# Intra-host genomic variation of serologically nontypeable *Haemophilus influenzae* isolates from otitis media

**DOI:** 10.1128/spectrum.03089-24

**Published:** 2025-03-31

**Authors:** Randall J. Olsen, S. Wesley Long, Yuvanesh Vedaraju, Sandra Tomasdottir, Helga Erlendsdottir, Karl G. Kristinsson, James M. Musser, Gunnsteinn Haraldsson

**Affiliations:** 1 Department of Pathology and Genomic Medicine, Laboratory for Molecular and Translational Human Infectious Diseases Research, Center for Infectious Diseases, Houston Methodist Research Institute, and Houston Methodist Hospital 23534 , Houston, Texas, USA; 2 Department of Pathology and Laboratory Medicine and Microbiology and Immunology, Weill Medical College of Cornell University 5922, New York City, New York, USA; 3 Department of Clinical Microbiology, Landspítali - the National University Hospital of Iceland, Reykjavik, Iceland; 4 Faculty of Medicine, School of Health Science, University of Iceland 63541, , Reykjavík, Capital Region, Iceland; Griffith University-Gold Coast Campus, Gold Coast, Australia

**Keywords:** intra-host variation, *Haemophilus influenzae*, genomics

## Abstract

Serologically nontypeable *Haemophilus influenzae* is a human pathogen that causes infections ranging in severity from mild otitis media and sinusitis to life-threatening pneumonia, bacteremia, and meningitis. Although intra-host genomic variation in infected humans has been studied, many important questions remain unanswered. To address this knowledge deficit, we sequenced the genomes of 500 isolates recovered from ear drainage fluid collected from 11 Icelandic children with otorrhea. We discovered substantial genomic diversity among the *H. influenzae* strains infecting each patient. In total, we identified 88 genes that acquired nonsynonymous (amino acid-changing) or nonsense (protein-truncating) single-nucleotide polymorphisms, insertions, or deletions in at least one isolate. Of these, 13 genes were recurrently polymorphic. The polymorphic genes encoded proteins with varied inferred functions, including carbohydrate metabolism, cell wall biosynthesis, environmental stress response, glycolipid metabolism, iron metabolism, recombination, small molecule transport, and transcription and translation. No amino acid substitutions or protein truncations were identified in proven *H. influenzae* virulence factors or major transcription regulators. However, many of the polymorphic genes likely contribute to fitness, virulence, or host-pathogen molecular interactions. Our study of intra-host variation in otitis media provides a framework for understanding the genomic adaptability of *H. influenzae* during human infections.

**IMPORTANCE:**

Serologically nontypeable *H. influenzae* is a human pathogen responsible for a range of diseases, including mild otitis media (middle ear infection) and sinusitis, and severe pneumonia, bacteremia, and meningitis. While research has begun advancing our understanding of the population genomic structure of *H. influenza* strains infecting humans, little is known about intra-host genomic variation. To address this knowledge gap, we sequenced the genomes of 500 *H*. *influenzae* isolates recovered from ear drainage fluid of Icelandic children diagnosed with otitis media. Our findings revealed that intra-host genomic variation involves many different genes encoding proteins with diverse functions. The data provide novel information bearing on the complexity of intra-host diversity and improve our understanding of *H. influenzae* strain fitness and molecular pathogenesis. This information could generate new hypotheses bearing on host-pathogen interactions and identify new therapeutic and vaccine targets.

## INTRODUCTION

*Haemophilus influenzae*, historically known as Pfeiffer’s bacillus or *Bacillus influenzae* (1), is a human pathogen that causes infections ranging in severity from mild otitis media (middle ear infection) and sinusitis to life-threatening pneumonia, bacteremia, and meningitis (2). *H. influenzae* also commonly colonizes the nasopharynx of young children and genitourinary tract of adult females (3–7). Strains are typically categorized based on expression of six antigenically distinct capsular polysaccharides—types a, b, c, d, e, f—or as unencapsulated (nontypeable). Multilocus sequence typing (MLST) is often used as a low-resolution estimate of genetic relationships between unencapsulated strains (8).

Prior to the introduction and widespread use of a vaccine against *H. influenzae* type b (Hib) in the late 20th century, Hib was a leading cause of meningitis and epiglottitis (9). Hib infections have markedly declined in countries with extensive Hib vaccination programs, while infections caused by unencapsulated strains have significantly increased (10). In the United States and Europe, the estimated annual incidence of invasive *H. influenzae* disease is 0.8–1.70 cases per 100,000 population (10–12), with most cases occurring in children under 1 year and adults over 65 years of age (10).

*H. influenzae* strain Rd was the first bacterial genome to be sequenced (13). Investigators have since studied the population genomic structure of *H. influenzae*, particularly unencapsulated strains (14–18). These studies have begun providing some insights into gene content, gene polymorphisms, and genomic diversity (14–18). Although intra-host genomic variation in infected humans has been studied, many important questions remain unanswered. Specifically, the genes or gene-encoded protein functions that most frequently acquire nonsynonymous (amino acid-changing) or nonsense (protein-truncating) single-nucleotide polymorphisms (SNPs), insertions, and deletions (indels) during infection remain unidentified. Studies of intra-host variation in other human pathogens, including SARS-CoV-2 (19, 20), influenza A virus (19), Ebola virus (21), *Streptococcus pyogenes* (22), *Staphylococcus aureus* (23, 24), *Klebsiella pneumoniae* (25), and others (26–28), have provided key insights into strain fitness, molecular pathogenesis, and host-pathogen interactions. However, research on *H. influenzae* intra-host variation has been limited by the use of low-resolution genetic techniques such as pulsed-field gel electrophoresis (29) and small strain collections or small patient cohorts (30, 31). To address this knowledge deficit, we sequenced the genomes of 500 *H*. *influenzae* isolates recovered from clinical samples of ear drainage fluid collected from 11 Icelandic children with otitis media.

## MATERIALS AND METHODS

### Specimen collection and *Haemophilus influenzae* identification

**Fig 1:**
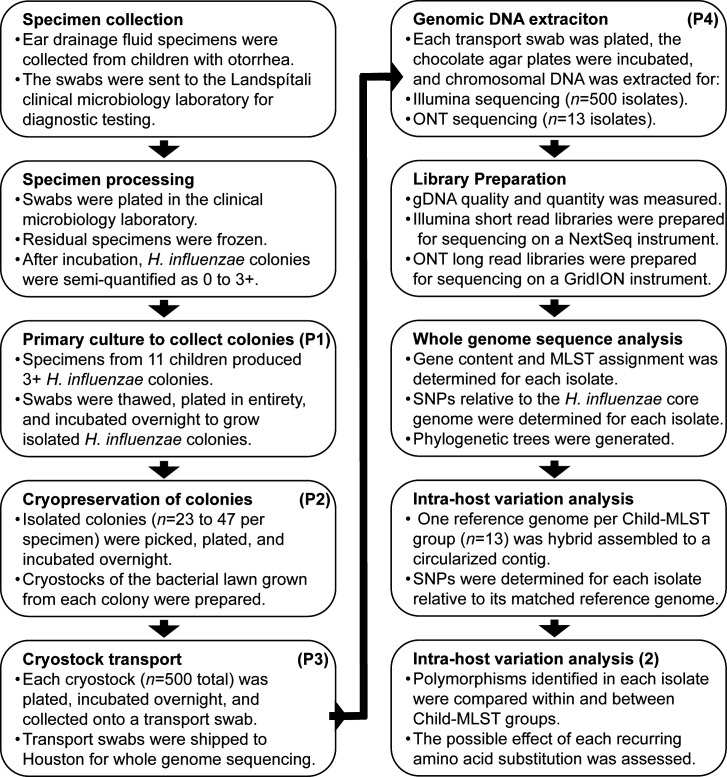
Isolate collection, genome sequencing, and intra-host variation analysis method. Passage number is marked as P# in the caption of each step.

Landspítali, the National University Hospital of Iceland, is the primary healthcare provider serving the diverse socioeconomic patient population of the Capital Region that includes Reykjavik and six adjacent municipalities (32), accounting for 64% of the population of Iceland (1 January 2024; www.statice.is). Landspítali’s clinical microbiology laboratory serves as the only reference laboratory in Iceland (3–5). As part of a routine diagnostic workup, ear drainage fluid specimens recovered from patients with otorrhea, with or without tympanostomy tubes, are submitted for pathogen identification and archival (Fig. 1). All samples used in this intra-host variation study were collected for diagnostic purposes by physicians treating symptomatic patients. Ear drainage fluid was collected from the external auditory canal using a swab, placed in transport media (Copan Italia s.p.a., Brescia, Italy), and cultured within 18 hours. Samples were plated on chocolate blood agar with a bacitracin disc (Becton, Dickinson, and Company, Vaud, Switzerland) and incubated aerobically with 5% CO_2_ for 18–20 hours at 36°C to recover *H. influenzae*. Samples were also plated on two tryptic soy agars supplemented with 5% sheep blood, one MacConkey agar, one SS agar, and one n-agar to recover other organisms that were not used in this study. Of note, two samples contained *Streptococcus pneumoniae* at low density and other samples contained various organisms, including *Staphylococcus aureus*, *Staphylococcus epidermidis*, *Staphylococcus hominis*, *Staphylococcus warneri*, *Streptococcus constellatus*, *Streptococcus intermedius*, *Turicella otitidis,* and yeasts. Residual specimen was cryopreserved at −80°C within 6 hours of primary plating without adding cryopreservatives. *H. influenzae* was identified based on colony morphology, resistance to bacitracin, and MALDI-TOF mass spectrometry (Bruker Daltonics, Billerica, MA). When present, *H. influenzae* colonies were semi-quantified as 0 to 3+ in each sample using standard clinical microbiology laboratory procedures (33).

### Specimen selection and isolate cultivation for intra-host variation analysis

Ear drainage fluid samples (*n* = 15) collected from 11 children in 2020 produced 3+ colonies in primary cultivation of *H. influenzae* (Fig. 1). The patients were randomly assigned a letter designation A–K for anonymity (Table 1). Nine patients (Child A–F, H, and J–K) had one sample collected from one ear on a single day. One patient (Child G) had one sample collected from each ear on a single day, and one patient (Child I) had samples collected from one ear on four separate days (Table 1). Within 2 days of the original plating, the 15 samples with 3+ *H. influenzae* growth were retrieved from the freezer, thawed, plated in entirety on chocolate blood agar plates, and incubated overnight. All isolated colonies (*n* = 23–47 colonies per sample; *n* = 500 colonies total) were picked (Table S1), labeled systematically, subcultured, and confirmed as *H. influenzae* using MALDI-TOF mass spectrometry. A loopful of colonial material from each subculture, representing one colony from the primary sample, was cryopreserved as a stock. Each stock was then plated and incubated for growth before being transferred onto a transport swab immersed in charcoal media (Medical Wire and Equipment Co Ltd, UK) and shipped to Houston Methodist Research Institute at room temperature for whole-genome sequence analysis (Fig. 1).

**TABLE 1:** Demographic information for the 11 children with otitis media

**Child**	**Age (years**)	**Sex**	**Laterality**	**Sampling day**	**Isolates (number**)
A	0.8	Male	Unilateral	0	47
B	0.1	Male	Unilateral	0	40
C	1.4	Male	Unilateral	0	31
D	1.8	Male	Unilateral	0	40
E	2.7	Female	Unilateral	0	35
F	2.5	Female	Unilateral	0	25
G	1.5	Female	Bilateral	0	23, 24
H	3.9	Male	Unilateral	0	29
I	3.6	Male	Multilateral	0, 2, 28, 35	25, 29, 39, 37
J	1.9	Female	Unilateral	0	37
K	1.1	Female	Unilateral	0	39

### Whole-genome sequence data generation

The genomes of the 500 *H*. *influenzae* isolates were sequenced using previously described methods (34–37). Briefly, all isolates (*n* = 500) underwent Illumina short-read sequencing. To provide a closed reference genome for each patient, one isolate per Child-MLST group (described below, *n* = 13) was sequenced by Oxford Nanopore Technology long-read sequencing and hybrid assembled to closure. Isolates were grown overnight at 37°C with 5% CO_2_ on chocolate agar. Chromosomal DNA was extracted using the DNeasy Blood and Tissue kit (Qiagen, Germantown, MD). Short-read sequencing libraries were prepared using the Nextera XT kit (Illumina, San Diego, CA) and sequenced on a NextSeq 550 instrument (Illumina) with a 2 × 150 base pair protocol. Long-read sequencing libraries were prepared with the Native Barcoding Kit V14 (Oxford Nanopore Technologies, OX4 4DQ, UK) and sequenced on a GridION instrument using version R10.4 flow cells.

### Whole-genome sequence analysis

Illumina short-reads were generated for each isolate (*n* = 500) to provide approximately 160.9-fold coverage per genome (mean: 305.8 mb/isolate, range: 36.9–589.3 mb/isolate) with mean 99.7% completeness and 1.9% contamination (38). The short reads were trimmed for quality and adaptor contamination using Trimmomatic (39) and error-corrected using Musket (40). Unpaired reads were removed using Fastq-pair (41). Gene content, including multilocus sequence type alleles, capsule genotype, and fucose operon genotype were determined using SRST2 with custom database files (42). Plasmid replicons and antimicrobial resistance genes were identified using SRST2 with database files PlasmidFinder.fasta and ARGannot.r1.fasta, respectively (42). The *H. influenzae* core and accessory genomes were generated from 99 closed genome sequences with diverse MLST lacking ambiguous bases (Table S2) deposited in the NCBI Microbial Genome Database (accessed 03/07/2023) using Bifrost (43) and Corer (44). Phylogenetic relationships among the 500 isolates were assessed by determining polymorphisms relative to the core genome using MUMMER dna-diff (45). Phylogenetic trees were generated using rapidnj, SplitsTree, and Dendroscope (46).

### Reference genome assembly, closure, and annotation

Oxford Nanopore long reads were generated for one isolate from each Child-MLST group (*n* = 13) to provide approximately 202.7-fold coverage per genome (mean 385.1 mb/isolate, range 32.3–1,304.2 mb/isolate) with >99.7% completeness and <2% contamination (38). Read length was assessed by FastQC (mean 2,199 b/isolate, range 66–112,293 b/isolate). Nucleotides were called using minKnow v.24.11.8 with high accuracy setting and 10 genomes per flow cell. Reference genomes were hybrid assembled from the short- and long-read sequencing data. Each reference strain was selected to be genetically representative of all isolates in the Child-MLST group. Illumina FASTQ files were trimmed and filtered using Trimmomatic 0.39 (39). Oxford Nanopore Technology FASTQ files were filtered using a 1 kb minimum length and 90% retention threshold with Filtlong 0.2.1 (47). Hybrid genome assembly was performed using Unicycler 0.5.0 with SPAdes 3.15.4 (48). Racon 1.5.0 (49) was used for assembly polishing using the “normal” Unicycler setting (50). Genomes that did not assemble to closure with the first approach were reattempted using the “bold” setting. Genomes that did not assemble using either hybrid approach were reattempted using a long-read-first approach with Trycycler, Minimap2 v2.17-r941, Miniasm v0.3-r179, Raven 1.8.1, Minipolish, Polypolish, and POLCA, as described previously (36). The closed reference genomes were annotated with PROKKA using the default settings (51). Each reference genome was assembled to a single circularized contig with N50 = genome size (Table 2) and >99.67% completeness (38). Artemis (52) and CLC Genomics Workbench (Qiagen) were used as genome browser and bioinformatics visualization tools. Graphing and statistical analysis were performed with Prism v10 (GraphPad Software, Boston, MA).

**TABLE 2:** Cohort of *H. influenzae* otitis media reference genomes

**Isolate**	**Child**	**MLST**	**Genome (bp**)	**Genes (number**)
MHI4419	A	155	1,815,833	1,789
MHI4466	B	155	1,906,308	1,856
MHI4506	C	145	1,850,644	1,795
MHI4537	D	1,013	1,824,312	1,744
MHI4578	E	1,030	1,903,238	1,902
MHI4615	F	1,030	1,836,726	1,760
MHI4640	G	145	1,851,026	1,793
MHI4688	H	3	1,977,871	1,977
MHI4743	I	1,927	1,884,791	1,854
MHI4772	I	266	1,911,825	1,884
MHI4812	J	583	1,901,692	1,911
MHI4814	J	99	1,957,403	2,004
MHI4890	K	590	1,939,587	1,948

### Intra-host variation determination

To assess intra-host variation, the trimmed, error-corrected, paired short-reads for each isolate were assembled into contigs using SPAdes (48). Polymorphisms in each genome were then determined relative to the child-specific MLST-matched reference genome using MUMMER dna-diff (45). An exclusion file, identifying regions of each closed reference genome with possibly mismapped reads, was created by mapping the corresponding reads to the closed reference genome. Genes encoding ribosomal proteins, transfer RNAs, and pilis proteins were also excluded. Polymorphism data were then processed using in-house scripts Prephix, Phrecon, pre2snpfx, and snpfx as previously described (25). Pairwise distances were determined using MEGA v11 (53). Phylogenetic trees were generated using rapidnj, SplitsTree, and Dendroscope (46). Annotated genes (hypothetical genes were excluded from further analysis, Table S3) in each isolate containing nonsynonymous (amino acid changing) or nonsense (protein truncating) SNPs or indels relative to the reference genome were manually curated. Gene function was determined using UniProt (54) and literature review.

## RESULTS

### Gene content analysis

Otorhea samples were collected from 11 children with symptomatic otitis media for clinical diagnosis. Whole-genome sequence analysis of 500 nontypeable *H. influenzae* isolates recovered from the samples were performed using Illumina short-read sequencing. To establish genetic relationships, the multilocus sequence type (MLST) of each isolate was determined. In total, 13 distinct Child-MLST groups were identified (Table 2; S1). Nine patients (Child A–H, K) were infected with strains from a single MLST at one timepoint. One patient (Child I) was infected with one MLST on days 0 and 2 but a different MLST on days 28 and 35. The two MLSTs differed at all seven alleles, suggesting two distinct episodes of otorrhea caused by genomically distinct clones. One patient (Child J) was simultaneously infected with two MLSTs that differed at six loci, suggesting a concurrent infection with two distantly related clones. Additionally, two patients (Child A and B) were both infected with MLST155 isolates. Similarly, two patients (Child C and G) and (Child E and F) were infected with MLST145 and MLST1030 isolates, respectively. The clonal complexes containing the 11 MLST infecting the Icelandic children are known causes of human upper respiratory tract infection, including otitis media (8).

Next, we analyzed gene content across the 500 *H*. *influenzae* isolates (Table S1). First, we assessed the presence of the fucose (*fuc*) operon, as its deletion has been reported in some *H. influenzae* lineages and may lead to a non-typeable MLST and species misidentification (55, 56). All isolates had an intact *fuc* operon with no SNPs or indels. Second, we analyzed genes encoding proteins required for capsule production, plasmid replication, and antimicrobial resistance (AMR). All isolates were unencapsulated (nontypeable) and lacked plasmid replicons (Table S1). While most isolates lacked AMR genes, genes encoding a TEM-1D beta-lactamase were present in some isolates recovered from Child J (*n* = 9/9 MLST99 isolates and *n* = 5/28 MLST583 isolates). Additionally, genes encoding an aminoglycoside 3′-phosphotransferase and TEM-1D beta-lactamase were present in all isolates recovered from Child K (*n* = 39/39 isolates).

### Phylogenetic analysis

**Fig 2:**
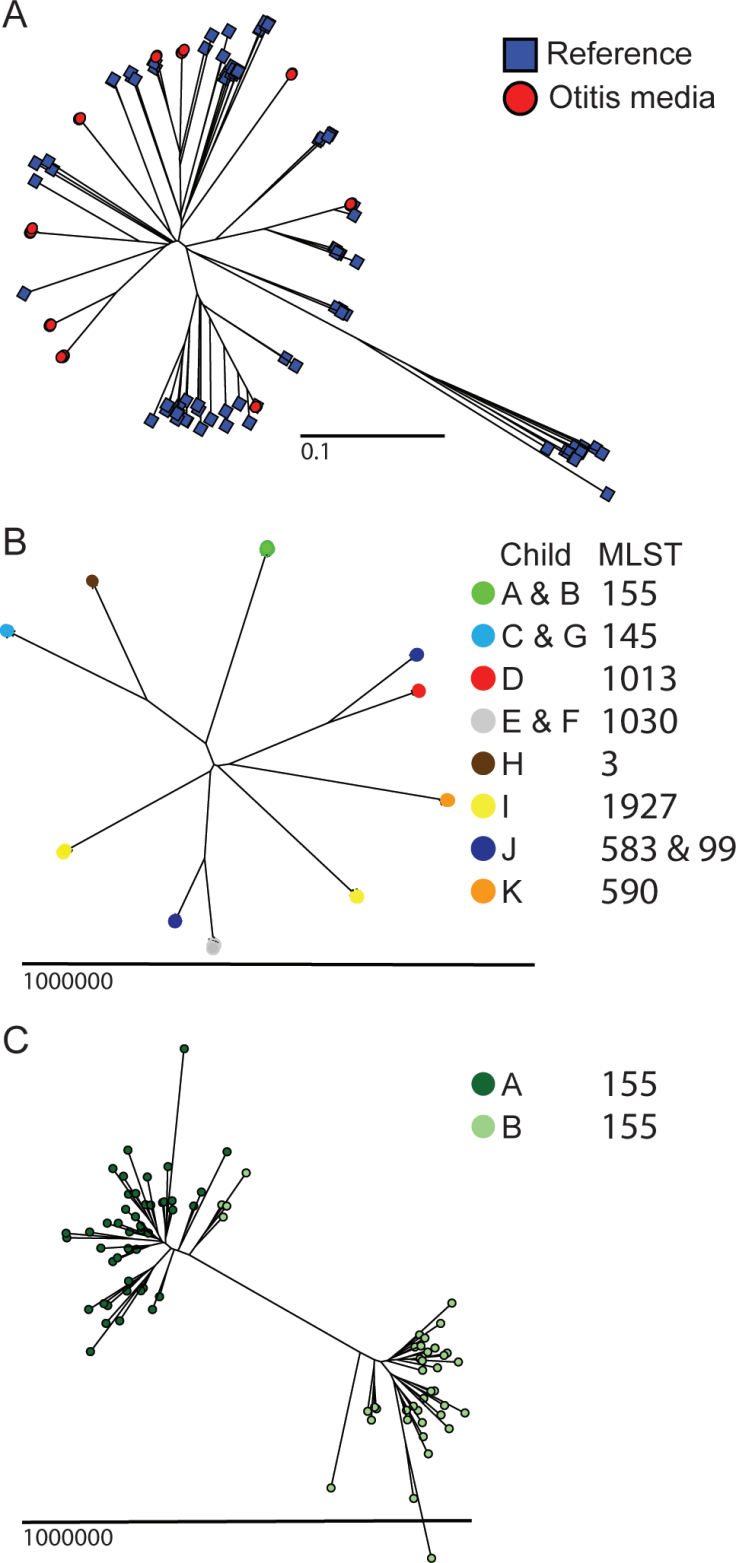
Phylogeny of *H. influenzae* isolates. Phylogeny was inferred by neighbor-joining based on SNPs identified by Illumina short-read whole-genome sequencing, with read mapping relative to the *H. influenzae* core genome. (A) The 99 reference strains (blue squares) and 500 otitis media isolates (red circles) are shown. (B) Whole-genome sequence analysis of otitis media isolates recovered from eleven anonymized patients (Child A–K). Because of clonal relatedness, closely related isolates appear overlapping at this scale. (C) At higher magnification, MLST155 strains recovered from two patients (Child A, dark green, and Child B, light green) form distinct subpopulations.

We constructed a phylogenetic tree to place the 500 *H*. *influenzae* otitis media isolates into a broader phylogenetic context. The *H. influenzae* core genome was determined using 99 publicly available closed genome sequences (Table S2), which served as a reference for determining SNPs and indels. This analysis revealed four key findings. First, the 13 Child-MLST groups were interspersed with the reference genomes, indicating that they represent a genomically diverse population (Fig. 2A). For the most part, reference and otitis media strains with the same MLST cluster closely on the phylogenetic tree. Second, we identified 120,500 unique SNP loci within the 500 otitis media isolates relative to the core genome (Table S4), underscoring their high level of genomic diversity (Fig. 2B). Third, isolates of the same MLST recovered from different patients formed closely related but separate subpopulations (Fig. 2C). For example, although the MLST155 isolates recovered from Child A and B were closely aligned relative to strains of other MLSTs (Fig. 2B), they formed distinct subpopulations (Fig. 2C), so they were further analyzed independently. In comparison, the MLST145 isolates recovered from the bilateral ears of Child G were intermingled on the phylogenetic tree, so they were analyzed as one group. Fourth, isolates of the same MLST collected at different time points for Child I did not form distinct subpopulations; MLST266 isolates from days 0 and 2 were interspersed, as were MLST1960 isolates from days 28 and 35 (Fig. 2B).

### Intra-host genetic variation analysis

To identify *H. influenzae* genes undergoing intra-host variation within each patient’s infected middle ear, we generated a closed circularized reference genome for each Child-MLST group using hybrid sequence assemblies (Table 2). We have successfully applied a similar approach in previous investigations of closely related strains of *Streptococcus pyogenes* (35), *Streptococcus dysgalactiae subspecies equisimilis* (36), and *Klebsiella pneumoniae* (25). This strategy enables highly accurate SNP determination by using clonally related reference strains (57). The 13 closed circularized reference genomes ranged from 1,815,833 to 1,977,871 base pairs and included 1,744 to 2,004 genes (Table 2), which is consistent with the genome size and gene content of other publicly available *H. influenzae* genomes (Table S2).

**Fig 3:**
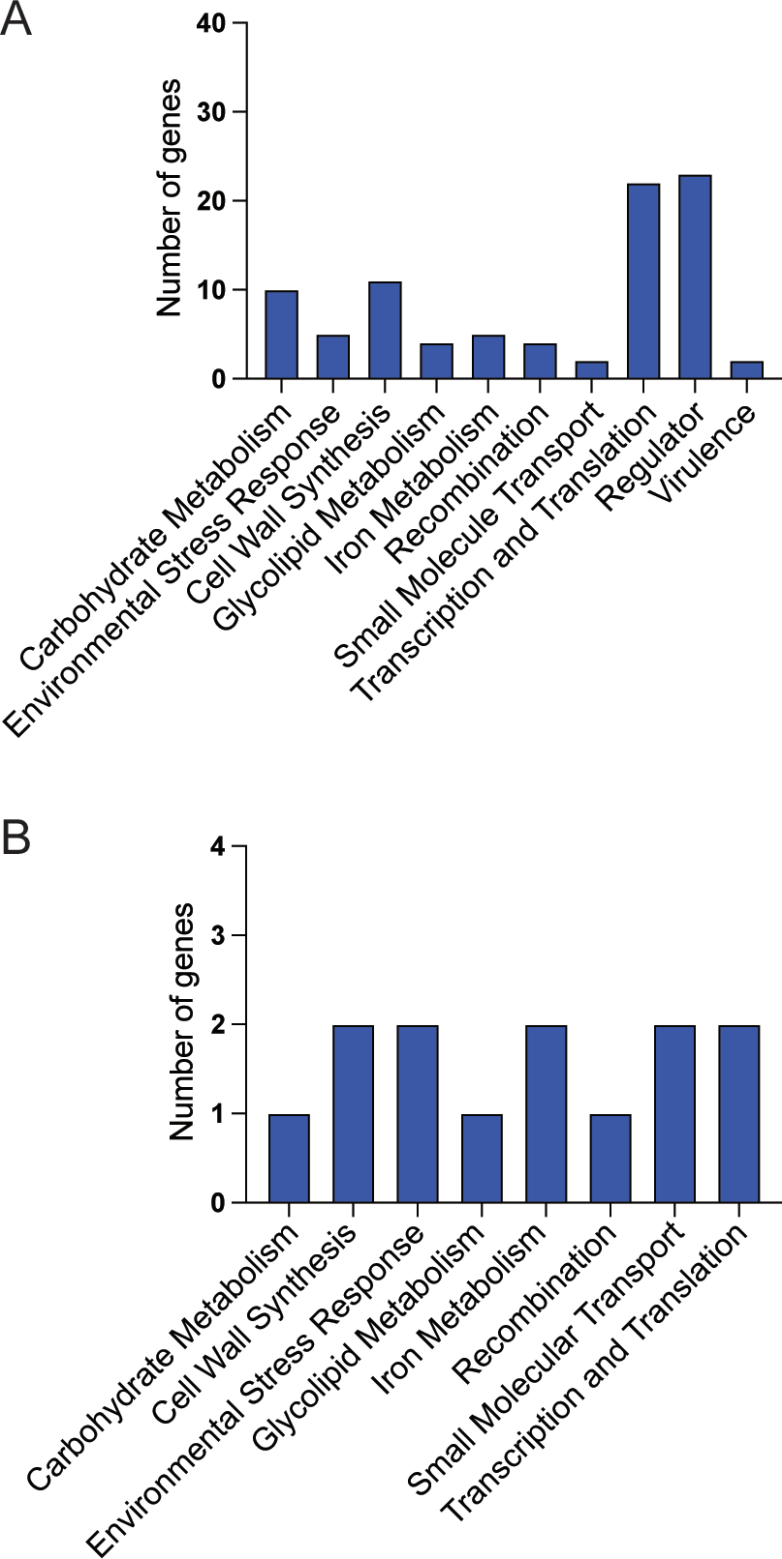
Function of genes undergoing intra-host variation. (A) Functional categorization of the 88 genes that acquired a nonsynonymous or nonsense SNP or indel in at least one otitis media isolate. (B) Functional categorization of the 13 genes identified as recurrently polymorphic.

For each *H. influenzae* otitis media isolate, SNPs were determined relative to the Child-MLST matched reference genome. The mean pairwise distances among strains in each group ranged from 4.4 to 25.8 SNPs (Table S5). Relative to the matched reference genome, no gene deletions were identified in any individual isolate. We next tabulated annotated genes with nonsynonymous (amino acid-changing) or nonsense (protein-truncating) mutations. In total, 88 polymorphic genes were found in at least one isolate (Table S6; Fig. 3A), including 13 recurrently polymorphic genes (Table 3, Fig. 3B). The function of the proteins encoded by the recurrently polymorphic genes included cell wall biosynthesis (*n* = 2 genes), environmental stress response (*n* = 2 genes), iron metabolism (*n* = 2 genes), small molecule transport (*n* = 2 genes), transcription and translation (*n* = 2 genes), carbohydrate metabolism (*n* = 1 gene), glycolipid metabolism (*n* = 1 gene), and recombination (*n* = 1 gene). For the most part, the polymorphic genes are present and have high nucleotide identity in the majority of the Child-MLST groups (Table S5). That is, the genes acquiring intra-host polymorphisms are well conserved and do not have high population-level diversity.

**TABLE 3:** Genes with recurrent polymorphisms^*a*^

Gene	Protein	Function	Polymorphism		Substitution		Position in codon	Child-MLST	Isolate
*ftnA_2*	Bacterial non-heme ferritin	Iron metabolism	C86A		A29D		2	D_1013	MHI4553
			A406G		S136G		1	K_590	MHI4911
							
*mukB*	Chromosome partition protein	Cell wall biosynthesis	C1610T		S537N		2	I_266	MHI4867
			C3455A		A1152E		2	E_1030	MHI4586
							
*nanK*	N-acetylmannosamine kinase	Carbohydrate metabolism	C622G		E208Q		1	H_3	MHI4716
			C622G		E208Q		1	I_266	MHI4809
			C436A		G146C		1	I_266	MHI4867
							
*opgE_1*	Phosphoethanolamine transferase	Cell wall biosynthesis	A1321C		I441L		1	D_1013	MHI4541
			T618G		F206L		3	I_266	MHI4867
							
*patB*	Cystathionine beta-lyase	Iron metabolism	A83C		I28S		2	D_1013	MHI4573
			C794A		P265Q		2	K_590	MHI4904
			C815A		A272D		2	K_590	MHI4904
							
*pglH*	Protein glycosylation H	Transcription/ translation	C697A		A233B		1	B_155	MHI4478
			C184A		P62T		1	E_1030	MHI4592
			C860A		C287F		2	H_3	MHI4710
			C1015A		D339Y		1	I_266	MHI4487
			T582G		F191L		3	I_1927	MHI4747
			A573C		K194N		3	I_1927	MHI4747
							
*phoR*	Phosphate regulon sensor protein	Small molecule transport	G424T		Q424K		1	A_155	MHI4446
			T137G		L46*		2	J_583	MHI4813
			G341T		C114F		2	J_583	MHI4821
			G479T		T160N		2	J_99	MHI4845
			T384A		K128N		3	K_590	MHI4907
			T356A		Q119L		2	K_590	MHI4907
			G296T		P99Q		2	K_590	MHI4907
			G139T		L47I		1	K_590	MHI4904
							
*plsB*	Glycerol-3-phosphate acyltransferase	Glycolipid metabolism	A2027C		I676S		2	I_266	MHI4867
			G262A		G88S		1	K_590	MHI4909
							
*recD2*	RecD-like DNA helicase	Recombination	T2516A		F839Y		2	D_1013	MHI4552
			G2519T		S840I		2	D_1013	MHI4552
			T2528A		F843Y		2	D_1013	MHI4552
			G2551T		V851L		1	D_1013	MHI4552
			G2593T		V865L		1	D_1013	MHI4552
			A2729C		N910T		2	D_1013	MHI4547
			A2639C		D880A		2	J_99	MHI4842
			A2653C		M885L		1	J_99	MHI4842
							
*sohB*	Inner membrane peptidase	Environmental stress response	C111A		N37K		3	I_266	MHI4867
			G151T		E51*		1	I_266	MHI4867
			G149T		S50*		2	K_590	MHI4910
							
*sppA_1*	Stringent starvation protein A	Environmental stress response	G1711A		A571T		1	C_145	MHI4519, MHI4528, MHI4534
			C187T		A63T		1	G_1030	MHI4648, MHI4652, MHI4664, MHI4666, MHI4669
							
*ydgA*	DNA topoisomerase	Transcription/ translation	A172G		K58E		1	I_266	MHI4867
			A190T		R64*		1	I_266	MHI4867
			G226T		E76*		1	I_1927	MHI4750
							
*yjhB*	Putative metabolite transport protein	Small molecule transport	G1173T		M391I		3	I_266	MHI4867
			C1214A		A405D		2	I_266	MHI4867
			T734G		L245*		2	I_1927	MHI4740

^*a*^ *, nonsense codon.

### Diversifying selection of recurrently polymorphic genes

To test the hypothesis that nonsynonymous and nonsense mutations identified in the 13 recurrently polymorphic genes occurred through diversifying selection, we evaluated the molecular consequence of each genetic change defining the 46 unique variant alleles. Only three nonsynonymous (silent, not resulting in an amino acid replacement or protein truncation; *ftnA_2* A381G, *phoR* G141T, and *sspA_1* 312C) changes were identified among the 13 genes in the 500 isolates. That is, compared with the wild-type sequence, 46/49 (93.9%) variant alleles encode an altered protein sequence. The nucleotide changes were significantly overrepresented in the first and second positions of the variant codons (Table 3), enabling us to reject the hypothesis of selective neutrality (*χ*^2^ test, *P* = 0.025, *χ*^2^ = 4.998, *z* = 2.236, calculated using 40 nucleotide changes observed in the first 2 positions and 31 nucleotide changes expected if the 46 polymorphisms were randomly distributed across three codon positions). Most alleles occurred in a single isolate or very few isolates (Table 3). Taken together, these data are consistent with a model of intra-host variation in which new alleles emerge through diversifying selection.

## DISCUSSION

Intra-host variation studies have provided valuable insights into strain fitness, virulence, and molecular pathogenesis in numerous human pathogens (19–28). Although intra-host genomic variation of nontypeable *H. influenzae* in infected humans has been studied (15), many important questions remain unanswered. To address this gap, we sequenced the genomes of 500 colonies isolated from 11 Icelandic children with otorrhea. Our goal was to identify genes with nonsynonymous or nonsense SNPs and indels acquired during human infection. Nonsense mutations, in particular, may have a substantial impact to *in vivo* protein structure or function. This information could generate new hypotheses bearing on host-pathogen interactions and identify new therapeutic and vaccine targets.

In total, we identified 88 genes, representing 4.7% of the mean 1,863 genes in the 13 closed circularized *H. influenzae* otitis media reference genomes that acquired nonsynonymous or nonsense mutations. Of these, 13 genes encoding proteins with diverse inferred functions were recurrently polymorphic. Notably, we found no amino acid substitutions or protein truncations in proven *H. influenzae* virulence factors such as secreted toxins or major transcription regulators, and no clear signal of convergent evolution. However, many of the polymorphic genes likely contribute to fitness, virulence, or host-pathogen molecular interactions.

Three key results emerged regarding the frequency and distribution of SNPs and indels. First, most polymorphisms were unique to individual isolates or patients (Table S6). Similarly, most polymorphisms in the 13 recurrently polymorphic genes occurred only once. For example, FtnA_2 A29D occurred in Child D and K590G occurred in Child K; MukB S537N occurred in Child I and A1152E occurred in Child E; and OpgE_1 I441L occurred in Child D and F206L occurred in Child I. Second, some recurrently polymorphic genes encoded proteins with two amino acid changes within the same isolate. For example, PatB P265Q and A272D occurred in one isolate recovered from Child K, while SohB N37K and E51*, YdgA K58E and R64*, and YjhB M391I and A405D each occurred in one isolate recovered from Child I. The proximity of the two altered codons could indicate recombination events in this naturally competent organism (58). Alternatively, the multiple amino acid substitutions could act synergistically to alter protein function (59, 60). Third, only two proteins, NanK and SppA, had identical amino acid substitutions in isolates recovered from multiple patients. Neither protein has been well studied in *H. influenzae*. NanK, which belongs to the ROK (repressor, open reading frame, kinase) superfamily of enzymes (61), had an E208Q substitution in one isolate each from Child H and I. E208 is a highly conserved residue across *H. influenzae* and other species (62). Although E208 is not part of the NanK ATP (amino acids 5–12 and 132–139) or Zn^2+^ binding motifs (amino acids 156, 166, 168, and 173), it is predicted to be surface exposed (63). Notably, a second strain recovered from Child I had a G146C amino acid substitution located between the second ATP and first Zn^2+^ binding site. The glutamic acid to glutamine and glycine to cysteine substitutions likely alter side chain charge and polarity to disrupt electrostatic interactions within NanK or between NanK and other proteins. NanK converts sialic acids into a phosphorylated form before catabolism of the sugar. Studies in other organisms suggest that NanK enables scavenging of host sialic acids, which are abundant in mucous membranes such as the middle ear (62, 64). Incorporation of sialic acids into bacterial surface molecules facilitates host immune evasion. We hypothesize that the intra-host substitutions in NanK alter sialic acid catabolism in a manner that favors *H. influenzae* intra-host immune evasion, persistence, or virulence (62, 65). In comparison, SppA, which is implicated in environmental stress response, had an A571T substitution in three isolates from Child C and A63T substitution in five isolates from Child G. A63 is located between the transmembrane and topological domains of SppA, and A571 is located within the topological domain (63). The alanine to threonine amino acid substitutions at either position may alter side chain polarity and similarly disrupt SppA structure or function. Importantly, SppA expression is induced by iron starvation to increase iron uptake (66), a critical function given the low concentrations of iron in the middle ear (67). We hypothesize that SppA substitutions may enhance intra-host strain fitness in this environment. The roles of NanK and SppA in *H. influenzae* host-pathogen interactions and fitness in otitis media merit further investigation.

The most polymorphic gene in our intra-host variation study was *phoR*. In total, we observed eight distinct PhoR amino acid substitutions or protein truncations across six isolates recovered from four patients. PhoR has not been previously studied in *H. influenzae,* so its regulon is undefined. However, PhoR functions in other organisms as the signaling histidine kinase for the PhoR/PhoB two-component regulatory system (68) that controls expression of phosphate regulon genes (47). Phosphate is naturally present in all body fluids, including the middle ear, and its levels increase in chronic otitis (69, 70). In other organisms, the PhoR phosphate regulon contains genes encoding proteins involved in virulence activities such as resistance to serum bactericidal activity, response to acid stress, and defense against cationic antimicrobial peptides (71). Inactivation of genes in the phosphate transport system, the PhoR/PhoB two-component system, or the phosphate regulon alters strain virulence in various *in vitro* and *in vivo* infection models (71). We hypothesize that intra-host variation in *phoR* promotes phenotypic diversity, enhancing *H. influenzae* fitness in the middle ear by enabling adaptive responses to fluctuating environmental conditions and host defenses. That is, intra-host variation of PhoR could favor *H. influenzae* immune evasion, persistence, or virulence.

This is the largest study to investigate intra-host variability of *H. influenzae* isolates causing human infections. However, there are some limitations in the study design. First, although we conject a minimal detriment from sample cryopreservation, multiple passages, and shipping in charcoal transport medium, an underestimated effect on sample recovery and isolate diversity is possible. Second, restricting the intra-host variation study to children with swab samples having dense growth from the primary culture could also lead to unforeseen bias in sample recovery and isolate diversity. Third, SARS-CoV-2 is well documented to have altered the prevalence and transmission pattern of many respiratory pathogens during and after the pandemic (72, 73). The first SARS-CoV-2 positive patient in Iceland was identified on 28 February 2020 and initial Covid-related restrictions were implemented on 16 March 2020 but did not apply to schools and day care centers (74). Since the otorrhea samples were collected during 2020, a pandemic-related effect on *H influenzae* infection, transmission, or intra-host variation is possible. Whereas, Child A–J were sampled before 1 April 2020 and are unlikely to be affected by Covid, Child K was sampled in late 2020.

In summary, whole-genome sequence analysis of 500 *H. influenzae* isolates recovered from 11 Icelandic children with otorrhea identified 88 polymorphic genes, including 13 recurrently polymorphic genes encoding proteins with diverse functions. No single convergent evolutionary pathway was detected, indicating that *H. influenzae* intra-host variation is complex. Additional intra-host variation studies using strains from infections occurring at different anatomic sites or asymptomatic carriers could provide further insights to host-pathogen molecular interactions, strain fitness, and potential targets for therapies or vaccines.

## Data Availability

Whole genome sequencing data for this investigation was submitted to the National Center for Biotechnology Information Sequence Read Archive and closed genomes were submitted to GenBank under BioProject accession PRJNA1130518.

## SUPPLEMENTAL MATERIAL

Table S1 spectrum.03089-24-s0001.pdf Cohort of 500 *H. influenzae* isolates recovered from 11 children.

Table S2 spectrum.03089-24-s0002.pdf Cohort of 99 publicly available *H. influenzae* genomes.

Table S3 spectrum.03089-24-s0003.pdf Hypothetical genes with a nonsynonymous or nonsense polymorphism.

Table S4 spectrum.03089-24-s0004.pdf 120,500 unique SNP loci relative to the core genome.

Table S5 spectrum.03089-24-s0005.pdf Genes with a polymorphism in at least one otitis media isolate.

Table S6 spectrum.03089-24-s0006.pdf Mean pairwise distances among isolates in each Child-MLST group.

ASM does not own the copyrights to Supplemental Material that may be linked to, or accessed through, an article. The authors have granted ASM a non-exclusive, world-wide license to publish the Supplemental Material files. Please contact the corresponding author directly for reuse.
