# Supplementary material for: Intra-host genomic variation of serologically nontypeable Haemophilus influenzae isolates from otitis media
Source: Microbiol Spectr. 2025 Mar 31;13(5):e03089-24. doi: 10.1128/spectrum.03089-24 (PMC12053901; doi:10.1128/spectrum.03089-24)
Supplement: Table S1 — Cohort of 500 H. influenzae isolates recovered from 11 children. [file spectrum.03089-24-s0001.pdf]

Table S1. Cohort of 500 *H. influenzae* isolates recovered from 11 children

Strain	Child	MLST	*fuc*	Capsule Genotype	Plasmid Replicons
MHI4419	A	155	present	unencapsulated	none detected
MHI4420	A	155	present	unencapsulated	none detected
MHI4421	A	155	present	unencapsulated	none detected
MHI4422	A	155	present	unencapsulated	none detected
MHI4423	A	155	present	unencapsulated	none detected
MHI4424	A	155	present	unencapsulated	none detected
MHI4425	A	155	present	unencapsulated	none detected
MHI4426	A	155	present	unencapsulated	none detected
MHI4427	A	155	present	unencapsulated	none detected
MHI4428	A	155	present	unencapsulated	none detected
MHI4429	A	155	present	unencapsulated	none detected
MHI4430	A	155	present	unencapsulated	none detected
MHI4431	A	155	present	unencapsulated	none detected
MHI4432	A	155	present	unencapsulated	none detected
MHI4433	A	155	present	unencapsulated	none detected
MHI4434	A	155	present	unencapsulated	none detected
MHI4435	A	155	present	unencapsulated	none detected
MHI4436	A	155	present	unencapsulated	none detected
MHI4437	A	155	present	unencapsulated	none detected
MHI4438	A	155	present	unencapsulated	none detected
MHI4439	A	155	present	unencapsulated	none detected
MHI4440	A	155	present	unencapsulated	none detected
MHI4441	A	155	present	unencapsulated	none detected
MHI4442	A	155	present	unencapsulated	none detected
MHI4443	A	155	present	unencapsulated	none detected
MHI4444	A	155	present	unencapsulated	none detected
MHI4445	A	155	present	unencapsulated	none detected
MHI4446	A	155	present	unencapsulated	none detected
MHI4447	A	155	present	unencapsulated	none detected
MHI4448	A	155	present	unencapsulated	none detected
MHI4449	A	155	present	unencapsulated	none detected
MHI4450	A	155	present	unencapsulated	none detected
MHI4451	A	155	present	unencapsulated	none detected
MHI4452	A	155	present	unencapsulated	none detected
MHI4453	A	155	present	unencapsulated	none detected
MHI4454	A	155	present	unencapsulated	none detected
MHI4455	A	155	present	unencapsulated	none detected
MHI4456	A	155	present	unencapsulated	none detected
MHI4457	A	155	present	unencapsulated	none detected
MHI4458	A	155	present	unencapsulated	none detected

MHI4459	A	155	present	unencapsulated	none detected
MHI4460	A	155	present	unencapsulated	none detected
MHI4461	A	155	present	unencapsulated	none detected
MHI4462	A	155	present	unencapsulated	none detected
MHI4463	A	155	present	unencapsulated	none detected
MHI4464	A	155	present	unencapsulated	none detected
MHI4465	A	155	present	unencapsulated	none detected
MHI4466	B	155	present	unencapsulated	none detected
MHI4467	B	155	present	unencapsulated	none detected
MHI4468	B	155	present	unencapsulated	none detected
MHI4469	B	155	present	unencapsulated	none detected
MHI4470	B	155	present	unencapsulated	none detected
MHI4471	B	155	present	unencapsulated	none detected
MHI4472	B	155	present	unencapsulated	none detected
MHI4473	B	155	present	unencapsulated	none detected
MHI4474	B	155	present	unencapsulated	none detected
MHI4475	B	155	present	unencapsulated	none detected
MHI4476	B	155	present	unencapsulated	none detected
MHI4477	B	155	present	unencapsulated	none detected
MHI4478	B	155	present	unencapsulated	none detected
MHI4479	B	155	present	unencapsulated	none detected
MHI4480	B	155	present	unencapsulated	none detected
MHI4481	B	155	present	unencapsulated	none detected
MHI4482	B	155	present	unencapsulated	none detected
MHI4483	B	155	present	unencapsulated	none detected
MHI4484	B	155	present	unencapsulated	none detected
MHI4485	B	155	present	unencapsulated	none detected
MHI4486	B	155	present	unencapsulated	none detected
MHI4487	B	155	present	unencapsulated	none detected
MHI4488	B	155	present	unencapsulated	none detected
MHI4489	B	155	present	unencapsulated	none detected
MHI4490	B	155	present	unencapsulated	none detected
MHI4491	B	155	present	unencapsulated	none detected
MHI4492	B	155	present	unencapsulated	none detected
MHI4493	B	155	present	unencapsulated	none detected
MHI4494	B	155	present	unencapsulated	none detected
MHI4495	B	155	present	unencapsulated	none detected
MHI4496	B	155	present	unencapsulated	none detected
MHI4497	B	155	present	unencapsulated	none detected
MHI4498	B	155	present	unencapsulated	none detected
MHI4499	B	155	present	unencapsulated	none detected
MHI4500	B	155	present	unencapsulated	none detected
MHI4501	B	155	present	unencapsulated	none detected

MHI4502	B	155	present	unencapsulated	none detected
MHI4503	B	155	present	unencapsulated	none detected
MHI4504	B	155	present	unencapsulated	none detected
MHI4505	B	155	present	unencapsulated	none detected
MHI4506	C	145	present	unencapsulated	none detected
MHI4507	C	145	present	unencapsulated	none detected
MHI4508	C	145	present	unencapsulated	none detected
MHI4509	C	145	present	unencapsulated	none detected
MHI4510	C	145	present	unencapsulated	none detected
MHI4511	C	145	present	unencapsulated	none detected
MHI4512	C	145	present	unencapsulated	none detected
MHI4513	C	145	present	unencapsulated	none detected
MHI4514	C	145	present	unencapsulated	none detected
MHI4515	C	145	present	unencapsulated	none detected
MHI4516	C	145	present	unencapsulated	none detected
MHI4517	C	145	present	unencapsulated	none detected
MHI4518	C	145	present	unencapsulated	none detected
MHI4519	C	145	present	unencapsulated	none detected
MHI4520	C	145	present	unencapsulated	none detected
MHI4521	C	145	present	unencapsulated	none detected
MHI4522	C	145	present	unencapsulated	none detected
MHI4523	C	145	present	unencapsulated	none detected
MHI4524	C	145	present	unencapsulated	none detected
MHI4525	C	145	present	unencapsulated	none detected
MHI4526	C	145	present	unencapsulated	none detected
MHI4527	C	145	present	unencapsulated	none detected
MHI4528	C	145	present	unencapsulated	none detected
MHI4529	C	145	present	unencapsulated	none detected
MHI4530	C	145	present	unencapsulated	none detected
MHI4531	C	145	present	unencapsulated	none detected
MHI4532	C	145	present	unencapsulated	none detected
MHI4533	C	145	present	unencapsulated	none detected
MHI4534	C	145	present	unencapsulated	none detected
MHI4535	C	145	present	unencapsulated	none detected
MHI4536	C	145	present	unencapsulated	none detected
MHI4537	D	1013	present	unencapsulated	none detected
MHI4538	D	1013	present	unencapsulated	none detected
MHI4539	D	1013	present	unencapsulated	none detected
MHI4540	D	1013	present	unencapsulated	none detected
MHI4541	D	1013	present	unencapsulated	none detected
MHI4542	D	1013	present	unencapsulated	none detected
MHI4543	D	1013	present	unencapsulated	none detected
MHI4544	D	1013	present	unencapsulated	none detected

MHI4545	D	1013	present	unencapsulated	none detected
MHI4546	D	1013	present	unencapsulated	none detected
MHI4547	D	1013	present	unencapsulated	none detected
MHI4548	D	1013	present	unencapsulated	none detected
MHI4549	D	1013	present	unencapsulated	none detected
MHI4550	D	1013	present	unencapsulated	none detected
MHI4551	D	1013	present	unencapsulated	none detected
MHI4552	D	1013	present	unencapsulated	none detected
MHI4553	D	1013	present	unencapsulated	none detected
MHI4554	D	1013	present	unencapsulated	none detected
MHI4555	D	1013	present	unencapsulated	none detected
MHI4556	D	1013	present	unencapsulated	none detected
MHI4557	D	1013	present	unencapsulated	none detected
MHI4558	D	1013	present	unencapsulated	none detected
MHI4559	D	1013	present	unencapsulated	none detected
MHI4560	D	1013	present	unencapsulated	none detected
MHI4561	D	1013	present	unencapsulated	none detected
MHI4562	D	1013	present	unencapsulated	none detected
MHI4563	D	1013	present	unencapsulated	none detected
MHI4564	D	1013	present	unencapsulated	none detected
MHI4565	D	1013	present	unencapsulated	none detected
MHI4566	D	1013	present	unencapsulated	none detected
MHI4567	D	1013	present	unencapsulated	none detected
MHI4568	D	1013	present	unencapsulated	none detected
MHI4569	D	1013	present	unencapsulated	none detected
MHI4570	D	1013	present	unencapsulated	none detected
MHI4571	D	1013	present	unencapsulated	none detected
MHI4572	D	1013	present	unencapsulated	none detected
MHI4573	D	1013	present	unencapsulated	none detected
MHI4574	D	1013	present	unencapsulated	none detected
MHI4575	D	1013	present	unencapsulated	none detected
MHI4576	D	1013	present	unencapsulated	none detected
MHI4577	E	1030	present	unencapsulated	none detected
MHI4578	E	1030	present	unencapsulated	none detected
MHI4579	E	1030	present	unencapsulated	none detected
MHI4580	E	1030	present	unencapsulated	none detected
MHI4581	E	1030	present	unencapsulated	none detected
MHI4582	E	1030	present	unencapsulated	none detected
MHI4584	E	1030	present	unencapsulated	none detected
MHI4585	E	1030	present	unencapsulated	none detected
MHI4586	E	1030	present	unencapsulated	none detected
MHI4587	E	1030	present	unencapsulated	none detected
MHI4588	E	1030	present	unencapsulated	none detected

MHI4589	E	1030	present	unencapsulated	none detected
MHI4590	E	1030	present	unencapsulated	none detected
MHI4591	E	1030	present	unencapsulated	none detected
MHI4592	E	1030	present	unencapsulated	none detected
MHI4593	E	1030	present	unencapsulated	none detected
MHI4594	E	1030	present	unencapsulated	none detected
MHI4595	E	1030	present	unencapsulated	none detected
MHI4596	E	1030	present	unencapsulated	none detected
MHI4597	E	1030	present	unencapsulated	none detected
MHI4598	E	1030	present	unencapsulated	none detected
MHI4599	E	1030	present	unencapsulated	none detected
MHI4600	E	1030	present	unencapsulated	none detected
MHI4601	E	1030	present	unencapsulated	none detected
MHI4602	E	1030	present	unencapsulated	none detected
MHI4603	E	1030	present	unencapsulated	none detected
MHI4604	E	1030	present	unencapsulated	none detected
MHI4605	E	1030	present	unencapsulated	none detected
MHI4606	E	1030	present	unencapsulated	none detected
MHI4607	E	1030	present	unencapsulated	none detected
MHI4608	E	1030	present	unencapsulated	none detected
MHI4611	E	1030	present	unencapsulated	none detected
MHI4612	E	1030	present	unencapsulated	none detected
MHI4613	E	1030	present	unencapsulated	none detected
MHI4614	E	1030	present	unencapsulated	none detected
MHI4615	F	1030	present	unencapsulated	none detected
MHI4616	F	1030	present	unencapsulated	none detected
MHI4617	F	1030	present	unencapsulated	none detected
MHI4618	F	1030	present	unencapsulated	none detected
MHI4619	F	1030	present	unencapsulated	none detected
MHI4620	F	1030	present	unencapsulated	none detected
MHI4621	F	1030	present	unencapsulated	none detected
MHI4622	F	1030	present	unencapsulated	none detected
MHI4623	F	1030	present	unencapsulated	none detected
MHI4624	F	1030	present	unencapsulated	none detected
MHI4625	F	1030	present	unencapsulated	none detected
MHI4626	F	1030	present	unencapsulated	none detected
MHI4627	F	1030	present	unencapsulated	none detected
MHI4628	F	1030	present	unencapsulated	none detected
MHI4629	F	1030	present	unencapsulated	none detected
MHI4630	F	1030	present	unencapsulated	none detected
MHI4631	F	1030	present	unencapsulated	none detected
MHI4632	F	1030	present	unencapsulated	none detected
MHI4633	F	1030	present	unencapsulated	none detected

MHI4634	F	1030	present	unencapsulated	none detected
MHI4635	F	1030	present	unencapsulated	none detected
MHI4636	F	1030	present	unencapsulated	none detected
MHI4637	F	1030	present	unencapsulated	none detected
MHI4638	F	1030	present	unencapsulated	none detected
MHI4639	F	1030	present	unencapsulated	none detected
MHI4640	G	145	present	unencapsulated	none detected
MHI4641	G	145	present	unencapsulated	none detected
MHI4642	G	145	present	unencapsulated	none detected
MHI4643	G	145	present	unencapsulated	none detected
MHI4644	G	145	present	unencapsulated	none detected
MHI4645	G	145	present	unencapsulated	none detected
MHI4646	G	145	present	unencapsulated	none detected
MHI4647	G	145	present	unencapsulated	none detected
MHI4648	G	145	present	unencapsulated	none detected
MHI4649	G	145	present	unencapsulated	none detected
MHI4650	G	145	present	unencapsulated	none detected
MHI4651	G	145	present	unencapsulated	none detected
MHI4652	G	145	present	unencapsulated	none detected
MHI4653	G	145	present	unencapsulated	none detected
MHI4654	G	145	present	unencapsulated	none detected
MHI4655	G	145	present	unencapsulated	none detected
MHI4656	G	145	present	unencapsulated	none detected
MHI4657	G	145	present	unencapsulated	none detected
MHI4658	G	145	present	unencapsulated	none detected
MHI4659	G	145	present	unencapsulated	none detected
MHI4660	G	145	present	unencapsulated	none detected
MHI4661	G	145	present	unencapsulated	none detected
MHI4662	G	145	present	unencapsulated	none detected
MHI4663	G	145	present	unencapsulated	none detected
MHI4664	G	145	present	unencapsulated	none detected
MHI4665	G	145	present	unencapsulated	none detected
MHI4666	G	145	present	unencapsulated	none detected
MHI4667	G	145	present	unencapsulated	none detected
MHI4668	G	145	present	unencapsulated	none detected
MHI4669	G	145	present	unencapsulated	none detected
MHI4670	G	145	present	unencapsulated	none detected
MHI4671	G	145	present	unencapsulated	none detected
MHI4672	G	145	present	unencapsulated	none detected
MHI4673	G	145	present	unencapsulated	none detected
MHI4674	G	145	present	unencapsulated	none detected
MHI4675	G	145	present	unencapsulated	none detected
MHI4676	G	145	present	unencapsulated	none detected

MHI4677	G	145	present	unencapsulated	none detected
MHI4678	G	145	present	unencapsulated	none detected
MHI4679	G	145	present	unencapsulated	none detected
MHI4680	G	145	present	unencapsulated	none detected
MHI4681	G	145	present	unencapsulated	none detected
MHI4682	G	145	present	unencapsulated	none detected
MHI4683	G	145	present	unencapsulated	none detected
MHI4684	G	145	present	unencapsulated	none detected
MHI4686	G	145	present	unencapsulated	none detected
MHI4687	G	145	present	unencapsulated	none detected
MHI4688	H	3	present	unencapsulated	none detected
MHI4689	H	3	present	unencapsulated	none detected
MHI4690	H	3	present	unencapsulated	none detected
MHI4691	H	3	present	unencapsulated	none detected
MHI4692	H	3	present	unencapsulated	none detected
MHI4693	H	3	present	unencapsulated	none detected
MHI4694	H	3	present	unencapsulated	none detected
MHI4695	H	3	present	unencapsulated	none detected
MHI4696	H	3	present	unencapsulated	none detected
MHI4697	H	3	present	unencapsulated	none detected
MHI4698	H	3	present	unencapsulated	none detected
MHI4699	H	3	present	unencapsulated	none detected
MHI4700	H	3	present	unencapsulated	none detected
MHI4701	H	3	present	unencapsulated	none detected
MHI4702	H	3	present	unencapsulated	none detected
MHI4703	H	3	present	unencapsulated	none detected
MHI4704	H	3	present	unencapsulated	none detected
MHI4705	H	3	present	unencapsulated	none detected
MHI4707	H	3	present	unencapsulated	none detected
MHI4708	H	3	present	unencapsulated	none detected
MHI4709	H	3	present	unencapsulated	none detected
MHI4710	H	3	present	unencapsulated	none detected
MHI4711	H	3	present	unencapsulated	none detected
MHI4712	H	3	present	unencapsulated	none detected
MHI4713	H	3	present	unencapsulated	none detected
MHI4714	H	3	present	unencapsulated	none detected
MHI4715	H	3	present	unencapsulated	none detected
MHI4716	H	3	present	unencapsulated	none detected
MHI4717	H	3	present	unencapsulated	none detected
MHI4718	I	1927	present	unencapsulated	none detected
MHI4719	I	1927	present	unencapsulated	none detected
MHI4720	I	1927	present	unencapsulated	none detected
MHI4721	I	1927	present	unencapsulated	none detected

MHI4722	I	1927	present	unencapsulated	none detected
MHI4723	I	1927	present	unencapsulated	none detected
MHI4724	I	1927	present	unencapsulated	none detected
MHI4725	I	1927	present	unencapsulated	none detected
MHI4726	I	1927	present	unencapsulated	none detected
MHI4727	I	1927	present	unencapsulated	none detected
MHI4728	I	1927	present	unencapsulated	none detected
MHI4729	I	1927	present	unencapsulated	none detected
MHI4730	I	1927	present	unencapsulated	none detected
MHI4731	I	1927	present	unencapsulated	none detected
MHI4732	I	1927	present	unencapsulated	none detected
MHI4733	I	1927	present	unencapsulated	none detected
MHI4734	I	1927	present	unencapsulated	none detected
MHI4735	I	1927	present	unencapsulated	none detected
MHI4736	I	1927	present	unencapsulated	none detected
MHI4737	I	1927	present	unencapsulated	none detected
MHI4738	I	1927	present	unencapsulated	none detected
MHI4739	I	1927	present	unencapsulated	none detected
MHI4740	I	1927	present	unencapsulated	none detected
MHI4741	I	1927	present	unencapsulated	none detected
MHI4742	I	1927	present	unencapsulated	none detected
MHI4743	I	1927	present	unencapsulated	none detected
MHI4744	I	1927	present	unencapsulated	none detected
MHI4745	I	1927	present	unencapsulated	none detected
MHI4746	I	1927	present	unencapsulated	none detected
MHI4747	I	1927	present	unencapsulated	none detected
MHI4748	I	1927	present	unencapsulated	none detected
MHI4749	I	1927	present	unencapsulated	none detected
MHI4750	I	1927	present	unencapsulated	none detected
MHI4751	I	1927	present	unencapsulated	none detected
MHI4752	I	1927	present	unencapsulated	none detected
MHI4753	I	1927	present	unencapsulated	none detected
MHI4754	I	1927	present	unencapsulated	none detected
MHI4755	I	1927	present	unencapsulated	none detected
MHI4756	I	1927	present	unencapsulated	none detected
MHI4757	I	1927	present	unencapsulated	none detected
MHI4758	I	1927	present	unencapsulated	none detected
MHI4759	I	1927	present	unencapsulated	none detected
MHI4760	I	1927	present	unencapsulated	none detected
MHI4761	I	1927	present	unencapsulated	none detected
MHI4762	I	1927	present	unencapsulated	none detected
MHI4763	I	1927	present	unencapsulated	none detected
MHI4764	I	1927	present	unencapsulated	none detected

MHI4765	I	1927	present	unencapsulated	none detected
MHI4766	I	1927	present	unencapsulated	none detected
MHI4767	I	1927	present	unencapsulated	none detected
MHI4768	I	1927	present	unencapsulated	none detected
MHI4769	I	1927	present	unencapsulated	none detected
MHI4770	I	1927	present	unencapsulated	none detected
MHI4771	I	1927	present	unencapsulated	none detected
MHI4772	I	266	present	unencapsulated	none detected
MHI4773	I	266	present	unencapsulated	none detected
MHI4774	I	266	present	unencapsulated	none detected
MHI4775	I	266	present	unencapsulated	none detected
MHI4776	I	266	present	unencapsulated	none detected
MHI4777	I	266	present	unencapsulated	none detected
MHI4778	I	266	present	unencapsulated	none detected
MHI4779	I	266	present	unencapsulated	none detected
MHI4780	I	266	present	unencapsulated	none detected
MHI4781	I	266	present	unencapsulated	none detected
MHI4782	I	266	present	unencapsulated	none detected
MHI4783	I	266	present	unencapsulated	none detected
MHI4784	I	266	present	unencapsulated	none detected
MHI4785	I	266	present	unencapsulated	none detected
MHI4786	I	266	present	unencapsulated	none detected
MHI4787	I	266	present	unencapsulated	none detected
MHI4788	I	266	present	unencapsulated	none detected
MHI4790	I	266	present	unencapsulated	none detected
MHI4791	I	266	present	unencapsulated	none detected
MHI4792	I	266	present	unencapsulated	none detected
MHI4793	I	266	present	unencapsulated	none detected
MHI4794	I	266	present	unencapsulated	none detected
MHI4795	I	266	present	unencapsulated	none detected
MHI4796	I	266	present	unencapsulated	none detected
MHI4797	I	266	present	unencapsulated	none detected
MHI4798	I	266	present	unencapsulated	none detected
MHI4799	I	266	present	unencapsulated	none detected
MHI4800	I	266	present	unencapsulated	none detected
MHI4801	I	266	present	unencapsulated	none detected
MHI4802	I	266	present	unencapsulated	none detected
MHI4803	I	266	present	unencapsulated	none detected
MHI4804	I	266	present	unencapsulated	none detected
MHI4805	I	266	present	unencapsulated	none detected
MHI4806	I	266	present	unencapsulated	none detected
MHI4807	I	266	present	unencapsulated	none detected
MHI4808	I	266	present	unencapsulated	none detected

MHI4809	I	266	present	unencapsulated	none detected
MHI4810	I	266	present	unencapsulated	none detected
MHI4811	I	266	present	unencapsulated	none detected
MHI4812	J	583	present	unencapsulated	none detected
MHI4813	J	583	present	unencapsulated	none detected
MHI4814	J	99	present	unencapsulated	none detected
MHI4815	J	583	present	unencapsulated	none detected
MHI4816	J	583	present	unencapsulated	none detected
MHI4817	J	583	present	unencapsulated	none detected
MHI4818	J	583	present	unencapsulated	none detected
MHI4819	J	583	present	unencapsulated	none detected
MHI4820	J	583	present	unencapsulated	none detected
MHI4821	J	583	present	unencapsulated	none detected
MHI4822	J	583	present	unencapsulated	none detected
MHI4823	J	583	present	unencapsulated	none detected
MHI4824	J	583	present	unencapsulated	none detected
MHI4825	J	583	present	unencapsulated	none detected
MHI4826	J	99	present	unencapsulated	none detected
MHI4827	J	583	present	unencapsulated	none detected
MHI4828	J	583	present	unencapsulated	none detected
MHI4829	J	99	present	unencapsulated	none detected
MHI4830	J	583	present	unencapsulated	none detected
MHI4832	J	583	present	unencapsulated	none detected
MHI4833	J	99	present	unencapsulated	none detected
MHI4834	J	583	present	unencapsulated	none detected
MHI4835	J	99	present	unencapsulated	none detected
MHI4836	J	583	present	unencapsulated	none detected
MHI4837	J	583	present	unencapsulated	none detected
MHI4838	J	99	present	unencapsulated	none detected
MHI4839	J	583	present	unencapsulated	none detected
MHI4840	J	583	present	unencapsulated	none detected
MHI4841	J	583	present	unencapsulated	none detected
MHI4842	J	99	present	unencapsulated	none detected
MHI4844	J	583	present	unencapsulated	none detected
MHI4845	J	99	present	unencapsulated	none detected
MHI4846	J	583	present	unencapsulated	none detected
MHI4847	J	583	present	unencapsulated	none detected
MHI4848	J	99	present	unencapsulated	none detected
MHI4849	J	583	present	unencapsulated	none detected
MHI4850	J	583	present	unencapsulated	none detected
MHI4851	I	266	present	unencapsulated	none detected
MHI4852	I	266	present	unencapsulated	none detected
MHI4853	I	266	present	unencapsulated	none detected

MHI4854	I	266	present	unencapsulated	none detected
MHI4856	I	266	present	unencapsulated	none detected
MHI4857	I	266	present	unencapsulated	none detected
MHI4858	I	266	present	unencapsulated	none detected
MHI4859	I	266	present	unencapsulated	none detected
MHI4860	I	266	present	unencapsulated	none detected
MHI4861	I	266	present	unencapsulated	none detected
MHI4862	I	266	present	unencapsulated	none detected
MHI4863	I	266	present	unencapsulated	none detected
MHI4864	I	266	present	unencapsulated	none detected
MHI4865	I	266	present	unencapsulated	none detected
MHI4866	I	266	present	unencapsulated	none detected
MHI4867	I	266	present	unencapsulated	none detected
MHI4868	I	266	present	unencapsulated	none detected
MHI4869	I	266	present	unencapsulated	none detected
MHI4870	I	266	present	unencapsulated	none detected
MHI4871	I	266	present	unencapsulated	none detected
MHI4872	I	266	present	unencapsulated	none detected
MHI4873	I	266	present	unencapsulated	none detected
MHI4874	I	266	present	unencapsulated	none detected
MHI4875	I	266	present	unencapsulated	none detected
MHI4876	I	266	present	unencapsulated	none detected
MHI4877	I	266	present	unencapsulated	none detected
MHI4878	I	266	present	unencapsulated	none detected
MHI4879	I	266	present	unencapsulated	none detected
MHI4880	I	266	present	unencapsulated	none detected
MHI4881	I	266	present	unencapsulated	none detected
MHI4882	I	266	present	unencapsulated	none detected
MHI4883	I	266	present	unencapsulated	none detected
MHI4884	I	266	present	unencapsulated	none detected
MHI4885	I	266	present	unencapsulated	none detected
MHI4886	I	266	present	unencapsulated	none detected
MHI4887	I	266	present	unencapsulated	none detected
MHI4888	I	266	present	unencapsulated	none detected
MHI4890	K	590	present	unencapsulated	none detected
MHI4891	K	590	present	unencapsulated	none detected
MHI4892	K	590	present	unencapsulated	none detected
MHI4893	K	590	present	unencapsulated	none detected
MHI4894	K	590	present	unencapsulated	none detected
MHI4895	K	590	present	unencapsulated	none detected
MHI4896	K	590	present	unencapsulated	none detected
MHI4897	K	590	present	unencapsulated	none detected
MHI4898	K	590	present	unencapsulated	none detected

MHI4899	K	590	present	unencapsulated	none detected
MHI4900	K	590	present	unencapsulated	none detected
MHI4901	K	590	present	unencapsulated	none detected
MHI4902	K	590	present	unencapsulated	none detected
MHI4903	K	590	present	unencapsulated	none detected
MHI4904	K	590	present	unencapsulated	none detected
MHI4905	K	590	present	unencapsulated	none detected
MHI4906	K	590	present	unencapsulated	none detected
MHI4907	K	590	present	unencapsulated	none detected
MHI4908	K	590	present	unencapsulated	none detected
MHI4909	K	590	present	unencapsulated	none detected
MHI4910	K	590	present	unencapsulated	none detected
MHI4911	K	590	present	unencapsulated	none detected
MHI4912	K	590	present	unencapsulated	none detected
MHI4913	K	590	present	unencapsulated	none detected
MHI4914	K	590	present	unencapsulated	none detected
MHI4915	K	590	present	unencapsulated	none detected
MHI4916	K	590	present	unencapsulated	none detected
MHI4917	K	590	present	unencapsulated	none detected
MHI4918	K	590	present	unencapsulated	none detected
MHI4919	K	590	present	unencapsulated	none detected
MHI4920	K	590	present	unencapsulated	none detected
MHI4921	K	590	present	unencapsulated	none detected
MHI4922	K	590	present	unencapsulated	none detected
MHI4923	K	590	present	unencapsulated	none detected
MHI4924	K	590	present	unencapsulated	none detected
MHI4925	K	590	present	unencapsulated	none detected
MHI4926	K	590	present	unencapsulated	none detected
MHI4927	K	590	present	unencapsulated	none detected
MHI4928	K	590	present	unencapsulated	none detected

n with otitis media.

AMR Genes
none detected
none detected
none detected
none detected
none detected
none detected
none detected
none detected
none detected
none detected
none detected
none detected
none detected
none detected
none detected
none detected
none detected
none detected
none detected
none detected
none detected
none detected
none detected
none detected
none detected
none detected
none detected
none detected
none detected
none detected
none detected
none detected
none detected
none detected
none detected
none detected
none detected
none detected
none detected
none detected

none detected
none detected
none detected
none detected
none detected
none detected
none detected
none detected
none detected
none detected
none detected
none detected
none detected
none detected
none detected
none detected
none detected
none detected
none detected
none detected
none detected
none detected
none detected
none detected
none detected
none detected
none detected
none detected
none detected
none detected
none detected
none detected
none detected
none detected
none detected
none detected
none detected
none detected
none detected
none detected
none detected
none detected
none detected

none detected
none detected
none detected
none detected
none detected
none detected
none detected
none detected
none detected
none detected
none detected
none detected
none detected
none detected
none detected
none detected
none detected
none detected
none detected
none detected
none detected
none detected
none detected
none detected
none detected
none detected
none detected
none detected
none detected
none detected
none detected
none detected
none detected
none detected
none detected
none detected
none detected
none detected
none detected
none detected
none detected
none detected
none detected

none detected
none detected
none detected
none detected
none detected
none detected
none detected
none detected
none detected
none detected
none detected
none detected
none detected
none detected
none detected
none detected
none detected
none detected
none detected
none detected
none detected
none detected
none detected
none detected
none detected
none detected
none detected
none detected
none detected
none detected
none detected
none detected
none detected
none detected
none detected
none detected
none detected
none detected
none detected
none detected
none detected
none detected
none detected

none detected
none detected
none detected
none detected
none detected
none detected
none detected
none detected
none detected
none detected
none detected
none detected
none detected
none detected
none detected
none detected
none detected
none detected
none detected
none detected
none detected
none detected
none detected
none detected
none detected
none detected
none detected
none detected
none detected
none detected
none detected
none detected
none detected
none detected
none detected
none detected
none detected
none detected
none detected
none detected
none detected
none detected
none detected

none detected
none detected
none detected
none detected
none detected
none detected
none detected
none detected
none detected
none detected
none detected
none detected
none detected
none detected
none detected
none detected
none detected
none detected
none detected
none detected
none detected
none detected
none detected
none detected
none detected
none detected
none detected
none detected
none detected
none detected
none detected
none detected
none detected
none detected
none detected
none detected
none detected
none detected
none detected
none detected
none detected
none detected
none detected

none detected
none detected
none detected
none detected
none detected
none detected
none detected
none detected
none detected
none detected
none detected
none detected
none detected
none detected
none detected
none detected
none detected
none detected
none detected
none detected
none detected
none detected
none detected
none detected
none detected
none detected
none detected
none detected
none detected
none detected
none detected
none detected
none detected
none detected
none detected
none detected
none detected
none detected
none detected
none detected
none detected
none detected
none detected

none detected
none detected
none detected
none detected
none detected
none detected
none detected
none detected
none detected
none detected
none detected
none detected
none detected
none detected
none detected
none detected
none detected
none detected
none detected
none detected
none detected
none detected
none detected
none detected
none detected
none detected
none detected
none detected
none detected
none detected
none detected
none detected
none detected
none detected
none detected
none detected
none detected
none detected
none detected
none detected
none detected
none detected
none detected

none detected
none detected
none detected
none detected
none detected
none detected
none detected
none detected
none detected
none detected
none detected
none detected
none detected
none detected
none detected
none detected
none detected
none detected
none detected
none detected
none detected
none detected
none detected
none detected
none detected
none detected
none detected
none detected
none detected
none detected
none detected
none detected
none detected
none detected
none detected
none detected
none detected
none detected
none detected
none detected
none detected
none detected
none detected

none detected
none detected
none detected
none detected
none detected
TEM-1D_Bla
none detected
none detected
none detected
none detected
none detected
none detected
none detected
none detected
none detected
none detected
none detected
TEM-1D_Bla
none detected
none detected
TEM-1D_Bla
TEM-1D_Bla
none detected
TEM-1D_Bla
TEM-1D_Bla
TEM-1D_Bla
TEM-1D_Bla
TEM-1D_Bla
TEM-1D_Bla
none detected
none detected
none detected
TEM-1D_Bla
none detected
TEM-1D_Bla
none detected
none detected
TEM-1D_Bla
none detected
TEM-1D_Bla
none detected
none detected
none detected

none detected
none detected
TEM-1D_Bla
none detected
none detected
none detected
none detected
TEM-1D_Bla
none detected
none detected
none detected
none detected
none detected
none detected
none detected
none detected
none detected
none detected
none detected
none detected
none detected
none detected
none detected
none detected
none detected
none detected
none detected
none detected
none detected
none detected
none detected
none detected
none detected
Aph3''Ia_AGly, TEM-1D_Bla
Aph3''Ia_AGly, TEM-1D_Bla
Aph3''Ia_AGly, TEM-1D_Bla
Aph3''Ia_AGly, TEM-1D_Bla
Aph3''Ia_AGly, TEM-1D_Bla
Aph3''Ia_AGly, TEM-1D_Bla
Aph3''Ia_AGly, TEM-1D_Bla
Aph3''Ia_AGly, TEM-1D_Bla
Aph3''Ia_AGly, TEM-1D_Bla

Aph3''Ia_AGly, TEM-1D_Bla
Aph3''Ia_AGly, TEM-1D_Bla
Aph3''Ia_AGly, TEM-1D_Bla
Aph3''Ia_AGly, TEM-1D_Bla
Aph3''Ia_AGly, TEM-1D_Bla
Aph3''Ia_AGly, TEM-1D_Bla
Aph3''Ia_AGly, TEM-1D_Bla
Aph3''Ia_AGly, TEM-1D_Bla
Aph3''Ia_AGly, TEM-1D_Bla
Aph3''Ia_AGly, TEM-1D_Bla
Aph3''Ia_AGly, TEM-1D_Bla
Aph3''Ia_AGly, TEM-1D_Bla
Aph3''Ia_AGly, TEM-1D_Bla
Aph3''Ia_AGly, TEM-1D_Bla
Aph3''Ia_AGly, TEM-1D_Bla
Aph3''Ia_AGly, TEM-1D_Bla
Aph3''Ia_AGly, TEM-1D_Bla
Aph3''Ia_AGly, TEM-1D_Bla
Aph3''Ia_AGly, TEM-1D_Bla
Aph3''Ia_AGly, TEM-1D_Bla
Aph3''Ia_AGly, TEM-1D_Bla
Aph3''Ia_AGly, TEM-1D_Bla
Aph3''Ia_AGly, TEM-1D_Bla
Aph3''Ia_AGly, TEM-1D_Bla
Aph3''Ia_AGly, TEM-1D_Bla
Aph3''Ia_AGly, TEM-1D_Bla
Aph3''Ia_AGly, TEM-1D_Bla
Aph3''Ia_AGly, TEM-1D_Bla
Aph3''Ia_AGly, TEM-1D_Bla
Aph3''Ia_AGly, TEM-1D_Bla

NF*	171	3	16	14	30	15*
266	3	18	53	15	86	14

21
23
