# Supplementary material for: Intra-host genomic variation of serologically nontypeable Haemophilus influenzae isolates from otitis media
Source: Microbiol Spectr. 2025 Mar 31;13(5):e03089-24. doi: 10.1128/spectrum.03089-24 (PMC12053901; doi:10.1128/spectrum.03089-24)
Supplement: Table S2 — Cohort of 99 publicly available H. influenzae genomes. [file spectrum.03089-24-s0002.pdf]

Table S2. Cohort of 99 publically available *H. influenzae* genomes.

Assembly Accession	Assembly Name	MLST	Bases
GCA_000012185.1	ASM1218v1	33	1,914,490
GCA_000016465.1	ASM1646v1	107	1,813,033
GCA_000165525.1	ASM16552v1	99	1,932,306
GCA_000165575.1	ASM16557v1	1622	1,819,370
GCA_000197875.1	ASM19787v1	65	1,985,832
GCA_000200475.1	ASM20047v1	70	2,007,018
GCA_000210875.1	ASM21087v1	6	1,981,535
GCA_001856725.1	ASM185672v1	23	1,829,217
GCA_002073475.1	ASM207347v2	47	1,830,681
GCA_002966575.1	ASM296657v1	155	1,857,175
GCA_002966595.1	ASM296659v1	155	1,857,048
GCA_002966615.1	ASM296661v1	103	1,886,450
GCA_002966635.1	ASM296663v1	103	1,886,411
GCA_002966655.1	ASM296665v1	156	1,823,096
GCA_002966675.1	ASM296667v1	156	1,823,172
GCA_002966695.1	ASM296669v1	155	1,901,558
GCA_002966715.1	ASM296671v1	155	1,901,533
GCA_002966735.1	ASM296673v1	159	1,813,865
GCA_002988215.1	ASM298821v2	107	1,812,418
GCA_002988885.1	ASM298888v2	107	1,812,134
GCA_003184385.1	ASM318438v1	1327	2,047,595
GCA_003184405.1	ASM318440v1	263	2,025,527
GCA_013394405.1	ASM1339440v1	422	1,957,393
GCA_014701215.1	ASM1470121v1	1511	1,809,645
GCA_014931495.1	ASM1493149v1	2592	1,894,676
GCA_016861285.1	ASM1686128v1	56	1,852,308
GCA_019703365.1	ASM1970336v1	3	1,979,718
GCA_019703385.1	ASM1970338v1	3	1,979,786
GCA_019703405.1	ASM1970340v1	n.d.	1,853,393
GCA_019703425.1	ASM1970342v1	436	1,832,515
GCA_019703455.1	ASM1970345v1	14	1,805,925
GCA_019703495.1	ASM1970349v1	160	1,819,364
GCA_019703525.1	ASM1970352v1	1017	1,886,480
GCA_019703545.1	ASM1970354v1	1683	1,902,536
GCA_019703575.1	ASM1970357v1	155	1,816,076
GCA_019703595.1	ASM1970359v1	159	1,914,722

GCA_019703615.1	ASM1970361v1	107	1,810,977
GCA_019703635.1	ASM1970363v1	396	1,818,266
GCA_019703655.1	ASM1970365v1	156	1,822,115
GCA_019703675.1	ASM1970367v1	1736	1,884,503
GCA_019703695.1	ASM1970369v1	1444	1,911,890
GCA_019703715.1	ASM1970371v1	393	1,791,343
GCA_019703735.1	ASM1970373v1	1782	1,882,642
GCA_019703755.1	ASM1970375v1	1434	1,953,394
GCA_019703775.1	ASM1970377v1	584	1,790,924
GCA_019703795.1	ASM1970379v1	187	1,936,686
GCA_019930705.1	ASM1993070v1	44	1,902,597
GCA_020736025.1	ASM2073602v1	123	1,834,285
GCA_020736045.1	ASM2073604v1	3	1,890,662
GCA_028535035.1	ASM2853503v1	n.d.	1,812,138
GCA_900475535.1	44310_E01	559	2,044,007
GCA_900475755.1	45214_B02	123	1,834,484
GCA_900475995.1	47555_F02	145	1,850,791
GCA_900478275.1	34211_D02	44	1,890,469
GCA_900478325.1	33962_G01	142	1,915,356
GCA_900478735.1	33763_D01	27	1,865,137
GCA_900635795.1	33962_B02	6	1,860,106
GCA_900635805.1	34211_C02	368	1,879,945
GCA_900638105.1	56433_A01	478	1,948,880
GCA_901472485.1	35860_G01	464	1,876,886
GCF_000465255.1	ASM46525v1	124	1,856,176
GCF_000698365.1	ASM69836v1	712	1,811,802
GCF_000767075.1	ASM76707v1	3	1,850,897
GCF_000931575.1	ASM93157v1	1	1,846,259
GCF_000931605.1	ASM93160v1	244	1,846,503
GCF_000931625.1	ASM93162v1	14	1,887,620
GCF_000968335.1	ASM96833v1	321	1,969,659
GCF_001457655.1	NCTC8143	3	1,890,645
GCF_003351425.1	ASM335142v1	62	1,817,261
GCF_003351445.1	ASM335144v1	139	1,860,196
GCF_003351465.1	ASM335146v1	9	1,816,295
GCF_003351585.1	ASM335158v1	56	1,848,871
GCF_003351605.1	ASM335160v1	7	1,804,746
GCF_003352345.1	ASM335234v1	2449	1,914,782
GCF_003352365.1	ASM335236v1	66	1,887,933

GCF_003352405.1	ASM335240v1	18	1,919,901
GCF_003425445.1	ASM342544v1	1025	1,838,740
GCF_003425465.1	ASM342546v1	2022	1,833,710
GCF_003425485.1	ASM342548v1	485	1,811,303
GCF_003425505.1	ASM342550v1	3	2,013,003
GCF_003425525.1	ASM342552v1	145	1,897,311
GCF_003425565.1	ASM342556v1	1069	1,858,634
GCF_003425585.1	ASM342558v1	12	1,840,498
GCF_003425605.1	ASM342560v1	1069	1,858,630
GCF_003425625.1	ASM342562v1	1789	1,833,305
GCF_003425645.1	ASM342564v1	474	1,908,143
GCF_003425715.1	ASM342571v1	12	1,848,210
GCF_003425765.1	ASM342576v1	2022	1,833,864
GCF_003425815.1	ASM342581v1	1025	1,840,062
GCF_003425935.1	ASM342593v1	145	1,849,483
GCF_003425955.1	ASM342595v1	46	1,904,311
GCF_008586745.1	ASM858674v1	65	1,985,844
GCF_008586765.1	ASM858676v1	n.d.	2,000,194
GCF_008586785.1	ASM858678v1	n.d.	1,877,864
GCF_008586805.1	ASM858680v1	65	1,987,687
GCF_008586825.1	ASM858682v1	65	1,984,979
GCF_008831525.1	ASM883152v1	43	1,887,343
GCF_919946725.1	KRLund_NTHi_Assembly_Genome271	14	1,805,497
GCF_919949215.1	KRLund_NTHi_Assembly_Genome3655	411	1,917,048

n.d.=not defined

Genes	Release Date
1,899	08/30/07
1,695	06/07/07
1,892	10/22/10
1,744	10/25/10
1,810	01/11/11
1,820	01/11/11
1,914	07/23/10
1,781	10/31/16
1,823	02/23/18
1,850	03/02/18
1,851	03/02/18
1,922	03/02/18
1,932	03/02/18
1,818	03/02/18
1,818	03/02/18
1,908	03/02/18
1,911	03/02/18
1,799	03/02/18
1,777	02/06/23
1,772	02/06/23
2,079	06/04/18
2,115	06/04/18
2,003	06/09/20
1,773	09/15/20
1,878	10/26/20
1,842	01/28/21
2,002	05/22/21
2,002	05/22/21
1,825	05/22/21
1,806	05/22/21
1,769	05/22/21
1,783	05/22/21
1,883	05/22/21
1,909	05/22/21
1,760	05/22/21
1,899	05/22/21

1,753	05/22/21
1,780	05/22/21
1,779	05/22/21
1,879	05/22/21
1,929	05/22/21
1,749	05/22/21
1,866	05/22/21
1,949	05/22/21
1,768	05/22/21
1,941	05/22/21
1,899	09/12/21
1,789	11/03/21
1,896	11/03/21
1,775	02/07/23
2,092	06/17/18
1,772	06/17/18
1,808	06/17/18
1,861	06/18/18
1,938	06/18/18
1,817	06/18/18
1,828	12/20/18
1,831	12/20/18
1,944	12/20/18
1,840	05/11/19
1,812	09/09/13
1,773	06/05/14
1,842	10/16/14
1,834	02/23/15
1,813	02/23/15
1,899	02/23/15
2,009	04/02/15
1,890	03/22/15
1,761	08/01/18
1,850	08/01/18
1,783	08/01/18
1,846	08/01/18
1,772	08/01/18
1,895	08/01/18
1,862	08/01/18

1,922	08/01/18
1,820	08/23/18
1,817	08/23/18
1,767	08/23/18
2,061	08/23/18
1,905	08/23/18
1,839	08/23/18
1,817	08/23/18
1,839	08/23/18
1,799	08/23/18
1,941	08/23/18
1,832	08/23/18
1,819	08/23/18
1,824	08/23/18
1,836	08/23/18
1,903	08/23/18
2,024	09/19/19
2,038	09/19/19
1,896	09/19/19
2,030	09/19/19
2,024	09/19/19
1,879	10/07/19
1,785	12/03/21
1,928	12/03/21
