# Supplementary material for: Intra-host genomic variation of serologically nontypeable Haemophilus influenzae isolates from otitis media
Source: Microbiol Spectr. 2025 Mar 31;13(5):e03089-24. doi: 10.1128/spectrum.03089-24 (PMC12053901; doi:10.1128/spectrum.03089-24)
Supplement: Table S3 — Hypothetical genes with a nonsynonymous or nonsense polymorphism. [file spectrum.03089-24-s0003.pdf]

Table S3. Hypothetical genes with a nonsynonymous or nonsense polymorphism.

Reference genome	Locus tag	Position in genome	Position in gene
MHI-4419	MHI-4419-pc__00609	621136	928
MHI-4419	MHI-4419-pc__00838	873910	952
MHI-4419	MHI-4419-pc__00838	873918	960
MHI-4419	MHI-4419-pc__00890	926153	4060
MHI-4419	MHI-4419-pc__00890	926197	4016
MHI-4419	MHI-4419-pc__00890	926519	3694
MHI-4419	MHI-4419-pc__00890	926885	3328
MHI-4419	MHI-4419-pc__00890	926929	3284
MHI-4419	MHI-4419-pc__00890	927110	3103
MHI-4419	MHI-4419-pc__01044	1094957	148
MHI-4419	MHI-4419-pc__01086	1130548	520
MHI-4419	MHI-4419-pc__01087	1132524	195
MHI-4419	MHI-4419-pc__01107	1152133	641
MHI-4419	MHI-4419-pc__01107	1152160	614
MHI-4419	MHI-4419-pc__01107	1152293	481
MHI-4419	MHI-4419-pc__01107	1152320	454
MHI-4419	MHI-4419-pc__01107	1152358	416
MHI-4419	MHI-4419-pc__01107	1152376	398
MHI-4419	MHI-4419-pc__01107	1152377	397
MHI-4419	MHI-4419-pc__01107	1152404	370
MHI-4419	MHI-4419-pc__01107	1152431	343
MHI-4419	MHI-4419-pc__01107	1152511	263
MHI-4419	MHI-4419-pc__01107	1152516	258
MHI-4419	MHI-4419-pc__01107	1152520	254
MHI-4419	MHI-4419-pc__01420	1443711	261
MHI-4419	MHI-4419-pc__01420	1443713	259
MHI-4419	MHI-4419-pc__01420	1443718	254
MHI-4419	MHI-4419-pc__01420	1443730	242
MHI-4419	MHI-4419-pc__01420	1443731	241
MHI-4419	MHI-4419-pc__01420	1443739	233
MHI-4419	MHI-4419-pc__01420	1443892	80
MHI-4419	MHI-4419-pc__01420	1443904	68
MHI-4419	MHI-4419-pc__01420	1443907	65
MHI-4419	MHI-4419-pc__01420	1443917	55
MHI-4419	MHI-4419-pc__01420	1443928	44
MHI-4419	MHI-4419-pc__01420	1443957	15
MHI-4419	MHI-4419-pc__01648	1667720	886
MHI-4419	MHI-4419-pc__01648	1667727	879

MHI-4419	MHI-4419-pc__01648	1667788	818
MHI-4419	MHI-4419-pc__01648	1667798	808
MHI-4419	MHI-4419-pc__01648	1668320	286
MHI-4419	MHI-4419-pc__01648	1668363	243
MHI-4419	MHI-4419-pc__01648	1668367	239
MHI-4419	MHI-4419-pc__01654	1674678	69
MHI-4419	MHI-4419-pc__01655	1675600	193
MHI-4419	MHI-4419-pc__01655	1675610	203
MHI-4419	MHI-4419-pc__01655	1675618	211
MHI-4419	MHI-4419-pc__01655	1675630	223
MHI-4466	MHI-4466__00256	271788	73
MHI-4466	MHI-4466__00283	298618	4
MHI-4466	MHI-4466__00585	575212	1134
MHI-4466	MHI-4466__00932	971999	4060
MHI-4466	MHI-4466__00932	972590	3469
MHI-4466	MHI-4466__00932	972956	3103
MHI-4466	MHI-4466__01121	1174709	252
MHI-4466	MHI-4466__01412	1445556	222
MHI-4466	MHI-4466__01413	1445556	20
MHI-4466	MHI-4466__01413	1445559	23
MHI-4466	MHI-4466__01412	1445565	231
MHI-4466	MHI-4466__01413	1445565	29
MHI-4466	MHI-4466__01442	1476747	4
MHI-4466	MHI-4466__01733	1766127	235
MHI-4466	MHI-4466__01733	1766143	251
MHI-4466	MHI-4466__01733	1766151	259
MHI-4466	MHI-4466__01733	1766161	269
MHI-4466	MHI-4466__01733	1766176	284
MHI-4466	MHI-4466__01733	1766196	304
MHI-4506	MHI-4506__00032	33766	1426
MHI-4506	MHI-4506__00032	34720	472
MHI-4506	MHI-4506__00307	321702	748
MHI-4506	MHI-4506__00307	321819	631
MHI-4506	MHI-4506__00307	322245	205
MHI-4506	MHI-4506__00424	425554	688
MHI-4506	MHI-4506__00424	425571	671
MHI-4506	MHI-4506__00424	425608	634
MHI-4506	MHI-4506__00424	425631	611
MHI-4506	MHI-4506__00424	425635	607
MHI-4506	MHI-4506__00424	425851	391
MHI-4506	MHI-4506__00424	425874	368
MHI-4506	MHI-4506__00424	425878	364
MHI-4506	MHI-4506__00567	568087	1070

MHI-4506	MHI-4506__00567	568109	1092
MHI-4506	MHI-4506__00826	849569	1079
MHI-4506	MHI-4506__01334	1373932	115
MHI-4506	MHI-4506__01334	1374023	206
MHI-4506	MHI-4506__01335	1374023	4
MHI-4506	MHI-4506__01334	1374038	221
MHI-4506	MHI-4506__01335	1374038	19
MHI-4506	MHI-4506__01334	1374041	224
MHI-4506	MHI-4506__01335	1374041	22
MHI-4506	MHI-4506__01334	1374041	224
MHI-4506	MHI-4506__01335	1374041	22
MHI-4506	MHI-4506__01334	1374043	226
MHI-4506	MHI-4506__01335	1374043	24
MHI-4506	MHI-4506__01334	1374048	231
MHI-4506	MHI-4506__01335	1374048	29
MHI-4506	MHI-4506__01670	1713300	223
MHI-4506	MHI-4506__01670	1713308	215
MHI-4506	MHI-4506__01670	1713311	212
MHI-4506	MHI-4506__01670	1713314	209
MHI-4506	MHI-4506__01670	1713316	207
MHI-4506	MHI-4506__01670	1713321	202
MHI-4506	MHI-4506__01790	1840670	1233
MHI-4506	MHI-4506__01790	1840679	1224
MHI-4506	MHI-4506__01790	1840692	1211
MHI-4506	MHI-4506__01790	1840692	1211
MHI-4537	MHI-4537__00037	37896	510
MHI-4537	MHI-4537__00040	41308	1369
MHI-4537	MHI-4537__00040	42081	596
MHI-4537	MHI-4537__00040	42285	392
MHI-4537	MHI-4537__00233	242445	143
MHI-4537	MHI-4537__00282	292741	954
MHI-4537	MHI-4537__00282	292748	947
MHI-4537	MHI-4537__00490	500892	1716
MHI-4537	MHI-4537__00490	502573	35
MHI-4537	MHI-4537__00590	615973	258
MHI-4537	MHI-4537__00638	668788	556
MHI-4537	MHI-4537__00808	859887	116
MHI-4537	MHI-4537__00808	859892	121
MHI-4537	MHI-4537__00808	859893	122
MHI-4537	MHI-4537__01282	1342267	753
MHI-4537	MHI-4537__01355	1413428	1366
MHI-4537	MHI-4537__01360	1419097	116
MHI-4537	MHI-4537__01360	1419138	75

MHI-4537	MHI-4537__01361	1419517	22
MHI-4537	MHI-4537__01361	1419535	4
MHI-4537	MHI-4537__01362	1420160	107
MHI-4537	MHI-4537__01362	1420188	135
MHI-4537	MHI-4537__01462	1535581	412
MHI-4537	MHI-4537__01621	1687114	218
MHI-4537	MHI-4537__01621	1687345	449
MHI-4537	MHI-4537__01621	1687740	844
MHI-4577	MHI-4577__00132	135570	292
MHI-4577	MHI-4577__00132	135572	290
MHI-4577	MHI-4577__00132	135578	284
MHI-4577	MHI-4577__00132	135587	275
MHI-4577	MHI-4577__00132	135591	271
MHI-4577	MHI-4577__00132	135593	269
MHI-4577	MHI-4577__00140	143002	328
MHI-4577	MHI-4577__00140	143035	361
MHI-4577	MHI-4577__00140	143038	364
MHI-4577	MHI-4577__00140	143039	365
MHI-4577	MHI-4577__00140	143041	367
MHI-4577	MHI-4577__00140	143044	370
MHI-4577	MHI-4577__00140	143056	382
MHI-4577	MHI-4577__00140	143122	448
MHI-4577	MHI-4577__00140	143345	671
MHI-4577	MHI-4577__00140	143353	679
MHI-4577	MHI-4577__00140	143377	703
MHI-4577	MHI-4577__00744	734109	122
MHI-4577	MHI-4577__00780	772810	518
MHI-4577	MHI-4577__01096	1064678	742
MHI-4577	MHI-4577__01320	1303504	622
MHI-4577	MHI-4577__01320	1303514	612
MHI-4577	MHI-4577__01320	1303531	595
MHI-4577	MHI-4577__01693	1683556	1079
MHI-4615	MHI-4615__00034	33907	560
MHI-4615	MHI-4615__00034	33911	556
MHI-4615	MHI-4615__00060	63579	417
MHI-4615	MHI-4615__00518	559229	1333
MHI-4615	MHI-4615__00518	559230	1334
MHI-4615	MHI-4615__00519	559633	395
MHI-4615	MHI-4615__00519	559689	451
MHI-4615	MHI-4615__00519	559781	543
MHI-4615	MHI-4615__00630	674647	589
MHI-4615	MHI-4615__00630	674653	583
MHI-4615	MHI-4615__00630	674662	574

MHI-4615	MHI-4615__00630	674695	541
MHI-4615	MHI-4615__00630	674703	533
MHI-4615	MHI-4615__01036	1098266	122
MHI-4615	MHI-4615__01094	1149785	374
MHI-4615	MHI-4615__01094	1149798	361
MHI-4615	MHI-4615__01094	1149816	343
MHI-4615	MHI-4615__01094	1149820	339
MHI-4615	MHI-4615__01629	1695996	92
MHI-4615	MHI-4615__01629	1696143	239
MHI-4664	MHI-4664__00632	637943	685
MHI-4664	MHI-4664__00632	637951	693
MHI-4664	MHI-4664__00632	637954	696
MHI-4664	MHI-4664__00632	638363	1105
MHI-4664	MHI-4664__00632	638365	1107
MHI-4664	MHI-4664__00632	638369	1111
MHI-4664	MHI-4664__00632	638486	1228
MHI-4664	MHI-4664__01330	1368899	1655
MHI-4664	MHI-4664__01334	1374080	265
MHI-4664	MHI-4664__01789	1840699	1228
MHI-4664	MHI-4664__01789	1840707	1220
MHI-4664	MHI-4664__01789	1840708	1219
MHI-4664	MHI-4664__01789	1840711	1216
MHI-4664	MHI-4664__01789	1840713	1214
MHI-4664	MHI-4664__01789	1840715	1212
MHI-4664	MHI-4664__01789	1840719	1208
MHI-4664	MHI-4664__01789	1840720	1207
MHI-4664	MHI-4664__01789	1840728	1199
MHI-4664	MHI-4664__01789	1840729	1198
MHI-4688	MHI-4688__00246	252496	611
MHI-4688	MHI-4688__00338	342381	988
MHI-4688	MHI-4688__00401	396978	28
MHI-4688	MHI-4688__00597	583786	299
MHI-4688	MHI-4688__00597	583829	256
MHI-4688	MHI-4688__00597	583852	233
MHI-4688	MHI-4688__00597	583927	158
MHI-4688	MHI-4688__00597	583930	155
MHI-4688	MHI-4688__00597	583947	138
MHI-4688	MHI-4688__00597	583948	137
MHI-4688	MHI-4688__00597	583952	133
MHI-4688	MHI-4688__00597	583956	129
MHI-4688	MHI-4688__00597	583958	127
MHI-4688	MHI-4688__00598	584124	2495
MHI-4688	MHI-4688__00598	584125	2494

MHI-4688	MHI-4688__00598	584127	2492
MHI-4688	MHI-4688__00598	584128	2491
MHI-4688	MHI-4688__00598	584131	2488
MHI-4688	MHI-4688__00598	584135	2484
MHI-4688	MHI-4688__00598	584144	2475
MHI-4688	MHI-4688__00598	584146	2473
MHI-4688	MHI-4688__00598	584161	2458
MHI-4688	MHI-4688__00598	584166	2453
MHI-4688	MHI-4688__00598	584172	2447
MHI-4688	MHI-4688__00598	584173	2446
MHI-4688	MHI-4688__00598	584185	2434
MHI-4688	MHI-4688__00598	584188	2431
MHI-4688	MHI-4688__00598	584190	2429
MHI-4688	MHI-4688__00598	584193	2426
MHI-4688	MHI-4688__00598	584199	2420
MHI-4688	MHI-4688__00598	584200	2419
MHI-4688	MHI-4688__00598	584206	2413
MHI-4688	MHI-4688__00598	584209	2410
MHI-4688	MHI-4688__00598	584211	2408
MHI-4688	MHI-4688__00598	584223	2396
MHI-4688	MHI-4688__00598	584232	2387
MHI-4688	MHI-4688__00598	584237	2382
MHI-4688	MHI-4688__00598	584239	2380
MHI-4688	MHI-4688__00598	584241	2378
MHI-4688	MHI-4688__00598	584244	2375
MHI-4688	MHI-4688__00598	584247	2372
MHI-4688	MHI-4688__00598	584248	2371
MHI-4688	MHI-4688__00598	584250	2369
MHI-4688	MHI-4688__00598	584251	2368
MHI-4688	MHI-4688__00598	584252	2367
MHI-4688	MHI-4688__01005	1032310	793
MHI-4688	MHI-4688__01213	1232471	1210
MHI-4772	MHI-4772__00041	42616	541
MHI-4772	MHI-4772__00041	42620	537
MHI-4772	MHI-4772__00041	42677	480
MHI-4772	MHI-4772__00041	42686	471
MHI-4772	MHI-4772__00160	167623	348
MHI-4772	MHI-4772__00167	176179	550
MHI-4772	MHI-4772__00167	176200	529
MHI-4772	MHI-4772__00234	230169	742
MHI-4772	MHI-4772__00234	230184	757
MHI-4772	MHI-4772__00252	246115	1108
MHI-4772	MHI-4772__00313	304303	522

MHI-4772	MHI-4772__00346	338804	265
MHI-4772	MHI-4772__00346	338837	298
MHI-4772	MHI-4772__00346	339152	613
MHI-4772	MHI-4772__00346	339158	619
MHI-4772	MHI-4772__00346	339159	620
MHI-4772	MHI-4772__00347	339595	391
MHI-4772	MHI-4772__00347	339615	411
MHI-4772	MHI-4772__00347	339629	425
MHI-4772	MHI-4772__00372	363125	542
MHI-4772	MHI-4772__00417	409447	257
MHI-4772	MHI-4772__00417	409455	265
MHI-4772	MHI-4772__00527	496670	571
MHI-4772	MHI-4772__00536	509781	1933
MHI-4772	MHI-4772__00576	563715	26
MHI-4772	MHI-4772__00577	564343	87
MHI-4772	MHI-4772__00601	583477	429
MHI-4772	MHI-4772__00609	588365	2009
MHI-4772	MHI-4772__00664	645887	695
MHI-4772	MHI-4772__00664	645958	624
MHI-4772	MHI-4772__00664	646106	476
MHI-4772	MHI-4772__00664	646241	341
MHI-4772	MHI-4772__00664	646261	321
MHI-4772	MHI-4772__00664	646265	317
MHI-4772	MHI-4772__00664	646281	301
MHI-4772	MHI-4772__00664	646350	232
MHI-4772	MHI-4772__00664	646435	147
MHI-4772	MHI-4772__00664	646476	106
MHI-4772	MHI-4772__00725	718108	336
MHI-4772	MHI-4772__00734	727496	853
MHI-4772	MHI-4772__00744	736596	788
MHI-4772	MHI-4772__00744	736597	787
MHI-4772	MHI-4772__00969	977026	238
MHI-4772	MHI-4772__01005	1012647	686
MHI-4772	MHI-4772__01053	1061081	541
MHI-4772	MHI-4772__01053	1061085	545
MHI-4772	MHI-4772__01053	1061102	562
MHI-4772	MHI-4772__01053	1061115	575
MHI-4772	MHI-4772__01072	1081143	862
MHI-4772	MHI-4772__01114	1133466	575
MHI-4772	MHI-4772__01119	1138408	3
MHI-4772	MHI-4772__01128	1147002	290
MHI-4772	MHI-4772__01186	1208287	154
MHI-4772	MHI-4772__01186	1208319	122

MHI-4772	MHI-4772__01187	1208567	154
MHI-4772	MHI-4772__01187	1208599	122
MHI-4772	MHI-4772__01244	1259627	710
MHI-4772	MHI-4772__01244	1259985	352
MHI-4772	MHI-4772__01244	1260041	296
MHI-4772	MHI-4772__01284	1294255	1069
MHI-4772	MHI-4772__01329	1339432	22
MHI-4772	MHI-4772__01329	1339446	8
MHI-4772	MHI-4772__01429	1440893	800
MHI-4772	MHI-4772__01433	1444509	611
MHI-4772	MHI-4772__01504	1516163	1967
MHI-4772	MHI-4772__01504	1516170	1960
MHI-4772	MHI-4772__01504	1517886	244
MHI-4772	MHI-4772__01504	1517895	235
MHI-4772	MHI-4772__01536	1548519	28
MHI-4772	MHI-4772__01536	1548523	24
MHI-4772	MHI-4772__01588	1605551	220
MHI-4772	MHI-4772__01588	1605560	211
MHI-4772	MHI-4772__01589	1606566	26
MHI-4772	MHI-4772__01590	1607977	1849
MHI-4772	MHI-4772__01590	1608003	1823
MHI-4772	MHI-4772__01590	1608006	1820
MHI-4772	MHI-4772__01590	1608525	1301
MHI-4772	MHI-4772__01590	1608533	1293
MHI-4772	MHI-4772__01590	1608534	1292
MHI-4772	MHI-4772__01590	1608543	1283
MHI-4772	MHI-4772__01590	1608552	1274
MHI-4772	MHI-4772__01590	1608564	1262
MHI-4772	MHI-4772__01590	1608565	1261
MHI-4772	MHI-4772__01590	1608566	1260
MHI-4772	MHI-4772__01590	1608568	1258
MHI-4772	MHI-4772__01590	1608571	1255
MHI-4772	MHI-4772__01590	1608964	862
MHI-4772	MHI-4772__01590	1609004	822
MHI-4772	MHI-4772__01590	1609068	758
MHI-4772	MHI-4772__01590	1609071	755
MHI-4772	MHI-4772__01590	1609072	754
MHI-4772	MHI-4772__01590	1609074	752
MHI-4772	MHI-4772__01590	1609078	748
MHI-4772	MHI-4772__01590	1609089	737
MHI-4772	MHI-4772__01590	1609240	586
MHI-4772	MHI-4772__01814	1833407	1575
MHI-4772	MHI-4772__01814	1833420	1562

MHI-4772	MHI-4772__01814	1833445	1537
MHI-4772	MHI-4772__01814	1833479	1503
MHI-4772	MHI-4772__01814	1833483	1499
MHI-4772	MHI-4772__01814	1833745	1237
MHI-4772	MHI-4772__01814	1833808	1174
MHI-4772	MHI-4772__01814	1834417	565
MHI-4772	MHI-4772__01829	1849574	579
MHI-4718	MHI-4718__00008	8026	104
MHI-4718	MHI-4718__00157	172064	952
MHI-4718	MHI-4718__00238	253556	88
MHI-4718	MHI-4718__00300	325490	973
MHI-4718	MHI-4718__00300	325504	959
MHI-4718	MHI-4718__00300	325510	953
MHI-4718	MHI-4718__00300	325516	947
MHI-4718	MHI-4718__00300	325520	943
MHI-4718	MHI-4718__00300	325522	941
MHI-4718	MHI-4718__00300	325536	927
MHI-4718	MHI-4718__00300	325568	895
MHI-4718	MHI-4718__00300	325733	730
MHI-4718	MHI-4718__00300	325763	700
MHI-4718	MHI-4718__00300	325773	690
MHI-4718	MHI-4718__00300	325777	686
MHI-4718	MHI-4718__00300	325897	566
MHI-4718	MHI-4718__00338	365301	122
MHI-4718	MHI-4718__00339	365581	122
MHI-4718	MHI-4718__00397	416902	919
MHI-4718	MHI-4718__00397	416905	916
MHI-4718	MHI-4718__00397	416917	904
MHI-4718	MHI-4718__00397	416969	852
MHI-4718	MHI-4718__00397	416970	851
MHI-4718	MHI-4718__00397	416974	847
MHI-4718	MHI-4718__00397	417018	803
MHI-4718	MHI-4718__00436	451692	1054
MHI-4718	MHI-4718__00436	451699	1047
MHI-4718	MHI-4718__00436	451738	1008
MHI-4718	MHI-4718__00436	451740	1006
MHI-4718	MHI-4718__00755	788183	99
MHI-4718	MHI-4718__00756	790381	1753
MHI-4718	MHI-4718__00756	790389	1745
MHI-4718	MHI-4718__00756	790395	1739
MHI-4718	MHI-4718__00756	790413	1721
MHI-4718	MHI-4718__00756	790421	1713
MHI-4718	MHI-4718__00756	790423	1711

MHI-4718	MHI-4718__00756	790425	1709
MHI-4718	MHI-4718__00756	790519	1615
MHI-4718	MHI-4718__00756	790535	1599
MHI-4718	MHI-4718__00756	790536	1598
MHI-4718	MHI-4718__00756	790537	1597
MHI-4718	MHI-4718__00756	790557	1577
MHI-4718	MHI-4718__00756	790558	1576
MHI-4718	MHI-4718__00756	790642	1492
MHI-4718	MHI-4718__00756	791011	1123
MHI-4718	MHI-4718__00756	791026	1108
MHI-4718	MHI-4718__00756	791043	1091
MHI-4718	MHI-4718__00756	791044	1090
MHI-4718	MHI-4718__00756	791052	1082
MHI-4718	MHI-4718__00756	791053	1081
MHI-4718	MHI-4718__00756	791226	908
MHI-4718	MHI-4718__00756	791227	907
MHI-4718	MHI-4718__00756	791232	902
MHI-4718	MHI-4718__00756	791233	901
MHI-4718	MHI-4718__00756	791234	900
MHI-4718	MHI-4718__00756	791235	899
MHI-4718	MHI-4718__00756	791239	895
MHI-4718	MHI-4718__00756	791247	887
MHI-4718	MHI-4718__00756	791249	885
MHI-4718	MHI-4718__00756	791250	884
MHI-4718	MHI-4718__00756	791257	877
MHI-4718	MHI-4718__00770	807010	122
MHI-4718	MHI-4718__00770	807011	121
MHI-4718	MHI-4718__00770	807016	116
MHI-4718	MHI-4718__00918	950079	423
MHI-4718	MHI-4718__00918	950129	373
MHI-4718	MHI-4718__00918	950131	371
MHI-4718	MHI-4718__00924	956392	839
MHI-4718	MHI-4718__00926	957763	182
MHI-4718	MHI-4718__00926	957931	350
MHI-4718	MHI-4718__00926	957972	391
MHI-4718	MHI-4718__00926	958090	509
MHI-4718	MHI-4718__00926	958094	513
MHI-4718	MHI-4718__00926	958147	566
MHI-4718	MHI-4718__00948	981464	353
MHI-4718	MHI-4718__00986	1020997	1170
MHI-4718	MHI-4718__00986	1021006	1161
MHI-4718	MHI-4718__00986	1021014	1153
MHI-4718	MHI-4718__00986	1021046	1121

MHI-4718	MHI-4718__00986	1021068	1099
MHI-4718	MHI-4718__01086	1131180	1223
MHI-4718	MHI-4718__01086	1131204	1199
MHI-4718	MHI-4718__01086	1131237	1166
MHI-4718	MHI-4718__01388	1422266	903
MHI-4718	MHI-4718__01388	1422995	174
MHI-4718	MHI-4718__01491	1546142	8
MHI-4718	MHI-4718__01505	1554597	91
MHI-4718	MHI-4718__01577	1604014	480
MHI-4718	MHI-4718__01675	1691671	1860
MHI-4812	MHI-4812__00014	15533	246
MHI-4812	MHI-4812__00084	100808	356
MHI-4812	MHI-4812__00107	120745	79
MHI-4812	MHI-4812__00123	140723	194
MHI-4812	MHI-4812__00315	340521	400
MHI-4812	MHI-4812__00315	340689	568
MHI-4812	MHI-4812__00315	340713	592
MHI-4812	MHI-4812__00315	340729	608
MHI-4812	MHI-4812__00317	342541	737
MHI-4812	MHI-4812__00317	342543	739
MHI-4812	MHI-4812__00425	450657	546
MHI-4812	MHI-4812__00838	864405	16
MHI-4812	MHI-4812__00938	955862	262
MHI-4812	MHI-4812__00938	956092	32
MHI-4812	MHI-4812__01226	1250303	238
MHI-4812	MHI-4812__01471	1495830	216
MHI-4812	MHI-4812__01471	1495838	224
MHI-4812	MHI-4812__01472	1496134	278
MHI-4812	MHI-4812__01473	1496150	14
MHI-4812	MHI-4812__01473	1496213	77
MHI-4812	MHI-4812__01474	1497113	143
MHI-4812	MHI-4812__01477	1500614	1021
MHI-4812	MHI-4812__01637	1670121	325
MHI-4812	MHI-4812__01637	1670169	277
MHI-4812	MHI-4812__01653	1685342	1267
MHI-4814	MHI-4814-pc__00061	62702	184
MHI-4814	MHI-4814-pc__00568	536930	1652
MHI-4814	MHI-4814-pc__00568	537586	996
MHI-4814	MHI-4814-pc__00953	945318	729
MHI-4814	MHI-4814-pc__00953	945602	445
MHI-4814	MHI-4814-pc__00953	945885	162
MHI-4814	MHI-4814-pc__00953	945933	114
MHI-4814	MHI-4814-pc__00953	945987	60

MHI-4814	MHI-4814-pc__00953	945989	58
MHI-4814	MHI-4814-pc__00953	946007	40
MHI-4814	MHI-4814-pc__00953	946015	32
MHI-4814	MHI-4814-pc__00953	946022	25
MHI-4814	MHI-4814-pc__00954	946035	628
MHI-4814	MHI-4814-pc__00954	946038	625
MHI-4814	MHI-4814-pc__00954	946049	614
MHI-4814	MHI-4814-pc__00954	946052	611
MHI-4814	MHI-4814-pc__00954	946079	584
MHI-4814	MHI-4814-pc__00954	946083	580
MHI-4814	MHI-4814-pc__00954	946085	578
MHI-4814	MHI-4814-pc__00954	946106	557
MHI-4814	MHI-4814-pc__00954	946122	541
MHI-4814	MHI-4814-pc__00954	946127	536
MHI-4814	MHI-4814-pc__00954	946170	493
MHI-4814	MHI-4814-pc__00954	946200	463
MHI-4814	MHI-4814-pc__00954	946214	449
MHI-4814	MHI-4814-pc__00955	946726	691
MHI-4814	MHI-4814-pc__00979	967405	506
MHI-4814	MHI-4814-pc__01137	1125886	963
MHI-4814	MHI-4814-pc__01369	1320513	552
MHI-4814	MHI-4814-pc__01879	1820699	81
MHI-4814	MHI-4814-pc__01879	1820711	93
MHI-4814	MHI-4814-pc__01879	1820712	94
MHI-4814	MHI-4814-pc__01879	1820746	128
MHI-4814	MHI-4814-pc__01879	1820761	143
MHI-4890	MHI-4890__00347	345295	188
MHI-4890	MHI-4890__00347	345304	179
MHI-4890	MHI-4890__00347	345340	143
MHI-4890	MHI-4890__00375	375081	1651
MHI-4890	MHI-4890__00375	375132	1702
MHI-4890	MHI-4890__00375	375133	1703
MHI-4890	MHI-4890__01580	1583263	211
MHI-4890	MHI-4890__01669	1648905	882
MHI-4890	MHI-4890__01669	1648941	918
