# Supplementary material for: Intra-host genomic variation of serologically nontypeable Haemophilus influenzae isolates from otitis media
Source: Microbiol Spectr. 2025 Mar 31;13(5):e03089-24. doi: 10.1128/spectrum.03089-24 (PMC12053901; doi:10.1128/spectrum.03089-24)
Supplement: Table S5 — Genes with a polymorphism in at least one otitis media isolate. [file spectrum.03089-24-s0005.pdf]

Table S5. Genes with a polymorphism in at least one otitis media isolate.

Gene	Protein	Function	MHI4419	MHI4466	MHI4506	MHI4537	MHI4578	MHI4615	MHI4640	MHI4688	MHI4743	MHI4772	MHI4812	MHI4814	MHI4890	Nucleotide identity (%)	Polymorphism	Substitution	Child_MLST	Isolate
atoD	Acetate CoA-transferase sub	Carbohydrate metabolism	absent	absent	present	present	absent	absent	present	present	absent	present	absent	present	absent	99.6	G5T	T2K	I_266	MHI4867
dlgD	2,3-diketo-L-gulonate reducta	Carbohydrate metabolism	absent	absent	present	present	absent	absent	present	absent	absent	present	absent	absent	absent	99.9	A212C	L71*	I_266	MHI4867
gapA	Glyceraldehyde-3-phosphate	Carbohydrate metabolism	present	present	present	present	present	present	present	present	present	present	present	present	absent	98.4	A117C	E39D	I_266	MHI4867
glgA	Glycogen synthase	Carbohydrate metabolism	present	present	present	present	present	present	present	present	present	present	present	present	absent	94.8	C1028T	A343V	I_266	MHI4769
glmM_2	Phosphoglucosamine mutase	Carbohydrate metabolism	present	present	absent	absent	absent	absent	absent	absent	absent	absent	absent	absent	absent	97.7	T47C	N16S	A_155	MHI4448
glmM_3	Phosphoglucosamine mutase	Carbohydrate metabolism	present	present	absent	absent	absent	absent	absent	absent	absent	absent	absent	absent	absent	97.7	A301G	*101Q	A_155	MHI4448
malP	Maltodextrin phosphorylase	Carbohydrate metabolism	present	present	present	present	present	present	present	present	present	present	present	present	absent	96.2	CG1612TC	R538S	A_155	MHI4769
nanK	N-acetylmannosamine kinase	Carbohydrate metabolism	present	present	present	present	present	present	present	present	present	present	present	present	absent	99.0	C622G	E208Q	H_3	MHI4716
																	C622G	E208Q	I_266	MHI4809
																	C436A	G146C	I_266	MHI4867
pflB	Formate acetyltransferase 1	Carbohydrate metabolism	present	present	present	present	present	present	present	present	present	present	present	present	absent	98.5	A2032C	F678V	I_266	MHI4867
pykA	Pyruvate kinase II	Carbohydrate metabolism	present	present	present	present	present	present	present	present	present	present	present	present	absent	98.9	C576A	F192L	K_590	MHI4902
ispU	Isoprenyl transferase	Cell wall biosynthesis	present	present	present	present	present	present	present	present	present	present	present	present	absent	98.6	C670T	A224T	I_1927	MHI4729
mreC	Cell shape-determining protei	Cell wall biosynthesis	present	present	present	present	present	present	present	present	present	present	present	present	absent	98.8	T1025C	I342T	I_1927	MHI4727
mtgA	Biosynthetic peptidoglycan tr	Cell wall biosynthesis	present	present	present	present	present	present	present	present	present	present	present	present	absent	98.3	C577A	H193N	F_1030	MHI4623
mukB	Chromosome partition protei	Cell wall biosynthesis	present	present	present	present	present	present	present	present	present	present	present	present	absent	97.0	C1610T	S537N	I_266	MHI4867
																	C3455A	A1152E	E_1030	MHI4586
opgE_1	Phosphoethanolamine transfe	Cell wall biosynthesis	present	present	present	present	present	present	present	present	present	present	present	present	absent	97.9	A1321C	I441L	D_1013	MHI4541
																	T618G	F206L	I_266	MHI4867
grxA	Glutaredoxin 1	Envlronmental stress response	present	present	present	present	present	present	present	present	present	present	present	present	absent	98.6	A257C	V86G	C_145	MHI4528
htpX	Protease HtpX	Envlronmental stress response	present	present	present	present	present	present	present	present	present	present	present	present	absent	97.1	A320C	F107C	K_590	MHI4916
katA	Catalase	Envlronmental stress response	present	present	present	present	present	present	present	present	present	present	present	present	absent	97.2	G1474T	A492S	I_266	MHI4867
maeB	NADP-dependent malic enzy	Envlronmental stress response	present	present	present	present	present	present	present	present	present	present	present	present	absent	97.9	G2258T	A753E	I_266	MHI4867
menF	Isochorismate synthase	Envlronmental stress response	present	present	present	present	present	present	present	present	present	present	present	present	absent	97.9	A153C	F51L	I_266	MHI4867
mopII	Molybdenum-pterin-binding p	Envlronmental stress response	present	present	present	present	present	present	present	present	present	present	present	present	absent	98.1	G160T	E54*	I_266	MHI4867
ndh	Type II NADH:quinone oxidor	Envlronmental stress response	present	present	present	present	present	present	present	present	present	present	present	present	absent	99.0	G1273T	G425C	I_266	MHI4867
sohB	Inner membrane peptidase	Envlronmental stress response	present	present	present	present	present	present	present	present	present	present	present	present	absent	98.9	C111A	N37K	I_266	MHI4867
																	G151T	E51*	I_266	MHI4867
																	G149T	S50*	K_590	MHI4910
spoT	Bifunctional (p)ppGpp syntha	Envlronmental stress response	present	present	present	present	present	present	present	present	present	present	present	present	absent	98.6	G149T	S50*	J_583	MHI4840
sppA_1	Stringent starvation protein A	Envlronmental stress response	present	present	present	present	present	present	present	present	present	present	present	present	absent	99.3	C111A	N37K	I_266	MHI4867
																	G151T	E51*	I_266	MHI4867
																	G149T	S50*	K_590	MHI4910
ybgC	Acyl-CoA thioesterase	Envlronmental stress response	present	present	present	present	present	present	present	present	present	present	present	present	absent	97.9	G149T	S50*	I_266	MHI4867
lapA	Lipopolysaccharide assembly	Glycolipid metabolism	present	present	present	present	present	present	present	present	present	present	present	present	absent	99.2	G149T	S50*	I_1927	MHI4750
lex1	Lipooligosaccharide biosynth	Glycolipid metabolism	present	present	present	present	present	present	present	present	present	present	present	present	absent	97.2	GA215TT	S72N	I_266	MHI4854
lst	CMP-N-acetylneuraminate:be	Glycolipid metabolism	present	present	present	present	present	present	present	present	present	present	present	present	absent	97.7				
plsB	Glycerol-3-phosphate acyltra	Glycolipid metabolism	present	present	present	present	present	present	present	present	present	present	present	present	absent	99.5	A2027C	I676S	I_266	MHI4867
																	G262A	G88S	K_590	MHI4909
ftnA_2	Bacterial non-heme ferritin	Iron metabolism	present	present	present	present	present	present	present	present	present	present	present	present	absent	99.8	C86A	A29D	D_1013	MHI4553
																	A406G	S136G	K_590	MHI4911
hgpA	Hemoglobin and hemoglobin-	Iron metabolism	present	absent	present	present	present	present	present	present	present	absent	present	present	present	97.8	C1478A	R493L	D_1013	MHI4555
patB	Cystathionine beta-lyase	Iron metabolism	present	present	present	present	present	present	present	present	present	present	present	present	absent	99.5	A83C	I28S	D_1013	MHI4573
																	C794A	P265Q	K_590	MHI4904
																	C815A	A272D	K_590	MHI4904
tbpA	Transferrin-binding protein A	Iron metabolism	present	present	present	present	present	present	present	present	present	present	present	present	absent	96.2	C1897A	V633F	I_266	MHI4867
tbpB	Transferrin-binding protein B	Iron metabolism	present	present	present	present	present	present	present	present	present	present	present	present	absent	84.6	C1355T	G452D	I_266	MHI4867
hindIIIR	Type II restriction endonuclea	recombination	absent	absent	present	absent	absent	absent	present	absent	absent	present	absent	absent	absent	99.8	G714T	E238D	I_266	MHI4887
rdgC_1	Recombination associated pr	recombination	present	present	present	present	present	present	present	present	present	present	present	present	absent	97.3	G440A	A147V	B_155	MHI4479
recB	RecBCD enzyme subunit Re	recombination	present	present	present	present	present	present	present	present	present	present	present	present	absent	97.5	C682A	E228*	I_266	MHI4854
recD2	RecD-like DNA helicase	recombination	absent	absent	absent	present	absent	absent	absent	absent	absent	absent	absent	present	absent	100	T2516A	F839Y	D_1013	MHI4552
																	G2519T	S840I	D_1013	MHI4552
																	T2528A	F843Y	D_1013	MHI4552
																	G2551T	V851L	D_1013	MHI4552
																	G2593T	V865L	D_1013	MHI4552
																	A2729C	N910T	D_1013	MHI4547
																	A2639C	D880A	J_99	MHI4842
																	A2653C	M885L	J_99	MHI4842
cpxR	Transcriptional Regulatory pr	Regulator	present	present	present	present	present	present	present	present	present	present	present	present	absent	97.8	A2653C	M885L	I_266	MHI4867
glpR_1	Glycerol-3-phosphate regulor	Regulator	absent	absent	absent	absent	present	present	absent	absent	present	present	present	present	absent	97.2	A742C	*248E	D_1013	MHI4878
asd	Aspartate-semialdehyde deh	Transcription/Translation	present	present	present	present	present	present	present	present	present	present	present	present	present	96.3	C372A	L124F	I_266	MHI4867
birA	Bifunctional ligase/repressor	Transcription/Translation	present	present	present	present	present	present	present	present	present	present	present	present	present	98.4	T843A	K281N	I_1927	MHI4748
citC	Citrate [pro-3S]-lyase ligase	Transcription/Translation	present	present	present	present	present	present	present	present	present	present	present	present	absent	99.7	C1001A	R334L	I_266	MHI4867
citG	2-(5''-triphosphoribosyl)-3'-de	Transcription/Translation	present	present	present	present	present	present	present	present	present	present	present	present	absent	99.3	C6A	Q2H	I_266	MHI4867
cpdB	2',3'-cyclic-nucleotide 2'-phos	Transcription/Translation	present	present	present	present	present	present	present	present	present	present	present	present	present	94.8	G980A	A327V	J_583	4830
epmB	L-lysine 2,3-aminomutase	Transcription/Translation	present	present	present	present	present	present	present	present	present	present	present	present	absent	98.2	C983T	A328V	I_266	MHI4867
ihfB	Integration host factor subuni	Transcription/Translation	present	present	present	present	present	present	present	present	present	present	present	present	absent	98.8	A59C	K20T	K_590	MHI4907
lysA	Diaminopimelate decarboxyla	Transcription/Translation	present	present	present	present	present	present	present	present	present	present	present	present	absent	97.5	C536T	R179Q	G_145	MHI4671
pglH	Protein glycosylation H	Transcription/Translation	present	present	present	present	present	present	present	present	present	present	present	present	absent	98.1	C697A	A233B	B_155	MHI4478
																	C184A	P62T	E_1030	MHI4592
																	C860A	C287F	H_3	MHI4710
																	C1015A	D339Y	I_266	MHI4487
																	T582G	F191L	I_1927	MHI4747
																	A573C	K194N	I_1927	MHI4747
ppiD	Peptidyl-prolyl cis-trans isom	Transcription/Translation	present	present	present	present	present	present	present	present	present	present	present	present	absent	98.1	A1375G	I459V	H_3	MHI4704
purF	Amidophosphoribosyltransfer	Transcription/Translation	present	present	present	present	present	present	present	present	present	present	present	present	absent	96.1	A866T	E289V	I_266	MHI4867
sdhE	FAD assembly factor	Transcription/Translation	present	present	present	present	present	present	present	present	present	present	present	present	absent	91.7	A49C	M17L	A_155	MHI4448
ssb_1	Single stranded DNA binding	Transcription/Translation	present	present	present	present	present	present	present	present	present	present	present	present	absent	97.1	T424C	I142V	B_155	MHI4472
tldD	Metalloprotease TldD	Transcription/Translation	present	present	present	present	present	present	present	present	present	present	present	present	absent	97.6	G1040T	P347Q	I_266	MHI4865

trkH	Trk system potassium uptake	Transcription/Translation	present	present	present	present	present	present	present	present	present	present	present	present	absent	98.7	G1454T	W485L	I_266	MHI4810
trpC	Tryptophan biosynthesis prot	Transcription/Translation	present	present	present	present	present	present	present	present	present	present	present	present	absent	97.8	A549T	E183D	I_1927	MHI4769
trpE	Anthranilate synthase compo	Transcription/Translation	present	present	present	present	present	present	present	present	present	present	present	present	absent	96.3	G16T	Q6K	I_266	MHI4867
trpGD	Anthranilate phosphoribosyl t	Transcription/Translation	present	present	present	present	present	present	present	present	present	present	present	present	absent	97.8	G910A	H304Y	I_266	MHI4867
tuaG	Putative teichuronic acid bios	Transcription/Translation	present	present	absent	present	present	present	absent	absent	present	absent	present	present	absent	99.7	G118T	D40Y	A_155	MHI4464
tusC	SulfurTransferase complex su	Transcription/Translation	present	present	present	present	present	present	present	present	present	present	present	present	absent	96.7	C280A	L94I	I_266	MHI4867
uup	ATP-binding protein	Transcription/Translation	present	present	present	present	present	present	present	present	present	present	present	present	absent	97.8	G1808T	A603E	I_266	MHI4867
uvrD	DNA helicase II	Transcription/Translation	present	present	present	present	present	present	present	present	present	present	present	present	absent	98.4	T434G	K145T	I_1927	MHI4747
ydgA	DNA topoisomerase	Transcription/Translation	present	present	present	present	present	present	present	present	present	present	present	present	absent	93.9	A172G	K58E	I_266	MHI4867
																	A190T	R64*	I_266	MHI4867
																	G226T	E76*	I_1927	MHI4750
ampG	Anhydromuropeptide permea	Transporter	present	present	present	present	present	present	present	present	present	present	present	present	present	98.7	A752G	K251R	E_1030	MHI4602
btuC	Vitamin B12 import system p	Transporter	absent	absent	absent	absent	absent	absent	absent	absent	present	present	absent	absent	absent	99.7	A179C	I60S	I_266	MHI4867
cntI	Pseudopaline exporter	Transporter	present	present	present	present	absent	absent	present	present	present	present	absent	absent	absent	99.2	T838C	S280G	I_266	MHI4867
gltC	Sodium/glutamate symporter	Transporter	present	present	present	present	present	present	present	present	present	present	present	present	absent	98.8	G610A	R204W	I_266	MHI4862
lysO	Lysine exporter	Transporter	absent	absent	absent	present	absent	absent	absent	present	absent	present	absent	absent	absent	96.2	T113C	L38S	I_266	MHI4867
metN	Methionine import ATP-bindi	Transporter	present	present	present	present	present	present	present	present	present	present	present	present	absent	98.9	T148A	C50S	I_266	MHI4867
mleN	Malate-2H(+)/Na(+)-lactate ar	Transporter	present	present	present	present	present	present	present	present	present	present	present	present	absent	98.9	T574C	T192A	J_583	MHI4830
nqrA	Na(+)-translocating NADH-qu	Transporter	present	present	present	present	present	present	present	present	present	present	present	present	absent	99.0	T980A	V327E	I_266	MHI4867
phoR	Phosphate regulon sensor pro	Transporter	present	present	present	present	present	present	present	present	present	present	present	present	absent	99.7	G424T	Q424K	A_155	MHI4446
																	T137G	L46*	J_583	MHI4813
																	G341T	C114F	J_583	MHI4821
																	G479T	T160N	J_99	MHI4845
																	T384A	K128N	K_590	MHI4907
																	T356A	Q119L	K_590	MHI4907
																	G296T	P99Q	K_590	MHI4907
																	G139T	L47I	K_590	MHI4904
pnuC	Nicotinamide riboside transpo	Transporter	present	present	present	present	present	present	present	present	present	present	present	present	absent	97.9	T579A	L193F	J_583	MHI4830
potD_2	Spermidine/putrescine-bindin	Transporter	present	present	present	present	present	present	present	present	present	present	present	present	absent	99.5	G1007T	A336D	I_266	MHI4867
proY	Proline-specific permease	Transporter	absent	absent	absent	absent	absent	absent	absent	absent	present	absent	absent	absent	absent	100	A418C	N140H	I_1927	MHI4750
ptsH	Phosphocarrier protein	Transporter	present	present	present	present	present	present	present	present	present	present	present	present	absent	97.8	T213G	Q71H	I_1927	MHI4735
rnfE	Ion-translocating oxidoreducta	Transporter	present	present	present	present	present	present	present	present	present	present	present	present	absent	97.4	C185A	A62E	G_145	MHI4680
rsxC	Ion-translocating oxidoreducta	Transporter	present	present	present	present	present	present	present	present	present	present	present	present	absent	98.4	T1765A	S589T	I_266	MHI4804
sapA	Peptide transport periplasmic	Transporter	present	present	present	present	present	present	present	present	present	present	present	present	absent	98.2	T889G	I297L	I_266	MHI4867
secA	Protein translocase subunit S	Transporter	present	present	present	present	present	present	present	present	present	present	present	present	absent	97.7	A2706T	*902Y	I_266	MHI4867
siaP	Sialic acid-binding periplasmi	Transporter	present	present	present	present	present	present	present	present	present	present	present	present	absent	99.1	C622T	Q208*	I_266	MHI4775
tehA	Tellurite resistance protein	Transporter	present	present	present	present	present	present	present	present	present	present	present	present	absent	94.8	T520G	M174L	A_155	MHI4451
tyrP_2	Aromatic amino acid permea	Transporter	present	present	present	present	present	present	present	present	present	present	present	present	absent	99.2	C1162A	V388L	B_155	MHI4474
ydcV	Inner membrane ABC transp	Transporter	present	present	present	present	present	present	present	present	present	present	present	present	absent	99.8	C13A	L5I	I_266	MHI4867
yjhB	Putative metabolite transport	Transporter	present	present	present	present	present	present	present	present	present	present	present	present	absent	98.6	G1173T	M391I	I_266	MHI4867
																	C1214A	A405D	I_266	MHI4867
																	T734G	L245*	I_1927	MHI4740
esiB	Secretory immunoglobulin A-	Virulence	present	present	absent	absent	present	present	absent	absent	absent	present	present	present	present	90.3	G725A	R242Q	I_266	MHI4867
iga	Immunoglobulin A1 protease	Virulence	present	present	present	present	present	present	present	present	present	present	present	present	absent	95.3	G4804T	H1602N	I_266	MHI4867
