# Supplementary material for: Intra-host genomic variation of serologically nontypeable Haemophilus influenzae isolates from otitis media
Source: Microbiol Spectr. 2025 Mar 31;13(5):e03089-24. doi: 10.1128/spectrum.03089-24 (PMC12053901; doi:10.1128/spectrum.03089-24)
Supplement: Table S6 — Mean pairwise distances among isolates in each Child-MLST group. [file spectrum.03089-24-s0006.pdf]

Table S6. Mean pairwise distances among isolates in each Child-MLST group.

Child_MLST group	Isolates (number)	Mean Pairwise Distance (SNPs/strain)
A_155	47	15.9
B_155	40	16.1
C_145	31	6.2
D_1013	40	9.4
E_1030	35	7.0
F_1030	25	6.3
G_145	47	24.5
H_3	29	4.4
I_266	76	7.2
I_1927	54	12.5
J_99	9	25.8
J_583	28	12.5
K_590	39	20.0
