# Supplementary material for: Intra-host genomic variation of serologically nontypeable Haemophilus influenzae isolates from otitis media
Source: Microbiol Spectr. 2025 Mar 31;13(5):e03089-24. doi: 10.1128/spectrum.03089-24 (PMC12053901; doi:10.1128/spectrum.03089-24)
Supplement: Table S4 — 120,500 unique SNP loci relative to the core genome. [file spectrum.03089-24-s0004.pdf]

Table S4. 120,500 unique SNP loci relative to the core genome.

Position	Locus tag	Position	Reference	Sample	Substitution
244	intergenic	intergenic	C	G	intergenic
245	intergenic	intergenic	A	C	intergenic
247	intergenic	intergenic	G	A	intergenic
263	intergenic	intergenic	G	A	intergenic
304	intergenic	intergenic	T	C	intergenic
307	intergenic	intergenic	T	C	intergenic
310	intergenic	intergenic	T	A	intergenic
316	intergenic	intergenic	A	G	intergenic
322	intergenic	intergenic	T	G	intergenic
356	intergenic	intergenic	G	C	intergenic
367	intergenic	intergenic	T	A	intergenic
375	intergenic	intergenic	A	C	intergenic
386	intergenic	intergenic	T	G	intergenic
387	intergenic	intergenic	A	G	intergenic
401	intergenic	intergenic	C	G	intergenic
743	intergenic	intergenic	A	C	intergenic
802	intergenic	intergenic	C	T	intergenic
881	intergenic	intergenic	G	A	intergenic
882	intergenic	intergenic	T	G	intergenic
882	intergenic	intergenic	T	A	intergenic
883	intergenic	intergenic	C	G	intergenic
884	intergenic	intergenic	C	G	intergenic
884	intergenic	intergenic	C	A	intergenic
885	intergenic	intergenic	C	T	intergenic
887	intergenic	intergenic	A	T	intergenic
888	intergenic	intergenic	G	A	intergenic
890	intergenic	intergenic	G	C	intergenic
890	intergenic	intergenic	G	T	intergenic
891	intergenic	intergenic	G	C	intergenic
892	intergenic	intergenic	G	C	intergenic
892	intergenic	intergenic	G	T	intergenic
893	intergenic	intergenic	C	T	intergenic
973	intergenic	intergenic	C	G	intergenic
1022	intergenic	intergenic	C	A	intergenic
1027	intergenic	intergenic	C	A	intergenic
1285	intergenic	intergenic	C	A	intergenic
1289	intergenic	intergenic	T	A	intergenic
1294	intergenic	intergenic	C	T	intergenic
1297	intergenic	intergenic	T	G	intergenic
1298	intergenic	intergenic	G	C	intergenic
1298	intergenic	intergenic	G	A	intergenic
1307	intergenic	intergenic	C	G	intergenic

1307	intergenic	intergenic	C	T	intergenic
1308	intergenic	intergenic	A	T	intergenic
1309	intergenic	intergenic	T	C	intergenic
1311	intergenic	intergenic	G	A	intergenic
1317	intergenic	intergenic	G	T	intergenic
1319	intergenic	intergenic	G	A	intergenic
1320	intergenic	intergenic	A	T	intergenic
1364	intergenic	intergenic	G	T	intergenic
1365	intergenic	intergenic	A	G	intergenic
1368	intergenic	intergenic	A	G	intergenic
1373	intergenic	intergenic	T	C	intergenic
1457	intergenic	intergenic	C	G	intergenic
1473	intergenic	intergenic	G	C	intergenic
1786	intergenic	intergenic	T	A	intergenic
1795	intergenic	intergenic	G	T	intergenic
1837	intergenic	intergenic	T	C	intergenic
1850	intergenic	intergenic	A	G	intergenic
1856	intergenic	intergenic	C	G	intergenic
1865	intergenic	intergenic	G	C	intergenic
1873	intergenic	intergenic	T	C	intergenic
1884	intergenic	intergenic	A	G	intergenic
1941	intergenic	intergenic	C	T	intergenic
1942	intergenic	intergenic	A	C	intergenic
1950	intergenic	intergenic	C	A	intergenic
1952	intergenic	intergenic	T	C	intergenic
1953	intergenic	intergenic	T	A	intergenic
1957	intergenic	intergenic	T	A	intergenic
1958	intergenic	intergenic	C	G	intergenic
1994	intergenic	intergenic	C	T	intergenic
1995	intergenic	intergenic	A	G	intergenic
1995	intergenic	intergenic	A	T	intergenic
2008	intergenic	intergenic	T	A	intergenic
2009	intergenic	intergenic	A	T	intergenic
2014	intergenic	intergenic	T	G	intergenic
2015	intergenic	intergenic	A	T	intergenic
2028	intergenic	intergenic	A	C	intergenic
2029	intergenic	intergenic	G	A	intergenic
2039	intergenic	intergenic	G	T	intergenic
2078	intergenic	intergenic	G	T	intergenic
2121	intergenic	intergenic	T	C	intergenic
2126	intergenic	intergenic	C	A	intergenic
2132	intergenic	intergenic	C	G	intergenic
2133	intergenic	intergenic	C	A	intergenic
2159	intergenic	intergenic	G	T	intergenic
2160	intergenic	intergenic	T	C	intergenic

2163	intergenic	intergenic	A	G	intergenic
2175	intergenic	intergenic	T	C	intergenic
2179	intergenic	intergenic	A	G	intergenic
2182	intergenic	intergenic	G	A	intergenic
2190	intergenic	intergenic	T	C	intergenic
2192	intergenic	intergenic	A	G	intergenic
2193	intergenic	intergenic	G	A	intergenic
2196	intergenic	intergenic	C	T	intergenic
2204	intergenic	intergenic	G	A	intergenic
2205	intergenic	intergenic	G	A	intergenic
2218	intergenic	intergenic	A	C	intergenic
2232	intergenic	intergenic	G	T	intergenic
2249	intergenic	intergenic	G	A	intergenic
2250	intergenic	intergenic	A	C	intergenic
2271	intergenic	intergenic	A	G	intergenic
2276	intergenic	intergenic	G	A	intergenic
2281	intergenic	intergenic	C	T	intergenic
2301	intergenic	intergenic	T	C	intergenic
2322	intergenic	intergenic	A	C	intergenic
2323	intergenic	intergenic	C	A	intergenic
2357	intergenic	intergenic	C	T	intergenic
2359	intergenic	intergenic	C	T	intergenic
2365	intergenic	intergenic	T	C	intergenic
2368	intergenic	intergenic	C	T	intergenic
2408	intergenic	intergenic	C	T	intergenic
2414	intergenic	intergenic	G	A	intergenic
2418	intergenic	intergenic	C	T	intergenic
2431	intergenic	intergenic	C	A	intergenic
2437	intergenic	intergenic	A	T	intergenic
2443	intergenic	intergenic	T	C	intergenic
2523	intergenic	intergenic	T	G	intergenic
2526	intergenic	intergenic	G	T	intergenic
2527	intergenic	intergenic	G	A	intergenic
2539	intergenic	intergenic	G	T	intergenic
2562	intergenic	intergenic	T	C	intergenic
2584	intergenic	intergenic	A	C	intergenic
2586	intergenic	intergenic	C	A	intergenic
2603	intergenic	intergenic	T	G	intergenic
2615	intergenic	intergenic	G	A	intergenic
2712	NTHI477_RS00015	63	C	T	synonymous
2715	NTHI477_RS00015	66	C	T	synonymous
2733	NTHI477_RS00015	84	G	A	synonymous
2735	NTHI477_RS00015	86	A	G	nonsynonymous
2736	NTHI477_RS00015	87	C	T	synonymous
2757	NTHI477_RS00015	108	A	C	synonymous

2811	NTHI477_RS00015	162	G	A	synonymous
2868	NTHI477_RS00015	219	A	G	synonymous
2880	NTHI477_RS00015	231	G	A	nonsynonymous
2883	NTHI477_RS00015	234	G	A	synonymous
2883	NTHI477_RS00015	234	G	T	synonymous
2914	NTHI477_RS00015	265	C	A	synonymous
2926	NTHI477_RS00015	277	G	T	nonsynonymous
2940	NTHI477_RS00015	291	A	G	synonymous
2941	NTHI477_RS00015	292	T	C	synonymous
2997	NTHI477_RS00015	348	C	T	synonymous
3028	NTHI477_RS00015	379	T	C	synonymous
3054	NTHI477_RS00015	405	G	T	synonymous
3066	NTHI477_RS00015	417	C	T	synonymous
3109	NTHI477_RS00015	460	G	A	nonsynonymous
3129	NTHI477_RS00015	480	T	C	synonymous
3165	NTHI477_RS00015	516	A	G	synonymous
3191	NTHI477_RS00015	542	C	T	nonsynonymous
3201	NTHI477_RS00015	552	C	T	synonymous
3213	NTHI477_RS00015	564	A	G	synonymous
3246	NTHI477_RS00015	597	C	T	synonymous
3292	NTHI477_RS00015	643	C	T	nonsynonymous
3311	NTHI477_RS00015	662	C	T	nonsynonymous
3360	NTHI477_RS00015	711	C	T	synonymous
3361	NTHI477_RS00015	712	A	C	nonsynonymous
3362	NTHI477_RS00015	713	A	G	nonsynonymous
3363	NTHI477_RS00015	714	A	G	synonymous
3363	NTHI477_RS00015	714	A	T	nonsynonymous
3366	NTHI477_RS00015	717	C	T	synonymous
3369	NTHI477_RS00015	720	T	C	synonymous
3375	NTHI477_RS00015	726	C	G	synonymous
3378	NTHI477_RS00015	729	A	T	synonymous
3379	NTHI477_RS00015	730	G	A	nonsynonymous
3381	NTHI477_RS00015	732	G	C	synonymous
3396	NTHI477_RS00015	747	T	C	synonymous
3396	NTHI477_RS00015	747	T	G	synonymous
3397	NTHI477_RS00015	748	T	C	synonymous
3414	NTHI477_RS00015	765	A	G	synonymous
3423	NTHI477_RS00015	774	T	C	synonymous
3436	NTHI477_RS00015	787	A	G	nonsynonymous
3445	NTHI477_RS00015	796	A	G	nonsynonymous
3446	NTHI477_RS00015	797	G	A	nonsynonymous
3447	NTHI477_RS00015	798	T	A	nonsynonymous
3450	NTHI477_RS00015	801	C	T	synonymous
3456	NTHI477_RS00015	807	C	T	synonymous
3462	NTHI477_RS00015	813	C	T	synonymous

3463	NTHI477_RS00015	814	T	C	synonymous
3471	NTHI477_RS00015	822	C	G	synonymous
3473	NTHI477_RS00015	824	C	T	nonsynonymous
3489	NTHI477_RS00015	840	C	T	synonymous
3495	NTHI477_RS00015	846	C	T	synonymous
3528	NTHI477_RS00015	879	C	T	synonymous
3555	NTHI477_RS00015	906	G	T	synonymous
3555	NTHI477_RS00015	906	G	C	synonymous
3581	intergenic	intergenic	C	T	intergenic
3582	intergenic	intergenic	G	A	intergenic
3615	NTHI477_RS00020	27	G	C	nonsynonymous
3616	NTHI477_RS00020	28	C	T	nonsynonymous
3617	NTHI477_RS00020	29	C	T	nonsynonymous
3623	NTHI477_RS00020	35	C	A	nonsynonymous
3634	NTHI477_RS00020	46	G	T	nonsynonymous
3642	NTHI477_RS00020	54	G	A	synonymous
3657	NTHI477_RS00020	69	C	T	synonymous
3702	NTHI477_RS00020	114	T	C	synonymous
3744	NTHI477_RS00020	156	C	T	synonymous
3750	NTHI477_RS00020	162	A	G	synonymous
3762	NTHI477_RS00020	174	G	T	synonymous
3774	NTHI477_RS00020	186	T	C	synonymous
3792	NTHI477_RS00020	204	G	A	synonymous
3807	NTHI477_RS00020	219	C	T	synonymous
3816	NTHI477_RS00020	228	C	T	synonymous
3858	NTHI477_RS00020	270	C	T	synonymous
3859	NTHI477_RS00020	271	A	C	synonymous
3861	NTHI477_RS00020	273	A	C	nonsynonymous
3886	NTHI477_RS00020	298	T	C	synonymous
3921	NTHI477_RS00020	333	C	T	synonymous
3942	NTHI477_RS00020	354	G	T	synonymous
3942	NTHI477_RS00020	354	G	C	synonymous
3961	NTHI477_RS00020	373	C	T	synonymous
3984	NTHI477_RS00020	396	T	A	synonymous
3986	NTHI477_RS00020	398	C	A	nonsynonymous
4002	NTHI477_RS00020	414	A	C	synonymous
4002	NTHI477_RS00020	414	A	T	synonymous
4032	NTHI477_RS00020	444	G	A	synonymous
4053	NTHI477_RS00020	465	T	C	synonymous
4080	NTHI477_RS00020	492	T	G	synonymous
4109	NTHI477_RS00020	521	A	G	nonsynonymous
4122	NTHI477_RS00020	534	A	C	synonymous
4126	NTHI477_RS00020	538	C	T	synonymous
4146	NTHI477_RS00020	558	C	A	synonymous
4156	NTHI477_RS00020	568	T	C	synonymous

4170	NTHI477_RS00020	582	C	T	synonymous
4185	NTHI477_RS00020	597	A	C	synonymous
4197	NTHI477_RS00020	609	C	G	synonymous
4200	NTHI477_RS00020	612	C	T	synonymous
4206	NTHI477_RS00020	618	A	T	nonsynonymous
4208	NTHI477_RS00020	620	T	C	nonsynonymous
4216	NTHI477_RS00020	628	T	C	nonsynonymous
4258	NTHI477_RS00020	670	A	G	nonsynonymous
4273	NTHI477_RS00020	685	T	C	synonymous
4284	NTHI477_RS00020	696	A	C	synonymous
4288	NTHI477_RS00020	700	G	A	nonsynonymous
4304	NTHI477_RS00020	716	C	A	nonsynonymous
4311	NTHI477_RS00025	7	C	T	synonymous
4315	NTHI477_RS00025	11	C	T	nonsynonymous
4325	NTHI477_RS00025	21	T	C	synonymous
4334	NTHI477_RS00025	30	C	T	synonymous
4349	NTHI477_RS00025	45	C	G	synonymous
4385	NTHI477_RS00025	81	G	A	synonymous
4427	NTHI477_RS00025	123	C	T	synonymous
4442	NTHI477_RS00025	138	T	C	synonymous
4445	NTHI477_RS00025	141	A	T	synonymous
4457	NTHI477_RS00025	153	T	A	synonymous
4490	NTHI477_RS00025	186	T	G	nonsynonymous
4502	NTHI477_RS00025	198	C	T	synonymous
4511	NTHI477_RS00025	207	C	T	synonymous
4532	NTHI477_RS00025	228	G	A	synonymous
4538	NTHI477_RS00025	234	C	T	synonymous
4547	NTHI477_RS00025	243	C	T	synonymous
4586	NTHI477_RS00025	282	C	T	synonymous
4589	NTHI477_RS00025	285	A	T	synonymous
4592	NTHI477_RS00025	288	C	A	synonymous
4592	NTHI477_RS00025	288	C	T	synonymous
4595	NTHI477_RS00025	291	A	G	synonymous
4609	NTHI477_RS00025	305	C	T	nonsynonymous
4613	NTHI477_RS00025	309	T	C	synonymous
4635	NTHI477_RS00025	331	T	A	nonsynonymous
4675	intergenic	intergenic	C	T	intergenic
4678	intergenic	intergenic	G	A	intergenic
4680	intergenic	intergenic	A	G	intergenic
4700	intergenic	intergenic	G	C	intergenic
4704	intergenic	intergenic	A	C	intergenic
4705	intergenic	intergenic	T	C	intergenic
4730	intergenic	intergenic	C	A	intergenic
4733	intergenic	intergenic	C	A	intergenic
4742	NTHI477_RS00030	6	A	C	nonsynonymous

4742	NTHI477_RS00030	6	A	T	nonsynonymous
4790	NTHI477_RS00030	54	T	G	synonymous
4792	NTHI477_RS00030	56	C	T	nonsynonymous
4799	NTHI477_RS00030	63	C	T	synonymous
4818	NTHI477_RS00030	82	C	T	nonsynonymous
4847	NTHI477_RS00030	111	T	G	synonymous
4869	NTHI477_RS00030	133	G	C	nonsynonymous
4879	NTHI477_RS00030	143	C	T	nonsynonymous
4883	NTHI477_RS00030	147	G	A	synonymous
4886	NTHI477_RS00030	150	T	C	synonymous
4889	NTHI477_RS00030	153	T	C	synonymous
4907	NTHI477_RS00030	171	G	C	synonymous
4940	NTHI477_RS00030	204	G	C	nonsynonymous
4958	NTHI477_RS00030	222	C	T	synonymous
4958	NTHI477_RS00030	222	C	A	nonsynonymous
4959	NTHI477_RS00030	223	G	C	nonsynonymous
4960	NTHI477_RS00030	224	G	T	nonsynonymous
4961	NTHI477_RS00030	225	A	C	synonymous
5010	intergenic	intergenic	G	A	intergenic
5017	intergenic	intergenic	G	A	intergenic
5021	intergenic	intergenic	T	C	intergenic
5042	intergenic	intergenic	T	C	intergenic
5052	NTHI477_RS00035	1575	C	T	synonymous
5055	NTHI477_RS00035	1572	C	G	synonymous
5058	NTHI477_RS00035	1569	C	A	synonymous
5064	NTHI477_RS00035	1563	A	G	synonymous
5070	NTHI477_RS00035	1557	C	A	synonymous
5071	NTHI477_RS00035	1556	G	A	nonsynonymous
5073	NTHI477_RS00035	1554	T	G	synonymous
5073	NTHI477_RS00035	1554	T	C	synonymous
5076	NTHI477_RS00035	1551	C	A	nonsynonymous
5079	NTHI477_RS00035	1548	G	A	synonymous
5082	NTHI477_RS00035	1545	G	A	synonymous
5094	NTHI477_RS00035	1533	G	A	synonymous
5103	NTHI477_RS00035	1524	T	C	synonymous
5121	NTHI477_RS00035	1506	C	T	synonymous
5124	NTHI477_RS00035	1503	A	G	synonymous
5130	NTHI477_RS00035	1497	A	G	synonymous
5159	NTHI477_RS00035	1468	G	A	synonymous
5169	NTHI477_RS00035	1458	G	A	synonymous
5175	NTHI477_RS00035	1452	A	G	synonymous
5199	NTHI477_RS00035	1428	C	T	synonymous
5200	NTHI477_RS00035	1427	C	T	nonsynonymous
5207	NTHI477_RS00035	1420	T	C	nonsynonymous
5208	NTHI477_RS00035	1419	G	A	synonymous

5214	NTHI477_RS00035	1413	T	C	synonymous
5226	NTHI477_RS00035	1401	C	T	synonymous
5238	NTHI477_RS00035	1389	T	C	synonymous
5253	NTHI477_RS00035	1374	C	T	synonymous
5277	NTHI477_RS00035	1350	C	T	synonymous
5279	NTHI477_RS00035	1348	A	G	synonymous
5283	NTHI477_RS00035	1344	A	C	synonymous
5319	NTHI477_RS00035	1308	A	C	synonymous
5322	NTHI477_RS00035	1305	C	A	synonymous
5325	NTHI477_RS00035	1302	G	A	synonymous
5370	NTHI477_RS00035	1257	T	C	synonymous
5390	NTHI477_RS00035	1237	A	G	synonymous
5418	NTHI477_RS00035	1209	A	C	synonymous
5427	NTHI477_RS00035	1200	C	T	synonymous
5436	NTHI477_RS00035	1191	G	A	synonymous
5445	NTHI477_RS00035	1182	T	C	synonymous
5454	NTHI477_RS00035	1173	C	T	synonymous
5460	NTHI477_RS00035	1167	G	A	synonymous
5485	NTHI477_RS00035	1142	A	G	nonsynonymous
5502	NTHI477_RS00035	1125	A	C	synonymous
5517	NTHI477_RS00035	1110	A	G	synonymous
5520	NTHI477_RS00035	1107	G	C	synonymous
5535	NTHI477_RS00035	1092	C	T	synonymous
5541	NTHI477_RS00035	1086	A	G	synonymous
5550	NTHI477_RS00035	1077	G	A	synonymous
5553	NTHI477_RS00035	1074	T	A	synonymous
5568	NTHI477_RS00035	1059	A	G	synonymous
5577	NTHI477_RS00035	1050	G	A	synonymous
5580	NTHI477_RS00035	1047	A	G	synonymous
5586	NTHI477_RS00035	1041	C	T	synonymous
5598	NTHI477_RS00035	1029	C	T	synonymous
5601	NTHI477_RS00035	1026	C	T	synonymous
5613	NTHI477_RS00035	1014	A	G	synonymous
5625	NTHI477_RS00035	1002	T	A	synonymous
5630	NTHI477_RS00035	997	T	G	nonsynonymous
5631	NTHI477_RS00035	996	A	T	synonymous
5634	NTHI477_RS00035	993	G	A	synonymous
5637	NTHI477_RS00035	990	G	T	nonsynonymous
5667	NTHI477_RS00035	960	T	G	synonymous
5670	NTHI477_RS00035	957	C	T	synonymous
5721	NTHI477_RS00035	906	A	G	synonymous
5724	NTHI477_RS00035	903	T	C	synonymous
5736	NTHI477_RS00035	891	T	C	synonymous
5772	NTHI477_RS00035	855	C	T	synonymous
5799	NTHI477_RS00035	828	C	T	synonymous

5805	NTHI477_RS00035	822	T	C	synonymous
5808	NTHI477_RS00035	819	A	C	synonymous
5808	NTHI477_RS00035	819	A	T	synonymous
5810	NTHI477_RS00035	817	C	A	nonsynonymous
5817	NTHI477_RS00035	810	C	T	synonymous
5832	NTHI477_RS00035	795	G	A	synonymous
5844	NTHI477_RS00035	783	C	T	synonymous
5853	NTHI477_RS00035	774	G	A	synonymous
5862	NTHI477_RS00035	765	T	C	synonymous
5877	NTHI477_RS00035	750	A	T	synonymous
5880	NTHI477_RS00035	747	G	T	synonymous
5880	NTHI477_RS00035	747	G	A	synonymous
5886	NTHI477_RS00035	741	C	T	synonymous
5889	NTHI477_RS00035	738	C	G	synonymous
5892	NTHI477_RS00035	735	T	C	synonymous
5898	NTHI477_RS00035	729	A	G	synonymous
5901	NTHI477_RS00035	726	C	G	synonymous
5907	NTHI477_RS00035	720	C	T	synonymous
5928	NTHI477_RS00035	699	A	G	synonymous
5937	NTHI477_RS00035	690	C	T	synonymous
5967	NTHI477_RS00035	660	C	T	synonymous
5982	NTHI477_RS00035	645	A	C	synonymous
5985	NTHI477_RS00035	642	A	G	synonymous
5986	NTHI477_RS00035	641	A	G	nonsynonymous
5988	NTHI477_RS00035	639	A	G	synonymous
6000	NTHI477_RS00035	627	A	G	synonymous
6001	NTHI477_RS00035	626	T	C	nonsynonymous
6006	NTHI477_RS00035	621	C	T	synonymous
6009	NTHI477_RS00035	618	G	A	synonymous
6015	NTHI477_RS00035	612	A	C	synonymous
6024	NTHI477_RS00035	603	A	C	synonymous
6028	NTHI477_RS00035	599	G	T	nonsynonymous
6028	NTHI477_RS00035	599	G	A	nonsynonymous
6030	NTHI477_RS00035	597	C	T	synonymous
6036	NTHI477_RS00035	591	G	A	synonymous
6042	NTHI477_RS00035	585	A	G	synonymous
6045	NTHI477_RS00035	582	C	T	synonymous
6048	NTHI477_RS00035	579	C	A	synonymous
6051	NTHI477_RS00035	576	T	A	synonymous
6060	NTHI477_RS00035	567	T	A	nonsynonymous
6062	NTHI477_RS00035	565	A	G	synonymous
6093	NTHI477_RS00035	534	G	A	synonymous
6105	NTHI477_RS00035	522	C	T	synonymous
6120	NTHI477_RS00035	507	T	C	synonymous
6135	NTHI477_RS00035	492	C	T	synonymous

6189	NTHI477_RS00035	438	A	G	synonymous
6219	NTHI477_RS00035	408	A	C	synonymous
6222	NTHI477_RS00035	405	G	A	synonymous
6228	NTHI477_RS00035	399	T	G	synonymous
6246	NTHI477_RS00035	381	T	G	synonymous
6261	NTHI477_RS00035	366	C	T	synonymous
6264	NTHI477_RS00035	363	A	C	nonsynonymous
6279	NTHI477_RS00035	348	A	C	synonymous
6294	NTHI477_RS00035	333	G	A	synonymous
6366	NTHI477_RS00035	261	G	A	synonymous
6375	NTHI477_RS00035	252	G	A	synonymous
6390	NTHI477_RS00035	237	A	G	synonymous
6395	NTHI477_RS00035	232	C	T	nonsynonymous
6396	NTHI477_RS00035	231	A	G	synonymous
6403	NTHI477_RS00035	224	G	T	nonsynonymous
6474	NTHI477_RS00035	153	G	A	synonymous
6486	NTHI477_RS00035	141	G	A	synonymous
6494	NTHI477_RS00035	133	C	T	nonsynonymous
6495	NTHI477_RS00035	132	C	T	synonymous
6498	NTHI477_RS00035	129	G	T	synonymous
6501	NTHI477_RS00035	126	T	A	synonymous
6504	NTHI477_RS00035	123	G	A	synonymous
6507	NTHI477_RS00035	120	A	T	synonymous
6510	NTHI477_RS00035	117	G	A	synonymous
6519	NTHI477_RS00035	108	T	G	synonymous
6606	NTHI477_RS00035	21	C	A	synonymous
6716	intergenic	intergenic	C	A	intergenic
6718	intergenic	intergenic	T	C	intergenic
6745	intergenic	intergenic	A	G	intergenic
6780	intergenic	intergenic	A	T	intergenic
6788	intergenic	intergenic	G	T	intergenic
6797	intergenic	intergenic	T	C	intergenic
6801	intergenic	intergenic	A	G	intergenic
6823	intergenic	intergenic	G	A	intergenic
6836	intergenic	intergenic	G	T	intergenic
6889	NTHI477_RS00040	33	C	A	synonymous
6889	NTHI477_RS00040	33	C	T	synonymous
6892	NTHI477_RS00040	36	A	T	synonymous
6916	NTHI477_RS00040	60	C	T	synonymous
6952	NTHI477_RS00040	96	T	C	synonymous
6979	NTHI477_RS00040	123	T	C	synonymous
6982	NTHI477_RS00040	126	A	T	nonsynonymous
6997	NTHI477_RS00040	141	T	C	synonymous
7024	NTHI477_RS00040	168	C	T	synonymous
7030	NTHI477_RS00040	174	T	C	synonymous

7057	NTHI477_RS00040	201	T	C	synonymous
7069	NTHI477_RS00040	213	T	C	synonymous
7075	NTHI477_RS00040	219	T	C	synonymous
7081	NTHI477_RS00040	225	C	T	synonymous
7093	NTHI477_RS00040	237	C	T	synonymous
7096	NTHI477_RS00040	240	A	G	synonymous
7098	NTHI477_RS00040	242	G	A	nonsynonymous
7105	NTHI477_RS00040	249	A	T	nonsynonymous
7121	NTHI477_RS00040	265	G	A	nonsynonymous
7129	NTHI477_RS00040	273	A	C	synonymous
7132	NTHI477_RS00040	276	C	T	synonymous
7144	NTHI477_RS00040	288	G	A	synonymous
7147	NTHI477_RS00040	291	C	T	synonymous
7154	NTHI477_RS00040	298	C	T	synonymous
7171	NTHI477_RS00040	315	A	C	synonymous
7174	NTHI477_RS00040	318	T	C	synonymous
7177	NTHI477_RS00040	321	G	C	synonymous
7183	NTHI477_RS00040	327	T	C	synonymous
7189	NTHI477_RS00040	333	A	G	synonymous
7195	NTHI477_RS00040	339	T	C	synonymous
7198	NTHI477_RS00040	342	T	C	synonymous
7204	NTHI477_RS00040	348	T	A	synonymous
7207	NTHI477_RS00040	351	T	C	synonymous
7210	NTHI477_RS00040	354	T	C	synonymous
7213	NTHI477_RS00040	357	T	C	synonymous
7216	NTHI477_RS00040	360	C	T	synonymous
7228	NTHI477_RS00040	372	T	C	synonymous
7231	NTHI477_RS00040	375	T	C	synonymous
7252	NTHI477_RS00040	396	T	C	synonymous
7258	NTHI477_RS00040	402	C	T	synonymous
7264	NTHI477_RS00040	408	T	A	nonsynonymous
7264	NTHI477_RS00040	408	T	C	synonymous
7271	NTHI477_RS00040	415	G	T	nonsynonymous
7273	NTHI477_RS00040	417	C	A	synonymous
7276	NTHI477_RS00040	420	A	G	synonymous
7285	NTHI477_RS00040	429	G	C	synonymous
7294	NTHI477_RS00040	438	T	C	synonymous
7303	NTHI477_RS00040	447	A	T	synonymous
7306	NTHI477_RS00040	450	A	G	synonymous
7321	NTHI477_RS00040	465	C	T	synonymous
7339	NTHI477_RS00040	483	A	C	synonymous
7360	NTHI477_RS00040	504	T	C	synonymous
7372	NTHI477_RS00040	516	G	A	synonymous
7378	NTHI477_RS00040	522	T	A	synonymous
7381	NTHI477_RS00040	525	T	A	synonymous

7385	NTHI477_RS00040	529	T	C	synonymous
7390	NTHI477_RS00040	534	G	T	synonymous
7390	NTHI477_RS00040	534	G	C	synonymous
7396	NTHI477_RS00040	540	A	G	synonymous
7399	NTHI477_RS00040	543	T	C	synonymous
7405	NTHI477_RS00040	549	T	C	synonymous
7408	NTHI477_RS00040	552	G	A	synonymous
7411	NTHI477_RS00040	555	T	C	synonymous
7414	NTHI477_RS00040	558	A	T	synonymous
7417	NTHI477_RS00040	561	C	T	synonymous
7420	NTHI477_RS00040	564	T	A	synonymous
7438	NTHI477_RS00040	582	C	T	synonymous
7444	NTHI477_RS00040	588	C	G	synonymous
7444	NTHI477_RS00040	588	C	T	synonymous
7468	NTHI477_RS00040	612	A	G	synonymous
7480	NTHI477_RS00040	624	C	T	synonymous
7483	NTHI477_RS00040	627	C	T	synonymous
7489	NTHI477_RS00040	633	G	A	synonymous
7495	NTHI477_RS00040	639	G	C	synonymous
7495	NTHI477_RS00040	639	G	T	synonymous
7498	NTHI477_RS00040	642	T	C	synonymous
7516	NTHI477_RS00040	660	C	G	synonymous
7543	NTHI477_RS00040	687	G	A	synonymous
7549	NTHI477_RS00040	693	T	C	synonymous
7550	NTHI477_RS00040	694	T	C	synonymous
7567	NTHI477_RS00040	711	A	C	synonymous
7570	NTHI477_RS00040	714	G	T	synonymous
7573	NTHI477_RS00040	717	C	T	synonymous
7579	NTHI477_RS00040	723	A	T	synonymous
7588	NTHI477_RS00040	732	C	T	synonymous
7597	NTHI477_RS00040	741	T	A	synonymous
7615	NTHI477_RS00040	759	C	T	synonymous
7618	NTHI477_RS00040	762	T	G	synonymous
7627	NTHI477_RS00040	771	C	T	synonymous
7636	NTHI477_RS00040	780	C	T	synonymous
7646	NTHI477_RS00040	790	T	G	nonsynonymous
7646	NTHI477_RS00040	790	T	A	nonsynonymous
7654	intergenic	intergenic	A	C	intergenic
7654	intergenic	intergenic	A	T	intergenic
7655	intergenic	intergenic	C	T	intergenic
7661	intergenic	intergenic	T	A	intergenic
7692	intergenic	intergenic	G	T	intergenic
7700	intergenic	intergenic	A	T	intergenic
7710	intergenic	intergenic	G	T	intergenic
7736	intergenic	intergenic	G	A	intergenic

7793	NTHI477_RS00045	51	C	A	nonsynonymous
7835	NTHI477_RS00045	93	A	G	synonymous
7836	NTHI477_RS00045	94	G	A	nonsynonymous
7844	NTHI477_RS00045	102	T	C	synonymous
7862	NTHI477_RS00045	120	A	G	synonymous
7867	NTHI477_RS00045	125	C	A	nonsynonymous
7907	NTHI477_RS00045	165	C	T	synonymous
7928	NTHI477_RS00045	186	T	C	synonymous
7940	NTHI477_RS00045	198	A	G	synonymous
7968	NTHI477_RS00045	226	G	T	nonsynonymous
7969	NTHI477_RS00045	227	C	T	nonsynonymous
7976	NTHI477_RS00045	234	T	A	synonymous
7997	NTHI477_RS00045	255	T	C	synonymous
8000	NTHI477_RS00045	258	C	T	synonymous
8027	NTHI477_RS00045	285	G	T	nonsynonymous
8027	NTHI477_RS00045	285	G	C	nonsynonymous
8039	NTHI477_RS00045	297	G	T	synonymous
8040	NTHI477_RS00045	298	C	T	synonymous
8045	NTHI477_RS00045	303	G	C	synonymous
8056	NTHI477_RS00045	314	G	A	nonsynonymous
8057	NTHI477_RS00045	315	C	T	synonymous
8060	NTHI477_RS00045	318	T	C	synonymous
8066	NTHI477_RS00045	324	A	G	synonymous
8075	NTHI477_RS00045	333	T	C	synonymous
8078	NTHI477_RS00045	336	A	G	synonymous
8093	NTHI477_RS00045	351	G	T	synonymous
8093	NTHI477_RS00045	351	G	C	synonymous
8096	NTHI477_RS00045	354	A	T	synonymous
8106	NTHI477_RS00045	364	T	C	synonymous
8120	NTHI477_RS00045	378	C	T	synonymous
8124	NTHI477_RS00045	382	G	A	nonsynonymous
8133	NTHI477_RS00045	391	A	C	nonsynonymous
8143	NTHI477_RS00045	401	C	A	nonsynonymous
8153	NTHI477_RS00045	411	A	G	synonymous
8174	NTHI477_RS00045	432	C	T	synonymous
8180	NTHI477_RS00045	438	A	T	synonymous
8180	NTHI477_RS00045	438	A	C	synonymous
8189	NTHI477_RS00045	447	G	A	synonymous
8190	NTHI477_RS00045	448	T	C	nonsynonymous
8201	NTHI477_RS00045	459	A	T	synonymous
8204	NTHI477_RS00045	462	T	G	synonymous
8204	NTHI477_RS00045	462	T	C	synonymous
8207	NTHI477_RS00045	465	C	T	synonymous
8207	NTHI477_RS00045	465	C	G	nonsynonymous
8210	NTHI477_RS00045	468	T	C	synonymous

8214	NTHI477_RS00045	472	T	C	synonymous
8216	NTHI477_RS00045	474	A	T	nonsynonymous
8231	NTHI477_RS00045	489	C	T	synonymous
8237	NTHI477_RS00045	495	G	A	synonymous
8238	NTHI477_RS00045	496	G	T	nonsynonymous
8243	NTHI477_RS00045	501	C	T	synonymous
8255	NTHI477_RS00045	513	G	T	synonymous
8255	NTHI477_RS00045	513	G	C	synonymous
8266	NTHI477_RS00045	524	T	C	nonsynonymous
8273	NTHI477_RS00045	531	C	A	synonymous
8274	NTHI477_RS00045	532	G	A	nonsynonymous
8275	NTHI477_RS00045	533	C	T	nonsynonymous
8281	NTHI477_RS00045	539	A	C	nonsynonymous
8297	NTHI477_RS00045	555	T	G	nonsynonymous
8309	NTHI477_RS00045	567	C	T	synonymous
8372	NTHI477_RS00045	630	T	C	synonymous
8375	NTHI477_RS00045	633	C	T	synonymous
8393	NTHI477_RS00045	651	A	C	synonymous
8394	NTHI477_RS00045	652	G	A	nonsynonymous
8397	NTHI477_RS00045	655	C	A	nonsynonymous
8404	NTHI477_RS00045	662	G	A	nonsynonymous
8406	NTHI477_RS00045	664	A	G	nonsynonymous
8407	NTHI477_RS00045	665	C	A	nonsynonymous
8407	NTHI477_RS00045	665	C	T	nonsynonymous
8408	NTHI477_RS00045	666	A	G	synonymous
8413	NTHI477_RS00045	671	C	T	nonsynonymous
8424	NTHI477_RS00045	682	T	C	synonymous
8426	NTHI477_RS00045	684	A	C	nonsynonymous
8441	NTHI477_RS00045	699	T	C	synonymous
8444	NTHI477_RS00045	702	C	T	synonymous
8447	NTHI477_RS00045	705	A	C	synonymous
8459	NTHI477_RS00045	717	C	T	synonymous
8463	NTHI477_RS00045	721	T	C	synonymous
8470	NTHI477_RS00045	728	C	G	nonsynonymous
8471	NTHI477_RS00045	729	T	C	synonymous
8477	NTHI477_RS00045	735	T	A	synonymous
8489	NTHI477_RS00045	747	C	A	synonymous
8489	NTHI477_RS00045	747	C	G	synonymous
8525	NTHI477_RS00045	783	G	T	nonsynonymous
8543	NTHI477_RS00045	801	C	T	synonymous
8555	NTHI477_RS00045	813	C	T	synonymous
8555	NTHI477_RS00045	813	C	A	synonymous
8567	NTHI477_RS00045	825	T	C	synonymous
8570	NTHI477_RS00045	828	C	T	synonymous
8588	NTHI477_RS00045	846	C	T	synonymous

8597	NTHI477_RS00045	855	A	G	synonymous
8600	NTHI477_RS00045	858	T	G	synonymous
8603	NTHI477_RS00045	861	C	T	synonymous
8625	NTHI477_RS00045	883	G	A	nonsynonymous
8669	NTHI477_RS00045	927	A	T	synonymous
8669	NTHI477_RS00045	927	A	G	synonymous
8685	NTHI477_RS00045	943	C	T	synonymous
8689	NTHI477_RS00045	947	C	T	nonsynonymous
8693	NTHI477_RS00045	951	T	C	synonymous
8699	NTHI477_RS00045	957	A	C	nonsynonymous
8702	NTHI477_RS00045	960	G	C	synonymous
8705	NTHI477_RS00045	963	G	A	synonymous
8708	NTHI477_RS00045	966	T	C	synonymous
8711	NTHI477_RS00045	969	T	C	synonymous
8717	NTHI477_RS00045	975	A	C	nonsynonymous
8720	NTHI477_RS00045	978	G	A	synonymous
8721	NTHI477_RS00045	979	G	A	nonsynonymous
8723	NTHI477_RS00045	981	A	C	synonymous
8724	NTHI477_RS00045	982	G	A	nonsynonymous
8724	NTHI477_RS00045	982	G	C	nonsynonymous
8726	NTHI477_RS00045	984	C	T	synonymous
8726	NTHI477_RS00045	984	C	A	nonsynonymous
8729	NTHI477_RS00045	987	T	C	synonymous
8730	NTHI477_RS00045	988	G	C	nonsynonymous
8731	NTHI477_RS00045	989	C	A	nonsynonymous
8731	NTHI477_RS00045	989	C	T	nonsynonymous
8732	NTHI477_RS00045	990	A	G	synonymous
8737	NTHI477_RS00045	995	A	G	nonsynonymous
8746	NTHI477_RS00045	1004	C	T	nonsynonymous
8755	NTHI477_RS00045	1013	C	T	nonsynonymous
8756	NTHI477_RS00045	1014	A	T	synonymous
8771	NTHI477_RS00045	1029	C	T	synonymous
8775	NTHI477_RS00045	1033	C	T	synonymous
8795	NTHI477_RS00045	1053	C	A	synonymous
8795	NTHI477_RS00045	1053	C	T	synonymous
8798	NTHI477_RS00045	1056	C	T	synonymous
8801	NTHI477_RS00045	1059	T	A	synonymous
8809	NTHI477_RS00045	1067	A	G	nonsynonymous
8810	NTHI477_RS00045	1068	C	T	synonymous
8823	NTHI477_RS00045	1081	T	C	synonymous
8825	NTHI477_RS00045	1083	A	G	synonymous
8826	NTHI477_RS00045	1084	C	T	nonsynonymous
8828	NTHI477_RS00045	1086	C	T	synonymous
8840	NTHI477_RS00045	1098	A	G	synonymous
8843	NTHI477_RS00045	1101	C	A	synonymous

8843	NTHI477_RS00045	1101	C	T	synonymous
8849	NTHI477_RS00045	1107	T	C	synonymous
8870	NTHI477_RS00045	1128	C	T	synonymous
8883	NTHI477_RS00045	1141	G	C	nonsynonymous
8912	NTHI477_RS00045	1170	C	T	synonymous
8942	NTHI477_RS00045	1200	T	C	synonymous
8954	NTHI477_RS00045	1212	C	T	synonymous
8957	NTHI477_RS00045	1215	C	T	synonymous
8960	NTHI477_RS00045	1218	A	C	synonymous
8966	NTHI477_RS00045	1224	T	C	synonymous
8970	NTHI477_RS00045	1228	C	G	nonsynonymous
8976	NTHI477_RS00045	1234	C	A	nonsynonymous
8978	NTHI477_RS00045	1236	G	C	nonsynonymous
8996	NTHI477_RS00045	1254	C	T	synonymous
9002	NTHI477_RS00045	1260	C	T	synonymous
9005	NTHI477_RS00045	1263	C	T	synonymous
9025	NTHI477_RS00045	1283	T	A	nonsynonymous
9032	NTHI477_RS00045	1290	G	A	synonymous
9035	NTHI477_RS00045	1293	C	T	synonymous
9038	NTHI477_RS00045	1296	A	G	synonymous
9041	NTHI477_RS00045	1299	C	T	synonymous
9042	NTHI477_RS00045	1300	C	A	nonsynonymous
9055	NTHI477_RS00045	1313	C	T	nonsynonymous
9065	NTHI477_RS00045	1323	C	T	synonymous
9078	NTHI477_RS00045	1336	C	A	nonsynonymous
9080	NTHI477_RS00045	1338	A	C	nonsynonymous
9081	NTHI477_RS00045	1339	A	G	nonsynonymous
9083	NTHI477_RS00045	1341	T	A	synonymous
9108	NTHI477_RS00045	1366	G	A	nonsynonymous
9122	NTHI477_RS00045	1380	C	A	synonymous
9146	NTHI477_RS00045	1404	C	T	synonymous
9152	NTHI477_RS00045	1410	T	C	synonymous
9155	NTHI477_RS00045	1413	T	C	synonymous
9170	NTHI477_RS00045	1428	C	T	synonymous
9177	NTHI477_RS00045	1435	T	G	nonsynonymous
9181	NTHI477_RS00045	1439	A	C	nonsynonymous
9182	NTHI477_RS00045	1440	A	C	nonsynonymous
9185	NTHI477_RS00045	1443	G	A	synonymous
9188	NTHI477_RS00045	1446	T	C	synonymous
9189	NTHI477_RS00045	1447	T	C	synonymous
9206	NTHI477_RS00045	1464	T	C	synonymous
9210	NTHI477_RS00045	1468	G	T	nonsynonymous
9231	NTHI477_RS00045	1489	G	T	nonsynonymous
9242	NTHI477_RS00045	1500	T	C	synonymous
9245	NTHI477_RS00045	1503	T	G	synonymous

9248	NTHI477_RS00045	1506	T	G	synonymous
9251	NTHI477_RS00045	1509	T	G	synonymous
9252	NTHI477_RS00045	1510	T	C	synonymous
9255	NTHI477_RS00045	1513	G	A	nonsynonymous
9257	NTHI477_RS00045	1515	A	G	synonymous
9257	NTHI477_RS00045	1515	A	T	nonsynonymous
9260	NTHI477_RS00045	1518	C	T	synonymous
9263	NTHI477_RS00045	1521	T	C	synonymous
9278	NTHI477_RS00045	1536	G	C	synonymous
9281	NTHI477_RS00045	1539	C	A	synonymous
9284	NTHI477_RS00045	1542	C	T	synonymous
9287	NTHI477_RS00045	1545	C	T	synonymous
9296	NTHI477_RS00045	1554	A	T	synonymous
9299	NTHI477_RS00045	1557	T	C	synonymous
9332	NTHI477_RS00045	1590	G	T	synonymous
9333	NTHI477_RS00045	1591	G	T	nonsynonymous
9335	NTHI477_RS00045	1593	A	C	synonymous
9336	NTHI477_RS00045	1594	A	C	nonsynonymous
9346	NTHI477_RS00045	1604	A	G	nonsynonymous
9347	NTHI477_RS00045	1605	T	C	synonymous
9354	NTHI477_RS00045	1612	A	C	nonsynonymous
9357	NTHI477_RS00045	1615	C	A	nonsynonymous
9361	NTHI477_RS00045	1619	A	G	nonsynonymous
9365	NTHI477_RS00045	1623	C	T	synonymous
9365	NTHI477_RS00045	1623	C	A	nonsynonymous
9368	NTHI477_RS00045	1626	G	A	synonymous
9368	NTHI477_RS00045	1626	G	T	synonymous
9373	NTHI477_RS00045	1631	C	T	nonsynonymous
9386	NTHI477_RS00045	1644	G	A	synonymous
9389	NTHI477_RS00045	1647	T	C	synonymous
9389	NTHI477_RS00045	1647	T	A	synonymous
9392	NTHI477_RS00045	1650	C	T	synonymous
9407	NTHI477_RS00045	1665	C	A	synonymous
9407	NTHI477_RS00045	1665	C	T	synonymous
9410	NTHI477_RS00045	1668	A	G	synonymous
9410	NTHI477_RS00045	1668	A	C	synonymous
9425	NTHI477_RS00045	1683	A	C	synonymous
9440	NTHI477_RS00045	1698	C	T	synonymous
9443	NTHI477_RS00045	1701	T	C	synonymous
9450	NTHI477_RS00045	1708	G	T	nonsynonymous
9452	NTHI477_RS00045	1710	A	T	synonymous
9452	NTHI477_RS00045	1710	A	G	synonymous
9453	NTHI477_RS00045	1711	C	G	nonsynonymous
9454	NTHI477_RS00045	1712	C	A	nonsynonymous
9455	NTHI477_RS00045	1713	G	T	synonymous

9455	NTHI477_RS00045	1713	G	C	synonymous
9464	NTHI477_RS00045	1722	T	A	synonymous
9465	NTHI477_RS00045	1723	G	T	nonsynonymous
9466	NTHI477_RS00045	1724	A	C	nonsynonymous
9472	NTHI477_RS00045	1730	T	C	nonsynonymous
9473	NTHI477_RS00045	1731	G	T	synonymous
9480	NTHI477_RS00045	1738	A	G	nonsynonymous
9482	NTHI477_RS00045	1740	T	G	nonsynonymous
9485	NTHI477_RS00045	1743	A	G	synonymous
9500	NTHI477_RS00045	1758	A	G	synonymous
9506	NTHI477_RS00045	1764	T	G	synonymous
9508	NTHI477_RS00045	1766	G	C	nonsynonymous
9509	NTHI477_RS00045	1767	T	A	synonymous
9512	NTHI477_RS00045	1770	A	C	synonymous
9515	NTHI477_RS00045	1773	A	G	synonymous
9518	NTHI477_RS00045	1776	T	C	synonymous
9521	NTHI477_RS00045	1779	G	A	synonymous
9525	NTHI477_RS00045	1783	T	C	synonymous
9527	NTHI477_RS00045	1785	A	G	synonymous
9528	NTHI477_RS00045	1786	T	C	synonymous
9530	NTHI477_RS00045	1788	A	C	nonsynonymous
9539	NTHI477_RS00045	1797	C	T	synonymous
9542	NTHI477_RS00045	1800	C	A	synonymous
9545	NTHI477_RS00045	1803	A	G	synonymous
9546	NTHI477_RS00045	1804	A	C	nonsynonymous
9548	NTHI477_RS00045	1806	G	A	nonsynonymous
9551	NTHI477_RS00045	1809	T	C	synonymous
9554	NTHI477_RS00045	1812	A	T	synonymous
9560	NTHI477_RS00045	1818	G	T	synonymous
9561	NTHI477_RS00045	1819	T	G	nonsynonymous
9566	NTHI477_RS00045	1824	T	C	synonymous
9569	NTHI477_RS00045	1827	A	G	synonymous
9572	NTHI477_RS00045	1830	T	C	synonymous
9575	NTHI477_RS00045	1833	T	C	synonymous
9578	NTHI477_RS00045	1836	T	C	synonymous
9590	NTHI477_RS00045	1848	G	A	nonsynonymous
9593	NTHI477_RS00045	1851	G	A	synonymous
9594	NTHI477_RS00045	1852	G	C	nonsynonymous
9596	NTHI477_RS00045	1854	G	A	synonymous
9599	NTHI477_RS00045	1857	T	C	synonymous
9600	NTHI477_RS00045	1858	T	C	nonsynonymous
9602	NTHI477_RS00045	1860	A	T	synonymous
9608	NTHI477_RS00045	1866	G	A	synonymous
9609	NTHI477_RS00045	1867	A	C	nonsynonymous
9611	NTHI477_RS00045	1869	T	C	synonymous

9614	NTHI477_RS00045	1872	C	T	synonymous
9620	NTHI477_RS00045	1878	T	C	synonymous
9623	NTHI477_RS00045	1881	T	A	synonymous
9629	NTHI477_RS00045	1887	C	T	synonymous
9632	NTHI477_RS00045	1890	A	C	nonsynonymous
9636	NTHI477_RS00045	1894	A	C	nonsynonymous
9637	NTHI477_RS00045	1895	C	G	nonsynonymous
9641	NTHI477_RS00045	1899	C	T	synonymous
9647	NTHI477_RS00045	1905	A	C	synonymous
9650	NTHI477_RS00045	1908	C	G	synonymous
9650	NTHI477_RS00045	1908	C	A	synonymous
9653	NTHI477_RS00045	1911	C	T	synonymous
9654	NTHI477_RS00045	1912	A	T	nonsynonymous
9656	NTHI477_RS00045	1914	T	A	synonymous
9656	NTHI477_RS00045	1914	T	G	nonsynonymous
9659	NTHI477_RS00045	1917	G	A	synonymous
9665	NTHI477_RS00045	1923	G	A	synonymous
9674	NTHI477_RS00045	1932	A	C	synonymous
9677	NTHI477_RS00045	1935	A	G	synonymous
9680	NTHI477_RS00045	1938	G	T	synonymous
9681	NTHI477_RS00045	1939	A	C	nonsynonymous
9682	NTHI477_RS00045	1940	A	G	nonsynonymous
9687	NTHI477_RS00045	1945	G	A	nonsynonymous
9688	NTHI477_RS00045	1946	C	A	nonsynonymous
9689	NTHI477_RS00045	1947	A	C	synonymous
9692	NTHI477_RS00045	1950	T	A	synonymous
9695	NTHI477_RS00045	1953	C	T	synonymous
9698	NTHI477_RS00045	1956	T	C	synonymous
9702	NTHI477_RS00045	1960	G	A	nonsynonymous
9704	NTHI477_RS00045	1962	G	C	synonymous
9707	NTHI477_RS00045	1965	C	T	synonymous
9713	NTHI477_RS00045	1971	A	C	synonymous
9714	NTHI477_RS00045	1972	C	G	nonsynonymous
9763	intergenic	intergenic	G	A	intergenic
9768	intergenic	intergenic	C	T	intergenic
9799	intergenic	intergenic	T	A	intergenic
9810	intergenic	intergenic	C	T	intergenic
9821	intergenic	intergenic	G	A	intergenic
9826	intergenic	intergenic	A	G	intergenic
9826	intergenic	intergenic	A	T	intergenic
9834	intergenic	intergenic	C	A	intergenic
9845	intergenic	intergenic	C	T	intergenic
9851	intergenic	intergenic	A	T	intergenic
9861	intergenic	intergenic	G	T	intergenic
9866	intergenic	intergenic	A	T	intergenic

9867	intergenic	intergenic	C	T	intergenic
9878	intergenic	intergenic	C	A	intergenic
9912	intergenic	intergenic	C	T	intergenic
9926	intergenic	intergenic	T	C	intergenic
9957	intergenic	intergenic	C	T	intergenic
9974	intergenic	intergenic	A	C	intergenic
9975	intergenic	intergenic	G	A	intergenic
9981	intergenic	intergenic	C	A	intergenic
10007	NTHI477_RS00050	24	A	G	synonymous
10013	NTHI477_RS00050	30	G	A	synonymous
10068	NTHI477_RS00050	85	G	A	nonsynonymous
10070	NTHI477_RS00050	87	G	A	synonymous
10073	NTHI477_RS00050	90	T	C	synonymous
10115	NTHI477_RS00050	132	C	A	synonymous
10157	NTHI477_RS00050	174	G	A	synonymous
10169	NTHI477_RS00050	186	G	A	synonymous
10202	NTHI477_RS00050	219	G	A	synonymous
10226	NTHI477_RS00050	243	G	A	synonymous
10229	NTHI477_RS00050	246	T	C	synonymous
10259	NTHI477_RS00050	276	A	G	synonymous
10280	NTHI477_RS00050	297	T	C	synonymous
10286	NTHI477_RS00050	303	A	C	synonymous
10301	NTHI477_RS00050	318	G	T	nonsynonymous
10301	NTHI477_RS00050	318	G	C	nonsynonymous
10304	NTHI477_RS00050	321	A	G	synonymous
10311	NTHI477_RS00050	328	A	G	nonsynonymous
10316	NTHI477_RS00050	333	G	A	synonymous
10364	NTHI477_RS00050	381	C	T	synonymous
10373	NTHI477_RS00050	390	G	A	synonymous
10376	NTHI477_RS00050	393	C	T	synonymous
10406	NTHI477_RS00050	423	T	C	synonymous
10418	NTHI477_RS00050	435	G	A	synonymous
10421	NTHI477_RS00050	438	T	C	synonymous
10424	NTHI477_RS00050	441	C	T	synonymous
10427	NTHI477_RS00050	444	C	G	synonymous
10428	NTHI477_RS00050	445	A	T	nonsynonymous
10435	NTHI477_RS00050	452	G	T	nonsynonymous
10437	NTHI477_RS00050	454	A	G	nonsynonymous
10441	NTHI477_RS00050	458	T	G	nonsynonymous
10449	NTHI477_RS00050	466	G	T	nonsynonymous
10451	NTHI477_RS00050	468	T	G	synonymous
10455	NTHI477_RS00050	472	T	G	nonsynonymous
10459	NTHI477_RS00050	476	T	G	nonsynonymous
10464	NTHI477_RS00050	481	A	C	nonsynonymous
10469	NTHI477_RS00050	486	G	T	synonymous

10472	NTHI477_RS00050	489	T	G	synonymous
10472	NTHI477_RS00050	489	T	C	synonymous
10473	NTHI477_RS00050	490	G	A	nonsynonymous
10474	NTHI477_RS00050	491	C	T	nonsynonymous
10475	NTHI477_RS00050	492	G	A	synonymous
10486	NTHI477_RS00050	503	T	G	nonsynonymous
10487	NTHI477_RS00050	504	T	G	nonsynonymous
10497	NTHI477_RS00050	514	T	G	nonsynonymous
10502	NTHI477_RS00050	519	G	T	synonymous
10502	NTHI477_RS00050	519	G	A	synonymous
10535	NTHI477_RS00050	552	A	G	synonymous
10586	NTHI477_RS00050	603	C	T	synonymous
10591	NTHI477_RS00050	608	C	T	nonsynonymous
10610	NTHI477_RS00050	627	T	G	nonsynonymous
10631	NTHI477_RS00055	10	A	G	nonsynonymous
10633	NTHI477_RS00055	12	T	G	nonsynonymous
10643	NTHI477_RS00055	22	A	C	nonsynonymous
10650	NTHI477_RS00055	29	G	A	nonsynonymous
10651	NTHI477_RS00055	30	C	T	synonymous
10654	NTHI477_RS00055	33	C	G	synonymous
10657	NTHI477_RS00055	36	C	T	synonymous
10672	NTHI477_RS00055	51	G	A	synonymous
10696	NTHI477_RS00055	75	C	T	synonymous
10729	NTHI477_RS00055	108	A	C	synonymous
10735	NTHI477_RS00055	114	C	T	synonymous
10738	NTHI477_RS00055	117	A	G	synonymous
10777	NTHI477_RS00055	156	C	A	synonymous
10777	NTHI477_RS00055	156	C	T	synonymous
10798	NTHI477_RS00055	177	T	C	synonymous
10820	NTHI477_RS00055	199	G	A	nonsynonymous
10840	NTHI477_RS00055	219	T	A	synonymous
10860	NTHI477_RS00055	239	T	C	nonsynonymous
10876	NTHI477_RS00055	255	C	T	synonymous
10902	NTHI477_RS00055	281	T	C	nonsynonymous
10928	NTHI477_RS00055	307	A	G	nonsynonymous
10930	NTHI477_RS00055	309	A	C	synonymous
10954	NTHI477_RS00055	333	T	C	synonymous
10963	NTHI477_RS00055	342	T	G	nonsynonymous
10972	NTHI477_RS00055	351	T	C	synonymous
10998	NTHI477_RS00055	377	A	C	nonsynonymous
11005	NTHI477_RS00055	384	C	T	synonymous
11008	NTHI477_RS00055	387	C	T	synonymous
11024	NTHI477_RS00055	403	G	C	nonsynonymous
11024	NTHI477_RS00055	403	G	A	nonsynonymous
11044	NTHI477_RS00055	423	G	A	synonymous

11057	NTHI477_RS00055	436	G	A	nonsynonymous
11059	NTHI477_RS00055	438	G	A	synonymous
11068	NTHI477_RS00055	447	A	G	synonymous
11079	NTHI477_RS00055	458	C	A	nonsynonymous
11111	NTHI477_RS00055	490	A	G	nonsynonymous
11116	NTHI477_RS00055	495	A	G	synonymous
11119	NTHI477_RS00055	498	G	C	synonymous
11128	NTHI477_RS00055	507	T	G	synonymous
11129	NTHI477_RS00055	508	C	A	synonymous
11131	NTHI477_RS00055	510	A	C	synonymous
11170	NTHI477_RS00055	549	G	A	synonymous
11188	NTHI477_RS00055	567	A	G	synonymous
11198	NTHI477_RS00055	577	T	G	nonsynonymous
11218	NTHI477_RS00055	597	G	A	synonymous
11227	NTHI477_RS00055	606	C	T	synonymous
11242	NTHI477_RS00055	621	T	G	synonymous
11277	NTHI477_RS00055	656	A	G	nonsynonymous
11316	NTHI477_RS00055	695	G	C	nonsynonymous
11338	NTHI477_RS00055	717	C	T	synonymous
11359	NTHI477_RS00055	738	G	T	synonymous
11392	NTHI477_RS00055	771	T	C	synonymous
11409	NTHI477_RS00055	788	C	T	nonsynonymous
11410	NTHI477_RS00055	789	C	T	synonymous
11421	NTHI477_RS00055	800	C	T	nonsynonymous
11434	NTHI477_RS00055	813	G	A	synonymous
11482	NTHI477_RS00055	861	T	G	synonymous
11495	NTHI477_RS00055	874	T	C	nonsynonymous
11534	NTHI477_RS00055	913	A	G	nonsynonymous
11597	NTHI477_RS00060	60	G	A	synonymous
11627	NTHI477_RS00060	90	C	T	synonymous
11630	NTHI477_RS00060	93	T	C	synonymous
11648	NTHI477_RS00060	111	C	T	synonymous
11671	NTHI477_RS00060	134	T	G	nonsynonymous
11679	NTHI477_RS00060	142	G	T	nonsynonymous
11705	NTHI477_RS00060	168	T	C	synonymous
11708	NTHI477_RS00060	171	G	C	synonymous
11708	NTHI477_RS00060	171	G	A	synonymous
11711	NTHI477_RS00060	174	C	T	synonymous
11721	NTHI477_RS00060	184	T	C	nonsynonymous
11725	NTHI477_RS00060	188	A	T	nonsynonymous
11729	NTHI477_RS00060	192	A	C	synonymous
11764	NTHI477_RS00060	227	C	T	nonsynonymous
11768	NTHI477_RS00060	231	G	A	synonymous
11777	NTHI477_RS00060	240	C	T	synonymous
11780	NTHI477_RS00060	243	C	A	synonymous

11781	NTHI477_RS00060	244	C	T	nonsynonymous
11784	NTHI477_RS00060	247	T	G	nonsynonymous
11798	NTHI477_RS00060	261	A	G	synonymous
11801	NTHI477_RS00060	264	C	G	synonymous
11801	NTHI477_RS00060	264	C	T	synonymous
11813	NTHI477_RS00060	276	A	G	synonymous
11822	NTHI477_RS00060	285	T	A	nonsynonymous
11833	NTHI477_RS00060	296	C	T	nonsynonymous
11847	NTHI477_RS00060	310	G	A	nonsynonymous
11852	NTHI477_RS00060	315	A	G	synonymous
11879	NTHI477_RS00060	342	G	A	synonymous
11884	NTHI477_RS00060	347	A	C	nonsynonymous
11917	NTHI477_RS00060	380	C	G	nonsynonymous
11918	NTHI477_RS00060	381	T	C	synonymous
11960	NTHI477_RS00060	423	G	A	synonymous
11963	NTHI477_RS00060	426	T	C	synonymous
11969	NTHI477_RS00060	432	G	A	synonymous
11977	NTHI477_RS00060	440	G	A	nonsynonymous
11978	NTHI477_RS00060	441	T	C	synonymous
11994	NTHI477_RS00060	457	G	A	nonsynonymous
11995	NTHI477_RS00060	458	G	A	nonsynonymous
11996	NTHI477_RS00060	459	G	A	synonymous
12005	NTHI477_RS00060	468	C	T	synonymous
12005	NTHI477_RS00060	468	C	G	synonymous
12017	NTHI477_RS00060	480	G	T	synonymous
12023	NTHI477_RS00060	486	T	C	synonymous
12047	NTHI477_RS00060	510	A	G	synonymous
12053	NTHI477_RS00060	516	A	G	synonymous
12071	NTHI477_RS00060	534	A	T	synonymous
12083	NTHI477_RS00060	546	G	A	synonymous
12084	NTHI477_RS00060	547	G	A	nonsynonymous
12085	NTHI477_RS00060	548	T	A	nonsynonymous
12101	NTHI477_RS00060	564	C	T	synonymous
12121	NTHI477_RS00060	584	T	C	nonsynonymous
12149	NTHI477_RS00060	612	G	T	nonsynonymous
12161	NTHI477_RS00060	624	C	T	synonymous
12165	NTHI477_RS00060	628	T	C	nonsynonymous
12175	NTHI477_RS00060	638	C	T	nonsynonymous
12215	NTHI477_RS00060	678	A	G	synonymous
12218	NTHI477_RS00060	681	C	T	synonymous
12218	NTHI477_RS00060	681	C	A	nonsynonymous
12225	NTHI477_RS00060	688	G	A	nonsynonymous
12271	NTHI477_RS00060	734	C	T	nonsynonymous
12297	intergenic	intergenic	A	C	intergenic
12360	NTHI477_RS00065	42	A	G	synonymous

12384	NTHI477_RS00065	66	T	C	synonymous
12387	NTHI477_RS00065	69	T	A	synonymous
12390	NTHI477_RS00065	72	G	C	synonymous
12390	NTHI477_RS00065	72	G	T	synonymous
12393	NTHI477_RS00065	75	T	C	synonymous
12396	NTHI477_RS00065	78	T	G	synonymous
12396	NTHI477_RS00065	78	T	A	synonymous
12411	NTHI477_RS00065	93	C	T	synonymous
12423	NTHI477_RS00065	105	C	A	synonymous
12438	NTHI477_RS00065	120	A	C	synonymous
12438	NTHI477_RS00065	120	A	G	synonymous
12442	NTHI477_RS00065	124	T	G	nonsynonymous
12449	NTHI477_RS00065	131	T	G	nonsynonymous
12461	NTHI477_RS00065	143	T	G	nonsynonymous
12483	NTHI477_RS00065	165	T	G	synonymous
12516	NTHI477_RS00065	198	G	A	synonymous
12528	NTHI477_RS00065	210	C	T	synonymous
12531	NTHI477_RS00065	213	A	G	synonymous
12582	NTHI477_RS00065	264	G	A	synonymous
12583	NTHI477_RS00065	265	T	C	synonymous
12600	NTHI477_RS00065	282	A	G	synonymous
12601	NTHI477_RS00065	283	C	T	synonymous
12612	NTHI477_RS00065	294	C	T	synonymous
12618	NTHI477_RS00065	300	T	C	synonymous
12630	NTHI477_RS00065	312	C	T	synonymous
12660	NTHI477_RS00065	342	T	C	synonymous
12663	NTHI477_RS00065	345	C	A	synonymous
12669	NTHI477_RS00065	351	C	A	synonymous
12675	NTHI477_RS00065	357	G	A	nonsynonymous
12683	NTHI477_RS00065	365	T	C	nonsynonymous
12686	NTHI477_RS00065	368	T	G	nonsynonymous
12690	NTHI477_RS00065	372	C	T	synonymous
12729	NTHI477_RS00065	411	A	G	synonymous
12733	NTHI477_RS00065	415	T	G	nonsynonymous
12745	NTHI477_RS00065	427	C	A	nonsynonymous
12753	NTHI477_RS00065	435	A	G	synonymous
12777	NTHI477_RS00065	459	C	T	synonymous
12780	NTHI477_RS00065	462	A	G	synonymous
12786	NTHI477_RS00065	468	G	A	synonymous
12810	NTHI477_RS00065	492	G	A	synonymous
12813	NTHI477_RS00065	495	G	A	synonymous
12828	NTHI477_RS00065	510	A	G	synonymous
12831	NTHI477_RS00065	513	C	G	synonymous
12831	NTHI477_RS00065	513	C	A	synonymous
12853	NTHI477_RS00065	535	C	A	nonsynonymous

12864	NTHI477_RS00065	546	C	T	synonymous
12876	NTHI477_RS00065	558	T	C	synonymous
12888	NTHI477_RS00065	570	A	G	synonymous
12900	NTHI477_RS00065	582	C	T	synonymous
12924	NTHI477_RS00065	606	A	G	synonymous
12930	NTHI477_RS00065	612	C	T	synonymous
12942	NTHI477_RS00065	624	G	T	synonymous
12966	NTHI477_RS00065	648	G	A	nonsynonymous
12990	NTHI477_RS00065	672	T	A	synonymous
12996	NTHI477_RS00065	678	T	C	synonymous
13014	NTHI477_RS00065	696	G	A	synonymous
13021	NTHI477_RS00065	703	A	G	nonsynonymous
13041	NTHI477_RS00065	723	A	G	synonymous
13047	NTHI477_RS00065	729	C	T	synonymous
13053	NTHI477_RS00065	735	G	C	synonymous
13140	NTHI477_RS00065	822	G	A	synonymous
13143	NTHI477_RS00065	825	G	A	synonymous
13155	NTHI477_RS00065	837	A	T	synonymous
13162	NTHI477_RS00065	844	A	G	nonsynonymous
13164	NTHI477_RS00065	846	C	T	synonymous
13170	NTHI477_RS00065	852	G	T	synonymous
13191	NTHI477_RS00065	873	C	T	synonymous
13218	NTHI477_RS00065	900	C	T	synonymous
13224	NTHI477_RS00065	906	C	T	synonymous
13257	NTHI477_RS00065	939	C	T	synonymous
13275	NTHI477_RS00065	957	T	C	synonymous
13290	NTHI477_RS00065	972	T	A	synonymous
13293	NTHI477_RS00065	975	G	A	synonymous
13299	NTHI477_RS00065	981	A	G	synonymous
13317	NTHI477_RS00065	999	T	C	synonymous
13345	NTHI477_RS00065	1027	C	T	synonymous
13350	NTHI477_RS00065	1032	A	G	synonymous
13377	NTHI477_RS00065	1059	C	T	synonymous
13383	NTHI477_RS00065	1065	T	C	synonymous
13384	NTHI477_RS00065	1066	C	A	synonymous
13389	NTHI477_RS00065	1071	C	T	synonymous
13418	NTHI477_RS00065	1100	T	C	nonsynonymous
13492	NTHI477_RS00065	1174	G	A	nonsynonymous
13498	NTHI477_RS00065	1180	A	C	nonsynonymous
13503	NTHI477_RS00065	1185	T	G	nonsynonymous
13515	intergenic	intergenic	G	A	intergenic
13516	intergenic	intergenic	A	C	intergenic
13530	intergenic	intergenic	G	A	intergenic
13559	intergenic	intergenic	A	G	intergenic
13567	intergenic	intergenic	A	G	intergenic

13595	NTHI477_RS00070	1329	T	C	synonymous
13598	NTHI477_RS00070	1326	A	G	synonymous
13610	NTHI477_RS00070	1314	T	C	synonymous
13615	NTHI477_RS00070	1309	C	T	nonsynonymous
13627	NTHI477_RS00070	1297	C	T	nonsynonymous
13632	NTHI477_RS00070	1292	T	C	nonsynonymous
13649	NTHI477_RS00070	1275	T	C	synonymous
13658	NTHI477_RS00070	1266	G	A	synonymous
13676	NTHI477_RS00070	1248	A	G	synonymous
13679	NTHI477_RS00070	1245	A	T	synonymous
13682	NTHI477_RS00070	1242	C	T	synonymous
13700	NTHI477_RS00070	1224	G	T	synonymous
13723	NTHI477_RS00070	1201	T	C	nonsynonymous
13724	NTHI477_RS00070	1200	A	T	synonymous
13736	NTHI477_RS00070	1188	C	T	synonymous
13757	NTHI477_RS00070	1167	A	G	synonymous
13766	NTHI477_RS00070	1158	T	C	synonymous
13767	NTHI477_RS00070	1157	T	G	nonsynonymous
13789	NTHI477_RS00070	1135	G	C	nonsynonymous
13790	NTHI477_RS00070	1134	T	C	synonymous
13805	NTHI477_RS00070	1119	A	G	synonymous
13808	NTHI477_RS00070	1116	T	A	synonymous
13832	NTHI477_RS00070	1092	C	T	synonymous
13838	NTHI477_RS00070	1086	G	C	synonymous
13877	NTHI477_RS00070	1047	T	A	synonymous
13891	NTHI477_RS00070	1033	A	G	synonymous
13898	NTHI477_RS00070	1026	C	T	synonymous
13919	NTHI477_RS00070	1005	A	C	synonymous
13925	NTHI477_RS00070	999	A	G	synonymous
13931	NTHI477_RS00070	993	C	T	synonymous
13937	NTHI477_RS00070	987	A	G	synonymous
13943	NTHI477_RS00070	981	A	G	synonymous
13946	NTHI477_RS00070	978	A	G	synonymous
14015	NTHI477_RS00070	909	A	G	synonymous
14021	NTHI477_RS00070	903	C	T	synonymous
14033	NTHI477_RS00070	891	C	T	synonymous
14036	NTHI477_RS00070	888	T	C	synonymous
14057	NTHI477_RS00070	867	C	A	synonymous
14078	NTHI477_RS00070	846	G	A	synonymous
14093	NTHI477_RS00070	831	A	T	synonymous
14108	NTHI477_RS00070	816	G	A	synonymous
14114	NTHI477_RS00070	810	T	C	synonymous
14114	NTHI477_RS00070	810	T	A	synonymous
14117	NTHI477_RS00070	807	G	A	synonymous
14120	NTHI477_RS00070	804	A	G	synonymous

14129	NTHI477_RS00070	795	T	G	synonymous
14135	NTHI477_RS00070	789	T	C	synonymous
14143	NTHI477_RS00070	781	G	T	nonsynonymous
14150	NTHI477_RS00070	774	C	A	synonymous
14150	NTHI477_RS00070	774	C	T	synonymous
14163	NTHI477_RS00070	761	T	C	nonsynonymous
14168	NTHI477_RS00070	756	G	A	synonymous
14171	NTHI477_RS00070	753	A	G	synonymous
14186	NTHI477_RS00070	738	C	T	synonymous
14186	NTHI477_RS00070	738	C	G	synonymous
14189	NTHI477_RS00070	735	G	A	synonymous
14192	NTHI477_RS00070	732	A	T	nonsynonymous
14204	NTHI477_RS00070	720	G	A	synonymous
14206	NTHI477_RS00070	718	C	T	nonsynonymous
14207	NTHI477_RS00070	717	T	G	synonymous
14219	NTHI477_RS00070	705	A	T	synonymous
14231	NTHI477_RS00070	693	A	T	synonymous
14234	NTHI477_RS00070	690	C	A	nonsynonymous
14237	NTHI477_RS00070	687	T	G	nonsynonymous
14252	NTHI477_RS00070	672	G	T	synonymous
14264	NTHI477_RS00070	660	C	A	synonymous
14282	NTHI477_RS00070	642	T	G	synonymous
14282	NTHI477_RS00070	642	T	A	synonymous
14315	NTHI477_RS00070	609	A	G	synonymous
14318	NTHI477_RS00070	606	G	A	synonymous
14324	NTHI477_RS00070	600	G	A	synonymous
14324	NTHI477_RS00070	600	G	T	synonymous
14372	NTHI477_RS00070	552	A	G	synonymous
14381	NTHI477_RS00070	543	A	G	synonymous
14390	NTHI477_RS00070	534	A	G	synonymous
14396	NTHI477_RS00070	528	A	G	synonymous
14442	NTHI477_RS00070	482	A	G	nonsynonymous
14447	NTHI477_RS00070	477	T	C	synonymous
14474	NTHI477_RS00070	450	C	T	synonymous
14480	NTHI477_RS00070	444	C	T	synonymous
14489	NTHI477_RS00070	435	A	G	synonymous
14507	NTHI477_RS00070	417	T	C	synonymous
14531	NTHI477_RS00070	393	G	T	synonymous
14576	NTHI477_RS00070	348	T	A	synonymous
14579	NTHI477_RS00070	345	A	G	synonymous
14588	NTHI477_RS00070	336	G	T	synonymous
14591	NTHI477_RS00070	333	T	C	synonymous
14594	NTHI477_RS00070	330	T	A	synonymous
14597	NTHI477_RS00070	327	G	T	synonymous
14600	NTHI477_RS00070	324	G	A	synonymous

14609	NTHI477_RS00070	315	A	T	synonymous
14612	NTHI477_RS00070	312	C	A	synonymous
14612	NTHI477_RS00070	312	C	T	synonymous
14615	NTHI477_RS00070	309	T	C	synonymous
14615	NTHI477_RS00070	309	T	G	synonymous
14618	NTHI477_RS00070	306	A	G	synonymous
14621	NTHI477_RS00070	303	G	A	synonymous
14624	NTHI477_RS00070	300	G	A	synonymous
14627	NTHI477_RS00070	297	A	G	synonymous
14633	NTHI477_RS00070	291	G	T	synonymous
14642	NTHI477_RS00070	282	A	C	synonymous
14657	NTHI477_RS00070	267	A	G	synonymous
14678	NTHI477_RS00070	246	G	T	synonymous
14678	NTHI477_RS00070	246	G	A	synonymous
14681	NTHI477_RS00070	243	T	C	synonymous
14684	NTHI477_RS00070	240	A	G	synonymous
14693	NTHI477_RS00070	231	T	C	synonymous
14696	NTHI477_RS00070	228	A	T	synonymous
14698	NTHI477_RS00070	226	A	C	nonsynonymous
14699	NTHI477_RS00070	225	G	A	synonymous
14702	NTHI477_RS00070	222	G	A	synonymous
14714	NTHI477_RS00070	210	T	G	synonymous
14717	NTHI477_RS00070	207	A	C	synonymous
14726	NTHI477_RS00070	198	A	T	synonymous
14737	NTHI477_RS00070	187	C	T	nonsynonymous
14741	NTHI477_RS00070	183	G	T	synonymous
14759	NTHI477_RS00070	165	A	G	synonymous
14768	NTHI477_RS00070	156	A	G	synonymous
14792	NTHI477_RS00070	132	A	G	synonymous
14795	NTHI477_RS00070	129	G	A	synonymous
14801	NTHI477_RS00070	123	G	A	synonymous
14810	NTHI477_RS00070	114	C	T	synonymous
14813	NTHI477_RS00070	111	T	C	synonymous
14816	NTHI477_RS00070	108	G	A	synonymous
14846	NTHI477_RS00070	78	A	G	synonymous
14849	NTHI477_RS00070	75	A	G	synonymous
14852	NTHI477_RS00070	72	G	A	synonymous
14858	NTHI477_RS00070	66	A	G	synonymous
14861	NTHI477_RS00070	63	A	G	synonymous
14876	NTHI477_RS00070	48	C	T	synonymous
14879	NTHI477_RS00070	45	T	C	synonymous
14888	NTHI477_RS00070	36	T	A	synonymous
14900	NTHI477_RS00070	24	A	G	synonymous
14901	NTHI477_RS00070	23	T	C	nonsynonymous
14908	NTHI477_RS00070	16	G	A	synonymous

14947	intergenic	intergenic	A	G	intergenic
14970	intergenic	intergenic	G	A	intergenic
14981	intergenic	intergenic	T	G	intergenic
14981	intergenic	intergenic	T	A	intergenic
14984	intergenic	intergenic	G	T	intergenic
14988	intergenic	intergenic	G	A	intergenic
14990	intergenic	intergenic	G	A	intergenic
15009	intergenic	intergenic	C	G	intergenic
15020	intergenic	intergenic	G	A	intergenic
15025	intergenic	intergenic	G	A	intergenic
15027	intergenic	intergenic	G	A	intergenic
15035	intergenic	intergenic	A	G	intergenic
15052	intergenic	intergenic	A	G	intergenic
15053	intergenic	intergenic	G	T	intergenic
15054	intergenic	intergenic	C	A	intergenic
15055	intergenic	intergenic	T	A	intergenic
15057	intergenic	intergenic	T	A	intergenic
15058	intergenic	intergenic	G	A	intergenic
15069	intergenic	intergenic	A	T	intergenic
15070	intergenic	intergenic	T	C	intergenic
15141	NTHI477_RS00075	811	C	T	nonsynonymous
15160	NTHI477_RS00075	792	A	G	synonymous
15194	NTHI477_RS00075	758	T	G	nonsynonymous
15199	NTHI477_RS00075	753	C	T	synonymous
15223	NTHI477_RS00075	729	G	A	synonymous
15247	NTHI477_RS00075	705	A	G	synonymous
15271	NTHI477_RS00075	681	A	G	synonymous
15277	NTHI477_RS00075	675	C	T	synonymous
15289	NTHI477_RS00075	663	G	A	synonymous
15298	NTHI477_RS00075	654	A	G	synonymous
15301	NTHI477_RS00075	651	G	A	synonymous
15343	NTHI477_RS00075	609	A	G	synonymous
15355	NTHI477_RS00075	597	C	G	synonymous
15355	NTHI477_RS00075	597	C	T	synonymous
15364	NTHI477_RS00075	588	G	C	synonymous
15378	NTHI477_RS00075	574	C	A	nonsynonymous
15383	NTHI477_RS00075	569	A	T	nonsynonymous
15397	NTHI477_RS00075	555	G	A	synonymous
15432	NTHI477_RS00075	520	C	A	nonsynonymous
15433	NTHI477_RS00075	519	T	C	synonymous
15433	NTHI477_RS00075	519	T	G	synonymous
15448	NTHI477_RS00075	504	G	A	synonymous
15451	NTHI477_RS00075	501	T	C	synonymous
15454	NTHI477_RS00075	498	T	G	synonymous
15456	NTHI477_RS00075	496	C	T	nonsynonymous

15463	NTHI477_RS00075	489	A	G	synonymous
15478	NTHI477_RS00075	474	G	A	synonymous
15481	NTHI477_RS00075	471	G	A	synonymous
15502	NTHI477_RS00075	450	T	G	synonymous
15505	NTHI477_RS00075	447	C	T	synonymous
15529	NTHI477_RS00075	423	A	G	synonymous
15532	NTHI477_RS00075	420	C	T	synonymous
15538	NTHI477_RS00075	414	C	A	synonymous
15541	NTHI477_RS00075	411	C	T	synonymous
15545	NTHI477_RS00075	407	C	T	nonsynonymous
15546	NTHI477_RS00075	406	C	T	nonsynonymous
15547	NTHI477_RS00075	405	T	C	synonymous
15553	NTHI477_RS00075	399	C	T	synonymous
15562	NTHI477_RS00075	390	T	A	synonymous
15568	NTHI477_RS00075	384	A	G	synonymous
15571	NTHI477_RS00075	381	C	A	synonymous
15574	NTHI477_RS00075	378	G	A	synonymous
15577	NTHI477_RS00075	375	A	T	synonymous
15616	NTHI477_RS00075	336	A	G	synonymous
15623	NTHI477_RS00075	329	A	G	nonsynonymous
15625	NTHI477_RS00075	327	G	A	synonymous
15643	NTHI477_RS00075	309	G	T	synonymous
15646	NTHI477_RS00075	306	A	T	nonsynonymous
15646	NTHI477_RS00075	306	A	G	synonymous
15647	NTHI477_RS00075	305	T	G	nonsynonymous
15649	NTHI477_RS00075	303	C	T	synonymous
15653	NTHI477_RS00075	299	C	A	nonsynonymous
15661	NTHI477_RS00075	291	C	T	synonymous
15669	NTHI477_RS00075	283	T	C	nonsynonymous
15670	NTHI477_RS00075	282	G	A	synonymous
15672	NTHI477_RS00075	280	G	A	nonsynonymous
15677	NTHI477_RS00075	275	C	T	nonsynonymous
15688	NTHI477_RS00075	264	C	T	synonymous
15700	NTHI477_RS00075	252	T	G	synonymous
15705	NTHI477_RS00075	247	A	G	synonymous
15706	NTHI477_RS00075	246	C	T	synonymous
15784	NTHI477_RS00075	168	A	C	synonymous
15787	NTHI477_RS00075	165	T	C	synonymous
15787	NTHI477_RS00075	165	T	G	synonymous
15790	NTHI477_RS00075	162	G	A	synonymous
15820	NTHI477_RS00075	132	T	C	synonymous
15847	NTHI477_RS00075	105	G	A	synonymous
15853	NTHI477_RS00075	99	A	G	synonymous
15855	NTHI477_RS00075	97	C	T	nonsynonymous
15864	NTHI477_RS00075	88	G	A	synonymous

15868	NTHI477_RS00075	84	G	A	synonymous
15880	NTHI477_RS00075	72	G	A	synonymous
15895	NTHI477_RS00075	57	G	A	synonymous
15904	NTHI477_RS00075	48	G	A	synonymous
15907	NTHI477_RS00075	45	T	A	synonymous
15913	NTHI477_RS00075	39	T	G	synonymous
15930	NTHI477_RS00075	22	A	G	synonymous
15931	NTHI477_RS00075	21	A	C	nonsynonymous
15932	NTHI477_RS00075	20	A	G	nonsynonymous
15952	NTHI477_RS00075	0	T	C	synonymous
15953	intergenic	intergenic	G	C	intergenic
15959	intergenic	intergenic	C	T	intergenic
15983	intergenic	intergenic	T	C	intergenic
15985	intergenic	intergenic	C	A	intergenic
15986	intergenic	intergenic	G	A	intergenic
15991	intergenic	intergenic	G	A	intergenic
16006	intergenic	intergenic	A	T	intergenic
16006	intergenic	intergenic	A	C	intergenic
16007	intergenic	intergenic	T	A	intergenic
16052	intergenic	intergenic	C	T	intergenic
16054	intergenic	intergenic	T	C	intergenic
16062	intergenic	intergenic	G	T	intergenic
16064	intergenic	intergenic	T	C	intergenic
16104	intergenic	intergenic	T	C	intergenic
16116	intergenic	intergenic	C	T	intergenic
16184	NTHI477_RS00080	2286	T	A	nonsynonymous
16185	NTHI477_RS00080	2285	T	G	nonsynonymous
16186	NTHI477_RS00080	2284	C	T	nonsynonymous
16193	NTHI477_RS00080	2277	A	T	synonymous
16205	NTHI477_RS00080	2265	G	A	synonymous
16207	NTHI477_RS00080	2263	G	A	nonsynonymous
16211	NTHI477_RS00080	2259	G	C	synonymous
16213	NTHI477_RS00080	2257	G	A	nonsynonymous
16219	NTHI477_RS00080	2251	A	C	nonsynonymous
16221	NTHI477_RS00080	2249	G	A	nonsynonymous
16226	NTHI477_RS00080	2244	G	T	synonymous
16258	NTHI477_RS00080	2212	C	T	nonsynonymous
16269	NTHI477_RS00080	2201	C	T	nonsynonymous
16290	NTHI477_RS00080	2180	T	C	nonsynonymous
16310	NTHI477_RS00080	2160	C	T	synonymous
16327	NTHI477_RS00080	2143	T	C	nonsynonymous
16331	NTHI477_RS00080	2139	G	A	synonymous
16334	NTHI477_RS00080	2136	A	C	synonymous
16342	NTHI477_RS00080	2128	C	T	nonsynonymous
16358	NTHI477_RS00080	2112	T	C	synonymous

16424	NTHI477_RS00080	2046	A	G	synonymous
16445	NTHI477_RS00080	2025	G	A	synonymous
16448	NTHI477_RS00080	2022	G	T	synonymous
16493	NTHI477_RS00080	1977	T	C	synonymous
16493	NTHI477_RS00080	1977	T	A	synonymous
16495	NTHI477_RS00080	1975	A	C	nonsynonymous
16544	NTHI477_RS00080	1926	C	T	synonymous
16556	NTHI477_RS00080	1914	G	A	synonymous
16559	NTHI477_RS00080	1911	A	T	synonymous
16562	NTHI477_RS00080	1908	T	A	synonymous
16565	NTHI477_RS00080	1905	A	G	synonymous
16568	NTHI477_RS00080	1902	A	T	synonymous
16598	NTHI477_RS00080	1872	G	T	synonymous
16624	NTHI477_RS00080	1846	C	T	nonsynonymous
16635	NTHI477_RS00080	1835	G	A	nonsynonymous
16637	NTHI477_RS00080	1833	A	G	synonymous
16676	NTHI477_RS00080	1794	T	A	synonymous
16678	NTHI477_RS00080	1792	C	T	nonsynonymous
16682	NTHI477_RS00080	1788	A	G	synonymous
16683	NTHI477_RS00080	1787	C	T	nonsynonymous
16688	NTHI477_RS00080	1782	G	T	synonymous
16697	NTHI477_RS00080	1773	A	G	synonymous
16702	NTHI477_RS00080	1768	C	A	nonsynonymous
16708	NTHI477_RS00080	1762	T	G	synonymous
16712	NTHI477_RS00080	1758	A	C	synonymous
16726	NTHI477_RS00080	1744	G	T	nonsynonymous
16727	NTHI477_RS00080	1743	G	A	synonymous
16760	NTHI477_RS00080	1710	A	G	synonymous
16764	NTHI477_RS00080	1706	C	G	nonsynonymous
16778	NTHI477_RS00080	1692	C	T	synonymous
16805	NTHI477_RS00080	1665	C	T	synonymous
16808	NTHI477_RS00080	1662	G	A	synonymous
16811	NTHI477_RS00080	1659	G	A	synonymous
16832	NTHI477_RS00080	1638	C	T	synonymous
16841	NTHI477_RS00080	1629	G	A	synonymous
16847	NTHI477_RS00080	1623	G	A	synonymous
16856	NTHI477_RS00080	1614	A	G	synonymous
16865	NTHI477_RS00080	1605	A	T	synonymous
16874	NTHI477_RS00080	1596	T	C	synonymous
16877	NTHI477_RS00080	1593	A	T	synonymous
16880	NTHI477_RS00080	1590	G	A	synonymous
16880	NTHI477_RS00080	1590	G	C	synonymous
16898	NTHI477_RS00080	1572	G	T	synonymous
16898	NTHI477_RS00080	1572	G	C	synonymous
16900	NTHI477_RS00080	1570	C	T	nonsynonymous

16922	NTHI477_RS00080	1548	T	A	synonymous
16922	NTHI477_RS00080	1548	T	C	synonymous
16926	NTHI477_RS00080	1544	T	C	nonsynonymous
16928	NTHI477_RS00080	1542	A	G	synonymous
16961	NTHI477_RS00080	1509	C	A	synonymous
16964	NTHI477_RS00080	1506	C	A	synonymous
16964	NTHI477_RS00080	1506	C	T	synonymous
16967	NTHI477_RS00080	1503	G	A	synonymous
16976	NTHI477_RS00080	1494	G	A	synonymous
16977	NTHI477_RS00080	1493	A	T	nonsynonymous
16985	NTHI477_RS00080	1485	T	C	synonymous
16988	NTHI477_RS00080	1482	T	A	synonymous
16994	NTHI477_RS00080	1476	T	C	synonymous
17007	NTHI477_RS00080	1463	G	A	nonsynonymous
17012	NTHI477_RS00080	1458	A	G	synonymous
17015	NTHI477_RS00080	1455	C	T	synonymous
17033	NTHI477_RS00080	1437	G	A	synonymous
17057	NTHI477_RS00080	1413	A	C	synonymous
17060	NTHI477_RS00080	1410	A	T	synonymous
17123	NTHI477_RS00080	1347	C	G	synonymous
17129	NTHI477_RS00080	1341	T	C	synonymous
17141	NTHI477_RS00080	1329	T	C	synonymous
17144	NTHI477_RS00080	1326	T	G	synonymous
17183	NTHI477_RS00080	1287	C	T	synonymous
17186	NTHI477_RS00080	1284	T	C	synonymous
17186	NTHI477_RS00080	1284	T	A	synonymous
17188	NTHI477_RS00080	1282	C	T	nonsynonymous
17189	NTHI477_RS00080	1281	C	T	synonymous
17208	NTHI477_RS00080	1262	A	G	nonsynonymous
17238	NTHI477_RS00080	1232	A	C	nonsynonymous
17243	NTHI477_RS00080	1227	T	G	synonymous
17258	NTHI477_RS00080	1212	A	G	synonymous
17297	NTHI477_RS00080	1173	G	A	synonymous
17306	NTHI477_RS00080	1164	A	G	synonymous
17309	NTHI477_RS00080	1161	G	C	synonymous
17312	NTHI477_RS00080	1158	T	C	nonsynonymous
17330	NTHI477_RS00080	1140	T	C	synonymous
17332	NTHI477_RS00080	1138	C	T	nonsynonymous
17336	NTHI477_RS00080	1134	G	A	synonymous
17339	NTHI477_RS00080	1131	T	C	synonymous
17345	NTHI477_RS00080	1125	T	C	synonymous
17348	NTHI477_RS00080	1122	T	C	synonymous
17366	NTHI477_RS00080	1104	T	C	synonymous
17372	NTHI477_RS00080	1098	G	A	synonymous
17402	NTHI477_RS00080	1068	C	T	synonymous

17459	NTHI477_RS00080	1011	T	C	synonymous
17472	NTHI477_RS00080	998	C	T	nonsynonymous
17498	NTHI477_RS00080	972	T	C	synonymous
17498	NTHI477_RS00080	972	T	G	synonymous
17507	NTHI477_RS00080	963	T	C	synonymous
17513	NTHI477_RS00080	957	A	T	synonymous
17519	NTHI477_RS00080	951	A	C	synonymous
17519	NTHI477_RS00080	951	A	T	synonymous
17537	NTHI477_RS00080	933	G	C	synonymous
17576	NTHI477_RS00080	894	G	A	synonymous
17591	NTHI477_RS00080	879	C	T	synonymous
17597	NTHI477_RS00080	873	A	C	synonymous
17606	NTHI477_RS00080	864	A	G	synonymous
17615	NTHI477_RS00080	855	G	A	synonymous
17672	NTHI477_RS00080	798	A	G	synonymous
17690	NTHI477_RS00080	780	G	T	synonymous
17693	NTHI477_RS00080	777	A	G	synonymous
17717	NTHI477_RS00080	753	A	C	synonymous
17723	NTHI477_RS00080	747	T	A	synonymous
17741	NTHI477_RS00080	729	G	A	synonymous
17747	NTHI477_RS00080	723	A	G	synonymous
17764	NTHI477_RS00080	706	C	T	nonsynonymous
17768	NTHI477_RS00080	702	C	T	synonymous
17786	NTHI477_RS00080	684	G	C	synonymous
17804	NTHI477_RS00080	666	C	T	synonymous
17810	NTHI477_RS00080	660	T	G	synonymous
17813	NTHI477_RS00080	657	G	T	synonymous
17822	NTHI477_RS00080	648	C	A	synonymous
17831	NTHI477_RS00080	639	T	A	synonymous
17843	NTHI477_RS00080	627	C	G	synonymous
17855	NTHI477_RS00080	615	G	A	synonymous
17870	NTHI477_RS00080	600	T	G	synonymous
17951	NTHI477_RS00080	519	T	C	synonymous
17960	NTHI477_RS00080	510	A	T	synonymous
17963	NTHI477_RS00080	507	T	A	nonsynonymous
17965	NTHI477_RS00080	505	A	G	synonymous
17966	NTHI477_RS00080	504	A	G	synonymous
17999	NTHI477_RS00080	471	C	T	synonymous
18002	NTHI477_RS00080	468	C	T	synonymous
18026	NTHI477_RS00080	444	A	G	synonymous
18028	NTHI477_RS00080	442	C	T	nonsynonymous
18029	NTHI477_RS00080	441	A	T	synonymous
18031	NTHI477_RS00080	439	T	C	nonsynonymous
18035	NTHI477_RS00080	435	G	T	synonymous
18038	NTHI477_RS00080	432	T	C	synonymous

18038	NTHI477_RS00080	432	T	G	synonymous
18040	NTHI477_RS00080	430	T	C	nonsynonymous
18041	NTHI477_RS00080	429	G	C	synonymous
18041	NTHI477_RS00080	429	G	A	synonymous
18044	NTHI477_RS00080	426	T	G	nonsynonymous
18046	NTHI477_RS00080	424	A	G	synonymous
18056	NTHI477_RS00080	414	G	T	synonymous
18061	NTHI477_RS00080	409	A	T	nonsynonymous
18065	NTHI477_RS00080	405	C	T	synonymous
18068	NTHI477_RS00080	402	A	G	synonymous
18073	NTHI477_RS00080	397	G	T	nonsynonymous
18075	NTHI477_RS00080	395	C	T	nonsynonymous
18086	NTHI477_RS00080	384	G	A	synonymous
18089	NTHI477_RS00080	381	C	T	synonymous
18098	NTHI477_RS00080	372	C	T	synonymous
18101	NTHI477_RS00080	369	A	T	synonymous
18104	NTHI477_RS00080	366	C	T	synonymous
18131	NTHI477_RS00080	339	A	G	synonymous
18134	NTHI477_RS00080	336	G	A	synonymous
18139	NTHI477_RS00080	331	G	A	nonsynonymous
18140	NTHI477_RS00080	330	T	C	nonsynonymous
18142	NTHI477_RS00080	328	T	C	nonsynonymous
18164	NTHI477_RS00080	306	T	C	synonymous
18179	NTHI477_RS00080	291	T	G	synonymous
18182	NTHI477_RS00080	288	G	A	synonymous
18191	NTHI477_RS00080	279	T	G	synonymous
18194	NTHI477_RS00080	276	A	C	synonymous
18194	NTHI477_RS00080	276	A	G	synonymous
18275	NTHI477_RS00080	195	G	T	synonymous
18311	NTHI477_RS00080	159	A	T	synonymous
18314	NTHI477_RS00080	156	G	A	synonymous
18341	NTHI477_RS00080	129	G	A	synonymous
18374	NTHI477_RS00080	96	T	C	synonymous
18375	NTHI477_RS00080	95	G	A	nonsynonymous
18376	NTHI477_RS00080	94	C	T	nonsynonymous
18377	NTHI477_RS00080	93	A	T	synonymous
18383	NTHI477_RS00080	87	G	A	synonymous
18392	NTHI477_RS00080	78	A	C	synonymous
18551	NTHI477_RS00085	279	A	G	synonymous
18560	NTHI477_RS00085	270	A	G	synonymous
18611	NTHI477_RS00085	219	G	T	synonymous
18611	NTHI477_RS00085	219	G	A	synonymous
18613	NTHI477_RS00085	217	G	A	nonsynonymous
18631	NTHI477_RS00085	199	C	A	nonsynonymous
18643	NTHI477_RS00085	187	A	G	synonymous

18644	NTHI477_RS00085	186	C	T	synonymous
18655	NTHI477_RS00085	175	C	T	nonsynonymous
18656	NTHI477_RS00085	174	A	G	synonymous
18659	NTHI477_RS00085	171	G	A	synonymous
18661	NTHI477_RS00085	169	C	T	nonsynonymous
18667	NTHI477_RS00085	163	C	T	nonsynonymous
18700	NTHI477_RS00085	130	T	A	nonsynonymous
18701	NTHI477_RS00085	129	A	G	synonymous
18746	NTHI477_RS00085	84	G	A	synonymous
18763	NTHI477_RS00085	67	G	A	nonsynonymous
18779	NTHI477_RS00085	51	C	T	synonymous
18786	NTHI477_RS00085	44	T	G	nonsynonymous
18788	NTHI477_RS00085	42	G	T	synonymous
18791	NTHI477_RS00085	39	G	A	synonymous
18821	NTHI477_RS00085	9	A	G	synonymous
18870	NTHI477_RS00090	306	A	G	synonymous
18894	NTHI477_RS00090	282	A	G	synonymous
18912	NTHI477_RS00090	264	C	T	synonymous
18915	NTHI477_RS00090	261	A	T	synonymous
18918	NTHI477_RS00090	258	A	G	synonymous
18927	NTHI477_RS00090	249	T	G	synonymous
18945	NTHI477_RS00090	231	T	C	synonymous
18951	NTHI477_RS00090	225	G	A	synonymous
18978	NTHI477_RS00090	198	C	A	synonymous
19053	NTHI477_RS00090	123	A	C	synonymous
19056	NTHI477_RS00090	120	A	C	synonymous
19088	NTHI477_RS00090	88	T	C	nonsynonymous
19128	NTHI477_RS00090	48	T	C	synonymous
19131	NTHI477_RS00090	45	C	T	synonymous
19158	NTHI477_RS00090	18	C	T	synonymous
19161	NTHI477_RS00090	15	C	A	nonsynonymous
19207	intergenic	intergenic	C	T	intergenic
19208	intergenic	intergenic	C	T	intergenic
19244	intergenic	intergenic	C	T	intergenic
19332	NTHI477_RS00095	22	G	A	nonsynonymous
19349	NTHI477_RS00095	39	G	A	synonymous
19350	NTHI477_RS00095	40	T	C	synonymous
19352	NTHI477_RS00095	42	A	G	synonymous
19355	NTHI477_RS00095	45	T	C	synonymous
19360	NTHI477_RS00095	50	C	T	nonsynonymous
19361	NTHI477_RS00095	51	A	C	synonymous
19364	NTHI477_RS00095	54	C	T	synonymous
19376	NTHI477_RS00095	66	C	G	synonymous
19385	NTHI477_RS00095	75	G	T	synonymous
19388	NTHI477_RS00095	78	A	G	synonymous

19415	NTHI477_RS00095	105	C	T	synonymous
19421	NTHI477_RS00095	111	C	T	synonymous
19427	NTHI477_RS00095	117	C	T	synonymous
19430	NTHI477_RS00095	120	A	G	synonymous
19478	NTHI477_RS00095	168	C	T	synonymous
19481	NTHI477_RS00095	171	T	C	synonymous
19484	NTHI477_RS00095	174	G	T	synonymous
19484	NTHI477_RS00095	174	G	C	synonymous
19499	NTHI477_RS00095	189	C	T	synonymous
19505	NTHI477_RS00095	195	C	T	synonymous
19514	NTHI477_RS00095	204	G	A	synonymous
19526	NTHI477_RS00095	216	C	T	synonymous
19538	NTHI477_RS00095	228	C	T	synonymous
19541	NTHI477_RS00095	231	T	C	synonymous
19544	NTHI477_RS00095	234	T	C	synonymous
19562	NTHI477_RS00095	252	C	T	synonymous
19572	NTHI477_RS00095	262	T	C	synonymous
19574	NTHI477_RS00095	264	A	T	nonsynonymous
19590	NTHI477_RS00095	280	G	A	nonsynonymous
19614	NTHI477_RS00095	304	A	C	nonsynonymous
19619	NTHI477_RS00095	309	C	T	synonymous
19652	NTHI477_RS00095	342	C	T	synonymous
19658	NTHI477_RS00095	348	T	C	synonymous
19661	NTHI477_RS00095	351	C	A	synonymous
19667	NTHI477_RS00095	357	G	A	synonymous
19685	NTHI477_RS00095	375	A	G	synonymous
19706	NTHI477_RS00095	396	A	G	synonymous
19740	NTHI477_RS00095	430	G	A	nonsynonymous
19769	NTHI477_RS00095	459	C	T	synonymous
19784	NTHI477_RS00095	474	G	T	synonymous
19793	NTHI477_RS00095	483	G	A	synonymous
19802	NTHI477_RS00095	492	C	T	synonymous
19829	NTHI477_RS00095	519	C	T	synonymous
19838	NTHI477_RS00095	528	G	A	synonymous
19874	NTHI477_RS00095	564	C	T	synonymous
19877	NTHI477_RS00095	567	C	A	synonymous
19886	NTHI477_RS00095	576	C	A	synonymous
19886	NTHI477_RS00095	576	C	T	synonymous
19889	NTHI477_RS00095	579	C	T	synonymous
19892	NTHI477_RS00095	582	G	A	synonymous
19904	NTHI477_RS00095	594	C	T	synonymous
19919	NTHI477_RS00095	609	T	C	synonymous
19925	NTHI477_RS00095	615	G	A	synonymous
19940	NTHI477_RS00095	630	A	G	synonymous
19961	NTHI477_RS00095	651	A	G	synonymous

19997	NTHI477_RS00095	687	C	T	synonymous
20015	NTHI477_RS00095	705	C	T	synonymous
20036	NTHI477_RS00095	726	G	C	synonymous
20054	NTHI477_RS00095	744	A	G	synonymous
20063	NTHI477_RS00095	753	C	T	synonymous
20087	NTHI477_RS00095	777	T	G	synonymous
20096	NTHI477_RS00095	786	G	A	synonymous
20106	NTHI477_RS00095	796	G	A	nonsynonymous
20108	NTHI477_RS00095	798	G	A	synonymous
20108	NTHI477_RS00095	798	G	T	synonymous
20121	intergenic	intergenic	A	G	intergenic
20125	intergenic	intergenic	A	T	intergenic
20134	intergenic	intergenic	A	G	intergenic
20137	intergenic	intergenic	T	C	intergenic
20181	intergenic	intergenic	A	G	intergenic
20183	intergenic	intergenic	G	C	intergenic
20189	intergenic	intergenic	G	T	intergenic
20191	intergenic	intergenic	T	A	intergenic
20193	intergenic	intergenic	T	C	intergenic
20195	intergenic	intergenic	A	C	intergenic
20196	intergenic	intergenic	C	T	intergenic
20200	intergenic	intergenic	A	G	intergenic
20207	intergenic	intergenic	T	C	intergenic
20208	intergenic	intergenic	C	A	intergenic
20210	intergenic	intergenic	T	C	intergenic
20214	NTHI477_RS00100	4	C	T	synonymous
20220	NTHI477_RS00100	10	T	C	nonsynonymous
20223	NTHI477_RS00100	13	C	T	nonsynonymous
20224	NTHI477_RS00100	14	C	T	nonsynonymous
20226	NTHI477_RS00100	16	A	T	nonsynonymous
20228	NTHI477_RS00100	18	A	G	synonymous
20229	NTHI477_RS00100	19	C	T	synonymous
20267	NTHI477_RS00100	57	A	G	synonymous
20277	NTHI477_RS00100	67	C	T	nonsynonymous
20294	NTHI477_RS00100	84	C	A	synonymous
20307	NTHI477_RS00100	97	C	T	nonsynonymous
20327	NTHI477_RS00100	117	T	G	synonymous
20328	NTHI477_RS00100	118	A	G	nonsynonymous
20340	NTHI477_RS00100	130	T	A	nonsynonymous
20354	NTHI477_RS00100	144	T	C	synonymous
20357	NTHI477_RS00100	147	C	A	synonymous
20357	NTHI477_RS00100	147	C	T	synonymous
20384	NTHI477_RS00100	174	A	G	nonsynonymous
20386	NTHI477_RS00100	176	A	G	nonsynonymous
20399	NTHI477_RS00100	189	A	G	synonymous

20401	NTHI477_RS00100	191	T	A	nonsynonymous
20402	NTHI477_RS00100	192	G	A	synonymous
20403	NTHI477_RS00100	193	G	A	nonsynonymous
20405	NTHI477_RS00100	195	G	C	synonymous
20405	NTHI477_RS00100	195	G	A	synonymous
20409	NTHI477_RS00100	199	G	T	nonsynonymous
20441	NTHI477_RS00100	231	T	A	synonymous
20459	NTHI477_RS00100	249	G	A	synonymous
20474	NTHI477_RS00100	264	G	A	synonymous
20480	NTHI477_RS00100	270	A	G	synonymous
20497	NTHI477_RS00100	287	G	T	nonsynonymous
20501	NTHI477_RS00100	291	T	A	synonymous
20502	NTHI477_RS00100	292	G	A	nonsynonymous
20504	NTHI477_RS00100	294	G	A	synonymous
20516	NTHI477_RS00100	306	G	A	synonymous
20522	NTHI477_RS00100	312	T	C	synonymous
20534	NTHI477_RS00100	324	G	A	synonymous
20549	NTHI477_RS00100	339	C	T	synonymous
20552	NTHI477_RS00100	342	T	C	synonymous
20565	NTHI477_RS00100	355	A	C	nonsynonymous
20570	NTHI477_RS00100	360	T	C	synonymous
20583	NTHI477_RS00100	373	G	C	nonsynonymous
20584	NTHI477_RS00100	374	A	C	nonsynonymous
20596	NTHI477_RS00100	386	C	T	nonsynonymous
20603	NTHI477_RS00100	393	G	A	synonymous
20627	NTHI477_RS00100	417	C	T	synonymous
20642	NTHI477_RS00100	432	A	C	synonymous
20651	NTHI477_RS00100	441	T	C	synonymous
20652	NTHI477_RS00100	442	G	A	nonsynonymous
20653	NTHI477_RS00100	443	C	T	nonsynonymous
20678	NTHI477_RS00100	468	A	G	synonymous
20679	NTHI477_RS00100	469	T	C	synonymous
20702	NTHI477_RS00100	492	A	T	synonymous
20720	NTHI477_RS00100	510	A	G	synonymous
20722	NTHI477_RS00100	512	A	G	nonsynonymous
20809	NTHI477_RS00100	599	A	T	nonsynonymous
20816	NTHI477_RS00100	606	T	C	synonymous
20819	NTHI477_RS00100	609	A	T	synonymous
20822	NTHI477_RS00100	612	C	T	synonymous
20860	NTHI477_RS00100	650	G	A	nonsynonymous
20861	NTHI477_RS00100	651	C	T	synonymous
20867	NTHI477_RS00100	657	T	G	synonymous
20870	NTHI477_RS00100	660	G	C	synonymous
20873	NTHI477_RS00100	663	T	G	synonymous
20874	NTHI477_RS00100	664	T	C	synonymous

20875	NTHI477_RS00100	665	T	A	nonsynonymous
20879	NTHI477_RS00100	669	C	T	synonymous
20887	NTHI477_RS00100	677	C	A	nonsynonymous
20888	NTHI477_RS00100	678	T	C	synonymous
20913	NTHI477_RS00100	703	C	T	nonsynonymous
20945	NTHI477_RS00100	735	T	C	synonymous
20969	NTHI477_RS00100	759	C	T	synonymous
20989	NTHI477_RS00100	779	A	C	nonsynonymous
21020	NTHI477_RS00100	810	T	C	synonymous
21032	NTHI477_RS00100	822	C	T	synonymous
21041	NTHI477_RS00100	831	A	G	synonymous
21044	NTHI477_RS00100	834	G	A	synonymous
21047	NTHI477_RS00100	837	T	C	synonymous
21050	NTHI477_RS00100	840	C	T	synonymous
21061	NTHI477_RS00100	851	G	A	nonsynonymous
21067	NTHI477_RS00100	857	C	A	nonsynonymous
21074	NTHI477_RS00100	864	G	A	synonymous
21113	NTHI477_RS00100	903	A	C	synonymous
21122	NTHI477_RS00100	912	G	T	synonymous
21134	NTHI477_RS00100	924	A	G	synonymous
21135	NTHI477_RS00100	925	C	T	synonymous
21165	NTHI477_RS00100	955	C	T	nonsynonymous
21174	NTHI477_RS00100	964	C	A	nonsynonymous
21177	NTHI477_RS00100	967	G	A	nonsynonymous
21178	NTHI477_RS00100	968	A	C	nonsynonymous
21185	NTHI477_RS00100	975	A	G	synonymous
21191	NTHI477_RS00100	981	C	T	synonymous
21191	NTHI477_RS00100	981	C	G	nonsynonymous
21194	NTHI477_RS00100	984	A	G	synonymous
21209	NTHI477_RS00100	999	T	C	synonymous
21221	NTHI477_RS00100	1011	C	T	synonymous
21226	NTHI477_RS00100	1016	G	C	nonsynonymous
21227	NTHI477_RS00100	1017	C	T	synonymous
21245	NTHI477_RS00100	1035	C	T	synonymous
21246	NTHI477_RS00100	1036	T	C	nonsynonymous
21247	NTHI477_RS00100	1037	T	G	nonsynonymous
21269	NTHI477_RS00100	1059	G	A	synonymous
21305	NTHI477_RS00100	1095	C	T	synonymous
21306	NTHI477_RS00100	1096	G	A	nonsynonymous
21308	NTHI477_RS00100	1098	A	G	synonymous
21311	NTHI477_RS00100	1101	T	G	nonsynonymous
21314	NTHI477_RS00100	1104	C	G	synonymous
21314	NTHI477_RS00100	1104	C	T	synonymous
21315	NTHI477_RS00100	1105	A	G	nonsynonymous
21332	NTHI477_RS00100	1122	T	C	synonymous

21335	NTHI477_RS00100	1125	G	A	synonymous
21359	NTHI477_RS00100	1149	T	G	nonsynonymous
21366	NTHI477_RS00100	1156	T	C	nonsynonymous
21380	NTHI477_RS00100	1170	C	T	synonymous
21391	NTHI477_RS00100	1181	C	A	nonsynonymous
21392	NTHI477_RS00100	1182	C	A	synonymous
21392	NTHI477_RS00100	1182	C	T	synonymous
21392	NTHI477_RS00100	1182	C	G	synonymous
21398	NTHI477_RS00100	1188	G	A	synonymous
21410	NTHI477_RS00100	1200	A	G	synonymous
21416	NTHI477_RS00100	1206	A	G	synonymous
21432	NTHI477_RS00100	1222	G	T	nonsynonymous
21434	NTHI477_RS00100	1224	A	G	synonymous
21440	NTHI477_RS00100	1230	A	G	synonymous
21470	NTHI477_RS00100	1260	T	C	synonymous
21479	NTHI477_RS00100	1269	T	A	synonymous
21479	NTHI477_RS00100	1269	T	G	synonymous
21482	NTHI477_RS00100	1272	G	T	synonymous
21503	NTHI477_RS00100	1293	C	T	synonymous
21509	NTHI477_RS00100	1299	G	A	synonymous
21512	NTHI477_RS00100	1302	C	A	synonymous
21512	NTHI477_RS00100	1302	C	T	synonymous
21530	NTHI477_RS00100	1320	G	A	synonymous
21536	NTHI477_RS00100	1326	C	T	synonymous
21597	NTHI477_RS00100	1387	C	A	nonsynonymous
21601	NTHI477_RS00100	1391	A	G	nonsynonymous
21607	NTHI477_RS00100	1397	C	A	nonsynonymous
21608	NTHI477_RS00100	1398	G	T	synonymous
21609	NTHI477_RS00100	1399	C	G	nonsynonymous
21617	NTHI477_RS00100	1407	T	C	synonymous
21626	NTHI477_RS00100	1416	T	C	synonymous
21645	NTHI477_RS00100	1435	C	T	nonsynonymous
21662	NTHI477_RS00100	1452	G	A	synonymous
21672	NTHI477_RS00100	1462	G	A	nonsynonymous
21674	NTHI477_RS00100	1464	G	T	synonymous
21680	NTHI477_RS00100	1470	G	A	synonymous
21683	NTHI477_RS00100	1473	C	G	nonsynonymous
21686	NTHI477_RS00100	1476	C	T	synonymous
21701	NTHI477_RS00100	1491	G	A	synonymous
21704	NTHI477_RS00100	1494	A	G	synonymous
21707	NTHI477_RS00100	1497	T	C	synonymous
21710	NTHI477_RS00100	1500	T	C	synonymous
21728	NTHI477_RS00100	1518	T	C	synonymous
21730	NTHI477_RS00100	1520	C	T	nonsynonymous
21731	NTHI477_RS00100	1521	T	A	synonymous

21732	NTHI477_RS00100	1522	G	A	nonsynonymous
21733	NTHI477_RS00100	1523	T	A	nonsynonymous
21734	NTHI477_RS00100	1524	G	A	synonymous
21738	NTHI477_RS00100	1528	A	G	nonsynonymous
21740	NTHI477_RS00100	1530	T	G	nonsynonymous
21740	NTHI477_RS00100	1530	T	A	synonymous
21744	NTHI477_RS00100	1534	G	A	nonsynonymous
21746	NTHI477_RS00100	1536	C	T	synonymous
21752	NTHI477_RS00100	1542	A	G	synonymous
21754	NTHI477_RS00100	1544	A	G	nonsynonymous
21773	NTHI477_RS00100	1563	T	C	synonymous
21800	NTHI477_RS00100	1590	C	A	synonymous
21800	NTHI477_RS00100	1590	C	T	synonymous
21803	NTHI477_RS00100	1593	A	T	synonymous
21803	NTHI477_RS00100	1593	A	C	synonymous
21804	NTHI477_RS00100	1594	A	C	nonsynonymous
21809	NTHI477_RS00100	1599	C	T	synonymous
21830	NTHI477_RS00100	1620	G	T	synonymous
21833	NTHI477_RS00100	1623	G	A	synonymous
21863	NTHI477_RS00100	1653	C	G	synonymous
21866	NTHI477_RS00100	1656	C	T	synonymous
21876	NTHI477_RS00100	1666	G	A	nonsynonymous
21914	NTHI477_RS00100	1704	G	C	synonymous
21917	NTHI477_RS00100	1707	C	T	synonymous
21932	NTHI477_RS00100	1722	G	A	synonymous
21941	NTHI477_RS00100	1731	T	C	synonymous
21942	NTHI477_RS00100	1732	G	T	nonsynonymous
21947	NTHI477_RS00100	1737	C	T	synonymous
21950	NTHI477_RS00100	1740	T	C	synonymous
21951	NTHI477_RS00100	1741	G	A	nonsynonymous
21953	NTHI477_RS00100	1743	T	C	synonymous
22007	NTHI477_RS00100	1797	A	T	synonymous
22007	NTHI477_RS00100	1797	A	C	synonymous
22009	NTHI477_RS00100	1799	G	A	nonsynonymous
22012	NTHI477_RS00100	1802	G	A	nonsynonymous
22013	NTHI477_RS00100	1803	G	A	synonymous
22020	NTHI477_RS00100	1810	T	G	nonsynonymous
22028	NTHI477_RS00100	1818	C	T	synonymous
22049	NTHI477_RS00100	1839	T	C	synonymous
22085	NTHI477_RS00100	1875	A	G	synonymous
22096	NTHI477_RS00100	1886	T	C	nonsynonymous
22099	NTHI477_RS00100	1889	G	A	nonsynonymous
22101	NTHI477_RS00100	1891	G	A	nonsynonymous
22109	NTHI477_RS00100	1899	T	C	synonymous
22110	NTHI477_RS00100	1900	G	T	nonsynonymous

22116	NTHI477_RS00100	1906	A	G	nonsynonymous
22117	NTHI477_RS00100	1907	C	T	nonsynonymous
22118	NTHI477_RS00100	1908	G	A	synonymous
22119	NTHI477_RS00100	1909	C	T	nonsynonymous
22124	NTHI477_RS00100	1914	T	C	synonymous
22127	NTHI477_RS00100	1917	G	T	synonymous
22133	NTHI477_RS00100	1923	G	C	nonsynonymous
22134	NTHI477_RS00100	1924	C	A	nonsynonymous
22143	NTHI477_RS00100	1933	G	A	nonsynonymous
22144	NTHI477_RS00100	1934	C	A	nonsynonymous
22145	NTHI477_RS00100	1935	T	G	synonymous
22151	NTHI477_RS00100	1941	T	C	synonymous
22154	NTHI477_RS00100	1944	A	T	synonymous
22169	NTHI477_RS00100	1959	G	T	synonymous
22184	NTHI477_RS00100	1974	A	G	synonymous
22190	NTHI477_RS00100	1980	T	C	synonymous
22193	NTHI477_RS00100	1983	G	A	synonymous
22217	NTHI477_RS00100	2007	T	C	synonymous
22220	NTHI477_RS00100	2010	C	A	nonsynonymous
22222	NTHI477_RS00100	2012	T	C	nonsynonymous
22223	NTHI477_RS00100	2013	T	A	synonymous
22259	NTHI477_RS00100	2049	A	G	synonymous
22265	NTHI477_RS00100	2055	C	T	synonymous
22271	NTHI477_RS00100	2061	A	G	synonymous
22295	NTHI477_RS00100	2085	C	T	synonymous
22319	NTHI477_RS00100	2109	G	C	synonymous
22334	NTHI477_RS00100	2124	C	G	synonymous
22334	NTHI477_RS00100	2124	C	T	synonymous
22334	NTHI477_RS00100	2124	C	A	synonymous
22335	NTHI477_RS00100	2125	G	A	nonsynonymous
22355	NTHI477_RS00100	2145	C	T	synonymous
22361	NTHI477_RS00100	2151	A	G	synonymous
22373	NTHI477_RS00100	2163	G	A	synonymous
22401	NTHI477_RS00100	2191	T	C	nonsynonymous
22421	NTHI477_RS00100	2211	C	T	synonymous
22424	NTHI477_RS00100	2214	A	T	synonymous
22430	NTHI477_RS00100	2220	G	A	synonymous
22433	NTHI477_RS00100	2223	T	G	synonymous
22455	NTHI477_RS00100	2245	T	C	synonymous
22466	NTHI477_RS00100	2256	C	T	synonymous
22469	NTHI477_RS00100	2259	A	C	synonymous
22473	NTHI477_RS00100	2263	T	G	nonsynonymous
22478	NTHI477_RS00100	2268	C	T	synonymous
22488	NTHI477_RS00100	2278	G	A	nonsynonymous
22496	NTHI477_RS00100	2286	G	A	synonymous

22497	NTHI477_RS00100	2287	C	T	nonsynonymous
22512	NTHI477_RS00100	2302	G	A	nonsynonymous
22513	NTHI477_RS00100	2303	T	C	nonsynonymous
22517	NTHI477_RS00100	2307	T	G	synonymous
22523	NTHI477_RS00100	2313	C	T	synonymous
22538	NTHI477_RS00100	2328	T	C	synonymous
22540	NTHI477_RS00100	2330	C	T	nonsynonymous
22544	NTHI477_RS00100	2334	A	G	synonymous
22550	NTHI477_RS00100	2340	C	T	synonymous
22553	NTHI477_RS00100	2343	C	T	synonymous
22568	NTHI477_RS00100	2358	G	T	nonsynonymous
22568	NTHI477_RS00100	2358	G	C	nonsynonymous
22574	NTHI477_RS00100	2364	C	T	synonymous
22583	NTHI477_RS00100	2373	A	G	synonymous
22590	NTHI477_RS00100	2380	C	G	nonsynonymous
22617	NTHI477_RS00100	2407	G	C	nonsynonymous
22619	NTHI477_RS00100	2409	A	C	synonymous
22622	NTHI477_RS00100	2412	G	T	synonymous
22631	NTHI477_RS00100	2421	C	G	synonymous
22649	NTHI477_RS00100	2439	T	C	synonymous
22651	NTHI477_RS00100	2441	C	G	nonsynonymous
22664	NTHI477_RS00100	2454	A	C	synonymous
22673	NTHI477_RS00100	2463	A	G	synonymous
22679	NTHI477_RS00100	2469	G	A	synonymous
22691	NTHI477_RS00100	2481	C	A	synonymous
22694	NTHI477_RS00100	2484	A	G	synonymous
22700	NTHI477_RS00100	2490	A	G	synonymous
22707	NTHI477_RS00100	2497	G	A	nonsynonymous
22854	intergenic	intergenic	A	G	intergenic
22869	intergenic	intergenic	G	A	intergenic
22875	intergenic	intergenic	C	T	intergenic
22880	NTHI477_RS00105	4	C	T	synonymous
22906	NTHI477_RS00105	30	C	T	synonymous
22907	NTHI477_RS00105	31	G	T	nonsynonymous
22909	NTHI477_RS00105	33	G	A	synonymous
22936	NTHI477_RS00105	60	A	T	synonymous
22936	NTHI477_RS00105	60	A	C	synonymous
22937	NTHI477_RS00105	61	C	G	nonsynonymous
22960	NTHI477_RS00105	84	C	T	synonymous
22966	NTHI477_RS00105	90	A	G	synonymous
22987	NTHI477_RS00105	111	T	C	synonymous
23002	NTHI477_RS00105	126	A	G	synonymous
23029	NTHI477_RS00105	153	T	C	synonymous
23032	NTHI477_RS00105	156	T	A	synonymous
23047	NTHI477_RS00105	171	T	C	synonymous

23048	NTHI477_RS00105	172	C	T	synonymous
23065	NTHI477_RS00105	189	A	G	synonymous
23083	NTHI477_RS00105	207	T	C	synonymous
23092	NTHI477_RS00105	216	C	T	synonymous
23173	NTHI477_RS00105	297	A	G	synonymous
23200	NTHI477_RS00105	324	T	A	synonymous
23203	NTHI477_RS00105	327	G	C	synonymous
23215	NTHI477_RS00105	339	T	C	synonymous
23257	NTHI477_RS00105	381	T	C	synonymous
23257	NTHI477_RS00105	381	T	A	synonymous
23260	NTHI477_RS00105	384	T	G	synonymous
23263	NTHI477_RS00105	387	A	T	synonymous
23269	NTHI477_RS00105	393	T	G	synonymous
23270	NTHI477_RS00105	394	T	C	synonymous
23278	NTHI477_RS00105	402	T	C	synonymous
23281	NTHI477_RS00105	405	C	T	synonymous
23284	NTHI477_RS00105	408	C	T	synonymous
23284	NTHI477_RS00105	408	C	A	synonymous
23290	NTHI477_RS00105	414	C	T	synonymous
23344	NTHI477_RS00105	468	T	C	synonymous
23347	NTHI477_RS00105	471	G	T	synonymous
23350	NTHI477_RS00105	474	A	G	synonymous
23353	NTHI477_RS00105	477	C	T	synonymous
23359	NTHI477_RS00105	483	T	C	synonymous
23371	NTHI477_RS00105	495	G	C	synonymous
23371	NTHI477_RS00105	495	G	T	synonymous
23374	NTHI477_RS00105	498	G	A	synonymous
23416	NTHI477_RS00105	540	G	A	synonymous
23425	NTHI477_RS00105	549	T	C	synonymous
23438	NTHI477_RS00105	562	G	C	nonsynonymous
23440	NTHI477_RS00105	564	A	G	synonymous
23464	NTHI477_RS00105	588	C	A	synonymous
23488	NTHI477_RS00105	612	A	T	synonymous
23488	NTHI477_RS00105	612	A	G	synonymous
23509	NTHI477_RS00105	633	G	C	synonymous
23515	NTHI477_RS00105	639	C	T	synonymous
23515	NTHI477_RS00105	639	C	G	synonymous
23533	NTHI477_RS00105	657	C	T	synonymous
23539	NTHI477_RS00105	663	C	T	synonymous
23560	NTHI477_RS00105	684	A	G	synonymous
23566	NTHI477_RS00105	690	G	A	synonymous
23584	NTHI477_RS00105	708	T	G	synonymous
23599	NTHI477_RS00105	723	C	G	synonymous
23611	NTHI477_RS00105	735	T	C	synonymous
23618	NTHI477_RS00105	742	A	G	nonsynonymous

23677	NTHI477_RS00105	801	A	G	synonymous
23683	NTHI477_RS00105	807	T	C	synonymous
23698	NTHI477_RS00105	822	T	C	synonymous
23716	NTHI477_RS00105	840	A	G	synonymous
23722	NTHI477_RS00105	846	G	A	synonymous
23759	NTHI477_RS00105	883	T	C	nonsynonymous
23762	NTHI477_RS00105	886	G	A	nonsynonymous
23767	NTHI477_RS00105	891	T	G	synonymous
23785	NTHI477_RS00105	909	C	T	synonymous
23801	NTHI477_RS00105	925	G	A	nonsynonymous
23818	NTHI477_RS00105	942	A	G	synonymous
23833	NTHI477_RS00105	957	T	C	synonymous
23836	NTHI477_RS00105	960	C	T	synonymous
23877	NTHI477_RS00105	1001	A	G	nonsynonymous
23881	NTHI477_RS00105	1005	A	G	synonymous
23902	NTHI477_RS00105	1026	A	C	synonymous
23917	NTHI477_RS00105	1041	T	C	synonymous
23923	NTHI477_RS00105	1047	C	A	synonymous
23929	NTHI477_RS00105	1053	G	A	synonymous
23935	NTHI477_RS00105	1059	G	A	synonymous
23953	intergenic	intergenic	G	C	intergenic
23964	intergenic	intergenic	T	C	intergenic
23966	intergenic	intergenic	A	C	intergenic
24010	intergenic	intergenic	A	G	intergenic
24011	intergenic	intergenic	C	A	intergenic
24011	intergenic	intergenic	C	G	intergenic
24012	intergenic	intergenic	A	G	intergenic
24016	intergenic	intergenic	T	G	intergenic
24018	intergenic	intergenic	A	G	intergenic
24019	intergenic	intergenic	T	C	intergenic
24023	intergenic	intergenic	C	A	intergenic
24026	intergenic	intergenic	T	G	intergenic
24031	intergenic	intergenic	G	A	intergenic
24058	intergenic	intergenic	G	A	intergenic
24063	intergenic	intergenic	A	G	intergenic
24269	intergenic	intergenic	A	G	intergenic
24270	intergenic	intergenic	G	A	intergenic
24271	intergenic	intergenic	C	G	intergenic
24272	intergenic	intergenic	C	T	intergenic
24273	intergenic	intergenic	C	G	intergenic
24274	intergenic	intergenic	G	A	intergenic
24275	intergenic	intergenic	A	C	intergenic
24275	intergenic	intergenic	A	T	intergenic
24307	intergenic	intergenic	C	A	intergenic
24321	intergenic	intergenic	G	T	intergenic

24322	intergenic	intergenic	A	C	intergenic
24354	NTHI477_RS00120	539	T	C	nonsynonymous
24356	NTHI477_RS00120	537	C	T	nonsynonymous
24362	NTHI477_RS00120	531	G	A	synonymous
24367	NTHI477_RS00120	526	C	T	nonsynonymous
24383	NTHI477_RS00120	510	C	T	synonymous
24404	NTHI477_RS00120	489	G	A	synonymous
24410	NTHI477_RS00120	483	T	A	synonymous
24413	NTHI477_RS00120	480	T	C	synonymous
24413	NTHI477_RS00120	480	T	A	synonymous
24416	NTHI477_RS00120	477	T	C	synonymous
24422	NTHI477_RS00120	471	C	G	synonymous
24428	NTHI477_RS00120	465	C	T	synonymous
24440	NTHI477_RS00120	453	G	A	synonymous
24442	NTHI477_RS00120	451	C	T	nonsynonymous
24467	NTHI477_RS00120	426	T	C	synonymous
24470	NTHI477_RS00120	423	A	T	synonymous
24491	NTHI477_RS00120	402	C	T	synonymous
24491	NTHI477_RS00120	402	C	A	synonymous
24494	NTHI477_RS00120	399	G	A	synonymous
24502	NTHI477_RS00120	391	G	A	nonsynonymous
24530	NTHI477_RS00120	363	T	A	synonymous
24551	NTHI477_RS00120	342	G	T	synonymous
24578	NTHI477_RS00120	315	G	A	synonymous
24590	NTHI477_RS00120	303	T	C	synonymous
24599	NTHI477_RS00120	294	G	C	synonymous
24599	NTHI477_RS00120	294	G	T	synonymous
24602	NTHI477_RS00120	291	A	C	synonymous
24608	NTHI477_RS00120	285	C	T	synonymous
24644	NTHI477_RS00120	249	T	A	synonymous
24659	NTHI477_RS00120	234	G	A	synonymous
24670	NTHI477_RS00120	223	C	T	nonsynonymous
24695	NTHI477_RS00120	198	A	G	synonymous
24698	NTHI477_RS00120	195	C	A	synonymous
24735	NTHI477_RS00120	158	T	C	nonsynonymous
24742	NTHI477_RS00120	151	A	G	nonsynonymous
24764	NTHI477_RS00120	129	G	A	synonymous
24767	NTHI477_RS00120	126	C	T	synonymous
24773	NTHI477_RS00120	120	C	T	synonymous
24779	NTHI477_RS00120	114	A	G	synonymous
24836	NTHI477_RS00120	57	A	T	nonsynonymous
24845	NTHI477_RS00120	48	G	A	synonymous
24889	NTHI477_RS00120	4	G	A	nonsynonymous
24995	NTHI477_RS00125	33	T	C	synonymous
24996	NTHI477_RS00125	34	A	C	synonymous

25010	NTHI477_RS00125	48	G	A	synonymous
25023	NTHI477_RS00125	61	A	G	nonsynonymous
25037	NTHI477_RS00125	75	T	C	synonymous
25071	NTHI477_RS00125	109	C	T	nonsynonymous
25073	NTHI477_RS00125	111	T	G	synonymous
25076	NTHI477_RS00125	114	C	A	synonymous
25077	NTHI477_RS00125	115	G	A	nonsynonymous
25082	NTHI477_RS00125	120	A	G	synonymous
25097	NTHI477_RS00125	135	G	T	synonymous
25103	NTHI477_RS00125	141	A	G	synonymous
25112	NTHI477_RS00125	150	T	G	synonymous
25118	NTHI477_RS00125	156	C	T	synonymous
25127	NTHI477_RS00125	165	C	T	synonymous
25143	NTHI477_RS00125	181	G	A	nonsynonymous
25173	NTHI477_RS00125	211	T	C	synonymous
25190	NTHI477_RS00125	228	G	A	synonymous
25217	NTHI477_RS00125	255	C	T	synonymous
25241	NTHI477_RS00125	279	T	C	synonymous
25244	NTHI477_RS00125	282	C	T	synonymous
25247	NTHI477_RS00125	285	G	A	synonymous
25253	NTHI477_RS00125	291	G	A	synonymous
25259	NTHI477_RS00125	297	C	T	synonymous
25262	NTHI477_RS00125	300	T	C	synonymous
25265	NTHI477_RS00125	303	C	T	synonymous
25304	NTHI477_RS00125	342	T	C	synonymous
25313	NTHI477_RS00125	351	T	C	synonymous
25316	NTHI477_RS00125	354	T	C	synonymous
25322	NTHI477_RS00125	360	G	A	synonymous
25329	NTHI477_RS00125	367	G	A	nonsynonymous
25352	NTHI477_RS00125	390	G	A	synonymous
25355	NTHI477_RS00125	393	G	A	synonymous
25376	NTHI477_RS00125	414	T	C	synonymous
25409	NTHI477_RS00125	447	T	A	synonymous
25412	NTHI477_RS00125	450	G	T	synonymous
25414	NTHI477_RS00125	452	G	A	nonsynonymous
25420	NTHI477_RS00125	458	C	A	nonsynonymous
25424	NTHI477_RS00125	462	T	A	synonymous
25425	NTHI477_RS00125	463	C	T	nonsynonymous
25427	NTHI477_RS00125	465	C	A	synonymous
25452	NTHI477_RS00125	490	T	C	synonymous
25455	NTHI477_RS00125	493	G	A	nonsynonymous
25457	NTHI477_RS00125	495	A	G	synonymous
25460	NTHI477_RS00125	498	A	G	synonymous
25468	NTHI477_RS00125	506	G	A	nonsynonymous
25484	NTHI477_RS00125	522	C	A	nonsynonymous

25485	NTHI477_RS00125	523	C	A	nonsynonymous
25487	NTHI477_RS00125	525	A	G	synonymous
25488	NTHI477_RS00125	526	C	T	nonsynonymous
25544	NTHI477_RS00125	582	T	C	synonymous
25569	NTHI477_RS00125	607	C	A	nonsynonymous
25586	NTHI477_RS00125	624	G	A	synonymous
25607	NTHI477_RS00125	645	T	A	synonymous
25655	NTHI477_RS00125	693	C	T	synonymous
25667	NTHI477_RS00125	705	G	A	synonymous
25670	NTHI477_RS00125	708	T	C	synonymous
25688	NTHI477_RS00125	726	C	G	synonymous
25689	NTHI477_RS00125	727	T	C	synonymous
25712	NTHI477_RS00125	750	G	A	synonymous
25715	NTHI477_RS00125	753	A	G	synonymous
25724	NTHI477_RS00125	762	C	T	synonymous
25736	NTHI477_RS00125	774	A	G	synonymous
25778	NTHI477_RS00125	816	C	G	synonymous
25793	NTHI477_RS00125	831	T	C	synonymous
25796	NTHI477_RS00125	834	A	C	synonymous
25796	NTHI477_RS00125	834	A	G	synonymous
25814	NTHI477_RS00125	852	C	T	synonymous
25842	NTHI477_RS00125	880	A	T	nonsynonymous
25844	NTHI477_RS00125	882	C	T	synonymous
25877	NTHI477_RS00125	915	C	T	synonymous
25880	NTHI477_RS00125	918	C	A	synonymous
25880	NTHI477_RS00125	918	C	T	synonymous
25883	NTHI477_RS00125	921	A	G	synonymous
25892	NTHI477_RS00125	930	G	A	synonymous
25913	NTHI477_RS00125	951	C	G	synonymous
25926	NTHI477_RS00125	964	G	A	nonsynonymous
25939	NTHI477_RS00125	977	G	A	nonsynonymous
25940	NTHI477_RS00125	978	C	T	synonymous
25995	NTHI477_RS00125	1033	T	G	nonsynonymous
26003	NTHI477_RS00125	1041	A	G	synonymous
26030	intergenic	intergenic	A	G	intergenic
26059	intergenic	intergenic	C	A	intergenic
26060	intergenic	intergenic	A	G	intergenic
26069	intergenic	intergenic	A	G	intergenic
26092	intergenic	intergenic	T	C	intergenic
26094	intergenic	intergenic	T	C	intergenic
26114	intergenic	intergenic	A	G	intergenic
26116	intergenic	intergenic	T	C	intergenic
26119	intergenic	intergenic	T	C	intergenic
26121	intergenic	intergenic	T	C	intergenic
26122	intergenic	intergenic	G	A	intergenic

26125	intergenic	intergenic	T	G	intergenic
26128	intergenic	intergenic	A	T	intergenic
26128	intergenic	intergenic	A	C	intergenic
26130	intergenic	intergenic	A	C	intergenic
26138	intergenic	intergenic	T	C	intergenic
26139	intergenic	intergenic	T	C	intergenic
26143	intergenic	intergenic	G	A	intergenic
26144	intergenic	intergenic	A	T	intergenic
26150	intergenic	intergenic	C	A	intergenic
26167	NTHI477_RS00130	6	T	C	synonymous
26179	NTHI477_RS00130	18	G	T	synonymous
26185	NTHI477_RS00130	24	T	C	synonymous
26187	NTHI477_RS00130	26	T	C	nonsynonymous
26188	NTHI477_RS00130	27	T	C	synonymous
26191	NTHI477_RS00130	30	A	G	synonymous
26192	NTHI477_RS00130	31	C	T	nonsynonymous
26196	NTHI477_RS00130	35	A	T	nonsynonymous
26209	NTHI477_RS00130	48	G	T	synonymous
26212	NTHI477_RS00130	51	C	T	synonymous
26218	NTHI477_RS00130	57	T	A	synonymous
26218	NTHI477_RS00130	57	T	C	synonymous
26257	NTHI477_RS00130	96	A	T	nonsynonymous
26260	NTHI477_RS00130	99	T	C	synonymous
26269	NTHI477_RS00130	108	C	G	synonymous
26272	NTHI477_RS00130	111	A	T	synonymous
26274	NTHI477_RS00130	113	G	C	nonsynonymous
26275	NTHI477_RS00130	114	G	C	synonymous
26278	NTHI477_RS00130	117	G	T	synonymous
26278	NTHI477_RS00130	117	G	A	synonymous
26281	NTHI477_RS00130	120	A	G	synonymous
26284	NTHI477_RS00130	123	T	C	synonymous
26285	NTHI477_RS00130	124	G	A	nonsynonymous
26296	NTHI477_RS00130	135	G	A	synonymous
26299	NTHI477_RS00130	138	C	T	synonymous
26302	NTHI477_RS00130	141	A	G	synonymous
26309	NTHI477_RS00130	148	T	C	synonymous
26311	NTHI477_RS00130	150	G	A	synonymous
26322	NTHI477_RS00130	161	T	G	nonsynonymous
26322	NTHI477_RS00130	161	T	C	nonsynonymous
26323	NTHI477_RS00130	162	T	G	synonymous
26329	NTHI477_RS00130	168	G	A	synonymous
26339	NTHI477_RS00130	178	A	G	nonsynonymous
26340	NTHI477_RS00130	179	T	C	nonsynonymous
26341	NTHI477_RS00130	180	A	G	nonsynonymous
26342	NTHI477_RS00130	181	C	A	nonsynonymous

26356	NTHI477_RS00130	195	T	A	synonymous
26359	NTHI477_RS00130	198	T	A	nonsynonymous
26362	NTHI477_RS00130	201	A	T	synonymous
26368	NTHI477_RS00130	207	C	T	synonymous
26374	NTHI477_RS00130	213	A	C	nonsynonymous
26380	NTHI477_RS00130	219	T	A	synonymous
26383	NTHI477_RS00130	222	A	T	synonymous
26386	NTHI477_RS00130	225	C	A	nonsynonymous
26387	NTHI477_RS00130	226	C	T	nonsynonymous
26395	NTHI477_RS00130	234	C	T	synonymous
26398	NTHI477_RS00130	237	G	T	synonymous
26406	NTHI477_RS00130	245	C	G	nonsynonymous
26407	NTHI477_RS00130	246	A	T	synonymous
26410	NTHI477_RS00130	249	T	C	synonymous
26421	intergenic	intergenic	T	A	intergenic
26424	intergenic	intergenic	T	G	intergenic
26442	intergenic	intergenic	T	A	intergenic
26466	intergenic	intergenic	T	C	intergenic
26479	intergenic	intergenic	A	C	intergenic
26484	intergenic	intergenic	G	T	intergenic
26491	intergenic	intergenic	G	A	intergenic
26524	NTHI477_RS00135	18	T	C	synonymous
26530	NTHI477_RS00135	24	T	C	synonymous
26533	NTHI477_RS00135	27	G	A	synonymous
26590	NTHI477_RS00135	84	A	G	synonymous
26602	NTHI477_RS00135	96	T	A	synonymous
26617	NTHI477_RS00135	111	C	T	synonymous
26619	NTHI477_RS00135	113	T	C	nonsynonymous
26619	NTHI477_RS00135	113	T	A	nonsynonymous
26626	NTHI477_RS00135	120	C	T	synonymous
26632	NTHI477_RS00135	126	A	G	synonymous
26638	NTHI477_RS00135	132	T	C	synonymous
26644	NTHI477_RS00135	138	G	T	synonymous
26644	NTHI477_RS00135	138	G	A	synonymous
26647	NTHI477_RS00135	141	T	A	synonymous
26648	NTHI477_RS00135	142	G	A	nonsynonymous
26649	NTHI477_RS00135	143	C	A	nonsynonymous
26650	NTHI477_RS00135	144	T	A	synonymous
26650	NTHI477_RS00135	144	T	C	synonymous
26651	NTHI477_RS00135	145	G	T	nonsynonymous
26652	NTHI477_RS00135	146	C	T	nonsynonymous
26654	NTHI477_RS00135	148	G	A	nonsynonymous
26659	NTHI477_RS00135	153	A	G	synonymous
26677	NTHI477_RS00135	171	C	T	synonymous
26683	NTHI477_RS00135	177	A	G	synonymous

26686	NTHI477_RS00135	180	T	C	synonymous
26693	NTHI477_RS00135	187	A	G	nonsynonymous
26707	NTHI477_RS00135	201	A	G	synonymous
26716	NTHI477_RS00135	210	G	C	synonymous
26719	NTHI477_RS00135	213	A	G	synonymous
26755	NTHI477_RS00135	249	A	G	synonymous
26797	NTHI477_RS00135	291	G	A	synonymous
26800	NTHI477_RS00135	294	G	C	synonymous
26806	NTHI477_RS00135	300	A	C	synonymous
26808	NTHI477_RS00135	302	G	C	nonsynonymous
26809	NTHI477_RS00135	303	C	A	synonymous
26809	NTHI477_RS00135	303	C	G	synonymous
26815	NTHI477_RS00135	309	G	A	synonymous
26828	NTHI477_RS00135	322	T	A	nonsynonymous
26830	NTHI477_RS00135	324	A	T	nonsynonymous
26842	NTHI477_RS00135	336	G	C	synonymous
26851	NTHI477_RS00135	345	A	G	synonymous
26854	NTHI477_RS00135	348	T	G	synonymous
26857	NTHI477_RS00135	351	A	G	synonymous
26860	NTHI477_RS00135	354	T	C	synonymous
26881	NTHI477_RS00135	375	A	G	synonymous
26887	NTHI477_RS00135	381	A	G	synonymous
26896	NTHI477_RS00135	390	T	C	synonymous
26899	NTHI477_RS00135	393	G	T	synonymous
26907	NTHI477_RS00135	401	C	G	nonsynonymous
26911	NTHI477_RS00135	405	C	T	synonymous
26918	NTHI477_RS00135	412	G	A	nonsynonymous
26935	NTHI477_RS00135	429	A	C	synonymous
26939	NTHI477_RS00135	433	T	C	nonsynonymous
26944	NTHI477_RS00135	438	T	A	synonymous
26947	NTHI477_RS00135	441	T	A	synonymous
26956	NTHI477_RS00135	450	T	C	synonymous
26965	NTHI477_RS00135	459	G	A	synonymous
26974	NTHI477_RS00135	468	T	A	synonymous
27010	NTHI477_RS00135	504	A	C	synonymous
27013	NTHI477_RS00135	507	T	C	synonymous
27025	NTHI477_RS00135	519	T	C	synonymous
27040	NTHI477_RS00135	534	G	A	synonymous
27061	NTHI477_RS00135	555	T	C	synonymous
27067	NTHI477_RS00135	561	C	T	synonymous
27070	NTHI477_RS00135	564	A	C	synonymous
27079	NTHI477_RS00135	573	T	G	synonymous
27088	NTHI477_RS00135	582	G	A	synonymous
27088	NTHI477_RS00135	582	G	T	synonymous
27157	NTHI477_RS00135	651	T	C	synonymous

27172	NTHI477_RS00135	666	T	G	synonymous
27175	NTHI477_RS00135	669	C	A	synonymous
27181	NTHI477_RS00135	675	C	T	synonymous
27196	NTHI477_RS00135	690	T	C	synonymous
27202	NTHI477_RS00135	696	A	G	synonymous
27203	NTHI477_RS00135	697	G	C	nonsynonymous
27211	NTHI477_RS00135	705	G	A	synonymous
27286	NTHI477_RS00135	780	T	A	synonymous
27289	NTHI477_RS00135	783	A	T	synonymous
27292	NTHI477_RS00135	786	C	T	synonymous
27298	NTHI477_RS00135	792	A	G	synonymous
27317	NTHI477_RS00135	811	G	A	nonsynonymous
27319	NTHI477_RS00135	813	G	C	synonymous
27322	NTHI477_RS00135	816	T	C	synonymous
27325	NTHI477_RS00135	819	G	A	synonymous
27340	NTHI477_RS00135	834	T	C	synonymous
27352	NTHI477_RS00135	846	C	T	synonymous
27355	NTHI477_RS00135	849	C	G	synonymous
27370	NTHI477_RS00135	864	A	G	synonymous
27382	NTHI477_RS00135	876	T	A	synonymous
27386	NTHI477_RS00135	880	T	C	synonymous
27403	NTHI477_RS00135	897	C	T	synonymous
27421	NTHI477_RS00135	915	A	G	synonymous
27451	NTHI477_RS00135	945	T	A	synonymous
27457	NTHI477_RS00135	951	T	C	synonymous
27487	NTHI477_RS00135	981	T	C	synonymous
27493	NTHI477_RS00135	987	A	C	synonymous
27493	NTHI477_RS00135	987	A	T	synonymous
27497	NTHI477_RS00135	991	C	A	nonsynonymous
27505	NTHI477_RS00135	999	T	G	synonymous
27517	NTHI477_RS00135	1011	T	C	synonymous
27533	NTHI477_RS00135	1027	T	C	nonsynonymous
27583	NTHI477_RS00135	1077	G	A	synonymous
27595	NTHI477_RS00135	1089	A	G	synonymous
27604	NTHI477_RS00135	1098	T	C	synonymous
27631	NTHI477_RS00135	1125	T	C	synonymous
27637	NTHI477_RS00135	1131	G	A	synonymous
27640	NTHI477_RS00135	1134	G	A	synonymous
27652	NTHI477_RS00135	1146	G	C	synonymous
27652	NTHI477_RS00135	1146	G	T	synonymous
27667	NTHI477_RS00135	1161	G	T	synonymous
27667	NTHI477_RS00135	1161	G	A	synonymous
27670	NTHI477_RS00135	1164	G	A	synonymous
27676	NTHI477_RS00135	1170	T	C	synonymous
27691	NTHI477_RS00135	1185	T	G	synonymous

27706	NTHI477_RS00135	1200	G	A	synonymous
27736	NTHI477_RS00135	1230	A	G	synonymous
27750	NTHI477_RS00135	1244	A	C	nonsynonymous
27754	NTHI477_RS00135	1248	G	A	synonymous
27761	NTHI477_RS00135	1255	C	G	nonsynonymous
27763	NTHI477_RS00135	1257	G	T	synonymous
27775	NTHI477_RS00135	1269	T	C	synonymous
27778	NTHI477_RS00135	1272	C	A	synonymous
27784	NTHI477_RS00135	1278	T	G	synonymous
27787	NTHI477_RS00135	1281	T	G	synonymous
27790	NTHI477_RS00135	1284	A	C	synonymous
27796	NTHI477_RS00135	1290	C	A	synonymous
27796	NTHI477_RS00135	1290	C	T	synonymous
27802	NTHI477_RS00135	1296	C	T	synonymous
27808	NTHI477_RS00135	1302	T	A	synonymous
27811	NTHI477_RS00135	1305	C	T	synonymous
27811	NTHI477_RS00135	1305	C	G	synonymous
27814	NTHI477_RS00135	1308	G	A	synonymous
27844	NTHI477_RS00135	1338	A	G	synonymous
27853	NTHI477_RS00135	1347	T	C	synonymous
27859	NTHI477_RS00135	1353	C	T	synonymous
27862	NTHI477_RS00135	1356	T	A	synonymous
27868	NTHI477_RS00135	1362	C	T	synonymous
27877	NTHI477_RS00135	1371	T	C	synonymous
27934	NTHI477_RS00135	1428	G	A	synonymous
27940	NTHI477_RS00135	1434	C	T	synonymous
27943	NTHI477_RS00135	1437	C	T	synonymous
27979	NTHI477_RS00135	1473	C	T	synonymous
27986	NTHI477_RS00135	1480	G	A	nonsynonymous
27988	NTHI477_RS00135	1482	A	T	synonymous
27991	NTHI477_RS00135	1485	A	G	synonymous
27994	NTHI477_RS00135	1488	A	C	synonymous
28003	NTHI477_RS00135	1497	G	A	synonymous
28024	NTHI477_RS00135	1518	A	T	synonymous
28036	NTHI477_RS00135	1530	T	C	synonymous
28039	NTHI477_RS00135	1533	G	A	synonymous
28048	NTHI477_RS00135	1542	A	G	synonymous
28051	NTHI477_RS00135	1545	G	A	synonymous
28063	NTHI477_RS00135	1557	A	G	synonymous
28066	NTHI477_RS00135	1560	A	G	synonymous
28078	NTHI477_RS00135	1572	T	C	synonymous
28102	NTHI477_RS00135	1596	A	C	synonymous
28126	NTHI477_RS00135	1620	A	G	synonymous
28132	NTHI477_RS00135	1626	T	C	synonymous
28133	NTHI477_RS00135	1627	C	G	nonsynonymous

28146	NTHI477_RS00135	1640	T	C	nonsynonymous
28157	NTHI477_RS00135	1651	A	G	nonsynonymous
28159	NTHI477_RS00135	1653	T	C	synonymous
28177	NTHI477_RS00135	1671	C	T	synonymous
28241	intergenic	intergenic	T	G	intergenic
28267	intergenic	intergenic	G	A	intergenic
28267	intergenic	intergenic	G	T	intergenic
28398	NTHI477_RS00140	105	C	T	synonymous
28404	NTHI477_RS00140	111	G	A	synonymous
28422	NTHI477_RS00140	129	T	C	synonymous
28428	NTHI477_RS00140	135	T	C	synonymous
28435	NTHI477_RS00140	142	G	A	nonsynonymous
28464	NTHI477_RS00140	171	C	T	synonymous
28470	NTHI477_RS00140	177	A	G	synonymous
28539	NTHI477_RS00140	246	C	G	synonymous
28581	NTHI477_RS00140	288	A	G	synonymous
28584	NTHI477_RS00140	291	G	A	synonymous
28587	NTHI477_RS00140	294	G	C	synonymous
28608	NTHI477_RS00140	315	A	G	synonymous
28617	NTHI477_RS00140	324	G	T	synonymous
28624	NTHI477_RS00140	331	G	A	nonsynonymous
28633	NTHI477_RS00140	340	A	G	nonsynonymous
28635	NTHI477_RS00140	342	C	T	synonymous
28641	NTHI477_RS00140	348	T	C	synonymous
28660	NTHI477_RS00140	367	G	C	nonsynonymous
28662	NTHI477_RS00140	369	G	T	synonymous
28663	NTHI477_RS00140	370	C	T	synonymous
28719	NTHI477_RS00140	426	C	T	synonymous
28773	NTHI477_RS00140	480	G	A	synonymous
28805	intergenic	intergenic	G	A	intergenic
28831	intergenic	intergenic	C	T	intergenic
28846	intergenic	intergenic	A	T	intergenic
28847	intergenic	intergenic	G	A	intergenic
28849	intergenic	intergenic	T	G	intergenic
28860	intergenic	intergenic	A	G	intergenic
28925	intergenic	intergenic	G	T	intergenic
28926	intergenic	intergenic	A	T	intergenic
28958	intergenic	intergenic	G	A	intergenic
28959	intergenic	intergenic	T	C	intergenic
28975	intergenic	intergenic	C	A	intergenic
29029	NTHI477_RS00145	53	A	G	nonsynonymous
29072	NTHI477_RS00145	96	C	T	synonymous
29084	NTHI477_RS00145	108	C	T	synonymous
29087	NTHI477_RS00145	111	A	G	synonymous
29105	NTHI477_RS00145	129	C	T	synonymous

29106	NTHI477_RS00145	130	G	A	nonsynonymous
29110	NTHI477_RS00145	134	C	T	nonsynonymous
29111	NTHI477_RS00145	135	T	A	synonymous
29117	NTHI477_RS00145	141	T	C	synonymous
29120	NTHI477_RS00145	144	T	A	synonymous
29125	NTHI477_RS00145	149	C	T	nonsynonymous
29127	NTHI477_RS00145	151	A	G	nonsynonymous
29127	NTHI477_RS00145	151	A	C	nonsynonymous
29128	NTHI477_RS00145	152	A	G	nonsynonymous
29132	NTHI477_RS00145	156	T	G	synonymous
29133	NTHI477_RS00145	157	T	G	nonsynonymous
29146	NTHI477_RS00145	170	A	G	nonsynonymous
29147	NTHI477_RS00145	171	C	A	nonsynonymous
29150	NTHI477_RS00145	174	C	A	synonymous
29155	NTHI477_RS00145	179	T	C	nonsynonymous
29177	NTHI477_RS00145	201	G	A	synonymous
29193	intergenic	intergenic	A	G	intergenic
29199	intergenic	intergenic	A	T	intergenic
29200	intergenic	intergenic	C	T	intergenic
29253	intergenic	intergenic	A	G	intergenic
29270	intergenic	intergenic	T	A	intergenic
29301	intergenic	intergenic	G	A	intergenic
29303	intergenic	intergenic	T	C	intergenic
29400	NTHI477_RS00150	97	A	G	nonsynonymous
29411	NTHI477_RS00150	108	T	A	synonymous
29420	NTHI477_RS00150	117	C	T	synonymous
29441	NTHI477_RS00150	138	G	A	synonymous
29465	NTHI477_RS00150	162	G	A	synonymous
29471	NTHI477_RS00150	168	T	G	synonymous
29477	NTHI477_RS00150	174	T	C	synonymous
29480	NTHI477_RS00150	177	A	T	synonymous
29483	NTHI477_RS00150	180	A	G	synonymous
29501	NTHI477_RS00150	198	A	G	synonymous
29522	NTHI477_RS00150	219	A	T	synonymous
29543	NTHI477_RS00150	240	C	T	synonymous
29552	NTHI477_RS00150	249	T	C	synonymous
29570	NTHI477_RS00150	267	T	C	synonymous
29576	NTHI477_RS00150	273	G	T	nonsynonymous
29579	NTHI477_RS00150	276	A	T	synonymous
29609	NTHI477_RS00150	306	G	A	synonymous
29615	NTHI477_RS00150	312	T	C	synonymous
29618	NTHI477_RS00150	315	A	G	synonymous
29620	NTHI477_RS00150	317	C	T	nonsynonymous
29621	NTHI477_RS00150	318	C	A	synonymous
29633	NTHI477_RS00150	330	A	G	synonymous

29636	NTHI477_RS00150	333	A	G	synonymous
29648	NTHI477_RS00150	345	G	A	synonymous
29651	NTHI477_RS00150	348	C	T	synonymous
29652	NTHI477_RS00150	349	G	A	nonsynonymous
29654	NTHI477_RS00150	351	T	C	synonymous
29662	NTHI477_RS00150	359	C	T	nonsynonymous
29671	intergenic	intergenic	T	C	intergenic
29678	intergenic	intergenic	G	C	intergenic
29681	intergenic	intergenic	G	A	intergenic
29684	intergenic	intergenic	A	G	intergenic
29691	intergenic	intergenic	G	A	intergenic
29710	intergenic	intergenic	C	T	intergenic
29747	NTHI477_RS00155	15	G	A	synonymous
29756	NTHI477_RS00155	24	C	T	synonymous
29771	NTHI477_RS00155	39	C	T	synonymous
29778	NTHI477_RS00155	46	T	C	synonymous
29780	NTHI477_RS00155	48	G	A	synonymous
29795	NTHI477_RS00155	63	T	C	synonymous
29825	NTHI477_RS00155	93	C	T	synonymous
29844	NTHI477_RS00155	112	G	A	nonsynonymous
29845	NTHI477_RS00155	113	C	T	nonsynonymous
29861	NTHI477_RS00155	129	A	G	synonymous
29862	NTHI477_RS00155	130	C	T	nonsynonymous
29891	NTHI477_RS00155	159	C	T	synonymous
29906	NTHI477_RS00155	174	T	C	synonymous
29915	NTHI477_RS00155	183	T	G	nonsynonymous
29931	NTHI477_RS00155	199	A	C	synonymous
29933	NTHI477_RS00155	201	A	T	nonsynonymous
29939	NTHI477_RS00155	207	T	G	nonsynonymous
29951	NTHI477_RS00155	219	T	A	synonymous
29960	NTHI477_RS00155	228	A	G	synonymous
29966	NTHI477_RS00155	234	G	A	synonymous
29972	NTHI477_RS00155	240	T	C	synonymous
29975	NTHI477_RS00155	243	T	C	synonymous
29981	NTHI477_RS00155	249	T	C	synonymous
29993	NTHI477_RS00155	261	A	T	synonymous
29996	NTHI477_RS00155	264	T	C	synonymous
30011	NTHI477_RS00155	279	T	C	synonymous
30017	NTHI477_RS00155	285	A	C	synonymous
30026	NTHI477_RS00155	294	T	C	synonymous
30056	NTHI477_RS00155	324	C	T	synonymous
30074	NTHI477_RS00155	342	C	T	synonymous
30095	NTHI477_RS00155	363	A	T	synonymous
30096	NTHI477_RS00155	364	C	T	nonsynonymous
30107	NTHI477_RS00155	375	G	T	synonymous

30110	NTHI477_RS00155	378	A	G	synonymous
30120	NTHI477_RS00155	388	T	G	nonsynonymous
30125	NTHI477_RS00155	393	T	G	synonymous
30134	NTHI477_RS00155	402	C	A	synonymous
30151	NTHI477_RS00155	419	C	T	nonsynonymous
30152	NTHI477_RS00155	420	T	C	synonymous
30173	NTHI477_RS00155	441	C	T	synonymous
30203	NTHI477_RS00155	471	A	G	synonymous
30204	NTHI477_RS00155	472	T	C	synonymous
30206	NTHI477_RS00155	474	A	T	nonsynonymous
30213	NTHI477_RS00155	481	C	T	nonsynonymous
30219	NTHI477_RS00155	487	C	T	synonymous
30233	NTHI477_RS00155	501	T	C	synonymous
30236	NTHI477_RS00155	504	G	A	synonymous
30246	NTHI477_RS00155	514	A	T	nonsynonymous
30251	NTHI477_RS00155	519	G	C	synonymous
30251	NTHI477_RS00155	519	G	A	synonymous
30281	NTHI477_RS00155	549	A	G	synonymous
30290	NTHI477_RS00155	558	C	T	synonymous
30296	NTHI477_RS00155	564	C	T	synonymous
30341	NTHI477_RS00155	609	A	G	synonymous
30353	NTHI477_RS00155	621	A	C	synonymous
30356	NTHI477_RS00155	624	A	G	synonymous
30371	NTHI477_RS00155	639	T	G	synonymous
30371	NTHI477_RS00155	639	T	A	synonymous
30377	NTHI477_RS00155	645	G	T	synonymous
30378	NTHI477_RS00155	646	C	T	synonymous
30380	NTHI477_RS00155	648	G	A	synonymous
30387	NTHI477_RS00155	655	G	A	nonsynonymous
30409	NTHI477_RS00160	15	T	C	synonymous
30410	NTHI477_RS00160	16	C	A	nonsynonymous
30412	NTHI477_RS00160	18	C	T	synonymous
30415	NTHI477_RS00160	21	T	G	synonymous
30430	NTHI477_RS00160	36	C	T	synonymous
30438	NTHI477_RS00160	44	C	G	nonsynonymous
30454	NTHI477_RS00160	60	A	T	nonsynonymous
30461	NTHI477_RS00160	67	C	T	nonsynonymous
30463	NTHI477_RS00160	69	T	G	synonymous
30464	NTHI477_RS00160	70	A	G	nonsynonymous
30473	NTHI477_RS00160	79	C	T	nonsynonymous
30475	NTHI477_RS00160	81	T	G	synonymous
30478	NTHI477_RS00160	84	G	C	synonymous
30493	NTHI477_RS00160	99	A	G	synonymous
30493	NTHI477_RS00160	99	A	T	synonymous
30496	NTHI477_RS00160	102	T	C	synonymous

30541	NTHI477_RS00160	147	C	T	synonymous
30553	NTHI477_RS00160	159	T	A	synonymous
30559	NTHI477_RS00160	165	T	A	synonymous
30569	NTHI477_RS00160	175	G	A	nonsynonymous
30589	NTHI477_RS00160	195	C	T	synonymous
30594	NTHI477_RS00160	200	A	C	nonsynonymous
30599	NTHI477_RS00160	205	G	A	nonsynonymous
30608	NTHI477_RS00160	214	T	C	nonsynonymous
30617	NTHI477_RS00160	223	G	T	nonsynonymous
30618	NTHI477_RS00160	224	C	T	nonsynonymous
30668	NTHI477_RS00160	274	C	A	nonsynonymous
30673	NTHI477_RS00160	279	A	T	synonymous
30688	NTHI477_RS00160	294	T	C	synonymous
30697	NTHI477_RS00160	303	G	A	synonymous
30709	NTHI477_RS00160	315	A	G	synonymous
30710	NTHI477_RS00160	316	C	G	nonsynonymous
30711	NTHI477_RS00160	317	G	A	nonsynonymous
30725	NTHI477_RS00160	331	A	T	nonsynonymous
30732	NTHI477_RS00160	338	T	C	nonsynonymous
30736	NTHI477_RS00160	342	G	T	synonymous
30740	NTHI477_RS00160	346	C	T	nonsynonymous
30745	NTHI477_RS00160	351	C	T	synonymous
30746	NTHI477_RS00160	352	G	T	nonsynonymous
30747	NTHI477_RS00160	353	C	A	nonsynonymous
30748	NTHI477_RS00160	354	G	T	synonymous
30749	NTHI477_RS00160	355	A	C	nonsynonymous
30753	NTHI477_RS00160	359	T	C	nonsynonymous
30772	NTHI477_RS00160	378	A	C	nonsynonymous
30784	NTHI477_RS00160	390	T	C	synonymous
30792	NTHI477_RS00160	398	T	C	nonsynonymous
30812	NTHI477_RS00160	418	T	G	nonsynonymous
30833	NTHI477_RS00160	439	T	C	synonymous
30871	NTHI477_RS00160	477	T	G	nonsynonymous
30895	NTHI477_RS00160	501	C	G	synonymous
30913	NTHI477_RS00160	519	C	T	synonymous
30916	NTHI477_RS00160	522	T	C	synonymous
30924	NTHI477_RS00160	530	G	T	nonsynonymous
30952	NTHI477_RS00160	558	G	A	synonymous
30981	NTHI477_RS00160	587	G	C	nonsynonymous
31096	NTHI477_RS00160	702	A	C	synonymous
31105	NTHI477_RS00160	711	C	T	synonymous
31114	NTHI477_RS00160	720	A	T	synonymous
31120	NTHI477_RS00160	726	T	A	synonymous
31123	NTHI477_RS00160	729	A	T	synonymous
31174	NTHI477_RS00160	780	A	G	synonymous

31180	NTHI477_RS00160	786	C	A	synonymous
31183	NTHI477_RS00160	789	A	T	synonymous
31184	NTHI477_RS00160	790	A	G	nonsynonymous
31185	NTHI477_RS00160	791	A	G	nonsynonymous
31186	NTHI477_RS00160	792	T	C	synonymous
31194	NTHI477_RS00160	800	C	T	nonsynonymous
31195	NTHI477_RS00160	801	G	A	synonymous
31196	NTHI477_RS00160	802	T	G	nonsynonymous
31198	NTHI477_RS00160	804	T	G	synonymous
31201	NTHI477_RS00160	807	G	A	synonymous
31204	NTHI477_RS00160	810	T	A	synonymous
31207	NTHI477_RS00160	813	G	A	synonymous
31213	NTHI477_RS00160	819	C	A	synonymous
31217	NTHI477_RS00160	823	A	T	nonsynonymous
31218	NTHI477_RS00160	824	C	T	nonsynonymous
31219	NTHI477_RS00160	825	C	A	synonymous
31228	NTHI477_RS00160	834	G	C	synonymous
31234	NTHI477_RS00160	840	C	G	synonymous
31243	NTHI477_RS00160	849	G	T	synonymous
31246	NTHI477_RS00160	852	T	A	synonymous
31252	NTHI477_RS00160	858	A	G	synonymous
31255	NTHI477_RS00160	861	G	A	synonymous
31261	NTHI477_RS00160	867	A	G	synonymous
31265	NTHI477_RS00160	871	T	C	synonymous
31267	NTHI477_RS00160	873	A	T	nonsynonymous
31276	NTHI477_RS00160	882	C	T	synonymous
31279	NTHI477_RS00160	885	T	C	synonymous
31279	NTHI477_RS00160	885	T	A	synonymous
31280	NTHI477_RS00160	886	C	A	nonsynonymous
31281	NTHI477_RS00160	887	G	A	nonsynonymous
31282	NTHI477_RS00160	888	T	C	synonymous
31282	NTHI477_RS00160	888	T	A	synonymous
31283	NTHI477_RS00160	889	T	C	synonymous
31285	NTHI477_RS00160	891	G	A	synonymous
31294	NTHI477_RS00160	900	G	A	synonymous
31299	NTHI477_RS00160	905	C	A	nonsynonymous
31300	NTHI477_RS00160	906	C	A	synonymous
31303	NTHI477_RS00160	909	A	G	synonymous
31307	NTHI477_RS00160	913	A	G	nonsynonymous
31308	NTHI477_RS00160	914	C	A	nonsynonymous
31310	NTHI477_RS00160	916	C	T	synonymous
31317	NTHI477_RS00160	923	C	T	nonsynonymous
31318	NTHI477_RS00160	924	G	T	synonymous
31319	NTHI477_RS00160	925	C	A	nonsynonymous
31332	NTHI477_RS00160	938	C	G	nonsynonymous

31333	NTHI477_RS00160	939	T	C	synonymous
31336	NTHI477_RS00160	942	T	C	synonymous
31337	NTHI477_RS00160	943	G	A	nonsynonymous
31345	NTHI477_RS00160	951	A	G	synonymous
31346	NTHI477_RS00160	952	T	C	synonymous
31348	NTHI477_RS00160	954	A	G	synonymous
31352	NTHI477_RS00160	958	T	G	nonsynonymous
31390	NTHI477_RS00160	996	C	T	synonymous
31407	NTHI477_RS00160	1013	A	C	nonsynonymous
31409	NTHI477_RS00160	1015	T	A	nonsynonymous
31474	NTHI477_RS00160	1080	C	T	synonymous
31480	NTHI477_RS00160	1086	C	T	synonymous
31495	NTHI477_RS00160	1101	A	T	synonymous
31501	NTHI477_RS00160	1107	A	T	nonsynonymous
31506	NTHI477_RS00160	1112	C	T	nonsynonymous
31522	NTHI477_RS00160	1128	A	T	synonymous
31523	NTHI477_RS00160	1129	G	T	nonsynonymous
31528	NTHI477_RS00160	1134	T	C	synonymous
31531	NTHI477_RS00160	1137	T	A	synonymous
31546	NTHI477_RS00160	1152	A	G	synonymous
31549	NTHI477_RS00160	1155	T	C	synonymous
31558	NTHI477_RS00160	1164	C	T	synonymous
31567	NTHI477_RS00160	1173	G	A	synonymous
31576	NTHI477_RS00160	1182	T	C	synonymous
31591	NTHI477_RS00160	1197	T	A	synonymous
31630	NTHI477_RS00160	1236	C	A	synonymous
31642	NTHI477_RS00160	1248	G	A	synonymous
31699	NTHI477_RS00160	1305	T	C	synonymous
31702	NTHI477_RS00160	1308	A	T	synonymous
31703	NTHI477_RS00160	1309	A	G	nonsynonymous
31704	NTHI477_RS00160	1310	C	T	nonsynonymous
31711	NTHI477_RS00160	1317	A	G	synonymous
31712	NTHI477_RS00160	1318	A	G	nonsynonymous
31714	NTHI477_RS00160	1320	T	G	nonsynonymous
31715	NTHI477_RS00160	1321	C	A	nonsynonymous
31716	NTHI477_RS00160	1322	A	G	nonsynonymous
31733	NTHI477_RS00160	1339	G	T	nonsynonymous
31743	NTHI477_RS00160	1349	A	C	nonsynonymous
31809	intergenic	intergenic	C	T	intergenic
31814	intergenic	intergenic	C	T	intergenic
31816	intergenic	intergenic	C	G	intergenic
31827	intergenic	intergenic	G	A	intergenic
31858	intergenic	intergenic	A	G	intergenic
31863	intergenic	intergenic	T	C	intergenic
31934	intergenic	intergenic	C	T	intergenic

31976	intergenic	intergenic	T	G	intergenic
32043	NTHI477_RS00165	28	A	C	synonymous
32078	NTHI477_RS00165	63	A	G	synonymous
32088	NTHI477_RS00165	73	G	A	nonsynonymous
32090	NTHI477_RS00165	75	G	A	synonymous
32120	NTHI477_RS00165	105	G	A	synonymous
32132	NTHI477_RS00165	117	A	T	synonymous
32135	NTHI477_RS00165	120	G	A	synonymous
32237	NTHI477_RS00165	222	C	A	synonymous
32259	NTHI477_RS00165	244	C	T	synonymous
32267	NTHI477_RS00165	252	G	A	synonymous
32336	NTHI477_RS00165	321	G	T	synonymous
32340	NTHI477_RS00165	325	T	C	synonymous
32395	NTHI477_RS00165	380	C	T	nonsynonymous
32439	NTHI477_RS00165	424	C	T	synonymous
32453	NTHI477_RS00165	438	C	A	synonymous
32462	NTHI477_RS00165	447	T	G	synonymous
32477	NTHI477_RS00165	462	C	T	synonymous
32478	NTHI477_RS00165	463	A	G	nonsynonymous
32582	NTHI477_RS00165	567	C	T	synonymous
32591	NTHI477_RS00165	576	G	A	synonymous
32606	NTHI477_RS00165	591	A	G	synonymous
32615	NTHI477_RS00165	600	C	T	synonymous
32618	NTHI477_RS00165	603	A	G	synonymous
32621	NTHI477_RS00165	606	G	A	synonymous
32648	NTHI477_RS00165	633	C	T	synonymous
32669	NTHI477_RS00165	654	A	G	synonymous
32672	NTHI477_RS00165	657	A	G	synonymous
32696	NTHI477_RS00165	681	C	T	synonymous
32710	NTHI477_RS00165	695	G	C	nonsynonymous
32711	NTHI477_RS00165	696	T	C	synonymous
32721	NTHI477_RS00165	706	G	A	nonsynonymous
32730	NTHI477_RS00165	715	G	A	nonsynonymous
32735	NTHI477_RS00165	720	G	A	synonymous
32738	NTHI477_RS00165	723	G	A	synonymous
32753	NTHI477_RS00165	738	G	A	synonymous
32762	NTHI477_RS00165	747	T	C	synonymous
32772	NTHI477_RS00165	757	G	A	nonsynonymous
32834	NTHI477_RS00165	819	C	T	synonymous
32834	NTHI477_RS00165	819	C	G	synonymous
32837	NTHI477_RS00165	822	T	C	synonymous
32847	NTHI477_RS00165	832	G	T	nonsynonymous
32852	NTHI477_RS00165	837	A	G	synonymous
32852	NTHI477_RS00165	837	A	C	synonymous
32858	NTHI477_RS00165	843	G	A	synonymous

32891	NTHI477_RS00165	876	G	T	nonsynonymous
32894	NTHI477_RS00165	879	G	T	synonymous
32918	NTHI477_RS00165	903	A	G	synonymous
32933	NTHI477_RS00165	918	C	T	synonymous
32958	NTHI477_RS00165	943	A	G	nonsynonymous
32969	NTHI477_RS00165	954	T	C	synonymous
33035	NTHI477_RS00165	1020	C	G	synonymous
33050	NTHI477_RS00165	1035	A	G	synonymous
33077	NTHI477_RS00165	1062	T	C	synonymous
33096	NTHI477_RS00165	1081	T	C	synonymous
33105	NTHI477_RS00165	1090	A	G	nonsynonymous
33137	NTHI477_RS00165	1122	A	G	synonymous
33167	NTHI477_RS00165	1152	T	C	synonymous
33227	NTHI477_RS00165	1212	G	A	synonymous
33233	NTHI477_RS00165	1218	G	A	synonymous
33240	NTHI477_RS00165	1225	G	A	nonsynonymous
33257	NTHI477_RS00165	1242	T	C	synonymous
33302	NTHI477_RS00165	1287	C	T	synonymous
33320	NTHI477_RS00165	1305	C	T	synonymous
33323	NTHI477_RS00165	1308	T	C	synonymous
33332	NTHI477_RS00165	1317	T	A	synonymous
33350	NTHI477_RS00165	1335	G	T	synonymous
33377	NTHI477_RS00165	1362	A	C	synonymous
33380	NTHI477_RS00165	1365	T	G	synonymous
33383	NTHI477_RS00165	1368	A	G	synonymous
33389	NTHI477_RS00165	1374	T	C	synonymous
33389	NTHI477_RS00165	1374	T	A	synonymous
33398	NTHI477_RS00165	1383	A	G	synonymous
33407	NTHI477_RS00165	1392	T	A	synonymous
33413	NTHI477_RS00165	1398	G	A	synonymous
33422	NTHI477_RS00165	1407	A	G	synonymous
33476	NTHI477_RS00165	1461	A	G	synonymous
33491	NTHI477_RS00165	1476	C	T	synonymous
33557	NTHI477_RS00165	1542	C	T	synonymous
33560	NTHI477_RS00165	1545	T	C	synonymous
33638	NTHI477_RS00165	1623	T	C	synonymous
33650	NTHI477_RS00165	1635	G	A	synonymous
33721	NTHI477_RS00165	1706	G	C	nonsynonymous
33756	NTHI477_RS00165	1741	G	T	nonsynonymous
33846	NTHI477_RS00165	1831	T	C	synonymous
33866	NTHI477_RS00165	1851	C	T	synonymous
33867	NTHI477_RS00165	1852	A	G	nonsynonymous
33868	NTHI477_RS00165	1853	C	T	nonsynonymous
33875	NTHI477_RS00165	1860	T	C	synonymous
33878	NTHI477_RS00165	1863	A	G	synonymous

33885	NTHI477_RS00165	1870	G	A	nonsynonymous
33893	NTHI477_RS00165	1878	T	C	synonymous
33908	NTHI477_RS00165	1893	A	C	synonymous
33914	NTHI477_RS00165	1899	C	T	synonymous
33917	NTHI477_RS00165	1902	T	C	synonymous
33950	NTHI477_RS00165	1935	T	C	synonymous
33978	NTHI477_RS00165	1963	T	C	synonymous
34001	NTHI477_RS00165	1986	T	G	synonymous
34004	NTHI477_RS00165	1989	G	A	synonymous
34007	NTHI477_RS00165	1992	A	G	synonymous
34008	NTHI477_RS00165	1993	C	T	synonymous
34010	NTHI477_RS00165	1995	A	G	synonymous
34065	intergenic	intergenic	T	C	intergenic
34070	intergenic	intergenic	C	A	intergenic
34076	intergenic	intergenic	T	G	intergenic
34089	NTHI477_RS00170	1467	T	C	synonymous
34098	NTHI477_RS00170	1458	C	T	synonymous
34115	NTHI477_RS00170	1441	G	A	synonymous
34116	NTHI477_RS00170	1440	T	C	synonymous
34116	NTHI477_RS00170	1440	T	A	synonymous
34122	NTHI477_RS00170	1434	T	A	synonymous
34128	NTHI477_RS00170	1428	A	G	synonymous
34131	NTHI477_RS00170	1425	A	T	synonymous
34137	NTHI477_RS00170	1419	A	G	synonymous
34138	NTHI477_RS00170	1418	A	C	nonsynonymous
34157	NTHI477_RS00170	1399	A	G	synonymous
34164	NTHI477_RS00170	1392	C	A	synonymous
34164	NTHI477_RS00170	1392	C	T	synonymous
34167	NTHI477_RS00170	1389	A	G	synonymous
34170	NTHI477_RS00170	1386	T	A	synonymous
34173	NTHI477_RS00170	1383	A	G	synonymous
34185	NTHI477_RS00170	1371	T	A	synonymous
34227	NTHI477_RS00170	1329	G	C	synonymous
34227	NTHI477_RS00170	1329	G	A	synonymous
34230	NTHI477_RS00170	1326	A	T	synonymous
34239	NTHI477_RS00170	1317	G	A	synonymous
34242	NTHI477_RS00170	1314	A	C	synonymous
34248	NTHI477_RS00170	1308	G	A	synonymous
34254	NTHI477_RS00170	1302	C	G	synonymous
34254	NTHI477_RS00170	1302	C	A	synonymous
34257	NTHI477_RS00170	1299	A	G	synonymous
34260	NTHI477_RS00170	1296	A	G	synonymous
34281	NTHI477_RS00170	1275	C	T	synonymous
34287	NTHI477_RS00170	1269	C	A	synonymous
34293	NTHI477_RS00170	1263	C	T	synonymous

34338	NTHI477_RS00170	1218	A	G	synonymous
34349	NTHI477_RS00170	1207	C	A	nonsynonymous
34375	NTHI477_RS00170	1181	T	G	nonsynonymous
34376	NTHI477_RS00170	1180	C	T	nonsynonymous
34377	NTHI477_RS00170	1179	G	A	synonymous
34392	NTHI477_RS00170	1164	C	T	synonymous
34395	NTHI477_RS00170	1161	G	T	synonymous
34398	NTHI477_RS00170	1158	A	G	synonymous
34413	NTHI477_RS00170	1143	A	T	synonymous
34416	NTHI477_RS00170	1140	C	T	synonymous
34431	NTHI477_RS00170	1125	C	A	synonymous
34434	NTHI477_RS00170	1122	C	A	synonymous
34437	NTHI477_RS00170	1119	T	C	synonymous
34464	NTHI477_RS00170	1092	T	A	synonymous
34473	NTHI477_RS00170	1083	A	G	synonymous
34476	NTHI477_RS00170	1080	C	T	synonymous
34488	NTHI477_RS00170	1068	G	A	synonymous
34491	NTHI477_RS00170	1065	G	T	synonymous
34493	NTHI477_RS00170	1063	C	T	nonsynonymous
34503	NTHI477_RS00170	1053	C	G	synonymous
34509	NTHI477_RS00170	1047	T	G	synonymous
34518	NTHI477_RS00170	1038	C	T	synonymous
34524	NTHI477_RS00170	1032	A	G	synonymous
34527	NTHI477_RS00170	1029	A	G	synonymous
34530	NTHI477_RS00170	1026	A	G	synonymous
34533	NTHI477_RS00170	1023	A	C	synonymous
34542	NTHI477_RS00170	1014	G	A	synonymous
34561	NTHI477_RS00170	995	T	G	nonsynonymous
34590	NTHI477_RS00170	966	G	A	synonymous
34596	NTHI477_RS00170	960	A	T	synonymous
34608	NTHI477_RS00170	948	G	A	synonymous
34614	NTHI477_RS00170	942	T	A	synonymous
34631	NTHI477_RS00170	925	C	T	nonsynonymous
34632	NTHI477_RS00170	924	G	A	synonymous
34638	NTHI477_RS00170	918	T	A	synonymous
34641	NTHI477_RS00170	915	C	T	synonymous
34644	NTHI477_RS00170	912	A	G	synonymous
34652	NTHI477_RS00170	904	C	G	nonsynonymous
34674	NTHI477_RS00170	882	G	A	synonymous
34677	NTHI477_RS00170	879	A	G	synonymous
34683	NTHI477_RS00170	873	A	G	synonymous
34686	NTHI477_RS00170	870	T	A	synonymous
34689	NTHI477_RS00170	867	A	C	synonymous
34692	NTHI477_RS00170	864	G	A	synonymous
34698	NTHI477_RS00170	858	A	G	synonymous

34719	NTHI477_RS00170	837	A	G	synonymous
34722	NTHI477_RS00170	834	T	C	synonymous
34722	NTHI477_RS00170	834	T	A	synonymous
34728	NTHI477_RS00170	828	A	G	synonymous
34749	NTHI477_RS00170	807	T	A	synonymous
34749	NTHI477_RS00170	807	T	C	synonymous
34763	NTHI477_RS00170	793	A	G	synonymous
34770	NTHI477_RS00170	786	G	A	synonymous
34773	NTHI477_RS00170	783	G	T	synonymous
34782	NTHI477_RS00170	774	A	G	synonymous
34785	NTHI477_RS00170	771	G	T	synonymous
34791	NTHI477_RS00170	765	T	G	synonymous
34806	NTHI477_RS00170	750	T	C	synonymous
34817	NTHI477_RS00170	739	G	A	nonsynonymous
34818	NTHI477_RS00170	738	G	T	synonymous
34821	NTHI477_RS00170	735	C	T	synonymous
34826	NTHI477_RS00170	730	T	C	nonsynonymous
34829	NTHI477_RS00170	727	A	G	nonsynonymous
34833	NTHI477_RS00170	723	A	G	synonymous
34839	NTHI477_RS00170	717	T	A	synonymous
34860	NTHI477_RS00170	696	G	T	synonymous
34875	NTHI477_RS00170	681	A	G	synonymous
34890	NTHI477_RS00170	666	T	A	synonymous
34914	NTHI477_RS00170	642	T	G	nonsynonymous
34917	NTHI477_RS00170	639	T	C	synonymous
34920	NTHI477_RS00170	636	T	A	synonymous
34941	NTHI477_RS00170	615	G	T	synonymous
34974	NTHI477_RS00170	582	G	T	synonymous
34977	NTHI477_RS00170	579	G	A	synonymous
34980	NTHI477_RS00170	576	G	A	synonymous
34983	NTHI477_RS00170	573	G	A	synonymous
34989	NTHI477_RS00170	567	G	T	synonymous
34992	NTHI477_RS00170	564	T	C	synonymous
35007	NTHI477_RS00170	549	A	G	synonymous
35010	NTHI477_RS00170	546	G	A	synonymous
35028	NTHI477_RS00170	528	A	T	synonymous
35050	NTHI477_RS00170	506	G	A	nonsynonymous
35051	NTHI477_RS00170	505	C	A	nonsynonymous
35055	NTHI477_RS00170	501	G	A	synonymous
35071	NTHI477_RS00170	485	T	G	nonsynonymous
35072	NTHI477_RS00170	484	C	T	nonsynonymous
35079	NTHI477_RS00170	477	T	C	synonymous
35081	NTHI477_RS00170	475	T	C	nonsynonymous
35085	NTHI477_RS00170	471	G	A	synonymous
35094	NTHI477_RS00170	462	G	A	synonymous

35110	NTHI477_RS00170	446	G	A	nonsynonymous
35111	NTHI477_RS00170	445	C	T	nonsynonymous
35115	NTHI477_RS00170	441	C	T	synonymous
35117	NTHI477_RS00170	439	T	C	nonsynonymous
35144	NTHI477_RS00170	412	T	G	nonsynonymous
35151	NTHI477_RS00170	405	A	G	synonymous
35234	NTHI477_RS00170	322	C	T	nonsynonymous
35235	NTHI477_RS00170	321	A	G	synonymous
35262	NTHI477_RS00170	294	C	T	synonymous
35265	NTHI477_RS00170	291	T	C	synonymous
35304	NTHI477_RS00170	252	C	T	synonymous
35342	NTHI477_RS00170	214	C	G	nonsynonymous
35381	NTHI477_RS00170	175	C	T	nonsynonymous
35383	NTHI477_RS00170	173	A	G	nonsynonymous
35392	NTHI477_RS00170	164	A	G	nonsynonymous
35397	NTHI477_RS00170	159	T	G	synonymous
35397	NTHI477_RS00170	159	T	A	synonymous
35403	NTHI477_RS00170	153	T	G	synonymous
35403	NTHI477_RS00170	153	T	A	synonymous
35406	NTHI477_RS00170	150	C	A	nonsynonymous
35436	NTHI477_RS00170	120	G	A	synonymous
35437	NTHI477_RS00170	119	C	T	nonsynonymous
35451	NTHI477_RS00170	105	G	A	synonymous
35459	NTHI477_RS00170	97	C	T	nonsynonymous
35480	NTHI477_RS00170	76	C	T	nonsynonymous
35495	NTHI477_RS00170	61	C	T	nonsynonymous
35519	NTHI477_RS00170	37	T	C	nonsynonymous
35587	intergenic	intergenic	G	A	intergenic
35596	intergenic	intergenic	G	T	intergenic
35604	intergenic	intergenic	C	T	intergenic
35608	intergenic	intergenic	A	G	intergenic
35629	intergenic	intergenic	C	T	intergenic
35643	intergenic	intergenic	G	A	intergenic
35728	NTHI477_RS00175	67	C	T	synonymous
35757	NTHI477_RS00175	96	A	G	synonymous
35823	NTHI477_RS00175	162	A	G	synonymous
35832	NTHI477_RS00175	171	G	A	synonymous
35841	NTHI477_RS00175	180	T	G	synonymous
35856	NTHI477_RS00175	195	A	G	synonymous
35874	NTHI477_RS00175	213	A	G	synonymous
35895	NTHI477_RS00175	234	T	C	synonymous
35928	NTHI477_RS00175	267	C	T	synonymous
35931	NTHI477_RS00175	270	A	G	synonymous
35934	NTHI477_RS00175	273	T	C	synonymous
35940	NTHI477_RS00175	279	A	G	synonymous

35968	NTHI477_RS00175	307	T	C	synonymous
35988	NTHI477_RS00175	327	A	G	synonymous
36021	NTHI477_RS00175	360	C	T	synonymous
36033	NTHI477_RS00175	372	G	A	synonymous
36054	NTHI477_RS00175	393	G	A	nonsynonymous
36058	NTHI477_RS00175	397	A	G	nonsynonymous
36087	NTHI477_RS00175	426	C	T	synonymous
36091	NTHI477_RS00175	430	C	T	synonymous
36120	NTHI477_RS00175	459	C	T	synonymous
36123	NTHI477_RS00175	462	C	T	synonymous
36129	NTHI477_RS00175	468	C	A	nonsynonymous
36162	NTHI477_RS00175	501	C	T	synonymous
36195	NTHI477_RS00175	534	T	A	synonymous
36195	NTHI477_RS00175	534	T	G	synonymous
36223	NTHI477_RS00175	562	G	A	nonsynonymous
36228	NTHI477_RS00175	567	G	A	synonymous
36255	NTHI477_RS00175	594	A	G	synonymous
36264	NTHI477_RS00175	603	C	T	synonymous
36273	NTHI477_RS00175	612	A	C	synonymous
36295	NTHI477_RS00175	634	A	C	synonymous
36309	NTHI477_RS00175	648	T	C	synonymous
36312	NTHI477_RS00175	651	T	C	synonymous
36314	NTHI477_RS00175	653	T	C	nonsynonymous
36336	NTHI477_RS00175	675	A	G	synonymous
36353	NTHI477_RS00175	692	G	A	nonsynonymous
36354	NTHI477_RS00175	693	C	A	synonymous
36373	NTHI477_RS00175	712	G	T	nonsynonymous
36375	NTHI477_RS00175	714	A	C	synonymous
36398	NTHI477_RS00175	737	C	T	nonsynonymous
36414	NTHI477_RS00175	753	A	G	synonymous
36426	NTHI477_RS00175	765	T	C	synonymous
36622	NTHI477_RS00175	961	G	T	nonsynonymous
36648	NTHI477_RS00175	987	C	T	synonymous
36687	NTHI477_RS00175	1026	G	T	synonymous
36712	NTHI477_RS00175	1051	A	G	nonsynonymous
36741	NTHI477_RS00175	1080	T	C	synonymous
36745	NTHI477_RS00175	1084	C	A	nonsynonymous
36806	NTHI477_RS00180	45	C	T	synonymous
36807	NTHI477_RS00180	46	G	A	nonsynonymous
36812	NTHI477_RS00180	51	T	C	synonymous
36839	NTHI477_RS00180	78	G	A	synonymous
36842	NTHI477_RS00180	81	A	G	synonymous
36849	NTHI477_RS00180	88	G	A	nonsynonymous
36935	NTHI477_RS00180	174	T	A	synonymous
36938	NTHI477_RS00180	177	T	G	synonymous

36951	NTHI477_RS00180	190	A	G	nonsynonymous
36980	NTHI477_RS00180	219	T	G	synonymous
37026	NTHI477_RS00180	265	G	A	nonsynonymous
37040	NTHI477_RS00180	279	A	T	synonymous
37061	NTHI477_RS00180	300	T	A	synonymous
37064	NTHI477_RS00180	303	A	T	synonymous
37067	NTHI477_RS00180	306	G	A	nonsynonymous
37070	NTHI477_RS00180	309	C	G	synonymous
37073	NTHI477_RS00180	312	G	A	synonymous
37159	NTHI477_RS00180	398	T	C	nonsynonymous
37163	NTHI477_RS00180	402	C	T	synonymous
37187	NTHI477_RS00180	426	T	A	synonymous
37199	NTHI477_RS00180	438	A	T	synonymous
37208	NTHI477_RS00180	447	C	T	synonymous
37227	NTHI477_RS00180	466	A	C	nonsynonymous
37230	NTHI477_RS00180	469	A	C	nonsynonymous
37250	NTHI477_RS00180	489	G	T	synonymous
37253	NTHI477_RS00180	492	A	G	synonymous
37263	NTHI477_RS00180	502	G	A	nonsynonymous
37315	NTHI477_RS00180	554	C	T	nonsynonymous
37317	NTHI477_RS00180	556	G	A	nonsynonymous
37329	NTHI477_RS00180	568	T	G	nonsynonymous
37337	NTHI477_RS00180	576	A	G	synonymous
37399	NTHI477_RS00180	638	C	T	nonsynonymous
37400	NTHI477_RS00180	639	A	G	synonymous
37406	NTHI477_RS00180	645	T	G	nonsynonymous
37415	NTHI477_RS00180	654	T	C	synonymous
37427	NTHI477_RS00180	666	T	C	synonymous
37432	NTHI477_RS00180	671	T	C	nonsynonymous
37454	NTHI477_RS00180	693	A	G	synonymous
37457	NTHI477_RS00180	696	G	T	synonymous
37487	NTHI477_RS00180	726	C	T	synonymous
37508	NTHI477_RS00180	747	T	A	synonymous
37599	NTHI477_RS00180	838	A	G	nonsynonymous
37640	NTHI477_RS00180	879	C	T	synonymous
37646	NTHI477_RS00180	885	A	C	synonymous
37658	NTHI477_RS00180	897	C	T	synonymous
37664	NTHI477_RS00180	903	A	T	synonymous
37670	NTHI477_RS00180	909	C	T	synonymous
37703	NTHI477_RS00180	942	C	T	synonymous
37715	NTHI477_RS00180	954	C	T	synonymous
37738	NTHI477_RS00180	977	T	C	nonsynonymous
37742	NTHI477_RS00180	981	C	T	synonymous
37745	NTHI477_RS00180	984	G	A	synonymous
37751	NTHI477_RS00180	990	T	C	synonymous

37760	NTHI477_RS00180	999	A	T	synonymous
37763	NTHI477_RS00180	1002	C	T	synonymous
37769	NTHI477_RS00180	1008	C	T	synonymous
37772	NTHI477_RS00180	1011	C	T	synonymous
37802	NTHI477_RS00180	1041	G	A	synonymous
37817	NTHI477_RS00180	1056	G	A	synonymous
37825	NTHI477_RS00180	1064	A	G	nonsynonymous
37830	NTHI477_RS00180	1069	C	A	synonymous
37838	NTHI477_RS00180	1077	G	A	synonymous
37839	NTHI477_RS00180	1078	C	T	nonsynonymous
37847	intergenic	intergenic	G	A	intergenic
37848	intergenic	intergenic	G	A	intergenic
37853	intergenic	intergenic	A	G	intergenic
37858	intergenic	intergenic	T	C	intergenic
37872	intergenic	intergenic	T	C	intergenic
37880	intergenic	intergenic	A	G	intergenic
37884	intergenic	intergenic	A	G	intergenic
37888	intergenic	intergenic	G	A	intergenic
37889	intergenic	intergenic	G	A	intergenic
37890	intergenic	intergenic	G	A	intergenic
37891	intergenic	intergenic	G	T	intergenic
37896	intergenic	intergenic	T	C	intergenic
37899	intergenic	intergenic	A	G	intergenic
37899	intergenic	intergenic	A	T	intergenic
37919	intergenic	intergenic	C	T	intergenic
37925	intergenic	intergenic	C	A	intergenic
37942	intergenic	intergenic	G	T	intergenic
37953	intergenic	intergenic	A	G	intergenic
37982	intergenic	intergenic	C	T	intergenic
38005	intergenic	intergenic	G	A	intergenic
38020	intergenic	intergenic	G	T	intergenic
38041	NTHI477_RS00185	10	G	T	nonsynonymous
38049	NTHI477_RS00185	18	C	T	synonymous
38058	NTHI477_RS00185	27	T	C	synonymous
38091	NTHI477_RS00185	60	T	C	synonymous
38122	NTHI477_RS00185	91	T	G	nonsynonymous
38124	NTHI477_RS00185	93	T	A	synonymous
38124	NTHI477_RS00185	93	T	C	synonymous
38127	NTHI477_RS00185	96	A	G	synonymous
38154	NTHI477_RS00185	123	A	G	synonymous
38166	NTHI477_RS00185	135	G	A	synonymous
38184	NTHI477_RS00185	153	A	G	synonymous
38185	NTHI477_RS00185	154	G	A	nonsynonymous
38187	NTHI477_RS00185	156	G	T	synonymous
38190	NTHI477_RS00185	159	G	C	synonymous

38195	NTHI477_RS00185	164	T	C	nonsynonymous
38214	NTHI477_RS00185	183	C	T	synonymous
38268	NTHI477_RS00185	237	C	T	synonymous
38274	NTHI477_RS00185	243	C	T	synonymous
38319	NTHI477_RS00185	288	C	T	synonymous
38349	NTHI477_RS00185	318	T	C	synonymous
38352	NTHI477_RS00185	321	G	A	synonymous
38388	NTHI477_RS00185	357	T	C	synonymous
38394	NTHI477_RS00185	363	C	T	synonymous
38400	NTHI477_RS00185	369	A	T	synonymous
38403	NTHI477_RS00185	372	A	G	synonymous
38406	NTHI477_RS00185	375	T	C	synonymous
38409	NTHI477_RS00185	378	T	A	nonsynonymous
38413	NTHI477_RS00185	382	A	T	nonsynonymous
38414	NTHI477_RS00185	383	A	C	nonsynonymous
38415	NTHI477_RS00185	384	T	G	nonsynonymous
38415	NTHI477_RS00185	384	T	A	nonsynonymous
38418	NTHI477_RS00185	387	A	C	synonymous
38419	NTHI477_RS00185	388	C	G	nonsynonymous
38420	NTHI477_RS00185	389	A	C	nonsynonymous
38421	NTHI477_RS00185	390	A	T	nonsynonymous
38440	NTHI477_RS00185	409	C	T	nonsynonymous
38451	NTHI477_RS00185	420	T	C	synonymous
38472	NTHI477_RS00185	441	C	G	synonymous
38478	NTHI477_RS00185	447	T	C	synonymous
38484	NTHI477_RS00185	453	A	C	nonsynonymous
38493	NTHI477_RS00185	462	G	T	synonymous
38505	NTHI477_RS00185	474	T	C	synonymous
38526	NTHI477_RS00185	495	A	G	synonymous
38545	NTHI477_RS00185	514	A	G	nonsynonymous
38578	NTHI477_RS00185	547	A	G	nonsynonymous
38598	NTHI477_RS00185	567	C	A	synonymous
38598	NTHI477_RS00185	567	C	T	synonymous
38604	NTHI477_RS00185	573	C	T	synonymous
38610	NTHI477_RS00185	579	G	A	synonymous
38616	NTHI477_RS00185	585	A	T	synonymous
38622	NTHI477_RS00185	591	T	G	synonymous
38625	NTHI477_RS00185	594	A	G	synonymous
38628	NTHI477_RS00185	597	G	A	synonymous
38661	NTHI477_RS00185	630	A	G	synonymous
38670	NTHI477_RS00185	639	A	T	synonymous
38704	NTHI477_RS00185	673	G	A	nonsynonymous
38727	NTHI477_RS00185	696	G	A	synonymous
38736	NTHI477_RS00185	705	G	A	synonymous
38737	NTHI477_RS00185	706	T	C	synonymous

38742	NTHI477_RS00185	711	T	C	synonymous
38757	NTHI477_RS00185	726	C	A	synonymous
38760	NTHI477_RS00185	729	C	T	synonymous
38775	NTHI477_RS00185	744	G	A	synonymous
38785	NTHI477_RS00185	754	G	T	nonsynonymous
38787	NTHI477_RS00185	756	T	C	synonymous
38799	NTHI477_RS00185	768	G	A	synonymous
38820	NTHI477_RS00185	789	G	T	synonymous
38826	NTHI477_RS00185	795	A	G	synonymous
38841	NTHI477_RS00185	810	G	A	synonymous
38843	NTHI477_RS00185	812	C	T	nonsynonymous
38844	NTHI477_RS00185	813	A	C	synonymous
38871	NTHI477_RS00185	840	T	A	synonymous
38874	NTHI477_RS00185	843	A	T	synonymous
38877	NTHI477_RS00185	846	T	C	synonymous
38889	NTHI477_RS00185	858	C	T	synonymous
38908	NTHI477_RS00185	877	A	G	nonsynonymous
38922	NTHI477_RS00185	891	G	A	synonymous
38958	NTHI477_RS00185	927	C	T	synonymous
38982	NTHI477_RS00185	951	C	T	synonymous
38994	NTHI477_RS00185	963	G	A	synonymous
38997	NTHI477_RS00185	966	T	C	synonymous
39054	NTHI477_RS00185	1023	C	T	synonymous
39117	NTHI477_RS00185	1086	T	C	synonymous
39129	NTHI477_RS00185	1098	G	A	synonymous
39141	NTHI477_RS00185	1110	A	T	synonymous
39161	NTHI477_RS00185	1130	C	T	nonsynonymous
39177	NTHI477_RS00185	1146	T	C	synonymous
39182	NTHI477_RS00185	1151	C	T	nonsynonymous
39183	NTHI477_RS00185	1152	C	G	synonymous
39345	NTHI477_RS00185	1314	T	G	synonymous
39348	NTHI477_RS00185	1317	T	A	synonymous
39411	NTHI477_RS00185	1380	G	A	synonymous
39426	NTHI477_RS00185	1395	G	A	synonymous
39438	NTHI477_RS00185	1407	A	C	synonymous
39441	NTHI477_RS00185	1410	A	G	synonymous
39570	NTHI477_RS00185	1539	T	C	synonymous
39579	NTHI477_RS00185	1548	T	C	synonymous
39597	NTHI477_RS00185	1566	G	A	synonymous
39612	NTHI477_RS00185	1581	A	T	synonymous
39614	NTHI477_RS00185	1583	C	T	nonsynonymous
39615	NTHI477_RS00185	1584	T	C	synonymous
39621	NTHI477_RS00185	1590	A	T	synonymous
39639	NTHI477_RS00185	1608	C	T	synonymous
39645	NTHI477_RS00185	1614	T	A	synonymous

39654	NTHI477_RS00185	1623	A	G	synonymous
39660	NTHI477_RS00185	1629	C	T	synonymous
39661	NTHI477_RS00185	1630	C	T	nonsynonymous
39663	NTHI477_RS00185	1632	C	A	synonymous
39666	NTHI477_RS00185	1635	A	T	synonymous
39732	NTHI477_RS00185	1701	C	T	synonymous
39774	NTHI477_RS00185	1743	A	G	synonymous
39831	NTHI477_RS00185	1800	C	T	synonymous
39834	NTHI477_RS00185	1803	A	G	synonymous
39855	NTHI477_RS00185	1824	A	G	synonymous
39858	NTHI477_RS00185	1827	G	A	synonymous
39879	NTHI477_RS00185	1848	C	T	synonymous
39954	NTHI477_RS00185	1923	T	A	synonymous
39957	NTHI477_RS00185	1926	C	T	synonymous
39981	NTHI477_RS00185	1950	C	T	synonymous
40023	NTHI477_RS00185	1992	C	T	synonymous
40059	NTHI477_RS00185	2028	G	C	synonymous
40098	NTHI477_RS00185	2067	A	G	synonymous
40107	NTHI477_RS00185	2076	C	T	synonymous
40128	NTHI477_RS00185	2097	A	G	synonymous
40137	NTHI477_RS00185	2106	G	A	synonymous
40138	NTHI477_RS00185	2107	G	A	nonsynonymous
40146	NTHI477_RS00185	2115	G	A	synonymous
40161	NTHI477_RS00185	2130	T	C	synonymous
40176	NTHI477_RS00185	2145	A	C	synonymous
40230	NTHI477_RS00185	2199	C	T	synonymous
40304	intergenic	intergenic	T	C	intergenic
40312	intergenic	intergenic	C	T	intergenic
40359	intergenic	intergenic	T	C	intergenic
40360	intergenic	intergenic	G	A	intergenic
40415	intergenic	intergenic	T	C	intergenic
40463	intergenic	intergenic	T	G	intergenic
40474	intergenic	intergenic	G	C	intergenic
40481	intergenic	intergenic	T	C	intergenic
40511	intergenic	intergenic	G	A	intergenic
40517	intergenic	intergenic	T	G	intergenic
40534	NTHI477_RS00190	735	A	G	synonymous
40537	NTHI477_RS00190	732	A	G	synonymous
40588	NTHI477_RS00190	681	T	C	synonymous
40590	NTHI477_RS00190	679	A	G	synonymous
40591	NTHI477_RS00190	678	C	T	synonymous
40624	NTHI477_RS00190	645	T	G	synonymous
40641	NTHI477_RS00190	628	C	T	nonsynonymous
40663	NTHI477_RS00190	606	A	G	synonymous
40666	NTHI477_RS00190	603	A	T	synonymous

40717	NTHI477_RS00190	552	T	C	synonymous
40735	NTHI477_RS00190	534	A	G	synonymous
40749	NTHI477_RS00190	520	G	T	nonsynonymous
40756	NTHI477_RS00190	513	C	A	synonymous
40758	NTHI477_RS00190	511	C	T	nonsynonymous
40774	NTHI477_RS00190	495	G	A	synonymous
40822	NTHI477_RS00190	447	T	C	synonymous
40832	NTHI477_RS00190	437	T	C	nonsynonymous
40845	NTHI477_RS00190	424	T	G	nonsynonymous
40864	NTHI477_RS00190	405	A	G	synonymous
40867	NTHI477_RS00190	402	G	A	synonymous
40882	NTHI477_RS00190	387	T	G	synonymous
40897	NTHI477_RS00190	372	C	T	synonymous
40900	NTHI477_RS00190	369	G	A	synonymous
40908	NTHI477_RS00190	361	T	C	nonsynonymous
40912	NTHI477_RS00190	357	C	T	synonymous
40924	NTHI477_RS00190	345	C	T	synonymous
40968	NTHI477_RS00190	301	C	A	nonsynonymous
40969	NTHI477_RS00190	300	A	G	synonymous
41003	NTHI477_RS00190	266	T	G	nonsynonymous
41026	NTHI477_RS00190	243	G	C	synonymous
41050	NTHI477_RS00190	219	C	G	nonsynonymous
41059	NTHI477_RS00190	210	C	G	synonymous
41072	NTHI477_RS00190	197	G	A	nonsynonymous
41073	NTHI477_RS00190	196	A	T	nonsynonymous
41074	NTHI477_RS00190	195	A	G	synonymous
41076	NTHI477_RS00190	193	C	T	nonsynonymous
41085	NTHI477_RS00190	184	T	C	nonsynonymous
41110	NTHI477_RS00190	159	C	T	synonymous
41111	NTHI477_RS00190	158	A	G	nonsynonymous
41112	NTHI477_RS00190	157	C	T	nonsynonymous
41113	NTHI477_RS00190	156	A	G	synonymous
41116	NTHI477_RS00190	153	C	T	nonsynonymous
41131	NTHI477_RS00190	138	A	G	synonymous
41140	NTHI477_RS00190	129	T	G	synonymous
41143	NTHI477_RS00190	126	A	G	synonymous
41145	NTHI477_RS00190	124	T	G	nonsynonymous
41151	NTHI477_RS00190	118	G	T	nonsynonymous
41180	NTHI477_RS00190	89	C	A	nonsynonymous
41181	NTHI477_RS00190	88	A	G	nonsynonymous
41187	NTHI477_RS00190	82	T	G	nonsynonymous
41193	NTHI477_RS00190	76	C	T	nonsynonymous
41209	NTHI477_RS00190	60	T	C	synonymous
41221	NTHI477_RS00190	48	A	G	synonymous
41223	NTHI477_RS00190	46	G	A	nonsynonymous

41227	NTHI477_RS00190	42	T	C	synonymous
41239	NTHI477_RS00190	30	T	A	synonymous
41241	NTHI477_RS00190	28	T	C	nonsynonymous
41257	NTHI477_RS00190	12	G	T	synonymous
41285	intergenic	intergenic	A	G	intergenic
41292	intergenic	intergenic	G	A	intergenic
41324	intergenic	intergenic	C	T	intergenic
41368	intergenic	intergenic	G	A	intergenic
41374	intergenic	intergenic	T	C	intergenic
41375	intergenic	intergenic	C	T	intergenic
41376	intergenic	intergenic	G	A	intergenic
41390	intergenic	intergenic	T	C	intergenic
41398	intergenic	intergenic	T	C	intergenic
41420	NTHI477_RS00195	18	C	T	synonymous
41425	NTHI477_RS00195	23	T	C	nonsynonymous
41426	NTHI477_RS00195	24	T	C	synonymous
41437	NTHI477_RS00195	35	T	C	nonsynonymous
41471	NTHI477_RS00195	69	G	A	synonymous
41489	NTHI477_RS00195	87	C	G	synonymous
41507	NTHI477_RS00195	105	A	G	synonymous
41510	NTHI477_RS00195	108	G	T	synonymous
41573	NTHI477_RS00195	171	T	C	synonymous
41633	NTHI477_RS00195	231	G	A	synonymous
41636	NTHI477_RS00195	234	G	A	synonymous
41642	NTHI477_RS00195	240	C	T	synonymous
41645	NTHI477_RS00195	243	C	T	synonymous
41666	NTHI477_RS00195	264	T	C	synonymous
41670	NTHI477_RS00195	268	T	A	nonsynonymous
41672	NTHI477_RS00195	270	C	T	synonymous
41684	NTHI477_RS00195	282	T	G	nonsynonymous
41687	NTHI477_RS00195	285	C	T	synonymous
41720	NTHI477_RS00195	318	C	T	synonymous
41721	NTHI477_RS00195	319	G	A	nonsynonymous
41723	NTHI477_RS00195	321	T	C	synonymous
41741	NTHI477_RS00195	339	C	T	synonymous
41744	NTHI477_RS00195	342	A	G	synonymous
41763	NTHI477_RS00195	361	C	T	synonymous
41771	NTHI477_RS00195	369	A	G	synonymous
41802	NTHI477_RS00195	400	G	A	nonsynonymous
41822	NTHI477_RS00195	420	T	C	synonymous
41828	NTHI477_RS00195	426	T	C	synonymous
41831	NTHI477_RS00195	429	G	T	synonymous
41834	NTHI477_RS00195	432	A	T	synonymous
41837	NTHI477_RS00195	435	T	G	synonymous
41846	NTHI477_RS00195	444	G	C	synonymous

41847	NTHI477_RS00195	445	G	A	nonsynonymous
41858	NTHI477_RS00195	456	T	C	synonymous
41880	NTHI477_RS00195	478	G	A	nonsynonymous
41909	NTHI477_RS00195	507	G	A	synonymous
41912	NTHI477_RS00195	510	C	T	synonymous
41921	NTHI477_RS00195	519	C	T	synonymous
41924	NTHI477_RS00195	522	C	T	synonymous
41935	NTHI477_RS00195	533	C	A	nonsynonymous
41943	NTHI477_RS00195	541	C	T	nonsynonymous
41943	NTHI477_RS00195	541	C	A	nonsynonymous
41948	NTHI477_RS00195	546	C	A	nonsynonymous
41956	NTHI477_RS00195	554	G	A	nonsynonymous
41958	NTHI477_RS00195	556	G	A	nonsynonymous
41958	NTHI477_RS00195	556	G	T	nonsynonymous
41960	NTHI477_RS00195	558	A	G	synonymous
41962	NTHI477_RS00195	560	T	C	nonsynonymous
41962	NTHI477_RS00195	560	T	A	nonsynonymous
41964	NTHI477_RS00195	562	C	T	nonsynonymous
41966	NTHI477_RS00195	564	T	C	synonymous
41969	NTHI477_RS00195	567	G	A	synonymous
41976	NTHI477_RS00195	574	C	A	nonsynonymous
41985	NTHI477_RS00195	583	C	A	nonsynonymous
41991	NTHI477_RS00195	589	C	A	nonsynonymous
41996	NTHI477_RS00195	594	C	T	synonymous
42026	NTHI477_RS00195	624	G	A	synonymous
42032	NTHI477_RS00195	630	T	C	synonymous
42033	NTHI477_RS00195	631	A	G	nonsynonymous
42038	NTHI477_RS00195	636	A	G	synonymous
42047	NTHI477_RS00195	645	A	G	synonymous
42050	NTHI477_RS00195	648	T	C	synonymous
42065	NTHI477_RS00195	663	G	A	synonymous
42074	NTHI477_RS00195	672	T	C	synonymous
42086	NTHI477_RS00195	684	C	T	synonymous
42125	NTHI477_RS00195	723	T	C	synonymous
42128	NTHI477_RS00195	726	G	A	nonsynonymous
42131	NTHI477_RS00195	729	G	C	synonymous
42134	NTHI477_RS00195	732	T	A	synonymous
42146	NTHI477_RS00195	744	A	G	synonymous
42147	NTHI477_RS00195	745	A	G	nonsynonymous
42149	NTHI477_RS00195	747	T	C	synonymous
42152	NTHI477_RS00195	750	T	C	synonymous
42155	NTHI477_RS00195	753	T	A	synonymous
42155	NTHI477_RS00195	753	T	G	synonymous
42161	NTHI477_RS00195	759	T	A	synonymous
42185	NTHI477_RS00195	783	T	C	synonymous

42188	NTHI477_RS00195	786	C	T	synonymous
42189	NTHI477_RS00195	787	C	A	nonsynonymous
42190	NTHI477_RS00195	788	C	A	nonsynonymous
42191	NTHI477_RS00195	789	C	T	synonymous
42204	NTHI477_RS00195	802	T	G	nonsynonymous
42206	NTHI477_RS00195	804	C	T	synonymous
42218	NTHI477_RS00195	816	A	G	synonymous
42227	NTHI477_RS00195	825	G	T	synonymous
42231	NTHI477_RS00195	829	A	C	nonsynonymous
42236	NTHI477_RS00195	834	G	A	synonymous
42237	NTHI477_RS00195	835	T	C	synonymous
42269	NTHI477_RS00195	867	A	G	synonymous
42275	NTHI477_RS00195	873	T	G	synonymous
42308	NTHI477_RS00195	906	G	A	synonymous
42309	NTHI477_RS00195	907	G	A	nonsynonymous
42314	NTHI477_RS00195	912	G	A	synonymous
42315	NTHI477_RS00195	913	T	C	synonymous
42434	NTHI477_RS00195	1032	G	A	synonymous
42461	NTHI477_RS00195	1059	C	G	synonymous
42474	NTHI477_RS00195	1072	A	G	nonsynonymous
42476	NTHI477_RS00195	1074	C	T	synonymous
42485	NTHI477_RS00195	1083	G	A	synonymous
42488	NTHI477_RS00195	1086	A	G	synonymous
42497	NTHI477_RS00195	1095	A	G	synonymous
42499	NTHI477_RS00195	1097	C	T	nonsynonymous
42625	NTHI477_RS00200	21	T	C	synonymous
42676	NTHI477_RS00200	72	C	T	synonymous
42679	NTHI477_RS00200	75	G	A	synonymous
42685	NTHI477_RS00200	81	C	T	synonymous
42691	NTHI477_RS00200	87	C	A	synonymous
42694	NTHI477_RS00200	90	A	C	synonymous
42697	NTHI477_RS00200	93	T	A	synonymous
42697	NTHI477_RS00200	93	T	G	nonsynonymous
42706	NTHI477_RS00200	102	T	G	synonymous
42733	NTHI477_RS00200	129	T	C	synonymous
42745	NTHI477_RS00200	141	G	A	synonymous
42751	NTHI477_RS00200	147	G	A	synonymous
42761	NTHI477_RS00200	157	G	T	nonsynonymous
42766	NTHI477_RS00200	162	G	T	synonymous
42767	NTHI477_RS00200	163	A	G	nonsynonymous
42772	NTHI477_RS00200	168	T	C	synonymous
42807	NTHI477_RS00200	203	T	C	nonsynonymous
42808	NTHI477_RS00200	204	T	C	synonymous
42825	NTHI477_RS00200	221	A	G	nonsynonymous
42844	NTHI477_RS00200	240	G	A	synonymous

42847	NTHI477_RS00200	243	C	T	synonymous
42871	NTHI477_RS00200	267	C	T	synonymous
42914	NTHI477_RS00200	310	G	A	nonsynonymous
42928	NTHI477_RS00200	324	G	A	synonymous
42967	NTHI477_RS00200	363	A	T	synonymous
42970	NTHI477_RS00200	366	T	C	synonymous
42973	NTHI477_RS00200	369	T	C	synonymous
42973	NTHI477_RS00200	369	T	A	synonymous
42994	NTHI477_RS00200	390	C	T	synonymous
42997	NTHI477_RS00200	393	A	G	synonymous
43021	NTHI477_RS00200	417	T	C	synonymous
43030	NTHI477_RS00200	426	C	T	synonymous
43045	NTHI477_RS00200	441	G	A	synonymous
43051	NTHI477_RS00200	447	A	G	synonymous
43060	NTHI477_RS00200	456	A	T	nonsynonymous
43096	NTHI477_RS00200	492	C	T	synonymous
43111	NTHI477_RS00200	507	T	C	synonymous
43117	NTHI477_RS00200	513	T	G	nonsynonymous
43121	NTHI477_RS00200	517	C	T	nonsynonymous
43121	NTHI477_RS00200	517	C	G	nonsynonymous
43123	NTHI477_RS00200	519	A	T	synonymous
43123	NTHI477_RS00200	519	A	G	synonymous
43124	NTHI477_RS00200	520	A	G	nonsynonymous
43129	NTHI477_RS00200	525	T	C	synonymous
43130	NTHI477_RS00200	526	T	C	synonymous
43142	NTHI477_RS00200	538	A	C	nonsynonymous
43145	NTHI477_RS00200	541	G	A	nonsynonymous
43151	NTHI477_RS00200	547	G	T	nonsynonymous
43156	NTHI477_RS00200	552	T	G	nonsynonymous
43166	NTHI477_RS00200	562	A	G	nonsynonymous
43168	NTHI477_RS00200	564	G	A	synonymous
43183	NTHI477_RS00200	579	T	C	synonymous
43186	NTHI477_RS00200	582	G	A	synonymous
43195	NTHI477_RS00200	591	G	A	synonymous
43210	NTHI477_RS00200	606	G	A	synonymous
43216	NTHI477_RS00200	612	T	C	synonymous
43217	NTHI477_RS00200	613	A	C	nonsynonymous
43243	NTHI477_RS00200	639	A	T	nonsynonymous
43246	NTHI477_RS00200	642	A	G	synonymous
43247	NTHI477_RS00200	643	A	C	nonsynonymous
43250	NTHI477_RS00200	646	T	C	nonsynonymous
43257	NTHI477_RS00200	653	A	G	nonsynonymous
43258	NTHI477_RS00200	654	T	C	synonymous
43270	NTHI477_RS00200	666	C	T	synonymous
43288	NTHI477_RS00200	684	T	C	synonymous

43293	NTHI477_RS00200	689	T	G	nonsynonymous
43294	NTHI477_RS00200	690	T	G	synonymous
43297	NTHI477_RS00200	693	T	G	nonsynonymous
43315	NTHI477_RS00200	711	A	C	nonsynonymous
43318	NTHI477_RS00200	714	T	A	synonymous
43322	NTHI477_RS00200	718	T	C	synonymous
43324	NTHI477_RS00200	720	A	C	nonsynonymous
43348	NTHI477_RS00200	744	A	G	synonymous
43349	NTHI477_RS00200	745	T	A	nonsynonymous
43349	NTHI477_RS00200	745	T	C	nonsynonymous
43355	NTHI477_RS00200	751	T	G	nonsynonymous
43356	NTHI477_RS00200	752	C	T	nonsynonymous
43357	NTHI477_RS00200	753	A	G	synonymous
43358	NTHI477_RS00200	754	A	G	nonsynonymous
43364	NTHI477_RS00200	760	A	C	nonsynonymous
43366	NTHI477_RS00200	762	A	G	synonymous
43367	NTHI477_RS00200	763	A	G	nonsynonymous
43369	NTHI477_RS00200	765	A	G	nonsynonymous
43378	NTHI477_RS00200	774	T	C	synonymous
43399	NTHI477_RS00200	795	A	G	synonymous
43408	NTHI477_RS00200	804	G	A	synonymous
43447	NTHI477_RS00200	843	G	C	synonymous
43483	NTHI477_RS00200	879	T	C	synonymous
43484	NTHI477_RS00200	880	C	T	nonsynonymous
43486	NTHI477_RS00200	882	A	T	synonymous
43487	NTHI477_RS00200	883	G	A	nonsynonymous
43488	NTHI477_RS00200	884	A	T	nonsynonymous
43489	NTHI477_RS00200	885	C	G	nonsynonymous
43504	NTHI477_RS00200	900	T	A	synonymous
43520	NTHI477_RS00200	916	G	A	nonsynonymous
43544	NTHI477_RS00205	24	T	A	synonymous
43565	NTHI477_RS00205	45	T	G	synonymous
43568	NTHI477_RS00205	48	A	G	synonymous
43577	NTHI477_RS00205	57	A	C	synonymous
43583	NTHI477_RS00205	63	C	T	synonymous
43594	NTHI477_RS00205	74	T	C	nonsynonymous
43606	NTHI477_RS00205	86	C	T	nonsynonymous
43616	NTHI477_RS00205	96	A	T	synonymous
43627	NTHI477_RS00205	107	C	A	nonsynonymous
43642	NTHI477_RS00205	122	G	T	nonsynonymous
43644	NTHI477_RS00205	124	G	A	nonsynonymous
43649	NTHI477_RS00205	129	C	T	synonymous
43665	NTHI477_RS00205	145	G	A	nonsynonymous
43667	NTHI477_RS00205	147	T	C	synonymous
43689	NTHI477_RS00205	169	G	C	nonsynonymous

43704	NTHI477_RS00205	184	C	A	nonsynonymous
43724	NTHI477_RS00205	204	T	C	synonymous
43739	NTHI477_RS00205	219	T	A	synonymous
43752	NTHI477_RS00205	232	G	A	nonsynonymous
43757	NTHI477_RS00205	237	A	G	synonymous
43769	NTHI477_RS00205	249	A	G	synonymous
43780	NTHI477_RS00205	260	C	G	nonsynonymous
43780	NTHI477_RS00205	260	C	A	nonsynonymous
43802	NTHI477_RS00205	282	A	G	synonymous
43847	NTHI477_RS00205	327	A	G	synonymous
43877	NTHI477_RS00205	357	T	C	synonymous
43883	NTHI477_RS00205	363	G	T	synonymous
43893	NTHI477_RS00205	373	T	C	nonsynonymous
43895	NTHI477_RS00205	375	C	A	nonsynonymous
43901	NTHI477_RS00205	381	C	T	synonymous
43904	NTHI477_RS00205	384	G	T	synonymous
43908	NTHI477_RS00205	388	G	A	nonsynonymous
43970	NTHI477_RS00205	450	G	T	synonymous
43972	NTHI477_RS00205	452	T	A	nonsynonymous
43993	NTHI477_RS00205	473	C	G	nonsynonymous
43994	NTHI477_RS00205	474	A	G	synonymous
43997	NTHI477_RS00205	477	T	C	synonymous
44006	NTHI477_RS00205	486	T	C	synonymous
44008	NTHI477_RS00205	488	G	C	nonsynonymous
44031	NTHI477_RS00205	511	C	T	nonsynonymous
44033	NTHI477_RS00205	513	T	G	synonymous
44042	NTHI477_RS00205	522	T	C	synonymous
44050	NTHI477_RS00205	530	C	A	nonsynonymous
44056	NTHI477_RS00205	536	G	T	nonsynonymous
44069	NTHI477_RS00205	549	T	C	synonymous
44070	NTHI477_RS00205	550	G	A	nonsynonymous
44084	NTHI477_RS00205	564	T	G	synonymous
44093	NTHI477_RS00205	573	T	G	nonsynonymous
44102	NTHI477_RS00205	582	A	C	nonsynonymous
44125	NTHI477_RS00205	605	C	T	nonsynonymous
44126	NTHI477_RS00205	606	C	T	synonymous
44138	NTHI477_RS00205	618	A	G	synonymous
44140	NTHI477_RS00205	620	C	A	nonsynonymous
44147	NTHI477_RS00205	627	T	A	nonsynonymous
44148	NTHI477_RS00205	628	C	T	nonsynonymous
44150	NTHI477_RS00205	630	C	T	synonymous
44154	NTHI477_RS00205	634	A	G	nonsynonymous
44165	NTHI477_RS00205	645	A	G	synonymous
44174	NTHI477_RS00205	654	A	G	synonymous
44177	NTHI477_RS00205	657	A	T	synonymous

44180	NTHI477_RS00205	660	G	T	synonymous
44180	NTHI477_RS00205	660	G	A	synonymous
44183	NTHI477_RS00205	663	T	A	synonymous
44183	NTHI477_RS00205	663	T	C	synonymous
44183	NTHI477_RS00205	663	T	G	synonymous
44195	NTHI477_RS00205	675	G	A	synonymous
44216	NTHI477_RS00205	696	A	G	synonymous
44217	NTHI477_RS00205	697	G	T	nonsynonymous
44218	NTHI477_RS00205	698	C	A	nonsynonymous
44223	NTHI477_RS00205	703	A	G	nonsynonymous
44229	NTHI477_RS00205	709	G	A	nonsynonymous
44248	NTHI477_RS00205	728	T	C	nonsynonymous
44255	NTHI477_RS00205	735	T	A	synonymous
44258	NTHI477_RS00205	738	T	C	synonymous
44267	NTHI477_RS00205	747	T	C	synonymous
44268	NTHI477_RS00205	748	T	G	nonsynonymous
44284	NTHI477_RS00205	764	A	G	nonsynonymous
44285	NTHI477_RS00205	765	T	C	synonymous
44318	NTHI477_RS00205	798	G	A	synonymous
44321	NTHI477_RS00205	801	C	G	synonymous
44328	NTHI477_RS00205	808	C	T	synonymous
44333	NTHI477_RS00205	813	G	C	synonymous
44334	NTHI477_RS00205	814	C	G	nonsynonymous
44342	NTHI477_RS00205	822	G	A	synonymous
44345	NTHI477_RS00205	825	G	A	synonymous
44357	NTHI477_RS00205	837	C	T	synonymous
44360	NTHI477_RS00205	840	A	G	synonymous
44363	NTHI477_RS00205	843	T	C	synonymous
44372	NTHI477_RS00205	852	C	T	synonymous
44376	NTHI477_RS00205	856	A	T	nonsynonymous
44384	NTHI477_RS00205	864	T	A	synonymous
44384	NTHI477_RS00205	864	T	G	synonymous
44385	NTHI477_RS00205	865	C	T	nonsynonymous
44387	NTHI477_RS00205	867	T	G	synonymous
44405	NTHI477_RS00205	885	G	T	synonymous
44408	NTHI477_RS00205	888	G	A	synonymous
44409	NTHI477_RS00205	889	C	A	nonsynonymous
44415	NTHI477_RS00205	895	G	A	nonsynonymous
44416	NTHI477_RS00205	896	A	C	nonsynonymous
44423	NTHI477_RS00205	903	T	A	synonymous
44436	NTHI477_RS00205	916	A	C	nonsynonymous
44443	NTHI477_RS00205	923	G	A	nonsynonymous
44462	NTHI477_RS00205	942	C	T	synonymous
44462	NTHI477_RS00205	942	C	A	nonsynonymous
44464	NTHI477_RS00205	944	G	C	nonsynonymous

44480	NTHI477_RS00205	960	G	C	nonsynonymous
44480	NTHI477_RS00205	960	G	A	nonsynonymous
44498	NTHI477_RS00205	978	T	C	synonymous
44500	NTHI477_RS00205	980	A	T	nonsynonymous
44501	NTHI477_RS00205	981	A	G	synonymous
44513	NTHI477_RS00205	993	T	A	nonsynonymous
44513	NTHI477_RS00205	993	T	C	synonymous
44520	NTHI477_RS00205	1000	C	T	nonsynonymous
44523	NTHI477_RS00205	1003	G	T	nonsynonymous
44528	NTHI477_RS00205	1008	G	C	nonsynonymous
44528	NTHI477_RS00205	1008	G	A	nonsynonymous
44535	NTHI477_RS00205	1015	G	T	nonsynonymous
44571	NTHI477_RS00205	1051	G	A	nonsynonymous
44572	NTHI477_RS00205	1052	C	T	nonsynonymous
44572	NTHI477_RS00205	1052	C	G	nonsynonymous
44573	NTHI477_RS00205	1053	G	A	synonymous
44592	intergenic	intergenic	T	G	intergenic
44635	NTHI477_RS00210	42	G	A	synonymous
44642	NTHI477_RS00210	49	G	A	nonsynonymous
44650	NTHI477_RS00210	57	T	C	synonymous
44665	NTHI477_RS00210	72	T	C	synonymous
44668	NTHI477_RS00210	75	T	C	synonymous
44681	NTHI477_RS00210	88	A	G	nonsynonymous
44683	NTHI477_RS00210	90	T	A	synonymous
44701	NTHI477_RS00210	108	C	T	synonymous
44716	NTHI477_RS00210	123	G	T	nonsynonymous
44717	NTHI477_RS00210	124	G	A	nonsynonymous
44728	NTHI477_RS00210	135	C	T	synonymous
44729	NTHI477_RS00210	136	T	A	nonsynonymous
44729	NTHI477_RS00210	136	T	G	nonsynonymous
44765	NTHI477_RS00210	172	G	T	nonsynonymous
44766	NTHI477_RS00210	173	G	T	nonsynonymous
44767	NTHI477_RS00210	174	C	A	synonymous
44767	NTHI477_RS00210	174	C	T	synonymous
44772	NTHI477_RS00210	179	G	A	nonsynonymous
44776	NTHI477_RS00210	183	C	G	synonymous
44785	NTHI477_RS00210	192	T	C	synonymous
44788	NTHI477_RS00210	195	A	G	synonymous
44791	NTHI477_RS00210	198	A	C	synonymous
44794	NTHI477_RS00210	201	C	T	synonymous
44800	NTHI477_RS00210	207	A	C	synonymous
44800	NTHI477_RS00210	207	A	T	synonymous
44809	NTHI477_RS00210	216	T	C	synonymous
44821	NTHI477_RS00210	228	C	T	synonymous
44831	NTHI477_RS00210	238	G	A	nonsynonymous

44842	NTHI477_RS00210	249	C	T	synonymous
44845	NTHI477_RS00210	252	T	C	synonymous
44848	NTHI477_RS00210	255	T	C	synonymous
44855	NTHI477_RS00210	262	G	A	nonsynonymous
44860	NTHI477_RS00210	267	C	T	synonymous
44864	NTHI477_RS00210	271	G	A	nonsynonymous
44866	NTHI477_RS00210	273	G	A	synonymous
44867	NTHI477_RS00210	274	T	C	synonymous
44881	NTHI477_RS00210	288	T	C	synonymous
44884	NTHI477_RS00210	291	T	C	synonymous
44896	NTHI477_RS00210	303	C	T	synonymous
44897	NTHI477_RS00210	304	C	T	nonsynonymous
44898	NTHI477_RS00210	305	A	C	nonsynonymous
44900	NTHI477_RS00210	307	G	A	nonsynonymous
44909	NTHI477_RS00210	316	C	A	nonsynonymous
44930	NTHI477_RS00210	337	G	T	nonsynonymous
44933	NTHI477_RS00210	340	G	A	nonsynonymous
44939	NTHI477_RS00210	346	A	C	nonsynonymous
44945	NTHI477_RS00210	352	T	G	nonsynonymous
44954	NTHI477_RS00210	361	G	A	nonsynonymous
44960	NTHI477_RS00210	367	G	C	nonsynonymous
44965	NTHI477_RS00210	372	G	A	synonymous
44974	NTHI477_RS00210	381	A	G	synonymous
44981	NTHI477_RS00210	388	G	A	nonsynonymous
44982	NTHI477_RS00210	389	A	G	nonsynonymous
44993	NTHI477_RS00210	400	T	A	nonsynonymous
44993	NTHI477_RS00210	400	T	G	nonsynonymous
44994	NTHI477_RS00210	401	T	C	nonsynonymous
45016	NTHI477_RS00210	423	G	C	synonymous
45022	NTHI477_RS00210	429	C	T	synonymous
45025	NTHI477_RS00210	432	C	T	synonymous
45032	NTHI477_RS00210	439	T	C	synonymous
45050	NTHI477_RS00210	457	A	G	nonsynonymous
45067	NTHI477_RS00210	474	C	T	synonymous
45088	NTHI477_RS00210	495	T	A	synonymous
45088	NTHI477_RS00210	495	T	C	synonymous
45091	NTHI477_RS00210	498	A	C	synonymous
45094	NTHI477_RS00210	501	T	G	synonymous
45094	NTHI477_RS00210	501	T	A	synonymous
45109	NTHI477_RS00210	516	T	A	synonymous
45121	NTHI477_RS00210	528	T	C	synonymous
45121	NTHI477_RS00210	528	T	G	synonymous
45122	NTHI477_RS00210	529	A	G	nonsynonymous
45136	NTHI477_RS00210	543	T	C	synonymous
45148	NTHI477_RS00210	555	T	C	synonymous

45154	NTHI477_RS00210	561	C	T	synonymous
45155	NTHI477_RS00210	562	A	G	nonsynonymous
45172	NTHI477_RS00210	579	C	G	synonymous
45185	NTHI477_RS00210	592	C	T	nonsynonymous
45187	NTHI477_RS00210	594	C	A	synonymous
45197	NTHI477_RS00210	604	G	A	nonsynonymous
45221	NTHI477_RS00210	628	G	A	nonsynonymous
45226	NTHI477_RS00210	633	C	T	synonymous
45232	NTHI477_RS00210	639	C	T	synonymous
45238	NTHI477_RS00210	645	C	A	synonymous
45259	NTHI477_RS00210	666	A	C	synonymous
45262	NTHI477_RS00210	669	C	T	synonymous
45275	NTHI477_RS00210	682	A	G	nonsynonymous
45277	NTHI477_RS00210	684	C	T	synonymous
45292	NTHI477_RS00210	699	A	T	synonymous
45292	NTHI477_RS00210	699	A	C	synonymous
45301	NTHI477_RS00210	708	A	G	synonymous
45302	NTHI477_RS00210	709	T	C	nonsynonymous
45328	NTHI477_RS00210	735	G	T	nonsynonymous
45360	NTHI477_RS00210	767	C	T	nonsynonymous
45459	NTHI477_RS00215	81	G	A	synonymous
45474	NTHI477_RS00215	96	G	A	synonymous
45491	NTHI477_RS00215	113	G	A	nonsynonymous
45492	NTHI477_RS00215	114	T	C	synonymous
45495	NTHI477_RS00215	117	G	C	synonymous
45501	NTHI477_RS00215	123	C	G	nonsynonymous
45502	NTHI477_RS00215	124	C	A	nonsynonymous
45503	NTHI477_RS00215	125	C	G	nonsynonymous
45504	NTHI477_RS00215	126	C	A	synonymous
45504	NTHI477_RS00215	126	C	G	synonymous
45507	NTHI477_RS00215	129	T	A	synonymous
45509	NTHI477_RS00215	131	A	G	nonsynonymous
45509	NTHI477_RS00215	131	A	T	nonsynonymous
45510	NTHI477_RS00215	132	A	C	nonsynonymous
45516	NTHI477_RS00215	138	A	G	synonymous
45520	NTHI477_RS00215	142	T	C	nonsynonymous
45521	NTHI477_RS00215	143	C	G	nonsynonymous
45522	NTHI477_RS00215	144	T	C	synonymous
45526	NTHI477_RS00215	148	C	T	nonsynonymous
45528	NTHI477_RS00215	150	A	T	synonymous
45532	NTHI477_RS00215	154	G	C	nonsynonymous
45533	NTHI477_RS00215	155	T	C	nonsynonymous
45534	NTHI477_RS00215	156	G	A	synonymous
45537	NTHI477_RS00215	159	A	T	synonymous
45539	NTHI477_RS00215	161	A	C	nonsynonymous

45540	NTHI477_RS00215	162	G	A	synonymous
45543	NTHI477_RS00215	165	C	G	synonymous
45546	NTHI477_RS00215	168	C	T	synonymous
45558	NTHI477_RS00215	180	C	G	synonymous
45573	NTHI477_RS00215	195	G	A	synonymous
45582	NTHI477_RS00215	204	C	T	synonymous
45591	NTHI477_RS00215	213	T	C	synonymous
45595	NTHI477_RS00215	217	C	T	nonsynonymous
45596	NTHI477_RS00215	218	G	A	nonsynonymous
45597	NTHI477_RS00215	219	T	C	synonymous
45609	NTHI477_RS00215	231	C	A	synonymous
45627	NTHI477_RS00215	249	C	T	synonymous
45642	NTHI477_RS00215	264	T	G	synonymous
45649	NTHI477_RS00215	271	T	C	nonsynonymous
45651	NTHI477_RS00215	273	G	A	synonymous
45652	NTHI477_RS00215	274	G	A	nonsynonymous
45659	NTHI477_RS00215	281	C	G	nonsynonymous
45667	NTHI477_RS00215	289	G	A	nonsynonymous
45670	NTHI477_RS00215	292	T	C	synonymous
45678	NTHI477_RS00215	300	T	G	synonymous
45690	NTHI477_RS00215	312	T	C	synonymous
45723	NTHI477_RS00215	345	G	A	synonymous
45749	NTHI477_RS00215	371	C	G	nonsynonymous
45761	NTHI477_RS00215	383	A	G	nonsynonymous
45783	NTHI477_RS00215	405	C	T	synonymous
45788	NTHI477_RS00215	410	T	C	nonsynonymous
45794	NTHI477_RS00215	416	G	A	nonsynonymous
45832	NTHI477_RS00215	454	G	A	nonsynonymous
45837	NTHI477_RS00215	459	A	G	synonymous
45856	NTHI477_RS00215	478	T	C	synonymous
45858	NTHI477_RS00215	480	A	T	nonsynonymous
45912	NTHI477_RS00215	534	G	A	synonymous
45922	NTHI477_RS00215	544	T	C	synonymous
45931	NTHI477_RS00215	553	C	T	nonsynonymous
45933	NTHI477_RS00215	555	A	G	synonymous
45941	NTHI477_RS00215	563	C	A	nonsynonymous
45942	NTHI477_RS00215	564	C	A	synonymous
45942	NTHI477_RS00215	564	C	T	synonymous
45960	NTHI477_RS00215	582	A	G	synonymous
45966	NTHI477_RS00215	588	A	G	synonymous
45972	NTHI477_RS00215	594	T	C	synonymous
45981	NTHI477_RS00215	603	C	T	synonymous
45987	NTHI477_RS00215	609	T	C	synonymous
45993	NTHI477_RS00215	615	C	T	synonymous
46002	NTHI477_RS00215	624	G	A	synonymous

46005	NTHI477_RS00215	627	T	A	synonymous
46014	NTHI477_RS00215	636	C	T	synonymous
46016	NTHI477_RS00215	638	C	A	nonsynonymous
46017	NTHI477_RS00215	639	T	C	synonymous
46047	NTHI477_RS00215	669	C	T	synonymous
46075	NTHI477_RS00215	697	A	G	nonsynonymous
46095	NTHI477_RS00215	717	C	T	synonymous
46098	NTHI477_RS00215	720	G	A	synonymous
46120	NTHI477_RS00215	742	C	T	nonsynonymous
46125	NTHI477_RS00215	747	C	T	synonymous
46146	NTHI477_RS00215	768	G	A	synonymous
46147	NTHI477_RS00215	769	G	A	nonsynonymous
46158	NTHI477_RS00215	780	T	C	synonymous
46173	NTHI477_RS00215	795	A	G	synonymous
46185	NTHI477_RS00215	807	T	C	synonymous
46191	NTHI477_RS00215	813	G	A	synonymous
46197	NTHI477_RS00215	819	C	T	synonymous
46197	NTHI477_RS00215	819	C	G	nonsynonymous
46206	NTHI477_RS00215	828	T	G	synonymous
46224	NTHI477_RS00215	846	G	A	synonymous
46234	NTHI477_RS00215	856	G	A	nonsynonymous
46236	NTHI477_RS00215	858	T	C	synonymous
46282	NTHI477_RS00220	18	T	A	synonymous
46321	NTHI477_RS00220	57	G	T	synonymous
46324	NTHI477_RS00220	60	G	A	synonymous
46342	NTHI477_RS00220	78	T	G	synonymous
46345	NTHI477_RS00220	81	A	T	synonymous
46350	NTHI477_RS00220	86	C	T	nonsynonymous
46372	NTHI477_RS00220	108	C	A	synonymous
46378	NTHI477_RS00220	114	T	A	synonymous
46387	NTHI477_RS00220	123	C	A	synonymous
46387	NTHI477_RS00220	123	C	T	synonymous
46388	NTHI477_RS00220	124	G	A	nonsynonymous
46389	NTHI477_RS00220	125	T	C	nonsynonymous
46390	NTHI477_RS00220	126	A	T	synonymous
46411	NTHI477_RS00220	147	T	C	synonymous
46418	NTHI477_RS00220	154	A	G	nonsynonymous
46420	NTHI477_RS00220	156	A	G	synonymous
46423	NTHI477_RS00220	159	A	G	synonymous
46436	NTHI477_RS00220	172	T	C	synonymous
46453	NTHI477_RS00220	189	C	T	synonymous
46460	NTHI477_RS00220	196	C	T	nonsynonymous
46462	NTHI477_RS00220	198	A	G	synonymous
46469	NTHI477_RS00220	205	C	A	synonymous
46471	NTHI477_RS00220	207	A	G	synonymous

46474	NTHI477_RS00220	210	A	C	synonymous
46480	NTHI477_RS00220	216	C	T	synonymous
46489	NTHI477_RS00220	225	G	A	synonymous
46495	NTHI477_RS00220	231	A	G	synonymous
46498	NTHI477_RS00220	234	T	G	synonymous
46511	NTHI477_RS00220	247	G	A	nonsynonymous
46512	NTHI477_RS00220	248	C	A	nonsynonymous
46525	NTHI477_RS00220	261	T	G	synonymous
46559	NTHI477_RS00220	295	G	T	nonsynonymous
46561	NTHI477_RS00220	297	T	G	nonsynonymous
46579	NTHI477_RS00220	315	A	G	synonymous
46581	NTHI477_RS00220	317	C	A	nonsynonymous
46592	NTHI477_RS00220	328	C	A	nonsynonymous
46595	NTHI477_RS00220	331	C	T	nonsynonymous
46597	NTHI477_RS00220	333	C	T	synonymous
46603	NTHI477_RS00220	339	A	G	synonymous
46605	NTHI477_RS00220	341	C	G	nonsynonymous
46640	NTHI477_RS00220	376	A	G	nonsynonymous
46646	NTHI477_RS00220	382	G	A	nonsynonymous
46648	NTHI477_RS00220	384	A	C	synonymous
46676	NTHI477_RS00220	412	C	T	nonsynonymous
46680	NTHI477_RS00220	416	C	T	nonsynonymous
46681	NTHI477_RS00220	417	A	G	synonymous
46681	NTHI477_RS00220	417	A	T	synonymous
46684	NTHI477_RS00220	420	G	T	synonymous
46699	NTHI477_RS00220	435	T	C	synonymous
46702	NTHI477_RS00220	438	G	A	synonymous
46723	NTHI477_RS00220	459	G	A	synonymous
46726	NTHI477_RS00220	462	T	C	synonymous
46732	NTHI477_RS00220	468	T	C	synonymous
46743	NTHI477_RS00220	479	C	T	nonsynonymous
46807	NTHI477_RS00220	543	G	A	synonymous
46813	NTHI477_RS00220	549	C	A	synonymous
46822	NTHI477_RS00220	558	C	T	synonymous
46838	NTHI477_RS00220	574	A	G	nonsynonymous
46849	NTHI477_RS00220	585	G	A	synonymous
46852	NTHI477_RS00220	588	A	T	synonymous
46857	NTHI477_RS00220	593	T	C	nonsynonymous
46864	NTHI477_RS00220	600	T	C	synonymous
46870	NTHI477_RS00220	606	G	T	synonymous
46870	NTHI477_RS00220	606	G	A	synonymous
46871	NTHI477_RS00220	607	A	C	synonymous
46873	NTHI477_RS00220	609	A	C	nonsynonymous
46876	NTHI477_RS00220	612	G	C	synonymous
46876	NTHI477_RS00220	612	G	A	synonymous

46885	NTHI477_RS00220	621	T	C	synonymous
46891	NTHI477_RS00220	627	G	A	synonymous
46893	NTHI477_RS00220	629	G	A	nonsynonymous
46894	NTHI477_RS00220	630	T	C	synonymous
46900	NTHI477_RS00220	636	T	C	synonymous
46906	NTHI477_RS00220	642	C	T	synonymous
46918	NTHI477_RS00220	654	T	C	synonymous
46945	NTHI477_RS00220	681	G	A	synonymous
46951	NTHI477_RS00220	687	C	T	synonymous
46957	NTHI477_RS00220	693	G	A	synonymous
46960	NTHI477_RS00220	696	A	T	synonymous
46966	NTHI477_RS00220	702	G	A	synonymous
46969	NTHI477_RS00220	705	T	A	synonymous
46976	NTHI477_RS00220	712	T	G	nonsynonymous
47011	NTHI477_RS00220	747	T	C	synonymous
47023	NTHI477_RS00220	759	A	G	synonymous
47035	NTHI477_RS00220	771	G	A	synonymous
47041	NTHI477_RS00220	777	A	C	nonsynonymous
47074	intergenic	intergenic	A	C	intergenic
47075	intergenic	intergenic	C	T	intergenic
47085	intergenic	intergenic	A	G	intergenic
47091	intergenic	intergenic	T	G	intergenic
47112	intergenic	intergenic	T	G	intergenic
47115	intergenic	intergenic	A	G	intergenic
47150	intergenic	intergenic	T	C	intergenic
47171	intergenic	intergenic	C	A	intergenic
47181	NTHI477_RS00225	765	T	C	synonymous
47190	NTHI477_RS00225	756	C	T	synonymous
47196	NTHI477_RS00225	750	T	A	synonymous
47217	NTHI477_RS00225	729	A	G	synonymous
47235	NTHI477_RS00225	711	C	T	synonymous
47253	NTHI477_RS00225	693	T	C	synonymous
47254	NTHI477_RS00225	692	G	A	nonsynonymous
47259	NTHI477_RS00225	687	G	C	nonsynonymous
47265	NTHI477_RS00225	681	A	T	synonymous
47283	NTHI477_RS00225	663	A	G	synonymous
47285	NTHI477_RS00225	661	C	T	nonsynonymous
47289	NTHI477_RS00225	657	G	T	nonsynonymous
47313	NTHI477_RS00225	633	C	T	synonymous
47322	NTHI477_RS00225	624	G	T	synonymous
47340	NTHI477_RS00225	606	G	A	synonymous
47342	NTHI477_RS00225	604	G	A	nonsynonymous
47349	NTHI477_RS00225	597	A	G	synonymous
47362	NTHI477_RS00225	584	G	A	nonsynonymous
47364	NTHI477_RS00225	582	T	C	synonymous

47379	NTHI477_RS00225	567	G	A	synonymous
47385	NTHI477_RS00225	561	T	C	synonymous
47394	NTHI477_RS00225	552	A	T	synonymous
47397	NTHI477_RS00225	549	C	T	synonymous
47424	NTHI477_RS00225	522	C	T	synonymous
47430	NTHI477_RS00225	516	G	C	synonymous
47442	NTHI477_RS00225	504	A	C	synonymous
47478	NTHI477_RS00225	468	C	T	synonymous
47499	NTHI477_RS00225	447	C	T	synonymous
47505	NTHI477_RS00225	441	C	T	synonymous
47508	NTHI477_RS00225	438	A	G	synonymous
47511	NTHI477_RS00225	435	A	G	synonymous
47517	NTHI477_RS00225	429	A	G	synonymous
47519	NTHI477_RS00225	427	A	C	nonsynonymous
47526	NTHI477_RS00225	420	A	G	synonymous
47529	NTHI477_RS00225	417	G	T	synonymous
47531	NTHI477_RS00225	415	G	T	nonsynonymous
47532	NTHI477_RS00225	414	T	C	synonymous
47539	NTHI477_RS00225	407	G	A	nonsynonymous
47565	NTHI477_RS00225	381	T	C	synonymous
47592	NTHI477_RS00225	354	G	A	synonymous
47616	NTHI477_RS00225	330	C	T	synonymous
47619	NTHI477_RS00225	327	C	T	synonymous
47637	NTHI477_RS00225	309	G	A	synonymous
47646	NTHI477_RS00225	300	A	C	synonymous
47646	NTHI477_RS00225	300	A	G	synonymous
47649	NTHI477_RS00225	297	G	A	synonymous
47685	NTHI477_RS00225	261	G	T	synonymous
47697	NTHI477_RS00225	249	G	T	synonymous
47706	NTHI477_RS00225	240	G	A	synonymous
47712	NTHI477_RS00225	234	C	T	synonymous
47713	NTHI477_RS00225	233	A	G	nonsynonymous
47715	NTHI477_RS00225	231	C	T	synonymous
47718	NTHI477_RS00225	228	G	A	synonymous
47721	NTHI477_RS00225	225	T	C	synonymous
47727	NTHI477_RS00225	219	A	G	synonymous
47736	NTHI477_RS00225	210	A	G	synonymous
47820	NTHI477_RS00225	126	T	G	synonymous
47832	NTHI477_RS00225	114	T	A	synonymous
47844	NTHI477_RS00225	102	C	T	synonymous
47850	NTHI477_RS00225	96	G	A	synonymous
47877	NTHI477_RS00225	69	A	C	synonymous
47902	NTHI477_RS00225	44	G	A	nonsynonymous
47919	NTHI477_RS00225	27	C	T	synonymous
47919	NTHI477_RS00225	27	C	G	synonymous

47954	intergenic	intergenic	C	T	intergenic
47956	intergenic	intergenic	C	T	intergenic
47973	intergenic	intergenic	G	A	intergenic
48029	intergenic	intergenic	T	G	intergenic
48049	intergenic	intergenic	C	T	intergenic
48064	intergenic	intergenic	T	C	intergenic
48069	intergenic	intergenic	A	G	intergenic
48070	intergenic	intergenic	T	G	intergenic
48078	intergenic	intergenic	T	G	intergenic
48082	intergenic	intergenic	C	T	intergenic
48097	NTHI477_RS00230	15	T	G	synonymous
48124	NTHI477_RS00230	42	G	A	synonymous
48133	NTHI477_RS00230	51	C	A	synonymous
48151	NTHI477_RS00230	69	C	T	synonymous
48175	NTHI477_RS00230	93	C	G	synonymous
48175	NTHI477_RS00230	93	C	T	synonymous
48187	NTHI477_RS00230	105	A	T	synonymous
48205	NTHI477_RS00230	123	T	A	synonymous
48205	NTHI477_RS00230	123	T	C	synonymous
48244	NTHI477_RS00230	162	A	G	synonymous
48247	NTHI477_RS00230	165	G	A	synonymous
48259	NTHI477_RS00230	177	T	G	synonymous
48284	NTHI477_RS00230	202	G	A	nonsynonymous
48295	NTHI477_RS00230	213	C	T	synonymous
48298	NTHI477_RS00230	216	G	A	synonymous
48298	NTHI477_RS00230	216	G	T	synonymous
48304	NTHI477_RS00230	222	G	A	synonymous
48316	NTHI477_RS00230	234	A	C	synonymous
48319	NTHI477_RS00230	237	T	G	synonymous
48325	NTHI477_RS00230	243	C	T	synonymous
48358	NTHI477_RS00230	276	T	C	synonymous
48364	NTHI477_RS00230	282	C	T	synonymous
48367	NTHI477_RS00230	285	A	G	synonymous
48370	NTHI477_RS00230	288	A	G	synonymous
48376	NTHI477_RS00230	294	A	T	synonymous
48376	NTHI477_RS00230	294	A	C	synonymous
48392	NTHI477_RS00230	310	G	A	nonsynonymous
48394	NTHI477_RS00230	312	C	T	synonymous
48406	NTHI477_RS00230	324	C	A	synonymous
48420	NTHI477_RS00230	338	C	T	nonsynonymous
48427	NTHI477_RS00230	345	T	A	nonsynonymous
48450	NTHI477_RS00230	368	C	T	nonsynonymous
48451	NTHI477_RS00230	369	A	T	synonymous
48457	NTHI477_RS00230	375	T	C	synonymous
48466	NTHI477_RS00230	384	C	T	synonymous

48469	NTHI477_RS00230	387	T	C	synonymous
48473	NTHI477_RS00230	391	T	C	synonymous
48484	NTHI477_RS00230	402	C	T	synonymous
48508	NTHI477_RS00230	426	A	G	synonymous
48511	NTHI477_RS00230	429	C	T	synonymous
48513	NTHI477_RS00230	431	T	G	nonsynonymous
48514	NTHI477_RS00230	432	A	G	synonymous
48517	NTHI477_RS00230	435	T	A	synonymous
48520	NTHI477_RS00230	438	T	G	synonymous
48523	NTHI477_RS00230	441	T	G	synonymous
48529	NTHI477_RS00230	447	C	T	synonymous
48534	NTHI477_RS00230	452	C	A	nonsynonymous
48547	NTHI477_RS00230	465	C	T	synonymous
48549	NTHI477_RS00230	467	A	C	nonsynonymous
48568	NTHI477_RS00230	486	A	G	synonymous
48571	NTHI477_RS00230	489	G	A	synonymous
48572	NTHI477_RS00230	490	C	A	nonsynonymous
48586	NTHI477_RS00230	504	G	A	synonymous
48586	NTHI477_RS00230	504	G	T	synonymous
48591	NTHI477_RS00230	509	C	G	nonsynonymous
48592	NTHI477_RS00230	510	T	A	synonymous
48607	NTHI477_RS00230	525	C	T	synonymous
48613	NTHI477_RS00230	531	A	G	synonymous
48658	NTHI477_RS00230	576	C	T	synonymous
48664	NTHI477_RS00230	582	T	C	synonymous
48665	NTHI477_RS00230	583	G	T	nonsynonymous
48671	NTHI477_RS00230	589	G	A	nonsynonymous
48673	NTHI477_RS00230	591	T	G	synonymous
48689	NTHI477_RS00230	607	A	G	nonsynonymous
48691	NTHI477_RS00230	609	G	T	synonymous
48697	NTHI477_RS00230	615	A	G	synonymous
48700	NTHI477_RS00230	618	G	T	synonymous
48703	NTHI477_RS00230	621	C	T	synonymous
48706	NTHI477_RS00230	624	T	A	synonymous
48709	NTHI477_RS00230	627	A	T	synonymous
48709	NTHI477_RS00230	627	A	G	synonymous
48715	NTHI477_RS00230	633	C	T	synonymous
48724	NTHI477_RS00230	642	A	G	synonymous
48724	NTHI477_RS00230	642	A	C	synonymous
48728	NTHI477_RS00230	646	G	A	nonsynonymous
48736	NTHI477_RS00230	654	C	T	synonymous
48757	NTHI477_RS00230	675	T	C	synonymous
48775	NTHI477_RS00230	693	C	T	synonymous
48805	NTHI477_RS00230	723	T	A	synonymous
48820	NTHI477_RS00230	738	T	G	synonymous

48823	NTHI477_RS00230	741	A	G	synonymous
48826	NTHI477_RS00230	744	G	A	synonymous
48837	NTHI477_RS00230	755	C	A	nonsynonymous
48851	intergenic	intergenic	A	G	intergenic
48853	intergenic	intergenic	A	C	intergenic
48853	intergenic	intergenic	A	T	intergenic
48856	intergenic	intergenic	C	T	intergenic
48861	intergenic	intergenic	T	C	intergenic
48863	intergenic	intergenic	G	T	intergenic
48865	intergenic	intergenic	C	T	intergenic
48868	intergenic	intergenic	G	T	intergenic
48875	intergenic	intergenic	G	A	intergenic
48900	intergenic	intergenic	T	G	intergenic
48900	intergenic	intergenic	T	C	intergenic
48901	intergenic	intergenic	A	C	intergenic
48901	intergenic	intergenic	A	G	intergenic
48903	intergenic	intergenic	A	G	intergenic
48904	intergenic	intergenic	G	T	intergenic
48905	intergenic	intergenic	C	T	intergenic
48908	intergenic	intergenic	C	T	intergenic
48909	intergenic	intergenic	T	A	intergenic
48912	intergenic	intergenic	C	T	intergenic
48917	intergenic	intergenic	C	T	intergenic
48918	intergenic	intergenic	A	G	intergenic
48919	intergenic	intergenic	T	A	intergenic
48920	intergenic	intergenic	C	A	intergenic
48931	intergenic	intergenic	A	T	intergenic
48939	intergenic	intergenic	A	C	intergenic
48942	intergenic	intergenic	C	T	intergenic
48943	intergenic	intergenic	A	C	intergenic
48944	intergenic	intergenic	A	T	intergenic
48947	intergenic	intergenic	G	T	intergenic
48947	intergenic	intergenic	G	C	intergenic
48955	intergenic	intergenic	G	C	intergenic
48967	intergenic	intergenic	A	G	intergenic
48968	intergenic	intergenic	G	T	intergenic
48985	NTHI477_RS00235	4	C	A	nonsynonymous
48994	NTHI477_RS00235	13	T	A	nonsynonymous
49008	NTHI477_RS00235	27	C	T	synonymous
49012	NTHI477_RS00235	31	C	T	synonymous
49014	NTHI477_RS00235	33	G	A	synonymous
49017	NTHI477_RS00235	36	G	T	synonymous
49035	NTHI477_RS00235	54	A	G	synonymous
49047	NTHI477_RS00235	66	C	T	synonymous
49048	NTHI477_RS00235	67	C	T	synonymous

49056	NTHI477_RS00235	75	T	C	synonymous
49059	NTHI477_RS00235	78	A	T	synonymous
49062	NTHI477_RS00235	81	C	T	synonymous
49068	NTHI477_RS00235	87	T	C	synonymous
49083	NTHI477_RS00235	102	G	A	synonymous
49086	NTHI477_RS00235	105	G	T	synonymous
49101	NTHI477_RS00235	120	G	T	synonymous
49101	NTHI477_RS00235	120	G	C	synonymous
49104	NTHI477_RS00235	123	G	A	synonymous
49110	NTHI477_RS00235	129	C	A	synonymous
49110	NTHI477_RS00235	129	C	T	synonymous
49116	NTHI477_RS00235	135	G	A	synonymous
49119	NTHI477_RS00235	138	C	T	synonymous
49128	NTHI477_RS00235	147	C	T	synonymous
49134	NTHI477_RS00235	153	C	A	synonymous
49140	NTHI477_RS00235	159	T	C	synonymous
49140	NTHI477_RS00235	159	T	A	synonymous
49143	NTHI477_RS00235	162	G	T	synonymous
49155	NTHI477_RS00235	174	A	C	synonymous
49173	NTHI477_RS00235	192	G	A	synonymous
49182	NTHI477_RS00235	201	A	T	synonymous
49191	NTHI477_RS00235	210	C	T	synonymous
49198	NTHI477_RS00235	217	C	T	synonymous
49203	NTHI477_RS00235	222	A	G	synonymous
49209	NTHI477_RS00235	228	A	G	synonymous
49209	NTHI477_RS00235	228	A	C	synonymous
49218	NTHI477_RS00235	237	G	A	synonymous
49230	NTHI477_RS00235	249	C	T	synonymous
49236	NTHI477_RS00235	255	C	T	synonymous
49239	NTHI477_RS00235	258	G	A	synonymous
49272	NTHI477_RS00235	291	T	C	synonymous
49284	NTHI477_RS00235	303	A	G	synonymous
49287	NTHI477_RS00235	306	T	G	synonymous
49287	NTHI477_RS00235	306	T	C	synonymous
49290	NTHI477_RS00235	309	G	T	synonymous
49294	NTHI477_RS00235	313	C	T	nonsynonymous
49299	NTHI477_RS00235	318	C	G	synonymous
49302	NTHI477_RS00235	321	T	C	synonymous
49302	NTHI477_RS00235	321	T	G	synonymous
49305	NTHI477_RS00235	324	C	A	synonymous
49308	NTHI477_RS00235	327	G	A	synonymous
49311	NTHI477_RS00235	330	G	T	synonymous
49317	NTHI477_RS00235	336	T	C	synonymous
49327	NTHI477_RS00235	346	G	A	nonsynonymous
49332	NTHI477_RS00235	351	C	T	synonymous

49341	NTHI477_RS00235	360	A	G	synonymous
49380	NTHI477_RS00235	399	G	A	synonymous
49383	NTHI477_RS00235	402	G	A	synonymous
49390	NTHI477_RS00235	409	C	T	nonsynonymous
49392	NTHI477_RS00235	411	C	G	synonymous
49395	NTHI477_RS00235	414	T	C	synonymous
49407	NTHI477_RS00235	426	G	T	nonsynonymous
49426	NTHI477_RS00235	445	A	C	nonsynonymous
49434	NTHI477_RS00235	453	G	A	synonymous
49434	NTHI477_RS00235	453	G	C	synonymous
49441	NTHI477_RS00235	460	T	G	nonsynonymous
49443	NTHI477_RS00235	462	A	C	nonsynonymous
49446	NTHI477_RS00235	465	T	G	synonymous
49446	NTHI477_RS00235	465	T	A	synonymous
49449	NTHI477_RS00235	468	G	A	synonymous
49449	NTHI477_RS00235	468	G	T	synonymous
49455	NTHI477_RS00235	474	A	C	nonsynonymous
49458	NTHI477_RS00235	477	T	G	synonymous
49461	NTHI477_RS00235	480	A	G	synonymous
49462	NTHI477_RS00235	481	C	T	nonsynonymous
49463	NTHI477_RS00235	482	T	G	nonsynonymous
49464	NTHI477_RS00235	483	C	G	synonymous
49464	NTHI477_RS00235	483	C	T	synonymous
49467	NTHI477_RS00235	486	G	T	synonymous
49469	NTHI477_RS00235	488	T	G	nonsynonymous
49473	NTHI477_RS00235	492	T	G	synonymous
49479	NTHI477_RS00235	498	A	T	synonymous
49479	NTHI477_RS00235	498	A	G	synonymous
49491	NTHI477_RS00235	510	G	A	synonymous
49500	NTHI477_RS00235	519	C	T	synonymous
49506	NTHI477_RS00235	525	C	T	synonymous
49509	NTHI477_RS00235	528	T	A	synonymous
49512	NTHI477_RS00235	531	C	G	synonymous
49529	NTHI477_RS00235	548	C	T	nonsynonymous
49542	NTHI477_RS00235	561	A	G	synonymous
49545	NTHI477_RS00235	564	G	C	synonymous
49551	NTHI477_RS00235	570	C	A	synonymous
49551	NTHI477_RS00235	570	C	T	synonymous
49581	NTHI477_RS00235	600	C	A	synonymous
49583	NTHI477_RS00235	602	C	G	nonsynonymous
49584	NTHI477_RS00235	603	A	T	synonymous
49599	NTHI477_RS00235	618	T	C	synonymous
49605	NTHI477_RS00235	624	C	G	synonymous
49605	NTHI477_RS00235	624	C	T	synonymous
49608	NTHI477_RS00235	627	A	G	synonymous

49620	NTHI477_RS00235	639	T	C	synonymous
49623	NTHI477_RS00235	642	C	T	synonymous
49645	NTHI477_RS00235	664	T	C	synonymous
49648	NTHI477_RS00235	667	C	T	synonymous
49653	NTHI477_RS00235	672	T	A	synonymous
49656	NTHI477_RS00235	675	A	G	synonymous
49671	NTHI477_RS00235	690	A	G	synonymous
49695	NTHI477_RS00235	714	A	T	synonymous
49705	NTHI477_RS00235	724	A	G	nonsynonymous
49705	NTHI477_RS00240	9	A	G	synonymous
49730	NTHI477_RS00240	34	A	C	nonsynonymous
49744	NTHI477_RS00240	48	A	C	nonsynonymous
49759	NTHI477_RS00240	63	G	A	synonymous
49771	NTHI477_RS00240	75	C	T	synonymous
49774	NTHI477_RS00240	78	A	G	synonymous
49777	NTHI477_RS00240	81	C	A	synonymous
49780	NTHI477_RS00240	84	C	T	synonymous
49798	NTHI477_RS00240	102	C	T	synonymous
49814	NTHI477_RS00240	118	C	T	synonymous
49825	NTHI477_RS00240	129	A	T	synonymous
49825	NTHI477_RS00240	129	A	C	synonymous
49828	NTHI477_RS00240	132	C	G	synonymous
49834	NTHI477_RS00240	138	A	C	synonymous
49849	NTHI477_RS00240	153	T	C	synonymous
49858	NTHI477_RS00240	162	C	T	synonymous
49883	NTHI477_RS00240	187	G	A	nonsynonymous
49896	NTHI477_RS00240	200	G	C	nonsynonymous
49897	NTHI477_RS00240	201	C	G	nonsynonymous
49905	NTHI477_RS00240	209	C	G	nonsynonymous
49906	NTHI477_RS00240	210	A	C	synonymous
49915	NTHI477_RS00240	219	C	T	synonymous
49939	NTHI477_RS00240	243	A	G	synonymous
49951	NTHI477_RS00240	255	G	A	synonymous
49954	NTHI477_RS00240	258	T	C	synonymous
49971	NTHI477_RS00240	275	C	G	nonsynonymous
49972	NTHI477_RS00240	276	T	A	synonymous
49981	NTHI477_RS00240	285	T	C	synonymous
49990	NTHI477_RS00240	294	C	A	synonymous
49990	NTHI477_RS00240	294	C	T	synonymous
49996	NTHI477_RS00240	300	C	T	synonymous
50003	NTHI477_RS00240	307	C	A	nonsynonymous
50026	NTHI477_RS00240	330	C	T	synonymous
50035	NTHI477_RS00240	339	C	G	synonymous
50047	NTHI477_RS00240	351	T	C	synonymous
50056	NTHI477_RS00240	360	T	C	synonymous

50077	NTHI477_RS00240	381	T	C	synonymous
50089	NTHI477_RS00240	393	C	T	synonymous
50092	NTHI477_RS00240	396	A	T	synonymous
50097	NTHI477_RS00240	401	A	T	nonsynonymous
50098	NTHI477_RS00240	402	A	G	synonymous
50101	NTHI477_RS00240	405	A	G	synonymous
50107	NTHI477_RS00240	411	G	A	synonymous
50113	NTHI477_RS00240	417	A	C	synonymous
50113	NTHI477_RS00240	417	A	T	synonymous
50116	NTHI477_RS00240	420	G	T	synonymous
50119	NTHI477_RS00240	423	T	A	synonymous
50119	NTHI477_RS00240	423	T	C	synonymous
50122	NTHI477_RS00240	426	G	A	synonymous
50123	NTHI477_RS00240	427	C	T	synonymous
50126	NTHI477_RS00240	430	C	T	synonymous
50129	NTHI477_RS00240	433	A	G	nonsynonymous
50134	NTHI477_RS00240	438	T	G	nonsynonymous
50137	NTHI477_RS00240	441	T	C	synonymous
50140	NTHI477_RS00240	444	T	C	synonymous
50177	NTHI477_RS00240	481	G	T	nonsynonymous
50191	NTHI477_RS00240	495	C	T	synonymous
50213	NTHI477_RS00240	517	C	T	nonsynonymous
50254	NTHI477_RS00240	558	A	C	synonymous
50261	NTHI477_RS00240	565	T	G	nonsynonymous
50290	NTHI477_RS00240	594	G	C	synonymous
50293	NTHI477_RS00240	597	C	T	synonymous
50296	NTHI477_RS00240	600	A	G	synonymous
50297	NTHI477_RS00240	601	T	C	synonymous
50317	NTHI477_RS00240	621	G	A	synonymous
50318	NTHI477_RS00240	622	C	A	nonsynonymous
50353	NTHI477_RS00240	657	C	T	synonymous
50382	NTHI477_RS00240	686	A	G	nonsynonymous
50392	NTHI477_RS00240	696	C	T	synonymous
50404	NTHI477_RS00240	708	C	A	synonymous
50410	NTHI477_RS00240	714	T	C	synonymous
50414	NTHI477_RS00240	718	C	T	nonsynonymous
50428	NTHI477_RS00240	732	A	T	synonymous
50452	NTHI477_RS00240	756	G	C	synonymous
50453	NTHI477_RS00240	757	C	T	synonymous
50458	NTHI477_RS00240	762	C	T	synonymous
50466	NTHI477_RS00240	770	C	T	nonsynonymous
50467	NTHI477_RS00240	771	A	G	synonymous
50485	NTHI477_RS00240	789	A	G	synonymous
50486	NTHI477_RS00240	790	C	T	nonsynonymous
50488	NTHI477_RS00240	792	C	T	synonymous

50489	NTHI477_RS00240	793	G	T	nonsynonymous
50491	NTHI477_RS00240	795	G	A	synonymous
50509	NTHI477_RS00240	813	G	A	synonymous
50511	NTHI477_RS00240	815	T	G	nonsynonymous
50544	NTHI477_RS00240	848	T	C	nonsynonymous
50545	NTHI477_RS00240	849	A	G	nonsynonymous
50548	NTHI477_RS00240	852	G	A	synonymous
50561	NTHI477_RS00240	865	C	A	nonsynonymous
50569	NTHI477_RS00240	873	C	T	synonymous
50572	NTHI477_RS00240	876	T	C	synonymous
50593	NTHI477_RS00240	897	G	A	synonymous
50596	NTHI477_RS00240	900	T	C	synonymous
50597	NTHI477_RS00240	901	A	G	nonsynonymous
50626	NTHI477_RS00240	930	G	A	synonymous
50635	NTHI477_RS00240	939	C	T	synonymous
50641	NTHI477_RS00240	945	A	C	nonsynonymous
50651	NTHI477_RS00240	955	A	C	nonsynonymous
50675	NTHI477_RS00240	979	A	C	nonsynonymous
50683	NTHI477_RS00240	987	G	A	synonymous
50695	NTHI477_RS00240	999	A	G	synonymous
50702	NTHI477_RS00240	1006	G	A	nonsynonymous
50702	NTHI477_RS00240	1006	G	T	nonsynonymous
50705	NTHI477_RS00240	1009	G	A	nonsynonymous
50713	NTHI477_RS00240	1017	A	G	synonymous
50719	NTHI477_RS00240	1023	C	T	synonymous
50771	intergenic	intergenic	A	C	intergenic
50772	intergenic	intergenic	A	T	intergenic
50785	NTHI477_RS00245	1365	A	G	synonymous
50791	NTHI477_RS00245	1359	A	G	synonymous
50797	NTHI477_RS00245	1353	A	G	synonymous
50800	NTHI477_RS00245	1350	C	A	synonymous
50814	NTHI477_RS00245	1336	C	A	nonsynonymous
50818	NTHI477_RS00245	1332	G	T	synonymous
50830	NTHI477_RS00245	1320	T	C	synonymous
50833	NTHI477_RS00245	1317	A	G	synonymous
50889	NTHI477_RS00245	1261	T	G	nonsynonymous
50890	NTHI477_RS00245	1260	A	G	synonymous
50905	NTHI477_RS00245	1245	T	C	synonymous
50908	NTHI477_RS00245	1242	A	G	synonymous
50920	NTHI477_RS00245	1230	G	A	synonymous
50923	NTHI477_RS00245	1227	T	A	synonymous
50935	NTHI477_RS00245	1215	T	A	synonymous
50966	NTHI477_RS00245	1184	G	A	nonsynonymous
50968	NTHI477_RS00245	1182	G	A	synonymous
50974	NTHI477_RS00245	1176	G	A	synonymous

51007	NTHI477_RS00245	1143	T	G	synonymous
51010	NTHI477_RS00245	1140	G	A	synonymous
51028	NTHI477_RS00245	1122	G	C	synonymous
51040	NTHI477_RS00245	1110	A	T	synonymous
51043	NTHI477_RS00245	1107	G	A	synonymous
51049	NTHI477_RS00245	1101	G	A	synonymous
51061	NTHI477_RS00245	1089	A	G	synonymous
51062	NTHI477_RS00245	1088	A	C	nonsynonymous
51064	NTHI477_RS00245	1086	G	C	synonymous
51067	NTHI477_RS00245	1083	A	T	synonymous
51069	NTHI477_RS00245	1081	C	T	nonsynonymous
51073	NTHI477_RS00245	1077	G	C	synonymous
51076	NTHI477_RS00245	1074	T	A	nonsynonymous
51079	NTHI477_RS00245	1071	G	A	synonymous
51081	NTHI477_RS00245	1069	C	T	nonsynonymous
51091	NTHI477_RS00245	1059	G	A	synonymous
51097	NTHI477_RS00245	1053	G	A	synonymous
51109	NTHI477_RS00245	1041	C	T	synonymous
51118	NTHI477_RS00245	1032	T	C	synonymous
51124	NTHI477_RS00245	1026	T	C	synonymous
51127	NTHI477_RS00245	1023	A	G	synonymous
51133	NTHI477_RS00245	1017	T	C	synonymous
51151	NTHI477_RS00245	999	G	A	synonymous
51157	NTHI477_RS00245	993	A	C	synonymous
51160	NTHI477_RS00245	990	C	T	synonymous
51163	NTHI477_RS00245	987	C	T	synonymous
51166	NTHI477_RS00245	984	A	G	synonymous
51190	NTHI477_RS00245	960	G	A	synonymous
51196	NTHI477_RS00245	954	C	T	synonymous
51202	NTHI477_RS00245	948	G	A	synonymous
51208	NTHI477_RS00245	942	G	A	synonymous
51235	NTHI477_RS00245	915	T	A	synonymous
51250	NTHI477_RS00245	900	C	T	synonymous
51256	NTHI477_RS00245	894	A	T	synonymous
51262	NTHI477_RS00245	888	C	T	synonymous
51267	NTHI477_RS00245	883	C	T	nonsynonymous
51280	NTHI477_RS00245	870	C	T	synonymous
51286	NTHI477_RS00245	864	A	T	synonymous
51297	NTHI477_RS00245	853	A	C	nonsynonymous
51298	NTHI477_RS00245	852	G	T	synonymous
51316	NTHI477_RS00245	834	A	C	synonymous
51325	NTHI477_RS00245	825	G	A	synonymous
51328	NTHI477_RS00245	822	A	G	synonymous
51337	NTHI477_RS00245	813	G	A	synonymous
51343	NTHI477_RS00245	807	G	A	synonymous

51352	NTHI477_RS00245	798	A	G	synonymous
51355	NTHI477_RS00245	795	C	A	synonymous
51355	NTHI477_RS00245	795	C	T	synonymous
51358	NTHI477_RS00245	792	T	G	synonymous
51361	NTHI477_RS00245	789	A	G	synonymous
51364	NTHI477_RS00245	786	A	G	synonymous
51388	NTHI477_RS00245	762	T	G	nonsynonymous
51390	NTHI477_RS00245	760	A	G	synonymous
51415	NTHI477_RS00245	735	A	C	synonymous
51415	NTHI477_RS00245	735	A	G	synonymous
51427	NTHI477_RS00245	723	A	G	synonymous
51442	NTHI477_RS00245	708	G	T	synonymous
51448	NTHI477_RS00245	702	C	A	synonymous
51448	NTHI477_RS00245	702	C	T	synonymous
51457	NTHI477_RS00245	693	A	G	synonymous
51470	NTHI477_RS00245	680	T	A	nonsynonymous
51475	NTHI477_RS00245	675	G	C	synonymous
51493	NTHI477_RS00245	657	A	T	synonymous
51502	NTHI477_RS00245	648	A	G	synonymous
51517	NTHI477_RS00245	633	C	T	synonymous
51526	NTHI477_RS00245	624	G	A	synonymous
51529	NTHI477_RS00245	621	C	T	synonymous
51538	NTHI477_RS00245	612	T	C	synonymous
51544	NTHI477_RS00245	606	G	A	synonymous
51550	NTHI477_RS00245	600	A	T	synonymous
51577	NTHI477_RS00245	573	C	T	synonymous
51579	NTHI477_RS00245	571	G	A	synonymous
51598	NTHI477_RS00245	552	T	C	synonymous
51602	NTHI477_RS00245	548	C	T	nonsynonymous
51610	NTHI477_RS00245	540	T	C	synonymous
51622	NTHI477_RS00245	528	A	C	synonymous
51625	NTHI477_RS00245	525	T	C	synonymous
51651	NTHI477_RS00245	499	T	C	nonsynonymous
51663	NTHI477_RS00245	487	G	A	nonsynonymous
51664	NTHI477_RS00245	486	G	T	synonymous
51664	NTHI477_RS00245	486	G	A	synonymous
51673	NTHI477_RS00245	477	T	C	nonsynonymous
51691	NTHI477_RS00245	459	A	G	synonymous
51700	NTHI477_RS00245	450	A	G	synonymous
51709	NTHI477_RS00245	441	G	A	synonymous
51724	NTHI477_RS00245	426	A	G	synonymous
51743	NTHI477_RS00245	407	G	C	nonsynonymous
51744	NTHI477_RS00245	406	C	T	nonsynonymous
51748	NTHI477_RS00245	402	G	A	synonymous
51751	NTHI477_RS00245	399	A	G	synonymous

51757	NTHI477_RS00245	393	G	T	synonymous
51763	NTHI477_RS00245	387	G	A	synonymous
51764	NTHI477_RS00245	386	C	T	nonsynonymous
51766	NTHI477_RS00245	384	G	A	synonymous
51773	NTHI477_RS00245	377	C	T	nonsynonymous
51775	NTHI477_RS00245	375	A	G	synonymous
51778	NTHI477_RS00245	372	G	A	synonymous
51796	NTHI477_RS00245	354	A	G	synonymous
51835	NTHI477_RS00245	315	G	A	synonymous
51858	NTHI477_RS00245	292	A	G	synonymous
51895	NTHI477_RS00245	255	G	T	synonymous
51898	NTHI477_RS00245	252	A	G	synonymous
51913	NTHI477_RS00245	237	T	C	synonymous
51925	NTHI477_RS00245	225	A	G	synonymous
51928	NTHI477_RS00245	222	T	G	synonymous
51937	NTHI477_RS00245	213	G	A	synonymous
52000	NTHI477_RS00245	150	G	A	synonymous
52015	NTHI477_RS00245	135	G	A	synonymous
52018	NTHI477_RS00245	132	T	A	synonymous
52018	NTHI477_RS00245	132	T	G	synonymous
52027	NTHI477_RS00245	123	G	A	synonymous
52033	NTHI477_RS00245	117	G	C	synonymous
52033	NTHI477_RS00245	117	G	A	synonymous
52036	NTHI477_RS00245	114	C	A	synonymous
52042	NTHI477_RS00245	108	A	G	synonymous
52048	NTHI477_RS00245	102	G	A	synonymous
52051	NTHI477_RS00245	99	G	A	synonymous
52069	NTHI477_RS00245	81	A	G	synonymous
52072	NTHI477_RS00245	78	G	A	synonymous
52075	NTHI477_RS00245	75	C	A	synonymous
52087	NTHI477_RS00245	63	G	A	synonymous
52090	NTHI477_RS00245	60	C	A	synonymous
52096	NTHI477_RS00245	54	T	A	synonymous
52096	NTHI477_RS00245	54	T	G	synonymous
52108	NTHI477_RS00245	42	A	G	synonymous
52108	NTHI477_RS00245	42	A	T	synonymous
52114	NTHI477_RS00245	36	C	T	synonymous
52138	NTHI477_RS00245	12	G	A	synonymous
52164	intergenic	intergenic	T	C	intergenic
52188	intergenic	intergenic	T	C	intergenic
52202	intergenic	intergenic	G	A	intergenic
52203	intergenic	intergenic	C	A	intergenic
52247	NTHI477_RS00250	597	G	A	synonymous
52262	NTHI477_RS00250	582	G	A	synonymous
52265	NTHI477_RS00250	579	A	G	synonymous

52268	NTHI477_RS00250	576	T	A	synonymous
52271	NTHI477_RS00250	573	T	C	synonymous
52274	NTHI477_RS00250	570	A	G	synonymous
52277	NTHI477_RS00250	567	C	T	synonymous
52280	NTHI477_RS00250	564	G	A	synonymous
52283	NTHI477_RS00250	561	A	G	synonymous
52289	NTHI477_RS00250	555	G	A	synonymous
52292	NTHI477_RS00250	552	G	T	synonymous
52295	NTHI477_RS00250	549	T	A	synonymous
52307	NTHI477_RS00250	537	T	C	synonymous
52309	NTHI477_RS00250	535	G	A	synonymous
52316	NTHI477_RS00250	528	A	G	synonymous
52325	NTHI477_RS00250	519	C	T	synonymous
52339	NTHI477_RS00250	505	T	C	nonsynonymous
52343	NTHI477_RS00250	501	T	C	synonymous
52358	NTHI477_RS00250	486	C	T	synonymous
52376	NTHI477_RS00250	468	C	T	synonymous
52388	NTHI477_RS00250	456	T	C	synonymous
52390	NTHI477_RS00250	454	C	G	nonsynonymous
52403	NTHI477_RS00250	441	A	G	synonymous
52403	NTHI477_RS00250	441	A	T	synonymous
52412	NTHI477_RS00250	432	A	T	synonymous
52433	NTHI477_RS00250	411	C	T	synonymous
52436	NTHI477_RS00250	408	G	C	synonymous
52442	NTHI477_RS00250	402	G	A	synonymous
52448	NTHI477_RS00250	396	G	A	synonymous
52451	NTHI477_RS00250	393	A	G	synonymous
52451	NTHI477_RS00250	393	A	T	synonymous
52460	NTHI477_RS00250	384	T	C	synonymous
52463	NTHI477_RS00250	381	A	G	synonymous
52468	NTHI477_RS00250	376	G	A	synonymous
52469	NTHI477_RS00250	375	T	C	synonymous
52469	NTHI477_RS00250	375	T	A	synonymous
52472	NTHI477_RS00250	372	A	C	synonymous
52475	NTHI477_RS00250	369	A	G	synonymous
52478	NTHI477_RS00250	366	G	A	synonymous
52489	NTHI477_RS00250	355	G	T	synonymous
52490	NTHI477_RS00250	354	C	T	synonymous
52490	NTHI477_RS00250	354	C	A	synonymous
52493	NTHI477_RS00250	351	A	G	synonymous
52493	NTHI477_RS00250	351	A	C	synonymous
52499	NTHI477_RS00250	345	T	A	synonymous
52520	NTHI477_RS00250	324	A	G	synonymous
52532	NTHI477_RS00250	312	G	C	nonsynonymous
52534	NTHI477_RS00250	310	T	C	nonsynonymous

52538	NTHI477_RS00250	306	G	A	synonymous
52553	NTHI477_RS00250	291	G	A	synonymous
52562	NTHI477_RS00250	282	A	T	synonymous
52568	NTHI477_RS00250	276	G	A	synonymous
52592	NTHI477_RS00250	252	A	G	synonymous
52604	NTHI477_RS00250	240	C	A	synonymous
52634	NTHI477_RS00250	210	G	A	synonymous
52646	NTHI477_RS00250	198	A	G	synonymous
52661	NTHI477_RS00250	183	A	T	synonymous
52673	NTHI477_RS00250	171	C	A	synonymous
52676	NTHI477_RS00250	168	G	A	synonymous
52688	NTHI477_RS00250	156	C	T	synonymous
52694	NTHI477_RS00250	150	T	C	synonymous
52715	NTHI477_RS00250	129	C	G	synonymous
52724	NTHI477_RS00250	120	T	C	synonymous
52727	NTHI477_RS00250	117	C	T	synonymous
52763	NTHI477_RS00250	81	G	A	synonymous
52764	NTHI477_RS00250	80	T	C	nonsynonymous
52769	NTHI477_RS00250	75	C	T	synonymous
52778	NTHI477_RS00250	66	T	A	synonymous
52784	NTHI477_RS00250	60	T	C	synonymous
52787	NTHI477_RS00250	57	G	A	synonymous
52793	NTHI477_RS00250	51	A	G	synonymous
52794	NTHI477_RS00250	50	T	G	nonsynonymous
52795	NTHI477_RS00250	49	T	G	nonsynonymous
52802	NTHI477_RS00250	42	C	T	synonymous
52860	intergenic	intergenic	T	C	intergenic
52867	intergenic	intergenic	T	A	intergenic
52867	intergenic	intergenic	T	G	intergenic
52876	intergenic	intergenic	A	G	intergenic
52883	intergenic	intergenic	T	C	intergenic
52894	intergenic	intergenic	G	A	intergenic
52895	intergenic	intergenic	T	C	intergenic
52898	intergenic	intergenic	C	T	intergenic
52914	intergenic	intergenic	A	G	intergenic
52918	intergenic	intergenic	C	T	intergenic
52926	intergenic	intergenic	C	T	intergenic
52934	intergenic	intergenic	G	A	intergenic
52941	intergenic	intergenic	G	A	intergenic
52963	intergenic	intergenic	A	G	intergenic
52968	intergenic	intergenic	T	C	intergenic
52986	intergenic	intergenic	A	C	intergenic
52988	intergenic	intergenic	C	T	intergenic
52989	intergenic	intergenic	G	C	intergenic
52993	intergenic	intergenic	T	C	intergenic

52995	intergenic	intergenic	T	C	intergenic
52999	intergenic	intergenic	T	C	intergenic
53008	intergenic	intergenic	C	A	intergenic
53020	intergenic	intergenic	G	A	intergenic
53022	intergenic	intergenic	C	T	intergenic
53034	NTHI477_RS00255	697	C	T	nonsynonymous
53176	NTHI477_RS00255	555	G	T	synonymous
53191	NTHI477_RS00255	540	C	A	synonymous
53200	NTHI477_RS00255	531	T	C	synonymous
53215	NTHI477_RS00255	516	T	C	synonymous
53220	NTHI477_RS00255	511	T	C	nonsynonymous
53221	NTHI477_RS00255	510	G	A	synonymous
53227	NTHI477_RS00255	504	T	A	synonymous
53229	NTHI477_RS00255	502	T	C	nonsynonymous
53230	NTHI477_RS00255	501	G	T	synonymous
53235	NTHI477_RS00255	496	G	A	synonymous
53236	NTHI477_RS00255	495	A	G	synonymous
53242	NTHI477_RS00255	489	T	C	synonymous
53254	NTHI477_RS00255	477	T	C	synonymous
53266	NTHI477_RS00255	465	C	T	synonymous
53278	NTHI477_RS00255	453	G	C	synonymous
53317	NTHI477_RS00255	414	G	A	synonymous
53332	NTHI477_RS00255	399	A	G	synonymous
53338	NTHI477_RS00255	393	G	A	synonymous
53362	NTHI477_RS00255	369	C	T	synonymous
53367	NTHI477_RS00255	364	G	A	synonymous
53416	NTHI477_RS00255	315	G	T	synonymous
53416	NTHI477_RS00255	315	G	A	synonymous
53425	NTHI477_RS00255	306	T	G	synonymous
53428	NTHI477_RS00255	303	C	T	synonymous
53434	NTHI477_RS00255	297	A	G	synonymous
53455	NTHI477_RS00255	276	A	G	synonymous
53467	NTHI477_RS00255	264	A	G	synonymous
53494	NTHI477_RS00255	237	A	G	synonymous
53496	NTHI477_RS00255	235	T	C	nonsynonymous
53512	NTHI477_RS00255	219	A	G	synonymous
53515	NTHI477_RS00255	216	C	T	synonymous
53527	NTHI477_RS00255	204	C	T	synonymous
53533	NTHI477_RS00255	198	G	A	synonymous
53535	NTHI477_RS00255	196	C	T	nonsynonymous
53542	NTHI477_RS00255	189	C	A	synonymous
53545	NTHI477_RS00255	186	A	T	synonymous
53545	NTHI477_RS00255	186	A	C	synonymous
53546	NTHI477_RS00255	185	G	T	nonsynonymous
53548	NTHI477_RS00255	183	A	G	synonymous

53557	NTHI477_RS00255	174	T	G	synonymous
53578	NTHI477_RS00255	153	C	T	synonymous
53587	NTHI477_RS00255	144	C	A	synonymous
53599	NTHI477_RS00255	132	A	G	synonymous
53604	NTHI477_RS00255	127	C	T	nonsynonymous
53605	NTHI477_RS00255	126	C	G	synonymous
53608	NTHI477_RS00255	123	T	A	synonymous
53611	NTHI477_RS00255	120	A	G	synonymous
53634	NTHI477_RS00255	97	T	C	nonsynonymous
53638	NTHI477_RS00255	93	A	G	synonymous
53641	NTHI477_RS00255	90	T	G	nonsynonymous
53675	NTHI477_RS00255	56	G	A	nonsynonymous
53688	NTHI477_RS00255	43	T	G	nonsynonymous
53696	NTHI477_RS00255	35	T	G	nonsynonymous
53754	NTHI477_RS00260	602	T	C	nonsynonymous
53762	NTHI477_RS00260	594	C	T	synonymous
53769	NTHI477_RS00260	587	T	C	nonsynonymous
53771	NTHI477_RS00260	585	A	G	synonymous
53772	NTHI477_RS00260	584	T	G	nonsynonymous
53783	NTHI477_RS00260	573	G	A	synonymous
53785	NTHI477_RS00260	571	T	C	nonsynonymous
53792	NTHI477_RS00260	564	T	G	synonymous
53795	NTHI477_RS00260	561	G	A	synonymous
53795	NTHI477_RS00260	561	G	T	synonymous
53796	NTHI477_RS00260	560	C	G	nonsynonymous
53797	NTHI477_RS00260	559	G	C	nonsynonymous
53812	NTHI477_RS00260	544	C	T	nonsynonymous
53819	NTHI477_RS00260	537	G	A	synonymous
53822	NTHI477_RS00260	534	A	G	synonymous
53825	NTHI477_RS00260	531	T	G	synonymous
53840	NTHI477_RS00260	516	A	C	synonymous
53873	NTHI477_RS00260	483	T	G	synonymous
53876	NTHI477_RS00260	480	C	T	synonymous
53878	NTHI477_RS00260	478	A	C	nonsynonymous
53886	NTHI477_RS00260	470	C	T	nonsynonymous
53906	NTHI477_RS00260	450	T	C	synonymous
53908	NTHI477_RS00260	448	A	G	nonsynonymous
53922	NTHI477_RS00260	434	C	T	nonsynonymous
53969	NTHI477_RS00260	387	T	C	synonymous
53987	NTHI477_RS00260	369	G	A	synonymous
53999	NTHI477_RS00260	357	G	T	synonymous
53999	NTHI477_RS00260	357	G	A	synonymous
54017	NTHI477_RS00260	339	C	A	synonymous
54029	NTHI477_RS00260	327	C	T	synonymous
54047	NTHI477_RS00260	309	A	G	synonymous

54053	NTHI477_RS00260	303	G	A	synonymous
54056	NTHI477_RS00260	300	A	G	synonymous
54062	NTHI477_RS00260	294	T	G	synonymous
54062	NTHI477_RS00260	294	T	A	synonymous
54065	NTHI477_RS00260	291	T	C	synonymous
54068	NTHI477_RS00260	288	G	A	synonymous
54094	NTHI477_RS00260	262	T	C	nonsynonymous
54094	NTHI477_RS00260	262	T	G	nonsynonymous
54125	NTHI477_RS00260	231	T	C	synonymous
54128	NTHI477_RS00260	228	G	A	synonymous
54131	NTHI477_RS00260	225	A	G	synonymous
54135	NTHI477_RS00260	221	A	T	nonsynonymous
54136	NTHI477_RS00260	220	C	G	nonsynonymous
54137	NTHI477_RS00260	219	A	C	nonsynonymous
54182	NTHI477_RS00260	174	G	A	synonymous
54200	NTHI477_RS00260	156	G	A	synonymous
54203	NTHI477_RS00260	153	A	G	synonymous
54206	NTHI477_RS00260	150	T	C	synonymous
54211	NTHI477_RS00260	145	G	A	synonymous
54224	NTHI477_RS00260	132	C	T	synonymous
54226	NTHI477_RS00260	130	G	T	nonsynonymous
54230	NTHI477_RS00260	126	T	A	synonymous
54233	NTHI477_RS00260	123	T	C	synonymous
54242	NTHI477_RS00260	114	C	G	synonymous
54242	NTHI477_RS00260	114	C	T	synonymous
54254	NTHI477_RS00260	102	A	G	synonymous
54260	NTHI477_RS00260	96	G	T	synonymous
54269	NTHI477_RS00260	87	A	G	synonymous
54270	NTHI477_RS00260	86	T	A	nonsynonymous
54275	NTHI477_RS00260	81	T	C	synonymous
54280	NTHI477_RS00260	76	C	T	nonsynonymous
54288	NTHI477_RS00260	68	A	G	nonsynonymous
54289	NTHI477_RS00260	67	T	C	nonsynonymous
54296	NTHI477_RS00260	60	A	G	synonymous
54305	NTHI477_RS00260	51	A	G	synonymous
54307	NTHI477_RS00260	49	T	C	nonsynonymous
54323	NTHI477_RS00260	33	G	A	synonymous
54326	NTHI477_RS00260	30	A	G	synonymous
54338	NTHI477_RS00260	18	G	T	synonymous
54347	NTHI477_RS00260	9	T	A	synonymous
54350	NTHI477_RS00260	6	A	G	synonymous
54362	NTHI477_RS00265	1070	C	T	nonsynonymous
54371	NTHI477_RS00265	1061	G	A	nonsynonymous
54379	NTHI477_RS00265	1053	A	G	synonymous
54391	NTHI477_RS00265	1041	C	A	synonymous

54418	NTHI477_RS00265	1014	G	A	synonymous
54442	NTHI477_RS00265	990	G	A	synonymous
54445	NTHI477_RS00265	987	G	A	synonymous
54467	NTHI477_RS00265	965	T	A	nonsynonymous
54490	NTHI477_RS00265	942	A	G	synonymous
54499	NTHI477_RS00265	933	C	T	synonymous
54511	NTHI477_RS00265	921	G	A	synonymous
54519	NTHI477_RS00265	913	T	A	nonsynonymous
54529	NTHI477_RS00265	903	G	T	synonymous
54562	NTHI477_RS00265	870	T	C	synonymous
54562	NTHI477_RS00265	870	T	G	synonymous
54577	NTHI477_RS00265	855	A	G	synonymous
54592	NTHI477_RS00265	840	G	A	synonymous
54598	NTHI477_RS00265	834	A	C	synonymous
54616	NTHI477_RS00265	816	A	G	synonymous
54625	NTHI477_RS00265	807	C	T	synonymous
54631	NTHI477_RS00265	801	A	G	synonymous
54637	NTHI477_RS00265	795	A	G	synonymous
54643	NTHI477_RS00265	789	G	A	synonymous
54646	NTHI477_RS00265	786	C	G	synonymous
54649	NTHI477_RS00265	783	A	G	synonymous
54670	NTHI477_RS00265	762	G	C	synonymous
54673	NTHI477_RS00265	759	A	G	synonymous
54675	NTHI477_RS00265	757	G	T	nonsynonymous
54679	NTHI477_RS00265	753	C	T	synonymous
54703	NTHI477_RS00265	729	A	G	synonymous
54705	NTHI477_RS00265	727	T	C	nonsynonymous
54706	NTHI477_RS00265	726	G	A	synonymous
54730	NTHI477_RS00265	702	G	A	synonymous
54733	NTHI477_RS00265	699	C	A	synonymous
54734	NTHI477_RS00265	698	G	A	nonsynonymous
54748	NTHI477_RS00265	684	G	A	synonymous
54753	NTHI477_RS00265	679	T	C	nonsynonymous
54763	NTHI477_RS00265	669	G	T	synonymous
54769	NTHI477_RS00265	663	A	C	synonymous
54772	NTHI477_RS00265	660	A	G	synonymous
54775	NTHI477_RS00265	657	A	G	synonymous
54778	NTHI477_RS00265	654	G	A	synonymous
54796	NTHI477_RS00265	636	A	T	synonymous
54799	NTHI477_RS00265	633	C	A	synonymous
54802	NTHI477_RS00265	630	C	T	synonymous
54811	NTHI477_RS00265	621	T	C	synonymous
54827	NTHI477_RS00265	605	C	T	nonsynonymous
54836	NTHI477_RS00265	596	T	G	nonsynonymous
54842	NTHI477_RS00265	590	C	T	nonsynonymous

54862	NTHI477_RS00265	570	G	A	synonymous
54868	NTHI477_RS00265	564	C	T	synonymous
54871	NTHI477_RS00265	561	A	G	synonymous
54874	NTHI477_RS00265	558	C	G	synonymous
54877	NTHI477_RS00265	555	T	G	synonymous
54886	NTHI477_RS00265	546	G	A	synonymous
54889	NTHI477_RS00265	543	T	G	synonymous
54889	NTHI477_RS00265	543	T	A	synonymous
54904	NTHI477_RS00265	528	T	C	synonymous
54919	NTHI477_RS00265	513	G	C	synonymous
54925	NTHI477_RS00265	507	T	C	synonymous
54928	NTHI477_RS00265	504	A	G	synonymous
54931	NTHI477_RS00265	501	C	A	synonymous
54940	NTHI477_RS00265	492	G	A	synonymous
54961	NTHI477_RS00265	471	T	A	synonymous
54961	NTHI477_RS00265	471	T	G	synonymous
54963	NTHI477_RS00265	469	C	T	nonsynonymous
54976	NTHI477_RS00265	456	C	T	synonymous
54979	NTHI477_RS00265	453	T	C	synonymous
54981	NTHI477_RS00265	451	G	T	nonsynonymous
55003	NTHI477_RS00265	429	T	A	synonymous
55006	NTHI477_RS00265	426	C	T	nonsynonymous
55022	NTHI477_RS00265	410	T	G	nonsynonymous
55027	NTHI477_RS00265	405	C	T	synonymous
55030	NTHI477_RS00265	402	A	G	synonymous
55066	NTHI477_RS00265	366	G	A	synonymous
55072	NTHI477_RS00265	360	G	A	synonymous
55078	NTHI477_RS00265	354	G	A	synonymous
55090	NTHI477_RS00265	342	A	G	synonymous
55093	NTHI477_RS00265	339	C	A	synonymous
55102	NTHI477_RS00265	330	C	T	synonymous
55138	NTHI477_RS00265	294	G	A	synonymous
55144	NTHI477_RS00265	288	G	A	synonymous
55146	NTHI477_RS00265	286	C	T	nonsynonymous
55180	NTHI477_RS00265	252	C	T	synonymous
55189	NTHI477_RS00265	243	C	T	synonymous
55189	NTHI477_RS00265	243	C	A	synonymous
55198	NTHI477_RS00265	234	G	C	synonymous
55200	NTHI477_RS00265	232	C	A	nonsynonymous
55231	NTHI477_RS00265	201	T	C	synonymous
55237	NTHI477_RS00265	195	A	G	synonymous
55240	NTHI477_RS00265	192	T	G	synonymous
55254	NTHI477_RS00265	178	C	T	nonsynonymous
55255	NTHI477_RS00265	177	A	C	nonsynonymous
55261	NTHI477_RS00265	171	T	C	synonymous

55267	NTHI477_RS00265	165	A	G	synonymous
55270	NTHI477_RS00265	162	G	A	synonymous
55272	NTHI477_RS00265	160	T	C	nonsynonymous
55279	NTHI477_RS00265	153	A	G	synonymous
55281	NTHI477_RS00265	151	C	T	nonsynonymous
55285	NTHI477_RS00265	147	G	A	synonymous
55302	NTHI477_RS00265	130	G	T	nonsynonymous
55303	NTHI477_RS00265	129	C	T	synonymous
55305	NTHI477_RS00265	127	G	C	nonsynonymous
55333	NTHI477_RS00265	99	C	T	synonymous
55360	NTHI477_RS00265	72	A	T	synonymous
55363	NTHI477_RS00265	69	G	T	synonymous
55379	NTHI477_RS00265	53	C	T	nonsynonymous
55381	NTHI477_RS00265	51	C	T	synonymous
55384	NTHI477_RS00265	48	A	T	synonymous
55393	NTHI477_RS00265	39	T	C	synonymous
55396	NTHI477_RS00265	36	T	C	synonymous
55402	NTHI477_RS00265	30	A	G	synonymous
55411	NTHI477_RS00265	21	G	A	synonymous
55458	NTHI477_RS00270	2102	G	T	nonsynonymous
55462	NTHI477_RS00270	2098	T	C	nonsynonymous
55471	NTHI477_RS00270	2089	T	G	nonsynonymous
55475	NTHI477_RS00270	2085	C	T	synonymous
55475	NTHI477_RS00270	2085	C	A	synonymous
55477	NTHI477_RS00270	2083	G	A	synonymous
55481	NTHI477_RS00270	2079	C	T	synonymous
55484	NTHI477_RS00270	2076	C	T	synonymous
55492	NTHI477_RS00270	2068	C	T	nonsynonymous
55493	NTHI477_RS00270	2067	C	T	synonymous
55493	NTHI477_RS00270	2067	C	A	synonymous
55502	NTHI477_RS00270	2058	G	A	synonymous
55505	NTHI477_RS00270	2055	G	A	synonymous
55505	NTHI477_RS00270	2055	G	T	synonymous
55514	NTHI477_RS00270	2046	G	T	synonymous
55517	NTHI477_RS00270	2043	T	G	synonymous
55517	NTHI477_RS00270	2043	T	C	synonymous
55529	NTHI477_RS00270	2031	G	A	synonymous
55537	NTHI477_RS00270	2023	C	T	nonsynonymous
55552	NTHI477_RS00270	2008	G	C	nonsynonymous
55569	NTHI477_RS00270	1991	C	T	nonsynonymous
55584	NTHI477_RS00270	1976	A	T	nonsynonymous
55586	NTHI477_RS00270	1974	T	A	nonsynonymous
55596	NTHI477_RS00270	1964	G	A	nonsynonymous
55603	NTHI477_RS00270	1957	G	T	nonsynonymous
55604	NTHI477_RS00270	1956	A	T	synonymous

55606	NTHI477_RS00270	1954	A	C	nonsynonymous
55607	NTHI477_RS00270	1953	G	A	synonymous
55615	NTHI477_RS00270	1945	T	C	nonsynonymous
55623	NTHI477_RS00270	1937	C	G	nonsynonymous
55624	NTHI477_RS00270	1936	T	C	nonsynonymous
55635	NTHI477_RS00270	1925	G	A	nonsynonymous
55640	NTHI477_RS00270	1920	C	T	synonymous
55652	NTHI477_RS00270	1908	T	C	synonymous
55661	NTHI477_RS00270	1899	A	T	synonymous
55664	NTHI477_RS00270	1896	G	A	synonymous
55670	NTHI477_RS00270	1890	C	G	synonymous
55673	NTHI477_RS00270	1887	T	G	synonymous
55676	NTHI477_RS00270	1884	A	G	synonymous
55682	NTHI477_RS00270	1878	G	A	synonymous
55685	NTHI477_RS00270	1875	T	C	synonymous
55694	NTHI477_RS00270	1866	A	C	synonymous
55696	NTHI477_RS00270	1864	G	C	nonsynonymous
55697	NTHI477_RS00270	1863	G	A	synonymous
55700	NTHI477_RS00270	1860	A	G	synonymous
55704	NTHI477_RS00270	1856	G	T	nonsynonymous
55756	NTHI477_RS00270	1804	C	G	nonsynonymous
55765	NTHI477_RS00270	1795	C	T	nonsynonymous
55774	NTHI477_RS00270	1786	A	C	nonsynonymous
55784	NTHI477_RS00270	1776	A	G	synonymous
55791	NTHI477_RS00270	1769	C	G	nonsynonymous
55795	NTHI477_RS00270	1765	T	A	nonsynonymous
55796	NTHI477_RS00270	1764	G	A	synonymous
55804	NTHI477_RS00270	1756	C	T	nonsynonymous
55805	NTHI477_RS00270	1755	T	C	synonymous
55808	NTHI477_RS00270	1752	T	C	synonymous
55832	NTHI477_RS00270	1728	A	G	synonymous
55837	NTHI477_RS00270	1723	T	G	nonsynonymous
55838	NTHI477_RS00270	1722	C	T	synonymous
55841	NTHI477_RS00270	1719	T	G	synonymous
55844	NTHI477_RS00270	1716	A	T	synonymous
55850	NTHI477_RS00270	1710	A	T	synonymous
55851	NTHI477_RS00270	1709	A	G	nonsynonymous
55853	NTHI477_RS00270	1707	C	T	synonymous
55867	NTHI477_RS00270	1693	C	T	nonsynonymous
55874	NTHI477_RS00270	1686	T	C	synonymous
55878	NTHI477_RS00270	1682	G	A	nonsynonymous
55879	NTHI477_RS00270	1681	T	C	nonsynonymous
55880	NTHI477_RS00270	1680	T	A	nonsynonymous
55881	NTHI477_RS00270	1679	T	G	nonsynonymous
55882	NTHI477_RS00270	1678	G	A	nonsynonymous

55892	NTHI477_RS00270	1668	G	A	synonymous
55916	NTHI477_RS00270	1644	C	T	synonymous
55955	NTHI477_RS00270	1605	G	A	synonymous
55979	NTHI477_RS00270	1581	C	T	synonymous
56015	NTHI477_RS00270	1545	T	C	synonymous
56045	NTHI477_RS00270	1515	T	G	synonymous
56087	NTHI477_RS00270	1473	G	T	synonymous
56117	NTHI477_RS00270	1443	A	G	synonymous
56129	NTHI477_RS00270	1431	A	T	synonymous
56129	NTHI477_RS00270	1431	A	C	synonymous
56204	NTHI477_RS00270	1356	C	T	synonymous
56207	NTHI477_RS00270	1353	T	C	synonymous
56219	NTHI477_RS00270	1341	G	T	synonymous
56228	NTHI477_RS00270	1332	A	G	synonymous
56234	NTHI477_RS00270	1326	T	A	synonymous
56249	NTHI477_RS00270	1311	G	A	synonymous
56255	NTHI477_RS00270	1305	C	T	synonymous
56288	NTHI477_RS00270	1272	G	T	synonymous
56291	NTHI477_RS00270	1269	G	A	synonymous
56297	NTHI477_RS00270	1263	C	T	synonymous
56321	NTHI477_RS00270	1239	G	A	synonymous
56357	NTHI477_RS00270	1203	C	T	synonymous
56420	NTHI477_RS00270	1140	G	A	synonymous
56443	NTHI477_RS00270	1117	C	T	nonsynonymous
56471	NTHI477_RS00270	1089	A	C	synonymous
56491	NTHI477_RS00270	1069	T	G	nonsynonymous
56504	NTHI477_RS00270	1056	T	C	synonymous
56510	NTHI477_RS00270	1050	G	T	nonsynonymous
56512	NTHI477_RS00270	1048	A	G	nonsynonymous
56513	NTHI477_RS00270	1047	G	A	synonymous
56528	NTHI477_RS00270	1032	T	C	synonymous
56546	NTHI477_RS00270	1014	C	T	synonymous
56547	NTHI477_RS00270	1013	A	G	nonsynonymous
56554	NTHI477_RS00270	1006	C	T	nonsynonymous
56555	NTHI477_RS00270	1005	A	G	synonymous
56563	NTHI477_RS00270	997	A	G	nonsynonymous
56582	NTHI477_RS00270	978	A	C	synonymous
56583	NTHI477_RS00270	977	A	G	nonsynonymous
56621	NTHI477_RS00270	939	A	C	synonymous
56633	NTHI477_RS00270	927	A	G	synonymous
56666	NTHI477_RS00270	894	A	G	synonymous
56669	NTHI477_RS00270	891	G	A	synonymous
56675	NTHI477_RS00270	885	T	C	synonymous
56701	NTHI477_RS00270	859	C	T	nonsynonymous
56717	NTHI477_RS00270	843	G	A	synonymous

56747	NTHI477_RS00270	813	G	C	synonymous
56759	NTHI477_RS00270	801	C	T	synonymous
56765	NTHI477_RS00270	795	T	C	synonymous
56766	NTHI477_RS00270	794	G	T	nonsynonymous
56771	NTHI477_RS00270	789	A	G	synonymous
56825	NTHI477_RS00270	735	G	A	synonymous
56843	NTHI477_RS00270	717	G	A	synonymous
56843	NTHI477_RS00270	717	G	T	synonymous
56845	NTHI477_RS00270	715	T	C	nonsynonymous
56846	NTHI477_RS00270	714	C	T	synonymous
56851	NTHI477_RS00270	709	C	T	nonsynonymous
56858	NTHI477_RS00270	702	G	A	synonymous
56879	NTHI477_RS00270	681	T	A	nonsynonymous
56880	NTHI477_RS00270	680	T	G	nonsynonymous
56892	NTHI477_RS00270	668	T	G	nonsynonymous
56893	NTHI477_RS00270	667	C	A	nonsynonymous
56903	NTHI477_RS00270	657	A	T	synonymous
56906	NTHI477_RS00270	654	C	T	synonymous
56942	NTHI477_RS00270	618	A	G	synonymous
56943	NTHI477_RS00270	617	C	T	nonsynonymous
56974	NTHI477_RS00270	586	C	T	nonsynonymous
56978	NTHI477_RS00270	582	T	G	synonymous
56990	NTHI477_RS00270	570	G	A	synonymous
56992	NTHI477_RS00270	568	G	A	nonsynonymous
57008	NTHI477_RS00270	552	G	A	synonymous
57020	NTHI477_RS00270	540	A	G	synonymous
57047	NTHI477_RS00270	513	A	G	synonymous
57098	NTHI477_RS00270	462	C	T	synonymous
57107	NTHI477_RS00270	453	C	T	synonymous
57122	NTHI477_RS00270	438	T	A	synonymous
57140	NTHI477_RS00270	420	G	A	synonymous
57149	NTHI477_RS00270	411	G	A	synonymous
57163	NTHI477_RS00270	397	G	A	nonsynonymous
57217	NTHI477_RS00270	343	T	C	nonsynonymous
57221	NTHI477_RS00270	339	T	C	synonymous
57223	NTHI477_RS00270	337	C	A	nonsynonymous
57224	NTHI477_RS00270	336	T	C	synonymous
57263	NTHI477_RS00270	297	G	C	synonymous
57269	NTHI477_RS00270	291	G	A	synonymous
57294	NTHI477_RS00270	266	A	G	nonsynonymous
57389	NTHI477_RS00270	171	G	A	synonymous
57404	NTHI477_RS00270	156	T	A	synonymous
57409	NTHI477_RS00270	151	C	G	nonsynonymous
57454	NTHI477_RS00270	106	G	A	nonsynonymous
57503	NTHI477_RS00270	57	G	T	synonymous

57605	NTHI477_RS00275	537	T	G	synonymous
57617	NTHI477_RS00275	525	A	G	synonymous
57650	NTHI477_RS00275	492	G	A	synonymous
57698	NTHI477_RS00275	444	C	A	synonymous
57719	NTHI477_RS00275	423	C	T	synonymous
57722	NTHI477_RS00275	420	G	A	synonymous
57737	NTHI477_RS00275	405	A	G	synonymous
57779	NTHI477_RS00275	363	A	G	synonymous
57794	NTHI477_RS00275	348	G	A	synonymous
57836	NTHI477_RS00275	306	G	A	synonymous
57842	NTHI477_RS00275	300	C	T	synonymous
57859	NTHI477_RS00275	283	C	T	nonsynonymous
57863	NTHI477_RS00275	279	A	C	nonsynonymous
57875	NTHI477_RS00275	267	C	T	synonymous
57889	NTHI477_RS00275	253	G	A	synonymous
57986	NTHI477_RS00275	156	A	G	synonymous
58070	NTHI477_RS00275	72	A	G	synonymous
58091	NTHI477_RS00275	51	T	C	synonymous
58109	NTHI477_RS00275	33	A	G	synonymous
58136	NTHI477_RS00275	6	A	G	synonymous
58173	intergenic	intergenic	A	T	intergenic
58174	intergenic	intergenic	C	G	intergenic
58176	intergenic	intergenic	G	A	intergenic
58195	intergenic	intergenic	G	C	intergenic
58215	intergenic	intergenic	A	G	intergenic
58216	intergenic	intergenic	G	T	intergenic
58232	NTHI477_RS00280	576	C	T	synonymous
58280	NTHI477_RS00280	528	A	G	synonymous
58297	NTHI477_RS00280	511	C	A	nonsynonymous
58314	NTHI477_RS00280	494	A	G	nonsynonymous
58316	NTHI477_RS00280	492	T	A	synonymous
58321	NTHI477_RS00280	487	C	T	nonsynonymous
58328	NTHI477_RS00280	480	T	G	synonymous
58333	NTHI477_RS00280	475	C	T	nonsynonymous
58349	NTHI477_RS00280	459	C	T	synonymous
58354	NTHI477_RS00280	454	C	T	nonsynonymous
58379	NTHI477_RS00280	429	G	A	synonymous
58412	NTHI477_RS00280	396	T	C	synonymous
58430	NTHI477_RS00280	378	A	G	synonymous
58438	NTHI477_RS00280	370	T	C	nonsynonymous
58447	NTHI477_RS00280	361	G	A	synonymous
58475	NTHI477_RS00280	333	G	A	synonymous
58553	NTHI477_RS00280	255	C	T	synonymous
58587	NTHI477_RS00280	221	A	G	nonsynonymous
58592	NTHI477_RS00280	216	T	C	synonymous

58601	NTHI477_RS00280	207	G	A	synonymous
58718	NTHI477_RS00280	90	G	A	synonymous
58736	NTHI477_RS00280	72	T	A	synonymous
58778	NTHI477_RS00280	30	T	G	synonymous
58780	NTHI477_RS00280	28	C	T	nonsynonymous
58787	NTHI477_RS00280	21	T	C	synonymous
58823	intergenic	intergenic	G	T	intergenic
58823	intergenic	intergenic	G	A	intergenic
58853	intergenic	intergenic	G	A	intergenic
58862	intergenic	intergenic	C	A	intergenic
58890	intergenic	intergenic	C	G	intergenic
58894	intergenic	intergenic	A	G	intergenic
58896	intergenic	intergenic	A	T	intergenic
59066	intergenic	intergenic	C	A	intergenic
59086	intergenic	intergenic	G	A	intergenic
59110	intergenic	intergenic	A	G	intergenic
59146	intergenic	intergenic	A	G	intergenic
59171	NTHI477_RS00285	1046	T	C	nonsynonymous
59184	NTHI477_RS00285	1033	C	T	nonsynonymous
59206	NTHI477_RS00285	1011	T	A	synonymous
59209	NTHI477_RS00285	1008	C	T	synonymous
59278	NTHI477_RS00285	939	C	T	synonymous
59303	NTHI477_RS00285	914	A	G	nonsynonymous
59335	NTHI477_RS00285	882	G	T	synonymous
59348	NTHI477_RS00285	869	T	G	nonsynonymous
59352	NTHI477_RS00285	865	T	A	nonsynonymous
59353	NTHI477_RS00285	864	G	A	synonymous
59377	NTHI477_RS00285	840	G	T	synonymous
59386	NTHI477_RS00285	831	G	A	synonymous
59428	NTHI477_RS00285	789	C	T	synonymous
59432	NTHI477_RS00285	785	T	G	nonsynonymous
59468	NTHI477_RS00285	749	A	T	nonsynonymous
59488	NTHI477_RS00285	729	C	A	synonymous
59491	NTHI477_RS00285	726	A	G	synonymous
59494	NTHI477_RS00285	723	T	C	synonymous
59497	NTHI477_RS00285	720	G	A	synonymous
59575	NTHI477_RS00285	642	A	G	synonymous
59614	NTHI477_RS00285	603	G	A	synonymous
59623	NTHI477_RS00285	594	G	A	synonymous
59683	NTHI477_RS00285	534	C	T	synonymous
59692	NTHI477_RS00285	525	A	G	synonymous
59701	NTHI477_RS00285	516	G	A	synonymous
59734	NTHI477_RS00285	483	C	T	synonymous
59770	NTHI477_RS00285	447	G	A	synonymous
59773	NTHI477_RS00285	444	A	G	synonymous

59776	NTHI477_RS00285	441	A	T	synonymous
59779	NTHI477_RS00285	438	A	G	synonymous
59803	NTHI477_RS00285	414	A	G	synonymous
59809	NTHI477_RS00285	408	A	T	synonymous
59811	NTHI477_RS00285	406	A	G	nonsynonymous
59815	NTHI477_RS00285	402	G	T	synonymous
59818	NTHI477_RS00285	399	G	T	synonymous
59827	NTHI477_RS00285	390	G	A	synonymous
59830	NTHI477_RS00285	387	A	T	synonymous
59844	NTHI477_RS00285	373	T	C	nonsynonymous
59845	NTHI477_RS00285	372	G	A	synonymous
59845	NTHI477_RS00285	372	G	T	synonymous
59929	NTHI477_RS00285	288	A	C	nonsynonymous
59932	NTHI477_RS00285	285	G	T	synonymous
59953	NTHI477_RS00285	264	A	G	synonymous
59965	NTHI477_RS00285	252	C	A	nonsynonymous
59977	NTHI477_RS00285	240	C	A	synonymous
59989	NTHI477_RS00285	228	A	T	synonymous
59995	NTHI477_RS00285	222	A	G	synonymous
59997	NTHI477_RS00285	220	T	C	nonsynonymous
59998	NTHI477_RS00285	219	G	T	synonymous
59999	NTHI477_RS00285	218	C	T	nonsynonymous
60001	NTHI477_RS00285	216	T	C	synonymous
60008	NTHI477_RS00285	209	T	A	nonsynonymous
60010	NTHI477_RS00285	207	T	C	synonymous
60054	NTHI477_RS00285	163	C	T	nonsynonymous
60066	NTHI477_RS00285	151	C	A	nonsynonymous
60068	NTHI477_RS00285	149	G	T	nonsynonymous
60094	NTHI477_RS00285	123	C	T	synonymous
60095	NTHI477_RS00285	122	G	A	nonsynonymous
60096	NTHI477_RS00285	121	T	C	nonsynonymous
60106	NTHI477_RS00285	111	G	T	nonsynonymous
60138	NTHI477_RS00285	79	C	G	nonsynonymous
60177	NTHI477_RS00285	40	A	G	synonymous
60261	intergenic	intergenic	G	A	intergenic
60307	intergenic	intergenic	T	G	intergenic
60308	intergenic	intergenic	T	C	intergenic
60364	intergenic	intergenic	C	G	intergenic
60365	intergenic	intergenic	T	C	intergenic
60422	NTHI477_RS00290	7	T	G	nonsynonymous
60427	NTHI477_RS00290	12	T	C	synonymous
60451	NTHI477_RS00290	36	T	A	synonymous
60457	NTHI477_RS00290	42	C	T	synonymous
60458	NTHI477_RS00290	43	G	A	nonsynonymous
60472	NTHI477_RS00290	57	C	T	synonymous

60490	NTHI477_RS00290	75	T	C	synonymous
60493	NTHI477_RS00290	78	G	T	synonymous
60502	NTHI477_RS00290	87	T	C	synonymous
60585	NTHI477_RS00290	170	G	A	nonsynonymous
60605	NTHI477_RS00290	190	T	C	synonymous
60622	NTHI477_RS00290	207	T	C	synonymous
60703	NTHI477_RS00290	288	T	C	synonymous
60711	NTHI477_RS00290	296	T	C	nonsynonymous
60715	NTHI477_RS00290	300	C	T	synonymous
60722	NTHI477_RS00290	307	C	A	nonsynonymous
60727	NTHI477_RS00290	312	C	T	synonymous
60751	NTHI477_RS00290	336	G	T	nonsynonymous
60762	NTHI477_RS00290	347	C	T	nonsynonymous
60814	NTHI477_RS00290	399	A	G	synonymous
60837	NTHI477_RS00290	422	G	C	nonsynonymous
60883	NTHI477_RS00290	468	T	G	synonymous
60883	NTHI477_RS00290	468	T	A	synonymous
60890	NTHI477_RS00290	475	G	A	nonsynonymous
60896	NTHI477_RS00290	481	G	A	nonsynonymous
60897	NTHI477_RS00290	482	C	A	nonsynonymous
60902	NTHI477_RS00290	487	G	A	nonsynonymous
60913	NTHI477_RS00290	498	T	A	synonymous
60914	NTHI477_RS00290	499	T	G	nonsynonymous
60915	NTHI477_RS00290	500	C	T	nonsynonymous
60919	NTHI477_RS00290	504	T	G	synonymous
60921	NTHI477_RS00290	506	A	G	nonsynonymous
60926	NTHI477_RS00290	511	A	G	nonsynonymous
60928	NTHI477_RS00290	513	G	A	nonsynonymous
60930	NTHI477_RS00290	515	T	C	nonsynonymous
60933	NTHI477_RS00290	518	C	T	nonsynonymous
60936	NTHI477_RS00290	521	C	T	nonsynonymous
60937	NTHI477_RS00290	522	T	G	synonymous
60937	NTHI477_RS00290	522	T	A	synonymous
60967	NTHI477_RS00290	552	C	A	synonymous
60991	NTHI477_RS00290	576	T	C	synonymous
61009	NTHI477_RS00290	594	T	C	synonymous
61048	NTHI477_RS00290	633	A	T	synonymous
61060	NTHI477_RS00290	645	G	C	nonsynonymous
61072	NTHI477_RS00290	657	C	T	synonymous
61088	intergenic	intergenic	A	C	intergenic
61100	intergenic	intergenic	C	T	intergenic
61167	NTHI477_RS00295	19	G	A	nonsynonymous
61187	NTHI477_RS00295	39	T	C	synonymous
61202	NTHI477_RS00295	54	T	C	synonymous
61244	NTHI477_RS00295	96	T	G	synonymous

61268	NTHI477_RS00295	120	A	C	synonymous
61271	NTHI477_RS00295	123	G	C	synonymous
61337	NTHI477_RS00295	189	T	C	synonymous
61340	NTHI477_RS00295	192	C	G	synonymous
61361	NTHI477_RS00295	213	T	A	synonymous
61368	NTHI477_RS00295	220	A	G	nonsynonymous
61424	NTHI477_RS00295	276	G	A	synonymous
61427	NTHI477_RS00295	279	G	A	synonymous
61461	NTHI477_RS00295	313	C	A	nonsynonymous
61523	NTHI477_RS00295	375	C	A	synonymous
61568	NTHI477_RS00295	420	T	C	synonymous
61574	NTHI477_RS00295	426	T	C	synonymous
61578	NTHI477_RS00295	430	C	T	nonsynonymous
61580	NTHI477_RS00295	432	T	G	synonymous
61586	NTHI477_RS00295	438	T	C	synonymous
61592	NTHI477_RS00295	444	C	G	synonymous
61592	NTHI477_RS00295	444	C	A	synonymous
61593	NTHI477_RS00295	445	G	A	nonsynonymous
61596	NTHI477_RS00295	448	A	C	nonsynonymous
61598	NTHI477_RS00295	450	C	G	nonsynonymous
61625	NTHI477_RS00295	477	T	C	synonymous
61628	NTHI477_RS00295	480	T	C	synonymous
61631	NTHI477_RS00295	483	A	G	synonymous
61661	NTHI477_RS00295	513	G	A	synonymous
61667	NTHI477_RS00295	519	C	T	synonymous
61667	NTHI477_RS00295	519	C	G	synonymous
61682	NTHI477_RS00295	534	A	T	synonymous
61682	NTHI477_RS00295	534	A	C	synonymous
61733	NTHI477_RS00295	585	T	C	synonymous
61736	NTHI477_RS00295	588	C	T	synonymous
61739	NTHI477_RS00295	591	A	T	nonsynonymous
61742	NTHI477_RS00295	594	T	C	synonymous
61745	NTHI477_RS00295	597	A	G	synonymous
61755	NTHI477_RS00295	607	G	A	nonsynonymous
61757	NTHI477_RS00295	609	A	G	synonymous
61781	NTHI477_RS00295	633	C	T	synonymous
61784	NTHI477_RS00295	636	G	T	synonymous
61787	NTHI477_RS00295	639	A	C	synonymous
61787	NTHI477_RS00295	639	A	T	synonymous
61793	NTHI477_RS00295	645	A	G	synonymous
61802	NTHI477_RS00295	654	C	T	synonymous
61805	NTHI477_RS00295	657	C	T	synonymous
61809	NTHI477_RS00295	661	A	C	synonymous
61811	NTHI477_RS00295	663	G	A	synonymous
61814	NTHI477_RS00295	666	G	A	synonymous

61830	NTHI477_RS00295	682	A	C	nonsynonymous
61835	NTHI477_RS00295	687	C	T	synonymous
61853	NTHI477_RS00295	705	C	T	synonymous
61862	NTHI477_RS00295	714	C	T	synonymous
61865	NTHI477_RS00295	717	G	A	synonymous
61871	NTHI477_RS00295	723	T	C	synonymous
61877	NTHI477_RS00295	729	C	T	synonymous
61880	NTHI477_RS00295	732	A	G	synonymous
61883	NTHI477_RS00295	735	C	T	synonymous
61884	NTHI477_RS00295	736	G	A	nonsynonymous
61964	NTHI477_RS00295	816	A	G	synonymous
61970	NTHI477_RS00295	822	A	G	synonymous
61985	NTHI477_RS00295	837	T	C	synonymous
62000	NTHI477_RS00295	852	C	G	synonymous
62009	NTHI477_RS00295	861	G	A	synonymous
62022	NTHI477_RS00295	874	C	T	nonsynonymous
62040	NTHI477_RS00295	892	T	C	nonsynonymous
62045	NTHI477_RS00295	897	G	C	synonymous
62051	NTHI477_RS00295	903	T	A	synonymous
62052	NTHI477_RS00295	904	G	T	nonsynonymous
62054	NTHI477_RS00295	906	G	C	synonymous
62066	NTHI477_RS00295	918	T	C	synonymous
62075	NTHI477_RS00295	927	C	T	synonymous
62081	NTHI477_RS00295	933	G	A	synonymous
62081	NTHI477_RS00295	933	G	T	nonsynonymous
62082	NTHI477_RS00295	934	T	C	synonymous
62084	NTHI477_RS00295	936	A	G	synonymous
62084	NTHI477_RS00295	936	A	C	nonsynonymous
62089	NTHI477_RS00295	941	A	G	nonsynonymous
62091	NTHI477_RS00295	943	A	G	nonsynonymous
62093	NTHI477_RS00295	945	T	A	synonymous
62100	NTHI477_RS00295	952	A	C	nonsynonymous
62101	NTHI477_RS00295	953	C	T	nonsynonymous
62107	NTHI477_RS00295	959	T	A	nonsynonymous
62108	NTHI477_RS00295	960	G	T	synonymous
62114	NTHI477_RS00295	966	T	C	synonymous
62118	NTHI477_RS00295	970	T	C	synonymous
62129	NTHI477_RS00295	981	T	C	synonymous
62153	NTHI477_RS00295	1005	G	A	synonymous
62156	NTHI477_RS00295	1008	T	C	synonymous
62159	NTHI477_RS00295	1011	T	C	synonymous
62177	NTHI477_RS00295	1029	G	A	synonymous
62180	NTHI477_RS00295	1032	A	G	synonymous
62182	NTHI477_RS00295	1034	C	G	nonsynonymous
62189	NTHI477_RS00295	1041	A	C	nonsynonymous

62190	NTHI477_RS00295	1042	A	G	nonsynonymous
62197	NTHI477_RS00295	1049	A	T	nonsynonymous
62201	NTHI477_RS00295	1053	A	C	synonymous
62210	NTHI477_RS00295	1062	G	A	synonymous
62237	NTHI477_RS00295	1089	A	T	synonymous
62252	NTHI477_RS00295	1104	C	A	synonymous
62285	NTHI477_RS00295	1137	G	A	synonymous
62288	NTHI477_RS00295	1140	G	A	synonymous
62297	NTHI477_RS00295	1149	T	C	synonymous
62300	NTHI477_RS00295	1152	T	A	synonymous
62303	NTHI477_RS00295	1155	T	C	synonymous
62304	NTHI477_RS00295	1156	T	G	nonsynonymous
62306	NTHI477_RS00295	1158	A	G	synonymous
62309	NTHI477_RS00295	1161	G	A	synonymous
62312	NTHI477_RS00295	1164	T	C	synonymous
62331	NTHI477_RS00295	1183	T	C	synonymous
62333	NTHI477_RS00295	1185	G	A	synonymous
62336	NTHI477_RS00295	1188	T	C	synonymous
62342	NTHI477_RS00295	1194	C	A	synonymous
62351	NTHI477_RS00295	1203	G	A	synonymous
62369	NTHI477_RS00295	1221	T	G	synonymous
62394	NTHI477_RS00295	1246	G	A	nonsynonymous
62405	NTHI477_RS00295	1257	T	C	synonymous
62408	NTHI477_RS00295	1260	G	A	synonymous
62414	NTHI477_RS00295	1266	C	T	synonymous
62438	NTHI477_RS00295	1290	A	G	synonymous
62447	NTHI477_RS00295	1299	C	T	synonymous
62453	NTHI477_RS00295	1305	T	C	synonymous
62456	NTHI477_RS00295	1308	T	C	synonymous
62468	NTHI477_RS00295	1320	T	C	synonymous
62477	NTHI477_RS00295	1329	G	A	synonymous
62480	NTHI477_RS00295	1332	G	A	synonymous
62483	NTHI477_RS00295	1335	A	T	synonymous
62484	NTHI477_RS00295	1336	T	C	synonymous
62507	NTHI477_RS00295	1359	C	T	synonymous
62521	NTHI477_RS00295	1373	T	A	nonsynonymous
62537	NTHI477_RS00295	1389	T	C	synonymous
62543	NTHI477_RS00295	1395	T	C	synonymous
62546	NTHI477_RS00295	1398	G	T	synonymous
62547	NTHI477_RS00295	1399	C	T	synonymous
62549	NTHI477_RS00295	1401	A	G	synonymous
62561	NTHI477_RS00295	1413	C	T	synonymous
62564	NTHI477_RS00295	1416	C	T	synonymous
62576	NTHI477_RS00295	1428	C	T	synonymous
62582	NTHI477_RS00295	1434	C	T	synonymous

62585	NTHI477_RS00295	1437	G	T	synonymous
62588	NTHI477_RS00295	1440	T	C	synonymous
62601	NTHI477_RS00295	1453	T	C	synonymous
62603	NTHI477_RS00295	1455	A	G	synonymous
62609	NTHI477_RS00295	1461	C	T	synonymous
62609	NTHI477_RS00295	1461	C	A	synonymous
62612	NTHI477_RS00295	1464	G	A	synonymous
62615	NTHI477_RS00295	1467	G	C	synonymous
62648	NTHI477_RS00295	1500	T	C	synonymous
62651	NTHI477_RS00295	1503	G	A	synonymous
62651	NTHI477_RS00295	1503	G	C	synonymous
62654	NTHI477_RS00295	1506	A	G	synonymous
62664	NTHI477_RS00295	1516	T	G	nonsynonymous
62669	NTHI477_RS00295	1521	C	T	synonymous
62675	NTHI477_RS00295	1527	T	A	synonymous
62678	NTHI477_RS00295	1530	G	A	synonymous
62687	NTHI477_RS00295	1539	A	C	synonymous
62687	NTHI477_RS00295	1539	A	T	synonymous
62692	NTHI477_RS00295	1544	T	G	nonsynonymous
62708	NTHI477_RS00295	1560	T	A	synonymous
62714	NTHI477_RS00295	1566	T	C	synonymous
62723	NTHI477_RS00295	1575	A	G	synonymous
62756	NTHI477_RS00295	1608	T	C	synonymous
62771	NTHI477_RS00295	1623	T	G	synonymous
62774	NTHI477_RS00295	1626	C	T	synonymous
62786	NTHI477_RS00295	1638	T	C	synonymous
62804	NTHI477_RS00295	1656	C	T	synonymous
62807	NTHI477_RS00295	1659	A	G	synonymous
62825	NTHI477_RS00295	1677	T	C	synonymous
62828	NTHI477_RS00295	1680	T	C	synonymous
62855	NTHI477_RS00295	1707	C	T	synonymous
62861	NTHI477_RS00295	1713	C	G	synonymous
62861	NTHI477_RS00295	1713	C	A	synonymous
62876	NTHI477_RS00295	1728	C	T	synonymous
62886	NTHI477_RS00295	1738	G	A	nonsynonymous
62891	NTHI477_RS00295	1743	T	A	synonymous
62891	NTHI477_RS00295	1743	T	G	synonymous
62897	NTHI477_RS00295	1749	T	C	synonymous
62912	NTHI477_RS00295	1764	A	G	synonymous
62918	NTHI477_RS00295	1770	G	T	synonymous
62924	NTHI477_RS00295	1776	C	T	synonymous
62928	NTHI477_RS00295	1780	G	A	nonsynonymous
62939	NTHI477_RS00295	1791	G	A	synonymous
62945	NTHI477_RS00295	1797	T	C	synonymous
62954	NTHI477_RS00295	1806	G	A	synonymous

62960	NTHI477_RS00295	1812	G	A	synonymous
62963	NTHI477_RS00295	1815	T	G	synonymous
62973	NTHI477_RS00295	1825	T	C	synonymous
62990	NTHI477_RS00295	1842	T	G	synonymous
62990	NTHI477_RS00295	1842	T	A	synonymous
62993	NTHI477_RS00295	1845	C	T	synonymous
62993	NTHI477_RS00295	1845	C	A	synonymous
62994	NTHI477_RS00295	1846	C	T	nonsynonymous
62996	NTHI477_RS00295	1848	T	G	synonymous
62999	NTHI477_RS00295	1851	T	C	synonymous
62999	NTHI477_RS00295	1851	T	A	synonymous
63008	NTHI477_RS00295	1860	C	A	synonymous
63024	NTHI477_RS00295	1876	C	T	synonymous
63035	NTHI477_RS00295	1887	C	T	synonymous
63041	NTHI477_RS00295	1893	G	A	synonymous
63050	NTHI477_RS00295	1902	A	T	synonymous
63062	NTHI477_RS00295	1914	T	C	synonymous
63080	NTHI477_RS00295	1932	T	C	synonymous
63095	NTHI477_RS00295	1947	C	T	synonymous
63107	NTHI477_RS00295	1959	C	A	synonymous
63110	NTHI477_RS00295	1962	C	T	synonymous
63111	NTHI477_RS00295	1963	G	A	nonsynonymous
63138	NTHI477_RS00295	1990	A	C	nonsynonymous
63143	NTHI477_RS00295	1995	C	T	synonymous
63158	NTHI477_RS00295	2010	G	A	synonymous
63164	NTHI477_RS00295	2016	G	A	synonymous
63176	NTHI477_RS00295	2028	A	G	synonymous
63176	NTHI477_RS00295	2028	A	C	nonsynonymous
63182	NTHI477_RS00295	2034	C	T	synonymous
63184	NTHI477_RS00295	2036	T	C	nonsynonymous
63185	NTHI477_RS00295	2037	G	C	synonymous
63185	NTHI477_RS00295	2037	G	A	synonymous
63185	NTHI477_RS00295	2037	G	T	synonymous
63194	NTHI477_RS00295	2046	A	C	synonymous
63195	NTHI477_RS00295	2047	G	A	nonsynonymous
63209	NTHI477_RS00295	2061	A	G	synonymous
63218	NTHI477_RS00295	2070	G	A	synonymous
63224	NTHI477_RS00295	2076	A	G	synonymous
63236	NTHI477_RS00295	2088	C	G	synonymous
63242	NTHI477_RS00295	2094	A	C	synonymous
63245	NTHI477_RS00295	2097	G	A	synonymous
63248	NTHI477_RS00295	2100	A	G	synonymous
63251	NTHI477_RS00295	2103	A	G	synonymous
63254	NTHI477_RS00295	2106	T	C	synonymous
63259	NTHI477_RS00295	2111	G	A	nonsynonymous

63268	NTHI477_RS00295	2120	A	G	nonsynonymous
63278	NTHI477_RS00295	2130	A	C	synonymous
63279	NTHI477_RS00295	2131	A	T	nonsynonymous
63280	NTHI477_RS00295	2132	A	T	nonsynonymous
63281	NTHI477_RS00295	2133	C	G	nonsynonymous
63283	NTHI477_RS00295	2135	G	T	nonsynonymous
63284	NTHI477_RS00295	2136	T	A	nonsynonymous
63285	NTHI477_RS00295	2137	C	A	nonsynonymous
63285	NTHI477_RS00295	2137	C	G	nonsynonymous
63288	NTHI477_RS00295	2140	A	C	nonsynonymous
63289	NTHI477_RS00295	2141	A	G	nonsynonymous
63290	NTHI477_RS00295	2142	C	T	synonymous
63291	NTHI477_RS00295	2143	A	G	nonsynonymous
63292	NTHI477_RS00295	2144	T	C	nonsynonymous
63293	NTHI477_RS00295	2145	A	G	nonsynonymous
63296	NTHI477_RS00295	2148	A	G	synonymous
63297	NTHI477_RS00295	2149	G	T	nonsynonymous
63307	intergenic	intergenic	T	G	intergenic
63336	intergenic	intergenic	T	A	intergenic
63346	intergenic	intergenic	G	T	intergenic
63348	intergenic	intergenic	T	C	intergenic
63358	intergenic	intergenic	G	T	intergenic
63359	intergenic	intergenic	G	A	intergenic
63377	intergenic	intergenic	T	A	intergenic
71916	intergenic	intergenic	A	T	intergenic
71923	intergenic	intergenic	A	G	intergenic
71944	intergenic	intergenic	T	C	intergenic
71946	intergenic	intergenic	A	G	intergenic
71951	intergenic	intergenic	T	G	intergenic
71953	intergenic	intergenic	C	T	intergenic
71955	intergenic	intergenic	T	C	intergenic
71961	intergenic	intergenic	T	C	intergenic
71968	intergenic	intergenic	G	C	intergenic
71970	intergenic	intergenic	T	C	intergenic
71974	intergenic	intergenic	C	T	intergenic
71975	intergenic	intergenic	T	C	intergenic
71976	intergenic	intergenic	T	C	intergenic
71977	intergenic	intergenic	T	C	intergenic
71978	intergenic	intergenic	C	T	intergenic
71979	intergenic	intergenic	C	T	intergenic
71980	intergenic	intergenic	C	T	intergenic
71981	intergenic	intergenic	T	C	intergenic
71983	intergenic	intergenic	T	C	intergenic
71985	intergenic	intergenic	T	C	intergenic
71987	intergenic	intergenic	T	C	intergenic

71989	intergenic	intergenic	A	T	intergenic
71990	intergenic	intergenic	C	T	intergenic
71991	intergenic	intergenic	G	C	intergenic
71992	intergenic	intergenic	G	A	intergenic
72003	intergenic	intergenic	G	T	intergenic
72004	intergenic	intergenic	A	C	intergenic
72006	intergenic	intergenic	A	T	intergenic
72079	intergenic	intergenic	T	A	intergenic
72170	intergenic	intergenic	C	T	intergenic
72172	intergenic	intergenic	G	A	intergenic
72177	intergenic	intergenic	A	G	intergenic
72181	intergenic	intergenic	G	C	intergenic
72201	intergenic	intergenic	T	C	intergenic
72206	intergenic	intergenic	T	C	intergenic
72208	intergenic	intergenic	A	G	intergenic
72225	intergenic	intergenic	C	T	intergenic
72230	intergenic	intergenic	G	A	intergenic
72232	intergenic	intergenic	A	C	intergenic
72242	intergenic	intergenic	C	T	intergenic
72245	intergenic	intergenic	A	C	intergenic
72251	intergenic	intergenic	A	G	intergenic
72256	intergenic	intergenic	G	A	intergenic
72258	intergenic	intergenic	G	A	intergenic
72260	intergenic	intergenic	A	G	intergenic
72261	intergenic	intergenic	A	G	intergenic
72262	intergenic	intergenic	G	A	intergenic
72263	intergenic	intergenic	G	A	intergenic
72264	intergenic	intergenic	G	A	intergenic
72273	NTHI477_RS00315	4	C	T	synonymous
72302	NTHI477_RS00315	33	T	C	synonymous
72314	NTHI477_RS00315	45	A	C	synonymous
72315	NTHI477_RS00315	46	G	A	nonsynonymous
72340	NTHI477_RS00315	71	C	A	nonsynonymous
72380	NTHI477_RS00315	111	T	C	synonymous
72401	NTHI477_RS00315	132	C	G	synonymous
72405	NTHI477_RS00315	136	C	T	nonsynonymous
72425	NTHI477_RS00315	156	A	G	synonymous
72431	NTHI477_RS00315	162	G	A	synonymous
72434	NTHI477_RS00315	165	G	A	synonymous
72452	NTHI477_RS00315	183	T	A	nonsynonymous
72455	NTHI477_RS00315	186	A	G	synonymous
72470	NTHI477_RS00315	201	G	A	synonymous
72479	NTHI477_RS00315	210	T	C	synonymous
72485	NTHI477_RS00315	216	C	T	synonymous
72486	NTHI477_RS00315	217	G	A	nonsynonymous

72511	NTHI477_RS00315	242	G	A	nonsynonymous
72512	NTHI477_RS00315	243	T	C	synonymous
72527	NTHI477_RS00315	258	A	G	synonymous
72530	NTHI477_RS00315	261	C	A	synonymous
72535	NTHI477_RS00315	266	C	T	nonsynonymous
72554	NTHI477_RS00315	285	C	T	synonymous
72560	NTHI477_RS00315	291	G	C	synonymous
72571	NTHI477_RS00315	302	A	G	nonsynonymous
72572	NTHI477_RS00315	303	A	G	synonymous
72579	NTHI477_RS00315	310	T	C	nonsynonymous
72597	NTHI477_RS00315	328	A	C	nonsynonymous
72599	NTHI477_RS00315	330	C	T	synonymous
72606	NTHI477_RS00315	337	T	C	synonymous
72623	NTHI477_RS00315	354	C	T	synonymous
72648	NTHI477_RS00315	379	G	T	nonsynonymous
72668	NTHI477_RS00315	399	C	T	synonymous
72680	NTHI477_RS00315	411	G	A	synonymous
72683	NTHI477_RS00315	414	A	T	synonymous
72683	NTHI477_RS00315	414	A	G	synonymous
72695	NTHI477_RS00315	426	T	C	synonymous
72710	NTHI477_RS00315	441	C	T	synonymous
72715	NTHI477_RS00315	446	C	T	nonsynonymous
72715	NTHI477_RS00315	446	C	A	nonsynonymous
72719	NTHI477_RS00315	450	G	A	synonymous
72722	NTHI477_RS00315	453	A	G	synonymous
72725	NTHI477_RS00315	456	T	G	synonymous
72728	NTHI477_RS00315	459	T	C	synonymous
72729	NTHI477_RS00315	460	T	A	nonsynonymous
72740	NTHI477_RS00315	471	G	A	synonymous
72749	NTHI477_RS00315	480	A	G	synonymous
72758	NTHI477_RS00315	489	T	C	synonymous
72758	NTHI477_RS00315	489	T	A	synonymous
72797	NTHI477_RS00315	528	T	G	synonymous
72800	NTHI477_RS00315	531	T	C	synonymous
72806	NTHI477_RS00315	537	G	A	synonymous
72824	NTHI477_RS00315	555	C	T	synonymous
72842	NTHI477_RS00315	573	T	G	synonymous
72852	NTHI477_RS00315	583	T	C	synonymous
72854	NTHI477_RS00315	585	A	G	synonymous
72857	NTHI477_RS00315	588	T	C	synonymous
72872	NTHI477_RS00315	603	G	A	synonymous
72875	NTHI477_RS00315	606	G	A	synonymous
72882	NTHI477_RS00315	613	T	C	synonymous
72884	NTHI477_RS00315	615	A	C	nonsynonymous
72902	NTHI477_RS00315	633	C	T	synonymous

72906	NTHI477_RS00315	637	A	G	nonsynonymous
72926	NTHI477_RS00315	657	T	C	synonymous
72942	NTHI477_RS00315	673	G	A	nonsynonymous
72943	NTHI477_RS00315	674	C	T	nonsynonymous
72954	NTHI477_RS00315	685	T	G	nonsynonymous
72956	NTHI477_RS00315	687	C	T	synonymous
72956	NTHI477_RS00315	687	C	G	synonymous
72960	NTHI477_RS00315	691	T	C	synonymous
72962	NTHI477_RS00315	693	G	T	nonsynonymous
72967	NTHI477_RS00315	698	T	C	nonsynonymous
72968	NTHI477_RS00315	699	A	G	nonsynonymous
72998	NTHI477_RS00315	729	C	A	synonymous
73000	NTHI477_RS00315	731	A	T	nonsynonymous
73005	NTHI477_RS00315	736	T	C	nonsynonymous
73008	NTHI477_RS00315	739	G	A	nonsynonymous
73009	NTHI477_RS00315	740	C	T	nonsynonymous
73040	NTHI477_RS00315	771	G	A	synonymous
73049	NTHI477_RS00315	780	C	T	synonymous
73054	NTHI477_RS00315	785	C	T	nonsynonymous
73083	NTHI477_RS00315	814	G	A	nonsynonymous
73091	NTHI477_RS00315	822	G	A	synonymous
73098	NTHI477_RS00315	829	T	C	synonymous
73106	NTHI477_RS00315	837	A	G	synonymous
73107	NTHI477_RS00315	838	G	A	nonsynonymous
73118	NTHI477_RS00315	849	A	G	synonymous
73125	NTHI477_RS00315	856	A	G	nonsynonymous
73135	NTHI477_RS00315	866	T	G	nonsynonymous
73142	NTHI477_RS00315	873	A	T	synonymous
73174	NTHI477_RS00315	905	C	T	nonsynonymous
73181	NTHI477_RS00315	912	C	T	synonymous
73187	NTHI477_RS00315	918	T	C	synonymous
73202	NTHI477_RS00315	933	C	T	synonymous
73260	NTHI477_RS00315	991	A	C	synonymous
73263	NTHI477_RS00315	994	G	A	nonsynonymous
73265	NTHI477_RS00315	996	T	C	synonymous
73272	NTHI477_RS00315	1003	G	A	nonsynonymous
73283	NTHI477_RS00315	1014	C	T	synonymous
73289	NTHI477_RS00315	1020	A	G	synonymous
73314	NTHI477_RS00315	1045	T	C	nonsynonymous
73324	intergenic	intergenic	C	T	intergenic
73332	intergenic	intergenic	A	G	intergenic
73336	intergenic	intergenic	C	A	intergenic
73341	intergenic	intergenic	T	C	intergenic
73345	intergenic	intergenic	T	C	intergenic
73352	intergenic	intergenic	G	A	intergenic

73353	intergenic	intergenic	G	A	intergenic
73355	intergenic	intergenic	T	C	intergenic
73356	intergenic	intergenic	A	G	intergenic
73358	intergenic	intergenic	T	C	intergenic
73368	intergenic	intergenic	C	T	intergenic
73369	intergenic	intergenic	C	T	intergenic
73378	intergenic	intergenic	A	G	intergenic
73380	intergenic	intergenic	G	C	intergenic
73381	intergenic	intergenic	C	T	intergenic
73383	intergenic	intergenic	T	C	intergenic
73383	intergenic	intergenic	T	G	intergenic
73390	intergenic	intergenic	C	T	intergenic
73401	intergenic	intergenic	G	A	intergenic
73402	intergenic	intergenic	A	T	intergenic
73406	intergenic	intergenic	G	T	intergenic
73409	intergenic	intergenic	T	A	intergenic
73413	intergenic	intergenic	T	A	intergenic
73416	intergenic	intergenic	A	G	intergenic
73443	intergenic	intergenic	G	A	intergenic
73452	intergenic	intergenic	C	T	intergenic
73478	intergenic	intergenic	T	C	intergenic
73492	intergenic	intergenic	C	T	intergenic
73494	intergenic	intergenic	C	G	intergenic
73498	intergenic	intergenic	A	T	intergenic
73500	intergenic	intergenic	A	C	intergenic
73512	intergenic	intergenic	T	C	intergenic
73514	intergenic	intergenic	T	C	intergenic
73522	intergenic	intergenic	C	A	intergenic
73526	intergenic	intergenic	C	T	intergenic
73547	intergenic	intergenic	G	A	intergenic
73564	NTHI477_RS00320	15	G	A	synonymous
73597	NTHI477_RS00320	48	A	G	synonymous
73627	NTHI477_RS00320	78	G	C	synonymous
73648	NTHI477_RS00320	99	C	A	synonymous
73651	NTHI477_RS00320	102	T	C	synonymous
73663	NTHI477_RS00320	114	G	C	synonymous
73705	NTHI477_RS00320	156	C	T	synonymous
73714	NTHI477_RS00320	165	A	G	synonymous
73714	NTHI477_RS00320	165	A	T	synonymous
73737	NTHI477_RS00320	188	C	T	nonsynonymous
73738	NTHI477_RS00320	189	C	G	synonymous
73741	NTHI477_RS00320	192	T	C	synonymous
73751	NTHI477_RS00320	202	A	T	nonsynonymous
73752	NTHI477_RS00320	203	G	C	nonsynonymous
73765	NTHI477_RS00320	216	A	G	synonymous

73768	NTHI477_RS00320	219	A	T	synonymous
73783	NTHI477_RS00320	234	C	T	synonymous
73788	NTHI477_RS00320	239	A	G	nonsynonymous
73795	NTHI477_RS00320	246	A	G	synonymous
73799	NTHI477_RS00320	250	C	A	synonymous
73801	NTHI477_RS00320	252	G	T	synonymous
73801	NTHI477_RS00320	252	G	C	synonymous
73805	NTHI477_RS00320	256	C	T	synonymous
73807	NTHI477_RS00320	258	A	T	synonymous
73807	NTHI477_RS00320	258	A	G	synonymous
73810	NTHI477_RS00320	261	C	T	synonymous
73816	NTHI477_RS00320	267	G	A	synonymous
73819	NTHI477_RS00320	270	C	T	synonymous
73824	NTHI477_RS00320	275	C	T	nonsynonymous
73828	NTHI477_RS00320	279	C	T	synonymous
73828	NTHI477_RS00320	279	C	G	synonymous
73828	NTHI477_RS00320	279	C	A	synonymous
73846	NTHI477_RS00320	297	T	A	synonymous
73858	NTHI477_RS00320	309	T	A	synonymous
73880	NTHI477_RS00320	331	T	C	synonymous
73888	NTHI477_RS00320	339	C	T	synonymous
73900	NTHI477_RS00320	351	A	G	synonymous
73903	NTHI477_RS00320	354	G	A	synonymous
73927	NTHI477_RS00320	378	G	A	synonymous
73942	NTHI477_RS00320	393	G	A	synonymous
73957	NTHI477_RS00320	408	G	A	synonymous
73963	NTHI477_RS00320	414	T	C	synonymous
73966	NTHI477_RS00320	417	T	A	synonymous
73969	NTHI477_RS00320	420	T	C	synonymous
73987	NTHI477_RS00320	438	C	T	synonymous
73987	NTHI477_RS00320	438	C	G	nonsynonymous
74023	NTHI477_RS00320	474	A	G	synonymous
74038	NTHI477_RS00320	489	G	A	synonymous
74047	NTHI477_RS00320	498	G	A	synonymous
74065	NTHI477_RS00320	516	A	C	synonymous
74071	NTHI477_RS00320	522	A	G	synonymous
74074	NTHI477_RS00320	525	C	T	synonymous
74077	NTHI477_RS00320	528	G	T	synonymous
74093	NTHI477_RS00320	544	C	T	synonymous
74098	NTHI477_RS00320	549	G	A	synonymous
74125	NTHI477_RS00320	576	G	A	synonymous
74128	NTHI477_RS00320	579	A	T	synonymous
74143	NTHI477_RS00320	594	T	C	synonymous
74152	NTHI477_RS00320	603	C	T	synonymous
74158	NTHI477_RS00320	609	T	C	synonymous

74173	NTHI477_RS00320	624	T	C	synonymous
74176	NTHI477_RS00320	627	C	A	synonymous
74182	NTHI477_RS00320	633	T	G	synonymous
74182	NTHI477_RS00320	633	T	C	synonymous
74185	NTHI477_RS00320	636	C	G	synonymous
74197	NTHI477_RS00320	648	G	T	synonymous
74200	NTHI477_RS00320	651	G	A	synonymous
74203	NTHI477_RS00320	654	T	C	synonymous
74206	NTHI477_RS00320	657	A	T	synonymous
74218	NTHI477_RS00320	669	T	A	synonymous
74227	NTHI477_RS00320	678	T	A	synonymous
74233	NTHI477_RS00320	684	G	T	synonymous
74251	NTHI477_RS00320	702	G	C	synonymous
74266	NTHI477_RS00320	717	A	G	synonymous
74267	NTHI477_RS00320	718	C	T	synonymous
74281	NTHI477_RS00320	732	G	A	synonymous
74284	NTHI477_RS00320	735	T	C	synonymous
74293	NTHI477_RS00320	744	C	T	synonymous
74311	NTHI477_RS00320	762	C	T	synonymous
74335	NTHI477_RS00320	786	A	C	synonymous
74344	NTHI477_RS00320	795	G	A	synonymous
74347	NTHI477_RS00320	798	C	T	synonymous
74361	NTHI477_RS00320	812	C	T	nonsynonymous
74377	NTHI477_RS00320	828	C	A	synonymous
74401	NTHI477_RS00320	852	C	T	synonymous
74413	NTHI477_RS00320	864	T	C	synonymous
74416	NTHI477_RS00320	867	G	T	synonymous
74416	NTHI477_RS00320	867	G	A	synonymous
74422	NTHI477_RS00320	873	G	A	synonymous
74428	NTHI477_RS00320	879	A	G	synonymous
74431	NTHI477_RS00320	882	G	A	synonymous
74437	NTHI477_RS00320	888	T	C	synonymous
74440	NTHI477_RS00320	891	C	T	synonymous
74443	NTHI477_RS00320	894	G	T	synonymous
74449	NTHI477_RS00320	900	G	A	synonymous
74458	NTHI477_RS00320	909	A	G	synonymous
74461	NTHI477_RS00320	912	C	T	synonymous
74464	NTHI477_RS00320	915	C	T	synonymous
74467	NTHI477_RS00320	918	C	T	synonymous
74479	NTHI477_RS00320	930	T	G	synonymous
74491	NTHI477_RS00320	942	T	C	synonymous
74494	NTHI477_RS00320	945	A	G	synonymous
74497	NTHI477_RS00320	948	A	G	synonymous
74510	NTHI477_RS00320	961	T	C	synonymous
74515	NTHI477_RS00320	966	G	A	synonymous

74554	NTHI477_RS00320	1005	C	T	synonymous
74557	NTHI477_RS00320	1008	A	G	synonymous
74558	NTHI477_RS00320	1009	G	T	nonsynonymous
74560	NTHI477_RS00320	1011	G	T	synonymous
74561	NTHI477_RS00320	1012	C	T	synonymous
74563	NTHI477_RS00320	1014	A	G	synonymous
74567	NTHI477_RS00320	1018	T	C	synonymous
74575	NTHI477_RS00320	1026	G	T	synonymous
74581	NTHI477_RS00320	1032	T	C	synonymous
74582	NTHI477_RS00320	1033	C	A	synonymous
74587	NTHI477_RS00320	1038	T	C	synonymous
74590	NTHI477_RS00320	1041	T	A	synonymous
74593	NTHI477_RS00320	1044	C	T	synonymous
74596	NTHI477_RS00320	1047	A	G	synonymous
74617	NTHI477_RS00320	1068	C	T	synonymous
74629	NTHI477_RS00320	1080	C	G	synonymous
74647	NTHI477_RS00320	1098	C	T	synonymous
74656	NTHI477_RS00320	1107	A	C	synonymous
74662	NTHI477_RS00320	1113	G	A	synonymous
74668	NTHI477_RS00320	1119	G	A	synonymous
74671	NTHI477_RS00320	1122	T	C	synonymous
74674	NTHI477_RS00320	1125	G	A	synonymous
74683	NTHI477_RS00320	1134	A	G	synonymous
74692	NTHI477_RS00320	1143	T	A	synonymous
74716	NTHI477_RS00320	1167	G	A	synonymous
74731	NTHI477_RS00320	1182	T	C	synonymous
74737	NTHI477_RS00320	1188	C	T	synonymous
74740	NTHI477_RS00320	1191	A	G	synonymous
74767	NTHI477_RS00320	1218	T	A	synonymous
74770	NTHI477_RS00320	1221	G	A	synonymous
74779	NTHI477_RS00320	1230	A	G	synonymous
74821	NTHI477_RS00320	1272	T	C	synonymous
74824	NTHI477_RS00320	1275	T	C	synonymous
74827	NTHI477_RS00320	1278	T	A	synonymous
74836	NTHI477_RS00320	1287	T	C	synonymous
74839	NTHI477_RS00320	1290	T	C	synonymous
74842	NTHI477_RS00320	1293	T	C	synonymous
74875	NTHI477_RS00320	1326	T	C	synonymous
74884	NTHI477_RS00320	1335	A	G	synonymous
74896	NTHI477_RS00320	1347	G	A	synonymous
74923	NTHI477_RS00320	1374	A	G	synonymous
74931	intergenic	intergenic	A	G	intergenic
74936	intergenic	intergenic	T	C	intergenic
74955	intergenic	intergenic	T	G	intergenic
74956	intergenic	intergenic	G	A	intergenic

75080	NTHI477_RS00325	75	A	G	synonymous
75103	NTHI477_RS00325	98	G	A	nonsynonymous
75125	NTHI477_RS00325	120	G	A	synonymous
75137	NTHI477_RS00325	132	A	G	synonymous
75140	NTHI477_RS00325	135	A	C	synonymous
75185	NTHI477_RS00325	180	G	A	synonymous
75212	NTHI477_RS00325	207	G	A	synonymous
75219	NTHI477_RS00325	214	C	T	synonymous
75224	NTHI477_RS00325	219	A	G	synonymous
75248	NTHI477_RS00325	243	G	A	synonymous
75257	NTHI477_RS00325	252	C	T	synonymous
75281	NTHI477_RS00325	276	G	A	synonymous
75292	NTHI477_RS00325	287	A	C	nonsynonymous
75293	NTHI477_RS00325	288	G	A	synonymous
75296	NTHI477_RS00325	291	A	G	synonymous
75299	NTHI477_RS00325	294	T	A	nonsynonymous
75371	NTHI477_RS00325	366	T	G	synonymous
75389	NTHI477_RS00325	384	G	A	synonymous
75392	NTHI477_RS00325	387	G	C	synonymous
75401	NTHI477_RS00325	396	A	G	synonymous
75404	NTHI477_RS00325	399	G	A	synonymous
75428	NTHI477_RS00325	423	T	G	synonymous
75464	NTHI477_RS00325	459	C	T	synonymous
75484	NTHI477_RS00325	479	A	G	synonymous
75495	NTHI477_RS00330	9	G	A	synonymous
75525	NTHI477_RS00330	39	G	A	synonymous
75534	NTHI477_RS00330	48	T	G	synonymous
75546	NTHI477_RS00330	60	A	G	synonymous
75552	NTHI477_RS00330	66	G	A	synonymous
75555	NTHI477_RS00330	69	C	T	synonymous
75568	NTHI477_RS00330	82	C	T	nonsynonymous
75621	NTHI477_RS00330	135	T	A	synonymous
75624	NTHI477_RS00330	138	A	G	synonymous
75624	NTHI477_RS00330	138	A	C	synonymous
75630	NTHI477_RS00330	144	C	T	synonymous
75657	NTHI477_RS00330	171	G	C	synonymous
75657	NTHI477_RS00330	171	G	A	synonymous
75660	NTHI477_RS00330	174	G	T	synonymous
75661	NTHI477_RS00330	175	A	T	nonsynonymous
75669	NTHI477_RS00330	183	A	G	synonymous
75702	NTHI477_RS00330	216	T	A	nonsynonymous
75705	NTHI477_RS00330	219	C	T	synonymous
75711	NTHI477_RS00330	225	C	T	synonymous
75731	NTHI477_RS00330	245	C	T	nonsynonymous
75744	NTHI477_RS00330	258	G	A	synonymous

75777	NTHI477_RS00330	291	A	G	synonymous
75780	NTHI477_RS00330	294	T	G	nonsynonymous
75783	NTHI477_RS00330	297	T	G	synonymous
75789	NTHI477_RS00330	303	C	T	synonymous
75795	NTHI477_RS00330	309	A	C	synonymous
75802	NTHI477_RS00330	316	C	T	synonymous
75804	NTHI477_RS00330	318	G	A	synonymous
75813	NTHI477_RS00330	327	C	T	synonymous
75816	NTHI477_RS00330	330	A	C	synonymous
75821	NTHI477_RS00330	335	T	C	nonsynonymous
75822	NTHI477_RS00330	336	T	C	synonymous
75828	NTHI477_RS00330	342	G	A	synonymous
75900	NTHI477_RS00330	414	G	A	synonymous
75903	NTHI477_RS00330	417	T	C	synonymous
75933	NTHI477_RS00330	447	T	C	synonymous
75948	NTHI477_RS00330	462	C	T	synonymous
75971	NTHI477_RS00330	485	T	C	nonsynonymous
75973	NTHI477_RS00330	487	T	C	nonsynonymous
75987	NTHI477_RS00330	501	C	T	synonymous
76002	NTHI477_RS00330	516	T	A	synonymous
76006	NTHI477_RS00330	520	G	A	nonsynonymous
76030	NTHI477_RS00330	544	G	A	nonsynonymous
76039	NTHI477_RS00330	553	T	C	nonsynonymous
76056	NTHI477_RS00330	570	G	A	synonymous
76077	NTHI477_RS00330	591	T	C	synonymous
76077	NTHI477_RS00330	591	T	A	synonymous
76078	NTHI477_RS00330	592	G	A	nonsynonymous
76080	NTHI477_RS00330	594	A	G	synonymous
76094	NTHI477_RS00330	608	C	T	nonsynonymous
76095	NTHI477_RS00330	609	A	G	synonymous
76113	NTHI477_RS00330	627	C	T	synonymous
76162	NTHI477_RS00330	676	C	T	nonsynonymous
76182	NTHI477_RS00330	696	C	T	synonymous
76188	NTHI477_RS00330	702	A	G	synonymous
76191	NTHI477_RS00330	705	A	T	synonymous
76197	NTHI477_RS00330	711	A	T	synonymous
76198	NTHI477_RS00330	712	G	C	nonsynonymous
76199	NTHI477_RS00330	713	T	A	nonsynonymous
76203	NTHI477_RS00330	717	G	A	synonymous
76204	NTHI477_RS00330	718	C	T	nonsynonymous
76205	NTHI477_RS00330	719	C	T	nonsynonymous
76209	NTHI477_RS00330	723	A	C	synonymous
76209	NTHI477_RS00330	723	A	T	synonymous
76215	NTHI477_RS00330	729	A	G	synonymous
76217	NTHI477_RS00330	731	C	T	nonsynonymous

76222	NTHI477_RS00330	736	A	G	nonsynonymous
76224	NTHI477_RS00330	738	T	A	nonsynonymous
76226	NTHI477_RS00330	740	T	G	nonsynonymous
76227	NTHI477_RS00330	741	T	C	synonymous
76231	NTHI477_RS00330	745	A	G	nonsynonymous
76235	NTHI477_RS00330	749	G	A	nonsynonymous
76237	NTHI477_RS00330	751	G	A	nonsynonymous
76237	NTHI477_RS00330	751	G	T	nonsynonymous
76238	NTHI477_RS00330	752	A	C	nonsynonymous
76240	NTHI477_RS00330	754	T	C	nonsynonymous
76248	NTHI477_RS00330	762	A	G	synonymous
76259	NTHI477_RS00330	773	A	G	nonsynonymous
76264	NTHI477_RS00330	778	A	C	nonsynonymous
76265	NTHI477_RS00330	779	G	A	nonsynonymous
76266	NTHI477_RS00330	780	C	T	synonymous
76269	NTHI477_RS00330	783	G	T	synonymous
76290	NTHI477_RS00330	804	C	A	synonymous
76308	NTHI477_RS00330	822	T	C	synonymous
76320	NTHI477_RS00330	834	A	G	synonymous
76362	NTHI477_RS00330	876	T	C	synonymous
76426	NTHI477_RS00330	940	G	A	nonsynonymous
76428	NTHI477_RS00330	942	A	C	synonymous
76430	NTHI477_RS00330	944	T	C	nonsynonymous
76472	NTHI477_RS00330	986	C	T	nonsynonymous
76594	NTHI477_RS00330	1108	G	A	nonsynonymous
76606	NTHI477_RS00330	1120	A	G	nonsynonymous
76619	NTHI477_RS00330	1133	G	A	nonsynonymous
76648	NTHI477_RS00330	1162	C	G	nonsynonymous
76663	NTHI477_RS00330	1177	G	A	nonsynonymous
76666	NTHI477_RS00330	1180	A	G	nonsynonymous
76710	NTHI477_RS00330	1224	A	G	synonymous
76721	NTHI477_RS00330	1235	C	G	nonsynonymous
76729	NTHI477_RS00330	1243	A	G	nonsynonymous
76875	NTHI477_RS00330	1389	A	G	synonymous
76901	NTHI477_RS00330	1415	C	G	nonsynonymous
76912	NTHI477_RS00330	1426	G	A	nonsynonymous
76959	NTHI477_RS00330	1473	G	A	synonymous
76962	NTHI477_RS00330	1476	G	T	synonymous
76965	NTHI477_RS00330	1479	T	G	synonymous
76974	NTHI477_RS00330	1488	A	G	synonymous
76987	NTHI477_RS00330	1501	A	G	nonsynonymous
77022	NTHI477_RS00330	1536	T	G	nonsynonymous
77118	NTHI477_RS00330	1632	A	G	synonymous
77154	NTHI477_RS00330	1668	T	C	synonymous
77187	NTHI477_RS00330	1701	T	C	synonymous

77196	NTHI477_RS00330	1710	T	C	synonymous
77211	NTHI477_RS00330	1725	A	G	synonymous
77238	NTHI477_RS00330	1752	T	C	synonymous
77241	NTHI477_RS00330	1755	T	A	synonymous
77283	NTHI477_RS00330	1797	C	A	synonymous
77298	NTHI477_RS00330	1812	T	C	synonymous
77307	NTHI477_RS00330	1821	A	T	synonymous
77373	NTHI477_RS00330	1887	A	G	synonymous
77448	NTHI477_RS00330	1962	A	G	synonymous
77473	NTHI477_RS00330	1987	T	C	synonymous
77478	NTHI477_RS00330	1992	C	A	synonymous
77484	NTHI477_RS00330	1998	A	C	synonymous
77487	NTHI477_RS00330	2001	C	T	synonymous
77490	NTHI477_RS00330	2004	T	C	synonymous
77493	NTHI477_RS00330	2007	A	G	synonymous
77496	NTHI477_RS00330	2010	C	T	synonymous
77502	NTHI477_RS00330	2016	T	C	synonymous
77520	NTHI477_RS00330	2034	C	T	synonymous
77523	NTHI477_RS00330	2037	A	C	nonsynonymous
77538	NTHI477_RS00330	2052	T	A	synonymous
77544	NTHI477_RS00330	2058	A	G	synonymous
77561	NTHI477_RS00330	2075	C	A	nonsynonymous
77562	NTHI477_RS00330	2076	G	T	synonymous
77563	NTHI477_RS00330	2077	A	G	nonsynonymous
77571	NTHI477_RS00330	2085	G	A	synonymous
77586	NTHI477_RS00330	2100	A	C	synonymous
77589	NTHI477_RS00330	2103	A	C	synonymous
77592	NTHI477_RS00330	2106	G	A	synonymous
77646	NTHI477_RS00330	2160	G	T	nonsynonymous
77652	NTHI477_RS00330	2166	A	G	synonymous
77655	NTHI477_RS00330	2169	G	C	synonymous
77673	NTHI477_RS00330	2187	A	G	synonymous
77676	NTHI477_RS00330	2190	G	C	synonymous
77700	NTHI477_RS00330	2214	G	C	synonymous
77700	NTHI477_RS00330	2214	G	A	synonymous
77706	NTHI477_RS00330	2220	T	C	synonymous
77751	NTHI477_RS00330	2265	T	C	synonymous
77796	NTHI477_RS00330	2310	C	T	synonymous
77802	NTHI477_RS00330	2316	G	C	synonymous
77805	NTHI477_RS00330	2319	G	T	synonymous
77820	NTHI477_RS00330	2334	C	T	synonymous
77826	NTHI477_RS00330	2340	T	C	synonymous
77832	NTHI477_RS00330	2346	T	C	synonymous
77844	NTHI477_RS00330	2358	G	T	synonymous
77847	NTHI477_RS00330	2361	T	C	synonymous

77847	NTHI477_RS00330	2361	T	G	synonymous
77850	NTHI477_RS00330	2364	A	T	synonymous
77853	NTHI477_RS00330	2367	A	G	synonymous
77856	NTHI477_RS00330	2370	C	G	synonymous
77856	NTHI477_RS00330	2370	C	T	synonymous
77862	NTHI477_RS00330	2376	A	G	synonymous
77865	NTHI477_RS00330	2379	G	T	synonymous
77910	NTHI477_RS00330	2424	C	T	synonymous
77913	NTHI477_RS00330	2427	C	T	synonymous
77919	NTHI477_RS00330	2433	T	C	synonymous
77940	NTHI477_RS00330	2454	T	C	synonymous
77946	NTHI477_RS00330	2460	C	A	synonymous
77949	NTHI477_RS00330	2463	C	T	synonymous
77958	NTHI477_RS00330	2472	C	G	synonymous
77976	NTHI477_RS00330	2490	C	T	synonymous
77982	NTHI477_RS00330	2496	A	G	synonymous
77985	NTHI477_RS00330	2499	T	G	synonymous
77994	NTHI477_RS00330	2508	T	C	synonymous
78000	NTHI477_RS00330	2514	A	C	synonymous
78006	NTHI477_RS00330	2520	A	G	synonymous
78012	NTHI477_RS00330	2526	T	C	synonymous
78013	NTHI477_RS00330	2527	G	A	nonsynonymous
78014	NTHI477_RS00330	2528	C	T	nonsynonymous
78015	NTHI477_RS00330	2529	T	A	synonymous
78015	NTHI477_RS00330	2529	T	G	synonymous
78018	NTHI477_RS00330	2532	C	T	synonymous
78024	NTHI477_RS00330	2538	G	A	synonymous
78027	NTHI477_RS00330	2541	A	T	nonsynonymous
78029	NTHI477_RS00330	2543	G	A	nonsynonymous
78031	NTHI477_RS00330	2545	A	T	nonsynonymous
78031	NTHI477_RS00330	2545	A	G	nonsynonymous
78042	NTHI477_RS00330	2556	G	A	synonymous
78050	NTHI477_RS00330	2564	G	A	nonsynonymous
78051	NTHI477_RS00330	2565	C	T	synonymous
78057	NTHI477_RS00330	2571	A	G	synonymous
78090	NTHI477_RS00330	2604	C	T	synonymous
78102	NTHI477_RS00330	2616	T	C	synonymous
78119	NTHI477_RS00330	2633	T	C	nonsynonymous
78147	NTHI477_RS00330	2661	A	G	synonymous
78162	NTHI477_RS00330	2676	A	T	synonymous
78162	NTHI477_RS00330	2676	A	C	synonymous
78165	NTHI477_RS00330	2679	G	T	synonymous
78168	NTHI477_RS00330	2682	T	C	synonymous
78189	NTHI477_RS00330	2703	G	A	synonymous
78195	NTHI477_RS00330	2709	G	A	synonymous

78228	NTHI477_RS00330	2742	A	G	synonymous
78244	NTHI477_RS00330	2758	C	T	nonsynonymous
78252	NTHI477_RS00330	2766	C	T	synonymous
78264	intergenic	intergenic	G	A	intergenic
78269	intergenic	intergenic	T	C	intergenic
78329	NTHI477_RS00335	57	G	T	nonsynonymous
78344	NTHI477_RS00335	72	G	A	synonymous
78356	NTHI477_RS00335	84	A	G	synonymous
78368	NTHI477_RS00335	96	A	G	synonymous
78383	NTHI477_RS00335	111	A	T	synonymous
78389	NTHI477_RS00335	117	T	C	synonymous
78425	NTHI477_RS00335	153	A	C	synonymous
78473	NTHI477_RS00335	201	T	C	synonymous
78482	NTHI477_RS00335	210	T	C	synonymous
78545	NTHI477_RS00335	273	C	T	synonymous
78566	NTHI477_RS00335	294	G	A	synonymous
78569	NTHI477_RS00335	297	C	G	synonymous
78581	NTHI477_RS00335	309	A	T	synonymous
78585	NTHI477_RS00335	313	A	G	nonsynonymous
78593	NTHI477_RS00335	321	A	C	synonymous
78599	NTHI477_RS00335	327	T	C	synonymous
78602	NTHI477_RS00335	330	T	G	synonymous
78608	NTHI477_RS00335	336	T	C	synonymous
78611	NTHI477_RS00335	339	T	G	synonymous
78623	NTHI477_RS00335	351	C	T	synonymous
78624	NTHI477_RS00335	352	A	G	nonsynonymous
78630	NTHI477_RS00335	358	A	C	nonsynonymous
78632	NTHI477_RS00335	360	T	G	nonsynonymous
78634	NTHI477_RS00335	362	G	C	nonsynonymous
78656	NTHI477_RS00335	384	A	G	synonymous
78666	NTHI477_RS00335	394	T	G	nonsynonymous
78671	NTHI477_RS00335	399	T	C	synonymous
78692	NTHI477_RS00335	420	C	T	synonymous
78698	NTHI477_RS00335	426	C	A	synonymous
78707	NTHI477_RS00335	435	C	T	synonymous
78728	NTHI477_RS00335	456	T	C	synonymous
78736	NTHI477_RS00335	464	C	T	nonsynonymous
78761	NTHI477_RS00335	489	T	C	synonymous
78779	NTHI477_RS00335	507	G	T	synonymous
78809	NTHI477_RS00335	537	T	C	synonymous
78812	NTHI477_RS00335	540	G	T	synonymous
78819	NTHI477_RS00335	547	A	G	nonsynonymous
78849	NTHI477_RS00335	577	C	T	nonsynonymous
78850	NTHI477_RS00335	578	C	T	nonsynonymous
78861	NTHI477_RS00335	589	G	A	nonsynonymous

78869	NTHI477_RS00335	597	G	T	synonymous
78875	NTHI477_RS00335	603	C	T	synonymous
78881	NTHI477_RS00335	609	T	C	synonymous
78891	NTHI477_RS00335	619	C	T	nonsynonymous
78892	intergenic	intergenic	A	G	intergenic
78894	intergenic	intergenic	G	A	intergenic
78904	intergenic	intergenic	T	G	intergenic
78927	intergenic	intergenic	C	T	intergenic
78931	intergenic	intergenic	C	T	intergenic
78950	intergenic	intergenic	A	C	intergenic
78956	NTHI477_RS00340	6	T	G	synonymous
78976	NTHI477_RS00340	26	C	T	nonsynonymous
79019	NTHI477_RS00340	69	A	G	synonymous
79021	NTHI477_RS00340	71	A	G	nonsynonymous
79047	NTHI477_RS00340	97	C	T	nonsynonymous
79071	NTHI477_RS00340	121	C	T	synonymous
79094	NTHI477_RS00340	144	G	C	synonymous
79094	NTHI477_RS00340	144	G	T	synonymous
79106	NTHI477_RS00340	156	T	A	synonymous
79121	NTHI477_RS00340	171	C	A	synonymous
79124	NTHI477_RS00340	174	A	G	synonymous
79133	NTHI477_RS00340	183	G	A	synonymous
79136	NTHI477_RS00340	186	G	A	synonymous
79145	NTHI477_RS00340	195	C	G	synonymous
79148	NTHI477_RS00340	198	T	C	synonymous
79178	NTHI477_RS00340	228	A	G	synonymous
79211	NTHI477_RS00340	261	T	C	synonymous
79238	NTHI477_RS00340	288	T	G	nonsynonymous
79241	NTHI477_RS00340	291	T	A	synonymous
79267	NTHI477_RS00340	317	G	A	nonsynonymous
79334	NTHI477_RS00340	384	G	A	synonymous
79337	NTHI477_RS00340	387	C	T	synonymous
79346	NTHI477_RS00340	396	C	T	synonymous
79351	NTHI477_RS00340	401	C	G	nonsynonymous
79352	NTHI477_RS00340	402	C	T	synonymous
79361	NTHI477_RS00340	411	T	C	synonymous
79406	NTHI477_RS00340	456	G	A	synonymous
79421	NTHI477_RS00340	471	C	G	synonymous
79422	NTHI477_RS00340	472	C	A	synonymous
79424	NTHI477_RS00340	474	A	C	synonymous
79427	NTHI477_RS00340	477	G	A	synonymous
79433	NTHI477_RS00340	483	G	A	synonymous
79445	NTHI477_RS00340	495	C	T	synonymous
79482	NTHI477_RS00340	532	G	A	nonsynonymous
79494	NTHI477_RS00340	544	G	A	nonsynonymous

79499	NTHI477_RS00340	549	T	A	synonymous
79518	NTHI477_RS00340	568	T	C	synonymous
79547	NTHI477_RS00340	597	A	G	synonymous
79574	NTHI477_RS00340	624	A	G	synonymous
79574	NTHI477_RS00340	624	A	T	synonymous
79583	NTHI477_RS00340	633	C	T	synonymous
79592	NTHI477_RS00340	642	C	T	synonymous
79616	NTHI477_RS00340	666	A	G	synonymous
79619	NTHI477_RS00340	669	A	T	nonsynonymous
79624	NTHI477_RS00340	674	C	T	nonsynonymous
79625	NTHI477_RS00340	675	G	T	synonymous
79673	NTHI477_RS00340	723	A	T	synonymous
79673	NTHI477_RS00340	723	A	C	synonymous
79679	NTHI477_RS00340	729	C	T	synonymous
79703	NTHI477_RS00340	753	T	C	synonymous
79745	NTHI477_RS00340	795	T	C	synonymous
79748	NTHI477_RS00340	798	A	C	synonymous
79763	NTHI477_RS00340	813	T	C	synonymous
79766	NTHI477_RS00340	816	T	A	synonymous
79769	NTHI477_RS00340	819	G	A	synonymous
79796	NTHI477_RS00340	846	A	G	synonymous
79799	NTHI477_RS00340	849	G	A	synonymous
79805	NTHI477_RS00340	855	A	C	synonymous
79808	NTHI477_RS00340	858	C	T	synonymous
79812	NTHI477_RS00340	862	T	C	synonymous
79820	NTHI477_RS00340	870	T	C	synonymous
79826	NTHI477_RS00340	876	C	T	synonymous
79837	NTHI477_RS00340	887	C	T	nonsynonymous
79838	NTHI477_RS00340	888	C	T	synonymous
79874	NTHI477_RS00340	924	A	T	synonymous
79886	NTHI477_RS00340	936	C	T	synonymous
79934	NTHI477_RS00340	984	G	A	synonymous
79938	NTHI477_RS00340	988	C	A	synonymous
79940	NTHI477_RS00340	990	A	T	synonymous
79946	NTHI477_RS00340	996	C	T	synonymous
79949	NTHI477_RS00340	999	C	T	synonymous
79958	NTHI477_RS00340	1008	T	C	synonymous
79961	NTHI477_RS00340	1011	T	A	synonymous
79967	NTHI477_RS00340	1017	T	C	synonymous
79970	NTHI477_RS00340	1020	A	G	synonymous
79973	NTHI477_RS00340	1023	A	T	synonymous
79976	NTHI477_RS00340	1026	C	A	synonymous
79976	NTHI477_RS00340	1026	C	G	synonymous
79976	NTHI477_RS00340	1026	C	T	synonymous
79985	NTHI477_RS00340	1035	T	C	synonymous

79991	NTHI477_RS00340	1041	G	C	synonymous
79991	NTHI477_RS00340	1041	G	A	synonymous
80000	NTHI477_RS00340	1050	C	T	synonymous
80000	NTHI477_RS00340	1050	C	G	synonymous
80003	NTHI477_RS00340	1053	A	G	synonymous
80009	NTHI477_RS00340	1059	C	T	synonymous
80012	NTHI477_RS00340	1062	T	A	synonymous
80015	NTHI477_RS00340	1065	G	A	synonymous
80036	NTHI477_RS00340	1086	A	G	synonymous
80039	NTHI477_RS00340	1089	T	A	synonymous
80039	NTHI477_RS00340	1089	T	C	synonymous
80045	NTHI477_RS00340	1095	C	T	synonymous
80054	NTHI477_RS00340	1104	A	C	synonymous
80075	NTHI477_RS00340	1125	G	A	synonymous
80093	NTHI477_RS00340	1143	G	A	synonymous
80096	NTHI477_RS00340	1146	C	T	synonymous
80099	NTHI477_RS00340	1149	A	C	synonymous
80103	NTHI477_RS00340	1153	G	A	nonsynonymous
80105	NTHI477_RS00340	1155	C	T	synonymous
80105	NTHI477_RS00340	1155	C	G	synonymous
80106	NTHI477_RS00340	1156	A	G	nonsynonymous
80123	NTHI477_RS00340	1173	T	C	synonymous
80146	NTHI477_RS00340	1196	C	T	nonsynonymous
80178	NTHI477_RS00340	1228	A	C	nonsynonymous
80183	NTHI477_RS00340	1233	C	T	synonymous
80201	NTHI477_RS00340	1251	G	A	synonymous
80204	NTHI477_RS00340	1254	A	G	synonymous
80246	NTHI477_RS00340	1296	G	A	synonymous
80268	NTHI477_RS00340	1318	G	A	nonsynonymous
80273	NTHI477_RS00340	1323	G	A	synonymous
80293	intergenic	intergenic	C	T	intergenic
80293	intergenic	intergenic	C	A	intergenic
80312	intergenic	intergenic	A	G	intergenic
80332	intergenic	intergenic	C	T	intergenic
80339	intergenic	intergenic	G	A	intergenic
80345	intergenic	intergenic	T	C	intergenic
80346	intergenic	intergenic	T	G	intergenic
80357	intergenic	intergenic	G	A	intergenic
80371	intergenic	intergenic	C	T	intergenic
80377	NTHI477_RS00345	1294	C	T	nonsynonymous
80379	NTHI477_RS00345	1292	C	T	nonsynonymous
80384	NTHI477_RS00345	1287	A	G	synonymous
80390	NTHI477_RS00345	1281	C	T	synonymous
80393	NTHI477_RS00345	1278	T	C	synonymous
80403	NTHI477_RS00345	1268	T	C	nonsynonymous

80405	NTHI477_RS00345	1266	G	A	synonymous
80414	NTHI477_RS00345	1257	T	A	synonymous
80426	NTHI477_RS00345	1245	T	G	synonymous
80429	NTHI477_RS00345	1242	A	T	synonymous
80438	NTHI477_RS00345	1233	T	G	synonymous
80450	NTHI477_RS00345	1221	T	C	synonymous
80456	NTHI477_RS00345	1215	A	T	synonymous
80462	NTHI477_RS00345	1209	A	G	synonymous
80465	NTHI477_RS00345	1206	G	T	synonymous
80468	NTHI477_RS00345	1203	C	T	synonymous
80471	NTHI477_RS00345	1200	A	T	synonymous
80495	NTHI477_RS00345	1176	C	T	nonsynonymous
80518	NTHI477_RS00345	1153	T	C	nonsynonymous
80528	NTHI477_RS00345	1143	G	A	synonymous
80547	NTHI477_RS00345	1124	G	A	nonsynonymous
80548	NTHI477_RS00345	1123	C	G	nonsynonymous
80562	NTHI477_RS00345	1109	C	T	nonsynonymous
80576	NTHI477_RS00345	1095	G	A	synonymous
80599	NTHI477_RS00345	1072	C	T	nonsynonymous
80606	NTHI477_RS00345	1065	G	T	synonymous
80612	NTHI477_RS00345	1059	A	G	synonymous
80621	NTHI477_RS00345	1050	A	T	synonymous
80624	NTHI477_RS00345	1047	C	T	synonymous
80630	NTHI477_RS00345	1041	A	T	synonymous
80645	NTHI477_RS00345	1026	C	T	synonymous
80648	NTHI477_RS00345	1023	A	T	synonymous
80685	NTHI477_RS00345	986	T	C	nonsynonymous
80689	NTHI477_RS00345	982	C	A	nonsynonymous
80696	NTHI477_RS00345	975	A	T	synonymous
80699	NTHI477_RS00345	972	A	G	synonymous
80705	NTHI477_RS00345	966	C	T	synonymous
80755	NTHI477_RS00345	916	T	C	nonsynonymous
80768	NTHI477_RS00345	903	T	G	nonsynonymous
80780	NTHI477_RS00345	891	G	A	synonymous
80801	NTHI477_RS00345	870	G	A	synonymous
80819	NTHI477_RS00345	852	C	T	synonymous
80858	NTHI477_RS00345	813	G	A	synonymous
80876	NTHI477_RS00345	795	T	G	synonymous
80897	NTHI477_RS00345	774	G	T	synonymous
80899	NTHI477_RS00345	772	C	T	nonsynonymous
80900	NTHI477_RS00345	771	A	G	synonymous
80900	NTHI477_RS00345	771	A	T	synonymous
80909	NTHI477_RS00345	762	G	A	synonymous
80921	NTHI477_RS00345	750	T	G	synonymous
80924	NTHI477_RS00345	747	C	T	synonymous

80930	NTHI477_RS00345	741	G	T	synonymous
80936	NTHI477_RS00345	735	G	C	synonymous
80948	NTHI477_RS00345	723	G	A	synonymous
80978	NTHI477_RS00345	693	G	T	synonymous
80981	NTHI477_RS00345	690	T	C	synonymous
80986	NTHI477_RS00345	685	T	C	nonsynonymous
80987	NTHI477_RS00345	684	G	T	nonsynonymous
80989	NTHI477_RS00345	682	A	G	nonsynonymous
80990	NTHI477_RS00345	681	G	A	synonymous
81005	NTHI477_RS00345	666	A	C	nonsynonymous
81007	NTHI477_RS00345	664	G	T	nonsynonymous
81008	NTHI477_RS00345	663	A	G	synonymous
81017	NTHI477_RS00345	654	C	G	nonsynonymous
81019	NTHI477_RS00345	652	T	G	nonsynonymous
81119	NTHI477_RS00345	552	T	C	synonymous
81212	NTHI477_RS00345	459	C	T	synonymous
81248	NTHI477_RS00345	423	T	A	synonymous
81251	NTHI477_RS00345	420	A	C	synonymous
81254	NTHI477_RS00345	417	C	T	synonymous
81255	NTHI477_RS00345	416	G	A	nonsynonymous
81265	NTHI477_RS00345	406	G	A	synonymous
81266	NTHI477_RS00345	405	C	A	synonymous
81272	NTHI477_RS00345	399	T	G	synonymous
81275	NTHI477_RS00345	396	C	T	synonymous
81281	NTHI477_RS00345	390	A	G	synonymous
81287	NTHI477_RS00345	384	A	C	synonymous
81287	NTHI477_RS00345	384	A	T	synonymous
81290	NTHI477_RS00345	381	T	C	synonymous
81293	NTHI477_RS00345	378	T	C	synonymous
81302	NTHI477_RS00345	369	C	A	synonymous
81321	NTHI477_RS00345	350	A	G	nonsynonymous
81330	NTHI477_RS00345	341	A	C	nonsynonymous
81330	NTHI477_RS00345	341	A	T	nonsynonymous
81331	NTHI477_RS00345	340	C	T	nonsynonymous
81336	NTHI477_RS00345	335	T	G	nonsynonymous
81337	NTHI477_RS00345	334	G	T	nonsynonymous
81338	NTHI477_RS00345	333	G	T	nonsynonymous
81383	NTHI477_RS00345	288	C	G	synonymous
81404	NTHI477_RS00345	267	T	C	synonymous
81413	NTHI477_RS00345	258	A	G	synonymous
81415	NTHI477_RS00345	256	T	C	nonsynonymous
81419	NTHI477_RS00345	252	C	T	nonsynonymous
81430	NTHI477_RS00345	241	C	T	nonsynonymous
81434	NTHI477_RS00345	237	G	A	synonymous
81435	NTHI477_RS00345	236	C	T	nonsynonymous

81443	NTHI477_RS00345	228	C	T	synonymous
81448	NTHI477_RS00345	223	T	C	nonsynonymous
81488	NTHI477_RS00345	183	G	A	synonymous
81491	NTHI477_RS00345	180	C	T	synonymous
81496	NTHI477_RS00345	175	C	T	nonsynonymous
81500	NTHI477_RS00345	171	G	C	synonymous
81503	NTHI477_RS00345	168	G	T	synonymous
81546	NTHI477_RS00345	125	G	A	nonsynonymous
81560	NTHI477_RS00345	111	G	T	synonymous
81568	NTHI477_RS00345	103	C	A	nonsynonymous
81589	NTHI477_RS00345	82	C	T	nonsynonymous
81590	NTHI477_RS00345	81	G	A	synonymous
81637	NTHI477_RS00345	34	C	T	nonsynonymous
81682	NTHI477_RS00350	825	C	T	synonymous
81766	NTHI477_RS00350	741	A	T	synonymous
81865	NTHI477_RS00350	642	C	A	synonymous
81871	NTHI477_RS00350	636	G	A	synonymous
81874	NTHI477_RS00350	633	G	A	synonymous
81901	NTHI477_RS00350	606	T	C	synonymous
81919	NTHI477_RS00350	588	A	T	synonymous
81934	NTHI477_RS00350	573	T	C	synonymous
81976	NTHI477_RS00350	531	C	T	synonymous
81989	NTHI477_RS00350	518	G	T	nonsynonymous
82035	NTHI477_RS00350	472	T	C	nonsynonymous
82144	NTHI477_RS00350	363	A	C	nonsynonymous
82251	NTHI477_RS00350	256	C	A	nonsynonymous
82452	NTHI477_RS00350	55	C	T	nonsynonymous
82485	NTHI477_RS00350	22	A	G	nonsynonymous
82501	NTHI477_RS00350	6	A	G	synonymous
82560	intergenic	intergenic	G	T	intergenic
82563	intergenic	intergenic	T	C	intergenic
82721	NTHI477_RS00355	260	C	T	nonsynonymous
82752	NTHI477_RS00355	229	C	T	nonsynonymous
82936	NTHI477_RS00355	45	G	A	synonymous
83077	intergenic	intergenic	G	T	intergenic
83122	intergenic	intergenic	C	A	intergenic
83141	intergenic	intergenic	C	T	intergenic
83147	intergenic	intergenic	C	A	intergenic
83180	intergenic	intergenic	T	C	intergenic
83186	intergenic	intergenic	C	T	intergenic
83203	intergenic	intergenic	C	T	intergenic
83353	intergenic	intergenic	T	C	intergenic
83354	intergenic	intergenic	G	A	intergenic
83366	intergenic	intergenic	T	C	intergenic
83384	intergenic	intergenic	G	A	intergenic

83450	NTHI477_RS00360	28	G	T	nonsynonymous
83569	NTHI477_RS00360	147	T	C	synonymous
83576	NTHI477_RS00360	154	G	A	nonsynonymous
83601	NTHI477_RS00360	179	T	A	nonsynonymous
83731	NTHI477_RS00360	309	G	A	synonymous
83746	NTHI477_RS00360	324	G	A	synonymous
83748	NTHI477_RS00360	326	G	A	nonsynonymous
83749	NTHI477_RS00360	327	T	G	synonymous
83830	NTHI477_RS00360	408	T	C	synonymous
83833	NTHI477_RS00360	411	A	G	synonymous
83854	NTHI477_RS00360	432	T	C	synonymous
83929	NTHI477_RS00360	507	T	A	synonymous
83935	NTHI477_RS00360	513	A	C	synonymous
83944	NTHI477_RS00360	522	C	T	synonymous
83950	NTHI477_RS00360	528	C	T	synonymous
83968	NTHI477_RS00360	546	T	C	synonymous
84055	NTHI477_RS00360	633	C	T	synonymous
84134	NTHI477_RS00360	712	G	A	nonsynonymous
84166	NTHI477_RS00360	744	G	A	synonymous
84194	NTHI477_RS00360	772	G	A	nonsynonymous
84247	NTHI477_RS00360	825	G	A	synonymous
84250	NTHI477_RS00360	828	C	T	synonymous
84304	NTHI477_RS00360	882	A	G	synonymous
84400	NTHI477_RS00360	978	T	C	synonymous
84412	NTHI477_RS00360	990	T	C	synonymous
84421	NTHI477_RS00360	999	C	T	synonymous
84445	NTHI477_RS00360	1023	C	T	synonymous
84487	NTHI477_RS00360	1065	C	A	synonymous
84493	NTHI477_RS00360	1071	C	T	synonymous
84505	NTHI477_RS00360	1083	A	T	synonymous
84515	NTHI477_RS00360	1093	C	T	synonymous
84523	NTHI477_RS00360	1101	T	C	synonymous
84529	NTHI477_RS00360	1107	G	T	synonymous
84546	NTHI477_RS00360	1124	T	C	nonsynonymous
84556	NTHI477_RS00360	1134	A	G	synonymous
84559	NTHI477_RS00360	1137	A	T	synonymous
84562	NTHI477_RS00360	1140	G	T	synonymous
84571	NTHI477_RS00360	1149	C	T	synonymous
84572	NTHI477_RS00360	1150	A	G	nonsynonymous
84581	NTHI477_RS00360	1159	T	G	nonsynonymous
84583	NTHI477_RS00360	1161	T	A	synonymous
84586	NTHI477_RS00360	1164	A	G	synonymous
84592	NTHI477_RS00360	1170	A	G	synonymous
84595	NTHI477_RS00360	1173	T	C	synonymous
84616	NTHI477_RS00360	1194	A	G	synonymous

84622	NTHI477_RS00360	1200	C	T	synonymous
84625	NTHI477_RS00360	1203	T	C	synonymous
84634	NTHI477_RS00360	1212	G	A	synonymous
84643	NTHI477_RS00360	1221	C	A	synonymous
84646	NTHI477_RS00360	1224	A	G	synonymous
84649	NTHI477_RS00360	1227	A	T	synonymous
84655	NTHI477_RS00360	1233	A	T	synonymous
84658	NTHI477_RS00360	1236	G	A	synonymous
84664	NTHI477_RS00360	1242	C	T	synonymous
84679	NTHI477_RS00360	1257	G	A	synonymous
84682	NTHI477_RS00360	1260	T	C	synonymous
84700	NTHI477_RS00360	1278	A	G	synonymous
84706	NTHI477_RS00360	1284	A	T	synonymous
84712	NTHI477_RS00360	1290	T	A	synonymous
84721	NTHI477_RS00360	1299	C	T	synonymous
84739	NTHI477_RS00360	1317	G	A	synonymous
84769	NTHI477_RS00360	1347	C	T	synonymous
84794	NTHI477_RS00360	1372	G	T	nonsynonymous
84823	NTHI477_RS00360	1401	A	C	synonymous
84840	NTHI477_RS00360	1418	C	G	nonsynonymous
84844	NTHI477_RS00360	1422	T	C	synonymous
84846	NTHI477_RS00360	1424	G	T	nonsynonymous
84851	NTHI477_RS00360	1429	A	T	nonsynonymous
84857	NTHI477_RS00360	1435	G	A	nonsynonymous
84859	NTHI477_RS00360	1437	A	T	synonymous
84891	NTHI477_RS00360	1469	C	T	nonsynonymous
84951	intergenic	intergenic	A	C	intergenic
84965	intergenic	intergenic	C	T	intergenic
85037	intergenic	intergenic	T	A	intergenic
85039	intergenic	intergenic	A	G	intergenic
85039	intergenic	intergenic	A	T	intergenic
85072	intergenic	intergenic	C	A	intergenic
85093	intergenic	intergenic	T	A	intergenic
85146	intergenic	intergenic	G	T	intergenic
85152	intergenic	intergenic	A	G	intergenic
85155	intergenic	intergenic	C	T	intergenic
85198	intergenic	intergenic	T	A	intergenic
85227	NTHI477_RS00365	18	C	T	synonymous
85239	NTHI477_RS00365	30	G	A	synonymous
85253	NTHI477_RS00365	44	G	A	nonsynonymous
85296	NTHI477_RS00365	87	G	A	synonymous
85336	NTHI477_RS00365	127	A	G	nonsynonymous
85464	NTHI477_RS00365	255	T	C	synonymous
85569	NTHI477_RS00365	360	C	T	synonymous
85662	NTHI477_RS00365	453	G	T	synonymous

85674	NTHI477_RS00365	465	C	T	synonymous
85713	NTHI477_RS00365	504	T	G	synonymous
85737	NTHI477_RS00365	528	C	T	synonymous
85745	NTHI477_RS00365	536	A	C	nonsynonymous
85770	NTHI477_RS00365	561	C	A	synonymous
85806	NTHI477_RS00365	597	G	A	synonymous
85824	NTHI477_RS00365	615	G	A	synonymous
85830	NTHI477_RS00365	621	G	A	synonymous
85836	NTHI477_RS00365	627	A	G	synonymous
85845	NTHI477_RS00365	636	T	C	synonymous
85848	NTHI477_RS00365	639	C	T	synonymous
85854	NTHI477_RS00365	645	T	C	synonymous
85857	NTHI477_RS00365	648	T	A	synonymous
85857	NTHI477_RS00365	648	T	C	synonymous
85879	NTHI477_RS00365	670	G	A	nonsynonymous
85884	NTHI477_RS00365	675	G	A	synonymous
85885	NTHI477_RS00365	676	T	G	nonsynonymous
85917	NTHI477_RS00365	708	C	A	synonymous
85926	NTHI477_RS00365	717	C	A	synonymous
85929	NTHI477_RS00365	720	A	G	synonymous
85944	NTHI477_RS00365	735	T	G	synonymous
86044	NTHI477_RS00365	835	A	G	nonsynonymous
86067	NTHI477_RS00365	858	T	C	synonymous
86133	NTHI477_RS00365	924	C	T	synonymous
86241	NTHI477_RS00365	1032	G	A	synonymous
86241	NTHI477_RS00365	1032	G	T	synonymous
86242	NTHI477_RS00365	1033	G	A	nonsynonymous
86307	NTHI477_RS00365	1098	C	T	synonymous
86313	NTHI477_RS00365	1104	T	A	synonymous
86314	NTHI477_RS00365	1105	G	A	nonsynonymous
86373	NTHI477_RS00365	1164	T	C	synonymous
86430	NTHI477_RS00365	1221	C	T	synonymous
86439	NTHI477_RS00365	1230	G	T	synonymous
86460	NTHI477_RS00365	1251	T	C	synonymous
86466	NTHI477_RS00365	1257	A	T	synonymous
86499	NTHI477_RS00365	1290	T	A	synonymous
86529	NTHI477_RS00365	1320	T	G	synonymous
86538	NTHI477_RS00365	1329	C	T	synonymous
86547	NTHI477_RS00365	1338	C	T	synonymous
86553	NTHI477_RS00365	1344	T	C	synonymous
86592	NTHI477_RS00365	1383	G	A	synonymous
86592	NTHI477_RS00365	1383	G	C	synonymous
86595	NTHI477_RS00365	1386	G	A	synonymous
86601	NTHI477_RS00365	1392	T	C	synonymous
86605	NTHI477_RS00365	1396	C	T	nonsynonymous

86607	NTHI477_RS00365	1398	T	A	synonymous
86613	NTHI477_RS00365	1404	C	T	synonymous
86664	NTHI477_RS00365	1455	T	C	synonymous
86682	NTHI477_RS00365	1473	C	T	synonymous
86688	NTHI477_RS00365	1479	G	A	synonymous
86720	NTHI477_RS00365	1511	C	T	nonsynonymous
86730	NTHI477_RS00365	1521	A	T	synonymous
86883	NTHI477_RS00365	1674	A	T	synonymous
86883	NTHI477_RS00365	1674	A	C	synonymous
86924	NTHI477_RS00365	1715	C	G	nonsynonymous
86966	NTHI477_RS00370	36	G	A	synonymous
86969	NTHI477_RS00370	39	T	G	synonymous
86972	NTHI477_RS00370	42	T	A	synonymous
86975	NTHI477_RS00370	45	T	A	synonymous
86978	NTHI477_RS00370	48	A	G	synonymous
86996	NTHI477_RS00370	66	A	G	synonymous
87002	NTHI477_RS00370	72	A	C	synonymous
87002	NTHI477_RS00370	72	A	T	synonymous
87011	NTHI477_RS00370	81	C	T	synonymous
87035	NTHI477_RS00370	105	T	G	synonymous
87038	NTHI477_RS00370	108	A	G	synonymous
87063	NTHI477_RS00370	133	C	A	synonymous
87071	NTHI477_RS00370	141	C	T	synonymous
87080	NTHI477_RS00370	150	T	A	synonymous
87080	NTHI477_RS00370	150	T	C	synonymous
87090	NTHI477_RS00370	160	G	A	nonsynonymous
87095	NTHI477_RS00370	165	G	A	synonymous
87110	NTHI477_RS00370	180	C	T	synonymous
87122	NTHI477_RS00370	192	T	C	synonymous
87140	NTHI477_RS00370	210	G	A	synonymous
87164	NTHI477_RS00370	234	G	A	synonymous
87203	NTHI477_RS00370	273	A	C	synonymous
87212	NTHI477_RS00370	282	G	A	synonymous
87215	NTHI477_RS00370	285	G	T	synonymous
87215	NTHI477_RS00370	285	G	A	synonymous
87230	NTHI477_RS00370	300	A	G	synonymous
87245	NTHI477_RS00370	315	A	C	synonymous
87257	NTHI477_RS00370	327	C	T	synonymous
87356	NTHI477_RS00370	426	T	C	synonymous
87383	NTHI477_RS00370	453	A	C	synonymous
87395	NTHI477_RS00370	465	G	A	synonymous
87457	intergenic	intergenic	A	C	intergenic
87477	intergenic	intergenic	G	T	intergenic
87483	intergenic	intergenic	T	C	intergenic
87501	intergenic	intergenic	T	A	intergenic

87532	intergenic	intergenic	C	T	intergenic
87557	intergenic	intergenic	A	T	intergenic
87559	intergenic	intergenic	C	T	intergenic
87559	intergenic	intergenic	C	A	intergenic
87577	intergenic	intergenic	A	T	intergenic
87578	intergenic	intergenic	A	C	intergenic
87638	intergenic	intergenic	C	T	intergenic
87642	intergenic	intergenic	A	T	intergenic
87646	intergenic	intergenic	G	A	intergenic
87660	intergenic	intergenic	T	G	intergenic
87662	intergenic	intergenic	A	T	intergenic
87663	intergenic	intergenic	G	A	intergenic
87665	intergenic	intergenic	A	T	intergenic
87666	intergenic	intergenic	A	T	intergenic
87672	intergenic	intergenic	T	C	intergenic
87672	intergenic	intergenic	T	A	intergenic
87679	intergenic	intergenic	A	G	intergenic
87689	intergenic	intergenic	C	G	intergenic
87690	intergenic	intergenic	T	A	intergenic
87691	intergenic	intergenic	G	A	intergenic
87692	intergenic	intergenic	A	G	intergenic
87712	intergenic	intergenic	T	A	intergenic
87750	NTHI477_RS00375	1700	G	T	nonsynonymous
87751	NTHI477_RS00375	1699	T	C	nonsynonymous
87758	NTHI477_RS00375	1692	C	T	synonymous
87764	NTHI477_RS00375	1686	T	C	synonymous
87776	NTHI477_RS00375	1674	T	C	synonymous
87788	NTHI477_RS00375	1662	C	T	synonymous
87796	NTHI477_RS00375	1654	A	G	synonymous
87797	NTHI477_RS00375	1653	A	G	synonymous
87800	NTHI477_RS00375	1650	A	G	synonymous
87811	NTHI477_RS00375	1639	T	C	nonsynonymous
87812	NTHI477_RS00375	1638	A	G	synonymous
87814	NTHI477_RS00375	1636	A	C	nonsynonymous
87815	NTHI477_RS00375	1635	T	C	synonymous
87820	NTHI477_RS00375	1630	T	C	nonsynonymous
87839	NTHI477_RS00375	1611	G	A	synonymous
87845	NTHI477_RS00375	1605	T	C	synonymous
87848	NTHI477_RS00375	1602	A	G	synonymous
87857	NTHI477_RS00375	1593	C	T	synonymous
87863	NTHI477_RS00375	1587	G	T	synonymous
87869	NTHI477_RS00375	1581	A	T	synonymous
87878	NTHI477_RS00375	1572	A	G	synonymous
87902	NTHI477_RS00375	1548	T	C	synonymous
87905	NTHI477_RS00375	1545	T	C	synonymous

87947	NTHI477_RS00375	1503	T	G	synonymous
87953	NTHI477_RS00375	1497	C	T	synonymous
87965	NTHI477_RS00375	1485	C	G	synonymous
87996	NTHI477_RS00375	1454	G	A	nonsynonymous
87997	NTHI477_RS00375	1453	G	A	nonsynonymous
87999	NTHI477_RS00375	1451	G	A	nonsynonymous
88004	NTHI477_RS00375	1446	C	T	synonymous
88016	NTHI477_RS00375	1434	T	C	synonymous
88025	NTHI477_RS00375	1425	A	C	nonsynonymous
88028	NTHI477_RS00375	1422	T	A	synonymous
88052	NTHI477_RS00375	1398	T	A	synonymous
88055	NTHI477_RS00375	1395	A	G	synonymous
88061	NTHI477_RS00375	1389	G	A	synonymous
88079	NTHI477_RS00375	1371	T	C	synonymous
88082	NTHI477_RS00375	1368	A	C	synonymous
88088	NTHI477_RS00375	1362	A	G	synonymous
88090	NTHI477_RS00375	1360	C	T	nonsynonymous
88094	NTHI477_RS00375	1356	G	A	synonymous
88124	NTHI477_RS00375	1326	G	A	synonymous
88130	NTHI477_RS00375	1320	A	C	synonymous
88133	NTHI477_RS00375	1317	A	G	synonymous
88136	NTHI477_RS00375	1314	A	G	synonymous
88139	NTHI477_RS00375	1311	G	A	synonymous
88148	NTHI477_RS00375	1302	G	T	synonymous
88154	NTHI477_RS00375	1296	G	C	synonymous
88154	NTHI477_RS00375	1296	G	T	synonymous
88163	NTHI477_RS00375	1287	C	T	synonymous
88168	NTHI477_RS00375	1282	C	A	nonsynonymous
88169	NTHI477_RS00375	1281	A	T	synonymous
88178	NTHI477_RS00375	1272	A	G	synonymous
88180	NTHI477_RS00375	1270	C	T	nonsynonymous
88181	NTHI477_RS00375	1269	G	A	synonymous
88184	NTHI477_RS00375	1266	T	C	synonymous
88193	NTHI477_RS00375	1257	A	G	synonymous
88195	NTHI477_RS00375	1255	C	A	nonsynonymous
88198	NTHI477_RS00375	1252	C	G	nonsynonymous
88220	NTHI477_RS00375	1230	G	T	synonymous
88247	NTHI477_RS00375	1203	G	A	synonymous
88249	NTHI477_RS00375	1201	C	T	nonsynonymous
88250	NTHI477_RS00375	1200	G	A	synonymous
88256	NTHI477_RS00375	1194	A	G	synonymous
88280	NTHI477_RS00375	1170	T	C	synonymous
88286	NTHI477_RS00375	1164	T	C	nonsynonymous
88288	NTHI477_RS00375	1162	T	C	nonsynonymous
88289	NTHI477_RS00375	1161	C	T	synonymous

88295	NTHI477_RS00375	1155	G	A	synonymous
88304	NTHI477_RS00375	1146	G	A	synonymous
88316	NTHI477_RS00375	1134	T	C	synonymous
88322	NTHI477_RS00375	1128	A	G	synonymous
88355	NTHI477_RS00375	1095	T	C	synonymous
88361	NTHI477_RS00375	1089	A	C	synonymous
88369	NTHI477_RS00375	1081	T	A	nonsynonymous
88370	NTHI477_RS00375	1080	G	A	synonymous
88402	NTHI477_RS00375	1048	A	G	synonymous
88430	NTHI477_RS00375	1020	G	T	synonymous
88451	NTHI477_RS00375	999	G	T	synonymous
88454	NTHI477_RS00375	996	A	G	synonymous
88459	NTHI477_RS00375	991	T	C	nonsynonymous
88496	NTHI477_RS00375	954	A	T	synonymous
88502	NTHI477_RS00375	948	T	G	synonymous
88502	NTHI477_RS00375	948	T	A	synonymous
88511	NTHI477_RS00375	939	G	A	synonymous
88523	NTHI477_RS00375	927	G	A	synonymous
88535	NTHI477_RS00375	915	C	T	synonymous
88544	NTHI477_RS00375	906	C	T	synonymous
88547	NTHI477_RS00375	903	G	A	synonymous
88559	NTHI477_RS00375	891	G	T	synonymous
88562	NTHI477_RS00375	888	T	A	nonsynonymous
88574	NTHI477_RS00375	876	A	G	synonymous
88582	NTHI477_RS00375	868	C	A	nonsynonymous
88583	NTHI477_RS00375	867	T	C	synonymous
88595	NTHI477_RS00375	855	C	T	synonymous
88609	NTHI477_RS00375	841	T	C	nonsynonymous
88613	NTHI477_RS00375	837	C	T	synonymous
88619	NTHI477_RS00375	831	T	C	synonymous
88622	NTHI477_RS00375	828	A	G	synonymous
88642	NTHI477_RS00375	808	C	T	nonsynonymous
88646	NTHI477_RS00375	804	G	A	synonymous
88652	NTHI477_RS00375	798	T	C	synonymous
88653	NTHI477_RS00375	797	G	A	nonsynonymous
88664	NTHI477_RS00375	786	A	G	synonymous
88679	NTHI477_RS00375	771	G	A	synonymous
88685	NTHI477_RS00375	765	T	C	synonymous
88706	NTHI477_RS00375	744	C	G	synonymous
88709	NTHI477_RS00375	741	G	A	synonymous
88712	NTHI477_RS00375	738	T	C	synonymous
88719	NTHI477_RS00375	731	T	G	nonsynonymous
88720	NTHI477_RS00375	730	T	C	nonsynonymous
88727	NTHI477_RS00375	723	G	A	synonymous
88729	NTHI477_RS00375	721	G	A	nonsynonymous

88748	NTHI477_RS00375	702	G	A	synonymous
88774	NTHI477_RS00375	676	T	C	nonsynonymous
88778	NTHI477_RS00375	672	A	G	synonymous
88790	NTHI477_RS00375	660	C	T	synonymous
88795	NTHI477_RS00375	655	T	C	nonsynonymous
88805	NTHI477_RS00375	645	T	C	synonymous
88808	NTHI477_RS00375	642	A	G	synonymous
88811	NTHI477_RS00375	639	A	G	synonymous
88820	NTHI477_RS00375	630	T	C	synonymous
88829	NTHI477_RS00375	621	T	A	synonymous
88837	NTHI477_RS00375	613	C	T	nonsynonymous
88853	NTHI477_RS00375	597	A	G	synonymous
88862	NTHI477_RS00375	588	G	A	synonymous
88871	NTHI477_RS00375	579	A	G	synonymous
88892	NTHI477_RS00375	558	C	T	synonymous
88905	NTHI477_RS00375	545	C	T	nonsynonymous
88916	NTHI477_RS00375	534	A	G	synonymous
88918	NTHI477_RS00375	532	T	G	nonsynonymous
88985	NTHI477_RS00375	465	G	T	synonymous
88988	NTHI477_RS00375	462	G	A	synonymous
89021	NTHI477_RS00375	429	G	A	synonymous
89023	NTHI477_RS00375	427	C	T	nonsynonymous
89027	NTHI477_RS00375	423	A	T	synonymous
89042	NTHI477_RS00375	408	C	T	synonymous
89087	NTHI477_RS00375	363	A	C	synonymous
89090	NTHI477_RS00375	360	T	G	synonymous
89123	NTHI477_RS00375	327	A	G	synonymous
89153	NTHI477_RS00375	297	T	A	synonymous
89201	NTHI477_RS00375	249	G	A	synonymous
89207	NTHI477_RS00375	243	A	G	synonymous
89213	NTHI477_RS00375	237	A	G	synonymous
89219	NTHI477_RS00375	231	G	A	synonymous
89253	NTHI477_RS00375	197	A	G	nonsynonymous
89254	NTHI477_RS00375	196	G	C	nonsynonymous
89309	NTHI477_RS00375	141	C	T	synonymous
89321	NTHI477_RS00375	129	A	G	synonymous
89324	NTHI477_RS00375	126	A	G	synonymous
89354	NTHI477_RS00375	96	T	G	synonymous
89368	NTHI477_RS00375	82	T	C	nonsynonymous
89378	NTHI477_RS00375	72	G	A	synonymous
89379	NTHI477_RS00375	71	C	T	nonsynonymous
89392	NTHI477_RS00375	58	C	A	nonsynonymous
89393	NTHI477_RS00375	57	A	G	synonymous
89401	NTHI477_RS00375	49	G	T	nonsynonymous
89404	NTHI477_RS00375	46	T	C	nonsynonymous

89408	NTHI477_RS00375	42	C	T	synonymous
89533	NTHI477_RS00380	5	C	T	nonsynonymous
89552	NTHI477_RS00380	24	A	G	synonymous
89578	NTHI477_RS00380	50	T	C	nonsynonymous
89617	NTHI477_RS00380	89	C	T	nonsynonymous
89632	NTHI477_RS00380	104	A	T	nonsynonymous
89642	NTHI477_RS00380	114	T	C	synonymous
89663	NTHI477_RS00380	135	A	C	synonymous
89677	NTHI477_RS00380	149	G	C	nonsynonymous
89822	NTHI477_RS00380	294	T	G	synonymous
89849	NTHI477_RS00380	321	G	A	synonymous
89852	NTHI477_RS00380	324	C	A	synonymous
89852	NTHI477_RS00380	324	C	T	synonymous
89874	NTHI477_RS00380	346	A	G	nonsynonymous
89891	NTHI477_RS00380	363	A	T	nonsynonymous
89910	NTHI477_RS00380	382	A	G	nonsynonymous
89912	NTHI477_RS00380	384	T	A	synonymous
89913	NTHI477_RS00380	385	A	C	nonsynonymous
89913	NTHI477_RS00380	385	A	G	nonsynonymous
89915	NTHI477_RS00380	387	C	T	synonymous
89915	NTHI477_RS00380	387	C	G	nonsynonymous
89919	NTHI477_RS00380	391	G	A	nonsynonymous
89921	NTHI477_RS00380	393	T	C	synonymous
89925	NTHI477_RS00380	397	A	G	nonsynonymous
89928	NTHI477_RS00380	400	T	A	nonsynonymous
89929	NTHI477_RS00380	401	A	G	nonsynonymous
89936	NTHI477_RS00380	408	A	G	synonymous
89954	NTHI477_RS00380	426	A	C	nonsynonymous
89981	NTHI477_RS00380	453	A	G	synonymous
89983	NTHI477_RS00380	455	T	C	nonsynonymous
90028	NTHI477_RS00380	500	C	T	nonsynonymous
90036	NTHI477_RS00380	508	A	G	nonsynonymous
90077	NTHI477_RS00380	549	A	T	synonymous
90146	intergenic	intergenic	A	G	intergenic
90155	NTHI477_RS00385	8	C	A	nonsynonymous
90158	NTHI477_RS00385	11	T	G	nonsynonymous
90163	NTHI477_RS00385	16	G	T	nonsynonymous
90174	NTHI477_RS00385	27	G	A	synonymous
90176	NTHI477_RS00385	29	A	G	nonsynonymous
90177	NTHI477_RS00385	30	G	T	nonsynonymous
90180	NTHI477_RS00385	33	G	A	synonymous
90181	NTHI477_RS00385	34	G	A	nonsynonymous
90182	NTHI477_RS00385	35	G	A	nonsynonymous
90183	NTHI477_RS00385	36	T	G	synonymous
90184	NTHI477_RS00385	37	A	G	nonsynonymous

90201	NTHI477_RS00385	54	T	C	synonymous
90202	NTHI477_RS00385	55	A	G	nonsynonymous
90210	NTHI477_RS00385	63	T	C	synonymous
90223	NTHI477_RS00385	76	A	G	nonsynonymous
90231	NTHI477_RS00385	84	A	T	synonymous
90240	NTHI477_RS00385	93	T	A	synonymous
90241	NTHI477_RS00385	94	G	A	nonsynonymous
90241	NTHI477_RS00385	94	G	T	nonsynonymous
90263	NTHI477_RS00385	116	G	A	nonsynonymous
90280	NTHI477_RS00385	133	G	T	nonsynonymous
90285	NTHI477_RS00385	138	A	G	synonymous
90289	NTHI477_RS00385	142	G	A	nonsynonymous
90308	NTHI477_RS00385	161	A	G	nonsynonymous
90316	NTHI477_RS00385	169	T	C	synonymous
90318	NTHI477_RS00385	171	A	T	nonsynonymous
90320	NTHI477_RS00385	173	T	C	nonsynonymous
90324	NTHI477_RS00385	177	T	C	synonymous
90327	NTHI477_RS00385	180	T	G	synonymous
90336	NTHI477_RS00385	189	T	G	synonymous
90337	NTHI477_RS00385	190	T	A	nonsynonymous
90338	NTHI477_RS00385	191	T	C	nonsynonymous
90339	NTHI477_RS00385	192	A	G	synonymous
90342	NTHI477_RS00385	195	A	G	synonymous
90346	NTHI477_RS00385	199	G	A	nonsynonymous
90348	NTHI477_RS00385	201	C	T	synonymous
90352	NTHI477_RS00385	205	G	A	nonsynonymous
90354	NTHI477_RS00385	207	T	C	synonymous
90355	NTHI477_RS00385	208	A	C	nonsynonymous
90359	NTHI477_RS00385	212	C	G	nonsynonymous
90364	NTHI477_RS00385	217	A	G	nonsynonymous
90366	NTHI477_RS00385	219	C	T	synonymous
90373	NTHI477_RS00385	226	A	T	nonsynonymous
90375	NTHI477_RS00385	228	T	A	synonymous
90381	NTHI477_RS00385	234	T	G	synonymous
90390	NTHI477_RS00385	243	C	T	synonymous
90394	NTHI477_RS00385	247	C	T	nonsynonymous
90407	intergenic	intergenic	T	A	intergenic
90410	intergenic	intergenic	T	C	intergenic
90417	intergenic	intergenic	A	G	intergenic
90422	intergenic	intergenic	T	G	intergenic
90433	NTHI477_RS00390	9	A	G	synonymous
90439	NTHI477_RS00390	15	T	C	synonymous
90442	NTHI477_RS00390	18	G	A	nonsynonymous
90450	NTHI477_RS00390	26	C	T	nonsynonymous
90451	NTHI477_RS00390	27	A	T	synonymous

90452	NTHI477_RS00390	28	C	A	nonsynonymous
90454	NTHI477_RS00390	30	G	C	synonymous
90458	NTHI477_RS00390	34	G	A	nonsynonymous
90459	NTHI477_RS00390	35	T	C	nonsynonymous
90461	NTHI477_RS00390	37	G	A	nonsynonymous
90462	NTHI477_RS00390	38	C	T	nonsynonymous
90472	NTHI477_RS00390	48	A	G	synonymous
90473	NTHI477_RS00390	49	A	G	nonsynonymous
90478	NTHI477_RS00390	54	T	A	synonymous
90482	NTHI477_RS00390	58	G	A	nonsynonymous
90484	NTHI477_RS00390	60	A	G	synonymous
90490	NTHI477_RS00390	66	T	A	synonymous
90499	NTHI477_RS00390	75	C	T	synonymous
90502	NTHI477_RS00390	78	C	T	synonymous
90505	NTHI477_RS00390	81	T	C	synonymous
90511	NTHI477_RS00390	87	T	G	synonymous
90517	NTHI477_RS00390	93	C	T	synonymous
90529	NTHI477_RS00390	105	A	T	synonymous
90532	NTHI477_RS00390	108	G	A	synonymous
90538	NTHI477_RS00390	114	G	T	synonymous
90571	NTHI477_RS00390	147	A	G	synonymous
90574	NTHI477_RS00390	150	C	T	synonymous
90583	NTHI477_RS00390	159	A	G	synonymous
90586	NTHI477_RS00390	162	T	C	synonymous
90590	NTHI477_RS00390	166	G	A	nonsynonymous
90604	NTHI477_RS00390	180	T	C	synonymous
90608	NTHI477_RS00390	184	G	A	nonsynonymous
90610	NTHI477_RS00390	186	A	T	synonymous
90616	NTHI477_RS00390	192	G	T	synonymous
90625	NTHI477_RS00390	201	T	C	synonymous
90628	NTHI477_RS00390	204	A	T	synonymous
90631	NTHI477_RS00390	207	T	G	synonymous
90644	NTHI477_RS00390	220	G	A	nonsynonymous
90646	NTHI477_RS00390	222	T	G	synonymous
90653	NTHI477_RS00390	229	A	G	nonsynonymous
90678	NTHI477_RS00390	254	C	T	nonsynonymous
90682	NTHI477_RS00390	258	T	C	synonymous
90685	NTHI477_RS00390	261	A	T	synonymous
90697	NTHI477_RS00390	273	T	C	synonymous
90700	NTHI477_RS00390	276	A	G	synonymous
90706	NTHI477_RS00390	282	C	T	synonymous
90713	NTHI477_RS00390	289	A	G	nonsynonymous
90724	NTHI477_RS00390	300	T	C	synonymous
90730	NTHI477_RS00390	306	C	T	synonymous
90736	NTHI477_RS00390	312	A	G	synonymous

90740	NTHI477_RS00390	316	A	G	nonsynonymous
90742	NTHI477_RS00390	318	G	A	nonsynonymous
90775	NTHI477_RS00390	351	G	A	synonymous
90817	NTHI477_RS00390	393	C	T	synonymous
90822	NTHI477_RS00390	398	G	T	nonsynonymous
90859	NTHI477_RS00390	435	T	C	synonymous
90865	NTHI477_RS00390	441	A	G	synonymous
90867	NTHI477_RS00390	443	G	A	nonsynonymous
90891	intergenic	intergenic	C	T	intergenic
90925	intergenic	intergenic	T	C	intergenic
90940	intergenic	intergenic	A	G	intergenic
90962	intergenic	intergenic	T	C	intergenic
90987	intergenic	intergenic	C	T	intergenic
91017	intergenic	intergenic	A	C	intergenic
91018	intergenic	intergenic	G	T	intergenic
91025	intergenic	intergenic	G	C	intergenic
91040	intergenic	intergenic	C	T	intergenic
91073	intergenic	intergenic	T	C	intergenic
91082	NTHI477_RS00395	966	T	C	nonsynonymous
91094	NTHI477_RS00395	954	T	A	nonsynonymous
91100	NTHI477_RS00395	948	T	G	synonymous
91101	NTHI477_RS00395	947	A	C	nonsynonymous
91103	NTHI477_RS00395	945	G	A	synonymous
91106	NTHI477_RS00395	942	C	T	synonymous
91120	NTHI477_RS00395	928	T	G	nonsynonymous
91121	NTHI477_RS00395	927	T	A	nonsynonymous
91130	NTHI477_RS00395	918	C	A	nonsynonymous
91131	NTHI477_RS00395	917	T	G	nonsynonymous
91145	NTHI477_RS00395	903	T	A	nonsynonymous
91147	NTHI477_RS00395	901	C	T	nonsynonymous
91166	NTHI477_RS00395	882	A	T	nonsynonymous
91172	NTHI477_RS00395	876	G	T	synonymous
91211	NTHI477_RS00395	837	C	A	nonsynonymous
91271	NTHI477_RS00395	777	C	T	synonymous
91289	NTHI477_RS00395	759	T	G	nonsynonymous
91323	NTHI477_RS00395	725	G	A	nonsynonymous
91337	NTHI477_RS00395	711	C	A	nonsynonymous
91357	NTHI477_RS00395	691	C	T	nonsynonymous
91375	NTHI477_RS00395	673	A	G	nonsynonymous
91376	NTHI477_RS00395	672	A	C	synonymous
91387	NTHI477_RS00395	661	T	C	nonsynonymous
91411	NTHI477_RS00395	637	T	G	nonsynonymous
91415	NTHI477_RS00395	633	C	T	synonymous
91486	NTHI477_RS00395	562	A	G	nonsynonymous
91550	NTHI477_RS00395	498	A	G	synonymous

91559	NTHI477_RS00395	489	T	C	nonsynonymous
91592	NTHI477_RS00395	456	C	A	nonsynonymous
91603	NTHI477_RS00395	445	T	G	nonsynonymous
91625	NTHI477_RS00395	423	T	A	synonymous
91639	NTHI477_RS00395	409	G	T	nonsynonymous
91652	NTHI477_RS00395	396	T	G	synonymous
91662	NTHI477_RS00395	386	C	T	nonsynonymous
91718	NTHI477_RS00395	330	T	C	synonymous
91720	NTHI477_RS00395	328	G	A	synonymous
91778	NTHI477_RS00395	270	C	T	synonymous
91783	NTHI477_RS00395	265	T	A	nonsynonymous
91804	NTHI477_RS00395	244	C	T	nonsynonymous
91808	NTHI477_RS00395	240	A	G	synonymous
91839	NTHI477_RS00395	209	T	C	nonsynonymous
91853	NTHI477_RS00395	195	G	A	synonymous
91874	NTHI477_RS00395	174	T	G	nonsynonymous
91888	NTHI477_RS00395	160	T	C	nonsynonymous
91951	NTHI477_RS00395	97	C	T	nonsynonymous
91955	NTHI477_RS00395	93	A	G	synonymous
91956	NTHI477_RS00395	92	C	G	nonsynonymous
92036	NTHI477_RS00395	12	A	G	synonymous
92037	NTHI477_RS00395	11	G	A	nonsynonymous
92038	NTHI477_RS00395	10	G	A	nonsynonymous
92053	intergenic	intergenic	C	A	intergenic
92056	intergenic	intergenic	C	T	intergenic
92059	intergenic	intergenic	T	G	intergenic
92061	intergenic	intergenic	T	C	intergenic
92061	intergenic	intergenic	T	G	intergenic
92071	intergenic	intergenic	A	C	intergenic
92078	intergenic	intergenic	T	C	intergenic
92080	intergenic	intergenic	A	G	intergenic
92102	intergenic	intergenic	C	T	intergenic
92110	intergenic	intergenic	T	G	intergenic
92126	intergenic	intergenic	G	A	intergenic
92154	NTHI477_RS09085	471	A	G	synonymous
92155	NTHI477_RS09085	470	C	A	nonsynonymous
92167	NTHI477_RS09085	458	G	A	nonsynonymous
92184	NTHI477_RS09085	441	G	A	synonymous
92215	NTHI477_RS09085	410	A	T	nonsynonymous
92258	NTHI477_RS09085	367	C	T	nonsynonymous
92318	NTHI477_RS09085	307	C	A	nonsynonymous
92344	NTHI477_RS09085	281	C	T	nonsynonymous
92368	NTHI477_RS09085	257	C	G	nonsynonymous
92385	NTHI477_RS09085	240	C	A	nonsynonymous
92404	NTHI477_RS09085	221	T	G	nonsynonymous

92418	NTHI477_RS09085	207	C	T	synonymous
92423	NTHI477_RS09085	202	T	A	nonsynonymous
92448	NTHI477_RS09085	177	T	C	synonymous
92451	NTHI477_RS09085	174	C	T	synonymous
92460	NTHI477_RS09085	165	C	T	nonsynonymous
92461	NTHI477_RS09085	164	C	T	nonsynonymous
92463	NTHI477_RS09085	162	T	A	synonymous
92488	NTHI477_RS09085	137	T	C	nonsynonymous
92495	NTHI477_RS09085	130	A	C	nonsynonymous
92516	NTHI477_RS09085	109	G	T	nonsynonymous
92544	NTHI477_RS09085	81	C	T	synonymous
92547	NTHI477_RS09085	78	A	G	synonymous
92564	NTHI477_RS09085	61	T	G	nonsynonymous
92567	NTHI477_RS09085	58	C	T	nonsynonymous
92595	NTHI477_RS09085	30	C	T	synonymous
92646	intergenic	intergenic	A	G	intergenic
92663	intergenic	intergenic	T	C	intergenic
92669	intergenic	intergenic	T	C	intergenic
92691	intergenic	intergenic	C	T	intergenic
92709	NTHI477_RS00410	1639	C	T	nonsynonymous
92722	NTHI477_RS00410	1626	A	G	synonymous
92728	NTHI477_RS00410	1620	T	C	synonymous
92734	NTHI477_RS00410	1614	T	G	synonymous
92740	NTHI477_RS00410	1608	G	A	synonymous
92743	NTHI477_RS00410	1605	G	A	synonymous
92746	NTHI477_RS00410	1602	A	G	synonymous
92749	NTHI477_RS00410	1599	A	G	synonymous
92755	NTHI477_RS00410	1593	T	G	synonymous
92770	NTHI477_RS00410	1578	T	G	synonymous
92791	NTHI477_RS00410	1557	G	A	synonymous
92812	NTHI477_RS00410	1536	T	C	synonymous
92815	NTHI477_RS00410	1533	G	T	synonymous
92818	NTHI477_RS00410	1530	A	G	synonymous
92866	NTHI477_RS00410	1482	T	G	synonymous
92872	NTHI477_RS00410	1476	G	A	synonymous
92872	NTHI477_RS00410	1476	G	T	nonsynonymous
92884	NTHI477_RS00410	1464	C	A	synonymous
92896	NTHI477_RS00410	1452	C	A	synonymous
92899	NTHI477_RS00410	1449	T	C	synonymous
92902	NTHI477_RS00410	1446	A	G	synonymous
92903	NTHI477_RS00410	1445	G	A	nonsynonymous
92932	NTHI477_RS00410	1416	C	G	synonymous
92932	NTHI477_RS00410	1416	C	A	synonymous
92974	NTHI477_RS00410	1374	A	G	synonymous
92983	NTHI477_RS00410	1365	T	C	synonymous

93001	NTHI477_RS00410	1347	C	A	synonymous
93012	NTHI477_RS00410	1336	C	T	nonsynonymous
93079	NTHI477_RS00410	1269	T	G	synonymous
93103	NTHI477_RS00410	1245	T	C	synonymous
93105	NTHI477_RS00410	1243	C	A	nonsynonymous
93112	NTHI477_RS00410	1236	G	A	synonymous
93115	NTHI477_RS00410	1233	A	G	synonymous
93119	NTHI477_RS00410	1229	C	G	nonsynonymous
93124	NTHI477_RS00410	1224	A	G	synonymous
93127	NTHI477_RS00410	1221	G	A	synonymous
93127	NTHI477_RS00410	1221	G	T	synonymous
93157	NTHI477_RS00410	1191	A	G	synonymous
93160	NTHI477_RS00410	1188	G	A	synonymous
93166	NTHI477_RS00410	1182	A	G	synonymous
93187	NTHI477_RS00410	1161	G	A	synonymous
93196	NTHI477_RS00410	1152	G	A	synonymous
93205	NTHI477_RS00410	1143	A	T	synonymous
93220	NTHI477_RS00410	1128	G	A	synonymous
93223	NTHI477_RS00410	1125	A	C	synonymous
93229	NTHI477_RS00410	1119	A	C	synonymous
93235	NTHI477_RS00410	1113	A	G	synonymous
93238	NTHI477_RS00410	1110	A	G	synonymous
93241	NTHI477_RS00410	1107	G	A	synonymous
93244	NTHI477_RS00410	1104	A	G	synonymous
93247	NTHI477_RS00410	1101	G	A	synonymous
93249	NTHI477_RS00410	1099	C	T	nonsynonymous
93253	NTHI477_RS00410	1095	G	A	synonymous
93265	NTHI477_RS00410	1083	C	G	synonymous
93265	NTHI477_RS00410	1083	C	T	synonymous
93274	NTHI477_RS00410	1074	A	C	synonymous
93301	NTHI477_RS00410	1047	A	G	synonymous
93307	NTHI477_RS00410	1041	A	G	synonymous
93310	NTHI477_RS00410	1038	T	C	synonymous
93313	NTHI477_RS00410	1035	G	A	synonymous
93346	NTHI477_RS00410	1002	C	T	synonymous
93358	NTHI477_RS00410	990	C	G	synonymous
93358	NTHI477_RS00410	990	C	T	synonymous
93367	NTHI477_RS00410	981	A	G	synonymous
93373	NTHI477_RS00410	975	G	A	synonymous
93394	NTHI477_RS00410	954	T	G	synonymous
93400	NTHI477_RS00410	948	A	G	synonymous
93403	NTHI477_RS00410	945	G	A	synonymous
93406	NTHI477_RS00410	942	T	G	synonymous
93409	NTHI477_RS00410	939	G	A	synonymous
93417	NTHI477_RS00410	931	G	T	nonsynonymous

93421	NTHI477_RS00410	927	G	A	synonymous
93429	NTHI477_RS00410	919	T	C	nonsynonymous
93432	NTHI477_RS00410	916	A	T	nonsynonymous
93444	NTHI477_RS00410	904	C	T	nonsynonymous
93457	NTHI477_RS00410	891	C	T	synonymous
93460	NTHI477_RS00410	888	G	A	synonymous
93464	NTHI477_RS00410	884	T	C	nonsynonymous
93473	NTHI477_RS00410	875	G	T	nonsynonymous
93478	NTHI477_RS00410	870	A	G	synonymous
93496	NTHI477_RS00410	852	T	G	synonymous
93502	NTHI477_RS00410	846	C	T	synonymous
93520	NTHI477_RS00410	828	G	A	synonymous
93523	NTHI477_RS00410	825	T	A	synonymous
93535	NTHI477_RS00410	813	A	G	synonymous
93571	NTHI477_RS00410	777	G	A	synonymous
93586	NTHI477_RS00410	762	G	A	synonymous
93643	NTHI477_RS00410	705	C	T	synonymous
93654	NTHI477_RS00410	694	C	T	nonsynonymous
93661	NTHI477_RS00410	687	C	T	synonymous
93667	NTHI477_RS00410	681	C	T	synonymous
93682	NTHI477_RS00410	666	C	T	synonymous
93685	NTHI477_RS00410	663	A	G	synonymous
93691	NTHI477_RS00410	657	C	T	synonymous
93712	NTHI477_RS00410	636	A	T	synonymous
93715	NTHI477_RS00410	633	T	G	synonymous
93721	NTHI477_RS00410	627	A	G	synonymous
93721	NTHI477_RS00410	627	A	C	synonymous
93733	NTHI477_RS00410	615	T	C	synonymous
93745	NTHI477_RS00410	603	C	T	synonymous
93745	NTHI477_RS00410	603	C	A	synonymous
93748	NTHI477_RS00410	600	C	T	synonymous
93748	NTHI477_RS00410	600	C	G	synonymous
93775	NTHI477_RS00410	573	T	C	synonymous
93778	NTHI477_RS00410	570	T	C	synonymous
93784	NTHI477_RS00410	564	G	A	synonymous
93822	NTHI477_RS00410	526	G	A	synonymous
93823	NTHI477_RS00410	525	A	G	synonymous
93835	NTHI477_RS00410	513	C	T	synonymous
93837	NTHI477_RS00410	511	G	A	nonsynonymous
93844	NTHI477_RS00410	504	T	C	synonymous
93862	NTHI477_RS00410	486	A	C	synonymous
93865	NTHI477_RS00410	483	C	G	synonymous
93865	NTHI477_RS00410	483	C	A	synonymous
93871	NTHI477_RS00410	477	G	A	synonymous
93895	NTHI477_RS00410	453	C	T	synonymous

93898	NTHI477_RS00410	450	T	A	synonymous
93904	NTHI477_RS00410	444	C	A	synonymous
93905	NTHI477_RS00410	443	G	C	nonsynonymous
93910	NTHI477_RS00410	438	G	T	synonymous
93910	NTHI477_RS00410	438	G	C	synonymous
93916	NTHI477_RS00410	432	G	A	synonymous
93919	NTHI477_RS00410	429	T	C	synonymous
93937	NTHI477_RS00410	411	G	A	synonymous
93958	NTHI477_RS00410	390	G	A	synonymous
93978	NTHI477_RS00410	370	G	A	synonymous
93984	NTHI477_RS00410	364	T	C	nonsynonymous
94000	NTHI477_RS00410	348	G	A	synonymous
94009	NTHI477_RS00410	339	G	A	synonymous
94015	NTHI477_RS00410	333	A	T	synonymous
94027	NTHI477_RS00410	321	C	A	synonymous
94035	NTHI477_RS00410	313	T	C	nonsynonymous
94036	NTHI477_RS00410	312	G	A	synonymous
94045	NTHI477_RS00410	303	A	T	synonymous
94045	NTHI477_RS00410	303	A	G	synonymous
94051	NTHI477_RS00410	297	A	G	synonymous
94060	NTHI477_RS00410	288	C	T	synonymous
94063	NTHI477_RS00410	285	G	A	synonymous
94066	NTHI477_RS00410	282	A	C	synonymous
94075	NTHI477_RS00410	273	T	A	synonymous
94078	NTHI477_RS00410	270	A	G	synonymous
94084	NTHI477_RS00410	264	G	A	synonymous
94090	NTHI477_RS00410	258	T	A	nonsynonymous
94092	NTHI477_RS00410	256	C	T	nonsynonymous
94093	NTHI477_RS00410	255	G	A	synonymous
94096	NTHI477_RS00410	252	A	G	synonymous
94105	NTHI477_RS00410	243	C	A	synonymous
94105	NTHI477_RS00410	243	C	T	synonymous
94117	NTHI477_RS00410	231	G	A	synonymous
94126	NTHI477_RS00410	222	T	C	synonymous
94126	NTHI477_RS00410	222	T	A	synonymous
94128	NTHI477_RS00410	220	C	A	nonsynonymous
94154	NTHI477_RS00410	194	T	A	nonsynonymous
94162	NTHI477_RS00410	186	G	A	synonymous
94177	NTHI477_RS00410	171	G	A	synonymous
94180	NTHI477_RS00410	168	G	A	synonymous
94195	NTHI477_RS00410	153	G	T	synonymous
94219	NTHI477_RS00410	129	T	C	synonymous
94270	NTHI477_RS00410	78	G	A	synonymous
94273	NTHI477_RS00410	75	G	A	synonymous
94282	NTHI477_RS00410	66	C	T	synonymous

94303	NTHI477_RS00410	45	G	A	synonymous
94307	NTHI477_RS00410	41	G	A	nonsynonymous
94308	NTHI477_RS00410	40	A	C	nonsynonymous
94310	NTHI477_RS00410	38	T	C	nonsynonymous
94339	NTHI477_RS00410	9	A	G	synonymous
94348	NTHI477_RS00410	0	G	A	synonymous
94350	intergenic	intergenic	T	C	intergenic
94373	intergenic	intergenic	C	T	intergenic
94374	intergenic	intergenic	A	G	intergenic
94397	intergenic	intergenic	A	G	intergenic
94399	intergenic	intergenic	T	C	intergenic
94409	intergenic	intergenic	G	A	intergenic
94421	intergenic	intergenic	C	T	intergenic
94453	NTHI477_RS00415	1074	G	A	synonymous
94471	NTHI477_RS00415	1056	A	C	synonymous
94546	NTHI477_RS00415	981	G	A	synonymous
94554	NTHI477_RS00415	973	A	G	synonymous
94572	NTHI477_RS00415	955	C	T	nonsynonymous
94591	NTHI477_RS00415	936	G	A	synonymous
94606	NTHI477_RS00415	921	A	G	synonymous
94642	NTHI477_RS00415	885	A	G	synonymous
94648	NTHI477_RS00415	879	G	A	synonymous
94662	NTHI477_RS00415	865	C	T	nonsynonymous
94666	NTHI477_RS00415	861	T	C	synonymous
94678	NTHI477_RS00415	849	C	T	synonymous
94683	NTHI477_RS00415	844	C	T	nonsynonymous
94686	NTHI477_RS00415	841	G	T	nonsynonymous
94699	NTHI477_RS00415	828	G	A	synonymous
94708	NTHI477_RS00415	819	C	T	nonsynonymous
94729	NTHI477_RS00415	798	A	G	synonymous
94745	NTHI477_RS00415	782	G	A	nonsynonymous
94846	NTHI477_RS00415	681	A	G	synonymous
94849	NTHI477_RS00415	678	G	A	synonymous
94894	NTHI477_RS00415	633	G	A	synonymous
94903	NTHI477_RS00415	624	C	T	synonymous
94905	NTHI477_RS00415	622	G	A	synonymous
94906	NTHI477_RS00415	621	A	G	synonymous
94912	NTHI477_RS00415	615	T	G	nonsynonymous
94914	NTHI477_RS00415	613	C	T	nonsynonymous
94915	NTHI477_RS00415	612	A	T	synonymous
94976	NTHI477_RS00415	551	G	A	nonsynonymous
94977	NTHI477_RS00415	550	C	T	nonsynonymous
94999	NTHI477_RS00415	528	G	A	synonymous
95002	NTHI477_RS00415	525	C	T	synonymous
95005	NTHI477_RS00415	522	A	G	synonymous

95008	NTHI477_RS00415	519	G	A	synonymous
95029	NTHI477_RS00415	498	G	A	synonymous
95032	NTHI477_RS00415	495	T	A	synonymous
95035	NTHI477_RS00415	492	G	A	synonymous
95128	NTHI477_RS00415	399	C	T	synonymous
95131	NTHI477_RS00415	396	G	A	synonymous
95134	NTHI477_RS00415	393	A	G	synonymous
95164	NTHI477_RS00415	363	T	C	synonymous
95185	NTHI477_RS00415	342	C	T	synonymous
95187	NTHI477_RS00415	340	C	A	nonsynonymous
95200	NTHI477_RS00415	327	A	C	nonsynonymous
95218	NTHI477_RS00415	309	G	A	synonymous
95221	NTHI477_RS00415	306	T	C	synonymous
95272	NTHI477_RS00415	255	G	A	synonymous
95278	NTHI477_RS00415	249	A	G	synonymous
95287	NTHI477_RS00415	240	C	T	synonymous
95305	NTHI477_RS00415	222	A	G	synonymous
95314	NTHI477_RS00415	213	G	T	synonymous
95326	NTHI477_RS00415	201	T	C	synonymous
95344	NTHI477_RS00415	183	C	A	synonymous
95347	NTHI477_RS00415	180	C	T	synonymous
95350	NTHI477_RS00415	177	G	C	synonymous
95362	NTHI477_RS00415	165	A	G	synonymous
95365	NTHI477_RS00415	162	A	G	synonymous
95366	NTHI477_RS00415	161	T	C	nonsynonymous
95377	NTHI477_RS00415	150	G	A	synonymous
95383	NTHI477_RS00415	144	A	T	synonymous
95389	NTHI477_RS00415	138	G	A	synonymous
95392	NTHI477_RS00415	135	T	C	synonymous
95410	NTHI477_RS00415	117	G	A	synonymous
95413	NTHI477_RS00415	114	G	A	synonymous
95425	NTHI477_RS00415	102	C	T	synonymous
95428	NTHI477_RS00415	99	A	C	synonymous
95443	NTHI477_RS00415	84	A	T	nonsynonymous
95447	NTHI477_RS00415	80	G	C	nonsynonymous
95457	NTHI477_RS00415	70	G	T	nonsynonymous
95476	NTHI477_RS00415	51	C	T	synonymous
95478	NTHI477_RS00415	49	G	T	nonsynonymous
95494	NTHI477_RS00415	33	A	G	synonymous
95497	NTHI477_RS00415	30	A	G	synonymous
95566	NTHI477_RS00420	1377	G	A	synonymous
95569	NTHI477_RS00420	1374	G	A	synonymous
95578	NTHI477_RS00420	1365	G	A	synonymous
95587	NTHI477_RS00420	1356	A	G	synonymous
95626	NTHI477_RS00420	1317	G	A	synonymous

95638	NTHI477_RS00420	1305	T	C	synonymous
95644	NTHI477_RS00420	1299	G	A	synonymous
95662	NTHI477_RS00420	1281	A	G	synonymous
95689	NTHI477_RS00420	1254	G	C	synonymous
95695	NTHI477_RS00420	1248	G	A	synonymous
95698	NTHI477_RS00420	1245	T	A	synonymous
95701	NTHI477_RS00420	1242	G	A	synonymous
95737	NTHI477_RS00420	1206	A	G	synonymous
95743	NTHI477_RS00420	1200	T	A	synonymous
95761	NTHI477_RS00420	1182	G	A	synonymous
95780	NTHI477_RS00420	1163	G	C	nonsynonymous
95788	NTHI477_RS00420	1155	C	T	synonymous
95794	NTHI477_RS00420	1149	A	C	synonymous
95803	NTHI477_RS00420	1140	T	C	synonymous
95806	NTHI477_RS00420	1137	C	T	synonymous
95818	NTHI477_RS00420	1125	G	A	synonymous
95860	NTHI477_RS00420	1083	G	A	synonymous
95883	NTHI477_RS00420	1060	G	A	synonymous
95905	NTHI477_RS00420	1038	T	G	synonymous
95911	NTHI477_RS00420	1032	G	A	synonymous
95980	NTHI477_RS00420	963	C	G	synonymous
95983	NTHI477_RS00420	960	G	C	synonymous
96007	NTHI477_RS00420	936	A	G	synonymous
96016	NTHI477_RS00420	927	A	G	synonymous
96026	NTHI477_RS00420	917	A	T	nonsynonymous
96055	NTHI477_RS00420	888	C	T	synonymous
96064	NTHI477_RS00420	879	G	A	synonymous
96073	NTHI477_RS00420	870	G	A	synonymous
96085	NTHI477_RS00420	858	T	C	synonymous
96100	NTHI477_RS00420	843	C	A	synonymous
96106	NTHI477_RS00420	837	A	T	synonymous
96136	NTHI477_RS00420	807	G	C	synonymous
96139	NTHI477_RS00420	804	A	T	synonymous
96169	NTHI477_RS00420	774	T	A	synonymous
96247	NTHI477_RS00420	696	G	A	synonymous
96271	NTHI477_RS00420	672	T	C	synonymous
96289	NTHI477_RS00420	654	A	T	synonymous
96295	NTHI477_RS00420	648	T	C	synonymous
96304	NTHI477_RS00420	639	A	T	synonymous
96314	NTHI477_RS00420	629	T	C	nonsynonymous
96325	NTHI477_RS00420	618	G	A	synonymous
96328	NTHI477_RS00420	615	A	G	synonymous
96331	NTHI477_RS00420	612	G	A	synonymous
96334	NTHI477_RS00420	609	C	T	synonymous
96342	NTHI477_RS00420	601	C	T	nonsynonymous

96343	NTHI477_RS00420	600	A	G	synonymous
96346	NTHI477_RS00420	597	T	C	synonymous
96406	NTHI477_RS00420	537	T	C	synonymous
96436	NTHI477_RS00420	507	G	A	synonymous
96445	NTHI477_RS00420	498	C	T	synonymous
96448	NTHI477_RS00420	495	A	T	synonymous
96448	NTHI477_RS00420	495	A	C	synonymous
96484	NTHI477_RS00420	459	C	T	synonymous
96547	NTHI477_RS00420	396	T	C	synonymous
96574	NTHI477_RS00420	369	G	A	synonymous
96583	NTHI477_RS00420	360	G	A	synonymous
96589	NTHI477_RS00420	354	G	A	synonymous
96616	NTHI477_RS00420	327	G	C	synonymous
96622	NTHI477_RS00420	321	C	T	synonymous
96634	NTHI477_RS00420	309	G	A	synonymous
96640	NTHI477_RS00420	303	T	C	synonymous
96676	NTHI477_RS00420	267	T	G	synonymous
96682	NTHI477_RS00420	261	A	G	synonymous
96724	NTHI477_RS00420	219	T	C	synonymous
96872	NTHI477_RS00420	71	A	G	nonsynonymous
96922	NTHI477_RS00420	21	A	G	synonymous
96968	intergenic	intergenic	C	A	intergenic
96996	intergenic	intergenic	A	G	intergenic
97016	intergenic	intergenic	G	A	intergenic
97017	intergenic	intergenic	A	G	intergenic
97080	intergenic	intergenic	T	C	intergenic
97150	NTHI477_RS00425	1371	C	T	synonymous
97213	NTHI477_RS00425	1308	G	T	synonymous
97213	NTHI477_RS00425	1308	G	A	synonymous
97225	NTHI477_RS00425	1296	A	G	synonymous
97234	NTHI477_RS00425	1287	G	A	synonymous
97364	NTHI477_RS00425	1157	C	G	nonsynonymous
97432	NTHI477_RS00425	1089	C	T	synonymous
97441	NTHI477_RS00425	1080	G	A	synonymous
97537	NTHI477_RS00425	984	A	T	synonymous
97552	NTHI477_RS00425	969	G	A	synonymous
97552	NTHI477_RS00425	969	G	T	synonymous
97630	NTHI477_RS00425	891	C	T	synonymous
97642	NTHI477_RS00425	879	G	A	synonymous
97645	NTHI477_RS00425	876	A	G	synonymous
97711	NTHI477_RS00425	810	A	G	synonymous
97759	NTHI477_RS00425	762	A	G	synonymous
97792	NTHI477_RS00425	729	G	A	synonymous
97806	NTHI477_RS00425	715	T	C	nonsynonymous
97822	NTHI477_RS00425	699	G	A	synonymous

97876	NTHI477_RS00425	645	A	C	synonymous
97945	NTHI477_RS00425	576	G	T	nonsynonymous
97960	NTHI477_RS00425	561	G	A	synonymous
97995	NTHI477_RS00425	526	C	G	nonsynonymous
98099	NTHI477_RS00425	422	G	A	nonsynonymous
98152	NTHI477_RS00425	369	C	A	synonymous
98155	NTHI477_RS00425	366	C	A	synonymous
98167	NTHI477_RS00425	354	A	T	synonymous
98224	NTHI477_RS00425	297	T	C	synonymous
98253	NTHI477_RS00425	268	C	T	nonsynonymous
98597	intergenic	intergenic	C	T	intergenic
98600	intergenic	intergenic	T	C	intergenic
98613	intergenic	intergenic	C	A	intergenic
98626	intergenic	intergenic	G	T	intergenic
98631	intergenic	intergenic	C	A	intergenic
98667	intergenic	intergenic	C	T	intergenic
98685	intergenic	intergenic	C	T	intergenic
98716	intergenic	intergenic	T	G	intergenic
98737	intergenic	intergenic	C	A	intergenic
98741	intergenic	intergenic	T	C	intergenic
98808	intergenic	intergenic	G	A	intergenic
98841	intergenic	intergenic	C	T	intergenic
98843	intergenic	intergenic	A	G	intergenic
98849	intergenic	intergenic	C	T	intergenic
98854	intergenic	intergenic	T	C	intergenic
98860	intergenic	intergenic	T	G	intergenic
98862	intergenic	intergenic	G	A	intergenic
98892	intergenic	intergenic	G	T	intergenic
98905	intergenic	intergenic	C	A	intergenic
98919	intergenic	intergenic	T	C	intergenic
98932	intergenic	intergenic	A	G	intergenic
98940	intergenic	intergenic	A	C	intergenic
98945	intergenic	intergenic	C	T	intergenic
98962	intergenic	intergenic	T	C	intergenic
99005	intergenic	intergenic	C	T	intergenic
99006	intergenic	intergenic	A	G	intergenic
99009	intergenic	intergenic	T	C	intergenic
99025	intergenic	intergenic	T	C	intergenic
99026	intergenic	intergenic	A	T	intergenic
99043	intergenic	intergenic	G	A	intergenic
99046	intergenic	intergenic	A	G	intergenic
99052	intergenic	intergenic	A	C	intergenic
99064	NTHI477_RS09470	12	T	C	synonymous
99087	NTHI477_RS09470	35	A	G	nonsynonymous
99099	NTHI477_RS09470	47	G	A	nonsynonymous

99121	NTHI477_RS09470	69	A	C	synonymous
99124	NTHI477_RS09470	72	T	C	synonymous
99181	NTHI477_RS09470	129	C	T	synonymous
99247	NTHI477_RS09470	195	A	C	nonsynonymous
99256	NTHI477_RS09470	204	T	C	synonymous
99259	NTHI477_RS09470	207	A	G	synonymous
99266	NTHI477_RS09470	214	C	T	nonsynonymous
99267	NTHI477_RS09470	215	A	G	nonsynonymous
99292	NTHI477_RS09470	240	A	G	synonymous
99296	NTHI477_RS09470	244	C	T	synonymous
99309	NTHI477_RS09470	257	G	A	nonsynonymous
99361	NTHI477_RS09470	309	T	C	synonymous
99389	intergenic	intergenic	G	A	intergenic
99398	intergenic	intergenic	G	A	intergenic
99412	intergenic	intergenic	T	C	intergenic
99441	intergenic	intergenic	C	T	intergenic
99471	NTHI477_RS09475	18	C	A	nonsynonymous
99472	NTHI477_RS09475	19	C	A	nonsynonymous
99473	NTHI477_RS09475	20	C	A	nonsynonymous
99474	NTHI477_RS09475	21	C	A	synonymous
99476	NTHI477_RS09475	23	C	T	nonsynonymous
99499	NTHI477_RS09475	46	A	C	nonsynonymous
99504	NTHI477_RS09475	51	A	G	synonymous
99506	NTHI477_RS09475	53	C	T	nonsynonymous
99510	NTHI477_RS09475	57	G	A	synonymous
99529	NTHI477_RS09475	76	A	G	nonsynonymous
99599	NTHI477_RS09475	146	C	A	nonsynonymous
99663	NTHI477_RS09475	210	A	C	nonsynonymous
99690	NTHI477_RS09475	237	C	T	synonymous
99690	NTHI477_RS09475	237	C	A	synonymous
99704	NTHI477_RS09475	251	G	A	nonsynonymous
99742	NTHI477_RS09475	289	A	C	nonsynonymous
99773	NTHI477_RS09475	320	C	A	nonsynonymous
99774	NTHI477_RS09475	321	G	T	synonymous
99815	NTHI477_RS09475	362	T	C	nonsynonymous
99823	NTHI477_RS09475	370	T	G	nonsynonymous
99878	NTHI477_RS09475	425	A	C	nonsynonymous
99879	NTHI477_RS09475	426	A	T	nonsynonymous
99891	NTHI477_RS09475	438	T	C	synonymous
99916	NTHI477_RS09475	463	C	T	nonsynonymous
100144	NTHI477_RS09475	691	A	C	nonsynonymous
100146	NTHI477_RS09475	693	G	A	synonymous
100148	NTHI477_RS09475	695	T	C	nonsynonymous
100148	NTHI477_RS09475	695	T	G	nonsynonymous
100155	intergenic	intergenic	G	A	intergenic

100162	intergenic	intergenic	G	A	intergenic
100163	intergenic	intergenic	A	C	intergenic
100164	intergenic	intergenic	G	T	intergenic
100164	intergenic	intergenic	G	C	intergenic
100171	intergenic	intergenic	A	C	intergenic
100173	intergenic	intergenic	G	T	intergenic
100174	intergenic	intergenic	G	T	intergenic
100174	intergenic	intergenic	G	A	intergenic
100204	intergenic	intergenic	G	A	intergenic
100211	intergenic	intergenic	A	G	intergenic
100221	NTHI477_RS09480	276	G	A	synonymous
100226	NTHI477_RS09480	271	G	A	synonymous
100228	NTHI477_RS09480	269	T	C	nonsynonymous
100230	NTHI477_RS09480	267	G	T	synonymous
100230	NTHI477_RS09480	267	G	A	synonymous
100231	NTHI477_RS09480	266	A	C	nonsynonymous
100279	NTHI477_RS09480	218	T	G	nonsynonymous
100280	NTHI477_RS09480	217	C	T	nonsynonymous
100281	NTHI477_RS09480	216	G	A	synonymous
100298	NTHI477_RS09480	199	C	T	nonsynonymous
100304	NTHI477_RS09480	193	T	A	nonsynonymous
100343	NTHI477_RS09480	154	C	T	nonsynonymous
100368	NTHI477_RS09480	129	T	C	synonymous
100431	NTHI477_RS09480	66	A	C	synonymous
100448	NTHI477_RS09480	49	C	T	nonsynonymous
100452	NTHI477_RS09480	45	T	C	synonymous
100455	NTHI477_RS09480	42	T	C	synonymous
100473	NTHI477_RS09480	24	A	C	synonymous
100481	NTHI477_RS09480	16	C	A	nonsynonymous
100484	NTHI477_RS09480	13	A	C	nonsynonymous
100510	NTHI477_RS00450	15	G	A	synonymous
100516	NTHI477_RS00450	21	T	C	synonymous
100603	NTHI477_RS00450	108	C	T	synonymous
100606	NTHI477_RS00450	111	A	G	synonymous
100621	NTHI477_RS00450	126	C	T	synonymous
100630	NTHI477_RS00450	135	A	C	synonymous
100639	NTHI477_RS00450	144	T	A	nonsynonymous
100645	NTHI477_RS00450	150	C	T	synonymous
100657	NTHI477_RS00450	162	G	C	synonymous
100678	NTHI477_RS00450	183	G	T	synonymous
100691	NTHI477_RS00450	196	T	C	nonsynonymous
100710	NTHI477_RS00450	215	C	T	nonsynonymous
100734	intergenic	intergenic	A	T	intergenic
100743	NTHI477_RS00455	3	G	A	synonymous
100749	NTHI477_RS00455	9	C	A	synonymous

100755	NTHI477_RS00455	15	G	A	synonymous
100770	NTHI477_RS00455	30	C	T	synonymous
100773	NTHI477_RS00455	33	C	T	synonymous
100797	NTHI477_RS00455	57	A	C	synonymous
100806	NTHI477_RS00455	66	A	T	synonymous
100809	NTHI477_RS00455	69	A	G	synonymous
100827	NTHI477_RS00455	87	T	C	synonymous
100851	NTHI477_RS00455	111	G	A	synonymous
100860	NTHI477_RS00455	120	C	T	synonymous
100862	NTHI477_RS00455	122	C	G	nonsynonymous
100864	NTHI477_RS00455	124	A	G	nonsynonymous
100875	NTHI477_RS00455	135	G	A	synonymous
100879	NTHI477_RS00455	139	G	A	nonsynonymous
100882	NTHI477_RS00455	142	G	T	nonsynonymous
100897	NTHI477_RS00455	157	G	T	nonsynonymous
100950	NTHI477_RS00455	210	G	A	synonymous
100962	NTHI477_RS00455	222	G	C	synonymous
100964	NTHI477_RS00455	224	A	C	nonsynonymous
100965	NTHI477_RS00455	225	G	A	synonymous
100971	NTHI477_RS00455	231	G	A	synonymous
100978	NTHI477_RS00455	238	G	A	nonsynonymous
100986	NTHI477_RS00455	246	G	A	synonymous
101004	NTHI477_RS00455	264	G	A	synonymous
101018	NTHI477_RS00455	278	A	G	nonsynonymous
101020	NTHI477_RS00455	280	A	G	nonsynonymous
101022	NTHI477_RS00455	282	G	A	nonsynonymous
101025	NTHI477_RS00455	285	G	A	synonymous
101048	NTHI477_RS00455	308	T	C	nonsynonymous
101049	NTHI477_RS00455	309	A	T	synonymous
101052	NTHI477_RS00455	312	T	A	nonsynonymous
101069	NTHI477_RS00455	329	T	C	nonsynonymous
101073	NTHI477_RS00455	333	T	G	nonsynonymous
101073	NTHI477_RS00455	333	T	A	nonsynonymous
101076	NTHI477_RS00455	336	A	G	synonymous
101091	NTHI477_RS00455	351	A	T	synonymous
101097	NTHI477_RS00455	357	T	C	synonymous
101100	NTHI477_RS00455	360	T	A	synonymous
101109	NTHI477_RS00455	369	T	C	synonymous
101120	NTHI477_RS00455	380	C	T	nonsynonymous
101121	NTHI477_RS00455	381	G	A	synonymous
101139	NTHI477_RS00455	399	G	A	synonymous
101154	NTHI477_RS00455	414	A	C	nonsynonymous
101175	NTHI477_RS00455	435	A	T	synonymous
101205	NTHI477_RS00455	465	A	G	synonymous
101208	NTHI477_RS00455	468	G	A	synonymous

101217	NTHI477_RS00455	477	C	T	synonymous
101368	NTHI477_RS00455	628	A	G	nonsynonymous
101444	NTHI477_RS00455	704	C	T	nonsynonymous
101576	intergenic	intergenic	C	T	intergenic
101582	intergenic	intergenic	T	A	intergenic
101596	intergenic	intergenic	A	G	intergenic
101602	intergenic	intergenic	T	G	intergenic
101606	intergenic	intergenic	T	C	intergenic
101623	intergenic	intergenic	C	T	intergenic
101652	intergenic	intergenic	T	C	intergenic
101669	intergenic	intergenic	C	T	intergenic
101673	intergenic	intergenic	G	A	intergenic
101699	intergenic	intergenic	C	A	intergenic
101714	intergenic	intergenic	C	T	intergenic
101717	intergenic	intergenic	G	A	intergenic
101718	intergenic	intergenic	G	A	intergenic
101736	intergenic	intergenic	C	G	intergenic
101737	intergenic	intergenic	C	A	intergenic
101791	NTHI477_RS00460	54	G	A	synonymous
101805	NTHI477_RS00460	68	G	T	nonsynonymous
101821	NTHI477_RS00460	84	T	C	synonymous
101824	NTHI477_RS00460	87	T	A	nonsynonymous
101827	NTHI477_RS00460	90	G	A	synonymous
101830	NTHI477_RS00460	93	C	T	synonymous
101830	NTHI477_RS00460	93	C	G	synonymous
101857	NTHI477_RS00460	120	G	A	synonymous
101860	NTHI477_RS00460	123	T	C	synonymous
101875	NTHI477_RS00460	138	T	C	synonymous
101882	NTHI477_RS00460	145	C	A	synonymous
101887	NTHI477_RS00460	150	C	A	synonymous
101887	NTHI477_RS00460	150	C	T	synonymous
101893	NTHI477_RS00460	156	C	T	synonymous
101896	NTHI477_RS00460	159	C	T	synonymous
101905	NTHI477_RS00460	168	G	A	synonymous
101908	NTHI477_RS00460	171	C	T	synonymous
101950	NTHI477_RS00460	213	A	G	synonymous
101951	NTHI477_RS00460	214	C	T	synonymous
101992	NTHI477_RS00460	255	A	T	synonymous
102001	NTHI477_RS00460	264	T	C	synonymous
102010	NTHI477_RS00460	273	T	C	synonymous
102046	NTHI477_RS00460	309	G	A	synonymous
102058	NTHI477_RS00460	321	A	G	synonymous
102061	NTHI477_RS00460	324	G	A	synonymous
102073	NTHI477_RS00460	336	C	T	synonymous
102088	NTHI477_RS00460	351	T	C	synonymous

102091	NTHI477_RS00460	354	G	A	synonymous
102106	NTHI477_RS00460	369	C	T	synonymous
102112	NTHI477_RS00460	375	G	A	synonymous
102124	NTHI477_RS00460	387	G	A	synonymous
102139	NTHI477_RS00460	402	C	G	synonymous
102148	NTHI477_RS00460	411	A	G	synonymous
102159	NTHI477_RS00460	422	T	C	nonsynonymous
102163	NTHI477_RS00460	426	T	C	synonymous
102165	NTHI477_RS00460	428	C	T	nonsynonymous
102190	NTHI477_RS00460	453	G	T	synonymous
102195	NTHI477_RS00460	458	T	C	nonsynonymous
102263	NTHI477_RS00460	526	G	A	nonsynonymous
102264	NTHI477_RS00460	527	T	C	nonsynonymous
102266	NTHI477_RS00460	529	C	T	nonsynonymous
102276	intergenic	intergenic	A	G	intergenic
102317	intergenic	intergenic	G	T	intergenic
102324	intergenic	intergenic	A	C	intergenic
102330	intergenic	intergenic	T	C	intergenic
102337	intergenic	intergenic	T	C	intergenic
102347	intergenic	intergenic	A	C	intergenic
102348	intergenic	intergenic	A	C	intergenic
102365	intergenic	intergenic	A	C	intergenic
102385	intergenic	intergenic	T	C	intergenic
102393	intergenic	intergenic	T	G	intergenic
102396	intergenic	intergenic	C	A	intergenic
102416	intergenic	intergenic	A	C	intergenic
102417	intergenic	intergenic	A	T	intergenic
102437	intergenic	intergenic	G	A	intergenic
102441	intergenic	intergenic	G	A	intergenic
102450	intergenic	intergenic	G	A	intergenic
102453	intergenic	intergenic	G	T	intergenic
102506	NTHI477_RS00465	32	C	T	nonsynonymous
102513	NTHI477_RS00465	39	A	C	nonsynonymous
102523	NTHI477_RS00465	49	A	G	nonsynonymous
102525	NTHI477_RS00465	51	C	A	synonymous
102528	NTHI477_RS00465	54	T	G	nonsynonymous
102537	NTHI477_RS00465	63	A	C	nonsynonymous
102571	NTHI477_RS00465	97	T	G	nonsynonymous
102583	NTHI477_RS00465	109	T	C	nonsynonymous
102584	NTHI477_RS00465	110	C	T	nonsynonymous
102591	NTHI477_RS00465	117	C	T	synonymous
102594	NTHI477_RS00465	120	T	A	synonymous
102596	NTHI477_RS00465	122	C	A	nonsynonymous
102597	NTHI477_RS00465	123	G	A	synonymous
102600	NTHI477_RS00465	126	A	G	synonymous

102616	NTHI477_RS00465	142	A	G	nonsynonymous
102625	NTHI477_RS00465	151	T	C	nonsynonymous
102629	NTHI477_RS00465	155	T	G	nonsynonymous
102651	NTHI477_RS00465	177	A	C	synonymous
102658	NTHI477_RS00465	184	G	C	nonsynonymous
102659	NTHI477_RS00465	185	A	T	nonsynonymous
102661	NTHI477_RS00465	187	G	A	nonsynonymous
102662	NTHI477_RS00465	188	C	T	nonsynonymous
102667	NTHI477_RS00465	193	T	A	nonsynonymous
102669	NTHI477_RS00465	195	G	T	nonsynonymous
102671	NTHI477_RS00465	197	T	A	nonsynonymous
102672	NTHI477_RS00465	198	G	T	nonsynonymous
102679	NTHI477_RS00465	205	G	T	nonsynonymous
102688	NTHI477_RS00465	214	G	A	nonsynonymous
102689	NTHI477_RS00465	215	C	A	nonsynonymous
102692	NTHI477_RS00465	218	G	A	nonsynonymous
102693	NTHI477_RS00465	219	A	G	synonymous
102696	NTHI477_RS00465	222	A	C	synonymous
102697	NTHI477_RS00465	223	A	C	nonsynonymous
102698	NTHI477_RS00465	224	T	A	nonsynonymous
102718	NTHI477_RS00465	244	T	G	nonsynonymous
102732	NTHI477_RS00465	258	G	A	synonymous
102742	NTHI477_RS00465	268	C	A	nonsynonymous
102766	NTHI477_RS00465	292	A	G	nonsynonymous
102776	NTHI477_RS00465	302	C	T	nonsynonymous
102778	NTHI477_RS00465	304	A	C	nonsynonymous
102780	NTHI477_RS00465	306	A	G	nonsynonymous
102791	NTHI477_RS00465	317	C	T	nonsynonymous
102805	NTHI477_RS00465	331	C	G	nonsynonymous
102810	NTHI477_RS00465	336	C	A	nonsynonymous
102814	NTHI477_RS00465	340	G	A	nonsynonymous
102836	NTHI477_RS00465	362	C	A	nonsynonymous
102837	NTHI477_RS00465	363	C	T	synonymous
102847	NTHI477_RS00465	373	T	C	nonsynonymous
102850	NTHI477_RS00465	376	G	A	nonsynonymous
102870	NTHI477_RS00465	396	T	A	synonymous
102871	NTHI477_RS00465	397	G	A	nonsynonymous
102873	NTHI477_RS00465	399	T	A	nonsynonymous
102874	NTHI477_RS00465	400	C	G	nonsynonymous
102878	NTHI477_RS00465	404	A	T	nonsynonymous
102879	NTHI477_RS00465	405	T	A	nonsynonymous
102882	NTHI477_RS00465	408	T	A	synonymous
102882	NTHI477_RS00465	408	T	C	synonymous
102885	NTHI477_RS00465	411	C	T	synonymous
102894	NTHI477_RS00465	420	T	C	synonymous

102897	NTHI477_RS00465	423	C	T	synonymous
102898	NTHI477_RS00465	424	A	G	nonsynonymous
102901	NTHI477_RS00465	427	T	C	synonymous
102905	NTHI477_RS00465	431	A	C	nonsynonymous
102915	NTHI477_RS00465	441	G	A	synonymous
102917	NTHI477_RS00465	443	T	G	nonsynonymous
102959	NTHI477_RS09390	39	A	G	synonymous
102962	NTHI477_RS09390	42	G	A	synonymous
102994	NTHI477_RS09390	74	A	G	nonsynonymous
102998	NTHI477_RS09390	78	C	T	synonymous
103008	NTHI477_RS09390	88	C	T	nonsynonymous
103010	NTHI477_RS09390	90	T	C	synonymous
103105	intergenic	intergenic	A	C	intergenic
103106	intergenic	intergenic	C	A	intergenic
103117	NTHI477_RS00470	9	C	G	synonymous
103130	NTHI477_RS00470	22	C	T	nonsynonymous
103132	NTHI477_RS00470	24	T	A	synonymous
103147	NTHI477_RS00470	39	C	A	nonsynonymous
103157	NTHI477_RS00470	49	A	G	nonsynonymous
103159	NTHI477_RS00470	51	T	C	synonymous
103159	NTHI477_RS00470	51	T	A	synonymous
103194	NTHI477_RS00470	86	C	T	nonsynonymous
103195	NTHI477_RS00470	87	G	C	synonymous
103198	NTHI477_RS00470	90	A	G	synonymous
103219	NTHI477_RS00470	111	A	G	synonymous
103222	NTHI477_RS00470	114	C	A	synonymous
103228	NTHI477_RS00470	120	T	C	synonymous
103229	NTHI477_RS00470	121	G	A	nonsynonymous
103238	NTHI477_RS00470	130	A	G	nonsynonymous
103240	NTHI477_RS00470	132	C	T	synonymous
103271	NTHI477_RS00470	163	C	A	nonsynonymous
103276	NTHI477_RS00470	168	G	A	synonymous
103279	NTHI477_RS00470	171	G	A	synonymous
103285	NTHI477_RS00470	177	A	G	synonymous
103286	NTHI477_RS00470	178	G	T	nonsynonymous
103286	NTHI477_RS00470	178	G	A	nonsynonymous
103292	NTHI477_RS00470	184	C	G	nonsynonymous
103292	NTHI477_RS00470	184	C	A	nonsynonymous
103294	NTHI477_RS00470	186	G	T	nonsynonymous
103295	NTHI477_RS00470	187	G	A	nonsynonymous
103296	NTHI477_RS00470	188	C	T	nonsynonymous
103301	NTHI477_RS00470	193	G	T	nonsynonymous
103302	NTHI477_RS00470	194	G	T	nonsynonymous
103302	NTHI477_RS00470	194	G	A	nonsynonymous
103304	NTHI477_RS00470	196	G	C	nonsynonymous

103304	NTHI477_RS00470	196	G	A	nonsynonymous
103305	NTHI477_RS00470	197	G	A	nonsynonymous
103306	NTHI477_RS00470	198	T	A	synonymous
103312	NTHI477_RS00470	204	C	T	synonymous
103313	NTHI477_RS00470	205	A	G	nonsynonymous
103313	NTHI477_RS00470	205	A	T	nonsynonymous
103314	NTHI477_RS00470	206	T	A	nonsynonymous
103315	NTHI477_RS00470	207	G	T	nonsynonymous
103321	NTHI477_RS00470	213	T	C	synonymous
103322	NTHI477_RS00470	214	G	A	nonsynonymous
103323	NTHI477_RS00470	215	A	C	nonsynonymous
103323	NTHI477_RS00470	215	A	T	nonsynonymous
103324	NTHI477_RS00470	216	A	C	nonsynonymous
103324	NTHI477_RS00470	216	A	G	synonymous
103326	NTHI477_RS00470	218	G	A	nonsynonymous
103327	NTHI477_RS00470	219	A	T	nonsynonymous
103332	NTHI477_RS00470	224	T	G	nonsynonymous
103332	NTHI477_RS00470	224	T	A	nonsynonymous
103333	NTHI477_RS00470	225	T	A	synonymous
103336	NTHI477_RS00470	228	C	A	synonymous
103337	NTHI477_RS00470	229	G	A	nonsynonymous
103339	NTHI477_RS00470	231	A	C	synonymous
103349	NTHI477_RS00470	241	G	T	nonsynonymous
103350	NTHI477_RS00470	242	A	G	nonsynonymous
103352	NTHI477_RS00470	244	T	G	nonsynonymous
103354	NTHI477_RS00470	246	C	T	synonymous
103355	NTHI477_RS00470	247	A	G	nonsynonymous
103360	NTHI477_RS00470	252	A	G	synonymous
103360	NTHI477_RS00470	252	A	C	synonymous
103366	NTHI477_RS00470	258	C	G	nonsynonymous
103366	NTHI477_RS00470	258	C	A	nonsynonymous
103371	NTHI477_RS00470	263	A	T	nonsynonymous
103376	NTHI477_RS00470	268	A	C	nonsynonymous
103381	NTHI477_RS00470	273	G	A	synonymous
103384	NTHI477_RS00470	276	G	A	synonymous
103385	NTHI477_RS00470	277	G	A	nonsynonymous
103393	NTHI477_RS00470	285	G	A	synonymous
103394	NTHI477_RS00470	286	G	A	nonsynonymous
103394	NTHI477_RS00470	286	G	T	nonsynonymous
103395	NTHI477_RS00470	287	A	T	nonsynonymous
103396	NTHI477_RS00470	288	T	G	nonsynonymous
103400	NTHI477_RS00470	292	G	A	nonsynonymous
103402	NTHI477_RS00470	294	T	G	nonsynonymous
103409	NTHI477_RS00470	301	T	G	nonsynonymous
103411	NTHI477_RS00470	303	A	T	nonsynonymous

103411	NTHI477_RS00470	303	A	G	synonymous
103414	NTHI477_RS00470	306	T	A	nonsynonymous
103420	NTHI477_RS00470	312	T	C	synonymous
103421	NTHI477_RS00470	313	G	A	nonsynonymous
103422	NTHI477_RS00470	314	C	A	nonsynonymous
103422	NTHI477_RS00470	314	C	T	nonsynonymous
103423	NTHI477_RS00470	315	G	T	synonymous
103429	NTHI477_RS00470	321	T	C	synonymous
103431	NTHI477_RS00470	323	C	A	nonsynonymous
103432	NTHI477_RS00470	324	A	G	synonymous
103432	NTHI477_RS00470	324	A	T	synonymous
103433	NTHI477_RS00470	325	A	G	nonsynonymous
103434	NTHI477_RS00470	326	T	A	nonsynonymous
103435	NTHI477_RS00470	327	C	G	nonsynonymous
103438	NTHI477_RS00470	330	A	G	synonymous
103440	NTHI477_RS00470	332	G	A	nonsynonymous
103440	NTHI477_RS00470	332	G	T	nonsynonymous
103441	NTHI477_RS00470	333	T	A	synonymous
103444	NTHI477_RS00470	336	C	T	synonymous
103447	NTHI477_RS00470	339	A	C	synonymous
103457	NTHI477_RS00470	349	G	A	nonsynonymous
103457	NTHI477_RS00470	349	G	T	nonsynonymous
103458	NTHI477_RS00470	350	G	A	nonsynonymous
103460	NTHI477_RS00470	352	G	T	nonsynonymous
103467	NTHI477_RS00470	359	C	A	nonsynonymous
103468	NTHI477_RS00470	360	G	A	synonymous
103468	NTHI477_RS00470	360	G	C	synonymous
103471	NTHI477_RS00470	363	G	A	synonymous
103474	NTHI477_RS00470	366	G	A	synonymous
103474	NTHI477_RS00470	366	G	C	nonsynonymous
103474	NTHI477_RS00470	366	G	T	nonsynonymous
103479	NTHI477_RS00470	371	T	A	nonsynonymous
103484	NTHI477_RS00470	376	A	C	nonsynonymous
103489	NTHI477_RS00470	381	G	A	synonymous
103491	NTHI477_RS00470	383	C	A	nonsynonymous
103492	NTHI477_RS00470	384	A	G	synonymous
103495	NTHI477_RS00470	387	G	A	synonymous
103498	NTHI477_RS00470	390	G	A	synonymous
103501	NTHI477_RS00470	393	A	G	synonymous
103502	NTHI477_RS00470	394	A	T	nonsynonymous
103502	NTHI477_RS00470	394	A	C	nonsynonymous
103502	NTHI477_RS00470	394	A	G	nonsynonymous
103504	NTHI477_RS00470	396	T	G	nonsynonymous
103508	NTHI477_RS00470	400	A	G	nonsynonymous
103510	NTHI477_RS00470	402	G	A	synonymous

103510	NTHI477_RS00470	402	G	T	nonsynonymous
103514	NTHI477_RS00470	406	C	A	nonsynonymous
103517	NTHI477_RS00470	409	A	G	nonsynonymous
103517	NTHI477_RS00470	409	A	T	nonsynonymous
103518	NTHI477_RS00470	410	A	G	nonsynonymous
103518	NTHI477_RS00470	410	A	T	nonsynonymous
103520	NTHI477_RS00470	412	G	A	nonsynonymous
103521	NTHI477_RS00470	413	G	A	nonsynonymous
103528	NTHI477_RS00470	420	C	G	synonymous
103528	NTHI477_RS00470	420	C	T	synonymous
103529	NTHI477_RS00470	421	A	G	nonsynonymous
103529	NTHI477_RS00470	421	A	T	nonsynonymous
103531	NTHI477_RS00470	423	G	A	nonsynonymous
103538	NTHI477_RS00470	430	G	C	nonsynonymous
103539	NTHI477_RS00470	431	A	G	nonsynonymous
103539	NTHI477_RS00470	431	A	T	nonsynonymous
103540	NTHI477_RS00470	432	C	A	nonsynonymous
103540	NTHI477_RS00470	432	C	T	synonymous
103541	NTHI477_RS00470	433	G	A	nonsynonymous
103541	NTHI477_RS00470	433	G	T	nonsynonymous
103542	NTHI477_RS00470	434	G	A	nonsynonymous
103543	NTHI477_RS00470	435	A	T	synonymous
103544	NTHI477_RS00470	436	G	A	nonsynonymous
103548	NTHI477_RS00470	440	T	G	nonsynonymous
103549	NTHI477_RS00470	441	T	A	synonymous
103553	NTHI477_RS00470	445	A	G	nonsynonymous
103555	NTHI477_RS00470	447	A	C	synonymous
103557	NTHI477_RS00470	449	A	C	nonsynonymous
103565	NTHI477_RS00470	457	T	G	nonsynonymous
103566	NTHI477_RS00470	458	A	G	nonsynonymous
103568	NTHI477_RS00470	460	T	G	nonsynonymous
103570	NTHI477_RS00470	462	C	T	synonymous
103571	NTHI477_RS00470	463	A	G	nonsynonymous
103575	NTHI477_RS00470	467	C	A	nonsynonymous
103576	NTHI477_RS00470	468	G	T	synonymous
103576	NTHI477_RS00470	468	G	A	synonymous
103581	NTHI477_RS00470	473	A	C	nonsynonymous
103582	NTHI477_RS00470	474	A	C	nonsynonymous
103582	NTHI477_RS00470	474	A	G	synonymous
103586	NTHI477_RS00470	478	C	T	nonsynonymous
103587	NTHI477_RS00470	479	A	T	nonsynonymous
103592	NTHI477_RS00470	484	A	C	nonsynonymous
103593	NTHI477_RS00470	485	A	G	nonsynonymous
103597	NTHI477_RS00470	489	G	A	synonymous
103606	NTHI477_RS00470	498	G	A	synonymous

103610	NTHI477_RS00470	502	T	G	nonsynonymous
103614	NTHI477_RS00470	506	G	C	nonsynonymous
103615	NTHI477_RS00470	507	A	G	synonymous
103616	NTHI477_RS00470	508	G	A	nonsynonymous
103617	NTHI477_RS00470	509	G	C	nonsynonymous
103617	NTHI477_RS00470	509	G	A	nonsynonymous
103625	NTHI477_RS00470	517	G	T	nonsynonymous
103626	NTHI477_RS00470	518	T	C	nonsynonymous
103628	NTHI477_RS00470	520	A	C	nonsynonymous
103629	NTHI477_RS00470	521	T	A	nonsynonymous
103630	NTHI477_RS00470	522	G	A	nonsynonymous
103630	NTHI477_RS00470	522	G	T	nonsynonymous
103641	NTHI477_RS00470	533	C	T	nonsynonymous
103650	NTHI477_RS00470	542	C	A	nonsynonymous
103651	NTHI477_RS00470	543	G	A	synonymous
103718	NTHI477_RS00470	610	G	C	nonsynonymous
103718	NTHI477_RS00470	610	G	A	nonsynonymous
103720	NTHI477_RS00470	612	A	T	nonsynonymous
103724	NTHI477_RS00470	616	G	A	nonsynonymous
103725	NTHI477_RS00470	617	T	C	nonsynonymous
103725	NTHI477_RS00470	617	T	A	nonsynonymous
103726	NTHI477_RS00470	618	G	T	synonymous
103729	NTHI477_RS00470	621	T	C	synonymous
103732	NTHI477_RS00470	624	T	C	synonymous
103742	NTHI477_RS00470	634	G	A	nonsynonymous
103762	NTHI477_RS00470	654	A	G	synonymous
103764	NTHI477_RS00470	656	G	T	nonsynonymous
103834	NTHI477_RS09395	67	A	G	nonsynonymous
103840	NTHI477_RS09395	73	G	A	nonsynonymous
103847	NTHI477_RS09395	80	A	C	nonsynonymous
103848	NTHI477_RS09395	81	A	G	synonymous
103850	NTHI477_RS09395	83	C	A	nonsynonymous
103882	NTHI477_RS09395	115	C	A	nonsynonymous
103928	NTHI477_RS09395	161	C	T	nonsynonymous
103929	NTHI477_RS09395	162	C	T	synonymous
103965	NTHI477_RS00475	9	C	A	synonymous
103978	NTHI477_RS00475	22	C	T	nonsynonymous
103980	NTHI477_RS00475	24	T	A	synonymous
103995	NTHI477_RS00475	39	C	A	nonsynonymous
104005	NTHI477_RS00475	49	G	A	nonsynonymous
104007	NTHI477_RS00475	51	A	T	synonymous
104007	NTHI477_RS00475	51	A	C	synonymous
104022	NTHI477_RS00475	66	C	A	synonymous
104025	NTHI477_RS00475	69	C	T	synonymous
104030	NTHI477_RS00475	74	G	C	nonsynonymous

104032	NTHI477_RS00475	76	G	C	nonsynonymous
104033	NTHI477_RS00475	77	C	T	nonsynonymous
104042	NTHI477_RS00475	86	T	C	nonsynonymous
104042	NTHI477_RS00475	86	T	A	nonsynonymous
104043	NTHI477_RS00475	87	G	A	synonymous
104044	NTHI477_RS00475	88	G	T	nonsynonymous
104045	NTHI477_RS00475	89	A	T	nonsynonymous
104046	NTHI477_RS00475	90	A	C	nonsynonymous
104049	NTHI477_RS00475	93	G	A	synonymous
104053	NTHI477_RS00475	97	T	G	nonsynonymous
104066	NTHI477_RS00475	110	T	C	nonsynonymous
104067	NTHI477_RS00475	111	A	G	synonymous
104070	NTHI477_RS00475	114	C	A	synonymous
104076	NTHI477_RS00475	120	T	C	synonymous
104077	NTHI477_RS00475	121	G	A	nonsynonymous
104086	NTHI477_RS00475	130	A	G	nonsynonymous
104088	NTHI477_RS00475	132	C	T	synonymous
104119	NTHI477_RS00475	163	C	A	nonsynonymous
104124	NTHI477_RS00475	168	G	A	synonymous
104134	NTHI477_RS00475	178	G	A	nonsynonymous
104134	NTHI477_RS00475	178	G	T	nonsynonymous
104139	NTHI477_RS00475	183	A	G	synonymous
104140	NTHI477_RS00475	184	A	C	nonsynonymous
104140	NTHI477_RS00475	184	A	G	nonsynonymous
104141	NTHI477_RS00475	185	A	G	nonsynonymous
104142	NTHI477_RS00475	186	T	G	nonsynonymous
104143	NTHI477_RS00475	187	G	A	nonsynonymous
104144	NTHI477_RS00475	188	T	C	nonsynonymous
104149	NTHI477_RS00475	193	T	G	nonsynonymous
104150	NTHI477_RS00475	194	T	G	nonsynonymous
104152	NTHI477_RS00475	196	A	G	nonsynonymous
104153	NTHI477_RS00475	197	A	G	nonsynonymous
104161	NTHI477_RS00475	205	G	A	nonsynonymous
104161	NTHI477_RS00475	205	G	T	nonsynonymous
104162	NTHI477_RS00475	206	T	A	nonsynonymous
104163	NTHI477_RS00475	207	G	T	synonymous
104170	NTHI477_RS00475	214	G	A	nonsynonymous
104170	NTHI477_RS00475	214	G	T	nonsynonymous
104171	NTHI477_RS00475	215	C	A	nonsynonymous
104172	NTHI477_RS00475	216	T	A	synonymous
104172	NTHI477_RS00475	216	T	C	synonymous
104173	NTHI477_RS00475	217	G	A	nonsynonymous
104175	NTHI477_RS00475	219	G	A	synonymous
104175	NTHI477_RS00475	219	G	C	nonsynonymous
104176	NTHI477_RS00475	220	G	C	nonsynonymous

104178	NTHI477_RS00475	222	G	A	synonymous
104180	NTHI477_RS00475	224	A	T	nonsynonymous
104181	NTHI477_RS00475	225	A	T	nonsynonymous
104184	NTHI477_RS00475	228	C	A	synonymous
104185	NTHI477_RS00475	229	G	A	nonsynonymous
104187	NTHI477_RS00475	231	C	A	synonymous
104200	NTHI477_RS00475	244	T	G	nonsynonymous
104208	NTHI477_RS00475	252	G	A	synonymous
104214	NTHI477_RS00475	258	A	G	synonymous
104214	NTHI477_RS00475	258	A	C	nonsynonymous
104219	NTHI477_RS00475	263	A	T	nonsynonymous
104221	NTHI477_RS00475	265	C	A	nonsynonymous
104223	NTHI477_RS00475	267	C	A	synonymous
104229	NTHI477_RS00475	273	G	A	synonymous
104231	NTHI477_RS00475	275	C	A	nonsynonymous
104232	NTHI477_RS00475	276	G	A	synonymous
104235	NTHI477_RS00475	279	G	A	synonymous
104238	NTHI477_RS00475	282	G	A	synonymous
104241	NTHI477_RS00475	285	A	T	synonymous
104241	NTHI477_RS00475	285	A	G	synonymous
104242	NTHI477_RS00475	286	G	A	nonsynonymous
104242	NTHI477_RS00475	286	G	C	nonsynonymous
104242	NTHI477_RS00475	286	G	T	nonsynonymous
104248	NTHI477_RS00475	292	A	G	nonsynonymous
104250	NTHI477_RS00475	294	T	G	nonsynonymous
104254	NTHI477_RS00475	298	C	A	nonsynonymous
104256	NTHI477_RS00475	300	G	A	synonymous
104257	NTHI477_RS00475	301	G	T	nonsynonymous
104257	NTHI477_RS00475	301	G	A	nonsynonymous
104258	NTHI477_RS00475	302	C	T	nonsynonymous
104258	NTHI477_RS00475	302	C	G	nonsynonymous
104258	NTHI477_RS00475	302	C	A	nonsynonymous
104260	NTHI477_RS00475	304	T	A	nonsynonymous
104260	NTHI477_RS00475	304	T	G	nonsynonymous
104265	NTHI477_RS00475	309	A	G	synonymous
104268	NTHI477_RS00475	312	T	C	synonymous
104269	NTHI477_RS00475	313	T	A	nonsynonymous
104269	NTHI477_RS00475	313	T	G	nonsynonymous
104270	NTHI477_RS00475	314	T	A	nonsynonymous
104270	NTHI477_RS00475	314	T	C	nonsynonymous
104271	NTHI477_RS00475	315	G	T	nonsynonymous
104272	NTHI477_RS00475	316	G	A	nonsynonymous
104273	NTHI477_RS00475	317	C	T	nonsynonymous
104274	NTHI477_RS00475	318	T	G	synonymous
104278	NTHI477_RS00475	322	A	G	nonsynonymous

104279	NTHI477_RS00475	323	C	T	nonsynonymous
104279	NTHI477_RS00475	323	C	G	nonsynonymous
104280	NTHI477_RS00475	324	T	C	synonymous
104280	NTHI477_RS00475	324	T	A	synonymous
104281	NTHI477_RS00475	325	G	A	nonsynonymous
104282	NTHI477_RS00475	326	A	C	nonsynonymous
104283	NTHI477_RS00475	327	A	T	nonsynonymous
104283	NTHI477_RS00475	327	A	C	nonsynonymous
104283	NTHI477_RS00475	327	A	G	synonymous
104286	NTHI477_RS00475	330	G	A	synonymous
104288	NTHI477_RS00475	332	G	A	nonsynonymous
104289	NTHI477_RS00475	333	T	A	synonymous
104292	NTHI477_RS00475	336	T	C	synonymous
104295	NTHI477_RS00475	339	A	C	synonymous
104297	NTHI477_RS00475	341	G	A	nonsynonymous
104305	NTHI477_RS00475	349	T	A	nonsynonymous
104305	NTHI477_RS00475	349	T	G	nonsynonymous
104308	NTHI477_RS00475	352	A	T	nonsynonymous
104309	NTHI477_RS00475	353	C	T	nonsynonymous
104310	NTHI477_RS00475	354	C	T	synonymous
104311	NTHI477_RS00475	355	G	A	nonsynonymous
104316	NTHI477_RS00475	360	G	C	synonymous
104319	NTHI477_RS00475	363	G	A	synonymous
104322	NTHI477_RS00475	366	G	A	synonymous
104327	NTHI477_RS00475	371	T	A	nonsynonymous
104331	NTHI477_RS00475	375	C	T	synonymous
104343	NTHI477_RS00475	387	G	T	nonsynonymous
104346	NTHI477_RS00475	390	G	A	synonymous
104349	NTHI477_RS00475	393	A	G	synonymous
104350	NTHI477_RS00475	394	C	T	nonsynonymous
104350	NTHI477_RS00475	394	C	G	nonsynonymous
104350	NTHI477_RS00475	394	C	A	nonsynonymous
104352	NTHI477_RS00475	396	T	G	nonsynonymous
104356	NTHI477_RS00475	400	A	G	nonsynonymous
104358	NTHI477_RS00475	402	T	A	nonsynonymous
104358	NTHI477_RS00475	402	T	G	nonsynonymous
104364	NTHI477_RS00475	408	A	G	synonymous
104366	NTHI477_RS00475	410	C	G	nonsynonymous
104366	NTHI477_RS00475	410	C	T	nonsynonymous
104368	NTHI477_RS00475	412	A	C	nonsynonymous
104369	NTHI477_RS00475	413	T	G	nonsynonymous
104370	NTHI477_RS00475	414	A	T	synonymous
104370	NTHI477_RS00475	414	A	G	nonsynonymous
104379	NTHI477_RS00475	423	C	A	nonsynonymous
104386	NTHI477_RS00475	430	C	A	nonsynonymous

104396	NTHI477_RS00475	440	A	G	nonsynonymous
104401	NTHI477_RS00475	445	G	A	nonsynonymous
104416	NTHI477_RS00475	460	T	G	nonsynonymous
104417	NTHI477_RS00475	461	C	T	nonsynonymous
104424	NTHI477_RS00475	468	T	C	synonymous
104437	NTHI477_RS00475	481	G	A	nonsynonymous
104457	NTHI477_RS00475	501	T	A	synonymous
104457	NTHI477_RS00475	501	T	C	synonymous
104458	NTHI477_RS00475	502	G	A	nonsynonymous
104460	NTHI477_RS00475	504	T	A	nonsynonymous
104461	NTHI477_RS00475	505	C	G	nonsynonymous
104464	NTHI477_RS00475	508	G	A	nonsynonymous
104464	NTHI477_RS00475	508	G	C	nonsynonymous
104464	NTHI477_RS00475	508	G	T	nonsynonymous
104465	NTHI477_RS00475	509	G	T	nonsynonymous
104466	NTHI477_RS00475	510	T	A	synonymous
104466	NTHI477_RS00475	510	T	G	synonymous
104469	NTHI477_RS00475	513	T	A	synonymous
104472	NTHI477_RS00475	516	C	T	synonymous
104473	NTHI477_RS00475	517	A	G	nonsynonymous
104481	NTHI477_RS00475	525	T	C	synonymous
104484	NTHI477_RS00475	528	C	T	synonymous
104488	NTHI477_RS00475	532	C	T	synonymous
104504	NTHI477_RS00475	548	G	T	nonsynonymous
104515	intergenic	intergenic	T	A	intergenic
104517	intergenic	intergenic	T	C	intergenic
104535	intergenic	intergenic	T	C	intergenic
104558	intergenic	intergenic	G	C	intergenic
104573	intergenic	intergenic	G	A	intergenic
104595	intergenic	intergenic	T	G	intergenic
104596	intergenic	intergenic	A	G	intergenic
104600	intergenic	intergenic	C	T	intergenic
104605	intergenic	intergenic	T	C	intergenic
104606	intergenic	intergenic	T	C	intergenic
104608	intergenic	intergenic	C	T	intergenic
104609	intergenic	intergenic	C	T	intergenic
104610	intergenic	intergenic	C	T	intergenic
104612	intergenic	intergenic	T	C	intergenic
104626	intergenic	intergenic	A	G	intergenic
104632	intergenic	intergenic	T	C	intergenic
104633	intergenic	intergenic	G	T	intergenic
104646	intergenic	intergenic	C	A	intergenic
104664	intergenic	intergenic	A	G	intergenic
104666	intergenic	intergenic	A	G	intergenic
104737	intergenic	intergenic	C	T	intergenic

104738	intergenic	intergenic	C	T	intergenic
104755	intergenic	intergenic	G	A	intergenic
104757	intergenic	intergenic	A	G	intergenic
104781	intergenic	intergenic	T	C	intergenic
104831	NTHI477_RS00480	45	T	C	synonymous
104837	NTHI477_RS00480	51	A	G	synonymous
104840	NTHI477_RS00480	54	G	A	synonymous
104841	NTHI477_RS00480	55	C	T	synonymous
104846	NTHI477_RS00480	60	A	G	synonymous
104849	NTHI477_RS00480	63	C	A	synonymous
104852	NTHI477_RS00480	66	A	G	synonymous
104853	NTHI477_RS00480	67	T	C	synonymous
104855	NTHI477_RS00480	69	G	A	synonymous
104863	NTHI477_RS00480	77	T	C	nonsynonymous
104864	NTHI477_RS00480	78	T	G	synonymous
104867	NTHI477_RS00480	81	T	G	synonymous
104870	NTHI477_RS00480	84	A	G	synonymous
104874	NTHI477_RS00480	88	A	T	nonsynonymous
104875	NTHI477_RS00480	89	G	C	nonsynonymous
104876	NTHI477_RS00480	90	T	G	nonsynonymous
104915	NTHI477_RS00480	129	G	A	synonymous
104927	NTHI477_RS00480	141	A	T	nonsynonymous
104930	NTHI477_RS00480	144	A	C	synonymous
104930	NTHI477_RS00480	144	A	G	synonymous
104948	NTHI477_RS00480	162	T	C	synonymous
104957	NTHI477_RS00480	171	A	G	synonymous
104978	NTHI477_RS00480	192	A	G	synonymous
104993	NTHI477_RS00480	207	C	T	synonymous
104996	NTHI477_RS00480	210	C	T	synonymous
105032	NTHI477_RS00480	246	C	T	synonymous
105046	NTHI477_RS00480	260	C	T	nonsynonymous
105068	NTHI477_RS00480	282	A	G	synonymous
105087	NTHI477_RS00480	301	C	T	synonymous
105131	NTHI477_RS00480	345	C	T	synonymous
105134	NTHI477_RS00480	348	T	A	synonymous
105149	NTHI477_RS00480	363	T	C	synonymous
105158	NTHI477_RS00480	372	A	G	synonymous
105161	NTHI477_RS00480	375	T	C	synonymous
105170	NTHI477_RS00480	384	G	C	synonymous
105173	NTHI477_RS00480	387	T	A	synonymous
105191	NTHI477_RS00480	405	C	T	synonymous
105194	NTHI477_RS00480	408	C	T	synonymous
105197	NTHI477_RS00480	411	T	C	synonymous
105203	NTHI477_RS00480	417	G	T	synonymous
105215	NTHI477_RS00480	429	C	T	synonymous

105219	NTHI477_RS00480	433	T	C	nonsynonymous
105219	NTHI477_RS00480	433	T	A	nonsynonymous
105221	NTHI477_RS00480	435	T	C	synonymous
105222	NTHI477_RS00480	436	G	A	nonsynonymous
105224	NTHI477_RS00480	438	A	G	synonymous
105228	NTHI477_RS00480	442	A	G	nonsynonymous
105230	NTHI477_RS00480	444	G	A	nonsynonymous
105231	NTHI477_RS00480	445	C	T	nonsynonymous
105233	NTHI477_RS00480	447	C	A	synonymous
105238	NTHI477_RS00480	452	C	A	nonsynonymous
105239	NTHI477_RS00480	453	C	T	synonymous
105239	NTHI477_RS00480	453	C	A	synonymous
105242	NTHI477_RS00480	456	C	T	synonymous
105248	NTHI477_RS00480	462	C	T	synonymous
105254	NTHI477_RS00480	468	G	A	synonymous
105260	NTHI477_RS00480	474	T	A	synonymous
105262	NTHI477_RS00480	476	A	T	nonsynonymous
105269	NTHI477_RS00480	483	A	G	synonymous
105270	NTHI477_RS00480	484	G	A	nonsynonymous
105272	NTHI477_RS00480	486	G	T	synonymous
105275	NTHI477_RS00480	489	C	T	synonymous
105278	NTHI477_RS00480	492	A	T	synonymous
105279	NTHI477_RS00480	493	A	T	nonsynonymous
105280	NTHI477_RS00480	494	A	T	nonsynonymous
105284	NTHI477_RS00480	498	A	C	synonymous
105284	NTHI477_RS00480	498	A	G	synonymous
105286	NTHI477_RS00480	500	G	C	nonsynonymous
105287	NTHI477_RS00480	501	T	C	synonymous
105290	NTHI477_RS00480	504	C	A	synonymous
105290	NTHI477_RS00480	504	C	T	synonymous
105293	NTHI477_RS00480	507	A	G	synonymous
105296	NTHI477_RS00480	510	C	T	synonymous
105305	NTHI477_RS00480	519	C	T	synonymous
105308	NTHI477_RS00480	522	G	A	synonymous
105309	NTHI477_RS00480	523	C	T	nonsynonymous
105311	NTHI477_RS00480	525	T	G	synonymous
105314	NTHI477_RS00480	528	A	G	synonymous
105320	NTHI477_RS00480	534	A	G	synonymous
105323	NTHI477_RS00480	537	G	T	synonymous
105326	NTHI477_RS00480	540	C	T	synonymous
105329	NTHI477_RS00480	543	T	C	synonymous
105341	NTHI477_RS00480	555	A	G	synonymous
105344	NTHI477_RS00480	558	T	C	synonymous
105356	NTHI477_RS00480	570	A	T	synonymous
105360	NTHI477_RS00480	574	A	T	nonsynonymous

105361	NTHI477_RS00480	575	G	C	nonsynonymous
105362	NTHI477_RS00480	576	C	A	nonsynonymous
105362	NTHI477_RS00480	576	C	T	synonymous
105362	NTHI477_RS00480	576	C	G	nonsynonymous
105368	NTHI477_RS00480	582	G	T	synonymous
105368	NTHI477_RS00480	582	G	A	synonymous
105374	NTHI477_RS00480	588	C	T	synonymous
105377	NTHI477_RS00480	591	C	T	synonymous
105380	NTHI477_RS00480	594	G	T	synonymous
105380	NTHI477_RS00480	594	G	C	synonymous
105383	NTHI477_RS00480	597	T	A	synonymous
105392	NTHI477_RS00480	606	C	T	synonymous
105395	NTHI477_RS00480	609	T	C	synonymous
105416	NTHI477_RS00480	630	T	A	synonymous
105416	NTHI477_RS00480	630	T	G	synonymous
105419	NTHI477_RS00480	633	A	G	synonymous
105422	NTHI477_RS00480	636	T	C	synonymous
105431	NTHI477_RS00480	645	A	G	synonymous
105443	NTHI477_RS00480	657	A	T	synonymous
105446	NTHI477_RS00480	660	A	C	synonymous
105446	NTHI477_RS00480	660	A	G	synonymous
105452	NTHI477_RS00480	666	A	G	synonymous
105467	NTHI477_RS00480	681	T	C	synonymous
105470	NTHI477_RS00480	684	C	T	synonymous
105479	NTHI477_RS00480	693	T	C	synonymous
105482	NTHI477_RS00480	696	A	T	synonymous
105500	NTHI477_RS00480	714	T	C	synonymous
105512	NTHI477_RS00480	726	T	A	synonymous
105515	NTHI477_RS00480	729	G	A	synonymous
105530	NTHI477_RS00480	744	T	C	synonymous
105542	NTHI477_RS00480	756	C	T	synonymous
105548	NTHI477_RS00480	762	T	G	synonymous
105602	NTHI477_RS00480	816	A	G	synonymous
105626	NTHI477_RS00480	840	A	G	synonymous
105647	NTHI477_RS00480	861	C	T	synonymous
105665	NTHI477_RS00480	879	G	A	nonsynonymous
105668	NTHI477_RS00480	882	C	T	synonymous
105689	NTHI477_RS00480	903	C	A	synonymous
105713	NTHI477_RS00480	927	A	T	synonymous
105737	NTHI477_RS00480	951	A	G	synonymous
105740	NTHI477_RS00480	954	C	T	synonymous
105746	NTHI477_RS00480	960	C	T	synonymous
105752	NTHI477_RS00480	966	C	T	synonymous
105767	NTHI477_RS00480	981	T	C	synonymous
105773	NTHI477_RS00480	987	G	A	synonymous

105782	NTHI477_RS00480	996	G	A	synonymous
105785	NTHI477_RS00480	999	G	A	nonsynonymous
105804	NTHI477_RS00480	1018	C	T	synonymous
105824	NTHI477_RS00480	1038	C	G	synonymous
105842	NTHI477_RS00480	1056	G	A	synonymous
105845	NTHI477_RS00480	1059	G	A	synonymous
105848	NTHI477_RS00480	1062	G	T	synonymous
105879	intergenic	intergenic	C	G	intergenic
105885	intergenic	intergenic	A	G	intergenic
105887	intergenic	intergenic	T	C	intergenic
105895	intergenic	intergenic	T	C	intergenic
105898	intergenic	intergenic	T	C	intergenic
105899	intergenic	intergenic	T	C	intergenic
105910	intergenic	intergenic	C	A	intergenic
105912	intergenic	intergenic	G	A	intergenic
105914	intergenic	intergenic	G	A	intergenic
105927	NTHI477_RS00485	6	C	T	synonymous
105927	NTHI477_RS00485	6	C	G	nonsynonymous
105961	NTHI477_RS00485	40	G	A	nonsynonymous
105967	NTHI477_RS00485	46	A	C	nonsynonymous
105969	NTHI477_RS00485	48	A	G	synonymous
105970	NTHI477_RS00485	49	C	T	nonsynonymous
105972	NTHI477_RS00485	51	T	A	synonymous
105976	NTHI477_RS00485	55	C	A	nonsynonymous
105981	NTHI477_RS00485	60	T	C	synonymous
105982	NTHI477_RS00485	61	G	A	nonsynonymous
105983	NTHI477_RS00485	62	C	T	nonsynonymous
105990	NTHI477_RS00485	69	A	C	synonymous
106005	NTHI477_RS00485	84	T	C	synonymous
106009	NTHI477_RS00485	88	C	A	nonsynonymous
106014	NTHI477_RS00485	93	A	C	synonymous
106014	NTHI477_RS00485	93	A	G	synonymous
106014	NTHI477_RS00485	93	A	T	synonymous
106030	NTHI477_RS00485	109	C	A	nonsynonymous
106035	NTHI477_RS00485	114	G	A	synonymous
106040	NTHI477_RS00485	119	G	C	nonsynonymous
106042	NTHI477_RS00485	121	G	C	nonsynonymous
106049	NTHI477_RS00485	128	T	C	nonsynonymous
106054	NTHI477_RS00485	133	C	A	nonsynonymous
106065	NTHI477_RS00485	144	A	C	synonymous
106068	NTHI477_RS00485	147	G	A	synonymous
106071	NTHI477_RS00485	150	G	A	synonymous
106080	NTHI477_RS00485	159	C	T	synonymous
106094	NTHI477_RS00485	173	T	C	nonsynonymous
106095	NTHI477_RS00485	174	G	T	nonsynonymous

106098	NTHI477_RS00485	177	T	G	nonsynonymous
106105	NTHI477_RS00485	184	G	A	nonsynonymous
106106	NTHI477_RS00485	185	C	T	nonsynonymous
106107	NTHI477_RS00485	186	T	A	synonymous
106125	NTHI477_RS00485	204	C	T	synonymous
106129	NTHI477_RS00485	208	A	T	nonsynonymous
106134	NTHI477_RS00485	213	T	C	synonymous
106154	NTHI477_RS00485	233	A	G	nonsynonymous
106167	NTHI477_RS00485	246	C	T	synonymous
106173	NTHI477_RS00485	252	C	T	synonymous
106190	NTHI477_RS00485	269	C	T	nonsynonymous
106197	NTHI477_RS00485	276	G	T	nonsynonymous
106221	NTHI477_RS00485	300	T	C	synonymous
106224	NTHI477_RS00485	303	C	T	synonymous
106245	NTHI477_RS00485	324	A	G	synonymous
106248	NTHI477_RS00485	327	G	A	synonymous
106251	NTHI477_RS00485	330	A	G	synonymous
106257	NTHI477_RS00485	336	A	G	synonymous
106293	NTHI477_RS00485	372	T	G	synonymous
106299	NTHI477_RS00485	378	T	A	synonymous
106299	NTHI477_RS00485	378	T	C	synonymous
106305	NTHI477_RS00485	384	T	C	synonymous
106308	NTHI477_RS00485	387	C	T	synonymous
106320	NTHI477_RS00485	399	G	T	synonymous
106341	NTHI477_RS00485	420	T	A	synonymous
106341	NTHI477_RS00485	420	T	G	nonsynonymous
106359	NTHI477_RS00485	438	A	G	synonymous
106380	NTHI477_RS00490	19	C	T	synonymous
106382	NTHI477_RS00490	21	G	A	synonymous
106391	NTHI477_RS00490	30	A	G	synonymous
106392	NTHI477_RS00490	31	A	G	nonsynonymous
106393	NTHI477_RS00490	32	T	A	nonsynonymous
106394	NTHI477_RS00490	33	G	T	nonsynonymous
106395	NTHI477_RS00490	34	G	T	nonsynonymous
106396	NTHI477_RS00490	35	C	T	nonsynonymous
106397	NTHI477_RS00490	36	G	A	synonymous
106403	NTHI477_RS00490	42	A	G	synonymous
106418	NTHI477_RS00490	57	C	T	synonymous
106419	NTHI477_RS00490	58	A	T	nonsynonymous
106442	NTHI477_RS00490	81	A	G	synonymous
106458	NTHI477_RS00490	97	C	T	nonsynonymous
106460	NTHI477_RS00490	99	T	C	synonymous
106462	NTHI477_RS00490	101	C	T	nonsynonymous
106469	NTHI477_RS00490	108	C	T	synonymous
106473	NTHI477_RS00490	112	T	C	nonsynonymous

106474	NTHI477_RS00490	113	C	T	nonsynonymous
106477	NTHI477_RS00490	116	G	A	nonsynonymous
106491	NTHI477_RS00490	130	G	A	nonsynonymous
106505	NTHI477_RS00490	144	C	T	synonymous
106525	NTHI477_RS00490	164	G	A	nonsynonymous
106557	NTHI477_RS00490	196	T	C	synonymous
106577	NTHI477_RS00490	216	C	T	synonymous
106589	NTHI477_RS00490	228	C	T	synonymous
106607	NTHI477_RS00490	246	A	G	synonymous
106610	NTHI477_RS00490	249	G	A	synonymous
106613	NTHI477_RS00490	252	A	G	synonymous
106613	NTHI477_RS00490	252	A	C	synonymous
106622	NTHI477_RS00490	261	C	T	synonymous
106629	NTHI477_RS00490	268	G	A	nonsynonymous
106631	NTHI477_RS00490	270	C	T	synonymous
106634	NTHI477_RS00490	273	A	C	synonymous
106644	NTHI477_RS00490	283	A	C	synonymous
106652	NTHI477_RS00490	291	C	T	synonymous
106655	NTHI477_RS00490	294	G	T	synonymous
106655	NTHI477_RS00490	294	G	A	synonymous
106659	NTHI477_RS00490	298	A	G	nonsynonymous
106660	NTHI477_RS00490	299	T	G	nonsynonymous
106661	NTHI477_RS00490	300	T	A	synonymous
106662	NTHI477_RS00490	301	T	C	synonymous
106664	NTHI477_RS00490	303	A	C	nonsynonymous
106664	NTHI477_RS00490	303	A	G	synonymous
106667	NTHI477_RS00490	306	G	A	synonymous
106670	NTHI477_RS00490	309	A	G	synonymous
106673	NTHI477_RS00490	312	A	G	synonymous
106686	NTHI477_RS00490	325	G	T	nonsynonymous
106688	NTHI477_RS00490	327	G	T	synonymous
106691	NTHI477_RS00490	330	A	G	synonymous
106698	NTHI477_RS00490	337	C	T	synonymous
106704	NTHI477_RS00490	343	G	C	nonsynonymous
106706	NTHI477_RS00490	345	A	G	synonymous
106716	NTHI477_RS00490	355	C	T	nonsynonymous
106722	NTHI477_RS00490	361	C	T	nonsynonymous
106736	NTHI477_RS00490	375	T	C	synonymous
106739	NTHI477_RS00490	378	T	C	synonymous
106739	NTHI477_RS00490	378	T	A	synonymous
106742	NTHI477_RS00490	381	A	T	synonymous
106745	NTHI477_RS00490	384	C	T	synonymous
106754	NTHI477_RS00490	393	A	T	synonymous
106757	NTHI477_RS00490	396	C	T	synonymous
106760	NTHI477_RS00490	399	C	G	synonymous

106761	NTHI477_RS00490	400	C	T	nonsynonymous
106763	NTHI477_RS00490	402	C	A	synonymous
106763	NTHI477_RS00490	402	C	G	synonymous
106766	NTHI477_RS00490	405	T	A	synonymous
106769	NTHI477_RS00490	408	G	A	synonymous
106772	NTHI477_RS00490	411	T	A	synonymous
106775	NTHI477_RS00490	414	C	G	synonymous
106782	NTHI477_RS00490	421	G	T	nonsynonymous
106783	NTHI477_RS00490	422	C	T	nonsynonymous
106783	NTHI477_RS00490	422	C	A	nonsynonymous
106784	NTHI477_RS00490	423	G	A	synonymous
106784	NTHI477_RS00490	423	G	C	synonymous
106785	NTHI477_RS00490	424	C	T	nonsynonymous
106787	NTHI477_RS00490	426	C	T	synonymous
106788	NTHI477_RS00490	427	A	G	nonsynonymous
106793	NTHI477_RS00490	432	C	T	synonymous
106799	NTHI477_RS00490	438	G	A	synonymous
106802	NTHI477_RS00490	441	G	A	synonymous
106804	NTHI477_RS00490	443	A	T	nonsynonymous
106805	NTHI477_RS00490	444	T	A	nonsynonymous
106806	NTHI477_RS00490	445	A	C	nonsynonymous
106809	NTHI477_RS00490	448	C	T	nonsynonymous
106811	NTHI477_RS00490	450	G	A	synonymous
106812	NTHI477_RS00490	451	C	T	synonymous
106814	NTHI477_RS00490	453	G	A	synonymous
106820	NTHI477_RS00490	459	A	C	synonymous
106829	NTHI477_RS00490	468	G	T	synonymous
106839	NTHI477_RS00490	478	C	T	nonsynonymous
106850	NTHI477_RS00490	489	G	A	synonymous
106853	NTHI477_RS00490	492	A	G	synonymous
106861	NTHI477_RS00490	500	A	G	nonsynonymous
106892	NTHI477_RS00490	531	C	A	synonymous
106893	NTHI477_RS00490	532	C	G	nonsynonymous
106908	NTHI477_RS00490	547	T	C	nonsynonymous
106908	NTHI477_RS00490	547	T	A	nonsynonymous
106932	NTHI477_RS00490	571	A	C	nonsynonymous
106972	NTHI477_RS00490	611	C	T	nonsynonymous
107003	NTHI477_RS00490	642	G	A	synonymous
107015	NTHI477_RS00490	654	G	C	synonymous
107015	NTHI477_RS00490	654	G	T	synonymous
107030	NTHI477_RS00490	669	T	G	synonymous
107033	NTHI477_RS00490	672	C	G	synonymous
107041	NTHI477_RS00490	680	A	C	nonsynonymous
107042	NTHI477_RS00490	681	G	A	synonymous
107045	NTHI477_RS00490	684	G	T	synonymous

107061	NTHI477_RS00490	700	C	T	nonsynonymous
107074	NTHI477_RS00490	713	T	C	nonsynonymous
107079	NTHI477_RS00490	718	T	C	nonsynonymous
107083	NTHI477_RS00490	722	G	A	nonsynonymous
107095	NTHI477_RS00490	734	C	T	nonsynonymous
107103	NTHI477_RS00490	742	G	A	nonsynonymous
107104	NTHI477_RS00490	743	T	C	nonsynonymous
107120	NTHI477_RS00490	759	C	T	synonymous
107135	NTHI477_RS00490	774	C	A	synonymous
107158	NTHI477_RS00490	797	A	T	nonsynonymous
107166	NTHI477_RS00490	805	C	T	nonsynonymous
107186	NTHI477_RS00490	825	T	C	synonymous
107199	NTHI477_RS00490	838	G	A	nonsynonymous
107213	NTHI477_RS00490	852	C	T	synonymous
107214	NTHI477_RS00490	853	G	A	nonsynonymous
107253	NTHI477_RS00495	14	G	A	nonsynonymous
107260	NTHI477_RS00495	21	A	G	synonymous
107270	NTHI477_RS00495	31	A	C	nonsynonymous
107276	NTHI477_RS00495	37	G	A	nonsynonymous
107299	NTHI477_RS00495	60	T	A	synonymous
107311	NTHI477_RS00495	72	C	T	synonymous
107313	NTHI477_RS00495	74	C	T	nonsynonymous
107321	NTHI477_RS00495	82	C	A	nonsynonymous
107334	NTHI477_RS00495	95	A	C	nonsynonymous
107338	NTHI477_RS00495	99	G	T	nonsynonymous
107341	NTHI477_RS00495	102	T	G	synonymous
107341	NTHI477_RS00495	102	T	A	synonymous
107350	NTHI477_RS00495	111	G	A	synonymous
107366	NTHI477_RS00495	127	A	G	nonsynonymous
107384	NTHI477_RS00495	145	C	T	nonsynonymous
107393	NTHI477_RS00495	154	G	A	nonsynonymous
107395	NTHI477_RS00495	156	G	A	synonymous
107405	NTHI477_RS00495	166	C	T	nonsynonymous
107407	NTHI477_RS00495	168	G	T	nonsynonymous
107410	NTHI477_RS00495	171	A	G	synonymous
107416	NTHI477_RS00495	177	G	A	synonymous
107425	NTHI477_RS00495	186	T	C	synonymous
107428	NTHI477_RS00495	189	T	C	synonymous
107441	NTHI477_RS00495	202	C	T	nonsynonymous
107442	NTHI477_RS00495	203	A	G	nonsynonymous
107473	NTHI477_RS00495	234	C	T	synonymous
107498	NTHI477_RS00495	259	G	C	nonsynonymous
107501	NTHI477_RS00495	262	C	A	nonsynonymous
107506	NTHI477_RS00495	267	T	C	synonymous
107512	NTHI477_RS00495	273	A	T	synonymous

107533	NTHI477_RS00495	294	A	G	synonymous
107533	NTHI477_RS00495	294	A	T	synonymous
107536	NTHI477_RS00495	297	G	A	nonsynonymous
107540	NTHI477_RS00495	301	T	C	nonsynonymous
107554	NTHI477_RS00495	315	G	C	nonsynonymous
107556	NTHI477_RS00495	317	C	T	nonsynonymous
107560	NTHI477_RS00495	321	G	A	synonymous
107566	NTHI477_RS00495	327	G	A	synonymous
107569	NTHI477_RS00495	330	A	G	synonymous
107575	NTHI477_RS00495	336	C	T	synonymous
107578	NTHI477_RS00495	339	A	T	synonymous
107581	NTHI477_RS00495	342	A	C	synonymous
107587	NTHI477_RS00495	348	C	T	synonymous
107593	NTHI477_RS00495	354	A	G	synonymous
107597	NTHI477_RS00495	358	T	C	synonymous
107599	NTHI477_RS00495	360	A	C	nonsynonymous
107611	NTHI477_RS00495	372	T	C	synonymous
107615	NTHI477_RS00495	376	G	A	nonsynonymous
107617	NTHI477_RS00495	378	T	A	synonymous
107617	NTHI477_RS00495	378	T	C	synonymous
107662	NTHI477_RS00495	423	T	A	synonymous
107665	NTHI477_RS00495	426	A	G	synonymous
107677	NTHI477_RS00495	438	G	A	synonymous
107704	NTHI477_RS00495	465	G	C	synonymous
107704	NTHI477_RS00495	465	G	A	synonymous
107713	NTHI477_RS00495	474	T	G	synonymous
107726	NTHI477_RS00495	487	G	A	nonsynonymous
107743	NTHI477_RS00495	504	C	T	synonymous
107824	NTHI477_RS00495	585	C	T	synonymous
107875	NTHI477_RS00495	636	C	T	synonymous
107881	NTHI477_RS00495	642	C	T	synonymous
107884	NTHI477_RS00495	645	T	C	synonymous
107887	NTHI477_RS00495	648	C	T	synonymous
107890	NTHI477_RS00495	651	A	G	synonymous
107896	NTHI477_RS00495	657	A	G	synonymous
107902	NTHI477_RS00495	663	A	G	synonymous
107976	NTHI477_RS00495	737	C	T	nonsynonymous
108013	NTHI477_RS00495	774	G	A	synonymous
108061	NTHI477_RS00500	4	C	A	nonsynonymous
108094	NTHI477_RS00500	37	G	A	nonsynonymous
108102	NTHI477_RS00500	45	C	T	synonymous
108114	NTHI477_RS00500	57	T	C	synonymous
108135	NTHI477_RS00500	78	C	T	synonymous
108138	NTHI477_RS00500	81	G	T	synonymous
108138	NTHI477_RS00500	81	G	C	synonymous

108139	NTHI477_RS00500	82	C	T	synonymous
108141	NTHI477_RS00500	84	G	T	synonymous
108147	NTHI477_RS00500	90	C	T	synonymous
108159	NTHI477_RS00500	102	A	G	synonymous
108175	NTHI477_RS00500	118	G	T	nonsynonymous
108177	NTHI477_RS00500	120	T	C	synonymous
108191	NTHI477_RS00500	134	C	T	nonsynonymous
108192	NTHI477_RS00500	135	A	G	synonymous
108195	NTHI477_RS00500	138	G	A	synonymous
108199	NTHI477_RS00500	142	C	T	nonsynonymous
108201	NTHI477_RS00500	144	T	G	synonymous
108216	NTHI477_RS00500	159	A	G	synonymous
108219	NTHI477_RS00500	162	T	C	synonymous
108222	NTHI477_RS00500	165	A	G	synonymous
108225	NTHI477_RS00500	168	G	T	synonymous
108246	NTHI477_RS00500	189	T	C	synonymous
108249	NTHI477_RS00500	192	A	G	synonymous
108270	NTHI477_RS00500	213	A	C	synonymous
108273	NTHI477_RS00500	216	T	A	synonymous
108276	NTHI477_RS00500	219	C	A	synonymous
108288	NTHI477_RS00500	231	A	C	synonymous
108309	NTHI477_RS00500	252	A	G	synonymous
108315	NTHI477_RS00500	258	T	G	nonsynonymous
108318	NTHI477_RS00500	261	A	G	synonymous
108318	NTHI477_RS00500	261	A	C	synonymous
108321	NTHI477_RS00500	264	C	T	synonymous
108324	NTHI477_RS00500	267	G	T	synonymous
108339	NTHI477_RS00500	282	C	T	synonymous
108363	NTHI477_RS00500	306	T	C	synonymous
108369	NTHI477_RS00500	312	C	T	synonymous
108375	NTHI477_RS00500	318	T	A	synonymous
108381	NTHI477_RS00500	324	T	G	synonymous
108384	NTHI477_RS00500	327	C	A	synonymous
108384	NTHI477_RS00500	327	C	G	synonymous
108388	NTHI477_RS00500	331	A	G	nonsynonymous
108390	NTHI477_RS00500	333	T	G	nonsynonymous
108397	NTHI477_RS00500	340	C	T	nonsynonymous
108399	NTHI477_RS00500	342	T	A	synonymous
108402	NTHI477_RS00500	345	C	A	synonymous
108402	NTHI477_RS00500	345	C	G	synonymous
108405	NTHI477_RS00500	348	C	A	synonymous
108408	NTHI477_RS00500	351	T	C	synonymous
108414	NTHI477_RS00500	357	G	T	synonymous
108424	NTHI477_RS00500	367	G	T	nonsynonymous
108451	NTHI477_RS00500	394	G	C	nonsynonymous

108456	NTHI477_RS00500	399	A	T	synonymous
108459	NTHI477_RS00500	402	G	A	synonymous
108472	NTHI477_RS00500	415	A	T	nonsynonymous
108481	NTHI477_RS00500	424	T	A	nonsynonymous
108492	NTHI477_RS00500	435	A	T	synonymous
108493	NTHI477_RS00500	436	G	A	nonsynonymous
108502	NTHI477_RS00500	445	G	A	nonsynonymous
108504	NTHI477_RS00500	447	C	T	synonymous
108510	NTHI477_RS00500	453	C	T	synonymous
108511	NTHI477_RS00500	454	G	A	nonsynonymous
108531	NTHI477_RS00500	474	T	C	synonymous
108534	NTHI477_RS00500	477	T	C	synonymous
108537	NTHI477_RS00500	480	T	C	synonymous
108555	NTHI477_RS00500	498	T	C	synonymous
108561	NTHI477_RS00500	504	T	C	synonymous
108564	NTHI477_RS00500	507	A	T	synonymous
108570	NTHI477_RS00500	513	T	C	synonymous
108576	NTHI477_RS00500	519	T	A	synonymous
108594	NTHI477_RS00500	537	G	A	synonymous
108615	NTHI477_RS00500	558	A	T	synonymous
108627	NTHI477_RS00500	570	C	A	synonymous
108642	NTHI477_RS00500	585	T	A	synonymous
108663	NTHI477_RS00500	606	T	C	synonymous
108693	NTHI477_RS00500	636	A	G	synonymous
108699	NTHI477_RS00500	642	G	A	synonymous
108700	NTHI477_RS00500	643	A	G	nonsynonymous
108705	NTHI477_RS00500	648	T	C	synonymous
108711	NTHI477_RS00500	654	T	C	synonymous
108714	NTHI477_RS00500	657	G	A	synonymous
108723	NTHI477_RS00500	666	C	G	synonymous
108726	NTHI477_RS00500	669	A	G	synonymous
108727	NTHI477_RS00500	670	T	C	synonymous
108729	NTHI477_RS00500	672	A	C	nonsynonymous
108732	NTHI477_RS00500	675	G	A	synonymous
108747	NTHI477_RS00500	690	A	G	synonymous
108747	NTHI477_RS00500	690	A	T	synonymous
108750	NTHI477_RS00500	693	A	C	synonymous
108753	NTHI477_RS00500	696	C	A	synonymous
108768	NTHI477_RS00500	711	C	T	synonymous
108777	NTHI477_RS00500	720	A	G	synonymous
108798	NTHI477_RS00500	741	A	G	synonymous
108813	NTHI477_RS00500	756	T	A	synonymous
108816	NTHI477_RS00500	759	T	A	synonymous
108819	NTHI477_RS00500	762	A	G	synonymous
108822	NTHI477_RS00500	765	G	A	synonymous

108834	NTHI477_RS00500	777	G	T	synonymous
108840	NTHI477_RS00500	783	A	C	nonsynonymous
108843	NTHI477_RS00500	786	C	T	synonymous
108849	NTHI477_RS00500	792	T	G	synonymous
108849	NTHI477_RS00500	792	T	C	synonymous
108851	NTHI477_RS00500	794	C	T	nonsynonymous
108852	NTHI477_RS00500	795	C	T	synonymous
108860	NTHI477_RS00500	803	A	G	nonsynonymous
108862	NTHI477_RS00500	805	G	T	nonsynonymous
108864	NTHI477_RS00500	807	C	G	synonymous
108867	NTHI477_RS00500	810	C	T	synonymous
108870	NTHI477_RS00500	813	T	C	synonymous
108888	NTHI477_RS00500	831	T	C	synonymous
108894	NTHI477_RS00500	837	A	G	synonymous
108903	NTHI477_RS00500	846	C	T	synonymous
109018	NTHI477_RS00505	47	C	T	nonsynonymous
109061	NTHI477_RS00505	90	T	G	nonsynonymous
109075	NTHI477_RS00505	104	C	T	nonsynonymous
109088	NTHI477_RS00505	117	G	A	synonymous
109096	NTHI477_RS00505	125	A	G	nonsynonymous
109097	NTHI477_RS00505	126	G	A	synonymous
109103	NTHI477_RS00505	132	G	A	synonymous
109106	NTHI477_RS00505	135	C	T	synonymous
109112	NTHI477_RS00505	141	T	G	synonymous
109127	NTHI477_RS00505	156	G	C	synonymous
109127	NTHI477_RS00505	156	G	T	synonymous
109139	NTHI477_RS00505	168	T	A	synonymous
109145	NTHI477_RS00505	174	T	G	synonymous
109148	NTHI477_RS00505	177	G	A	synonymous
109149	NTHI477_RS00505	178	C	A	nonsynonymous
109155	NTHI477_RS00505	184	C	T	synonymous
109157	NTHI477_RS00505	186	G	A	synonymous
109160	NTHI477_RS00505	189	A	C	synonymous
109160	NTHI477_RS00505	189	A	T	synonymous
109163	NTHI477_RS00505	192	A	G	synonymous
109170	NTHI477_RS00505	199	C	T	synonymous
109172	NTHI477_RS00505	201	G	A	synonymous
109178	NTHI477_RS00505	207	G	C	synonymous
109187	NTHI477_RS00505	216	G	A	synonymous
109190	NTHI477_RS00505	219	A	T	synonymous
109214	NTHI477_RS00505	243	G	A	synonymous
109220	NTHI477_RS00505	249	A	C	synonymous
109221	NTHI477_RS00505	250	G	A	nonsynonymous
109232	NTHI477_RS00505	261	C	A	synonymous
109232	NTHI477_RS00505	261	C	T	synonymous

109235	NTHI477_RS00505	264	C	T	synonymous
109238	NTHI477_RS00505	267	C	T	synonymous
109265	NTHI477_RS00505	294	T	C	synonymous
109268	NTHI477_RS00505	297	A	G	synonymous
109271	NTHI477_RS00505	300	C	A	synonymous
109271	NTHI477_RS00505	300	C	T	synonymous
109274	NTHI477_RS00505	303	G	A	synonymous
109283	NTHI477_RS00505	312	C	T	synonymous
109286	NTHI477_RS00505	315	A	T	synonymous
109289	NTHI477_RS00505	318	C	G	nonsynonymous
109289	NTHI477_RS00505	318	C	T	synonymous
109292	NTHI477_RS00505	321	T	A	synonymous
109293	NTHI477_RS00505	322	C	T	synonymous
109295	NTHI477_RS00505	324	G	A	synonymous
109301	NTHI477_RS00505	330	T	C	synonymous
109302	NTHI477_RS00505	331	T	G	nonsynonymous
109313	NTHI477_RS00505	342	C	T	synonymous
109319	NTHI477_RS00505	348	G	A	synonymous
109322	NTHI477_RS00505	351	A	T	synonymous
109332	NTHI477_RS00505	361	C	T	nonsynonymous
109332	NTHI477_RS00505	361	C	G	nonsynonymous
109334	NTHI477_RS00505	363	C	T	synonymous
109342	NTHI477_RS00505	371	C	G	nonsynonymous
109356	NTHI477_RS00505	385	G	T	nonsynonymous
109356	NTHI477_RS00505	385	G	A	nonsynonymous
109364	NTHI477_RS00505	393	C	T	synonymous
109391	NTHI477_RS00505	420	A	C	synonymous
109406	NTHI477_RS00505	435	C	T	synonymous
109412	NTHI477_RS00505	441	A	G	synonymous
109445	NTHI477_RS00505	474	A	G	synonymous
109463	NTHI477_RS00505	492	G	A	synonymous
109466	NTHI477_RS00505	495	T	G	synonymous
109472	NTHI477_RS00505	501	C	T	synonymous
109472	NTHI477_RS00505	501	C	A	synonymous
109475	NTHI477_RS00505	504	G	A	synonymous
109505	NTHI477_RS00505	534	A	G	synonymous
109511	NTHI477_RS00505	540	T	C	synonymous
109532	NTHI477_RS00505	561	T	C	synonymous
109538	NTHI477_RS00505	567	T	C	synonymous
109605	NTHI477_RS00505	634	G	A	nonsynonymous
109617	NTHI477_RS00505	646	A	G	nonsynonymous
109618	NTHI477_RS00505	647	C	T	nonsynonymous
109631	NTHI477_RS00505	660	C	T	synonymous
109664	NTHI477_RS00505	693	A	T	synonymous
109715	NTHI477_RS00505	744	G	A	synonymous

109719	NTHI477_RS00505	748	C	T	nonsynonymous
109736	NTHI477_RS00505	765	T	C	synonymous
109790	NTHI477_RS00505	819	T	C	synonymous
109793	NTHI477_RS00505	822	A	C	synonymous
109798	NTHI477_RS00505	827	C	T	nonsynonymous
109799	NTHI477_RS00505	828	G	A	synonymous
109799	NTHI477_RS00505	828	G	T	synonymous
109820	NTHI477_RS00505	849	A	C	synonymous
109820	NTHI477_RS00505	849	A	T	synonymous
109823	NTHI477_RS00505	852	A	G	synonymous
109841	NTHI477_RS00505	870	T	C	synonymous
109850	NTHI477_RS00505	879	A	G	synonymous
109853	NTHI477_RS00505	882	G	A	synonymous
109868	NTHI477_RS00505	897	C	T	synonymous
109886	NTHI477_RS00505	915	C	T	synonymous
109900	NTHI477_RS00505	929	A	T	nonsynonymous
109901	NTHI477_RS00505	930	A	G	synonymous
109908	NTHI477_RS00505	937	C	A	nonsynonymous
109911	NTHI477_RS00505	940	C	T	nonsynonymous
109927	NTHI477_RS00510	9	T	C	synonymous
109936	NTHI477_RS00510	18	T	C	synonymous
109937	NTHI477_RS00510	19	C	T	nonsynonymous
109945	NTHI477_RS00510	27	T	C	synonymous
109948	NTHI477_RS00510	30	A	G	synonymous
109948	NTHI477_RS00510	30	A	C	synonymous
109949	NTHI477_RS00510	31	C	T	synonymous
109951	NTHI477_RS00510	33	A	G	synonymous
109966	NTHI477_RS00510	48	G	A	synonymous
109981	NTHI477_RS00510	63	T	C	synonymous
109988	NTHI477_RS00510	70	C	A	synonymous
109996	NTHI477_RS00510	78	A	T	synonymous
109996	NTHI477_RS00510	78	A	G	synonymous
109996	NTHI477_RS00510	78	A	C	synonymous
109999	NTHI477_RS00510	81	T	A	synonymous
110011	NTHI477_RS00510	93	C	T	synonymous
110012	NTHI477_RS00510	94	C	T	synonymous
110014	NTHI477_RS00510	96	G	A	synonymous
110020	NTHI477_RS00510	102	T	A	synonymous
110026	NTHI477_RS00510	108	A	G	synonymous
110029	NTHI477_RS00510	111	T	A	synonymous
110030	NTHI477_RS00510	112	G	T	nonsynonymous
110032	NTHI477_RS00510	114	G	T	synonymous
110047	NTHI477_RS00510	129	C	T	synonymous
110051	NTHI477_RS00510	133	C	T	nonsynonymous
110056	NTHI477_RS00510	138	G	T	synonymous

110068	NTHI477_RS00510	150	A	T	synonymous
110074	NTHI477_RS00510	156	A	G	synonymous
110075	NTHI477_RS00510	157	G	A	nonsynonymous
110076	NTHI477_RS00510	158	G	A	nonsynonymous
110096	NTHI477_RS00510	178	C	T	synonymous
110104	NTHI477_RS00510	186	C	T	synonymous
110113	NTHI477_RS00510	195	T	C	synonymous
110116	NTHI477_RS00510	198	G	A	synonymous
110122	NTHI477_RS00510	204	G	C	synonymous
110125	NTHI477_RS00510	207	C	T	synonymous
110126	NTHI477_RS00510	208	G	A	nonsynonymous
110131	NTHI477_RS00510	213	A	T	nonsynonymous
110131	NTHI477_RS00510	213	A	G	synonymous
110137	NTHI477_RS00510	219	T	C	synonymous
110146	NTHI477_RS00510	228	A	G	synonymous
110155	NTHI477_RS00510	237	G	A	synonymous
110156	NTHI477_RS00510	238	C	T	synonymous
110179	NTHI477_RS00510	261	C	T	synonymous
110191	NTHI477_RS00510	273	G	T	synonymous
110224	NTHI477_RS00510	306	G	A	synonymous
110225	NTHI477_RS00510	307	G	A	nonsynonymous
110236	NTHI477_RS00510	318	A	G	synonymous
110251	NTHI477_RS00510	333	T	C	synonymous
110254	NTHI477_RS00510	336	A	T	synonymous
110265	NTHI477_RS00510	347	C	T	nonsynonymous
110290	NTHI477_RS00510	372	A	T	nonsynonymous
110299	NTHI477_RS00510	381	T	G	nonsynonymous
110314	NTHI477_RS00510	396	T	C	synonymous
110323	NTHI477_RS00510	405	G	A	synonymous
110329	NTHI477_RS00510	411	A	T	nonsynonymous
110344	NTHI477_RS00510	426	G	A	synonymous
110350	NTHI477_RS00510	432	T	C	synonymous
110353	NTHI477_RS00510	435	T	C	synonymous
110360	NTHI477_RS00510	442	C	T	synonymous
110365	NTHI477_RS00510	447	G	A	synonymous
110378	NTHI477_RS00510	460	G	A	nonsynonymous
110416	NTHI477_RS00510	498	A	C	nonsynonymous
110431	NTHI477_RS00510	513	A	C	synonymous
110434	NTHI477_RS00510	516	A	C	synonymous
110437	NTHI477_RS00510	519	C	T	synonymous
110443	NTHI477_RS00510	525	G	C	synonymous
110461	NTHI477_RS00510	543	A	G	synonymous
110487	NTHI477_RS00510	569	G	A	nonsynonymous
110492	NTHI477_RS00510	574	G	A	nonsynonymous
110503	NTHI477_RS00510	585	T	A	synonymous

110503	NTHI477_RS00510	585	T	G	synonymous
110504	NTHI477_RS00510	586	G	A	nonsynonymous
110509	NTHI477_RS00510	591	C	T	synonymous
110515	NTHI477_RS00510	597	C	T	synonymous
110560	NTHI477_RS00510	642	T	G	synonymous
110568	NTHI477_RS00510	650	C	T	nonsynonymous
110569	NTHI477_RS00510	651	G	A	synonymous
110588	NTHI477_RS00510	670	C	T	synonymous
110624	NTHI477_RS00510	706	C	T	nonsynonymous
110630	NTHI477_RS00510	712	C	T	nonsynonymous
110632	NTHI477_RS00510	714	G	A	synonymous
110647	NTHI477_RS00510	729	A	G	synonymous
110668	NTHI477_RS00510	750	C	A	synonymous
110683	NTHI477_RS00510	765	A	G	synonymous
110686	NTHI477_RS00510	768	G	A	synonymous
110710	NTHI477_RS00510	792	T	A	synonymous
110710	NTHI477_RS00510	792	T	G	synonymous
110728	NTHI477_RS00510	810	T	C	synonymous
110734	NTHI477_RS00510	816	C	T	synonymous
110740	NTHI477_RS00510	822	G	T	synonymous
110755	NTHI477_RS00510	837	C	T	synonymous
110755	NTHI477_RS00510	837	C	G	synonymous
110758	NTHI477_RS00510	840	C	T	synonymous
110761	NTHI477_RS00510	843	T	A	synonymous
110764	NTHI477_RS00510	846	A	G	synonymous
110770	NTHI477_RS00510	852	G	A	synonymous
110803	NTHI477_RS00510	885	T	C	synonymous
110815	NTHI477_RS00510	897	A	G	synonymous
110830	NTHI477_RS00510	912	C	T	synonymous
110836	NTHI477_RS00510	918	G	T	synonymous
110842	NTHI477_RS00510	924	A	G	synonymous
110848	NTHI477_RS00510	930	C	T	synonymous
110887	NTHI477_RS00510	969	A	G	synonymous
110890	NTHI477_RS00510	972	C	A	synonymous
110902	NTHI477_RS00510	984	A	G	synonymous
110905	NTHI477_RS00510	987	T	C	synonymous
110913	NTHI477_RS00510	995	C	T	nonsynonymous
110917	NTHI477_RS00510	999	G	C	synonymous
110923	NTHI477_RS00510	1005	T	C	synonymous
110929	NTHI477_RS00510	1011	G	A	synonymous
110932	NTHI477_RS00510	1014	C	G	nonsynonymous
110953	NTHI477_RS00510	1035	A	T	synonymous
110965	NTHI477_RS00510	1047	T	C	synonymous
110968	NTHI477_RS00510	1050	A	G	synonymous
110977	NTHI477_RS00510	1059	A	G	synonymous

111004	NTHI477_RS00510	1086	C	T	synonymous
111007	NTHI477_RS00510	1089	T	A	synonymous
111009	NTHI477_RS00510	1091	G	T	nonsynonymous
111016	NTHI477_RS00510	1098	G	C	synonymous
111022	NTHI477_RS00510	1104	A	G	synonymous
111028	NTHI477_RS00510	1110	C	T	synonymous
111031	NTHI477_RS00510	1113	G	A	synonymous
111037	NTHI477_RS00510	1119	A	T	synonymous
111040	NTHI477_RS00510	1122	C	A	synonymous
111046	NTHI477_RS00510	1128	T	C	synonymous
111049	NTHI477_RS00510	1131	T	C	synonymous
111058	NTHI477_RS00510	1140	A	T	synonymous
111061	NTHI477_RS00510	1143	T	G	synonymous
111085	NTHI477_RS00510	1167	T	C	synonymous
111089	NTHI477_RS00510	1171	A	C	synonymous
111097	NTHI477_RS00510	1179	A	G	synonymous
111129	NTHI477_RS00515	15	A	T	synonymous
111219	NTHI477_RS00515	105	T	C	synonymous
111222	NTHI477_RS00515	108	G	A	synonymous
111231	NTHI477_RS00515	117	T	C	synonymous
111261	NTHI477_RS00515	147	T	G	synonymous
111279	NTHI477_RS00515	165	G	A	synonymous
111288	NTHI477_RS00515	174	T	C	synonymous
111330	NTHI477_RS00515	216	A	G	synonymous
111333	NTHI477_RS00515	219	A	G	synonymous
111378	NTHI477_RS00515	264	T	A	synonymous
111462	NTHI477_RS00515	348	A	T	synonymous
111474	NTHI477_RS00515	360	G	A	synonymous
111516	NTHI477_RS00515	402	G	A	synonymous
111531	NTHI477_RS00515	417	T	C	synonymous
111537	NTHI477_RS00515	423	T	C	synonymous
111540	NTHI477_RS00515	426	T	C	synonymous
111546	NTHI477_RS00515	432	A	G	synonymous
111546	NTHI477_RS00515	432	A	T	synonymous
111555	NTHI477_RS00515	441	C	T	synonymous
111564	NTHI477_RS00515	450	G	T	synonymous
111579	NTHI477_RS00515	465	A	T	synonymous
111585	NTHI477_RS00515	471	C	T	synonymous
111597	NTHI477_RS00515	483	A	T	synonymous
111600	NTHI477_RS00515	486	G	T	synonymous
111630	NTHI477_RS00515	516	T	C	synonymous
111657	NTHI477_RS00515	543	C	T	synonymous
111689	NTHI477_RS00515	575	T	C	nonsynonymous
111690	NTHI477_RS00515	576	C	T	synonymous
111715	NTHI477_RS00515	601	A	G	nonsynonymous

111717	NTHI477_RS00515	603	G	A	synonymous
111718	NTHI477_RS00515	604	A	C	nonsynonymous
111719	NTHI477_RS00515	605	A	G	nonsynonymous
111726	NTHI477_RS00515	612	T	C	synonymous
111728	NTHI477_RS00515	614	G	A	nonsynonymous
111729	NTHI477_RS00515	615	C	T	synonymous
111768	NTHI477_RS00515	654	T	A	synonymous
111771	NTHI477_RS00515	657	G	A	synonymous
111771	NTHI477_RS00515	657	G	T	synonymous
111774	NTHI477_RS00515	660	T	C	synonymous
111818	NTHI477_RS00515	704	C	T	nonsynonymous
111819	NTHI477_RS00515	705	G	A	synonymous
111837	NTHI477_RS00515	723	T	C	synonymous
111840	NTHI477_RS00515	726	A	T	synonymous
111846	NTHI477_RS00515	732	G	A	synonymous
111849	NTHI477_RS00515	735	C	T	synonymous
111885	NTHI477_RS00515	771	G	A	synonymous
111945	NTHI477_RS00515	831	A	C	synonymous
111951	NTHI477_RS00515	837	G	A	synonymous
111951	NTHI477_RS00515	837	G	T	synonymous
111957	NTHI477_RS00515	843	T	G	synonymous
111963	NTHI477_RS00515	849	A	G	synonymous
111972	NTHI477_RS00515	858	G	T	synonymous
111977	NTHI477_RS00515	863	G	C	nonsynonymous
111978	NTHI477_RS00515	864	T	C	synonymous
111987	NTHI477_RS00515	873	C	T	synonymous
111999	NTHI477_RS00515	885	G	A	synonymous
112004	NTHI477_RS00515	890	T	C	nonsynonymous
112012	NTHI477_RS00515	898	A	C	nonsynonymous
112029	NTHI477_RS00515	915	C	T	synonymous
112038	NTHI477_RS00515	924	C	T	synonymous
112041	NTHI477_RS00515	927	G	C	synonymous
112044	NTHI477_RS00515	930	C	A	synonymous
112059	NTHI477_RS00515	945	C	T	synonymous
112071	NTHI477_RS00515	957	T	C	synonymous
112074	NTHI477_RS00515	960	A	G	synonymous
112080	NTHI477_RS00515	966	A	G	synonymous
112081	NTHI477_RS00515	967	G	A	nonsynonymous
112083	NTHI477_RS00515	969	G	A	synonymous
112086	NTHI477_RS00515	972	A	G	synonymous
112110	NTHI477_RS00515	996	T	C	synonymous
112132	NTHI477_RS00515	1018	C	A	synonymous
112145	NTHI477_RS00515	1031	C	A	nonsynonymous
112151	NTHI477_RS00515	1037	A	C	nonsynonymous
112171	NTHI477_RS00515	1057	G	T	nonsynonymous

112200	NTHI477_RS00515	1086	T	C	synonymous
112202	NTHI477_RS00515	1088	C	T	nonsynonymous
112212	NTHI477_RS00515	1098	G	A	synonymous
112260	NTHI477_RS00515	1146	T	C	synonymous
112291	NTHI477_RS00515	1177	C	A	synonymous
112293	NTHI477_RS00515	1179	A	G	synonymous
112339	NTHI477_RS00515	1225	G	A	nonsynonymous
112352	NTHI477_RS00515	1238	A	T	nonsynonymous
112368	NTHI477_RS00515	1254	C	T	synonymous
112375	NTHI477_RS00515	1261	T	C	synonymous
112385	NTHI477_RS00515	1271	T	C	nonsynonymous
112386	NTHI477_RS00515	1272	T	G	nonsynonymous
112408	intergenic	intergenic	A	G	intergenic
112420	NTHI477_RS00520	8	C	G	nonsynonymous
112448	NTHI477_RS00520	36	C	T	synonymous
112452	NTHI477_RS00520	40	C	A	nonsynonymous
112478	NTHI477_RS00520	66	T	C	synonymous
112504	NTHI477_RS00520	92	T	C	nonsynonymous
112514	NTHI477_RS00520	102	T	C	synonymous
112571	NTHI477_RS00520	159	C	T	synonymous
112589	NTHI477_RS00520	177	A	G	synonymous
112598	NTHI477_RS00520	186	C	T	synonymous
112603	NTHI477_RS00520	191	G	A	nonsynonymous
112610	NTHI477_RS00520	198	C	T	synonymous
112613	NTHI477_RS00520	201	C	T	synonymous
112631	NTHI477_RS00520	219	A	G	synonymous
112634	NTHI477_RS00520	222	G	T	synonymous
112649	NTHI477_RS00520	237	C	T	synonymous
112673	NTHI477_RS00520	261	G	A	synonymous
112703	NTHI477_RS00520	291	G	A	synonymous
112709	NTHI477_RS00520	297	T	C	synonymous
112712	NTHI477_RS00520	300	G	T	synonymous
112718	NTHI477_RS00520	306	A	G	synonymous
112721	NTHI477_RS00520	309	T	C	synonymous
112727	NTHI477_RS00520	315	C	T	synonymous
112743	NTHI477_RS00520	331	A	C	nonsynonymous
112745	NTHI477_RS00520	333	C	T	synonymous
112748	NTHI477_RS00520	336	C	T	synonymous
112754	NTHI477_RS00520	342	G	A	synonymous
112757	NTHI477_RS00520	345	T	G	synonymous
112766	NTHI477_RS00520	354	G	A	synonymous
112769	NTHI477_RS00520	357	G	A	synonymous
112778	NTHI477_RS00520	366	G	A	synonymous
112832	NTHI477_RS00520	420	C	T	synonymous
112854	NTHI477_RS00520	442	C	T	nonsynonymous

112875	NTHI477_RS00520	463	T	C	synonymous
112877	NTHI477_RS00520	465	G	A	synonymous
112880	NTHI477_RS00520	468	A	G	synonymous
112882	NTHI477_RS00520	470	A	G	nonsynonymous
112889	NTHI477_RS00520	477	T	C	synonymous
112898	NTHI477_RS00520	486	C	T	synonymous
112900	NTHI477_RS00520	488	T	C	nonsynonymous
112901	NTHI477_RS00520	489	C	T	synonymous
112925	NTHI477_RS00520	513	C	T	synonymous
112928	NTHI477_RS00520	516	T	G	synonymous
112931	NTHI477_RS00520	519	C	A	synonymous
112931	NTHI477_RS00520	519	C	G	synonymous
112934	NTHI477_RS00520	522	A	G	synonymous
112937	NTHI477_RS00520	525	T	C	synonymous
112943	NTHI477_RS00520	531	C	T	synonymous
112952	NTHI477_RS00520	540	C	T	synonymous
112958	NTHI477_RS00520	546	C	T	synonymous
112961	NTHI477_RS00520	549	G	A	synonymous
112964	NTHI477_RS00520	552	G	T	synonymous
112976	NTHI477_RS00520	564	G	A	synonymous
112982	NTHI477_RS00520	570	T	C	synonymous
113001	NTHI477_RS00520	589	C	T	nonsynonymous
113008	NTHI477_RS00520	596	C	T	nonsynonymous
113033	NTHI477_RS00520	621	T	C	synonymous
113036	NTHI477_RS00520	624	A	C	synonymous
113037	NTHI477_RS00520	625	A	G	nonsynonymous
113039	NTHI477_RS00520	627	A	C	synonymous
113042	NTHI477_RS00520	630	C	T	synonymous
113048	NTHI477_RS00520	636	C	T	synonymous
113051	NTHI477_RS00520	639	T	G	synonymous
113052	NTHI477_RS00520	640	A	C	nonsynonymous
113055	NTHI477_RS00520	643	A	G	nonsynonymous
113063	NTHI477_RS00520	651	A	G	synonymous
113072	NTHI477_RS00520	660	C	T	synonymous
113075	NTHI477_RS00520	663	A	G	synonymous
113078	NTHI477_RS00520	666	A	G	synonymous
113080	NTHI477_RS00520	668	C	A	nonsynonymous
113082	NTHI477_RS00520	670	A	G	nonsynonymous
113085	NTHI477_RS00520	673	G	T	nonsynonymous
113090	NTHI477_RS00520	678	A	T	synonymous
113093	NTHI477_RS00520	681	T	C	synonymous
113096	NTHI477_RS00520	684	T	G	nonsynonymous
113099	NTHI477_RS00520	687	A	T	synonymous
113105	NTHI477_RS00520	693	G	A	synonymous
113109	NTHI477_RS00520	697	G	A	nonsynonymous

113114	NTHI477_RS00520	702	C	T	synonymous
113126	NTHI477_RS00520	714	G	A	synonymous
113177	NTHI477_RS00520	765	A	G	synonymous
113183	NTHI477_RS00520	771	G	A	synonymous
113198	NTHI477_RS00520	786	C	T	synonymous
113216	NTHI477_RS00520	804	C	T	synonymous
113220	NTHI477_RS00520	808	T	C	synonymous
113222	NTHI477_RS00520	810	G	T	nonsynonymous
113228	NTHI477_RS00520	816	G	C	synonymous
113228	NTHI477_RS00520	816	G	A	synonymous
113240	NTHI477_RS00520	828	T	G	synonymous
113247	NTHI477_RS00520	835	T	C	nonsynonymous
113267	NTHI477_RS00520	855	G	A	synonymous
113270	NTHI477_RS00520	858	G	A	synonymous
113274	NTHI477_RS00520	862	T	C	nonsynonymous
113276	NTHI477_RS00520	864	C	T	synonymous
113290	NTHI477_RS00520	878	G	C	nonsynonymous
113291	NTHI477_RS00520	879	C	G	nonsynonymous
113302	NTHI477_RS00520	890	G	A	nonsynonymous
113332	NTHI477_RS00520	920	A	C	nonsynonymous
113333	NTHI477_RS00520	921	A	G	synonymous
113336	NTHI477_RS00520	924	C	T	synonymous
113337	NTHI477_RS00520	925	A	C	synonymous
113339	NTHI477_RS00520	927	A	G	synonymous
113342	NTHI477_RS00520	930	G	C	synonymous
113354	NTHI477_RS00520	942	A	G	synonymous
113363	NTHI477_RS00520	951	T	C	synonymous
113369	NTHI477_RS00520	957	C	A	synonymous
113375	NTHI477_RS00520	963	C	T	synonymous
113390	NTHI477_RS00520	978	A	G	synonymous
113398	NTHI477_RS00520	986	C	T	nonsynonymous
113399	NTHI477_RS00520	987	A	G	synonymous
113414	NTHI477_RS00520	1002	G	A	synonymous
113418	NTHI477_RS00520	1006	G	A	nonsynonymous
113419	NTHI477_RS00520	1007	A	C	nonsynonymous
113444	NTHI477_RS00520	1032	T	C	synonymous
113450	NTHI477_RS00520	1038	A	G	synonymous
113453	NTHI477_RS00520	1041	C	T	synonymous
113459	NTHI477_RS00520	1047	G	A	synonymous
113462	NTHI477_RS00520	1050	G	A	synonymous
113570	NTHI477_RS00525	9	G	A	synonymous
113593	NTHI477_RS00525	32	A	G	nonsynonymous
113627	NTHI477_RS00525	66	A	G	synonymous
113642	NTHI477_RS00525	81	A	G	synonymous
113644	NTHI477_RS00525	83	T	C	nonsynonymous

113662	NTHI477_RS00525	101	C	T	nonsynonymous
113687	NTHI477_RS00525	126	T	C	synonymous
113702	NTHI477_RS00525	141	A	G	synonymous
113705	NTHI477_RS00525	144	T	G	nonsynonymous
113708	NTHI477_RS00525	147	G	A	synonymous
113723	NTHI477_RS00525	162	C	T	synonymous
113730	NTHI477_RS00525	169	C	G	nonsynonymous
113731	NTHI477_RS00525	170	G	A	nonsynonymous
113735	NTHI477_RS00525	174	C	A	nonsynonymous
113738	NTHI477_RS00525	177	C	T	synonymous
113742	NTHI477_RS00525	181	T	G	nonsynonymous
113744	NTHI477_RS00525	183	G	A	synonymous
113750	NTHI477_RS00525	189	G	A	synonymous
113756	NTHI477_RS00525	195	T	A	synonymous
113762	NTHI477_RS00525	201	C	T	synonymous
113770	NTHI477_RS00525	209	C	T	nonsynonymous
113778	NTHI477_RS00525	217	C	A	synonymous
113780	NTHI477_RS00525	219	A	G	synonymous
113782	NTHI477_RS00525	221	T	C	nonsynonymous
113786	NTHI477_RS00525	225	A	G	synonymous
113795	NTHI477_RS00525	234	C	A	synonymous
113797	NTHI477_RS00525	236	C	G	nonsynonymous
113798	NTHI477_RS00525	237	C	A	synonymous
113802	NTHI477_RS00525	241	C	A	nonsynonymous
113807	NTHI477_RS00525	246	A	C	synonymous
113813	NTHI477_RS00525	252	A	G	synonymous
113825	NTHI477_RS00525	264	A	C	synonymous
113825	NTHI477_RS00525	264	A	T	synonymous
113834	NTHI477_RS00525	273	A	G	synonymous
113837	NTHI477_RS00525	276	T	C	synonymous
113846	NTHI477_RS00525	285	T	C	synonymous
113848	NTHI477_RS00525	287	G	A	nonsynonymous
113849	NTHI477_RS00525	288	C	T	synonymous
113852	NTHI477_RS00525	291	C	T	synonymous
113855	NTHI477_RS00525	294	T	C	synonymous
113861	NTHI477_RS00525	300	C	T	synonymous
113862	NTHI477_RS00525	301	T	G	nonsynonymous
113864	NTHI477_RS00525	303	T	C	synonymous
113865	NTHI477_RS00525	304	G	A	nonsynonymous
113878	NTHI477_RS00525	317	G	C	nonsynonymous
113879	NTHI477_RS00525	318	T	G	synonymous
113882	NTHI477_RS00525	321	G	A	synonymous
113883	NTHI477_RS00525	322	T	C	synonymous
113885	NTHI477_RS00525	324	A	G	synonymous
113888	NTHI477_RS00525	327	T	G	nonsynonymous

113888	NTHI477_RS00525	327	T	C	synonymous
113906	NTHI477_RS00525	345	T	C	synonymous
113911	NTHI477_RS00525	350	C	T	nonsynonymous
113915	NTHI477_RS00525	354	G	A	synonymous
113924	NTHI477_RS00525	363	T	C	synonymous
113930	NTHI477_RS00525	369	T	C	synonymous
113935	NTHI477_RS00525	374	G	T	nonsynonymous
113936	NTHI477_RS00525	375	T	C	synonymous
113939	NTHI477_RS00525	378	C	T	synonymous
113944	NTHI477_RS00525	383	T	C	nonsynonymous
113960	NTHI477_RS00525	399	T	C	synonymous
113965	NTHI477_RS00525	404	A	T	nonsynonymous
113970	NTHI477_RS00525	409	T	C	nonsynonymous
113972	NTHI477_RS00525	411	T	A	synonymous
113975	NTHI477_RS00525	414	A	C	synonymous
113978	NTHI477_RS00525	417	A	T	synonymous
113981	NTHI477_RS00525	420	A	G	synonymous
113985	NTHI477_RS00525	424	A	G	nonsynonymous
113988	NTHI477_RS00525	427	A	G	nonsynonymous
114005	NTHI477_RS00525	444	C	T	synonymous
114012	NTHI477_RS00525	451	C	A	nonsynonymous
114043	NTHI477_RS00525	482	C	T	nonsynonymous
114044	NTHI477_RS00525	483	A	C	synonymous
114045	NTHI477_RS00525	484	A	G	nonsynonymous
114060	NTHI477_RS00525	499	A	G	nonsynonymous
114104	NTHI477_RS00525	543	C	T	synonymous
114111	NTHI477_RS00525	550	A	C	nonsynonymous
114127	NTHI477_RS00525	566	C	T	nonsynonymous
114128	NTHI477_RS00525	567	C	G	synonymous
114137	NTHI477_RS00525	576	C	A	synonymous
114137	NTHI477_RS00525	576	C	G	synonymous
114138	NTHI477_RS00525	577	G	A	nonsynonymous
114142	NTHI477_RS00525	581	A	G	nonsynonymous
114144	NTHI477_RS00525	583	A	G	nonsynonymous
114181	NTHI477_RS00525	620	C	G	nonsynonymous
114201	NTHI477_RS00525	640	A	G	nonsynonymous
114201	NTHI477_RS00530	5	A	G	nonsynonymous
114218	NTHI477_RS00530	22	A	G	nonsynonymous
114228	NTHI477_RS00530	32	G	A	nonsynonymous
114229	NTHI477_RS00530	33	T	C	synonymous
114234	NTHI477_RS00530	38	A	G	nonsynonymous
114235	NTHI477_RS00530	39	A	G	synonymous
114238	NTHI477_RS00530	42	A	G	synonymous
114238	NTHI477_RS00530	42	A	C	nonsynonymous
114241	NTHI477_RS00530	45	C	T	synonymous

114253	NTHI477_RS00530	57	C	A	synonymous
114259	NTHI477_RS00530	63	T	C	synonymous
114260	NTHI477_RS00530	64	A	G	nonsynonymous
114261	NTHI477_RS00530	65	G	A	nonsynonymous
114262	NTHI477_RS00530	66	C	T	synonymous
114268	NTHI477_RS00530	72	T	C	synonymous
114269	NTHI477_RS00530	73	G	A	nonsynonymous
114270	NTHI477_RS00530	74	A	G	nonsynonymous
114271	NTHI477_RS00530	75	A	G	synonymous
114274	NTHI477_RS00530	78	T	C	synonymous
114277	NTHI477_RS00530	81	G	C	synonymous
114283	NTHI477_RS00530	87	T	C	synonymous
114289	NTHI477_RS00530	93	A	G	synonymous
114296	NTHI477_RS00530	100	A	G	nonsynonymous
114297	NTHI477_RS00530	101	A	C	nonsynonymous
114298	NTHI477_RS00530	102	T	G	nonsynonymous
114304	NTHI477_RS00530	108	G	T	nonsynonymous
114308	NTHI477_RS00530	112	A	G	nonsynonymous
114311	NTHI477_RS00530	115	A	G	nonsynonymous
114312	NTHI477_RS00530	116	T	C	nonsynonymous
114313	NTHI477_RS00530	117	G	A	nonsynonymous
114325	NTHI477_RS00530	129	C	T	synonymous
114344	NTHI477_RS00530	148	T	C	synonymous
114352	NTHI477_RS00530	156	C	T	synonymous
114361	NTHI477_RS00530	165	A	G	synonymous
114364	NTHI477_RS00530	168	A	T	synonymous
114388	NTHI477_RS00530	192	T	C	synonymous
114389	NTHI477_RS00530	193	A	T	nonsynonymous
114391	NTHI477_RS00530	195	G	A	synonymous
114400	NTHI477_RS00530	204	G	A	synonymous
114415	NTHI477_RS00530	219	C	G	synonymous
114416	NTHI477_RS00530	220	A	G	nonsynonymous
114418	NTHI477_RS00530	222	C	T	synonymous
114450	NTHI477_RS00530	254	G	A	nonsynonymous
114451	NTHI477_RS00530	255	C	G	synonymous
114452	NTHI477_RS00530	256	A	G	nonsynonymous
114454	NTHI477_RS00530	258	T	C	synonymous
114456	NTHI477_RS00530	260	T	A	nonsynonymous
114460	NTHI477_RS00530	264	C	G	synonymous
114461	NTHI477_RS00530	265	A	G	nonsynonymous
114469	NTHI477_RS00530	273	G	A	synonymous
114470	NTHI477_RS00530	274	C	T	synonymous
114473	NTHI477_RS00530	277	A	C	nonsynonymous
114474	NTHI477_RS00530	278	T	C	nonsynonymous
114475	NTHI477_RS00530	279	T	G	nonsynonymous

114479	NTHI477_RS00530	283	C	T	nonsynonymous
114491	NTHI477_RS00530	295	T	C	nonsynonymous
114496	NTHI477_RS00530	300	T	C	synonymous
114499	NTHI477_RS00530	303	T	C	synonymous
114505	NTHI477_RS00530	309	A	C	synonymous
114510	NTHI477_RS00530	314	A	C	nonsynonymous
114511	NTHI477_RS00530	315	C	A	nonsynonymous
114521	NTHI477_RS00530	325	C	T	nonsynonymous
114522	NTHI477_RS00530	326	A	T	nonsynonymous
114523	NTHI477_RS00530	327	A	G	synonymous
114525	NTHI477_RS00530	329	G	A	nonsynonymous
114528	NTHI477_RS00530	332	G	A	nonsynonymous
114538	NTHI477_RS00530	342	G	A	synonymous
114541	NTHI477_RS00530	345	T	C	synonymous
114544	NTHI477_RS00530	348	A	G	synonymous
114547	NTHI477_RS00530	351	T	C	synonymous
114577	NTHI477_RS00530	381	C	T	synonymous
114591	NTHI477_RS00530	395	C	A	nonsynonymous
114652	NTHI477_RS00530	456	C	T	synonymous
114658	NTHI477_RS00530	462	G	A	synonymous
114688	NTHI477_RS00530	492	A	C	synonymous
114709	NTHI477_RS00530	513	C	T	synonymous
114718	NTHI477_RS00530	522	A	G	synonymous
114720	NTHI477_RS00530	524	A	C	nonsynonymous
114727	NTHI477_RS00530	531	A	G	synonymous
114733	NTHI477_RS00530	537	T	A	nonsynonymous
114757	NTHI477_RS00530	561	G	A	synonymous
114766	NTHI477_RS00530	570	T	G	nonsynonymous
114787	NTHI477_RS00530	591	A	G	synonymous
114790	NTHI477_RS00530	594	A	G	synonymous
114793	NTHI477_RS00530	597	G	A	synonymous
114808	NTHI477_RS00530	612	T	C	synonymous
114811	NTHI477_RS00530	615	G	T	synonymous
114820	NTHI477_RS00530	624	C	A	synonymous
114823	NTHI477_RS00530	627	T	C	synonymous
114832	NTHI477_RS00530	636	G	C	synonymous
114835	NTHI477_RS00530	639	T	G	synonymous
114841	NTHI477_RS00530	645	A	G	synonymous
114868	NTHI477_RS00530	672	T	C	synonymous
114869	NTHI477_RS00530	673	G	A	nonsynonymous
114870	NTHI477_RS00530	674	G	A	nonsynonymous
114879	NTHI477_RS00530	683	G	A	nonsynonymous
114880	NTHI477_RS00530	684	C	T	synonymous
114881	NTHI477_RS00530	685	G	A	nonsynonymous
114890	NTHI477_RS00530	694	T	A	nonsynonymous

114891	NTHI477_RS00530	695	C	A	nonsynonymous
114895	NTHI477_RS00530	699	G	T	synonymous
114895	NTHI477_RS00530	699	G	A	synonymous
114898	NTHI477_RS00530	702	C	T	synonymous
114902	NTHI477_RS00530	706	A	T	nonsynonymous
114907	NTHI477_RS00530	711	G	A	synonymous
114912	NTHI477_RS00530	716	A	G	nonsynonymous
114913	NTHI477_RS00530	717	C	T	synonymous
114922	NTHI477_RS00530	726	C	G	synonymous
114922	NTHI477_RS00530	726	C	T	synonymous
114923	NTHI477_RS00530	727	C	T	synonymous
114925	NTHI477_RS00530	729	G	A	synonymous
114937	NTHI477_RS00530	741	G	A	synonymous
114940	NTHI477_RS00530	744	A	G	synonymous
114952	NTHI477_RS00530	756	A	G	synonymous
114955	NTHI477_RS00530	759	C	T	synonymous
114964	NTHI477_RS00530	768	C	G	nonsynonymous
114965	NTHI477_RS00530	769	A	C	nonsynonymous
114982	intergenic	intergenic	A	C	intergenic
114983	intergenic	intergenic	A	G	intergenic
114994	NTHI477_RS00535	6	C	A	synonymous
115007	NTHI477_RS00535	19	A	G	nonsynonymous
115012	NTHI477_RS00535	24	T	G	synonymous
115015	NTHI477_RS00535	27	C	T	synonymous
115029	NTHI477_RS00535	41	T	C	nonsynonymous
115033	NTHI477_RS00535	45	A	G	synonymous
115042	NTHI477_RS00535	54	T	C	synonymous
115045	NTHI477_RS00535	57	C	T	synonymous
115066	NTHI477_RS00535	78	A	G	synonymous
115081	NTHI477_RS00535	93	C	T	synonymous
115083	NTHI477_RS00535	95	G	T	nonsynonymous
115090	NTHI477_RS00535	102	G	T	synonymous
115117	NTHI477_RS00535	129	C	T	synonymous
115129	NTHI477_RS00535	141	C	T	synonymous
115141	NTHI477_RS00535	153	A	G	synonymous
115147	NTHI477_RS00535	159	G	A	synonymous
115164	NTHI477_RS00535	176	A	G	nonsynonymous
115165	NTHI477_RS00535	177	C	G	nonsynonymous
115169	NTHI477_RS00535	181	G	A	nonsynonymous
115174	NTHI477_RS00535	186	A	G	synonymous
115177	NTHI477_RS00535	189	G	A	synonymous
115179	NTHI477_RS00535	191	A	C	nonsynonymous
115196	NTHI477_RS00535	208	A	G	nonsynonymous
115198	NTHI477_RS00535	210	A	C	nonsynonymous
115211	NTHI477_RS00535	223	G	C	nonsynonymous

115231	NTHI477_RS00535	243	G	T	synonymous
115234	NTHI477_RS00535	246	A	T	synonymous
115243	NTHI477_RS00535	255	T	G	synonymous
115251	NTHI477_RS00535	263	G	T	nonsynonymous
115252	NTHI477_RS00535	264	A	C	synonymous
115256	NTHI477_RS00535	268	A	G	nonsynonymous
115258	NTHI477_RS00535	270	T	G	nonsynonymous
115259	NTHI477_RS00535	271	A	C	synonymous
115262	NTHI477_RS00535	274	A	G	nonsynonymous
115268	NTHI477_RS00535	280	G	A	nonsynonymous
115270	NTHI477_RS00535	282	A	T	nonsynonymous
115272	NTHI477_RS00535	284	C	A	nonsynonymous
115279	NTHI477_RS00535	291	C	T	synonymous
115291	NTHI477_RS00535	303	T	C	synonymous
115294	NTHI477_RS00535	306	C	T	synonymous
115324	NTHI477_RS00535	336	A	G	synonymous
115325	NTHI477_RS00535	337	C	T	synonymous
115327	NTHI477_RS00535	339	A	G	synonymous
115328	NTHI477_RS00535	340	T	C	synonymous
115336	NTHI477_RS00535	348	T	G	synonymous
115339	NTHI477_RS00535	351	T	C	synonymous
115345	NTHI477_RS00535	357	C	G	synonymous
115345	NTHI477_RS00535	357	C	A	synonymous
115354	NTHI477_RS00535	366	A	G	synonymous
115363	NTHI477_RS00535	375	T	C	synonymous
115364	NTHI477_RS00535	376	G	A	nonsynonymous
115366	NTHI477_RS00535	378	A	C	nonsynonymous
115369	NTHI477_RS00535	381	A	G	synonymous
115369	NTHI477_RS00535	381	A	T	nonsynonymous
115371	NTHI477_RS00535	383	A	C	nonsynonymous
115381	NTHI477_RS00535	393	G	A	synonymous
115395	NTHI477_RS00535	407	T	C	nonsynonymous
115399	NTHI477_RS00535	411	T	C	synonymous
115411	NTHI477_RS00535	423	A	G	synonymous
115421	NTHI477_RS00535	433	A	G	nonsynonymous
115423	NTHI477_RS00535	435	A	G	synonymous
115433	NTHI477_RS00535	445	C	T	synonymous
115438	NTHI477_RS00535	450	C	T	synonymous
115444	NTHI477_RS00535	456	A	T	synonymous
115450	NTHI477_RS00535	462	T	C	synonymous
115471	NTHI477_RS00535	483	T	C	synonymous
115474	NTHI477_RS00535	486	T	C	synonymous
115483	NTHI477_RS00535	495	A	C	synonymous
115495	NTHI477_RS00535	507	A	G	synonymous
115562	NTHI477_RS00535	574	T	G	nonsynonymous

115577	NTHI477_RS00535	589	G	A	nonsynonymous
115583	NTHI477_RS00535	595	C	T	nonsynonymous
115619	NTHI477_RS00535	631	A	C	nonsynonymous
115626	NTHI477_RS00535	638	T	C	nonsynonymous
115658	intergenic	intergenic	G	A	intergenic
115717	NTHI477_RS00540	6	C	T	synonymous
115744	NTHI477_RS00540	33	C	T	synonymous
115762	NTHI477_RS00540	51	G	A	synonymous
115783	NTHI477_RS00540	72	G	A	synonymous
115808	NTHI477_RS00540	97	G	A	nonsynonymous
115819	NTHI477_RS00540	108	G	A	synonymous
115837	NTHI477_RS00540	126	G	A	synonymous
115840	NTHI477_RS00540	129	T	C	synonymous
115843	NTHI477_RS00540	132	A	T	synonymous
115861	NTHI477_RS00540	150	T	G	synonymous
115867	NTHI477_RS00540	156	A	G	synonymous
115880	NTHI477_RS00540	169	G	T	nonsynonymous
115882	NTHI477_RS00540	171	A	G	synonymous
115934	NTHI477_RS00540	223	A	C	nonsynonymous
115963	NTHI477_RS00540	252	G	A	synonymous
115987	NTHI477_RS00540	276	G	A	synonymous
115990	NTHI477_RS00540	279	C	T	synonymous
115996	NTHI477_RS00540	285	C	T	synonymous
116005	NTHI477_RS00540	294	A	G	synonymous
116038	NTHI477_RS00540	327	G	A	nonsynonymous
116069	NTHI477_RS00540	358	A	G	nonsynonymous
116071	NTHI477_RS00540	360	T	A	synonymous
116084	NTHI477_RS00540	373	C	T	nonsynonymous
116086	NTHI477_RS00540	375	C	T	synonymous
116089	NTHI477_RS00540	378	T	C	synonymous
116119	NTHI477_RS00540	408	C	T	synonymous
116130	NTHI477_RS00540	419	C	G	nonsynonymous
116146	NTHI477_RS00540	435	G	A	synonymous
116182	NTHI477_RS00540	471	G	A	synonymous
116194	NTHI477_RS00540	483	G	A	synonymous
116206	NTHI477_RS00540	495	G	A	synonymous
116215	NTHI477_RS00540	504	A	G	synonymous
116224	NTHI477_RS00540	513	A	G	synonymous
116227	NTHI477_RS00540	516	C	A	synonymous
116233	NTHI477_RS00540	522	A	G	synonymous
116239	NTHI477_RS00540	528	C	T	synonymous
116254	NTHI477_RS00540	543	T	A	synonymous
116260	NTHI477_RS00540	549	T	C	synonymous
116269	NTHI477_RS00540	558	G	A	synonymous
116272	NTHI477_RS00540	561	G	C	synonymous

116305	NTHI477_RS00540	594	C	T	synonymous
116359	NTHI477_RS00540	648	A	G	synonymous
116362	NTHI477_RS00540	651	T	G	synonymous
116371	NTHI477_RS00540	660	T	C	synonymous
116374	NTHI477_RS00540	663	T	C	synonymous
116375	NTHI477_RS00540	664	C	A	nonsynonymous
116386	NTHI477_RS00540	675	G	A	synonymous
116389	NTHI477_RS00540	678	G	T	synonymous
116392	NTHI477_RS00540	681	G	A	synonymous
116394	NTHI477_RS00540	683	G	A	synonymous
116403	NTHI477_RS00545	9	G	T	synonymous
116406	NTHI477_RS00545	12	G	T	synonymous
116412	NTHI477_RS00545	18	T	C	synonymous
116439	NTHI477_RS00545	45	A	G	synonymous
116454	NTHI477_RS00545	60	A	G	synonymous
116457	NTHI477_RS00545	63	C	T	synonymous
116475	NTHI477_RS00545	81	C	T	synonymous
116493	NTHI477_RS00545	99	A	G	synonymous
116494	NTHI477_RS00545	100	T	C	synonymous
116499	NTHI477_RS00545	105	T	C	synonymous
116520	NTHI477_RS00545	126	C	T	synonymous
116538	NTHI477_RS00545	144	T	G	synonymous
116556	NTHI477_RS00545	162	T	C	synonymous
116562	NTHI477_RS00545	168	G	A	synonymous
116566	NTHI477_RS00545	172	T	C	synonymous
116568	NTHI477_RS00545	174	G	T	nonsynonymous
116574	NTHI477_RS00545	180	C	G	synonymous
116577	NTHI477_RS00545	183	G	T	synonymous
116580	NTHI477_RS00545	186	G	C	synonymous
116584	NTHI477_RS00545	190	A	G	nonsynonymous
116585	NTHI477_RS00545	191	T	C	nonsynonymous
116586	NTHI477_RS00545	192	T	A	synonymous
116595	NTHI477_RS00545	201	T	A	nonsynonymous
116601	NTHI477_RS00545	207	G	A	synonymous
116603	NTHI477_RS00545	209	A	C	nonsynonymous
116604	NTHI477_RS00545	210	T	C	synonymous
116612	NTHI477_RS00545	218	A	C	nonsynonymous
116613	NTHI477_RS00545	219	A	G	synonymous
116619	NTHI477_RS00545	225	C	A	synonymous
116622	NTHI477_RS00545	228	A	C	synonymous
116653	NTHI477_RS00545	259	C	T	synonymous
116665	NTHI477_RS00545	271	C	G	nonsynonymous
116667	NTHI477_RS00545	273	A	C	synonymous
116679	NTHI477_RS00545	285	A	T	synonymous
116679	NTHI477_RS00545	285	A	C	synonymous

116682	NTHI477_RS00545	288	C	T	synonymous
116685	NTHI477_RS00545	291	T	C	synonymous
116694	NTHI477_RS00545	300	T	G	synonymous
116703	NTHI477_RS00545	309	G	T	synonymous
116709	NTHI477_RS00545	315	G	A	synonymous
116724	NTHI477_RS00545	330	A	G	synonymous
116745	NTHI477_RS00545	351	C	T	synonymous
116790	NTHI477_RS00545	396	C	T	synonymous
116796	NTHI477_RS00545	402	T	C	synonymous
116805	NTHI477_RS00545	411	G	A	synonymous
116811	NTHI477_RS00545	417	T	G	synonymous
116815	NTHI477_RS00545	421	A	G	nonsynonymous
116817	NTHI477_RS00545	423	C	T	synonymous
116825	NTHI477_RS00545	431	A	G	nonsynonymous
116841	NTHI477_RS00545	447	G	A	synonymous
116851	NTHI477_RS00545	457	G	T	nonsynonymous
116869	NTHI477_RS00545	475	C	T	nonsynonymous
116901	NTHI477_RS00545	507	G	A	synonymous
116919	NTHI477_RS00545	525	C	T	synonymous
116935	NTHI477_RS00545	541	G	A	nonsynonymous
117000	NTHI477_RS00545	606	T	C	synonymous
117018	NTHI477_RS00545	624	G	A	synonymous
117024	NTHI477_RS00545	630	T	C	synonymous
117036	NTHI477_RS00545	642	C	G	synonymous
117042	NTHI477_RS00545	648	G	A	synonymous
117054	NTHI477_RS00545	660	T	A	synonymous
117101	NTHI477_RS00545	707	C	T	nonsynonymous
117102	NTHI477_RS00545	708	G	A	synonymous
117138	NTHI477_RS00545	744	G	T	synonymous
117189	NTHI477_RS00545	795	C	A	synonymous
117205	NTHI477_RS00545	811	A	C	synonymous
117210	NTHI477_RS00545	816	C	T	synonymous
117213	NTHI477_RS00545	819	A	G	synonymous
117219	NTHI477_RS00545	825	T	C	synonymous
117225	NTHI477_RS00545	831	A	T	synonymous
117234	NTHI477_RS00545	840	T	A	synonymous
117240	NTHI477_RS00545	846	C	T	synonymous
117246	NTHI477_RS00545	852	T	G	synonymous
117282	NTHI477_RS00545	888	G	A	synonymous
117297	NTHI477_RS00545	903	G	A	synonymous
117306	NTHI477_RS00545	912	T	A	synonymous
117318	NTHI477_RS00545	924	C	T	synonymous
117321	NTHI477_RS00545	927	G	A	synonymous
117327	NTHI477_RS00545	933	G	A	synonymous
117330	NTHI477_RS00545	936	G	A	synonymous

117336	NTHI477_RS00545	942	T	C	synonymous
117339	NTHI477_RS00545	945	G	T	synonymous
117342	NTHI477_RS00545	948	T	C	synonymous
117375	NTHI477_RS00545	981	G	A	synonymous
117393	NTHI477_RS00545	999	T	C	synonymous
117402	NTHI477_RS00545	1008	A	T	synonymous
117435	NTHI477_RS00545	1041	T	G	synonymous
117435	NTHI477_RS00545	1041	T	C	synonymous
117441	NTHI477_RS00545	1047	C	T	synonymous
117453	NTHI477_RS00545	1059	T	C	synonymous
117463	NTHI477_RS00545	1069	G	A	nonsynonymous
117489	NTHI477_RS00545	1095	C	A	synonymous
117504	NTHI477_RS00545	1110	G	C	synonymous
117510	NTHI477_RS00545	1116	C	T	synonymous
117528	NTHI477_RS00545	1134	T	C	synonymous
117533	NTHI477_RS00545	1139	T	C	nonsynonymous
117553	NTHI477_RS00545	1159	G	A	nonsynonymous
117555	NTHI477_RS00545	1161	T	C	synonymous
117558	NTHI477_RS00545	1164	A	T	synonymous
117567	NTHI477_RS00545	1173	A	C	synonymous
117579	NTHI477_RS00545	1185	G	A	nonsynonymous
117579	NTHI477_RS00545	1185	G	T	nonsynonymous
117582	NTHI477_RS00545	1188	A	G	synonymous
117606	NTHI477_RS00545	1212	T	C	synonymous
117624	NTHI477_RS00545	1230	A	G	synonymous
117639	NTHI477_RS00545	1245	C	T	synonymous
117648	intergenic	intergenic	T	C	intergenic
117658	intergenic	intergenic	A	C	intergenic
117662	intergenic	intergenic	C	T	intergenic
117710	intergenic	intergenic	T	C	intergenic
117725	intergenic	intergenic	A	G	intergenic
117749	intergenic	intergenic	G	A	intergenic
117750	intergenic	intergenic	G	A	intergenic
117752	intergenic	intergenic	A	C	intergenic
117768	intergenic	intergenic	C	T	intergenic
117781	intergenic	intergenic	T	A	intergenic
117782	intergenic	intergenic	C	A	intergenic
117800	intergenic	intergenic	C	A	intergenic
117803	intergenic	intergenic	A	G	intergenic
117829	intergenic	intergenic	G	T	intergenic
117830	intergenic	intergenic	A	T	intergenic
117849	intergenic	intergenic	T	C	intergenic
117850	intergenic	intergenic	T	A	intergenic
117851	intergenic	intergenic	T	A	intergenic
117858	intergenic	intergenic	G	A	intergenic

117861	intergenic	intergenic	A	G	intergenic
117895	NTHI477_RS00550	12	A	G	synonymous
117895	NTHI477_RS00550	12	A	T	synonymous
117901	NTHI477_RS00550	18	C	T	synonymous
117908	NTHI477_RS00550	25	C	A	synonymous
117922	NTHI477_RS00550	39	C	T	synonymous
117931	NTHI477_RS00550	48	G	A	synonymous
117940	NTHI477_RS00550	57	C	G	synonymous
117946	NTHI477_RS00550	63	C	A	synonymous
117961	NTHI477_RS00550	78	C	T	synonymous
117967	NTHI477_RS00550	84	A	T	synonymous
117967	NTHI477_RS00550	84	A	G	synonymous
117974	NTHI477_RS00550	91	C	T	nonsynonymous
117976	NTHI477_RS00550	93	G	T	nonsynonymous
117985	NTHI477_RS00550	102	G	A	synonymous
118027	NTHI477_RS00550	144	T	G	nonsynonymous
118029	NTHI477_RS00550	146	G	C	nonsynonymous
118033	NTHI477_RS00550	150	A	G	synonymous
118086	NTHI477_RS00550	203	T	C	nonsynonymous
118104	NTHI477_RS00550	221	C	T	nonsynonymous
118126	NTHI477_RS00550	243	A	T	nonsynonymous
118132	NTHI477_RS00550	249	C	T	synonymous
118144	NTHI477_RS00550	261	T	C	synonymous
118165	NTHI477_RS00550	282	T	C	synonymous
118186	NTHI477_RS00550	303	G	T	synonymous
118192	NTHI477_RS00550	309	T	C	synonymous
118216	NTHI477_RS00550	333	A	G	synonymous
118222	NTHI477_RS00550	339	G	A	synonymous
118249	NTHI477_RS00550	366	G	T	synonymous
118252	NTHI477_RS00550	369	A	G	synonymous
118255	NTHI477_RS00550	372	C	T	synonymous
118303	NTHI477_RS00550	420	A	T	synonymous
118345	NTHI477_RS00550	462	A	T	synonymous
118447	NTHI477_RS00550	564	C	T	synonymous
118462	NTHI477_RS00550	579	G	T	synonymous
118492	NTHI477_RS00550	609	G	A	synonymous
118492	NTHI477_RS00550	609	G	T	synonymous
118505	NTHI477_RS00550	622	T	A	nonsynonymous
118600	NTHI477_RS00550	717	T	C	synonymous
118756	NTHI477_RS00550	873	A	C	synonymous
118758	NTHI477_RS00550	875	T	C	nonsynonymous
118837	NTHI477_RS00550	954	G	A	synonymous
118849	NTHI477_RS00550	966	A	G	synonymous
118915	NTHI477_RS00550	1032	T	G	nonsynonymous
118960	intergenic	intergenic	T	C	intergenic

118963	intergenic	intergenic	T	C	intergenic
119015	intergenic	intergenic	T	C	intergenic
119017	intergenic	intergenic	T	C	intergenic
119026	NTHI477_RS00555	436	C	T	nonsynonymous
119044	NTHI477_RS00555	418	T	C	nonsynonymous
119078	NTHI477_RS00555	384	G	T	synonymous
119086	NTHI477_RS00555	376	C	T	nonsynonymous
119099	NTHI477_RS00555	363	T	C	synonymous
119114	NTHI477_RS00555	348	A	G	synonymous
119124	NTHI477_RS00555	338	T	G	nonsynonymous
119129	NTHI477_RS00555	333	T	G	synonymous
119147	NTHI477_RS00555	315	A	G	synonymous
119153	NTHI477_RS00555	309	G	A	synonymous
119162	NTHI477_RS00555	300	G	A	synonymous
119163	NTHI477_RS00555	299	C	T	nonsynonymous
119168	NTHI477_RS00555	294	C	T	synonymous
119169	NTHI477_RS00555	293	G	A	nonsynonymous
119170	NTHI477_RS00555	292	T	C	nonsynonymous
119207	NTHI477_RS00555	255	T	A	synonymous
119210	NTHI477_RS00555	252	C	T	synonymous
119215	NTHI477_RS00555	247	C	T	nonsynonymous
119225	NTHI477_RS00555	237	A	T	synonymous
119235	NTHI477_RS00555	227	T	G	nonsynonymous
119407	NTHI477_RS00555	55	G	A	synonymous
119468	intergenic	intergenic	A	G	intergenic
119474	intergenic	intergenic	T	C	intergenic
119502	intergenic	intergenic	T	C	intergenic
119503	intergenic	intergenic	A	G	intergenic
119505	intergenic	intergenic	C	T	intergenic
119537	intergenic	intergenic	A	G	intergenic
119544	intergenic	intergenic	A	G	intergenic
119557	intergenic	intergenic	A	G	intergenic
119557	intergenic	intergenic	A	C	intergenic
119567	intergenic	intergenic	A	C	intergenic
119573	intergenic	intergenic	T	A	intergenic
119577	intergenic	intergenic	T	G	intergenic
119583	intergenic	intergenic	G	T	intergenic
119590	intergenic	intergenic	T	G	intergenic
119590	intergenic	intergenic	T	A	intergenic
119592	intergenic	intergenic	T	G	intergenic
119593	intergenic	intergenic	A	T	intergenic
119595	intergenic	intergenic	A	T	intergenic
119624	intergenic	intergenic	C	A	intergenic
119626	intergenic	intergenic	C	G	intergenic
119630	intergenic	intergenic	T	G	intergenic

119633	intergenic	intergenic	A	C	intergenic
119664	intergenic	intergenic	T	C	intergenic
119687	intergenic	intergenic	C	T	intergenic
120012	NTHI477_RS00560	267	A	C	synonymous
120074	NTHI477_RS00560	329	T	C	nonsynonymous
120083	NTHI477_RS00560	338	C	T	nonsynonymous
120090	NTHI477_RS00560	345	T	G	synonymous
120094	NTHI477_RS00560	349	C	T	nonsynonymous
120096	NTHI477_RS00560	351	T	G	synonymous
120099	NTHI477_RS00560	354	A	T	synonymous
120100	NTHI477_RS00560	355	A	G	nonsynonymous
120102	NTHI477_RS00560	357	C	G	synonymous
120112	NTHI477_RS00560	367	G	T	nonsynonymous
120117	NTHI477_RS00560	372	C	T	synonymous
120120	NTHI477_RS00560	375	A	T	synonymous
120126	NTHI477_RS00560	381	G	A	synonymous
120132	NTHI477_RS00560	387	T	C	synonymous
120138	NTHI477_RS00560	393	C	T	synonymous
120144	NTHI477_RS00560	399	T	G	synonymous
120147	NTHI477_RS00560	402	A	G	synonymous
120148	NTHI477_RS00560	403	T	C	nonsynonymous
120150	NTHI477_RS00560	405	T	A	nonsynonymous
120153	NTHI477_RS00560	408	C	A	synonymous
120154	NTHI477_RS00560	409	C	T	nonsynonymous
120156	NTHI477_RS00560	411	T	A	synonymous
120159	NTHI477_RS00560	414	C	T	synonymous
120162	NTHI477_RS00560	417	A	G	synonymous
120165	NTHI477_RS00560	420	C	T	synonymous
120189	NTHI477_RS00560	444	A	G	synonymous
120201	NTHI477_RS00560	456	A	G	synonymous
120222	NTHI477_RS00560	477	A	T	synonymous
120231	NTHI477_RS00560	486	C	T	synonymous
120273	NTHI477_RS00560	528	G	A	synonymous
120276	NTHI477_RS00560	531	C	T	synonymous
120279	NTHI477_RS00560	534	A	G	synonymous
120285	NTHI477_RS00560	540	T	A	synonymous
120300	NTHI477_RS00560	555	C	T	synonymous
120336	NTHI477_RS00560	591	A	G	synonymous
120372	NTHI477_RS00560	627	A	G	synonymous
120375	NTHI477_RS00560	630	T	A	synonymous
120390	NTHI477_RS00560	645	C	T	synonymous
120420	NTHI477_RS00560	675	T	A	synonymous
120471	NTHI477_RS00560	726	A	G	synonymous
120477	NTHI477_RS00560	732	T	C	synonymous
120552	NTHI477_RS00560	807	T	C	synonymous

120552	NTHI477_RS00560	807	T	A	synonymous
120642	NTHI477_RS00560	897	G	A	synonymous
120651	NTHI477_RS00560	906	C	T	synonymous
120699	NTHI477_RS00560	954	C	T	synonymous
120714	NTHI477_RS00560	969	A	T	synonymous
120714	NTHI477_RS00560	969	A	G	synonymous
120729	NTHI477_RS00560	984	C	T	synonymous
120741	NTHI477_RS00560	996	G	A	synonymous
120811	NTHI477_RS00560	1066	T	C	synonymous
120816	NTHI477_RS00560	1071	C	T	synonymous
120825	NTHI477_RS00560	1080	A	C	synonymous
120834	NTHI477_RS00560	1089	A	G	synonymous
120858	NTHI477_RS00560	1113	A	T	synonymous
120882	NTHI477_RS00560	1137	T	C	synonymous
120888	NTHI477_RS00560	1143	C	T	synonymous
120894	NTHI477_RS00560	1149	T	C	synonymous
120903	NTHI477_RS00560	1158	C	T	synonymous
120909	NTHI477_RS00560	1164	C	T	synonymous
120918	NTHI477_RS00560	1173	A	G	synonymous
120924	NTHI477_RS00560	1179	C	T	synonymous
120963	NTHI477_RS00560	1218	G	A	nonsynonymous
120981	NTHI477_RS00560	1236	C	T	synonymous
120986	NTHI477_RS00560	1241	C	T	nonsynonymous
120992	intergenic	intergenic	G	T	intergenic
120999	intergenic	intergenic	C	A	intergenic
121011	intergenic	intergenic	C	T	intergenic
121015	intergenic	intergenic	C	A	intergenic
121016	intergenic	intergenic	T	A	intergenic
121017	intergenic	intergenic	A	T	intergenic
121035	intergenic	intergenic	G	A	intergenic
121055	intergenic	intergenic	C	T	intergenic
121057	intergenic	intergenic	C	G	intergenic
121075	intergenic	intergenic	C	T	intergenic
121078	intergenic	intergenic	T	C	intergenic
121087	intergenic	intergenic	A	G	intergenic
121094	intergenic	intergenic	T	C	intergenic
121096	intergenic	intergenic	C	T	intergenic
121138	NTHI477_RS00565	27	C	T	synonymous
121149	NTHI477_RS00565	38	T	A	nonsynonymous
121150	NTHI477_RS00565	39	G	A	synonymous
121151	NTHI477_RS00565	40	C	A	nonsynonymous
121152	NTHI477_RS00565	41	G	A	nonsynonymous
121168	NTHI477_RS00565	57	G	A	synonymous
121174	NTHI477_RS00565	63	C	T	synonymous
121192	NTHI477_RS00565	81	T	C	synonymous

121204	NTHI477_RS00565	93	T	G	nonsynonymous
121276	NTHI477_RS00565	165	G	A	synonymous
121301	NTHI477_RS00565	190	G	A	nonsynonymous
121312	NTHI477_RS00565	201	G	A	synonymous
121339	NTHI477_RS00565	228	A	G	synonymous
121378	NTHI477_RS00565	267	G	A	synonymous
121381	NTHI477_RS00565	270	C	T	synonymous
121384	NTHI477_RS00565	273	A	G	synonymous
121385	NTHI477_RS00565	274	G	T	nonsynonymous
121399	NTHI477_RS00565	288	A	C	synonymous
121412	NTHI477_RS00565	301	G	A	nonsynonymous
121420	NTHI477_RS00565	309	T	C	synonymous
121423	NTHI477_RS00565	312	C	T	synonymous
121426	NTHI477_RS00565	315	T	C	synonymous
121432	NTHI477_RS00565	321	C	T	synonymous
121441	NTHI477_RS00565	330	G	A	synonymous
121462	NTHI477_RS00565	351	G	A	synonymous
121465	NTHI477_RS00565	354	G	A	synonymous
121468	NTHI477_RS00565	357	A	C	synonymous
121498	NTHI477_RS00565	387	G	T	synonymous
121517	NTHI477_RS00565	406	C	A	nonsynonymous
121552	NTHI477_RS00565	441	T	C	synonymous
121561	NTHI477_RS00565	450	G	A	synonymous
121573	NTHI477_RS00565	462	A	G	synonymous
121576	NTHI477_RS00565	465	C	T	synonymous
121582	NTHI477_RS00565	471	T	C	synonymous
121588	NTHI477_RS00565	477	A	C	synonymous
121600	NTHI477_RS00565	489	C	T	synonymous
121615	NTHI477_RS00565	504	T	C	synonymous
121618	NTHI477_RS00565	507	A	T	synonymous
121640	NTHI477_RS00565	529	G	A	nonsynonymous
121643	NTHI477_RS00565	532	G	C	nonsynonymous
121645	NTHI477_RS00565	534	T	G	synonymous
121651	NTHI477_RS00565	540	G	A	synonymous
121654	NTHI477_RS00565	543	G	A	synonymous
121663	NTHI477_RS00565	552	A	C	nonsynonymous
121669	NTHI477_RS00565	558	T	C	synonymous
121674	NTHI477_RS00565	563	A	T	nonsynonymous
121681	NTHI477_RS00565	570	T	A	synonymous
121686	NTHI477_RS00565	575	C	A	nonsynonymous
121716	NTHI477_RS00565	605	A	C	nonsynonymous
121732	NTHI477_RS00570	13	C	A	nonsynonymous
121791	NTHI477_RS00570	72	A	T	synonymous
121794	NTHI477_RS00570	75	A	C	synonymous
121798	NTHI477_RS00570	79	A	G	nonsynonymous

121803	NTHI477_RS00570	84	A	G	synonymous
121803	NTHI477_RS00570	84	A	T	synonymous
121806	NTHI477_RS00570	87	C	A	synonymous
121806	NTHI477_RS00570	87	C	T	synonymous
121809	NTHI477_RS00570	90	T	A	synonymous
121811	NTHI477_RS00570	92	A	G	nonsynonymous
121815	NTHI477_RS00570	96	A	G	synonymous
121820	NTHI477_RS00570	101	T	C	nonsynonymous
121820	NTHI477_RS00570	101	T	A	nonsynonymous
121845	NTHI477_RS00570	126	T	C	synonymous
121857	NTHI477_RS00570	138	G	A	synonymous
121866	NTHI477_RS00570	147	T	A	synonymous
121886	NTHI477_RS00570	167	C	T	nonsynonymous
121887	NTHI477_RS00570	168	A	G	synonymous
121890	NTHI477_RS00570	171	G	T	synonymous
121897	NTHI477_RS00570	178	T	C	synonymous
121899	NTHI477_RS00570	180	G	A	synonymous
121904	NTHI477_RS00570	185	T	C	nonsynonymous
121911	NTHI477_RS00570	192	C	T	synonymous
121923	NTHI477_RS00570	204	A	G	synonymous
121930	NTHI477_RS00570	211	G	C	nonsynonymous
121952	NTHI477_RS00570	233	G	C	nonsynonymous
121962	NTHI477_RS00570	243	G	A	synonymous
121991	NTHI477_RS00570	272	G	A	nonsynonymous
122011	NTHI477_RS00570	292	G	A	nonsynonymous
122022	NTHI477_RS00570	303	C	T	synonymous
122054	NTHI477_RS00570	335	C	A	nonsynonymous
122079	NTHI477_RS00570	360	C	T	synonymous
122082	NTHI477_RS00570	363	T	A	synonymous
122082	NTHI477_RS00570	363	T	C	synonymous
122085	NTHI477_RS00570	366	T	C	synonymous
122100	NTHI477_RS00570	381	T	C	synonymous
122105	NTHI477_RS00570	386	C	T	nonsynonymous
122118	NTHI477_RS00570	399	G	C	synonymous
122124	NTHI477_RS00570	405	A	G	synonymous
122130	NTHI477_RS00570	411	A	G	synonymous
122142	NTHI477_RS00570	423	T	C	synonymous
122149	NTHI477_RS00570	430	C	T	synonymous
122175	NTHI477_RS00570	456	T	C	synonymous
122181	NTHI477_RS00570	462	T	A	synonymous
122186	NTHI477_RS00570	467	C	A	nonsynonymous
122187	NTHI477_RS00570	468	G	T	synonymous
122196	NTHI477_RS00570	477	T	C	synonymous
122217	NTHI477_RS00570	498	G	A	synonymous
122219	NTHI477_RS00570	500	C	G	nonsynonymous

122221	NTHI477_RS00570	502	C	A	nonsynonymous
122244	NTHI477_RS00570	525	A	G	synonymous
122247	NTHI477_RS00570	528	G	A	synonymous
122280	intergenic	intergenic	A	G	intergenic
122291	intergenic	intergenic	C	T	intergenic
122299	intergenic	intergenic	C	A	intergenic
122313	intergenic	intergenic	G	A	intergenic
122351	NTHI477_RS00575	525	A	G	synonymous
122356	NTHI477_RS00575	520	T	C	nonsynonymous
122357	NTHI477_RS00575	519	A	T	synonymous
122361	NTHI477_RS00575	515	C	T	nonsynonymous
122372	NTHI477_RS00575	504	G	T	synonymous
122374	NTHI477_RS00575	502	C	T	nonsynonymous
122378	NTHI477_RS00575	498	C	T	synonymous
122381	NTHI477_RS00575	495	C	T	nonsynonymous
122384	NTHI477_RS00575	492	A	G	synonymous
122386	NTHI477_RS00575	490	T	C	nonsynonymous
122387	NTHI477_RS00575	489	T	C	synonymous
122399	NTHI477_RS00575	477	G	A	synonymous
122405	NTHI477_RS00575	471	C	G	synonymous
122438	NTHI477_RS00575	438	C	A	synonymous
122447	NTHI477_RS00575	429	T	C	synonymous
122454	NTHI477_RS00575	422	G	C	nonsynonymous
122455	NTHI477_RS00575	421	T	C	nonsynonymous
122456	NTHI477_RS00575	420	G	T	synonymous
122459	NTHI477_RS00575	417	A	C	nonsynonymous
122461	NTHI477_RS00575	415	T	C	nonsynonymous
122471	NTHI477_RS00575	405	C	T	synonymous
122486	NTHI477_RS00575	390	A	G	synonymous
122495	NTHI477_RS00575	381	T	C	synonymous
122507	NTHI477_RS00575	369	T	G	synonymous
122510	NTHI477_RS00575	366	A	C	synonymous
122521	NTHI477_RS00575	355	A	T	nonsynonymous
122525	NTHI477_RS00575	351	A	G	synonymous
122537	NTHI477_RS00575	339	A	G	synonymous
122540	NTHI477_RS00575	336	T	C	synonymous
122546	NTHI477_RS00575	330	C	T	synonymous
122548	NTHI477_RS00575	328	A	G	synonymous
122549	NTHI477_RS00575	327	C	T	nonsynonymous
122562	NTHI477_RS00575	314	T	C	nonsynonymous
122564	NTHI477_RS00575	312	T	A	synonymous
122567	NTHI477_RS00575	309	C	T	synonymous
122580	NTHI477_RS00575	296	G	A	nonsynonymous
122594	NTHI477_RS00575	282	T	G	synonymous
122597	NTHI477_RS00575	279	T	G	synonymous

122600	NTHI477_RS00575	276	C	T	synonymous
122603	NTHI477_RS00575	273	G	A	synonymous
122618	NTHI477_RS00575	258	T	C	synonymous
122621	NTHI477_RS00575	255	G	A	synonymous
122621	NTHI477_RS00575	255	G	T	synonymous
122626	NTHI477_RS00575	250	G	T	nonsynonymous
122639	NTHI477_RS00575	237	G	T	synonymous
122639	NTHI477_RS00575	237	G	A	synonymous
122645	NTHI477_RS00575	231	C	T	nonsynonymous
122646	NTHI477_RS00575	230	A	C	nonsynonymous
122653	NTHI477_RS00575	223	T	G	nonsynonymous
122657	NTHI477_RS00575	219	T	C	synonymous
122657	NTHI477_RS00575	219	T	G	nonsynonymous
122660	NTHI477_RS00575	216	C	T	synonymous
122660	NTHI477_RS00575	216	C	G	synonymous
122675	NTHI477_RS00575	201	T	C	synonymous
122684	NTHI477_RS00575	192	A	T	synonymous
122695	NTHI477_RS00575	181	G	A	synonymous
122696	NTHI477_RS00575	180	C	T	synonymous
122697	NTHI477_RS00575	179	G	T	nonsynonymous
122702	NTHI477_RS00575	174	T	C	synonymous
122710	NTHI477_RS00575	166	T	C	nonsynonymous
122720	NTHI477_RS00575	156	A	G	synonymous
122725	NTHI477_RS00575	151	C	T	nonsynonymous
122744	NTHI477_RS00575	132	A	G	synonymous
122756	NTHI477_RS00575	120	T	C	synonymous
122768	NTHI477_RS00575	108	C	T	synonymous
122769	NTHI477_RS00575	107	A	G	nonsynonymous
122780	NTHI477_RS00575	96	T	C	synonymous
122782	NTHI477_RS00575	94	C	A	nonsynonymous
122785	NTHI477_RS00575	91	C	T	nonsynonymous
122786	NTHI477_RS00575	90	G	A	synonymous
122793	NTHI477_RS00575	83	C	T	nonsynonymous
122793	NTHI477_RS00575	83	C	A	nonsynonymous
122816	NTHI477_RS00575	60	T	C	synonymous
122823	NTHI477_RS00575	53	A	G	nonsynonymous
122824	NTHI477_RS00575	52	C	T	nonsynonymous
122828	NTHI477_RS00575	48	C	T	synonymous
122837	NTHI477_RS00575	39	G	A	synonymous
122852	NTHI477_RS00575	24	G	T	synonymous
122861	NTHI477_RS00575	15	T	A	synonymous
122864	NTHI477_RS00575	12	A	T	synonymous
122866	NTHI477_RS00575	10	G	A	nonsynonymous
122867	NTHI477_RS00575	9	T	A	synonymous
122870	NTHI477_RS00575	6	A	G	synonymous

122884	intergenic	intergenic	C	T	intergenic
122887	intergenic	intergenic	T	C	intergenic
122887	intergenic	intergenic	T	A	intergenic
122890	intergenic	intergenic	T	A	intergenic
122891	intergenic	intergenic	A	G	intergenic
122893	intergenic	intergenic	G	A	intergenic
122906	intergenic	intergenic	G	A	intergenic
122914	intergenic	intergenic	G	A	intergenic
122926	intergenic	intergenic	A	G	intergenic
122932	intergenic	intergenic	C	A	intergenic
122948	intergenic	intergenic	T	G	intergenic
122948	intergenic	intergenic	T	C	intergenic
122949	intergenic	intergenic	A	G	intergenic
122980	NTHI477_RS00580	8	G	A	nonsynonymous
122993	NTHI477_RS00580	21	T	C	synonymous
123004	NTHI477_RS00580	32	T	C	nonsynonymous
123005	NTHI477_RS00580	33	G	A	synonymous
123008	NTHI477_RS00580	36	G	T	synonymous
123038	NTHI477_RS00580	66	T	C	synonymous
123050	NTHI477_RS00580	78	C	T	synonymous
123057	NTHI477_RS00580	85	C	T	synonymous
123059	NTHI477_RS00580	87	A	G	synonymous
123060	NTHI477_RS00580	88	T	C	synonymous
123063	NTHI477_RS00580	91	C	T	synonymous
123071	NTHI477_RS00580	99	G	C	synonymous
123072	NTHI477_RS00580	100	A	G	nonsynonymous
123074	NTHI477_RS00580	102	T	A	synonymous
123089	NTHI477_RS00580	117	T	C	synonymous
123092	NTHI477_RS00580	120	C	T	synonymous
123095	NTHI477_RS00580	123	G	A	synonymous
123107	NTHI477_RS00580	135	T	A	synonymous
123108	NTHI477_RS00580	136	G	A	nonsynonymous
123115	NTHI477_RS00580	143	C	T	nonsynonymous
123116	NTHI477_RS00580	144	G	A	synonymous
123117	NTHI477_RS00580	145	A	G	nonsynonymous
123119	NTHI477_RS00580	147	C	G	nonsynonymous
123122	NTHI477_RS00580	150	A	T	nonsynonymous
123125	NTHI477_RS00580	153	C	G	synonymous
123125	NTHI477_RS00580	153	C	T	synonymous
123128	NTHI477_RS00580	156	A	G	synonymous
123134	NTHI477_RS00580	162	G	A	synonymous
123135	NTHI477_RS00580	163	C	G	nonsynonymous
123137	NTHI477_RS00580	165	C	T	synonymous
123137	NTHI477_RS00580	165	C	G	synonymous
123149	NTHI477_RS00580	177	T	C	synonymous

123153	NTHI477_RS00580	181	T	C	synonymous
123167	NTHI477_RS00580	195	A	T	synonymous
123167	NTHI477_RS00580	195	A	G	synonymous
123175	NTHI477_RS00580	203	C	T	nonsynonymous
123194	NTHI477_RS00580	222	G	A	synonymous
123215	NTHI477_RS00580	243	G	A	synonymous
123217	NTHI477_RS00580	245	A	G	nonsynonymous
123218	NTHI477_RS00580	246	T	A	nonsynonymous
123218	NTHI477_RS00580	246	T	C	synonymous
123221	NTHI477_RS00580	249	C	T	synonymous
123225	NTHI477_RS00580	253	A	C	synonymous
123233	NTHI477_RS00580	261	T	C	synonymous
123239	NTHI477_RS00580	267	G	T	synonymous
123245	NTHI477_RS00580	273	T	C	synonymous
123246	NTHI477_RS00580	274	G	A	nonsynonymous
123257	NTHI477_RS00580	285	C	T	synonymous
123270	NTHI477_RS00580	298	G	A	nonsynonymous
123302	NTHI477_RS00580	330	A	C	synonymous
123302	NTHI477_RS00580	330	A	T	synonymous
123320	NTHI477_RS00580	348	A	G	synonymous
123321	NTHI477_RS00580	349	G	A	nonsynonymous
123326	NTHI477_RS00580	354	A	G	synonymous
123332	NTHI477_RS00580	360	A	G	synonymous
123335	NTHI477_RS00580	363	C	T	synonymous
123353	NTHI477_RS00580	381	T	C	synonymous
123354	NTHI477_RS00580	382	G	A	nonsynonymous
123356	NTHI477_RS00580	384	G	T	synonymous
123365	NTHI477_RS00580	393	T	C	synonymous
123368	NTHI477_RS00580	396	C	G	nonsynonymous
123368	NTHI477_RS00580	396	C	T	synonymous
123375	NTHI477_RS00580	403	G	A	nonsynonymous
123376	NTHI477_RS00580	404	A	G	nonsynonymous
123383	NTHI477_RS00580	411	G	A	synonymous
123404	NTHI477_RS00580	432	A	G	synonymous
123416	NTHI477_RS00580	444	A	C	synonymous
123422	NTHI477_RS00580	450	A	G	synonymous
123437	NTHI477_RS00580	465	G	C	synonymous
123440	NTHI477_RS00580	468	C	T	synonymous
123446	NTHI477_RS00580	474	T	G	synonymous
123515	NTHI477_RS00580	543	T	C	synonymous
123521	NTHI477_RS00580	549	G	A	synonymous
123527	NTHI477_RS00580	555	C	A	synonymous
123545	NTHI477_RS00580	573	G	A	synonymous
123563	NTHI477_RS00580	591	A	G	synonymous
123578	NTHI477_RS00580	606	C	A	synonymous

123581	NTHI477_RS00580	609	G	A	synonymous
123584	NTHI477_RS00580	612	A	T	synonymous
123635	NTHI477_RS00580	663	A	G	synonymous
123647	NTHI477_RS00580	675	C	T	synonymous
123680	NTHI477_RS00580	708	T	C	synonymous
123705	NTHI477_RS00580	733	A	C	nonsynonymous
123706	NTHI477_RS00580	734	A	G	nonsynonymous
123708	NTHI477_RS00580	736	G	A	nonsynonymous
123728	NTHI477_RS00580	756	G	A	synonymous
123732	NTHI477_RS00580	760	C	T	nonsynonymous
123734	NTHI477_RS00580	762	T	A	synonymous
123735	NTHI477_RS00580	763	G	T	nonsynonymous
123737	NTHI477_RS00580	765	A	G	synonymous
123740	NTHI477_RS00580	768	A	T	nonsynonymous
123767	NTHI477_RS00580	795	G	C	synonymous
123770	NTHI477_RS00580	798	A	T	synonymous
123773	NTHI477_RS00580	801	C	T	synonymous
123775	NTHI477_RS00580	803	T	C	nonsynonymous
123776	NTHI477_RS00580	804	A	C	synonymous
123797	NTHI477_RS00580	825	T	C	synonymous
123800	NTHI477_RS00580	828	T	C	synonymous
123815	NTHI477_RS00580	843	G	A	synonymous
123831	NTHI477_RS00580	859	C	T	synonymous
123833	NTHI477_RS00580	861	A	C	synonymous
123833	NTHI477_RS00580	861	A	T	synonymous
123834	NTHI477_RS00580	862	G	A	nonsynonymous
123848	NTHI477_RS00580	876	C	T	synonymous
123854	NTHI477_RS00580	882	A	G	synonymous
123857	NTHI477_RS00580	885	T	C	synonymous
123868	NTHI477_RS00580	896	C	T	nonsynonymous
123872	NTHI477_RS00580	900	C	T	synonymous
123896	NTHI477_RS00580	924	G	A	synonymous
123903	NTHI477_RS00580	931	C	T	synonymous
123914	NTHI477_RS00580	942	T	C	synonymous
123959	NTHI477_RS00580	987	T	G	synonymous
123962	NTHI477_RS00580	990	A	G	synonymous
123966	NTHI477_RS00580	994	A	G	nonsynonymous
123974	NTHI477_RS00580	1002	T	C	synonymous
123992	NTHI477_RS00580	1020	A	T	nonsynonymous
123999	NTHI477_RS00580	1027	G	T	nonsynonymous
124001	NTHI477_RS00580	1029	T	A	synonymous
124004	NTHI477_RS00580	1032	T	C	synonymous
124007	NTHI477_RS00580	1035	T	C	synonymous
124010	NTHI477_RS00580	1038	T	C	synonymous
124013	NTHI477_RS00580	1041	T	C	synonymous

124019	NTHI477_RS00580	1047	G	A	synonymous
124020	NTHI477_RS00580	1048	C	A	synonymous
124028	NTHI477_RS00580	1056	T	C	synonymous
124037	NTHI477_RS00580	1065	C	T	synonymous
124038	NTHI477_RS00580	1066	C	T	nonsynonymous
124049	NTHI477_RS00580	1077	T	C	synonymous
124052	NTHI477_RS00580	1080	T	A	synonymous
124055	NTHI477_RS00580	1083	G	A	synonymous
124061	NTHI477_RS00580	1089	C	T	synonymous
124064	NTHI477_RS00580	1092	G	T	synonymous
124065	NTHI477_RS00580	1093	A	G	nonsynonymous
124068	NTHI477_RS00580	1096	C	T	synonymous
124073	NTHI477_RS00580	1101	C	A	synonymous
124076	NTHI477_RS00580	1104	T	G	synonymous
124085	NTHI477_RS00580	1113	T	A	synonymous
124088	NTHI477_RS00580	1116	C	T	synonymous
124091	NTHI477_RS00580	1119	A	T	synonymous
124106	NTHI477_RS00580	1134	T	A	synonymous
124115	NTHI477_RS00580	1143	C	T	synonymous
124118	NTHI477_RS00580	1146	A	T	synonymous
124127	NTHI477_RS00580	1155	T	A	synonymous
124133	NTHI477_RS00580	1161	C	T	synonymous
124145	NTHI477_RS00580	1173	T	C	synonymous
124148	NTHI477_RS00580	1176	A	T	synonymous
124172	NTHI477_RS00580	1200	T	C	synonymous
124181	NTHI477_RS00580	1209	A	T	synonymous
124181	NTHI477_RS00580	1209	A	C	synonymous
124184	NTHI477_RS00580	1212	A	G	synonymous
124187	NTHI477_RS00580	1215	A	T	synonymous
124190	NTHI477_RS00580	1218	T	C	synonymous
124234	NTHI477_RS00580	1262	C	A	nonsynonymous
124238	NTHI477_RS00580	1266	T	C	synonymous
124241	NTHI477_RS00580	1269	A	T	synonymous
124247	NTHI477_RS00580	1275	G	A	synonymous
124250	NTHI477_RS00580	1278	T	C	synonymous
124266	NTHI477_RS00580	1294	A	G	nonsynonymous
124283	NTHI477_RS00580	1311	C	T	synonymous
124292	NTHI477_RS00580	1320	T	C	synonymous
124295	NTHI477_RS00580	1323	T	A	synonymous
124304	NTHI477_RS00580	1332	A	C	synonymous
124307	NTHI477_RS00580	1335	C	A	synonymous
124311	NTHI477_RS00580	1339	A	C	nonsynonymous
124312	NTHI477_RS00580	1340	A	G	nonsynonymous
124314	NTHI477_RS00580	1342	G	T	nonsynonymous
124346	NTHI477_RS00580	1374	T	G	nonsynonymous

124352	NTHI477_RS00580	1380	T	A	synonymous
124352	NTHI477_RS00580	1380	T	C	synonymous
124363	NTHI477_RS00580	1391	C	G	nonsynonymous
124364	NTHI477_RS00580	1392	A	G	synonymous
124376	NTHI477_RS00580	1404	C	A	synonymous
124407	NTHI477_RS00580	1435	T	C	nonsynonymous
124409	NTHI477_RS00580	1437	C	T	synonymous
124419	NTHI477_RS00580	1447	A	C	synonymous
124436	NTHI477_RS00580	1464	T	C	synonymous
124448	NTHI477_RS00580	1476	C	T	synonymous
124462	NTHI477_RS00580	1490	T	C	nonsynonymous
124478	NTHI477_RS00580	1506	G	T	synonymous
124487	NTHI477_RS00580	1515	G	T	synonymous
124499	NTHI477_RS00580	1527	G	A	synonymous
124502	NTHI477_RS00580	1530	C	T	synonymous
124508	NTHI477_RS00580	1536	T	C	synonymous
124526	NTHI477_RS00580	1554	A	G	synonymous
124538	NTHI477_RS00580	1566	A	C	synonymous
124538	NTHI477_RS00580	1566	A	T	synonymous
124556	NTHI477_RS00580	1584	G	A	synonymous
124559	NTHI477_RS00580	1587	C	T	synonymous
124571	NTHI477_RS00580	1599	G	A	synonymous
124580	NTHI477_RS00580	1608	G	A	synonymous
124604	NTHI477_RS00580	1632	G	A	synonymous
124607	NTHI477_RS00580	1635	A	G	synonymous
124616	NTHI477_RS00580	1644	C	T	synonymous
124628	NTHI477_RS00580	1656	T	C	synonymous
124631	NTHI477_RS00580	1659	A	T	synonymous
124640	NTHI477_RS00580	1668	C	T	synonymous
124643	NTHI477_RS00580	1671	T	G	synonymous
124655	NTHI477_RS00580	1683	C	T	synonymous
124658	NTHI477_RS00580	1686	C	T	synonymous
124664	NTHI477_RS00580	1692	G	A	synonymous
124682	NTHI477_RS00580	1710	G	T	synonymous
124683	NTHI477_RS00580	1711	G	A	nonsynonymous
124685	NTHI477_RS00580	1713	G	T	synonymous
124691	NTHI477_RS00580	1719	G	A	synonymous
124694	NTHI477_RS00580	1722	G	A	synonymous
124699	NTHI477_RS00580	1727	T	C	nonsynonymous
124709	NTHI477_RS00580	1737	T	A	synonymous
124713	NTHI477_RS00580	1741	T	C	synonymous
124718	NTHI477_RS00580	1746	T	A	synonymous
124718	NTHI477_RS00580	1746	T	G	synonymous
124719	NTHI477_RS00580	1747	G	C	nonsynonymous
124751	NTHI477_RS00580	1779	T	G	nonsynonymous

124752	NTHI477_RS00580	1780	A	G	nonsynonymous
124784	NTHI477_RS00580	1812	A	G	synonymous
124784	NTHI477_RS00580	1812	A	C	nonsynonymous
124786	NTHI477_RS00580	1814	A	C	nonsynonymous
124796	NTHI477_RS00580	1824	T	C	synonymous
124806	NTHI477_RS00580	1834	G	A	nonsynonymous
124808	NTHI477_RS00580	1836	A	G	synonymous
124811	NTHI477_RS00580	1839	A	C	nonsynonymous
124811	NTHI477_RS00580	1839	A	G	synonymous
124814	NTHI477_RS00580	1842	G	T	synonymous
124815	NTHI477_RS00580	1843	A	C	nonsynonymous
124840	intergenic	intergenic	T	C	intergenic
124855	intergenic	intergenic	C	T	intergenic
124857	intergenic	intergenic	T	C	intergenic
124889	NTHI477_RS00585	767	G	T	nonsynonymous
124891	NTHI477_RS00585	765	C	T	synonymous
124897	NTHI477_RS00585	759	G	C	nonsynonymous
124906	NTHI477_RS00585	750	T	A	synonymous
124907	NTHI477_RS00585	749	G	A	nonsynonymous
124907	NTHI477_RS00585	749	G	T	nonsynonymous
124909	NTHI477_RS00585	747	T	G	synonymous
124912	NTHI477_RS00585	744	A	G	synonymous
124915	NTHI477_RS00585	741	C	T	synonymous
124917	NTHI477_RS00585	739	T	G	nonsynonymous
124924	NTHI477_RS00585	732	A	G	synonymous
124927	NTHI477_RS00585	729	T	G	synonymous
124930	NTHI477_RS00585	726	A	G	synonymous
124939	NTHI477_RS00585	717	T	C	synonymous
124980	NTHI477_RS00585	676	G	T	synonymous
124981	NTHI477_RS00585	675	T	A	synonymous
124981	NTHI477_RS00585	675	T	C	synonymous
124984	NTHI477_RS00585	672	T	C	synonymous
125001	NTHI477_RS00585	655	T	C	nonsynonymous
125002	NTHI477_RS00585	654	A	C	synonymous
125004	NTHI477_RS00585	652	C	T	nonsynonymous
125005	NTHI477_RS00585	651	A	G	synonymous
125008	NTHI477_RS00585	648	G	A	synonymous
125020	NTHI477_RS00585	636	C	T	synonymous
125023	NTHI477_RS00585	633	C	T	synonymous
125023	NTHI477_RS00585	633	C	A	synonymous
125025	NTHI477_RS00585	631	G	A	nonsynonymous
125034	NTHI477_RS00585	622	A	C	nonsynonymous
125038	NTHI477_RS00585	618	T	A	nonsynonymous
125040	NTHI477_RS00585	616	G	T	nonsynonymous
125047	NTHI477_RS00585	609	A	G	synonymous

125053	NTHI477_RS00585	603	G	A	synonymous
125068	NTHI477_RS00585	588	A	G	synonymous
125088	NTHI477_RS00585	568	T	C	nonsynonymous
125107	NTHI477_RS00585	549	G	A	synonymous
125116	NTHI477_RS00585	540	T	A	nonsynonymous
125118	NTHI477_RS00585	538	A	G	synonymous
125125	NTHI477_RS00585	531	T	C	synonymous
125142	NTHI477_RS00585	514	G	A	synonymous
125142	NTHI477_RS00585	514	G	T	nonsynonymous
125143	NTHI477_RS00585	513	A	T	nonsynonymous
125146	NTHI477_RS00585	510	G	A	synonymous
125152	NTHI477_RS00585	504	T	C	synonymous
125158	NTHI477_RS00585	498	A	G	synonymous
125159	NTHI477_RS00585	497	T	C	nonsynonymous
125160	NTHI477_RS00585	496	T	C	nonsynonymous
125167	NTHI477_RS00585	489	A	C	synonymous
125167	NTHI477_RS00585	489	A	T	synonymous
125169	NTHI477_RS00585	487	C	T	nonsynonymous
125171	NTHI477_RS00585	485	T	C	nonsynonymous
125173	NTHI477_RS00585	483	T	C	synonymous
125174	NTHI477_RS00585	482	T	C	nonsynonymous
125175	NTHI477_RS00585	481	T	G	nonsynonymous
125175	NTHI477_RS00585	481	T	A	nonsynonymous
125176	NTHI477_RS00585	480	C	T	synonymous
125178	NTHI477_RS00585	478	C	A	nonsynonymous
125179	NTHI477_RS00585	477	G	A	synonymous
125179	NTHI477_RS00585	477	G	C	synonymous
125192	NTHI477_RS00585	464	G	T	nonsynonymous
125193	NTHI477_RS00585	463	C	A	nonsynonymous
125194	NTHI477_RS00585	462	C	T	synonymous
125209	NTHI477_RS00585	447	T	C	synonymous
125212	NTHI477_RS00585	444	G	A	synonymous
125218	NTHI477_RS00585	438	G	A	synonymous
125219	NTHI477_RS00585	437	G	T	nonsynonymous
125221	NTHI477_RS00585	435	A	G	synonymous
125224	NTHI477_RS00585	432	G	T	synonymous
125224	NTHI477_RS00585	432	G	A	synonymous
125225	NTHI477_RS00585	431	C	A	nonsynonymous
125228	NTHI477_RS00585	428	A	C	nonsynonymous
125230	NTHI477_RS00585	426	T	A	nonsynonymous
125232	NTHI477_RS00585	424	T	G	nonsynonymous
125234	NTHI477_RS00585	422	A	G	nonsynonymous
125241	NTHI477_RS00585	415	G	T	synonymous
125242	NTHI477_RS00585	414	A	G	synonymous
125244	NTHI477_RS00585	412	G	A	nonsynonymous

125245	NTHI477_RS00585	411	C	T	synonymous
125254	NTHI477_RS00585	402	C	A	synonymous
125256	NTHI477_RS00585	400	G	A	synonymous
125257	NTHI477_RS00585	399	T	A	synonymous
125259	NTHI477_RS00585	397	G	A	nonsynonymous
125260	NTHI477_RS00585	396	G	A	synonymous
125261	NTHI477_RS00585	395	T	C	nonsynonymous
125263	NTHI477_RS00585	393	G	A	synonymous
125275	NTHI477_RS00585	381	T	A	nonsynonymous
125275	NTHI477_RS00585	381	T	C	synonymous
125277	NTHI477_RS00585	379	C	T	nonsynonymous
125281	NTHI477_RS00585	375	C	A	synonymous
125281	NTHI477_RS00585	375	C	T	synonymous
125283	NTHI477_RS00585	373	G	T	nonsynonymous
125284	NTHI477_RS00585	372	T	C	synonymous
125287	NTHI477_RS00585	369	G	A	synonymous
125287	NTHI477_RS00585	369	G	C	synonymous
125293	NTHI477_RS00585	363	A	G	synonymous
125299	NTHI477_RS00585	357	A	G	synonymous
125308	NTHI477_RS00585	348	C	T	nonsynonymous
125308	NTHI477_RS00585	348	C	G	nonsynonymous
125317	NTHI477_RS00585	339	T	C	synonymous
125319	NTHI477_RS00585	337	G	C	nonsynonymous
125327	NTHI477_RS00585	329	C	T	nonsynonymous
125332	NTHI477_RS00585	324	T	C	synonymous
125336	NTHI477_RS00585	320	G	A	nonsynonymous
125337	NTHI477_RS00585	319	C	T	nonsynonymous
125338	NTHI477_RS00585	318	A	T	nonsynonymous
125341	NTHI477_RS00585	315	T	G	synonymous
125353	NTHI477_RS00585	303	G	A	synonymous
125356	NTHI477_RS00585	300	G	A	synonymous
125362	NTHI477_RS00585	294	G	A	synonymous
125365	NTHI477_RS00585	291	A	T	synonymous
125365	NTHI477_RS00585	291	A	G	synonymous
125373	NTHI477_RS00585	283	C	T	nonsynonymous
125380	NTHI477_RS00585	276	A	T	synonymous
125383	NTHI477_RS00585	273	A	G	synonymous
125383	NTHI477_RS00585	273	A	T	synonymous
125386	NTHI477_RS00585	270	A	C	nonsynonymous
125388	NTHI477_RS00585	268	A	T	nonsynonymous
125390	NTHI477_RS00585	266	T	A	nonsynonymous
125395	NTHI477_RS00585	261	A	T	synonymous
125398	NTHI477_RS00585	258	G	A	synonymous
125398	NTHI477_RS00585	258	G	T	synonymous
125404	NTHI477_RS00585	252	T	C	synonymous

125407	NTHI477_RS00585	249	C	T	synonymous
125408	NTHI477_RS00585	248	G	A	nonsynonymous
125410	NTHI477_RS00585	246	C	T	synonymous
125413	NTHI477_RS00585	243	T	C	synonymous
125414	NTHI477_RS00585	242	T	C	nonsynonymous
125416	NTHI477_RS00585	240	C	T	nonsynonymous
125419	NTHI477_RS00585	237	A	G	synonymous
125420	NTHI477_RS00585	236	T	C	nonsynonymous
125422	NTHI477_RS00585	234	G	A	synonymous
125425	NTHI477_RS00585	231	G	T	nonsynonymous
125426	NTHI477_RS00585	230	T	C	nonsynonymous
125427	NTHI477_RS00585	229	G	A	nonsynonymous
125431	NTHI477_RS00585	225	G	A	synonymous
125434	NTHI477_RS00585	222	A	T	synonymous
125435	NTHI477_RS00585	221	G	T	nonsynonymous
125440	NTHI477_RS00585	216	T	A	nonsynonymous
125441	NTHI477_RS00585	215	T	C	nonsynonymous
125443	NTHI477_RS00585	213	A	T	nonsynonymous
125445	NTHI477_RS00585	211	A	G	nonsynonymous
125450	NTHI477_RS00585	206	A	G	nonsynonymous
125451	NTHI477_RS00585	205	C	T	nonsynonymous
125454	NTHI477_RS00585	202	C	T	nonsynonymous
125455	NTHI477_RS00585	201	A	G	synonymous
125457	NTHI477_RS00585	199	C	T	nonsynonymous
125458	NTHI477_RS00585	198	G	A	synonymous
125461	NTHI477_RS00585	195	C	T	synonymous
125462	NTHI477_RS00585	194	C	T	nonsynonymous
125476	NTHI477_RS00585	180	A	G	synonymous
125488	NTHI477_RS00585	168	A	T	synonymous
125497	NTHI477_RS00585	159	A	G	synonymous
125500	NTHI477_RS00585	156	A	G	synonymous
125503	NTHI477_RS00585	153	A	G	synonymous
125518	NTHI477_RS00585	138	G	T	synonymous
125523	NTHI477_RS00585	133	T	G	nonsynonymous
125524	NTHI477_RS00585	132	G	A	synonymous
125527	NTHI477_RS00585	129	G	A	synonymous
125533	NTHI477_RS00585	123	A	T	synonymous
125536	NTHI477_RS00585	120	A	T	synonymous
125539	NTHI477_RS00585	117	G	T	synonymous
125542	NTHI477_RS00585	114	T	C	synonymous
125544	NTHI477_RS00585	112	A	G	synonymous
125545	NTHI477_RS00585	111	T	G	synonymous
125554	NTHI477_RS00585	102	A	G	synonymous
125557	NTHI477_RS00585	99	A	G	synonymous
125567	NTHI477_RS00585	89	T	C	nonsynonymous

125579	NTHI477_RS00585	77	C	T	nonsynonymous
125580	NTHI477_RS00585	76	G	T	synonymous
125589	NTHI477_RS00585	67	A	G	synonymous
125590	NTHI477_RS00585	66	T	A	synonymous
125608	NTHI477_RS00585	48	T	G	synonymous
125613	NTHI477_RS00585	43	T	C	nonsynonymous
125623	NTHI477_RS00585	33	G	T	synonymous
125626	NTHI477_RS00585	30	T	C	synonymous
125632	NTHI477_RS00585	24	T	C	synonymous
125638	NTHI477_RS00585	18	G	A	synonymous
125641	NTHI477_RS00585	15	C	T	synonymous
125641	NTHI477_RS00585	15	C	A	synonymous
125644	NTHI477_RS00585	12	T	C	synonymous
125668	NTHI477_RS00590	689	G	T	nonsynonymous
125672	NTHI477_RS00590	685	G	T	nonsynonymous
125676	NTHI477_RS00590	681	C	A	nonsynonymous
125678	NTHI477_RS00590	679	A	G	synonymous
125691	NTHI477_RS00590	666	A	G	synonymous
125692	NTHI477_RS00590	665	T	C	nonsynonymous
125694	NTHI477_RS00590	663	C	G	nonsynonymous
125700	NTHI477_RS00590	657	A	G	synonymous
125718	NTHI477_RS00590	639	T	C	synonymous
125732	NTHI477_RS00590	625	C	T	nonsynonymous
125733	NTHI477_RS00590	624	A	G	synonymous
125734	NTHI477_RS00590	623	T	C	nonsynonymous
125735	NTHI477_RS00590	622	T	G	nonsynonymous
125745	NTHI477_RS00590	612	T	C	synonymous
125747	NTHI477_RS00590	610	C	G	nonsynonymous
125748	NTHI477_RS00590	609	C	T	synonymous
125753	NTHI477_RS00590	604	A	G	nonsynonymous
125754	NTHI477_RS00590	603	G	A	synonymous
125756	NTHI477_RS00590	601	T	C	nonsynonymous
125760	NTHI477_RS00590	597	C	T	synonymous
125766	NTHI477_RS00590	591	C	A	nonsynonymous
125781	NTHI477_RS00590	576	C	T	synonymous
125790	NTHI477_RS00590	567	A	G	synonymous
125803	NTHI477_RS00590	554	T	G	nonsynonymous
125804	NTHI477_RS00590	553	T	C	nonsynonymous
125805	NTHI477_RS00590	552	T	G	synonymous
125815	NTHI477_RS00590	542	A	G	nonsynonymous
125817	NTHI477_RS00590	540	C	T	synonymous
125818	NTHI477_RS00590	539	C	T	nonsynonymous
125827	NTHI477_RS00590	530	C	T	nonsynonymous
125830	NTHI477_RS00590	527	G	A	nonsynonymous
125831	NTHI477_RS00590	526	T	A	nonsynonymous

125832	NTHI477_RS00590	525	G	A	synonymous
125835	NTHI477_RS00590	522	T	A	nonsynonymous
125844	NTHI477_RS00590	513	C	T	synonymous
125846	NTHI477_RS00590	511	A	G	synonymous
125848	NTHI477_RS00590	509	G	T	nonsynonymous
125849	NTHI477_RS00590	508	T	C	nonsynonymous
125852	NTHI477_RS00590	505	G	A	synonymous
125856	NTHI477_RS00590	501	G	T	synonymous
125856	NTHI477_RS00590	501	G	A	synonymous
125857	NTHI477_RS00590	500	A	G	nonsynonymous
125861	NTHI477_RS00590	496	T	C	nonsynonymous
125869	NTHI477_RS00590	488	C	T	nonsynonymous
125871	NTHI477_RS00590	486	A	T	synonymous
125874	NTHI477_RS00590	483	T	A	synonymous
125876	NTHI477_RS00590	481	T	C	nonsynonymous
125880	NTHI477_RS00590	477	A	G	synonymous
125881	NTHI477_RS00590	476	T	A	nonsynonymous
125883	NTHI477_RS00590	474	A	T	synonymous
125888	NTHI477_RS00590	469	C	T	nonsynonymous
125889	NTHI477_RS00590	468	C	T	synonymous
125895	NTHI477_RS00590	462	C	T	synonymous
125898	NTHI477_RS00590	459	G	A	synonymous
125901	NTHI477_RS00590	456	A	G	synonymous
125903	NTHI477_RS00590	454	G	A	nonsynonymous
125904	NTHI477_RS00590	453	C	T	synonymous
125907	NTHI477_RS00590	450	G	A	synonymous
125910	NTHI477_RS00590	447	T	C	synonymous
125912	NTHI477_RS00590	445	A	G	synonymous
125916	NTHI477_RS00590	441	T	A	synonymous
125922	NTHI477_RS00590	435	C	A	nonsynonymous
125929	NTHI477_RS00590	428	C	T	nonsynonymous
125931	NTHI477_RS00590	426	C	T	nonsynonymous
125944	NTHI477_RS00590	413	T	C	nonsynonymous
125946	NTHI477_RS00590	411	A	G	synonymous
125954	NTHI477_RS00590	403	A	G	synonymous
125958	NTHI477_RS00590	399	C	T	synonymous
125963	NTHI477_RS00590	394	G	A	synonymous
125973	NTHI477_RS00590	384	G	A	synonymous
125976	NTHI477_RS00590	381	C	A	synonymous
125979	NTHI477_RS00590	378	T	G	nonsynonymous
125981	NTHI477_RS00590	376	T	C	nonsynonymous
125982	NTHI477_RS00590	375	A	C	synonymous
125988	NTHI477_RS00590	369	A	G	synonymous
125991	NTHI477_RS00590	366	A	G	synonymous
125994	NTHI477_RS00590	363	T	C	synonymous

126004	NTHI477_RS00590	353	T	G	nonsynonymous
126006	NTHI477_RS00590	351	G	A	synonymous
126012	NTHI477_RS00590	345	T	C	synonymous
126014	NTHI477_RS00590	343	A	G	nonsynonymous
126016	NTHI477_RS00590	341	G	A	nonsynonymous
126018	NTHI477_RS00590	339	T	C	synonymous
126021	NTHI477_RS00590	336	C	T	synonymous
126042	NTHI477_RS00590	315	A	G	synonymous
126048	NTHI477_RS00590	309	T	A	synonymous
126054	NTHI477_RS00590	303	C	T	synonymous
126063	NTHI477_RS00590	294	A	C	synonymous
126076	NTHI477_RS00590	281	T	C	nonsynonymous
126090	NTHI477_RS00590	267	T	C	synonymous
126114	NTHI477_RS00590	243	G	A	synonymous
126122	NTHI477_RS00590	235	C	T	nonsynonymous
126144	NTHI477_RS00590	213	C	T	synonymous
126154	NTHI477_RS00590	203	T	C	nonsynonymous
126189	NTHI477_RS00590	168	T	C	synonymous
126189	NTHI477_RS00590	168	T	G	nonsynonymous
126190	NTHI477_RS00590	167	A	C	nonsynonymous
126194	NTHI477_RS00590	163	A	G	nonsynonymous
126195	NTHI477_RS00590	162	C	T	synonymous
126198	NTHI477_RS00590	159	T	A	synonymous
126198	NTHI477_RS00590	159	T	C	synonymous
126203	NTHI477_RS00590	154	C	T	nonsynonymous
126213	NTHI477_RS00590	144	A	T	nonsynonymous
126216	NTHI477_RS00590	141	G	A	synonymous
126218	NTHI477_RS00590	139	C	T	nonsynonymous
126219	NTHI477_RS00590	138	G	A	synonymous
126225	NTHI477_RS00590	132	T	C	synonymous
126231	NTHI477_RS00590	126	A	T	synonymous
126231	NTHI477_RS00590	126	A	C	synonymous
126232	NTHI477_RS00590	125	C	T	nonsynonymous
126234	NTHI477_RS00590	123	T	C	synonymous
126236	NTHI477_RS00590	121	G	A	synonymous
126240	NTHI477_RS00590	117	A	C	synonymous
126242	NTHI477_RS00590	115	T	C	nonsynonymous
126243	NTHI477_RS00590	114	G	C	synonymous
126249	NTHI477_RS00590	108	C	T	synonymous
126255	NTHI477_RS00590	102	T	C	synonymous
126287	NTHI477_RS00590	70	A	C	nonsynonymous
126306	NTHI477_RS00590	51	G	A	synonymous
126309	NTHI477_RS00590	48	G	T	nonsynonymous
126310	NTHI477_RS00590	47	T	G	nonsynonymous
126318	NTHI477_RS00590	39	A	T	synonymous

126321	NTHI477_RS00590	36	G	A	synonymous
126329	NTHI477_RS00590	28	A	G	synonymous
126330	NTHI477_RS00590	27	T	G	synonymous
126336	NTHI477_RS00590	21	T	A	synonymous
126339	NTHI477_RS00590	18	T	C	synonymous
126345	NTHI477_RS00590	12	G	A	synonymous
126348	NTHI477_RS00590	9	T	C	synonymous
126413	NTHI477_RS00595	819	A	G	synonymous
126445	NTHI477_RS00595	787	G	T	nonsynonymous
126451	NTHI477_RS00595	781	T	G	nonsynonymous
126452	NTHI477_RS00595	780	A	C	synonymous
126461	NTHI477_RS00595	771	A	T	synonymous
126463	NTHI477_RS00595	769	G	A	nonsynonymous
126467	NTHI477_RS00595	765	A	T	synonymous
126470	NTHI477_RS00595	762	T	C	synonymous
126476	NTHI477_RS00595	756	T	A	synonymous
126478	NTHI477_RS00595	754	C	T	nonsynonymous
126485	NTHI477_RS00595	747	T	A	synonymous
126488	NTHI477_RS00595	744	G	A	synonymous
126546	NTHI477_RS00595	686	C	T	nonsynonymous
126560	NTHI477_RS00595	672	C	A	synonymous
126569	NTHI477_RS00595	663	T	G	synonymous
126622	NTHI477_RS00595	610	T	C	nonsynonymous
126692	NTHI477_RS00595	540	C	T	synonymous
126701	NTHI477_RS00595	531	A	T	synonymous
126704	NTHI477_RS00595	528	G	T	synonymous
126706	NTHI477_RS00595	526	G	A	nonsynonymous
126707	NTHI477_RS00595	525	A	C	synonymous
126721	NTHI477_RS00595	511	A	G	nonsynonymous
126722	NTHI477_RS00595	510	G	A	synonymous
126725	NTHI477_RS00595	507	G	A	synonymous
126731	NTHI477_RS00595	501	C	G	synonymous
126734	NTHI477_RS00595	498	T	G	synonymous
126749	NTHI477_RS00595	483	C	A	synonymous
126758	NTHI477_RS00595	474	A	G	synonymous
126764	NTHI477_RS00595	468	T	G	synonymous
126765	NTHI477_RS00595	467	A	G	nonsynonymous
126766	NTHI477_RS00595	466	T	C	nonsynonymous
126775	NTHI477_RS00595	457	T	A	nonsynonymous
126776	NTHI477_RS00595	456	A	G	synonymous
126778	NTHI477_RS00595	454	A	G	nonsynonymous
126783	NTHI477_RS00595	449	A	C	nonsynonymous
126785	NTHI477_RS00595	447	A	T	synonymous
126791	NTHI477_RS00595	441	A	G	synonymous
126795	NTHI477_RS00595	437	G	T	nonsynonymous

126798	NTHI477_RS00595	434	A	G	nonsynonymous
126799	NTHI477_RS00595	433	T	C	nonsynonymous
126802	NTHI477_RS00595	430	C	T	nonsynonymous
126806	NTHI477_RS00595	426	T	C	synonymous
126809	NTHI477_RS00595	423	T	G	synonymous
126821	NTHI477_RS00595	411	A	C	synonymous
126823	NTHI477_RS00595	409	G	A	nonsynonymous
126827	NTHI477_RS00595	405	C	T	synonymous
126829	NTHI477_RS00595	403	A	C	nonsynonymous
126835	NTHI477_RS00595	397	A	C	nonsynonymous
126841	NTHI477_RS00595	391	T	C	nonsynonymous
126842	NTHI477_RS00595	390	A	C	synonymous
126848	NTHI477_RS00595	384	G	T	synonymous
126849	NTHI477_RS00595	383	G	C	nonsynonymous
126851	NTHI477_RS00595	381	G	A	synonymous
126857	NTHI477_RS00595	375	G	A	synonymous
126860	NTHI477_RS00595	372	T	C	synonymous
126863	NTHI477_RS00595	369	G	T	synonymous
126863	NTHI477_RS00595	369	G	C	synonymous
126870	NTHI477_RS00595	362	T	C	nonsynonymous
126872	NTHI477_RS00595	360	G	A	synonymous
126874	NTHI477_RS00595	358	T	A	nonsynonymous
126875	NTHI477_RS00595	357	A	G	synonymous
126908	NTHI477_RS00595	324	C	T	synonymous
126916	NTHI477_RS00595	316	A	T	nonsynonymous
126920	NTHI477_RS00595	312	A	G	synonymous
126944	NTHI477_RS00595	288	A	T	synonymous
126944	NTHI477_RS00595	288	A	C	synonymous
126956	NTHI477_RS00595	276	T	C	synonymous
126984	NTHI477_RS00595	248	G	C	nonsynonymous
127001	NTHI477_RS00595	231	T	C	synonymous
127004	NTHI477_RS00595	228	G	A	synonymous
127012	NTHI477_RS00595	220	C	T	nonsynonymous
127029	NTHI477_RS00595	203	C	T	nonsynonymous
127030	NTHI477_RS00595	202	G	T	synonymous
127040	NTHI477_RS00595	192	G	A	synonymous
127059	NTHI477_RS00595	173	C	T	nonsynonymous
127065	NTHI477_RS00595	167	C	T	nonsynonymous
127066	NTHI477_RS00595	166	G	T	synonymous
127070	NTHI477_RS00595	162	T	C	synonymous
127074	NTHI477_RS00595	158	G	A	nonsynonymous
127076	NTHI477_RS00595	156	G	T	synonymous
127082	NTHI477_RS00595	150	G	T	synonymous
127085	NTHI477_RS00595	147	A	T	synonymous
127088	NTHI477_RS00595	144	T	A	nonsynonymous

127118	NTHI477_RS00595	114	T	C	synonymous
127127	NTHI477_RS00595	105	T	C	synonymous
127129	NTHI477_RS00595	103	C	T	nonsynonymous
127139	NTHI477_RS00595	93	C	T	synonymous
127142	NTHI477_RS00595	90	A	G	synonymous
127143	NTHI477_RS00595	89	G	T	nonsynonymous
127144	NTHI477_RS00595	88	T	C	nonsynonymous
127160	NTHI477_RS00595	72	C	T	synonymous
127166	NTHI477_RS00595	66	G	A	synonymous
127172	NTHI477_RS00595	60	C	T	synonymous
127177	NTHI477_RS00595	55	G	A	synonymous
127205	NTHI477_RS00595	27	C	T	synonymous
127227	NTHI477_RS00595	5	C	T	nonsynonymous
127237	NTHI477_RS00600	921	C	T	synonymous
127249	NTHI477_RS00600	909	C	T	synonymous
127264	NTHI477_RS00600	894	G	A	synonymous
127279	NTHI477_RS00600	879	C	T	synonymous
127297	NTHI477_RS00600	861	C	T	synonymous
127312	NTHI477_RS00600	846	T	C	synonymous
127354	NTHI477_RS00600	804	A	G	synonymous
127360	NTHI477_RS00600	798	G	A	synonymous
127420	NTHI477_RS00600	738	G	A	synonymous
127475	NTHI477_RS00600	683	A	G	nonsynonymous
127480	NTHI477_RS00600	678	A	C	synonymous
127483	NTHI477_RS00600	675	A	C	synonymous
127497	NTHI477_RS00600	661	G	A	synonymous
127498	NTHI477_RS00600	660	C	T	synonymous
127513	NTHI477_RS00600	645	C	A	synonymous
127513	NTHI477_RS00600	645	C	T	synonymous
127525	NTHI477_RS00600	633	A	G	synonymous
127528	NTHI477_RS00600	630	G	A	synonymous
127539	NTHI477_RS00600	619	G	A	synonymous
127543	NTHI477_RS00600	615	A	G	synonymous
127546	NTHI477_RS00600	612	A	G	synonymous
127552	NTHI477_RS00600	606	T	C	synonymous
127557	NTHI477_RS00600	601	A	G	synonymous
127585	NTHI477_RS00600	573	G	A	synonymous
127588	NTHI477_RS00600	570	C	T	synonymous
127602	NTHI477_RS00600	556	T	A	nonsynonymous
127603	NTHI477_RS00600	555	C	G	nonsynonymous
127615	NTHI477_RS00600	543	T	A	nonsynonymous
127617	NTHI477_RS00600	541	C	T	nonsynonymous
127630	NTHI477_RS00600	528	A	G	synonymous
127636	NTHI477_RS00600	522	A	G	synonymous
127655	NTHI477_RS00600	503	C	T	nonsynonymous

127657	NTHI477_RS00600	501	C	A	synonymous
127659	NTHI477_RS00600	499	A	G	nonsynonymous
127663	NTHI477_RS00600	495	A	T	synonymous
127670	NTHI477_RS00600	488	T	A	nonsynonymous
127705	NTHI477_RS00600	453	T	C	synonymous
127711	NTHI477_RS00600	447	A	G	synonymous
127738	NTHI477_RS00600	420	A	G	synonymous
127741	NTHI477_RS00600	417	G	A	synonymous
127756	NTHI477_RS00600	402	A	G	synonymous
127769	NTHI477_RS00600	389	C	T	nonsynonymous
127804	NTHI477_RS00600	354	C	T	synonymous
127817	NTHI477_RS00600	341	A	G	nonsynonymous
127819	NTHI477_RS00600	339	C	T	synonymous
127822	NTHI477_RS00600	336	G	A	synonymous
127828	NTHI477_RS00600	330	C	A	synonymous
127830	NTHI477_RS00600	328	G	T	nonsynonymous
127831	NTHI477_RS00600	327	C	A	nonsynonymous
127849	NTHI477_RS00600	309	A	G	synonymous
127855	NTHI477_RS00600	303	C	T	synonymous
127861	NTHI477_RS00600	297	T	C	synonymous
127864	NTHI477_RS00600	294	G	T	synonymous
127872	NTHI477_RS00600	286	T	G	nonsynonymous
127903	NTHI477_RS00600	255	A	G	synonymous
127917	NTHI477_RS00600	241	T	C	nonsynonymous
127921	NTHI477_RS00600	237	T	A	synonymous
127960	NTHI477_RS00600	198	C	T	synonymous
127983	NTHI477_RS00600	175	T	G	synonymous
127998	NTHI477_RS00600	160	T	C	nonsynonymous
128044	NTHI477_RS00600	114	G	A	synonymous
128060	NTHI477_RS00600	98	A	G	nonsynonymous
128080	NTHI477_RS00600	78	A	C	nonsynonymous
128089	NTHI477_RS00600	69	C	T	synonymous
128099	NTHI477_RS00600	59	C	T	nonsynonymous
128242	intergenic	intergenic	T	C	intergenic
128272	intergenic	intergenic	G	T	intergenic
128274	intergenic	intergenic	C	T	intergenic
128293	intergenic	intergenic	T	C	intergenic
128307	intergenic	intergenic	G	A	intergenic
128341	NTHI477_RS00605	1982	C	T	nonsynonymous
128343	NTHI477_RS00605	1980	C	T	synonymous
128344	NTHI477_RS00605	1979	G	A	nonsynonymous
128367	NTHI477_RS00605	1956	G	A	synonymous
128380	NTHI477_RS00605	1943	G	C	nonsynonymous
128424	NTHI477_RS00605	1899	G	A	synonymous
128430	NTHI477_RS00605	1893	G	A	synonymous

128436	NTHI477_RS00605	1887	G	T	synonymous
128439	NTHI477_RS00605	1884	T	A	synonymous
128442	NTHI477_RS00605	1881	A	G	synonymous
128445	NTHI477_RS00605	1878	G	A	synonymous
128448	NTHI477_RS00605	1875	G	A	synonymous
128475	NTHI477_RS00605	1848	G	C	nonsynonymous
128476	NTHI477_RS00605	1847	A	G	nonsynonymous
128478	NTHI477_RS00605	1845	T	C	synonymous
128486	NTHI477_RS00605	1837	G	T	nonsynonymous
128490	NTHI477_RS00605	1833	C	T	synonymous
128492	NTHI477_RS00605	1831	G	C	nonsynonymous
128500	NTHI477_RS00605	1823	G	A	nonsynonymous
128501	NTHI477_RS00605	1822	C	T	nonsynonymous
128511	NTHI477_RS00605	1812	A	G	synonymous
128515	NTHI477_RS00605	1808	G	T	nonsynonymous
128526	NTHI477_RS00605	1797	G	A	synonymous
128538	NTHI477_RS00605	1785	G	A	synonymous
128543	NTHI477_RS00605	1780	G	A	synonymous
128565	NTHI477_RS00605	1758	C	T	synonymous
128574	NTHI477_RS00605	1749	A	G	synonymous
128576	NTHI477_RS00605	1747	T	G	nonsynonymous
128577	NTHI477_RS00605	1746	C	A	synonymous
128580	NTHI477_RS00605	1743	G	C	synonymous
128589	NTHI477_RS00605	1734	A	C	synonymous
128591	NTHI477_RS00605	1732	C	T	nonsynonymous
128612	NTHI477_RS00605	1711	C	T	nonsynonymous
128613	NTHI477_RS00605	1710	A	G	synonymous
128619	NTHI477_RS00605	1704	G	A	synonymous
128622	NTHI477_RS00605	1701	A	G	synonymous
128625	NTHI477_RS00605	1698	G	T	synonymous
128628	NTHI477_RS00605	1695	C	T	synonymous
128631	NTHI477_RS00605	1692	T	A	synonymous
128634	NTHI477_RS00605	1689	A	C	synonymous
128637	NTHI477_RS00605	1686	C	G	nonsynonymous
128637	NTHI477_RS00605	1686	C	T	synonymous
128639	NTHI477_RS00605	1684	A	G	synonymous
128643	NTHI477_RS00605	1680	T	A	synonymous
128644	NTHI477_RS00605	1679	C	T	nonsynonymous
128646	NTHI477_RS00605	1677	A	T	synonymous
128649	NTHI477_RS00605	1674	A	T	synonymous
128654	NTHI477_RS00605	1669	C	T	nonsynonymous
128658	NTHI477_RS00605	1665	G	A	synonymous
128661	NTHI477_RS00605	1662	C	T	synonymous
128663	NTHI477_RS00605	1660	T	G	nonsynonymous
128664	NTHI477_RS00605	1659	T	A	synonymous

128664	NTHI477_RS00605	1659	T	C	synonymous
128671	NTHI477_RS00605	1652	T	G	nonsynonymous
128672	NTHI477_RS00605	1651	T	G	nonsynonymous
128673	NTHI477_RS00605	1650	C	T	synonymous
128676	NTHI477_RS00605	1647	C	G	nonsynonymous
128677	NTHI477_RS00605	1646	A	G	nonsynonymous
128678	NTHI477_RS00605	1645	T	C	nonsynonymous
128688	NTHI477_RS00605	1635	C	T	synonymous
128691	NTHI477_RS00605	1632	G	A	synonymous
128703	NTHI477_RS00605	1620	A	G	synonymous
128724	NTHI477_RS00605	1599	G	A	synonymous
128730	NTHI477_RS00605	1593	A	G	synonymous
128741	NTHI477_RS00605	1582	C	T	nonsynonymous
128748	NTHI477_RS00605	1575	A	T	synonymous
128753	NTHI477_RS00605	1570	T	C	nonsynonymous
128763	NTHI477_RS00605	1560	G	A	synonymous
128792	NTHI477_RS00605	1531	C	T	nonsynonymous
128793	NTHI477_RS00605	1530	G	T	synonymous
128802	NTHI477_RS00605	1521	G	A	synonymous
128865	NTHI477_RS00605	1458	G	A	synonymous
128868	NTHI477_RS00605	1455	A	G	synonymous
128880	NTHI477_RS00605	1443	A	G	synonymous
128912	NTHI477_RS00605	1411	T	C	nonsynonymous
128917	NTHI477_RS00605	1406	T	C	nonsynonymous
128934	NTHI477_RS00605	1389	G	A	synonymous
128940	NTHI477_RS00605	1383	A	C	synonymous
128958	NTHI477_RS00605	1365	G	A	synonymous
128977	NTHI477_RS00605	1346	G	A	nonsynonymous
128984	NTHI477_RS00605	1339	C	T	nonsynonymous
129004	NTHI477_RS00605	1319	A	T	nonsynonymous
129005	NTHI477_RS00605	1318	G	C	nonsynonymous
129006	NTHI477_RS00605	1317	C	T	synonymous
129032	NTHI477_RS00605	1291	A	C	nonsynonymous
129045	NTHI477_RS00605	1278	T	G	synonymous
129051	NTHI477_RS00605	1272	T	C	synonymous
129114	NTHI477_RS00605	1209	T	C	synonymous
129141	NTHI477_RS00605	1182	T	C	synonymous
129159	NTHI477_RS00605	1164	G	A	synonymous
129162	NTHI477_RS00605	1161	T	G	synonymous
129165	NTHI477_RS00605	1158	C	T	synonymous
129171	NTHI477_RS00605	1152	T	A	synonymous
129175	NTHI477_RS00605	1148	T	C	nonsynonymous
129177	NTHI477_RS00605	1146	T	C	synonymous
129179	NTHI477_RS00605	1144	A	G	synonymous
129186	NTHI477_RS00605	1137	A	G	synonymous

129195	NTHI477_RS00605	1128	A	G	synonymous
129196	NTHI477_RS00605	1127	C	T	nonsynonymous
129201	NTHI477_RS00605	1122	C	T	synonymous
129210	NTHI477_RS00605	1113	C	T	synonymous
129224	NTHI477_RS00605	1099	C	T	nonsynonymous
129225	NTHI477_RS00605	1098	T	C	synonymous
129231	NTHI477_RS00605	1092	G	A	synonymous
129245	NTHI477_RS00605	1078	C	T	nonsynonymous
129264	NTHI477_RS00605	1059	C	T	synonymous
129270	NTHI477_RS00605	1053	A	G	synonymous
129279	NTHI477_RS00605	1044	G	A	synonymous
129289	NTHI477_RS00605	1034	G	T	nonsynonymous
129291	NTHI477_RS00605	1032	T	G	synonymous
129291	NTHI477_RS00605	1032	T	A	synonymous
129301	NTHI477_RS00605	1022	G	T	nonsynonymous
129308	NTHI477_RS00605	1015	C	T	nonsynonymous
129334	NTHI477_RS00605	989	C	T	nonsynonymous
129339	NTHI477_RS00605	984	C	A	synonymous
129345	NTHI477_RS00605	978	C	T	synonymous
129359	NTHI477_RS00605	964	C	T	nonsynonymous
129363	NTHI477_RS00605	960	G	A	synonymous
129367	NTHI477_RS00605	956	G	A	nonsynonymous
129387	NTHI477_RS00605	936	C	T	synonymous
129399	NTHI477_RS00605	924	G	A	synonymous
129411	NTHI477_RS00605	912	G	A	synonymous
129414	NTHI477_RS00605	909	T	C	synonymous
129417	NTHI477_RS00605	906	G	A	synonymous
129423	NTHI477_RS00605	900	G	A	synonymous
129426	NTHI477_RS00605	897	T	A	synonymous
129429	NTHI477_RS00605	894	G	A	synonymous
129430	NTHI477_RS00605	893	A	C	nonsynonymous
129432	NTHI477_RS00605	891	C	T	synonymous
129434	NTHI477_RS00605	889	C	A	nonsynonymous
129435	NTHI477_RS00605	888	A	T	nonsynonymous
129435	NTHI477_RS00605	888	A	G	synonymous
129441	NTHI477_RS00605	882	T	C	synonymous
129453	NTHI477_RS00605	870	G	A	synonymous
129456	NTHI477_RS00605	867	G	A	synonymous
129468	NTHI477_RS00605	855	A	G	synonymous
129468	NTHI477_RS00605	855	A	T	synonymous
129471	NTHI477_RS00605	852	T	C	synonymous
129476	NTHI477_RS00605	847	C	T	nonsynonymous
129478	NTHI477_RS00605	845	A	G	nonsynonymous
129486	NTHI477_RS00605	837	G	A	synonymous
129489	NTHI477_RS00605	834	T	A	synonymous

129501	NTHI477_RS00605	822	A	G	synonymous
129501	NTHI477_RS00605	822	A	T	synonymous
129506	NTHI477_RS00605	817	T	C	nonsynonymous
129513	NTHI477_RS00605	810	G	A	synonymous
129536	NTHI477_RS00605	787	T	G	nonsynonymous
129555	NTHI477_RS00605	768	A	C	synonymous
129571	NTHI477_RS00605	752	G	A	nonsynonymous
129573	NTHI477_RS00605	750	G	T	synonymous
129588	NTHI477_RS00605	735	T	G	nonsynonymous
129605	NTHI477_RS00605	718	G	A	synonymous
129606	NTHI477_RS00605	717	T	C	synonymous
129621	NTHI477_RS00605	702	C	T	synonymous
129624	NTHI477_RS00605	699	T	C	synonymous
129633	NTHI477_RS00605	690	A	G	synonymous
129645	NTHI477_RS00605	678	T	C	synonymous
129648	NTHI477_RS00605	675	T	G	synonymous
129654	NTHI477_RS00605	669	A	G	synonymous
129654	NTHI477_RS00605	669	A	C	nonsynonymous
129663	NTHI477_RS00605	660	T	A	synonymous
129666	NTHI477_RS00605	657	G	A	synonymous
129675	NTHI477_RS00605	648	C	T	synonymous
129684	NTHI477_RS00605	639	A	G	synonymous
129687	NTHI477_RS00605	636	C	T	synonymous
129705	NTHI477_RS00605	618	A	G	synonymous
129708	NTHI477_RS00605	615	G	A	synonymous
129716	NTHI477_RS00605	607	A	T	nonsynonymous
129717	NTHI477_RS00605	606	A	T	synonymous
129729	NTHI477_RS00605	594	T	C	synonymous
129744	NTHI477_RS00605	579	T	C	synonymous
129756	NTHI477_RS00605	567	A	T	nonsynonymous
129783	NTHI477_RS00605	540	G	A	synonymous
129786	NTHI477_RS00605	537	T	C	synonymous
129795	NTHI477_RS00605	528	G	A	synonymous
129796	NTHI477_RS00605	527	C	T	nonsynonymous
129815	NTHI477_RS00605	508	C	T	nonsynonymous
129817	NTHI477_RS00605	506	A	G	nonsynonymous
129866	NTHI477_RS00605	457	T	C	nonsynonymous
129870	NTHI477_RS00605	453	A	C	synonymous
129870	NTHI477_RS00605	453	A	G	synonymous
129872	NTHI477_RS00605	451	G	A	nonsynonymous
129888	NTHI477_RS00605	435	G	T	synonymous
129895	NTHI477_RS00605	428	T	C	nonsynonymous
129924	NTHI477_RS00605	399	C	T	synonymous
129940	NTHI477_RS00605	383	C	T	nonsynonymous
129945	NTHI477_RS00605	378	C	T	synonymous

129954	NTHI477_RS00605	369	A	G	synonymous
129969	NTHI477_RS00605	354	C	T	synonymous
129976	NTHI477_RS00605	347	T	A	nonsynonymous
129984	NTHI477_RS00605	339	G	A	synonymous
129993	NTHI477_RS00605	330	C	T	synonymous
129994	NTHI477_RS00605	329	G	A	nonsynonymous
130041	NTHI477_RS00605	282	T	A	synonymous
130071	NTHI477_RS00605	252	T	C	synonymous
130072	NTHI477_RS00605	251	T	A	nonsynonymous
130076	NTHI477_RS00605	247	G	A	nonsynonymous
130084	NTHI477_RS00605	239	C	T	nonsynonymous
130089	NTHI477_RS00605	234	T	C	synonymous
130094	NTHI477_RS00605	229	G	T	nonsynonymous
130101	NTHI477_RS00605	222	T	C	synonymous
130104	NTHI477_RS00605	219	G	A	synonymous
130131	NTHI477_RS00605	192	C	T	nonsynonymous
130132	NTHI477_RS00605	191	A	G	nonsynonymous
130137	NTHI477_RS00605	186	T	A	synonymous
130140	NTHI477_RS00605	183	G	A	synonymous
130143	NTHI477_RS00605	180	A	G	synonymous
130152	NTHI477_RS00605	171	G	A	synonymous
130155	NTHI477_RS00605	168	T	C	synonymous
130158	NTHI477_RS00605	165	T	G	nonsynonymous
130164	NTHI477_RS00605	159	G	T	synonymous
130165	NTHI477_RS00605	158	G	C	nonsynonymous
130193	NTHI477_RS00605	130	C	A	nonsynonymous
130212	NTHI477_RS00605	111	G	T	synonymous
130218	NTHI477_RS00605	105	T	G	synonymous
130221	NTHI477_RS00605	102	C	T	synonymous
130253	NTHI477_RS00605	70	T	C	nonsynonymous
130282	NTHI477_RS00605	41	T	C	nonsynonymous
130284	NTHI477_RS00605	39	G	A	synonymous
130299	NTHI477_RS00605	24	A	T	synonymous
130308	NTHI477_RS00605	15	G	C	synonymous
130331	intergenic	intergenic	C	T	intergenic
130367	intergenic	intergenic	G	A	intergenic
130420	intergenic	intergenic	G	A	intergenic
130432	intergenic	intergenic	G	A	intergenic
130432	intergenic	intergenic	G	T	intergenic
130434	intergenic	intergenic	T	C	intergenic
130480	NTHI477_RS00610	10	A	G	nonsynonymous
130553	NTHI477_RS00610	83	C	A	nonsynonymous
130590	NTHI477_RS00610	120	A	T	synonymous
130605	NTHI477_RS00610	135	T	C	synonymous
130645	NTHI477_RS00610	175	C	T	nonsynonymous

130722	NTHI477_RS00610	252	A	G	synonymous
130728	NTHI477_RS00610	258	A	T	synonymous
130767	NTHI477_RS00610	297	C	T	synonymous
130773	NTHI477_RS00610	303	G	A	synonymous
130779	NTHI477_RS00610	309	C	T	synonymous
130782	NTHI477_RS00610	312	A	G	synonymous
130791	NTHI477_RS00610	321	G	C	synonymous
130791	NTHI477_RS00610	321	G	T	synonymous
130803	NTHI477_RS00610	333	T	C	synonymous
130806	NTHI477_RS00610	336	A	G	synonymous
130827	NTHI477_RS00610	357	T	G	synonymous
130833	NTHI477_RS00610	363	C	T	synonymous
130837	NTHI477_RS00610	367	C	A	nonsynonymous
130839	NTHI477_RS00610	369	C	A	synonymous
130848	NTHI477_RS00610	378	A	G	synonymous
130866	NTHI477_RS00610	396	T	A	synonymous
130878	NTHI477_RS00610	408	C	A	synonymous
130896	NTHI477_RS00610	426	A	T	synonymous
130899	NTHI477_RS00610	429	T	C	synonymous
130908	NTHI477_RS00610	438	A	T	synonymous
130911	NTHI477_RS00610	441	A	T	synonymous
130923	NTHI477_RS00610	453	T	C	synonymous
130935	NTHI477_RS00610	465	T	C	synonymous
130941	NTHI477_RS00610	471	T	A	synonymous
130992	NTHI477_RS00610	522	A	G	synonymous
131017	NTHI477_RS00610	547	A	G	nonsynonymous
131019	NTHI477_RS00610	549	C	G	nonsynonymous
131028	NTHI477_RS00610	558	T	A	synonymous
131037	NTHI477_RS00610	567	A	G	synonymous
131043	NTHI477_RS00610	573	G	A	synonymous
131061	NTHI477_RS00610	591	T	A	synonymous
131067	NTHI477_RS00610	597	G	A	synonymous
131076	NTHI477_RS00610	606	A	G	synonymous
131085	NTHI477_RS00610	615	A	C	synonymous
131136	NTHI477_RS00610	666	C	T	synonymous
131142	NTHI477_RS00610	672	T	C	synonymous
131145	NTHI477_RS00610	675	T	C	synonymous
131148	NTHI477_RS00610	678	C	T	synonymous
131166	NTHI477_RS00610	696	T	C	synonymous
131184	NTHI477_RS00610	714	A	T	synonymous
131200	NTHI477_RS00610	730	C	T	nonsynonymous
131202	NTHI477_RS00610	732	T	C	synonymous
131226	NTHI477_RS00610	756	T	C	synonymous
131229	NTHI477_RS00610	759	C	T	synonymous
131232	NTHI477_RS00610	762	T	G	synonymous

131241	NTHI477_RS00610	771	A	C	synonymous
131244	NTHI477_RS00610	774	T	C	synonymous
131262	NTHI477_RS00610	792	T	C	synonymous
131289	NTHI477_RS00610	819	T	C	synonymous
131316	NTHI477_RS00610	846	A	G	synonymous
131331	NTHI477_RS00610	861	C	A	synonymous
131352	NTHI477_RS00610	882	C	T	synonymous
131355	NTHI477_RS00610	885	C	T	synonymous
131361	NTHI477_RS00610	891	C	T	synonymous
131364	NTHI477_RS00610	894	C	T	synonymous
131379	NTHI477_RS00610	909	A	T	synonymous
131385	NTHI477_RS00610	915	C	T	synonymous
131421	NTHI477_RS00610	951	T	C	synonymous
131424	NTHI477_RS00610	954	C	T	synonymous
131430	NTHI477_RS00610	960	C	T	synonymous
131460	NTHI477_RS00610	990	T	A	synonymous
131475	NTHI477_RS00610	1005	G	T	synonymous
131481	NTHI477_RS00610	1011	A	G	synonymous
131502	NTHI477_RS00610	1032	T	C	synonymous
131508	NTHI477_RS00610	1038	A	G	synonymous
131515	NTHI477_RS00610	1045	T	C	synonymous
131535	NTHI477_RS00610	1065	A	G	synonymous
131568	NTHI477_RS00610	1098	T	C	synonymous
131577	NTHI477_RS00610	1107	T	C	synonymous
131580	NTHI477_RS00610	1110	C	T	synonymous
131617	NTHI477_RS00610	1147	C	G	nonsynonymous
131634	NTHI477_RS00610	1164	C	T	synonymous
131695	intergenic	intergenic	T	G	intergenic
131696	intergenic	intergenic	C	A	intergenic
131714	intergenic	intergenic	C	T	intergenic
131746	intergenic	intergenic	T	G	intergenic
131765	NTHI477_RS00615	257	A	C	nonsynonymous
131800	NTHI477_RS00615	222	A	G	synonymous
131836	NTHI477_RS00615	186	G	A	synonymous
131863	NTHI477_RS00615	159	A	G	synonymous
131866	NTHI477_RS00615	156	A	G	synonymous
131869	NTHI477_RS00615	153	A	G	synonymous
131887	NTHI477_RS00615	135	T	C	synonymous
131890	NTHI477_RS00615	132	A	G	synonymous
131954	NTHI477_RS00615	68	T	C	nonsynonymous
131974	NTHI477_RS00615	48	T	A	synonymous
131977	NTHI477_RS00615	45	T	C	synonymous
131980	NTHI477_RS00615	42	A	G	synonymous
131995	NTHI477_RS00615	27	A	G	synonymous
132011	NTHI477_RS00615	11	A	G	nonsynonymous

132012	NTHI477_RS00615	10	C	T	nonsynonymous
132054	intergenic	intergenic	C	T	intergenic
132075	intergenic	intergenic	G	A	intergenic
132087	intergenic	intergenic	T	C	intergenic
132117	intergenic	intergenic	G	C	intergenic
132122	intergenic	intergenic	T	C	intergenic
132126	intergenic	intergenic	A	G	intergenic
132128	intergenic	intergenic	A	G	intergenic
132130	intergenic	intergenic	T	G	intergenic
132135	intergenic	intergenic	G	A	intergenic
132147	NTHI477_RS00620	10	T	C	synonymous
132161	NTHI477_RS00620	24	A	C	synonymous
132215	NTHI477_RS00620	78	G	A	synonymous
132216	NTHI477_RS00620	79	C	T	nonsynonymous
132244	NTHI477_RS00620	107	A	G	nonsynonymous
132245	NTHI477_RS00620	108	C	T	synonymous
132248	NTHI477_RS00620	111	T	C	synonymous
132249	NTHI477_RS00620	112	T	C	nonsynonymous
132251	NTHI477_RS00620	114	C	A	nonsynonymous
132263	NTHI477_RS00620	126	G	A	synonymous
132301	NTHI477_RS00620	164	A	C	nonsynonymous
132305	NTHI477_RS00620	168	T	A	synonymous
132327	NTHI477_RS00620	190	C	T	nonsynonymous
132328	NTHI477_RS00620	191	C	T	nonsynonymous
132341	NTHI477_RS00620	204	A	T	synonymous
132342	NTHI477_RS00620	205	G	A	nonsynonymous
132359	NTHI477_RS00620	222	C	T	synonymous
132365	NTHI477_RS00620	228	G	A	synonymous
132371	NTHI477_RS00620	234	G	A	synonymous
132383	NTHI477_RS00620	246	T	C	synonymous
132395	NTHI477_RS00620	258	G	A	synonymous
132410	NTHI477_RS00620	273	A	G	synonymous
132452	NTHI477_RS00620	315	A	G	synonymous
132506	NTHI477_RS00620	369	C	T	synonymous
132518	NTHI477_RS00620	381	T	A	nonsynonymous
132519	NTHI477_RS00620	382	A	G	nonsynonymous
132524	NTHI477_RS00620	387	C	T	synonymous
132525	NTHI477_RS00620	388	G	A	nonsynonymous
132533	NTHI477_RS00620	396	A	G	synonymous
132542	NTHI477_RS00620	405	T	G	nonsynonymous
132550	NTHI477_RS00620	413	T	C	nonsynonymous
132560	NTHI477_RS00620	423	C	T	synonymous
132626	NTHI477_RS00620	489	A	G	synonymous
132641	NTHI477_RS00620	504	G	A	synonymous
132644	NTHI477_RS00620	507	C	T	synonymous

132655	NTHI477_RS00620	518	C	T	nonsynonymous
132668	NTHI477_RS00620	531	C	T	synonymous
132681	NTHI477_RS00620	544	T	C	synonymous
132683	NTHI477_RS00620	546	G	A	synonymous
132686	NTHI477_RS00620	549	A	G	synonymous
132695	NTHI477_RS00620	558	T	C	synonymous
132698	NTHI477_RS00620	561	C	T	synonymous
132737	NTHI477_RS00620	600	G	A	nonsynonymous
132737	NTHI477_RS00620	600	G	T	nonsynonymous
132749	NTHI477_RS00620	612	G	T	synonymous
132764	NTHI477_RS00620	627	G	A	synonymous
132765	NTHI477_RS00620	628	A	C	synonymous
132770	NTHI477_RS00620	633	T	C	synonymous
132770	NTHI477_RS00620	633	T	A	synonymous
132773	NTHI477_RS00620	636	G	A	synonymous
132777	NTHI477_RS00620	640	A	G	nonsynonymous
132782	NTHI477_RS00620	645	T	C	synonymous
132791	NTHI477_RS00620	654	G	A	synonymous
132800	NTHI477_RS00620	663	C	T	synonymous
132809	NTHI477_RS00620	672	C	T	synonymous
132818	NTHI477_RS00620	681	C	T	synonymous
132829	NTHI477_RS00620	692	C	T	nonsynonymous
132830	NTHI477_RS00620	693	C	A	synonymous
132842	NTHI477_RS00620	705	A	C	nonsynonymous
132845	NTHI477_RS00620	708	A	G	synonymous
132848	NTHI477_RS00620	711	G	A	synonymous
132857	NTHI477_RS00620	720	C	T	synonymous
132861	NTHI477_RS00620	724	C	A	nonsynonymous
132862	NTHI477_RS00620	725	A	G	nonsynonymous
132866	NTHI477_RS00620	729	A	T	synonymous
132875	NTHI477_RS00620	738	T	C	synonymous
132890	NTHI477_RS00620	753	A	G	synonymous
132896	NTHI477_RS00620	759	C	T	synonymous
132910	NTHI477_RS00620	773	G	T	nonsynonymous
132941	NTHI477_RS00620	804	G	A	synonymous
132953	NTHI477_RS00620	816	A	T	synonymous
133039	NTHI477_RS00620	902	C	T	nonsynonymous
133055	intergenic	intergenic	C	T	intergenic
133063	intergenic	intergenic	C	T	intergenic
133091	NTHI477_RS00625	1209	A	G	synonymous
133094	NTHI477_RS00625	1206	C	T	nonsynonymous
133157	NTHI477_RS00625	1143	A	T	synonymous
133190	NTHI477_RS00625	1110	C	T	synonymous
133196	NTHI477_RS00625	1104	C	T	synonymous
133211	NTHI477_RS00625	1089	G	A	synonymous

133213	NTHI477_RS00625	1087	C	T	nonsynonymous
133226	NTHI477_RS00625	1074	T	A	synonymous
133247	NTHI477_RS00625	1053	G	A	synonymous
133274	NTHI477_RS00625	1026	A	G	synonymous
133325	NTHI477_RS00625	975	A	G	synonymous
133503	NTHI477_RS00625	797	G	C	nonsynonymous
133517	NTHI477_RS00625	783	A	G	synonymous
133583	NTHI477_RS00625	717	T	C	synonymous
133585	NTHI477_RS00625	715	C	T	nonsynonymous
133686	NTHI477_RS00625	614	A	G	nonsynonymous
133852	NTHI477_RS00625	448	T	C	nonsynonymous
133919	NTHI477_RS00625	381	G	A	synonymous
133952	NTHI477_RS00625	348	A	G	synonymous
133979	NTHI477_RS00625	321	G	T	nonsynonymous
134005	NTHI477_RS00625	295	G	A	synonymous
134022	NTHI477_RS00625	278	T	C	nonsynonymous
134027	NTHI477_RS00625	273	G	A	synonymous
134051	NTHI477_RS00625	249	G	A	synonymous
134111	NTHI477_RS00625	189	G	A	synonymous
134174	NTHI477_RS00625	126	A	G	synonymous
134316	intergenic	intergenic	T	A	intergenic
134345	NTHI477_RS00630	2240	G	A	nonsynonymous
134352	NTHI477_RS00630	2233	C	T	nonsynonymous
134418	NTHI477_RS00630	2167	C	T	nonsynonymous
134419	NTHI477_RS00630	2166	A	G	synonymous
134472	NTHI477_RS00630	2113	A	G	nonsynonymous
134560	NTHI477_RS00630	2025	T	C	nonsynonymous
134599	NTHI477_RS00630	1986	C	T	synonymous
134613	NTHI477_RS00630	1972	T	G	nonsynonymous
134724	NTHI477_RS00630	1861	C	T	nonsynonymous
134770	NTHI477_RS00630	1815	T	C	synonymous
134773	NTHI477_RS00630	1812	C	T	synonymous
134826	NTHI477_RS00630	1759	T	C	nonsynonymous
134872	NTHI477_RS00630	1713	G	A	synonymous
134896	NTHI477_RS00630	1689	T	G	synonymous
134901	NTHI477_RS00630	1684	G	T	nonsynonymous
134902	NTHI477_RS00630	1683	C	T	synonymous
134920	NTHI477_RS00630	1665	C	T	synonymous
134926	NTHI477_RS00630	1659	C	T	synonymous
134929	NTHI477_RS00630	1656	G	A	synonymous
134989	NTHI477_RS00630	1596	G	A	synonymous
135034	NTHI477_RS00630	1551	T	A	synonymous
135058	NTHI477_RS00630	1527	T	A	synonymous
135079	NTHI477_RS00630	1506	T	C	synonymous
135088	NTHI477_RS00630	1497	C	T	synonymous

135089	NTHI477_RS00630	1496	G	A	nonsynonymous
135098	NTHI477_RS00630	1487	C	T	nonsynonymous
135150	NTHI477_RS00630	1435	G	A	nonsynonymous
135152	NTHI477_RS00630	1433	C	T	nonsynonymous
135153	NTHI477_RS00630	1432	T	A	nonsynonymous
135205	NTHI477_RS00630	1380	G	A	synonymous
135208	NTHI477_RS00630	1377	A	G	synonymous
135268	NTHI477_RS00630	1317	A	T	synonymous
135359	NTHI477_RS00630	1226	G	A	nonsynonymous
135364	NTHI477_RS00630	1221	A	C	synonymous
135388	NTHI477_RS00630	1197	G	T	nonsynonymous
135391	NTHI477_RS00630	1194	G	A	synonymous
135466	NTHI477_RS00630	1119	T	C	synonymous
135477	NTHI477_RS00630	1108	A	G	synonymous
135507	NTHI477_RS00630	1078	T	C	nonsynonymous
135520	NTHI477_RS00630	1065	G	A	synonymous
135559	NTHI477_RS00630	1026	T	A	synonymous
135619	NTHI477_RS00630	966	G	T	synonymous
135622	NTHI477_RS00630	963	C	A	synonymous
135625	NTHI477_RS00630	960	G	A	synonymous
135636	NTHI477_RS00630	949	C	T	nonsynonymous
135658	NTHI477_RS00630	927	G	T	synonymous
135673	NTHI477_RS00630	912	A	G	synonymous
135679	NTHI477_RS00630	906	T	C	synonymous
135685	NTHI477_RS00630	900	T	G	nonsynonymous
135687	NTHI477_RS00630	898	T	G	synonymous
135694	NTHI477_RS00630	891	G	A	synonymous
135736	NTHI477_RS00630	849	T	C	synonymous
135745	NTHI477_RS00630	840	T	G	synonymous
135748	NTHI477_RS00630	837	C	T	synonymous
135757	NTHI477_RS00630	828	A	T	nonsynonymous
135775	NTHI477_RS00630	810	A	G	synonymous
135781	NTHI477_RS00630	804	G	T	synonymous
135784	NTHI477_RS00630	801	T	G	synonymous
135787	NTHI477_RS00630	798	C	A	synonymous
135805	NTHI477_RS00630	780	C	T	synonymous
135808	NTHI477_RS00630	777	A	T	synonymous
135811	NTHI477_RS00630	774	A	G	synonymous
135817	NTHI477_RS00630	768	G	A	synonymous
135820	NTHI477_RS00630	765	C	T	synonymous
135823	NTHI477_RS00630	762	G	A	synonymous
135842	NTHI477_RS00630	743	G	A	nonsynonymous
135868	NTHI477_RS00630	717	G	A	synonymous
135898	NTHI477_RS00630	687	C	T	synonymous
135916	NTHI477_RS00630	669	C	A	synonymous

135934	NTHI477_RS00630	651	T	G	synonymous
135957	NTHI477_RS00630	628	C	T	nonsynonymous
135968	NTHI477_RS00630	617	C	T	nonsynonymous
135969	NTHI477_RS00630	616	T	C	nonsynonymous
135976	NTHI477_RS00630	609	T	A	synonymous
135987	NTHI477_RS00630	598	G	A	synonymous
136000	NTHI477_RS00630	585	C	T	synonymous
136042	NTHI477_RS00630	543	G	A	synonymous
136042	NTHI477_RS00630	543	G	C	synonymous
136054	NTHI477_RS00630	531	T	C	synonymous
136057	NTHI477_RS00630	528	G	A	synonymous
136075	NTHI477_RS00630	510	C	T	synonymous
136087	NTHI477_RS00630	498	C	A	synonymous
136099	NTHI477_RS00630	486	A	G	synonymous
136117	NTHI477_RS00630	468	A	C	synonymous
136117	NTHI477_RS00630	468	A	G	synonymous
136120	NTHI477_RS00630	465	C	T	synonymous
136153	NTHI477_RS00630	432	T	A	synonymous
136172	NTHI477_RS00630	413	T	C	nonsynonymous
136174	NTHI477_RS00630	411	C	T	synonymous
136176	NTHI477_RS00630	409	A	G	synonymous
136201	NTHI477_RS00630	384	G	A	synonymous
136225	NTHI477_RS00630	360	A	T	synonymous
136249	NTHI477_RS00630	336	T	C	synonymous
136279	NTHI477_RS00630	306	T	A	synonymous
136279	NTHI477_RS00630	306	T	C	synonymous
136291	NTHI477_RS00630	294	A	G	synonymous
136318	NTHI477_RS00630	267	A	C	synonymous
136333	NTHI477_RS00630	252	A	G	synonymous
136351	NTHI477_RS00630	234	G	A	synonymous
136360	NTHI477_RS00630	225	G	A	synonymous
136381	NTHI477_RS00630	204	A	G	synonymous
136429	NTHI477_RS00630	156	C	T	synonymous
136444	NTHI477_RS00630	141	A	G	synonymous
136465	NTHI477_RS00630	120	A	G	synonymous
136507	NTHI477_RS00630	78	A	G	synonymous
136531	NTHI477_RS00630	54	A	G	synonymous
136537	NTHI477_RS00630	48	G	A	synonymous
136540	NTHI477_RS00630	45	G	A	synonymous
136628	intergenic	intergenic	A	G	intergenic
136747	NTHI477_RS00635	1803	T	A	synonymous
136752	NTHI477_RS00635	1798	T	C	nonsynonymous
136864	NTHI477_RS00635	1686	G	A	synonymous
136876	NTHI477_RS00635	1674	C	T	synonymous
136882	NTHI477_RS00635	1668	G	A	synonymous

136891	NTHI477_RS00635	1659	G	A	synonymous
136900	NTHI477_RS00635	1650	C	G	synonymous
136925	NTHI477_RS00635	1625	T	C	nonsynonymous
136930	NTHI477_RS00635	1620	A	G	synonymous
136966	NTHI477_RS00635	1584	G	T	synonymous
136987	NTHI477_RS00635	1563	T	G	synonymous
137037	NTHI477_RS00635	1513	G	T	nonsynonymous
137059	NTHI477_RS00635	1491	A	G	synonymous
137062	NTHI477_RS00635	1488	C	A	synonymous
137101	NTHI477_RS00635	1449	A	G	synonymous
137131	NTHI477_RS00635	1419	A	G	synonymous
137140	NTHI477_RS00635	1410	G	A	synonymous
137154	NTHI477_RS00635	1396	C	T	nonsynonymous
137173	NTHI477_RS00635	1377	G	T	synonymous
137218	NTHI477_RS00635	1332	A	G	synonymous
137233	NTHI477_RS00635	1317	C	T	synonymous
137263	NTHI477_RS00635	1287	C	T	synonymous
137278	NTHI477_RS00635	1272	A	G	synonymous
137284	NTHI477_RS00635	1266	C	A	synonymous
137332	NTHI477_RS00635	1218	T	C	synonymous
137365	NTHI477_RS00635	1185	A	T	synonymous
137401	NTHI477_RS00635	1149	T	G	synonymous
137404	NTHI477_RS00635	1146	T	G	synonymous
137407	NTHI477_RS00635	1143	A	G	synonymous
137434	NTHI477_RS00635	1116	G	A	synonymous
137445	NTHI477_RS00635	1105	C	T	nonsynonymous
137446	NTHI477_RS00635	1104	T	C	synonymous
137458	NTHI477_RS00635	1092	A	G	synonymous
137467	NTHI477_RS00635	1083	A	G	synonymous
137473	NTHI477_RS00635	1077	A	G	synonymous
137476	NTHI477_RS00635	1074	T	G	synonymous
137512	NTHI477_RS00635	1038	A	G	synonymous
137569	NTHI477_RS00635	981	C	T	synonymous
137605	NTHI477_RS00635	945	G	A	synonymous
137641	NTHI477_RS00635	909	G	A	synonymous
137674	NTHI477_RS00635	876	A	T	synonymous
137680	NTHI477_RS00635	870	G	A	synonymous
137698	NTHI477_RS00635	852	G	A	synonymous
137704	NTHI477_RS00635	846	G	A	synonymous
137731	NTHI477_RS00635	819	T	A	synonymous
137734	NTHI477_RS00635	816	T	C	synonymous
137749	NTHI477_RS00635	801	G	A	synonymous
137761	NTHI477_RS00635	789	A	G	synonymous
137764	NTHI477_RS00635	786	C	T	synonymous
137770	NTHI477_RS00635	780	A	G	synonymous

137817	NTHI477_RS00635	733	T	C	nonsynonymous
137847	NTHI477_RS00635	703	C	T	nonsynonymous
137848	NTHI477_RS00635	702	T	G	synonymous
137848	NTHI477_RS00635	702	T	C	synonymous
137854	NTHI477_RS00635	696	C	G	synonymous
137896	NTHI477_RS00635	654	C	A	synonymous
137917	NTHI477_RS00635	633	A	G	synonymous
137965	NTHI477_RS00635	585	G	A	synonymous
137971	NTHI477_RS00635	579	T	C	synonymous
137971	NTHI477_RS00635	579	T	G	synonymous
137974	NTHI477_RS00635	576	C	T	synonymous
137975	NTHI477_RS00635	575	G	C	nonsynonymous
138023	NTHI477_RS00635	527	C	T	nonsynonymous
138043	NTHI477_RS00635	507	C	T	synonymous
138058	NTHI477_RS00635	492	A	G	synonymous
138059	NTHI477_RS00635	491	G	A	nonsynonymous
138067	NTHI477_RS00635	483	A	C	synonymous
138079	NTHI477_RS00635	471	G	A	synonymous
138088	NTHI477_RS00635	462	C	T	synonymous
138096	NTHI477_RS00635	454	C	T	nonsynonymous
138100	NTHI477_RS00635	450	T	C	synonymous
138142	NTHI477_RS00635	408	C	G	synonymous
138144	NTHI477_RS00635	406	C	G	nonsynonymous
138144	NTHI477_RS00635	406	C	T	nonsynonymous
138148	NTHI477_RS00635	402	A	G	synonymous
138148	NTHI477_RS00635	402	A	C	synonymous
138160	NTHI477_RS00635	390	A	G	synonymous
138187	NTHI477_RS00635	363	C	T	synonymous
138193	NTHI477_RS00635	357	C	T	synonymous
138211	NTHI477_RS00635	339	A	T	synonymous
138223	NTHI477_RS00635	327	T	A	synonymous
138226	NTHI477_RS00635	324	C	T	synonymous
138280	NTHI477_RS00635	270	G	A	synonymous
138283	NTHI477_RS00635	267	T	C	synonymous
138286	NTHI477_RS00635	264	T	C	synonymous
138317	NTHI477_RS00635	233	A	G	nonsynonymous
138343	NTHI477_RS00635	207	G	A	synonymous
138346	NTHI477_RS00635	204	T	C	synonymous
138349	NTHI477_RS00635	201	G	T	synonymous
138354	NTHI477_RS00635	196	C	T	nonsynonymous
138358	NTHI477_RS00635	192	G	A	synonymous
138367	NTHI477_RS00635	183	T	A	synonymous
138370	NTHI477_RS00635	180	G	A	synonymous
138373	NTHI477_RS00635	177	T	C	synonymous
138379	NTHI477_RS00635	171	T	G	synonymous

138393	NTHI477_RS00635	157	T	A	nonsynonymous
138403	NTHI477_RS00635	147	C	G	synonymous
138418	NTHI477_RS00635	132	T	C	synonymous
138421	NTHI477_RS00635	129	G	A	synonymous
138499	NTHI477_RS00635	51	T	C	synonymous
138553	intergenic	intergenic	G	T	intergenic
138556	intergenic	intergenic	A	G	intergenic
138558	intergenic	intergenic	T	G	intergenic
138560	intergenic	intergenic	T	G	intergenic
138580	intergenic	intergenic	G	A	intergenic
138644	NTHI477_RS00640	915	A	C	nonsynonymous
138653	NTHI477_RS00640	906	T	G	synonymous
138656	NTHI477_RS00640	903	A	G	synonymous
138659	NTHI477_RS00640	900	T	C	synonymous
138671	NTHI477_RS00640	888	G	A	synonymous
138690	NTHI477_RS00640	869	T	A	nonsynonymous
138691	NTHI477_RS00640	868	G	T	nonsynonymous
138692	NTHI477_RS00640	867	T	C	synonymous
138694	NTHI477_RS00640	865	A	T	nonsynonymous
138698	NTHI477_RS00640	861	C	G	synonymous
138699	NTHI477_RS00640	860	C	T	nonsynonymous
138707	NTHI477_RS00640	852	G	T	synonymous
138719	NTHI477_RS00640	840	A	T	synonymous
138739	NTHI477_RS00640	820	C	T	nonsynonymous
138796	NTHI477_RS00640	763	C	T	nonsynonymous
138811	NTHI477_RS00640	748	T	C	nonsynonymous
138830	NTHI477_RS00640	729	T	C	synonymous
138848	NTHI477_RS00640	711	G	A	synonymous
138855	NTHI477_RS00640	704	T	G	nonsynonymous
138857	NTHI477_RS00640	702	T	C	synonymous
138860	NTHI477_RS00640	699	A	G	synonymous
138887	NTHI477_RS00640	672	G	A	synonymous
138890	NTHI477_RS00640	669	T	A	synonymous
138898	NTHI477_RS00640	661	C	T	nonsynonymous
138899	NTHI477_RS00640	660	A	G	synonymous
138902	NTHI477_RS00640	657	G	A	synonymous
138903	NTHI477_RS00640	656	A	G	nonsynonymous
138905	NTHI477_RS00640	654	C	T	synonymous
138908	NTHI477_RS00640	651	C	T	synonymous
138917	NTHI477_RS00640	642	T	G	synonymous
138917	NTHI477_RS00640	642	T	A	synonymous
138920	NTHI477_RS00640	639	T	A	synonymous
138921	NTHI477_RS00640	638	G	A	nonsynonymous
138922	NTHI477_RS00640	637	C	T	nonsynonymous
138959	NTHI477_RS00640	600	T	C	synonymous

139037	NTHI477_RS00640	522	A	G	synonymous
139049	NTHI477_RS00640	510	A	G	synonymous
139055	NTHI477_RS00640	504	T	C	synonymous
139091	NTHI477_RS00640	468	A	G	synonymous
139094	NTHI477_RS00640	465	A	G	synonymous
139100	NTHI477_RS00640	459	G	A	synonymous
139100	NTHI477_RS00640	459	G	T	synonymous
139106	NTHI477_RS00640	453	G	A	synonymous
139109	NTHI477_RS00640	450	A	G	synonymous
139118	NTHI477_RS00640	441	C	T	synonymous
139126	NTHI477_RS00640	433	G	T	nonsynonymous
139127	NTHI477_RS00640	432	A	G	synonymous
139130	NTHI477_RS00640	429	G	A	synonymous
139133	NTHI477_RS00640	426	C	T	synonymous
139133	NTHI477_RS00640	426	C	A	synonymous
139136	NTHI477_RS00640	423	G	A	synonymous
139148	NTHI477_RS00640	411	C	T	synonymous
139166	NTHI477_RS00640	393	A	G	synonymous
139174	NTHI477_RS00640	385	C	T	nonsynonymous
139178	NTHI477_RS00640	381	T	G	synonymous
139198	NTHI477_RS00640	361	T	C	nonsynonymous
139213	NTHI477_RS00640	346	C	T	nonsynonymous
139214	NTHI477_RS00640	345	T	C	synonymous
139223	NTHI477_RS00640	336	T	C	synonymous
139247	NTHI477_RS00640	312	C	T	synonymous
139265	NTHI477_RS00640	294	A	G	synonymous
139268	NTHI477_RS00640	291	T	A	synonymous
139291	NTHI477_RS00640	268	C	T	nonsynonymous
139292	NTHI477_RS00640	267	T	A	synonymous
139295	NTHI477_RS00640	264	G	A	synonymous
139298	NTHI477_RS00640	261	G	A	synonymous
139316	NTHI477_RS00640	243	C	A	synonymous
139318	NTHI477_RS00640	241	C	T	nonsynonymous
139319	NTHI477_RS00640	240	C	T	synonymous
139325	NTHI477_RS00640	234	G	C	nonsynonymous
139325	NTHI477_RS00640	234	G	T	nonsynonymous
139327	NTHI477_RS00640	232	C	T	nonsynonymous
139328	NTHI477_RS00640	231	G	A	synonymous
139331	NTHI477_RS00640	228	T	C	synonymous
139334	NTHI477_RS00640	225	A	T	synonymous
139336	NTHI477_RS00640	223	G	A	nonsynonymous
139340	NTHI477_RS00640	219	A	G	synonymous
139342	NTHI477_RS00640	217	C	T	nonsynonymous
139343	NTHI477_RS00640	216	A	T	synonymous
139346	NTHI477_RS00640	213	G	A	synonymous

139352	NTHI477_RS00640	207	A	T	synonymous
139364	NTHI477_RS00640	195	A	G	synonymous
139367	NTHI477_RS00640	192	G	A	synonymous
139376	NTHI477_RS00640	183	G	A	synonymous
139379	NTHI477_RS00640	180	A	T	synonymous
139412	NTHI477_RS00640	147	C	T	synonymous
139423	NTHI477_RS00640	136	G	A	nonsynonymous
139425	NTHI477_RS00640	134	G	T	nonsynonymous
139425	NTHI477_RS00640	134	G	C	nonsynonymous
139429	NTHI477_RS00640	130	T	G	nonsynonymous
139441	NTHI477_RS00640	118	G	T	nonsynonymous
139487	NTHI477_RS00640	72	G	A	synonymous
139492	NTHI477_RS00640	67	G	A	synonymous
139549	NTHI477_RS00640	10	C	T	nonsynonymous
139617	intergenic	intergenic	C	T	intergenic
139708	NTHI477_RS00645	54	C	T	synonymous
139721	NTHI477_RS00645	67	C	T	nonsynonymous
139735	NTHI477_RS00645	81	C	T	synonymous
139736	NTHI477_RS00645	82	A	G	nonsynonymous
139744	NTHI477_RS00645	90	T	C	synonymous
139762	NTHI477_RS00645	108	A	T	synonymous
139768	NTHI477_RS00645	114	G	T	synonymous
139774	NTHI477_RS00645	120	G	A	synonymous
139780	NTHI477_RS00645	126	T	C	synonymous
139801	NTHI477_RS00645	147	C	T	synonymous
139802	NTHI477_RS00645	148	C	G	nonsynonymous
139804	NTHI477_RS00645	150	A	G	synonymous
139807	NTHI477_RS00645	153	T	C	synonymous
139810	NTHI477_RS00645	156	A	T	synonymous
139810	NTHI477_RS00645	156	A	G	synonymous
139813	NTHI477_RS00645	159	T	A	synonymous
139822	NTHI477_RS00645	168	G	A	synonymous
139837	NTHI477_RS00645	183	T	G	synonymous
139843	NTHI477_RS00645	189	T	C	synonymous
139850	NTHI477_RS00645	196	C	T	nonsynonymous
139852	NTHI477_RS00645	198	C	T	synonymous
139870	NTHI477_RS00645	216	A	G	synonymous
139885	NTHI477_RS00645	231	A	G	synonymous
139888	NTHI477_RS00645	234	T	A	synonymous
139905	NTHI477_RS00645	251	T	A	nonsynonymous
139906	NTHI477_RS00645	252	C	T	synonymous
139924	NTHI477_RS00645	270	A	G	synonymous
139955	NTHI477_RS00645	301	C	T	nonsynonymous
139963	NTHI477_RS00645	309	T	C	synonymous
139964	NTHI477_RS00645	310	A	G	nonsynonymous

139993	NTHI477_RS00645	339	C	T	synonymous
140000	NTHI477_RS00645	346	T	C	synonymous
140002	NTHI477_RS00645	348	A	G	synonymous
140005	NTHI477_RS00645	351	T	C	synonymous
140110	NTHI477_RS00645	456	T	C	synonymous
140113	NTHI477_RS00645	459	T	C	synonymous
140158	NTHI477_RS00645	504	C	T	synonymous
140164	NTHI477_RS00645	510	G	A	synonymous
140176	NTHI477_RS00645	522	G	A	synonymous
140179	NTHI477_RS00645	525	G	A	synonymous
140209	NTHI477_RS00645	555	T	C	synonymous
140218	NTHI477_RS00645	564	G	T	synonymous
140221	NTHI477_RS00645	567	T	G	synonymous
140236	NTHI477_RS00645	582	C	A	synonymous
140257	NTHI477_RS00645	603	T	C	synonymous
140263	NTHI477_RS00645	609	G	A	synonymous
140266	NTHI477_RS00645	612	T	C	synonymous
140272	NTHI477_RS00645	618	T	C	synonymous
140278	NTHI477_RS00645	624	A	G	synonymous
140287	NTHI477_RS00645	633	G	A	synonymous
140294	NTHI477_RS00645	640	G	A	nonsynonymous
140299	NTHI477_RS00645	645	A	G	synonymous
140318	NTHI477_RS00645	664	G	A	nonsynonymous
140335	NTHI477_RS00645	681	A	G	synonymous
140344	NTHI477_RS00645	690	A	G	synonymous
140389	NTHI477_RS00645	735	A	G	synonymous
140440	NTHI477_RS00645	786	C	T	synonymous
140458	NTHI477_RS00645	804	C	T	synonymous
140470	NTHI477_RS00645	816	A	C	synonymous
140471	NTHI477_RS00645	817	G	A	nonsynonymous
140476	NTHI477_RS00645	822	A	G	synonymous
140494	NTHI477_RS00645	840	C	T	synonymous
140497	NTHI477_RS00645	843	A	G	synonymous
140509	NTHI477_RS00645	855	C	T	synonymous
140510	NTHI477_RS00645	856	C	T	synonymous
140521	NTHI477_RS00645	867	T	A	synonymous
140527	NTHI477_RS00645	873	A	T	synonymous
140530	NTHI477_RS00645	876	A	G	synonymous
140533	NTHI477_RS00645	879	T	C	synonymous
140542	NTHI477_RS00645	888	T	G	synonymous
140551	NTHI477_RS00645	897	G	C	synonymous
140551	NTHI477_RS00645	897	G	T	synonymous
140569	NTHI477_RS00645	915	C	T	synonymous
140587	NTHI477_RS00645	933	G	T	synonymous
140599	NTHI477_RS00645	945	C	T	synonymous

140605	NTHI477_RS00645	951	C	A	nonsynonymous
140608	NTHI477_RS00645	954	T	G	synonymous
140617	NTHI477_RS00645	963	C	T	synonymous
140626	NTHI477_RS00645	972	C	T	synonymous
140632	NTHI477_RS00645	978	A	T	synonymous
140664	NTHI477_RS00645	1010	T	C	nonsynonymous
140665	NTHI477_RS00645	1011	G	A	synonymous
140668	NTHI477_RS00645	1014	T	C	synonymous
140701	NTHI477_RS00645	1047	C	T	synonymous
140716	NTHI477_RS00645	1062	A	C	synonymous
140719	NTHI477_RS00645	1065	C	G	synonymous
140723	NTHI477_RS00645	1069	G	A	nonsynonymous
140746	NTHI477_RS00645	1092	T	C	synonymous
140746	NTHI477_RS00645	1092	T	A	synonymous
140752	NTHI477_RS00645	1098	A	G	synonymous
140762	NTHI477_RS00645	1108	T	C	synonymous
140786	NTHI477_RS00645	1132	G	T	nonsynonymous
140809	NTHI477_RS00645	1155	C	T	synonymous
140812	NTHI477_RS00645	1158	A	T	nonsynonymous
140818	NTHI477_RS00645	1164	T	C	synonymous
140827	NTHI477_RS00645	1173	T	C	synonymous
140830	NTHI477_RS00645	1176	T	C	synonymous
140838	NTHI477_RS00645	1184	A	C	nonsynonymous
140839	NTHI477_RS00645	1185	C	T	synonymous
140842	NTHI477_RS00645	1188	T	A	synonymous
140852	NTHI477_RS00645	1198	T	C	synonymous
140869	NTHI477_RS00645	1215	C	A	synonymous
140872	NTHI477_RS00645	1218	G	A	synonymous
140884	NTHI477_RS00645	1230	G	A	synonymous
140887	NTHI477_RS00645	1233	G	T	synonymous
140893	NTHI477_RS00645	1239	C	T	synonymous
140899	NTHI477_RS00645	1245	C	T	synonymous
140908	NTHI477_RS00645	1254	G	A	synonymous
140950	NTHI477_RS00645	1296	T	C	synonymous
140953	NTHI477_RS00645	1299	A	C	synonymous
141004	NTHI477_RS00645	1350	T	C	synonymous
141007	NTHI477_RS00645	1353	C	T	synonymous
141019	NTHI477_RS00645	1365	G	A	synonymous
141052	NTHI477_RS00645	1398	C	T	synonymous
141071	NTHI477_RS00645	1417	T	G	nonsynonymous
141076	NTHI477_RS00645	1422	G	A	synonymous
141096	intergenic	intergenic	G	A	intergenic
141100	intergenic	intergenic	G	A	intergenic
141102	intergenic	intergenic	A	T	intergenic
141105	intergenic	intergenic	T	C	intergenic

141145	intergenic	intergenic	G	A	intergenic
141153	intergenic	intergenic	T	C	intergenic
141173	intergenic	intergenic	C	T	intergenic
141188	intergenic	intergenic	G	T	intergenic
141227	intergenic	intergenic	A	G	intergenic
141288	NTHI477_RS09100	11	C	T	nonsynonymous
141345	NTHI477_RS09100	68	A	G	nonsynonymous
141378	NTHI477_RS09100	101	C	T	nonsynonymous
141395	NTHI477_RS09100	118	T	C	nonsynonymous
141412	NTHI477_RS09100	135	G	A	synonymous
141464	intergenic	intergenic	G	A	intergenic
141473	intergenic	intergenic	G	A	intergenic
141489	intergenic	intergenic	C	T	intergenic
141512	intergenic	intergenic	A	G	intergenic
141515	intergenic	intergenic	G	A	intergenic
141518	intergenic	intergenic	T	G	intergenic
141527	intergenic	intergenic	A	G	intergenic
141544	intergenic	intergenic	T	A	intergenic
141548	intergenic	intergenic	G	A	intergenic
141551	intergenic	intergenic	T	C	intergenic
141553	intergenic	intergenic	C	A	intergenic
141562	intergenic	intergenic	G	T	intergenic
141573	intergenic	intergenic	C	A	intergenic
141581	intergenic	intergenic	A	T	intergenic
141591	intergenic	intergenic	A	G	intergenic
141593	intergenic	intergenic	A	T	intergenic
141609	intergenic	intergenic	C	A	intergenic
141643	intergenic	intergenic	A	G	intergenic
141654	intergenic	intergenic	G	A	intergenic
141675	intergenic	intergenic	T	C	intergenic
141679	intergenic	intergenic	C	A	intergenic
141681	intergenic	intergenic	T	C	intergenic
141684	intergenic	intergenic	C	T	intergenic
141685	intergenic	intergenic	A	G	intergenic
141690	intergenic	intergenic	G	A	intergenic
141714	intergenic	intergenic	T	A	intergenic
141722	intergenic	intergenic	A	G	intergenic
141731	intergenic	intergenic	T	G	intergenic
141741	intergenic	intergenic	G	A	intergenic
141774	NTHI477_RS00650	339	C	G	synonymous
141777	NTHI477_RS00650	336	T	C	synonymous
141808	NTHI477_RS00650	305	A	C	nonsynonymous
141821	NTHI477_RS00650	292	T	C	nonsynonymous
141837	NTHI477_RS00650	276	A	C	nonsynonymous
141844	NTHI477_RS00650	269	G	A	nonsynonymous

141899	NTHI477_RS00650	214	A	C	nonsynonymous
141903	NTHI477_RS00650	210	G	A	synonymous
141904	NTHI477_RS00650	209	T	G	nonsynonymous
141914	NTHI477_RS00650	199	G	C	nonsynonymous
141915	NTHI477_RS00650	198	A	T	nonsynonymous
141917	NTHI477_RS00650	196	T	A	nonsynonymous
141920	NTHI477_RS00650	193	C	G	nonsynonymous
141928	NTHI477_RS00650	185	T	C	nonsynonymous
141933	NTHI477_RS00650	180	C	T	synonymous
141935	NTHI477_RS00650	178	T	G	synonymous
141942	NTHI477_RS00650	171	T	A	nonsynonymous
141943	NTHI477_RS00650	170	C	T	nonsynonymous
141945	NTHI477_RS00650	168	A	T	synonymous
141945	NTHI477_RS00650	168	A	C	synonymous
141948	NTHI477_RS00650	165	G	A	synonymous
141954	NTHI477_RS00650	159	A	G	synonymous
141960	NTHI477_RS00650	153	A	C	synonymous
142030	NTHI477_RS00650	83	C	T	nonsynonymous
142037	NTHI477_RS00650	76	T	C	nonsynonymous
142041	NTHI477_RS00650	72	T	C	synonymous
142044	NTHI477_RS00650	69	G	A	synonymous
142049	NTHI477_RS00650	64	C	T	nonsynonymous
142050	NTHI477_RS00650	63	T	A	synonymous
142081	NTHI477_RS00650	32	C	T	nonsynonymous
142086	NTHI477_RS00650	27	G	A	synonymous
142103	NTHI477_RS00650	10	T	C	nonsynonymous
142104	NTHI477_RS00650	9	A	G	synonymous
142106	NTHI477_RS00650	7	A	G	nonsynonymous
142110	NTHI477_RS00650	3	C	A	nonsynonymous
142112	NTHI477_RS00650	1	A	G	synonymous
142116	intergenic	intergenic	C	T	intergenic
142120	intergenic	intergenic	C	G	intergenic
142169	NTHI477_RS09485	48	A	C	synonymous
142175	NTHI477_RS09485	54	T	C	synonymous
142184	NTHI477_RS09485	63	A	G	synonymous
142236	NTHI477_RS09485	115	T	C	synonymous
142247	NTHI477_RS09485	126	C	T	synonymous
142303	NTHI477_RS09485	182	T	C	nonsynonymous
142330	NTHI477_RS09485	209	C	G	nonsynonymous
142368	NTHI477_RS09485	247	T	C	nonsynonymous
142415	NTHI477_RS09485	294	G	A	synonymous
142457	NTHI477_RS09485	336	C	T	synonymous
142468	NTHI477_RS09485	347	G	A	nonsynonymous
142481	NTHI477_RS09485	360	C	G	nonsynonymous
142590	NTHI477_RS09485	469	A	G	nonsynonymous

142602	NTHI477_RS09485	481	G	A	nonsynonymous
142605	NTHI477_RS09485	484	G	A	nonsynonymous
142641	NTHI477_RS09485	520	G	A	nonsynonymous
142644	NTHI477_RS09485	523	C	T	nonsynonymous
142654	NTHI477_RS09485	533	A	G	synonymous
142752	NTHI477_RS09485	631	C	G	nonsynonymous
142777	NTHI477_RS09485	656	G	A	synonymous
142784	NTHI477_RS09485	663	A	G	synonymous
142803	NTHI477_RS09485	682	A	T	nonsynonymous
142848	NTHI477_RS09485	727	T	C	nonsynonymous
142856	NTHI477_RS09485	735	T	C	synonymous
142857	NTHI477_RS09485	736	G	A	nonsynonymous
142863	NTHI477_RS09485	742	G	A	nonsynonymous
142872	NTHI477_RS09485	751	A	G	nonsynonymous
142882	NTHI477_RS09485	761	A	T	nonsynonymous
142890	NTHI477_RS09485	769	G	A	nonsynonymous
142900	NTHI477_RS09485	779	G	A	nonsynonymous
142917	NTHI477_RS09485	796	T	C	nonsynonymous
142934	NTHI477_RS09485	813	C	T	synonymous
142935	NTHI477_RS09485	814	G	T	nonsynonymous
142941	NTHI477_RS09485	820	C	A	nonsynonymous
142968	NTHI477_RS09485	847	T	A	nonsynonymous
142972	NTHI477_RS09485	851	T	C	nonsynonymous
142980	NTHI477_RS09485	859	G	A	nonsynonymous
142983	NTHI477_RS09485	862	G	T	nonsynonymous
142992	NTHI477_RS09485	871	A	T	nonsynonymous
142999	NTHI477_RS09485	878	G	A	nonsynonymous
143001	NTHI477_RS09485	880	G	A	nonsynonymous
143004	NTHI477_RS09485	883	G	A	nonsynonymous
143043	NTHI477_RS09485	922	T	G	nonsynonymous
143046	NTHI477_RS09485	925	A	T	nonsynonymous
143061	NTHI477_RS09485	940	G	A	nonsynonymous
143067	NTHI477_RS09485	946	C	T	nonsynonymous
143076	NTHI477_RS09485	955	C	T	nonsynonymous
143079	NTHI477_RS09485	958	C	T	nonsynonymous
143093	NTHI477_RS09485	972	C	A	nonsynonymous
143148	NTHI477_RS09485	1027	A	G	nonsynonymous
143172	NTHI477_RS09485	1051	C	A	synonymous
143198	intergenic	intergenic	A	G	intergenic
143302	NTHI477_RS00665	600	G	A	synonymous
143311	NTHI477_RS00665	591	G	A	synonymous
143335	NTHI477_RS00665	567	C	T	synonymous
143341	NTHI477_RS00665	561	A	G	synonymous
143344	NTHI477_RS00665	558	T	A	synonymous
143356	NTHI477_RS00665	546	A	G	synonymous

143374	NTHI477_RS00665	528	T	C	synonymous
143394	NTHI477_RS00665	508	G	A	nonsynonymous
143416	NTHI477_RS00665	486	T	A	synonymous
143419	NTHI477_RS00665	483	T	A	synonymous
143422	NTHI477_RS00665	480	G	T	synonymous
143425	NTHI477_RS00665	477	C	A	synonymous
143425	NTHI477_RS00665	477	C	T	synonymous
143428	NTHI477_RS00665	474	T	A	synonymous
143449	NTHI477_RS00665	453	T	A	synonymous
143461	NTHI477_RS00665	441	A	C	synonymous
143464	NTHI477_RS00665	438	T	C	synonymous
143542	NTHI477_RS00665	360	A	G	synonymous
143584	NTHI477_RS00665	318	A	G	synonymous
143587	NTHI477_RS00665	315	A	G	synonymous
143713	NTHI477_RS00665	189	A	G	synonymous
143716	NTHI477_RS00665	186	T	A	synonymous
143722	NTHI477_RS00665	180	A	G	synonymous
143722	NTHI477_RS00665	180	A	T	synonymous
143724	NTHI477_RS00665	178	G	A	nonsynonymous
143725	NTHI477_RS00665	177	C	T	synonymous
143728	NTHI477_RS00665	174	A	G	synonymous
143731	NTHI477_RS00665	171	T	C	synonymous
143740	NTHI477_RS00665	162	A	G	synonymous
143743	NTHI477_RS00665	159	C	T	synonymous
143752	NTHI477_RS00665	150	C	T	synonymous
143758	NTHI477_RS00665	144	G	A	synonymous
143761	NTHI477_RS00665	141	T	C	synonymous
143836	NTHI477_RS00665	66	A	G	synonymous
143845	NTHI477_RS00665	57	T	C	synonymous
143857	NTHI477_RS00665	45	T	C	synonymous
143863	NTHI477_RS00665	39	A	G	synonymous
143922	intergenic	intergenic	G	T	intergenic
143931	intergenic	intergenic	A	G	intergenic
143956	intergenic	intergenic	C	T	intergenic
143964	intergenic	intergenic	A	C	intergenic
144001	NTHI477_RS00670	1380	G	A	synonymous
144034	NTHI477_RS00670	1347	A	C	synonymous
144052	NTHI477_RS00670	1329	G	A	synonymous
144058	NTHI477_RS00670	1323	A	G	synonymous
144091	NTHI477_RS00670	1290	A	T	synonymous
144094	NTHI477_RS00670	1287	A	G	synonymous
144095	NTHI477_RS00670	1286	C	G	nonsynonymous
144097	NTHI477_RS00670	1284	G	A	synonymous
144100	NTHI477_RS00670	1281	G	A	synonymous
144106	NTHI477_RS00670	1275	A	G	synonymous

144109	NTHI477_RS00670	1272	A	C	synonymous
144115	NTHI477_RS00670	1266	G	A	synonymous
144127	NTHI477_RS00670	1254	A	G	synonymous
144133	NTHI477_RS00670	1248	A	T	nonsynonymous
144136	NTHI477_RS00670	1245	T	A	synonymous
144160	NTHI477_RS00670	1221	G	A	synonymous
144172	NTHI477_RS00670	1209	C	T	synonymous
144175	NTHI477_RS00670	1206	C	G	nonsynonymous
144223	NTHI477_RS00670	1158	T	A	synonymous
144226	NTHI477_RS00670	1155	A	G	synonymous
144226	NTHI477_RS00670	1155	A	T	synonymous
144232	NTHI477_RS00670	1149	C	T	synonymous
144235	NTHI477_RS00670	1146	G	A	synonymous
144244	NTHI477_RS00670	1137	C	T	synonymous
144292	NTHI477_RS00670	1089	G	A	synonymous
144310	NTHI477_RS00670	1071	C	T	synonymous
144340	NTHI477_RS00670	1041	C	T	synonymous
144373	NTHI477_RS00670	1008	A	G	synonymous
144376	NTHI477_RS00670	1005	G	A	synonymous
144403	NTHI477_RS00670	978	C	T	synonymous
144451	NTHI477_RS00670	930	C	T	synonymous
144529	NTHI477_RS00670	852	G	A	synonymous
144556	NTHI477_RS00670	825	A	G	synonymous
144604	NTHI477_RS00670	777	G	A	synonymous
144622	NTHI477_RS00670	759	A	G	synonymous
144643	NTHI477_RS00670	738	A	C	synonymous
144676	NTHI477_RS00670	705	A	G	synonymous
144691	NTHI477_RS00670	690	A	G	synonymous
144721	NTHI477_RS00670	660	A	G	synonymous
144793	NTHI477_RS00670	588	C	T	synonymous
144793	NTHI477_RS00670	588	C	A	synonymous
144810	NTHI477_RS00670	571	T	C	nonsynonymous
144814	NTHI477_RS00670	567	T	A	synonymous
144871	NTHI477_RS00670	510	G	A	synonymous
144880	NTHI477_RS00670	501	A	C	synonymous
144910	NTHI477_RS00670	471	A	G	synonymous
144946	NTHI477_RS00670	435	C	T	synonymous
144955	NTHI477_RS00670	426	A	G	synonymous
144964	NTHI477_RS00670	417	C	T	synonymous
144973	NTHI477_RS00670	408	G	A	synonymous
145006	NTHI477_RS00670	375	T	C	synonymous
145048	NTHI477_RS00670	333	T	C	synonymous
145060	NTHI477_RS00670	321	T	A	synonymous
145111	NTHI477_RS00670	270	C	A	synonymous
145123	NTHI477_RS00670	258	A	T	synonymous

145125	NTHI477_RS00670	256	A	C	nonsynonymous
145147	NTHI477_RS00670	234	A	G	synonymous
145152	NTHI477_RS00670	229	C	T	nonsynonymous
145203	NTHI477_RS00670	178	T	C	nonsynonymous
145222	NTHI477_RS00670	159	T	C	synonymous
145240	NTHI477_RS00670	141	G	A	synonymous
145424	intergenic	intergenic	C	T	intergenic
145438	intergenic	intergenic	A	G	intergenic
145465	intergenic	intergenic	G	A	intergenic
145472	intergenic	intergenic	C	T	intergenic
145476	intergenic	intergenic	C	T	intergenic
145492	intergenic	intergenic	A	C	intergenic
145507	intergenic	intergenic	C	T	intergenic
145523	intergenic	intergenic	G	A	intergenic
145532	intergenic	intergenic	C	T	intergenic
145548	intergenic	intergenic	A	G	intergenic
145561	intergenic	intergenic	T	C	intergenic
145630	NTHI477_RS00675	24	T	C	synonymous
145659	NTHI477_RS00675	53	C	T	nonsynonymous
145705	NTHI477_RS00675	99	C	T	synonymous
145706	NTHI477_RS00675	100	G	A	nonsynonymous
145714	NTHI477_RS00675	108	T	C	synonymous
145729	NTHI477_RS00675	123	C	T	synonymous
145766	NTHI477_RS00675	160	A	G	nonsynonymous
145767	NTHI477_RS00675	161	C	T	nonsynonymous
145768	NTHI477_RS00675	162	C	T	synonymous
145771	NTHI477_RS00675	165	A	G	synonymous
145774	NTHI477_RS00675	168	C	T	synonymous
145829	NTHI477_RS00675	223	G	T	nonsynonymous
145831	NTHI477_RS00675	225	G	A	synonymous
145837	NTHI477_RS00675	231	G	T	synonymous
145867	NTHI477_RS00675	261	A	T	synonymous
145870	NTHI477_RS00675	264	A	G	synonymous
145873	NTHI477_RS00675	267	T	C	synonymous
145885	NTHI477_RS00675	279	A	G	synonymous
145891	NTHI477_RS00675	285	G	A	synonymous
145939	NTHI477_RS00675	333	T	C	synonymous
145951	NTHI477_RS00675	345	T	G	synonymous
145960	NTHI477_RS00675	354	T	C	synonymous
145975	NTHI477_RS00675	369	A	G	synonymous
145978	NTHI477_RS00675	372	G	A	synonymous
145981	NTHI477_RS00675	375	G	A	synonymous
145996	NTHI477_RS00675	390	T	C	synonymous
146005	NTHI477_RS00675	399	G	A	synonymous
146008	NTHI477_RS00675	402	T	A	synonymous

146011	NTHI477_RS00675	405	T	C	synonymous
146083	intergenic	intergenic	C	G	intergenic
146092	NTHI477_RS00680	9	A	G	synonymous
146131	NTHI477_RS00680	48	G	A	synonymous
146137	NTHI477_RS00680	54	G	A	synonymous
146143	NTHI477_RS00680	60	T	C	synonymous
146167	NTHI477_RS00680	84	A	G	synonymous
146167	NTHI477_RS00680	84	A	C	synonymous
146173	NTHI477_RS00680	90	C	T	synonymous
146173	NTHI477_RS00680	90	C	A	synonymous
146184	NTHI477_RS00680	101	C	T	nonsynonymous
146227	NTHI477_RS00680	144	T	A	nonsynonymous
146268	NTHI477_RS00680	185	T	C	nonsynonymous
146270	NTHI477_RS00680	187	G	A	nonsynonymous
146313	NTHI477_RS00680	230	T	C	nonsynonymous
146320	NTHI477_RS00680	237	C	T	synonymous
146321	NTHI477_RS00680	238	G	A	nonsynonymous
146350	NTHI477_RS00680	267	G	T	synonymous
146359	NTHI477_RS00680	276	A	G	synonymous
146365	NTHI477_RS00680	282	G	A	synonymous
146368	NTHI477_RS00680	285	C	T	synonymous
146381	NTHI477_RS00680	298	T	C	nonsynonymous
146382	NTHI477_RS00680	299	G	A	nonsynonymous
146386	NTHI477_RS00680	303	C	T	synonymous
146389	NTHI477_RS00680	306	G	A	synonymous
146432	NTHI477_RS00680	349	G	A	nonsynonymous
146443	NTHI477_RS00680	360	A	C	synonymous
146501	NTHI477_RS00680	418	G	A	nonsynonymous
146533	intergenic	intergenic	G	A	intergenic
146552	intergenic	intergenic	T	C	intergenic
146568	intergenic	intergenic	T	A	intergenic
146598	NTHI477_RS00685	14	A	T	nonsynonymous
146615	NTHI477_RS00685	31	C	G	nonsynonymous
146621	NTHI477_RS00685	37	T	G	nonsynonymous
146651	NTHI477_RS00685	67	G	A	nonsynonymous
146659	NTHI477_RS00685	75	A	G	synonymous
146671	NTHI477_RS00685	87	C	T	synonymous
146680	NTHI477_RS00685	96	G	A	synonymous
146681	NTHI477_RS00685	97	C	A	nonsynonymous
146689	NTHI477_RS00685	105	C	T	synonymous
146690	NTHI477_RS00685	106	G	A	nonsynonymous
146692	NTHI477_RS00685	108	G	T	synonymous
146695	NTHI477_RS00685	111	A	T	synonymous
146722	NTHI477_RS00685	138	C	G	synonymous
146734	NTHI477_RS00685	150	G	A	synonymous

146743	NTHI477_RS00685	159	G	T	synonymous
146755	NTHI477_RS00685	171	A	G	synonymous
146757	NTHI477_RS00685	173	C	G	nonsynonymous
146758	NTHI477_RS00685	174	C	T	synonymous
146763	NTHI477_RS00685	179	C	T	nonsynonymous
146764	NTHI477_RS00685	180	A	G	synonymous
146764	NTHI477_RS00685	180	A	T	synonymous
146768	NTHI477_RS00685	184	G	A	nonsynonymous
146769	NTHI477_RS00685	185	A	C	nonsynonymous
146770	NTHI477_RS00685	186	G	A	synonymous
146773	NTHI477_RS00685	189	C	T	synonymous
146776	NTHI477_RS00685	192	G	A	synonymous
146782	NTHI477_RS00685	198	T	C	synonymous
146785	NTHI477_RS00685	201	G	A	synonymous
146788	NTHI477_RS00685	204	G	C	synonymous
146827	NTHI477_RS00685	243	A	T	synonymous
146828	NTHI477_RS00685	244	C	T	nonsynonymous
146829	NTHI477_RS00685	245	A	T	nonsynonymous
146845	NTHI477_RS00685	261	A	T	synonymous
146846	NTHI477_RS00685	262	C	T	synonymous
146872	NTHI477_RS00685	288	C	T	synonymous
146882	NTHI477_RS00685	298	A	C	nonsynonymous
146896	NTHI477_RS00685	312	C	T	synonymous
146908	NTHI477_RS00685	324	A	G	synonymous
146911	NTHI477_RS00685	327	C	T	synonymous
146913	NTHI477_RS00685	329	C	G	nonsynonymous
146914	NTHI477_RS00685	330	G	A	synonymous
146965	NTHI477_RS00685	381	T	C	synonymous
146968	NTHI477_RS00685	384	G	A	synonymous
146977	NTHI477_RS00685	393	G	C	synonymous
146983	NTHI477_RS00685	399	G	A	synonymous
146992	NTHI477_RS00685	408	T	C	synonymous
146993	NTHI477_RS00685	409	T	C	nonsynonymous
146998	NTHI477_RS00685	414	C	T	synonymous
147005	NTHI477_RS00685	421	A	G	nonsynonymous
147006	NTHI477_RS00685	422	A	G	nonsynonymous
147007	NTHI477_RS00685	423	C	G	nonsynonymous
147010	NTHI477_RS00685	426	A	G	synonymous
147012	NTHI477_RS00685	428	G	C	nonsynonymous
147013	NTHI477_RS00685	429	A	G	synonymous
147030	NTHI477_RS00685	446	C	T	nonsynonymous
147031	NTHI477_RS00685	447	A	G	synonymous
147035	NTHI477_RS00685	451	A	G	nonsynonymous
147037	NTHI477_RS00685	453	C	T	synonymous
147040	NTHI477_RS00685	456	T	C	synonymous

147064	NTHI477_RS00685	480	T	C	synonymous
147076	NTHI477_RS00685	492	C	T	synonymous
147079	NTHI477_RS00685	495	G	A	synonymous
147082	NTHI477_RS00685	498	A	T	synonymous
147091	NTHI477_RS00685	507	A	G	synonymous
147102	NTHI477_RS00685	518	T	C	nonsynonymous
147148	NTHI477_RS00685	564	A	T	synonymous
147160	NTHI477_RS00685	576	C	T	synonymous
147163	NTHI477_RS00685	579	A	G	synonymous
147181	NTHI477_RS00685	597	G	A	synonymous
147186	NTHI477_RS00685	602	C	T	nonsynonymous
147194	NTHI477_RS00685	610	G	A	nonsynonymous
147195	NTHI477_RS00685	611	A	G	nonsynonymous
147196	NTHI477_RS00685	612	T	C	synonymous
147202	NTHI477_RS00685	618	T	C	synonymous
147208	NTHI477_RS00685	624	T	A	synonymous
147230	NTHI477_RS00685	646	C	T	synonymous
147235	NTHI477_RS00685	651	A	C	synonymous
147259	NTHI477_RS00685	675	A	G	synonymous
147265	NTHI477_RS00685	681	T	C	synonymous
147277	NTHI477_RS00685	693	T	C	synonymous
147282	NTHI477_RS00685	698	T	C	nonsynonymous
147287	NTHI477_RS00685	703	C	T	synonymous
147289	NTHI477_RS00685	705	G	A	synonymous
147289	NTHI477_RS00685	705	G	T	synonymous
147290	NTHI477_RS00685	706	A	T	nonsynonymous
147292	NTHI477_RS00685	708	T	C	synonymous
147293	NTHI477_RS00685	709	G	T	nonsynonymous
147311	NTHI477_RS00685	727	T	G	nonsynonymous
147319	NTHI477_RS00685	735	A	G	synonymous
147320	NTHI477_RS00685	736	A	C	nonsynonymous
147335	NTHI477_RS00685	751	A	G	nonsynonymous
147336	NTHI477_RS00685	752	C	T	nonsynonymous
147354	NTHI477_RS00685	770	C	T	nonsynonymous
147360	NTHI477_RS00685	776	G	C	nonsynonymous
147364	NTHI477_RS00685	780	T	C	synonymous
147367	NTHI477_RS00685	783	C	A	synonymous
147373	NTHI477_RS00685	789	C	T	synonymous
147382	NTHI477_RS00685	798	T	C	synonymous
147391	NTHI477_RS00685	807	C	T	synonymous
147394	NTHI477_RS00685	810	A	T	synonymous
147398	NTHI477_RS00685	814	C	A	nonsynonymous
147400	NTHI477_RS00685	816	C	A	synonymous
147401	NTHI477_RS00685	817	G	A	nonsynonymous
147405	NTHI477_RS00685	821	A	C	nonsynonymous

147442	NTHI477_RS00685	858	C	T	synonymous
147446	NTHI477_RS00685	862	C	T	nonsynonymous
147451	NTHI477_RS00685	867	C	T	synonymous
147455	NTHI477_RS00685	871	T	C	nonsynonymous
147457	NTHI477_RS00685	873	T	C	synonymous
147457	NTHI477_RS00685	873	T	A	nonsynonymous
147463	NTHI477_RS00685	879	T	C	synonymous
147464	NTHI477_RS00685	880	A	C	nonsynonymous
147466	NTHI477_RS00685	882	A	G	synonymous
147469	NTHI477_RS00685	885	T	C	synonymous
147471	NTHI477_RS00685	887	A	C	nonsynonymous
147472	NTHI477_RS00685	888	T	A	nonsynonymous
147485	NTHI477_RS00685	901	G	A	nonsynonymous
147493	NTHI477_RS00685	909	T	G	synonymous
147493	NTHI477_RS00685	909	T	A	synonymous
147496	NTHI477_RS00685	912	C	T	synonymous
147508	NTHI477_RS00685	924	G	T	synonymous
147524	NTHI477_RS00685	940	A	T	nonsynonymous
147531	NTHI477_RS00685	947	T	C	nonsynonymous
147532	NTHI477_RS00685	948	T	C	synonymous
147541	NTHI477_RS00685	957	C	T	synonymous
147544	NTHI477_RS00685	960	T	G	synonymous
147547	NTHI477_RS00685	963	C	T	synonymous
147599	NTHI477_RS00690	32	C	T	nonsynonymous
147621	NTHI477_RS00690	54	G	A	synonymous
147622	NTHI477_RS00690	55	C	T	nonsynonymous
147624	NTHI477_RS00690	57	T	C	synonymous
147627	NTHI477_RS00690	60	C	T	synonymous
147639	NTHI477_RS00690	72	C	A	synonymous
147642	NTHI477_RS00690	75	G	A	synonymous
147645	NTHI477_RS00690	78	T	C	synonymous
147652	NTHI477_RS00690	85	T	C	nonsynonymous
147657	NTHI477_RS00690	90	A	T	synonymous
147660	NTHI477_RS00690	93	A	T	synonymous
147663	NTHI477_RS00690	96	C	T	synonymous
147666	NTHI477_RS00690	99	C	T	synonymous
147669	NTHI477_RS00690	102	T	G	synonymous
147678	NTHI477_RS00690	111	C	T	synonymous
147681	NTHI477_RS00690	114	A	G	synonymous
147693	NTHI477_RS00690	126	T	G	nonsynonymous
147699	NTHI477_RS00690	132	T	C	synonymous
147708	NTHI477_RS00690	141	A	G	synonymous
147714	NTHI477_RS00690	147	C	T	synonymous
147717	NTHI477_RS00690	150	A	G	synonymous
147718	NTHI477_RS00690	151	T	C	synonymous

147723	NTHI477_RS00690	156	C	T	synonymous
147731	NTHI477_RS00690	164	T	C	nonsynonymous
147744	NTHI477_RS00690	177	C	T	synonymous
147760	NTHI477_RS00690	193	C	T	synonymous
147780	NTHI477_RS00690	213	C	T	synonymous
147789	NTHI477_RS00690	222	T	C	synonymous
147792	NTHI477_RS00690	225	T	A	synonymous
147804	NTHI477_RS00690	237	C	T	synonymous
147807	NTHI477_RS00690	240	A	G	synonymous
147810	NTHI477_RS00690	243	C	T	synonymous
147841	NTHI477_RS00690	274	G	A	nonsynonymous
147843	NTHI477_RS00690	276	C	T	synonymous
147846	NTHI477_RS00690	279	C	T	synonymous
147864	NTHI477_RS00690	297	A	G	synonymous
147870	NTHI477_RS00690	303	A	C	synonymous
147895	NTHI477_RS00690	328	T	G	nonsynonymous
147898	NTHI477_RS00690	331	G	A	nonsynonymous
147898	NTHI477_RS00690	331	G	T	nonsynonymous
147899	NTHI477_RS00690	332	C	T	nonsynonymous
147908	NTHI477_RS00690	341	C	T	nonsynonymous
147910	NTHI477_RS00690	343	T	C	synonymous
147915	NTHI477_RS00690	348	C	T	synonymous
147924	NTHI477_RS00690	357	T	C	synonymous
147942	NTHI477_RS00690	375	T	C	synonymous
147957	NTHI477_RS00690	390	T	G	nonsynonymous
147961	NTHI477_RS00690	394	G	A	nonsynonymous
147969	NTHI477_RS00690	402	C	A	synonymous
147974	NTHI477_RS00690	407	A	G	nonsynonymous
147984	NTHI477_RS00690	417	C	T	synonymous
147991	NTHI477_RS00690	424	A	G	nonsynonymous
147999	NTHI477_RS00690	432	C	T	synonymous
148002	NTHI477_RS00690	435	T	A	synonymous
148008	NTHI477_RS00690	441	G	A	synonymous
148020	NTHI477_RS00690	453	T	C	synonymous
148020	NTHI477_RS00690	453	T	G	synonymous
148029	NTHI477_RS00690	462	T	C	synonymous
148038	NTHI477_RS00690	471	T	A	synonymous
148044	NTHI477_RS00690	477	A	G	synonymous
148053	NTHI477_RS00690	486	G	A	nonsynonymous
148064	NTHI477_RS00695	6	A	G	synonymous
148076	NTHI477_RS00695	18	T	A	synonymous
148091	NTHI477_RS00695	33	A	G	synonymous
148091	NTHI477_RS00695	33	A	C	synonymous
148100	NTHI477_RS00695	42	G	C	synonymous
148100	NTHI477_RS00695	42	G	A	synonymous

148106	NTHI477_RS00695	48	G	C	synonymous
148115	NTHI477_RS00695	57	C	T	synonymous
148118	NTHI477_RS00695	60	T	C	synonymous
148130	NTHI477_RS00695	72	A	G	synonymous
148136	NTHI477_RS00695	78	G	A	synonymous
148139	NTHI477_RS00695	81	A	G	synonymous
148145	NTHI477_RS00695	87	C	T	synonymous
148169	NTHI477_RS00695	111	C	T	synonymous
148178	NTHI477_RS00695	120	C	T	synonymous
148185	NTHI477_RS00695	127	C	T	nonsynonymous
148190	NTHI477_RS00695	132	T	C	synonymous
148191	NTHI477_RS00695	133	A	G	nonsynonymous
148196	NTHI477_RS00695	138	T	C	synonymous
148204	NTHI477_RS00695	146	G	C	nonsynonymous
148214	NTHI477_RS00695	156	T	A	synonymous
148226	NTHI477_RS00695	168	G	A	synonymous
148235	NTHI477_RS00695	177	G	A	synonymous
148244	NTHI477_RS00695	186	C	T	synonymous
148250	NTHI477_RS00695	192	A	T	synonymous
148262	NTHI477_RS00695	204	C	T	synonymous
148280	NTHI477_RS00695	222	C	T	synonymous
148290	NTHI477_RS00695	232	T	C	synonymous
148301	NTHI477_RS00695	243	C	T	synonymous
148304	NTHI477_RS00695	246	T	C	synonymous
148310	NTHI477_RS00695	252	A	T	nonsynonymous
148322	NTHI477_RS00695	264	C	T	synonymous
148329	NTHI477_RS00695	271	T	C	nonsynonymous
148335	NTHI477_RS00695	277	C	A	nonsynonymous
148358	NTHI477_RS00695	300	T	C	synonymous
148359	NTHI477_RS00695	301	G	A	nonsynonymous
148370	NTHI477_RS00695	312	G	A	synonymous
148379	NTHI477_RS00695	321	A	G	synonymous
148382	NTHI477_RS00695	324	C	T	synonymous
148403	NTHI477_RS00695	345	G	A	synonymous
148409	NTHI477_RS00695	351	A	C	nonsynonymous
148412	NTHI477_RS00695	354	G	T	synonymous
148416	NTHI477_RS00695	358	T	C	synonymous
148421	NTHI477_RS00695	363	C	T	synonymous
148466	NTHI477_RS00695	408	C	T	synonymous
148472	NTHI477_RS00695	414	A	G	synonymous
148499	NTHI477_RS00695	441	C	T	synonymous
148509	NTHI477_RS00695	451	A	G	nonsynonymous
148510	NTHI477_RS00695	452	T	C	nonsynonymous
148511	NTHI477_RS00695	453	G	A	nonsynonymous
148601	NTHI477_RS00695	543	C	T	synonymous

148622	NTHI477_RS00695	564	A	G	synonymous
148651	NTHI477_RS00695	593	C	T	nonsynonymous
148656	NTHI477_RS00695	598	G	C	nonsynonymous
148658	NTHI477_RS00695	600	T	C	synonymous
148664	NTHI477_RS00695	606	C	T	synonymous
148691	intergenic	intergenic	G	A	intergenic
148750	NTHI477_RS00700	46	C	T	nonsynonymous
148752	NTHI477_RS00700	48	T	C	synonymous
148778	NTHI477_RS00700	74	C	T	nonsynonymous
148779	NTHI477_RS00700	75	G	T	synonymous
148800	NTHI477_RS00700	96	G	A	synonymous
148803	NTHI477_RS00700	99	T	G	synonymous
148830	NTHI477_RS00700	126	T	C	synonymous
148833	NTHI477_RS00700	129	G	A	synonymous
148836	NTHI477_RS00700	132	G	A	synonymous
148841	NTHI477_RS00700	137	C	T	nonsynonymous
148854	NTHI477_RS00700	150	A	G	synonymous
148857	NTHI477_RS00700	153	A	C	synonymous
148866	NTHI477_RS00700	162	T	A	nonsynonymous
148867	NTHI477_RS00700	163	C	A	nonsynonymous
148872	NTHI477_RS00700	168	C	T	synonymous
148875	NTHI477_RS00700	171	T	A	synonymous
148876	NTHI477_RS00700	172	G	A	nonsynonymous
148877	NTHI477_RS00700	173	C	T	nonsynonymous
148883	NTHI477_RS00700	179	C	T	nonsynonymous
148887	NTHI477_RS00700	183	C	T	synonymous
148926	NTHI477_RS00700	222	C	T	synonymous
148935	NTHI477_RS00700	231	C	T	synonymous
148947	NTHI477_RS00700	243	C	T	synonymous
148948	NTHI477_RS00700	244	T	A	nonsynonymous
148956	NTHI477_RS00700	252	A	G	synonymous
148964	NTHI477_RS00700	260	C	T	nonsynonymous
148971	NTHI477_RS00700	267	C	T	synonymous
149007	NTHI477_RS00700	303	G	A	synonymous
149017	NTHI477_RS00700	313	C	G	nonsynonymous
149029	NTHI477_RS00700	325	G	A	nonsynonymous
149030	NTHI477_RS00700	326	C	T	nonsynonymous
149031	NTHI477_RS00700	327	A	G	synonymous
149091	NTHI477_RS00700	387	C	T	synonymous
149124	NTHI477_RS00700	420	A	G	synonymous
149148	NTHI477_RS00700	444	C	T	synonymous
149168	NTHI477_RS00700	464	C	T	nonsynonymous
149169	NTHI477_RS00700	465	T	G	synonymous
149175	NTHI477_RS00700	471	C	T	synonymous
149178	NTHI477_RS00700	474	T	C	synonymous

149181	NTHI477_RS00700	477	T	C	synonymous
149187	NTHI477_RS00700	483	T	G	synonymous
149190	NTHI477_RS00700	486	T	C	synonymous
149193	NTHI477_RS00700	489	G	A	synonymous
149196	NTHI477_RS00700	492	A	T	synonymous
149205	NTHI477_RS00700	501	T	C	synonymous
149214	NTHI477_RS00700	510	T	A	synonymous
149232	NTHI477_RS00700	528	A	G	synonymous
149238	NTHI477_RS00700	534	C	T	synonymous
149243	NTHI477_RS00700	539	G	A	nonsynonymous
149247	NTHI477_RS00700	543	C	T	synonymous
149259	NTHI477_RS00700	555	G	T	synonymous
149268	NTHI477_RS00700	564	C	G	synonymous
149286	NTHI477_RS00700	582	T	C	synonymous
149292	NTHI477_RS00700	588	C	A	synonymous
149304	NTHI477_RS00700	600	G	C	synonymous
149304	NTHI477_RS00700	600	G	T	synonymous
149322	NTHI477_RS00700	618	A	T	synonymous
149331	NTHI477_RS00700	627	C	T	synonymous
149331	NTHI477_RS00700	627	C	A	synonymous
149357	NTHI477_RS00700	653	C	T	nonsynonymous
149358	NTHI477_RS00700	654	G	T	synonymous
149367	NTHI477_RS00700	663	C	T	synonymous
149370	NTHI477_RS00700	666	A	T	synonymous
149379	NTHI477_RS00700	675	C	T	synonymous
149382	NTHI477_RS00700	678	A	G	synonymous
149442	NTHI477_RS00700	738	T	A	synonymous
149529	NTHI477_RS00705	232	G	T	nonsynonymous
149587	NTHI477_RS00705	174	A	G	synonymous
149677	NTHI477_RS00705	84	A	G	synonymous
149688	NTHI477_RS00705	73	C	T	nonsynonymous
149689	NTHI477_RS00705	72	G	A	synonymous
149707	NTHI477_RS00705	54	T	C	synonymous
149749	NTHI477_RS00705	12	G	A	synonymous
149787	intergenic	intergenic	A	G	intergenic
149789	intergenic	intergenic	T	C	intergenic
149865	NTHI477_RS00710	60	T	C	synonymous
149884	NTHI477_RS00710	79	G	T	nonsynonymous
149910	NTHI477_RS00710	105	G	T	synonymous
149946	NTHI477_RS00710	141	T	G	synonymous
149946	NTHI477_RS00710	141	T	A	synonymous
149976	NTHI477_RS00710	171	T	G	synonymous
149979	NTHI477_RS00710	174	T	G	synonymous
150007	NTHI477_RS00710	202	G	A	nonsynonymous
150031	NTHI477_RS00710	226	T	C	synonymous

150046	NTHI477_RS00710	241	C	A	nonsynonymous
150046	NTHI477_RS00710	241	C	T	synonymous
150058	NTHI477_RS00710	253	A	G	nonsynonymous
150064	NTHI477_RS00710	259	A	G	nonsynonymous
150069	NTHI477_RS00710	264	A	G	synonymous
150076	NTHI477_RS00710	271	T	G	nonsynonymous
150096	NTHI477_RS00710	291	C	T	synonymous
150126	NTHI477_RS00710	321	C	T	synonymous
150138	NTHI477_RS00710	333	C	T	synonymous
150157	NTHI477_RS00710	352	G	A	nonsynonymous
150183	NTHI477_RS00710	378	T	A	synonymous
150183	NTHI477_RS00710	378	T	G	synonymous
150189	NTHI477_RS00710	384	T	C	synonymous
150192	NTHI477_RS00710	387	T	C	synonymous
150198	NTHI477_RS00710	393	C	T	synonymous
150199	NTHI477_RS00710	394	A	G	nonsynonymous
150219	NTHI477_RS00710	414	T	A	nonsynonymous
150220	NTHI477_RS00710	415	T	C	synonymous
150233	NTHI477_RS00710	428	T	C	nonsynonymous
150258	NTHI477_RS00710	453	C	T	synonymous
150297	NTHI477_RS00710	492	T	C	synonymous
150298	NTHI477_RS00710	493	G	A	nonsynonymous
150312	NTHI477_RS00710	507	A	G	synonymous
150317	NTHI477_RS00710	512	G	T	nonsynonymous
150319	NTHI477_RS00710	514	G	T	nonsynonymous
150321	NTHI477_RS00710	516	T	A	synonymous
150349	NTHI477_RS00710	544	G	A	nonsynonymous
150351	NTHI477_RS00710	546	G	A	synonymous
150367	NTHI477_RS00710	562	G	A	nonsynonymous
150388	NTHI477_RS00710	583	G	A	nonsynonymous
150417	NTHI477_RS00710	612	A	G	synonymous
150424	NTHI477_RS00710	619	T	G	nonsynonymous
150426	NTHI477_RS00710	621	G	A	synonymous
150505	NTHI477_RS00710	700	A	G	nonsynonymous
150597	intergenic	intergenic	G	A	intergenic
150655	intergenic	intergenic	G	A	intergenic
150694	intergenic	intergenic	T	G	intergenic
150716	NTHI477_RS00715	22	G	A	nonsynonymous
150756	NTHI477_RS00715	62	A	G	nonsynonymous
150802	NTHI477_RS00715	108	G	A	synonymous
150809	NTHI477_RS00715	115	T	C	nonsynonymous
150827	NTHI477_RS00715	133	C	A	nonsynonymous
150832	NTHI477_RS00715	138	A	G	synonymous
150841	NTHI477_RS00715	147	T	C	synonymous
150850	NTHI477_RS00715	156	T	A	synonymous

150862	NTHI477_RS00715	168	A	T	synonymous
150863	NTHI477_RS00715	169	G	T	nonsynonymous
150865	NTHI477_RS00715	171	T	G	synonymous
150871	NTHI477_RS00715	177	C	T	synonymous
150919	NTHI477_RS00715	225	A	T	synonymous
150926	NTHI477_RS00715	232	G	A	nonsynonymous
150928	NTHI477_RS00715	234	G	A	synonymous
150931	NTHI477_RS00715	237	A	G	synonymous
150976	NTHI477_RS00715	282	T	A	synonymous
150991	NTHI477_RS00715	297	T	A	synonymous
151009	NTHI477_RS00715	315	T	A	synonymous
151009	NTHI477_RS00715	315	T	C	synonymous
151030	NTHI477_RS00715	336	T	C	synonymous
151037	NTHI477_RS00715	343	A	G	nonsynonymous
151045	NTHI477_RS00715	351	T	C	synonymous
151120	NTHI477_RS00715	426	C	T	synonymous
151159	NTHI477_RS00715	465	G	C	synonymous
151174	NTHI477_RS00715	480	C	T	synonymous
151198	NTHI477_RS00715	504	T	C	synonymous
151201	NTHI477_RS00715	507	C	T	synonymous
151204	NTHI477_RS00715	510	C	T	synonymous
151210	NTHI477_RS00715	516	T	G	synonymous
151216	NTHI477_RS00715	522	C	T	synonymous
151267	NTHI477_RS00715	573	T	A	synonymous
151270	NTHI477_RS00715	576	G	A	synonymous
151303	NTHI477_RS00715	609	C	G	synonymous
151306	NTHI477_RS00715	612	C	T	synonymous
151321	NTHI477_RS00715	627	C	T	synonymous
151330	NTHI477_RS00715	636	A	G	synonymous
151331	NTHI477_RS00715	637	T	C	synonymous
151333	NTHI477_RS00715	639	G	A	synonymous
151363	NTHI477_RS00715	669	A	C	synonymous
151369	NTHI477_RS00715	675	G	A	synonymous
151375	NTHI477_RS00715	681	C	T	synonymous
151381	NTHI477_RS00715	687	A	G	synonymous
151390	NTHI477_RS00715	696	C	T	synonymous
151396	NTHI477_RS00715	702	C	T	synonymous
151423	NTHI477_RS00715	729	G	A	synonymous
151447	NTHI477_RS00715	753	G	A	synonymous
151450	NTHI477_RS00715	756	T	C	synonymous
151450	NTHI477_RS00715	756	T	A	nonsynonymous
151453	NTHI477_RS00715	759	T	A	synonymous
151456	NTHI477_RS00715	762	A	G	synonymous
151465	NTHI477_RS00715	771	C	G	synonymous
151489	NTHI477_RS00715	795	C	T	synonymous

151528	NTHI477_RS00715	834	C	T	synonymous
151546	NTHI477_RS00715	852	C	T	synonymous
151582	NTHI477_RS00715	888	C	A	synonymous
151588	NTHI477_RS00715	894	A	G	synonymous
151594	NTHI477_RS00715	900	C	T	synonymous
151627	NTHI477_RS00715	933	C	A	synonymous
151666	NTHI477_RS00715	972	T	C	synonymous
151696	intergenic	intergenic	A	T	intergenic
151756	NTHI477_RS00720	39	T	C	synonymous
151759	NTHI477_RS00720	42	A	T	synonymous
151760	NTHI477_RS00720	43	T	G	nonsynonymous
151762	NTHI477_RS00720	45	C	G	synonymous
151765	NTHI477_RS00720	48	T	G	synonymous
151768	NTHI477_RS00720	51	A	C	synonymous
151768	NTHI477_RS00720	51	A	T	synonymous
151795	NTHI477_RS00720	78	T	C	synonymous
151813	NTHI477_RS00720	96	G	A	synonymous
151816	NTHI477_RS00720	99	C	T	synonymous
151822	NTHI477_RS00720	105	A	G	synonymous
151826	NTHI477_RS00720	109	G	A	nonsynonymous
151827	NTHI477_RS00720	110	C	G	nonsynonymous
151840	NTHI477_RS00720	123	G	A	synonymous
151856	NTHI477_RS00720	139	A	G	nonsynonymous
151867	NTHI477_RS00720	150	G	A	synonymous
151894	NTHI477_RS00720	177	G	A	synonymous
151908	NTHI477_RS00720	191	T	C	nonsynonymous
151927	NTHI477_RS00720	210	C	T	synonymous
151930	NTHI477_RS00720	213	T	C	synonymous
151948	NTHI477_RS00720	231	T	C	synonymous
151954	NTHI477_RS00720	237	C	T	synonymous
151957	NTHI477_RS00720	240	T	C	synonymous
151961	NTHI477_RS00720	244	C	G	nonsynonymous
151987	NTHI477_RS00720	270	G	C	synonymous
151993	NTHI477_RS00720	276	C	T	synonymous
152017	NTHI477_RS00720	300	G	T	synonymous
152041	NTHI477_RS00720	324	C	G	synonymous
152041	NTHI477_RS00720	324	C	T	synonymous
152050	NTHI477_RS00720	333	C	T	synonymous
152065	NTHI477_RS00720	348	A	G	synonymous
152068	NTHI477_RS00720	351	G	A	synonymous
152077	NTHI477_RS00720	360	T	C	synonymous
152102	NTHI477_RS00720	385	G	A	nonsynonymous
152110	NTHI477_RS00720	393	G	T	synonymous
152113	NTHI477_RS00720	396	T	C	synonymous
152132	NTHI477_RS00720	415	G	C	nonsynonymous

152138	NTHI477_RS00720	421	G	A	nonsynonymous
152146	NTHI477_RS00720	429	C	T	synonymous
152146	NTHI477_RS00720	429	C	A	synonymous
152152	NTHI477_RS00720	435	T	A	synonymous
152174	NTHI477_RS00720	457	C	T	synonymous
152191	NTHI477_RS00720	474	T	C	synonymous
152218	NTHI477_RS00720	501	G	A	synonymous
152245	NTHI477_RS00720	528	A	G	synonymous
152257	NTHI477_RS00720	540	C	T	synonymous
152261	NTHI477_RS00720	544	G	A	nonsynonymous
152272	NTHI477_RS00720	555	G	A	synonymous
152274	NTHI477_RS00720	557	C	T	nonsynonymous
152275	NTHI477_RS00720	558	G	C	synonymous
152350	NTHI477_RS00720	633	A	T	nonsynonymous
152353	NTHI477_RS00720	636	T	C	synonymous
152359	NTHI477_RS00720	642	G	A	synonymous
152396	NTHI477_RS00720	679	G	A	nonsynonymous
152437	NTHI477_RS00720	720	T	C	synonymous
152449	NTHI477_RS00720	732	C	T	synonymous
152455	NTHI477_RS00720	738	T	C	synonymous
152467	NTHI477_RS00720	750	T	C	synonymous
152482	NTHI477_RS00720	765	C	T	synonymous
152495	NTHI477_RS00720	778	C	T	nonsynonymous
152497	NTHI477_RS00720	780	T	C	synonymous
152515	NTHI477_RS00720	798	A	G	synonymous
152533	NTHI477_RS00720	816	C	T	synonymous
152554	NTHI477_RS00720	837	C	T	synonymous
152557	NTHI477_RS00720	840	C	A	nonsynonymous
152560	NTHI477_RS00720	843	G	A	synonymous
152569	NTHI477_RS00720	852	G	A	synonymous
152572	NTHI477_RS00720	855	G	A	synonymous
152598	NTHI477_RS00720	881	T	C	nonsynonymous
152620	NTHI477_RS00720	903	G	T	synonymous
152632	NTHI477_RS00720	915	C	T	synonymous
152645	NTHI477_RS00720	928	C	G	nonsynonymous
152676	NTHI477_RS00720	959	T	C	nonsynonymous
152678	NTHI477_RS00720	961	G	A	nonsynonymous
152679	NTHI477_RS00720	962	C	T	nonsynonymous
152679	NTHI477_RS00720	962	C	A	nonsynonymous
152680	NTHI477_RS00720	963	G	C	synonymous
152694	NTHI477_RS00720	977	G	A	nonsynonymous
152695	NTHI477_RS00720	978	T	C	synonymous
152710	NTHI477_RS00720	993	C	G	synonymous
152722	NTHI477_RS00720	1005	C	T	synonymous
152731	NTHI477_RS00720	1014	T	C	synonymous

152743	NTHI477_RS00720	1026	G	C	synonymous
152743	NTHI477_RS00720	1026	G	T	synonymous
152779	NTHI477_RS00720	1062	T	G	synonymous
152788	NTHI477_RS00720	1071	A	G	synonymous
152791	NTHI477_RS00720	1074	C	T	synonymous
152800	NTHI477_RS00720	1083	G	A	synonymous
152824	NTHI477_RS00720	1107	C	G	synonymous
152849	NTHI477_RS00720	1132	G	A	nonsynonymous
152857	NTHI477_RS00720	1140	G	A	synonymous
152875	NTHI477_RS00720	1158	A	T	synonymous
152881	NTHI477_RS00720	1164	T	G	synonymous
152884	NTHI477_RS00720	1167	G	T	synonymous
152887	NTHI477_RS00720	1170	A	G	synonymous
152893	NTHI477_RS00720	1176	C	T	synonymous
152909	NTHI477_RS00720	1192	A	G	nonsynonymous
152910	NTHI477_RS00720	1193	A	C	nonsynonymous
152949	NTHI477_RS00720	1232	A	G	nonsynonymous
152988	NTHI477_RS00720	1271	A	G	nonsynonymous
152988	NTHI477_RS00720	1271	A	T	nonsynonymous
152991	NTHI477_RS00720	1274	G	T	nonsynonymous
152998	NTHI477_RS00720	1281	A	G	synonymous
153010	NTHI477_RS00720	1293	C	T	synonymous
153012	NTHI477_RS00720	1295	G	A	nonsynonymous
153022	NTHI477_RS00720	1305	C	T	synonymous
153052	NTHI477_RS00720	1335	G	T	synonymous
153101	NTHI477_RS00720	1384	G	A	nonsynonymous
153106	NTHI477_RS00720	1389	G	A	synonymous
153112	NTHI477_RS00720	1395	G	A	synonymous
153115	NTHI477_RS00720	1398	G	A	synonymous
153134	NTHI477_RS00720	1417	A	T	nonsynonymous
153136	NTHI477_RS00720	1419	T	C	synonymous
153145	NTHI477_RS00720	1428	C	T	synonymous
153151	NTHI477_RS00720	1434	G	A	synonymous
153158	NTHI477_RS00720	1441	A	G	nonsynonymous
153160	NTHI477_RS00720	1443	A	G	synonymous
153161	NTHI477_RS00720	1444	C	A	nonsynonymous
153163	NTHI477_RS00720	1446	C	T	synonymous
153192	NTHI477_RS00720	1475	A	G	nonsynonymous
153206	NTHI477_RS00720	1489	A	C	synonymous
153226	NTHI477_RS00720	1509	G	A	synonymous
153247	NTHI477_RS00720	1530	C	T	synonymous
153259	NTHI477_RS00720	1542	C	T	synonymous
153298	NTHI477_RS00720	1581	G	T	nonsynonymous
153299	NTHI477_RS00720	1582	C	A	nonsynonymous
153311	NTHI477_RS00720	1594	G	A	nonsynonymous

153322	NTHI477_RS00720	1605	T	C	synonymous
153325	NTHI477_RS00720	1608	T	A	synonymous
153343	NTHI477_RS00720	1626	A	C	synonymous
153352	NTHI477_RS00720	1635	C	T	synonymous
153352	NTHI477_RS00720	1635	C	A	synonymous
153361	NTHI477_RS00720	1644	G	A	synonymous
153364	NTHI477_RS00720	1647	T	G	nonsynonymous
153364	NTHI477_RS00720	1647	T	C	synonymous
153367	NTHI477_RS00720	1650	T	C	synonymous
153382	NTHI477_RS00720	1665	A	T	synonymous
153412	NTHI477_RS00720	1695	T	C	synonymous
153415	NTHI477_RS00720	1698	T	C	synonymous
153433	NTHI477_RS00720	1716	T	A	synonymous
153439	NTHI477_RS00720	1722	A	G	synonymous
153448	NTHI477_RS00720	1731	A	G	synonymous
153454	NTHI477_RS00720	1737	T	C	synonymous
153457	NTHI477_RS00720	1740	C	T	synonymous
153463	NTHI477_RS00720	1746	T	A	synonymous
153502	NTHI477_RS00720	1785	G	A	synonymous
153547	NTHI477_RS00720	1830	A	G	synonymous
153553	NTHI477_RS00720	1836	A	T	synonymous
153586	NTHI477_RS00720	1869	T	A	synonymous
153601	NTHI477_RS00720	1884	A	T	synonymous
153607	NTHI477_RS00720	1890	G	T	synonymous
153610	NTHI477_RS00720	1893	G	T	synonymous
153617	NTHI477_RS00720	1900	A	G	nonsynonymous
153619	NTHI477_RS00720	1902	C	T	synonymous
153630	NTHI477_RS00720	1913	C	A	nonsynonymous
153645	NTHI477_RS00720	1928	T	C	nonsynonymous
153649	NTHI477_RS00720	1932	A	G	synonymous
153652	NTHI477_RS00720	1935	C	T	synonymous
153655	NTHI477_RS00720	1938	T	A	synonymous
153658	NTHI477_RS00720	1941	T	C	synonymous
153658	NTHI477_RS00720	1941	T	A	synonymous
153664	NTHI477_RS00720	1947	T	A	synonymous
153667	NTHI477_RS00720	1950	T	G	synonymous
153670	NTHI477_RS00720	1953	T	C	synonymous
153685	NTHI477_RS00720	1968	C	T	synonymous
153686	NTHI477_RS00720	1969	C	G	nonsynonymous
153721	NTHI477_RS00720	2004	T	C	synonymous
153727	NTHI477_RS00720	2010	C	T	synonymous
153748	NTHI477_RS00720	2031	C	G	synonymous
153754	NTHI477_RS00720	2037	T	G	synonymous
153856	NTHI477_RS00720	2139	C	T	synonymous
153862	NTHI477_RS00720	2145	A	G	synonymous

153886	NTHI477_RS00720	2169	G	A	synonymous
153910	NTHI477_RS00720	2193	C	T	synonymous
153962	NTHI477_RS00720	2245	A	G	nonsynonymous
153965	NTHI477_RS00720	2248	T	G	nonsynonymous
153997	NTHI477_RS00720	2280	A	G	synonymous
154000	NTHI477_RS00720	2283	A	T	synonymous
154043	NTHI477_RS00720	2326	A	G	nonsynonymous
154057	NTHI477_RS00720	2340	A	T	synonymous
154121	NTHI477_RS00725	15	T	C	synonymous
154125	NTHI477_RS00725	19	C	A	nonsynonymous
154148	NTHI477_RS00725	42	T	C	synonymous
154160	NTHI477_RS00725	54	C	G	nonsynonymous
154187	NTHI477_RS00725	81	G	A	synonymous
154196	NTHI477_RS00725	90	C	T	synonymous
154202	NTHI477_RS00725	96	A	G	synonymous
154211	NTHI477_RS00725	105	T	C	synonymous
154214	NTHI477_RS00725	108	T	C	synonymous
154214	NTHI477_RS00725	108	T	A	synonymous
154216	NTHI477_RS00725	110	C	T	nonsynonymous
154235	NTHI477_RS00725	129	T	A	nonsynonymous
154241	NTHI477_RS00725	135	G	A	synonymous
154244	NTHI477_RS00725	138	A	G	synonymous
154269	NTHI477_RS00725	163	T	C	synonymous
154274	NTHI477_RS00725	168	C	T	synonymous
154286	NTHI477_RS00725	180	T	G	synonymous
154295	NTHI477_RS00725	189	A	G	synonymous
154295	NTHI477_RS00725	189	A	T	synonymous
154304	NTHI477_RS00725	198	G	A	synonymous
154310	NTHI477_RS00725	204	G	A	synonymous
154310	NTHI477_RS00725	204	G	T	synonymous
154313	NTHI477_RS00725	207	C	T	synonymous
154317	NTHI477_RS00725	211	A	G	nonsynonymous
154323	NTHI477_RS00725	217	C	G	nonsynonymous
154325	NTHI477_RS00725	219	G	A	synonymous
154334	NTHI477_RS00725	228	T	G	synonymous
154343	NTHI477_RS00725	237	G	A	synonymous
154343	NTHI477_RS00725	237	G	T	synonymous
154346	NTHI477_RS00725	240	G	C	synonymous
154358	NTHI477_RS00725	252	T	A	synonymous
154376	NTHI477_RS00725	270	C	T	synonymous
154394	NTHI477_RS00725	288	A	G	synonymous
154398	NTHI477_RS00725	292	T	C	nonsynonymous
154402	intergenic	intergenic	C	G	intergenic
154424	intergenic	intergenic	T	C	intergenic
154437	intergenic	intergenic	C	T	intergenic

154447	intergenic	intergenic	A	G	intergenic
154456	NTHI477_RS00730	7	G	A	nonsynonymous
154466	NTHI477_RS00730	17	C	T	nonsynonymous
154469	NTHI477_RS00730	20	T	G	nonsynonymous
154477	NTHI477_RS00730	28	A	G	nonsynonymous
154477	NTHI477_RS00730	28	A	T	nonsynonymous
154488	NTHI477_RS00730	39	A	G	synonymous
154489	NTHI477_RS00730	40	T	C	synonymous
154495	NTHI477_RS00730	46	C	T	synonymous
154509	NTHI477_RS00730	60	T	C	synonymous
154522	NTHI477_RS00730	73	G	A	nonsynonymous
154556	NTHI477_RS00730	107	A	G	nonsynonymous
154557	NTHI477_RS00730	108	C	T	synonymous
154566	NTHI477_RS00730	117	C	T	synonymous
154572	NTHI477_RS00730	123	C	T	synonymous
154608	NTHI477_RS00730	159	G	A	synonymous
154608	NTHI477_RS00730	159	G	T	synonymous
154611	NTHI477_RS00730	162	C	T	synonymous
154629	NTHI477_RS00730	180	C	T	synonymous
154632	NTHI477_RS00730	183	T	C	synonymous
154638	NTHI477_RS00730	189	A	T	synonymous
154641	NTHI477_RS00730	192	T	C	synonymous
154647	NTHI477_RS00730	198	C	G	synonymous
154659	NTHI477_RS00730	210	C	T	synonymous
154683	NTHI477_RS00730	234	A	C	nonsynonymous
154686	NTHI477_RS00730	237	T	C	synonymous
154707	NTHI477_RS00730	258	A	T	synonymous
154713	NTHI477_RS00730	264	C	T	synonymous
154716	NTHI477_RS00730	267	A	T	synonymous
154717	NTHI477_RS00730	268	A	G	nonsynonymous
154718	NTHI477_RS00730	269	C	T	nonsynonymous
154728	NTHI477_RS00730	279	C	T	synonymous
154730	NTHI477_RS00730	281	A	G	nonsynonymous
154740	NTHI477_RS00730	291	A	G	synonymous
154741	NTHI477_RS00730	292	C	T	nonsynonymous
154756	NTHI477_RS00730	307	G	C	nonsynonymous
154767	NTHI477_RS00730	318	A	T	synonymous
154767	NTHI477_RS00730	318	A	C	synonymous
154770	NTHI477_RS00730	321	T	C	synonymous
154791	NTHI477_RS00730	342	T	C	synonymous
154794	NTHI477_RS00730	345	C	T	synonymous
154795	NTHI477_RS00730	346	C	A	synonymous
154800	NTHI477_RS00730	351	C	T	synonymous
154809	NTHI477_RS00730	360	C	T	synonymous
154821	NTHI477_RS00730	372	A	G	synonymous

154838	NTHI477_RS00730	389	G	A	nonsynonymous
154839	NTHI477_RS00730	390	C	T	synonymous
154848	NTHI477_RS00730	399	G	T	synonymous
154851	NTHI477_RS00730	402	C	T	synonymous
154857	NTHI477_RS00730	408	C	T	synonymous
154866	NTHI477_RS00730	417	G	A	synonymous
154869	NTHI477_RS00730	420	A	G	synonymous
154872	NTHI477_RS00730	423	T	C	synonymous
154905	NTHI477_RS00730	456	G	A	synonymous
154935	NTHI477_RS00730	486	G	A	synonymous
154938	intergenic	intergenic	A	G	intergenic
154939	intergenic	intergenic	G	A	intergenic
154972	intergenic	intergenic	A	T	intergenic
154989	intergenic	intergenic	T	A	intergenic
165418	NTHI477_RS00790	231	G	A	synonymous
165448	NTHI477_RS00790	261	C	A	synonymous
165469	NTHI477_RS00790	282	T	G	synonymous
165471	NTHI477_RS00790	284	G	A	nonsynonymous
165472	NTHI477_RS00790	285	C	T	synonymous
165481	NTHI477_RS00790	294	C	T	synonymous
165482	NTHI477_RS00790	295	A	G	nonsynonymous
165487	NTHI477_RS00790	300	C	A	synonymous
165493	NTHI477_RS00790	306	C	T	synonymous
165511	NTHI477_RS00790	324	C	T	synonymous
165517	NTHI477_RS00790	330	G	A	synonymous
165520	NTHI477_RS00790	333	T	C	synonymous
165523	NTHI477_RS00790	336	T	C	synonymous
165527	NTHI477_RS00790	340	C	T	nonsynonymous
165529	NTHI477_RS00790	342	T	A	synonymous
165535	NTHI477_RS00790	348	T	C	synonymous
165541	NTHI477_RS00790	354	A	C	nonsynonymous
165541	NTHI477_RS00790	354	A	T	nonsynonymous
165544	NTHI477_RS00790	357	C	T	synonymous
165553	NTHI477_RS00790	366	G	A	synonymous
165559	NTHI477_RS00790	372	G	A	synonymous
165568	NTHI477_RS00790	381	T	G	nonsynonymous
165570	NTHI477_RS00790	383	G	A	nonsynonymous
165574	NTHI477_RS00790	387	A	T	synonymous
165587	NTHI477_RS00790	400	C	T	synonymous
165589	NTHI477_RS00790	402	G	A	synonymous
165595	NTHI477_RS00790	408	C	T	synonymous
165638	NTHI477_RS00790	451	T	C	nonsynonymous
165665	intergenic	intergenic	G	A	intergenic
165673	intergenic	intergenic	C	T	intergenic
165687	intergenic	intergenic	C	T	intergenic

165687	intergenic	intergenic	C	A	intergenic
165698	intergenic	intergenic	T	A	intergenic
165699	intergenic	intergenic	C	A	intergenic
165700	intergenic	intergenic	T	A	intergenic
165708	intergenic	intergenic	T	C	intergenic
165718	intergenic	intergenic	C	T	intergenic
165741	NTHI477_RS00795	23	C	T	nonsynonymous
165754	NTHI477_RS00795	36	A	G	synonymous
165758	NTHI477_RS00795	40	C	A	nonsynonymous
165775	NTHI477_RS00795	57	T	C	synonymous
165778	NTHI477_RS00795	60	T	C	synonymous
165784	NTHI477_RS00795	66	C	A	synonymous
165785	NTHI477_RS00795	67	A	G	nonsynonymous
165791	NTHI477_RS00795	73	T	C	nonsynonymous
165793	NTHI477_RS00795	75	T	C	synonymous
165794	NTHI477_RS00795	76	C	T	synonymous
165802	NTHI477_RS00795	84	C	T	synonymous
165805	NTHI477_RS00795	87	T	G	synonymous
165811	NTHI477_RS00795	93	A	C	synonymous
165823	NTHI477_RS00795	105	T	C	synonymous
165824	NTHI477_RS00795	106	T	G	nonsynonymous
165827	NTHI477_RS00795	109	C	T	synonymous
165829	NTHI477_RS00795	111	G	A	synonymous
165835	NTHI477_RS00795	117	G	C	synonymous
165838	NTHI477_RS00795	120	A	G	synonymous
165841	NTHI477_RS00795	123	G	A	synonymous
165845	NTHI477_RS00795	127	A	C	nonsynonymous
165847	NTHI477_RS00795	129	C	T	synonymous
165850	NTHI477_RS00795	132	T	C	synonymous
165854	NTHI477_RS00795	136	A	C	nonsynonymous
165859	NTHI477_RS00795	141	G	A	synonymous
165862	NTHI477_RS00795	144	A	C	nonsynonymous
165863	NTHI477_RS00795	145	A	G	nonsynonymous
165864	NTHI477_RS00795	146	A	G	nonsynonymous
165865	NTHI477_RS00795	147	T	G	nonsynonymous
165865	NTHI477_RS00795	147	T	C	synonymous
165867	NTHI477_RS00795	149	C	T	nonsynonymous
165869	NTHI477_RS00795	151	A	T	nonsynonymous
165869	NTHI477_RS00795	151	A	G	nonsynonymous
165870	NTHI477_RS00795	152	A	C	nonsynonymous
165871	NTHI477_RS00795	153	G	T	nonsynonymous
165887	NTHI477_RS00795	169	C	T	synonymous
165895	NTHI477_RS00795	177	A	G	synonymous
165901	NTHI477_RS00795	183	G	A	synonymous
165904	NTHI477_RS00795	186	A	C	synonymous

165908	NTHI477_RS00795	190	A	G	nonsynonymous
165919	NTHI477_RS00795	201	T	C	synonymous
165931	NTHI477_RS00795	213	T	G	synonymous
165934	NTHI477_RS00795	216	T	C	synonymous
165934	NTHI477_RS00795	216	T	A	synonymous
165935	NTHI477_RS00795	217	G	A	nonsynonymous
165937	NTHI477_RS00795	219	A	G	synonymous
165946	NTHI477_RS00795	228	C	T	synonymous
165964	NTHI477_RS00795	246	C	T	synonymous
165979	NTHI477_RS00795	261	G	A	synonymous
165985	NTHI477_RS00795	267	C	T	synonymous
166012	NTHI477_RS00795	294	A	G	synonymous
166015	NTHI477_RS00795	297	C	T	synonymous
166021	NTHI477_RS00795	303	T	C	synonymous
166022	NTHI477_RS00795	304	G	T	nonsynonymous
166032	NTHI477_RS00795	314	C	T	nonsynonymous
166033	NTHI477_RS00795	315	C	G	synonymous
166057	NTHI477_RS00795	339	G	C	nonsynonymous
166081	NTHI477_RS00795	363	C	T	synonymous
166087	NTHI477_RS00795	369	G	T	synonymous
166091	NTHI477_RS00795	373	A	G	nonsynonymous
166093	NTHI477_RS00795	375	T	C	synonymous
166094	NTHI477_RS00795	376	G	T	nonsynonymous
166095	NTHI477_RS00795	377	C	T	nonsynonymous
166096	NTHI477_RS00795	378	T	A	synonymous
166102	NTHI477_RS00795	384	A	G	synonymous
166105	NTHI477_RS00795	387	T	C	synonymous
166109	NTHI477_RS00795	391	G	A	nonsynonymous
166111	NTHI477_RS00795	393	C	T	synonymous
166132	NTHI477_RS00795	414	C	T	synonymous
166132	NTHI477_RS00795	414	C	A	synonymous
166148	NTHI477_RS00795	430	T	C	nonsynonymous
166150	NTHI477_RS00795	432	A	T	synonymous
166153	NTHI477_RS00795	435	C	A	synonymous
166162	NTHI477_RS00795	444	A	G	synonymous
166165	NTHI477_RS00795	447	A	G	synonymous
166177	NTHI477_RS00795	459	T	C	synonymous
166258	NTHI477_RS00795	540	T	C	synonymous
166259	NTHI477_RS00795	541	G	A	nonsynonymous
166281	NTHI477_RS00795	563	C	A	nonsynonymous
166291	NTHI477_RS00795	573	T	C	synonymous
166298	NTHI477_RS00795	580	G	A	nonsynonymous
166306	NTHI477_RS00795	588	C	T	synonymous
166314	NTHI477_RS00795	596	A	C	nonsynonymous
166315	NTHI477_RS00795	597	C	A	nonsynonymous

166321	NTHI477_RS00795	603	G	A	synonymous
166321	NTHI477_RS00795	603	G	T	nonsynonymous
166354	NTHI477_RS00795	636	A	G	synonymous
166381	NTHI477_RS00795	663	T	C	synonymous
166408	NTHI477_RS00795	690	C	T	synonymous
166411	NTHI477_RS00795	693	A	G	synonymous
166414	NTHI477_RS00795	696	G	T	nonsynonymous
166414	NTHI477_RS00795	696	G	A	synonymous
166419	NTHI477_RS00795	701	G	A	nonsynonymous
166420	NTHI477_RS00795	702	T	A	nonsynonymous
166428	NTHI477_RS00795	710	G	C	nonsynonymous
166435	NTHI477_RS00795	717	T	A	synonymous
166447	NTHI477_RS00795	729	G	A	synonymous
166457	NTHI477_RS00795	739	T	C	synonymous
166471	NTHI477_RS00795	753	C	T	synonymous
166474	NTHI477_RS00795	756	T	C	synonymous
166480	NTHI477_RS00795	762	C	T	synonymous
166480	NTHI477_RS00795	762	C	A	nonsynonymous
166482	NTHI477_RS00795	764	T	C	nonsynonymous
166483	NTHI477_RS00795	765	A	G	synonymous
166510	NTHI477_RS00795	792	G	A	synonymous
166519	NTHI477_RS00795	801	G	C	synonymous
166524	NTHI477_RS00795	806	A	G	nonsynonymous
166567	intergenic	intergenic	T	C	intergenic
166568	intergenic	intergenic	C	T	intergenic
166570	intergenic	intergenic	T	C	intergenic
166571	intergenic	intergenic	G	A	intergenic
166572	intergenic	intergenic	G	A	intergenic
166574	intergenic	intergenic	A	G	intergenic
166579	intergenic	intergenic	G	T	intergenic
166581	intergenic	intergenic	T	C	intergenic
166586	intergenic	intergenic	T	C	intergenic
166589	intergenic	intergenic	C	T	intergenic
166590	intergenic	intergenic	G	A	intergenic
166684	NTHI477_RS00800	21	A	G	synonymous
166719	NTHI477_RS00800	56	A	G	nonsynonymous
166759	NTHI477_RS00800	96	T	G	synonymous
166765	NTHI477_RS00800	102	G	A	synonymous
166771	NTHI477_RS00800	108	G	A	synonymous
166807	NTHI477_RS00800	144	A	G	synonymous
166816	NTHI477_RS00800	153	G	A	synonymous
166846	NTHI477_RS00800	183	A	T	synonymous
166849	NTHI477_RS00800	186	G	A	synonymous
166855	NTHI477_RS00800	192	G	T	synonymous
166861	NTHI477_RS00800	198	T	C	synonymous

166864	NTHI477_RS00800	201	C	T	synonymous
166939	NTHI477_RS00800	276	G	C	synonymous
166948	NTHI477_RS00800	285	G	A	synonymous
166960	NTHI477_RS00800	297	C	T	synonymous
166963	NTHI477_RS00800	300	C	T	synonymous
166966	NTHI477_RS00800	303	T	A	synonymous
167002	NTHI477_RS00800	339	C	T	synonymous
167038	NTHI477_RS00800	375	G	A	synonymous
167059	NTHI477_RS00800	396	C	T	synonymous
167077	NTHI477_RS00800	414	T	C	synonymous
167086	NTHI477_RS00800	423	C	T	synonymous
167089	NTHI477_RS00800	426	T	C	synonymous
167092	NTHI477_RS00800	429	A	T	nonsynonymous
167098	NTHI477_RS00800	435	A	G	synonymous
167098	NTHI477_RS00800	435	A	C	synonymous
167099	NTHI477_RS00800	436	T	C	synonymous
167134	NTHI477_RS00800	471	A	T	synonymous
167137	NTHI477_RS00800	474	G	C	synonymous
167167	NTHI477_RS00800	504	C	T	synonymous
167191	NTHI477_RS00800	528	A	G	synonymous
167191	NTHI477_RS00800	528	A	T	synonymous
167223	intergenic	intergenic	C	T	intergenic
167224	intergenic	intergenic	T	C	intergenic
167278	intergenic	intergenic	A	G	intergenic
167301	intergenic	intergenic	C	T	intergenic
167314	intergenic	intergenic	A	G	intergenic
167341	intergenic	intergenic	A	G	intergenic
167363	intergenic	intergenic	A	T	intergenic
167835	NTHI477_RS00810	156	A	G	synonymous
167895	NTHI477_RS00810	216	C	T	synonymous
167907	NTHI477_RS00810	228	C	T	synonymous
167916	NTHI477_RS00810	237	C	T	synonymous
167925	NTHI477_RS00810	246	C	A	synonymous
167982	NTHI477_RS00810	303	C	T	synonymous
167997	NTHI477_RS00810	318	T	C	synonymous
168091	intergenic	intergenic	T	C	intergenic
168094	intergenic	intergenic	G	A	intergenic
168094	intergenic	intergenic	G	T	intergenic
168096	intergenic	intergenic	G	A	intergenic
168101	intergenic	intergenic	C	T	intergenic
168116	intergenic	intergenic	G	A	intergenic
168119	intergenic	intergenic	G	A	intergenic
168151	NTHI477_RS00815	6	C	T	synonymous
168166	NTHI477_RS00815	21	G	A	synonymous
168172	NTHI477_RS00815	27	C	T	synonymous

168179	NTHI477_RS00815	34	C	T	synonymous
168181	NTHI477_RS00815	36	A	G	synonymous
168211	NTHI477_RS00815	66	A	G	synonymous
168214	NTHI477_RS00815	69	T	C	synonymous
168223	NTHI477_RS00815	78	G	A	synonymous
168226	NTHI477_RS00815	81	A	G	synonymous
168253	NTHI477_RS00815	108	T	C	synonymous
168277	NTHI477_RS00815	132	A	G	synonymous
168286	NTHI477_RS00815	141	T	C	synonymous
168292	NTHI477_RS00815	147	A	T	synonymous
168298	NTHI477_RS00815	153	A	G	synonymous
168328	NTHI477_RS00815	183	C	A	synonymous
168343	NTHI477_RS00815	198	A	G	synonymous
168349	NTHI477_RS00815	204	A	G	synonymous
168355	NTHI477_RS00815	210	C	T	synonymous
168392	NTHI477_RS00815	247	A	G	nonsynonymous
168402	NTHI477_RS00815	257	C	T	nonsynonymous
168412	NTHI477_RS00815	267	T	A	synonymous
168413	NTHI477_RS00815	268	A	G	nonsynonymous
168422	NTHI477_RS00815	277	G	A	nonsynonymous
168431	NTHI477_RS00815	286	G	T	nonsynonymous
168465	NTHI477_RS00815	320	G	C	nonsynonymous
168466	NTHI477_RS00815	321	T	C	synonymous
168467	NTHI477_RS00815	322	G	C	nonsynonymous
168469	NTHI477_RS00815	324	T	G	nonsynonymous
168470	NTHI477_RS00815	325	A	G	nonsynonymous
168471	NTHI477_RS00815	326	A	G	nonsynonymous
168475	NTHI477_RS00815	330	A	G	synonymous
168499	NTHI477_RS00815	354	C	T	synonymous
168502	NTHI477_RS00815	357	A	G	synonymous
168505	NTHI477_RS00815	360	G	A	synonymous
168514	NTHI477_RS00815	369	A	G	synonymous
168527	NTHI477_RS00815	382	C	T	synonymous
168529	NTHI477_RS00815	384	G	A	synonymous
168544	NTHI477_RS00815	399	C	T	synonymous
168550	NTHI477_RS00815	405	C	T	synonymous
168553	NTHI477_RS00815	408	T	C	synonymous
168558	NTHI477_RS00815	413	G	T	nonsynonymous
168569	NTHI477_RS00815	424	T	C	synonymous
168586	NTHI477_RS00815	441	C	T	synonymous
168592	NTHI477_RS00815	447	T	A	synonymous
168601	NTHI477_RS00815	456	T	C	synonymous
168619	NTHI477_RS00815	474	A	T	synonymous
168634	NTHI477_RS00815	489	T	C	synonymous
168637	NTHI477_RS00815	492	A	G	synonymous

168640	NTHI477_RS00815	495	A	G	synonymous
168667	NTHI477_RS00815	522	A	C	synonymous
168685	NTHI477_RS00815	540	G	A	synonymous
168698	NTHI477_RS00815	553	G	T	nonsynonymous
168709	NTHI477_RS00815	564	C	T	synonymous
168727	NTHI477_RS00815	582	A	G	synonymous
168730	NTHI477_RS00815	585	A	T	synonymous
168730	NTHI477_RS00815	585	A	G	synonymous
168732	NTHI477_RS00815	587	C	A	nonsynonymous
168736	NTHI477_RS00815	591	G	T	nonsynonymous
168736	NTHI477_RS00815	591	G	A	synonymous
168742	NTHI477_RS00815	597	G	A	synonymous
168751	NTHI477_RS00815	606	G	A	synonymous
168775	NTHI477_RS00815	630	G	A	synonymous
168781	NTHI477_RS00815	636	G	T	synonymous
168784	NTHI477_RS00815	639	G	A	synonymous
168799	NTHI477_RS00815	654	G	A	synonymous
168812	NTHI477_RS00815	667	C	T	nonsynonymous
168814	NTHI477_RS00815	669	A	T	synonymous
168817	NTHI477_RS00815	672	A	G	nonsynonymous
168818	NTHI477_RS00815	673	C	A	nonsynonymous
168823	NTHI477_RS00815	678	C	T	synonymous
168831	NTHI477_RS00815	686	C	A	nonsynonymous
168848	NTHI477_RS00815	703	T	C	nonsynonymous
168849	NTHI477_RS00815	704	A	G	nonsynonymous
168866	NTHI477_RS00815	721	G	A	nonsynonymous
168875	NTHI477_RS00815	730	G	A	nonsynonymous
168877	NTHI477_RS00815	732	A	C	synonymous
168886	NTHI477_RS00815	741	A	G	synonymous
168887	NTHI477_RS00815	742	G	A	nonsynonymous
168891	NTHI477_RS00815	746	G	A	nonsynonymous
168893	NTHI477_RS00815	748	G	T	nonsynonymous
168896	NTHI477_RS00815	751	G	T	nonsynonymous
168906	NTHI477_RS00815	761	T	C	nonsynonymous
168908	NTHI477_RS00815	763	G	C	nonsynonymous
168909	NTHI477_RS00815	764	C	T	nonsynonymous
168919	NTHI477_RS00815	774	C	A	synonymous
168925	NTHI477_RS00815	780	A	G	synonymous
168926	NTHI477_RS00815	781	C	T	synonymous
168930	NTHI477_RS00815	785	A	T	nonsynonymous
168931	NTHI477_RS00815	786	G	T	nonsynonymous
168996	NTHI477_RS00815	851	G	A	nonsynonymous
169015	NTHI477_RS00815	870	C	T	synonymous
169034	NTHI477_RS00815	889	C	T	nonsynonymous
169060	NTHI477_RS00815	915	A	T	synonymous

169069	NTHI477_RS00815	924	C	G	nonsynonymous
169075	NTHI477_RS00815	930	C	T	synonymous
169101	NTHI477_RS00815	956	G	A	nonsynonymous
169109	NTHI477_RS00815	964	T	C	nonsynonymous
169110	NTHI477_RS00815	965	C	A	nonsynonymous
169114	NTHI477_RS00815	969	G	T	synonymous
169136	NTHI477_RS00815	991	C	A	nonsynonymous
169138	NTHI477_RS00815	993	C	A	synonymous
169138	NTHI477_RS00815	993	C	G	synonymous
169143	NTHI477_RS00815	998	C	T	nonsynonymous
169154	NTHI477_RS00815	1009	T	G	nonsynonymous
169156	NTHI477_RS00815	1011	C	A	synonymous
169158	NTHI477_RS00815	1013	C	T	nonsynonymous
169167	NTHI477_RS00815	1022	C	A	nonsynonymous
169183	NTHI477_RS00815	1038	C	A	synonymous
169192	NTHI477_RS00815	1047	C	T	synonymous
169199	NTHI477_RS00815	1054	A	T	nonsynonymous
169201	NTHI477_RS00815	1056	A	C	synonymous
169213	NTHI477_RS00815	1068	A	C	synonymous
169214	NTHI477_RS00815	1069	A	C	nonsynonymous
169218	NTHI477_RS00815	1073	A	G	nonsynonymous
169220	NTHI477_RS00815	1075	C	A	nonsynonymous
169224	NTHI477_RS00815	1079	C	T	nonsynonymous
169240	NTHI477_RS00815	1095	A	T	synonymous
169240	NTHI477_RS00815	1095	A	C	synonymous
169246	NTHI477_RS00815	1101	C	G	synonymous
169246	NTHI477_RS00815	1101	C	T	synonymous
169247	NTHI477_RS00815	1102	T	C	synonymous
169264	NTHI477_RS00815	1119	A	G	synonymous
169267	NTHI477_RS00815	1122	C	T	synonymous
169276	NTHI477_RS00815	1131	A	T	synonymous
169282	NTHI477_RS00815	1137	T	C	synonymous
169303	NTHI477_RS00815	1158	T	C	synonymous
169312	NTHI477_RS00815	1167	T	C	synonymous
169316	NTHI477_RS00815	1171	C	T	nonsynonymous
169321	NTHI477_RS00815	1176	T	C	synonymous
169328	NTHI477_RS00815	1183	C	T	synonymous
169335	NTHI477_RS00815	1190	A	G	nonsynonymous
169336	NTHI477_RS00815	1191	T	C	synonymous
169339	NTHI477_RS00815	1194	G	A	synonymous
169345	NTHI477_RS00815	1200	G	A	synonymous
169369	NTHI477_RS00815	1224	T	C	synonymous
169384	NTHI477_RS00815	1239	T	C	synonymous
169411	NTHI477_RS00815	1266	G	A	synonymous
169418	NTHI477_RS00815	1273	C	A	nonsynonymous

169423	NTHI477_RS00815	1278	A	G	synonymous
169456	NTHI477_RS00815	1311	T	C	synonymous
169489	NTHI477_RS00815	1344	T	C	synonymous
169498	NTHI477_RS00815	1353	G	A	synonymous
169522	NTHI477_RS00815	1377	C	T	synonymous
169549	NTHI477_RS00815	1404	C	A	synonymous
169552	NTHI477_RS00815	1407	A	G	synonymous
169555	NTHI477_RS00815	1410	C	T	synonymous
169561	NTHI477_RS00815	1416	C	T	synonymous
169561	NTHI477_RS00815	1416	C	A	synonymous
169576	NTHI477_RS00815	1431	A	G	synonymous
169618	NTHI477_RS00815	1473	T	C	synonymous
169627	NTHI477_RS00815	1482	T	G	synonymous
169630	NTHI477_RS00815	1485	A	G	synonymous
169633	NTHI477_RS00815	1488	C	T	synonymous
169646	NTHI477_RS00815	1501	T	C	nonsynonymous
169647	NTHI477_RS00815	1502	C	T	nonsynonymous
169648	NTHI477_RS00815	1503	G	A	synonymous
169670	NTHI477_RS00815	1525	A	G	nonsynonymous
169672	NTHI477_RS00815	1527	A	C	nonsynonymous
169681	NTHI477_RS00815	1536	T	G	synonymous
169705	NTHI477_RS00815	1560	G	T	synonymous
169705	NTHI477_RS00815	1560	G	A	synonymous
169723	NTHI477_RS00815	1578	C	T	synonymous
169728	NTHI477_RS00815	1583	C	T	nonsynonymous
169729	NTHI477_RS00815	1584	C	T	synonymous
169735	NTHI477_RS00815	1590	A	T	synonymous
169765	NTHI477_RS00815	1620	C	T	synonymous
169801	NTHI477_RS00815	1656	T	G	synonymous
169807	NTHI477_RS00815	1662	T	C	synonymous
169833	NTHI477_RS00815	1688	C	T	nonsynonymous
169834	NTHI477_RS00815	1689	T	A	synonymous
169858	NTHI477_RS00815	1713	T	C	synonymous
169860	NTHI477_RS00815	1715	A	C	nonsynonymous
169867	NTHI477_RS00815	1722	T	C	synonymous
169868	NTHI477_RS00815	1723	C	T	nonsynonymous
169869	NTHI477_RS00815	1724	G	A	nonsynonymous
169876	NTHI477_RS00815	1731	A	C	synonymous
169879	NTHI477_RS00815	1734	G	A	synonymous
169888	NTHI477_RS00815	1743	A	G	synonymous
169897	NTHI477_RS00815	1752	C	T	synonymous
169901	NTHI477_RS00815	1756	T	C	synonymous
169903	NTHI477_RS00815	1758	A	G	synonymous
169903	NTHI477_RS00815	1758	A	T	nonsynonymous
169906	NTHI477_RS00815	1761	G	A	synonymous

169907	NTHI477_RS00815	1762	C	A	nonsynonymous
169908	NTHI477_RS00815	1763	G	A	nonsynonymous
169909	NTHI477_RS00815	1764	T	A	synonymous
169910	NTHI477_RS00815	1765	G	A	nonsynonymous
169913	NTHI477_RS00815	1768	A	C	nonsynonymous
169914	NTHI477_RS00815	1769	A	G	nonsynonymous
169915	NTHI477_RS00815	1770	A	T	nonsynonymous
169916	NTHI477_RS00815	1771	A	T	nonsynonymous
169924	NTHI477_RS00815	1779	G	T	synonymous
169927	NTHI477_RS00815	1782	T	G	synonymous
169928	NTHI477_RS00815	1783	T	G	nonsynonymous
169929	NTHI477_RS00815	1784	T	A	nonsynonymous
169930	NTHI477_RS00815	1785	A	T	nonsynonymous
169933	NTHI477_RS00815	1788	A	C	synonymous
169935	NTHI477_RS00815	1790	A	T	nonsynonymous
169936	NTHI477_RS00815	1791	A	T	nonsynonymous
169937	NTHI477_RS00815	1792	A	C	nonsynonymous
169938	NTHI477_RS00815	1793	A	G	nonsynonymous
169939	NTHI477_RS00815	1794	T	A	nonsynonymous
169942	NTHI477_RS00815	1797	A	G	synonymous
169943	NTHI477_RS00815	1798	T	C	synonymous
169945	NTHI477_RS00815	1800	G	A	synonymous
169951	NTHI477_RS00815	1806	A	G	synonymous
169952	NTHI477_RS00815	1807	T	C	synonymous
169954	NTHI477_RS00815	1809	A	G	synonymous
169954	NTHI477_RS00815	1809	A	T	nonsynonymous
169957	NTHI477_RS00815	1812	G	A	synonymous
169957	NTHI477_RS00815	1812	G	T	synonymous
169960	NTHI477_RS00815	1815	C	T	synonymous
169963	NTHI477_RS00815	1818	G	C	synonymous
169966	NTHI477_RS00815	1821	A	G	synonymous
169978	NTHI477_RS00815	1833	T	A	synonymous
169986	NTHI477_RS00815	1841	A	G	nonsynonymous
169990	NTHI477_RS00815	1845	C	T	synonymous
169993	NTHI477_RS00815	1848	A	T	synonymous
170003	NTHI477_RS00815	1858	A	C	nonsynonymous
170008	NTHI477_RS00815	1863	T	A	synonymous
170011	NTHI477_RS00815	1866	G	A	synonymous
170012	NTHI477_RS00815	1867	A	G	nonsynonymous
170013	NTHI477_RS00815	1868	A	T	nonsynonymous
170014	NTHI477_RS00815	1869	A	G	synonymous
170017	NTHI477_RS00815	1872	A	G	synonymous
170024	NTHI477_RS00815	1879	T	A	nonsynonymous
170026	NTHI477_RS00815	1881	G	A	nonsynonymous
170027	NTHI477_RS00815	1882	G	A	nonsynonymous

170029	NTHI477_RS00815	1884	G	A	synonymous
170029	NTHI477_RS00815	1884	G	T	synonymous
170030	NTHI477_RS00815	1885	C	T	nonsynonymous
170032	NTHI477_RS00815	1887	T	A	synonymous
170038	NTHI477_RS00815	1893	C	T	synonymous
170044	NTHI477_RS00815	1899	G	A	synonymous
170050	NTHI477_RS00815	1905	G	T	synonymous
170053	NTHI477_RS00815	1908	A	T	synonymous
170056	NTHI477_RS00815	1911	A	G	synonymous
170059	NTHI477_RS00815	1914	T	C	synonymous
170071	NTHI477_RS00815	1926	C	T	synonymous
170074	NTHI477_RS00815	1929	G	A	synonymous
170085	NTHI477_RS00815	1940	C	A	nonsynonymous
170134	NTHI477_RS00815	1989	A	G	synonymous
170140	NTHI477_RS00815	1995	T	C	synonymous
170155	NTHI477_RS00815	2010	A	T	synonymous
170161	NTHI477_RS00815	2016	T	C	synonymous
170161	NTHI477_RS00815	2016	T	A	nonsynonymous
170205	NTHI477_RS00815	2060	C	T	nonsynonymous
170206	NTHI477_RS00815	2061	A	C	synonymous
170210	NTHI477_RS00815	2065	T	C	synonymous
170215	NTHI477_RS00815	2070	T	C	synonymous
170230	NTHI477_RS00815	2085	G	A	synonymous
170245	NTHI477_RS00815	2100	A	G	synonymous
170248	NTHI477_RS00815	2103	C	A	synonymous
170263	NTHI477_RS00815	2118	C	A	synonymous
170269	NTHI477_RS00815	2124	C	A	synonymous
170273	NTHI477_RS00815	2128	C	A	synonymous
170308	NTHI477_RS00815	2163	T	G	nonsynonymous
170320	NTHI477_RS00815	2175	T	A	synonymous
170326	NTHI477_RS00815	2181	G	A	synonymous
170327	NTHI477_RS00815	2182	C	T	synonymous
170342	NTHI477_RS00815	2197	T	G	nonsynonymous
170344	NTHI477_RS00815	2199	G	T	synonymous
170347	NTHI477_RS00815	2202	A	C	synonymous
170380	NTHI477_RS00815	2235	C	T	synonymous
170386	NTHI477_RS00815	2241	T	G	synonymous
170416	NTHI477_RS00815	2271	T	C	synonymous
170422	NTHI477_RS00815	2277	C	T	synonymous
170425	NTHI477_RS00815	2280	G	A	synonymous
170428	NTHI477_RS00815	2283	G	A	synonymous
170431	NTHI477_RS00815	2286	A	G	synonymous
170437	NTHI477_RS00815	2292	A	T	synonymous
170437	NTHI477_RS00815	2292	A	C	synonymous
170442	NTHI477_RS00815	2297	C	G	nonsynonymous

170443	NTHI477_RS00815	2298	A	T	synonymous
170455	NTHI477_RS00815	2310	T	C	synonymous
170470	NTHI477_RS00815	2325	T	C	synonymous
170497	NTHI477_RS00815	2352	T	G	synonymous
170499	NTHI477_RS00815	2354	T	C	nonsynonymous
170500	NTHI477_RS00815	2355	G	A	synonymous
170503	NTHI477_RS00815	2358	G	A	synonymous
170506	NTHI477_RS00815	2361	C	T	synonymous
170510	NTHI477_RS00815	2365	G	A	nonsynonymous
170511	NTHI477_RS00815	2366	A	T	nonsynonymous
170517	NTHI477_RS00815	2372	G	C	nonsynonymous
170518	NTHI477_RS00815	2373	T	G	nonsynonymous
170519	NTHI477_RS00815	2374	A	G	nonsynonymous
170520	NTHI477_RS00815	2375	A	C	nonsynonymous
170521	NTHI477_RS00815	2376	A	T	nonsynonymous
170522	NTHI477_RS00815	2377	A	G	nonsynonymous
170523	NTHI477_RS00815	2378	A	G	nonsynonymous
170524	NTHI477_RS00815	2379	A	T	nonsynonymous
170525	NTHI477_RS00815	2380	G	T	nonsynonymous
170530	NTHI477_RS00815	2385	C	G	nonsynonymous
170534	NTHI477_RS00815	2389	A	G	nonsynonymous
170555	NTHI477_RS00815	2410	G	A	nonsynonymous
170575	NTHI477_RS00815	2430	A	G	nonsynonymous
170584	NTHI477_RS00815	2439	C	T	synonymous
170593	NTHI477_RS00815	2448	C	A	nonsynonymous
170596	NTHI477_RS00815	2451	T	A	synonymous
170601	NTHI477_RS00815	2456	G	A	nonsynonymous
170617	NTHI477_RS00815	2472	T	G	synonymous
170620	NTHI477_RS00815	2475	A	G	synonymous
170623	NTHI477_RS00815	2478	A	G	synonymous
170641	NTHI477_RS00815	2496	G	A	synonymous
170650	NTHI477_RS00815	2505	A	G	synonymous
170650	NTHI477_RS00815	2505	A	C	synonymous
170654	NTHI477_RS00815	2509	G	T	nonsynonymous
170675	NTHI477_RS00815	2530	G	A	nonsynonymous
170677	NTHI477_RS00815	2532	G	T	synonymous
170680	NTHI477_RS00815	2535	G	A	synonymous
170684	NTHI477_RS00815	2539	G	A	nonsynonymous
170686	NTHI477_RS00815	2541	A	G	synonymous
170713	NTHI477_RS00815	2568	A	G	synonymous
170723	NTHI477_RS00815	2578	T	C	synonymous
170753	NTHI477_RS00815	2608	A	G	nonsynonymous
170754	NTHI477_RS00815	2609	A	C	nonsynonymous
170755	NTHI477_RS00815	2610	T	G	nonsynonymous
170755	NTHI477_RS00815	2610	T	A	nonsynonymous

170758	NTHI477_RS00815	2613	G	A	synonymous
170761	NTHI477_RS00815	2616	G	A	synonymous
170762	NTHI477_RS00815	2617	A	G	nonsynonymous
170763	NTHI477_RS00815	2618	A	G	nonsynonymous
170763	NTHI477_RS00815	2618	A	C	nonsynonymous
170764	NTHI477_RS00815	2619	T	G	nonsynonymous
170765	NTHI477_RS00815	2620	T	C	nonsynonymous
170779	NTHI477_RS00815	2634	G	A	synonymous
170782	NTHI477_RS00815	2637	G	A	synonymous
170792	NTHI477_RS00815	2647	G	A	nonsynonymous
170805	NTHI477_RS00815	2660	A	C	nonsynonymous
170811	NTHI477_RS00815	2666	G	A	nonsynonymous
170831	NTHI477_RS00815	2686	A	C	nonsynonymous
170836	NTHI477_RS00815	2691	G	A	synonymous
170838	NTHI477_RS00815	2693	C	A	nonsynonymous
170846	NTHI477_RS00815	2701	G	T	nonsynonymous
170850	NTHI477_RS00815	2705	C	A	nonsynonymous
170854	NTHI477_RS00815	2709	T	G	nonsynonymous
170855	NTHI477_RS00815	2710	C	A	nonsynonymous
170856	NTHI477_RS00815	2711	C	A	nonsynonymous
170858	NTHI477_RS00815	2713	G	A	nonsynonymous
170860	NTHI477_RS00815	2715	T	C	synonymous
170866	NTHI477_RS00815	2721	T	A	synonymous
170869	NTHI477_RS00815	2724	C	T	synonymous
170874	NTHI477_RS00815	2729	C	T	nonsynonymous
170887	NTHI477_RS00815	2742	G	T	nonsynonymous
170888	NTHI477_RS00815	2743	C	T	nonsynonymous
170897	NTHI477_RS00815	2752	G	T	nonsynonymous
170961	NTHI477_RS00815	2816	G	C	nonsynonymous
170962	NTHI477_RS00815	2817	T	A	synonymous
170962	NTHI477_RS00815	2817	T	G	synonymous
170971	NTHI477_RS00815	2826	A	C	synonymous
170974	NTHI477_RS00815	2829	A	G	synonymous
170975	NTHI477_RS00815	2830	A	C	nonsynonymous
170984	NTHI477_RS00815	2839	G	A	nonsynonymous
170997	NTHI477_RS00815	2852	A	G	nonsynonymous
170998	NTHI477_RS00815	2853	G	T	nonsynonymous
171022	NTHI477_RS00815	2877	C	T	synonymous
171024	NTHI477_RS00815	2879	C	T	nonsynonymous
171040	NTHI477_RS00815	2895	T	C	synonymous
171047	NTHI477_RS00815	2902	G	T	nonsynonymous
171049	NTHI477_RS00815	2904	T	G	synonymous
171058	NTHI477_RS00815	2913	A	T	synonymous
171058	NTHI477_RS00815	2913	A	G	nonsynonymous
171061	NTHI477_RS00815	2916	C	G	synonymous

171081	NTHI477_RS00815	2936	A	G	nonsynonymous
171082	NTHI477_RS00815	2937	T	C	synonymous
171090	NTHI477_RS00815	2945	A	G	nonsynonymous
171091	NTHI477_RS00815	2946	T	C	synonymous
171105	NTHI477_RS00815	2960	C	T	nonsynonymous
171127	NTHI477_RS00815	2982	T	A	synonymous
171127	NTHI477_RS00815	2982	T	G	synonymous
171166	NTHI477_RS00815	3021	T	C	synonymous
171169	NTHI477_RS00815	3024	A	G	synonymous
171177	NTHI477_RS00815	3032	T	C	nonsynonymous
171190	NTHI477_RS00815	3045	C	G	nonsynonymous
171197	NTHI477_RS00815	3052	A	C	synonymous
171223	NTHI477_RS00815	3078	T	C	synonymous
171232	NTHI477_RS00815	3087	G	A	synonymous
171233	NTHI477_RS00815	3088	C	T	nonsynonymous
171268	NTHI477_RS00815	3123	G	A	synonymous
171278	NTHI477_RS00815	3133	C	A	nonsynonymous
171307	NTHI477_RS00815	3162	G	A	synonymous
171344	NTHI477_RS00815	3199	C	T	synonymous
171349	NTHI477_RS00815	3204	T	A	synonymous
171353	NTHI477_RS00815	3208	T	C	nonsynonymous
171353	NTHI477_RS00815	3208	T	A	nonsynonymous
171357	NTHI477_RS00815	3212	A	C	nonsynonymous
171375	NTHI477_RS00815	3230	A	C	nonsynonymous
171384	NTHI477_RS00815	3239	G	A	nonsynonymous
171385	NTHI477_RS00815	3240	C	T	synonymous
171386	NTHI477_RS00815	3241	A	G	nonsynonymous
171388	NTHI477_RS00815	3243	A	G	synonymous
171397	NTHI477_RS00815	3252	C	G	synonymous
171409	NTHI477_RS00815	3264	A	G	synonymous
171410	NTHI477_RS00815	3265	C	T	nonsynonymous
171419	NTHI477_RS00815	3274	C	T	nonsynonymous
171433	NTHI477_RS00815	3288	G	A	synonymous
171442	NTHI477_RS00815	3297	A	G	synonymous
171445	NTHI477_RS00815	3300	A	C	synonymous
171452	NTHI477_RS00815	3307	A	C	synonymous
171460	NTHI477_RS00815	3315	A	G	synonymous
171478	NTHI477_RS00815	3333	C	T	synonymous
171487	NTHI477_RS00815	3342	A	G	synonymous
171490	NTHI477_RS00815	3345	A	G	synonymous
171495	NTHI477_RS00815	3350	A	G	nonsynonymous
171500	NTHI477_RS00815	3355	A	G	nonsynonymous
171532	NTHI477_RS00815	3387	C	T	synonymous
171535	NTHI477_RS00815	3390	T	C	synonymous
171538	NTHI477_RS00815	3393	T	C	synonymous

171539	NTHI477_RS00815	3394	G	T	nonsynonymous
171540	NTHI477_RS00815	3395	A	C	nonsynonymous
171541	NTHI477_RS00815	3396	C	T	synonymous
171553	NTHI477_RS00815	3408	G	A	synonymous
171631	NTHI477_RS00815	3486	A	G	synonymous
171637	NTHI477_RS00815	3492	C	T	synonymous
171647	NTHI477_RS00815	3502	C	G	nonsynonymous
171652	NTHI477_RS00815	3507	C	T	synonymous
171660	NTHI477_RS00815	3515	C	T	nonsynonymous
171661	NTHI477_RS00815	3516	A	G	synonymous
171673	NTHI477_RS00815	3528	C	T	synonymous
171679	NTHI477_RS00815	3534	A	G	synonymous
171694	NTHI477_RS00815	3549	A	G	synonymous
171703	NTHI477_RS00815	3558	G	A	nonsynonymous
171706	NTHI477_RS00815	3561	C	T	synonymous
171709	NTHI477_RS00815	3564	T	A	synonymous
171712	NTHI477_RS00815	3567	A	G	synonymous
171733	NTHI477_RS00815	3588	C	T	synonymous
171738	NTHI477_RS00815	3593	G	A	nonsynonymous
171769	NTHI477_RS00815	3624	C	T	synonymous
171775	NTHI477_RS00815	3630	G	A	synonymous
171789	NTHI477_RS00820	9	T	C	synonymous
171798	NTHI477_RS00820	18	C	A	nonsynonymous
171812	NTHI477_RS00820	32	A	G	nonsynonymous
171814	NTHI477_RS00820	34	C	G	nonsynonymous
171815	NTHI477_RS00820	35	G	A	nonsynonymous
171832	NTHI477_RS00820	52	G	A	nonsynonymous
171840	NTHI477_RS00820	60	T	C	synonymous
171855	NTHI477_RS00820	75	A	T	synonymous
171874	NTHI477_RS00820	94	A	G	nonsynonymous
171875	NTHI477_RS00820	95	C	T	nonsynonymous
171883	NTHI477_RS00820	103	G	A	nonsynonymous
171892	NTHI477_RS00820	112	A	G	nonsynonymous
171904	NTHI477_RS00820	124	G	T	nonsynonymous
171906	NTHI477_RS00820	126	T	C	synonymous
171918	NTHI477_RS00820	138	C	T	synonymous
171930	NTHI477_RS00820	150	T	C	synonymous
171982	NTHI477_RS00820	202	C	A	nonsynonymous
171996	NTHI477_RS00820	216	C	T	synonymous
172008	NTHI477_RS00820	228	C	T	synonymous
172011	NTHI477_RS00820	231	T	A	synonymous
172014	NTHI477_RS00820	234	A	G	synonymous
172019	NTHI477_RS00820	239	A	T	nonsynonymous
172023	NTHI477_RS00820	243	C	T	synonymous
172026	NTHI477_RS00820	246	T	C	synonymous

172029	NTHI477_RS00820	249	A	G	synonymous
172032	NTHI477_RS00820	252	T	A	synonymous
172040	NTHI477_RS00820	260	G	A	nonsynonymous
172060	NTHI477_RS00820	280	G	C	nonsynonymous
172062	NTHI477_RS00820	282	T	C	synonymous
172065	NTHI477_RS00820	285	G	T	synonymous
172083	NTHI477_RS00820	303	G	A	synonymous
172087	NTHI477_RS00820	307	T	C	nonsynonymous
172089	NTHI477_RS00820	309	C	T	synonymous
172098	NTHI477_RS00820	318	G	A	nonsynonymous
172116	NTHI477_RS00820	336	C	T	synonymous
172131	NTHI477_RS00820	351	A	G	synonymous
172139	NTHI477_RS00820	359	T	C	nonsynonymous
172170	NTHI477_RS00820	390	T	C	synonymous
172171	NTHI477_RS00820	391	A	G	nonsynonymous
172191	NTHI477_RS00820	411	T	C	synonymous
172196	NTHI477_RS00820	416	C	T	nonsynonymous
172197	NTHI477_RS00820	417	G	A	synonymous
172212	NTHI477_RS00820	432	C	T	synonymous
172215	NTHI477_RS00820	435	T	C	synonymous
172224	NTHI477_RS00820	444	A	T	nonsynonymous
172225	NTHI477_RS00820	445	C	A	nonsynonymous
172225	NTHI477_RS00820	445	C	T	nonsynonymous
172259	NTHI477_RS00820	479	A	G	nonsynonymous
172272	NTHI477_RS00820	492	G	T	nonsynonymous
172323	NTHI477_RS00820	543	G	A	synonymous
172326	NTHI477_RS00820	546	G	A	synonymous
172328	NTHI477_RS00820	548	C	T	nonsynonymous
172341	NTHI477_RS00820	561	T	C	synonymous
172359	NTHI477_RS00820	579	G	A	synonymous
172362	NTHI477_RS00820	582	T	C	synonymous
172368	NTHI477_RS00820	588	A	T	synonymous
172386	NTHI477_RS00820	606	G	C	synonymous
172395	NTHI477_RS00820	615	G	T	synonymous
172420	NTHI477_RS00820	640	T	C	synonymous
172440	NTHI477_RS00820	660	C	T	synonymous
172455	NTHI477_RS00820	675	A	G	synonymous
172464	NTHI477_RS00820	684	C	T	synonymous
172467	NTHI477_RS00820	687	A	G	synonymous
172470	NTHI477_RS00820	690	C	T	synonymous
172470	NTHI477_RS00820	690	C	A	synonymous
172476	NTHI477_RS00820	696	C	T	synonymous
172531	NTHI477_RS00820	751	G	T	nonsynonymous
172537	NTHI477_RS00820	757	C	G	nonsynonymous
172554	NTHI477_RS00820	774	G	A	synonymous

172572	NTHI477_RS00820	792	C	T	synonymous
172575	NTHI477_RS00820	795	C	T	synonymous
172590	NTHI477_RS00820	810	G	A	synonymous
172599	NTHI477_RS00820	819	C	T	synonymous
172617	NTHI477_RS00820	837	G	A	synonymous
172617	NTHI477_RS00820	837	G	T	synonymous
172620	NTHI477_RS00820	840	C	A	synonymous
172629	NTHI477_RS00820	849	C	T	synonymous
172630	NTHI477_RS00820	850	C	T	nonsynonymous
172635	NTHI477_RS00820	855	C	T	synonymous
172644	NTHI477_RS00820	864	A	G	synonymous
172645	NTHI477_RS00820	865	T	C	synonymous
172647	NTHI477_RS00820	867	A	G	synonymous
172653	NTHI477_RS00820	873	G	A	synonymous
172662	NTHI477_RS00820	882	C	T	synonymous
172686	NTHI477_RS00820	906	G	A	synonymous
172697	NTHI477_RS00820	917	T	C	nonsynonymous
172707	NTHI477_RS00820	927	C	T	synonymous
172722	NTHI477_RS00820	942	C	T	synonymous
172728	NTHI477_RS00820	948	C	T	synonymous
172752	NTHI477_RS00820	972	G	A	synonymous
172755	NTHI477_RS00820	975	A	G	synonymous
172782	NTHI477_RS00820	1002	T	A	synonymous
172791	NTHI477_RS00820	1011	G	T	synonymous
172791	NTHI477_RS00820	1011	G	A	synonymous
172797	NTHI477_RS00820	1017	A	G	synonymous
172800	NTHI477_RS00820	1020	T	A	synonymous
172811	NTHI477_RS00820	1031	C	A	nonsynonymous
172815	NTHI477_RS00820	1035	A	G	synonymous
172833	NTHI477_RS00820	1053	A	G	synonymous
172835	NTHI477_RS00820	1055	T	C	nonsynonymous
172836	NTHI477_RS00820	1056	C	T	synonymous
172837	NTHI477_RS00820	1057	G	T	nonsynonymous
172837	NTHI477_RS00820	1057	G	A	nonsynonymous
172839	NTHI477_RS00820	1059	A	G	synonymous
172842	NTHI477_RS00820	1062	C	T	synonymous
172845	NTHI477_RS00820	1065	A	G	synonymous
172847	NTHI477_RS00820	1067	T	A	nonsynonymous
172850	NTHI477_RS00820	1070	C	A	nonsynonymous
172851	NTHI477_RS00820	1071	A	G	synonymous
172853	NTHI477_RS00820	1073	C	T	nonsynonymous
172858	NTHI477_RS00820	1078	G	A	nonsynonymous
172860	NTHI477_RS00820	1080	C	A	synonymous
172863	NTHI477_RS00820	1083	C	T	synonymous
172864	NTHI477_RS00820	1084	A	G	nonsynonymous

172867	NTHI477_RS00820	1087	G	A	nonsynonymous
172870	NTHI477_RS00820	1090	G	A	nonsynonymous
172874	NTHI477_RS00820	1094	G	C	nonsynonymous
172876	NTHI477_RS00820	1096	T	G	nonsynonymous
172878	NTHI477_RS00820	1098	C	T	synonymous
172905	NTHI477_RS00820	1125	C	T	synonymous
172915	NTHI477_RS00820	1135	C	T	synonymous
172926	NTHI477_RS00820	1146	T	C	synonymous
172929	NTHI477_RS00820	1149	C	T	synonymous
172950	NTHI477_RS00820	1170	C	T	synonymous
172956	NTHI477_RS00820	1176	A	C	synonymous
172959	NTHI477_RS00820	1179	A	G	synonymous
172971	NTHI477_RS00820	1191	C	T	synonymous
172996	NTHI477_RS00820	1216	A	G	nonsynonymous
173003	NTHI477_RS00820	1223	A	G	nonsynonymous
173013	NTHI477_RS00820	1233	C	A	nonsynonymous
173025	NTHI477_RS00820	1245	A	G	synonymous
173041	NTHI477_RS00820	1261	G	T	nonsynonymous
173043	NTHI477_RS00820	1263	C	T	synonymous
173044	NTHI477_RS00820	1264	C	T	synonymous
173056	NTHI477_RS00820	1276	A	G	nonsynonymous
173059	NTHI477_RS00820	1279	G	A	nonsynonymous
173064	NTHI477_RS00820	1284	G	A	synonymous
173065	NTHI477_RS00820	1285	G	A	nonsynonymous
173067	NTHI477_RS00820	1287	C	T	synonymous
173079	NTHI477_RS00820	1299	G	A	synonymous
173164	NTHI477_RS00820	1384	G	A	nonsynonymous
173190	NTHI477_RS00820	1410	C	T	synonymous
173197	NTHI477_RS00820	1417	G	T	nonsynonymous
173199	NTHI477_RS00820	1419	G	A	synonymous
173217	NTHI477_RS00820	1437	A	G	synonymous
173240	NTHI477_RS00820	1460	A	G	nonsynonymous
173242	NTHI477_RS00820	1462	A	G	nonsynonymous
173253	NTHI477_RS00820	1473	C	T	synonymous
173274	NTHI477_RS00820	1494	T	C	synonymous
173291	NTHI477_RS00820	1511	A	C	nonsynonymous
173293	NTHI477_RS00820	1513	C	G	nonsynonymous
173361	NTHI477_RS00820	1581	T	C	synonymous
173397	NTHI477_RS00820	1617	A	C	synonymous
173400	NTHI477_RS00820	1620	A	T	synonymous
173400	NTHI477_RS00820	1620	A	G	synonymous
173450	NTHI477_RS00820	1670	A	G	nonsynonymous
173463	NTHI477_RS00820	1683	G	A	synonymous
173466	NTHI477_RS00820	1686	C	T	synonymous
173469	NTHI477_RS00820	1689	A	G	synonymous

173471	NTHI477_RS00820	1691	C	T	nonsynonymous
173474	NTHI477_RS00820	1694	C	T	nonsynonymous
173508	NTHI477_RS00820	1728	C	A	synonymous
173515	NTHI477_RS00820	1735	G	A	nonsynonymous
173520	NTHI477_RS00820	1740	C	T	synonymous
173523	NTHI477_RS00820	1743	T	C	synonymous
173586	NTHI477_RS00820	1806	C	G	synonymous
173602	NTHI477_RS00820	1822	G	C	nonsynonymous
173607	NTHI477_RS00820	1827	C	T	synonymous
173610	NTHI477_RS00820	1830	T	A	synonymous
173617	NTHI477_RS00820	1837	G	A	nonsynonymous
173643	NTHI477_RS00820	1863	G	A	synonymous
173649	NTHI477_RS00820	1869	T	C	synonymous
173691	NTHI477_RS00820	1911	A	G	synonymous
173758	intergenic	intergenic	A	T	intergenic
173877	NTHI477_RS00825	342	T	C	synonymous
173894	NTHI477_RS00825	325	G	T	synonymous
173922	NTHI477_RS00825	297	A	C	synonymous
173952	NTHI477_RS00825	267	G	T	synonymous
173967	NTHI477_RS00825	252	T	A	synonymous
173991	NTHI477_RS00825	228	G	T	synonymous
174000	NTHI477_RS00825	219	G	A	synonymous
174012	NTHI477_RS00825	207	C	T	synonymous
174026	NTHI477_RS00825	193	C	T	nonsynonymous
174027	NTHI477_RS00825	192	T	C	synonymous
174030	NTHI477_RS00825	189	A	G	synonymous
174038	NTHI477_RS00825	181	G	T	nonsynonymous
174039	NTHI477_RS00825	180	A	G	synonymous
174042	NTHI477_RS00825	177	T	C	synonymous
174062	NTHI477_RS00825	157	G	T	nonsynonymous
174064	NTHI477_RS00825	155	G	T	nonsynonymous
174065	NTHI477_RS00825	154	A	C	nonsynonymous
174066	NTHI477_RS00825	153	A	T	synonymous
174079	NTHI477_RS00825	140	G	C	nonsynonymous
174096	NTHI477_RS00825	123	G	T	synonymous
174096	NTHI477_RS00825	123	G	C	synonymous
174098	NTHI477_RS00825	121	C	T	nonsynonymous
174102	NTHI477_RS00825	117	A	G	synonymous
174117	NTHI477_RS00825	102	A	T	synonymous
174129	NTHI477_RS00825	90	G	A	synonymous
174188	NTHI477_RS00825	31	A	C	nonsynonymous
174248	intergenic	intergenic	A	G	intergenic
174269	intergenic	intergenic	C	A	intergenic
174329	intergenic	intergenic	T	C	intergenic
174340	intergenic	intergenic	C	T	intergenic

174378	NTHI477_RS00830	34	G	C	nonsynonymous
174447	NTHI477_RS00830	103	G	A	nonsynonymous
174475	NTHI477_RS00830	131	C	A	nonsynonymous
174481	NTHI477_RS00830	137	G	C	nonsynonymous
174485	NTHI477_RS00830	141	C	T	synonymous
174500	NTHI477_RS00830	156	T	C	synonymous
174506	NTHI477_RS00830	162	G	A	synonymous
174509	NTHI477_RS00830	165	A	G	synonymous
174519	NTHI477_RS00830	175	C	T	synonymous
174521	NTHI477_RS00830	177	A	G	synonymous
174601	NTHI477_RS00830	257	C	T	nonsynonymous
174609	NTHI477_RS00830	265	G	T	nonsynonymous
174670	NTHI477_RS00830	326	C	A	nonsynonymous
174698	NTHI477_RS00830	354	T	C	synonymous
174705	NTHI477_RS00830	361	G	A	nonsynonymous
174707	NTHI477_RS00830	363	A	T	synonymous
174707	NTHI477_RS00830	363	A	G	synonymous
174710	NTHI477_RS00830	366	G	A	synonymous
174719	NTHI477_RS00830	375	T	C	synonymous
174720	NTHI477_RS00830	376	G	A	nonsynonymous
174797	NTHI477_RS00830	453	G	T	synonymous
174797	NTHI477_RS00830	453	G	A	synonymous
174803	NTHI477_RS00830	459	G	A	synonymous
174812	NTHI477_RS00830	468	G	A	synonymous
174821	NTHI477_RS00830	477	T	C	synonymous
174824	NTHI477_RS00830	480	C	A	synonymous
174869	NTHI477_RS00830	525	A	G	synonymous
174883	NTHI477_RS00830	539	A	T	nonsynonymous
174891	NTHI477_RS00830	547	C	T	synonymous
174902	NTHI477_RS00830	558	G	A	synonymous
174907	NTHI477_RS00830	563	T	G	nonsynonymous
174957	NTHI477_RS00830	613	A	G	nonsynonymous
175011	NTHI477_RS00830	667	A	T	nonsynonymous
175074	NTHI477_RS00830	730	A	G	nonsynonymous
175082	NTHI477_RS00830	738	A	G	synonymous
175111	NTHI477_RS00830	767	C	T	nonsynonymous
175113	NTHI477_RS00830	769	A	G	nonsynonymous
175125	NTHI477_RS00830	781	C	A	nonsynonymous
175161	NTHI477_RS00830	817	C	T	nonsynonymous
175162	NTHI477_RS00830	818	T	C	nonsynonymous
175163	NTHI477_RS00830	819	T	G	synonymous
175166	NTHI477_RS00830	822	T	G	synonymous
175178	NTHI477_RS00830	834	C	T	synonymous
175193	NTHI477_RS00830	849	T	G	synonymous
175206	NTHI477_RS00830	862	A	G	nonsynonymous

175211	NTHI477_RS00830	867	C	T	synonymous
175256	NTHI477_RS00830	912	G	A	synonymous
175262	NTHI477_RS00830	918	T	C	synonymous
175274	NTHI477_RS00830	930	T	A	synonymous
175283	NTHI477_RS00830	939	T	A	synonymous
175297	NTHI477_RS00830	953	T	A	nonsynonymous
175310	NTHI477_RS00830	966	T	C	synonymous
175331	NTHI477_RS00830	987	A	G	synonymous
175343	NTHI477_RS00830	999	C	T	synonymous
175358	NTHI477_RS00830	1014	T	C	synonymous
175364	NTHI477_RS00830	1020	C	G	synonymous
175391	NTHI477_RS00830	1047	C	T	synonymous
175397	NTHI477_RS00830	1053	G	T	synonymous
175403	NTHI477_RS00830	1059	C	T	synonymous
175415	NTHI477_RS00830	1071	A	G	synonymous
175427	NTHI477_RS00830	1083	G	C	synonymous
175448	NTHI477_RS00830	1104	T	A	synonymous
175451	NTHI477_RS00830	1107	T	C	synonymous
175454	NTHI477_RS00830	1110	G	A	synonymous
175496	NTHI477_RS00830	1152	G	A	synonymous
175502	NTHI477_RS00830	1158	T	C	synonymous
175505	NTHI477_RS00830	1161	C	T	synonymous
175508	NTHI477_RS00830	1164	T	A	nonsynonymous
175544	NTHI477_RS00830	1200	A	C	synonymous
175547	NTHI477_RS00830	1203	G	T	synonymous
175562	NTHI477_RS00830	1218	T	C	synonymous
175568	NTHI477_RS00830	1224	C	A	synonymous
175568	NTHI477_RS00830	1224	C	T	synonymous
175580	NTHI477_RS00830	1236	G	T	synonymous
175583	NTHI477_RS00830	1239	A	G	synonymous
175589	NTHI477_RS00830	1245	C	T	synonymous
175601	NTHI477_RS00830	1257	C	A	synonymous
175616	NTHI477_RS00830	1272	G	T	synonymous
175634	NTHI477_RS00830	1290	C	A	synonymous
175634	NTHI477_RS00830	1290	C	G	synonymous
175640	NTHI477_RS00830	1296	A	G	synonymous
175661	NTHI477_RS00830	1317	T	A	synonymous
175673	NTHI477_RS00830	1329	T	C	synonymous
175691	NTHI477_RS00830	1347	G	T	synonymous
175697	NTHI477_RS00830	1353	C	T	synonymous
175704	NTHI477_RS00830	1360	C	T	synonymous
175708	NTHI477_RS00830	1364	C	T	nonsynonymous
175712	NTHI477_RS00830	1368	C	T	synonymous
175718	NTHI477_RS00830	1374	A	G	synonymous
175721	NTHI477_RS00830	1377	T	C	synonymous

175740	NTHI477_RS00830	1396	C	A	nonsynonymous
175760	NTHI477_RS00830	1416	G	A	synonymous
175796	NTHI477_RS00830	1452	T	G	synonymous
175808	NTHI477_RS00830	1464	A	G	synonymous
175817	NTHI477_RS00830	1473	C	T	synonymous
175841	NTHI477_RS00830	1497	C	T	synonymous
175845	NTHI477_RS00830	1501	T	G	nonsynonymous
175880	NTHI477_RS00830	1536	G	A	synonymous
175883	NTHI477_RS00830	1539	T	A	synonymous
175886	NTHI477_RS00830	1542	T	C	synonymous
175901	NTHI477_RS00830	1557	G	A	synonymous
175905	NTHI477_RS00830	1561	A	G	nonsynonymous
175910	NTHI477_RS00830	1566	A	T	nonsynonymous
175910	NTHI477_RS00830	1566	A	C	nonsynonymous
175913	NTHI477_RS00830	1569	G	A	synonymous
175952	NTHI477_RS00830	1608	T	C	synonymous
175989	NTHI477_RS00830	1645	G	A	nonsynonymous
175990	NTHI477_RS00830	1646	G	A	nonsynonymous
176000	NTHI477_RS00830	1656	G	A	synonymous
176009	NTHI477_RS00830	1665	A	G	synonymous
176033	NTHI477_RS00830	1689	T	A	synonymous
176037	NTHI477_RS00830	1693	C	T	nonsynonymous
176072	NTHI477_RS00830	1728	A	G	synonymous
176073	NTHI477_RS00830	1729	C	A	nonsynonymous
176075	NTHI477_RS00830	1731	T	A	synonymous
176078	NTHI477_RS00830	1734	C	T	synonymous
176084	NTHI477_RS00830	1740	T	A	synonymous
176104	NTHI477_RS00830	1760	A	G	nonsynonymous
176113	NTHI477_RS00830	1769	G	T	nonsynonymous
176144	intergenic	intergenic	G	A	intergenic
176201	intergenic	intergenic	A	G	intergenic
176222	intergenic	intergenic	A	G	intergenic
176231	intergenic	intergenic	A	C	intergenic
176235	intergenic	intergenic	T	G	intergenic
176242	intergenic	intergenic	G	A	intergenic
176242	intergenic	intergenic	G	C	intergenic
176298	NTHI477_RS00835	10	G	A	nonsynonymous
176320	NTHI477_RS00835	32	G	A	nonsynonymous
176324	NTHI477_RS00835	36	C	T	synonymous
176348	NTHI477_RS00835	60	G	A	synonymous
176369	NTHI477_RS00835	81	T	C	synonymous
176381	NTHI477_RS00835	93	C	G	synonymous
176384	NTHI477_RS00835	96	G	A	synonymous
176396	NTHI477_RS00835	108	G	T	synonymous
176402	NTHI477_RS00835	114	A	C	synonymous

176406	NTHI477_RS00835	118	T	C	synonymous
176420	NTHI477_RS00835	132	T	C	synonymous
176423	NTHI477_RS00835	135	T	A	synonymous
176424	NTHI477_RS00835	136	A	G	nonsynonymous
176438	NTHI477_RS00835	150	A	G	synonymous
176448	NTHI477_RS00835	160	G	A	nonsynonymous
176465	NTHI477_RS00835	177	C	T	synonymous
176489	NTHI477_RS00835	201	A	G	synonymous
176501	NTHI477_RS00835	213	G	A	synonymous
176504	NTHI477_RS00835	216	C	T	synonymous
176510	NTHI477_RS00835	222	T	C	synonymous
176513	NTHI477_RS00835	225	C	T	synonymous
176540	NTHI477_RS00835	252	A	C	synonymous
176555	NTHI477_RS00835	267	G	A	synonymous
176561	NTHI477_RS00835	273	G	T	synonymous
176627	NTHI477_RS00835	339	C	G	synonymous
176633	NTHI477_RS00835	345	A	C	synonymous
176639	NTHI477_RS00835	351	T	A	synonymous
176639	NTHI477_RS00835	351	T	G	synonymous
176648	NTHI477_RS00835	360	A	T	synonymous
176651	NTHI477_RS00835	363	A	G	synonymous
176663	NTHI477_RS00835	375	A	G	synonymous
176666	NTHI477_RS00835	378	A	G	synonymous
176669	NTHI477_RS00835	381	T	G	synonymous
176678	NTHI477_RS00835	390	G	A	synonymous
176681	NTHI477_RS00835	393	G	T	synonymous
176684	NTHI477_RS00835	396	C	T	synonymous
176685	NTHI477_RS00835	397	C	A	synonymous
176705	NTHI477_RS00835	417	T	A	synonymous
176720	NTHI477_RS00835	432	A	G	synonymous
176741	NTHI477_RS00835	453	T	A	synonymous
176741	NTHI477_RS00835	453	T	C	synonymous
176792	NTHI477_RS00835	504	G	A	synonymous
176814	NTHI477_RS00835	526	A	G	nonsynonymous
176827	intergenic	intergenic	T	C	intergenic
176839	intergenic	intergenic	C	T	intergenic
176843	intergenic	intergenic	C	T	intergenic
176855	intergenic	intergenic	G	C	intergenic
176859	intergenic	intergenic	G	A	intergenic
176861	intergenic	intergenic	T	G	intergenic
176861	intergenic	intergenic	T	C	intergenic
176863	intergenic	intergenic	T	G	intergenic
176866	intergenic	intergenic	T	C	intergenic
176877	intergenic	intergenic	C	G	intergenic
176882	intergenic	intergenic	C	T	intergenic

176887	intergenic	intergenic	A	C	intergenic
176898	intergenic	intergenic	T	G	intergenic
176899	intergenic	intergenic	G	T	intergenic
176909	intergenic	intergenic	T	C	intergenic
176916	intergenic	intergenic	T	C	intergenic
176919	intergenic	intergenic	C	A	intergenic
176921	intergenic	intergenic	A	C	intergenic
176926	intergenic	intergenic	A	T	intergenic
176929	intergenic	intergenic	C	G	intergenic
176929	intergenic	intergenic	C	A	intergenic
176930	intergenic	intergenic	T	A	intergenic
176931	intergenic	intergenic	T	G	intergenic
176932	intergenic	intergenic	G	T	intergenic
176933	intergenic	intergenic	T	A	intergenic
176933	intergenic	intergenic	T	C	intergenic
176935	intergenic	intergenic	A	T	intergenic
176935	intergenic	intergenic	A	G	intergenic
176936	intergenic	intergenic	T	A	intergenic
176936	intergenic	intergenic	T	G	intergenic
176937	intergenic	intergenic	T	G	intergenic
176937	intergenic	intergenic	T	A	intergenic
176938	intergenic	intergenic	T	G	intergenic
176939	intergenic	intergenic	T	A	intergenic
176942	intergenic	intergenic	A	C	intergenic
176946	intergenic	intergenic	T	C	intergenic
176946	intergenic	intergenic	T	G	intergenic
176948	intergenic	intergenic	A	T	intergenic
176950	intergenic	intergenic	T	G	intergenic
176951	intergenic	intergenic	A	T	intergenic
176953	intergenic	intergenic	T	A	intergenic
176954	intergenic	intergenic	G	A	intergenic
176955	intergenic	intergenic	G	T	intergenic
176957	intergenic	intergenic	A	C	intergenic
176958	intergenic	intergenic	A	C	intergenic
176960	intergenic	intergenic	T	A	intergenic
176964	intergenic	intergenic	G	T	intergenic
176964	intergenic	intergenic	G	A	intergenic
176968	intergenic	intergenic	A	G	intergenic
176976	intergenic	intergenic	G	T	intergenic
176979	intergenic	intergenic	T	C	intergenic
176982	intergenic	intergenic	A	T	intergenic
176985	intergenic	intergenic	A	T	intergenic
176986	intergenic	intergenic	A	T	intergenic
176986	intergenic	intergenic	A	G	intergenic
176988	intergenic	intergenic	C	G	intergenic

176998	intergenic	intergenic	G	T	intergenic
177001	intergenic	intergenic	A	C	intergenic
177001	intergenic	intergenic	A	T	intergenic
177005	intergenic	intergenic	T	A	intergenic
177014	intergenic	intergenic	T	G	intergenic
177015	intergenic	intergenic	T	G	intergenic
177017	intergenic	intergenic	C	A	intergenic
177019	intergenic	intergenic	A	C	intergenic
177020	intergenic	intergenic	T	A	intergenic
177026	intergenic	intergenic	T	C	intergenic
177027	intergenic	intergenic	T	G	intergenic
177031	intergenic	intergenic	A	T	intergenic
177033	intergenic	intergenic	T	G	intergenic
177040	intergenic	intergenic	C	T	intergenic
177042	intergenic	intergenic	A	G	intergenic
177063	NTHI477_RS00840	8	T	C	nonsynonymous
177086	NTHI477_RS00840	31	G	T	nonsynonymous
177140	NTHI477_RS00840	85	G	A	nonsynonymous
177163	NTHI477_RS00840	108	G	A	synonymous
177184	NTHI477_RS00840	129	T	G	synonymous
177204	NTHI477_RS00840	149	C	T	nonsynonymous
177236	NTHI477_RS00840	181	T	C	nonsynonymous
177252	NTHI477_RS00840	197	T	G	nonsynonymous
177310	NTHI477_RS00840	255	T	G	synonymous
177313	NTHI477_RS00840	258	A	G	synonymous
177316	NTHI477_RS00840	261	G	A	synonymous
177320	NTHI477_RS00840	265	G	T	nonsynonymous
177328	NTHI477_RS00840	273	T	G	synonymous
177331	NTHI477_RS00840	276	C	T	synonymous
177349	NTHI477_RS00840	294	T	C	synonymous
177407	NTHI477_RS00840	352	T	C	synonymous
177421	NTHI477_RS00840	366	T	C	synonymous
177434	NTHI477_RS00840	379	G	A	nonsynonymous
177436	NTHI477_RS00840	381	G	A	synonymous
177439	NTHI477_RS00840	384	T	C	synonymous
177455	NTHI477_RS00840	400	C	A	synonymous
177472	NTHI477_RS00840	417	T	C	synonymous
177478	NTHI477_RS00840	423	G	T	synonymous
177480	NTHI477_RS00840	425	A	G	nonsynonymous
177481	NTHI477_RS00840	426	A	G	synonymous
177509	NTHI477_RS00840	454	T	G	nonsynonymous
177517	NTHI477_RS00840	462	C	T	synonymous
177519	NTHI477_RS00840	464	G	A	nonsynonymous
177592	NTHI477_RS00840	537	G	A	synonymous
177596	NTHI477_RS00840	541	A	G	nonsynonymous

177597	NTHI477_RS00840	542	C	A	nonsynonymous
177618	NTHI477_RS00840	563	T	C	nonsynonymous
177629	NTHI477_RS00840	574	A	G	nonsynonymous
177631	NTHI477_RS00840	576	A	T	synonymous
177632	NTHI477_RS00840	577	C	T	nonsynonymous
177638	NTHI477_RS00840	583	G	A	nonsynonymous
177639	NTHI477_RS00840	584	A	G	nonsynonymous
177642	NTHI477_RS00840	587	C	T	nonsynonymous
177643	NTHI477_RS00840	588	G	A	synonymous
177644	NTHI477_RS00840	589	A	G	nonsynonymous
177655	NTHI477_RS00840	600	A	G	synonymous
177676	NTHI477_RS00840	621	A	G	synonymous
177683	NTHI477_RS00840	628	A	C	nonsynonymous
177719	NTHI477_RS00840	664	A	T	nonsynonymous
177729	NTHI477_RS00840	674	C	A	nonsynonymous
177740	NTHI477_RS00840	685	T	G	nonsynonymous
177742	NTHI477_RS00840	687	G	A	synonymous
177743	NTHI477_RS00840	688	A	G	nonsynonymous
177766	NTHI477_RS00840	711	C	A	nonsynonymous
177769	NTHI477_RS00840	714	G	A	synonymous
177809	intergenic	intergenic	C	T	intergenic
177835	intergenic	intergenic	A	G	intergenic
177837	intergenic	intergenic	T	C	intergenic
177838	intergenic	intergenic	G	A	intergenic
177850	intergenic	intergenic	G	A	intergenic
177852	intergenic	intergenic	T	C	intergenic
177853	intergenic	intergenic	A	C	intergenic
177906	intergenic	intergenic	T	C	intergenic
177931	intergenic	intergenic	T	C	intergenic
177934	intergenic	intergenic	T	C	intergenic
177937	intergenic	intergenic	C	G	intergenic
177941	intergenic	intergenic	A	G	intergenic
177959	intergenic	intergenic	G	T	intergenic
177961	intergenic	intergenic	A	G	intergenic
177963	intergenic	intergenic	T	G	intergenic
177973	intergenic	intergenic	T	C	intergenic
177983	intergenic	intergenic	A	G	intergenic
177984	intergenic	intergenic	T	G	intergenic
177985	intergenic	intergenic	T	C	intergenic
177991	intergenic	intergenic	G	A	intergenic
178068	NTHI477_RS00850	15	T	A	synonymous
178119	NTHI477_RS00850	66	C	T	synonymous
178174	NTHI477_RS00850	121	A	G	nonsynonymous
178182	NTHI477_RS00850	129	T	C	synonymous
178191	NTHI477_RS00850	138	C	T	synonymous

178278	NTHI477_RS00850	225	T	A	synonymous
178296	NTHI477_RS00850	243	T	A	synonymous
178362	intergenic	intergenic	C	T	intergenic
178392	intergenic	intergenic	C	A	intergenic
178400	NTHI477_RS09110	603	C	A	nonsynonymous
178431	NTHI477_RS09110	572	G	A	nonsynonymous
178438	NTHI477_RS09110	565	G	T	nonsynonymous
178439	NTHI477_RS09110	564	A	G	synonymous
178444	NTHI477_RS09110	559	T	C	nonsynonymous
178445	NTHI477_RS09110	558	T	A	synonymous
178448	NTHI477_RS09110	555	T	G	nonsynonymous
178450	NTHI477_RS09110	553	C	T	nonsynonymous
178451	NTHI477_RS09110	552	A	G	synonymous
178452	NTHI477_RS09110	551	C	A	nonsynonymous
178454	NTHI477_RS09110	549	G	A	synonymous
178456	NTHI477_RS09110	547	T	A	nonsynonymous
178457	NTHI477_RS09110	546	G	A	synonymous
178511	NTHI477_RS09110	492	G	A	synonymous
178573	NTHI477_RS09110	430	A	C	nonsynonymous
178589	NTHI477_RS09110	414	A	G	synonymous
178619	NTHI477_RS09110	384	G	A	synonymous
178620	NTHI477_RS09110	383	C	A	nonsynonymous
178640	NTHI477_RS09110	363	C	T	synonymous
178642	NTHI477_RS09110	361	C	T	nonsynonymous
178651	NTHI477_RS09110	352	C	T	nonsynonymous
178656	NTHI477_RS09110	347	A	T	nonsynonymous
178658	NTHI477_RS09110	345	G	A	synonymous
178664	NTHI477_RS09110	339	C	A	synonymous
178667	NTHI477_RS09110	336	A	G	synonymous
178670	NTHI477_RS09110	333	C	A	synonymous
178673	NTHI477_RS09110	330	A	C	nonsynonymous
178683	NTHI477_RS09110	320	G	A	nonsynonymous
178686	NTHI477_RS09110	317	T	C	nonsynonymous
178688	NTHI477_RS09110	315	A	T	synonymous
178693	NTHI477_RS09110	310	T	G	nonsynonymous
178696	NTHI477_RS09110	307	C	T	nonsynonymous
178700	NTHI477_RS09110	303	G	A	synonymous
178706	NTHI477_RS09110	297	T	C	synonymous
178709	NTHI477_RS09110	294	C	A	nonsynonymous
178711	NTHI477_RS09110	292	A	G	synonymous
178713	NTHI477_RS09110	290	A	T	nonsynonymous
178714	NTHI477_RS09110	289	A	G	synonymous
178718	NTHI477_RS09110	285	C	T	synonymous
178719	NTHI477_RS09110	284	T	G	nonsynonymous
178721	NTHI477_RS09110	282	T	C	synonymous

178726	NTHI477_RS09110	277	G	A	nonsynonymous
178727	NTHI477_RS09110	276	C	A	synonymous
178728	NTHI477_RS09110	275	G	A	nonsynonymous
178729	NTHI477_RS09110	274	C	T	nonsynonymous
178736	NTHI477_RS09110	267	G	A	synonymous
178740	NTHI477_RS09110	263	A	C	nonsynonymous
178744	NTHI477_RS09110	259	C	T	nonsynonymous
178750	NTHI477_RS09110	253	A	G	nonsynonymous
178754	NTHI477_RS09110	249	C	A	nonsynonymous
178763	NTHI477_RS09110	240	T	A	nonsynonymous
178765	NTHI477_RS09110	238	A	G	nonsynonymous
178769	NTHI477_RS09110	234	G	A	synonymous
178784	NTHI477_RS09110	219	C	T	synonymous
178784	NTHI477_RS09110	219	C	A	synonymous
178787	NTHI477_RS09110	216	G	C	synonymous
178796	NTHI477_RS09110	207	T	A	synonymous
178808	NTHI477_RS09110	195	C	A	synonymous
178812	NTHI477_RS09110	191	C	T	nonsynonymous
178818	NTHI477_RS09110	185	C	A	nonsynonymous
178821	NTHI477_RS09110	182	T	A	nonsynonymous
178824	NTHI477_RS09110	179	C	A	nonsynonymous
178832	NTHI477_RS09110	171	A	G	synonymous
178861	NTHI477_RS09110	142	C	T	nonsynonymous
178887	NTHI477_RS09110	116	C	G	nonsynonymous
178914	NTHI477_RS09110	89	T	C	nonsynonymous
178938	NTHI477_RS09110	65	T	A	nonsynonymous
178974	NTHI477_RS09110	29	G	T	nonsynonymous
178986	NTHI477_RS09110	17	A	G	nonsynonymous
178989	NTHI477_RS09110	14	G	A	nonsynonymous
179014	intergenic	intergenic	A	G	intergenic
179024	intergenic	intergenic	G	A	intergenic
179025	intergenic	intergenic	C	A	intergenic
179031	intergenic	intergenic	T	C	intergenic
179065	intergenic	intergenic	A	G	intergenic
179099	NTHI477_RS00865	1437	A	G	synonymous
179110	NTHI477_RS00865	1426	G	T	nonsynonymous
179168	NTHI477_RS00865	1368	C	T	synonymous
179174	NTHI477_RS00865	1362	G	A	synonymous
179177	NTHI477_RS00865	1359	A	C	synonymous
179303	NTHI477_RS00865	1233	C	T	synonymous
179345	NTHI477_RS00865	1191	A	G	synonymous
179357	NTHI477_RS00865	1179	G	A	synonymous
179377	NTHI477_RS00865	1159	G	A	synonymous
179420	NTHI477_RS00865	1116	C	T	synonymous
179606	NTHI477_RS00865	930	G	C	synonymous

179624	NTHI477_RS00865	912	T	C	synonymous
179636	NTHI477_RS00865	900	T	A	synonymous
179648	NTHI477_RS00865	888	T	G	synonymous
179668	NTHI477_RS00865	868	G	A	synonymous
179678	NTHI477_RS00865	858	T	C	synonymous
179690	NTHI477_RS00865	846	G	A	synonymous
179711	NTHI477_RS00865	825	G	A	synonymous
179728	NTHI477_RS00865	808	G	T	nonsynonymous
179751	NTHI477_RS00865	785	G	T	nonsynonymous
179753	NTHI477_RS00865	783	G	A	synonymous
179787	NTHI477_RS00865	749	C	T	nonsynonymous
179879	NTHI477_RS00865	657	T	C	synonymous
179924	NTHI477_RS00865	612	T	A	synonymous
179993	NTHI477_RS00865	543	A	G	synonymous
180014	NTHI477_RS00865	522	T	C	synonymous
180152	NTHI477_RS00865	384	G	T	synonymous
180205	NTHI477_RS00865	331	G	T	nonsynonymous
180217	NTHI477_RS00865	319	G	T	nonsynonymous
180218	NTHI477_RS00865	318	A	G	synonymous
180234	NTHI477_RS00865	302	C	T	nonsynonymous
180245	NTHI477_RS00865	291	C	T	synonymous
180260	NTHI477_RS00865	276	T	A	synonymous
180284	NTHI477_RS00865	252	G	A	synonymous
180285	NTHI477_RS00865	251	C	T	nonsynonymous
180290	NTHI477_RS00865	246	T	C	synonymous
180292	NTHI477_RS00865	244	C	A	nonsynonymous
180292	NTHI477_RS00865	244	C	T	nonsynonymous
180293	NTHI477_RS00865	243	A	G	synonymous
180302	NTHI477_RS00865	234	G	A	synonymous
180303	NTHI477_RS00865	233	G	A	nonsynonymous
180305	NTHI477_RS00865	231	A	G	synonymous
180308	NTHI477_RS00865	228	G	A	synonymous
180311	NTHI477_RS00865	225	T	A	synonymous
180329	NTHI477_RS00865	207	G	A	synonymous
180332	NTHI477_RS00865	204	T	C	synonymous
180337	NTHI477_RS00865	199	A	G	nonsynonymous
180338	NTHI477_RS00865	198	A	C	synonymous
180364	NTHI477_RS00865	172	C	T	nonsynonymous
180371	NTHI477_RS00865	165	A	T	synonymous
180377	NTHI477_RS00865	159	G	A	synonymous
180386	NTHI477_RS00865	150	T	G	synonymous
180395	NTHI477_RS00865	141	G	A	synonymous
180398	NTHI477_RS00865	138	G	A	synonymous
180401	NTHI477_RS00865	135	G	A	synonymous
180403	NTHI477_RS00865	133	A	C	nonsynonymous

180408	NTHI477_RS00865	128	A	G	nonsynonymous
180427	NTHI477_RS00865	109	G	A	synonymous
180437	NTHI477_RS00865	99	T	A	nonsynonymous
180440	NTHI477_RS00865	96	A	G	synonymous
180455	NTHI477_RS00865	81	A	G	synonymous
180458	NTHI477_RS00865	78	G	T	synonymous
180464	NTHI477_RS00865	72	G	A	synonymous
180470	NTHI477_RS00865	66	A	T	synonymous
180479	NTHI477_RS00865	57	A	G	synonymous
180482	NTHI477_RS00865	54	C	T	synonymous
180485	NTHI477_RS00865	51	T	G	synonymous
180500	NTHI477_RS00865	36	A	T	synonymous
180512	NTHI477_RS00865	24	A	G	synonymous
180606	intergenic	intergenic	C	T	intergenic
180632	intergenic	intergenic	G	A	intergenic
180743	NTHI477_RS00870	45	A	T	synonymous
180770	NTHI477_RS00870	72	A	T	synonymous
180806	NTHI477_RS00870	108	C	A	synonymous
180809	NTHI477_RS00870	111	C	T	synonymous
180875	NTHI477_RS00870	177	G	A	synonymous
180878	NTHI477_RS00870	180	T	G	synonymous
180930	NTHI477_RS00870	232	A	T	nonsynonymous
180977	NTHI477_RS00870	279	A	G	synonymous
180995	NTHI477_RS00870	297	A	T	synonymous
180998	NTHI477_RS00870	300	C	T	synonymous
181034	NTHI477_RS00870	336	G	A	synonymous
181037	NTHI477_RS00870	339	T	G	synonymous
181040	NTHI477_RS00870	342	T	C	synonymous
181064	NTHI477_RS00870	366	T	A	synonymous
181067	NTHI477_RS00870	369	T	C	synonymous
181070	NTHI477_RS00870	372	A	G	synonymous
181073	NTHI477_RS00870	375	A	C	synonymous
181079	NTHI477_RS00870	381	G	A	synonymous
181085	NTHI477_RS00870	387	T	C	synonymous
181133	NTHI477_RS00870	435	C	T	synonymous
181145	NTHI477_RS00870	447	A	G	synonymous
181192	intergenic	intergenic	G	A	intergenic
181198	intergenic	intergenic	G	A	intergenic
181262	NTHI477_RS00875	252	C	T	synonymous
181327	NTHI477_RS00875	187	C	T	nonsynonymous
181415	NTHI477_RS00875	99	A	G	synonymous
181436	NTHI477_RS00875	78	A	C	synonymous
181436	NTHI477_RS00875	78	A	T	synonymous
181466	NTHI477_RS00875	48	C	T	synonymous
181520	intergenic	intergenic	T	C	intergenic

181559	intergenic	intergenic	T	C	intergenic
181561	intergenic	intergenic	C	T	intergenic
181572	intergenic	intergenic	C	T	intergenic
181576	intergenic	intergenic	T	C	intergenic
181580	intergenic	intergenic	G	A	intergenic
181681	NTHI477_RS00880	42	G	A	synonymous
181688	NTHI477_RS00880	49	C	T	nonsynonymous
181792	NTHI477_RS00880	153	G	A	synonymous
181801	NTHI477_RS00880	162	A	C	synonymous
181807	NTHI477_RS00880	168	C	T	synonymous
181816	NTHI477_RS00880	177	T	C	synonymous
181819	NTHI477_RS00880	180	C	A	synonymous
181873	NTHI477_RS00880	234	T	A	synonymous
181894	NTHI477_RS00880	255	G	A	synonymous
181927	NTHI477_RS00880	288	A	G	synonymous
181951	NTHI477_RS00880	312	T	C	synonymous
181966	NTHI477_RS00880	327	A	G	synonymous
181967	NTHI477_RS00880	328	T	C	synonymous
181971	NTHI477_RS00880	332	C	A	nonsynonymous
181972	NTHI477_RS00880	333	A	G	synonymous
181973	NTHI477_RS00880	334	T	C	nonsynonymous
181978	NTHI477_RS00880	339	A	G	synonymous
181981	NTHI477_RS00880	342	T	C	synonymous
181986	NTHI477_RS00880	347	A	C	nonsynonymous
181999	NTHI477_RS00880	360	T	G	synonymous
182002	NTHI477_RS00880	363	G	A	synonymous
182056	NTHI477_RS00880	417	T	C	synonymous
182109	NTHI477_RS00880	470	G	A	nonsynonymous
182134	NTHI477_RS00880	495	T	C	synonymous
182137	NTHI477_RS00880	498	C	T	synonymous
182155	NTHI477_RS00880	516	T	C	synonymous
182157	NTHI477_RS00880	518	A	C	nonsynonymous
182158	NTHI477_RS00880	519	A	G	synonymous
182186	NTHI477_RS00880	547	A	G	nonsynonymous
182194	NTHI477_RS00880	555	G	A	synonymous
182212	NTHI477_RS00880	573	A	G	synonymous
182266	NTHI477_RS00880	627	T	A	synonymous
182581	NTHI477_RS00885	222	T	C	synonymous
182617	NTHI477_RS00885	258	G	C	synonymous
182653	NTHI477_RS00885	294	C	T	synonymous
182710	NTHI477_RS00885	351	A	T	synonymous
182725	NTHI477_RS00885	366	G	A	synonymous
182851	NTHI477_RS00885	492	C	G	synonymous
182871	NTHI477_RS00885	512	G	T	nonsynonymous
182941	NTHI477_RS00885	582	T	C	synonymous

183151	NTHI477_RS00885	792	C	T	synonymous
183226	NTHI477_RS00885	867	T	C	synonymous
183241	NTHI477_RS00885	882	C	G	synonymous
183241	NTHI477_RS00885	882	C	A	synonymous
183262	NTHI477_RS00885	903	G	A	synonymous
183280	NTHI477_RS00885	921	T	C	synonymous
183371	NTHI477_RS00885	1012	T	C	nonsynonymous
183374	NTHI477_RS00885	1015	G	A	nonsynonymous
183379	NTHI477_RS00885	1020	G	A	synonymous
183400	NTHI477_RS00885	1041	G	A	synonymous
183415	NTHI477_RS00885	1056	G	A	synonymous
183418	NTHI477_RS00885	1059	G	C	synonymous
183421	NTHI477_RS00885	1062	C	T	synonymous
183457	NTHI477_RS00885	1098	G	A	synonymous
183460	NTHI477_RS00885	1101	G	T	synonymous
183461	NTHI477_RS00885	1102	G	A	nonsynonymous
183463	NTHI477_RS00885	1104	T	C	synonymous
183466	NTHI477_RS00885	1107	G	A	synonymous
183481	NTHI477_RS00885	1122	G	C	synonymous
183491	NTHI477_RS00885	1132	A	G	nonsynonymous
183520	NTHI477_RS00885	1161	G	A	synonymous
183526	NTHI477_RS00885	1167	T	C	synonymous
183547	NTHI477_RS00885	1188	G	A	synonymous
183556	NTHI477_RS00885	1197	T	C	synonymous
183580	NTHI477_RS00885	1221	A	G	synonymous
183586	NTHI477_RS00885	1227	C	A	synonymous
183589	NTHI477_RS00885	1230	T	C	synonymous
183598	NTHI477_RS00885	1239	T	C	synonymous
183604	NTHI477_RS00885	1245	T	A	synonymous
183604	NTHI477_RS00885	1245	T	C	synonymous
183610	NTHI477_RS00885	1251	C	T	synonymous
183613	NTHI477_RS00885	1254	C	G	synonymous
183613	NTHI477_RS00885	1254	C	T	synonymous
183622	NTHI477_RS00885	1263	T	G	synonymous
183631	NTHI477_RS00885	1272	G	A	synonymous
183637	NTHI477_RS00885	1278	A	G	synonymous
183640	NTHI477_RS00885	1281	C	T	synonymous
183649	NTHI477_RS00885	1290	T	A	synonymous
183652	NTHI477_RS00885	1293	C	T	synonymous
183661	NTHI477_RS00885	1302	C	G	synonymous
183673	NTHI477_RS00885	1314	A	T	synonymous
183676	NTHI477_RS00885	1317	A	T	synonymous
183676	NTHI477_RS00885	1317	A	C	synonymous
183677	NTHI477_RS00885	1318	C	A	nonsynonymous
183679	NTHI477_RS00885	1320	T	A	synonymous

183682	NTHI477_RS00885	1323	C	G	synonymous
183685	NTHI477_RS00885	1326	G	A	synonymous
183691	NTHI477_RS00885	1332	G	A	synonymous
183703	NTHI477_RS00885	1344	A	G	synonymous
183709	NTHI477_RS00885	1350	G	A	synonymous
183712	NTHI477_RS00885	1353	C	T	synonymous
183769	NTHI477_RS00885	1410	A	G	synonymous
183772	NTHI477_RS00885	1413	G	T	synonymous
183781	NTHI477_RS00885	1422	A	G	synonymous
183796	NTHI477_RS00885	1437	C	T	synonymous
183799	NTHI477_RS00885	1440	C	T	synonymous
183802	NTHI477_RS00885	1443	A	G	synonymous
183817	NTHI477_RS00885	1458	A	G	synonymous
183841	NTHI477_RS00885	1482	C	A	synonymous
183841	NTHI477_RS00885	1482	C	G	synonymous
183844	NTHI477_RS00885	1485	A	G	synonymous
183853	NTHI477_RS00885	1494	T	C	synonymous
183856	NTHI477_RS00885	1497	G	T	synonymous
183868	NTHI477_RS00885	1509	A	G	synonymous
183874	NTHI477_RS00885	1515	A	G	synonymous
183889	NTHI477_RS00885	1530	A	C	nonsynonymous
183892	NTHI477_RS00885	1533	G	A	synonymous
183898	NTHI477_RS00885	1539	T	G	synonymous
183901	NTHI477_RS00885	1542	G	A	synonymous
183913	NTHI477_RS00885	1554	A	G	synonymous
183922	NTHI477_RS00885	1563	G	A	synonymous
183925	NTHI477_RS00885	1566	C	T	synonymous
183931	NTHI477_RS00885	1572	T	G	synonymous
183934	NTHI477_RS00885	1575	C	T	synonymous
183958	NTHI477_RS00885	1599	G	A	synonymous
183979	NTHI477_RS00885	1620	T	C	synonymous
183982	NTHI477_RS00885	1623	A	T	synonymous
183986	NTHI477_RS00885	1627	T	G	nonsynonymous
183988	NTHI477_RS00885	1629	G	A	synonymous
184003	NTHI477_RS00885	1644	G	T	synonymous
184021	NTHI477_RS00885	1662	T	C	synonymous
184040	NTHI477_RS00885	1681	G	C	nonsynonymous
184050	NTHI477_RS00885	1691	T	C	nonsynonymous
184051	NTHI477_RS00885	1692	C	T	synonymous
184054	NTHI477_RS00885	1695	C	T	synonymous
184087	NTHI477_RS00885	1728	G	A	synonymous
184105	NTHI477_RS00885	1746	C	T	synonymous
184117	NTHI477_RS00885	1758	G	A	synonymous
184126	NTHI477_RS00885	1767	A	G	synonymous
184147	NTHI477_RS00885	1788	T	C	synonymous

184170	NTHI477_RS00885	1811	G	A	nonsynonymous
184186	NTHI477_RS00885	1827	G	A	synonymous
184209	NTHI477_RS00885	1850	C	T	nonsynonymous
184228	NTHI477_RS00885	1869	C	T	synonymous
184253	NTHI477_RS00885	1894	A	G	nonsynonymous
184270	intergenic	intergenic	C	T	intergenic
184306	intergenic	intergenic	C	T	intergenic
184317	intergenic	intergenic	G	T	intergenic
184324	intergenic	intergenic	G	T	intergenic
184334	intergenic	intergenic	G	T	intergenic
184342	intergenic	intergenic	C	T	intergenic
184352	intergenic	intergenic	A	G	intergenic
184366	intergenic	intergenic	T	C	intergenic
184387	NTHI477_RS00890	9	T	A	synonymous
184393	NTHI477_RS00890	15	A	G	synonymous
184396	NTHI477_RS00890	18	T	C	synonymous
184405	NTHI477_RS00890	27	T	C	synonymous
184411	NTHI477_RS00890	33	T	C	synonymous
184414	NTHI477_RS00890	36	A	G	synonymous
184417	NTHI477_RS00890	39	C	T	synonymous
184420	NTHI477_RS00890	42	G	T	synonymous
184423	NTHI477_RS00890	45	T	C	synonymous
184424	NTHI477_RS00890	46	C	A	nonsynonymous
184426	NTHI477_RS00890	48	A	G	synonymous
184435	NTHI477_RS00890	57	G	A	synonymous
184438	NTHI477_RS00890	60	T	C	synonymous
184486	NTHI477_RS00890	108	T	C	synonymous
184487	NTHI477_RS00890	109	C	T	synonymous
184489	NTHI477_RS00890	111	A	G	synonymous
184498	NTHI477_RS00890	120	A	G	synonymous
184520	NTHI477_RS00890	142	C	T	synonymous
184522	NTHI477_RS00890	144	A	G	synonymous
184528	NTHI477_RS00890	150	G	A	synonymous
184531	NTHI477_RS00890	153	G	T	synonymous
184535	NTHI477_RS00890	157	A	G	nonsynonymous
184555	NTHI477_RS00890	177	A	T	synonymous
184561	NTHI477_RS00890	183	C	T	synonymous
184564	NTHI477_RS00890	186	T	G	synonymous
184570	NTHI477_RS00890	192	G	T	synonymous
184576	NTHI477_RS00890	198	G	A	synonymous
184585	NTHI477_RS00890	207	C	T	synonymous
184585	NTHI477_RS00890	207	C	G	synonymous
184597	NTHI477_RS00890	219	G	A	synonymous
184615	NTHI477_RS00890	237	G	A	synonymous
184633	NTHI477_RS00890	255	G	T	synonymous

184638	NTHI477_RS00890	260	A	G	nonsynonymous
184663	NTHI477_RS00890	285	T	C	synonymous
184669	NTHI477_RS00890	291	C	T	synonymous
184678	NTHI477_RS00890	300	T	A	synonymous
184679	NTHI477_RS00890	301	G	A	nonsynonymous
184696	NTHI477_RS00890	318	G	A	synonymous
184701	NTHI477_RS00890	323	A	G	nonsynonymous
184747	NTHI477_RS00890	369	G	A	synonymous
184753	NTHI477_RS00890	375	G	C	synonymous
184753	NTHI477_RS00890	375	G	A	synonymous
184762	NTHI477_RS00890	384	G	C	synonymous
184762	NTHI477_RS00890	384	G	A	synonymous
184763	NTHI477_RS00890	385	G	A	nonsynonymous
184765	NTHI477_RS00890	387	G	A	synonymous
184774	NTHI477_RS00890	396	G	A	synonymous
184789	NTHI477_RS00890	411	T	C	synonymous
184792	NTHI477_RS00890	414	T	C	synonymous
184814	NTHI477_RS00890	436	C	A	nonsynonymous
184816	NTHI477_RS00890	438	C	A	synonymous
184825	NTHI477_RS00890	447	A	G	synonymous
184835	NTHI477_RS00890	457	T	C	nonsynonymous
184843	NTHI477_RS00890	465	A	G	synonymous
184852	NTHI477_RS00890	474	G	A	synonymous
184864	NTHI477_RS00890	486	G	C	nonsynonymous
184876	NTHI477_RS00890	498	A	G	synonymous
184894	NTHI477_RS00890	516	C	T	synonymous
184906	NTHI477_RS00890	528	C	T	synonymous
184907	NTHI477_RS00890	529	A	G	nonsynonymous
184921	NTHI477_RS00890	543	T	C	synonymous
184933	NTHI477_RS00890	555	T	C	synonymous
184939	NTHI477_RS00890	561	C	A	synonymous
184943	NTHI477_RS00890	565	G	T	nonsynonymous
184945	NTHI477_RS00890	567	C	T	synonymous
184951	NTHI477_RS00890	573	T	C	synonymous
184954	NTHI477_RS00890	576	A	G	synonymous
185003	NTHI477_RS00890	625	A	C	nonsynonymous
185005	NTHI477_RS00890	627	G	T	nonsynonymous
185007	NTHI477_RS00890	629	A	G	nonsynonymous
185011	NTHI477_RS00890	633	C	A	synonymous
185017	NTHI477_RS00890	639	A	T	synonymous
185017	NTHI477_RS00890	639	A	C	synonymous
185020	NTHI477_RS00890	642	A	G	synonymous
185026	NTHI477_RS00890	648	G	A	synonymous
185029	NTHI477_RS00890	651	A	G	synonymous
185053	NTHI477_RS00890	675	C	T	synonymous

185056	NTHI477_RS00890	678	A	G	synonymous
185071	NTHI477_RS00890	693	T	C	synonymous
185071	NTHI477_RS00890	693	T	G	synonymous
185074	NTHI477_RS00890	696	T	C	synonymous
185080	NTHI477_RS00890	702	A	G	synonymous
185084	NTHI477_RS00890	706	A	G	nonsynonymous
185086	NTHI477_RS00890	708	A	G	nonsynonymous
185089	NTHI477_RS00890	711	T	A	synonymous
185092	NTHI477_RS00890	714	C	T	synonymous
185104	NTHI477_RS00890	726	C	T	synonymous
185104	NTHI477_RS00890	726	C	A	synonymous
185105	NTHI477_RS00890	727	G	A	nonsynonymous
185116	NTHI477_RS00890	738	A	T	synonymous
185118	NTHI477_RS00890	740	T	C	nonsynonymous
185128	NTHI477_RS00890	750	C	T	synonymous
185157	NTHI477_RS00890	779	C	T	nonsynonymous
185158	NTHI477_RS00890	780	T	A	synonymous
185161	NTHI477_RS00890	783	A	G	synonymous
185191	NTHI477_RS00890	813	A	G	synonymous
185209	intergenic	intergenic	G	A	intergenic
185210	intergenic	intergenic	T	C	intergenic
185215	intergenic	intergenic	T	G	intergenic
185239	intergenic	intergenic	T	G	intergenic
185241	intergenic	intergenic	A	G	intergenic
185248	NTHI477_RS00895	5	C	T	nonsynonymous
185279	NTHI477_RS00895	36	A	T	synonymous
185291	NTHI477_RS00895	48	A	G	synonymous
185294	NTHI477_RS00895	51	T	C	synonymous
185295	NTHI477_RS00895	52	G	T	nonsynonymous
185295	NTHI477_RS00895	52	G	A	nonsynonymous
185296	NTHI477_RS00895	53	C	G	nonsynonymous
185300	NTHI477_RS00895	57	C	T	synonymous
185306	NTHI477_RS00895	63	T	C	synonymous
185312	NTHI477_RS00895	69	A	T	synonymous
185333	NTHI477_RS00895	90	T	C	synonymous
185342	NTHI477_RS00895	99	G	A	synonymous
185351	NTHI477_RS00895	108	G	A	synonymous
185354	NTHI477_RS00895	111	G	A	synonymous
185355	NTHI477_RS00895	112	G	T	nonsynonymous
185360	NTHI477_RS00895	117	T	C	synonymous
185369	NTHI477_RS00895	126	G	T	synonymous
185393	NTHI477_RS00895	150	T	C	synonymous
185411	NTHI477_RS00895	168	T	C	synonymous
185429	NTHI477_RS00895	186	T	C	synonymous
185441	NTHI477_RS00895	198	A	G	synonymous

185444	NTHI477_RS00895	201	C	T	synonymous
185445	NTHI477_RS00895	202	G	A	nonsynonymous
185450	NTHI477_RS00895	207	G	A	synonymous
185453	NTHI477_RS00895	210	G	T	synonymous
185459	NTHI477_RS00895	216	C	T	synonymous
185465	NTHI477_RS00895	222	A	G	synonymous
185471	NTHI477_RS00895	228	C	T	synonymous
185474	NTHI477_RS00895	231	C	T	synonymous
185477	NTHI477_RS00895	234	T	G	synonymous
185483	NTHI477_RS00895	240	G	T	synonymous
185504	NTHI477_RS00895	261	A	G	synonymous
185516	NTHI477_RS00895	273	C	T	synonymous
185519	NTHI477_RS00895	276	T	A	synonymous
185528	NTHI477_RS00895	285	A	G	synonymous
185534	NTHI477_RS00895	291	T	C	synonymous
185537	NTHI477_RS00895	294	G	A	synonymous
185573	NTHI477_RS00895	330	C	G	synonymous
185597	NTHI477_RS00895	354	A	C	synonymous
185600	NTHI477_RS00895	357	A	T	synonymous
185627	NTHI477_RS00895	384	C	T	synonymous
185636	NTHI477_RS00895	393	A	G	synonymous
185645	NTHI477_RS00895	402	G	A	synonymous
185651	NTHI477_RS00895	408	G	T	synonymous
185654	NTHI477_RS00895	411	A	G	nonsynonymous
185672	NTHI477_RS00895	429	C	T	synonymous
185687	NTHI477_RS00895	444	A	C	synonymous
185702	NTHI477_RS00895	459	T	C	synonymous
185717	NTHI477_RS00895	474	T	C	synonymous
185735	NTHI477_RS00895	492	G	C	synonymous
185735	NTHI477_RS00895	492	G	T	synonymous
185744	NTHI477_RS00895	501	A	C	synonymous
185744	NTHI477_RS00895	501	A	T	synonymous
185747	NTHI477_RS00895	504	A	T	synonymous
185750	NTHI477_RS00895	507	C	T	synonymous
185762	NTHI477_RS00895	519	G	A	synonymous
185771	NTHI477_RS00895	528	A	G	synonymous
185778	NTHI477_RS00895	535	G	A	nonsynonymous
185801	NTHI477_RS00895	558	C	T	synonymous
185825	NTHI477_RS00895	582	T	G	nonsynonymous
185834	NTHI477_RS00895	591	A	G	synonymous
185846	NTHI477_RS00895	603	C	T	synonymous
185855	NTHI477_RS00895	612	T	G	nonsynonymous
185863	NTHI477_RS00895	620	A	G	nonsynonymous
185879	NTHI477_RS00895	636	T	C	synonymous
185909	NTHI477_RS00895	666	A	G	synonymous

185912	NTHI477_RS00895	669	T	A	synonymous
185914	NTHI477_RS00895	671	C	A	nonsynonymous
185924	NTHI477_RS00895	681	T	C	synonymous
185930	NTHI477_RS00895	687	A	T	synonymous
185938	NTHI477_RS00895	695	T	C	nonsynonymous
185939	NTHI477_RS00895	696	G	T	nonsynonymous
185948	NTHI477_RS00895	705	T	C	synonymous
185954	NTHI477_RS00895	711	C	T	synonymous
185957	NTHI477_RS00895	714	A	G	synonymous
185975	NTHI477_RS00895	732	C	T	synonymous
185978	NTHI477_RS00895	735	C	T	synonymous
185981	NTHI477_RS00895	738	C	A	synonymous
186010	NTHI477_RS00895	767	A	G	nonsynonymous
186020	NTHI477_RS00895	777	C	T	synonymous
186039	NTHI477_RS00895	796	A	G	nonsynonymous
186044	NTHI477_RS00895	801	G	A	synonymous
186044	NTHI477_RS00895	801	G	C	synonymous
186053	NTHI477_RS00895	810	A	G	synonymous
186059	NTHI477_RS00895	816	A	C	synonymous
186059	NTHI477_RS00895	816	A	T	synonymous
186083	NTHI477_RS00895	840	C	T	synonymous
186092	NTHI477_RS00895	849	C	A	synonymous
186111	NTHI477_RS00895	868	T	C	synonymous
186137	NTHI477_RS00895	894	C	T	synonymous
186143	NTHI477_RS00895	900	T	C	synonymous
186146	NTHI477_RS00895	903	C	T	synonymous
186158	NTHI477_RS00895	915	C	T	synonymous
186164	NTHI477_RS00895	921	A	C	synonymous
186179	NTHI477_RS00895	936	G	A	synonymous
186189	NTHI477_RS00895	946	C	G	nonsynonymous
186212	NTHI477_RS00895	969	G	T	synonymous
186215	NTHI477_RS00895	972	G	C	synonymous
186218	NTHI477_RS00895	975	G	A	synonymous
186224	NTHI477_RS00895	981	A	T	synonymous
186227	NTHI477_RS00895	984	A	T	synonymous
186242	NTHI477_RS00895	999	G	A	synonymous
186248	NTHI477_RS00895	1005	G	A	synonymous
186257	NTHI477_RS00895	1014	T	G	synonymous
186260	NTHI477_RS00895	1017	C	A	synonymous
186275	NTHI477_RS00895	1032	G	A	synonymous
186281	NTHI477_RS00895	1038	G	A	synonymous
186293	NTHI477_RS00895	1050	A	G	synonymous
186296	NTHI477_RS00895	1053	G	T	synonymous
186302	NTHI477_RS00895	1059	G	A	synonymous
186302	NTHI477_RS00895	1059	G	T	synonymous

186332	NTHI477_RS00895	1089	A	G	synonymous
186353	NTHI477_RS00895	1110	A	T	synonymous
186359	NTHI477_RS00895	1116	G	A	synonymous
186378	NTHI477_RS00895	1135	A	G	nonsynonymous
186380	NTHI477_RS00895	1137	G	A	synonymous
186389	NTHI477_RS00895	1146	T	A	nonsynonymous
186407	NTHI477_RS00895	1164	C	T	synonymous
186425	NTHI477_RS00895	1182	C	T	synonymous
186443	NTHI477_RS00895	1200	C	T	synonymous
186452	NTHI477_RS00895	1209	T	C	synonymous
186455	NTHI477_RS00895	1212	A	G	synonymous
186458	NTHI477_RS00895	1215	T	A	synonymous
186458	NTHI477_RS00895	1215	T	C	synonymous
186461	NTHI477_RS00895	1218	T	C	synonymous
186470	NTHI477_RS00895	1227	G	A	synonymous
186482	NTHI477_RS00895	1239	T	G	synonymous
186485	NTHI477_RS00895	1242	G	A	synonymous
186488	NTHI477_RS00895	1245	A	G	synonymous
186500	NTHI477_RS00895	1257	C	T	synonymous
186524	NTHI477_RS00895	1281	T	C	synonymous
186525	NTHI477_RS00895	1282	G	T	nonsynonymous
186526	NTHI477_RS00895	1283	A	C	nonsynonymous
186562	NTHI477_RS00895	1319	C	T	nonsynonymous
186563	NTHI477_RS00895	1320	C	T	synonymous
186569	NTHI477_RS00895	1326	A	G	synonymous
186598	intergenic	intergenic	G	C	intergenic
186663	NTHI477_RS00900	24	C	T	synonymous
186670	NTHI477_RS00900	31	G	T	nonsynonymous
186685	NTHI477_RS00900	46	A	G	nonsynonymous
186690	NTHI477_RS00900	51	T	C	synonymous
186697	NTHI477_RS00900	58	G	A	nonsynonymous
186702	NTHI477_RS00900	63	C	T	synonymous
186704	NTHI477_RS00900	65	A	G	nonsynonymous
186705	NTHI477_RS00900	66	T	C	synonymous
186712	NTHI477_RS00900	73	G	A	nonsynonymous
186713	NTHI477_RS00900	74	T	C	nonsynonymous
186721	NTHI477_RS00900	82	T	A	nonsynonymous
186722	NTHI477_RS00900	83	C	T	nonsynonymous
186729	NTHI477_RS00900	90	G	A	synonymous
186737	NTHI477_RS00900	98	T	C	nonsynonymous
186738	NTHI477_RS00900	99	A	T	nonsynonymous
186744	NTHI477_RS00900	105	A	G	synonymous
186745	NTHI477_RS00900	106	C	G	nonsynonymous
186747	NTHI477_RS00900	108	G	A	synonymous
186768	NTHI477_RS00900	129	C	T	synonymous

186775	NTHI477_RS00900	136	G	T	nonsynonymous
186777	NTHI477_RS00900	138	A	C	synonymous
186792	NTHI477_RS00900	153	C	T	synonymous
186804	NTHI477_RS00900	165	G	A	synonymous
186831	NTHI477_RS00900	192	C	T	synonymous
186843	NTHI477_RS00900	204	T	A	synonymous
186864	NTHI477_RS00900	225	A	G	synonymous
186877	NTHI477_RS00900	238	T	C	nonsynonymous
186882	NTHI477_RS00900	243	A	G	synonymous
186894	NTHI477_RS00900	255	G	A	synonymous
186897	NTHI477_RS00900	258	T	A	synonymous
186900	NTHI477_RS00900	261	G	C	synonymous
186909	NTHI477_RS00900	270	C	A	synonymous
186910	NTHI477_RS00900	271	C	T	nonsynonymous
186915	NTHI477_RS00900	276	G	A	synonymous
186920	NTHI477_RS00900	281	C	G	nonsynonymous
186921	NTHI477_RS00900	282	C	A	synonymous
186924	NTHI477_RS00900	285	A	C	synonymous
186949	NTHI477_RS00900	310	G	A	nonsynonymous
186957	NTHI477_RS00900	318	C	T	synonymous
186976	NTHI477_RS00900	337	G	A	nonsynonymous
187150	intergenic	intergenic	C	A	intergenic
187158	intergenic	intergenic	A	C	intergenic
187198	intergenic	intergenic	A	G	intergenic
187199	intergenic	intergenic	T	C	intergenic
187246	intergenic	intergenic	A	G	intergenic
187252	intergenic	intergenic	C	A	intergenic
187258	intergenic	intergenic	C	T	intergenic
187456	NTHI477_RS00905	109	G	A	nonsynonymous
187553	NTHI477_RS00905	206	T	C	nonsynonymous
187562	NTHI477_RS00905	215	A	C	nonsynonymous
187603	NTHI477_RS00905	256	A	G	nonsynonymous
187638	NTHI477_RS00905	291	G	A	synonymous
187655	NTHI477_RS00905	308	G	C	nonsynonymous
187659	NTHI477_RS00905	312	G	A	synonymous
187662	NTHI477_RS00905	315	G	A	nonsynonymous
187668	NTHI477_RS00905	321	A	G	synonymous
187671	NTHI477_RS00905	324	A	G	synonymous
187671	NTHI477_RS00905	324	A	T	synonymous
187673	NTHI477_RS00905	326	C	T	nonsynonymous
187674	NTHI477_RS00905	327	A	T	synonymous
187685	NTHI477_RS00905	338	C	T	nonsynonymous
187687	NTHI477_RS00905	340	A	G	nonsynonymous
187689	NTHI477_RS00905	342	T	C	synonymous
187692	NTHI477_RS00905	345	A	G	synonymous

187702	NTHI477_RS00905	355	G	A	nonsynonymous
187704	NTHI477_RS00905	357	T	A	synonymous
187707	NTHI477_RS00905	360	A	G	synonymous
187718	NTHI477_RS00905	371	T	C	nonsynonymous
187720	NTHI477_RS00905	373	G	A	nonsynonymous
187721	NTHI477_RS00905	374	G	C	nonsynonymous
187722	NTHI477_RS00905	375	C	T	synonymous
187725	NTHI477_RS00905	378	A	G	synonymous
187728	NTHI477_RS00905	381	G	A	nonsynonymous
187734	NTHI477_RS00905	387	A	G	synonymous
187737	NTHI477_RS00905	390	A	G	synonymous
187739	NTHI477_RS00905	392	C	T	nonsynonymous
187740	NTHI477_RS00905	393	A	T	synonymous
187751	NTHI477_RS00905	404	C	T	nonsynonymous
187753	NTHI477_RS00905	406	A	G	nonsynonymous
187754	NTHI477_RS00905	407	G	C	nonsynonymous
187755	NTHI477_RS00905	408	T	C	synonymous
187758	NTHI477_RS00905	411	G	A	synonymous
187761	NTHI477_RS00905	414	G	A	nonsynonymous
187767	NTHI477_RS00905	420	A	G	synonymous
187770	NTHI477_RS00905	423	T	A	synonymous
187772	NTHI477_RS00905	425	C	T	nonsynonymous
187773	NTHI477_RS00905	426	A	G	synonymous
187773	NTHI477_RS00905	426	A	T	synonymous
187784	NTHI477_RS00905	437	T	C	nonsynonymous
187784	NTHI477_RS00905	437	T	A	nonsynonymous
187786	NTHI477_RS00905	439	G	A	nonsynonymous
187788	NTHI477_RS00905	441	C	T	synonymous
187788	NTHI477_RS00905	441	C	A	synonymous
187793	NTHI477_RS00905	446	T	A	nonsynonymous
187804	NTHI477_RS00905	457	G	A	nonsynonymous
187808	NTHI477_RS00905	461	C	G	nonsynonymous
187815	NTHI477_RS00905	468	G	A	nonsynonymous
187821	NTHI477_RS00905	474	G	C	nonsynonymous
187836	NTHI477_RS00905	489	A	G	synonymous
187855	NTHI477_RS00905	508	G	T	nonsynonymous
187870	NTHI477_RS00905	523	A	G	nonsynonymous
187871	NTHI477_RS00905	524	C	T	nonsynonymous
187933	intergenic	intergenic	T	C	intergenic
187944	intergenic	intergenic	C	T	intergenic
187951	intergenic	intergenic	G	T	intergenic
187953	intergenic	intergenic	T	A	intergenic
187959	NTHI477_RS00910	1362	C	G	nonsynonymous
188004	NTHI477_RS00910	1317	T	C	synonymous
188006	NTHI477_RS00910	1315	G	A	synonymous

188007	NTHI477_RS00910	1314	C	T	synonymous
188058	NTHI477_RS00910	1263	G	A	synonymous
188066	NTHI477_RS00910	1255	C	T	nonsynonymous
188067	NTHI477_RS00910	1254	T	C	synonymous
188076	NTHI477_RS00910	1245	G	A	synonymous
188085	NTHI477_RS00910	1236	G	T	synonymous
188088	NTHI477_RS00910	1233	T	C	synonymous
188094	NTHI477_RS00910	1227	C	T	synonymous
188106	NTHI477_RS00910	1215	G	A	synonymous
188121	NTHI477_RS00910	1200	T	C	synonymous
188174	NTHI477_RS00910	1147	G	T	nonsynonymous
188187	NTHI477_RS00910	1134	G	T	synonymous
188190	NTHI477_RS00910	1131	A	G	synonymous
188193	NTHI477_RS00910	1128	A	G	synonymous
188220	NTHI477_RS00910	1101	G	T	synonymous
188220	NTHI477_RS00910	1101	G	C	synonymous
188250	NTHI477_RS00910	1071	T	A	synonymous
188250	NTHI477_RS00910	1071	T	C	synonymous
188253	NTHI477_RS00910	1068	T	C	synonymous
188253	NTHI477_RS00910	1068	T	G	synonymous
188282	NTHI477_RS00910	1039	C	T	nonsynonymous
188313	NTHI477_RS00910	1008	C	T	synonymous
188316	NTHI477_RS00910	1005	T	C	synonymous
188318	NTHI477_RS00910	1003	G	A	synonymous
188328	NTHI477_RS00910	993	T	G	synonymous
188334	NTHI477_RS00910	987	A	G	synonymous
188338	NTHI477_RS00910	983	G	A	nonsynonymous
188349	NTHI477_RS00910	972	G	A	synonymous
188352	NTHI477_RS00910	969	A	G	synonymous
188358	NTHI477_RS00910	963	C	T	synonymous
188364	NTHI477_RS00910	957	A	G	synonymous
188370	NTHI477_RS00910	951	C	T	synonymous
188370	NTHI477_RS00910	951	C	A	synonymous
188373	NTHI477_RS00910	948	G	A	synonymous
188388	NTHI477_RS00910	933	A	T	synonymous
188391	NTHI477_RS00910	930	G	A	synonymous
188395	NTHI477_RS00910	926	G	C	nonsynonymous
188409	NTHI477_RS00910	912	C	T	synonymous
188412	NTHI477_RS00910	909	T	A	synonymous
188457	NTHI477_RS00910	864	G	A	synonymous
188475	NTHI477_RS00910	846	T	A	synonymous
188523	NTHI477_RS00910	798	G	A	synonymous
188535	NTHI477_RS00910	786	C	T	synonymous
188562	NTHI477_RS00910	759	C	A	synonymous
188565	NTHI477_RS00910	756	G	A	synonymous

188574	NTHI477_RS00910	747	T	C	synonymous
188575	NTHI477_RS00910	746	G	C	nonsynonymous
188577	NTHI477_RS00910	744	T	A	nonsynonymous
188591	NTHI477_RS00910	730	G	A	synonymous
188592	NTHI477_RS00910	729	G	A	synonymous
188607	NTHI477_RS00910	714	C	T	synonymous
188610	NTHI477_RS00910	711	T	C	synonymous
188622	NTHI477_RS00910	699	T	C	synonymous
188661	NTHI477_RS00910	660	T	C	synonymous
188671	NTHI477_RS00910	650	A	G	nonsynonymous
188682	NTHI477_RS00910	639	T	A	synonymous
188702	NTHI477_RS00910	619	C	T	nonsynonymous
188712	NTHI477_RS00910	609	G	A	synonymous
188718	NTHI477_RS00910	603	C	G	synonymous
188719	NTHI477_RS00910	602	G	A	nonsynonymous
188732	NTHI477_RS00910	589	G	T	nonsynonymous
188766	NTHI477_RS00910	555	C	T	synonymous
188772	NTHI477_RS00910	549	G	A	synonymous
188774	NTHI477_RS00910	547	T	C	nonsynonymous
188778	NTHI477_RS00910	543	T	C	synonymous
188787	NTHI477_RS00910	534	T	A	synonymous
188883	NTHI477_RS00910	438	C	T	synonymous
188895	NTHI477_RS00910	426	C	T	synonymous
188902	NTHI477_RS00910	419	A	G	nonsynonymous
188904	NTHI477_RS00910	417	A	G	synonymous
188906	NTHI477_RS00910	415	T	C	nonsynonymous
188913	NTHI477_RS00910	408	C	T	synonymous
188931	NTHI477_RS00910	390	T	C	synonymous
188940	NTHI477_RS00910	381	G	A	synonymous
188968	NTHI477_RS00910	353	A	G	nonsynonymous
188970	NTHI477_RS00910	351	G	A	synonymous
188973	NTHI477_RS00910	348	A	G	synonymous
188981	NTHI477_RS00910	340	T	C	nonsynonymous
188986	NTHI477_RS00910	335	A	G	nonsynonymous
188997	NTHI477_RS00910	324	G	A	synonymous
189006	NTHI477_RS00910	315	T	C	synonymous
189009	NTHI477_RS00910	312	T	A	synonymous
189048	NTHI477_RS00910	273	G	A	synonymous
189060	NTHI477_RS00910	261	T	C	synonymous
189066	NTHI477_RS00910	255	G	A	synonymous
189072	NTHI477_RS00910	249	C	T	synonymous
189075	NTHI477_RS00910	246	A	G	synonymous
189078	NTHI477_RS00910	243	G	A	synonymous
189087	NTHI477_RS00910	234	C	T	synonymous
189090	NTHI477_RS00910	231	G	A	synonymous

189093	NTHI477_RS00910	228	T	A	synonymous
189111	NTHI477_RS00910	210	G	A	synonymous
189118	NTHI477_RS00910	203	A	G	nonsynonymous
189123	NTHI477_RS00910	198	T	A	synonymous
189123	NTHI477_RS00910	198	T	C	synonymous
189129	NTHI477_RS00910	192	T	C	synonymous
189134	NTHI477_RS00910	187	C	T	nonsynonymous
189135	NTHI477_RS00910	186	T	A	synonymous
189135	NTHI477_RS00910	186	T	G	synonymous
189138	NTHI477_RS00910	183	G	A	synonymous
189150	NTHI477_RS00910	171	T	C	synonymous
189151	NTHI477_RS00910	170	G	A	nonsynonymous
189156	NTHI477_RS00910	165	A	G	synonymous
189158	NTHI477_RS00910	163	C	T	nonsynonymous
189162	NTHI477_RS00910	159	T	C	synonymous
189165	NTHI477_RS00910	156	C	T	synonymous
189230	NTHI477_RS00910	91	G	A	nonsynonymous
189234	NTHI477_RS00910	87	A	T	synonymous
189270	NTHI477_RS00910	51	T	C	synonymous
189278	NTHI477_RS00910	43	T	C	nonsynonymous
189282	NTHI477_RS00910	39	C	T	nonsynonymous
189291	NTHI477_RS00910	30	A	C	synonymous
189337	intergenic	intergenic	C	T	intergenic
189354	intergenic	intergenic	A	T	intergenic
189356	intergenic	intergenic	G	A	intergenic
189357	intergenic	intergenic	A	T	intergenic
189362	intergenic	intergenic	A	C	intergenic
189376	intergenic	intergenic	A	G	intergenic
189399	intergenic	intergenic	T	C	intergenic
189400	intergenic	intergenic	T	A	intergenic
189408	intergenic	intergenic	T	G	intergenic
189415	intergenic	intergenic	T	C	intergenic
189416	intergenic	intergenic	A	G	intergenic
189420	intergenic	intergenic	G	T	intergenic
189428	intergenic	intergenic	G	A	intergenic
189468	intergenic	intergenic	A	T	intergenic
189479	intergenic	intergenic	C	T	intergenic
189490	intergenic	intergenic	C	T	intergenic
189494	intergenic	intergenic	T	G	intergenic
189511	intergenic	intergenic	G	A	intergenic
189512	intergenic	intergenic	C	T	intergenic
189513	intergenic	intergenic	G	A	intergenic
189514	intergenic	intergenic	C	T	intergenic
189546	intergenic	intergenic	T	C	intergenic
189577	intergenic	intergenic	T	A	intergenic

189606	intergenic	intergenic	T	C	intergenic
189612	intergenic	intergenic	C	T	intergenic
189617	intergenic	intergenic	G	T	intergenic
189621	intergenic	intergenic	A	C	intergenic
189622	intergenic	intergenic	A	G	intergenic
189640	intergenic	intergenic	A	G	intergenic
189643	intergenic	intergenic	C	T	intergenic
189668	intergenic	intergenic	G	A	intergenic
189704	intergenic	intergenic	T	C	intergenic
189731	intergenic	intergenic	A	C	intergenic
189740	intergenic	intergenic	A	G	intergenic
190146	NTHI477_RS09495	69	A	G	synonymous
190152	NTHI477_RS09495	75	T	G	synonymous
190156	NTHI477_RS09495	79	A	G	nonsynonymous
190159	NTHI477_RS09495	82	A	C	nonsynonymous
190175	NTHI477_RS09495	98	G	A	nonsynonymous
190176	NTHI477_RS09495	99	C	T	synonymous
190186	NTHI477_RS09495	109	T	C	synonymous
190198	NTHI477_RS09495	121	T	A	nonsynonymous
190200	NTHI477_RS09495	123	A	T	nonsynonymous
190203	NTHI477_RS09495	126	G	T	synonymous
190203	NTHI477_RS09495	126	G	A	synonymous
190206	NTHI477_RS09495	129	A	G	synonymous
190209	NTHI477_RS09495	132	A	G	synonymous
190215	NTHI477_RS09495	138	T	C	synonymous
190227	NTHI477_RS09495	150	T	C	synonymous
190236	NTHI477_RS09495	159	A	G	synonymous
190239	NTHI477_RS09495	162	A	G	synonymous
190248	NTHI477_RS09495	171	C	A	synonymous
190262	NTHI477_RS09495	185	T	A	nonsynonymous
190263	NTHI477_RS09495	186	A	G	synonymous
190263	NTHI477_RS09495	186	A	C	synonymous
190266	NTHI477_RS09495	189	G	A	synonymous
190269	NTHI477_RS09495	192	A	T	synonymous
190275	NTHI477_RS09495	198	G	C	synonymous
190278	NTHI477_RS09495	201	T	C	synonymous
190299	NTHI477_RS09495	222	T	A	synonymous
190301	NTHI477_RS09495	224	T	C	nonsynonymous
190308	NTHI477_RS09495	231	C	T	synonymous
190317	NTHI477_RS09495	240	T	C	synonymous
190319	NTHI477_RS09495	242	G	T	nonsynonymous
190321	NTHI477_RS09495	244	C	T	nonsynonymous
190323	NTHI477_RS09495	246	T	C	synonymous
190325	NTHI477_RS09495	248	A	C	nonsynonymous
190329	NTHI477_RS09495	252	G	A	synonymous

190330	NTHI477_RS09495	253	G	A	nonsynonymous
190334	NTHI477_RS09495	257	A	C	nonsynonymous
190335	NTHI477_RS09495	258	A	G	synonymous
190344	NTHI477_RS09495	267	C	A	synonymous
190347	NTHI477_RS09495	270	T	C	synonymous
190355	NTHI477_RS09495	278	C	A	nonsynonymous
190360	NTHI477_RS09495	283	A	G	nonsynonymous
190366	NTHI477_RS09495	289	C	T	nonsynonymous
190368	NTHI477_RS09495	291	C	A	nonsynonymous
190369	NTHI477_RS09495	292	G	A	nonsynonymous
190392	NTHI477_RS09495	315	C	T	synonymous
190407	intergenic	intergenic	T	A	intergenic
190417	intergenic	intergenic	C	T	intergenic
190424	intergenic	intergenic	C	T	intergenic
190427	intergenic	intergenic	A	G	intergenic
190431	intergenic	intergenic	T	C	intergenic
190437	intergenic	intergenic	C	T	intergenic
190441	intergenic	intergenic	G	A	intergenic
190445	intergenic	intergenic	A	T	intergenic
190446	intergenic	intergenic	A	T	intergenic
190496	intergenic	intergenic	T	A	intergenic
190498	intergenic	intergenic	A	G	intergenic
190504	intergenic	intergenic	G	A	intergenic
190511	intergenic	intergenic	G	A	intergenic
190525	intergenic	intergenic	C	T	intergenic
190541	intergenic	intergenic	G	A	intergenic
190586	NTHI477_RS00920	40	A	G	nonsynonymous
190627	NTHI477_RS00920	81	C	T	synonymous
190636	NTHI477_RS00920	90	T	C	synonymous
190657	NTHI477_RS00920	111	T	C	synonymous
190679	NTHI477_RS00920	133	C	A	nonsynonymous
190681	NTHI477_RS00920	135	T	C	synonymous
190683	NTHI477_RS00920	137	A	C	nonsynonymous
190695	NTHI477_RS00920	149	A	G	nonsynonymous
190711	NTHI477_RS00920	165	A	G	synonymous
190802	NTHI477_RS00920	256	A	G	nonsynonymous
190807	NTHI477_RS00920	261	C	T	synonymous
190810	NTHI477_RS00920	264	G	T	nonsynonymous
190835	NTHI477_RS00920	289	T	C	nonsynonymous
190844	NTHI477_RS00920	298	C	T	nonsynonymous
190850	NTHI477_RS00920	304	C	T	nonsynonymous
190853	NTHI477_RS00920	307	T	C	nonsynonymous
190873	NTHI477_RS00920	327	A	C	synonymous
190885	NTHI477_RS00920	339	A	G	synonymous
190885	NTHI477_RS00920	339	A	T	synonymous

190888	NTHI477_RS00920	342	T	G	synonymous
190920	NTHI477_RS00920	374	C	G	nonsynonymous
190939	NTHI477_RS00920	393	T	C	synonymous
190942	NTHI477_RS00920	396	T	C	synonymous
190955	NTHI477_RS00920	409	A	G	nonsynonymous
190972	NTHI477_RS00920	426	G	A	synonymous
190981	NTHI477_RS00920	435	G	T	nonsynonymous
191011	NTHI477_RS00920	465	A	G	synonymous
191053	NTHI477_RS00920	507	A	T	synonymous
191053	NTHI477_RS00920	507	A	G	synonymous
191059	NTHI477_RS00920	513	G	A	synonymous
191098	intergenic	intergenic	A	G	intergenic
191111	NTHI477_RS00925	10	C	T	nonsynonymous
191112	NTHI477_RS00925	11	A	G	nonsynonymous
191171	NTHI477_RS00925	70	A	G	nonsynonymous
191173	NTHI477_RS00925	72	G	T	nonsynonymous
191173	NTHI477_RS00925	72	G	A	nonsynonymous
191177	NTHI477_RS00925	76	T	C	synonymous
191181	NTHI477_RS00925	80	A	G	nonsynonymous
191183	NTHI477_RS00925	82	A	G	nonsynonymous
191185	NTHI477_RS00925	84	G	T	nonsynonymous
191192	NTHI477_RS00925	91	T	C	synonymous
191195	NTHI477_RS00925	94	A	C	nonsynonymous
191196	NTHI477_RS00925	95	T	C	nonsynonymous
191197	NTHI477_RS00925	96	G	C	nonsynonymous
191199	NTHI477_RS00925	98	G	C	nonsynonymous
191200	NTHI477_RS00925	99	T	C	synonymous
191202	NTHI477_RS00925	101	A	C	nonsynonymous
191203	NTHI477_RS00925	102	T	A	nonsynonymous
191203	NTHI477_RS00925	102	T	G	nonsynonymous
191205	NTHI477_RS00925	104	G	A	nonsynonymous
191209	NTHI477_RS00925	108	T	C	synonymous
191212	NTHI477_RS00925	111	T	C	synonymous
191222	NTHI477_RS00925	121	C	A	synonymous
191230	NTHI477_RS00925	129	C	T	synonymous
191236	NTHI477_RS00925	135	T	C	synonymous
191242	NTHI477_RS00925	141	T	C	synonymous
191243	NTHI477_RS00925	142	A	G	nonsynonymous
191244	NTHI477_RS00925	143	T	A	nonsynonymous
191245	NTHI477_RS00925	144	T	G	nonsynonymous
191255	NTHI477_RS00925	154	C	A	nonsynonymous
191272	NTHI477_RS00925	171	A	G	synonymous
191310	intergenic	intergenic	G	A	intergenic
191337	intergenic	intergenic	G	A	intergenic
191347	intergenic	intergenic	C	T	intergenic

191404	NTHI477_RS00930	350	G	A	nonsynonymous
191428	NTHI477_RS00930	326	G	T	nonsynonymous
191441	NTHI477_RS00930	313	C	T	nonsynonymous
191646	NTHI477_RS00930	108	T	C	synonymous
191658	NTHI477_RS00930	96	A	G	synonymous
191674	NTHI477_RS00930	80	A	C	nonsynonymous
191742	NTHI477_RS00930	12	G	A	synonymous
191759	intergenic	intergenic	T	G	intergenic
191771	intergenic	intergenic	G	T	intergenic
191773	intergenic	intergenic	A	C	intergenic
191786	intergenic	intergenic	T	G	intergenic
191793	intergenic	intergenic	A	C	intergenic
191794	intergenic	intergenic	A	C	intergenic
191796	intergenic	intergenic	T	C	intergenic
191802	intergenic	intergenic	C	T	intergenic
191802	intergenic	intergenic	C	G	intergenic
191803	intergenic	intergenic	T	G	intergenic
191804	intergenic	intergenic	A	G	intergenic
191806	intergenic	intergenic	T	A	intergenic
191815	intergenic	intergenic	T	G	intergenic
191819	intergenic	intergenic	T	G	intergenic
191856	intergenic	intergenic	C	A	intergenic
191865	intergenic	intergenic	C	T	intergenic
191867	intergenic	intergenic	T	G	intergenic
191964	NTHI477_RS00935	56	C	A	nonsynonymous
191968	NTHI477_RS00935	60	A	C	nonsynonymous
191980	NTHI477_RS00935	72	A	T	nonsynonymous
191981	NTHI477_RS00935	73	A	G	nonsynonymous
192014	NTHI477_RS00935	106	G	C	nonsynonymous
192017	NTHI477_RS00935	109	T	G	nonsynonymous
192053	NTHI477_RS00935	145	A	C	nonsynonymous
192081	NTHI477_RS00935	173	A	C	nonsynonymous
192095	NTHI477_RS00935	187	G	A	nonsynonymous
192103	NTHI477_RS00935	195	T	G	nonsynonymous
192129	NTHI477_RS00935	221	A	C	nonsynonymous
192140	NTHI477_RS00935	232	A	G	nonsynonymous
192144	NTHI477_RS00935	236	T	C	nonsynonymous
192176	NTHI477_RS00935	268	C	T	nonsynonymous
192182	NTHI477_RS00935	274	G	A	nonsynonymous
192239	NTHI477_RS00935	331	A	G	nonsynonymous
192249	NTHI477_RS00935	341	C	G	nonsynonymous
192250	NTHI477_RS00935	342	A	G	synonymous
192267	NTHI477_RS00935	359	T	A	nonsynonymous
192270	NTHI477_RS00935	362	G	A	nonsynonymous
192272	NTHI477_RS00935	364	G	A	nonsynonymous

192273	NTHI477_RS00935	365	C	T	nonsynonymous
192274	NTHI477_RS00935	366	A	T	synonymous
192277	NTHI477_RS00935	369	A	C	synonymous
192282	NTHI477_RS00935	374	C	A	nonsynonymous
192292	NTHI477_RS00935	384	C	T	synonymous
192294	NTHI477_RS00935	386	A	G	nonsynonymous
192301	NTHI477_RS00935	393	A	C	nonsynonymous
192350	NTHI477_RS00935	442	G	C	nonsynonymous
192381	NTHI477_RS00935	473	G	A	nonsynonymous
192396	NTHI477_RS00935	488	C	T	nonsynonymous
192396	NTHI477_RS00940	4	C	T	nonsynonymous
192450	NTHI477_RS00940	58	G	A	nonsynonymous
192485	NTHI477_RS00940	93	G	A	synonymous
192546	NTHI477_RS00940	154	A	T	nonsynonymous
192575	NTHI477_RS00940	183	A	C	nonsynonymous
192599	NTHI477_RS00940	207	C	T	synonymous
192633	NTHI477_RS00940	241	G	A	nonsynonymous
192740	NTHI477_RS00940	348	T	C	synonymous
192867	NTHI477_RS00940	475	A	G	nonsynonymous
192939	intergenic	intergenic	G	A	intergenic
192977	intergenic	intergenic	T	C	intergenic
192988	intergenic	intergenic	C	A	intergenic
192997	intergenic	intergenic	T	C	intergenic
192999	intergenic	intergenic	T	A	intergenic
193006	intergenic	intergenic	A	T	intergenic
193028	intergenic	intergenic	C	T	intergenic
193041	intergenic	intergenic	G	A	intergenic
193139	NTHI477_RS00945	43	T	A	nonsynonymous
193144	NTHI477_RS00945	48	A	T	synonymous
193145	NTHI477_RS00945	49	G	A	nonsynonymous
193176	NTHI477_RS00945	80	C	T	nonsynonymous
193200	NTHI477_RS00945	104	C	T	nonsynonymous
193213	NTHI477_RS00945	117	A	G	synonymous
193222	NTHI477_RS00945	126	T	C	synonymous
193243	NTHI477_RS00945	147	C	T	synonymous
193246	NTHI477_RS00945	150	C	T	synonymous
193252	NTHI477_RS00945	156	T	C	synonymous
193288	NTHI477_RS00945	192	T	C	synonymous
193309	NTHI477_RS00945	213	T	C	synonymous
193315	NTHI477_RS00945	219	C	A	synonymous
193329	NTHI477_RS00945	233	A	G	nonsynonymous
193345	NTHI477_RS00945	249	G	T	nonsynonymous
193355	NTHI477_RS00945	259	C	G	nonsynonymous
193384	NTHI477_RS00945	288	C	T	synonymous
193453	NTHI477_RS00945	357	C	G	synonymous

193456	NTHI477_RS00945	360	T	C	synonymous
193462	NTHI477_RS00945	366	C	T	synonymous
193483	NTHI477_RS00945	387	C	T	synonymous
193519	NTHI477_RS00945	423	A	G	synonymous
193522	NTHI477_RS00945	426	A	G	synonymous
193531	NTHI477_RS00945	435	G	A	synonymous
193546	NTHI477_RS00945	450	A	T	synonymous
193582	NTHI477_RS00945	486	A	G	synonymous
193586	NTHI477_RS00945	490	G	A	nonsynonymous
193594	NTHI477_RS00945	498	G	A	synonymous
193597	NTHI477_RS00945	501	A	G	synonymous
193601	NTHI477_RS00945	505	C	A	nonsynonymous
193621	NTHI477_RS00945	525	A	G	synonymous
193636	NTHI477_RS00945	540	G	A	synonymous
193663	NTHI477_RS00945	567	C	T	synonymous
193666	NTHI477_RS00945	570	C	T	synonymous
193708	NTHI477_RS00945	612	T	C	synonymous
193711	NTHI477_RS00945	615	G	A	synonymous
193738	NTHI477_RS00945	642	A	G	synonymous
193744	NTHI477_RS00945	648	C	T	synonymous
193789	NTHI477_RS00945	693	A	G	synonymous
193807	NTHI477_RS00945	711	T	C	synonymous
193828	NTHI477_RS00945	732	T	C	synonymous
193837	NTHI477_RS00945	741	T	C	synonymous
193837	NTHI477_RS00945	741	T	A	synonymous
193837	NTHI477_RS00945	741	T	G	synonymous
193853	NTHI477_RS00945	757	A	G	nonsynonymous
193855	NTHI477_RS00945	759	C	A	synonymous
193858	NTHI477_RS00945	762	T	A	synonymous
193858	NTHI477_RS00945	762	T	C	synonymous
193864	NTHI477_RS00945	768	C	A	synonymous
193918	NTHI477_RS00945	822	T	C	synonymous
193945	NTHI477_RS00945	849	A	G	synonymous
193963	NTHI477_RS00945	867	C	T	synonymous
193966	NTHI477_RS00945	870	C	T	synonymous
193990	NTHI477_RS00945	894	A	G	synonymous
193993	NTHI477_RS00945	897	A	G	synonymous
193999	NTHI477_RS00945	903	C	T	synonymous
194008	NTHI477_RS00945	912	C	T	synonymous
194014	NTHI477_RS00945	918	C	T	synonymous
194020	NTHI477_RS00945	924	G	A	synonymous
194023	NTHI477_RS00945	927	T	G	synonymous
194023	NTHI477_RS00945	927	T	A	synonymous
194029	NTHI477_RS00945	933	A	T	synonymous
194035	NTHI477_RS00945	939	T	C	synonymous

194041	NTHI477_RS00945	945	C	T	synonymous
194056	NTHI477_RS00945	960	C	T	synonymous
194062	NTHI477_RS00945	966	G	A	synonymous
194071	NTHI477_RS00945	975	C	T	synonymous
194098	NTHI477_RS00945	1002	T	C	synonymous
194107	NTHI477_RS00945	1011	A	T	synonymous
194128	NTHI477_RS00945	1032	T	C	synonymous
194160	intergenic	intergenic	G	A	intergenic
194161	intergenic	intergenic	A	G	intergenic
194163	intergenic	intergenic	A	G	intergenic
194164	intergenic	intergenic	T	G	intergenic
194165	intergenic	intergenic	G	A	intergenic
194166	intergenic	intergenic	T	C	intergenic
194181	NTHI477_RS00950	12	A	G	synonymous
194199	NTHI477_RS00950	30	T	C	synonymous
194211	NTHI477_RS00950	42	T	G	synonymous
194253	NTHI477_RS00950	84	T	C	synonymous
194256	NTHI477_RS00950	87	A	G	synonymous
194265	NTHI477_RS00950	96	T	C	synonymous
194283	NTHI477_RS00950	114	C	T	synonymous
194296	NTHI477_RS00950	127	C	T	synonymous
194298	NTHI477_RS00950	129	A	G	synonymous
194361	NTHI477_RS00950	192	T	C	synonymous
194385	NTHI477_RS00950	216	T	C	synonymous
194388	NTHI477_RS00950	219	C	T	synonymous
194391	NTHI477_RS00950	222	C	T	synonymous
194394	NTHI477_RS00950	225	C	A	synonymous
194394	NTHI477_RS00950	225	C	T	synonymous
194397	NTHI477_RS00950	228	C	T	synonymous
194403	NTHI477_RS00950	234	C	T	synonymous
194406	NTHI477_RS00950	237	T	A	synonymous
194409	NTHI477_RS00950	240	C	G	synonymous
194410	NTHI477_RS00950	241	A	G	nonsynonymous
194415	NTHI477_RS00950	246	T	C	synonymous
194418	NTHI477_RS00950	249	G	T	synonymous
194427	NTHI477_RS00950	258	G	A	synonymous
194446	NTHI477_RS00950	277	A	G	nonsynonymous
194451	NTHI477_RS00950	282	C	T	synonymous
194463	NTHI477_RS00950	294	G	A	synonymous
194464	NTHI477_RS00950	295	T	C	synonymous
194466	NTHI477_RS00950	297	G	T	nonsynonymous
194475	NTHI477_RS00950	306	A	G	synonymous
194508	NTHI477_RS00950	339	A	G	synonymous
194529	NTHI477_RS00950	360	G	C	synonymous
194529	NTHI477_RS00950	360	G	T	synonymous

194541	NTHI477_RS00950	372	C	G	synonymous
194541	NTHI477_RS00950	372	C	T	synonymous
194547	NTHI477_RS00950	378	C	T	synonymous
194559	NTHI477_RS00950	390	A	G	synonymous
194571	NTHI477_RS00950	402	C	T	synonymous
194578	NTHI477_RS00950	409	T	C	synonymous
194580	NTHI477_RS00950	411	G	A	synonymous
194601	NTHI477_RS00950	432	G	A	synonymous
194613	NTHI477_RS00950	444	A	C	synonymous
194616	NTHI477_RS00950	447	C	T	synonymous
194634	NTHI477_RS00950	465	A	T	synonymous
194643	NTHI477_RS00950	474	C	T	synonymous
194652	NTHI477_RS00950	483	T	C	synonymous
194658	NTHI477_RS00950	489	C	T	synonymous
194664	NTHI477_RS00950	495	A	G	synonymous
194667	NTHI477_RS00950	498	G	C	synonymous
194677	NTHI477_RS00950	508	T	C	synonymous
194679	NTHI477_RS00950	510	A	G	synonymous
194685	NTHI477_RS00950	516	A	G	synonymous
194691	NTHI477_RS00950	522	C	A	nonsynonymous
194694	NTHI477_RS00950	525	A	T	synonymous
194706	NTHI477_RS00950	537	A	C	synonymous
194715	NTHI477_RS00950	546	T	C	synonymous
194753	NTHI477_RS00950	584	C	G	nonsynonymous
194781	NTHI477_RS00950	612	T	C	synonymous
194790	NTHI477_RS00950	621	G	A	synonymous
194812	NTHI477_RS00950	643	A	G	nonsynonymous
194813	intergenic	intergenic	A	G	intergenic
194834	intergenic	intergenic	T	C	intergenic
194835	intergenic	intergenic	C	T	intergenic
194865	NTHI477_RS00955	13	C	T	synonymous
194891	NTHI477_RS00955	39	C	T	synonymous
194898	NTHI477_RS00955	46	G	A	nonsynonymous
194903	NTHI477_RS00955	51	T	C	synonymous
194918	NTHI477_RS00955	66	G	A	synonymous
194921	NTHI477_RS00955	69	G	A	synonymous
194924	NTHI477_RS00955	72	C	T	synonymous
194939	NTHI477_RS00955	87	T	C	synonymous
194976	NTHI477_RS00955	124	A	G	nonsynonymous
194993	NTHI477_RS00955	141	A	C	synonymous
195017	NTHI477_RS00955	165	G	A	synonymous
195023	NTHI477_RS00955	171	C	T	synonymous
195029	NTHI477_RS00955	177	A	T	synonymous
195032	NTHI477_RS00955	180	A	C	synonymous
195059	NTHI477_RS00955	207	G	A	synonymous

195071	NTHI477_RS00955	219	C	T	synonymous
195072	NTHI477_RS00955	220	A	G	nonsynonymous
195073	NTHI477_RS00955	221	A	G	nonsynonymous
195074	NTHI477_RS00955	222	T	A	nonsynonymous
195083	NTHI477_RS00955	231	G	C	synonymous
195086	NTHI477_RS00955	234	T	A	synonymous
195092	NTHI477_RS00955	240	T	C	synonymous
195140	NTHI477_RS00955	288	A	G	synonymous
195149	NTHI477_RS00955	297	A	G	synonymous
195155	NTHI477_RS00955	303	G	A	synonymous
195173	NTHI477_RS00955	321	G	A	synonymous
195206	NTHI477_RS00955	354	C	T	synonymous
195221	NTHI477_RS00955	369	T	A	synonymous
195227	NTHI477_RS00955	375	T	C	synonymous
195233	NTHI477_RS00955	381	C	T	synonymous
195251	NTHI477_RS00955	399	C	T	synonymous
195255	NTHI477_RS00955	403	T	C	synonymous
195261	NTHI477_RS00955	409	C	T	synonymous
195281	NTHI477_RS00955	429	G	A	synonymous
195287	NTHI477_RS00955	435	G	A	synonymous
195290	NTHI477_RS00955	438	G	T	synonymous
195290	NTHI477_RS00955	438	G	C	synonymous
195293	NTHI477_RS00955	441	T	A	synonymous
195302	NTHI477_RS00955	450	A	T	synonymous
195303	NTHI477_RS00955	451	A	C	nonsynonymous
195304	NTHI477_RS00955	452	A	G	nonsynonymous
195308	NTHI477_RS00955	456	T	G	synonymous
195314	NTHI477_RS00955	462	A	T	synonymous
195316	NTHI477_RS00955	464	G	T	nonsynonymous
195316	NTHI477_RS00955	464	G	A	nonsynonymous
195317	NTHI477_RS00955	465	G	A	synonymous
195324	NTHI477_RS00955	472	T	C	nonsynonymous
195328	intergenic	intergenic	G	A	intergenic
195330	intergenic	intergenic	A	T	intergenic
195330	intergenic	intergenic	A	C	intergenic
195346	intergenic	intergenic	A	T	intergenic
195352	intergenic	intergenic	T	C	intergenic
195362	intergenic	intergenic	T	C	intergenic
195365	intergenic	intergenic	A	G	intergenic
195374	intergenic	intergenic	G	T	intergenic
195376	intergenic	intergenic	G	A	intergenic
195377	intergenic	intergenic	T	C	intergenic
195383	intergenic	intergenic	G	A	intergenic
195385	intergenic	intergenic	C	T	intergenic
195389	intergenic	intergenic	G	A	intergenic

195392	intergenic	intergenic	T	G	intergenic
195394	intergenic	intergenic	T	C	intergenic
195395	intergenic	intergenic	A	T	intergenic
195397	intergenic	intergenic	G	C	intergenic
195399	intergenic	intergenic	T	G	intergenic
195400	intergenic	intergenic	T	G	intergenic
195401	intergenic	intergenic	A	G	intergenic
195402	intergenic	intergenic	G	A	intergenic
195403	intergenic	intergenic	C	A	intergenic
195410	intergenic	intergenic	A	G	intergenic
195410	intergenic	intergenic	A	T	intergenic
195419	intergenic	intergenic	A	G	intergenic
195427	intergenic	intergenic	G	T	intergenic
195440	intergenic	intergenic	G	T	intergenic
195440	intergenic	intergenic	G	A	intergenic
195441	intergenic	intergenic	A	C	intergenic
195481	intergenic	intergenic	A	G	intergenic
195498	intergenic	intergenic	C	T	intergenic
195516	intergenic	intergenic	C	T	intergenic
195559	intergenic	intergenic	C	T	intergenic
195593	NTHI477_RS09405	33	G	A	synonymous
195614	NTHI477_RS09405	54	G	T	synonymous
195617	NTHI477_RS09405	57	T	C	synonymous
195622	NTHI477_RS09405	62	T	C	nonsynonymous
195623	NTHI477_RS09405	63	C	T	synonymous
195642	NTHI477_RS09405	82	G	A	nonsynonymous
195643	NTHI477_RS09405	83	T	C	nonsynonymous
195644	NTHI477_RS09405	84	G	C	synonymous
195653	NTHI477_RS09405	93	T	C	synonymous
195656	NTHI477_RS09405	96	T	A	synonymous
195665	NTHI477_RS09405	105	T	A	synonymous
195673	NTHI477_RS09405	113	T	C	nonsynonymous
195677	NTHI477_RS09405	117	G	A	synonymous
195680	NTHI477_RS09405	120	T	G	synonymous
195684	NTHI477_RS09405	124	G	A	nonsynonymous
195686	NTHI477_RS09405	126	C	T	synonymous
195694	NTHI477_RS09405	134	T	G	nonsynonymous
195713	NTHI477_RS09405	153	C	T	synonymous
195716	NTHI477_RS09405	156	G	T	synonymous
195761	NTHI477_RS09405	201	C	G	synonymous
195767	NTHI477_RS09405	207	G	A	synonymous
195770	NTHI477_RS09405	210	G	A	synonymous
195771	NTHI477_RS09405	211	C	T	synonymous
195773	NTHI477_RS09405	213	A	G	synonymous
195776	NTHI477_RS09405	216	C	G	synonymous

195778	NTHI477_RS09405	218	C	T	nonsynonymous
195779	NTHI477_RS09405	219	T	C	synonymous
195788	NTHI477_RS09405	228	G	C	synonymous
195801	NTHI477_RS09405	241	C	G	nonsynonymous
195806	NTHI477_RS09405	246	C	T	synonymous
195807	NTHI477_RS09405	247	T	C	synonymous
195812	NTHI477_RS09405	252	C	T	synonymous
195815	NTHI477_RS09405	255	T	G	nonsynonymous
195822	NTHI477_RS09405	262	C	A	nonsynonymous
195848	NTHI477_RS09405	288	T	C	synonymous
195866	NTHI477_RS09405	306	A	C	synonymous
195878	NTHI477_RS09405	318	T	G	nonsynonymous
195885	NTHI477_RS09405	325	A	G	nonsynonymous
195887	NTHI477_RS09405	327	G	A	nonsynonymous
195920	NTHI477_RS09405	360	T	C	synonymous
195925	NTHI477_RS09405	365	T	G	nonsynonymous
195926	NTHI477_RS09405	366	C	T	synonymous
195928	NTHI477_RS09405	368	T	G	nonsynonymous
195935	NTHI477_RS09405	375	T	G	synonymous
195939	NTHI477_RS09405	379	C	T	synonymous
195944	NTHI477_RS09405	384	C	T	synonymous
195966	NTHI477_RS09405	406	C	A	nonsynonymous
195970	NTHI477_RS09405	410	G	C	nonsynonymous
195971	NTHI477_RS09405	411	T	C	synonymous
195974	NTHI477_RS09405	414	C	T	synonymous
195976	NTHI477_RS09405	416	A	C	nonsynonymous
195993	NTHI477_RS09405	433	T	G	nonsynonymous
195995	NTHI477_RS09405	435	T	A	synonymous
195995	NTHI477_RS09405	435	T	G	synonymous
196004	NTHI477_RS09405	444	T	G	nonsynonymous
196010	NTHI477_RS09405	450	G	C	synonymous
196010	NTHI477_RS09405	450	G	T	synonymous
196013	NTHI477_RS09405	453	A	G	synonymous
196019	NTHI477_RS09405	459	A	G	synonymous
196028	NTHI477_RS09405	468	C	T	synonymous
196031	NTHI477_RS09405	471	T	C	synonymous
196034	NTHI477_RS09405	474	C	A	synonymous
196052	NTHI477_RS09405	492	C	T	synonymous
196055	NTHI477_RS09405	495	G	A	synonymous
196056	NTHI477_RS09405	496	C	T	synonymous
196070	NTHI477_RS09405	510	T	C	synonymous
196070	NTHI477_RS09405	510	T	A	synonymous
196085	NTHI477_RS09405	525	G	A	synonymous
196107	NTHI477_RS09405	547	G	A	nonsynonymous
196109	NTHI477_RS09405	549	C	T	synonymous

196115	NTHI477_RS09405	555	T	C	synonymous
196116	NTHI477_RS09405	556	G	A	nonsynonymous
196117	NTHI477_RS09405	557	T	G	nonsynonymous
196124	NTHI477_RS09405	564	G	T	synonymous
196125	NTHI477_RS09405	565	G	T	nonsynonymous
196136	NTHI477_RS09405	576	G	A	synonymous
196142	NTHI477_RS09405	582	G	T	nonsynonymous
196148	NTHI477_RS09405	588	T	G	nonsynonymous
196151	NTHI477_RS09405	591	T	C	synonymous
196202	NTHI477_RS09405	642	C	T	synonymous
196206	NTHI477_RS09405	646	C	T	synonymous
196208	NTHI477_RS09405	648	G	T	synonymous
196210	NTHI477_RS09405	650	C	A	nonsynonymous
196211	NTHI477_RS09405	651	A	T	synonymous
196216	NTHI477_RS09405	656	C	A	nonsynonymous
196217	NTHI477_RS09405	657	T	G	synonymous
196220	NTHI477_RS09405	660	G	T	nonsynonymous
196223	NTHI477_RS09405	663	G	A	synonymous
196232	NTHI477_RS09405	672	G	A	synonymous
196234	NTHI477_RS09405	674	G	T	nonsynonymous
196235	NTHI477_RS09405	675	T	C	synonymous
196235	NTHI477_RS09405	675	T	G	synonymous
196236	NTHI477_RS09405	676	A	T	nonsynonymous
196244	NTHI477_RS09405	684	T	C	synonymous
196250	NTHI477_RS09405	690	C	T	synonymous
196259	NTHI477_RS09405	699	A	C	nonsynonymous
196259	NTHI477_RS09405	699	A	G	synonymous
196262	NTHI477_RS09405	702	G	A	synonymous
196265	NTHI477_RS09405	705	T	C	synonymous
196271	NTHI477_RS09405	711	T	A	synonymous
196277	NTHI477_RS09405	717	C	T	synonymous
196286	NTHI477_RS09405	726	A	G	synonymous
196293	NTHI477_RS09405	733	T	C	synonymous
196305	NTHI477_RS09405	745	A	C	nonsynonymous
196313	NTHI477_RS09405	753	T	C	synonymous
196328	NTHI477_RS09405	768	G	A	synonymous
196337	NTHI477_RS09405	777	C	T	synonymous
196343	NTHI477_RS09405	783	G	A	synonymous
196347	NTHI477_RS09405	787	T	C	synonymous
196361	NTHI477_RS09405	801	A	G	synonymous
196373	NTHI477_RS09405	813	A	G	synonymous
196382	NTHI477_RS09405	822	C	T	synonymous
196416	NTHI477_RS09405	856	G	T	nonsynonymous
196424	NTHI477_RS09405	864	C	A	synonymous
196427	NTHI477_RS09405	867	A	G	synonymous

196439	NTHI477_RS09405	879	A	G	synonymous
196443	NTHI477_RS09405	883	C	T	synonymous
196448	NTHI477_RS09405	888	A	G	synonymous
196454	NTHI477_RS09405	894	C	T	synonymous
196458	NTHI477_RS09405	898	T	A	nonsynonymous
196463	NTHI477_RS09405	903	G	A	synonymous
196477	NTHI477_RS09405	917	A	G	synonymous
196481	intergenic	intergenic	T	G	intergenic
196486	intergenic	intergenic	T	G	intergenic
196496	intergenic	intergenic	C	A	intergenic
196497	intergenic	intergenic	G	C	intergenic
196505	intergenic	intergenic	T	A	intergenic
196518	intergenic	intergenic	A	C	intergenic
196538	intergenic	intergenic	A	G	intergenic
196538	intergenic	intergenic	A	T	intergenic
196548	NTHI477_RS00965	321	C	G	nonsynonymous
196569	NTHI477_RS00965	300	T	C	synonymous
196572	NTHI477_RS00965	297	A	G	synonymous
196611	NTHI477_RS00965	258	T	G	synonymous
196619	NTHI477_RS00965	250	C	T	nonsynonymous
196632	NTHI477_RS00965	237	C	T	synonymous
196634	NTHI477_RS00965	235	C	T	nonsynonymous
196644	NTHI477_RS00965	225	T	G	synonymous
196644	NTHI477_RS00965	225	T	A	synonymous
196653	NTHI477_RS00965	216	C	T	synonymous
196667	NTHI477_RS00965	202	C	T	nonsynonymous
196671	NTHI477_RS00965	198	A	T	synonymous
196674	NTHI477_RS00965	195	A	G	synonymous
196680	NTHI477_RS00965	189	C	T	synonymous
196706	NTHI477_RS00965	163	C	T	nonsynonymous
196713	NTHI477_RS00965	156	A	G	synonymous
196731	NTHI477_RS00965	138	A	G	synonymous
196738	NTHI477_RS00965	131	T	C	nonsynonymous
196754	NTHI477_RS00965	115	G	A	synonymous
196770	NTHI477_RS00965	99	G	A	synonymous
196772	NTHI477_RS00965	97	C	A	nonsynonymous
196773	NTHI477_RS00965	96	G	A	synonymous
196773	NTHI477_RS00965	96	G	T	synonymous
196785	NTHI477_RS00965	84	A	G	synonymous
196791	NTHI477_RS00965	78	T	C	synonymous
196839	NTHI477_RS00965	30	G	T	synonymous
196870	intergenic	intergenic	A	G	intergenic
196872	intergenic	intergenic	G	T	intergenic
196884	intergenic	intergenic	G	A	intergenic
196891	intergenic	intergenic	A	G	intergenic

196904	intergenic	intergenic	A	G	intergenic
196913	intergenic	intergenic	A	T	intergenic
196926	intergenic	intergenic	T	G	intergenic
196930	intergenic	intergenic	G	C	intergenic
196942	NTHI477_RS00970	724	C	T	nonsynonymous
196967	NTHI477_RS00970	699	G	A	synonymous
196976	NTHI477_RS00970	690	C	T	synonymous
196985	NTHI477_RS00970	681	A	G	synonymous
196997	NTHI477_RS00970	669	T	C	synonymous
197003	NTHI477_RS00970	663	G	T	synonymous
197009	NTHI477_RS00970	657	T	C	synonymous
197061	NTHI477_RS00970	605	G	C	nonsynonymous
197062	NTHI477_RS00970	604	T	A	nonsynonymous
197063	NTHI477_RS00970	603	G	T	synonymous
197072	NTHI477_RS00970	594	G	A	synonymous
197084	NTHI477_RS00970	582	T	C	synonymous
197096	NTHI477_RS00970	570	A	T	synonymous
197102	NTHI477_RS00970	564	T	C	synonymous
197108	NTHI477_RS00970	558	G	A	synonymous
197116	NTHI477_RS00970	550	C	T	nonsynonymous
197123	NTHI477_RS00970	543	T	C	synonymous
197126	NTHI477_RS00970	540	A	G	synonymous
197135	NTHI477_RS00970	531	A	G	synonymous
197138	NTHI477_RS00970	528	G	T	synonymous
197143	NTHI477_RS00970	523	C	T	nonsynonymous
197145	NTHI477_RS00970	521	C	T	nonsynonymous
197153	NTHI477_RS00970	513	A	G	synonymous
197156	NTHI477_RS00970	510	C	T	synonymous
197162	NTHI477_RS00970	504	C	T	synonymous
197185	NTHI477_RS00970	481	C	T	nonsynonymous
197198	NTHI477_RS00970	468	T	C	synonymous
197204	NTHI477_RS00970	462	C	T	synonymous
197207	NTHI477_RS00970	459	C	A	synonymous
197207	NTHI477_RS00970	459	C	G	synonymous
197207	NTHI477_RS00970	459	C	T	synonymous
197210	NTHI477_RS00970	456	A	G	synonymous
197213	NTHI477_RS00970	453	T	A	synonymous
197219	NTHI477_RS00970	447	C	A	nonsynonymous
197221	NTHI477_RS00970	445	A	G	synonymous
197222	NTHI477_RS00970	444	T	A	synonymous
197225	NTHI477_RS00970	441	A	G	synonymous
197228	NTHI477_RS00970	438	T	C	synonymous
197236	NTHI477_RS00970	430	G	T	nonsynonymous
197244	NTHI477_RS00970	422	T	C	nonsynonymous
197248	NTHI477_RS00970	418	G	A	nonsynonymous

197269	NTHI477_RS00970	397	C	T	nonsynonymous
197270	NTHI477_RS00970	396	C	T	synonymous
197276	NTHI477_RS00970	390	G	A	synonymous
197315	NTHI477_RS00970	351	T	C	synonymous
197318	NTHI477_RS00970	348	T	A	synonymous
197321	NTHI477_RS00970	345	G	C	synonymous
197329	NTHI477_RS00970	337	T	C	nonsynonymous
197330	NTHI477_RS00970	336	T	C	synonymous
197333	NTHI477_RS00970	333	G	A	synonymous
197336	NTHI477_RS00970	330	T	C	synonymous
197342	NTHI477_RS00970	324	A	C	synonymous
197351	NTHI477_RS00970	315	C	T	synonymous
197353	NTHI477_RS00970	313	T	C	nonsynonymous
197357	NTHI477_RS00970	309	A	T	synonymous
197357	NTHI477_RS00970	309	A	G	synonymous
197359	NTHI477_RS00970	307	T	G	nonsynonymous
197360	NTHI477_RS00970	306	G	C	nonsynonymous
197372	NTHI477_RS00970	294	A	G	synonymous
197381	NTHI477_RS00970	285	T	C	synonymous
197383	NTHI477_RS00970	283	C	T	nonsynonymous
197384	NTHI477_RS00970	282	C	G	synonymous
197399	NTHI477_RS00970	267	T	G	synonymous
197402	NTHI477_RS00970	264	T	C	synonymous
197405	NTHI477_RS00970	261	T	C	synonymous
197408	NTHI477_RS00970	258	T	C	synonymous
197411	NTHI477_RS00970	255	G	T	synonymous
197423	NTHI477_RS00970	243	G	A	synonymous
197426	NTHI477_RS00970	240	A	T	synonymous
197426	NTHI477_RS00970	240	A	C	synonymous
197427	NTHI477_RS00970	239	G	A	nonsynonymous
197428	NTHI477_RS00970	238	C	T	nonsynonymous
197432	NTHI477_RS00970	234	G	A	synonymous
197435	NTHI477_RS00970	231	G	A	synonymous
197438	NTHI477_RS00970	228	C	T	synonymous
197440	NTHI477_RS00970	226	A	G	synonymous
197447	NTHI477_RS00970	219	T	G	synonymous
197450	NTHI477_RS00970	216	T	C	synonymous
197459	NTHI477_RS00970	207	C	A	synonymous
197465	NTHI477_RS00970	201	A	C	synonymous
197471	NTHI477_RS00970	195	T	G	synonymous
197471	NTHI477_RS00970	195	T	A	synonymous
197474	NTHI477_RS00970	192	G	A	synonymous
197480	NTHI477_RS00970	186	A	G	synonymous
197483	NTHI477_RS00970	183	C	T	synonymous
197489	NTHI477_RS00970	177	T	C	synonymous

197498	NTHI477_RS00970	168	C	T	synonymous
197498	NTHI477_RS00970	168	C	G	synonymous
197504	NTHI477_RS00970	162	G	T	synonymous
197504	NTHI477_RS00970	162	G	A	synonymous
197510	NTHI477_RS00970	156	T	C	synonymous
197534	NTHI477_RS00970	132	A	G	synonymous
197537	NTHI477_RS00970	129	A	G	synonymous
197537	NTHI477_RS00970	129	A	T	synonymous
197543	NTHI477_RS00970	123	A	G	synonymous
197546	NTHI477_RS00970	120	G	A	synonymous
197557	NTHI477_RS00970	109	C	T	nonsynonymous
197564	NTHI477_RS00970	102	C	T	nonsynonymous
197579	NTHI477_RS00970	87	A	G	synonymous
197581	NTHI477_RS00970	85	G	T	nonsynonymous
197594	NTHI477_RS00970	72	G	A	synonymous
197605	NTHI477_RS00970	61	C	T	nonsynonymous
197648	NTHI477_RS00970	18	G	A	synonymous
197657	NTHI477_RS00970	9	T	C	synonymous
197668	intergenic	intergenic	G	T	intergenic
197700	NTHI477_RS00975	1184	A	G	nonsynonymous
197704	NTHI477_RS00975	1180	C	A	nonsynonymous
197705	NTHI477_RS00975	1179	T	C	synonymous
197711	NTHI477_RS00975	1173	G	A	synonymous
197714	NTHI477_RS00975	1170	C	T	synonymous
197714	NTHI477_RS00975	1170	C	G	synonymous
197718	NTHI477_RS00975	1166	G	A	nonsynonymous
197719	NTHI477_RS00975	1165	A	T	nonsynonymous
197720	NTHI477_RS00975	1164	A	G	synonymous
197729	NTHI477_RS00975	1155	A	T	synonymous
197744	NTHI477_RS00975	1140	C	A	synonymous
197773	NTHI477_RS00975	1111	C	T	nonsynonymous
197774	NTHI477_RS00975	1110	A	G	synonymous
197789	NTHI477_RS00975	1095	G	A	synonymous
197789	NTHI477_RS00975	1095	G	T	synonymous
197795	NTHI477_RS00975	1089	A	G	synonymous
197798	NTHI477_RS00975	1086	T	A	synonymous
197801	NTHI477_RS00975	1083	C	A	synonymous
197801	NTHI477_RS00975	1083	C	T	synonymous
197810	NTHI477_RS00975	1074	T	C	synonymous
197816	NTHI477_RS00975	1068	A	C	nonsynonymous
197828	NTHI477_RS00975	1056	T	C	synonymous
197839	NTHI477_RS00975	1045	G	A	nonsynonymous
197843	NTHI477_RS00975	1041	A	G	synonymous
197849	NTHI477_RS00975	1035	T	C	synonymous
197858	NTHI477_RS00975	1026	G	A	synonymous

197870	NTHI477_RS00975	1014	C	T	synonymous
197875	NTHI477_RS00975	1009	C	A	nonsynonymous
197875	NTHI477_RS00975	1009	C	T	nonsynonymous
197894	NTHI477_RS00975	990	G	A	synonymous
197918	NTHI477_RS00975	966	A	T	nonsynonymous
197936	NTHI477_RS00975	948	C	G	synonymous
197936	NTHI477_RS00975	948	C	T	synonymous
197942	NTHI477_RS00975	942	C	A	synonymous
197951	NTHI477_RS00975	933	G	A	synonymous
197954	NTHI477_RS00975	930	T	G	nonsynonymous
197956	NTHI477_RS00975	928	A	G	synonymous
197963	NTHI477_RS00975	921	T	A	synonymous
197975	NTHI477_RS00975	909	A	G	synonymous
197981	NTHI477_RS00975	903	C	T	synonymous
197990	NTHI477_RS00975	894	A	G	synonymous
198002	NTHI477_RS00975	882	A	G	synonymous
198005	NTHI477_RS00975	879	G	T	synonymous
198011	NTHI477_RS00975	873	A	G	synonymous
198025	NTHI477_RS00975	859	C	A	nonsynonymous
198026	NTHI477_RS00975	858	G	A	synonymous
198056	NTHI477_RS00975	828	G	A	synonymous
198059	NTHI477_RS00975	825	C	G	synonymous
198059	NTHI477_RS00975	825	C	A	synonymous
198070	NTHI477_RS00975	814	C	A	nonsynonymous
198071	NTHI477_RS00975	813	G	A	synonymous
198080	NTHI477_RS00975	804	A	G	synonymous
198080	NTHI477_RS00975	804	A	T	nonsynonymous
198091	NTHI477_RS00975	793	T	G	nonsynonymous
198092	NTHI477_RS00975	792	G	A	synonymous
198095	NTHI477_RS00975	789	G	T	synonymous
198110	NTHI477_RS00975	774	C	T	synonymous
198113	NTHI477_RS00975	771	T	C	synonymous
198119	NTHI477_RS00975	765	G	A	synonymous
198131	NTHI477_RS00975	753	T	A	synonymous
198143	NTHI477_RS00975	741	A	T	synonymous
198146	NTHI477_RS00975	738	G	A	synonymous
198206	NTHI477_RS00975	678	A	C	synonymous
198207	NTHI477_RS00975	677	G	A	nonsynonymous
198226	NTHI477_RS00975	658	G	A	synonymous
198227	NTHI477_RS00975	657	C	T	synonymous
198250	NTHI477_RS00975	634	T	G	nonsynonymous
198251	NTHI477_RS00975	633	A	T	synonymous
198259	NTHI477_RS00975	625	C	T	nonsynonymous
198260	NTHI477_RS00975	624	A	G	synonymous
198260	NTHI477_RS00975	624	A	T	synonymous

198263	NTHI477_RS00975	621	T	C	synonymous
198265	NTHI477_RS00975	619	A	G	synonymous
198275	NTHI477_RS00975	609	A	G	synonymous
198277	NTHI477_RS00975	607	T	A	nonsynonymous
198278	NTHI477_RS00975	606	G	A	synonymous
198284	NTHI477_RS00975	600	G	A	synonymous
198287	NTHI477_RS00975	597	G	A	synonymous
198290	NTHI477_RS00975	594	A	G	synonymous
198293	NTHI477_RS00975	591	A	G	synonymous
198296	NTHI477_RS00975	588	G	T	synonymous
198302	NTHI477_RS00975	582	T	C	synonymous
198305	NTHI477_RS00975	579	A	G	synonymous
198311	NTHI477_RS00975	573	T	C	synonymous
198317	NTHI477_RS00975	567	C	T	synonymous
198341	NTHI477_RS00975	543	G	A	synonymous
198356	NTHI477_RS00975	528	G	A	synonymous
198371	NTHI477_RS00975	513	G	A	synonymous
198400	NTHI477_RS00975	484	G	A	synonymous
198413	NTHI477_RS00975	471	T	C	synonymous
198425	NTHI477_RS00975	459	A	T	synonymous
198427	NTHI477_RS00975	457	G	A	nonsynonymous
198428	NTHI477_RS00975	456	A	G	synonymous
198431	NTHI477_RS00975	453	A	G	synonymous
198434	NTHI477_RS00975	450	C	A	synonymous
198447	NTHI477_RS00975	437	T	G	nonsynonymous
198455	NTHI477_RS00975	429	C	A	synonymous
198461	NTHI477_RS00975	423	G	C	synonymous
198467	NTHI477_RS00975	417	T	C	synonymous
198469	NTHI477_RS00975	415	T	C	nonsynonymous
198479	NTHI477_RS00975	405	G	A	synonymous
198479	NTHI477_RS00975	405	G	T	synonymous
198488	NTHI477_RS00975	396	A	G	synonymous
198500	NTHI477_RS00975	384	A	G	synonymous
198504	NTHI477_RS00975	380	T	G	nonsynonymous
198512	NTHI477_RS00975	372	C	T	synonymous
198527	NTHI477_RS00975	357	G	A	synonymous
198529	NTHI477_RS00975	355	C	T	nonsynonymous
198533	NTHI477_RS00975	351	G	A	synonymous
198551	NTHI477_RS00975	333	C	T	synonymous
198554	NTHI477_RS00975	330	T	G	synonymous
198555	NTHI477_RS00975	329	A	T	nonsynonymous
198572	NTHI477_RS00975	312	T	G	synonymous
198580	NTHI477_RS00975	304	C	T	nonsynonymous
198596	NTHI477_RS00975	288	C	T	synonymous
198599	NTHI477_RS00975	285	G	A	synonymous

198624	NTHI477_RS00975	260	A	T	nonsynonymous
198625	NTHI477_RS00975	259	G	T	nonsynonymous
198630	NTHI477_RS00975	254	T	G	nonsynonymous
198631	NTHI477_RS00975	253	G	A	nonsynonymous
198638	NTHI477_RS00975	246	C	T	synonymous
198639	NTHI477_RS00975	245	T	C	nonsynonymous
198643	NTHI477_RS00975	241	G	T	nonsynonymous
198668	NTHI477_RS00975	216	T	C	synonymous
198674	NTHI477_RS00975	210	T	C	nonsynonymous
198676	NTHI477_RS00975	208	T	C	nonsynonymous
198677	NTHI477_RS00975	207	C	T	synonymous
198683	NTHI477_RS00975	201	C	G	synonymous
198689	NTHI477_RS00975	195	A	G	synonymous
198722	NTHI477_RS00975	162	G	A	synonymous
198737	NTHI477_RS00975	147	A	G	synonymous
198758	NTHI477_RS00975	126	G	A	synonymous
198764	NTHI477_RS00975	120	C	T	synonymous
198769	NTHI477_RS00975	115	T	C	nonsynonymous
198772	NTHI477_RS00975	112	T	C	nonsynonymous
198775	NTHI477_RS00975	109	C	T	nonsynonymous
198798	NTHI477_RS00975	86	G	A	nonsynonymous
198814	NTHI477_RS00975	70	A	G	synonymous
198816	NTHI477_RS00975	68	C	T	nonsynonymous
198817	NTHI477_RS00975	67	T	A	nonsynonymous
198822	NTHI477_RS00975	62	G	A	nonsynonymous
198830	NTHI477_RS00975	54	T	C	synonymous
198838	NTHI477_RS00975	46	G	A	nonsynonymous
198841	NTHI477_RS00975	43	T	G	nonsynonymous
198843	NTHI477_RS00975	41	T	C	nonsynonymous
198848	NTHI477_RS00975	36	G	A	synonymous
198885	intergenic	intergenic	G	C	intergenic
198899	intergenic	intergenic	A	G	intergenic
198907	intergenic	intergenic	A	T	intergenic
198908	intergenic	intergenic	A	T	intergenic
198913	intergenic	intergenic	G	C	intergenic
198918	intergenic	intergenic	T	C	intergenic
198922	intergenic	intergenic	C	A	intergenic
198923	intergenic	intergenic	T	A	intergenic
198930	intergenic	intergenic	A	C	intergenic
198933	intergenic	intergenic	T	A	intergenic
198957	intergenic	intergenic	A	C	intergenic
198984	intergenic	intergenic	T	C	intergenic
199006	intergenic	intergenic	C	T	intergenic
199016	intergenic	intergenic	G	T	intergenic
199019	intergenic	intergenic	G	T	intergenic

199024	NTHI477_RS00980	5	A	G	nonsynonymous
199025	NTHI477_RS00980	6	T	C	synonymous
199036	NTHI477_RS00980	17	T	A	nonsynonymous
199043	NTHI477_RS00980	24	G	A	synonymous
199045	NTHI477_RS00980	26	A	T	nonsynonymous
199070	NTHI477_RS00980	51	G	A	synonymous
199076	NTHI477_RS00980	57	C	T	synonymous
199082	NTHI477_RS00980	63	A	G	synonymous
199088	NTHI477_RS00980	69	C	T	synonymous
199094	NTHI477_RS00980	75	T	A	synonymous
199103	NTHI477_RS00980	84	A	G	synonymous
199113	NTHI477_RS00980	94	A	G	nonsynonymous
199121	NTHI477_RS00980	102	C	T	synonymous
199136	NTHI477_RS00980	117	A	G	synonymous
199142	NTHI477_RS00980	123	T	C	synonymous
199146	NTHI477_RS00980	127	A	C	nonsynonymous
199154	NTHI477_RS00980	135	G	A	synonymous
199160	NTHI477_RS00980	141	G	A	synonymous
199193	NTHI477_RS00980	174	A	G	synonymous
199205	NTHI477_RS00980	186	C	A	synonymous
199241	NTHI477_RS00980	222	C	T	synonymous
199250	NTHI477_RS00980	231	G	A	nonsynonymous
199259	NTHI477_RS00980	240	C	G	synonymous
199268	NTHI477_RS00980	249	T	A	synonymous
199271	NTHI477_RS00980	252	A	G	synonymous
199277	NTHI477_RS00980	258	G	A	synonymous
199283	NTHI477_RS00980	264	C	A	synonymous
199292	NTHI477_RS00980	273	G	A	synonymous
199301	NTHI477_RS00980	282	T	C	synonymous
199304	NTHI477_RS00980	285	C	T	synonymous
199313	NTHI477_RS00980	294	G	A	synonymous
199328	NTHI477_RS00980	309	C	T	synonymous
199331	NTHI477_RS00980	312	G	T	synonymous
199385	NTHI477_RS00980	366	G	A	synonymous
199388	NTHI477_RS00980	369	A	G	synonymous
199409	NTHI477_RS00980	390	G	A	synonymous
199439	NTHI477_RS00980	420	C	T	synonymous
199442	NTHI477_RS00980	423	G	A	synonymous
199451	NTHI477_RS00980	432	G	T	synonymous
199451	NTHI477_RS00980	432	G	A	synonymous
199481	NTHI477_RS00980	462	T	C	synonymous
199496	NTHI477_RS00980	477	A	G	synonymous
199517	NTHI477_RS00980	498	G	A	synonymous
199520	NTHI477_RS00980	501	A	C	synonymous
199529	NTHI477_RS00980	510	T	G	synonymous

199529	NTHI477_RS00980	510	T	C	synonymous
199535	NTHI477_RS00980	516	A	G	synonymous
199556	NTHI477_RS00980	537	T	C	synonymous
199559	NTHI477_RS00980	540	C	T	synonymous
199568	NTHI477_RS00980	549	A	G	synonymous
199586	NTHI477_RS00980	567	A	G	synonymous
199600	NTHI477_RS00980	581	T	C	nonsynonymous
199625	NTHI477_RS00980	606	C	T	synonymous
199661	NTHI477_RS00980	642	T	C	synonymous
199730	intergenic	intergenic	G	A	intergenic
199732	intergenic	intergenic	T	C	intergenic
199740	NTHI477_RS00985	4	C	T	synonymous
199778	NTHI477_RS00985	42	T	C	synonymous
199793	NTHI477_RS00985	57	T	C	synonymous
199794	NTHI477_RS00985	58	A	G	nonsynonymous
199813	NTHI477_RS00985	77	C	T	nonsynonymous
199859	NTHI477_RS00985	123	G	A	synonymous
199881	NTHI477_RS00985	145	C	A	nonsynonymous
199890	NTHI477_RS00985	154	C	T	synonymous
199893	NTHI477_RS00985	157	C	T	nonsynonymous
199899	NTHI477_RS00985	163	G	A	nonsynonymous
199933	NTHI477_RS00985	197	G	A	nonsynonymous
199946	NTHI477_RS00985	210	C	T	synonymous
200015	intergenic	intergenic	A	C	intergenic
200077	intergenic	intergenic	A	C	intergenic
200079	intergenic	intergenic	C	T	intergenic
200126	NTHI477_RS00990	21	A	T	synonymous
200144	NTHI477_RS00990	39	A	G	synonymous
200162	NTHI477_RS00990	57	G	A	synonymous
200188	NTHI477_RS00990	83	G	A	nonsynonymous
200195	NTHI477_RS00990	90	A	C	synonymous
200198	NTHI477_RS00990	93	G	A	synonymous
200219	NTHI477_RS00990	114	T	C	synonymous
200241	NTHI477_RS00990	136	A	G	nonsynonymous
200253	NTHI477_RS00990	148	G	A	nonsynonymous
200255	NTHI477_RS00990	150	T	C	synonymous
200258	NTHI477_RS00990	153	G	A	synonymous
200261	NTHI477_RS00990	156	A	G	synonymous
200264	NTHI477_RS00990	159	A	T	synonymous
200286	NTHI477_RS00990	181	C	A	nonsynonymous
200291	NTHI477_RS00990	186	C	A	nonsynonymous
200330	NTHI477_RS00990	225	A	G	synonymous
200360	NTHI477_RS00990	255	T	C	synonymous
200372	NTHI477_RS00990	267	C	T	synonymous
200375	NTHI477_RS00990	270	C	T	synonymous

200378	NTHI477_RS00990	273	T	C	synonymous
200380	NTHI477_RS00990	275	C	T	nonsynonymous
200384	NTHI477_RS00990	279	C	T	synonymous
200384	NTHI477_RS00990	279	C	A	synonymous
200393	NTHI477_RS00990	288	A	G	synonymous
200408	NTHI477_RS00990	303	G	A	synonymous
200431	NTHI477_RS00990	326	G	A	nonsynonymous
200440	NTHI477_RS00990	335	C	T	nonsynonymous
200443	NTHI477_RS00990	338	G	A	nonsynonymous
200585	NTHI477_RS00990	480	C	T	synonymous
200603	NTHI477_RS00990	498	A	G	synonymous
200624	NTHI477_RS00990	519	A	G	synonymous
200636	NTHI477_RS00990	531	G	A	synonymous
200712	NTHI477_RS00990	607	C	A	nonsynonymous
200819	NTHI477_RS00990	714	A	T	synonymous
200822	NTHI477_RS00990	717	G	A	synonymous
200825	NTHI477_RS00990	720	C	A	nonsynonymous
200826	NTHI477_RS00990	721	A	G	nonsynonymous
200835	NTHI477_RS00990	730	T	C	nonsynonymous
200836	intergenic	intergenic	A	G	intergenic
200863	intergenic	intergenic	G	A	intergenic
200980	NTHI477_RS00995	822	T	C	synonymous
201052	NTHI477_RS00995	750	T	C	synonymous
201187	NTHI477_RS00995	615	G	T	synonymous
201232	NTHI477_RS00995	570	T	C	synonymous
201262	NTHI477_RS00995	540	T	C	synonymous
201268	NTHI477_RS00995	534	G	C	synonymous
201274	NTHI477_RS00995	528	G	A	synonymous
201285	NTHI477_RS00995	517	C	A	nonsynonymous
201310	NTHI477_RS00995	492	T	C	synonymous
201400	NTHI477_RS00995	402	C	A	synonymous
201430	NTHI477_RS00995	372	C	T	synonymous
201439	NTHI477_RS00995	363	T	G	nonsynonymous
201448	NTHI477_RS00995	354	G	A	synonymous
201553	NTHI477_RS00995	249	G	T	synonymous
201565	NTHI477_RS00995	237	A	C	synonymous
201616	NTHI477_RS00995	186	C	A	synonymous
201625	NTHI477_RS00995	177	A	G	synonymous
201658	NTHI477_RS00995	144	T	G	synonymous
201676	NTHI477_RS00995	126	T	G	synonymous
201715	NTHI477_RS00995	87	T	A	synonymous
201730	NTHI477_RS00995	72	G	A	synonymous
201793	NTHI477_RS00995	9	G	A	synonymous
201829	intergenic	intergenic	T	A	intergenic
201835	intergenic	intergenic	T	C	intergenic

201910	intergenic	intergenic	A	G	intergenic
201911	intergenic	intergenic	A	G	intergenic
201912	intergenic	intergenic	G	A	intergenic
201921	intergenic	intergenic	T	G	intergenic
201925	intergenic	intergenic	G	T	intergenic
201937	intergenic	intergenic	C	T	intergenic
201953	intergenic	intergenic	C	A	intergenic
201956	intergenic	intergenic	A	G	intergenic
201960	intergenic	intergenic	T	C	intergenic
201967	intergenic	intergenic	T	C	intergenic
201994	intergenic	intergenic	T	C	intergenic
201995	intergenic	intergenic	T	C	intergenic
201997	intergenic	intergenic	C	T	intergenic
202007	intergenic	intergenic	C	T	intergenic
202011	intergenic	intergenic	A	G	intergenic
202035	intergenic	intergenic	G	A	intergenic
202083	intergenic	intergenic	G	A	intergenic
202092	intergenic	intergenic	C	T	intergenic
202103	intergenic	intergenic	A	T	intergenic
202104	intergenic	intergenic	G	T	intergenic
202176	intergenic	intergenic	C	T	intergenic
202179	intergenic	intergenic	G	T	intergenic
202183	intergenic	intergenic	G	A	intergenic
202438	NTHI477_RS01000	174	C	T	synonymous
202561	NTHI477_RS01000	297	A	C	synonymous
202609	NTHI477_RS01000	345	C	T	synonymous
202647	NTHI477_RS01000	383	C	G	nonsynonymous
202648	NTHI477_RS01000	384	T	A	synonymous
202663	NTHI477_RS01000	399	A	T	synonymous
202796	NTHI477_RS01005	87	T	C	synonymous
202802	NTHI477_RS01005	93	C	T	synonymous
202841	NTHI477_RS01005	132	G	A	synonymous
202868	NTHI477_RS01005	159	A	G	synonymous
202901	NTHI477_RS01005	192	C	T	synonymous
202913	NTHI477_RS01005	204	G	A	synonymous
202937	NTHI477_RS01005	228	G	A	synonymous
202943	NTHI477_RS01005	234	A	G	synonymous
202970	NTHI477_RS01005	261	G	A	synonymous
203042	NTHI477_RS01005	333	A	T	synonymous
203187	intergenic	intergenic	A	G	intergenic
203204	intergenic	intergenic	A	G	intergenic
203214	intergenic	intergenic	A	G	intergenic
203220	intergenic	intergenic	T	G	intergenic
203291	NTHI477_RS01010	9	C	T	synonymous
203292	NTHI477_RS01010	10	G	A	nonsynonymous

203372	NTHI477_RS01010	90	T	A	synonymous
203402	NTHI477_RS01010	120	G	A	synonymous
203417	NTHI477_RS01010	135	A	T	synonymous
203451	NTHI477_RS01010	169	G	A	nonsynonymous
203465	NTHI477_RS01010	183	T	G	synonymous
203504	NTHI477_RS01010	222	T	C	synonymous
203525	NTHI477_RS01010	243	A	G	synonymous
203555	NTHI477_RS01010	273	G	A	synonymous
203555	NTHI477_RS01010	273	G	C	synonymous
203561	NTHI477_RS01010	279	T	C	synonymous
203621	NTHI477_RS01010	339	C	A	synonymous
203659	NTHI477_RS01010	377	T	C	nonsynonymous
203688	NTHI477_RS01010	406	T	C	synonymous
203693	NTHI477_RS01010	411	A	G	synonymous
203798	NTHI477_RS01010	516	T	C	synonymous
203810	NTHI477_RS01010	528	G	A	synonymous
203861	NTHI477_RS01010	579	C	T	synonymous
203873	NTHI477_RS01010	591	A	G	synonymous
203885	NTHI477_RS01010	603	G	T	synonymous
203927	NTHI477_RS01015	7	C	T	nonsynonymous
203962	NTHI477_RS01015	42	T	C	synonymous
203991	NTHI477_RS01015	71	A	G	nonsynonymous
204005	NTHI477_RS01015	85	C	T	synonymous
204028	NTHI477_RS01015	108	G	A	synonymous
204031	NTHI477_RS01015	111	A	T	synonymous
204034	NTHI477_RS01015	114	A	G	synonymous
204088	NTHI477_RS01015	168	T	C	synonymous
204090	NTHI477_RS01015	170	A	G	nonsynonymous
204100	NTHI477_RS01015	180	T	C	synonymous
204136	NTHI477_RS01015	216	C	T	synonymous
204139	NTHI477_RS01015	219	A	G	synonymous
204142	NTHI477_RS01015	222	C	T	synonymous
204151	NTHI477_RS01015	231	T	C	synonymous
204154	NTHI477_RS01015	234	T	C	synonymous
204181	NTHI477_RS01015	261	C	T	synonymous
204185	NTHI477_RS01015	265	G	A	nonsynonymous
204235	NTHI477_RS01015	315	A	T	synonymous
204259	NTHI477_RS01015	339	C	A	nonsynonymous
204262	NTHI477_RS01015	342	C	T	synonymous
204281	NTHI477_RS01015	361	C	T	nonsynonymous
204293	NTHI477_RS01015	373	T	G	nonsynonymous
204364	NTHI477_RS01015	444	T	C	synonymous
204381	intergenic	intergenic	C	T	intergenic
204418	NTHI477_RS01020	1869	G	A	synonymous
204419	NTHI477_RS01020	1868	T	C	nonsynonymous

204421	NTHI477_RS01020	1866	C	T	synonymous
204439	NTHI477_RS01020	1848	A	G	synonymous
204441	NTHI477_RS01020	1846	T	G	nonsynonymous
204497	NTHI477_RS01020	1790	T	C	nonsynonymous
204502	NTHI477_RS01020	1785	T	C	synonymous
204508	NTHI477_RS01020	1779	G	T	synonymous
204529	NTHI477_RS01020	1758	A	G	synonymous
204577	NTHI477_RS01020	1710	T	C	synonymous
204580	NTHI477_RS01020	1707	C	A	synonymous
204583	NTHI477_RS01020	1704	C	A	synonymous
204619	NTHI477_RS01020	1668	T	C	synonymous
204628	NTHI477_RS01020	1659	T	C	synonymous
204637	NTHI477_RS01020	1650	A	G	synonymous
204703	NTHI477_RS01020	1584	T	C	synonymous
204723	NTHI477_RS01020	1564	C	A	nonsynonymous
204725	NTHI477_RS01020	1562	G	A	nonsynonymous
204726	NTHI477_RS01020	1561	C	A	nonsynonymous
204727	NTHI477_RS01020	1560	C	T	synonymous
204728	NTHI477_RS01020	1559	A	T	nonsynonymous
204733	NTHI477_RS01020	1554	C	A	synonymous
204736	NTHI477_RS01020	1551	A	G	synonymous
204745	NTHI477_RS01020	1542	C	T	synonymous
204748	NTHI477_RS01020	1539	G	A	synonymous
204757	NTHI477_RS01020	1530	G	A	synonymous
204781	NTHI477_RS01020	1506	C	T	synonymous
204782	NTHI477_RS01020	1505	T	C	nonsynonymous
204783	NTHI477_RS01020	1504	C	T	nonsynonymous
204805	NTHI477_RS01020	1482	A	G	synonymous
204811	NTHI477_RS01020	1476	T	A	synonymous
204820	NTHI477_RS01020	1467	C	T	synonymous
204859	NTHI477_RS01020	1428	A	G	synonymous
204862	NTHI477_RS01020	1425	A	G	synonymous
204865	NTHI477_RS01020	1422	C	T	synonymous
204872	NTHI477_RS01020	1415	G	A	nonsynonymous
204874	NTHI477_RS01020	1413	A	T	synonymous
204880	NTHI477_RS01020	1407	T	G	synonymous
204916	NTHI477_RS01020	1371	T	C	synonymous
204931	NTHI477_RS01020	1356	T	C	synonymous
204934	NTHI477_RS01020	1353	C	T	synonymous
204940	NTHI477_RS01020	1347	A	G	synonymous
204943	NTHI477_RS01020	1344	G	A	synonymous
204952	NTHI477_RS01020	1335	T	G	synonymous
204958	NTHI477_RS01020	1329	G	A	synonymous
204964	NTHI477_RS01020	1323	C	A	nonsynonymous
204973	NTHI477_RS01020	1314	A	G	synonymous

204975	NTHI477_RS01020	1312	T	G	nonsynonymous
204979	NTHI477_RS01020	1308	A	G	synonymous
204985	NTHI477_RS01020	1302	A	C	synonymous
204991	NTHI477_RS01020	1296	A	G	synonymous
205000	NTHI477_RS01020	1287	C	A	synonymous
205009	NTHI477_RS01020	1278	A	G	synonymous
205018	NTHI477_RS01020	1269	T	C	synonymous
205021	NTHI477_RS01020	1266	C	T	synonymous
205033	NTHI477_RS01020	1254	C	T	synonymous
205041	NTHI477_RS01020	1246	A	G	synonymous
205042	NTHI477_RS01020	1245	T	C	synonymous
205045	NTHI477_RS01020	1242	A	G	synonymous
205069	NTHI477_RS01020	1218	G	A	synonymous
205120	NTHI477_RS01020	1167	A	G	synonymous
205129	NTHI477_RS01020	1158	G	A	synonymous
205143	NTHI477_RS01020	1144	C	A	nonsynonymous
205144	NTHI477_RS01020	1143	A	G	synonymous
205150	NTHI477_RS01020	1137	T	A	synonymous
205153	NTHI477_RS01020	1134	A	G	synonymous
205159	NTHI477_RS01020	1128	A	C	synonymous
205165	NTHI477_RS01020	1122	A	G	synonymous
205183	NTHI477_RS01020	1104	T	C	synonymous
205261	NTHI477_RS01020	1026	A	G	synonymous
205264	NTHI477_RS01020	1023	A	T	synonymous
205294	NTHI477_RS01020	993	G	A	synonymous
205339	NTHI477_RS01020	948	G	A	synonymous
205348	NTHI477_RS01020	939	T	C	synonymous
205366	NTHI477_RS01020	921	G	A	synonymous
205375	NTHI477_RS01020	912	T	A	synonymous
205381	NTHI477_RS01020	906	A	G	synonymous
205396	NTHI477_RS01020	891	C	A	synonymous
205411	NTHI477_RS01020	876	A	G	synonymous
205414	NTHI477_RS01020	873	C	G	synonymous
205414	NTHI477_RS01020	873	C	T	synonymous
205417	NTHI477_RS01020	870	A	G	synonymous
205435	NTHI477_RS01020	852	T	C	synonymous
205447	NTHI477_RS01020	840	T	C	synonymous
205456	NTHI477_RS01020	831	C	T	synonymous
205486	NTHI477_RS01020	801	T	C	synonymous
205487	NTHI477_RS01020	800	G	A	nonsynonymous
205489	NTHI477_RS01020	798	T	A	synonymous
205490	NTHI477_RS01020	797	C	G	nonsynonymous
205501	NTHI477_RS01020	786	C	A	nonsynonymous
205516	NTHI477_RS01020	771	G	A	synonymous
205522	NTHI477_RS01020	765	T	A	synonymous

205534	NTHI477_RS01020	753	A	G	synonymous
205627	NTHI477_RS01020	660	A	C	synonymous
205666	NTHI477_RS01020	621	T	C	synonymous
205675	NTHI477_RS01020	612	G	A	synonymous
205681	NTHI477_RS01020	606	G	T	synonymous
205684	NTHI477_RS01020	603	A	G	synonymous
205690	NTHI477_RS01020	597	A	G	synonymous
205696	NTHI477_RS01020	591	C	T	synonymous
205717	NTHI477_RS01020	570	A	G	synonymous
205777	NTHI477_RS01020	510	C	T	synonymous
205786	NTHI477_RS01020	501	T	C	synonymous
205786	NTHI477_RS01020	501	T	A	synonymous
205819	NTHI477_RS01020	468	C	A	synonymous
205827	NTHI477_RS01020	460	A	C	nonsynonymous
205831	NTHI477_RS01020	456	G	A	synonymous
205837	NTHI477_RS01020	450	G	A	synonymous
205840	NTHI477_RS01020	447	G	T	synonymous
205843	NTHI477_RS01020	444	A	G	synonymous
205860	NTHI477_RS01020	427	T	C	nonsynonymous
205879	NTHI477_RS01020	408	A	G	synonymous
205882	NTHI477_RS01020	405	G	A	synonymous
205885	NTHI477_RS01020	402	T	G	synonymous
205891	NTHI477_RS01020	396	A	G	synonymous
205915	NTHI477_RS01020	372	A	G	synonymous
205966	NTHI477_RS01020	321	G	A	synonymous
205982	NTHI477_RS01020	305	C	T	nonsynonymous
205990	NTHI477_RS01020	297	A	G	synonymous
206005	NTHI477_RS01020	282	C	T	synonymous
206025	NTHI477_RS01020	262	A	G	synonymous
206026	NTHI477_RS01020	261	A	G	synonymous
206053	NTHI477_RS01020	234	C	T	synonymous
206053	NTHI477_RS01020	234	C	A	synonymous
206080	NTHI477_RS01020	207	C	T	synonymous
206083	NTHI477_RS01020	204	C	T	synonymous
206089	NTHI477_RS01020	198	C	T	synonymous
206092	NTHI477_RS01020	195	A	G	synonymous
206098	NTHI477_RS01020	189	C	T	synonymous
206101	NTHI477_RS01020	186	C	T	synonymous
206107	NTHI477_RS01020	180	T	G	synonymous
206107	NTHI477_RS01020	180	T	A	synonymous
206110	NTHI477_RS01020	177	A	T	synonymous
206110	NTHI477_RS01020	177	A	C	synonymous
206113	NTHI477_RS01020	174	T	C	synonymous
206116	NTHI477_RS01020	171	C	T	synonymous
206119	NTHI477_RS01020	168	C	A	synonymous

206122	NTHI477_RS01020	165	A	G	synonymous
206122	NTHI477_RS01020	165	A	T	synonymous
206134	NTHI477_RS01020	153	C	T	synonymous
206143	NTHI477_RS01020	144	T	C	synonymous
206161	NTHI477_RS01020	126	T	A	synonymous
206191	NTHI477_RS01020	96	G	A	synonymous
206209	NTHI477_RS01020	78	A	G	synonymous
206227	NTHI477_RS01020	60	C	T	synonymous
206278	NTHI477_RS01020	9	G	A	synonymous
206298	intergenic	intergenic	G	T	intergenic
206311	intergenic	intergenic	C	G	intergenic
206318	intergenic	intergenic	G	T	intergenic
206323	intergenic	intergenic	G	A	intergenic
206325	intergenic	intergenic	T	C	intergenic
206326	intergenic	intergenic	A	G	intergenic
206348	NTHI477_RS01025	871	T	C	nonsynonymous
206376	NTHI477_RS01025	843	C	T	synonymous
206381	NTHI477_RS01025	838	C	T	nonsynonymous
206409	NTHI477_RS01025	810	A	C	synonymous
206430	NTHI477_RS01025	789	C	T	synonymous
206445	NTHI477_RS01025	774	G	A	synonymous
206475	NTHI477_RS01025	744	A	G	synonymous
206499	NTHI477_RS01025	720	G	A	synonymous
206502	NTHI477_RS01025	717	C	G	synonymous
206505	NTHI477_RS01025	714	C	T	synonymous
206550	NTHI477_RS01025	669	G	A	synonymous
206571	NTHI477_RS01025	648	G	A	synonymous
206582	NTHI477_RS01025	637	C	T	nonsynonymous
206583	NTHI477_RS01025	636	C	T	synonymous
206592	NTHI477_RS01025	627	T	C	synonymous
206610	NTHI477_RS01025	609	C	T	synonymous
206611	NTHI477_RS01025	608	C	T	nonsynonymous
206628	NTHI477_RS01025	591	C	T	synonymous
206652	NTHI477_RS01025	567	C	T	synonymous
206673	NTHI477_RS01025	546	A	G	synonymous
206682	NTHI477_RS01025	537	G	A	synonymous
206691	NTHI477_RS01025	528	A	G	synonymous
206706	NTHI477_RS01025	513	G	A	synonymous
206718	NTHI477_RS01025	501	C	T	synonymous
206727	NTHI477_RS01025	492	C	T	synonymous
206745	NTHI477_RS01025	474	A	G	synonymous
206751	NTHI477_RS01025	468	C	T	synonymous
206757	NTHI477_RS01025	462	A	G	synonymous
206763	NTHI477_RS01025	456	T	C	synonymous
206769	NTHI477_RS01025	450	C	T	synonymous

206772	NTHI477_RS01025	447	A	G	synonymous
206775	NTHI477_RS01025	444	A	T	synonymous
206777	NTHI477_RS01025	442	C	A	nonsynonymous
206784	NTHI477_RS01025	435	C	A	synonymous
206798	NTHI477_RS01025	421	T	C	nonsynonymous
206817	NTHI477_RS01025	402	A	C	synonymous
206819	NTHI477_RS01025	400	C	T	nonsynonymous
206832	NTHI477_RS01025	387	T	A	synonymous
206841	NTHI477_RS01025	378	T	C	synonymous
206865	NTHI477_RS01025	354	C	T	synonymous
206868	NTHI477_RS01025	351	T	A	synonymous
206868	NTHI477_RS01025	351	T	C	synonymous
206931	NTHI477_RS01025	288	G	A	synonymous
206936	NTHI477_RS01025	283	G	A	synonymous
206940	NTHI477_RS01025	279	A	G	synonymous
206946	NTHI477_RS01025	273	A	G	synonymous
206952	NTHI477_RS01025	267	C	T	synonymous
207006	NTHI477_RS01025	213	A	G	synonymous
207009	NTHI477_RS01025	210	A	G	synonymous
207010	NTHI477_RS01025	209	G	A	nonsynonymous
207018	NTHI477_RS01025	201	C	T	synonymous
207051	NTHI477_RS01025	168	T	A	synonymous
207063	NTHI477_RS01025	156	T	C	synonymous
207066	NTHI477_RS01025	153	G	A	synonymous
207072	NTHI477_RS01025	147	G	T	synonymous
207074	NTHI477_RS01025	145	G	T	nonsynonymous
207081	NTHI477_RS01025	138	C	T	synonymous
207087	NTHI477_RS01025	132	G	A	synonymous
207114	NTHI477_RS01025	105	C	G	nonsynonymous
207126	NTHI477_RS01025	93	T	C	synonymous
207133	NTHI477_RS01025	86	T	C	nonsynonymous
207134	NTHI477_RS01025	85	G	A	nonsynonymous
207144	NTHI477_RS01025	75	G	A	synonymous
207146	NTHI477_RS01025	73	T	C	nonsynonymous
207147	NTHI477_RS01025	72	C	T	synonymous
207147	NTHI477_RS01025	72	C	A	nonsynonymous
207169	NTHI477_RS01025	50	C	T	nonsynonymous
207174	NTHI477_RS01025	45	G	A	synonymous
207181	NTHI477_RS01025	38	G	T	nonsynonymous
207181	NTHI477_RS01025	38	G	A	nonsynonymous
207212	NTHI477_RS01025	7	G	T	nonsynonymous
207213	NTHI477_RS01025	6	A	C	synonymous
207229	NTHI477_RS01030	244	C	A	nonsynonymous
207232	NTHI477_RS01030	241	C	T	nonsynonymous
207263	NTHI477_RS01030	210	C	G	synonymous

207263	NTHI477_RS01030	210	C	A	synonymous
207263	NTHI477_RS01030	210	C	T	synonymous
207266	NTHI477_RS01030	207	G	A	synonymous
207272	NTHI477_RS01030	201	T	A	synonymous
207296	NTHI477_RS01030	177	A	G	synonymous
207309	NTHI477_RS01030	164	T	A	nonsynonymous
207326	NTHI477_RS01030	147	C	T	synonymous
207331	NTHI477_RS01030	142	A	G	synonymous
207335	NTHI477_RS01030	138	A	G	synonymous
207338	NTHI477_RS01030	135	G	T	synonymous
207340	NTHI477_RS01030	133	G	A	nonsynonymous
207344	NTHI477_RS01030	129	C	T	synonymous
207368	NTHI477_RS01030	105	C	T	synonymous
207380	NTHI477_RS01030	93	C	T	synonymous
207383	NTHI477_RS01030	90	T	C	synonymous
207389	NTHI477_RS01030	84	G	A	synonymous
207404	NTHI477_RS01030	69	T	A	synonymous
207404	NTHI477_RS01030	69	T	C	synonymous
207406	NTHI477_RS01030	67	T	C	nonsynonymous
207424	NTHI477_RS01030	49	T	G	nonsynonymous
207426	NTHI477_RS01030	47	G	A	nonsynonymous
207431	NTHI477_RS01030	42	T	C	synonymous
207457	NTHI477_RS01030	16	T	C	nonsynonymous
207457	NTHI477_RS01030	16	T	A	nonsynonymous
207458	NTHI477_RS01030	15	G	A	synonymous
207467	NTHI477_RS01030	6	T	C	synonymous
207473	NTHI477_RS01030	0	G	T	synonymous
207474	intergenic	intergenic	G	T	intergenic
207478	intergenic	intergenic	T	C	intergenic
207479	intergenic	intergenic	A	G	intergenic
207501	intergenic	intergenic	A	G	intergenic
207502	intergenic	intergenic	C	T	intergenic
207524	intergenic	intergenic	C	T	intergenic
207605	intergenic	intergenic	C	A	intergenic
207609	intergenic	intergenic	A	G	intergenic
207628	intergenic	intergenic	T	C	intergenic
207640	NTHI477_RS01035	10	A	G	nonsynonymous
207663	NTHI477_RS01035	33	C	T	synonymous
207672	NTHI477_RS01035	42	G	A	synonymous
207678	NTHI477_RS01035	48	A	G	synonymous
207684	NTHI477_RS01035	54	G	T	synonymous
207708	NTHI477_RS01035	78	A	G	synonymous
207753	NTHI477_RS01035	123	C	T	synonymous
207762	NTHI477_RS01035	132	A	G	synonymous
207765	NTHI477_RS01035	135	C	T	synonymous

207766	NTHI477_RS01035	136	C	A	nonsynonymous
207804	NTHI477_RS01035	174	A	G	synonymous
207822	NTHI477_RS01035	192	C	T	synonymous
207825	NTHI477_RS01035	195	C	T	synonymous
207843	NTHI477_RS01035	213	T	C	synonymous
207879	NTHI477_RS01035	249	A	G	synonymous
207891	NTHI477_RS01035	261	A	G	synonymous
207918	NTHI477_RS01035	288	T	G	synonymous
207926	NTHI477_RS01035	296	C	T	nonsynonymous
207927	NTHI477_RS01035	297	A	G	synonymous
207941	NTHI477_RS01035	311	A	G	nonsynonymous
207947	NTHI477_RS01035	317	C	G	nonsynonymous
207948	NTHI477_RS01035	318	C	T	synonymous
207972	NTHI477_RS01035	342	A	G	synonymous
207975	NTHI477_RS01035	345	A	T	synonymous
207993	NTHI477_RS01035	363	A	G	synonymous
208033	NTHI477_RS01035	403	T	C	nonsynonymous
208045	NTHI477_RS01035	415	G	A	nonsynonymous
208059	NTHI477_RS01035	429	G	A	synonymous
208068	NTHI477_RS01035	438	A	T	synonymous
208074	NTHI477_RS01035	444	G	A	synonymous
208077	NTHI477_RS01035	447	T	A	synonymous
208120	NTHI477_RS01035	490	G	A	nonsynonymous
208131	NTHI477_RS01035	501	T	A	synonymous
208134	NTHI477_RS01035	504	T	G	synonymous
208140	NTHI477_RS01035	510	T	C	synonymous
208155	NTHI477_RS01035	525	G	T	synonymous
208164	NTHI477_RS01035	534	G	A	synonymous
208170	NTHI477_RS01035	540	G	A	synonymous
208179	NTHI477_RS01035	549	A	G	synonymous
208185	NTHI477_RS01035	555	G	A	synonymous
208185	NTHI477_RS01035	555	G	T	synonymous
208188	NTHI477_RS01035	558	G	A	synonymous
208203	NTHI477_RS01035	573	C	T	synonymous
208209	NTHI477_RS01035	579	G	A	synonymous
208215	NTHI477_RS01035	585	T	C	synonymous
208219	NTHI477_RS01035	589	C	T	synonymous
208236	NTHI477_RS01035	606	C	T	synonymous
208245	NTHI477_RS01035	615	T	C	synonymous
208266	NTHI477_RS01035	636	T	C	synonymous
208269	NTHI477_RS01035	639	C	T	synonymous
208270	NTHI477_RS01035	640	A	G	nonsynonymous
208274	NTHI477_RS01035	644	T	C	nonsynonymous
208290	NTHI477_RS01035	660	T	A	synonymous
208293	NTHI477_RS01035	663	A	G	synonymous

208326	NTHI477_RS01035	696	C	T	synonymous
208327	NTHI477_RS01035	697	T	G	nonsynonymous
208327	NTHI477_RS01035	697	T	A	nonsynonymous
208341	NTHI477_RS01035	711	A	G	synonymous
208351	NTHI477_RS01035	721	G	A	nonsynonymous
208356	NTHI477_RS01035	726	T	C	synonymous
208365	NTHI477_RS01035	735	G	A	synonymous
208368	NTHI477_RS01035	738	A	G	synonymous
208369	NTHI477_RS01035	739	G	A	nonsynonymous
208374	NTHI477_RS01035	744	T	C	synonymous
208404	NTHI477_RS01035	774	C	A	synonymous
208413	NTHI477_RS01035	783	C	G	synonymous
208413	NTHI477_RS01035	783	C	T	synonymous
208416	NTHI477_RS01035	786	T	G	synonymous
208440	NTHI477_RS01035	810	A	G	synonymous
208440	NTHI477_RS01035	810	A	T	synonymous
208443	NTHI477_RS01035	813	C	A	synonymous
208467	NTHI477_RS01035	837	T	C	synonymous
208479	NTHI477_RS01035	849	A	G	synonymous
208484	NTHI477_RS01035	854	T	C	nonsynonymous
208503	NTHI477_RS01035	873	G	A	synonymous
208509	NTHI477_RS01035	879	A	T	synonymous
208512	NTHI477_RS01035	882	T	C	synonymous
208524	NTHI477_RS01035	894	A	G	synonymous
208551	NTHI477_RS01035	921	C	G	synonymous
208552	NTHI477_RS01035	922	G	T	nonsynonymous
208556	NTHI477_RS01035	926	A	G	nonsynonymous
208572	NTHI477_RS01035	942	C	T	synonymous
208584	NTHI477_RS01035	954	C	G	synonymous
208587	NTHI477_RS01035	957	T	C	synonymous
208605	NTHI477_RS01035	975	C	T	synonymous
208608	NTHI477_RS01035	978	A	G	synonymous
208626	NTHI477_RS01035	996	T	C	synonymous
208629	NTHI477_RS01035	999	C	T	synonymous
208653	NTHI477_RS01035	1023	G	T	synonymous
208665	NTHI477_RS01035	1035	C	T	synonymous
208689	NTHI477_RS01035	1059	C	T	synonymous
208695	NTHI477_RS01035	1065	T	G	synonymous
208698	NTHI477_RS01035	1068	T	G	synonymous
208701	NTHI477_RS01035	1071	T	C	synonymous
208704	NTHI477_RS01035	1074	A	G	synonymous
208707	NTHI477_RS01035	1077	G	A	synonymous
208711	NTHI477_RS01035	1081	C	G	nonsynonymous
208713	NTHI477_RS01035	1083	T	A	synonymous
208714	NTHI477_RS01035	1084	A	G	nonsynonymous

208715	NTHI477_RS01035	1085	A	C	nonsynonymous
208723	NTHI477_RS01035	1093	G	A	nonsynonymous
208726	NTHI477_RS01035	1096	C	T	nonsynonymous
208737	NTHI477_RS01035	1107	T	C	synonymous
208746	NTHI477_RS01035	1116	T	A	synonymous
208753	NTHI477_RS01035	1123	G	T	nonsynonymous
208758	NTHI477_RS01035	1128	A	G	synonymous
208767	NTHI477_RS01035	1137	A	G	synonymous
208791	NTHI477_RS01035	1161	T	C	synonymous
208791	NTHI477_RS01035	1161	T	A	synonymous
208811	NTHI477_RS01035	1181	T	A	nonsynonymous
208818	NTHI477_RS01035	1188	G	A	synonymous
208824	NTHI477_RS01035	1194	C	T	synonymous
208837	NTHI477_RS01035	1207	G	A	nonsynonymous
208842	NTHI477_RS01035	1212	A	G	synonymous
208843	NTHI477_RS01035	1213	G	A	nonsynonymous
208845	NTHI477_RS01035	1215	T	C	synonymous
208872	NTHI477_RS01035	1242	T	C	synonymous
208884	NTHI477_RS01035	1254	A	G	synonymous
208896	NTHI477_RS01035	1266	T	G	nonsynonymous
208903	NTHI477_RS01035	1273	G	A	nonsynonymous
208908	NTHI477_RS01035	1278	A	G	synonymous
208910	NTHI477_RS01035	1280	G	A	nonsynonymous
208912	NTHI477_RS01035	1282	A	G	nonsynonymous
208914	NTHI477_RS01035	1284	A	T	synonymous
208917	NTHI477_RS01035	1287	T	C	synonymous
208920	NTHI477_RS01035	1290	C	A	nonsynonymous
208921	NTHI477_RS01035	1291	G	A	nonsynonymous
208923	NTHI477_RS01035	1293	C	T	synonymous
208926	NTHI477_RS01035	1296	T	G	nonsynonymous
208932	NTHI477_RS01035	1302	G	A	nonsynonymous
208950	NTHI477_RS01035	1320	T	C	synonymous
208965	NTHI477_RS01035	1335	C	T	synonymous
208982	NTHI477_RS01035	1352	G	A	nonsynonymous
209010	NTHI477_RS01035	1380	A	G	synonymous
209016	NTHI477_RS01035	1386	T	C	synonymous
209025	NTHI477_RS01035	1395	A	G	synonymous
209067	NTHI477_RS01035	1437	A	G	synonymous
209079	NTHI477_RS01035	1449	C	T	synonymous
209130	NTHI477_RS01040	28	A	G	nonsynonymous
209135	NTHI477_RS01040	33	A	G	synonymous
209147	NTHI477_RS01040	45	C	T	synonymous
209163	NTHI477_RS01040	61	T	C	synonymous
209219	NTHI477_RS01040	117	A	C	synonymous
209234	NTHI477_RS01040	132	T	A	synonymous

209243	NTHI477_RS01040	141	G	A	synonymous
209267	NTHI477_RS01040	165	A	G	synonymous
209270	NTHI477_RS01040	168	C	T	synonymous
209274	NTHI477_RS01040	172	C	T	nonsynonymous
209275	NTHI477_RS01040	173	A	G	nonsynonymous
209279	NTHI477_RS01040	177	A	T	synonymous
209282	NTHI477_RS01040	180	G	A	synonymous
209297	NTHI477_RS01040	195	T	A	synonymous
209298	NTHI477_RS01040	196	A	G	nonsynonymous
209300	NTHI477_RS01040	198	T	C	synonymous
209303	NTHI477_RS01040	201	A	G	synonymous
209307	NTHI477_RS01040	205	A	C	nonsynonymous
209318	NTHI477_RS01040	216	T	C	synonymous
209323	NTHI477_RS01040	221	C	T	nonsynonymous
209324	NTHI477_RS01040	222	T	G	synonymous
209348	NTHI477_RS01040	246	G	A	synonymous
209354	NTHI477_RS01040	252	C	T	synonymous
209355	NTHI477_RS01040	253	G	A	nonsynonymous
209360	NTHI477_RS01040	258	G	A	synonymous
209363	NTHI477_RS01040	261	A	G	synonymous
209367	NTHI477_RS01040	265	C	A	nonsynonymous
209370	NTHI477_RS01040	268	C	T	synonymous
209376	NTHI477_RS01040	274	A	G	nonsynonymous
209377	NTHI477_RS01040	275	T	C	nonsynonymous
209378	NTHI477_RS01040	276	T	C	synonymous
209384	NTHI477_RS01040	282	A	G	synonymous
209396	NTHI477_RS01040	294	A	G	synonymous
209398	NTHI477_RS01040	296	A	C	nonsynonymous
209402	NTHI477_RS01040	300	T	G	synonymous
209403	NTHI477_RS01040	301	T	C	synonymous
209411	NTHI477_RS01040	309	T	G	nonsynonymous
209414	NTHI477_RS01040	312	C	A	nonsynonymous
209415	NTHI477_RS01040	313	G	C	nonsynonymous
209420	NTHI477_RS01040	318	C	T	synonymous
209420	NTHI477_RS01045	4	C	T	synonymous
209442	NTHI477_RS01045	26	A	C	nonsynonymous
209452	NTHI477_RS01045	36	T	A	synonymous
209464	NTHI477_RS01045	48	A	G	synonymous
209469	NTHI477_RS01045	53	C	A	nonsynonymous
209473	NTHI477_RS01045	57	T	C	synonymous
209477	NTHI477_RS01045	61	T	C	synonymous
209488	NTHI477_RS01045	72	T	C	synonymous
209506	NTHI477_RS01045	90	C	T	synonymous
209530	NTHI477_RS01045	114	T	C	synonymous
209533	NTHI477_RS01045	117	G	A	synonymous

209542	NTHI477_RS01045	126	A	G	synonymous
209548	NTHI477_RS01045	132	C	T	synonymous
209551	NTHI477_RS01045	135	A	G	synonymous
209554	NTHI477_RS01045	138	T	C	synonymous
209593	NTHI477_RS01045	177	C	T	synonymous
209599	NTHI477_RS01045	183	G	A	synonymous
209617	NTHI477_RS01045	201	T	C	synonymous
209629	NTHI477_RS01045	213	T	C	synonymous
209644	NTHI477_RS01045	228	G	A	synonymous
209658	NTHI477_RS01045	242	A	G	nonsynonymous
209678	NTHI477_RS01045	262	G	A	nonsynonymous
209686	NTHI477_RS01045	270	G	A	synonymous
209689	NTHI477_RS01045	273	T	A	synonymous
209689	NTHI477_RS01045	273	T	C	synonymous
209709	NTHI477_RS01045	293	A	C	nonsynonymous
209710	NTHI477_RS01045	294	G	A	synonymous
209712	NTHI477_RS01045	296	G	A	nonsynonymous
209791	NTHI477_RS01045	375	A	T	synonymous
209791	NTHI477_RS01045	375	A	C	synonymous
209794	NTHI477_RS01045	378	G	T	synonymous
209798	NTHI477_RS01045	382	G	T	nonsynonymous
209799	NTHI477_RS01045	383	A	C	nonsynonymous
209821	NTHI477_RS01045	405	C	A	synonymous
209830	NTHI477_RS01045	414	C	T	synonymous
209839	NTHI477_RS01045	423	T	A	synonymous
209854	NTHI477_RS01045	438	G	A	synonymous
209857	NTHI477_RS01045	441	G	A	synonymous
209878	NTHI477_RS01045	462	A	G	synonymous
209893	NTHI477_RS01045	477	T	C	synonymous
209907	NTHI477_RS01045	491	T	G	nonsynonymous
209935	NTHI477_RS01045	519	C	T	synonymous
209938	NTHI477_RS01045	522	A	G	synonymous
209947	NTHI477_RS01045	531	G	A	synonymous
209947	NTHI477_RS01045	531	G	T	synonymous
209955	NTHI477_RS01045	539	A	G	nonsynonymous
209988	NTHI477_RS01045	572	T	C	nonsynonymous
209991	NTHI477_RS01045	575	C	T	nonsynonymous
209992	NTHI477_RS01045	576	A	T	synonymous
209996	NTHI477_RS01045	580	T	C	synonymous
210001	NTHI477_RS01045	585	T	C	synonymous
210003	NTHI477_RS01045	587	G	T	nonsynonymous
210010	NTHI477_RS01045	594	A	G	synonymous
210026	NTHI477_RS01045	610	A	G	nonsynonymous
210054	NTHI477_RS01045	638	C	A	nonsynonymous
210082	NTHI477_RS01045	666	T	G	synonymous

210083	NTHI477_RS01045	667	T	C	synonymous
210100	NTHI477_RS01045	684	T	G	nonsynonymous
210109	NTHI477_RS01045	693	G	A	nonsynonymous
210121	NTHI477_RS01045	705	A	G	synonymous
210143	NTHI477_RS01050	15	C	T	synonymous
210176	NTHI477_RS01050	48	A	G	synonymous
210197	NTHI477_RS01050	69	A	G	synonymous
210203	NTHI477_RS01050	75	G	A	synonymous
210212	NTHI477_RS01050	84	T	C	synonymous
210262	NTHI477_RS01050	134	A	G	nonsynonymous
210281	NTHI477_RS01050	153	A	T	nonsynonymous
210357	intergenic	intergenic	G	A	intergenic
210360	intergenic	intergenic	A	G	intergenic
210376	intergenic	intergenic	A	G	intergenic
210416	intergenic	intergenic	G	T	intergenic
210567	NTHI477_RS01055	132	G	A	synonymous
210745	intergenic	intergenic	A	T	intergenic
210843	NTHI477_RS01060	447	G	A	synonymous
210887	NTHI477_RS01060	403	G	A	synonymous
210937	NTHI477_RS01060	353	G	A	nonsynonymous
210939	NTHI477_RS01060	351	G	A	synonymous
210945	NTHI477_RS01060	345	C	T	synonymous
210954	NTHI477_RS01060	336	C	T	synonymous
210963	NTHI477_RS01060	327	C	T	synonymous
210972	NTHI477_RS01060	318	T	C	synonymous
210975	NTHI477_RS01060	315	T	C	synonymous
210987	NTHI477_RS01060	303	A	C	synonymous
210987	NTHI477_RS01060	303	A	T	synonymous
210990	NTHI477_RS01060	300	T	C	synonymous
210999	NTHI477_RS01060	291	G	A	synonymous
211014	NTHI477_RS01060	276	T	C	synonymous
211017	NTHI477_RS01060	273	T	C	synonymous
211020	NTHI477_RS01060	270	A	G	synonymous
211026	NTHI477_RS01060	264	G	A	synonymous
211029	NTHI477_RS01060	261	C	T	synonymous
211038	NTHI477_RS01060	252	C	T	synonymous
211047	NTHI477_RS01060	243	C	T	synonymous
211065	NTHI477_RS01060	225	T	C	synonymous
211113	NTHI477_RS01060	177	G	A	synonymous
211127	NTHI477_RS01060	163	C	T	nonsynonymous
211140	NTHI477_RS01060	150	C	T	synonymous
211176	NTHI477_RS01060	114	A	G	synonymous
211188	NTHI477_RS01060	102	T	G	synonymous
211191	NTHI477_RS01060	99	C	T	synonymous
211194	NTHI477_RS01060	96	T	C	synonymous

211215	NTHI477_RS01060	75	A	G	synonymous
211236	NTHI477_RS01060	54	A	G	synonymous
211243	NTHI477_RS01060	47	A	G	nonsynonymous
211281	NTHI477_RS01060	9	G	A	synonymous
211299	intergenic	intergenic	C	T	intergenic
211323	intergenic	intergenic	T	C	intergenic
211411	NTHI477_RS01065	64	C	T	nonsynonymous
211426	NTHI477_RS01065	79	G	A	nonsynonymous
211428	NTHI477_RS01065	81	G	A	synonymous
211440	NTHI477_RS01065	93	C	T	synonymous
211442	NTHI477_RS01065	95	A	G	nonsynonymous
211443	NTHI477_RS01065	96	A	C	nonsynonymous
211444	NTHI477_RS01065	97	G	A	nonsynonymous
211463	NTHI477_RS01065	116	T	C	nonsynonymous
211479	NTHI477_RS01065	132	A	G	synonymous
211497	NTHI477_RS01065	150	T	C	synonymous
211497	NTHI477_RS01065	150	T	A	synonymous
211507	NTHI477_RS01065	160	G	A	nonsynonymous
211519	NTHI477_RS01065	172	T	G	nonsynonymous
211533	NTHI477_RS01065	186	A	T	synonymous
211536	NTHI477_RS01065	189	G	A	synonymous
211578	NTHI477_RS01065	231	G	T	nonsynonymous
211585	NTHI477_RS01065	238	T	G	nonsynonymous
211596	NTHI477_RS01065	249	G	A	synonymous
211605	NTHI477_RS01065	258	G	A	synonymous
211638	NTHI477_RS01065	291	C	T	synonymous
211644	NTHI477_RS01065	297	T	C	synonymous
211650	NTHI477_RS01065	303	G	A	synonymous
211662	NTHI477_RS01065	315	G	A	synonymous
211665	NTHI477_RS01065	318	T	A	synonymous
211699	NTHI477_RS01065	352	C	T	nonsynonymous
211701	NTHI477_RS01065	354	T	G	synonymous
211705	NTHI477_RS01065	358	T	C	synonymous
211707	NTHI477_RS01065	360	G	A	synonymous
211740	NTHI477_RS01065	393	A	T	synonymous
211763	NTHI477_RS01065	416	T	C	nonsynonymous
211764	NTHI477_RS01065	417	C	G	nonsynonymous
211794	NTHI477_RS01065	447	T	C	synonymous
211803	NTHI477_RS01065	456	G	A	synonymous
211825	NTHI477_RS01065	478	G	A	nonsynonymous
211842	NTHI477_RS01065	495	G	A	synonymous
211890	NTHI477_RS01065	543	T	C	synonymous
211899	NTHI477_RS01065	552	A	G	synonymous
211902	NTHI477_RS01065	555	C	T	synonymous
211914	NTHI477_RS01065	567	G	T	synonymous

211932	NTHI477_RS01065	585	T	C	synonymous
211939	NTHI477_RS01065	592	C	T	nonsynonymous
211945	NTHI477_RS01065	598	G	A	nonsynonymous
211952	NTHI477_RS01065	605	A	G	nonsynonymous
211959	NTHI477_RS01065	612	A	T	synonymous
211959	NTHI477_RS01065	612	A	G	synonymous
211983	NTHI477_RS01065	636	G	T	synonymous
211992	NTHI477_RS01065	645	G	T	nonsynonymous
212004	NTHI477_RS01065	657	T	C	synonymous
212007	NTHI477_RS01065	660	T	C	synonymous
212008	NTHI477_RS01065	661	G	T	nonsynonymous
212019	NTHI477_RS01065	672	A	G	synonymous
212101	NTHI477_RS01065	754	G	A	nonsynonymous
212118	NTHI477_RS01065	771	C	G	synonymous
212119	NTHI477_RS01065	772	T	C	synonymous
212127	NTHI477_RS01065	780	G	A	synonymous
212157	NTHI477_RS01065	810	C	G	synonymous
212217	NTHI477_RS01065	870	T	C	synonymous
212238	NTHI477_RS01065	891	A	G	synonymous
212253	NTHI477_RS01065	906	T	C	synonymous
212275	NTHI477_RS01065	928	G	A	nonsynonymous
212317	intergenic	intergenic	G	T	intergenic
212332	intergenic	intergenic	T	C	intergenic
212341	intergenic	intergenic	T	A	intergenic
212344	intergenic	intergenic	T	C	intergenic
212351	intergenic	intergenic	T	G	intergenic
212361	intergenic	intergenic	G	A	intergenic
212373	intergenic	intergenic	C	T	intergenic
212376	intergenic	intergenic	T	C	intergenic
212398	intergenic	intergenic	T	C	intergenic
212417	intergenic	intergenic	C	T	intergenic
212418	intergenic	intergenic	T	C	intergenic
212419	intergenic	intergenic	G	A	intergenic
212479	NTHI477_RS01070	769	C	T	nonsynonymous
212487	NTHI477_RS01070	761	G	A	nonsynonymous
212488	NTHI477_RS01070	760	C	T	nonsynonymous
212531	NTHI477_RS01070	717	C	T	synonymous
212534	NTHI477_RS01070	714	C	T	synonymous
212540	NTHI477_RS01070	708	C	T	synonymous
212540	NTHI477_RS01070	708	C	A	synonymous
212552	NTHI477_RS01070	696	T	C	synonymous
212558	NTHI477_RS01070	690	A	C	synonymous
212561	NTHI477_RS01070	687	C	A	synonymous
212561	NTHI477_RS01070	687	C	T	synonymous
212562	NTHI477_RS01070	686	A	G	nonsynonymous

212563	NTHI477_RS01070	685	C	T	nonsynonymous
212564	NTHI477_RS01070	684	C	G	synonymous
212564	NTHI477_RS01070	684	C	T	synonymous
212572	NTHI477_RS01070	676	C	A	nonsynonymous
212573	NTHI477_RS01070	675	T	C	synonymous
212576	NTHI477_RS01070	672	A	C	synonymous
212576	NTHI477_RS01070	672	A	G	synonymous
212576	NTHI477_RS01070	672	A	T	synonymous
212585	NTHI477_RS01070	663	T	A	synonymous
212585	NTHI477_RS01070	663	T	C	synonymous
212591	NTHI477_RS01070	657	T	C	synonymous
212592	NTHI477_RS01070	656	T	G	nonsynonymous
212594	NTHI477_RS01070	654	T	A	synonymous
212597	NTHI477_RS01070	651	C	T	synonymous
212600	NTHI477_RS01070	648	T	A	synonymous
212602	NTHI477_RS01070	646	C	T	nonsynonymous
212627	NTHI477_RS01070	621	C	T	synonymous
212635	NTHI477_RS01070	613	T	A	nonsynonymous
212636	NTHI477_RS01070	612	A	G	synonymous
212639	NTHI477_RS01070	609	T	G	synonymous
212642	NTHI477_RS01070	606	C	T	synonymous
212648	NTHI477_RS01070	600	C	T	synonymous
212660	NTHI477_RS01070	588	G	A	synonymous
212663	NTHI477_RS01070	585	A	G	synonymous
212666	NTHI477_RS01070	582	C	T	synonymous
212681	NTHI477_RS01070	567	G	A	synonymous
212693	NTHI477_RS01070	555	T	A	synonymous
212711	NTHI477_RS01070	537	A	G	synonymous
212732	NTHI477_RS01070	516	G	T	synonymous
212759	NTHI477_RS01070	489	G	A	synonymous
212762	NTHI477_RS01070	486	C	T	synonymous
212762	NTHI477_RS01070	486	C	A	synonymous
212767	NTHI477_RS01070	481	T	C	nonsynonymous
212771	NTHI477_RS01070	477	G	A	synonymous
212774	NTHI477_RS01070	474	C	T	synonymous
212780	NTHI477_RS01070	468	T	G	synonymous
212780	NTHI477_RS01070	468	T	A	synonymous
212783	NTHI477_RS01070	465	T	C	synonymous
212783	NTHI477_RS01070	465	T	A	synonymous
212795	NTHI477_RS01070	453	G	T	synonymous
212798	NTHI477_RS01070	450	G	C	synonymous
212798	NTHI477_RS01070	450	G	T	synonymous
212807	NTHI477_RS01070	441	A	G	synonymous
212819	NTHI477_RS01070	429	A	G	synonymous
212822	NTHI477_RS01070	426	T	C	synonymous

212824	NTHI477_RS01070	424	T	C	nonsynonymous
212838	NTHI477_RS01070	410	C	T	nonsynonymous
212846	NTHI477_RS01070	402	C	G	synonymous
212864	NTHI477_RS01070	384	T	C	synonymous
212870	NTHI477_RS01070	378	T	C	synonymous
212882	NTHI477_RS01070	366	A	C	synonymous
212885	NTHI477_RS01070	363	A	G	synonymous
212897	NTHI477_RS01070	351	A	G	synonymous
212954	NTHI477_RS01070	294	A	G	synonymous
212960	NTHI477_RS01070	288	C	T	synonymous
212969	NTHI477_RS01070	279	T	G	synonymous
213153	NTHI477_RS01070	95	C	T	nonsynonymous
213154	NTHI477_RS01070	94	G	A	nonsynonymous
213188	NTHI477_RS01070	60	C	A	synonymous
213199	NTHI477_RS01070	49	C	T	nonsynonymous
213250	NTHI477_RS01075	1194	T	G	nonsynonymous
213254	NTHI477_RS01075	1190	A	G	nonsynonymous
213260	NTHI477_RS01075	1184	T	C	nonsynonymous
213261	NTHI477_RS01075	1183	T	C	nonsynonymous
213262	NTHI477_RS01075	1182	T	A	nonsynonymous
213283	NTHI477_RS01075	1161	G	A	synonymous
213403	NTHI477_RS01075	1041	A	G	synonymous
213409	NTHI477_RS01075	1035	G	A	synonymous
213415	NTHI477_RS01075	1029	A	G	synonymous
213442	NTHI477_RS01075	1002	G	A	synonymous
213511	NTHI477_RS01075	933	C	T	synonymous
213517	NTHI477_RS01075	927	A	G	synonymous
213520	NTHI477_RS01075	924	G	A	synonymous
213523	NTHI477_RS01075	921	A	G	synonymous
213526	NTHI477_RS01075	918	A	G	synonymous
213532	NTHI477_RS01075	912	C	T	synonymous
213535	NTHI477_RS01075	909	T	A	synonymous
213538	NTHI477_RS01075	906	A	T	synonymous
213543	NTHI477_RS01075	901	T	C	nonsynonymous
213544	NTHI477_RS01075	900	G	A	synonymous
213547	NTHI477_RS01075	897	A	G	synonymous
213550	NTHI477_RS01075	894	G	A	synonymous
213556	NTHI477_RS01075	888	C	T	synonymous
213559	NTHI477_RS01075	885	A	T	synonymous
213561	NTHI477_RS01075	883	T	C	nonsynonymous
213565	NTHI477_RS01075	879	T	A	synonymous
213573	NTHI477_RS01075	871	T	C	nonsynonymous
213574	NTHI477_RS01075	870	A	G	synonymous
213575	NTHI477_RS01075	869	T	G	nonsynonymous
213576	NTHI477_RS01075	868	C	T	nonsynonymous

213583	NTHI477_RS01075	861	A	T	synonymous
213585	NTHI477_RS01075	859	T	A	nonsynonymous
213586	NTHI477_RS01075	858	T	A	synonymous
213589	NTHI477_RS01075	855	C	G	synonymous
213598	NTHI477_RS01075	846	T	C	synonymous
213604	NTHI477_RS01075	840	G	A	synonymous
213607	NTHI477_RS01075	837	G	A	synonymous
213613	NTHI477_RS01075	831	T	A	synonymous
213614	NTHI477_RS01075	830	G	C	nonsynonymous
213617	NTHI477_RS01075	827	G	T	nonsynonymous
213622	NTHI477_RS01075	822	G	A	synonymous
213626	NTHI477_RS01075	818	T	C	nonsynonymous
213630	NTHI477_RS01075	814	G	A	nonsynonymous
213631	NTHI477_RS01075	813	C	T	synonymous
213637	NTHI477_RS01075	807	A	G	synonymous
213640	NTHI477_RS01075	804	A	G	synonymous
213646	NTHI477_RS01075	798	G	A	synonymous
213649	NTHI477_RS01075	795	C	G	synonymous
213650	NTHI477_RS01075	794	G	C	nonsynonymous
213658	NTHI477_RS01075	786	A	G	synonymous
213664	NTHI477_RS01075	780	A	C	synonymous
213667	NTHI477_RS01075	777	C	T	synonymous
213673	NTHI477_RS01075	771	C	T	synonymous
213697	NTHI477_RS01075	747	T	G	synonymous
213699	NTHI477_RS01075	745	A	G	nonsynonymous
213717	NTHI477_RS01075	727	C	T	nonsynonymous
213733	NTHI477_RS01075	711	A	G	synonymous
213736	NTHI477_RS01075	708	C	A	synonymous
213739	NTHI477_RS01075	705	A	G	synonymous
213751	NTHI477_RS01075	693	A	C	synonymous
213760	NTHI477_RS01075	684	G	C	synonymous
213763	NTHI477_RS01075	681	T	A	synonymous
213765	NTHI477_RS01075	679	C	T	nonsynonymous
213777	NTHI477_RS01075	667	G	A	nonsynonymous
213784	NTHI477_RS01075	660	T	C	synonymous
213790	NTHI477_RS01075	654	G	T	synonymous
213800	NTHI477_RS01075	644	C	T	nonsynonymous
213805	NTHI477_RS01075	639	G	A	synonymous
213805	NTHI477_RS01075	639	G	C	synonymous
213808	NTHI477_RS01075	636	T	C	synonymous
213811	NTHI477_RS01075	633	T	A	nonsynonymous
213812	NTHI477_RS01075	632	T	A	nonsynonymous
213813	NTHI477_RS01075	631	C	T	nonsynonymous
213814	NTHI477_RS01075	630	G	T	synonymous
213836	NTHI477_RS01075	608	C	T	nonsynonymous

213837	NTHI477_RS01075	607	T	C	nonsynonymous
213844	NTHI477_RS01075	600	C	A	synonymous
213853	NTHI477_RS01075	591	A	G	synonymous
213859	NTHI477_RS01075	585	A	G	synonymous
213862	NTHI477_RS01075	582	G	A	synonymous
213868	NTHI477_RS01075	576	A	C	synonymous
213871	NTHI477_RS01075	573	G	C	synonymous
213892	NTHI477_RS01075	552	G	A	synonymous
213901	NTHI477_RS01075	543	A	G	synonymous
213922	NTHI477_RS01075	522	C	T	synonymous
213963	NTHI477_RS01075	481	C	G	nonsynonymous
213973	NTHI477_RS01075	471	T	C	synonymous
213979	NTHI477_RS01075	465	A	C	synonymous
213982	NTHI477_RS01075	462	C	G	synonymous
213982	NTHI477_RS01075	462	C	T	synonymous
214000	NTHI477_RS01075	444	G	A	synonymous
214003	NTHI477_RS01075	441	C	T	synonymous
214006	NTHI477_RS01075	438	G	A	synonymous
214009	NTHI477_RS01075	435	T	C	synonymous
214012	NTHI477_RS01075	432	T	C	synonymous
214036	NTHI477_RS01075	408	A	C	synonymous
214051	NTHI477_RS01075	393	A	G	synonymous
214054	NTHI477_RS01075	390	C	T	synonymous
214084	NTHI477_RS01075	360	G	T	synonymous
214087	NTHI477_RS01075	357	C	T	synonymous
214090	NTHI477_RS01075	354	A	C	synonymous
214093	NTHI477_RS01075	351	A	G	synonymous
214099	NTHI477_RS01075	345	C	T	synonymous
214102	NTHI477_RS01075	342	A	C	synonymous
214105	NTHI477_RS01075	339	C	T	synonymous
214129	NTHI477_RS01075	315	C	T	synonymous
214171	NTHI477_RS01075	273	C	T	synonymous
214177	NTHI477_RS01075	267	G	T	synonymous
214237	NTHI477_RS01075	207	A	G	synonymous
214243	NTHI477_RS01075	201	A	G	synonymous
214304	NTHI477_RS01075	140	G	A	nonsynonymous
214336	NTHI477_RS01075	108	A	T	synonymous
214339	NTHI477_RS01075	105	C	T	synonymous
214342	NTHI477_RS01075	102	C	T	synonymous
214348	NTHI477_RS01075	96	G	A	synonymous
214363	NTHI477_RS01075	81	C	T	synonymous
214366	NTHI477_RS01075	78	T	C	synonymous
214387	NTHI477_RS01075	57	G	T	synonymous
214390	NTHI477_RS01075	54	T	C	synonymous
214411	NTHI477_RS01075	33	G	A	synonymous

214487	NTHI477_RS01080	732	C	A	synonymous
214487	NTHI477_RS01080	732	C	T	synonymous
214520	NTHI477_RS01080	699	G	A	synonymous
214532	NTHI477_RS01080	687	C	T	synonymous
214544	NTHI477_RS01080	675	T	C	synonymous
214568	NTHI477_RS01080	651	G	A	synonymous
214601	NTHI477_RS01080	618	G	A	synonymous
214610	NTHI477_RS01080	609	G	C	synonymous
214623	NTHI477_RS01080	596	C	T	nonsynonymous
214655	NTHI477_RS01080	564	G	A	synonymous
214658	NTHI477_RS01080	561	C	T	synonymous
214661	NTHI477_RS01080	558	C	T	synonymous
214676	NTHI477_RS01080	543	A	T	synonymous
214679	NTHI477_RS01080	540	C	G	synonymous
214691	NTHI477_RS01080	528	G	A	synonymous
214694	NTHI477_RS01080	525	T	G	synonymous
214697	NTHI477_RS01080	522	T	A	synonymous
214700	NTHI477_RS01080	519	A	G	synonymous
214706	NTHI477_RS01080	513	T	A	synonymous
214715	NTHI477_RS01080	504	C	A	synonymous
214721	NTHI477_RS01080	498	G	T	synonymous
214724	NTHI477_RS01080	495	T	G	synonymous
214727	NTHI477_RS01080	492	T	C	synonymous
214748	NTHI477_RS01080	471	G	C	synonymous
214754	NTHI477_RS01080	465	A	G	synonymous
214760	NTHI477_RS01080	459	A	G	synonymous
214766	NTHI477_RS01080	453	A	G	synonymous
214778	NTHI477_RS01080	441	A	G	synonymous
214784	NTHI477_RS01080	435	T	C	synonymous
214784	NTHI477_RS01080	435	T	A	synonymous
214787	NTHI477_RS01080	432	A	G	synonymous
214793	NTHI477_RS01080	426	A	G	synonymous
214797	NTHI477_RS01080	422	G	T	nonsynonymous
214799	NTHI477_RS01080	420	A	T	synonymous
214802	NTHI477_RS01080	417	T	C	synonymous
214808	NTHI477_RS01080	411	T	C	synonymous
214820	NTHI477_RS01080	399	G	A	synonymous
214826	NTHI477_RS01080	393	C	G	nonsynonymous
214826	NTHI477_RS01080	393	C	T	synonymous
214834	NTHI477_RS01080	385	A	G	nonsynonymous
214844	NTHI477_RS01080	375	C	G	synonymous
214901	NTHI477_RS01080	318	A	G	synonymous
214929	NTHI477_RS01080	290	G	A	nonsynonymous
214936	NTHI477_RS01080	283	A	G	nonsynonymous
214940	NTHI477_RS01080	279	C	T	synonymous

214997	NTHI477_RS01080	222	G	A	synonymous
214999	NTHI477_RS01080	220	C	T	nonsynonymous
215031	NTHI477_RS01080	188	G	A	nonsynonymous
215048	NTHI477_RS01080	171	G	A	synonymous
215060	NTHI477_RS01080	159	C	T	synonymous
215071	NTHI477_RS01080	148	T	G	nonsynonymous
215099	NTHI477_RS01080	120	C	T	synonymous
215103	NTHI477_RS01080	116	C	A	nonsynonymous
215117	NTHI477_RS01080	102	G	A	synonymous
215143	NTHI477_RS01080	76	C	T	nonsynonymous
215147	NTHI477_RS01080	72	G	A	synonymous
215150	NTHI477_RS01080	69	C	T	synonymous
215153	NTHI477_RS01080	66	T	C	synonymous
215162	NTHI477_RS01080	57	G	A	synonymous
215165	NTHI477_RS01080	54	T	C	synonymous
215168	NTHI477_RS01080	51	C	T	synonymous
215188	NTHI477_RS01080	31	C	T	nonsynonymous
215195	NTHI477_RS01080	24	T	A	synonymous
215207	NTHI477_RS01080	12	A	T	synonymous
215210	NTHI477_RS01080	9	A	G	synonymous
215214	NTHI477_RS01080	5	T	C	nonsynonymous
215238	intergenic	intergenic	G	A	intergenic
215270	intergenic	intergenic	A	G	intergenic
215351	intergenic	intergenic	G	A	intergenic
215355	intergenic	intergenic	A	G	intergenic
215382	intergenic	intergenic	T	C	intergenic
215387	intergenic	intergenic	C	T	intergenic
215436	NTHI477_RS01085	39	C	T	synonymous
215446	NTHI477_RS01085	49	A	G	nonsynonymous
215479	NTHI477_RS01085	82	G	C	nonsynonymous
215494	NTHI477_RS01085	97	C	A	nonsynonymous
215495	NTHI477_RS01085	98	G	A	nonsynonymous
215508	NTHI477_RS01085	111	T	C	synonymous
215512	NTHI477_RS01085	115	G	A	nonsynonymous
215514	NTHI477_RS01085	117	C	A	synonymous
215550	NTHI477_RS01085	153	C	A	synonymous
215568	NTHI477_RS01085	171	A	G	synonymous
215583	NTHI477_RS01085	186	T	C	synonymous
215592	NTHI477_RS01085	195	C	T	synonymous
215610	NTHI477_RS01085	213	A	C	synonymous
215610	NTHI477_RS01085	213	A	T	synonymous
215625	NTHI477_RS01085	228	G	A	synonymous
215628	NTHI477_RS01085	231	A	C	nonsynonymous
215640	NTHI477_RS01085	243	C	T	synonymous
215643	NTHI477_RS01085	246	C	T	synonymous

215649	NTHI477_RS01085	252	A	G	synonymous
215655	NTHI477_RS01085	258	A	G	synonymous
215658	NTHI477_RS01085	261	G	A	synonymous
215670	NTHI477_RS01085	273	A	G	synonymous
215673	NTHI477_RS01085	276	C	T	synonymous
215676	NTHI477_RS01085	279	C	T	synonymous
215685	NTHI477_RS01085	288	A	G	synonymous
215691	NTHI477_RS01085	294	G	T	synonymous
215697	NTHI477_RS01085	300	A	G	synonymous
215724	NTHI477_RS01085	327	T	A	synonymous
215730	NTHI477_RS01085	333	G	A	synonymous
215757	NTHI477_RS01085	360	T	C	synonymous
215766	NTHI477_RS01085	369	A	G	synonymous
215776	NTHI477_RS01085	379	G	T	nonsynonymous
215778	NTHI477_RS01085	381	C	T	synonymous
215784	NTHI477_RS01085	387	C	T	synonymous
215808	NTHI477_RS01085	411	G	C	synonymous
215821	NTHI477_RS01085	424	G	A	nonsynonymous
215823	NTHI477_RS01085	426	C	T	synonymous
215832	NTHI477_RS01085	435	A	T	synonymous
215841	NTHI477_RS01085	444	T	C	synonymous
215850	NTHI477_RS01085	453	G	C	synonymous
215856	NTHI477_RS01085	459	T	C	synonymous
215859	NTHI477_RS01085	462	T	C	synonymous
215862	NTHI477_RS01085	465	G	A	synonymous
215865	NTHI477_RS01085	468	G	A	synonymous
215877	NTHI477_RS01085	480	A	C	synonymous
215880	NTHI477_RS01085	483	C	T	synonymous
215883	NTHI477_RS01085	486	G	A	synonymous
215886	NTHI477_RS01085	489	T	A	synonymous
215913	NTHI477_RS01085	516	A	G	synonymous
215919	NTHI477_RS01085	522	G	A	synonymous
215925	NTHI477_RS01085	528	A	G	synonymous
215943	NTHI477_RS01085	546	A	T	synonymous
215955	NTHI477_RS01085	558	C	T	synonymous
215961	NTHI477_RS01085	564	A	T	synonymous
215973	NTHI477_RS01085	576	T	C	synonymous
215979	NTHI477_RS01085	582	G	A	synonymous
215980	NTHI477_RS01085	583	T	C	synonymous
215982	NTHI477_RS01085	585	A	T	nonsynonymous
215988	NTHI477_RS01085	591	C	T	synonymous
215995	NTHI477_RS01085	598	C	T	nonsynonymous
216003	NTHI477_RS01085	606	A	G	synonymous
216006	NTHI477_RS01085	609	T	C	synonymous
216007	NTHI477_RS01085	610	G	A	nonsynonymous

216019	NTHI477_RS01085	622	A	G	nonsynonymous
216036	NTHI477_RS01085	639	C	T	synonymous
216042	NTHI477_RS01085	645	G	A	synonymous
216051	NTHI477_RS01085	654	G	A	synonymous
216054	NTHI477_RS01085	657	T	C	synonymous
216055	NTHI477_RS01085	658	C	T	nonsynonymous
216063	NTHI477_RS01085	666	C	A	synonymous
216087	NTHI477_RS01085	690	G	A	synonymous
216096	NTHI477_RS01085	699	G	A	synonymous
216097	NTHI477_RS01085	700	T	C	synonymous
216104	NTHI477_RS01085	707	A	G	nonsynonymous
216105	NTHI477_RS01085	708	G	A	synonymous
216144	NTHI477_RS01085	747	T	C	synonymous
216174	NTHI477_RS01085	777	A	G	synonymous
216178	NTHI477_RS01085	781	G	A	nonsynonymous
216179	NTHI477_RS01085	782	A	C	nonsynonymous
216180	NTHI477_RS01085	783	C	A	nonsynonymous
216183	NTHI477_RS01085	786	T	C	synonymous
216186	NTHI477_RS01085	789	T	C	synonymous
216186	NTHI477_RS01085	789	T	A	synonymous
216255	NTHI477_RS01085	858	T	C	synonymous
216258	NTHI477_RS01085	861	C	T	synonymous
216261	NTHI477_RS01085	864	C	T	synonymous
216276	NTHI477_RS01085	879	T	C	synonymous
216282	NTHI477_RS01085	885	C	T	synonymous
216285	NTHI477_RS01085	888	T	C	synonymous
216310	NTHI477_RS01085	913	G	T	nonsynonymous
216342	NTHI477_RS01085	945	T	C	synonymous
216343	NTHI477_RS01085	946	G	A	nonsynonymous
216348	NTHI477_RS01085	951	G	A	synonymous
216358	NTHI477_RS01085	961	T	G	nonsynonymous
216360	NTHI477_RS01085	963	G	A	synonymous
216392	NTHI477_RS01085	995	A	C	nonsynonymous
216395	NTHI477_RS01085	998	A	C	nonsynonymous
216402	NTHI477_RS01085	1005	C	T	synonymous
216407	NTHI477_RS01085	1010	G	A	nonsynonymous
216433	NTHI477_RS01085	1036	T	G	nonsynonymous
216448	intergenic	intergenic	A	C	intergenic
216455	intergenic	intergenic	T	G	intergenic
216525	NTHI477_RS01090	39	A	G	synonymous
216557	NTHI477_RS01090	71	T	C	nonsynonymous
216581	NTHI477_RS01090	95	C	T	nonsynonymous
216615	NTHI477_RS01090	129	C	A	synonymous
216673	NTHI477_RS01090	187	T	G	nonsynonymous
216682	NTHI477_RS01090	196	C	T	nonsynonymous

216696	NTHI477_RS01090	210	C	T	synonymous
216726	NTHI477_RS01090	240	C	A	synonymous
216741	NTHI477_RS01090	255	C	T	synonymous
216780	NTHI477_RS01090	294	C	T	synonymous
216819	NTHI477_RS01090	333	T	C	synonymous
216852	NTHI477_RS01090	366	T	C	synonymous
216888	NTHI477_RS01090	402	C	T	synonymous
216918	NTHI477_RS01090	432	G	A	synonymous
216942	NTHI477_RS01090	456	A	G	synonymous
216945	NTHI477_RS01090	459	A	G	synonymous
216963	NTHI477_RS01090	477	T	C	synonymous
216975	NTHI477_RS01090	489	C	A	synonymous
216987	NTHI477_RS01090	501	G	A	synonymous
216992	NTHI477_RS01090	506	G	A	nonsynonymous
216998	NTHI477_RS01090	512	G	A	nonsynonymous
217020	NTHI477_RS01090	534	T	C	synonymous
217023	NTHI477_RS01090	537	G	A	synonymous
217053	NTHI477_RS01090	567	G	A	synonymous
217081	NTHI477_RS01090	595	T	C	synonymous
217089	NTHI477_RS01090	603	G	A	synonymous
217093	NTHI477_RS01090	607	G	C	nonsynonymous
217095	NTHI477_RS01090	609	T	C	synonymous
217096	NTHI477_RS01090	610	C	T	synonymous
217107	NTHI477_RS01090	621	A	T	synonymous
217117	NTHI477_RS01090	631	A	G	nonsynonymous
217131	intergenic	intergenic	A	G	intergenic
217138	intergenic	intergenic	A	G	intergenic
217169	intergenic	intergenic	A	G	intergenic
217174	intergenic	intergenic	T	C	intergenic
217192	intergenic	intergenic	T	C	intergenic
217193	intergenic	intergenic	T	C	intergenic
217204	intergenic	intergenic	T	C	intergenic
217207	intergenic	intergenic	A	G	intergenic
217208	intergenic	intergenic	A	G	intergenic
217212	NTHI477_RS01095	834	G	A	synonymous
217236	NTHI477_RS01095	810	A	T	synonymous
217239	NTHI477_RS01095	807	T	G	synonymous
217248	NTHI477_RS01095	798	T	C	synonymous
217303	NTHI477_RS01095	743	A	G	nonsynonymous
217496	NTHI477_RS01095	550	A	G	synonymous
217500	NTHI477_RS01095	546	C	T	synonymous
217529	NTHI477_RS01095	517	A	G	nonsynonymous
217554	NTHI477_RS01095	492	A	T	synonymous
217578	NTHI477_RS01095	468	A	C	synonymous
217578	NTHI477_RS01095	468	A	T	synonymous

217581	NTHI477_RS01095	465	C	T	synonymous
217590	NTHI477_RS01095	456	T	A	synonymous
217623	NTHI477_RS01095	423	T	C	synonymous
217629	NTHI477_RS01095	417	A	C	synonymous
217632	NTHI477_RS01095	414	A	T	synonymous
217635	NTHI477_RS01095	411	G	A	synonymous
217640	NTHI477_RS01095	406	C	T	nonsynonymous
217641	NTHI477_RS01095	405	A	G	synonymous
217650	NTHI477_RS01095	396	G	A	synonymous
217662	NTHI477_RS01095	384	A	G	synonymous
217671	NTHI477_RS01095	375	A	G	synonymous
217680	NTHI477_RS01095	366	G	T	synonymous
217683	NTHI477_RS01095	363	A	G	synonymous
217693	NTHI477_RS01095	353	C	T	nonsynonymous
217743	NTHI477_RS01095	303	A	G	synonymous
217743	NTHI477_RS01095	303	A	T	synonymous
217755	NTHI477_RS01095	291	C	G	synonymous
217763	NTHI477_RS01095	283	T	C	nonsynonymous
217779	NTHI477_RS01095	267	G	A	synonymous
217847	NTHI477_RS01095	199	C	T	nonsynonymous
217852	NTHI477_RS01095	194	C	T	nonsynonymous
217869	NTHI477_RS01095	177	C	T	synonymous
217884	NTHI477_RS01095	162	A	G	synonymous
217950	NTHI477_RS01095	96	A	G	synonymous
217958	NTHI477_RS01095	88	T	C	nonsynonymous
217959	NTHI477_RS01095	87	T	C	synonymous
217965	NTHI477_RS01095	81	C	T	synonymous
217967	NTHI477_RS01095	79	T	C	nonsynonymous
217968	NTHI477_RS01095	78	C	A	synonymous
217998	NTHI477_RS01095	48	T	C	synonymous
218003	NTHI477_RS01095	43	T	C	nonsynonymous
218004	NTHI477_RS01095	42	G	A	synonymous
218022	NTHI477_RS01095	24	T	A	synonymous
218024	NTHI477_RS01095	22	C	T	nonsynonymous
218027	NTHI477_RS01095	19	T	C	nonsynonymous
218031	NTHI477_RS01095	15	C	T	synonymous
218037	NTHI477_RS01095	9	C	T	synonymous
218067	intergenic	intergenic	A	G	intergenic
218085	intergenic	intergenic	T	A	intergenic
218086	intergenic	intergenic	G	A	intergenic
218102	intergenic	intergenic	G	T	intergenic
218143	intergenic	intergenic	T	C	intergenic
218151	intergenic	intergenic	G	A	intergenic
218154	intergenic	intergenic	A	G	intergenic
218194	intergenic	intergenic	A	G	intergenic

218198	intergenic	intergenic	T	C	intergenic
218211	intergenic	intergenic	T	C	intergenic
218219	intergenic	intergenic	G	A	intergenic
218231	intergenic	intergenic	G	T	intergenic
218236	intergenic	intergenic	A	G	intergenic
218249	NTHI477_RS01100	924	G	A	synonymous
218258	NTHI477_RS01100	915	A	C	synonymous
218300	NTHI477_RS01100	873	C	T	synonymous
218345	NTHI477_RS01100	828	T	G	synonymous
218384	NTHI477_RS01100	789	C	T	synonymous
218393	NTHI477_RS01100	780	A	C	synonymous
218417	NTHI477_RS01100	756	A	G	synonymous
218453	NTHI477_RS01100	720	T	C	synonymous
218459	NTHI477_RS01100	714	T	C	synonymous
218462	NTHI477_RS01100	711	T	C	synonymous
218465	NTHI477_RS01100	708	G	A	synonymous
218471	NTHI477_RS01100	702	A	C	synonymous
218486	NTHI477_RS01100	687	A	G	synonymous
218537	NTHI477_RS01100	636	T	C	synonymous
218555	NTHI477_RS01100	618	G	A	synonymous
218561	NTHI477_RS01100	612	C	T	synonymous
218612	NTHI477_RS01100	561	C	T	synonymous
218615	NTHI477_RS01100	558	A	G	synonymous
218627	NTHI477_RS01100	546	T	A	synonymous
218641	NTHI477_RS01100	532	C	T	nonsynonymous
218669	NTHI477_RS01100	504	C	T	synonymous
218678	NTHI477_RS01100	495	A	T	synonymous
218684	NTHI477_RS01100	489	C	A	synonymous
218687	NTHI477_RS01100	486	C	A	synonymous
218789	NTHI477_RS01100	384	A	T	synonymous
218795	NTHI477_RS01100	378	C	T	synonymous
218798	NTHI477_RS01100	375	C	A	synonymous
218816	NTHI477_RS01100	357	G	A	synonymous
218831	NTHI477_RS01100	342	T	G	synonymous
218951	NTHI477_RS01100	222	C	A	synonymous
218952	NTHI477_RS01100	221	G	A	nonsynonymous
218954	NTHI477_RS01100	219	A	G	synonymous
218957	NTHI477_RS01100	216	G	A	synonymous
218969	NTHI477_RS01100	204	A	T	synonymous
219002	NTHI477_RS01100	171	G	A	synonymous
219037	NTHI477_RS01100	136	C	A	nonsynonymous
219137	NTHI477_RS01100	36	G	A	synonymous
219190	intergenic	intergenic	A	G	intergenic
219191	intergenic	intergenic	G	A	intergenic
219216	intergenic	intergenic	A	G	intergenic

219411	NTHI477_RS01105	657	A	G	synonymous
219493	NTHI477_RS01105	575	T	C	nonsynonymous
219720	NTHI477_RS01105	348	C	T	synonymous
219747	NTHI477_RS01105	321	C	A	nonsynonymous
219774	NTHI477_RS01105	294	G	A	synonymous
219840	NTHI477_RS01105	228	T	A	synonymous
219885	NTHI477_RS01105	183	C	T	synonymous
220052	NTHI477_RS01105	16	C	A	nonsynonymous
220073	intergenic	intergenic	C	T	intergenic
220149	intergenic	intergenic	G	T	intergenic
220229	intergenic	intergenic	T	A	intergenic
220290	intergenic	intergenic	G	A	intergenic
220454	intergenic	intergenic	C	T	intergenic
220454	intergenic	intergenic	C	A	intergenic
220455	intergenic	intergenic	C	T	intergenic
220456	intergenic	intergenic	G	A	intergenic
220466	intergenic	intergenic	G	T	intergenic
220467	intergenic	intergenic	G	T	intergenic
220469	intergenic	intergenic	T	G	intergenic
220470	intergenic	intergenic	T	G	intergenic
220473	intergenic	intergenic	C	A	intergenic
220480	intergenic	intergenic	G	A	intergenic
220481	intergenic	intergenic	C	T	intergenic
220486	intergenic	intergenic	C	T	intergenic
220489	intergenic	intergenic	C	T	intergenic
220494	intergenic	intergenic	A	G	intergenic
220504	intergenic	intergenic	C	A	intergenic
220505	intergenic	intergenic	G	T	intergenic
220511	intergenic	intergenic	T	C	intergenic
220515	intergenic	intergenic	C	T	intergenic
220517	intergenic	intergenic	T	G	intergenic
220519	intergenic	intergenic	A	C	intergenic
220519	intergenic	intergenic	A	T	intergenic
220520	intergenic	intergenic	G	A	intergenic
220521	intergenic	intergenic	C	T	intergenic
220522	intergenic	intergenic	C	T	intergenic
220528	intergenic	intergenic	C	T	intergenic
220529	intergenic	intergenic	C	T	intergenic
220530	intergenic	intergenic	C	T	intergenic
220531	intergenic	intergenic	C	A	intergenic
220535	intergenic	intergenic	T	C	intergenic
220536	intergenic	intergenic	T	C	intergenic
220540	intergenic	intergenic	T	C	intergenic
220545	intergenic	intergenic	C	T	intergenic
220556	intergenic	intergenic	G	A	intergenic

220624	intergenic	intergenic	C	G	intergenic
241882	intergenic	intergenic	C	A	intergenic
241898	intergenic	intergenic	T	A	intergenic
241902	intergenic	intergenic	A	G	intergenic
241902	intergenic	intergenic	A	C	intergenic
241914	intergenic	intergenic	A	C	intergenic
241945	intergenic	intergenic	T	C	intergenic
241967	intergenic	intergenic	G	C	intergenic
241998	intergenic	intergenic	G	A	intergenic
242027	intergenic	intergenic	T	C	intergenic
242028	intergenic	intergenic	G	T	intergenic
242063	NTHI477_RS01270	21	A	C	nonsynonymous
242090	NTHI477_RS01270	48	G	A	synonymous
242097	NTHI477_RS01270	55	G	A	nonsynonymous
242098	NTHI477_RS01270	56	A	G	nonsynonymous
242126	NTHI477_RS01270	84	A	C	synonymous
242126	NTHI477_RS01270	84	A	T	synonymous
242127	NTHI477_RS01270	85	G	A	nonsynonymous
242147	NTHI477_RS01270	105	G	T	synonymous
242174	NTHI477_RS01270	132	A	C	synonymous
242204	NTHI477_RS01270	162	G	A	synonymous
242210	NTHI477_RS01270	168	C	T	synonymous
242234	NTHI477_RS01270	192	A	G	synonymous
242237	NTHI477_RS01270	195	G	A	synonymous
242240	NTHI477_RS01270	198	A	G	synonymous
242252	NTHI477_RS01270	210	C	A	synonymous
242258	NTHI477_RS01270	216	G	A	synonymous
242262	NTHI477_RS01270	220	G	A	nonsynonymous
242264	NTHI477_RS01270	222	G	C	nonsynonymous
242264	NTHI477_RS01270	222	G	T	nonsynonymous
242267	NTHI477_RS01270	225	C	T	synonymous
242270	NTHI477_RS01270	228	T	C	synonymous
242274	NTHI477_RS01270	232	G	A	nonsynonymous
242285	NTHI477_RS01270	243	T	C	synonymous
242292	NTHI477_RS01270	250	T	C	synonymous
242294	NTHI477_RS01270	252	A	C	nonsynonymous
242297	NTHI477_RS01270	255	A	G	synonymous
242298	NTHI477_RS01270	256	C	G	nonsynonymous
242300	NTHI477_RS01270	258	G	A	synonymous
242306	NTHI477_RS01270	264	T	A	synonymous
242345	NTHI477_RS01270	303	C	T	synonymous
242354	NTHI477_RS01270	312	T	C	synonymous
242390	NTHI477_RS01270	348	C	T	synonymous
242405	NTHI477_RS01270	363	C	T	synonymous
242408	NTHI477_RS01270	366	A	T	synonymous

242435	NTHI477_RS01270	393	A	C	nonsynonymous
242450	NTHI477_RS01270	408	C	T	synonymous
242456	NTHI477_RS01270	414	T	A	synonymous
242459	NTHI477_RS01270	417	T	C	synonymous
242462	NTHI477_RS01270	420	C	T	synonymous
242483	NTHI477_RS01270	441	T	C	synonymous
242486	NTHI477_RS01270	444	G	A	synonymous
242487	NTHI477_RS01270	445	C	T	nonsynonymous
242546	NTHI477_RS01270	504	A	T	synonymous
242552	NTHI477_RS01270	510	C	T	synonymous
242573	NTHI477_RS01270	531	G	T	synonymous
242573	NTHI477_RS01270	531	G	C	synonymous
242576	NTHI477_RS01270	534	A	C	synonymous
242597	NTHI477_RS01270	555	G	A	synonymous
242612	NTHI477_RS01270	570	C	T	synonymous
242624	NTHI477_RS01270	582	C	T	synonymous
242634	NTHI477_RS01270	592	G	A	nonsynonymous
242639	NTHI477_RS01270	597	C	T	synonymous
242642	NTHI477_RS01270	600	G	A	synonymous
242652	NTHI477_RS01270	610	A	G	nonsynonymous
242672	NTHI477_RS01270	630	T	C	synonymous
242692	NTHI477_RS01270	650	C	T	nonsynonymous
242694	NTHI477_RS01270	652	A	G	nonsynonymous
242717	NTHI477_RS01270	675	A	G	synonymous
242723	NTHI477_RS01270	681	C	T	synonymous
242747	NTHI477_RS01270	705	G	A	synonymous
242751	NTHI477_RS01270	709	C	G	nonsynonymous
242759	NTHI477_RS01270	717	T	C	synonymous
242771	NTHI477_RS01270	729	G	C	synonymous
242783	NTHI477_RS01270	741	G	C	synonymous
242789	NTHI477_RS01270	747	C	T	synonymous
242792	NTHI477_RS01270	750	G	A	synonymous
242793	NTHI477_RS01270	751	G	A	nonsynonymous
242806	NTHI477_RS01270	764	C	T	nonsynonymous
242807	NTHI477_RS01270	765	G	T	synonymous
242810	NTHI477_RS01270	768	T	G	synonymous
242812	NTHI477_RS01270	770	T	C	nonsynonymous
242876	NTHI477_RS01270	834	G	A	synonymous
242921	NTHI477_RS01270	879	C	G	synonymous
242936	NTHI477_RS01270	894	T	C	synonymous
242948	NTHI477_RS01270	906	T	G	synonymous
242960	NTHI477_RS01270	918	A	G	synonymous
242981	NTHI477_RS01270	939	T	C	synonymous
242984	NTHI477_RS01270	942	C	A	synonymous
242993	NTHI477_RS01270	951	A	G	synonymous

243023	NTHI477_RS01270	981	T	C	synonymous
243065	NTHI477_RS01275	4	A	G	nonsynonymous
243079	NTHI477_RS01275	18	T	C	synonymous
243088	NTHI477_RS01275	27	C	T	synonymous
243091	NTHI477_RS01275	30	C	T	synonymous
243094	NTHI477_RS01275	33	C	T	synonymous
243095	NTHI477_RS01275	34	A	T	nonsynonymous
243124	NTHI477_RS01275	63	G	C	synonymous
243124	NTHI477_RS01275	63	G	T	synonymous
243134	NTHI477_RS01275	73	T	C	nonsynonymous
243148	NTHI477_RS01275	87	G	A	synonymous
243154	NTHI477_RS01275	93	C	T	synonymous
243160	NTHI477_RS01275	99	T	C	synonymous
243164	NTHI477_RS01275	103	T	C	synonymous
243166	NTHI477_RS01275	105	G	T	nonsynonymous
243181	NTHI477_RS01275	120	T	C	synonymous
243193	NTHI477_RS01275	132	A	G	synonymous
243196	NTHI477_RS01275	135	T	C	synonymous
243205	NTHI477_RS01275	144	G	A	synonymous
243241	NTHI477_RS01275	180	A	G	synonymous
243259	NTHI477_RS01275	198	C	T	synonymous
243262	NTHI477_RS01275	201	C	G	synonymous
243286	NTHI477_RS01275	225	C	T	synonymous
243322	NTHI477_RS01275	261	C	A	synonymous
243329	NTHI477_RS01275	268	A	C	nonsynonymous
243345	NTHI477_RS01275	284	G	A	nonsynonymous
243401	NTHI477_RS01275	340	G	T	nonsynonymous
243413	NTHI477_RS01275	352	A	C	nonsynonymous
243424	NTHI477_RS01275	363	C	A	nonsynonymous
243425	NTHI477_RS01275	364	G	A	nonsynonymous
243427	NTHI477_RS01275	366	G	A	synonymous
243427	NTHI477_RS01275	366	G	T	synonymous
243430	NTHI477_RS01275	369	A	G	synonymous
243436	NTHI477_RS01275	375	C	T	synonymous
243442	NTHI477_RS01275	381	T	C	synonymous
243448	NTHI477_RS01275	387	T	C	synonymous
243451	NTHI477_RS01275	390	A	G	synonymous
243454	NTHI477_RS01275	393	T	G	synonymous
243457	NTHI477_RS01275	396	G	A	synonymous
243469	NTHI477_RS01275	408	C	T	synonymous
243484	NTHI477_RS01275	423	G	T	synonymous
243517	NTHI477_RS01275	456	T	C	synonymous
243546	NTHI477_RS01275	485	C	T	nonsynonymous
243547	NTHI477_RS01275	486	G	A	synonymous
243569	NTHI477_RS01275	508	G	A	nonsynonymous

243575	NTHI477_RS01275	514	A	G	nonsynonymous
243583	NTHI477_RS01275	522	A	G	synonymous
243610	NTHI477_RS01275	549	C	T	synonymous
243614	NTHI477_RS01275	553	G	A	nonsynonymous
243619	NTHI477_RS01275	558	C	T	synonymous
243620	NTHI477_RS01275	559	G	A	nonsynonymous
243628	NTHI477_RS01275	567	G	A	synonymous
243637	NTHI477_RS01275	576	A	G	synonymous
243652	NTHI477_RS01275	591	G	A	synonymous
243671	NTHI477_RS01275	610	C	T	synonymous
243677	NTHI477_RS01275	616	G	A	nonsynonymous
243678	NTHI477_RS01275	617	T	C	nonsynonymous
243689	NTHI477_RS01275	628	G	T	nonsynonymous
243706	NTHI477_RS01275	645	A	C	synonymous
243730	NTHI477_RS01275	669	A	G	synonymous
243731	NTHI477_RS01275	670	A	G	nonsynonymous
243742	NTHI477_RS01275	681	G	A	synonymous
243760	NTHI477_RS01275	699	T	G	synonymous
243764	NTHI477_RS01275	703	G	T	nonsynonymous
243776	NTHI477_RS01275	715	A	G	nonsynonymous
243779	NTHI477_RS01275	718	C	T	synonymous
243781	NTHI477_RS01275	720	G	A	synonymous
243782	NTHI477_RS01275	721	C	T	synonymous
243783	NTHI477_RS01275	722	T	A	nonsynonymous
243787	NTHI477_RS01275	726	A	T	synonymous
243790	NTHI477_RS01275	729	C	G	synonymous
243796	NTHI477_RS01275	735	C	T	synonymous
243799	NTHI477_RS01275	738	C	A	synonymous
243799	NTHI477_RS01275	738	C	T	synonymous
243805	NTHI477_RS01275	744	T	C	synonymous
243820	NTHI477_RS01275	759	C	T	synonymous
243824	NTHI477_RS01275	763	A	G	nonsynonymous
243868	NTHI477_RS01275	807	A	T	synonymous
243918	intergenic	intergenic	G	T	intergenic
243924	intergenic	intergenic	C	T	intergenic
243941	intergenic	intergenic	G	C	intergenic
243941	intergenic	intergenic	G	T	intergenic
243961	intergenic	intergenic	G	A	intergenic
243963	intergenic	intergenic	C	T	intergenic
243964	intergenic	intergenic	A	G	intergenic
243965	intergenic	intergenic	C	A	intergenic
243984	intergenic	intergenic	T	G	intergenic
243996	NTHI477_RS01280	598	A	C	nonsynonymous
244003	NTHI477_RS01280	591	C	T	synonymous
244007	NTHI477_RS01280	587	C	T	nonsynonymous

244018	NTHI477_RS01280	576	A	G	synonymous
244087	NTHI477_RS01280	507	T	C	synonymous
244090	NTHI477_RS01280	504	A	T	synonymous
244092	NTHI477_RS01280	502	C	T	nonsynonymous
244105	NTHI477_RS01280	489	G	A	synonymous
244168	NTHI477_RS01280	426	G	A	synonymous
244171	NTHI477_RS01280	423	G	A	synonymous
244183	NTHI477_RS01280	411	C	T	synonymous
244213	NTHI477_RS01280	381	T	C	synonymous
244234	NTHI477_RS01280	360	G	A	synonymous
244237	NTHI477_RS01280	357	G	A	synonymous
244240	NTHI477_RS01280	354	G	A	synonymous
244244	NTHI477_RS01280	350	C	T	nonsynonymous
244249	NTHI477_RS01280	345	T	G	synonymous
244258	NTHI477_RS01280	336	A	T	synonymous
244270	NTHI477_RS01280	324	A	G	synonymous
244282	NTHI477_RS01280	312	G	T	synonymous
244285	NTHI477_RS01280	309	A	G	synonymous
244291	NTHI477_RS01280	303	A	C	synonymous
244297	NTHI477_RS01280	297	T	G	synonymous
244300	NTHI477_RS01280	294	G	C	synonymous
244302	NTHI477_RS01280	292	A	C	nonsynonymous
244315	NTHI477_RS01280	279	A	T	synonymous
244351	NTHI477_RS01280	243	G	A	synonymous
244373	NTHI477_RS01280	221	G	A	nonsynonymous
244384	NTHI477_RS01280	210	A	G	synonymous
244432	NTHI477_RS01280	162	A	G	synonymous
244456	NTHI477_RS01280	138	C	A	synonymous
244504	NTHI477_RS01280	90	A	G	synonymous
244534	NTHI477_RS01280	60	G	A	synonymous
244545	NTHI477_RS01280	49	T	C	nonsynonymous
244548	NTHI477_RS01280	46	C	T	nonsynonymous
244601	intergenic	intergenic	T	A	intergenic
244617	intergenic	intergenic	G	T	intergenic
244640	intergenic	intergenic	C	T	intergenic
244646	intergenic	intergenic	G	A	intergenic
244652	intergenic	intergenic	T	G	intergenic
244654	intergenic	intergenic	G	A	intergenic
244658	intergenic	intergenic	T	C	intergenic
244659	intergenic	intergenic	C	G	intergenic
244691	intergenic	intergenic	G	A	intergenic
244694	intergenic	intergenic	C	A	intergenic
244695	intergenic	intergenic	G	T	intergenic
244697	intergenic	intergenic	T	A	intergenic
244698	intergenic	intergenic	G	C	intergenic

244700	intergenic	intergenic	A	G	intergenic
244729	NTHI477_RS01285	1387	A	G	synonymous
244811	NTHI477_RS01285	1305	T	C	synonymous
244817	NTHI477_RS01285	1299	G	T	synonymous
244829	NTHI477_RS01285	1287	A	G	synonymous
244832	NTHI477_RS01285	1284	T	C	synonymous
244835	NTHI477_RS01285	1281	G	C	synonymous
244853	NTHI477_RS01285	1263	T	G	synonymous
244856	NTHI477_RS01285	1260	A	G	synonymous
244865	NTHI477_RS01285	1251	A	G	synonymous
244868	NTHI477_RS01285	1248	G	A	synonymous
244873	NTHI477_RS01285	1243	G	T	nonsynonymous
244874	NTHI477_RS01285	1242	C	T	synonymous
244894	NTHI477_RS01285	1222	G	A	synonymous
244925	NTHI477_RS01285	1191	G	T	synonymous
244926	NTHI477_RS01285	1190	C	A	nonsynonymous
244927	NTHI477_RS01285	1189	G	A	nonsynonymous
244928	NTHI477_RS01285	1188	T	A	synonymous
244940	NTHI477_RS01285	1176	T	C	synonymous
244949	NTHI477_RS01285	1167	C	T	synonymous
244952	NTHI477_RS01285	1164	T	A	synonymous
244967	NTHI477_RS01285	1149	G	A	synonymous
244976	NTHI477_RS01285	1140	A	G	synonymous
244979	NTHI477_RS01285	1137	G	A	synonymous
244985	NTHI477_RS01285	1131	G	A	synonymous
245015	NTHI477_RS01285	1101	T	G	synonymous
245030	NTHI477_RS01285	1086	G	A	synonymous
245066	NTHI477_RS01285	1050	C	A	synonymous
245078	NTHI477_RS01285	1038	G	T	synonymous
245081	NTHI477_RS01285	1035	G	A	synonymous
245087	NTHI477_RS01285	1029	G	A	synonymous
245105	NTHI477_RS01285	1011	A	G	synonymous
245126	NTHI477_RS01285	990	G	A	synonymous
245216	NTHI477_RS01285	900	T	A	synonymous
245237	NTHI477_RS01285	879	G	A	synonymous
245336	NTHI477_RS01285	780	T	A	synonymous
245353	NTHI477_RS01285	763	C	T	nonsynonymous
245354	NTHI477_RS01285	762	G	A	synonymous
245384	NTHI477_RS01285	732	C	A	synonymous
245386	NTHI477_RS01285	730	C	A	nonsynonymous
245405	NTHI477_RS01285	711	A	G	synonymous
245417	NTHI477_RS01285	699	G	A	synonymous
245426	NTHI477_RS01285	690	A	G	synonymous
245429	NTHI477_RS01285	687	T	A	synonymous
245429	NTHI477_RS01285	687	T	C	synonymous

245432	NTHI477_RS01285	684	A	C	synonymous
245447	NTHI477_RS01285	669	G	A	synonymous
245459	NTHI477_RS01285	657	C	A	synonymous
245459	NTHI477_RS01285	657	C	G	synonymous
245462	NTHI477_RS01285	654	C	G	synonymous
245501	NTHI477_RS01285	615	T	A	synonymous
245501	NTHI477_RS01285	615	T	C	synonymous
245502	NTHI477_RS01285	614	G	A	nonsynonymous
245510	NTHI477_RS01285	606	C	T	synonymous
245522	NTHI477_RS01285	594	A	G	synonymous
245534	NTHI477_RS01285	582	T	A	synonymous
245537	NTHI477_RS01285	579	C	T	synonymous
245582	NTHI477_RS01285	534	G	A	synonymous
245585	NTHI477_RS01285	531	G	A	synonymous
245629	NTHI477_RS01285	487	A	G	nonsynonymous
245720	NTHI477_RS01285	396	G	A	synonymous
245729	NTHI477_RS01285	387	G	A	synonymous
245732	NTHI477_RS01285	384	A	G	synonymous
245735	NTHI477_RS01285	381	A	G	synonymous
245738	NTHI477_RS01285	378	A	T	synonymous
245747	NTHI477_RS01285	369	A	G	synonymous
245762	NTHI477_RS01285	354	G	A	synonymous
245767	NTHI477_RS01285	349	T	G	nonsynonymous
245767	NTHI477_RS01285	349	T	A	nonsynonymous
245774	NTHI477_RS01285	342	T	C	synonymous
245774	NTHI477_RS01285	342	T	A	synonymous
245783	NTHI477_RS01285	333	G	T	synonymous
245789	NTHI477_RS01285	327	T	C	synonymous
245795	NTHI477_RS01285	321	G	A	synonymous
245798	NTHI477_RS01285	318	C	T	synonymous
245810	NTHI477_RS01285	306	A	G	synonymous
245819	NTHI477_RS01285	297	A	G	synonymous
245822	NTHI477_RS01285	294	A	T	synonymous
245891	NTHI477_RS01285	225	C	T	synonymous
245892	NTHI477_RS01285	224	A	T	nonsynonymous
245930	NTHI477_RS01285	186	T	C	synonymous
245951	NTHI477_RS01285	165	G	A	synonymous
245965	NTHI477_RS01285	151	A	G	synonymous
245969	NTHI477_RS01285	147	G	A	synonymous
245981	NTHI477_RS01285	135	G	A	synonymous
246008	NTHI477_RS01285	108	T	A	synonymous
246010	NTHI477_RS01285	106	G	A	nonsynonymous
246011	NTHI477_RS01285	105	G	A	synonymous
246014	NTHI477_RS01285	102	A	G	synonymous
246069	NTHI477_RS01285	47	G	A	nonsynonymous

246195	intergenic	intergenic	A	G	intergenic
246236	intergenic	intergenic	G	A	intergenic
246277	intergenic	intergenic	T	C	intergenic
246346	NTHI477_RS01290	850	T	G	nonsynonymous
246440	NTHI477_RS01290	756	A	G	synonymous
246453	NTHI477_RS01290	743	G	A	nonsynonymous
246460	NTHI477_RS01290	736	T	C	nonsynonymous
246509	NTHI477_RS01290	687	A	G	synonymous
246514	NTHI477_RS01290	682	G	T	nonsynonymous
246518	NTHI477_RS01290	678	A	G	synonymous
246546	NTHI477_RS01290	650	A	T	nonsynonymous
246567	NTHI477_RS01290	629	T	G	nonsynonymous
246568	NTHI477_RS01290	628	T	G	nonsynonymous
246592	NTHI477_RS01290	604	G	A	nonsynonymous
246606	NTHI477_RS01290	590	A	C	nonsynonymous
246607	NTHI477_RS01290	589	A	G	synonymous
246648	NTHI477_RS01290	548	T	A	nonsynonymous
246651	NTHI477_RS01290	545	A	T	nonsynonymous
246657	NTHI477_RS01290	539	A	T	nonsynonymous
246658	NTHI477_RS01290	538	A	T	nonsynonymous
246661	NTHI477_RS01290	535	C	T	nonsynonymous
246678	NTHI477_RS01290	518	T	A	nonsynonymous
246745	NTHI477_RS01290	451	T	A	nonsynonymous
246749	NTHI477_RS01290	447	A	T	synonymous
246762	NTHI477_RS01290	434	T	G	nonsynonymous
246768	NTHI477_RS01290	428	T	C	nonsynonymous
246795	NTHI477_RS01290	401	G	A	nonsynonymous
246796	NTHI477_RS01290	400	A	T	nonsynonymous
246881	NTHI477_RS01290	315	C	T	nonsynonymous
246977	NTHI477_RS01290	219	T	C	nonsynonymous
246985	NTHI477_RS01290	211	T	G	nonsynonymous
246991	NTHI477_RS01290	205	A	C	nonsynonymous
247021	NTHI477_RS01290	175	C	A	nonsynonymous
247079	NTHI477_RS01290	117	A	C	synonymous
247101	NTHI477_RS01290	95	T	A	nonsynonymous
247463	NTHI477_RS09500	72	A	C	nonsynonymous
247495	NTHI477_RS09500	104	T	G	nonsynonymous
247756	intergenic	intergenic	T	G	intergenic
247761	intergenic	intergenic	A	G	intergenic
247764	intergenic	intergenic	A	G	intergenic
247765	intergenic	intergenic	T	C	intergenic
247766	intergenic	intergenic	C	A	intergenic
247782	intergenic	intergenic	C	T	intergenic
247785	intergenic	intergenic	C	T	intergenic
247788	intergenic	intergenic	A	G	intergenic

247790	intergenic	intergenic	G	T	intergenic
247807	intergenic	intergenic	C	T	intergenic
247810	intergenic	intergenic	A	C	intergenic
247811	intergenic	intergenic	G	C	intergenic
247811	intergenic	intergenic	G	T	intergenic
247812	intergenic	intergenic	T	C	intergenic
247816	intergenic	intergenic	A	T	intergenic
247820	intergenic	intergenic	G	A	intergenic
247824	intergenic	intergenic	C	T	intergenic
247865	NTHI477_RS01300	15	A	G	synonymous
247874	NTHI477_RS01300	24	T	C	synonymous
247887	NTHI477_RS01300	37	A	G	nonsynonymous
247895	NTHI477_RS01300	45	T	G	synonymous
247919	NTHI477_RS01300	69	T	C	synonymous
247928	NTHI477_RS01300	78	T	A	synonymous
247931	NTHI477_RS01300	81	T	C	synonymous
247943	NTHI477_RS01300	93	T	C	synonymous
247988	NTHI477_RS01300	138	A	G	synonymous
247989	NTHI477_RS01300	139	T	C	synonymous
247994	NTHI477_RS01300	144	A	G	synonymous
248000	NTHI477_RS01300	150	T	C	synonymous
248003	NTHI477_RS01300	153	A	G	synonymous
248005	NTHI477_RS01300	155	T	G	nonsynonymous
248024	NTHI477_RS01300	174	C	T	synonymous
248039	NTHI477_RS01300	189	G	A	synonymous
248051	NTHI477_RS01300	201	A	G	synonymous
248054	NTHI477_RS01300	204	T	G	synonymous
248059	NTHI477_RS01300	209	C	A	nonsynonymous
248084	NTHI477_RS01300	234	G	A	synonymous
248090	NTHI477_RS01300	240	G	A	synonymous
248099	NTHI477_RS01300	249	A	G	synonymous
248116	NTHI477_RS01300	266	A	G	nonsynonymous
248117	NTHI477_RS01300	267	C	T	synonymous
248120	NTHI477_RS01300	270	C	T	synonymous
248124	NTHI477_RS01300	274	T	G	nonsynonymous
248127	NTHI477_RS01300	277	T	C	synonymous
248132	NTHI477_RS01300	282	C	T	synonymous
248150	NTHI477_RS01300	300	C	T	synonymous
248165	NTHI477_RS01300	315	T	C	synonymous
248168	NTHI477_RS01300	318	T	C	synonymous
248174	NTHI477_RS01300	324	C	A	synonymous
248177	NTHI477_RS01300	327	A	G	synonymous
248178	NTHI477_RS01300	328	A	C	nonsynonymous
248189	NTHI477_RS01300	339	T	C	synonymous
248192	NTHI477_RS01300	342	G	A	synonymous

248201	NTHI477_RS01300	351	T	C	synonymous
248204	NTHI477_RS01300	354	T	C	synonymous
248206	NTHI477_RS01300	356	C	T	nonsynonymous
248207	NTHI477_RS01300	357	G	C	synonymous
248207	NTHI477_RS01300	357	G	T	synonymous
248222	NTHI477_RS01300	372	T	C	synonymous
248237	NTHI477_RS01300	387	A	G	synonymous
248248	NTHI477_RS01300	398	T	A	nonsynonymous
248253	NTHI477_RS01300	403	G	A	nonsynonymous
248282	NTHI477_RS01300	432	T	G	synonymous
248294	intergenic	intergenic	C	T	intergenic
248297	intergenic	intergenic	T	C	intergenic
248301	intergenic	intergenic	G	A	intergenic
248336	intergenic	intergenic	C	T	intergenic
248453	NTHI477_RS09420	84	C	T	synonymous
248464	NTHI477_RS09420	95	T	C	nonsynonymous
248503	NTHI477_RS09420	134	T	C	nonsynonymous
248504	NTHI477_RS09420	135	G	A	synonymous
248512	NTHI477_RS09420	143	T	C	nonsynonymous
248521	NTHI477_RS09420	152	T	G	nonsynonymous
248604	NTHI477_RS01305	52	G	T	nonsynonymous
248626	NTHI477_RS01305	74	C	T	nonsynonymous
248662	NTHI477_RS01305	110	C	A	nonsynonymous
248701	NTHI477_RS01305	149	G	A	nonsynonymous
248725	NTHI477_RS01305	173	T	A	nonsynonymous
248741	NTHI477_RS01305	189	T	C	synonymous
248749	NTHI477_RS01305	197	C	T	nonsynonymous
248750	NTHI477_RS01305	198	A	G	synonymous
248753	NTHI477_RS01305	201	G	A	synonymous
248759	NTHI477_RS01305	207	G	A	synonymous
248762	NTHI477_RS01305	210	C	T	synonymous
248777	NTHI477_RS01305	225	G	A	synonymous
248783	NTHI477_RS01305	231	A	G	nonsynonymous
248792	NTHI477_RS01305	240	T	G	synonymous
248804	NTHI477_RS01305	252	T	C	synonymous
248807	NTHI477_RS01305	255	C	T	synonymous
248813	NTHI477_RS01305	261	A	T	synonymous
248815	NTHI477_RS01305	263	A	G	nonsynonymous
248816	NTHI477_RS01305	264	T	A	nonsynonymous
248831	NTHI477_RS01305	279	T	C	synonymous
248832	NTHI477_RS01305	280	A	T	nonsynonymous
248832	NTHI477_RS01305	280	A	G	nonsynonymous
248835	NTHI477_RS01305	283	A	G	nonsynonymous
248838	NTHI477_RS01305	286	C	A	nonsynonymous
248839	NTHI477_RS01305	287	G	A	nonsynonymous

248840	NTHI477_RS01305	288	C	A	synonymous
248841	NTHI477_RS01305	289	A	G	nonsynonymous
248844	NTHI477_RS01305	292	G	T	nonsynonymous
248845	NTHI477_RS01305	293	A	C	nonsynonymous
248846	NTHI477_RS01305	294	A	T	nonsynonymous
248849	NTHI477_RS01305	297	A	T	synonymous
248853	NTHI477_RS01305	301	C	T	nonsynonymous
248855	NTHI477_RS01305	303	T	A	synonymous
248857	NTHI477_RS01305	305	C	A	nonsynonymous
248858	NTHI477_RS01305	306	A	T	synonymous
248861	NTHI477_RS01305	309	G	T	synonymous
248862	NTHI477_RS01305	310	A	G	nonsynonymous
248864	NTHI477_RS01305	312	T	A	synonymous
248867	NTHI477_RS01305	315	G	A	synonymous
248873	NTHI477_RS01305	321	A	G	synonymous
248876	NTHI477_RS01305	324	A	T	synonymous
248880	NTHI477_RS01305	328	A	T	nonsynonymous
248881	NTHI477_RS01305	329	G	C	nonsynonymous
248882	NTHI477_RS01305	330	T	A	nonsynonymous
248885	NTHI477_RS01305	333	T	C	synonymous
248888	NTHI477_RS01305	336	T	G	synonymous
248907	NTHI477_RS01305	355	A	G	nonsynonymous
248916	NTHI477_RS01305	364	T	A	nonsynonymous
248924	NTHI477_RS01305	372	T	C	synonymous
248925	NTHI477_RS01305	373	T	C	nonsynonymous
248930	intergenic	intergenic	A	T	intergenic
248946	intergenic	intergenic	T	G	intergenic
248958	intergenic	intergenic	T	C	intergenic
248988	intergenic	intergenic	A	G	intergenic
248990	intergenic	intergenic	T	C	intergenic
248993	intergenic	intergenic	T	A	intergenic
248994	intergenic	intergenic	A	G	intergenic
249000	intergenic	intergenic	G	A	intergenic
249002	intergenic	intergenic	G	A	intergenic
249005	intergenic	intergenic	T	C	intergenic
249035	intergenic	intergenic	T	C	intergenic
249092	intergenic	intergenic	G	A	intergenic
249098	intergenic	intergenic	T	G	intergenic
249109	intergenic	intergenic	A	T	intergenic
249115	intergenic	intergenic	T	C	intergenic
249125	intergenic	intergenic	T	C	intergenic
249126	intergenic	intergenic	A	C	intergenic
249127	intergenic	intergenic	T	C	intergenic
249144	intergenic	intergenic	G	T	intergenic
249160	NTHI477_RS01310	12	C	T	synonymous

249163	NTHI477_RS01310	15	A	C	nonsynonymous
249181	NTHI477_RS01310	33	T	C	synonymous
249190	NTHI477_RS01310	42	C	G	synonymous
249202	NTHI477_RS01310	54	T	A	synonymous
249202	NTHI477_RS01310	54	T	C	synonymous
249205	NTHI477_RS01310	57	G	A	synonymous
249214	NTHI477_RS01310	66	C	G	synonymous
249217	NTHI477_RS01310	69	C	T	synonymous
249220	NTHI477_RS01310	72	C	G	synonymous
249226	NTHI477_RS01310	78	G	A	synonymous
249229	NTHI477_RS01310	81	C	T	synonymous
249230	NTHI477_RS01310	82	A	G	nonsynonymous
249232	NTHI477_RS01310	84	A	C	nonsynonymous
249235	NTHI477_RS01310	87	C	G	synonymous
249253	NTHI477_RS01310	105	A	G	synonymous
249256	NTHI477_RS01310	108	C	T	synonymous
249262	NTHI477_RS01310	114	T	C	synonymous
249263	NTHI477_RS01310	115	T	C	synonymous
249265	NTHI477_RS01310	117	G	A	synonymous
249266	NTHI477_RS01310	118	C	T	nonsynonymous
249268	NTHI477_RS01310	120	T	A	synonymous
249271	NTHI477_RS01310	123	T	G	synonymous
249274	NTHI477_RS01310	126	T	G	synonymous
249275	NTHI477_RS01310	127	A	C	synonymous
249277	NTHI477_RS01310	129	A	G	synonymous
249277	NTHI477_RS01310	129	A	C	nonsynonymous
249283	NTHI477_RS01310	135	A	G	synonymous
249295	NTHI477_RS01310	147	T	C	synonymous
249302	NTHI477_RS01310	154	A	T	nonsynonymous
249303	NTHI477_RS01310	155	C	A	nonsynonymous
249304	NTHI477_RS01310	156	C	G	synonymous
249311	NTHI477_RS01310	163	G	A	nonsynonymous
249313	NTHI477_RS01310	165	G	A	synonymous
249322	NTHI477_RS01310	174	A	G	synonymous
249325	NTHI477_RS01310	177	G	A	synonymous
249327	NTHI477_RS01310	179	C	T	nonsynonymous
249328	NTHI477_RS01310	180	G	A	synonymous
249334	NTHI477_RS01310	186	T	C	synonymous
249334	NTHI477_RS01310	186	T	G	synonymous
249336	NTHI477_RS01310	188	T	C	nonsynonymous
249337	NTHI477_RS01310	189	T	G	synonymous
249343	NTHI477_RS01310	195	A	G	synonymous
249346	NTHI477_RS01310	198	G	A	synonymous
249349	NTHI477_RS01310	201	G	A	synonymous
249354	NTHI477_RS01310	206	A	T	nonsynonymous

249355	NTHI477_RS01310	207	G	A	synonymous
249361	NTHI477_RS01310	213	G	C	synonymous
249362	NTHI477_RS01310	214	A	G	nonsynonymous
249363	NTHI477_RS01310	215	A	C	nonsynonymous
249364	NTHI477_RS01310	216	G	A	synonymous
249364	NTHI477_RS01310	216	G	T	nonsynonymous
249365	NTHI477_RS01310	217	C	G	nonsynonymous
249370	NTHI477_RS01310	222	G	A	synonymous
249377	NTHI477_RS01310	229	G	T	nonsynonymous
249378	NTHI477_RS01310	230	G	C	nonsynonymous
249379	NTHI477_RS01310	231	G	T	synonymous
249379	NTHI477_RS01310	231	G	A	synonymous
249385	NTHI477_RS01310	237	T	G	nonsynonymous
249385	NTHI477_RS01310	237	T	A	nonsynonymous
249386	NTHI477_RS01310	238	A	G	nonsynonymous
249388	NTHI477_RS01310	240	G	A	synonymous
249389	NTHI477_RS01310	241	C	T	synonymous
249392	NTHI477_RS01310	244	A	C	nonsynonymous
249393	NTHI477_RS01310	245	A	T	nonsynonymous
249397	NTHI477_RS01310	249	A	G	synonymous
249407	NTHI477_RS01310	259	A	G	nonsynonymous
249417	NTHI477_RS01310	269	C	T	nonsynonymous
249419	NTHI477_RS01310	271	A	G	nonsynonymous
249425	NTHI477_RS01310	277	C	A	nonsynonymous
249426	NTHI477_RS01310	278	A	G	nonsynonymous
249432	NTHI477_RS01310	284	C	T	nonsynonymous
249460	NTHI477_RS01310	312	G	A	synonymous
249491	intergenic	intergenic	G	T	intergenic
249509	NTHI477_RS01315	3	G	T	nonsynonymous
249541	NTHI477_RS01315	35	G	T	nonsynonymous
249549	NTHI477_RS01315	43	A	G	nonsynonymous
249570	NTHI477_RS01315	64	A	G	nonsynonymous
249624	NTHI477_RS01315	118	A	G	nonsynonymous
249627	NTHI477_RS01315	121	C	G	nonsynonymous
249630	NTHI477_RS01315	124	A	G	nonsynonymous
249634	NTHI477_RS01315	128	G	A	nonsynonymous
249678	NTHI477_RS01315	172	T	G	nonsynonymous
249679	NTHI477_RS01315	173	C	T	nonsynonymous
249742	NTHI477_RS01315	236	A	C	nonsynonymous
249756	NTHI477_RS01315	250	C	T	synonymous
249916	intergenic	intergenic	C	T	intergenic
249966	NTHI477_RS01320	7	C	A	nonsynonymous
249971	NTHI477_RS01320	12	A	C	nonsynonymous
249972	NTHI477_RS01320	13	T	C	nonsynonymous
249975	NTHI477_RS01320	16	G	C	nonsynonymous

249982	NTHI477_RS01320	23	C	T	nonsynonymous
250010	NTHI477_RS01320	51	A	G	synonymous
250041	NTHI477_RS01320	82	G	A	nonsynonymous
250056	NTHI477_RS01320	97	T	A	nonsynonymous
250060	NTHI477_RS01320	101	A	G	nonsynonymous
250061	NTHI477_RS01320	102	A	G	synonymous
250068	NTHI477_RS01320	109	A	C	nonsynonymous
250070	NTHI477_RS01320	111	T	C	synonymous
250073	NTHI477_RS01320	114	G	A	synonymous
250075	NTHI477_RS01320	116	C	T	nonsynonymous
250091	NTHI477_RS01320	132	A	G	synonymous
250100	NTHI477_RS01320	141	G	A	synonymous
250106	NTHI477_RS01320	147	C	T	synonymous
250107	NTHI477_RS01320	148	G	A	nonsynonymous
250109	NTHI477_RS01320	150	A	C	synonymous
250112	NTHI477_RS01320	153	A	G	synonymous
250118	NTHI477_RS01320	159	T	C	synonymous
250119	NTHI477_RS01320	160	T	C	synonymous
250124	NTHI477_RS01320	165	T	C	synonymous
250130	NTHI477_RS01320	171	A	G	synonymous
250133	NTHI477_RS01320	174	C	T	synonymous
250136	NTHI477_RS01320	177	A	T	synonymous
250142	NTHI477_RS01320	183	A	G	synonymous
250193	NTHI477_RS01320	234	C	T	synonymous
250220	NTHI477_RS01320	261	C	T	synonymous
250223	NTHI477_RS01320	264	A	G	synonymous
250229	NTHI477_RS01320	270	T	C	synonymous
250232	NTHI477_RS01320	273	A	G	synonymous
250247	NTHI477_RS01320	288	G	A	synonymous
250250	NTHI477_RS01320	291	A	G	synonymous
250262	NTHI477_RS01320	303	G	A	synonymous
250265	NTHI477_RS01320	306	T	A	synonymous
250280	NTHI477_RS01320	321	T	C	synonymous
250286	NTHI477_RS01320	327	C	T	synonymous
250298	NTHI477_RS01320	339	T	C	synonymous
250307	NTHI477_RS01320	348	C	T	synonymous
250313	NTHI477_RS01320	354	C	T	synonymous
250325	NTHI477_RS01320	366	T	C	synonymous
250334	NTHI477_RS01320	375	T	C	synonymous
250343	NTHI477_RS01320	384	T	C	synonymous
250367	NTHI477_RS01320	408	C	T	synonymous
250376	NTHI477_RS01320	417	G	T	synonymous
250400	NTHI477_RS01320	441	G	A	synonymous
250406	NTHI477_RS01320	447	C	T	synonymous
250409	NTHI477_RS01320	450	C	G	synonymous

250418	NTHI477_RS01320	459	T	A	nonsynonymous
250418	NTHI477_RS01320	459	T	G	nonsynonymous
250421	NTHI477_RS01320	462	T	C	synonymous
250424	NTHI477_RS01320	465	A	G	synonymous
250427	NTHI477_RS01320	468	G	C	synonymous
250427	NTHI477_RS01320	468	G	T	synonymous
250430	NTHI477_RS01320	471	T	C	synonymous
250433	NTHI477_RS01320	474	A	C	synonymous
250433	NTHI477_RS01320	474	A	G	synonymous
250451	NTHI477_RS01320	492	T	G	synonymous
250454	NTHI477_RS01320	495	T	C	synonymous
250460	NTHI477_RS01320	501	C	T	synonymous
250469	NTHI477_RS01320	510	T	G	synonymous
250484	NTHI477_RS01320	525	C	A	synonymous
250484	NTHI477_RS01320	525	C	T	synonymous
250490	NTHI477_RS01320	531	C	T	synonymous
250493	NTHI477_RS01320	534	A	G	synonymous
250496	NTHI477_RS01320	537	A	G	synonymous
250520	NTHI477_RS01320	561	C	T	synonymous
250523	NTHI477_RS01320	564	G	A	synonymous
250541	NTHI477_RS01320	582	G	T	synonymous
250541	NTHI477_RS01320	582	G	A	synonymous
250547	NTHI477_RS01320	588	T	C	synonymous
250553	NTHI477_RS01320	594	G	A	synonymous
250562	NTHI477_RS01320	603	T	C	synonymous
250565	NTHI477_RS01320	606	T	C	synonymous
250580	NTHI477_RS01320	621	T	C	synonymous
250591	NTHI477_RS01320	632	C	T	nonsynonymous
250592	NTHI477_RS01320	633	A	T	synonymous
250592	NTHI477_RS01320	633	A	C	synonymous
250593	NTHI477_RS01320	634	A	G	nonsynonymous
250595	NTHI477_RS01320	636	T	C	synonymous
250597	NTHI477_RS01320	638	G	A	nonsynonymous
250598	NTHI477_RS01320	639	T	C	synonymous
250610	NTHI477_RS01320	651	A	G	synonymous
250616	NTHI477_RS01320	657	T	C	synonymous
250634	NTHI477_RS01320	675	G	C	synonymous
250640	NTHI477_RS01320	681	T	C	synonymous
250643	NTHI477_RS01320	684	A	G	synonymous
250643	NTHI477_RS01320	684	A	C	synonymous
250649	NTHI477_RS01320	690	A	G	synonymous
250655	NTHI477_RS01320	696	T	C	synonymous
250670	NTHI477_RS01320	711	A	G	synonymous
250673	NTHI477_RS01320	714	G	T	synonymous
250676	NTHI477_RS01320	717	G	T	synonymous

250679	NTHI477_RS01320	720	G	A	synonymous
250682	NTHI477_RS01320	723	G	T	synonymous
250682	NTHI477_RS01320	723	G	A	synonymous
250688	NTHI477_RS01320	729	T	A	synonymous
250688	NTHI477_RS01320	729	T	G	synonymous
250697	NTHI477_RS01320	738	A	G	synonymous
250700	NTHI477_RS01320	741	C	T	synonymous
250703	NTHI477_RS01320	744	C	T	synonymous
250704	NTHI477_RS01320	745	A	C	nonsynonymous
250707	NTHI477_RS01320	748	G	T	nonsynonymous
250708	NTHI477_RS01320	749	A	C	nonsynonymous
250712	NTHI477_RS01320	753	G	A	synonymous
250713	NTHI477_RS01320	754	C	A	nonsynonymous
250713	NTHI477_RS01320	754	C	G	nonsynonymous
250714	NTHI477_RS01320	755	A	T	nonsynonymous
250715	NTHI477_RS01320	756	A	C	nonsynonymous
250715	NTHI477_RS01320	756	A	T	nonsynonymous
250721	NTHI477_RS01320	762	G	A	synonymous
250724	NTHI477_RS01320	765	G	A	synonymous
250724	NTHI477_RS01320	765	G	T	synonymous
250727	NTHI477_RS01320	768	A	G	synonymous
250727	NTHI477_RS01320	768	A	T	synonymous
250728	NTHI477_RS01320	769	A	G	nonsynonymous
250733	NTHI477_RS01320	774	A	G	synonymous
250736	NTHI477_RS01320	777	G	A	synonymous
250737	NTHI477_RS01320	778	C	T	nonsynonymous
250739	NTHI477_RS01320	780	T	G	synonymous
250742	NTHI477_RS01320	783	A	T	synonymous
250745	NTHI477_RS01320	786	C	T	synonymous
250757	NTHI477_RS01320	798	G	A	synonymous
250766	NTHI477_RS01320	807	C	T	synonymous
250775	NTHI477_RS01320	816	T	C	synonymous
250778	NTHI477_RS01320	819	G	C	synonymous
250796	NTHI477_RS01320	837	A	T	synonymous
250802	NTHI477_RS01320	843	C	G	synonymous
250805	NTHI477_RS01320	846	G	T	synonymous
250805	NTHI477_RS01320	846	G	A	synonymous
250811	NTHI477_RS01320	852	C	T	synonymous
250817	NTHI477_RS01320	858	A	T	synonymous
250820	NTHI477_RS01320	861	A	G	synonymous
250823	NTHI477_RS01320	864	C	T	synonymous
250826	NTHI477_RS01320	867	T	C	synonymous
250829	NTHI477_RS01320	870	C	T	synonymous
250832	NTHI477_RS01320	873	C	T	synonymous
250838	NTHI477_RS01320	879	C	T	synonymous

250856	NTHI477_RS01320	897	T	C	synonymous
250858	NTHI477_RS01320	899	A	G	nonsynonymous
250859	NTHI477_RS01320	900	C	T	synonymous
250862	NTHI477_RS01320	903	A	G	synonymous
250869	NTHI477_RS01320	910	G	A	nonsynonymous
250871	NTHI477_RS01320	912	A	T	synonymous
250874	NTHI477_RS01320	915	T	C	synonymous
250875	NTHI477_RS01320	916	G	A	nonsynonymous
250877	NTHI477_RS01320	918	A	G	synonymous
250878	NTHI477_RS01320	919	T	C	synonymous
250880	NTHI477_RS01320	921	G	A	synonymous
250883	NTHI477_RS01320	924	G	C	synonymous
250884	NTHI477_RS01320	925	C	T	synonymous
250886	NTHI477_RS01320	927	G	A	synonymous
250889	NTHI477_RS01320	930	C	T	synonymous
250892	NTHI477_RS01320	933	A	G	synonymous
250893	NTHI477_RS01320	934	A	G	nonsynonymous
250958	NTHI477_RS01320	999	A	T	synonymous
250965	NTHI477_RS01320	1006	G	T	nonsynonymous
250985	NTHI477_RS01320	1026	A	G	synonymous
250991	NTHI477_RS01320	1032	T	C	synonymous
250997	NTHI477_RS01320	1038	G	A	synonymous
251000	NTHI477_RS01320	1041	T	C	synonymous
251009	NTHI477_RS01320	1050	T	C	synonymous
251012	NTHI477_RS01320	1053	C	G	synonymous
251026	NTHI477_RS01320	1067	C	T	nonsynonymous
251027	NTHI477_RS01320	1068	A	G	synonymous
251030	NTHI477_RS01320	1071	G	T	synonymous
251033	NTHI477_RS01320	1074	T	C	synonymous
251042	NTHI477_RS01320	1083	C	T	synonymous
251061	NTHI477_RS01320	1102	T	C	synonymous
251063	NTHI477_RS01320	1104	G	C	nonsynonymous
251099	NTHI477_RS01320	1140	C	T	synonymous
251114	NTHI477_RS01320	1155	C	T	synonymous
251115	NTHI477_RS01320	1156	G	A	nonsynonymous
251129	NTHI477_RS01320	1170	G	A	synonymous
251135	NTHI477_RS01320	1176	G	A	synonymous
251138	NTHI477_RS01320	1179	G	A	synonymous
251141	NTHI477_RS01320	1182	G	A	synonymous
251156	NTHI477_RS01320	1197	A	G	synonymous
251165	NTHI477_RS01320	1206	G	A	synonymous
251172	NTHI477_RS01320	1213	G	T	nonsynonymous
251174	NTHI477_RS01320	1215	T	C	synonymous
251189	NTHI477_RS01320	1230	C	T	synonymous
251195	NTHI477_RS01320	1236	G	A	synonymous

251204	NTHI477_RS01320	1245	C	T	synonymous
251249	NTHI477_RS01320	1290	T	C	synonymous
251279	NTHI477_RS01320	1320	T	C	synonymous
251300	NTHI477_RS01320	1341	G	A	synonymous
251309	NTHI477_RS01320	1350	T	C	synonymous
251312	NTHI477_RS01320	1353	T	A	synonymous
251312	NTHI477_RS01320	1353	T	G	synonymous
251318	NTHI477_RS01320	1359	C	T	synonymous
251321	NTHI477_RS01320	1362	A	G	synonymous
251339	NTHI477_RS01320	1380	G	A	synonymous
251390	NTHI477_RS01320	1431	C	T	synonymous
251393	NTHI477_RS01320	1434	A	G	synonymous
251394	NTHI477_RS01320	1435	T	C	synonymous
251399	NTHI477_RS01320	1440	C	T	synonymous
251417	NTHI477_RS01320	1458	T	C	synonymous
251431	NTHI477_RS01320	1472	C	T	nonsynonymous
251435	NTHI477_RS01320	1476	T	C	synonymous
251456	NTHI477_RS01320	1497	T	C	synonymous
251462	NTHI477_RS01320	1503	G	A	synonymous
251477	NTHI477_RS01320	1518	C	T	synonymous
251495	NTHI477_RS01320	1536	C	T	synonymous
251498	NTHI477_RS01320	1539	T	A	synonymous
251501	NTHI477_RS01320	1542	C	T	synonymous
251504	NTHI477_RS01320	1545	C	T	synonymous
251507	NTHI477_RS01320	1548	T	C	synonymous
251513	NTHI477_RS01320	1554	C	T	synonymous
251537	NTHI477_RS01320	1578	T	A	synonymous
251556	NTHI477_RS01320	1597	G	A	nonsynonymous
251558	NTHI477_RS01320	1599	A	G	synonymous
251579	NTHI477_RS01320	1620	C	A	synonymous
251585	NTHI477_RS01320	1626	A	G	synonymous
251597	NTHI477_RS01320	1638	G	C	synonymous
251603	NTHI477_RS01320	1644	T	C	synonymous
251651	NTHI477_RS01320	1692	A	G	synonymous
251657	NTHI477_RS01320	1698	T	C	synonymous
251669	NTHI477_RS01320	1710	T	C	synonymous
251672	NTHI477_RS01320	1713	A	G	synonymous
251687	NTHI477_RS01320	1728	C	T	synonymous
251693	NTHI477_RS01320	1734	C	T	synonymous
251720	NTHI477_RS01320	1761	C	T	synonymous
251726	NTHI477_RS01320	1767	T	C	synonymous
251762	NTHI477_RS01320	1803	A	T	synonymous
251768	NTHI477_RS01320	1809	G	A	synonymous
251771	NTHI477_RS01320	1812	C	T	synonymous
251771	NTHI477_RS01320	1812	C	G	synonymous

251774	NTHI477_RS01320	1815	T	C	synonymous
251780	NTHI477_RS01320	1821	G	A	synonymous
251789	NTHI477_RS01320	1830	G	A	synonymous
251798	NTHI477_RS01320	1839	C	A	synonymous
251798	NTHI477_RS01320	1839	C	G	synonymous
251807	NTHI477_RS01320	1848	C	T	synonymous
251813	NTHI477_RS01320	1854	C	T	synonymous
251825	NTHI477_RS01320	1866	C	T	synonymous
251834	NTHI477_RS01320	1875	T	G	synonymous
251837	NTHI477_RS01320	1878	G	A	synonymous
251840	NTHI477_RS01320	1881	A	G	synonymous
251843	NTHI477_RS01320	1884	T	C	synonymous
251849	NTHI477_RS01320	1890	C	T	synonymous
251855	NTHI477_RS01320	1896	T	C	synonymous
251858	NTHI477_RS01320	1899	C	T	synonymous
251861	NTHI477_RS01320	1902	C	T	synonymous
251879	NTHI477_RS01320	1920	T	C	synonymous
251885	NTHI477_RS01320	1926	A	G	synonymous
251888	NTHI477_RS01320	1929	T	C	synonymous
251897	NTHI477_RS01320	1938	C	T	synonymous
251906	NTHI477_RS01320	1947	T	C	synonymous
251912	NTHI477_RS01320	1953	A	G	synonymous
251939	NTHI477_RS01320	1980	G	A	synonymous
251945	NTHI477_RS01320	1986	C	A	nonsynonymous
251960	NTHI477_RS01320	2001	T	C	synonymous
251961	NTHI477_RS01320	2002	C	G	nonsynonymous
251969	NTHI477_RS01320	2010	A	G	synonymous
251972	NTHI477_RS01320	2013	T	C	synonymous
251975	NTHI477_RS01320	2016	A	G	synonymous
251981	NTHI477_RS01320	2022	A	C	synonymous
251982	NTHI477_RS01320	2023	T	G	nonsynonymous
251984	NTHI477_RS01320	2025	A	G	synonymous
251987	NTHI477_RS01320	2028	G	A	synonymous
251990	NTHI477_RS01320	2031	T	C	synonymous
252005	NTHI477_RS01320	2046	T	G	synonymous
252005	NTHI477_RS01320	2046	T	A	synonymous
252017	NTHI477_RS01320	2058	C	T	synonymous
252020	NTHI477_RS01320	2061	A	C	synonymous
252023	NTHI477_RS01320	2064	G	A	synonymous
252037	NTHI477_RS01320	2078	G	A	nonsynonymous
252038	NTHI477_RS01320	2079	C	T	synonymous
252046	NTHI477_RS01320	2087	G	A	nonsynonymous
252092	NTHI477_RS01320	2133	T	C	synonymous
252107	NTHI477_RS01320	2148	T	C	synonymous
252143	NTHI477_RS01320	2184	C	A	synonymous

252146	NTHI477_RS01320	2187	C	T	synonymous
252152	NTHI477_RS01320	2193	C	T	synonymous
252182	NTHI477_RS01320	2223	C	T	synonymous
252191	NTHI477_RS01320	2232	C	T	synonymous
252194	NTHI477_RS01320	2235	G	T	synonymous
252195	NTHI477_RS01320	2236	T	C	synonymous
252200	NTHI477_RS01320	2241	A	G	synonymous
252218	NTHI477_RS01320	2259	G	A	synonymous
252218	NTHI477_RS01320	2259	G	C	synonymous
252227	NTHI477_RS01320	2268	T	C	synonymous
252227	NTHI477_RS01320	2268	T	G	synonymous
252263	NTHI477_RS01320	2304	G	A	synonymous
252275	NTHI477_RS01320	2316	C	G	synonymous
252276	NTHI477_RS01320	2317	G	A	nonsynonymous
252284	NTHI477_RS01320	2325	G	T	synonymous
252285	NTHI477_RS01320	2326	G	A	nonsynonymous
252293	NTHI477_RS01320	2334	C	T	synonymous
252300	NTHI477_RS01320	2341	C	T	synonymous
252302	NTHI477_RS01320	2343	G	A	synonymous
252326	NTHI477_RS01320	2367	G	A	synonymous
252328	NTHI477_RS01320	2369	A	G	nonsynonymous
252340	NTHI477_RS01320	2381	T	C	nonsynonymous
252341	NTHI477_RS01320	2382	A	T	synonymous
252344	NTHI477_RS01320	2385	G	A	synonymous
252362	NTHI477_RS01320	2403	G	A	synonymous
252380	NTHI477_RS01320	2421	T	C	synonymous
252386	NTHI477_RS01320	2427	G	T	synonymous
252387	NTHI477_RS01320	2428	G	A	nonsynonymous
252401	NTHI477_RS01320	2442	T	A	synonymous
252412	NTHI477_RS01320	2453	A	C	nonsynonymous
252425	NTHI477_RS01320	2466	C	T	synonymous
252431	NTHI477_RS01320	2472	T	C	synonymous
252446	NTHI477_RS01320	2487	C	T	synonymous
252458	NTHI477_RS01320	2499	T	C	synonymous
252459	NTHI477_RS01320	2500	G	A	nonsynonymous
252506	NTHI477_RS01320	2547	C	A	synonymous
252533	NTHI477_RS01320	2574	G	A	synonymous
252540	NTHI477_RS01320	2581	G	A	nonsynonymous
252545	NTHI477_RS01320	2586	C	T	synonymous
252550	NTHI477_RS01320	2591	C	T	nonsynonymous
252551	NTHI477_RS01320	2592	A	C	synonymous
252557	NTHI477_RS01320	2598	A	G	synonymous
252563	NTHI477_RS01320	2604	C	G	synonymous
252563	NTHI477_RS01320	2604	C	A	synonymous
252573	NTHI477_RS01320	2614	T	C	synonymous

252575	NTHI477_RS01320	2616	A	C	nonsynonymous
252578	NTHI477_RS01320	2619	C	G	synonymous
252596	NTHI477_RS01320	2637	T	C	synonymous
252608	NTHI477_RS01320	2649	T	A	synonymous
252611	NTHI477_RS01320	2652	A	C	synonymous
252629	NTHI477_RS01320	2670	A	C	synonymous
252647	NTHI477_RS01320	2688	T	C	synonymous
252677	NTHI477_RS01320	2718	G	T	synonymous
252683	NTHI477_RS01320	2724	T	C	synonymous
252689	NTHI477_RS01320	2730	A	G	synonymous
252692	NTHI477_RS01320	2733	C	T	synonymous
252698	NTHI477_RS01320	2739	T	C	synonymous
252704	NTHI477_RS01320	2745	T	C	synonymous
252713	NTHI477_RS01320	2754	C	T	synonymous
252725	NTHI477_RS01320	2766	A	G	synonymous
252734	NTHI477_RS01320	2775	A	G	synonymous
252737	NTHI477_RS01320	2778	G	T	synonymous
252743	NTHI477_RS01320	2784	G	A	synonymous
252752	NTHI477_RS01320	2793	T	C	synonymous
252764	NTHI477_RS01320	2805	A	G	synonymous
252765	NTHI477_RS01320	2806	G	A	nonsynonymous
252806	NTHI477_RS01320	2847	A	G	synonymous
252811	NTHI477_RS01320	2852	T	C	nonsynonymous
252836	intergenic	intergenic	A	G	intergenic
252885	NTHI477_RS01325	246	T	C	synonymous
252891	NTHI477_RS01325	240	T	G	nonsynonymous
252941	NTHI477_RS01325	190	C	T	nonsynonymous
252967	NTHI477_RS01325	164	G	A	nonsynonymous
252979	NTHI477_RS01325	152	T	C	nonsynonymous
253012	NTHI477_RS01325	119	G	T	nonsynonymous
253019	NTHI477_RS01325	112	C	T	nonsynonymous
253028	NTHI477_RS01325	103	T	C	nonsynonymous
253046	NTHI477_RS01325	85	C	T	nonsynonymous
253076	NTHI477_RS01325	55	T	C	nonsynonymous
253114	NTHI477_RS01325	17	C	T	nonsynonymous
253115	NTHI477_RS01325	16	C	T	nonsynonymous
253116	NTHI477_RS01325	15	G	A	synonymous
253122	NTHI477_RS01325	9	C	T	synonymous
253237	NTHI477_RS01330	1386	T	G	synonymous
253243	NTHI477_RS01330	1380	C	A	synonymous
253255	NTHI477_RS01330	1368	C	T	synonymous
253258	NTHI477_RS01330	1365	T	C	synonymous
253261	NTHI477_RS01330	1362	A	T	synonymous
253269	NTHI477_RS01330	1354	T	G	nonsynonymous
253273	NTHI477_RS01330	1350	C	T	synonymous

253276	NTHI477_RS01330	1347	G	A	synonymous
253286	NTHI477_RS01330	1337	T	C	nonsynonymous
253287	NTHI477_RS01330	1336	T	C	nonsynonymous
253291	NTHI477_RS01330	1332	T	C	synonymous
253292	NTHI477_RS01330	1331	T	G	nonsynonymous
253293	NTHI477_RS01330	1330	G	C	nonsynonymous
253306	NTHI477_RS01330	1317	C	T	synonymous
253312	NTHI477_RS01330	1311	A	G	synonymous
253321	NTHI477_RS01330	1302	A	G	synonymous
253324	NTHI477_RS01330	1299	G	A	synonymous
253327	NTHI477_RS01330	1296	G	T	synonymous
253381	NTHI477_RS01330	1242	A	G	synonymous
253387	NTHI477_RS01330	1236	C	A	synonymous
253387	NTHI477_RS01330	1236	C	T	synonymous
253390	NTHI477_RS01330	1233	C	T	synonymous
253402	NTHI477_RS01330	1221	C	T	synonymous
253410	NTHI477_RS01330	1213	C	A	nonsynonymous
253423	NTHI477_RS01330	1200	A	G	synonymous
253435	NTHI477_RS01330	1188	A	T	nonsynonymous
253438	NTHI477_RS01330	1185	C	T	synonymous
253444	NTHI477_RS01330	1179	C	T	synonymous
253449	NTHI477_RS01330	1174	C	T	nonsynonymous
253453	NTHI477_RS01330	1170	A	G	synonymous
253453	NTHI477_RS01330	1170	A	T	synonymous
253462	NTHI477_RS01330	1161	G	A	synonymous
253467	NTHI477_RS01330	1156	C	T	nonsynonymous
253476	NTHI477_RS01330	1147	T	A	nonsynonymous
253476	NTHI477_RS01330	1147	T	C	nonsynonymous
253480	NTHI477_RS01330	1143	A	G	synonymous
253481	NTHI477_RS01330	1142	G	A	nonsynonymous
253489	NTHI477_RS01330	1134	A	T	synonymous
253489	NTHI477_RS01330	1134	A	C	synonymous
253495	NTHI477_RS01330	1128	C	T	synonymous
253522	NTHI477_RS01330	1101	G	T	synonymous
253524	NTHI477_RS01330	1099	G	A	nonsynonymous
253525	NTHI477_RS01330	1098	C	T	synonymous
253528	NTHI477_RS01330	1095	C	T	synonymous
253531	NTHI477_RS01330	1092	T	G	synonymous
253540	NTHI477_RS01330	1083	G	A	synonymous
253552	NTHI477_RS01330	1071	C	T	synonymous
253556	NTHI477_RS01330	1067	T	G	nonsynonymous
253591	NTHI477_RS01330	1032	T	C	synonymous
253594	NTHI477_RS01330	1029	C	T	synonymous
253598	NTHI477_RS01330	1025	T	C	nonsynonymous
253609	NTHI477_RS01330	1014	T	G	synonymous

253626	NTHI477_RS01330	997	T	C	nonsynonymous
253639	NTHI477_RS01330	984	T	A	synonymous
253660	NTHI477_RS01330	963	A	C	synonymous
253660	NTHI477_RS01330	963	A	T	synonymous
253664	NTHI477_RS01330	959	G	A	nonsynonymous
253681	NTHI477_RS01330	942	T	C	synonymous
253702	NTHI477_RS01330	921	A	G	synonymous
253718	NTHI477_RS01330	905	A	G	nonsynonymous
253735	NTHI477_RS01330	888	A	G	synonymous
253738	NTHI477_RS01330	885	G	A	synonymous
253744	NTHI477_RS01330	879	A	G	synonymous
253744	NTHI477_RS01330	879	A	C	synonymous
253752	NTHI477_RS01330	871	T	C	nonsynonymous
253774	NTHI477_RS01330	849	C	T	synonymous
253776	NTHI477_RS01330	847	C	T	nonsynonymous
253780	NTHI477_RS01330	843	T	C	synonymous
253813	NTHI477_RS01330	810	C	T	synonymous
253816	NTHI477_RS01330	807	C	T	synonymous
253848	NTHI477_RS01330	775	C	T	nonsynonymous
253855	NTHI477_RS01330	768	G	A	synonymous
253858	NTHI477_RS01330	765	A	G	synonymous
253878	NTHI477_RS01330	745	G	A	nonsynonymous
253879	NTHI477_RS01330	744	T	C	synonymous
253885	NTHI477_RS01330	738	T	C	synonymous
253894	NTHI477_RS01330	729	T	G	nonsynonymous
253898	NTHI477_RS01330	725	C	T	nonsynonymous
253920	NTHI477_RS01330	703	T	G	nonsynonymous
253927	NTHI477_RS01330	696	T	G	nonsynonymous
253947	NTHI477_RS01330	676	C	T	nonsynonymous
253954	NTHI477_RS01330	669	G	A	synonymous
253957	NTHI477_RS01330	666	T	A	synonymous
253959	NTHI477_RS01330	664	G	A	nonsynonymous
253974	NTHI477_RS01330	649	C	T	nonsynonymous
253983	NTHI477_RS01330	640	C	T	nonsynonymous
254005	NTHI477_RS01330	618	A	T	synonymous
254032	NTHI477_RS01330	591	G	A	synonymous
254041	NTHI477_RS01330	582	A	G	synonymous
254047	NTHI477_RS01330	576	C	T	synonymous
254049	NTHI477_RS01330	574	T	C	nonsynonymous
254053	NTHI477_RS01330	570	A	C	synonymous
254056	NTHI477_RS01330	567	C	T	synonymous
254062	NTHI477_RS01330	561	A	G	synonymous
254065	NTHI477_RS01330	558	A	G	synonymous
254077	NTHI477_RS01330	546	T	A	nonsynonymous
254086	NTHI477_RS01330	537	A	G	synonymous

254104	NTHI477_RS01330	519	G	A	synonymous
254110	NTHI477_RS01330	513	A	G	synonymous
254122	NTHI477_RS01330	501	T	C	synonymous
254128	NTHI477_RS01330	495	A	C	nonsynonymous
254149	NTHI477_RS01330	474	C	T	synonymous
254155	NTHI477_RS01330	468	G	A	synonymous
254164	NTHI477_RS01330	459	T	A	synonymous
254182	NTHI477_RS01330	441	C	T	synonymous
254200	NTHI477_RS01330	423	A	G	synonymous
254208	NTHI477_RS01330	415	G	A	nonsynonymous
254215	NTHI477_RS01330	408	C	T	synonymous
254227	NTHI477_RS01330	396	G	A	synonymous
254248	NTHI477_RS01330	375	A	C	synonymous
254248	NTHI477_RS01330	375	A	T	synonymous
254260	NTHI477_RS01330	363	C	T	synonymous
254267	NTHI477_RS01330	356	T	C	nonsynonymous
254269	NTHI477_RS01330	354	T	G	synonymous
254278	NTHI477_RS01330	345	A	C	synonymous
254284	NTHI477_RS01330	339	G	A	synonymous
254287	NTHI477_RS01330	336	C	T	synonymous
254288	NTHI477_RS01330	335	T	C	nonsynonymous
254296	NTHI477_RS01330	327	A	C	synonymous
254296	NTHI477_RS01330	327	A	G	synonymous
254302	NTHI477_RS01330	321	G	A	synonymous
254314	NTHI477_RS01330	309	A	G	synonymous
254317	NTHI477_RS01330	306	T	A	synonymous
254320	NTHI477_RS01330	303	C	T	synonymous
254323	NTHI477_RS01330	300	C	A	synonymous
254331	NTHI477_RS01330	292	T	C	nonsynonymous
254335	NTHI477_RS01330	288	G	A	synonymous
254338	NTHI477_RS01330	285	C	G	synonymous
254365	NTHI477_RS01330	258	T	G	nonsynonymous
254368	NTHI477_RS01330	255	T	C	synonymous
254371	NTHI477_RS01330	252	T	C	synonymous
254401	NTHI477_RS01330	222	G	A	synonymous
254407	NTHI477_RS01330	216	G	A	synonymous
254436	NTHI477_RS01330	187	C	T	nonsynonymous
254452	NTHI477_RS01330	171	G	A	synonymous
254460	NTHI477_RS01330	163	A	C	nonsynonymous
254469	NTHI477_RS01330	154	T	C	nonsynonymous
254488	NTHI477_RS01330	135	T	G	nonsynonymous
254509	NTHI477_RS01330	114	C	T	synonymous
254512	NTHI477_RS01330	111	A	T	synonymous
254527	NTHI477_RS01330	96	T	C	synonymous
254538	NTHI477_RS01330	85	C	T	nonsynonymous

254551	NTHI477_RS01330	72	T	C	synonymous
254557	NTHI477_RS01330	66	G	A	synonymous
254565	NTHI477_RS01330	58	C	T	nonsynonymous
254587	NTHI477_RS01330	36	A	G	synonymous
254590	NTHI477_RS01330	33	G	A	synonymous
254596	NTHI477_RS01330	27	A	G	synonymous
254602	NTHI477_RS01330	21	T	A	synonymous
254602	NTHI477_RS01330	21	T	G	synonymous
254605	NTHI477_RS01330	18	G	A	synonymous
254614	NTHI477_RS01330	9	G	A	synonymous
254617	NTHI477_RS01330	6	A	G	synonymous
254623	NTHI477_RS01330	0	A	C	synonymous
254624	intergenic	intergenic	G	T	intergenic
254624	intergenic	intergenic	G	A	intergenic
254634	intergenic	intergenic	A	T	intergenic
254635	intergenic	intergenic	G	A	intergenic
254640	intergenic	intergenic	A	G	intergenic
254640	intergenic	intergenic	A	C	intergenic
254646	intergenic	intergenic	C	A	intergenic
254647	intergenic	intergenic	G	A	intergenic
254648	intergenic	intergenic	C	A	intergenic
254649	intergenic	intergenic	A	T	intergenic
254650	intergenic	intergenic	A	T	intergenic
254651	intergenic	intergenic	C	T	intergenic
254660	NTHI477_RS01335	995	T	G	nonsynonymous
254661	NTHI477_RS01335	994	T	C	nonsynonymous
254662	NTHI477_RS01335	993	C	G	synonymous
254668	NTHI477_RS01335	987	G	T	nonsynonymous
254671	NTHI477_RS01335	984	T	C	synonymous
254676	NTHI477_RS01335	979	G	A	synonymous
254677	NTHI477_RS01335	978	C	T	synonymous
254682	NTHI477_RS01335	973	C	T	nonsynonymous
254686	NTHI477_RS01335	969	C	G	synonymous
254689	NTHI477_RS01335	966	T	G	nonsynonymous
254692	NTHI477_RS01335	963	A	G	synonymous
254699	NTHI477_RS01335	956	G	A	nonsynonymous
254707	NTHI477_RS01335	948	C	T	synonymous
254708	NTHI477_RS01335	947	A	G	nonsynonymous
254712	NTHI477_RS01335	943	A	G	synonymous
254717	NTHI477_RS01335	938	C	T	nonsynonymous
254737	NTHI477_RS01335	918	G	A	synonymous
254743	NTHI477_RS01335	912	A	G	synonymous
254745	NTHI477_RS01335	910	G	A	nonsynonymous
254746	NTHI477_RS01335	909	C	A	synonymous
254779	NTHI477_RS01335	876	C	A	synonymous

254785	NTHI477_RS01335	870	A	C	synonymous
254791	NTHI477_RS01335	864	T	A	synonymous
254792	NTHI477_RS01335	863	A	G	nonsynonymous
254794	NTHI477_RS01335	861	G	A	synonymous
254803	NTHI477_RS01335	852	T	C	synonymous
254812	NTHI477_RS01335	843	G	A	synonymous
254830	NTHI477_RS01335	825	G	C	synonymous
254841	NTHI477_RS01335	814	C	T	nonsynonymous
254847	NTHI477_RS01335	808	C	T	nonsynonymous
254857	NTHI477_RS01335	798	G	A	synonymous
254860	NTHI477_RS01335	795	G	A	synonymous
254869	NTHI477_RS01335	786	G	A	synonymous
254875	NTHI477_RS01335	780	T	G	synonymous
254989	NTHI477_RS01335	666	T	A	synonymous
254992	NTHI477_RS01335	663	G	A	synonymous
255019	NTHI477_RS01335	636	C	T	synonymous
255021	NTHI477_RS01335	634	C	T	nonsynonymous
255028	NTHI477_RS01335	627	G	A	synonymous
255052	NTHI477_RS01335	603	C	T	synonymous
255065	NTHI477_RS01335	590	A	G	nonsynonymous
255095	NTHI477_RS01335	560	C	T	nonsynonymous
255115	NTHI477_RS01335	540	C	A	synonymous
255130	NTHI477_RS01335	525	G	A	synonymous
255130	NTHI477_RS01335	525	G	T	synonymous
255136	NTHI477_RS01335	519	G	A	synonymous
255137	NTHI477_RS01335	518	C	T	nonsynonymous
255138	NTHI477_RS01335	517	A	G	nonsynonymous
255150	NTHI477_RS01335	505	A	C	nonsynonymous
255150	NTHI477_RS01335	505	A	T	nonsynonymous
255151	NTHI477_RS01335	504	T	G	synonymous
255153	NTHI477_RS01335	502	C	T	nonsynonymous
255163	NTHI477_RS01335	492	T	C	synonymous
255172	NTHI477_RS01335	483	G	A	synonymous
255178	NTHI477_RS01335	477	A	G	synonymous
255196	NTHI477_RS01335	459	C	A	synonymous
255208	NTHI477_RS01335	447	G	A	synonymous
255229	NTHI477_RS01335	426	T	C	synonymous
255245	NTHI477_RS01335	410	G	T	nonsynonymous
255246	NTHI477_RS01335	409	T	C	nonsynonymous
255256	NTHI477_RS01335	399	G	A	synonymous
255265	NTHI477_RS01335	390	C	A	synonymous
255268	NTHI477_RS01335	387	A	G	synonymous
255280	NTHI477_RS01335	375	G	A	synonymous
255283	NTHI477_RS01335	372	T	C	synonymous
255307	NTHI477_RS01335	348	C	T	synonymous

255334	NTHI477_RS01335	321	T	C	nonsynonymous
255336	NTHI477_RS01335	319	T	C	nonsynonymous
255341	NTHI477_RS01335	314	G	A	nonsynonymous
255355	NTHI477_RS01335	300	C	T	synonymous
255370	NTHI477_RS01335	285	A	G	synonymous
255372	NTHI477_RS01335	283	C	T	nonsynonymous
255373	NTHI477_RS01335	282	A	T	synonymous
255388	NTHI477_RS01335	267	G	A	synonymous
255397	NTHI477_RS01335	258	T	A	synonymous
255402	NTHI477_RS01335	253	C	T	nonsynonymous
255403	NTHI477_RS01335	252	G	A	synonymous
255406	NTHI477_RS01335	249	A	T	synonymous
255409	NTHI477_RS01335	246	C	T	synonymous
255415	NTHI477_RS01335	240	C	T	synonymous
255438	NTHI477_RS01335	217	C	T	nonsynonymous
255443	NTHI477_RS01335	212	C	T	nonsynonymous
255444	NTHI477_RS01335	211	G	A	nonsynonymous
255452	NTHI477_RS01335	203	A	G	nonsynonymous
255459	NTHI477_RS01335	196	C	A	nonsynonymous
255472	NTHI477_RS01335	183	C	G	synonymous
255477	NTHI477_RS01335	178	T	C	nonsynonymous
255481	NTHI477_RS01335	174	C	G	synonymous
255484	NTHI477_RS01335	171	G	A	synonymous
255487	NTHI477_RS01335	168	A	C	synonymous
255487	NTHI477_RS01335	168	A	T	synonymous
255489	NTHI477_RS01335	166	T	A	nonsynonymous
255496	NTHI477_RS01335	159	T	C	synonymous
255509	NTHI477_RS01335	146	C	T	nonsynonymous
255510	NTHI477_RS01335	145	T	C	nonsynonymous
255523	NTHI477_RS01335	132	G	A	synonymous
255526	NTHI477_RS01335	129	G	A	synonymous
255556	NTHI477_RS01335	99	G	A	synonymous
255574	NTHI477_RS01335	81	A	T	synonymous
255575	NTHI477_RS01335	80	A	G	nonsynonymous
255586	NTHI477_RS01335	69	T	A	synonymous
255589	NTHI477_RS01335	66	G	A	synonymous
255602	NTHI477_RS01335	53	C	G	nonsynonymous
255616	NTHI477_RS01335	39	T	C	synonymous
255631	NTHI477_RS01335	24	T	C	synonymous
255641	NTHI477_RS01335	14	T	G	nonsynonymous
255658	intergenic	intergenic	C	T	intergenic
255680	intergenic	intergenic	A	G	intergenic
255688	intergenic	intergenic	G	A	intergenic
255698	intergenic	intergenic	T	C	intergenic
255737	NTHI477_RS01340	357	C	T	synonymous

255740	NTHI477_RS01340	354	T	C	synonymous
255746	NTHI477_RS01340	348	G	A	synonymous
255749	NTHI477_RS01340	345	T	A	synonymous
255758	NTHI477_RS01340	336	T	G	synonymous
255761	NTHI477_RS01340	333	G	A	synonymous
255764	NTHI477_RS01340	330	G	A	synonymous
255776	NTHI477_RS01340	318	C	T	synonymous
255776	NTHI477_RS01340	318	C	G	synonymous
255779	NTHI477_RS01340	315	A	C	synonymous
255782	NTHI477_RS01340	312	T	C	synonymous
255800	NTHI477_RS01340	294	C	T	synonymous
255812	NTHI477_RS01340	282	G	A	synonymous
255821	NTHI477_RS01340	273	G	A	synonymous
255824	NTHI477_RS01340	270	G	A	synonymous
255839	NTHI477_RS01340	255	A	T	nonsynonymous
255848	NTHI477_RS01340	246	C	T	synonymous
255850	NTHI477_RS01340	244	G	A	synonymous
255872	NTHI477_RS01340	222	C	T	synonymous
255875	NTHI477_RS01340	219	G	A	synonymous
255887	NTHI477_RS01340	207	A	G	synonymous
255896	NTHI477_RS01340	198	C	T	synonymous
255899	NTHI477_RS01340	195	G	A	synonymous
255914	NTHI477_RS01340	180	C	G	synonymous
255926	NTHI477_RS01340	168	G	A	synonymous
255932	NTHI477_RS01340	162	G	A	synonymous
255935	NTHI477_RS01340	159	G	A	synonymous
255965	NTHI477_RS01340	129	A	G	synonymous
255980	NTHI477_RS01340	114	T	C	synonymous
255983	NTHI477_RS01340	111	A	G	synonymous
255986	NTHI477_RS01340	108	T	C	synonymous
255989	NTHI477_RS01340	105	A	G	synonymous
256001	NTHI477_RS01340	93	A	G	synonymous
256007	NTHI477_RS01340	87	G	T	synonymous
256016	NTHI477_RS01340	78	G	A	synonymous
256019	NTHI477_RS01340	75	G	A	synonymous
256022	NTHI477_RS01340	72	G	A	synonymous
256037	NTHI477_RS01340	57	T	C	synonymous
256040	NTHI477_RS01340	54	C	G	synonymous
256045	NTHI477_RS01340	49	G	T	nonsynonymous
256059	NTHI477_RS01340	35	A	G	nonsynonymous
256060	NTHI477_RS01340	34	A	C	nonsynonymous
256061	NTHI477_RS01340	33	A	G	synonymous
256085	NTHI477_RS01340	9	A	G	synonymous
256088	NTHI477_RS01340	6	G	A	synonymous
256124	intergenic	intergenic	T	C	intergenic

256137	intergenic	intergenic	T	G	intergenic
256138	intergenic	intergenic	C	A	intergenic
256177	NTHI477_RS01345	548	A	G	nonsynonymous
256209	NTHI477_RS01345	516	C	T	synonymous
256212	NTHI477_RS01345	513	A	G	synonymous
256215	NTHI477_RS01345	510	A	G	synonymous
256218	NTHI477_RS01345	507	G	A	synonymous
256254	NTHI477_RS01345	471	A	C	nonsynonymous
256263	NTHI477_RS01345	462	G	A	synonymous
256301	NTHI477_RS01345	424	G	T	nonsynonymous
256310	NTHI477_RS01345	415	T	C	nonsynonymous
256317	NTHI477_RS01345	408	C	T	nonsynonymous
256335	NTHI477_RS01345	390	T	C	synonymous
256339	NTHI477_RS01345	386	A	G	nonsynonymous
256353	NTHI477_RS01345	372	T	A	synonymous
256371	NTHI477_RS01345	354	T	C	synonymous
256371	NTHI477_RS01345	354	T	A	synonymous
256371	NTHI477_RS01345	354	T	G	synonymous
256386	NTHI477_RS01345	339	A	G	synonymous
256387	NTHI477_RS01345	338	C	G	nonsynonymous
256389	NTHI477_RS01345	336	G	T	synonymous
256413	NTHI477_RS01345	312	T	C	synonymous
256417	NTHI477_RS01345	308	T	G	nonsynonymous
256422	NTHI477_RS01345	303	A	T	synonymous
256425	NTHI477_RS01345	300	A	G	synonymous
256434	NTHI477_RS01345	291	G	C	nonsynonymous
256446	NTHI477_RS01345	279	A	G	synonymous
256458	NTHI477_RS01345	267	C	T	synonymous
256467	NTHI477_RS01345	258	C	A	synonymous
256467	NTHI477_RS01345	258	C	G	synonymous
256479	NTHI477_RS01345	246	T	G	synonymous
256482	NTHI477_RS01345	243	A	G	synonymous
256491	NTHI477_RS01345	234	T	C	synonymous
256496	NTHI477_RS01345	229	G	T	nonsynonymous
256506	NTHI477_RS01345	219	A	G	synonymous
256507	NTHI477_RS01345	218	C	T	nonsynonymous
256523	NTHI477_RS01345	202	G	A	synonymous
256533	NTHI477_RS01345	192	T	A	synonymous
256533	NTHI477_RS01345	192	T	C	synonymous
256541	NTHI477_RS01345	184	A	C	nonsynonymous
256545	NTHI477_RS01345	180	C	T	synonymous
256551	NTHI477_RS01345	174	T	A	synonymous
256553	NTHI477_RS01345	172	G	C	nonsynonymous
256557	NTHI477_RS01345	168	A	T	synonymous
256566	NTHI477_RS01345	159	C	T	synonymous

256582	NTHI477_RS01345	143	G	A	nonsynonymous
256583	NTHI477_RS01345	142	G	C	nonsynonymous
256590	NTHI477_RS01345	135	A	G	synonymous
256596	NTHI477_RS01345	129	G	A	synonymous
256601	NTHI477_RS01345	124	T	C	nonsynonymous
256604	NTHI477_RS01345	121	T	C	nonsynonymous
256617	NTHI477_RS01345	108	G	A	synonymous
256620	NTHI477_RS01345	105	G	A	synonymous
256629	NTHI477_RS01345	96	G	A	synonymous
256632	NTHI477_RS01345	93	A	G	synonymous
256653	NTHI477_RS01345	72	T	C	synonymous
256656	NTHI477_RS01345	69	T	C	synonymous
256662	NTHI477_RS01345	63	A	G	synonymous
256686	NTHI477_RS01345	39	A	G	synonymous
256755	NTHI477_RS01350	1539	A	T	synonymous
256782	NTHI477_RS01350	1512	T	C	synonymous
256785	NTHI477_RS01350	1509	G	A	synonymous
256785	NTHI477_RS01350	1509	G	T	synonymous
256791	NTHI477_RS01350	1503	G	A	synonymous
256806	NTHI477_RS01350	1488	G	A	synonymous
256824	NTHI477_RS01350	1470	A	G	synonymous
256833	NTHI477_RS01350	1461	T	C	synonymous
256839	NTHI477_RS01350	1455	G	A	synonymous
256839	NTHI477_RS01350	1455	G	T	synonymous
256851	NTHI477_RS01350	1443	T	C	synonymous
256854	NTHI477_RS01350	1440	C	T	synonymous
256860	NTHI477_RS01350	1434	G	A	synonymous
256865	NTHI477_RS01350	1429	C	T	nonsynonymous
256866	NTHI477_RS01350	1428	C	T	synonymous
256866	NTHI477_RS01350	1428	C	G	synonymous
256878	NTHI477_RS01350	1416	G	A	synonymous
256890	NTHI477_RS01350	1404	G	A	synonymous
256893	NTHI477_RS01350	1401	C	T	synonymous
256920	NTHI477_RS01350	1374	G	A	synonymous
256923	NTHI477_RS01350	1371	C	A	synonymous
256938	NTHI477_RS01350	1356	C	T	synonymous
256944	NTHI477_RS01350	1350	A	G	synonymous
256965	NTHI477_RS01350	1329	T	C	synonymous
256986	NTHI477_RS01350	1308	C	T	synonymous
256989	NTHI477_RS01350	1305	T	C	nonsynonymous
256992	NTHI477_RS01350	1302	T	C	synonymous
256998	NTHI477_RS01350	1296	G	A	synonymous
257004	NTHI477_RS01350	1290	T	G	synonymous
257007	NTHI477_RS01350	1287	C	T	synonymous
257007	NTHI477_RS01350	1287	C	G	synonymous

257010	NTHI477_RS01350	1284	A	T	synonymous
257034	NTHI477_RS01350	1260	G	A	synonymous
257055	NTHI477_RS01350	1239	C	T	synonymous
257064	NTHI477_RS01350	1230	C	T	synonymous
257085	NTHI477_RS01350	1209	T	C	synonymous
257088	NTHI477_RS01350	1206	A	G	synonymous
257094	NTHI477_RS01350	1200	G	C	synonymous
257097	NTHI477_RS01350	1197	C	T	synonymous
257106	NTHI477_RS01350	1188	G	A	synonymous
257127	NTHI477_RS01350	1167	T	C	synonymous
257136	NTHI477_RS01350	1158	T	C	synonymous
257139	NTHI477_RS01350	1155	T	A	synonymous
257142	NTHI477_RS01350	1152	C	G	synonymous
257142	NTHI477_RS01350	1152	C	T	synonymous
257151	NTHI477_RS01350	1143	G	C	synonymous
257157	NTHI477_RS01350	1137	A	G	synonymous
257158	NTHI477_RS01350	1136	C	T	nonsynonymous
257175	NTHI477_RS01350	1119	A	G	synonymous
257178	NTHI477_RS01350	1116	G	A	synonymous
257187	NTHI477_RS01350	1107	T	C	synonymous
257199	NTHI477_RS01350	1095	C	T	synonymous
257207	NTHI477_RS01350	1087	A	C	nonsynonymous
257214	NTHI477_RS01350	1080	C	T	synonymous
257226	NTHI477_RS01350	1068	G	A	synonymous
257235	NTHI477_RS01350	1059	G	A	synonymous
257244	NTHI477_RS01350	1050	T	C	synonymous
257256	NTHI477_RS01350	1038	A	C	synonymous
257256	NTHI477_RS01350	1038	A	T	synonymous
257262	NTHI477_RS01350	1032	C	T	synonymous
257274	NTHI477_RS01350	1020	G	A	synonymous
257277	NTHI477_RS01350	1017	A	G	synonymous
257280	NTHI477_RS01350	1014	G	T	synonymous
257291	NTHI477_RS01350	1003	C	T	nonsynonymous
257301	NTHI477_RS01350	993	A	C	synonymous
257307	NTHI477_RS01350	987	T	A	synonymous
257316	NTHI477_RS01350	978	G	A	synonymous
257319	NTHI477_RS01350	975	G	T	synonymous
257319	NTHI477_RS01350	975	G	A	synonymous
257322	NTHI477_RS01350	972	G	A	synonymous
257333	NTHI477_RS01350	961	A	G	synonymous
257346	NTHI477_RS01350	948	T	A	synonymous
257349	NTHI477_RS01350	945	T	C	synonymous
257350	NTHI477_RS01350	944	T	G	nonsynonymous
257350	NTHI477_RS01350	944	T	A	nonsynonymous
257352	NTHI477_RS01350	942	A	G	synonymous

257360	NTHI477_RS01350	934	A	G	synonymous
257403	NTHI477_RS01350	891	A	G	synonymous
257448	NTHI477_RS01350	846	G	A	synonymous
257451	NTHI477_RS01350	843	G	A	synonymous
257458	NTHI477_RS01350	836	G	A	nonsynonymous
257469	NTHI477_RS01350	825	C	A	synonymous
257475	NTHI477_RS01350	819	A	G	synonymous
257477	NTHI477_RS01350	817	C	T	nonsynonymous
257482	NTHI477_RS01350	812	G	A	nonsynonymous
257484	NTHI477_RS01350	810	A	G	synonymous
257490	NTHI477_RS01350	804	T	G	synonymous
257490	NTHI477_RS01350	804	T	A	synonymous
257496	NTHI477_RS01350	798	G	A	synonymous
257502	NTHI477_RS01350	792	T	A	synonymous
257511	NTHI477_RS01350	783	G	A	synonymous
257511	NTHI477_RS01350	783	G	T	synonymous
257517	NTHI477_RS01350	777	A	T	synonymous
257523	NTHI477_RS01350	771	A	G	synonymous
257535	NTHI477_RS01350	759	C	T	synonymous
257547	NTHI477_RS01350	747	C	T	synonymous
257550	NTHI477_RS01350	744	G	T	synonymous
257550	NTHI477_RS01350	744	G	A	synonymous
257553	NTHI477_RS01350	741	G	A	synonymous
257556	NTHI477_RS01350	738	G	A	synonymous
257589	NTHI477_RS01350	705	T	C	synonymous
257601	NTHI477_RS01350	693	T	C	synonymous
257602	NTHI477_RS01350	692	G	A	nonsynonymous
257603	NTHI477_RS01350	691	C	A	nonsynonymous
257607	NTHI477_RS01350	687	A	G	synonymous
257616	NTHI477_RS01350	678	A	G	synonymous
257621	NTHI477_RS01350	673	T	G	nonsynonymous
257643	NTHI477_RS01350	651	C	T	synonymous
257649	NTHI477_RS01350	645	A	G	synonymous
257655	NTHI477_RS01350	639	C	T	synonymous
257655	NTHI477_RS01350	639	C	G	synonymous
257658	NTHI477_RS01350	636	G	A	synonymous
257673	NTHI477_RS01350	621	G	A	synonymous
257706	NTHI477_RS01350	588	G	A	synonymous
257706	NTHI477_RS01350	588	G	T	synonymous
257712	NTHI477_RS01350	582	C	T	synonymous
257730	NTHI477_RS01350	564	A	G	synonymous
257736	NTHI477_RS01350	558	A	G	synonymous
257742	NTHI477_RS01350	552	G	T	synonymous
257744	NTHI477_RS01350	550	G	A	nonsynonymous
257745	NTHI477_RS01350	549	G	A	synonymous

257748	NTHI477_RS01350	546	C	T	synonymous
257751	NTHI477_RS01350	543	G	A	synonymous
257792	NTHI477_RS01350	502	G	T	nonsynonymous
257795	NTHI477_RS01350	499	G	T	nonsynonymous
257796	NTHI477_RS01350	498	T	C	synonymous
257805	NTHI477_RS01350	489	G	A	synonymous
257810	NTHI477_RS01350	484	C	T	nonsynonymous
257820	NTHI477_RS01350	474	G	A	synonymous
257824	NTHI477_RS01350	470	T	G	nonsynonymous
257853	NTHI477_RS01350	441	A	G	synonymous
257859	NTHI477_RS01350	435	G	A	synonymous
257879	NTHI477_RS01350	415	G	A	nonsynonymous
257888	NTHI477_RS01350	406	G	A	nonsynonymous
257890	NTHI477_RS01350	404	G	C	nonsynonymous
257897	NTHI477_RS01350	397	T	C	nonsynonymous
257904	NTHI477_RS01350	390	C	T	synonymous
257906	NTHI477_RS01350	388	G	A	synonymous
257907	NTHI477_RS01350	387	T	C	synonymous
257910	NTHI477_RS01350	384	G	A	synonymous
257922	NTHI477_RS01350	372	G	T	synonymous
257922	NTHI477_RS01350	372	G	A	synonymous
257923	NTHI477_RS01350	371	A	G	nonsynonymous
257928	NTHI477_RS01350	366	T	C	synonymous
257937	NTHI477_RS01350	357	G	A	synonymous
257938	NTHI477_RS01350	356	C	T	nonsynonymous
257940	NTHI477_RS01350	354	A	G	synonymous
257965	NTHI477_RS01350	329	A	G	nonsynonymous
257966	NTHI477_RS01350	328	G	A	synonymous
257967	NTHI477_RS01350	327	C	T	synonymous
257968	NTHI477_RS01350	326	G	A	nonsynonymous
257978	NTHI477_RS01350	316	C	T	nonsynonymous
257987	NTHI477_RS01350	307	C	T	nonsynonymous
257988	NTHI477_RS01350	306	G	C	nonsynonymous
257988	NTHI477_RS01350	306	G	T	nonsynonymous
257990	NTHI477_RS01350	304	C	T	nonsynonymous
258011	NTHI477_RS01350	283	C	T	nonsynonymous
258015	NTHI477_RS01350	279	T	C	synonymous
258038	NTHI477_RS01350	256	G	A	nonsynonymous
258068	NTHI477_RS01350	226	C	T	nonsynonymous
258069	NTHI477_RS01350	225	G	A	synonymous
258075	NTHI477_RS01350	219	T	A	synonymous
258078	NTHI477_RS01350	216	C	T	synonymous
258102	NTHI477_RS01350	192	G	A	synonymous
258105	NTHI477_RS01350	189	A	C	synonymous
258108	NTHI477_RS01350	186	G	T	synonymous

258111	NTHI477_RS01350	183	C	T	synonymous
258117	NTHI477_RS01350	177	C	T	synonymous
258117	NTHI477_RS01350	177	C	A	synonymous
258126	NTHI477_RS01350	168	G	A	synonymous
258132	NTHI477_RS01350	162	G	A	synonymous
258138	NTHI477_RS01350	156	G	A	synonymous
258150	NTHI477_RS01350	144	G	A	synonymous
258159	NTHI477_RS01350	135	C	T	synonymous
258162	NTHI477_RS01350	132	G	T	synonymous
258168	NTHI477_RS01350	126	A	G	synonymous
258180	NTHI477_RS01350	114	C	T	synonymous
258186	NTHI477_RS01350	108	A	G	synonymous
258228	NTHI477_RS01350	66	C	G	synonymous
258231	NTHI477_RS01350	63	A	G	synonymous
258243	NTHI477_RS01350	51	A	G	synonymous
258248	NTHI477_RS01350	46	C	T	nonsynonymous
258249	NTHI477_RS01350	45	G	T	synonymous
258251	NTHI477_RS01350	43	C	G	nonsynonymous
258251	NTHI477_RS01350	43	C	A	nonsynonymous
258257	NTHI477_RS01350	37	A	C	nonsynonymous
258261	NTHI477_RS01350	33	T	C	synonymous
258262	NTHI477_RS01350	32	A	G	nonsynonymous
258264	NTHI477_RS01350	30	C	T	synonymous
258278	NTHI477_RS01350	16	G	T	nonsynonymous
258281	NTHI477_RS01350	13	C	T	nonsynonymous
258288	NTHI477_RS01350	6	A	G	synonymous
258309	intergenic	intergenic	T	A	intergenic
258333	intergenic	intergenic	G	T	intergenic
258335	intergenic	intergenic	G	T	intergenic
258335	intergenic	intergenic	G	A	intergenic
258342	intergenic	intergenic	G	A	intergenic
258354	intergenic	intergenic	C	A	intergenic
258355	intergenic	intergenic	C	A	intergenic
258359	intergenic	intergenic	T	G	intergenic
258363	intergenic	intergenic	A	T	intergenic
258373	intergenic	intergenic	G	A	intergenic
258374	intergenic	intergenic	T	C	intergenic
258380	intergenic	intergenic	G	T	intergenic
258389	intergenic	intergenic	A	T	intergenic
258391	intergenic	intergenic	G	T	intergenic
258393	intergenic	intergenic	G	A	intergenic
258416	NTHI477_RS01355	849	G	A	synonymous
258456	NTHI477_RS01355	809	C	T	nonsynonymous
258495	NTHI477_RS01355	770	G	A	nonsynonymous
258500	NTHI477_RS01355	765	A	G	synonymous

258548	NTHI477_RS01355	717	G	T	synonymous
258548	NTHI477_RS01355	717	G	A	synonymous
258551	NTHI477_RS01355	714	T	C	synonymous
258554	NTHI477_RS01355	711	A	G	synonymous
258560	NTHI477_RS01355	705	T	A	synonymous
258596	NTHI477_RS01355	669	T	C	synonymous
258610	NTHI477_RS01355	655	T	C	nonsynonymous
258623	NTHI477_RS01355	642	A	G	synonymous
258625	NTHI477_RS01355	640	T	G	nonsynonymous
258635	NTHI477_RS01355	630	C	T	synonymous
258641	NTHI477_RS01355	624	A	C	synonymous
258641	NTHI477_RS01355	624	A	T	synonymous
258644	NTHI477_RS01355	621	A	G	synonymous
258650	NTHI477_RS01355	615	G	A	synonymous
258658	NTHI477_RS01355	607	C	A	nonsynonymous
258674	NTHI477_RS01355	591	T	C	synonymous
258677	NTHI477_RS01355	588	G	A	synonymous
258686	NTHI477_RS01355	579	A	G	synonymous
258711	NTHI477_RS01355	554	T	C	nonsynonymous
258714	NTHI477_RS01355	551	C	T	nonsynonymous
258715	NTHI477_RS01355	550	C	A	nonsynonymous
258717	NTHI477_RS01355	548	T	C	nonsynonymous
258721	NTHI477_RS01355	544	T	C	nonsynonymous
258722	NTHI477_RS01355	543	G	A	synonymous
258728	NTHI477_RS01355	537	G	A	synonymous
258729	NTHI477_RS01355	536	A	G	nonsynonymous
258733	NTHI477_RS01355	532	G	A	nonsynonymous
258737	NTHI477_RS01355	528	G	T	synonymous
258740	NTHI477_RS01355	525	A	T	synonymous
258741	NTHI477_RS01355	524	G	T	nonsynonymous
258742	NTHI477_RS01355	523	A	G	nonsynonymous
258743	NTHI477_RS01355	522	C	T	synonymous
258745	NTHI477_RS01355	520	G	T	nonsynonymous
258749	NTHI477_RS01355	516	T	A	synonymous
258754	NTHI477_RS01355	511	T	C	nonsynonymous
258755	NTHI477_RS01355	510	G	T	nonsynonymous
258757	NTHI477_RS01355	508	T	C	nonsynonymous
258764	NTHI477_RS01355	501	T	A	synonymous
258766	NTHI477_RS01355	499	T	C	nonsynonymous
258771	NTHI477_RS01355	494	G	A	nonsynonymous
258772	NTHI477_RS01355	493	A	C	nonsynonymous
258773	NTHI477_RS01355	492	A	G	synonymous
258778	NTHI477_RS01355	487	A	T	nonsynonymous
258781	NTHI477_RS01355	484	G	C	nonsynonymous
258783	NTHI477_RS01355	482	A	C	nonsynonymous

258787	NTHI477_RS01355	478	C	T	nonsynonymous
258789	NTHI477_RS01355	476	T	G	nonsynonymous
258791	NTHI477_RS01355	474	T	A	synonymous
258792	NTHI477_RS01355	473	G	T	nonsynonymous
258797	NTHI477_RS01355	468	G	A	synonymous
258800	NTHI477_RS01355	465	G	T	synonymous
258803	NTHI477_RS01355	462	A	G	synonymous
258809	NTHI477_RS01355	456	T	G	nonsynonymous
258835	NTHI477_RS01355	430	G	T	nonsynonymous
258843	NTHI477_RS01355	422	A	G	nonsynonymous
258845	NTHI477_RS01355	420	G	A	synonymous
258856	NTHI477_RS01355	409	C	A	nonsynonymous
258857	NTHI477_RS01355	408	G	T	synonymous
258862	NTHI477_RS01355	403	G	A	synonymous
258863	NTHI477_RS01355	402	T	G	synonymous
258869	NTHI477_RS01355	396	G	A	synonymous
258878	NTHI477_RS01355	387	T	A	synonymous
258887	NTHI477_RS01355	378	T	C	synonymous
258896	NTHI477_RS01355	369	C	G	nonsynonymous
258935	NTHI477_RS01355	330	A	G	synonymous
258944	NTHI477_RS01355	321	C	T	synonymous
258958	NTHI477_RS01355	307	T	G	nonsynonymous
258968	NTHI477_RS01355	297	C	A	synonymous
258980	NTHI477_RS01355	285	A	T	synonymous
258985	NTHI477_RS01355	280	T	C	nonsynonymous
258986	NTHI477_RS01355	279	A	T	synonymous
258989	NTHI477_RS01355	276	A	G	synonymous
258995	NTHI477_RS01355	270	A	T	synonymous
258996	NTHI477_RS01355	269	A	G	nonsynonymous
259001	NTHI477_RS01355	264	A	G	synonymous
259008	NTHI477_RS01355	257	A	G	nonsynonymous
259016	NTHI477_RS01355	249	C	T	synonymous
259019	NTHI477_RS01355	246	A	T	synonymous
259022	NTHI477_RS01355	243	A	T	synonymous
259034	NTHI477_RS01355	231	T	C	synonymous
259042	NTHI477_RS01355	223	C	T	nonsynonymous
259046	NTHI477_RS01355	219	T	A	synonymous
259049	NTHI477_RS01355	216	C	T	synonymous
259054	NTHI477_RS01355	211	T	C	nonsynonymous
259055	NTHI477_RS01355	210	T	A	synonymous
259058	NTHI477_RS01355	207	G	A	synonymous
259079	NTHI477_RS01355	186	T	G	synonymous
259087	NTHI477_RS01355	178	T	C	nonsynonymous
259091	NTHI477_RS01355	174	A	G	synonymous
259094	NTHI477_RS01355	171	T	A	synonymous

259097	NTHI477_RS01355	168	G	A	synonymous
259097	NTHI477_RS01355	168	G	C	nonsynonymous
259106	NTHI477_RS01355	159	T	A	synonymous
259108	NTHI477_RS01355	157	C	T	nonsynonymous
259114	NTHI477_RS01355	151	C	T	nonsynonymous
259121	NTHI477_RS01355	144	C	T	synonymous
259159	NTHI477_RS01355	106	C	A	nonsynonymous
259163	NTHI477_RS01355	102	C	A	synonymous
259165	NTHI477_RS01355	100	C	A	nonsynonymous
259176	NTHI477_RS01355	89	C	T	nonsynonymous
259181	NTHI477_RS01355	84	A	T	synonymous
259193	NTHI477_RS01355	72	C	T	synonymous
259199	NTHI477_RS01355	66	A	C	synonymous
259212	NTHI477_RS01355	53	C	T	nonsynonymous
259213	NTHI477_RS01355	52	C	T	nonsynonymous
259216	NTHI477_RS01355	49	T	C	nonsynonymous
259219	NTHI477_RS01355	46	G	A	nonsynonymous
259227	NTHI477_RS01355	38	G	A	nonsynonymous
259250	NTHI477_RS01355	15	G	A	synonymous
259272	intergenic	intergenic	G	A	intergenic
259305	intergenic	intergenic	C	T	intergenic
259313	intergenic	intergenic	C	T	intergenic
259327	intergenic	intergenic	T	C	intergenic
259330	intergenic	intergenic	T	G	intergenic
259351	intergenic	intergenic	G	A	intergenic
259365	intergenic	intergenic	T	C	intergenic
259367	intergenic	intergenic	C	A	intergenic
259368	intergenic	intergenic	T	G	intergenic
259369	intergenic	intergenic	C	A	intergenic
259375	intergenic	intergenic	T	C	intergenic
259379	intergenic	intergenic	C	G	intergenic
259380	intergenic	intergenic	T	C	intergenic
259413	intergenic	intergenic	G	A	intergenic
259421	intergenic	intergenic	A	G	intergenic
259435	intergenic	intergenic	C	A	intergenic
259451	intergenic	intergenic	G	A	intergenic
259468	NTHI477_RS01360	490	C	T	nonsynonymous
259469	NTHI477_RS01360	489	G	A	synonymous
259474	NTHI477_RS01360	484	C	A	nonsynonymous
259496	NTHI477_RS01360	462	A	G	synonymous
259501	NTHI477_RS01360	457	A	G	synonymous
259510	NTHI477_RS01360	448	T	C	nonsynonymous
259531	NTHI477_RS01360	427	G	A	nonsynonymous
259550	NTHI477_RS01360	408	A	T	nonsynonymous
259552	NTHI477_RS01360	406	T	C	nonsynonymous

259553	NTHI477_RS01360	405	A	G	synonymous
259556	NTHI477_RS01360	402	G	A	synonymous
259577	NTHI477_RS01360	381	T	C	synonymous
259693	NTHI477_RS01360	265	T	C	nonsynonymous
259702	NTHI477_RS01360	256	C	T	nonsynonymous
259729	NTHI477_RS01360	229	C	A	nonsynonymous
259733	NTHI477_RS01360	225	C	T	synonymous
259753	NTHI477_RS01360	205	T	C	nonsynonymous
259758	NTHI477_RS01360	200	C	A	nonsynonymous
259787	NTHI477_RS01360	171	C	T	synonymous
259802	NTHI477_RS01360	156	C	T	synonymous
259816	NTHI477_RS01360	142	T	C	nonsynonymous
259817	NTHI477_RS01360	141	T	A	synonymous
259820	NTHI477_RS01360	138	G	A	synonymous
259829	NTHI477_RS01360	129	C	T	synonymous
259832	NTHI477_RS01360	126	G	A	synonymous
259872	NTHI477_RS01360	86	G	T	nonsynonymous
259907	NTHI477_RS01360	51	C	T	synonymous
259936	NTHI477_RS01360	22	T	C	nonsynonymous
259959	intergenic	intergenic	T	C	intergenic
260102	NTHI477_RS01365	360	C	T	synonymous
260130	NTHI477_RS01365	332	C	T	nonsynonymous
260237	NTHI477_RS01365	225	T	A	synonymous
260411	NTHI477_RS01365	51	C	T	synonymous
260504	intergenic	intergenic	G	A	intergenic
260514	intergenic	intergenic	G	A	intergenic
260517	intergenic	intergenic	A	C	intergenic
260529	intergenic	intergenic	T	G	intergenic
260545	intergenic	intergenic	C	A	intergenic
260553	intergenic	intergenic	T	G	intergenic
260562	intergenic	intergenic	A	T	intergenic
260604	intergenic	intergenic	A	G	intergenic
260604	intergenic	intergenic	A	T	intergenic
260607	intergenic	intergenic	A	C	intergenic
260609	intergenic	intergenic	T	G	intergenic
260613	intergenic	intergenic	A	C	intergenic
260646	intergenic	intergenic	G	T	intergenic
260646	intergenic	intergenic	G	A	intergenic
260665	intergenic	intergenic	C	A	intergenic
260675	intergenic	intergenic	A	T	intergenic
260697	intergenic	intergenic	C	T	intergenic
260990	NTHI477_RS01370	12	T	C	synonymous
261018	NTHI477_RS01370	40	T	C	synonymous
261053	NTHI477_RS01370	75	T	G	synonymous
261092	NTHI477_RS01370	114	A	G	synonymous

261152	NTHI477_RS01370	174	C	T	synonymous
261206	NTHI477_RS01370	228	A	G	synonymous
261291	NTHI477_RS01370	313	G	A	nonsynonymous
261375	NTHI477_RS01370	397	G	A	nonsynonymous
261446	NTHI477_RS01370	468	C	T	synonymous
261486	NTHI477_RS01370	508	G	A	nonsynonymous
261554	NTHI477_RS01370	576	A	G	synonymous
261570	NTHI477_RS01370	592	G	A	nonsynonymous
261601	NTHI477_RS01370	623	G	C	nonsynonymous
261710	NTHI477_RS01370	732	A	C	synonymous
261743	NTHI477_RS01370	765	A	G	synonymous
261800	NTHI477_RS01370	822	G	A	synonymous
261836	NTHI477_RS01370	858	T	C	synonymous
261941	NTHI477_RS01370	963	G	A	synonymous
262022	intergenic	intergenic	A	G	intergenic
262026	intergenic	intergenic	C	T	intergenic
262029	intergenic	intergenic	A	G	intergenic
262323	NTHI477_RS01375	249	C	T	synonymous
262527	NTHI477_RS01375	453	G	T	synonymous
262528	NTHI477_RS01375	454	A	G	nonsynonymous
262529	NTHI477_RS01375	455	A	G	nonsynonymous
262710	NTHI477_RS01375	636	A	G	synonymous
262825	NTHI477_RS01375	751	T	G	nonsynonymous
263055	NTHI477_RS01380	32	G	T	nonsynonymous
263104	NTHI477_RS01380	81	C	A	synonymous
263203	NTHI477_RS01380	180	C	T	synonymous
263281	NTHI477_RS01380	258	G	T	synonymous
263500	NTHI477_RS01380	477	T	C	synonymous
263503	NTHI477_RS01380	480	G	A	synonymous
263602	NTHI477_RS01380	579	A	G	synonymous
263623	NTHI477_RS01380	600	T	C	synonymous
263629	NTHI477_RS01380	606	G	A	synonymous
263644	NTHI477_RS01380	621	C	T	synonymous
263645	NTHI477_RS01380	622	C	A	synonymous
263668	NTHI477_RS01380	645	G	A	synonymous
263683	NTHI477_RS01380	660	T	A	synonymous
263810	NTHI477_RS01380	787	A	G	nonsynonymous
263989	NTHI477_RS01385	108	G	A	synonymous
264058	NTHI477_RS01385	177	A	G	synonymous
264151	NTHI477_RS01385	270	G	A	synonymous
264376	NTHI477_RS01385	495	G	A	synonymous
264403	NTHI477_RS01385	522	G	A	synonymous
264404	NTHI477_RS01385	523	T	C	synonymous
264406	NTHI477_RS01385	525	A	C	nonsynonymous
264496	NTHI477_RS01385	615	A	G	nonsynonymous

264547	NTHI477_RS01385	666	C	T	synonymous
264634	NTHI477_RS01385	753	C	T	synonymous
264655	intergenic	intergenic	T	C	intergenic
264663	intergenic	intergenic	A	T	intergenic
264800	NTHI477_RS01390	55	G	A	nonsynonymous
264910	NTHI477_RS01390	165	A	C	synonymous
264913	NTHI477_RS01390	168	T	A	synonymous
264952	NTHI477_RS01390	207	G	A	synonymous
264988	NTHI477_RS01390	243	T	C	synonymous
265117	NTHI477_RS01390	372	A	G	synonymous
265166	NTHI477_RS01390	421	G	T	nonsynonymous
265351	NTHI477_RS01390	606	T	C	synonymous
265360	NTHI477_RS01390	615	T	C	synonymous
265376	NTHI477_RS01390	631	C	G	nonsynonymous
265381	NTHI477_RS01390	636	T	C	synonymous
265394	NTHI477_RS01390	649	C	A	synonymous
265402	NTHI477_RS01390	657	T	C	synonymous
265420	NTHI477_RS01390	675	T	C	synonymous
265478	NTHI477_RS01395	41	C	T	nonsynonymous
265574	NTHI477_RS01395	137	T	G	nonsynonymous
265576	NTHI477_RS01395	139	C	A	nonsynonymous
265578	NTHI477_RS01395	141	C	A	synonymous
265578	NTHI477_RS01395	141	C	T	synonymous
265629	NTHI477_RS01395	192	A	G	synonymous
265664	NTHI477_RS01395	227	A	G	nonsynonymous
265733	NTHI477_RS01395	296	C	A	nonsynonymous
265738	NTHI477_RS01395	301	G	T	nonsynonymous
265778	NTHI477_RS01395	341	G	T	nonsynonymous
265791	NTHI477_RS01395	354	G	A	synonymous
265793	NTHI477_RS01395	356	A	T	nonsynonymous
265812	NTHI477_RS01395	375	C	T	synonymous
265821	NTHI477_RS01395	384	A	T	nonsynonymous
265854	NTHI477_RS01395	417	T	G	nonsynonymous
265861	NTHI477_RS01395	424	C	A	nonsynonymous
265916	NTHI477_RS01395	479	C	A	nonsynonymous
265978	NTHI477_RS01395	541	A	G	nonsynonymous
266040	NTHI477_RS01395	603	T	C	synonymous
266111	NTHI477_RS01395	674	T	C	nonsynonymous
266116	NTHI477_RS01395	679	A	G	nonsynonymous
266172	NTHI477_RS01395	735	A	G	synonymous
266237	NTHI477_RS01395	800	A	C	nonsynonymous
266307	NTHI477_RS01395	870	T	C	synonymous
266325	NTHI477_RS01395	888	G	A	synonymous
266354	NTHI477_RS01395	917	C	T	nonsynonymous
266359	NTHI477_RS01395	922	C	T	synonymous

266376	NTHI477_RS01395	939	A	C	synonymous
266409	NTHI477_RS01395	972	T	C	synonymous
266431	NTHI477_RS01395	994	C	A	nonsynonymous
266649	NTHI477_RS01395	1212	G	T	synonymous
266697	NTHI477_RS01395	1260	T	G	nonsynonymous
266722	intergenic	intergenic	G	A	intergenic
266777	intergenic	intergenic	A	G	intergenic
266779	intergenic	intergenic	C	T	intergenic
266793	intergenic	intergenic	G	A	intergenic
266817	intergenic	intergenic	T	G	intergenic
266854	intergenic	intergenic	G	C	intergenic
266868	intergenic	intergenic	T	G	intergenic
266899	intergenic	intergenic	C	T	intergenic
266902	intergenic	intergenic	A	C	intergenic
266914	intergenic	intergenic	G	A	intergenic
266936	intergenic	intergenic	G	A	intergenic
266958	intergenic	intergenic	A	G	intergenic
267026	intergenic	intergenic	G	A	intergenic
267053	intergenic	intergenic	T	C	intergenic
267067	intergenic	intergenic	C	T	intergenic
267074	intergenic	intergenic	A	G	intergenic
267075	intergenic	intergenic	C	T	intergenic
267113	intergenic	intergenic	G	A	intergenic
267121	intergenic	intergenic	A	G	intergenic
267122	intergenic	intergenic	A	G	intergenic
267133	intergenic	intergenic	C	T	intergenic
267147	intergenic	intergenic	G	C	intergenic
267154	intergenic	intergenic	A	G	intergenic
267159	intergenic	intergenic	A	G	intergenic
267162	intergenic	intergenic	C	T	intergenic
267166	intergenic	intergenic	G	A	intergenic
267168	intergenic	intergenic	C	T	intergenic
267169	intergenic	intergenic	A	G	intergenic
267194	intergenic	intergenic	C	A	intergenic
267196	intergenic	intergenic	A	G	intergenic
267212	intergenic	intergenic	T	C	intergenic
267214	intergenic	intergenic	A	C	intergenic
267244	intergenic	intergenic	A	G	intergenic
267247	intergenic	intergenic	T	G	intergenic
267258	intergenic	intergenic	A	G	intergenic
267270	intergenic	intergenic	G	A	intergenic
267277	intergenic	intergenic	G	A	intergenic
267286	intergenic	intergenic	C	A	intergenic
267303	intergenic	intergenic	C	T	intergenic
267304	intergenic	intergenic	T	A	intergenic

267346	intergenic	intergenic	T	C	intergenic
267349	intergenic	intergenic	T	C	intergenic
267350	intergenic	intergenic	G	T	intergenic
267352	intergenic	intergenic	C	A	intergenic
267384	NTHI477_RS01400	1411	A	G	synonymous
267451	NTHI477_RS01400	1344	A	G	synonymous
267468	NTHI477_RS01400	1327	T	C	nonsynonymous
267474	NTHI477_RS01400	1321	C	G	nonsynonymous
267519	NTHI477_RS01400	1276	T	G	nonsynonymous
267559	NTHI477_RS01400	1236	A	G	synonymous
267562	NTHI477_RS01400	1233	A	G	synonymous
267568	NTHI477_RS01400	1227	G	T	synonymous
267568	NTHI477_RS01400	1227	G	A	synonymous
267576	NTHI477_RS01400	1219	G	A	nonsynonymous
267580	NTHI477_RS01400	1215	T	C	synonymous
267638	NTHI477_RS01400	1157	G	T	nonsynonymous
267667	NTHI477_RS01400	1128	A	G	synonymous
267708	NTHI477_RS01400	1087	T	C	nonsynonymous
267719	NTHI477_RS01400	1076	T	C	nonsynonymous
267840	NTHI477_RS01400	955	T	C	nonsynonymous
267892	NTHI477_RS01400	903	G	A	synonymous
267904	NTHI477_RS01400	891	C	T	synonymous
267916	NTHI477_RS01400	879	A	G	synonymous
267928	NTHI477_RS01400	867	T	C	synonymous
267942	NTHI477_RS01400	853	T	C	nonsynonymous
267943	NTHI477_RS01400	852	A	C	synonymous
267944	NTHI477_RS01400	851	G	A	nonsynonymous
267952	NTHI477_RS01400	843	G	T	synonymous
267966	NTHI477_RS01400	829	T	C	nonsynonymous
267967	NTHI477_RS01400	828	C	T	synonymous
267985	NTHI477_RS01400	810	T	C	synonymous
267990	NTHI477_RS01400	805	C	T	nonsynonymous
268024	NTHI477_RS01400	771	A	C	synonymous
268030	NTHI477_RS01400	765	G	T	synonymous
268032	NTHI477_RS01400	763	G	A	nonsynonymous
268035	NTHI477_RS01400	760	C	T	nonsynonymous
268036	NTHI477_RS01400	759	A	G	synonymous
268042	NTHI477_RS01400	753	A	G	synonymous
268059	NTHI477_RS01400	736	C	A	nonsynonymous
268132	NTHI477_RS01400	663	T	C	synonymous
268134	NTHI477_RS01400	661	C	A	nonsynonymous
268135	NTHI477_RS01400	660	G	T	synonymous
268146	NTHI477_RS01400	649	C	T	nonsynonymous
268147	NTHI477_RS01400	648	T	C	synonymous
268165	NTHI477_RS01400	630	T	C	synonymous

268171	NTHI477_RS01400	624	G	T	synonymous
268174	NTHI477_RS01400	621	A	G	synonymous
268204	NTHI477_RS01400	591	C	T	synonymous
268268	NTHI477_RS01400	527	G	A	nonsynonymous
268354	NTHI477_RS01400	441	T	C	synonymous
268368	NTHI477_RS01400	427	A	G	synonymous
268378	NTHI477_RS01400	417	G	A	synonymous
268384	NTHI477_RS01400	411	C	T	synonymous
268417	NTHI477_RS01400	378	A	G	synonymous
268447	NTHI477_RS01400	348	A	G	synonymous
268471	NTHI477_RS01400	324	C	T	synonymous
268567	NTHI477_RS01400	228	T	A	synonymous
268594	NTHI477_RS01400	201	A	C	synonymous
268594	NTHI477_RS01400	201	A	T	synonymous
268597	NTHI477_RS01400	198	C	T	synonymous
268609	NTHI477_RS01400	186	G	T	synonymous
268618	NTHI477_RS01400	177	G	C	synonymous
268630	NTHI477_RS01400	165	C	T	synonymous
268674	NTHI477_RS01400	121	T	C	nonsynonymous
268675	NTHI477_RS01400	120	A	G	synonymous
268702	NTHI477_RS01400	93	A	G	synonymous
268726	NTHI477_RS01400	69	G	A	synonymous
268733	NTHI477_RS01400	62	G	A	nonsynonymous
268785	NTHI477_RS01400	10	C	T	nonsynonymous
268796	intergenic	intergenic	A	G	intergenic
268813	NTHI477_RS01405	870	G	A	synonymous
268816	NTHI477_RS01405	867	A	G	synonymous
268818	NTHI477_RS01405	865	G	T	nonsynonymous
268840	NTHI477_RS01405	843	G	A	synonymous
268849	NTHI477_RS01405	834	C	T	synonymous
268858	NTHI477_RS01405	825	A	G	synonymous
268867	NTHI477_RS01405	816	A	G	synonymous
268870	NTHI477_RS01405	813	T	A	synonymous
268872	NTHI477_RS01405	811	G	T	nonsynonymous
268885	NTHI477_RS01405	798	A	G	synonymous
268888	NTHI477_RS01405	795	G	A	synonymous
268892	NTHI477_RS01405	791	T	C	nonsynonymous
268924	NTHI477_RS01405	759	A	G	synonymous
268929	NTHI477_RS01405	754	G	A	synonymous
268930	NTHI477_RS01405	753	G	A	synonymous
268936	NTHI477_RS01405	747	T	C	synonymous
268939	NTHI477_RS01405	744	C	G	synonymous
268942	NTHI477_RS01405	741	G	A	synonymous
268945	NTHI477_RS01405	738	A	G	synonymous
268954	NTHI477_RS01405	729	C	A	synonymous

268969	NTHI477_RS01405	714	C	T	synonymous
268993	NTHI477_RS01405	690	T	G	synonymous
269074	NTHI477_RS01405	609	C	G	synonymous
269101	NTHI477_RS01405	582	C	T	nonsynonymous
269110	NTHI477_RS01405	573	A	G	synonymous
269116	NTHI477_RS01405	567	A	C	synonymous
269125	NTHI477_RS01405	558	A	G	synonymous
269134	NTHI477_RS01405	549	C	T	synonymous
269146	NTHI477_RS01405	537	A	G	synonymous
269152	NTHI477_RS01405	531	A	G	synonymous
269155	NTHI477_RS01405	528	A	G	synonymous
269158	NTHI477_RS01405	525	G	A	synonymous
269167	NTHI477_RS01405	516	T	C	synonymous
269173	NTHI477_RS01405	510	T	C	synonymous
269193	NTHI477_RS01405	490	T	C	nonsynonymous
269194	NTHI477_RS01405	489	G	T	synonymous
269209	NTHI477_RS01405	474	G	C	synonymous
269212	NTHI477_RS01405	471	G	A	synonymous
269242	NTHI477_RS01405	441	G	C	synonymous
269284	NTHI477_RS01405	399	T	C	synonymous
269356	NTHI477_RS01405	327	T	A	synonymous
269374	NTHI477_RS01405	309	T	C	synonymous
269395	NTHI477_RS01405	288	A	G	synonymous
269398	NTHI477_RS01405	285	T	A	synonymous
269425	NTHI477_RS01405	258	T	C	synonymous
269518	NTHI477_RS01405	165	C	T	synonymous
269524	NTHI477_RS01405	159	G	A	synonymous
269535	NTHI477_RS01405	148	C	T	nonsynonymous
269566	NTHI477_RS01405	117	G	A	synonymous
269569	NTHI477_RS01405	114	T	C	synonymous
269572	NTHI477_RS01405	111	A	G	synonymous
269607	NTHI477_RS01405	76	T	C	nonsynonymous
269629	NTHI477_RS01405	54	C	A	synonymous
269632	NTHI477_RS01405	51	T	C	synonymous
269667	NTHI477_RS01405	16	A	G	nonsynonymous
269721	NTHI477_RS01410	874	T	C	nonsynonymous
269740	NTHI477_RS01410	855	G	A	synonymous
269748	NTHI477_RS01410	847	T	C	nonsynonymous
269749	NTHI477_RS01410	846	G	A	synonymous
269751	NTHI477_RS01410	844	T	C	nonsynonymous
269752	NTHI477_RS01410	843	G	A	synonymous
269770	NTHI477_RS01410	825	C	T	synonymous
269800	NTHI477_RS01410	795	T	G	synonymous
269818	NTHI477_RS01410	777	G	A	synonymous
269824	NTHI477_RS01410	771	C	T	synonymous

269869	NTHI477_RS01410	726	G	A	synonymous
269902	NTHI477_RS01410	693	G	A	synonymous
269920	NTHI477_RS01410	675	T	A	nonsynonymous
269923	NTHI477_RS01410	672	A	G	synonymous
269925	NTHI477_RS01410	670	T	C	nonsynonymous
269935	NTHI477_RS01410	660	G	T	synonymous
269940	NTHI477_RS01410	655	T	C	nonsynonymous
269941	NTHI477_RS01410	654	A	T	synonymous
269942	NTHI477_RS01410	653	G	T	nonsynonymous
269950	NTHI477_RS01410	645	A	G	synonymous
269959	NTHI477_RS01410	636	G	T	synonymous
269960	NTHI477_RS01410	635	C	G	nonsynonymous
269965	NTHI477_RS01410	630	T	C	synonymous
269966	NTHI477_RS01410	629	T	C	nonsynonymous
269971	NTHI477_RS01410	624	A	T	nonsynonymous
269972	NTHI477_RS01410	623	T	G	nonsynonymous
269973	NTHI477_RS01410	622	C	A	nonsynonymous
269974	NTHI477_RS01410	621	T	C	synonymous
269977	NTHI477_RS01410	618	G	A	synonymous
269982	NTHI477_RS01410	613	G	A	synonymous
269986	NTHI477_RS01410	609	T	C	synonymous
269998	NTHI477_RS01410	597	T	A	synonymous
270000	NTHI477_RS01410	595	A	T	nonsynonymous
270008	NTHI477_RS01410	587	C	G	nonsynonymous
270009	NTHI477_RS01410	586	T	C	nonsynonymous
270024	NTHI477_RS01410	571	C	A	nonsynonymous
270050	NTHI477_RS01410	545	G	A	nonsynonymous
270052	NTHI477_RS01410	543	A	T	synonymous
270055	NTHI477_RS01410	540	A	G	synonymous
270057	NTHI477_RS01410	538	A	G	nonsynonymous
270058	NTHI477_RS01410	537	T	C	synonymous
270061	NTHI477_RS01410	534	T	C	synonymous
270064	NTHI477_RS01410	531	A	T	nonsynonymous
270065	NTHI477_RS01410	530	T	C	nonsynonymous
270067	NTHI477_RS01410	528	T	C	synonymous
270074	NTHI477_RS01410	521	A	T	nonsynonymous
270082	NTHI477_RS01410	513	A	T	synonymous
270094	NTHI477_RS01410	501	C	A	synonymous
270107	NTHI477_RS01410	488	G	A	nonsynonymous
270148	NTHI477_RS01410	447	G	A	synonymous
270190	NTHI477_RS01410	405	T	C	synonymous
270219	NTHI477_RS01410	376	T	C	nonsynonymous
270278	NTHI477_RS01410	317	G	A	nonsynonymous
270279	NTHI477_RS01410	316	C	A	nonsynonymous
270313	NTHI477_RS01410	282	A	G	synonymous

270442	NTHI477_RS01410	153	C	T	synonymous
270463	NTHI477_RS01410	132	T	C	synonymous
270466	NTHI477_RS01410	129	G	A	synonymous
270490	NTHI477_RS01410	105	T	C	synonymous
270499	NTHI477_RS01410	96	A	G	synonymous
270534	NTHI477_RS01410	61	G	A	synonymous
270580	NTHI477_RS01410	15	T	C	synonymous
270630	intergenic	intergenic	T	A	intergenic
270632	intergenic	intergenic	T	C	intergenic
270633	intergenic	intergenic	T	G	intergenic
270636	intergenic	intergenic	A	G	intergenic
270637	intergenic	intergenic	A	T	intergenic
270641	intergenic	intergenic	T	C	intergenic
270644	intergenic	intergenic	C	T	intergenic
270653	intergenic	intergenic	C	T	intergenic
270657	intergenic	intergenic	C	T	intergenic
270663	intergenic	intergenic	T	C	intergenic
270667	NTHI477_RS01415	4530	T	A	nonsynonymous
270679	NTHI477_RS01415	4518	G	T	nonsynonymous
270685	NTHI477_RS01415	4512	T	G	synonymous
270700	NTHI477_RS01415	4497	G	A	synonymous
270706	NTHI477_RS01415	4491	A	G	synonymous
270709	NTHI477_RS01415	4488	C	T	synonymous
270715	NTHI477_RS01415	4482	G	A	synonymous
270724	NTHI477_RS01415	4473	G	A	synonymous
270745	NTHI477_RS01415	4452	G	A	synonymous
270748	NTHI477_RS01415	4449	T	G	synonymous
270751	NTHI477_RS01415	4446	T	A	synonymous
270751	NTHI477_RS01415	4446	T	G	synonymous
270760	NTHI477_RS01415	4437	C	T	synonymous
270766	NTHI477_RS01415	4431	A	G	synonymous
270769	NTHI477_RS01415	4428	G	A	synonymous
270775	NTHI477_RS01415	4422	T	A	synonymous
270778	NTHI477_RS01415	4419	C	T	synonymous
270781	NTHI477_RS01415	4416	A	G	synonymous
270784	NTHI477_RS01415	4413	A	G	synonymous
270790	NTHI477_RS01415	4407	C	T	synonymous
270802	NTHI477_RS01415	4395	C	T	synonymous
270817	NTHI477_RS01415	4380	T	C	synonymous
270820	NTHI477_RS01415	4377	G	A	synonymous
270844	NTHI477_RS01415	4353	G	A	synonymous
270853	NTHI477_RS01415	4344	C	T	synonymous
270856	NTHI477_RS01415	4341	T	C	synonymous
270883	NTHI477_RS01415	4314	A	T	synonymous
270886	NTHI477_RS01415	4311	C	A	synonymous

270898	NTHI477_RS01415	4299	G	A	synonymous
270901	NTHI477_RS01415	4296	T	A	synonymous
270913	NTHI477_RS01415	4284	A	G	synonymous
270928	NTHI477_RS01415	4269	G	A	synonymous
270967	NTHI477_RS01415	4230	G	A	synonymous
270979	NTHI477_RS01415	4218	A	C	synonymous
270988	NTHI477_RS01415	4209	A	T	synonymous
270988	NTHI477_RS01415	4209	A	C	synonymous
270991	NTHI477_RS01415	4206	G	A	synonymous
270991	NTHI477_RS01415	4206	G	T	synonymous
271000	NTHI477_RS01415	4197	A	G	synonymous
271000	NTHI477_RS01415	4197	A	T	synonymous
271003	NTHI477_RS01415	4194	G	A	synonymous
271012	NTHI477_RS01415	4185	A	G	synonymous
271018	NTHI477_RS01415	4179	A	T	synonymous
271021	NTHI477_RS01415	4176	G	A	synonymous
271045	NTHI477_RS01415	4152	T	C	synonymous
271072	NTHI477_RS01415	4125	T	C	synonymous
271078	NTHI477_RS01415	4119	G	A	synonymous
271081	NTHI477_RS01415	4116	G	A	synonymous
271090	NTHI477_RS01415	4107	T	C	synonymous
271102	NTHI477_RS01415	4095	T	C	synonymous
271123	NTHI477_RS01415	4074	G	A	synonymous
271126	NTHI477_RS01415	4071	A	G	synonymous
271156	NTHI477_RS01415	4041	A	G	synonymous
271159	NTHI477_RS01415	4038	G	A	synonymous
271162	NTHI477_RS01415	4035	G	C	nonsynonymous
271167	NTHI477_RS01415	4030	A	G	nonsynonymous
271168	NTHI477_RS01415	4029	G	A	synonymous
271171	NTHI477_RS01415	4026	G	A	synonymous
271177	NTHI477_RS01415	4020	A	G	synonymous
271183	NTHI477_RS01415	4014	G	A	synonymous
271197	NTHI477_RS01415	4000	C	T	nonsynonymous
271205	NTHI477_RS01415	3992	C	G	nonsynonymous
271213	NTHI477_RS01415	3984	T	C	synonymous
271216	NTHI477_RS01415	3981	G	A	synonymous
271221	NTHI477_RS01415	3976	A	G	synonymous
271228	NTHI477_RS01415	3969	G	C	synonymous
271231	NTHI477_RS01415	3966	A	G	synonymous
271234	NTHI477_RS01415	3963	G	C	synonymous
271240	NTHI477_RS01415	3957	A	G	synonymous
271249	NTHI477_RS01415	3948	C	T	synonymous
271252	NTHI477_RS01415	3945	T	C	synonymous
271261	NTHI477_RS01415	3936	A	C	synonymous
271270	NTHI477_RS01415	3927	T	C	synonymous

271273	NTHI477_RS01415	3924	T	C	synonymous
271284	NTHI477_RS01415	3913	A	C	nonsynonymous
271285	NTHI477_RS01415	3912	G	A	synonymous
271294	NTHI477_RS01415	3903	C	T	synonymous
271297	NTHI477_RS01415	3900	A	G	synonymous
271303	NTHI477_RS01415	3894	A	G	synonymous
271306	NTHI477_RS01415	3891	G	A	synonymous
271318	NTHI477_RS01415	3879	A	G	synonymous
271321	NTHI477_RS01415	3876	A	G	synonymous
271339	NTHI477_RS01415	3858	C	G	synonymous
271346	NTHI477_RS01415	3851	C	T	nonsynonymous
271372	NTHI477_RS01415	3825	A	G	synonymous
271378	NTHI477_RS01415	3819	A	C	synonymous
271378	NTHI477_RS01415	3819	A	T	synonymous
271396	NTHI477_RS01415	3801	G	A	synonymous
271465	NTHI477_RS01415	3732	G	C	synonymous
271465	NTHI477_RS01415	3732	G	T	synonymous
271474	NTHI477_RS01415	3723	G	A	synonymous
271477	NTHI477_RS01415	3720	G	A	synonymous
271483	NTHI477_RS01415	3714	A	G	synonymous
271501	NTHI477_RS01415	3696	A	G	synonymous
271510	NTHI477_RS01415	3687	C	T	synonymous
271516	NTHI477_RS01415	3681	A	G	synonymous
271525	NTHI477_RS01415	3672	C	T	synonymous
271528	NTHI477_RS01415	3669	C	A	synonymous
271541	NTHI477_RS01415	3656	G	A	nonsynonymous
271546	NTHI477_RS01415	3651	A	G	synonymous
271563	NTHI477_RS01415	3634	C	T	nonsynonymous
271570	NTHI477_RS01415	3627	A	G	synonymous
271581	NTHI477_RS01415	3616	G	A	synonymous
271594	NTHI477_RS01415	3603	T	C	synonymous
271597	NTHI477_RS01415	3600	C	T	synonymous
271600	NTHI477_RS01415	3597	C	T	synonymous
271609	NTHI477_RS01415	3588	C	T	synonymous
271612	NTHI477_RS01415	3585	T	C	synonymous
271615	NTHI477_RS01415	3582	C	A	synonymous
271615	NTHI477_RS01415	3582	C	G	synonymous
271621	NTHI477_RS01415	3576	G	T	synonymous
271630	NTHI477_RS01415	3567	C	T	synonymous
271645	NTHI477_RS01415	3552	T	C	synonymous
271666	NTHI477_RS01415	3531	G	A	synonymous
271675	NTHI477_RS01415	3522	A	G	synonymous
271681	NTHI477_RS01415	3516	G	A	synonymous
271699	NTHI477_RS01415	3498	A	G	synonymous
271702	NTHI477_RS01415	3495	A	G	synonymous

271705	NTHI477_RS01415	3492	A	T	synonymous
271705	NTHI477_RS01415	3492	A	G	synonymous
271708	NTHI477_RS01415	3489	T	G	synonymous
271713	NTHI477_RS01415	3484	G	A	synonymous
271714	NTHI477_RS01415	3483	A	G	synonymous
271723	NTHI477_RS01415	3474	T	C	synonymous
271725	NTHI477_RS01415	3472	C	T	nonsynonymous
271742	NTHI477_RS01415	3455	G	T	nonsynonymous
271743	NTHI477_RS01415	3454	T	C	nonsynonymous
271747	NTHI477_RS01415	3450	C	T	synonymous
271750	NTHI477_RS01415	3447	C	A	synonymous
271768	NTHI477_RS01415	3429	C	T	synonymous
271771	NTHI477_RS01415	3426	A	G	synonymous
271777	NTHI477_RS01415	3420	G	A	synonymous
271783	NTHI477_RS01415	3414	A	G	synonymous
271789	NTHI477_RS01415	3408	G	T	synonymous
271789	NTHI477_RS01415	3408	G	A	synonymous
271792	NTHI477_RS01415	3405	G	A	synonymous
271795	NTHI477_RS01415	3402	T	A	synonymous
271800	NTHI477_RS01415	3397	G	T	nonsynonymous
271813	NTHI477_RS01415	3384	C	A	synonymous
271813	NTHI477_RS01415	3384	C	T	synonymous
271834	NTHI477_RS01415	3363	C	G	synonymous
271852	NTHI477_RS01415	3345	A	G	synonymous
271855	NTHI477_RS01415	3342	C	A	synonymous
271870	NTHI477_RS01415	3327	C	T	synonymous
271908	NTHI477_RS01415	3289	G	A	nonsynonymous
271909	NTHI477_RS01415	3288	C	T	synonymous
271912	NTHI477_RS01415	3285	G	A	synonymous
271921	NTHI477_RS01415	3276	A	G	synonymous
271923	NTHI477_RS01415	3274	C	T	nonsynonymous
271924	NTHI477_RS01415	3273	A	G	synonymous
271939	NTHI477_RS01415	3258	C	T	synonymous
271945	NTHI477_RS01415	3252	A	C	synonymous
271948	NTHI477_RS01415	3249	A	C	nonsynonymous
271957	NTHI477_RS01415	3240	C	T	synonymous
271962	NTHI477_RS01415	3235	C	T	nonsynonymous
271969	NTHI477_RS01415	3228	T	C	synonymous
271978	NTHI477_RS01415	3219	A	G	synonymous
271981	NTHI477_RS01415	3216	C	T	synonymous
272002	NTHI477_RS01415	3195	A	G	synonymous
272017	NTHI477_RS01415	3180	T	C	synonymous
272022	NTHI477_RS01415	3175	T	C	nonsynonymous
272023	NTHI477_RS01415	3174	G	T	synonymous
272024	NTHI477_RS01415	3173	C	T	nonsynonymous

272026	NTHI477_RS01415	3171	G	A	synonymous
272035	NTHI477_RS01415	3162	A	G	synonymous
272044	NTHI477_RS01415	3153	G	A	synonymous
272056	NTHI477_RS01415	3141	G	A	synonymous
272074	NTHI477_RS01415	3123	C	T	nonsynonymous
272075	NTHI477_RS01415	3122	A	G	nonsynonymous
272080	NTHI477_RS01415	3117	T	G	synonymous
272083	NTHI477_RS01415	3114	C	T	synonymous
272086	NTHI477_RS01415	3111	A	T	synonymous
272089	NTHI477_RS01415	3108	C	T	synonymous
272110	NTHI477_RS01415	3087	G	A	synonymous
272122	NTHI477_RS01415	3075	G	A	synonymous
272125	NTHI477_RS01415	3072	A	G	synonymous
272125	NTHI477_RS01415	3072	A	T	synonymous
272127	NTHI477_RS01415	3070	G	A	nonsynonymous
272140	NTHI477_RS01415	3057	A	T	synonymous
272142	NTHI477_RS01415	3055	C	T	nonsynonymous
272151	NTHI477_RS01415	3046	C	T	nonsynonymous
272171	NTHI477_RS01415	3026	G	T	nonsynonymous
272185	NTHI477_RS01415	3012	A	T	synonymous
272197	NTHI477_RS01415	3000	C	T	synonymous
272204	NTHI477_RS01415	2993	A	G	nonsynonymous
272212	NTHI477_RS01415	2985	T	C	synonymous
272215	NTHI477_RS01415	2982	A	T	synonymous
272230	NTHI477_RS01415	2967	C	T	synonymous
272235	NTHI477_RS01415	2962	T	C	nonsynonymous
272242	NTHI477_RS01415	2955	A	G	synonymous
272248	NTHI477_RS01415	2949	T	A	nonsynonymous
272252	NTHI477_RS01415	2945	T	G	nonsynonymous
272253	NTHI477_RS01415	2944	T	C	nonsynonymous
272262	NTHI477_RS01415	2935	C	A	nonsynonymous
272263	NTHI477_RS01415	2934	T	G	nonsynonymous
272263	NTHI477_RS01415	2934	T	C	synonymous
272266	NTHI477_RS01415	2931	G	A	synonymous
272269	NTHI477_RS01415	2928	A	G	synonymous
272278	NTHI477_RS01415	2919	A	G	synonymous
272296	NTHI477_RS01415	2901	G	T	synonymous
272299	NTHI477_RS01415	2898	A	T	synonymous
272302	NTHI477_RS01415	2895	C	A	synonymous
272305	NTHI477_RS01415	2892	C	A	nonsynonymous
272331	NTHI477_RS01415	2866	A	G	nonsynonymous
272332	NTHI477_RS01415	2865	C	T	synonymous
272374	NTHI477_RS01415	2823	C	T	synonymous
272377	NTHI477_RS01415	2820	G	A	synonymous
272415	NTHI477_RS01415	2782	T	C	nonsynonymous

272416	NTHI477_RS01415	2781	C	T	synonymous
272428	NTHI477_RS01415	2769	C	T	synonymous
272443	NTHI477_RS01415	2754	G	A	synonymous
272452	NTHI477_RS01415	2745	C	T	synonymous
272455	NTHI477_RS01415	2742	T	C	synonymous
272504	NTHI477_RS01415	2693	T	C	nonsynonymous
272509	NTHI477_RS01415	2688	G	A	synonymous
272515	NTHI477_RS01415	2682	A	G	synonymous
272544	NTHI477_RS01415	2653	C	T	nonsynonymous
272554	NTHI477_RS01415	2643	T	C	synonymous
272567	NTHI477_RS01415	2630	G	T	nonsynonymous
272590	NTHI477_RS01415	2607	A	C	synonymous
272590	NTHI477_RS01415	2607	A	T	synonymous
272592	NTHI477_RS01415	2605	G	A	nonsynonymous
272593	NTHI477_RS01415	2604	T	C	synonymous
272595	NTHI477_RS01415	2602	A	C	nonsynonymous
272596	NTHI477_RS01415	2601	C	T	synonymous
272598	NTHI477_RS01415	2599	A	G	synonymous
272602	NTHI477_RS01415	2595	C	T	synonymous
272604	NTHI477_RS01415	2593	A	G	synonymous
272631	NTHI477_RS01415	2566	G	A	nonsynonymous
272659	NTHI477_RS01415	2538	T	C	synonymous
272668	NTHI477_RS01415	2529	G	T	synonymous
272704	NTHI477_RS01415	2493	T	C	synonymous
272734	NTHI477_RS01415	2463	G	A	synonymous
272737	NTHI477_RS01415	2460	T	C	synonymous
272740	NTHI477_RS01415	2457	A	G	synonymous
272746	NTHI477_RS01415	2451	C	T	synonymous
272794	NTHI477_RS01415	2403	C	T	synonymous
272797	NTHI477_RS01415	2400	G	T	synonymous
272806	NTHI477_RS01415	2391	T	C	synonymous
272824	NTHI477_RS01415	2373	G	A	synonymous
272830	NTHI477_RS01415	2367	T	C	synonymous
272836	NTHI477_RS01415	2361	G	A	synonymous
272872	NTHI477_RS01415	2325	T	C	synonymous
272881	NTHI477_RS01415	2316	A	G	synonymous
272887	NTHI477_RS01415	2310	A	C	nonsynonymous
272899	NTHI477_RS01415	2298	C	G	synonymous
272917	NTHI477_RS01415	2280	T	A	synonymous
272917	NTHI477_RS01415	2280	T	G	synonymous
272950	NTHI477_RS01415	2247	T	C	synonymous
272951	NTHI477_RS01415	2246	G	A	nonsynonymous
272956	NTHI477_RS01415	2241	T	C	synonymous
272959	NTHI477_RS01415	2238	G	A	synonymous
272980	NTHI477_RS01415	2217	C	T	synonymous

272991	NTHI477_RS01415	2206	T	C	nonsynonymous
272992	NTHI477_RS01415	2205	G	T	synonymous
272998	NTHI477_RS01415	2199	G	A	synonymous
273021	NTHI477_RS01415	2176	G	A	synonymous
273022	NTHI477_RS01415	2175	T	C	synonymous
273025	NTHI477_RS01415	2172	A	C	synonymous
273025	NTHI477_RS01415	2172	A	T	synonymous
273029	NTHI477_RS01415	2168	C	T	nonsynonymous
273034	NTHI477_RS01415	2163	A	G	synonymous
273042	NTHI477_RS01415	2155	C	T	nonsynonymous
273045	NTHI477_RS01415	2152	G	A	synonymous
273055	NTHI477_RS01415	2142	A	G	synonymous
273055	NTHI477_RS01415	2142	A	T	synonymous
273058	NTHI477_RS01415	2139	C	T	synonymous
273100	NTHI477_RS01415	2097	C	T	synonymous
273109	NTHI477_RS01415	2088	T	A	nonsynonymous
273111	NTHI477_RS01415	2086	G	A	nonsynonymous
273139	NTHI477_RS01415	2058	T	C	synonymous
273142	NTHI477_RS01415	2055	C	T	nonsynonymous
273157	NTHI477_RS01415	2040	T	C	synonymous
273165	NTHI477_RS01415	2032	C	G	nonsynonymous
273166	NTHI477_RS01415	2031	A	C	synonymous
273184	NTHI477_RS01415	2013	G	A	synonymous
273210	NTHI477_RS01415	1987	A	G	nonsynonymous
273212	NTHI477_RS01415	1985	G	C	nonsynonymous
273229	NTHI477_RS01415	1968	C	T	synonymous
273234	NTHI477_RS01415	1963	A	G	synonymous
273241	NTHI477_RS01415	1956	T	C	synonymous
273254	NTHI477_RS01415	1943	G	A	nonsynonymous
273256	NTHI477_RS01415	1941	T	C	synonymous
273265	NTHI477_RS01415	1932	T	A	synonymous
273271	NTHI477_RS01415	1926	T	G	synonymous
273271	NTHI477_RS01415	1926	T	C	synonymous
273280	NTHI477_RS01415	1917	C	T	synonymous
273281	NTHI477_RS01415	1916	G	A	nonsynonymous
273337	NTHI477_RS01415	1860	G	A	synonymous
273340	NTHI477_RS01415	1857	T	C	synonymous
273359	NTHI477_RS01415	1838	C	T	nonsynonymous
273360	NTHI477_RS01415	1837	T	C	nonsynonymous
273361	NTHI477_RS01415	1836	T	A	synonymous
273363	NTHI477_RS01415	1834	C	T	nonsynonymous
273364	NTHI477_RS01415	1833	T	A	synonymous
273370	NTHI477_RS01415	1827	A	G	synonymous
273371	NTHI477_RS01415	1826	T	C	nonsynonymous
273379	NTHI477_RS01415	1818	C	T	synonymous

273382	NTHI477_RS01415	1815	C	T	synonymous
273391	NTHI477_RS01415	1806	C	T	synonymous
273394	NTHI477_RS01415	1803	T	G	synonymous
273421	NTHI477_RS01415	1776	C	T	synonymous
273441	NTHI477_RS01415	1756	C	T	nonsynonymous
273583	NTHI477_RS01415	1614	C	G	synonymous
273587	NTHI477_RS01415	1610	C	T	nonsynonymous
273595	NTHI477_RS01415	1602	C	G	synonymous
273598	NTHI477_RS01415	1599	G	C	nonsynonymous
273598	NTHI477_RS01415	1599	G	T	nonsynonymous
273599	NTHI477_RS01415	1598	T	G	nonsynonymous
273599	NTHI477_RS01415	1598	T	C	nonsynonymous
273600	NTHI477_RS01415	1597	T	C	nonsynonymous
273600	NTHI477_RS01415	1597	T	G	nonsynonymous
273600	NTHI477_RS01415	1597	T	A	nonsynonymous
273601	NTHI477_RS01415	1596	C	A	synonymous
273604	NTHI477_RS01415	1593	G	A	synonymous
273607	NTHI477_RS01415	1590	T	A	synonymous
273610	NTHI477_RS01415	1587	A	C	nonsynonymous
273610	NTHI477_RS01415	1587	A	T	synonymous
273616	NTHI477_RS01415	1581	G	C	synonymous
273616	NTHI477_RS01415	1581	G	A	synonymous
273619	NTHI477_RS01415	1578	G	C	synonymous
273628	NTHI477_RS01415	1569	G	A	synonymous
273634	NTHI477_RS01415	1563	T	C	synonymous
273640	NTHI477_RS01415	1557	G	A	synonymous
273643	NTHI477_RS01415	1554	C	G	synonymous
273667	NTHI477_RS01415	1530	C	T	synonymous
273668	NTHI477_RS01415	1529	G	A	nonsynonymous
273694	NTHI477_RS01415	1503	A	G	synonymous
273697	NTHI477_RS01415	1500	T	C	synonymous
273703	NTHI477_RS01415	1494	C	T	synonymous
273706	NTHI477_RS01415	1491	A	G	synonymous
273712	NTHI477_RS01415	1485	C	A	synonymous
273715	NTHI477_RS01415	1482	T	C	synonymous
273724	NTHI477_RS01415	1473	A	T	synonymous
273727	NTHI477_RS01415	1470	A	G	synonymous
273730	NTHI477_RS01415	1467	G	T	synonymous
273730	NTHI477_RS01415	1467	G	A	synonymous
273733	NTHI477_RS01415	1464	T	G	nonsynonymous
273739	NTHI477_RS01415	1458	T	C	synonymous
273744	NTHI477_RS01415	1453	C	T	nonsynonymous
273778	NTHI477_RS01415	1419	A	C	synonymous
273784	NTHI477_RS01415	1413	G	A	synonymous
273787	NTHI477_RS01415	1410	A	G	synonymous

273790	NTHI477_RS01415	1407	C	T	synonymous
273793	NTHI477_RS01415	1404	G	T	synonymous
273799	NTHI477_RS01415	1398	A	C	synonymous
273802	NTHI477_RS01415	1395	C	T	synonymous
273808	NTHI477_RS01415	1389	G	A	synonymous
273811	NTHI477_RS01415	1386	T	A	synonymous
273811	NTHI477_RS01415	1386	T	G	synonymous
273814	NTHI477_RS01415	1383	A	T	synonymous
273816	NTHI477_RS01415	1381	T	C	nonsynonymous
273820	NTHI477_RS01415	1377	G	A	synonymous
273838	NTHI477_RS01415	1359	T	C	synonymous
273841	NTHI477_RS01415	1356	G	T	synonymous
273874	NTHI477_RS01415	1323	A	G	synonymous
273877	NTHI477_RS01415	1320	T	A	synonymous
273886	NTHI477_RS01415	1311	C	G	synonymous
273888	NTHI477_RS01415	1309	C	A	nonsynonymous
273946	NTHI477_RS01415	1251	C	T	synonymous
273948	NTHI477_RS01415	1249	C	T	nonsynonymous
273970	NTHI477_RS01415	1227	T	C	nonsynonymous
273997	NTHI477_RS01415	1200	G	A	synonymous
274005	NTHI477_RS01415	1192	C	T	nonsynonymous
274021	NTHI477_RS01415	1176	G	A	synonymous
274022	NTHI477_RS01415	1175	C	T	nonsynonymous
274027	NTHI477_RS01415	1170	G	A	synonymous
274045	NTHI477_RS01415	1152	C	T	synonymous
274051	NTHI477_RS01415	1146	G	A	synonymous
274063	NTHI477_RS01415	1134	G	A	synonymous
274105	NTHI477_RS01415	1092	A	C	synonymous
274112	NTHI477_RS01415	1085	T	C	nonsynonymous
274120	NTHI477_RS01415	1077	C	T	synonymous
274126	NTHI477_RS01415	1071	A	T	synonymous
274129	NTHI477_RS01415	1068	C	T	synonymous
274130	NTHI477_RS01415	1067	G	A	nonsynonymous
274146	NTHI477_RS01415	1051	C	T	nonsynonymous
274149	NTHI477_RS01415	1048	C	T	nonsynonymous
274150	NTHI477_RS01415	1047	T	C	synonymous
274162	NTHI477_RS01415	1035	A	G	synonymous
274177	NTHI477_RS01415	1020	T	C	synonymous
274198	NTHI477_RS01415	999	G	A	synonymous
274218	NTHI477_RS01415	979	C	T	nonsynonymous
274243	NTHI477_RS01415	954	C	T	synonymous
274258	NTHI477_RS01415	939	A	G	synonymous
274273	NTHI477_RS01415	924	A	G	synonymous
274276	NTHI477_RS01415	921	A	G	synonymous
274300	NTHI477_RS01415	897	A	G	synonymous

274308	NTHI477_RS01415	889	T	C	nonsynonymous
274309	NTHI477_RS01415	888	G	A	synonymous
274324	NTHI477_RS01415	873	G	A	synonymous
274327	NTHI477_RS01415	870	C	T	synonymous
274336	NTHI477_RS01415	861	C	T	synonymous
274354	NTHI477_RS01415	843	C	T	synonymous
274359	NTHI477_RS01415	838	G	A	nonsynonymous
274360	NTHI477_RS01415	837	C	T	synonymous
274390	NTHI477_RS01415	807	G	A	synonymous
274392	NTHI477_RS01415	805	T	G	nonsynonymous
274393	NTHI477_RS01415	804	C	T	synonymous
274420	NTHI477_RS01415	777	G	A	synonymous
274432	NTHI477_RS01415	765	G	A	synonymous
274444	NTHI477_RS01415	753	A	G	synonymous
274456	NTHI477_RS01415	741	G	A	synonymous
274459	NTHI477_RS01415	738	G	A	synonymous
274468	NTHI477_RS01415	729	T	C	synonymous
274468	NTHI477_RS01415	729	T	G	synonymous
274519	NTHI477_RS01415	678	T	C	synonymous
274549	NTHI477_RS01415	648	G	A	synonymous
274561	NTHI477_RS01415	636	G	A	synonymous
274564	NTHI477_RS01415	633	G	A	synonymous
274582	NTHI477_RS01415	615	A	G	synonymous
274588	NTHI477_RS01415	609	T	C	synonymous
274594	NTHI477_RS01415	603	G	A	synonymous
274597	NTHI477_RS01415	600	A	G	synonymous
274615	NTHI477_RS01415	582	G	A	synonymous
274621	NTHI477_RS01415	576	G	A	synonymous
274630	NTHI477_RS01415	567	A	G	synonymous
274633	NTHI477_RS01415	564	G	T	synonymous
274636	NTHI477_RS01415	561	G	A	synonymous
274639	NTHI477_RS01415	558	A	T	synonymous
274652	NTHI477_RS01415	545	C	T	nonsynonymous
274653	NTHI477_RS01415	544	C	T	nonsynonymous
274657	NTHI477_RS01415	540	G	T	synonymous
274658	NTHI477_RS01415	539	G	A	nonsynonymous
274663	NTHI477_RS01415	534	T	A	synonymous
274666	NTHI477_RS01415	531	G	A	synonymous
274672	NTHI477_RS01415	525	T	C	synonymous
274676	NTHI477_RS01415	521	G	A	nonsynonymous
274678	NTHI477_RS01415	519	C	T	synonymous
274696	NTHI477_RS01415	501	T	G	synonymous
274696	NTHI477_RS01415	501	T	A	synonymous
274699	NTHI477_RS01415	498	A	G	synonymous
274711	NTHI477_RS01415	486	A	G	synonymous

274726	NTHI477_RS01415	471	A	C	synonymous
274729	NTHI477_RS01415	468	C	T	synonymous
274744	NTHI477_RS01415	453	C	T	synonymous
274744	NTHI477_RS01415	453	C	G	synonymous
274753	NTHI477_RS01415	444	C	T	synonymous
274768	NTHI477_RS01415	429	G	A	synonymous
274778	NTHI477_RS01415	419	C	T	nonsynonymous
274786	NTHI477_RS01415	411	A	G	synonymous
274798	NTHI477_RS01415	399	G	A	synonymous
274813	NTHI477_RS01415	384	G	A	synonymous
274822	NTHI477_RS01415	375	A	C	synonymous
274825	NTHI477_RS01415	372	T	C	synonymous
274855	NTHI477_RS01415	342	G	A	synonymous
274861	NTHI477_RS01415	336	A	G	synonymous
274870	NTHI477_RS01415	327	G	A	synonymous
274873	NTHI477_RS01415	324	G	A	synonymous
274876	NTHI477_RS01415	321	A	T	synonymous
274879	NTHI477_RS01415	318	C	T	synonymous
274882	NTHI477_RS01415	315	T	A	synonymous
274906	NTHI477_RS01415	291	G	A	synonymous
274909	NTHI477_RS01415	288	T	C	synonymous
274915	NTHI477_RS01415	282	G	A	synonymous
274927	NTHI477_RS01415	270	G	T	synonymous
274948	NTHI477_RS01415	249	G	A	synonymous
274957	NTHI477_RS01415	240	A	C	synonymous
274960	NTHI477_RS01415	237	T	C	synonymous
274969	NTHI477_RS01415	228	A	C	synonymous
274972	NTHI477_RS01415	225	A	C	synonymous
274981	NTHI477_RS01415	216	C	T	synonymous
274993	NTHI477_RS01415	204	T	C	synonymous
274996	NTHI477_RS01415	201	A	G	synonymous
275005	NTHI477_RS01415	192	G	A	synonymous
275014	NTHI477_RS01415	183	G	T	synonymous
275053	NTHI477_RS01415	144	G	A	synonymous
275066	NTHI477_RS01415	131	T	G	nonsynonymous
275070	NTHI477_RS01415	127	C	T	nonsynonymous
275072	NTHI477_RS01415	125	C	T	nonsynonymous
275077	NTHI477_RS01415	120	A	G	synonymous
275082	NTHI477_RS01415	115	T	C	nonsynonymous
275083	NTHI477_RS01415	114	C	A	synonymous
275098	NTHI477_RS01415	99	C	T	nonsynonymous
275103	NTHI477_RS01415	94	G	A	nonsynonymous
275107	NTHI477_RS01415	90	G	T	nonsynonymous
275107	NTHI477_RS01415	90	G	A	synonymous
275153	NTHI477_RS01415	44	C	T	nonsynonymous

275244	NTHI477_RS01420	687	T	C	synonymous
275246	NTHI477_RS01420	685	T	C	nonsynonymous
275250	NTHI477_RS01420	681	G	T	nonsynonymous
275258	NTHI477_RS01420	673	C	T	nonsynonymous
275265	NTHI477_RS01420	666	T	C	synonymous
275282	NTHI477_RS01420	649	A	C	nonsynonymous
275292	NTHI477_RS01420	639	A	T	synonymous
275301	NTHI477_RS01420	630	C	A	synonymous
275301	NTHI477_RS01420	630	C	T	synonymous
275312	NTHI477_RS01420	619	C	A	nonsynonymous
275319	NTHI477_RS01420	612	G	A	synonymous
275324	NTHI477_RS01420	607	T	G	synonymous
275331	NTHI477_RS01420	600	G	C	synonymous
275331	NTHI477_RS01420	600	G	T	synonymous
275333	NTHI477_RS01420	598	C	A	nonsynonymous
275334	NTHI477_RS01420	597	C	T	synonymous
275340	NTHI477_RS01420	591	T	C	synonymous
275358	NTHI477_RS01420	573	C	T	synonymous
275364	NTHI477_RS01420	567	C	T	synonymous
275367	NTHI477_RS01420	564	C	A	synonymous
275394	NTHI477_RS01420	537	A	C	synonymous
275418	NTHI477_RS01420	513	G	A	synonymous
275424	NTHI477_RS01420	507	G	A	synonymous
275441	NTHI477_RS01420	490	A	G	synonymous
275448	NTHI477_RS01420	483	T	C	synonymous
275463	NTHI477_RS01420	468	A	T	synonymous
275466	NTHI477_RS01420	465	T	C	synonymous
275469	NTHI477_RS01420	462	C	T	synonymous
275499	NTHI477_RS01420	432	G	A	synonymous
275550	NTHI477_RS01420	381	A	G	synonymous
275574	NTHI477_RS01420	357	C	T	synonymous
275586	NTHI477_RS01420	345	G	A	synonymous
275595	NTHI477_RS01420	336	A	G	synonymous
275598	NTHI477_RS01420	333	T	C	synonymous
275604	NTHI477_RS01420	327	G	A	synonymous
275643	NTHI477_RS01420	288	T	C	synonymous
275663	NTHI477_RS01420	268	A	G	synonymous
275682	NTHI477_RS01420	249	T	C	synonymous
275706	NTHI477_RS01420	225	G	A	synonymous
275709	NTHI477_RS01420	222	A	G	synonymous
275718	NTHI477_RS01420	213	G	A	synonymous
275766	NTHI477_RS01420	165	T	G	synonymous
275766	NTHI477_RS01420	165	T	A	synonymous
275772	NTHI477_RS01420	159	T	C	synonymous
275817	NTHI477_RS01420	114	C	T	synonymous

275835	NTHI477_RS01420	96	A	C	synonymous
275838	NTHI477_RS01420	93	C	T	synonymous
275849	NTHI477_RS01420	82	G	A	synonymous
275856	NTHI477_RS01420	75	T	C	synonymous
275922	NTHI477_RS01420	9	G	A	synonymous
275969	NTHI477_RS01425	1024	A	G	nonsynonymous
276010	NTHI477_RS01425	983	G	T	nonsynonymous
276060	NTHI477_RS01425	933	C	A	synonymous
276127	NTHI477_RS01425	866	A	G	nonsynonymous
276132	NTHI477_RS01425	861	C	T	synonymous
276171	NTHI477_RS01425	822	C	T	synonymous
276252	NTHI477_RS01425	741	T	G	nonsynonymous
276759	NTHI477_RS01425	234	A	G	synonymous
277093	NTHI477_RS01430	859	T	C	nonsynonymous
277514	NTHI477_RS01430	438	G	T	synonymous
277579	NTHI477_RS01430	373	A	T	nonsynonymous
277741	NTHI477_RS01430	211	A	C	nonsynonymous
277896	NTHI477_RS01430	56	C	A	nonsynonymous
277965	NTHI477_RS01435	1334	T	C	synonymous
277997	NTHI477_RS01435	1302	A	T	synonymous
278000	NTHI477_RS01435	1299	A	G	synonymous
278008	NTHI477_RS01435	1291	T	C	nonsynonymous
278039	NTHI477_RS01435	1260	C	G	synonymous
278054	NTHI477_RS01435	1245	C	T	synonymous
278066	NTHI477_RS01435	1233	G	A	synonymous
278069	NTHI477_RS01435	1230	T	C	synonymous
278092	NTHI477_RS01435	1207	G	T	synonymous
278109	NTHI477_RS01435	1190	C	T	nonsynonymous
278173	NTHI477_RS01435	1126	T	G	nonsynonymous
278236	NTHI477_RS01435	1063	G	A	nonsynonymous
278261	NTHI477_RS01435	1038	T	C	synonymous
278290	NTHI477_RS01435	1009	A	G	synonymous
278291	NTHI477_RS01435	1008	C	T	synonymous
278306	NTHI477_RS01435	993	G	A	synonymous
278309	NTHI477_RS01435	990	C	T	synonymous
278315	NTHI477_RS01435	984	A	G	synonymous
278324	NTHI477_RS01435	975	C	T	synonymous
278327	NTHI477_RS01435	972	G	A	synonymous
278333	NTHI477_RS01435	966	C	T	synonymous
278336	NTHI477_RS01435	963	C	G	synonymous
278336	NTHI477_RS01435	963	C	T	synonymous
278342	NTHI477_RS01435	957	C	T	synonymous
278357	NTHI477_RS01435	942	A	C	nonsynonymous
278396	NTHI477_RS01435	903	G	A	synonymous
278402	NTHI477_RS01435	897	G	A	synonymous

278426	NTHI477_RS01435	873	A	G	synonymous
278426	NTHI477_RS01435	873	A	T	synonymous
278429	NTHI477_RS01435	870	A	G	synonymous
278444	NTHI477_RS01435	855	C	T	synonymous
278453	NTHI477_RS01435	846	G	A	synonymous
278471	NTHI477_RS01435	828	A	G	synonymous
278474	NTHI477_RS01435	825	A	G	synonymous
278474	NTHI477_RS01435	825	A	T	synonymous
278486	NTHI477_RS01435	813	C	T	synonymous
278492	NTHI477_RS01435	807	G	A	synonymous
278495	NTHI477_RS01435	804	G	A	synonymous
278501	NTHI477_RS01435	798	A	T	synonymous
278525	NTHI477_RS01435	774	C	G	synonymous
278525	NTHI477_RS01435	774	C	T	synonymous
278531	NTHI477_RS01435	768	C	T	synonymous
278534	NTHI477_RS01435	765	C	T	synonymous
278540	NTHI477_RS01435	759	G	A	synonymous
278546	NTHI477_RS01435	753	G	A	synonymous
278552	NTHI477_RS01435	747	G	A	synonymous
278558	NTHI477_RS01435	741	A	T	synonymous
278561	NTHI477_RS01435	738	C	T	synonymous
278564	NTHI477_RS01435	735	A	G	synonymous
278585	NTHI477_RS01435	714	C	T	synonymous
278594	NTHI477_RS01435	705	C	T	synonymous
278597	NTHI477_RS01435	702	T	C	synonymous
278600	NTHI477_RS01435	699	A	C	synonymous
278602	NTHI477_RS01435	697	G	A	nonsynonymous
278609	NTHI477_RS01435	690	C	G	synonymous
278615	NTHI477_RS01435	684	C	T	synonymous
278615	NTHI477_RS01435	684	C	G	synonymous
278618	NTHI477_RS01435	681	A	G	synonymous
278623	NTHI477_RS01435	676	G	A	synonymous
278624	NTHI477_RS01435	675	C	T	synonymous
278633	NTHI477_RS01435	666	C	T	synonymous
278639	NTHI477_RS01435	660	G	A	synonymous
278642	NTHI477_RS01435	657	C	T	synonymous
278648	NTHI477_RS01435	651	C	A	synonymous
278651	NTHI477_RS01435	648	A	T	synonymous
278663	NTHI477_RS01435	636	T	C	synonymous
278681	NTHI477_RS01435	618	T	A	synonymous
278687	NTHI477_RS01435	612	T	C	synonymous
278728	NTHI477_RS01435	571	C	T	nonsynonymous
278744	NTHI477_RS01435	555	G	A	synonymous
278756	NTHI477_RS01435	543	A	G	synonymous
278759	NTHI477_RS01435	540	G	A	synonymous

278792	NTHI477_RS01435	507	T	C	synonymous
278813	NTHI477_RS01435	486	T	C	synonymous
278835	NTHI477_RS01435	464	A	T	nonsynonymous
278843	NTHI477_RS01435	456	G	A	synonymous
278849	NTHI477_RS01435	450	G	A	synonymous
278864	NTHI477_RS01435	435	C	T	synonymous
278870	NTHI477_RS01435	429	T	C	synonymous
278873	NTHI477_RS01435	426	T	A	synonymous
278882	NTHI477_RS01435	417	A	C	synonymous
278885	NTHI477_RS01435	414	G	A	synonymous
278915	NTHI477_RS01435	384	T	C	synonymous
278918	NTHI477_RS01435	381	T	C	synonymous
278921	NTHI477_RS01435	378	A	T	synonymous
278936	NTHI477_RS01435	363	G	A	synonymous
278947	NTHI477_RS01435	352	G	A	nonsynonymous
278969	NTHI477_RS01435	330	T	C	synonymous
279002	NTHI477_RS01435	297	C	T	synonymous
279005	NTHI477_RS01435	294	C	T	synonymous
279008	NTHI477_RS01435	291	G	A	synonymous
279011	NTHI477_RS01435	288	C	T	synonymous
279035	NTHI477_RS01435	264	G	A	synonymous
279040	NTHI477_RS01435	259	A	G	synonymous
279046	NTHI477_RS01435	253	G	A	nonsynonymous
279056	NTHI477_RS01435	243	T	C	synonymous
279062	NTHI477_RS01435	237	A	G	synonymous
279092	NTHI477_RS01435	207	G	A	synonymous
279098	NTHI477_RS01435	201	C	G	synonymous
279101	NTHI477_RS01435	198	G	A	synonymous
279128	NTHI477_RS01435	171	G	A	synonymous
279131	NTHI477_RS01435	168	G	A	synonymous
279140	NTHI477_RS01435	159	C	T	synonymous
279149	NTHI477_RS01435	150	G	C	synonymous
279149	NTHI477_RS01435	150	G	A	synonymous
279155	NTHI477_RS01435	144	T	C	synonymous
279157	NTHI477_RS01435	142	A	G	synonymous
279193	NTHI477_RS01435	106	C	A	nonsynonymous
279198	NTHI477_RS01435	101	G	A	nonsynonymous
279200	NTHI477_RS01435	99	C	T	synonymous
279203	NTHI477_RS01435	96	C	T	synonymous
279206	NTHI477_RS01435	93	G	A	synonymous
279218	NTHI477_RS01435	81	C	T	synonymous
279224	NTHI477_RS01435	75	A	G	synonymous
279247	NTHI477_RS01435	52	G	A	nonsynonymous
279333	intergenic	intergenic	G	A	intergenic
279356	intergenic	intergenic	A	G	intergenic

279357	intergenic	intergenic	C	T	intergenic
279360	intergenic	intergenic	A	T	intergenic
279435	intergenic	intergenic	C	T	intergenic
279446	intergenic	intergenic	G	A	intergenic
279453	NTHI477_RS01440	6	C	A	nonsynonymous
279480	NTHI477_RS01440	33	G	A	synonymous
279491	NTHI477_RS01440	44	G	C	nonsynonymous
279501	NTHI477_RS01440	54	C	T	synonymous
279519	NTHI477_RS01440	72	T	C	synonymous
279525	NTHI477_RS01440	78	C	T	synonymous
279528	NTHI477_RS01440	81	T	C	synonymous
279531	NTHI477_RS01440	84	C	T	synonymous
279537	NTHI477_RS01440	90	C	T	synonymous
279540	NTHI477_RS01440	93	C	T	synonymous
279543	NTHI477_RS01440	96	T	C	synonymous
279543	NTHI477_RS01440	96	T	G	synonymous
279546	NTHI477_RS01440	99	T	A	synonymous
279547	NTHI477_RS01440	100	T	G	nonsynonymous
279549	NTHI477_RS01440	102	G	A	synonymous
279558	NTHI477_RS01440	111	G	T	synonymous
279558	NTHI477_RS01440	111	G	A	synonymous
279564	NTHI477_RS01440	117	T	C	synonymous
279581	NTHI477_RS01440	134	T	G	nonsynonymous
279597	NTHI477_RS01440	150	T	C	synonymous
279603	NTHI477_RS01440	156	C	T	synonymous
279603	NTHI477_RS01440	156	C	A	synonymous
279624	NTHI477_RS01440	177	C	T	synonymous
279627	NTHI477_RS01440	180	T	C	synonymous
279633	NTHI477_RS01440	186	G	A	synonymous
279645	NTHI477_RS01440	198	G	A	synonymous
279651	NTHI477_RS01440	204	T	C	synonymous
279654	NTHI477_RS01440	207	G	A	synonymous
279657	NTHI477_RS01440	210	G	A	synonymous
279660	NTHI477_RS01440	213	T	A	synonymous
279667	NTHI477_RS01440	220	G	A	nonsynonymous
279672	NTHI477_RS01440	225	T	C	synonymous
279675	NTHI477_RS01440	228	T	G	synonymous
279678	NTHI477_RS01440	231	G	A	synonymous
279681	NTHI477_RS01440	234	G	A	synonymous
279684	NTHI477_RS01440	237	C	T	synonymous
279687	NTHI477_RS01440	240	T	C	synonymous
279702	NTHI477_RS01440	255	A	G	synonymous
279705	NTHI477_RS01440	258	G	T	synonymous
279708	NTHI477_RS01440	261	T	C	synonymous
279711	NTHI477_RS01440	264	A	T	synonymous

279714	NTHI477_RS01440	267	A	G	synonymous
279723	NTHI477_RS01440	276	G	A	synonymous
279735	NTHI477_RS01440	288	G	A	synonymous
279741	NTHI477_RS01440	294	T	C	synonymous
279745	NTHI477_RS01440	298	G	A	nonsynonymous
279747	NTHI477_RS01440	300	C	A	synonymous
279750	NTHI477_RS01440	303	A	C	synonymous
279762	NTHI477_RS01440	315	T	C	synonymous
279783	NTHI477_RS01440	336	C	A	synonymous
279783	NTHI477_RS01440	336	C	T	synonymous
279807	NTHI477_RS01440	360	G	A	synonymous
279816	NTHI477_RS01440	369	C	A	synonymous
279825	NTHI477_RS01440	378	G	A	synonymous
279843	NTHI477_RS01440	396	C	T	synonymous
279849	NTHI477_RS01440	402	A	G	synonymous
279858	NTHI477_RS01440	411	C	T	synonymous
279861	NTHI477_RS01440	414	T	C	synonymous
279864	NTHI477_RS01440	417	T	C	synonymous
279864	NTHI477_RS01440	417	T	A	synonymous
279876	NTHI477_RS01440	429	T	G	synonymous
279879	NTHI477_RS01440	432	T	G	synonymous
279900	NTHI477_RS01440	453	A	G	synonymous
279903	NTHI477_RS01440	456	C	T	synonymous
279915	NTHI477_RS01440	468	T	C	synonymous
279924	NTHI477_RS01440	477	G	A	synonymous
279927	NTHI477_RS01440	480	G	A	synonymous
279960	NTHI477_RS01440	513	G	A	nonsynonymous
279966	NTHI477_RS01440	519	A	C	synonymous
279966	NTHI477_RS01440	519	A	G	synonymous
279981	NTHI477_RS01440	534	A	G	synonymous
279990	NTHI477_RS01440	543	C	T	synonymous
279993	NTHI477_RS01440	546	T	C	synonymous
279996	NTHI477_RS01440	549	C	T	synonymous
280021	NTHI477_RS01440	574	A	G	nonsynonymous
280026	NTHI477_RS01440	579	C	T	synonymous
280041	NTHI477_RS01440	594	T	C	synonymous
280047	NTHI477_RS01440	600	G	A	synonymous
280060	NTHI477_RS01440	613	G	A	nonsynonymous
280065	NTHI477_RS01440	618	A	G	synonymous
280066	NTHI477_RS01440	619	G	A	nonsynonymous
280089	NTHI477_RS01440	642	T	C	synonymous
280110	NTHI477_RS01440	663	T	C	synonymous
280125	NTHI477_RS01440	678	T	G	synonymous
280125	NTHI477_RS01440	678	T	C	synonymous
280128	NTHI477_RS01440	681	A	G	synonymous

280140	NTHI477_RS01440	693	C	T	synonymous
280155	NTHI477_RS01440	708	C	T	synonymous
280161	NTHI477_RS01440	714	T	C	synonymous
280164	NTHI477_RS01440	717	T	A	synonymous
280179	NTHI477_RS01440	732	T	C	synonymous
280185	NTHI477_RS01440	738	C	T	synonymous
280209	NTHI477_RS01440	762	G	A	synonymous
280219	NTHI477_RS01440	772	A	T	nonsynonymous
280227	NTHI477_RS01440	780	A	G	synonymous
280248	NTHI477_RS01440	801	A	C	synonymous
280257	NTHI477_RS01440	810	C	T	synonymous
280260	NTHI477_RS01440	813	A	G	synonymous
280264	NTHI477_RS01440	817	A	C	nonsynonymous
280266	NTHI477_RS01440	819	G	A	nonsynonymous
280270	NTHI477_RS01440	823	A	G	nonsynonymous
280275	NTHI477_RS01440	828	G	C	synonymous
280290	NTHI477_RS01440	843	C	T	synonymous
280307	NTHI477_RS01440	860	A	G	nonsynonymous
280314	NTHI477_RS01440	867	G	T	synonymous
280329	NTHI477_RS01440	882	G	A	synonymous
280330	NTHI477_RS01440	883	G	A	nonsynonymous
280343	NTHI477_RS01440	896	C	T	nonsynonymous
280344	NTHI477_RS01440	897	T	C	synonymous
280345	NTHI477_RS01440	898	G	A	nonsynonymous
280359	NTHI477_RS01440	912	C	G	nonsynonymous
280383	NTHI477_RS01440	936	G	A	synonymous
280384	NTHI477_RS01440	937	A	C	nonsynonymous
280401	NTHI477_RS01445	11	T	C	nonsynonymous
280412	NTHI477_RS01445	22	G	C	nonsynonymous
280421	NTHI477_RS01445	31	A	G	nonsynonymous
280426	NTHI477_RS01445	36	C	T	synonymous
280432	NTHI477_RS01445	42	C	T	synonymous
280444	NTHI477_RS01445	54	T	C	synonymous
280445	NTHI477_RS01445	55	A	C	nonsynonymous
280462	NTHI477_RS01445	72	C	T	synonymous
280476	NTHI477_RS01445	86	G	A	nonsynonymous
280477	NTHI477_RS01445	87	C	A	synonymous
280478	NTHI477_RS01445	88	T	G	nonsynonymous
280486	NTHI477_RS01445	96	A	G	synonymous
280486	NTHI477_RS01445	96	A	T	synonymous
280488	NTHI477_RS01445	98	A	G	nonsynonymous
280490	NTHI477_RS01445	100	C	T	synonymous
280490	NTHI477_RS01445	100	C	G	nonsynonymous
280492	NTHI477_RS01445	102	G	A	synonymous
280497	NTHI477_RS01445	107	G	A	nonsynonymous

280499	NTHI477_RS01445	109	A	C	nonsynonymous
280501	NTHI477_RS01445	111	C	T	synonymous
280510	NTHI477_RS01445	120	A	T	synonymous
280511	NTHI477_RS01445	121	G	A	nonsynonymous
280516	NTHI477_RS01445	126	C	A	synonymous
280534	NTHI477_RS01445	144	C	T	synonymous
280540	NTHI477_RS01445	150	A	G	synonymous
280546	NTHI477_RS01445	156	C	T	synonymous
280549	NTHI477_RS01445	159	T	C	synonymous
280564	NTHI477_RS01445	174	C	T	synonymous
280573	NTHI477_RS01445	183	A	T	synonymous
280579	NTHI477_RS01445	189	C	T	synonymous
280582	NTHI477_RS01445	192	T	C	synonymous
280603	NTHI477_RS01445	213	T	G	synonymous
280609	NTHI477_RS01445	219	A	G	synonymous
280612	NTHI477_RS01445	222	G	T	synonymous
280615	NTHI477_RS01445	225	T	A	synonymous
280616	NTHI477_RS01445	226	G	T	nonsynonymous
280618	NTHI477_RS01445	228	A	G	synonymous
280621	NTHI477_RS01445	231	T	C	synonymous
280627	NTHI477_RS01445	237	T	C	synonymous
280630	NTHI477_RS01445	240	C	T	synonymous
280633	NTHI477_RS01445	243	T	C	synonymous
280636	NTHI477_RS01445	246	T	C	synonymous
280642	NTHI477_RS01445	252	G	A	synonymous
280645	NTHI477_RS01445	255	A	G	synonymous
280648	NTHI477_RS01445	258	C	T	synonymous
280666	NTHI477_RS01445	276	T	A	synonymous
280666	NTHI477_RS01445	276	T	G	synonymous
280678	NTHI477_RS01445	288	C	T	synonymous
280681	NTHI477_RS01445	291	A	C	synonymous
280693	NTHI477_RS01445	303	T	C	synonymous
280696	NTHI477_RS01445	306	G	A	synonymous
280737	intergenic	intergenic	C	T	intergenic
280738	intergenic	intergenic	C	T	intergenic
280739	intergenic	intergenic	A	G	intergenic
280739	intergenic	intergenic	A	T	intergenic
280740	intergenic	intergenic	A	G	intergenic
280741	intergenic	intergenic	A	G	intergenic
280743	intergenic	intergenic	A	G	intergenic
280744	intergenic	intergenic	T	G	intergenic
280753	intergenic	intergenic	A	T	intergenic
280753	intergenic	intergenic	A	G	intergenic
280758	intergenic	intergenic	T	G	intergenic
280758	intergenic	intergenic	T	A	intergenic

280766	intergenic	intergenic	C	T	intergenic
280770	intergenic	intergenic	T	C	intergenic
280780	intergenic	intergenic	C	T	intergenic
280783	intergenic	intergenic	T	C	intergenic
280793	intergenic	intergenic	G	C	intergenic
280794	intergenic	intergenic	A	C	intergenic
280801	intergenic	intergenic	T	C	intergenic
280805	intergenic	intergenic	A	G	intergenic
280830	intergenic	intergenic	A	G	intergenic
280836	intergenic	intergenic	G	A	intergenic
280853	NTHI477_RS01450	15	A	T	synonymous
280863	NTHI477_RS01450	25	T	C	synonymous
280868	NTHI477_RS01450	30	A	G	synonymous
280888	NTHI477_RS01450	50	A	G	nonsynonymous
280898	NTHI477_RS01450	60	A	G	synonymous
280907	NTHI477_RS01450	69	A	C	synonymous
280919	NTHI477_RS01450	81	T	C	synonymous
280940	NTHI477_RS01450	102	G	T	synonymous
280940	NTHI477_RS01450	102	G	A	synonymous
280949	NTHI477_RS01450	111	T	A	synonymous
280953	NTHI477_RS01450	115	G	T	nonsynonymous
280955	NTHI477_RS01450	117	A	G	synonymous
280964	NTHI477_RS01450	126	A	C	synonymous
280998	NTHI477_RS01450	160	G	T	nonsynonymous
281006	NTHI477_RS01450	168	C	T	synonymous
281009	NTHI477_RS01450	171	G	A	synonymous
281024	NTHI477_RS01450	186	C	T	synonymous
281039	NTHI477_RS01450	201	C	G	synonymous
281066	intergenic	intergenic	A	C	intergenic
281109	intergenic	intergenic	C	A	intergenic
281128	intergenic	intergenic	C	T	intergenic
281134	intergenic	intergenic	T	C	intergenic
281142	intergenic	intergenic	T	C	intergenic
281163	intergenic	intergenic	T	A	intergenic
281184	intergenic	intergenic	C	G	intergenic
281184	intergenic	intergenic	C	A	intergenic
281191	intergenic	intergenic	T	G	intergenic
281193	intergenic	intergenic	A	G	intergenic
281237	NTHI477_RS01455	27	G	A	synonymous
281243	NTHI477_RS01455	33	G	C	nonsynonymous
281247	NTHI477_RS01455	37	T	C	nonsynonymous
281257	NTHI477_RS01455	47	C	T	nonsynonymous
281268	NTHI477_RS01455	58	A	G	nonsynonymous
281277	NTHI477_RS01455	67	C	A	nonsynonymous
281283	NTHI477_RS01455	73	G	A	nonsynonymous

281284	NTHI477_RS01455	74	C	T	nonsynonymous
281288	NTHI477_RS01455	78	A	C	nonsynonymous
281318	NTHI477_RS01455	108	C	T	synonymous
281339	NTHI477_RS01455	129	T	C	synonymous
281340	NTHI477_RS01455	130	G	A	nonsynonymous
281345	NTHI477_RS01455	135	T	G	synonymous
281345	NTHI477_RS01455	135	T	A	synonymous
281366	NTHI477_RS01455	156	G	A	synonymous
281375	NTHI477_RS01455	165	C	T	synonymous
281384	NTHI477_RS01455	174	G	A	synonymous
281384	NTHI477_RS01455	174	G	C	synonymous
281384	NTHI477_RS01455	174	G	T	synonymous
281387	NTHI477_RS01455	177	C	T	synonymous
281396	NTHI477_RS01455	186	C	T	synonymous
281411	NTHI477_RS01455	201	G	A	synonymous
281414	NTHI477_RS01455	204	T	A	synonymous
281414	NTHI477_RS01455	204	T	C	synonymous
281423	NTHI477_RS01455	213	T	C	synonymous
281429	NTHI477_RS01455	219	T	C	synonymous
281441	NTHI477_RS01455	231	C	T	synonymous
281450	NTHI477_RS01455	240	T	C	synonymous
281477	NTHI477_RS01455	267	C	T	synonymous
281501	NTHI477_RS01455	291	T	A	synonymous
281510	NTHI477_RS01455	300	G	A	synonymous
281513	NTHI477_RS01455	303	T	C	synonymous
281534	NTHI477_RS01455	324	C	T	synonymous
281534	NTHI477_RS01455	324	C	A	synonymous
281537	NTHI477_RS01455	327	C	T	synonymous
281552	NTHI477_RS01455	342	C	A	synonymous
281552	NTHI477_RS01455	342	C	T	synonymous
281555	NTHI477_RS01455	345	C	T	synonymous
281558	NTHI477_RS01455	348	C	T	synonymous
281588	NTHI477_RS01455	378	T	A	nonsynonymous
281592	NTHI477_RS01455	382	G	A	nonsynonymous
281595	NTHI477_RS01455	385	A	G	nonsynonymous
281596	NTHI477_RS01455	386	T	C	nonsynonymous
281597	NTHI477_RS01455	387	T	A	synonymous
281602	NTHI477_RS01455	392	G	A	nonsynonymous
281604	NTHI477_RS01455	394	A	G	nonsynonymous
281605	NTHI477_RS01455	395	G	A	nonsynonymous
281606	NTHI477_RS01455	396	T	C	synonymous
281615	NTHI477_RS01455	405	C	T	synonymous
281619	NTHI477_RS01455	409	G	A	nonsynonymous
281619	NTHI477_RS01455	409	G	T	nonsynonymous
281633	NTHI477_RS01455	423	T	G	synonymous

281633	NTHI477_RS01455	423	T	A	synonymous
281636	NTHI477_RS01455	426	A	C	synonymous
281636	NTHI477_RS01455	426	A	T	synonymous
281672	NTHI477_RS01455	462	G	A	synonymous
281690	NTHI477_RS01455	480	G	A	synonymous
281711	NTHI477_RS01455	501	T	C	synonymous
281717	NTHI477_RS01455	507	T	G	synonymous
281720	NTHI477_RS01455	510	T	C	synonymous
281726	NTHI477_RS01455	516	G	T	synonymous
281729	NTHI477_RS01455	519	C	A	synonymous
281738	NTHI477_RS01455	528	T	A	synonymous
281741	NTHI477_RS01455	531	T	G	synonymous
281744	NTHI477_RS01455	534	C	A	synonymous
281744	NTHI477_RS01455	534	C	T	synonymous
281750	NTHI477_RS01455	540	T	C	synonymous
281753	NTHI477_RS01455	543	T	C	synonymous
281759	NTHI477_RS01455	549	G	A	synonymous
281771	NTHI477_RS01455	561	T	C	synonymous
281775	NTHI477_RS01455	565	A	G	nonsynonymous
281837	NTHI477_RS01455	627	C	A	synonymous
281873	NTHI477_RS01455	663	T	C	synonymous
281873	NTHI477_RS01455	663	T	A	synonymous
281877	NTHI477_RS01455	667	A	G	nonsynonymous
281877	NTHI477_RS01455	667	A	C	nonsynonymous
281878	NTHI477_RS01455	668	A	T	nonsynonymous
281880	NTHI477_RS01455	670	T	G	nonsynonymous
281881	NTHI477_RS01455	671	C	A	nonsynonymous
281882	NTHI477_RS01455	672	T	C	synonymous
281885	NTHI477_RS01455	675	T	A	synonymous
281886	NTHI477_RS01455	676	A	G	nonsynonymous
281887	NTHI477_RS01455	677	C	T	nonsynonymous
281891	NTHI477_RS01455	681	G	A	synonymous
281894	NTHI477_RS01455	684	A	G	synonymous
281909	NTHI477_RS01455	699	C	T	synonymous
281910	NTHI477_RS01455	700	A	G	nonsynonymous
281945	NTHI477_RS01455	735	G	A	synonymous
281972	NTHI477_RS01455	762	A	T	nonsynonymous
281981	NTHI477_RS01455	771	C	T	synonymous
281984	NTHI477_RS01455	774	T	C	synonymous
281993	NTHI477_RS01455	783	A	G	synonymous
281996	NTHI477_RS01455	786	T	C	synonymous
282014	NTHI477_RS01455	804	A	G	synonymous
282027	NTHI477_RS01455	817	C	A	nonsynonymous
282027	NTHI477_RS01455	817	C	G	nonsynonymous
282030	NTHI477_RS01455	820	A	G	nonsynonymous

282032	NTHI477_RS01455	822	T	G	nonsynonymous
282032	NTHI477_RS01455	822	T	C	synonymous
282033	NTHI477_RS01455	823	G	T	nonsynonymous
282033	NTHI477_RS01455	823	G	A	nonsynonymous
282034	NTHI477_RS01455	824	C	G	nonsynonymous
282037	NTHI477_RS01455	827	A	G	nonsynonymous
282038	NTHI477_RS01455	828	T	C	synonymous
282039	NTHI477_RS01455	829	A	C	nonsynonymous
282047	NTHI477_RS01455	837	A	G	synonymous
282050	NTHI477_RS01455	840	G	A	synonymous
282051	NTHI477_RS01455	841	T	C	synonymous
282056	NTHI477_RS01455	846	C	T	synonymous
282059	NTHI477_RS01455	849	A	G	synonymous
282080	NTHI477_RS01455	870	T	C	synonymous
282086	NTHI477_RS01455	876	C	T	synonymous
282087	NTHI477_RS01455	877	A	C	nonsynonymous
282099	NTHI477_RS01455	889	C	T	nonsynonymous
282101	NTHI477_RS01455	891	T	C	synonymous
282128	NTHI477_RS01455	918	T	A	synonymous
282135	NTHI477_RS01455	925	T	C	nonsynonymous
282137	NTHI477_RS01455	927	T	C	synonymous
282138	NTHI477_RS01455	928	G	A	nonsynonymous
282161	NTHI477_RS01455	951	G	A	synonymous
282165	NTHI477_RS01455	955	T	C	synonymous
282176	NTHI477_RS01455	966	C	T	synonymous
282177	NTHI477_RS01455	967	G	A	nonsynonymous
282178	NTHI477_RS01455	968	A	G	nonsynonymous
282189	NTHI477_RS01455	979	A	G	nonsynonymous
282190	NTHI477_RS01455	980	A	G	nonsynonymous
282197	NTHI477_RS01455	987	C	T	synonymous
282224	NTHI477_RS01455	1014	G	A	synonymous
282225	NTHI477_RS01455	1015	A	G	nonsynonymous
282225	NTHI477_RS01455	1015	A	T	nonsynonymous
282230	NTHI477_RS01455	1020	A	G	synonymous
282233	NTHI477_RS01455	1023	A	T	synonymous
282241	NTHI477_RS01455	1031	T	C	nonsynonymous
282242	NTHI477_RS01455	1032	G	A	synonymous
282251	NTHI477_RS01455	1041	T	C	synonymous
282252	NTHI477_RS01455	1042	G	A	nonsynonymous
282263	NTHI477_RS01455	1053	T	C	synonymous
282275	NTHI477_RS01455	1065	C	T	synonymous
282278	NTHI477_RS01455	1068	A	G	synonymous
282294	NTHI477_RS01455	1084	A	C	nonsynonymous
282296	NTHI477_RS01455	1086	G	A	synonymous
282312	NTHI477_RS01455	1102	T	G	nonsynonymous

282313	NTHI477_RS01455	1103	C	T	nonsynonymous
282314	NTHI477_RS01455	1104	C	T	synonymous
282317	NTHI477_RS01455	1107	G	A	synonymous
282318	NTHI477_RS01455	1108	A	G	nonsynonymous
282319	NTHI477_RS01455	1109	A	C	nonsynonymous
282320	NTHI477_RS01455	1110	T	A	nonsynonymous
282321	NTHI477_RS01455	1111	A	G	nonsynonymous
282322	NTHI477_RS01455	1112	A	G	nonsynonymous
282324	NTHI477_RS01455	1114	A	G	nonsynonymous
282327	NTHI477_RS01455	1117	G	T	nonsynonymous
282328	NTHI477_RS01455	1118	A	C	nonsynonymous
282329	NTHI477_RS01455	1119	A	T	nonsynonymous
282330	NTHI477_RS01455	1120	A	T	nonsynonymous
282334	NTHI477_RS01455	1124	C	T	nonsynonymous
282335	NTHI477_RS01455	1125	A	G	synonymous
282337	NTHI477_RS01455	1127	C	A	nonsynonymous
282338	NTHI477_RS01455	1128	T	G	synonymous
282338	NTHI477_RS01455	1128	T	A	synonymous
282343	NTHI477_RS01455	1133	C	T	nonsynonymous
282344	NTHI477_RS01455	1134	A	G	synonymous
282345	NTHI477_RS01455	1135	A	G	nonsynonymous
282348	NTHI477_RS01455	1138	G	C	nonsynonymous
282349	NTHI477_RS01455	1139	A	C	nonsynonymous
282350	NTHI477_RS01455	1140	A	C	nonsynonymous
282351	NTHI477_RS01455	1141	G	T	nonsynonymous
282351	NTHI477_RS01455	1141	G	A	nonsynonymous
282354	NTHI477_RS01455	1144	T	G	nonsynonymous
282354	NTHI477_RS01455	1144	T	A	nonsynonymous
282355	NTHI477_RS01455	1145	A	G	nonsynonymous
282356	NTHI477_RS01455	1146	T	G	nonsynonymous
282358	NTHI477_RS01455	1148	A	G	nonsynonymous
282359	NTHI477_RS01455	1149	A	G	synonymous
282359	NTHI477_RS01455	1149	A	T	nonsynonymous
282360	NTHI477_RS01455	1150	C	T	nonsynonymous
282361	NTHI477_RS01455	1151	C	T	nonsynonymous
282362	NTHI477_RS01455	1152	A	G	synonymous
282362	NTHI477_RS01455	1152	A	T	synonymous
282363	NTHI477_RS01455	1153	C	T	synonymous
282364	NTHI477_RS01455	1154	T	C	nonsynonymous
282365	NTHI477_RS01455	1155	G	T	synonymous
282365	NTHI477_RS01455	1155	G	A	synonymous
282368	NTHI477_RS01455	1158	T	C	synonymous
282369	NTHI477_RS01455	1159	A	G	nonsynonymous
282371	NTHI477_RS01455	1161	T	C	synonymous
282373	NTHI477_RS01455	1163	T	A	nonsynonymous

282374	NTHI477_RS01455	1164	C	T	synonymous
282383	NTHI477_RS01455	1173	C	T	synonymous
282386	NTHI477_RS01455	1176	G	A	synonymous
282392	NTHI477_RS01455	1182	T	C	synonymous
282395	NTHI477_RS01455	1185	T	C	synonymous
282398	NTHI477_RS01455	1188	C	T	synonymous
282400	NTHI477_RS01455	1190	G	A	nonsynonymous
282401	NTHI477_RS01455	1191	T	A	synonymous
282405	NTHI477_RS01455	1195	A	G	nonsynonymous
282406	NTHI477_RS01455	1196	C	T	nonsynonymous
282407	NTHI477_RS01455	1197	C	T	synonymous
282410	NTHI477_RS01455	1200	A	G	synonymous
282419	NTHI477_RS01455	1209	G	A	synonymous
282428	NTHI477_RS01455	1218	A	T	synonymous
282439	NTHI477_RS01455	1229	T	C	nonsynonymous
282440	NTHI477_RS01455	1230	A	G	synonymous
282446	NTHI477_RS01455	1236	T	A	nonsynonymous
282453	NTHI477_RS01455	1243	T	A	nonsynonymous
282460	NTHI477_RS01455	1250	C	G	nonsynonymous
282464	NTHI477_RS01455	1254	T	C	synonymous
282467	NTHI477_RS01455	1257	G	C	synonymous
282470	NTHI477_RS01455	1260	G	A	synonymous
282488	NTHI477_RS01455	1278	C	A	synonymous
282488	NTHI477_RS01455	1278	C	T	synonymous
282489	NTHI477_RS01455	1279	G	A	nonsynonymous
282491	NTHI477_RS01455	1281	A	C	synonymous
282506	NTHI477_RS01455	1296	T	A	synonymous
282524	NTHI477_RS01455	1314	T	C	synonymous
282530	NTHI477_RS01455	1320	A	G	synonymous
282539	NTHI477_RS01455	1329	C	T	synonymous
282540	NTHI477_RS01455	1330	C	T	nonsynonymous
282541	NTHI477_RS01455	1331	A	C	nonsynonymous
282547	NTHI477_RS01455	1337	T	C	nonsynonymous
282548	NTHI477_RS01455	1338	A	G	synonymous
282549	NTHI477_RS01455	1339	A	G	nonsynonymous
282550	NTHI477_RS01455	1340	C	T	nonsynonymous
282551	NTHI477_RS01455	1341	C	A	synonymous
282551	NTHI477_RS01455	1341	C	T	synonymous
282552	NTHI477_RS01455	1342	C	T	synonymous
282553	NTHI477_RS01455	1343	T	A	nonsynonymous
282554	NTHI477_RS01455	1344	A	C	synonymous
282555	NTHI477_RS01455	1345	A	T	nonsynonymous
282555	NTHI477_RS01455	1345	A	G	nonsynonymous
282556	NTHI477_RS01455	1346	T	C	nonsynonymous
282557	NTHI477_RS01455	1347	T	G	nonsynonymous

282558	NTHI477_RS01455	1348	A	C	nonsynonymous
282560	NTHI477_RS01455	1350	G	A	nonsynonymous
282560	NTHI477_RS01455	1350	G	C	nonsynonymous
282560	NTHI477_RS01455	1350	G	T	nonsynonymous
282561	NTHI477_RS01455	1351	C	T	nonsynonymous
282563	NTHI477_RS01455	1353	T	A	synonymous
282564	NTHI477_RS01455	1354	G	T	nonsynonymous
282564	NTHI477_RS01455	1354	G	A	nonsynonymous
282565	NTHI477_RS01455	1355	A	G	nonsynonymous
282566	NTHI477_RS01455	1356	T	A	nonsynonymous
282567	NTHI477_RS01455	1357	A	G	nonsynonymous
282568	NTHI477_RS01455	1358	A	C	nonsynonymous
282570	NTHI477_RS01455	1360	G	A	nonsynonymous
282572	NTHI477_RS01455	1362	C	T	synonymous
282573	NTHI477_RS01455	1363	G	A	nonsynonymous
282575	NTHI477_RS01455	1365	A	G	synonymous
282578	NTHI477_RS01455	1368	C	G	nonsynonymous
282578	NTHI477_RS01455	1368	C	T	synonymous
282580	NTHI477_RS01455	1370	C	G	nonsynonymous
282580	NTHI477_RS01455	1370	C	T	nonsynonymous
282581	NTHI477_RS01455	1371	T	A	synonymous
282583	NTHI477_RS01455	1373	T	C	nonsynonymous
282585	NTHI477_RS01455	1375	G	A	nonsynonymous
282587	NTHI477_RS01455	1377	A	T	nonsynonymous
282588	NTHI477_RS01455	1378	A	C	nonsynonymous
282588	NTHI477_RS01455	1378	A	T	nonsynonymous
282589	NTHI477_RS01455	1379	C	T	nonsynonymous
282590	NTHI477_RS01455	1380	A	T	synonymous
282590	NTHI477_RS01455	1380	A	G	synonymous
282591	NTHI477_RS01455	1381	C	T	nonsynonymous
282591	NTHI477_RS01455	1381	C	A	nonsynonymous
282592	NTHI477_RS01455	1382	C	A	nonsynonymous
282592	NTHI477_RS01455	1382	C	T	nonsynonymous
282593	NTHI477_RS01455	1383	A	C	synonymous
282593	NTHI477_RS01455	1383	A	T	synonymous
282594	NTHI477_RS01455	1384	T	C	synonymous
282594	NTHI477_RS01455	1384	T	A	nonsynonymous
282594	NTHI477_RS01455	1384	T	G	nonsynonymous
282595	NTHI477_RS01455	1385	T	A	nonsynonymous
282596	NTHI477_RS01455	1386	A	C	nonsynonymous
282596	NTHI477_RS01455	1386	A	T	nonsynonymous
282596	NTHI477_RS01455	1386	A	G	synonymous
282597	NTHI477_RS01455	1387	T	A	nonsynonymous
282598	NTHI477_RS01455	1388	C	T	nonsynonymous
282599	NTHI477_RS01455	1389	A	T	synonymous

282599	NTHI477_RS01455	1389	A	G	synonymous
282600	NTHI477_RS01455	1390	A	G	nonsynonymous
282602	NTHI477_RS01455	1392	T	A	nonsynonymous
282605	NTHI477_RS01455	1395	C	T	synonymous
282606	NTHI477_RS01455	1396	A	T	nonsynonymous
282607	NTHI477_RS01455	1397	C	A	nonsynonymous
282608	NTHI477_RS01455	1398	A	T	synonymous
282623	NTHI477_RS01455	1413	C	T	synonymous
282623	NTHI477_RS01455	1413	C	A	synonymous
282626	NTHI477_RS01455	1416	T	A	synonymous
282634	NTHI477_RS01455	1424	C	G	nonsynonymous
282635	NTHI477_RS01455	1425	C	T	synonymous
282641	NTHI477_RS01455	1431	A	G	synonymous
282649	NTHI477_RS01455	1439	C	G	nonsynonymous
282649	NTHI477_RS01455	1439	C	T	nonsynonymous
282650	NTHI477_RS01455	1440	T	G	synonymous
282651	NTHI477_RS01455	1441	A	G	nonsynonymous
282652	NTHI477_RS01455	1442	T	C	nonsynonymous
282656	NTHI477_RS01455	1446	T	C	synonymous
282663	NTHI477_RS01455	1453	G	A	nonsynonymous
282666	NTHI477_RS01455	1456	C	T	synonymous
282668	NTHI477_RS01455	1458	A	G	synonymous
282671	NTHI477_RS01455	1461	A	T	synonymous
282678	NTHI477_RS01455	1468	A	C	nonsynonymous
282679	NTHI477_RS01455	1469	A	G	nonsynonymous
282689	NTHI477_RS01455	1479	A	G	synonymous
282692	NTHI477_RS01455	1482	G	T	nonsynonymous
282695	NTHI477_RS01455	1485	T	A	synonymous
282698	NTHI477_RS01455	1488	T	A	synonymous
282701	NTHI477_RS01455	1491	T	C	synonymous
282707	NTHI477_RS01455	1497	T	C	synonymous
282734	NTHI477_RS01455	1524	C	T	synonymous
282743	NTHI477_RS01455	1533	T	C	synonymous
282746	NTHI477_RS01455	1536	C	T	synonymous
282752	NTHI477_RS01455	1542	C	T	synonymous
282767	NTHI477_RS01455	1557	T	C	synonymous
282769	NTHI477_RS01455	1559	A	G	nonsynonymous
282770	NTHI477_RS01455	1560	C	T	synonymous
282770	NTHI477_RS01455	1560	C	A	nonsynonymous
282779	NTHI477_RS01455	1569	C	T	synonymous
282780	NTHI477_RS01455	1570	G	A	nonsynonymous
282782	NTHI477_RS01455	1572	G	T	nonsynonymous
282795	NTHI477_RS01455	1585	C	T	synonymous
282797	NTHI477_RS01455	1587	A	G	synonymous
282815	NTHI477_RS01455	1605	C	T	synonymous

282833	NTHI477_RS01455	1623	G	T	synonymous
282845	NTHI477_RS01455	1635	G	C	synonymous
282849	NTHI477_RS01455	1639	G	A	nonsynonymous
282851	NTHI477_RS01455	1641	T	A	synonymous
282854	NTHI477_RS01455	1644	T	C	synonymous
282861	NTHI477_RS01455	1651	C	A	nonsynonymous
282863	NTHI477_RS01455	1653	C	T	synonymous
282867	NTHI477_RS01455	1657	A	G	nonsynonymous
282873	NTHI477_RS01455	1663	T	C	nonsynonymous
282874	NTHI477_RS01455	1664	C	A	nonsynonymous
282876	NTHI477_RS01455	1666	A	G	nonsynonymous
282881	NTHI477_RS01455	1671	T	C	synonymous
282890	NTHI477_RS01455	1680	A	G	synonymous
282893	NTHI477_RS01455	1683	T	C	synonymous
282902	NTHI477_RS01455	1692	C	T	synonymous
282905	NTHI477_RS01455	1695	A	T	synonymous
282910	NTHI477_RS01455	1700	G	C	nonsynonymous
282911	NTHI477_RS01455	1701	C	G	nonsynonymous
282911	NTHI477_RS01455	1701	C	A	nonsynonymous
282932	NTHI477_RS01455	1722	T	C	synonymous
282944	NTHI477_RS01455	1734	T	C	synonymous
282947	NTHI477_RS01455	1737	A	G	synonymous
282954	NTHI477_RS01455	1744	G	A	nonsynonymous
282962	NTHI477_RS01455	1752	C	G	nonsynonymous
282962	NTHI477_RS01455	1752	C	A	nonsynonymous
282968	NTHI477_RS01455	1758	A	T	synonymous
282968	NTHI477_RS01455	1758	A	C	synonymous
282968	NTHI477_RS01455	1758	A	G	synonymous
282974	NTHI477_RS01455	1764	T	C	synonymous
282998	NTHI477_RS01455	1788	G	A	synonymous
283023	NTHI477_RS01455	1813	T	C	nonsynonymous
283023	NTHI477_RS01455	1813	T	A	nonsynonymous
283024	NTHI477_RS01455	1814	C	T	nonsynonymous
283024	NTHI477_RS01455	1814	C	G	nonsynonymous
283026	NTHI477_RS01455	1816	A	G	nonsynonymous
283027	NTHI477_RS01455	1817	T	A	nonsynonymous
283029	NTHI477_RS01455	1819	C	G	nonsynonymous
283029	NTHI477_RS01455	1819	C	A	nonsynonymous
283030	NTHI477_RS01455	1820	C	G	nonsynonymous
283030	NTHI477_RS01455	1820	C	A	nonsynonymous
283030	NTHI477_RS01455	1820	C	T	nonsynonymous
283031	NTHI477_RS01455	1821	G	A	synonymous
283032	NTHI477_RS01455	1822	G	A	nonsynonymous
283033	NTHI477_RS01455	1823	G	A	nonsynonymous
283034	NTHI477_RS01455	1824	T	A	synonymous

283034	NTHI477_RS01455	1824	T	G	synonymous
283035	NTHI477_RS01455	1825	G	A	nonsynonymous
283035	NTHI477_RS01455	1825	G	T	nonsynonymous
283039	NTHI477_RS01455	1829	G	A	nonsynonymous
283045	NTHI477_RS01455	1835	C	G	nonsynonymous
283055	NTHI477_RS01455	1845	C	T	synonymous
283061	NTHI477_RS01455	1851	A	G	synonymous
283067	NTHI477_RS01455	1857	G	A	synonymous
283070	NTHI477_RS01455	1860	T	C	synonymous
283094	NTHI477_RS01455	1884	A	G	synonymous
283097	NTHI477_RS01455	1887	C	T	synonymous
283100	NTHI477_RS01455	1890	T	C	synonymous
283106	NTHI477_RS01455	1896	T	A	synonymous
283112	NTHI477_RS01455	1902	C	T	synonymous
283121	NTHI477_RS01455	1911	A	G	synonymous
283139	NTHI477_RS01455	1929	T	A	synonymous
283140	NTHI477_RS01455	1930	A	G	nonsynonymous
283141	NTHI477_RS01455	1931	C	T	nonsynonymous
283142	NTHI477_RS01455	1932	G	A	synonymous
283148	NTHI477_RS01455	1938	T	C	synonymous
283161	NTHI477_RS01455	1951	T	C	synonymous
283178	NTHI477_RS01455	1968	T	C	synonymous
283178	NTHI477_RS01455	1968	T	A	synonymous
283184	NTHI477_RS01455	1974	T	C	synonymous
283185	NTHI477_RS01455	1975	G	A	nonsynonymous
283187	NTHI477_RS01455	1977	A	T	nonsynonymous
283194	NTHI477_RS01455	1984	G	A	nonsynonymous
283198	NTHI477_RS01455	1988	T	C	nonsynonymous
283199	NTHI477_RS01455	1989	G	A	synonymous
283203	NTHI477_RS01455	1993	A	G	nonsynonymous
283207	NTHI477_RS01455	1997	T	C	nonsynonymous
283207	NTHI477_RS01455	1997	T	G	nonsynonymous
283208	NTHI477_RS01455	1998	T	C	synonymous
283211	NTHI477_RS01455	2001	T	C	synonymous
283213	NTHI477_RS01455	2003	C	T	nonsynonymous
283213	NTHI477_RS01455	2003	C	A	nonsynonymous
283218	NTHI477_RS01455	2008	A	G	nonsynonymous
283220	NTHI477_RS01455	2010	T	C	synonymous
283223	NTHI477_RS01455	2013	T	A	synonymous
283236	NTHI477_RS01455	2026	T	C	nonsynonymous
283237	NTHI477_RS01455	2027	A	G	nonsynonymous
283239	NTHI477_RS01455	2029	C	A	nonsynonymous
283242	NTHI477_RS01455	2032	G	C	nonsynonymous
283248	NTHI477_RS01455	2038	T	C	synonymous
283252	NTHI477_RS01455	2042	G	A	nonsynonymous

283265	NTHI477_RS01455	2055	T	C	synonymous
283267	NTHI477_RS01455	2057	A	G	nonsynonymous
283268	NTHI477_RS01455	2058	C	T	synonymous
283270	NTHI477_RS01455	2060	C	G	nonsynonymous
283271	NTHI477_RS01455	2061	T	C	synonymous
283271	NTHI477_RS01455	2061	T	G	synonymous
283271	NTHI477_RS01455	2061	T	A	synonymous
283290	NTHI477_RS01455	2080	T	C	synonymous
283305	NTHI477_RS01455	2095	A	G	nonsynonymous
283306	NTHI477_RS01455	2096	C	A	nonsynonymous
283310	NTHI477_RS01455	2100	A	G	nonsynonymous
283320	NTHI477_RS01455	2110	G	A	nonsynonymous
283325	NTHI477_RS01455	2115	T	C	synonymous
283334	NTHI477_RS01455	2124	T	A	synonymous
283337	NTHI477_RS01455	2127	C	T	synonymous
283355	NTHI477_RS01455	2145	C	A	synonymous
283363	NTHI477_RS01455	2153	G	A	nonsynonymous
283365	NTHI477_RS01455	2155	C	A	nonsynonymous
283366	NTHI477_RS01455	2156	G	A	nonsynonymous
283367	NTHI477_RS01455	2157	T	C	synonymous
283373	NTHI477_RS01455	2163	T	G	nonsynonymous
283373	NTHI477_RS01455	2163	T	C	synonymous
283374	NTHI477_RS01455	2164	T	C	synonymous
283374	NTHI477_RS01455	2164	T	G	nonsynonymous
283375	NTHI477_RS01455	2165	T	C	nonsynonymous
283375	NTHI477_RS01455	2165	T	A	nonsynonymous
283376	NTHI477_RS01455	2166	G	A	synonymous
283377	NTHI477_RS01455	2167	A	C	nonsynonymous
283380	NTHI477_RS01455	2170	A	G	nonsynonymous
283381	NTHI477_RS01455	2171	A	G	nonsynonymous
283382	NTHI477_RS01455	2172	C	T	synonymous
283383	NTHI477_RS01455	2173	A	G	nonsynonymous
283384	NTHI477_RS01455	2174	A	G	nonsynonymous
283385	NTHI477_RS01455	2175	C	A	nonsynonymous
283385	NTHI477_RS01455	2175	C	T	synonymous
283386	NTHI477_RS01455	2176	G	A	nonsynonymous
283391	NTHI477_RS01455	2181	C	T	synonymous
283392	NTHI477_RS01455	2182	G	T	nonsynonymous
283392	NTHI477_RS01455	2182	G	A	nonsynonymous
283395	NTHI477_RS01455	2185	G	A	nonsynonymous
283397	NTHI477_RS01455	2187	T	C	synonymous
283399	NTHI477_RS01455	2189	A	G	nonsynonymous
283400	NTHI477_RS01455	2190	C	T	synonymous
283402	NTHI477_RS01455	2192	C	T	nonsynonymous
283404	NTHI477_RS01455	2194	C	T	nonsynonymous

283409	NTHI477_RS01455	2199	C	T	synonymous
283411	NTHI477_RS01455	2201	A	T	nonsynonymous
283412	NTHI477_RS01455	2202	T	C	synonymous
283424	NTHI477_RS01455	2214	C	T	synonymous
283427	NTHI477_RS01455	2217	C	T	synonymous
283433	NTHI477_RS01455	2223	A	G	synonymous
283448	NTHI477_RS01455	2238	C	T	synonymous
283451	NTHI477_RS01455	2241	T	C	synonymous
283454	NTHI477_RS01455	2244	T	C	synonymous
283457	NTHI477_RS01455	2247	C	T	synonymous
283469	NTHI477_RS01455	2259	C	A	synonymous
283476	NTHI477_RS01455	2266	C	A	nonsynonymous
283478	NTHI477_RS01455	2268	C	A	synonymous
283478	NTHI477_RS01455	2268	C	T	synonymous
283484	NTHI477_RS01455	2274	A	G	synonymous
283490	NTHI477_RS01455	2280	T	C	synonymous
283493	NTHI477_RS01455	2283	C	T	synonymous
283497	NTHI477_RS01455	2287	T	A	nonsynonymous
283520	NTHI477_RS01455	2310	G	A	synonymous
283520	NTHI477_RS01455	2310	G	T	synonymous
283523	NTHI477_RS01455	2313	C	T	synonymous
283548	NTHI477_RS01455	2338	A	G	nonsynonymous
283551	NTHI477_RS01455	2341	A	G	nonsynonymous
283559	NTHI477_RS01455	2349	C	T	synonymous
283561	NTHI477_RS01455	2351	C	T	nonsynonymous
283562	NTHI477_RS01455	2352	C	T	synonymous
283563	NTHI477_RS01455	2353	A	G	nonsynonymous
283564	NTHI477_RS01455	2354	G	A	nonsynonymous
283574	NTHI477_RS01455	2364	T	C	synonymous
283580	NTHI477_RS01455	2370	T	C	synonymous
283592	NTHI477_RS01455	2382	A	G	synonymous
283598	NTHI477_RS01455	2388	G	A	synonymous
283611	NTHI477_RS01455	2401	G	A	nonsynonymous
283630	intergenic	intergenic	T	A	intergenic
283631	intergenic	intergenic	A	T	intergenic
283633	intergenic	intergenic	C	T	intergenic
283651	intergenic	intergenic	G	A	intergenic
283653	intergenic	intergenic	G	T	intergenic
283665	intergenic	intergenic	C	T	intergenic
283666	intergenic	intergenic	A	G	intergenic
283670	intergenic	intergenic	T	A	intergenic
283686	intergenic	intergenic	G	A	intergenic
283690	intergenic	intergenic	A	C	intergenic
283691	intergenic	intergenic	T	G	intergenic
283697	intergenic	intergenic	C	T	intergenic

283750	NTHI477_RS01460	19	C	T	nonsynonymous
283758	NTHI477_RS01460	27	A	G	synonymous
283779	NTHI477_RS01460	48	T	C	synonymous
283788	NTHI477_RS01460	57	A	C	synonymous
283808	NTHI477_RS01460	77	A	G	nonsynonymous
283810	NTHI477_RS01460	79	A	G	nonsynonymous
283854	NTHI477_RS01460	123	T	C	synonymous
283858	NTHI477_RS01460	127	C	T	synonymous
283866	NTHI477_RS01460	135	T	C	synonymous
283890	NTHI477_RS01460	159	T	C	synonymous
283917	NTHI477_RS01460	186	G	A	synonymous
283920	NTHI477_RS01460	189	C	T	synonymous
283926	NTHI477_RS01460	195	A	T	synonymous
283929	NTHI477_RS01460	198	G	C	synonymous
283929	NTHI477_RS01460	198	G	A	synonymous
283932	NTHI477_RS01460	201	A	T	synonymous
283938	NTHI477_RS01460	207	T	C	synonymous
283947	NTHI477_RS01460	216	C	T	synonymous
283965	NTHI477_RS01460	234	T	C	synonymous
283977	NTHI477_RS01460	246	A	G	synonymous
283995	NTHI477_RS01460	264	C	T	synonymous
284040	NTHI477_RS01460	309	A	G	synonymous
284052	NTHI477_RS01460	321	G	A	synonymous
284076	NTHI477_RS01460	345	T	C	synonymous
284079	NTHI477_RS01460	348	C	T	synonymous
284097	NTHI477_RS01460	366	C	A	synonymous
284121	NTHI477_RS01460	390	C	T	synonymous
284142	NTHI477_RS01460	411	G	A	synonymous
284145	NTHI477_RS01460	414	G	T	synonymous
284187	NTHI477_RS01460	456	G	A	synonymous
284188	NTHI477_RS01460	457	C	T	nonsynonymous
284193	NTHI477_RS01460	462	A	G	synonymous
284199	NTHI477_RS01460	468	G	A	synonymous
284214	NTHI477_RS01460	483	T	C	synonymous
284254	NTHI477_RS01460	523	T	C	synonymous
284292	NTHI477_RS01460	561	G	A	synonymous
284295	NTHI477_RS01460	564	T	A	synonymous
284307	NTHI477_RS01460	576	G	A	synonymous
284310	NTHI477_RS01460	579	G	A	synonymous
284319	NTHI477_RS01460	588	T	A	synonymous
284376	NTHI477_RS01460	645	C	T	synonymous
284409	NTHI477_RS01460	678	A	G	synonymous
284421	NTHI477_RS01460	690	C	T	synonymous
284449	NTHI477_RS01460	718	C	T	synonymous
284462	NTHI477_RS01460	731	A	G	nonsynonymous

284463	NTHI477_RS01460	732	T	C	synonymous
284469	NTHI477_RS01460	738	T	C	synonymous
284470	NTHI477_RS01460	739	C	A	nonsynonymous
284484	NTHI477_RS01460	753	T	A	synonymous
284506	NTHI477_RS01460	775	A	G	nonsynonymous
284523	NTHI477_RS01460	792	T	G	synonymous
284535	NTHI477_RS01460	804	A	G	synonymous
284556	NTHI477_RS01460	825	T	A	synonymous
284568	NTHI477_RS01460	837	A	T	synonymous
284577	NTHI477_RS01460	846	G	A	synonymous
284640	NTHI477_RS01460	909	G	A	synonymous
284661	NTHI477_RS01460	930	C	T	synonymous
284666	NTHI477_RS01460	935	G	A	nonsynonymous
284670	NTHI477_RS01460	939	G	A	synonymous
284689	NTHI477_RS01460	958	C	A	nonsynonymous
284701	NTHI477_RS01460	970	G	A	nonsynonymous
284705	NTHI477_RS01460	974	C	T	nonsynonymous
284715	NTHI477_RS01460	984	C	T	synonymous
284727	NTHI477_RS01460	996	A	G	synonymous
284739	NTHI477_RS01460	1008	C	T	synonymous
284760	NTHI477_RS01460	1029	A	C	synonymous
284760	NTHI477_RS01460	1029	A	G	synonymous
284769	NTHI477_RS01460	1038	C	T	synonymous
284862	NTHI477_RS01460	1131	T	C	synonymous
284883	NTHI477_RS01460	1152	A	G	synonymous
284894	NTHI477_RS01460	1163	T	A	nonsynonymous
284910	NTHI477_RS01460	1179	A	C	nonsynonymous
284923	NTHI477_RS01460	1192	A	C	nonsynonymous
284937	NTHI477_RS01460	1206	C	T	synonymous
284964	NTHI477_RS01460	1233	T	G	synonymous
284979	NTHI477_RS01460	1248	G	T	nonsynonymous
284985	NTHI477_RS01460	1254	G	A	synonymous
284991	NTHI477_RS01460	1260	G	A	synonymous
284997	NTHI477_RS01460	1266	A	G	synonymous
285022	NTHI477_RS01460	1291	C	T	nonsynonymous
285046	NTHI477_RS01460	1315	G	T	nonsynonymous
285060	NTHI477_RS01460	1329	C	A	synonymous
285069	NTHI477_RS01460	1338	C	T	synonymous
285079	NTHI477_RS01460	1348	G	T	nonsynonymous
285080	NTHI477_RS01460	1349	C	T	nonsynonymous
285097	NTHI477_RS01460	1366	T	C	synonymous
285105	NTHI477_RS01460	1374	C	T	synonymous
285112	NTHI477_RS01460	1381	C	T	nonsynonymous
285144	NTHI477_RS01460	1413	T	A	synonymous
285146	NTHI477_RS01460	1415	C	T	nonsynonymous

285168	NTHI477_RS01460	1437	C	T	synonymous
285178	NTHI477_RS01460	1447	G	A	nonsynonymous
285192	NTHI477_RS01460	1461	C	T	synonymous
285214	NTHI477_RS01460	1483	A	C	nonsynonymous
285243	NTHI477_RS01460	1512	T	C	synonymous
285258	NTHI477_RS01460	1527	T	C	synonymous
285270	NTHI477_RS01460	1539	C	T	synonymous
285279	NTHI477_RS01460	1548	C	T	synonymous
285279	NTHI477_RS01460	1548	C	A	synonymous
285282	NTHI477_RS01460	1551	C	T	synonymous
285309	NTHI477_RS01460	1578	T	C	synonymous
285327	NTHI477_RS01460	1596	C	T	synonymous
285345	NTHI477_RS01460	1614	T	G	synonymous
285376	NTHI477_RS01460	1645	C	A	nonsynonymous
285390	NTHI477_RS01460	1659	T	C	synonymous
285391	NTHI477_RS01460	1660	A	T	nonsynonymous
285399	NTHI477_RS01460	1668	A	T	synonymous
285420	NTHI477_RS01460	1689	G	A	synonymous
285426	NTHI477_RS01460	1695	A	T	synonymous
285434	NTHI477_RS01460	1703	G	A	nonsynonymous
285477	NTHI477_RS01460	1746	G	T	synonymous
285477	NTHI477_RS01460	1746	G	A	synonymous
285495	NTHI477_RS01460	1764	G	A	synonymous
285497	NTHI477_RS01460	1766	G	A	nonsynonymous
285498	NTHI477_RS01460	1767	C	T	synonymous
285510	NTHI477_RS01460	1779	G	A	synonymous
285543	NTHI477_RS01460	1812	T	C	synonymous
285567	NTHI477_RS01460	1836	G	A	synonymous
285597	NTHI477_RS01460	1866	C	A	synonymous
285609	NTHI477_RS01460	1878	T	C	synonymous
285612	NTHI477_RS01460	1881	T	A	synonymous
285615	NTHI477_RS01460	1884	T	C	synonymous
285615	NTHI477_RS01460	1884	T	G	synonymous
285618	NTHI477_RS01460	1887	T	C	synonymous
285624	NTHI477_RS01460	1893	C	T	synonymous
285625	NTHI477_RS01460	1894	G	A	nonsynonymous
285675	NTHI477_RS01460	1944	G	A	synonymous
285700	NTHI477_RS01460	1969	C	A	nonsynonymous
285706	NTHI477_RS01460	1975	G	A	nonsynonymous
285717	NTHI477_RS01460	1986	A	G	synonymous
285720	NTHI477_RS01460	1989	T	C	synonymous
285746	NTHI477_RS01460	2015	C	G	nonsynonymous
285762	NTHI477_RS01460	2031	G	A	synonymous
285775	NTHI477_RS01460	2044	G	A	nonsynonymous
285791	NTHI477_RS01460	2060	G	A	nonsynonymous

285806	NTHI477_RS01460	2075	A	G	nonsynonymous
285817	NTHI477_RS01460	2086	G	A	nonsynonymous
285882	NTHI477_RS01460	2151	A	G	synonymous
285900	NTHI477_RS01460	2169	T	A	synonymous
285901	NTHI477_RS01460	2170	A	G	nonsynonymous
285921	NTHI477_RS01460	2190	C	A	synonymous
285921	NTHI477_RS01460	2190	C	G	nonsynonymous
285922	NTHI477_RS01460	2191	C	G	nonsynonymous
285924	NTHI477_RS01460	2193	C	T	synonymous
285927	NTHI477_RS01460	2196	T	C	synonymous
285936	NTHI477_RS01460	2205	A	G	synonymous
285939	NTHI477_RS01460	2208	G	T	synonymous
285969	NTHI477_RS01460	2238	T	G	nonsynonymous
285978	NTHI477_RS01460	2247	T	C	synonymous
286036	NTHI477_RS01460	2305	A	G	nonsynonymous
286038	NTHI477_RS01460	2307	A	G	synonymous
286066	NTHI477_RS01460	2335	G	T	nonsynonymous
286068	NTHI477_RS01460	2337	G	T	synonymous
286068	NTHI477_RS01460	2337	G	A	synonymous
286116	NTHI477_RS01460	2385	T	A	synonymous
286119	NTHI477_RS01460	2388	T	A	synonymous
286119	NTHI477_RS01460	2388	T	G	synonymous
286120	NTHI477_RS01460	2389	A	G	nonsynonymous
286156	NTHI477_RS01460	2425	T	C	nonsynonymous
286159	NTHI477_RS01460	2428	G	A	nonsynonymous
286176	NTHI477_RS01460	2445	G	A	synonymous
286197	NTHI477_RS01460	2466	C	T	synonymous
286201	NTHI477_RS01460	2470	A	C	nonsynonymous
286203	NTHI477_RS01460	2472	G	C	nonsynonymous
286206	NTHI477_RS01460	2475	C	A	synonymous
286206	NTHI477_RS01460	2475	C	T	synonymous
286207	NTHI477_RS01460	2476	A	G	nonsynonymous
286209	NTHI477_RS01460	2478	G	A	nonsynonymous
286212	NTHI477_RS01460	2481	G	A	synonymous
286215	NTHI477_RS01460	2484	A	G	synonymous
286239	NTHI477_RS01460	2508	A	G	synonymous
286248	NTHI477_RS01460	2517	C	T	synonymous
286254	NTHI477_RS01460	2523	A	G	synonymous
286275	NTHI477_RS01460	2544	C	T	synonymous
286277	NTHI477_RS01460	2546	T	C	nonsynonymous
286295	NTHI477_RS01460	2564	C	A	nonsynonymous
286342	NTHI477_RS01460	2611	A	G	nonsynonymous
286350	NTHI477_RS01460	2619	A	G	synonymous
286364	NTHI477_RS01460	2633	G	A	nonsynonymous
286392	NTHI477_RS01460	2661	T	C	synonymous

286398	NTHI477_RS01460	2667	T	C	synonymous
286399	NTHI477_RS01460	2668	A	G	nonsynonymous
286408	NTHI477_RS01460	2677	C	A	nonsynonymous
286427	NTHI477_RS01460	2696	C	T	nonsynonymous
286452	NTHI477_RS01460	2721	G	A	synonymous
286461	NTHI477_RS01460	2730	T	C	synonymous
286476	NTHI477_RS01460	2745	C	T	synonymous
286489	NTHI477_RS01460	2758	G	A	nonsynonymous
286501	NTHI477_RS01460	2770	G	A	nonsynonymous
286501	NTHI477_RS01460	2770	G	T	nonsynonymous
286504	NTHI477_RS01460	2773	T	A	nonsynonymous
286522	intergenic	intergenic	T	G	intergenic
286532	intergenic	intergenic	T	G	intergenic
286537	intergenic	intergenic	A	C	intergenic
286543	intergenic	intergenic	T	G	intergenic
286543	intergenic	intergenic	T	C	intergenic
286553	intergenic	intergenic	G	T	intergenic
286559	intergenic	intergenic	T	C	intergenic
286560	intergenic	intergenic	G	A	intergenic
286565	intergenic	intergenic	C	T	intergenic
286575	intergenic	intergenic	C	T	intergenic
286587	intergenic	intergenic	G	A	intergenic
286590	intergenic	intergenic	C	A	intergenic
286592	intergenic	intergenic	C	T	intergenic
286595	intergenic	intergenic	T	A	intergenic
286596	intergenic	intergenic	G	A	intergenic
286599	intergenic	intergenic	T	G	intergenic
286603	intergenic	intergenic	C	T	intergenic
286605	intergenic	intergenic	G	A	intergenic
286626	intergenic	intergenic	C	T	intergenic
286629	intergenic	intergenic	A	G	intergenic
286654	NTHI477_RS01465	1913	G	T	nonsynonymous
286665	NTHI477_RS01465	1902	G	A	synonymous
286683	NTHI477_RS01465	1884	C	T	synonymous
286707	NTHI477_RS01465	1860	A	T	synonymous
286715	NTHI477_RS01465	1852	T	A	nonsynonymous
286716	NTHI477_RS01465	1851	G	A	synonymous
286725	NTHI477_RS01465	1842	A	T	synonymous
286746	NTHI477_RS01465	1821	T	A	synonymous
286749	NTHI477_RS01465	1818	C	G	synonymous
286751	NTHI477_RS01465	1816	C	T	nonsynonymous
286755	NTHI477_RS01465	1812	G	A	synonymous
286755	NTHI477_RS01465	1812	G	T	synonymous
286761	NTHI477_RS01465	1806	T	A	synonymous
286773	NTHI477_RS01465	1794	A	G	synonymous

286776	NTHI477_RS01465	1791	A	G	synonymous
286779	NTHI477_RS01465	1788	A	G	synonymous
286785	NTHI477_RS01465	1782	A	G	synonymous
286800	NTHI477_RS01465	1767	A	G	synonymous
286803	NTHI477_RS01465	1764	G	A	synonymous
286815	NTHI477_RS01465	1752	T	C	synonymous
286821	NTHI477_RS01465	1746	A	G	synonymous
286827	NTHI477_RS01465	1740	G	A	synonymous
286830	NTHI477_RS01465	1737	G	A	synonymous
286833	NTHI477_RS01465	1734	C	T	synonymous
286842	NTHI477_RS01465	1725	G	A	synonymous
286845	NTHI477_RS01465	1722	A	T	synonymous
286848	NTHI477_RS01465	1719	T	G	nonsynonymous
286850	NTHI477_RS01465	1717	A	G	synonymous
286851	NTHI477_RS01465	1716	A	G	synonymous
286854	NTHI477_RS01465	1713	A	T	synonymous
286854	NTHI477_RS01465	1713	A	G	synonymous
286856	NTHI477_RS01465	1711	T	C	nonsynonymous
286860	NTHI477_RS01465	1707	C	A	synonymous
286860	NTHI477_RS01465	1707	C	T	synonymous
286863	NTHI477_RS01465	1704	G	A	synonymous
286873	NTHI477_RS01465	1694	A	G	nonsynonymous
286878	NTHI477_RS01465	1689	A	G	synonymous
286884	NTHI477_RS01465	1683	C	G	nonsynonymous
286884	NTHI477_RS01465	1683	C	T	synonymous
286890	NTHI477_RS01465	1677	T	A	synonymous
286890	NTHI477_RS01465	1677	T	C	synonymous
286891	NTHI477_RS01465	1676	G	A	nonsynonymous
286893	NTHI477_RS01465	1674	G	T	synonymous
286893	NTHI477_RS01465	1674	G	A	synonymous
286898	NTHI477_RS01465	1669	T	G	nonsynonymous
286899	NTHI477_RS01465	1668	C	T	synonymous
286911	NTHI477_RS01465	1656	T	A	synonymous
286923	NTHI477_RS01465	1644	G	A	synonymous
286926	NTHI477_RS01465	1641	G	A	synonymous
286935	NTHI477_RS01465	1632	T	C	synonymous
286935	NTHI477_RS01465	1632	T	A	synonymous
286938	NTHI477_RS01465	1629	A	T	synonymous
286941	NTHI477_RS01465	1626	T	C	synonymous
286953	NTHI477_RS01465	1614	G	A	synonymous
286953	NTHI477_RS01465	1614	G	T	synonymous
286962	NTHI477_RS01465	1605	T	C	synonymous
286965	NTHI477_RS01465	1602	A	G	synonymous
286977	NTHI477_RS01465	1590	T	A	synonymous
286992	NTHI477_RS01465	1575	A	G	synonymous

286995	NTHI477_RS01465	1572	A	G	synonymous
287016	NTHI477_RS01465	1551	A	T	synonymous
287019	NTHI477_RS01465	1548	A	G	synonymous
287034	NTHI477_RS01465	1533	G	A	synonymous
287043	NTHI477_RS01465	1524	G	T	synonymous
287043	NTHI477_RS01465	1524	G	A	synonymous
287064	NTHI477_RS01465	1503	A	G	synonymous
287070	NTHI477_RS01465	1497	T	A	synonymous
287073	NTHI477_RS01465	1494	T	C	synonymous
287091	NTHI477_RS01465	1476	A	G	synonymous
287097	NTHI477_RS01465	1470	T	A	synonymous
287097	NTHI477_RS01465	1470	T	C	synonymous
287103	NTHI477_RS01465	1464	T	C	synonymous
287106	NTHI477_RS01465	1461	G	A	synonymous
287118	NTHI477_RS01465	1449	C	A	synonymous
287121	NTHI477_RS01465	1446	G	A	synonymous
287121	NTHI477_RS01465	1446	G	T	synonymous
287147	NTHI477_RS01465	1420	A	G	synonymous
287154	NTHI477_RS01465	1413	T	G	synonymous
287159	NTHI477_RS01465	1408	G	A	synonymous
287163	NTHI477_RS01465	1404	A	G	synonymous
287172	NTHI477_RS01465	1395	T	C	synonymous
287175	NTHI477_RS01465	1392	T	C	synonymous
287196	NTHI477_RS01465	1371	A	G	synonymous
287205	NTHI477_RS01465	1362	A	G	synonymous
287226	NTHI477_RS01465	1341	G	A	synonymous
287229	NTHI477_RS01465	1338	T	C	synonymous
287235	NTHI477_RS01465	1332	G	T	synonymous
287238	NTHI477_RS01465	1329	T	A	synonymous
287241	NTHI477_RS01465	1326	A	T	synonymous
287241	NTHI477_RS01465	1326	A	G	synonymous
287256	NTHI477_RS01465	1311	C	T	synonymous
287259	NTHI477_RS01465	1308	A	G	synonymous
287259	NTHI477_RS01465	1308	A	T	synonymous
287271	NTHI477_RS01465	1296	T	C	synonymous
287283	NTHI477_RS01465	1284	G	A	synonymous
287295	NTHI477_RS01465	1272	A	C	synonymous
287298	NTHI477_RS01465	1269	G	A	synonymous
287313	NTHI477_RS01465	1254	T	C	synonymous
287319	NTHI477_RS01465	1248	G	A	synonymous
287331	NTHI477_RS01465	1236	G	A	synonymous
287364	NTHI477_RS01465	1203	G	A	synonymous
287382	NTHI477_RS01465	1185	G	A	synonymous
287412	NTHI477_RS01465	1155	G	A	synonymous
287415	NTHI477_RS01465	1152	A	C	synonymous

287439	NTHI477_RS01465	1128	C	A	synonymous
287457	NTHI477_RS01465	1110	T	C	synonymous
287463	NTHI477_RS01465	1104	A	G	synonymous
287466	NTHI477_RS01465	1101	T	C	synonymous
287472	NTHI477_RS01465	1095	T	C	synonymous
287484	NTHI477_RS01465	1083	G	A	synonymous
287499	NTHI477_RS01465	1068	C	T	synonymous
287505	NTHI477_RS01465	1062	A	G	synonymous
287523	NTHI477_RS01465	1044	G	A	synonymous
287550	NTHI477_RS01465	1017	T	C	synonymous
287559	NTHI477_RS01465	1008	G	T	synonymous
287559	NTHI477_RS01465	1008	G	A	synonymous
287562	NTHI477_RS01465	1005	T	A	synonymous
287565	NTHI477_RS01465	1002	G	A	synonymous
287586	NTHI477_RS01465	981	G	A	synonymous
287607	NTHI477_RS01465	960	A	G	synonymous
287610	NTHI477_RS01465	957	T	G	synonymous
287613	NTHI477_RS01465	954	G	A	synonymous
287646	NTHI477_RS01465	921	A	T	synonymous
287661	NTHI477_RS01465	906	T	C	synonymous
287664	NTHI477_RS01465	903	G	T	synonymous
287679	NTHI477_RS01465	888	T	C	synonymous
287682	NTHI477_RS01465	885	G	C	synonymous
287688	NTHI477_RS01465	879	C	T	synonymous
287757	NTHI477_RS01465	810	G	A	synonymous
287763	NTHI477_RS01465	804	A	G	synonymous
287766	NTHI477_RS01465	801	G	A	synonymous
287772	NTHI477_RS01465	795	G	A	synonymous
287775	NTHI477_RS01465	792	C	T	synonymous
287784	NTHI477_RS01465	783	T	C	synonymous
287787	NTHI477_RS01465	780	T	C	synonymous
287835	NTHI477_RS01465	732	G	A	synonymous
287838	NTHI477_RS01465	729	G	A	synonymous
287841	NTHI477_RS01465	726	A	G	synonymous
287863	NTHI477_RS01465	704	C	T	nonsynonymous
287871	NTHI477_RS01465	696	A	G	synonymous
287877	NTHI477_RS01465	690	A	C	synonymous
287883	NTHI477_RS01465	684	T	C	synonymous
287904	NTHI477_RS01465	663	T	C	synonymous
287913	NTHI477_RS01465	654	G	A	synonymous
287937	NTHI477_RS01465	630	A	G	synonymous
287952	NTHI477_RS01465	615	G	A	synonymous
288002	NTHI477_RS01465	565	T	C	nonsynonymous
288015	NTHI477_RS01465	552	G	C	synonymous
288015	NTHI477_RS01465	552	G	T	synonymous

288015	NTHI477_RS01465	552	G	A	synonymous
288027	NTHI477_RS01465	540	G	A	synonymous
288061	NTHI477_RS01465	506	G	A	nonsynonymous
288114	NTHI477_RS01465	453	T	G	synonymous
288118	NTHI477_RS01465	449	T	C	nonsynonymous
288135	NTHI477_RS01465	432	C	T	synonymous
288177	NTHI477_RS01465	390	T	G	synonymous
288183	NTHI477_RS01465	384	T	C	synonymous
288216	NTHI477_RS01465	351	A	T	synonymous
288218	NTHI477_RS01465	349	T	A	nonsynonymous
288267	NTHI477_RS01465	300	G	A	synonymous
288276	NTHI477_RS01465	291	A	G	synonymous
288282	NTHI477_RS01465	285	A	G	synonymous
288302	NTHI477_RS01465	265	C	T	nonsynonymous
288321	NTHI477_RS01465	246	A	T	synonymous
288330	NTHI477_RS01465	237	G	A	synonymous
288342	NTHI477_RS01465	225	G	A	synonymous
288382	NTHI477_RS01465	185	G	A	nonsynonymous
288390	NTHI477_RS01465	177	A	G	synonymous
288396	NTHI477_RS01465	171	T	A	nonsynonymous
288398	NTHI477_RS01465	169	A	G	synonymous
288399	NTHI477_RS01465	168	C	T	synonymous
288419	NTHI477_RS01465	148	C	T	nonsynonymous
288433	NTHI477_RS01465	134	C	T	nonsynonymous
288444	NTHI477_RS01465	123	G	A	synonymous
288481	NTHI477_RS01465	86	G	A	nonsynonymous
288520	NTHI477_RS01465	47	C	T	nonsynonymous
288521	NTHI477_RS01465	46	G	T	nonsynonymous
288528	NTHI477_RS01465	39	T	C	synonymous
288614	intergenic	intergenic	A	G	intergenic
288628	intergenic	intergenic	A	C	intergenic
288638	intergenic	intergenic	C	T	intergenic
288651	intergenic	intergenic	T	A	intergenic
288724	intergenic	intergenic	G	A	intergenic
288726	intergenic	intergenic	A	G	intergenic
288745	intergenic	intergenic	C	A	intergenic
288757	intergenic	intergenic	A	G	intergenic
288779	intergenic	intergenic	A	G	intergenic
288781	intergenic	intergenic	T	G	intergenic
288783	intergenic	intergenic	A	G	intergenic
288784	intergenic	intergenic	A	G	intergenic
288793	intergenic	intergenic	C	A	intergenic
288795	intergenic	intergenic	C	T	intergenic
288796	intergenic	intergenic	T	C	intergenic
288808	intergenic	intergenic	G	A	intergenic

288812	intergenic	intergenic	G	A	intergenic
288830	intergenic	intergenic	A	C	intergenic
288833	intergenic	intergenic	C	G	intergenic
288834	intergenic	intergenic	C	A	intergenic
288834	intergenic	intergenic	C	G	intergenic
288835	intergenic	intergenic	C	T	intergenic
288836	intergenic	intergenic	A	T	intergenic
288839	intergenic	intergenic	T	C	intergenic
288855	NTHI477_RS01470	5	G	A	nonsynonymous
288898	NTHI477_RS01470	48	G	T	synonymous
288901	NTHI477_RS01470	51	A	G	synonymous
288915	NTHI477_RS01470	65	C	T	nonsynonymous
288922	NTHI477_RS01470	72	T	C	synonymous
288924	NTHI477_RS01470	74	T	C	nonsynonymous
288926	NTHI477_RS01470	76	A	G	nonsynonymous
288953	NTHI477_RS01470	103	G	A	nonsynonymous
288955	NTHI477_RS01470	105	A	C	synonymous
288956	NTHI477_RS01470	106	G	A	nonsynonymous
288970	NTHI477_RS01470	120	C	T	synonymous
288979	NTHI477_RS01470	129	T	G	synonymous
288999	NTHI477_RS01470	149	C	T	nonsynonymous
289003	NTHI477_RS01470	153	T	C	synonymous
289015	NTHI477_RS01470	165	G	A	synonymous
289021	NTHI477_RS01470	171	A	G	synonymous
289021	NTHI477_RS01470	171	A	T	synonymous
289032	NTHI477_RS01470	182	C	T	nonsynonymous
289033	NTHI477_RS01470	183	A	G	synonymous
289035	NTHI477_RS01470	185	C	T	nonsynonymous
289036	NTHI477_RS01470	186	A	T	synonymous
289044	NTHI477_RS01470	194	A	C	nonsynonymous
289052	NTHI477_RS01470	202	C	T	synonymous
289054	NTHI477_RS01470	204	G	A	synonymous
289067	NTHI477_RS01470	217	A	G	nonsynonymous
289070	NTHI477_RS01470	220	C	T	synonymous
289090	NTHI477_RS01470	240	C	T	synonymous
289096	NTHI477_RS01470	246	A	G	synonymous
289102	NTHI477_RS01470	252	G	A	synonymous
289114	NTHI477_RS01470	264	G	A	synonymous
289117	NTHI477_RS01470	267	G	A	synonymous
289133	NTHI477_RS01470	283	G	A	nonsynonymous
289169	NTHI477_RS01470	319	G	T	nonsynonymous
289178	NTHI477_RS01470	328	C	A	nonsynonymous
289179	NTHI477_RS01470	329	A	G	nonsynonymous
289204	NTHI477_RS01470	354	C	G	nonsynonymous
289207	NTHI477_RS01470	357	C	T	synonymous

289208	NTHI477_RS01470	358	T	C	nonsynonymous
289213	NTHI477_RS01470	363	G	A	synonymous
289219	NTHI477_RS01470	369	A	G	synonymous
289222	NTHI477_RS01470	372	A	G	synonymous
289236	NTHI477_RS01470	386	C	T	nonsynonymous
289237	NTHI477_RS01470	387	C	G	synonymous
289261	NTHI477_RS01470	411	T	G	synonymous
289261	NTHI477_RS01470	411	T	A	synonymous
289280	NTHI477_RS01470	430	C	T	synonymous
289285	NTHI477_RS01470	435	G	A	synonymous
289318	NTHI477_RS01470	468	C	T	synonymous
289321	NTHI477_RS01470	471	T	C	synonymous
289363	NTHI477_RS01470	513	C	G	nonsynonymous
289366	NTHI477_RS01470	516	G	T	synonymous
289366	NTHI477_RS01470	516	G	A	synonymous
289367	NTHI477_RS01470	517	C	G	nonsynonymous
289367	NTHI477_RS01470	517	C	T	nonsynonymous
289369	NTHI477_RS01470	519	A	T	synonymous
289372	NTHI477_RS01470	522	G	A	synonymous
289375	NTHI477_RS01470	525	G	C	synonymous
289375	NTHI477_RS01470	525	G	A	synonymous
289387	NTHI477_RS01470	537	C	A	synonymous
289391	NTHI477_RS01470	541	G	T	nonsynonymous
289393	NTHI477_RS01470	543	G	A	synonymous
289396	NTHI477_RS01470	546	C	T	synonymous
289399	NTHI477_RS01470	549	A	G	synonymous
289416	NTHI477_RS01470	566	C	A	nonsynonymous
289418	NTHI477_RS01470	568	A	C	nonsynonymous
289421	NTHI477_RS01470	571	G	A	nonsynonymous
289423	NTHI477_RS01470	573	A	C	synonymous
289424	NTHI477_RS01470	574	G	A	nonsynonymous
289427	NTHI477_RS01470	577	G	A	nonsynonymous
289428	NTHI477_RS01470	578	C	G	nonsynonymous
289428	NTHI477_RS01470	578	C	A	nonsynonymous
289438	intergenic	intergenic	C	T	intergenic
289451	intergenic	intergenic	C	A	intergenic
289456	intergenic	intergenic	C	T	intergenic
289461	intergenic	intergenic	T	G	intergenic
289467	intergenic	intergenic	T	G	intergenic
289473	intergenic	intergenic	C	A	intergenic
289477	intergenic	intergenic	C	A	intergenic
289491	intergenic	intergenic	A	T	intergenic
289506	intergenic	intergenic	T	C	intergenic
289509	intergenic	intergenic	C	G	intergenic
289516	NTHI477_RS01475	2601	C	T	synonymous

289537	NTHI477_RS01475	2580	A	G	synonymous
289561	NTHI477_RS01475	2556	C	A	synonymous
289588	NTHI477_RS01475	2529	C	G	synonymous
289597	NTHI477_RS01475	2520	C	T	synonymous
289600	NTHI477_RS01475	2517	T	C	synonymous
289609	NTHI477_RS01475	2508	A	G	synonymous
289633	NTHI477_RS01475	2484	G	A	synonymous
289637	NTHI477_RS01475	2480	C	T	nonsynonymous
289642	NTHI477_RS01475	2475	A	T	synonymous
289645	NTHI477_RS01475	2472	A	G	synonymous
289657	NTHI477_RS01475	2460	T	A	synonymous
289663	NTHI477_RS01475	2454	C	G	synonymous
289708	NTHI477_RS01475	2409	A	T	synonymous
289708	NTHI477_RS01475	2409	A	C	synonymous
289714	NTHI477_RS01475	2403	T	A	synonymous
289716	NTHI477_RS01475	2401	A	C	nonsynonymous
289741	NTHI477_RS01475	2376	G	A	synonymous
289741	NTHI477_RS01475	2376	G	T	synonymous
289747	NTHI477_RS01475	2370	C	A	synonymous
289747	NTHI477_RS01475	2370	C	T	synonymous
289759	NTHI477_RS01475	2358	G	T	synonymous
289771	NTHI477_RS01475	2346	C	T	synonymous
289774	NTHI477_RS01475	2343	G	A	synonymous
289774	NTHI477_RS01475	2343	G	C	synonymous
289780	NTHI477_RS01475	2337	T	C	synonymous
289792	NTHI477_RS01475	2325	C	T	synonymous
289801	NTHI477_RS01475	2316	G	A	synonymous
289804	NTHI477_RS01475	2313	C	T	synonymous
289819	NTHI477_RS01475	2298	A	G	synonymous
289827	NTHI477_RS01475	2290	G	A	synonymous
289864	NTHI477_RS01475	2253	A	G	synonymous
289867	NTHI477_RS01475	2250	T	C	synonymous
289874	NTHI477_RS01475	2243	T	C	nonsynonymous
289898	NTHI477_RS01475	2219	T	C	nonsynonymous
289900	NTHI477_RS01475	2217	G	A	synonymous
289909	NTHI477_RS01475	2208	G	A	synonymous
289944	NTHI477_RS01475	2173	A	G	synonymous
289945	NTHI477_RS01475	2172	G	A	synonymous
289954	NTHI477_RS01475	2163	C	G	synonymous
289957	NTHI477_RS01475	2160	A	G	synonymous
289980	NTHI477_RS01475	2137	C	T	nonsynonymous
290023	NTHI477_RS01475	2094	C	T	synonymous
290029	NTHI477_RS01475	2088	T	C	synonymous
290053	NTHI477_RS01475	2064	A	G	synonymous
290065	NTHI477_RS01475	2052	T	C	synonymous

290074	NTHI477_RS01475	2043	G	T	synonymous
290076	NTHI477_RS01475	2041	C	A	nonsynonymous
290104	NTHI477_RS01475	2013	T	A	synonymous
290116	NTHI477_RS01475	2001	A	G	synonymous
290128	NTHI477_RS01475	1989	A	C	nonsynonymous
290143	NTHI477_RS01475	1974	G	A	synonymous
290166	NTHI477_RS01475	1951	C	T	nonsynonymous
290167	NTHI477_RS01475	1950	G	A	synonymous
290170	NTHI477_RS01475	1947	C	T	synonymous
290172	NTHI477_RS01475	1945	A	G	synonymous
290176	NTHI477_RS01475	1941	T	C	synonymous
290179	NTHI477_RS01475	1938	T	C	synonymous
290185	NTHI477_RS01475	1932	G	A	synonymous
290203	NTHI477_RS01475	1914	T	C	synonymous
290209	NTHI477_RS01475	1908	G	A	synonymous
290215	NTHI477_RS01475	1902	T	C	synonymous
290215	NTHI477_RS01475	1902	T	A	nonsynonymous
290226	NTHI477_RS01475	1891	A	G	nonsynonymous
290227	NTHI477_RS01475	1890	T	C	synonymous
290230	NTHI477_RS01475	1887	C	T	synonymous
290236	NTHI477_RS01475	1881	C	A	synonymous
290239	NTHI477_RS01475	1878	C	T	synonymous
290241	NTHI477_RS01475	1876	T	A	nonsynonymous
290242	NTHI477_RS01475	1875	G	A	synonymous
290254	NTHI477_RS01475	1863	C	T	synonymous
290255	NTHI477_RS01475	1862	A	C	nonsynonymous
290260	NTHI477_RS01475	1857	T	C	synonymous
290264	NTHI477_RS01475	1853	T	C	nonsynonymous
290272	NTHI477_RS01475	1845	G	A	synonymous
290278	NTHI477_RS01475	1839	T	G	synonymous
290278	NTHI477_RS01475	1839	T	A	synonymous
290284	NTHI477_RS01475	1833	T	A	nonsynonymous
290290	NTHI477_RS01475	1827	A	G	synonymous
290291	NTHI477_RS01475	1826	C	T	nonsynonymous
290293	NTHI477_RS01475	1824	A	G	synonymous
290296	NTHI477_RS01475	1821	A	G	synonymous
290314	NTHI477_RS01475	1803	T	G	synonymous
290320	NTHI477_RS01475	1797	C	T	synonymous
290323	NTHI477_RS01475	1794	A	G	synonymous
290326	NTHI477_RS01475	1791	G	A	synonymous
290327	NTHI477_RS01475	1790	C	T	nonsynonymous
290335	NTHI477_RS01475	1782	G	A	synonymous
290344	NTHI477_RS01475	1773	G	A	synonymous
290377	NTHI477_RS01475	1740	T	C	synonymous
290395	NTHI477_RS01475	1722	G	A	synonymous

290407	NTHI477_RS01475	1710	T	C	synonymous
290425	NTHI477_RS01475	1692	T	C	synonymous
290452	NTHI477_RS01475	1665	A	G	synonymous
290467	NTHI477_RS01475	1650	C	T	synonymous
290488	NTHI477_RS01475	1629	T	C	synonymous
290494	NTHI477_RS01475	1623	G	A	synonymous
290503	NTHI477_RS01475	1614	T	C	synonymous
290521	NTHI477_RS01475	1596	T	C	synonymous
290536	NTHI477_RS01475	1581	C	T	synonymous
290566	NTHI477_RS01475	1551	A	G	synonymous
290575	NTHI477_RS01475	1542	A	G	synonymous
290578	NTHI477_RS01475	1539	A	T	synonymous
290590	NTHI477_RS01475	1527	A	G	synonymous
290593	NTHI477_RS01475	1524	G	A	synonymous
290617	NTHI477_RS01475	1500	A	G	synonymous
290686	NTHI477_RS01475	1431	A	G	synonymous
290698	NTHI477_RS01475	1419	A	T	synonymous
290707	NTHI477_RS01475	1410	T	C	synonymous
290734	NTHI477_RS01475	1383	C	A	synonymous
290739	NTHI477_RS01475	1378	T	C	nonsynonymous
290744	NTHI477_RS01475	1373	G	A	nonsynonymous
290748	NTHI477_RS01475	1369	G	A	nonsynonymous
290749	NTHI477_RS01475	1368	C	T	synonymous
290773	NTHI477_RS01475	1344	G	A	synonymous
290779	NTHI477_RS01475	1338	C	T	synonymous
290785	NTHI477_RS01475	1332	C	T	synonymous
290803	NTHI477_RS01475	1314	A	G	synonymous
290821	NTHI477_RS01475	1296	G	T	synonymous
290824	NTHI477_RS01475	1293	T	C	synonymous
290833	NTHI477_RS01475	1284	T	C	synonymous
290836	NTHI477_RS01475	1281	G	A	synonymous
290848	NTHI477_RS01475	1269	T	A	synonymous
290848	NTHI477_RS01475	1269	T	G	synonymous
290851	NTHI477_RS01475	1266	T	G	synonymous
290853	NTHI477_RS01475	1264	A	T	nonsynonymous
290854	NTHI477_RS01475	1263	C	A	synonymous
290857	NTHI477_RS01475	1260	A	G	synonymous
290857	NTHI477_RS01475	1260	A	T	synonymous
290862	NTHI477_RS01475	1255	A	G	synonymous
290863	NTHI477_RS01475	1254	T	C	synonymous
290866	NTHI477_RS01475	1251	A	G	synonymous
290869	NTHI477_RS01475	1248	G	A	synonymous
290884	NTHI477_RS01475	1233	A	T	synonymous
290887	NTHI477_RS01475	1230	C	T	synonymous
290896	NTHI477_RS01475	1221	A	G	synonymous

290899	NTHI477_RS01475	1218	G	T	synonymous
290900	NTHI477_RS01475	1217	G	A	nonsynonymous
290905	NTHI477_RS01475	1212	G	A	synonymous
290908	NTHI477_RS01475	1209	C	T	synonymous
290913	NTHI477_RS01475	1204	G	A	nonsynonymous
290932	NTHI477_RS01475	1185	A	G	synonymous
290935	NTHI477_RS01475	1182	T	C	synonymous
290938	NTHI477_RS01475	1179	T	C	synonymous
290941	NTHI477_RS01475	1176	A	G	synonymous
290953	NTHI477_RS01475	1164	C	T	synonymous
290962	NTHI477_RS01475	1155	A	G	synonymous
290971	NTHI477_RS01475	1146	C	T	synonymous
290977	NTHI477_RS01475	1140	T	G	nonsynonymous
290978	NTHI477_RS01475	1139	T	C	nonsynonymous
290983	NTHI477_RS01475	1134	A	G	synonymous
290986	NTHI477_RS01475	1131	T	C	synonymous
290986	NTHI477_RS01475	1131	T	A	synonymous
290992	NTHI477_RS01475	1125	A	G	synonymous
291004	NTHI477_RS01475	1113	A	G	synonymous
291007	NTHI477_RS01475	1110	C	A	synonymous
291007	NTHI477_RS01475	1110	C	T	synonymous
291016	NTHI477_RS01475	1101	C	T	synonymous
291031	NTHI477_RS01475	1086	T	A	synonymous
291037	NTHI477_RS01475	1080	G	A	synonymous
291051	NTHI477_RS01475	1066	G	C	nonsynonymous
291055	NTHI477_RS01475	1062	T	C	synonymous
291061	NTHI477_RS01475	1056	T	C	synonymous
291064	NTHI477_RS01475	1053	C	T	synonymous
291065	NTHI477_RS01475	1052	G	A	nonsynonymous
291067	NTHI477_RS01475	1050	C	A	synonymous
291085	NTHI477_RS01475	1032	G	A	synonymous
291094	NTHI477_RS01475	1023	C	T	synonymous
291103	NTHI477_RS01475	1014	C	A	synonymous
291106	NTHI477_RS01475	1011	C	T	synonymous
291109	NTHI477_RS01475	1008	A	G	synonymous
291118	NTHI477_RS01475	999	T	C	synonymous
291130	NTHI477_RS01475	987	G	A	synonymous
291133	NTHI477_RS01475	984	T	C	synonymous
291154	NTHI477_RS01475	963	G	A	synonymous
291157	NTHI477_RS01475	960	T	C	synonymous
291172	NTHI477_RS01475	945	T	C	synonymous
291175	NTHI477_RS01475	942	G	A	synonymous
291190	NTHI477_RS01475	927	T	C	synonymous
291193	NTHI477_RS01475	924	C	T	synonymous
291199	NTHI477_RS01475	918	G	A	synonymous

291208	NTHI477_RS01475	909	T	C	synonymous
291211	NTHI477_RS01475	906	A	G	synonymous
291223	NTHI477_RS01475	894	C	A	synonymous
291223	NTHI477_RS01475	894	C	T	synonymous
291235	NTHI477_RS01475	882	T	C	synonymous
291253	NTHI477_RS01475	864	C	T	synonymous
291256	NTHI477_RS01475	861	A	T	synonymous
291256	NTHI477_RS01475	861	A	G	synonymous
291283	NTHI477_RS01475	834	G	T	nonsynonymous
291286	NTHI477_RS01475	831	T	C	synonymous
291291	NTHI477_RS01475	826	T	C	nonsynonymous
291307	NTHI477_RS01475	810	A	T	synonymous
291316	NTHI477_RS01475	801	A	C	synonymous
291340	NTHI477_RS01475	777	C	T	synonymous
291352	NTHI477_RS01475	765	C	T	synonymous
291385	NTHI477_RS01475	732	G	A	synonymous
291391	NTHI477_RS01475	726	C	T	synonymous
291394	NTHI477_RS01475	723	A	G	synonymous
291400	NTHI477_RS01475	717	G	A	synonymous
291402	NTHI477_RS01475	715	C	A	nonsynonymous
291418	NTHI477_RS01475	699	A	G	synonymous
291421	NTHI477_RS01475	696	T	C	synonymous
291424	NTHI477_RS01475	693	C	A	synonymous
291454	NTHI477_RS01475	663	C	T	synonymous
291460	NTHI477_RS01475	657	T	C	synonymous
291460	NTHI477_RS01475	657	T	A	synonymous
291461	NTHI477_RS01475	656	G	A	nonsynonymous
291475	NTHI477_RS01475	642	C	T	synonymous
291484	NTHI477_RS01475	633	T	G	synonymous
291505	NTHI477_RS01475	612	C	T	synonymous
291520	NTHI477_RS01475	597	A	T	synonymous
291529	NTHI477_RS01475	588	T	C	synonymous
291535	NTHI477_RS01475	582	T	A	synonymous
291538	NTHI477_RS01475	579	G	C	synonymous
291555	NTHI477_RS01475	562	A	G	synonymous
291556	NTHI477_RS01475	561	C	T	synonymous
291559	NTHI477_RS01475	558	A	C	synonymous
291559	NTHI477_RS01475	558	A	T	synonymous
291562	NTHI477_RS01475	555	C	T	synonymous
291607	NTHI477_RS01475	510	A	G	synonymous
291616	NTHI477_RS01475	501	A	T	synonymous
291622	NTHI477_RS01475	495	T	C	nonsynonymous
291628	NTHI477_RS01475	489	A	T	synonymous
291630	NTHI477_RS01475	487	G	A	nonsynonymous
291640	NTHI477_RS01475	477	T	C	synonymous

291652	NTHI477_RS01475	465	T	C	synonymous
291658	NTHI477_RS01475	459	T	A	synonymous
291661	NTHI477_RS01475	456	T	G	synonymous
291673	NTHI477_RS01475	444	G	T	synonymous
291708	NTHI477_RS01475	409	C	T	nonsynonymous
291709	NTHI477_RS01475	408	A	G	synonymous
291715	NTHI477_RS01475	402	G	A	synonymous
291721	NTHI477_RS01475	396	T	C	synonymous
291739	NTHI477_RS01475	378	T	C	synonymous
291784	NTHI477_RS01475	333	G	A	synonymous
291789	NTHI477_RS01475	328	C	T	nonsynonymous
291808	NTHI477_RS01475	309	C	T	synonymous
291814	NTHI477_RS01475	303	T	C	synonymous
291823	NTHI477_RS01475	294	C	T	synonymous
291838	NTHI477_RS01475	279	A	T	synonymous
291843	NTHI477_RS01475	274	A	G	synonymous
291868	NTHI477_RS01475	249	G	A	synonymous
291883	NTHI477_RS01475	234	C	A	synonymous
291891	NTHI477_RS01475	226	G	T	nonsynonymous
291922	NTHI477_RS01475	195	C	T	synonymous
291934	NTHI477_RS01475	183	T	C	synonymous
291944	NTHI477_RS01475	173	G	A	nonsynonymous
291947	NTHI477_RS01475	170	T	C	nonsynonymous
291958	NTHI477_RS01475	159	G	A	synonymous
291982	NTHI477_RS01475	135	A	G	synonymous
291997	NTHI477_RS01475	120	A	T	synonymous
292000	NTHI477_RS01475	117	C	A	synonymous
292003	NTHI477_RS01475	114	C	T	synonymous
292004	NTHI477_RS01475	113	G	T	nonsynonymous
292006	NTHI477_RS01475	111	C	T	synonymous
292024	NTHI477_RS01475	93	A	G	synonymous
292024	NTHI477_RS01475	93	A	C	synonymous
292027	NTHI477_RS01475	90	G	A	synonymous
292036	NTHI477_RS01475	81	C	A	synonymous
292039	NTHI477_RS01475	78	G	C	synonymous
292063	NTHI477_RS01475	54	A	G	synonymous
292102	NTHI477_RS01475	15	C	T	synonymous
292111	NTHI477_RS01475	6	A	T	nonsynonymous
292132	intergenic	intergenic	T	C	intergenic
292144	intergenic	intergenic	G	A	intergenic
292147	intergenic	intergenic	A	G	intergenic
292153	intergenic	intergenic	G	C	intergenic
292154	intergenic	intergenic	C	G	intergenic
292175	intergenic	intergenic	T	A	intergenic
292176	intergenic	intergenic	G	A	intergenic

292226	NTHI477_RS01480	15	C	T	synonymous
292244	NTHI477_RS01480	33	C	T	synonymous
292248	NTHI477_RS01480	37	G	A	nonsynonymous
292271	NTHI477_RS01480	60	T	A	synonymous
292286	NTHI477_RS01480	75	A	G	synonymous
292301	NTHI477_RS01480	90	G	A	synonymous
292313	NTHI477_RS01480	102	C	T	synonymous
292340	NTHI477_RS01480	129	T	C	synonymous
292348	NTHI477_RS01480	137	C	T	nonsynonymous
292355	NTHI477_RS01480	144	G	A	synonymous
292367	NTHI477_RS01480	156	C	T	synonymous
292388	NTHI477_RS01480	177	T	C	synonymous
292394	NTHI477_RS01480	183	T	A	synonymous
292398	NTHI477_RS01480	187	A	C	nonsynonymous
292400	NTHI477_RS01480	189	G	A	nonsynonymous
292409	NTHI477_RS01480	198	T	C	synonymous
292415	NTHI477_RS01480	204	C	T	synonymous
292418	NTHI477_RS01480	207	T	C	synonymous
292424	NTHI477_RS01480	213	C	A	synonymous
292430	NTHI477_RS01480	219	G	A	synonymous
292460	NTHI477_RS01480	249	T	C	synonymous
292463	NTHI477_RS01480	252	T	C	synonymous
292466	NTHI477_RS01480	255	G	C	nonsynonymous
292496	NTHI477_RS01480	285	G	T	synonymous
292502	NTHI477_RS01480	291	C	T	synonymous
292511	NTHI477_RS01480	300	T	G	synonymous
292569	NTHI477_RS01480	358	C	T	nonsynonymous
292586	NTHI477_RS01480	375	T	C	synonymous
292608	NTHI477_RS01480	397	G	A	nonsynonymous
292623	NTHI477_RS01480	412	G	A	nonsynonymous
292643	NTHI477_RS01480	432	T	C	synonymous
292670	NTHI477_RS01480	459	G	A	synonymous
292708	NTHI477_RS01480	497	C	T	nonsynonymous
292709	NTHI477_RS01480	498	A	C	synonymous
292731	NTHI477_RS01480	520	A	G	nonsynonymous
292755	NTHI477_RS01480	544	G	A	nonsynonymous
292767	NTHI477_RS01480	556	C	G	nonsynonymous
292772	NTHI477_RS01480	561	C	T	synonymous
292794	NTHI477_RS01480	583	G	T	nonsynonymous
292805	NTHI477_RS01480	594	G	A	synonymous
292826	NTHI477_RS01480	615	T	G	synonymous
292838	NTHI477_RS01480	627	C	T	synonymous
292847	NTHI477_RS01480	636	C	A	nonsynonymous
292859	NTHI477_RS01480	648	C	A	synonymous
292865	NTHI477_RS01480	654	G	T	synonymous

292865	NTHI477_RS01480	654	G	A	synonymous
292868	NTHI477_RS01480	657	A	G	synonymous
292871	NTHI477_RS01480	660	T	C	synonymous
292872	NTHI477_RS01480	661	A	G	nonsynonymous
292905	NTHI477_RS01480	694	G	A	nonsynonymous
292911	NTHI477_RS01480	700	C	T	nonsynonymous
292913	NTHI477_RS01480	702	C	G	synonymous
292913	NTHI477_RS01480	702	C	A	synonymous
292945	NTHI477_RS01480	734	G	A	nonsynonymous
292949	NTHI477_RS01480	738	A	C	nonsynonymous
292964	NTHI477_RS01480	753	C	A	synonymous
292967	NTHI477_RS01480	756	G	A	synonymous
292973	NTHI477_RS01480	762	T	C	synonymous
292977	NTHI477_RS01480	766	A	T	nonsynonymous
292982	NTHI477_RS01480	771	G	A	synonymous
292994	NTHI477_RS01480	783	A	G	synonymous
293015	NTHI477_RS01480	804	T	C	synonymous
293019	NTHI477_RS01480	808	G	A	nonsynonymous
293030	NTHI477_RS01480	819	C	T	synonymous
293042	NTHI477_RS01480	831	A	G	synonymous
293054	NTHI477_RS01480	843	A	G	synonymous
293079	NTHI477_RS01480	868	G	A	nonsynonymous
293081	NTHI477_RS01480	870	A	C	synonymous
293084	NTHI477_RS01480	873	G	A	synonymous
293090	NTHI477_RS01480	879	G	A	synonymous
293091	NTHI477_RS01480	880	T	A	nonsynonymous
293092	NTHI477_RS01480	881	G	A	nonsynonymous
293106	NTHI477_RS01480	895	G	C	nonsynonymous
293112	NTHI477_RS01480	901	C	T	nonsynonymous
293113	NTHI477_RS01480	902	A	C	nonsynonymous
293122	NTHI477_RS01480	911	A	G	nonsynonymous
293124	NTHI477_RS01480	913	A	C	nonsynonymous
293125	NTHI477_RS01480	914	T	A	nonsynonymous
293126	NTHI477_RS01480	915	T	C	synonymous
293131	intergenic	intergenic	T	C	intergenic
293132	intergenic	intergenic	G	A	intergenic
293134	intergenic	intergenic	T	C	intergenic
293157	intergenic	intergenic	A	G	intergenic
293158	intergenic	intergenic	G	A	intergenic
293159	intergenic	intergenic	C	T	intergenic
293163	intergenic	intergenic	C	T	intergenic
293165	intergenic	intergenic	A	C	intergenic
293184	intergenic	intergenic	G	A	intergenic
293211	NTHI477_RS01485	1407	A	G	synonymous
293220	NTHI477_RS01485	1398	G	A	synonymous

293241	NTHI477_RS01485	1377	G	T	synonymous
293256	NTHI477_RS01485	1362	C	A	nonsynonymous
293274	NTHI477_RS01485	1344	G	A	synonymous
293289	NTHI477_RS01485	1329	G	A	synonymous
293292	NTHI477_RS01485	1326	A	C	synonymous
293298	NTHI477_RS01485	1320	T	A	synonymous
293319	NTHI477_RS01485	1299	T	A	synonymous
293331	NTHI477_RS01485	1287	T	C	synonymous
293346	NTHI477_RS01485	1272	C	T	synonymous
293352	NTHI477_RS01485	1266	T	A	synonymous
293361	NTHI477_RS01485	1257	C	T	synonymous
293370	NTHI477_RS01485	1248	G	A	synonymous
293391	NTHI477_RS01485	1227	C	A	synonymous
293403	NTHI477_RS01485	1215	C	T	synonymous
293418	NTHI477_RS01485	1200	T	G	synonymous
293421	NTHI477_RS01485	1197	A	G	synonymous
293436	NTHI477_RS01485	1182	G	A	synonymous
293445	NTHI477_RS01485	1173	G	A	synonymous
293451	NTHI477_RS01485	1167	A	G	synonymous
293454	NTHI477_RS01485	1164	A	G	synonymous
293469	NTHI477_RS01485	1149	T	C	synonymous
293478	NTHI477_RS01485	1140	A	C	synonymous
293478	NTHI477_RS01485	1140	A	G	synonymous
293490	NTHI477_RS01485	1128	T	C	synonymous
293496	NTHI477_RS01485	1122	C	T	synonymous
293502	NTHI477_RS01485	1116	A	G	synonymous
293508	NTHI477_RS01485	1110	A	C	synonymous
293520	NTHI477_RS01485	1098	G	A	synonymous
293523	NTHI477_RS01485	1095	G	A	synonymous
293526	NTHI477_RS01485	1092	C	G	synonymous
293526	NTHI477_RS01485	1092	C	A	synonymous
293547	NTHI477_RS01485	1071	G	A	synonymous
293625	NTHI477_RS01485	993	A	C	synonymous
293643	NTHI477_RS01485	975	C	T	synonymous
293655	NTHI477_RS01485	963	C	T	synonymous
293666	NTHI477_RS01485	952	C	T	nonsynonymous
293679	NTHI477_RS01485	939	T	C	synonymous
293685	NTHI477_RS01485	933	C	T	synonymous
293694	NTHI477_RS01485	924	C	T	synonymous
293697	NTHI477_RS01485	921	T	G	synonymous
293697	NTHI477_RS01485	921	T	C	synonymous
293700	NTHI477_RS01485	918	A	G	synonymous
293749	NTHI477_RS01485	869	T	C	nonsynonymous
293763	NTHI477_RS01485	855	A	C	synonymous
293763	NTHI477_RS01485	855	A	T	synonymous

293769	NTHI477_RS01485	849	G	T	synonymous
293769	NTHI477_RS01485	849	G	C	synonymous
293787	NTHI477_RS01485	831	G	A	synonymous
293793	NTHI477_RS01485	825	C	T	synonymous
293808	NTHI477_RS01485	810	G	A	synonymous
293814	NTHI477_RS01485	804	A	C	synonymous
293817	NTHI477_RS01485	801	A	G	synonymous
293841	NTHI477_RS01485	777	C	T	synonymous
293844	NTHI477_RS01485	774	A	G	synonymous
293847	NTHI477_RS01485	771	T	A	synonymous
293850	NTHI477_RS01485	768	A	T	synonymous
293856	NTHI477_RS01485	762	G	C	synonymous
293856	NTHI477_RS01485	762	G	A	synonymous
293859	NTHI477_RS01485	759	C	T	synonymous
293862	NTHI477_RS01485	756	A	G	synonymous
293886	NTHI477_RS01485	732	T	C	synonymous
293889	NTHI477_RS01485	729	A	C	synonymous
293889	NTHI477_RS01485	729	A	T	synonymous
293892	NTHI477_RS01485	726	T	C	synonymous
293895	NTHI477_RS01485	723	T	A	synonymous
293904	NTHI477_RS01485	714	A	T	synonymous
293910	NTHI477_RS01485	708	T	A	synonymous
293928	NTHI477_RS01485	690	C	T	synonymous
293943	NTHI477_RS01485	675	T	A	synonymous
293946	NTHI477_RS01485	672	C	T	synonymous
293952	NTHI477_RS01485	666	G	A	synonymous
293958	NTHI477_RS01485	660	T	A	synonymous
293985	NTHI477_RS01485	633	C	A	synonymous
294018	NTHI477_RS01485	600	G	A	synonymous
294126	NTHI477_RS01485	492	T	C	synonymous
294132	NTHI477_RS01485	486	T	C	synonymous
294147	NTHI477_RS01485	471	G	A	synonymous
294168	NTHI477_RS01485	450	G	T	synonymous
294168	NTHI477_RS01485	450	G	C	synonymous
294171	NTHI477_RS01485	447	T	C	synonymous
294171	NTHI477_RS01485	447	T	G	synonymous
294195	NTHI477_RS01485	423	T	G	synonymous
294201	NTHI477_RS01485	417	G	A	synonymous
294225	NTHI477_RS01485	393	T	C	synonymous
294226	NTHI477_RS01485	392	G	A	nonsynonymous
294231	NTHI477_RS01485	387	T	C	synonymous
294234	NTHI477_RS01485	384	A	T	synonymous
294239	NTHI477_RS01485	379	C	T	nonsynonymous
294267	NTHI477_RS01485	351	C	T	synonymous
294273	NTHI477_RS01485	345	T	A	synonymous

294282	NTHI477_RS01485	336	T	C	synonymous
294288	NTHI477_RS01485	330	A	G	synonymous
294315	NTHI477_RS01485	303	A	T	synonymous
294327	NTHI477_RS01485	291	C	T	synonymous
294336	NTHI477_RS01485	282	A	G	synonymous
294339	NTHI477_RS01485	279	G	A	synonymous
294360	NTHI477_RS01485	258	A	C	synonymous
294412	NTHI477_RS01485	206	A	G	nonsynonymous
294414	NTHI477_RS01485	204	T	A	synonymous
294420	NTHI477_RS01485	198	A	G	synonymous
294429	NTHI477_RS01485	189	C	T	synonymous
294431	NTHI477_RS01485	187	C	A	nonsynonymous
294465	NTHI477_RS01485	153	G	T	synonymous
294474	NTHI477_RS01485	144	G	T	synonymous
294474	NTHI477_RS01485	144	G	A	synonymous
294492	NTHI477_RS01485	126	G	T	synonymous
294510	NTHI477_RS01485	108	G	A	synonymous
294522	NTHI477_RS01485	96	T	C	synonymous
294588	NTHI477_RS01485	30	A	G	synonymous
294591	NTHI477_RS01485	27	C	T	synonymous
294747	NTHI477_RS01490	1420	G	A	nonsynonymous
294766	NTHI477_RS01490	1401	T	C	synonymous
294769	NTHI477_RS01490	1398	A	G	synonymous
294805	NTHI477_RS01490	1362	T	C	synonymous
294859	NTHI477_RS01490	1308	T	C	synonymous
294907	NTHI477_RS01490	1260	G	T	synonymous
294910	NTHI477_RS01490	1257	C	T	synonymous
294921	NTHI477_RS01490	1246	C	A	nonsynonymous
294940	NTHI477_RS01490	1227	G	A	synonymous
294949	NTHI477_RS01490	1218	A	T	synonymous
294970	NTHI477_RS01490	1197	A	G	synonymous
294991	NTHI477_RS01490	1176	C	T	synonymous
295000	NTHI477_RS01490	1167	C	T	synonymous
295090	NTHI477_RS01490	1077	A	G	synonymous
295093	NTHI477_RS01490	1074	T	C	synonymous
295129	NTHI477_RS01490	1038	G	A	synonymous
295150	NTHI477_RS01490	1017	A	G	synonymous
295198	NTHI477_RS01490	969	A	T	synonymous
295207	NTHI477_RS01490	960	C	T	synonymous
295222	NTHI477_RS01490	945	G	A	synonymous
295282	NTHI477_RS01490	885	T	C	synonymous
295303	NTHI477_RS01490	864	A	T	synonymous
295312	NTHI477_RS01490	855	T	C	synonymous
295312	NTHI477_RS01490	855	T	G	synonymous
295321	NTHI477_RS01490	846	C	T	synonymous

295330	NTHI477_RS01490	837	C	A	synonymous
295351	NTHI477_RS01490	816	C	T	synonymous
295390	NTHI477_RS01490	777	C	T	synonymous
295399	NTHI477_RS01490	768	T	C	synonymous
295441	NTHI477_RS01490	726	A	G	synonymous
295453	NTHI477_RS01490	714	C	T	synonymous
295492	NTHI477_RS01490	675	A	G	synonymous
295498	NTHI477_RS01490	669	G	A	synonymous
295507	NTHI477_RS01490	660	A	G	synonymous
295513	NTHI477_RS01490	654	T	C	synonymous
295540	NTHI477_RS01490	627	C	A	synonymous
295543	NTHI477_RS01490	624	G	A	synonymous
295552	NTHI477_RS01490	615	C	T	synonymous
295618	NTHI477_RS01490	549	T	C	synonymous
295642	NTHI477_RS01490	525	C	T	synonymous
295648	NTHI477_RS01490	519	C	A	synonymous
295657	NTHI477_RS01490	510	A	T	synonymous
295690	NTHI477_RS01490	477	A	T	synonymous
295720	NTHI477_RS01490	447	G	A	synonymous
295729	NTHI477_RS01490	438	C	T	synonymous
295735	NTHI477_RS01490	432	T	C	synonymous
295738	NTHI477_RS01490	429	A	T	synonymous
295741	NTHI477_RS01490	426	G	T	synonymous
295780	NTHI477_RS01490	387	C	T	synonymous
295816	NTHI477_RS01490	351	C	T	synonymous
295825	NTHI477_RS01490	342	T	G	synonymous
295843	NTHI477_RS01490	324	C	T	synonymous
295849	NTHI477_RS01490	318	G	A	synonymous
295849	NTHI477_RS01490	318	G	T	synonymous
295860	NTHI477_RS01490	307	T	C	nonsynonymous
295882	NTHI477_RS01490	285	G	A	synonymous
295900	NTHI477_RS01490	267	A	G	synonymous
295903	NTHI477_RS01490	264	C	A	synonymous
295909	NTHI477_RS01490	258	G	C	synonymous
295909	NTHI477_RS01490	258	G	T	synonymous
295992	NTHI477_RS01490	175	T	A	nonsynonymous
296008	NTHI477_RS01490	159	C	T	synonymous
296011	NTHI477_RS01490	156	G	C	nonsynonymous
296095	NTHI477_RS01490	72	C	T	synonymous
296126	NTHI477_RS01490	41	C	T	nonsynonymous
296131	NTHI477_RS01490	36	A	G	synonymous
296196	intergenic	intergenic	A	G	intergenic
296201	intergenic	intergenic	A	G	intergenic
296216	intergenic	intergenic	C	T	intergenic
296221	intergenic	intergenic	C	T	intergenic

296364	intergenic	intergenic	T	G	intergenic
296388	intergenic	intergenic	A	G	intergenic
296412	intergenic	intergenic	T	C	intergenic
296434	NTHI477_RS01495	2459	T	C	nonsynonymous
296441	NTHI477_RS01495	2452	G	A	nonsynonymous
296490	NTHI477_RS01495	2403	A	G	synonymous
296496	NTHI477_RS01495	2397	C	T	synonymous
296532	NTHI477_RS01495	2361	G	A	synonymous
296559	NTHI477_RS01495	2334	G	A	synonymous
296584	NTHI477_RS01495	2309	G	A	nonsynonymous
296598	NTHI477_RS01495	2295	A	G	synonymous
296631	NTHI477_RS01495	2262	G	A	synonymous
296651	NTHI477_RS01495	2242	A	G	synonymous
296658	NTHI477_RS01495	2235	A	G	synonymous
296664	NTHI477_RS01495	2229	T	C	synonymous
296669	NTHI477_RS01495	2224	G	C	nonsynonymous
296670	NTHI477_RS01495	2223	A	T	nonsynonymous
296676	NTHI477_RS01495	2217	G	T	synonymous
296679	NTHI477_RS01495	2214	T	C	synonymous
296691	NTHI477_RS01495	2202	C	T	synonymous
296709	NTHI477_RS01495	2184	G	A	synonymous
296730	NTHI477_RS01495	2163	G	A	synonymous
296733	NTHI477_RS01495	2160	T	C	synonymous
296736	NTHI477_RS01495	2157	A	G	synonymous
296751	NTHI477_RS01495	2142	T	A	synonymous
296754	NTHI477_RS01495	2139	A	G	synonymous
296757	NTHI477_RS01495	2136	C	G	synonymous
296757	NTHI477_RS01495	2136	C	T	synonymous
296760	NTHI477_RS01495	2133	T	G	nonsynonymous
296763	NTHI477_RS01495	2130	T	G	synonymous
296775	NTHI477_RS01495	2118	T	C	synonymous
296778	NTHI477_RS01495	2115	A	C	synonymous
296787	NTHI477_RS01495	2106	C	T	synonymous
296790	NTHI477_RS01495	2103	T	A	synonymous
296790	NTHI477_RS01495	2103	T	C	synonymous
296793	NTHI477_RS01495	2100	G	A	synonymous
296796	NTHI477_RS01495	2097	A	G	synonymous
296802	NTHI477_RS01495	2091	A	G	synonymous
296805	NTHI477_RS01495	2088	A	G	synonymous
296808	NTHI477_RS01495	2085	G	A	synonymous
296813	NTHI477_RS01495	2080	T	G	nonsynonymous
296816	NTHI477_RS01495	2077	C	T	nonsynonymous
296820	NTHI477_RS01495	2073	A	G	synonymous
296820	NTHI477_RS01495	2073	A	T	synonymous
296826	NTHI477_RS01495	2067	G	A	synonymous

296841	NTHI477_RS01495	2052	T	C	synonymous
296844	NTHI477_RS01495	2049	G	A	synonymous
296847	NTHI477_RS01495	2046	C	T	synonymous
296853	NTHI477_RS01495	2040	G	A	synonymous
296877	NTHI477_RS01495	2016	T	G	synonymous
296877	NTHI477_RS01495	2016	T	A	synonymous
296880	NTHI477_RS01495	2013	A	G	synonymous
296883	NTHI477_RS01495	2010	T	A	nonsynonymous
296885	NTHI477_RS01495	2008	A	G	synonymous
296892	NTHI477_RS01495	2001	T	C	synonymous
296904	NTHI477_RS01495	1989	G	A	synonymous
296907	NTHI477_RS01495	1986	G	T	synonymous
296910	NTHI477_RS01495	1983	T	A	synonymous
296913	NTHI477_RS01495	1980	T	A	synonymous
296916	NTHI477_RS01495	1977	T	C	synonymous
296919	NTHI477_RS01495	1974	A	T	synonymous
296922	NTHI477_RS01495	1971	A	G	synonymous
296925	NTHI477_RS01495	1968	A	T	synonymous
296925	NTHI477_RS01495	1968	A	G	synonymous
296934	NTHI477_RS01495	1959	T	G	synonymous
296943	NTHI477_RS01495	1950	A	T	synonymous
296958	NTHI477_RS01495	1935	A	T	synonymous
296961	NTHI477_RS01495	1932	A	G	synonymous
296964	NTHI477_RS01495	1929	A	G	synonymous
296967	NTHI477_RS01495	1926	C	A	nonsynonymous
296969	NTHI477_RS01495	1924	A	G	synonymous
296973	NTHI477_RS01495	1920	C	T	synonymous
296982	NTHI477_RS01495	1911	T	C	synonymous
296991	NTHI477_RS01495	1902	A	G	synonymous
296994	NTHI477_RS01495	1899	T	A	synonymous
296997	NTHI477_RS01495	1896	A	G	synonymous
297003	NTHI477_RS01495	1890	T	C	synonymous
297006	NTHI477_RS01495	1887	A	T	synonymous
297012	NTHI477_RS01495	1881	T	C	synonymous
297015	NTHI477_RS01495	1878	A	G	synonymous
297019	NTHI477_RS01495	1874	C	T	nonsynonymous
297030	NTHI477_RS01495	1863	C	T	synonymous
297036	NTHI477_RS01495	1857	A	G	synonymous
297039	NTHI477_RS01495	1854	A	G	synonymous
297045	NTHI477_RS01495	1848	G	T	synonymous
297051	NTHI477_RS01495	1842	A	T	synonymous
297054	NTHI477_RS01495	1839	A	C	synonymous
297063	NTHI477_RS01495	1830	A	G	synonymous
297069	NTHI477_RS01495	1824	A	G	synonymous
297072	NTHI477_RS01495	1821	G	A	synonymous

297075	NTHI477_RS01495	1818	C	G	synonymous
297087	NTHI477_RS01495	1806	A	C	synonymous
297087	NTHI477_RS01495	1806	A	T	synonymous
297093	NTHI477_RS01495	1800	G	A	synonymous
297099	NTHI477_RS01495	1794	C	T	synonymous
297105	NTHI477_RS01495	1788	T	C	synonymous
297128	NTHI477_RS01495	1765	C	T	nonsynonymous
297129	NTHI477_RS01495	1764	G	A	synonymous
297132	NTHI477_RS01495	1761	C	A	synonymous
297154	NTHI477_RS01495	1739	C	T	nonsynonymous
297157	NTHI477_RS01495	1736	G	A	nonsynonymous
297158	NTHI477_RS01495	1735	C	T	nonsynonymous
297175	NTHI477_RS01495	1718	C	T	nonsynonymous
297180	NTHI477_RS01495	1713	G	A	synonymous
297189	NTHI477_RS01495	1704	T	C	synonymous
297192	NTHI477_RS01495	1701	T	C	synonymous
297198	NTHI477_RS01495	1695	C	T	synonymous
297213	NTHI477_RS01495	1680	A	G	synonymous
297231	NTHI477_RS01495	1662	T	G	synonymous
297237	NTHI477_RS01495	1656	C	A	synonymous
297240	NTHI477_RS01495	1653	G	A	synonymous
297249	NTHI477_RS01495	1644	G	A	synonymous
297249	NTHI477_RS01495	1644	G	C	synonymous
297252	NTHI477_RS01495	1641	G	A	synonymous
297254	NTHI477_RS01495	1639	G	A	nonsynonymous
297255	NTHI477_RS01495	1638	C	T	synonymous
297258	NTHI477_RS01495	1635	A	G	synonymous
297264	NTHI477_RS01495	1629	C	T	synonymous
297267	NTHI477_RS01495	1626	T	A	synonymous
297270	NTHI477_RS01495	1623	C	A	synonymous
297276	NTHI477_RS01495	1617	C	T	synonymous
297279	NTHI477_RS01495	1614	G	A	synonymous
297280	NTHI477_RS01495	1613	C	G	nonsynonymous
297280	NTHI477_RS01495	1613	C	T	nonsynonymous
297281	NTHI477_RS01495	1612	G	A	nonsynonymous
297285	NTHI477_RS01495	1608	T	C	synonymous
297291	NTHI477_RS01495	1602	T	C	synonymous
297297	NTHI477_RS01495	1596	T	C	synonymous
297309	NTHI477_RS01495	1584	A	G	synonymous
297312	NTHI477_RS01495	1581	A	G	synonymous
297312	NTHI477_RS01495	1581	A	C	synonymous
297324	NTHI477_RS01495	1569	G	A	synonymous
297327	NTHI477_RS01495	1566	A	T	synonymous
297330	NTHI477_RS01495	1563	A	G	synonymous
297332	NTHI477_RS01495	1561	C	T	nonsynonymous

297333	NTHI477_RS01495	1560	A	C	synonymous
297336	NTHI477_RS01495	1557	C	T	synonymous
297357	NTHI477_RS01495	1536	C	T	synonymous
297359	NTHI477_RS01495	1534	T	C	nonsynonymous
297360	NTHI477_RS01495	1533	A	G	synonymous
297363	NTHI477_RS01495	1530	T	C	synonymous
297364	NTHI477_RS01495	1529	G	A	nonsynonymous
297369	NTHI477_RS01495	1524	A	G	synonymous
297376	NTHI477_RS01495	1517	T	G	nonsynonymous
297384	NTHI477_RS01495	1509	A	G	synonymous
297393	NTHI477_RS01495	1500	A	G	synonymous
297408	NTHI477_RS01495	1485	A	G	synonymous
297411	NTHI477_RS01495	1482	G	A	synonymous
297414	NTHI477_RS01495	1479	A	G	synonymous
297423	NTHI477_RS01495	1470	A	G	synonymous
297426	NTHI477_RS01495	1467	C	T	synonymous
297429	NTHI477_RS01495	1464	T	G	synonymous
297430	NTHI477_RS01495	1463	G	T	nonsynonymous
297432	NTHI477_RS01495	1461	G	T	synonymous
297443	NTHI477_RS01495	1450	G	A	nonsynonymous
297444	NTHI477_RS01495	1449	G	A	synonymous
297447	NTHI477_RS01495	1446	C	T	synonymous
297450	NTHI477_RS01495	1443	C	A	synonymous
297453	NTHI477_RS01495	1440	G	C	synonymous
297468	NTHI477_RS01495	1425	C	T	synonymous
297468	NTHI477_RS01495	1425	C	A	synonymous
297474	NTHI477_RS01495	1419	G	A	synonymous
297477	NTHI477_RS01495	1416	A	T	synonymous
297489	NTHI477_RS01495	1404	G	A	synonymous
297495	NTHI477_RS01495	1398	A	G	synonymous
297498	NTHI477_RS01495	1395	G	A	synonymous
297513	NTHI477_RS01495	1380	A	T	synonymous
297513	NTHI477_RS01495	1380	A	G	synonymous
297519	NTHI477_RS01495	1374	A	G	synonymous
297528	NTHI477_RS01495	1365	A	G	synonymous
297531	NTHI477_RS01495	1362	G	A	synonymous
297534	NTHI477_RS01495	1359	T	G	synonymous
297537	NTHI477_RS01495	1356	A	T	synonymous
297540	NTHI477_RS01495	1353	T	C	synonymous
297552	NTHI477_RS01495	1341	G	A	synonymous
297558	NTHI477_RS01495	1335	A	G	synonymous
297564	NTHI477_RS01495	1329	G	A	synonymous
297573	NTHI477_RS01495	1320	G	A	synonymous
297591	NTHI477_RS01495	1302	T	A	synonymous
297594	NTHI477_RS01495	1299	T	C	synonymous

297594	NTHI477_RS01495	1299	T	A	synonymous
297597	NTHI477_RS01495	1296	G	A	synonymous
297600	NTHI477_RS01495	1293	T	C	synonymous
297602	NTHI477_RS01495	1291	G	A	synonymous
297606	NTHI477_RS01495	1287	T	G	synonymous
297621	NTHI477_RS01495	1272	G	A	synonymous
297665	NTHI477_RS01495	1228	C	T	nonsynonymous
297669	NTHI477_RS01495	1224	G	A	synonymous
297675	NTHI477_RS01495	1218	C	T	synonymous
297678	NTHI477_RS01495	1215	T	C	synonymous
297699	NTHI477_RS01495	1194	T	C	synonymous
297705	NTHI477_RS01495	1188	G	A	synonymous
297711	NTHI477_RS01495	1182	G	A	synonymous
297714	NTHI477_RS01495	1179	A	G	synonymous
297741	NTHI477_RS01495	1152	T	C	synonymous
297744	NTHI477_RS01495	1149	C	T	synonymous
297753	NTHI477_RS01495	1140	T	C	synonymous
297768	NTHI477_RS01495	1125	A	T	synonymous
297783	NTHI477_RS01495	1110	C	T	synonymous
297789	NTHI477_RS01495	1104	G	T	synonymous
297810	NTHI477_RS01495	1083	G	A	synonymous
297822	NTHI477_RS01495	1071	G	A	synonymous
297840	NTHI477_RS01495	1053	C	A	synonymous
297852	NTHI477_RS01495	1041	C	T	synonymous
297855	NTHI477_RS01495	1038	G	A	synonymous
297867	NTHI477_RS01495	1026	G	A	synonymous
297897	NTHI477_RS01495	996	C	T	synonymous
297903	NTHI477_RS01495	990	C	A	synonymous
297918	NTHI477_RS01495	975	A	G	synonymous
297921	NTHI477_RS01495	972	T	C	synonymous
297924	NTHI477_RS01495	969	G	A	synonymous
297927	NTHI477_RS01495	966	G	A	synonymous
297960	NTHI477_RS01495	933	G	A	synonymous
297962	NTHI477_RS01495	931	C	T	nonsynonymous
297999	NTHI477_RS01495	894	C	T	synonymous
298008	NTHI477_RS01495	885	G	C	synonymous
298035	NTHI477_RS01495	858	C	T	synonymous
298041	NTHI477_RS01495	852	G	A	synonymous
298044	NTHI477_RS01495	849	A	C	synonymous
298047	NTHI477_RS01495	846	A	G	synonymous
298053	NTHI477_RS01495	840	A	T	synonymous
298053	NTHI477_RS01495	840	A	C	synonymous
298065	NTHI477_RS01495	828	A	T	synonymous
298068	NTHI477_RS01495	825	G	A	synonymous
298074	NTHI477_RS01495	819	T	C	synonymous

298077	NTHI477_RS01495	816	A	T	synonymous
298077	NTHI477_RS01495	816	A	G	synonymous
298080	NTHI477_RS01495	813	T	A	synonymous
298086	NTHI477_RS01495	807	A	G	synonymous
298095	NTHI477_RS01495	798	T	C	synonymous
298098	NTHI477_RS01495	795	G	A	synonymous
298101	NTHI477_RS01495	792	G	A	synonymous
298110	NTHI477_RS01495	783	T	C	synonymous
298113	NTHI477_RS01495	780	T	C	synonymous
298143	NTHI477_RS01495	750	G	A	synonymous
298149	NTHI477_RS01495	744	G	A	synonymous
298191	NTHI477_RS01495	702	T	C	synonymous
298194	NTHI477_RS01495	699	T	A	synonymous
298215	NTHI477_RS01495	678	T	C	synonymous
298218	NTHI477_RS01495	675	G	A	synonymous
298227	NTHI477_RS01495	666	A	G	synonymous
298245	NTHI477_RS01495	648	T	C	synonymous
298269	NTHI477_RS01495	624	A	G	synonymous
298278	NTHI477_RS01495	615	G	A	synonymous
298281	NTHI477_RS01495	612	T	C	synonymous
298284	NTHI477_RS01495	609	G	A	synonymous
298287	NTHI477_RS01495	606	A	G	synonymous
298296	NTHI477_RS01495	597	T	A	synonymous
298299	NTHI477_RS01495	594	A	G	synonymous
298326	NTHI477_RS01495	567	T	G	synonymous
298329	NTHI477_RS01495	564	A	G	synonymous
298335	NTHI477_RS01495	558	A	G	synonymous
298338	NTHI477_RS01495	555	T	C	synonymous
298344	NTHI477_RS01495	549	A	C	synonymous
298347	NTHI477_RS01495	546	T	C	synonymous
298350	NTHI477_RS01495	543	T	A	synonymous
298350	NTHI477_RS01495	543	T	C	synonymous
298365	NTHI477_RS01495	528	T	C	synonymous
298371	NTHI477_RS01495	522	A	T	synonymous
298374	NTHI477_RS01495	519	A	G	synonymous
298377	NTHI477_RS01495	516	C	T	synonymous
298380	NTHI477_RS01495	513	G	T	synonymous
298392	NTHI477_RS01495	501	A	G	synonymous
298398	NTHI477_RS01495	495	A	G	synonymous
298440	NTHI477_RS01495	453	G	A	synonymous
298449	NTHI477_RS01495	444	C	T	synonymous
298455	NTHI477_RS01495	438	A	G	synonymous
298461	NTHI477_RS01495	432	G	C	synonymous
298461	NTHI477_RS01495	432	G	A	synonymous
298464	NTHI477_RS01495	429	C	T	synonymous

298467	NTHI477_RS01495	426	A	G	synonymous
298479	NTHI477_RS01495	414	A	G	synonymous
298482	NTHI477_RS01495	411	A	G	synonymous
298485	NTHI477_RS01495	408	A	C	synonymous
298488	NTHI477_RS01495	405	G	C	synonymous
298488	NTHI477_RS01495	405	G	T	synonymous
298497	NTHI477_RS01495	396	A	G	synonymous
298506	NTHI477_RS01495	387	A	G	synonymous
298512	NTHI477_RS01495	381	G	A	synonymous
298521	NTHI477_RS01495	372	A	T	synonymous
298527	NTHI477_RS01495	366	T	A	synonymous
298527	NTHI477_RS01495	366	T	G	synonymous
298529	NTHI477_RS01495	364	C	T	nonsynonymous
298539	NTHI477_RS01495	354	T	C	synonymous
298542	NTHI477_RS01495	351	A	C	synonymous
298545	NTHI477_RS01495	348	T	C	synonymous
298575	NTHI477_RS01495	318	T	A	synonymous
298581	NTHI477_RS01495	312	C	T	synonymous
298587	NTHI477_RS01495	306	T	C	synonymous
298607	NTHI477_RS01495	286	C	T	nonsynonymous
298614	NTHI477_RS01495	279	T	G	synonymous
298614	NTHI477_RS01495	279	T	C	synonymous
298623	NTHI477_RS01495	270	T	A	synonymous
298626	NTHI477_RS01495	267	C	G	synonymous
298632	NTHI477_RS01495	261	C	T	synonymous
298635	NTHI477_RS01495	258	G	A	synonymous
298641	NTHI477_RS01495	252	A	G	synonymous
298656	NTHI477_RS01495	237	G	A	synonymous
298682	NTHI477_RS01495	211	C	A	nonsynonymous
298698	NTHI477_RS01495	195	A	T	synonymous
298701	NTHI477_RS01495	192	A	G	synonymous
298704	NTHI477_RS01495	189	G	A	synonymous
298710	NTHI477_RS01495	183	A	C	synonymous
298713	NTHI477_RS01495	180	C	T	synonymous
298719	NTHI477_RS01495	174	T	C	synonymous
298728	NTHI477_RS01495	165	A	T	synonymous
298731	NTHI477_RS01495	162	C	A	synonymous
298737	NTHI477_RS01495	156	G	A	synonymous
298740	NTHI477_RS01495	153	A	G	synonymous
298740	NTHI477_RS01495	153	A	C	synonymous
298742	NTHI477_RS01495	151	G	A	nonsynonymous
298743	NTHI477_RS01495	150	T	A	synonymous
298746	NTHI477_RS01495	147	T	C	synonymous
298752	NTHI477_RS01495	141	G	T	synonymous
298754	NTHI477_RS01495	139	G	A	nonsynonymous

298758	NTHI477_RS01495	135	A	G	synonymous
298761	NTHI477_RS01495	132	G	C	synonymous
298770	NTHI477_RS01495	123	G	A	synonymous
298785	NTHI477_RS01495	108	G	A	synonymous
298803	NTHI477_RS01495	90	A	G	synonymous
298878	NTHI477_RS01495	15	G	A	synonymous
298924	intergenic	intergenic	G	A	intergenic
298938	intergenic	intergenic	G	A	intergenic
298940	intergenic	intergenic	G	A	intergenic
298948	intergenic	intergenic	T	G	intergenic
298949	intergenic	intergenic	C	T	intergenic
298957	intergenic	intergenic	A	G	intergenic
298967	intergenic	intergenic	G	A	intergenic
298997	intergenic	intergenic	T	G	intergenic
298999	intergenic	intergenic	A	C	intergenic
299000	intergenic	intergenic	A	G	intergenic
299003	intergenic	intergenic	C	T	intergenic
299004	intergenic	intergenic	G	A	intergenic
299010	intergenic	intergenic	G	A	intergenic
299016	intergenic	intergenic	T	C	intergenic
299018	intergenic	intergenic	C	T	intergenic
299025	intergenic	intergenic	T	G	intergenic
299059	intergenic	intergenic	C	T	intergenic
299071	intergenic	intergenic	A	C	intergenic
299075	intergenic	intergenic	C	T	intergenic
299092	intergenic	intergenic	T	G	intergenic
299109	intergenic	intergenic	T	A	intergenic
299157	NTHI477_RS01500	1413	T	C	synonymous
299178	NTHI477_RS01500	1392	C	T	synonymous
299208	NTHI477_RS01500	1362	A	G	synonymous
299211	NTHI477_RS01500	1359	T	C	synonymous
299211	NTHI477_RS01500	1359	T	G	synonymous
299214	NTHI477_RS01500	1356	A	G	synonymous
299217	NTHI477_RS01500	1353	A	T	synonymous
299219	NTHI477_RS01500	1351	C	T	nonsynonymous
299223	NTHI477_RS01500	1347	C	T	synonymous
299225	NTHI477_RS01500	1345	C	A	nonsynonymous
299225	NTHI477_RS01500	1345	C	T	nonsynonymous
299229	NTHI477_RS01500	1341	T	A	synonymous
299229	NTHI477_RS01500	1341	T	C	synonymous
299232	NTHI477_RS01500	1338	A	T	synonymous
299234	NTHI477_RS01500	1336	G	T	nonsynonymous
299235	NTHI477_RS01500	1335	C	T	synonymous
299236	NTHI477_RS01500	1334	G	A	nonsynonymous
299241	NTHI477_RS01500	1329	T	C	synonymous

299243	NTHI477_RS01500	1327	G	T	nonsynonymous
299247	NTHI477_RS01500	1323	T	A	nonsynonymous
299249	NTHI477_RS01500	1321	A	T	nonsynonymous
299250	NTHI477_RS01500	1320	G	T	synonymous
299252	NTHI477_RS01500	1318	C	A	nonsynonymous
299256	NTHI477_RS01500	1314	G	T	synonymous
299259	NTHI477_RS01500	1311	A	G	synonymous
299260	NTHI477_RS01500	1310	C	T	nonsynonymous
299262	NTHI477_RS01500	1308	T	C	synonymous
299265	NTHI477_RS01500	1305	C	A	nonsynonymous
299267	NTHI477_RS01500	1303	A	G	synonymous
299267	NTHI477_RS01500	1303	A	T	nonsynonymous
299269	NTHI477_RS01500	1301	C	G	nonsynonymous
299270	NTHI477_RS01500	1300	A	T	nonsynonymous
299271	NTHI477_RS01500	1299	A	G	synonymous
299272	NTHI477_RS01500	1298	T	C	nonsynonymous
299277	NTHI477_RS01500	1293	G	T	synonymous
299279	NTHI477_RS01500	1291	G	A	nonsynonymous
299281	NTHI477_RS01500	1289	G	T	nonsynonymous
299283	NTHI477_RS01500	1287	T	A	nonsynonymous
299286	NTHI477_RS01500	1284	A	T	synonymous
299288	NTHI477_RS01500	1282	G	C	nonsynonymous
299289	NTHI477_RS01500	1281	C	A	synonymous
299290	NTHI477_RS01500	1280	G	T	nonsynonymous
299291	NTHI477_RS01500	1279	T	C	nonsynonymous
299292	NTHI477_RS01500	1278	T	A	synonymous
299296	NTHI477_RS01500	1274	C	T	nonsynonymous
299304	NTHI477_RS01500	1266	C	T	synonymous
299334	NTHI477_RS01500	1236	G	A	synonymous
299343	NTHI477_RS01500	1227	A	G	synonymous
299345	NTHI477_RS01500	1225	T	C	nonsynonymous
299352	NTHI477_RS01500	1218	C	T	synonymous
299364	NTHI477_RS01500	1206	C	G	synonymous
299367	NTHI477_RS01500	1203	T	C	synonymous
299376	NTHI477_RS01500	1194	T	C	synonymous
299388	NTHI477_RS01500	1182	A	T	synonymous
299397	NTHI477_RS01500	1173	T	A	synonymous
299442	NTHI477_RS01500	1128	G	A	synonymous
299448	NTHI477_RS01500	1122	A	G	synonymous
299463	NTHI477_RS01500	1107	C	T	synonymous
299468	NTHI477_RS01500	1102	C	T	nonsynonymous
299469	NTHI477_RS01500	1101	G	A	synonymous
299481	NTHI477_RS01500	1089	C	T	nonsynonymous
299483	NTHI477_RS01500	1087	T	A	nonsynonymous
299490	NTHI477_RS01500	1080	C	T	synonymous

299493	NTHI477_RS01500	1077	A	C	synonymous
299495	NTHI477_RS01500	1075	G	T	nonsynonymous
299496	NTHI477_RS01500	1074	G	T	synonymous
299498	NTHI477_RS01500	1072	C	T	nonsynonymous
299504	NTHI477_RS01500	1066	C	T	nonsynonymous
299505	NTHI477_RS01500	1065	G	A	synonymous
299508	NTHI477_RS01500	1062	G	A	synonymous
299511	NTHI477_RS01500	1059	G	T	synonymous
299511	NTHI477_RS01500	1059	G	A	synonymous
299517	NTHI477_RS01500	1053	T	G	synonymous
299520	NTHI477_RS01500	1050	C	T	synonymous
299525	NTHI477_RS01500	1045	T	C	nonsynonymous
299529	NTHI477_RS01500	1041	T	G	nonsynonymous
299531	NTHI477_RS01500	1039	G	C	nonsynonymous
299531	NTHI477_RS01500	1039	G	T	nonsynonymous
299532	NTHI477_RS01500	1038	C	T	synonymous
299532	NTHI477_RS01500	1038	C	A	synonymous
299535	NTHI477_RS01500	1035	A	G	synonymous
299538	NTHI477_RS01500	1032	A	T	synonymous
299539	NTHI477_RS01500	1031	C	T	nonsynonymous
299541	NTHI477_RS01500	1029	T	G	nonsynonymous
299542	NTHI477_RS01500	1028	T	G	nonsynonymous
299542	NTHI477_RS01500	1028	T	A	nonsynonymous
299544	NTHI477_RS01500	1026	A	G	synonymous
299544	NTHI477_RS01500	1026	A	T	synonymous
299568	NTHI477_RS01500	1002	A	C	nonsynonymous
299580	NTHI477_RS01500	990	A	G	synonymous
299580	NTHI477_RS01500	990	A	T	synonymous
299583	NTHI477_RS01500	987	T	A	synonymous
299586	NTHI477_RS01500	984	A	G	synonymous
299604	NTHI477_RS01500	966	A	T	synonymous
299604	NTHI477_RS01500	966	A	C	synonymous
299610	NTHI477_RS01500	960	T	C	synonymous
299625	NTHI477_RS01500	945	G	A	synonymous
299633	NTHI477_RS01500	937	T	A	nonsynonymous
299649	NTHI477_RS01500	921	A	T	synonymous
299649	NTHI477_RS01500	921	A	G	synonymous
299676	NTHI477_RS01500	894	A	G	synonymous
299676	NTHI477_RS01500	894	A	C	synonymous
299689	NTHI477_RS01500	881	G	A	nonsynonymous
299694	NTHI477_RS01500	876	G	A	synonymous
299695	NTHI477_RS01500	875	G	C	nonsynonymous
299697	NTHI477_RS01500	873	A	T	synonymous
299698	NTHI477_RS01500	872	G	A	nonsynonymous
299699	NTHI477_RS01500	871	A	T	nonsynonymous

299702	NTHI477_RS01500	868	C	T	nonsynonymous
299724	NTHI477_RS01500	846	G	C	synonymous
299725	NTHI477_RS01500	845	G	A	nonsynonymous
299727	NTHI477_RS01500	843	T	C	synonymous
299732	NTHI477_RS01500	838	G	A	synonymous
299736	NTHI477_RS01500	834	G	A	synonymous
299742	NTHI477_RS01500	828	G	A	synonymous
299754	NTHI477_RS01500	816	G	A	synonymous
299759	NTHI477_RS01500	811	C	T	nonsynonymous
299769	NTHI477_RS01500	801	G	C	synonymous
299772	NTHI477_RS01500	798	C	T	synonymous
299784	NTHI477_RS01500	786	C	T	synonymous
299787	NTHI477_RS01500	783	A	G	synonymous
299817	NTHI477_RS01500	753	A	G	synonymous
299823	NTHI477_RS01500	747	G	A	synonymous
299826	NTHI477_RS01500	744	C	T	synonymous
299829	NTHI477_RS01500	741	A	G	synonymous
299832	NTHI477_RS01500	738	A	G	synonymous
299838	NTHI477_RS01500	732	A	T	synonymous
299854	NTHI477_RS01500	716	C	T	nonsynonymous
299871	NTHI477_RS01500	699	A	T	synonymous
299877	NTHI477_RS01500	693	C	T	synonymous
299880	NTHI477_RS01500	690	G	A	synonymous
299886	NTHI477_RS01500	684	C	T	synonymous
299889	NTHI477_RS01500	681	A	G	synonymous
299892	NTHI477_RS01500	678	G	A	synonymous
299904	NTHI477_RS01500	666	T	A	synonymous
299910	NTHI477_RS01500	660	C	T	synonymous
299921	NTHI477_RS01500	649	C	G	nonsynonymous
299922	NTHI477_RS01500	648	T	G	synonymous
299934	NTHI477_RS01500	636	G	A	synonymous
299937	NTHI477_RS01500	633	A	T	synonymous
299940	NTHI477_RS01500	630	T	C	synonymous
299940	NTHI477_RS01500	630	T	A	synonymous
299946	NTHI477_RS01500	624	G	A	synonymous
299949	NTHI477_RS01500	621	T	G	synonymous
299955	NTHI477_RS01500	615	A	G	synonymous
299967	NTHI477_RS01500	603	A	G	synonymous
299967	NTHI477_RS01500	603	A	T	synonymous
299976	NTHI477_RS01500	594	C	T	synonymous
299979	NTHI477_RS01500	591	G	A	synonymous
299988	NTHI477_RS01500	582	C	T	synonymous
299991	NTHI477_RS01500	579	C	T	synonymous
299994	NTHI477_RS01500	576	A	G	synonymous
300000	NTHI477_RS01500	570	T	C	synonymous

300006	NTHI477_RS01500	564	G	A	synonymous
300009	NTHI477_RS01500	561	T	G	nonsynonymous
300009	NTHI477_RS01500	561	T	A	nonsynonymous
300012	NTHI477_RS01500	558	T	C	synonymous
300015	NTHI477_RS01500	555	G	A	synonymous
300017	NTHI477_RS01500	553	G	T	nonsynonymous
300018	NTHI477_RS01500	552	G	A	synonymous
300024	NTHI477_RS01500	546	C	T	synonymous
300027	NTHI477_RS01500	543	T	C	synonymous
300029	NTHI477_RS01500	541	T	C	nonsynonymous
300030	NTHI477_RS01500	540	C	T	synonymous
300030	NTHI477_RS01500	540	C	A	synonymous
300036	NTHI477_RS01500	534	G	A	synonymous
300039	NTHI477_RS01500	531	G	A	synonymous
300045	NTHI477_RS01500	525	A	G	synonymous
300048	NTHI477_RS01500	522	C	T	synonymous
300078	NTHI477_RS01500	492	T	C	synonymous
300081	NTHI477_RS01500	489	C	T	synonymous
300087	NTHI477_RS01500	483	G	A	synonymous
300093	NTHI477_RS01500	477	G	A	synonymous
300096	NTHI477_RS01500	474	G	A	synonymous
300099	NTHI477_RS01500	471	C	T	synonymous
300099	NTHI477_RS01500	471	C	G	synonymous
300108	NTHI477_RS01500	462	C	T	synonymous
300111	NTHI477_RS01500	459	A	T	synonymous
300114	NTHI477_RS01500	456	G	T	synonymous
300115	NTHI477_RS01500	455	C	T	nonsynonymous
300116	NTHI477_RS01500	454	G	T	nonsynonymous
300117	NTHI477_RS01500	453	A	G	synonymous
300129	NTHI477_RS01500	441	C	T	synonymous
300129	NTHI477_RS01500	441	C	G	synonymous
300131	NTHI477_RS01500	439	G	A	synonymous
300135	NTHI477_RS01500	435	T	C	synonymous
300135	NTHI477_RS01500	435	T	A	synonymous
300138	NTHI477_RS01500	432	T	C	synonymous
300144	NTHI477_RS01500	426	C	T	synonymous
300144	NTHI477_RS01500	426	C	A	nonsynonymous
300146	NTHI477_RS01500	424	A	G	synonymous
300147	NTHI477_RS01500	423	A	G	synonymous
300147	NTHI477_RS01500	423	A	C	synonymous
300150	NTHI477_RS01500	420	T	A	synonymous
300153	NTHI477_RS01500	417	G	A	synonymous
300159	NTHI477_RS01500	411	A	G	synonymous
300162	NTHI477_RS01500	408	A	G	synonymous
300165	NTHI477_RS01500	405	A	G	synonymous

300168	NTHI477_RS01500	402	G	A	synonymous
300171	NTHI477_RS01500	399	T	C	synonymous
300180	NTHI477_RS01500	390	T	C	synonymous
300193	NTHI477_RS01500	377	C	T	nonsynonymous
300195	NTHI477_RS01500	375	A	G	synonymous
300200	NTHI477_RS01500	370	A	G	synonymous
300204	NTHI477_RS01500	366	T	A	synonymous
300219	NTHI477_RS01500	351	C	G	synonymous
300237	NTHI477_RS01500	333	A	G	synonymous
300267	NTHI477_RS01500	303	G	A	synonymous
300277	NTHI477_RS01500	293	G	A	nonsynonymous
300282	NTHI477_RS01500	288	G	A	synonymous
300297	NTHI477_RS01500	273	T	C	synonymous
300320	NTHI477_RS01500	250	C	A	nonsynonymous
300343	NTHI477_RS01500	227	C	T	nonsynonymous
300345	NTHI477_RS01500	225	A	G	synonymous
300363	NTHI477_RS01500	207	C	T	synonymous
300366	NTHI477_RS01500	204	C	T	synonymous
300378	NTHI477_RS01500	192	A	T	synonymous
300387	NTHI477_RS01500	183	G	A	synonymous
300408	NTHI477_RS01500	162	A	G	synonymous
300413	NTHI477_RS01500	157	T	G	nonsynonymous
300415	NTHI477_RS01500	155	T	G	nonsynonymous
300420	NTHI477_RS01500	150	G	A	synonymous
300421	NTHI477_RS01500	149	C	T	nonsynonymous
300427	NTHI477_RS01500	143	A	G	nonsynonymous
300428	NTHI477_RS01500	142	T	A	nonsynonymous
300432	NTHI477_RS01500	138	T	A	synonymous
300435	NTHI477_RS01500	135	G	T	synonymous
300435	NTHI477_RS01500	135	G	A	synonymous
300456	NTHI477_RS01500	114	G	A	synonymous
300459	NTHI477_RS01500	111	A	G	synonymous
300471	NTHI477_RS01500	99	A	G	synonymous
300483	NTHI477_RS01500	87	T	C	synonymous
300498	NTHI477_RS01500	72	C	T	synonymous
300519	NTHI477_RS01500	51	G	A	synonymous
300523	NTHI477_RS01500	47	G	A	nonsynonymous
300549	NTHI477_RS01500	21	A	G	synonymous
300560	NTHI477_RS01500	10	G	A	synonymous
300561	NTHI477_RS01500	9	T	G	synonymous
300576	intergenic	intergenic	T	G	intergenic
300580	intergenic	intergenic	A	G	intergenic
300589	intergenic	intergenic	A	C	intergenic
300589	intergenic	intergenic	A	G	intergenic
300595	intergenic	intergenic	A	G	intergenic

300597	intergenic	intergenic	G	A	intergenic
300607	intergenic	intergenic	C	A	intergenic
300607	intergenic	intergenic	C	T	intergenic
300614	intergenic	intergenic	C	A	intergenic
300616	intergenic	intergenic	C	T	intergenic
300617	intergenic	intergenic	C	G	intergenic
300619	intergenic	intergenic	C	T	intergenic
300627	intergenic	intergenic	C	A	intergenic
300628	intergenic	intergenic	T	A	intergenic
300637	intergenic	intergenic	A	G	intergenic
300638	intergenic	intergenic	T	G	intergenic
300642	intergenic	intergenic	G	T	intergenic
300653	intergenic	intergenic	T	C	intergenic
300656	intergenic	intergenic	T	A	intergenic
300657	intergenic	intergenic	T	A	intergenic
300667	intergenic	intergenic	C	T	intergenic
300689	NTHI477_RS01505	1290	A	T	synonymous
300689	NTHI477_RS01505	1290	A	C	synonymous
300692	NTHI477_RS01505	1287	T	A	nonsynonymous
300698	NTHI477_RS01505	1281	G	T	synonymous
300699	NTHI477_RS01505	1280	C	T	nonsynonymous
300704	NTHI477_RS01505	1275	C	T	synonymous
300707	NTHI477_RS01505	1272	G	T	nonsynonymous
300720	NTHI477_RS01505	1259	A	T	nonsynonymous
300727	NTHI477_RS01505	1252	G	A	synonymous
300736	NTHI477_RS01505	1243	G	A	nonsynonymous
300740	NTHI477_RS01505	1239	T	C	synonymous
300749	NTHI477_RS01505	1230	G	C	synonymous
300755	NTHI477_RS01505	1224	G	A	synonymous
300779	NTHI477_RS01505	1200	A	T	nonsynonymous
300781	NTHI477_RS01505	1198	T	G	nonsynonymous
300794	NTHI477_RS01505	1185	C	T	nonsynonymous
300795	NTHI477_RS01505	1184	A	C	nonsynonymous
300799	NTHI477_RS01505	1180	C	T	nonsynonymous
300800	NTHI477_RS01505	1179	C	T	synonymous
300830	NTHI477_RS01505	1149	G	A	synonymous
300839	NTHI477_RS01505	1140	G	A	synonymous
300863	NTHI477_RS01505	1116	G	A	synonymous
300872	NTHI477_RS01505	1107	A	G	synonymous
300892	NTHI477_RS01505	1087	A	G	synonymous
300908	NTHI477_RS01505	1071	G	C	synonymous
300920	NTHI477_RS01505	1059	G	T	synonymous
300922	NTHI477_RS01505	1057	A	C	nonsynonymous
300936	NTHI477_RS01505	1043	C	T	nonsynonymous
300938	NTHI477_RS01505	1041	A	G	synonymous

300941	NTHI477_RS01505	1038	A	G	synonymous
300944	NTHI477_RS01505	1035	A	T	synonymous
300946	NTHI477_RS01505	1033	G	A	nonsynonymous
300947	NTHI477_RS01505	1032	C	T	synonymous
300950	NTHI477_RS01505	1029	G	A	synonymous
300965	NTHI477_RS01505	1014	T	C	synonymous
300965	NTHI477_RS01505	1014	T	A	synonymous
300983	NTHI477_RS01505	996	G	A	synonymous
300986	NTHI477_RS01505	993	C	G	synonymous
300989	NTHI477_RS01505	990	G	A	synonymous
300992	NTHI477_RS01505	987	A	G	synonymous
300995	NTHI477_RS01505	984	C	A	nonsynonymous
300997	NTHI477_RS01505	982	A	G	synonymous
301010	NTHI477_RS01505	969	T	A	synonymous
301013	NTHI477_RS01505	966	G	A	synonymous
301016	NTHI477_RS01505	963	C	T	synonymous
301019	NTHI477_RS01505	960	G	A	synonymous
301020	NTHI477_RS01505	959	T	C	nonsynonymous
301022	NTHI477_RS01505	957	A	G	synonymous
301031	NTHI477_RS01505	948	A	G	synonymous
301037	NTHI477_RS01505	942	G	A	synonymous
301043	NTHI477_RS01505	936	A	T	synonymous
301046	NTHI477_RS01505	933	C	T	synonymous
301060	NTHI477_RS01505	919	C	T	nonsynonymous
301061	NTHI477_RS01505	918	C	A	synonymous
301067	NTHI477_RS01505	912	A	G	synonymous
301088	NTHI477_RS01505	891	G	A	synonymous
301097	NTHI477_RS01505	882	A	G	synonymous
301184	NTHI477_RS01505	795	C	T	synonymous
301187	NTHI477_RS01505	792	A	G	synonymous
301190	NTHI477_RS01505	789	G	A	synonymous
301193	NTHI477_RS01505	786	G	A	synonymous
301202	NTHI477_RS01505	777	G	A	synonymous
301214	NTHI477_RS01505	765	A	G	synonymous
301217	NTHI477_RS01505	762	T	A	synonymous
301220	NTHI477_RS01505	759	G	A	synonymous
301223	NTHI477_RS01505	756	A	C	synonymous
301226	NTHI477_RS01505	753	A	G	synonymous
301232	NTHI477_RS01505	747	C	A	synonymous
301232	NTHI477_RS01505	747	C	G	synonymous
301232	NTHI477_RS01505	747	C	T	synonymous
301234	NTHI477_RS01505	745	C	T	nonsynonymous
301235	NTHI477_RS01505	744	G	C	synonymous
301235	NTHI477_RS01505	744	G	T	synonymous
301236	NTHI477_RS01505	743	C	T	nonsynonymous

301241	NTHI477_RS01505	738	T	C	synonymous
301242	NTHI477_RS01505	737	T	G	nonsynonymous
301256	NTHI477_RS01505	723	C	T	synonymous
301259	NTHI477_RS01505	720	T	C	synonymous
301274	NTHI477_RS01505	705	A	G	synonymous
301277	NTHI477_RS01505	702	G	A	synonymous
301283	NTHI477_RS01505	696	G	A	synonymous
301286	NTHI477_RS01505	693	G	T	nonsynonymous
301288	NTHI477_RS01505	691	T	G	nonsynonymous
301289	NTHI477_RS01505	690	C	T	synonymous
301292	NTHI477_RS01505	687	G	A	synonymous
301292	NTHI477_RS01505	687	G	T	synonymous
301295	NTHI477_RS01505	684	G	A	synonymous
301296	NTHI477_RS01505	683	C	T	nonsynonymous
301297	NTHI477_RS01505	682	C	T	nonsynonymous
301298	NTHI477_RS01505	681	A	G	synonymous
301301	NTHI477_RS01505	678	T	C	synonymous
301304	NTHI477_RS01505	675	A	C	synonymous
301304	NTHI477_RS01505	675	A	T	synonymous
301305	NTHI477_RS01505	674	C	T	nonsynonymous
301306	NTHI477_RS01505	673	G	T	nonsynonymous
301307	NTHI477_RS01505	672	A	C	nonsynonymous
301310	NTHI477_RS01505	669	T	A	nonsynonymous
301310	NTHI477_RS01505	669	T	C	synonymous
301312	NTHI477_RS01505	667	A	G	synonymous
301319	NTHI477_RS01505	660	G	A	synonymous
301340	NTHI477_RS01505	639	C	T	synonymous
301355	NTHI477_RS01505	624	T	C	synonymous
301358	NTHI477_RS01505	621	A	G	synonymous
301358	NTHI477_RS01505	621	A	T	synonymous
301360	NTHI477_RS01505	619	T	C	nonsynonymous
301361	NTHI477_RS01505	618	A	G	synonymous
301364	NTHI477_RS01505	615	C	T	synonymous
301379	NTHI477_RS01505	600	A	G	synonymous
301382	NTHI477_RS01505	597	C	T	synonymous
301394	NTHI477_RS01505	585	C	A	synonymous
301403	NTHI477_RS01505	576	T	C	synonymous
301448	NTHI477_RS01505	531	C	T	nonsynonymous
301451	NTHI477_RS01505	528	T	C	synonymous
301454	NTHI477_RS01505	525	G	A	synonymous
301498	NTHI477_RS01505	481	T	C	nonsynonymous
301517	NTHI477_RS01505	462	A	C	nonsynonymous
301532	NTHI477_RS01505	447	T	C	synonymous
301535	NTHI477_RS01505	444	C	T	nonsynonymous
301541	NTHI477_RS01505	438	G	T	nonsynonymous

301634	NTHI477_RS01505	345	C	T	synonymous
301646	NTHI477_RS01505	333	G	A	synonymous
301649	NTHI477_RS01505	330	A	G	synonymous
301697	NTHI477_RS01505	282	G	A	synonymous
301699	NTHI477_RS01505	280	C	T	nonsynonymous
301706	NTHI477_RS01505	273	C	G	synonymous
301706	NTHI477_RS01505	273	C	T	synonymous
301709	NTHI477_RS01505	270	T	A	nonsynonymous
301711	NTHI477_RS01505	268	T	G	synonymous
301718	NTHI477_RS01505	261	T	C	synonymous
301724	NTHI477_RS01505	255	A	G	synonymous
301733	NTHI477_RS01505	246	T	G	synonymous
301778	NTHI477_RS01505	201	C	A	synonymous
301781	NTHI477_RS01505	198	G	T	synonymous
301784	NTHI477_RS01505	195	C	T	synonymous
301800	NTHI477_RS01505	179	C	T	nonsynonymous
301805	NTHI477_RS01505	174	G	A	synonymous
301826	NTHI477_RS01505	153	G	A	synonymous
301832	NTHI477_RS01505	147	A	G	synonymous
301865	NTHI477_RS01505	114	T	A	synonymous
301907	NTHI477_RS01505	72	A	T	synonymous
301919	NTHI477_RS01505	60	A	G	synonymous
301922	NTHI477_RS01505	57	T	A	synonymous
301969	NTHI477_RS01505	10	C	T	nonsynonymous
302096	NTHI477_RS01510	1882	C	T	nonsynonymous
302114	NTHI477_RS01510	1864	C	T	nonsynonymous
302115	NTHI477_RS01510	1863	C	T	synonymous
302224	NTHI477_RS01510	1754	G	A	nonsynonymous
302251	NTHI477_RS01510	1727	T	G	nonsynonymous
302269	NTHI477_RS01510	1709	T	C	nonsynonymous
302270	NTHI477_RS01510	1708	G	T	nonsynonymous
302271	NTHI477_RS01510	1707	G	A	synonymous
302283	NTHI477_RS01510	1695	C	A	nonsynonymous
302287	NTHI477_RS01510	1691	C	T	nonsynonymous
302288	NTHI477_RS01510	1690	T	G	synonymous
302289	NTHI477_RS01510	1689	T	C	synonymous
302289	NTHI477_RS01510	1689	T	G	nonsynonymous
302290	NTHI477_RS01510	1688	T	C	nonsynonymous
302292	NTHI477_RS01510	1686	G	A	synonymous
302295	NTHI477_RS01510	1683	G	A	synonymous
302301	NTHI477_RS01510	1677	G	A	synonymous
302310	NTHI477_RS01510	1668	C	T	synonymous
302333	NTHI477_RS01510	1645	C	T	nonsynonymous
302352	NTHI477_RS01510	1626	C	A	synonymous
302382	NTHI477_RS01510	1596	A	G	synonymous

302388	NTHI477_RS01510	1590	A	G	synonymous
302391	NTHI477_RS01510	1587	G	A	synonymous
302403	NTHI477_RS01510	1575	A	C	synonymous
302411	NTHI477_RS01510	1567	C	T	nonsynonymous
302424	NTHI477_RS01510	1554	C	A	synonymous
302427	NTHI477_RS01510	1551	C	T	synonymous
302429	NTHI477_RS01510	1549	A	G	synonymous
302430	NTHI477_RS01510	1548	A	C	synonymous
302432	NTHI477_RS01510	1546	G	A	nonsynonymous
302436	NTHI477_RS01510	1542	A	T	synonymous
302441	NTHI477_RS01510	1537	C	T	nonsynonymous
302442	NTHI477_RS01510	1536	G	A	synonymous
302472	NTHI477_RS01510	1506	T	G	synonymous
302475	NTHI477_RS01510	1503	A	G	synonymous
302517	NTHI477_RS01510	1461	C	T	synonymous
302520	NTHI477_RS01510	1458	T	C	synonymous
302523	NTHI477_RS01510	1455	A	G	synonymous
302525	NTHI477_RS01510	1453	A	C	nonsynonymous
302530	NTHI477_RS01510	1448	G	A	nonsynonymous
302531	NTHI477_RS01510	1447	A	G	nonsynonymous
302532	NTHI477_RS01510	1446	T	G	nonsynonymous
302535	NTHI477_RS01510	1443	T	C	synonymous
302538	NTHI477_RS01510	1440	A	T	synonymous
302538	NTHI477_RS01510	1440	A	C	synonymous
302544	NTHI477_RS01510	1434	T	C	synonymous
302547	NTHI477_RS01510	1431	T	A	synonymous
302549	NTHI477_RS01510	1429	C	T	nonsynonymous
302550	NTHI477_RS01510	1428	T	G	synonymous
302553	NTHI477_RS01510	1425	G	A	synonymous
302559	NTHI477_RS01510	1419	G	A	synonymous
302562	NTHI477_RS01510	1416	A	G	synonymous
302568	NTHI477_RS01510	1410	A	G	synonymous
302574	NTHI477_RS01510	1404	G	A	synonymous
302577	NTHI477_RS01510	1401	T	G	nonsynonymous
302579	NTHI477_RS01510	1399	T	G	synonymous
302580	NTHI477_RS01510	1398	G	A	synonymous
302583	NTHI477_RS01510	1395	G	A	synonymous
302586	NTHI477_RS01510	1392	G	A	synonymous
302587	NTHI477_RS01510	1391	T	C	nonsynonymous
302601	NTHI477_RS01510	1377	G	A	synonymous
302606	NTHI477_RS01510	1372	C	T	nonsynonymous
302610	NTHI477_RS01510	1368	A	G	synonymous
302621	NTHI477_RS01510	1357	G	C	nonsynonymous
302625	NTHI477_RS01510	1353	G	A	synonymous
302628	NTHI477_RS01510	1350	A	G	synonymous

302631	NTHI477_RS01510	1347	C	G	synonymous
302634	NTHI477_RS01510	1344	T	C	synonymous
302640	NTHI477_RS01510	1338	T	C	synonymous
302642	NTHI477_RS01510	1336	T	G	nonsynonymous
302642	NTHI477_RS01510	1336	T	C	nonsynonymous
302643	NTHI477_RS01510	1335	A	C	synonymous
302644	NTHI477_RS01510	1334	A	T	nonsynonymous
302645	NTHI477_RS01510	1333	G	T	nonsynonymous
302647	NTHI477_RS01510	1331	G	A	nonsynonymous
302648	NTHI477_RS01510	1330	T	C	nonsynonymous
302652	NTHI477_RS01510	1326	A	G	synonymous
302658	NTHI477_RS01510	1320	G	A	synonymous
302661	NTHI477_RS01510	1317	A	G	synonymous
302664	NTHI477_RS01510	1314	G	A	synonymous
302667	NTHI477_RS01510	1311	A	G	synonymous
302670	NTHI477_RS01510	1308	A	G	synonymous
302676	NTHI477_RS01510	1302	A	C	synonymous
302682	NTHI477_RS01510	1296	A	G	synonymous
302688	NTHI477_RS01510	1290	T	C	synonymous
302690	NTHI477_RS01510	1288	G	A	nonsynonymous
302709	NTHI477_RS01510	1269	A	G	synonymous
302712	NTHI477_RS01510	1266	T	C	synonymous
302718	NTHI477_RS01510	1260	G	A	synonymous
302719	NTHI477_RS01510	1259	A	C	nonsynonymous
302724	NTHI477_RS01510	1254	G	A	synonymous
302733	NTHI477_RS01510	1245	A	G	synonymous
302739	NTHI477_RS01510	1239	T	A	synonymous
302747	NTHI477_RS01510	1231	C	T	nonsynonymous
302748	NTHI477_RS01510	1230	A	G	synonymous
302750	NTHI477_RS01510	1228	C	T	nonsynonymous
302751	NTHI477_RS01510	1227	G	A	synonymous
302751	NTHI477_RS01510	1227	G	C	nonsynonymous
302771	NTHI477_RS01510	1207	G	A	synonymous
302775	NTHI477_RS01510	1203	G	A	synonymous
302783	NTHI477_RS01510	1195	A	T	nonsynonymous
302787	NTHI477_RS01510	1191	A	G	synonymous
302793	NTHI477_RS01510	1185	A	G	synonymous
302835	NTHI477_RS01510	1143	G	C	synonymous
302856	NTHI477_RS01510	1122	T	C	synonymous
302868	NTHI477_RS01510	1110	A	C	synonymous
302871	NTHI477_RS01510	1107	C	T	synonymous
302898	NTHI477_RS01510	1080	A	G	synonymous
302901	NTHI477_RS01510	1077	T	G	synonymous
302913	NTHI477_RS01510	1065	T	A	synonymous
302916	NTHI477_RS01510	1062	G	A	synonymous

302919	NTHI477_RS01510	1059	A	T	synonymous
302937	NTHI477_RS01510	1041	C	T	synonymous
302940	NTHI477_RS01510	1038	A	G	synonymous
302943	NTHI477_RS01510	1035	A	G	synonymous
302949	NTHI477_RS01510	1029	T	A	synonymous
302957	NTHI477_RS01510	1021	C	T	nonsynonymous
302961	NTHI477_RS01510	1017	A	C	synonymous
302964	NTHI477_RS01510	1014	A	T	synonymous
302966	NTHI477_RS01510	1012	G	A	nonsynonymous
302967	NTHI477_RS01510	1011	C	A	synonymous
302970	NTHI477_RS01510	1008	G	A	synonymous
302970	NTHI477_RS01510	1008	G	C	synonymous
302972	NTHI477_RS01510	1006	A	T	nonsynonymous
302991	NTHI477_RS01510	987	A	G	synonymous
302991	NTHI477_RS01510	987	A	T	synonymous
303030	NTHI477_RS01510	948	A	G	synonymous
303033	NTHI477_RS01510	945	C	T	synonymous
303036	NTHI477_RS01510	942	C	G	synonymous
303042	NTHI477_RS01510	936	C	T	synonymous
303081	NTHI477_RS01510	897	G	A	synonymous
303114	NTHI477_RS01510	864	G	A	synonymous
303144	NTHI477_RS01510	834	A	G	synonymous
303153	NTHI477_RS01510	825	G	A	synonymous
303155	NTHI477_RS01510	823	A	T	nonsynonymous
303186	NTHI477_RS01510	792	A	G	synonymous
303204	NTHI477_RS01510	774	A	G	synonymous
303210	NTHI477_RS01510	768	G	A	synonymous
303218	NTHI477_RS01510	760	C	T	nonsynonymous
303222	NTHI477_RS01510	756	A	G	synonymous
303225	NTHI477_RS01510	753	C	T	synonymous
303226	NTHI477_RS01510	752	T	G	nonsynonymous
303228	NTHI477_RS01510	750	A	T	nonsynonymous
303243	NTHI477_RS01510	735	T	C	synonymous
303264	NTHI477_RS01510	714	T	C	synonymous
303279	NTHI477_RS01510	699	T	C	synonymous
303297	NTHI477_RS01510	681	C	T	synonymous
303309	NTHI477_RS01510	669	C	T	synonymous
303345	NTHI477_RS01510	633	T	A	synonymous
303348	NTHI477_RS01510	630	T	A	synonymous
303359	NTHI477_RS01510	619	G	T	nonsynonymous
303420	NTHI477_RS01510	558	C	A	nonsynonymous
303437	NTHI477_RS01510	541	C	T	nonsynonymous
303444	NTHI477_RS01510	534	T	A	synonymous
303489	NTHI477_RS01510	489	G	A	synonymous
303494	NTHI477_RS01510	484	A	G	synonymous

303511	NTHI477_RS01510	467	A	G	nonsynonymous
303513	NTHI477_RS01510	465	G	A	synonymous
303536	NTHI477_RS01510	442	C	T	nonsynonymous
303537	NTHI477_RS01510	441	A	C	synonymous
303545	NTHI477_RS01510	433	G	A	nonsynonymous
303555	NTHI477_RS01510	423	T	G	nonsynonymous
303556	NTHI477_RS01510	422	A	G	nonsynonymous
303558	NTHI477_RS01510	420	A	G	synonymous
303561	NTHI477_RS01510	417	A	G	synonymous
303566	NTHI477_RS01510	412	T	C	nonsynonymous
303575	NTHI477_RS01510	403	T	C	nonsynonymous
303579	NTHI477_RS01510	399	A	T	nonsynonymous
303585	NTHI477_RS01510	393	C	T	synonymous
303585	NTHI477_RS01510	393	C	A	synonymous
303591	NTHI477_RS01510	387	C	A	synonymous
303593	NTHI477_RS01510	385	C	T	nonsynonymous
303594	NTHI477_RS01510	384	A	T	synonymous
303594	NTHI477_RS01510	384	A	C	synonymous
303595	NTHI477_RS01510	383	A	C	nonsynonymous
303606	NTHI477_RS01510	372	G	C	synonymous
303607	NTHI477_RS01510	371	G	A	nonsynonymous
303611	NTHI477_RS01510	367	G	T	nonsynonymous
303615	NTHI477_RS01510	363	C	G	synonymous
303615	NTHI477_RS01510	363	C	A	synonymous
303618	NTHI477_RS01510	360	G	A	synonymous
303621	NTHI477_RS01510	357	G	A	synonymous
303629	NTHI477_RS01510	349	T	G	nonsynonymous
303633	NTHI477_RS01510	345	C	A	synonymous
303634	NTHI477_RS01510	344	G	T	nonsynonymous
303635	NTHI477_RS01510	343	A	T	nonsynonymous
303636	NTHI477_RS01510	342	A	C	synonymous
303638	NTHI477_RS01510	340	G	T	nonsynonymous
303639	NTHI477_RS01510	339	C	T	synonymous
303642	NTHI477_RS01510	336	G	A	synonymous
303653	NTHI477_RS01510	325	A	G	nonsynonymous
303664	NTHI477_RS01510	314	C	G	nonsynonymous
303667	NTHI477_RS01510	311	C	G	nonsynonymous
303675	NTHI477_RS01510	303	A	C	synonymous
303687	NTHI477_RS01510	291	T	C	synonymous
303711	NTHI477_RS01510	267	C	T	nonsynonymous
303714	NTHI477_RS01510	264	T	C	synonymous
303720	NTHI477_RS01510	258	A	C	nonsynonymous
303722	NTHI477_RS01510	256	T	G	nonsynonymous
303723	NTHI477_RS01510	255	C	T	synonymous
303726	NTHI477_RS01510	252	G	A	synonymous

303733	NTHI477_RS01510	245	A	T	nonsynonymous
303753	NTHI477_RS01510	225	T	A	synonymous
303754	NTHI477_RS01510	224	C	G	nonsynonymous
303762	NTHI477_RS01510	216	C	T	synonymous
303768	NTHI477_RS01510	210	A	C	synonymous
303781	NTHI477_RS01510	197	G	A	nonsynonymous
303794	NTHI477_RS01510	184	G	T	nonsynonymous
303800	NTHI477_RS01510	178	C	T	nonsynonymous
303805	NTHI477_RS01510	173	T	C	nonsynonymous
303813	NTHI477_RS01510	165	C	T	synonymous
303819	NTHI477_RS01510	159	C	T	synonymous
303828	NTHI477_RS01510	150	T	A	synonymous
303837	NTHI477_RS01510	141	A	G	synonymous
303847	NTHI477_RS01510	131	T	G	nonsynonymous
303852	NTHI477_RS01510	126	A	T	synonymous
303864	NTHI477_RS01510	114	T	C	synonymous
303870	NTHI477_RS01510	108	T	A	synonymous
303871	NTHI477_RS01510	107	A	G	nonsynonymous
303872	NTHI477_RS01510	106	T	A	nonsynonymous
303876	NTHI477_RS01510	102	C	T	synonymous
303880	NTHI477_RS01510	98	G	T	nonsynonymous
303892	NTHI477_RS01510	86	G	A	nonsynonymous
303915	NTHI477_RS01510	63	G	A	synonymous
303956	NTHI477_RS01510	22	C	T	nonsynonymous
303957	NTHI477_RS01510	21	G	A	synonymous
303966	NTHI477_RS01510	12	G	A	synonymous
303969	NTHI477_RS01510	9	T	C	synonymous
303970	NTHI477_RS01510	8	T	G	nonsynonymous
303972	NTHI477_RS01510	6	G	A	synonymous
303980	intergenic	intergenic	T	C	intergenic
303996	intergenic	intergenic	G	C	intergenic
304005	intergenic	intergenic	G	T	intergenic
304084	NTHI477_RS01515	2185	C	T	nonsynonymous
304139	NTHI477_RS01515	2130	T	C	synonymous
304157	NTHI477_RS01515	2112	G	A	synonymous
304160	NTHI477_RS01515	2109	T	G	nonsynonymous
304162	NTHI477_RS01515	2107	T	G	nonsynonymous
304176	NTHI477_RS01515	2093	C	T	nonsynonymous
304187	NTHI477_RS01515	2082	G	A	synonymous
304193	NTHI477_RS01515	2076	G	A	synonymous
304196	NTHI477_RS01515	2073	T	A	synonymous
304199	NTHI477_RS01515	2070	A	G	synonymous
304204	NTHI477_RS01515	2065	C	G	nonsynonymous
304205	NTHI477_RS01515	2064	G	A	synonymous
304208	NTHI477_RS01515	2061	A	G	synonymous

304214	NTHI477_RS01515	2055	C	T	synonymous
304214	NTHI477_RS01515	2055	C	A	synonymous
304220	NTHI477_RS01515	2049	G	A	synonymous
304226	NTHI477_RS01515	2043	T	C	synonymous
304228	NTHI477_RS01515	2041	A	G	synonymous
304238	NTHI477_RS01515	2031	G	A	synonymous
304241	NTHI477_RS01515	2028	T	A	nonsynonymous
304242	NTHI477_RS01515	2027	T	G	nonsynonymous
304243	NTHI477_RS01515	2026	T	C	nonsynonymous
304244	NTHI477_RS01515	2025	T	G	synonymous
304247	NTHI477_RS01515	2022	T	C	synonymous
304251	NTHI477_RS01515	2018	A	G	nonsynonymous
304252	NTHI477_RS01515	2017	C	T	nonsynonymous
304256	NTHI477_RS01515	2013	C	T	synonymous
304259	NTHI477_RS01515	2010	G	C	synonymous
304259	NTHI477_RS01515	2010	G	A	synonymous
304262	NTHI477_RS01515	2007	G	A	synonymous
304264	NTHI477_RS01515	2005	T	C	nonsynonymous
304265	NTHI477_RS01515	2004	G	A	synonymous
304271	NTHI477_RS01515	1998	A	G	synonymous
304271	NTHI477_RS01515	1998	A	C	nonsynonymous
304273	NTHI477_RS01515	1996	T	C	nonsynonymous
304274	NTHI477_RS01515	1995	G	A	synonymous
304295	NTHI477_RS01515	1974	G	A	synonymous
304313	NTHI477_RS01515	1956	A	G	synonymous
304344	NTHI477_RS01515	1925	G	A	nonsynonymous
304385	NTHI477_RS01515	1884	G	A	synonymous
304403	NTHI477_RS01515	1866	G	A	synonymous
304463	NTHI477_RS01515	1806	C	T	synonymous
304466	NTHI477_RS01515	1803	T	C	synonymous
304475	NTHI477_RS01515	1794	G	A	synonymous
304481	NTHI477_RS01515	1788	A	G	synonymous
304484	NTHI477_RS01515	1785	T	A	synonymous
304488	NTHI477_RS01515	1781	C	T	nonsynonymous
304529	NTHI477_RS01515	1740	A	G	synonymous
304538	NTHI477_RS01515	1731	C	T	synonymous
304541	NTHI477_RS01515	1728	A	T	synonymous
304543	NTHI477_RS01515	1726	G	T	nonsynonymous
304544	NTHI477_RS01515	1725	A	G	synonymous
304550	NTHI477_RS01515	1719	G	A	synonymous
304562	NTHI477_RS01515	1707	C	A	synonymous
304574	NTHI477_RS01515	1695	C	T	synonymous
304583	NTHI477_RS01515	1686	G	A	synonymous
304589	NTHI477_RS01515	1680	G	A	synonymous
304592	NTHI477_RS01515	1677	G	C	synonymous

304604	NTHI477_RS01515	1665	A	G	synonymous
304610	NTHI477_RS01515	1659	A	G	synonymous
304613	NTHI477_RS01515	1656	T	A	synonymous
304616	NTHI477_RS01515	1653	A	C	synonymous
304628	NTHI477_RS01515	1641	G	A	synonymous
304640	NTHI477_RS01515	1629	C	T	synonymous
304645	NTHI477_RS01515	1624	C	T	nonsynonymous
304646	NTHI477_RS01515	1623	G	A	synonymous
304646	NTHI477_RS01515	1623	G	T	synonymous
304649	NTHI477_RS01515	1620	T	C	synonymous
304659	NTHI477_RS01515	1610	C	T	nonsynonymous
304706	NTHI477_RS01515	1563	T	C	synonymous
304709	NTHI477_RS01515	1560	A	C	synonymous
304724	NTHI477_RS01515	1545	G	A	synonymous
304733	NTHI477_RS01515	1536	T	C	synonymous
304733	NTHI477_RS01515	1536	T	A	synonymous
304735	NTHI477_RS01515	1534	C	T	nonsynonymous
304739	NTHI477_RS01515	1530	T	C	synonymous
304745	NTHI477_RS01515	1524	G	A	synonymous
304754	NTHI477_RS01515	1515	G	A	synonymous
304774	NTHI477_RS01515	1495	A	C	nonsynonymous
304778	NTHI477_RS01515	1491	G	C	synonymous
304778	NTHI477_RS01515	1491	G	T	synonymous
304789	NTHI477_RS01515	1480	T	G	nonsynonymous
304796	NTHI477_RS01515	1473	A	G	synonymous
304799	NTHI477_RS01515	1470	C	G	synonymous
304802	NTHI477_RS01515	1467	T	C	synonymous
304802	NTHI477_RS01515	1467	T	A	synonymous
304805	NTHI477_RS01515	1464	A	G	synonymous
304832	NTHI477_RS01515	1437	G	A	synonymous
304838	NTHI477_RS01515	1431	G	A	synonymous
304841	NTHI477_RS01515	1428	G	A	synonymous
304844	NTHI477_RS01515	1425	C	T	synonymous
304847	NTHI477_RS01515	1422	G	T	synonymous
304850	NTHI477_RS01515	1419	G	A	synonymous
304853	NTHI477_RS01515	1416	G	A	synonymous
304855	NTHI477_RS01515	1414	C	T	nonsynonymous
304868	NTHI477_RS01515	1401	G	A	synonymous
304886	NTHI477_RS01515	1383	G	A	synonymous
304889	NTHI477_RS01515	1380	A	T	synonymous
304895	NTHI477_RS01515	1374	C	T	synonymous
304904	NTHI477_RS01515	1365	A	G	synonymous
304913	NTHI477_RS01515	1356	T	A	synonymous
304913	NTHI477_RS01515	1356	T	C	synonymous
304918	NTHI477_RS01515	1351	C	T	nonsynonymous

304925	NTHI477_RS01515	1344	G	A	synonymous
304946	NTHI477_RS01515	1323	G	A	synonymous
304954	NTHI477_RS01515	1315	A	G	synonymous
304961	NTHI477_RS01515	1308	A	G	synonymous
305009	NTHI477_RS01515	1260	G	T	synonymous
305012	NTHI477_RS01515	1257	C	T	synonymous
305015	NTHI477_RS01515	1254	C	T	synonymous
305024	NTHI477_RS01515	1245	G	A	synonymous
305030	NTHI477_RS01515	1239	G	A	synonymous
305036	NTHI477_RS01515	1233	G	A	synonymous
305042	NTHI477_RS01515	1227	T	G	synonymous
305059	NTHI477_RS01515	1210	T	C	nonsynonymous
305069	NTHI477_RS01515	1200	A	G	synonymous
305081	NTHI477_RS01515	1188	G	A	synonymous
305093	NTHI477_RS01515	1176	G	A	synonymous
305096	NTHI477_RS01515	1173	A	C	synonymous
305099	NTHI477_RS01515	1170	G	T	synonymous
305105	NTHI477_RS01515	1164	G	A	synonymous
305111	NTHI477_RS01515	1158	C	T	synonymous
305113	NTHI477_RS01515	1156	G	A	synonymous
305116	NTHI477_RS01515	1153	A	T	nonsynonymous
305123	NTHI477_RS01515	1146	C	G	synonymous
305126	NTHI477_RS01515	1143	T	C	synonymous
305129	NTHI477_RS01515	1140	G	A	synonymous
305132	NTHI477_RS01515	1137	G	A	synonymous
305135	NTHI477_RS01515	1134	T	C	synonymous
305138	NTHI477_RS01515	1131	A	G	synonymous
305144	NTHI477_RS01515	1125	A	G	synonymous
305150	NTHI477_RS01515	1119	C	T	synonymous
305152	NTHI477_RS01515	1117	A	G	synonymous
305153	NTHI477_RS01515	1116	G	C	synonymous
305156	NTHI477_RS01515	1113	A	G	synonymous
305162	NTHI477_RS01515	1107	A	G	synonymous
305168	NTHI477_RS01515	1101	A	G	synonymous
305174	NTHI477_RS01515	1095	G	A	synonymous
305180	NTHI477_RS01515	1089	G	A	synonymous
305183	NTHI477_RS01515	1086	A	T	synonymous
305183	NTHI477_RS01515	1086	A	C	synonymous
305189	NTHI477_RS01515	1080	T	G	nonsynonymous
305190	NTHI477_RS01515	1079	T	G	nonsynonymous
305192	NTHI477_RS01515	1077	A	G	synonymous
305195	NTHI477_RS01515	1074	T	C	synonymous
305198	NTHI477_RS01515	1071	A	G	synonymous
305204	NTHI477_RS01515	1065	G	A	synonymous
305210	NTHI477_RS01515	1059	G	A	synonymous

305216	NTHI477_RS01515	1053	A	G	synonymous
305219	NTHI477_RS01515	1050	T	A	synonymous
305222	NTHI477_RS01515	1047	G	A	synonymous
305228	NTHI477_RS01515	1041	A	G	synonymous
305231	NTHI477_RS01515	1038	A	G	synonymous
305234	NTHI477_RS01515	1035	G	C	synonymous
305234	NTHI477_RS01515	1035	G	A	synonymous
305252	NTHI477_RS01515	1017	C	T	synonymous
305258	NTHI477_RS01515	1011	C	T	synonymous
305285	NTHI477_RS01515	984	T	C	synonymous
305313	NTHI477_RS01515	956	C	A	nonsynonymous
305315	NTHI477_RS01515	954	A	G	synonymous
305318	NTHI477_RS01515	951	T	G	synonymous
305321	NTHI477_RS01515	948	C	T	synonymous
305327	NTHI477_RS01515	942	T	C	synonymous
305330	NTHI477_RS01515	939	G	A	synonymous
305336	NTHI477_RS01515	933	A	G	synonymous
305357	NTHI477_RS01515	912	C	T	synonymous
305366	NTHI477_RS01515	903	T	C	synonymous
305372	NTHI477_RS01515	897	G	A	synonymous
305372	NTHI477_RS01515	897	G	C	synonymous
305390	NTHI477_RS01515	879	C	T	synonymous
305390	NTHI477_RS01515	879	C	A	synonymous
305408	NTHI477_RS01515	861	T	C	synonymous
305417	NTHI477_RS01515	852	T	A	synonymous
305420	NTHI477_RS01515	849	A	G	synonymous
305426	NTHI477_RS01515	843	A	G	synonymous
305429	NTHI477_RS01515	840	G	C	synonymous
305429	NTHI477_RS01515	840	G	A	synonymous
305444	NTHI477_RS01515	825	A	G	synonymous
305537	NTHI477_RS01515	732	A	G	synonymous
305543	NTHI477_RS01515	726	C	T	synonymous
305549	NTHI477_RS01515	720	A	G	synonymous
305552	NTHI477_RS01515	717	T	G	synonymous
305553	NTHI477_RS01515	716	G	C	nonsynonymous
305558	NTHI477_RS01515	711	A	G	synonymous
305561	NTHI477_RS01515	708	T	G	synonymous
305566	NTHI477_RS01515	703	G	T	nonsynonymous
305567	NTHI477_RS01515	702	A	T	synonymous
305568	NTHI477_RS01515	701	C	T	nonsynonymous
305569	NTHI477_RS01515	700	G	T	nonsynonymous
305570	NTHI477_RS01515	699	A	G	synonymous
305574	NTHI477_RS01515	695	T	G	nonsynonymous
305575	NTHI477_RS01515	694	G	C	nonsynonymous
305579	NTHI477_RS01515	690	C	G	synonymous

305579	NTHI477_RS01515	690	C	A	synonymous
305591	NTHI477_RS01515	678	C	T	synonymous
305600	NTHI477_RS01515	669	T	C	synonymous
305603	NTHI477_RS01515	666	T	G	synonymous
305609	NTHI477_RS01515	660	A	C	nonsynonymous
305609	NTHI477_RS01515	660	A	G	synonymous
305611	NTHI477_RS01515	658	T	C	nonsynonymous
305612	NTHI477_RS01515	657	T	C	synonymous
305615	NTHI477_RS01515	654	T	C	synonymous
305618	NTHI477_RS01515	651	A	T	synonymous
305621	NTHI477_RS01515	648	T	A	synonymous
305636	NTHI477_RS01515	633	T	C	synonymous
305642	NTHI477_RS01515	627	A	G	synonymous
305648	NTHI477_RS01515	621	G	C	synonymous
305652	NTHI477_RS01515	617	T	A	nonsynonymous
305654	NTHI477_RS01515	615	A	T	synonymous
305660	NTHI477_RS01515	609	C	G	synonymous
305663	NTHI477_RS01515	606	T	C	synonymous
305678	NTHI477_RS01515	591	G	T	synonymous
305682	NTHI477_RS01515	587	T	A	nonsynonymous
305683	NTHI477_RS01515	586	A	G	nonsynonymous
305684	NTHI477_RS01515	585	G	A	synonymous
305693	NTHI477_RS01515	576	C	T	synonymous
305702	NTHI477_RS01515	567	T	C	synonymous
305708	NTHI477_RS01515	561	G	A	synonymous
305714	NTHI477_RS01515	555	A	T	synonymous
305717	NTHI477_RS01515	552	A	T	synonymous
305719	NTHI477_RS01515	550	G	A	nonsynonymous
305720	NTHI477_RS01515	549	G	A	synonymous
305723	NTHI477_RS01515	546	G	A	synonymous
305724	NTHI477_RS01515	545	G	A	nonsynonymous
305729	NTHI477_RS01515	540	C	T	synonymous
305732	NTHI477_RS01515	537	C	T	synonymous
305734	NTHI477_RS01515	535	G	A	synonymous
305735	NTHI477_RS01515	534	G	A	synonymous
305738	NTHI477_RS01515	531	T	G	nonsynonymous
305738	NTHI477_RS01515	531	T	C	synonymous
305740	NTHI477_RS01515	529	A	G	synonymous
305741	NTHI477_RS01515	528	T	C	synonymous
305747	NTHI477_RS01515	522	C	A	synonymous
305747	NTHI477_RS01515	522	C	T	synonymous
305753	NTHI477_RS01515	516	G	A	synonymous
305759	NTHI477_RS01515	510	C	T	synonymous
305759	NTHI477_RS01515	510	C	G	synonymous
305760	NTHI477_RS01515	509	G	A	nonsynonymous

305761	NTHI477_RS01515	508	G	A	nonsynonymous
305762	NTHI477_RS01515	507	G	A	synonymous
305765	NTHI477_RS01515	504	G	A	synonymous
305774	NTHI477_RS01515	495	C	T	synonymous
305774	NTHI477_RS01515	495	C	A	synonymous
305777	NTHI477_RS01515	492	G	A	synonymous
305780	NTHI477_RS01515	489	G	A	synonymous
305783	NTHI477_RS01515	486	G	A	synonymous
305789	NTHI477_RS01515	480	A	G	synonymous
305810	NTHI477_RS01515	459	T	C	synonymous
305810	NTHI477_RS01515	459	T	A	synonymous
305822	NTHI477_RS01515	447	T	A	synonymous
305822	NTHI477_RS01515	447	T	G	synonymous
305828	NTHI477_RS01515	441	T	G	synonymous
305834	NTHI477_RS01515	435	A	G	synonymous
305846	NTHI477_RS01515	423	G	A	synonymous
305855	NTHI477_RS01515	414	C	T	synonymous
305858	NTHI477_RS01515	411	C	T	synonymous
305885	NTHI477_RS01515	384	A	G	synonymous
305888	NTHI477_RS01515	381	T	C	synonymous
305893	NTHI477_RS01515	376	C	T	nonsynonymous
305906	NTHI477_RS01515	363	T	C	synonymous
305909	NTHI477_RS01515	360	G	A	synonymous
305921	NTHI477_RS01515	348	T	A	synonymous
305951	NTHI477_RS01515	318	G	A	synonymous
305960	NTHI477_RS01515	309	T	A	synonymous
305960	NTHI477_RS01515	309	T	C	synonymous
305965	NTHI477_RS01515	304	G	A	nonsynonymous
305986	NTHI477_RS01515	283	T	C	nonsynonymous
305987	NTHI477_RS01515	282	A	T	synonymous
305993	NTHI477_RS01515	276	T	A	synonymous
306002	NTHI477_RS01515	267	A	G	synonymous
306035	NTHI477_RS01515	234	T	A	nonsynonymous
306068	NTHI477_RS01515	201	T	G	synonymous
306074	NTHI477_RS01515	195	C	T	synonymous
306089	NTHI477_RS01515	180	C	A	synonymous
306107	NTHI477_RS01515	162	A	T	synonymous
306122	NTHI477_RS01515	147	A	T	synonymous
306125	NTHI477_RS01515	144	C	T	synonymous
306131	NTHI477_RS01515	138	C	A	nonsynonymous
306131	NTHI477_RS01515	138	C	T	nonsynonymous
306133	NTHI477_RS01515	136	T	C	nonsynonymous
306134	NTHI477_RS01515	135	T	G	synonymous
306138	NTHI477_RS01515	131	T	C	nonsynonymous
306141	NTHI477_RS01515	128	G	A	nonsynonymous

306143	NTHI477_RS01515	126	A	C	nonsynonymous
306155	NTHI477_RS01515	114	C	G	synonymous
306158	NTHI477_RS01515	111	A	G	synonymous
306161	NTHI477_RS01515	108	G	A	synonymous
306173	NTHI477_RS01515	96	T	G	nonsynonymous
306174	NTHI477_RS01515	95	T	C	nonsynonymous
306175	NTHI477_RS01515	94	G	A	nonsynonymous
306179	NTHI477_RS01515	90	G	A	synonymous
306194	NTHI477_RS01515	75	A	T	synonymous
306197	NTHI477_RS01515	72	C	T	synonymous
306204	NTHI477_RS01515	65	A	C	nonsynonymous
306212	NTHI477_RS01515	57	A	G	synonymous
306218	NTHI477_RS01515	51	G	A	synonymous
306243	NTHI477_RS01515	26	G	A	nonsynonymous
306247	NTHI477_RS01515	22	C	T	nonsynonymous
306260	NTHI477_RS01515	9	G	A	synonymous
306374	NTHI477_RS01520	1995	G	A	synonymous
306377	NTHI477_RS01520	1992	T	C	synonymous
306396	NTHI477_RS01520	1973	C	G	nonsynonymous
306398	NTHI477_RS01520	1971	C	T	synonymous
306401	NTHI477_RS01520	1968	T	C	synonymous
306408	NTHI477_RS01520	1961	G	A	nonsynonymous
306461	NTHI477_RS01520	1908	G	A	synonymous
306470	NTHI477_RS01520	1899	C	T	synonymous
306481	NTHI477_RS01520	1888	A	G	nonsynonymous
306493	NTHI477_RS01520	1876	G	T	synonymous
306494	NTHI477_RS01520	1875	A	G	synonymous
306502	NTHI477_RS01520	1867	A	G	nonsynonymous
306524	NTHI477_RS01520	1845	T	C	synonymous
306524	NTHI477_RS01520	1845	T	G	synonymous
306530	NTHI477_RS01520	1839	A	G	synonymous
306533	NTHI477_RS01520	1836	T	C	synonymous
306545	NTHI477_RS01520	1824	C	T	synonymous
306581	NTHI477_RS01520	1788	G	A	synonymous
306593	NTHI477_RS01520	1776	C	T	nonsynonymous
306596	NTHI477_RS01520	1773	A	G	synonymous
306599	NTHI477_RS01520	1770	A	G	synonymous
306608	NTHI477_RS01520	1761	A	G	synonymous
306637	NTHI477_RS01520	1732	G	A	synonymous
306644	NTHI477_RS01520	1725	G	A	synonymous
306671	NTHI477_RS01520	1698	G	A	synonymous
306692	NTHI477_RS01520	1677	T	C	synonymous
306707	NTHI477_RS01520	1662	T	C	synonymous
306710	NTHI477_RS01520	1659	A	G	synonymous
306732	NTHI477_RS01520	1637	C	T	nonsynonymous

306750	NTHI477_RS01520	1619	C	A	nonsynonymous
306752	NTHI477_RS01520	1617	A	G	synonymous
306776	NTHI477_RS01520	1593	A	G	synonymous
306826	NTHI477_RS01520	1543	G	T	synonymous
306827	NTHI477_RS01520	1542	C	T	synonymous
306842	NTHI477_RS01520	1527	A	G	synonymous
306860	NTHI477_RS01520	1509	A	G	synonymous
306893	NTHI477_RS01520	1476	G	A	synonymous
306902	NTHI477_RS01520	1467	C	A	synonymous
306928	NTHI477_RS01520	1441	G	A	nonsynonymous
306929	NTHI477_RS01520	1440	C	T	synonymous
306938	NTHI477_RS01520	1431	A	G	synonymous
306941	NTHI477_RS01520	1428	G	A	synonymous
306950	NTHI477_RS01520	1419	G	A	synonymous
306961	NTHI477_RS01520	1408	T	C	nonsynonymous
306980	NTHI477_RS01520	1389	G	A	synonymous
307007	NTHI477_RS01520	1362	T	A	synonymous
307009	NTHI477_RS01520	1360	G	A	synonymous
307016	NTHI477_RS01520	1353	G	A	synonymous
307036	NTHI477_RS01520	1333	G	A	nonsynonymous
307057	NTHI477_RS01520	1312	T	C	nonsynonymous
307067	NTHI477_RS01520	1302	T	C	synonymous
307075	NTHI477_RS01520	1294	G	A	synonymous
307076	NTHI477_RS01520	1293	C	T	synonymous
307079	NTHI477_RS01520	1290	T	G	synonymous
307079	NTHI477_RS01520	1290	T	C	synonymous
307094	NTHI477_RS01520	1275	T	C	synonymous
307112	NTHI477_RS01520	1257	C	T	synonymous
307115	NTHI477_RS01520	1254	G	T	synonymous
307118	NTHI477_RS01520	1251	T	A	synonymous
307118	NTHI477_RS01520	1251	T	G	synonymous
307133	NTHI477_RS01520	1236	A	G	synonymous
307136	NTHI477_RS01520	1233	T	C	synonymous
307139	NTHI477_RS01520	1230	C	T	synonymous
307142	NTHI477_RS01520	1227	A	G	synonymous
307159	NTHI477_RS01520	1210	C	A	nonsynonymous
307172	NTHI477_RS01520	1197	A	G	synonymous
307211	NTHI477_RS01520	1158	T	C	synonymous
307214	NTHI477_RS01520	1155	G	A	synonymous
307214	NTHI477_RS01520	1155	G	C	synonymous
307232	NTHI477_RS01520	1137	A	C	synonymous
307239	NTHI477_RS01520	1130	T	C	nonsynonymous
307240	NTHI477_RS01520	1129	T	C	nonsynonymous
307244	NTHI477_RS01520	1125	G	A	synonymous
307247	NTHI477_RS01520	1122	A	T	synonymous

307249	NTHI477_RS01520	1120	A	T	nonsynonymous
307275	NTHI477_RS01520	1094	G	A	nonsynonymous
307280	NTHI477_RS01520	1089	T	C	synonymous
307283	NTHI477_RS01520	1086	T	C	synonymous
307289	NTHI477_RS01520	1080	T	C	synonymous
307292	NTHI477_RS01520	1077	A	G	synonymous
307319	NTHI477_RS01520	1050	G	A	synonymous
307324	NTHI477_RS01520	1045	G	T	nonsynonymous
307325	NTHI477_RS01520	1044	C	A	nonsynonymous
307328	NTHI477_RS01520	1041	A	C	nonsynonymous
307331	NTHI477_RS01520	1038	G	A	synonymous
307335	NTHI477_RS01520	1034	A	G	nonsynonymous
307345	NTHI477_RS01520	1024	G	A	synonymous
307346	NTHI477_RS01520	1023	G	A	synonymous
307373	NTHI477_RS01520	996	C	T	synonymous
307383	NTHI477_RS01520	986	T	C	nonsynonymous
307386	NTHI477_RS01520	983	C	T	nonsynonymous
307403	NTHI477_RS01520	966	A	T	synonymous
307403	NTHI477_RS01520	966	A	G	synonymous
307427	NTHI477_RS01520	942	G	A	synonymous
307430	NTHI477_RS01520	939	A	G	synonymous
307430	NTHI477_RS01520	939	A	T	synonymous
307433	NTHI477_RS01520	936	G	A	synonymous
307442	NTHI477_RS01520	927	T	C	synonymous
307448	NTHI477_RS01520	921	C	T	synonymous
307450	NTHI477_RS01520	919	A	C	nonsynonymous
307451	NTHI477_RS01520	918	G	T	synonymous
307462	NTHI477_RS01520	907	A	G	synonymous
307463	NTHI477_RS01520	906	G	A	synonymous
307463	NTHI477_RS01520	906	G	C	synonymous
307484	NTHI477_RS01520	885	C	A	synonymous
307491	NTHI477_RS01520	878	C	T	nonsynonymous
307502	NTHI477_RS01520	867	A	C	nonsynonymous
307515	NTHI477_RS01520	854	T	G	nonsynonymous
307523	NTHI477_RS01520	846	T	C	synonymous
307527	NTHI477_RS01520	842	A	G	nonsynonymous
307536	NTHI477_RS01520	833	A	G	nonsynonymous
307538	NTHI477_RS01520	831	G	A	synonymous
307550	NTHI477_RS01520	819	C	T	synonymous
307556	NTHI477_RS01520	813	G	A	synonymous
307561	NTHI477_RS01520	808	A	C	nonsynonymous
307565	NTHI477_RS01520	804	C	T	synonymous
307568	NTHI477_RS01520	801	G	A	synonymous
307569	NTHI477_RS01520	800	G	A	nonsynonymous
307570	NTHI477_RS01520	799	A	T	nonsynonymous

307595	NTHI477_RS01520	774	C	T	synonymous
307598	NTHI477_RS01520	771	A	T	synonymous
307613	NTHI477_RS01520	756	G	A	synonymous
307618	NTHI477_RS01520	751	A	C	nonsynonymous
307627	NTHI477_RS01520	742	C	T	nonsynonymous
307635	NTHI477_RS01520	734	G	T	nonsynonymous
307636	NTHI477_RS01520	733	C	T	nonsynonymous
307638	NTHI477_RS01520	731	G	C	nonsynonymous
307646	NTHI477_RS01520	723	T	G	synonymous
307651	NTHI477_RS01520	718	T	G	nonsynonymous
307654	NTHI477_RS01520	715	T	C	nonsynonymous
307679	NTHI477_RS01520	690	T	C	synonymous
307688	NTHI477_RS01520	681	G	A	synonymous
307713	NTHI477_RS01520	656	G	A	nonsynonymous
307719	NTHI477_RS01520	650	T	G	nonsynonymous
307720	NTHI477_RS01520	649	C	T	nonsynonymous
307721	NTHI477_RS01520	648	C	T	synonymous
307730	NTHI477_RS01520	639	G	A	synonymous
307748	NTHI477_RS01520	621	A	G	synonymous
307751	NTHI477_RS01520	618	G	A	synonymous
307754	NTHI477_RS01520	615	T	G	synonymous
307766	NTHI477_RS01520	603	A	G	synonymous
307771	NTHI477_RS01520	598	C	T	nonsynonymous
307775	NTHI477_RS01520	594	A	G	synonymous
307784	NTHI477_RS01520	585	A	G	synonymous
307793	NTHI477_RS01520	576	T	C	synonymous
307817	NTHI477_RS01520	552	C	T	synonymous
307826	NTHI477_RS01520	543	C	T	synonymous
307826	NTHI477_RS01520	543	C	G	synonymous
307829	NTHI477_RS01520	540	C	G	synonymous
307829	NTHI477_RS01520	540	C	A	synonymous
307833	NTHI477_RS01520	536	T	A	nonsynonymous
307834	NTHI477_RS01520	535	G	A	nonsynonymous
307847	NTHI477_RS01520	522	T	C	synonymous
307850	NTHI477_RS01520	519	A	G	synonymous
307857	NTHI477_RS01520	512	G	T	nonsynonymous
307858	NTHI477_RS01520	511	C	A	nonsynonymous
307858	NTHI477_RS01520	511	C	T	nonsynonymous
307859	NTHI477_RS01520	510	A	G	synonymous
307862	NTHI477_RS01520	507	C	T	synonymous
307868	NTHI477_RS01520	501	G	A	synonymous
307877	NTHI477_RS01520	492	G	T	synonymous
307883	NTHI477_RS01520	486	C	G	synonymous
307901	NTHI477_RS01520	468	G	A	synonymous
307934	NTHI477_RS01520	435	C	T	synonymous

307948	NTHI477_RS01520	421	T	C	nonsynonymous
307957	NTHI477_RS01520	412	A	G	nonsynonymous
307960	NTHI477_RS01520	409	G	A	nonsynonymous
307967	NTHI477_RS01520	402	A	C	synonymous
307979	NTHI477_RS01520	390	A	G	synonymous
307997	NTHI477_RS01520	372	G	A	synonymous
307998	NTHI477_RS01520	371	C	T	nonsynonymous
308000	NTHI477_RS01520	369	A	G	synonymous
308012	NTHI477_RS01520	357	G	A	synonymous
308015	NTHI477_RS01520	354	A	G	synonymous
308059	NTHI477_RS01520	310	A	G	synonymous
308060	NTHI477_RS01520	309	A	G	synonymous
308085	NTHI477_RS01520	284	A	G	nonsynonymous
308093	NTHI477_RS01520	276	T	C	synonymous
308102	NTHI477_RS01520	267	A	G	synonymous
308103	NTHI477_RS01520	266	G	A	nonsynonymous
308122	NTHI477_RS01520	247	G	A	synonymous
308139	NTHI477_RS01520	230	G	A	nonsynonymous
308150	NTHI477_RS01520	219	G	C	synonymous
308152	NTHI477_RS01520	217	G	A	nonsynonymous
308165	NTHI477_RS01520	204	G	T	synonymous
308198	NTHI477_RS01520	171	A	T	synonymous
308204	NTHI477_RS01520	165	T	A	synonymous
308210	NTHI477_RS01520	159	G	A	synonymous
308249	NTHI477_RS01520	120	C	T	synonymous
308249	NTHI477_RS01520	120	C	A	nonsynonymous
308262	NTHI477_RS01520	107	A	G	nonsynonymous
308282	NTHI477_RS01520	87	A	G	synonymous
308294	NTHI477_RS01520	75	C	T	synonymous
308309	NTHI477_RS01520	60	G	A	synonymous
308318	NTHI477_RS01520	51	C	T	synonymous
308327	NTHI477_RS01520	42	G	A	synonymous
308380	intergenic	intergenic	G	C	intergenic
308395	intergenic	intergenic	A	C	intergenic
308411	intergenic	intergenic	G	A	intergenic
308423	intergenic	intergenic	T	G	intergenic
308434	intergenic	intergenic	T	A	intergenic
308434	intergenic	intergenic	T	C	intergenic
308493	NTHI477_RS01525	399	G	T	synonymous
308493	NTHI477_RS01525	399	G	A	synonymous
308574	NTHI477_RS01525	318	A	G	synonymous
308580	NTHI477_RS01525	312	T	C	synonymous
308607	NTHI477_RS01525	285	G	A	synonymous
308610	NTHI477_RS01525	282	G	A	synonymous
308616	NTHI477_RS01525	276	A	G	synonymous

308622	NTHI477_RS01525	270	C	T	synonymous
308624	NTHI477_RS01525	268	G	A	synonymous
308720	NTHI477_RS01525	172	C	G	nonsynonymous
308769	NTHI477_RS01525	123	A	T	nonsynonymous
308771	NTHI477_RS01525	121	T	C	nonsynonymous
308853	NTHI477_RS01525	39	C	G	synonymous
308862	NTHI477_RS01525	30	T	G	synonymous
308880	NTHI477_RS01525	12	T	G	nonsynonymous
308893	intergenic	intergenic	T	C	intergenic
308938	intergenic	intergenic	G	A	intergenic
308942	intergenic	intergenic	G	A	intergenic
308961	intergenic	intergenic	G	A	intergenic
308978	NTHI477_RS01530	1662	T	C	synonymous
308986	NTHI477_RS01530	1654	A	G	synonymous
308987	NTHI477_RS01530	1653	G	A	synonymous
308993	NTHI477_RS01530	1647	T	C	synonymous
308999	NTHI477_RS01530	1641	G	A	synonymous
309005	NTHI477_RS01530	1635	T	C	synonymous
309020	NTHI477_RS01530	1620	T	A	synonymous
309020	NTHI477_RS01530	1620	T	G	synonymous
309038	NTHI477_RS01530	1602	T	C	synonymous
309044	NTHI477_RS01530	1596	G	A	synonymous
309053	NTHI477_RS01530	1587	C	T	synonymous
309068	NTHI477_RS01530	1572	A	G	synonymous
309071	NTHI477_RS01530	1569	A	G	synonymous
309089	NTHI477_RS01530	1551	A	C	nonsynonymous
309090	NTHI477_RS01530	1550	C	T	nonsynonymous
309090	NTHI477_RS01530	1550	C	G	nonsynonymous
309091	NTHI477_RS01530	1549	T	C	nonsynonymous
309092	NTHI477_RS01530	1548	T	A	synonymous
309095	NTHI477_RS01530	1545	G	C	synonymous
309097	NTHI477_RS01530	1543	G	A	nonsynonymous
309140	NTHI477_RS01530	1500	G	A	synonymous
309146	NTHI477_RS01530	1494	C	T	synonymous
309155	NTHI477_RS01530	1485	G	A	synonymous
309168	NTHI477_RS01530	1472	G	A	nonsynonymous
309170	NTHI477_RS01530	1470	C	A	synonymous
309182	NTHI477_RS01530	1458	C	T	synonymous
309185	NTHI477_RS01530	1455	A	G	synonymous
309212	NTHI477_RS01530	1428	T	C	synonymous
309218	NTHI477_RS01530	1422	T	C	synonymous
309219	NTHI477_RS01530	1421	G	A	nonsynonymous
309229	NTHI477_RS01530	1411	T	G	nonsynonymous
309230	NTHI477_RS01530	1410	A	C	synonymous
309231	NTHI477_RS01530	1409	A	G	nonsynonymous

309232	NTHI477_RS01530	1408	C	T	nonsynonymous
309233	NTHI477_RS01530	1407	C	A	synonymous
309245	NTHI477_RS01530	1395	A	G	synonymous
309251	NTHI477_RS01530	1389	G	A	synonymous
309254	NTHI477_RS01530	1386	A	C	synonymous
309260	NTHI477_RS01530	1380	C	T	synonymous
309266	NTHI477_RS01530	1374	A	G	synonymous
309269	NTHI477_RS01530	1371	T	A	synonymous
309275	NTHI477_RS01530	1365	T	C	synonymous
309277	NTHI477_RS01530	1363	C	A	nonsynonymous
309293	NTHI477_RS01530	1347	A	G	synonymous
309305	NTHI477_RS01530	1335	G	A	synonymous
309317	NTHI477_RS01530	1323	A	G	synonymous
309332	NTHI477_RS01530	1308	G	A	synonymous
309336	NTHI477_RS01530	1304	C	T	nonsynonymous
309339	NTHI477_RS01530	1301	G	T	nonsynonymous
309343	NTHI477_RS01530	1297	C	T	nonsynonymous
309350	NTHI477_RS01530	1290	T	C	synonymous
309350	NTHI477_RS01530	1290	T	G	synonymous
309352	NTHI477_RS01530	1288	C	T	nonsynonymous
309353	NTHI477_RS01530	1287	A	G	synonymous
309359	NTHI477_RS01530	1281	C	A	synonymous
309371	NTHI477_RS01530	1269	A	G	synonymous
309377	NTHI477_RS01530	1263	A	G	synonymous
309383	NTHI477_RS01530	1257	C	T	synonymous
309389	NTHI477_RS01530	1251	C	T	synonymous
309398	NTHI477_RS01530	1242	A	G	synonymous
309399	NTHI477_RS01530	1241	T	C	nonsynonymous
309401	NTHI477_RS01530	1239	T	C	synonymous
309404	NTHI477_RS01530	1236	T	C	synonymous
309409	NTHI477_RS01530	1231	A	G	synonymous
309410	NTHI477_RS01530	1230	A	G	synonymous
309428	NTHI477_RS01530	1212	C	T	synonymous
309434	NTHI477_RS01530	1206	C	T	synonymous
309446	NTHI477_RS01530	1194	A	T	synonymous
309449	NTHI477_RS01530	1191	T	G	synonymous
309451	NTHI477_RS01530	1189	G	A	nonsynonymous
309464	NTHI477_RS01530	1176	T	C	synonymous
309470	NTHI477_RS01530	1170	C	T	synonymous
309470	NTHI477_RS01530	1170	C	A	synonymous
309473	NTHI477_RS01530	1167	A	G	synonymous
309479	NTHI477_RS01530	1161	C	T	synonymous
309488	NTHI477_RS01530	1152	C	T	synonymous
309491	NTHI477_RS01530	1149	C	G	synonymous
309500	NTHI477_RS01530	1140	A	T	synonymous

309503	NTHI477_RS01530	1137	A	G	synonymous
309519	NTHI477_RS01530	1121	G	A	nonsynonymous
309521	NTHI477_RS01530	1119	G	T	synonymous
309523	NTHI477_RS01530	1117	C	T	nonsynonymous
309524	NTHI477_RS01530	1116	A	C	synonymous
309530	NTHI477_RS01530	1110	A	G	synonymous
309531	NTHI477_RS01530	1109	A	C	nonsynonymous
309532	NTHI477_RS01530	1108	C	T	nonsynonymous
309533	NTHI477_RS01530	1107	C	T	synonymous
309534	NTHI477_RS01530	1106	G	T	nonsynonymous
309536	NTHI477_RS01530	1104	T	C	synonymous
309537	NTHI477_RS01530	1103	T	A	nonsynonymous
309541	NTHI477_RS01530	1099	T	C	nonsynonymous
309548	NTHI477_RS01530	1092	A	G	synonymous
309557	NTHI477_RS01530	1083	T	C	synonymous
309560	NTHI477_RS01530	1080	C	T	synonymous
309590	NTHI477_RS01530	1050	G	A	synonymous
309599	NTHI477_RS01530	1041	A	G	synonymous
309605	NTHI477_RS01530	1035	A	G	synonymous
309608	NTHI477_RS01530	1032	T	C	synonymous
309623	NTHI477_RS01530	1017	G	A	synonymous
309626	NTHI477_RS01530	1014	G	A	synonymous
309665	NTHI477_RS01530	975	A	C	synonymous
309680	NTHI477_RS01530	960	G	A	synonymous
309683	NTHI477_RS01530	957	A	G	synonymous
309686	NTHI477_RS01530	954	A	G	synonymous
309704	NTHI477_RS01530	936	T	A	synonymous
309706	NTHI477_RS01530	934	G	A	nonsynonymous
309743	NTHI477_RS01530	897	C	T	nonsynonymous
309746	NTHI477_RS01530	894	G	A	synonymous
309749	NTHI477_RS01530	891	G	T	synonymous
309749	NTHI477_RS01530	891	G	A	synonymous
309755	NTHI477_RS01530	885	G	A	synonymous
309767	NTHI477_RS01530	873	C	T	synonymous
309770	NTHI477_RS01530	870	G	A	synonymous
309773	NTHI477_RS01530	867	T	G	nonsynonymous
309774	NTHI477_RS01530	866	T	C	nonsynonymous
309776	NTHI477_RS01530	864	A	G	synonymous
309779	NTHI477_RS01530	861	A	G	synonymous
309782	NTHI477_RS01530	858	T	C	synonymous
309785	NTHI477_RS01530	855	C	T	synonymous
309797	NTHI477_RS01530	843	T	C	synonymous
309809	NTHI477_RS01530	831	G	C	synonymous
309812	NTHI477_RS01530	828	T	C	synonymous
309815	NTHI477_RS01530	825	A	T	synonymous

309830	NTHI477_RS01530	810	C	T	synonymous
309839	NTHI477_RS01530	801	G	A	synonymous
309866	NTHI477_RS01530	774	T	C	synonymous
309875	NTHI477_RS01530	765	G	A	synonymous
309887	NTHI477_RS01530	753	C	T	synonymous
309935	NTHI477_RS01530	705	T	C	synonymous
309938	NTHI477_RS01530	702	G	A	synonymous
309941	NTHI477_RS01530	699	T	A	synonymous
309944	NTHI477_RS01530	696	A	G	synonymous
309950	NTHI477_RS01530	690	C	T	synonymous
309953	NTHI477_RS01530	687	A	G	synonymous
309956	NTHI477_RS01530	684	G	T	synonymous
309974	NTHI477_RS01530	666	C	A	synonymous
309977	NTHI477_RS01530	663	G	A	synonymous
309992	NTHI477_RS01530	648	G	A	synonymous
309998	NTHI477_RS01530	642	A	G	synonymous
310007	NTHI477_RS01530	633	G	T	nonsynonymous
310010	NTHI477_RS01530	630	A	G	synonymous
310019	NTHI477_RS01530	621	G	A	synonymous
310028	NTHI477_RS01530	612	A	T	synonymous
310028	NTHI477_RS01530	612	A	G	synonymous
310031	NTHI477_RS01530	609	G	A	synonymous
310040	NTHI477_RS01530	600	A	G	synonymous
310052	NTHI477_RS01530	588	T	A	synonymous
310079	NTHI477_RS01530	561	A	T	synonymous
310097	NTHI477_RS01530	543	A	G	synonymous
310112	NTHI477_RS01530	528	A	C	synonymous
310118	NTHI477_RS01530	522	T	G	synonymous
310124	NTHI477_RS01530	516	C	T	synonymous
310127	NTHI477_RS01530	513	A	G	synonymous
310130	NTHI477_RS01530	510	G	A	synonymous
310133	NTHI477_RS01530	507	C	T	synonymous
310151	NTHI477_RS01530	489	C	A	synonymous
310199	NTHI477_RS01530	441	A	C	synonymous
310199	NTHI477_RS01530	441	A	T	synonymous
310201	NTHI477_RS01530	439	C	G	nonsynonymous
310202	NTHI477_RS01530	438	T	C	synonymous
310205	NTHI477_RS01530	435	A	G	synonymous
310211	NTHI477_RS01530	429	T	G	synonymous
310214	NTHI477_RS01530	426	G	A	synonymous
310235	NTHI477_RS01530	405	A	T	nonsynonymous
310241	NTHI477_RS01530	399	C	A	synonymous
310247	NTHI477_RS01530	393	C	T	synonymous
310253	NTHI477_RS01530	387	A	C	synonymous
310265	NTHI477_RS01530	375	A	G	synonymous

310277	NTHI477_RS01530	363	C	T	synonymous
310286	NTHI477_RS01530	354	C	G	synonymous
310294	NTHI477_RS01530	346	C	T	nonsynonymous
310301	NTHI477_RS01530	339	C	T	synonymous
310302	NTHI477_RS01530	338	G	A	nonsynonymous
310307	NTHI477_RS01530	333	G	A	synonymous
310310	NTHI477_RS01530	330	G	A	synonymous
310319	NTHI477_RS01530	321	T	C	synonymous
310337	NTHI477_RS01530	303	C	T	synonymous
310346	NTHI477_RS01530	294	A	G	synonymous
310349	NTHI477_RS01530	291	A	G	synonymous
310364	NTHI477_RS01530	276	A	G	synonymous
310367	NTHI477_RS01530	273	C	A	synonymous
310367	NTHI477_RS01530	273	C	G	synonymous
310373	NTHI477_RS01530	267	G	A	synonymous
310376	NTHI477_RS01530	264	A	G	synonymous
310382	NTHI477_RS01530	258	T	C	synonymous
310394	NTHI477_RS01530	246	G	A	synonymous
310403	NTHI477_RS01530	237	T	C	synonymous
310406	NTHI477_RS01530	234	T	A	synonymous
310415	NTHI477_RS01530	225	A	G	synonymous
310418	NTHI477_RS01530	222	G	A	synonymous
310433	NTHI477_RS01530	207	A	G	synonymous
310445	NTHI477_RS01530	195	G	A	synonymous
310457	NTHI477_RS01530	183	C	T	synonymous
310466	NTHI477_RS01530	174	A	G	synonymous
310469	NTHI477_RS01530	171	C	T	synonymous
310478	NTHI477_RS01530	162	A	C	synonymous
310481	NTHI477_RS01530	159	C	T	synonymous
310484	NTHI477_RS01530	156	T	G	synonymous
310487	NTHI477_RS01530	153	G	A	synonymous
310505	NTHI477_RS01530	135	A	G	synonymous
310592	NTHI477_RS01530	48	G	A	synonymous
310599	NTHI477_RS01530	41	C	T	nonsynonymous
310601	NTHI477_RS01530	39	G	A	synonymous
310610	NTHI477_RS01530	30	T	C	synonymous
310610	NTHI477_RS01530	30	T	A	nonsynonymous
310611	NTHI477_RS01530	29	T	G	nonsynonymous
310611	NTHI477_RS01530	29	T	A	nonsynonymous
310623	NTHI477_RS01530	17	G	A	nonsynonymous
310633	NTHI477_RS01530	7	G	A	nonsynonymous
310667	intergenic	intergenic	A	G	intergenic
310670	intergenic	intergenic	C	T	intergenic
310713	intergenic	intergenic	C	T	intergenic
310735	intergenic	intergenic	A	G	intergenic

310760	intergenic	intergenic	C	T	intergenic
310802	intergenic	intergenic	A	T	intergenic
310806	intergenic	intergenic	G	T	intergenic
310809	intergenic	intergenic	C	A	intergenic
310830	intergenic	intergenic	A	G	intergenic
310831	intergenic	intergenic	A	C	intergenic
310832	intergenic	intergenic	T	A	intergenic
310853	intergenic	intergenic	C	T	intergenic
310858	intergenic	intergenic	T	C	intergenic
310883	intergenic	intergenic	T	C	intergenic
310945	intergenic	intergenic	A	G	intergenic
310956	intergenic	intergenic	A	G	intergenic
311016	intergenic	intergenic	A	G	intergenic
311070	intergenic	intergenic	A	G	intergenic
311075	intergenic	intergenic	G	A	intergenic
311078	intergenic	intergenic	C	T	intergenic
311082	intergenic	intergenic	A	G	intergenic
311096	intergenic	intergenic	A	G	intergenic
311130	intergenic	intergenic	G	A	intergenic
311137	intergenic	intergenic	G	A	intergenic
311141	intergenic	intergenic	A	G	intergenic
311188	NTHI477_RS01535	1467	A	G	synonymous
311245	NTHI477_RS01535	1410	T	G	synonymous
311299	NTHI477_RS01535	1356	G	A	synonymous
311305	NTHI477_RS01535	1350	G	T	synonymous
311356	NTHI477_RS01535	1299	A	G	synonymous
311359	NTHI477_RS01535	1296	C	A	synonymous
311386	NTHI477_RS01535	1269	A	G	synonymous
311413	NTHI477_RS01535	1242	A	C	synonymous
311428	NTHI477_RS01535	1227	A	G	synonymous
311437	NTHI477_RS01535	1218	A	G	synonymous
311446	NTHI477_RS01535	1209	G	A	synonymous
311455	NTHI477_RS01535	1200	G	A	synonymous
311458	NTHI477_RS01535	1197	T	C	synonymous
311470	NTHI477_RS01535	1185	G	A	synonymous
311473	NTHI477_RS01535	1182	G	A	synonymous
311479	NTHI477_RS01535	1176	T	C	synonymous
311485	NTHI477_RS01535	1170	A	G	synonymous
311494	NTHI477_RS01535	1161	T	C	synonymous
311500	NTHI477_RS01535	1155	G	A	synonymous
311506	NTHI477_RS01535	1149	T	C	synonymous
311527	NTHI477_RS01535	1128	A	G	synonymous
311535	NTHI477_RS01535	1120	T	C	nonsynonymous
311551	NTHI477_RS01535	1104	T	C	synonymous
311568	NTHI477_RS01535	1087	G	C	nonsynonymous

311572	NTHI477_RS01535	1083	T	C	synonymous
311581	NTHI477_RS01535	1074	G	T	synonymous
311581	NTHI477_RS01535	1074	G	A	synonymous
311584	NTHI477_RS01535	1071	A	G	synonymous
311647	NTHI477_RS01535	1008	T	G	nonsynonymous
311649	NTHI477_RS01535	1006	A	G	synonymous
311656	NTHI477_RS01535	999	C	T	synonymous
311659	NTHI477_RS01535	996	A	G	synonymous
311674	NTHI477_RS01535	981	T	C	synonymous
311683	NTHI477_RS01535	972	G	A	synonymous
311755	NTHI477_RS01535	900	G	C	synonymous
311782	NTHI477_RS01535	873	G	A	synonymous
311785	NTHI477_RS01535	870	G	A	synonymous
311842	NTHI477_RS01535	813	G	A	synonymous
311854	NTHI477_RS01535	801	A	G	synonymous
311866	NTHI477_RS01535	789	G	A	synonymous
311866	NTHI477_RS01535	789	G	T	synonymous
311889	NTHI477_RS01535	766	C	T	nonsynonymous
311890	NTHI477_RS01535	765	G	A	synonymous
311895	NTHI477_RS01535	760	G	A	synonymous
311926	NTHI477_RS01535	729	T	C	synonymous
311938	NTHI477_RS01535	717	G	A	synonymous
311953	NTHI477_RS01535	702	A	G	synonymous
311989	NTHI477_RS01535	666	A	G	synonymous
311998	NTHI477_RS01535	657	A	G	synonymous
312004	NTHI477_RS01535	651	T	C	synonymous
312010	NTHI477_RS01535	645	T	G	synonymous
312019	NTHI477_RS01535	636	G	A	synonymous
312025	NTHI477_RS01535	630	A	G	synonymous
312043	NTHI477_RS01535	612	A	G	synonymous
312046	NTHI477_RS01535	609	T	C	synonymous
312058	NTHI477_RS01535	597	C	T	synonymous
312064	NTHI477_RS01535	591	A	G	synonymous
312070	NTHI477_RS01535	585	C	A	synonymous
312076	NTHI477_RS01535	579	T	C	synonymous
312079	NTHI477_RS01535	576	A	G	synonymous
312097	NTHI477_RS01535	558	A	G	synonymous
312154	NTHI477_RS01535	501	A	G	synonymous
312172	NTHI477_RS01535	483	A	T	synonymous
312181	NTHI477_RS01535	474	C	T	synonymous
312184	NTHI477_RS01535	471	C	T	synonymous
312184	NTHI477_RS01535	471	C	G	synonymous
312196	NTHI477_RS01535	459	G	T	synonymous
312198	NTHI477_RS01535	457	C	T	nonsynonymous
312199	NTHI477_RS01535	456	G	A	synonymous

312202	NTHI477_RS01535	453	C	T	synonymous
312220	NTHI477_RS01535	435	A	G	synonymous
312232	NTHI477_RS01535	423	G	A	synonymous
312250	NTHI477_RS01535	405	T	A	synonymous
312267	NTHI477_RS01535	388	A	G	nonsynonymous
312271	NTHI477_RS01535	384	T	C	synonymous
312283	NTHI477_RS01535	372	G	A	synonymous
312286	NTHI477_RS01535	369	A	G	synonymous
312289	NTHI477_RS01535	366	C	T	synonymous
312292	NTHI477_RS01535	363	T	G	synonymous
312301	NTHI477_RS01535	354	T	C	synonymous
312304	NTHI477_RS01535	351	A	G	synonymous
312304	NTHI477_RS01535	351	A	T	synonymous
312316	NTHI477_RS01535	339	T	A	synonymous
312319	NTHI477_RS01535	336	C	G	synonymous
312322	NTHI477_RS01535	333	C	A	nonsynonymous
312328	NTHI477_RS01535	327	C	T	synonymous
312334	NTHI477_RS01535	321	T	C	synonymous
312358	NTHI477_RS01535	297	T	A	synonymous
312376	NTHI477_RS01535	279	G	A	synonymous
312421	NTHI477_RS01535	234	C	T	synonymous
312424	NTHI477_RS01535	231	G	A	synonymous
312427	NTHI477_RS01535	228	A	G	synonymous
312436	NTHI477_RS01535	219	A	G	synonymous
312439	NTHI477_RS01535	216	A	C	synonymous
312439	NTHI477_RS01535	216	A	G	synonymous
312442	NTHI477_RS01535	213	G	A	synonymous
312478	NTHI477_RS01535	177	A	G	synonymous
312487	NTHI477_RS01535	168	C	A	synonymous
312496	NTHI477_RS01535	159	T	A	synonymous
312499	NTHI477_RS01535	156	T	A	synonymous
312499	NTHI477_RS01535	156	T	C	synonymous
312502	NTHI477_RS01535	153	T	C	synonymous
312504	NTHI477_RS01535	151	A	G	synonymous
312514	NTHI477_RS01535	141	G	C	synonymous
312532	NTHI477_RS01535	123	A	G	synonymous
312538	NTHI477_RS01535	117	G	A	synonymous
312577	NTHI477_RS01535	78	G	A	synonymous
312595	NTHI477_RS01535	60	G	T	synonymous
312600	NTHI477_RS01535	55	G	A	synonymous
312601	NTHI477_RS01535	54	C	T	synonymous
312616	NTHI477_RS01535	39	G	A	synonymous
312622	NTHI477_RS01535	33	C	T	synonymous
312643	NTHI477_RS01535	12	G	T	synonymous
312648	NTHI477_RS01535	7	C	A	nonsynonymous

312740	intergenic	intergenic	G	A	intergenic
312753	intergenic	intergenic	C	T	intergenic
312764	intergenic	intergenic	T	G	intergenic
312768	intergenic	intergenic	A	T	intergenic
312789	NTHI477_RS01540	20	G	C	nonsynonymous
312794	NTHI477_RS01540	25	A	G	nonsynonymous
312826	NTHI477_RS01540	57	G	A	synonymous
312841	NTHI477_RS01540	72	C	T	synonymous
312883	NTHI477_RS01540	114	T	C	synonymous
312901	NTHI477_RS01540	132	T	C	synonymous
312907	NTHI477_RS01540	138	T	G	synonymous
312907	NTHI477_RS01540	138	T	C	synonymous
312910	NTHI477_RS01540	141	T	C	synonymous
312919	NTHI477_RS01540	150	T	A	synonymous
312922	NTHI477_RS01540	153	A	T	synonymous
312928	NTHI477_RS01540	159	G	T	synonymous
312928	NTHI477_RS01540	159	G	A	synonymous
312940	NTHI477_RS01540	171	C	T	synonymous
312949	NTHI477_RS01540	180	T	C	synonymous
312961	NTHI477_RS01540	192	A	G	synonymous
313048	NTHI477_RS01540	279	G	C	synonymous
313072	NTHI477_RS01540	303	A	T	synonymous
313090	NTHI477_RS01540	321	G	A	synonymous
313090	NTHI477_RS01540	321	G	T	synonymous
313127	NTHI477_RS01540	358	T	C	nonsynonymous
313159	NTHI477_RS01540	390	C	T	synonymous
313162	NTHI477_RS01540	393	T	G	synonymous
313204	NTHI477_RS01540	435	C	A	synonymous
313210	NTHI477_RS01540	441	T	A	synonymous
313213	NTHI477_RS01540	444	C	G	synonymous
313213	NTHI477_RS01540	444	C	T	synonymous
313241	NTHI477_RS01540	472	G	A	nonsynonymous
313252	NTHI477_RS01540	483	C	T	synonymous
313255	NTHI477_RS01540	486	C	T	synonymous
313270	NTHI477_RS01540	501	C	T	synonymous
313276	NTHI477_RS01540	507	G	A	synonymous
313303	NTHI477_RS01540	534	A	T	synonymous
313309	NTHI477_RS01540	540	C	T	synonymous
313312	NTHI477_RS01540	543	C	T	synonymous
313361	NTHI477_RS01540	592	T	C	synonymous
313372	NTHI477_RS01540	603	C	T	synonymous
313390	NTHI477_RS01540	621	G	A	synonymous
313419	NTHI477_RS01540	650	C	T	nonsynonymous
313420	NTHI477_RS01540	651	A	C	synonymous
313422	NTHI477_RS01540	653	T	C	nonsynonymous

313424	NTHI477_RS01540	655	C	T	synonymous
313438	NTHI477_RS01540	669	G	A	synonymous
313441	NTHI477_RS01540	672	T	A	synonymous
313442	NTHI477_RS01540	673	G	A	nonsynonymous
313444	NTHI477_RS01540	675	C	A	synonymous
313445	NTHI477_RS01540	676	G	A	nonsynonymous
313447	NTHI477_RS01540	678	A	C	synonymous
313477	NTHI477_RS01540	708	T	A	synonymous
313501	NTHI477_RS01540	732	T	C	synonymous
313507	NTHI477_RS01540	738	G	T	synonymous
313507	NTHI477_RS01540	738	G	C	synonymous
313549	NTHI477_RS01540	780	A	G	synonymous
313552	NTHI477_RS01540	783	C	T	synonymous
313561	NTHI477_RS01540	792	T	G	synonymous
313564	NTHI477_RS01540	795	G	T	synonymous
313565	NTHI477_RS01540	796	G	T	nonsynonymous
313591	NTHI477_RS01540	822	A	T	synonymous
313597	NTHI477_RS01540	828	C	T	synonymous
313607	NTHI477_RS01540	838	A	G	nonsynonymous
313608	NTHI477_RS01540	839	C	G	nonsynonymous
313609	NTHI477_RS01540	840	A	T	synonymous
313627	NTHI477_RS01540	858	A	G	synonymous
313629	NTHI477_RS01540	860	G	C	nonsynonymous
313630	NTHI477_RS01540	861	G	A	synonymous
313630	NTHI477_RS01540	861	G	T	synonymous
313633	NTHI477_RS01540	864	T	C	synonymous
313675	NTHI477_RS01540	906	C	A	synonymous
313679	NTHI477_RS01540	910	C	T	nonsynonymous
313699	NTHI477_RS01540	930	C	T	synonymous
313703	NTHI477_RS01540	934	A	C	nonsynonymous
313704	NTHI477_RS01540	935	A	G	nonsynonymous
313705	NTHI477_RS01540	936	T	C	synonymous
313714	NTHI477_RS01540	945	G	A	synonymous
313717	NTHI477_RS01540	948	A	G	synonymous
313720	NTHI477_RS01540	951	A	T	synonymous
313730	NTHI477_RS01540	961	C	T	nonsynonymous
313732	NTHI477_RS01540	963	T	A	synonymous
313733	NTHI477_RS01540	964	G	A	nonsynonymous
313744	NTHI477_RS01540	975	G	A	synonymous
313749	NTHI477_RS01540	980	A	G	nonsynonymous
313750	NTHI477_RS01540	981	T	C	synonymous
313762	NTHI477_RS01540	993	G	A	synonymous
313777	NTHI477_RS01540	1008	C	A	synonymous
313777	NTHI477_RS01540	1008	C	T	synonymous
313783	NTHI477_RS01540	1014	C	T	synonymous

313789	NTHI477_RS01540	1020	C	T	synonymous
313792	NTHI477_RS01540	1023	T	A	synonymous
313810	NTHI477_RS01540	1041	T	C	synonymous
313813	NTHI477_RS01540	1044	T	G	synonymous
313819	NTHI477_RS01540	1050	A	G	synonymous
313828	NTHI477_RS01540	1059	T	A	synonymous
313828	NTHI477_RS01540	1059	T	G	synonymous
313831	NTHI477_RS01540	1062	A	T	synonymous
313840	NTHI477_RS01540	1071	C	A	synonymous
313843	NTHI477_RS01540	1074	T	A	synonymous
313855	NTHI477_RS01540	1086	T	C	synonymous
313876	NTHI477_RS01540	1107	C	G	nonsynonymous
313879	NTHI477_RS01540	1110	A	T	synonymous
313882	NTHI477_RS01540	1113	T	A	synonymous
313906	NTHI477_RS01540	1137	C	A	synonymous
313912	NTHI477_RS01540	1143	T	A	synonymous
313918	NTHI477_RS01540	1149	G	C	synonymous
313921	NTHI477_RS01540	1152	A	G	synonymous
313924	NTHI477_RS01540	1155	T	G	synonymous
313930	NTHI477_RS01540	1161	C	T	synonymous
313934	NTHI477_RS01540	1165	C	T	nonsynonymous
313942	NTHI477_RS01540	1173	C	T	synonymous
313984	NTHI477_RS01540	1215	A	C	synonymous
313990	NTHI477_RS01540	1221	T	C	synonymous
313999	NTHI477_RS01540	1230	G	T	synonymous
314002	NTHI477_RS01540	1233	G	T	synonymous
314002	NTHI477_RS01540	1233	G	A	synonymous
314014	NTHI477_RS01540	1245	A	G	synonymous
314020	NTHI477_RS01540	1251	C	T	synonymous
314023	NTHI477_RS01540	1254	T	A	synonymous
314026	NTHI477_RS01540	1257	T	G	synonymous
314066	NTHI477_RS01540	1297	G	A	nonsynonymous
314068	NTHI477_RS01540	1299	T	C	synonymous
314077	NTHI477_RS01540	1308	G	C	synonymous
314077	NTHI477_RS01540	1308	G	A	synonymous
314089	NTHI477_RS01540	1320	A	T	synonymous
314092	NTHI477_RS01540	1323	T	C	synonymous
314098	NTHI477_RS01540	1329	G	A	synonymous
314101	NTHI477_RS01540	1332	A	C	synonymous
314131	NTHI477_RS01540	1362	T	G	synonymous
314137	NTHI477_RS01540	1368	T	C	synonymous
314167	NTHI477_RS01540	1398	A	T	synonymous
314170	NTHI477_RS01540	1401	C	T	synonymous
314304	NTHI477_RS01545	24	T	C	synonymous
314316	NTHI477_RS01545	36	T	G	synonymous

314319	NTHI477_RS01545	39	T	C	synonymous
314331	NTHI477_RS01545	51	A	G	synonymous
314343	NTHI477_RS01545	63	G	A	synonymous
314344	NTHI477_RS01545	64	A	G	nonsynonymous
314352	NTHI477_RS01545	72	A	G	synonymous
314354	NTHI477_RS01545	74	T	G	nonsynonymous
314354	NTHI477_RS01545	74	T	A	nonsynonymous
314361	NTHI477_RS01545	81	G	A	synonymous
314367	NTHI477_RS01545	87	A	G	synonymous
314375	NTHI477_RS01545	95	C	A	nonsynonymous
314385	NTHI477_RS01545	105	T	C	synonymous
314387	NTHI477_RS01545	107	G	C	nonsynonymous
314388	NTHI477_RS01545	108	C	T	synonymous
314416	NTHI477_RS01545	136	G	A	nonsynonymous
314422	NTHI477_RS01545	142	C	T	synonymous
314424	NTHI477_RS01545	144	A	G	synonymous
314427	NTHI477_RS01545	147	T	A	synonymous
314428	NTHI477_RS01545	148	A	G	nonsynonymous
314436	NTHI477_RS01545	156	T	A	nonsynonymous
314442	NTHI477_RS01545	162	T	C	synonymous
314443	NTHI477_RS01545	163	G	A	nonsynonymous
314448	NTHI477_RS01545	168	C	T	synonymous
314454	NTHI477_RS01545	174	A	C	synonymous
314466	NTHI477_RS01545	186	G	A	synonymous
314483	NTHI477_RS01545	203	C	T	nonsynonymous
314486	NTHI477_RS01545	206	C	T	nonsynonymous
314494	NTHI477_RS01545	214	G	A	nonsynonymous
314495	NTHI477_RS01545	215	A	C	nonsynonymous
314499	NTHI477_RS01545	219	T	A	synonymous
314529	NTHI477_RS01545	249	A	G	synonymous
314553	NTHI477_RS01545	273	G	A	synonymous
314568	NTHI477_RS01545	288	C	T	synonymous
314571	NTHI477_RS01545	291	C	T	synonymous
314578	NTHI477_RS01545	298	G	A	nonsynonymous
314580	NTHI477_RS01545	300	A	T	synonymous
314599	NTHI477_RS01545	319	G	A	nonsynonymous
314628	NTHI477_RS01545	348	C	T	synonymous
314631	NTHI477_RS01545	351	G	A	synonymous
314632	NTHI477_RS01545	352	T	G	nonsynonymous
314634	NTHI477_RS01545	354	G	T	synonymous
314637	NTHI477_RS01545	357	C	A	synonymous
314637	NTHI477_RS01545	357	C	T	synonymous
314638	NTHI477_RS01545	358	T	C	synonymous
314640	NTHI477_RS01545	360	G	A	synonymous
314646	NTHI477_RS01545	366	T	G	synonymous

314646	NTHI477_RS01545	366	T	C	synonymous
314648	NTHI477_RS01545	368	G	A	nonsynonymous
314661	NTHI477_RS01545	381	C	T	synonymous
314664	NTHI477_RS01545	384	G	A	synonymous
314667	NTHI477_RS01545	387	T	C	synonymous
314676	NTHI477_RS01545	396	C	T	synonymous
314703	NTHI477_RS01545	423	C	T	synonymous
314703	NTHI477_RS01545	423	C	G	synonymous
314709	NTHI477_RS01545	429	C	T	synonymous
314721	NTHI477_RS01545	441	G	C	synonymous
314724	NTHI477_RS01545	444	C	A	synonymous
314739	NTHI477_RS01545	459	C	T	synonymous
314766	NTHI477_RS01545	486	T	A	synonymous
314775	NTHI477_RS01545	495	A	G	synonymous
314778	NTHI477_RS01545	498	G	A	synonymous
314796	NTHI477_RS01545	516	A	G	synonymous
314811	NTHI477_RS01545	531	G	A	synonymous
314811	NTHI477_RS01545	531	G	C	synonymous
314814	NTHI477_RS01545	534	T	A	synonymous
314826	NTHI477_RS01545	546	A	G	synonymous
314841	NTHI477_RS01545	561	G	A	synonymous
314850	NTHI477_RS01545	570	C	T	synonymous
314889	NTHI477_RS01545	609	C	G	synonymous
314910	NTHI477_RS01545	630	G	A	synonymous
314937	NTHI477_RS01545	657	C	T	synonymous
314955	NTHI477_RS01545	675	A	C	synonymous
314955	NTHI477_RS01545	675	A	G	synonymous
314961	NTHI477_RS01545	681	G	A	synonymous
314964	NTHI477_RS01545	684	G	A	synonymous
314976	NTHI477_RS01545	696	A	C	synonymous
314985	NTHI477_RS01545	705	G	A	synonymous
314988	NTHI477_RS01545	708	C	T	synonymous
314989	NTHI477_RS01545	709	G	A	nonsynonymous
314994	NTHI477_RS01545	714	A	G	synonymous
314995	NTHI477_RS01545	715	G	A	nonsynonymous
314996	NTHI477_RS01545	716	C	T	nonsynonymous
315009	NTHI477_RS01545	729	G	A	synonymous
315033	NTHI477_RS01545	753	C	T	synonymous
315045	NTHI477_RS01545	765	C	T	synonymous
315054	NTHI477_RS01545	774	T	C	synonymous
315057	NTHI477_RS01545	777	C	T	synonymous
315084	NTHI477_RS01545	804	C	T	synonymous
315087	NTHI477_RS01545	807	A	G	synonymous
315088	NTHI477_RS01545	808	T	C	nonsynonymous
315093	NTHI477_RS01545	813	T	C	synonymous

315096	NTHI477_RS01545	816	T	G	synonymous
315111	NTHI477_RS01545	831	T	C	synonymous
315144	NTHI477_RS01545	864	A	G	synonymous
315168	NTHI477_RS01545	888	T	C	synonymous
315175	NTHI477_RS01545	895	C	T	nonsynonymous
315180	NTHI477_RS01545	900	C	T	synonymous
315188	NTHI477_RS01545	908	G	A	nonsynonymous
315204	NTHI477_RS01545	924	T	C	synonymous
315237	NTHI477_RS01545	957	G	A	synonymous
315240	NTHI477_RS01545	960	C	T	synonymous
315253	intergenic	intergenic	G	A	intergenic
315261	intergenic	intergenic	C	T	intergenic
315263	intergenic	intergenic	C	T	intergenic
315272	intergenic	intergenic	G	T	intergenic
315280	intergenic	intergenic	G	A	intergenic
315287	intergenic	intergenic	A	C	intergenic
315292	NTHI477_RS01550	876	T	C	synonymous
315295	NTHI477_RS01550	873	A	C	synonymous
315309	NTHI477_RS01550	859	G	A	synonymous
315312	NTHI477_RS01550	856	C	T	nonsynonymous
315316	NTHI477_RS01550	852	C	T	synonymous
315316	NTHI477_RS01550	852	C	A	synonymous
315349	NTHI477_RS01550	819	C	T	synonymous
315361	NTHI477_RS01550	807	C	T	synonymous
315363	NTHI477_RS01550	805	A	G	synonymous
315388	NTHI477_RS01550	780	A	T	synonymous
315394	NTHI477_RS01550	774	C	T	synonymous
315413	NTHI477_RS01550	755	C	T	nonsynonymous
315499	NTHI477_RS01550	669	T	C	synonymous
315504	NTHI477_RS01550	664	C	T	nonsynonymous
315508	NTHI477_RS01550	660	T	A	synonymous
315538	NTHI477_RS01550	630	T	C	synonymous
315574	NTHI477_RS01550	594	C	T	synonymous
315594	NTHI477_RS01550	574	C	T	nonsynonymous
315598	NTHI477_RS01550	570	A	G	synonymous
315599	NTHI477_RS01550	569	C	T	nonsynonymous
315607	NTHI477_RS01550	561	A	G	synonymous
315613	NTHI477_RS01550	555	A	T	synonymous
315618	NTHI477_RS01550	550	C	T	nonsynonymous
315634	NTHI477_RS01550	534	T	A	nonsynonymous
315661	NTHI477_RS01550	507	A	G	synonymous
315673	NTHI477_RS01550	495	T	C	synonymous
315685	NTHI477_RS01550	483	A	C	synonymous
315685	NTHI477_RS01550	483	A	T	synonymous
315703	NTHI477_RS01550	465	T	C	synonymous

315747	NTHI477_RS01550	421	C	T	nonsynonymous
315748	NTHI477_RS01550	420	A	T	synonymous
315749	NTHI477_RS01550	419	C	G	nonsynonymous
315862	NTHI477_RS01550	306	C	T	synonymous
315910	NTHI477_RS01550	258	G	A	synonymous
315925	NTHI477_RS01550	243	C	A	synonymous
315934	NTHI477_RS01550	234	G	A	synonymous
315946	NTHI477_RS01550	222	T	G	synonymous
315973	NTHI477_RS01550	195	A	G	synonymous
315979	NTHI477_RS01550	189	A	G	synonymous
315988	NTHI477_RS01550	180	T	C	synonymous
315991	NTHI477_RS01550	177	A	T	synonymous
315992	NTHI477_RS01550	176	G	A	nonsynonymous
315996	NTHI477_RS01550	172	G	T	nonsynonymous
315997	NTHI477_RS01550	171	T	C	synonymous
316006	NTHI477_RS01550	162	G	A	synonymous
316015	NTHI477_RS01550	153	C	T	synonymous
316023	NTHI477_RS01550	145	G	A	synonymous
316027	NTHI477_RS01550	141	G	T	synonymous
316041	NTHI477_RS01550	127	T	C	nonsynonymous
316043	NTHI477_RS01550	125	T	C	nonsynonymous
316056	NTHI477_RS01550	112	G	C	nonsynonymous
316067	NTHI477_RS01550	101	T	C	nonsynonymous
316068	NTHI477_RS01550	100	A	G	nonsynonymous
316078	NTHI477_RS01550	90	A	G	synonymous
316093	NTHI477_RS01550	75	T	C	synonymous
316094	NTHI477_RS01550	74	G	A	nonsynonymous
316145	NTHI477_RS01550	23	G	A	nonsynonymous
316148	NTHI477_RS01550	20	C	T	nonsynonymous
316200	intergenic	intergenic	A	C	intergenic
316226	intergenic	intergenic	T	C	intergenic
316233	intergenic	intergenic	C	T	intergenic
316234	intergenic	intergenic	C	T	intergenic
316246	intergenic	intergenic	A	G	intergenic
316264	intergenic	intergenic	C	T	intergenic
316292	intergenic	intergenic	T	C	intergenic
316318	intergenic	intergenic	A	G	intergenic
316323	intergenic	intergenic	G	A	intergenic
316324	intergenic	intergenic	C	A	intergenic
316324	intergenic	intergenic	C	T	intergenic
316327	intergenic	intergenic	A	G	intergenic
316329	intergenic	intergenic	A	G	intergenic
316362	intergenic	intergenic	T	G	intergenic
316376	intergenic	intergenic	C	G	intergenic
316399	intergenic	intergenic	A	G	intergenic

316542	NTHI477_RS01555	102	A	G	synonymous
316545	NTHI477_RS01555	105	G	A	synonymous
316551	NTHI477_RS01555	111	C	T	synonymous
316560	NTHI477_RS01555	120	C	T	synonymous
316777	NTHI477_RS01555	337	A	G	nonsynonymous
316807	NTHI477_RS01555	367	G	T	nonsynonymous
316902	NTHI477_RS01555	462	G	T	synonymous
316946	intergenic	intergenic	G	A	intergenic
316970	intergenic	intergenic	C	T	intergenic
316986	intergenic	intergenic	A	T	intergenic
317006	intergenic	intergenic	C	T	intergenic
317017	intergenic	intergenic	T	C	intergenic
317058	intergenic	intergenic	G	A	intergenic
317100	intergenic	intergenic	C	A	intergenic
317106	intergenic	intergenic	C	T	intergenic
317134	intergenic	intergenic	C	T	intergenic
317141	intergenic	intergenic	G	T	intergenic
317154	intergenic	intergenic	A	T	intergenic
317156	intergenic	intergenic	G	A	intergenic
317160	intergenic	intergenic	T	G	intergenic
317162	intergenic	intergenic	T	G	intergenic
317165	intergenic	intergenic	A	T	intergenic
317166	intergenic	intergenic	A	G	intergenic
317166	intergenic	intergenic	A	T	intergenic
317166	intergenic	intergenic	A	C	intergenic
317168	intergenic	intergenic	C	A	intergenic
317171	intergenic	intergenic	T	C	intergenic
317172	intergenic	intergenic	A	G	intergenic
317172	intergenic	intergenic	A	C	intergenic
317187	intergenic	intergenic	A	C	intergenic
317190	intergenic	intergenic	A	C	intergenic
317194	intergenic	intergenic	A	C	intergenic
317195	intergenic	intergenic	T	G	intergenic
317201	intergenic	intergenic	A	C	intergenic
317202	intergenic	intergenic	G	T	intergenic
317207	intergenic	intergenic	A	C	intergenic
317211	intergenic	intergenic	A	C	intergenic
317214	intergenic	intergenic	G	T	intergenic
317224	intergenic	intergenic	A	G	intergenic
317232	intergenic	intergenic	A	T	intergenic
317239	intergenic	intergenic	C	G	intergenic
317244	intergenic	intergenic	C	T	intergenic
317245	intergenic	intergenic	C	A	intergenic
317264	intergenic	intergenic	C	T	intergenic
317271	intergenic	intergenic	C	A	intergenic

317277	intergenic	intergenic	G	A	intergenic
317278	intergenic	intergenic	G	T	intergenic
317323	intergenic	intergenic	C	T	intergenic
317331	intergenic	intergenic	A	G	intergenic
317334	intergenic	intergenic	T	G	intergenic
317345	intergenic	intergenic	C	A	intergenic
317346	intergenic	intergenic	T	G	intergenic
317349	intergenic	intergenic	A	G	intergenic
317361	intergenic	intergenic	A	G	intergenic
317363	intergenic	intergenic	C	T	intergenic
317381	intergenic	intergenic	G	T	intergenic
317454	NTHI477_RS01560	1184	G	A	nonsynonymous
317517	NTHI477_RS01560	1121	G	A	nonsynonymous
317534	NTHI477_RS01560	1104	A	T	synonymous
317547	NTHI477_RS01560	1091	T	C	nonsynonymous
317592	NTHI477_RS01560	1046	C	T	nonsynonymous
317607	NTHI477_RS01560	1031	A	G	nonsynonymous
317706	NTHI477_RS01560	932	C	T	nonsynonymous
317821	NTHI477_RS01560	817	G	A	synonymous
317919	NTHI477_RS01560	719	C	T	nonsynonymous
317946	NTHI477_RS01560	692	A	C	nonsynonymous
318024	NTHI477_RS01560	614	T	C	nonsynonymous
318191	NTHI477_RS01560	447	T	C	synonymous
318194	NTHI477_RS01560	444	C	T	synonymous
318200	NTHI477_RS01560	438	A	G	synonymous
318203	NTHI477_RS01560	435	C	A	synonymous
318345	NTHI477_RS01560	293	G	A	nonsynonymous
318347	NTHI477_RS01560	291	G	A	synonymous
318371	NTHI477_RS01560	267	T	C	synonymous
318377	NTHI477_RS01560	261	C	T	synonymous
318487	NTHI477_RS01560	151	G	A	nonsynonymous
318506	NTHI477_RS01560	132	C	T	synonymous
318533	NTHI477_RS01560	105	G	A	synonymous
318536	NTHI477_RS01560	102	T	A	synonymous
318542	NTHI477_RS01560	96	A	G	synonymous
318559	NTHI477_RS01560	79	G	T	nonsynonymous
318615	NTHI477_RS01560	23	G	A	nonsynonymous
318616	NTHI477_RS01560	22	T	C	nonsynonymous
318735	intergenic	intergenic	T	A	intergenic
318740	intergenic	intergenic	G	A	intergenic
318750	intergenic	intergenic	T	G	intergenic
318775	intergenic	intergenic	C	T	intergenic
318776	intergenic	intergenic	G	T	intergenic
318779	intergenic	intergenic	A	G	intergenic
318798	intergenic	intergenic	C	T	intergenic

318805	intergenic	intergenic	T	C	intergenic
318821	intergenic	intergenic	C	T	intergenic
318827	intergenic	intergenic	A	G	intergenic
318838	intergenic	intergenic	G	A	intergenic
318839	intergenic	intergenic	T	G	intergenic
318841	intergenic	intergenic	G	A	intergenic
318946	NTHI477_RS01565	14	T	C	nonsynonymous
318956	NTHI477_RS01565	24	G	T	nonsynonymous
318973	NTHI477_RS01565	41	G	A	nonsynonymous
319007	NTHI477_RS01565	75	T	C	synonymous
319013	NTHI477_RS01565	81	T	C	synonymous
319026	NTHI477_RS01565	94	A	G	nonsynonymous
319064	NTHI477_RS01565	132	T	C	synonymous
319079	NTHI477_RS01565	147	A	G	synonymous
319094	NTHI477_RS01565	162	C	A	synonymous
319103	NTHI477_RS01565	171	T	A	synonymous
319163	NTHI477_RS01565	231	G	A	synonymous
319166	NTHI477_RS01565	234	T	A	synonymous
319208	NTHI477_RS01565	276	G	A	synonymous
319247	NTHI477_RS01565	315	C	T	synonymous
319274	NTHI477_RS01565	342	C	T	synonymous
319319	NTHI477_RS01565	387	A	G	synonymous
319334	NTHI477_RS01565	402	T	G	synonymous
319382	NTHI477_RS01565	450	C	T	synonymous
319391	NTHI477_RS01565	459	C	T	synonymous
319520	NTHI477_RS01565	588	C	T	synonymous
319544	NTHI477_RS01565	612	G	A	synonymous
319565	NTHI477_RS01565	633	T	C	synonymous
319568	NTHI477_RS01565	636	A	G	synonymous
319574	NTHI477_RS01565	642	G	A	synonymous
319604	NTHI477_RS01565	672	T	C	synonymous
319628	NTHI477_RS01565	696	A	T	synonymous
319715	NTHI477_RS01565	783	G	C	synonymous
319716	NTHI477_RS01565	784	G	A	nonsynonymous
319796	NTHI477_RS01565	864	A	C	synonymous
319856	NTHI477_RS01565	924	T	C	synonymous
320400	NTHI477_RS01570	366	G	A	synonymous
320403	NTHI477_RS01570	369	A	G	synonymous
320462	NTHI477_RS01570	428	C	T	nonsynonymous
320462	NTHI477_RS01570	428	C	A	nonsynonymous
320523	NTHI477_RS01570	489	G	A	synonymous
320569	NTHI477_RS01570	535	A	C	nonsynonymous
320586	NTHI477_RS01570	552	A	G	synonymous
320595	NTHI477_RS01570	561	T	C	synonymous
320601	NTHI477_RS01570	567	C	A	synonymous

320643	NTHI477_RS01570	609	A	G	synonymous
320652	NTHI477_RS01570	618	A	T	synonymous
320655	NTHI477_RS01570	621	C	T	synonymous
320664	NTHI477_RS01570	630	A	G	synonymous
320685	NTHI477_RS01570	651	A	G	synonymous
320703	NTHI477_RS01570	669	T	C	synonymous
320803	NTHI477_RS01570	769	G	A	nonsynonymous
320856	NTHI477_RS01570	822	C	A	synonymous
320859	NTHI477_RS01570	825	A	G	synonymous
320862	NTHI477_RS01570	828	C	T	synonymous
320871	NTHI477_RS01570	837	G	A	synonymous
320883	NTHI477_RS01570	849	A	G	synonymous
320907	NTHI477_RS01575	13	C	A	nonsynonymous
321182	NTHI477_RS01575	288	C	T	synonymous
321252	NTHI477_RS01575	358	G	A	nonsynonymous
321404	NTHI477_RS01575	510	T	G	synonymous
321506	NTHI477_RS01575	612	T	C	synonymous
321512	NTHI477_RS01575	618	T	C	synonymous
321563	NTHI477_RS01575	669	C	T	synonymous
321599	NTHI477_RS01575	705	T	C	synonymous
321752	intergenic	intergenic	G	A	intergenic
321920	NTHI477_RS01580	126	C	A	synonymous
321932	NTHI477_RS01580	138	A	T	synonymous
321941	NTHI477_RS01580	147	A	C	synonymous
322028	NTHI477_RS01580	234	A	T	synonymous
322229	NTHI477_RS01580	435	G	A	synonymous
322232	NTHI477_RS01580	438	G	A	synonymous
322269	NTHI477_RS01580	475	C	G	nonsynonymous
322301	NTHI477_RS01580	507	T	A	synonymous
322322	NTHI477_RS01580	528	G	A	synonymous
322337	NTHI477_RS01580	543	G	A	synonymous
322340	NTHI477_RS01580	546	C	T	synonymous
322481	NTHI477_RS01580	687	G	A	synonymous
322487	NTHI477_RS01580	693	C	T	synonymous
322490	NTHI477_RS01580	696	A	T	synonymous
322493	NTHI477_RS01580	699	C	T	synonymous
322505	NTHI477_RS01580	711	G	A	synonymous
322557	NTHI477_RS01580	763	T	C	synonymous
322583	NTHI477_RS01580	789	T	G	synonymous
322604	NTHI477_RS01580	810	A	T	synonymous
322618	NTHI477_RS01580	824	T	C	nonsynonymous
322632	NTHI477_RS01580	838	C	T	nonsynonymous
322640	NTHI477_RS01580	846	C	T	synonymous
322881	intergenic	intergenic	G	A	intergenic
322884	intergenic	intergenic	A	T	intergenic

322892	intergenic	intergenic	A	C	intergenic
322945	intergenic	intergenic	C	A	intergenic
322975	NTHI477_RS01585	1927	C	T	nonsynonymous
322994	NTHI477_RS01585	1908	T	C	synonymous
323042	NTHI477_RS01585	1860	T	C	synonymous
323049	NTHI477_RS01585	1853	G	T	nonsynonymous
323081	NTHI477_RS01585	1821	T	C	synonymous
323094	NTHI477_RS01585	1808	G	T	nonsynonymous
323126	NTHI477_RS01585	1776	G	A	synonymous
323143	NTHI477_RS01585	1759	C	T	nonsynonymous
323171	NTHI477_RS01585	1731	T	C	synonymous
323174	NTHI477_RS01585	1728	T	A	synonymous
323226	NTHI477_RS01585	1676	T	C	nonsynonymous
323270	NTHI477_RS01585	1632	C	T	synonymous
323288	NTHI477_RS01585	1614	C	T	synonymous
323294	NTHI477_RS01585	1608	G	A	synonymous
323303	NTHI477_RS01585	1599	A	G	synonymous
323339	NTHI477_RS01585	1563	T	A	synonymous
323358	NTHI477_RS01585	1544	T	C	nonsynonymous
323365	NTHI477_RS01585	1537	C	T	nonsynonymous
323390	NTHI477_RS01585	1512	C	G	synonymous
323417	NTHI477_RS01585	1485	A	G	synonymous
323450	NTHI477_RS01585	1452	C	A	synonymous
323533	NTHI477_RS01585	1369	G	T	nonsynonymous
323537	NTHI477_RS01585	1365	C	T	synonymous
323540	NTHI477_RS01585	1362	C	T	synonymous
323552	NTHI477_RS01585	1350	T	C	synonymous
323595	NTHI477_RS01585	1307	G	A	nonsynonymous
323599	NTHI477_RS01585	1303	T	G	nonsynonymous
323603	NTHI477_RS01585	1299	T	A	synonymous
323609	NTHI477_RS01585	1293	T	G	synonymous
323624	NTHI477_RS01585	1278	C	A	nonsynonymous
323642	NTHI477_RS01585	1260	C	T	synonymous
323645	NTHI477_RS01585	1257	A	G	synonymous
323648	NTHI477_RS01585	1254	G	A	synonymous
323663	NTHI477_RS01585	1239	G	A	synonymous
323665	NTHI477_RS01585	1237	C	T	nonsynonymous
323681	NTHI477_RS01585	1221	G	A	synonymous
323687	NTHI477_RS01585	1215	C	T	synonymous
323705	NTHI477_RS01585	1197	A	G	synonymous
323714	NTHI477_RS01585	1188	A	G	synonymous
323732	NTHI477_RS01585	1170	G	A	synonymous
323738	NTHI477_RS01585	1164	G	A	synonymous
323753	NTHI477_RS01585	1149	A	C	nonsynonymous
323762	NTHI477_RS01585	1140	A	G	synonymous

323774	NTHI477_RS01585	1128	G	A	synonymous
323780	NTHI477_RS01585	1122	G	T	synonymous
323789	NTHI477_RS01585	1113	T	A	synonymous
323801	NTHI477_RS01585	1101	A	C	synonymous
323819	NTHI477_RS01585	1083	G	A	synonymous
323844	NTHI477_RS01585	1058	G	A	nonsynonymous
323845	NTHI477_RS01585	1057	G	C	nonsynonymous
323855	NTHI477_RS01585	1047	C	T	synonymous
323855	NTHI477_RS01585	1047	C	G	synonymous
323864	NTHI477_RS01585	1038	G	A	synonymous
323991	NTHI477_RS01585	911	A	G	nonsynonymous
324002	NTHI477_RS01585	900	A	C	nonsynonymous
324002	NTHI477_RS01585	900	A	G	synonymous
324026	NTHI477_RS01585	876	A	C	synonymous
324044	NTHI477_RS01585	858	G	T	synonymous
324068	NTHI477_RS01585	834	A	G	synonymous
324098	NTHI477_RS01585	804	T	C	synonymous
324113	NTHI477_RS01585	789	A	T	synonymous
324116	NTHI477_RS01585	786	T	A	synonymous
324137	NTHI477_RS01585	765	T	C	synonymous
324151	NTHI477_RS01585	751	C	T	nonsynonymous
324161	NTHI477_RS01585	741	T	G	nonsynonymous
324192	NTHI477_RS01585	710	T	C	nonsynonymous
324197	NTHI477_RS01585	705	T	C	synonymous
324198	NTHI477_RS01585	704	A	G	nonsynonymous
324207	NTHI477_RS01585	695	T	A	nonsynonymous
324208	NTHI477_RS01585	694	G	C	nonsynonymous
324209	NTHI477_RS01585	693	C	T	synonymous
324217	NTHI477_RS01585	685	C	T	nonsynonymous
324236	NTHI477_RS01585	666	G	C	synonymous
324286	NTHI477_RS01585	616	G	T	nonsynonymous
324287	NTHI477_RS01585	615	G	A	synonymous
324329	NTHI477_RS01585	573	A	G	synonymous
324335	NTHI477_RS01585	567	C	T	synonymous
324341	NTHI477_RS01585	561	T	C	synonymous
324350	NTHI477_RS01585	552	G	A	synonymous
324362	NTHI477_RS01585	540	A	G	synonymous
324386	NTHI477_RS01585	516	T	C	synonymous
324395	NTHI477_RS01585	507	G	A	synonymous
324401	NTHI477_RS01585	501	G	T	synonymous
324404	NTHI477_RS01585	498	A	T	synonymous
324413	NTHI477_RS01585	489	G	A	synonymous
324458	NTHI477_RS01585	444	C	T	synonymous
324476	NTHI477_RS01585	426	C	T	synonymous
324512	NTHI477_RS01585	390	A	G	synonymous

324528	NTHI477_RS01585	374	T	G	nonsynonymous
324544	NTHI477_RS01585	358	G	A	synonymous
324593	NTHI477_RS01585	309	C	A	nonsynonymous
324629	NTHI477_RS01585	273	G	T	synonymous
324629	NTHI477_RS01585	273	G	A	synonymous
324632	NTHI477_RS01585	270	A	G	synonymous
324638	NTHI477_RS01585	264	C	T	synonymous
324660	NTHI477_RS01585	242	C	T	nonsynonymous
324665	NTHI477_RS01585	237	C	T	synonymous
324675	NTHI477_RS01585	227	G	A	nonsynonymous
324785	NTHI477_RS01585	117	G	A	synonymous
324809	NTHI477_RS01585	93	A	G	synonymous
324845	NTHI477_RS01585	57	C	T	synonymous
324854	NTHI477_RS01585	48	T	C	synonymous
324855	NTHI477_RS01585	47	G	A	nonsynonymous
324860	NTHI477_RS01585	42	A	G	synonymous
324872	NTHI477_RS01585	30	G	A	synonymous
324903	intergenic	intergenic	G	A	intergenic
324907	intergenic	intergenic	C	T	intergenic
324976	intergenic	intergenic	A	G	intergenic
324985	intergenic	intergenic	C	A	intergenic
324986	intergenic	intergenic	T	G	intergenic
324989	intergenic	intergenic	C	T	intergenic
325000	NTHI477_RS01590	1351	G	C	nonsynonymous
325013	NTHI477_RS01590	1338	C	T	synonymous
325016	NTHI477_RS01590	1335	C	T	synonymous
325019	NTHI477_RS01590	1332	T	G	synonymous
325025	NTHI477_RS01590	1326	T	C	synonymous
325031	NTHI477_RS01590	1320	T	C	synonymous
325037	NTHI477_RS01590	1314	G	A	synonymous
325061	NTHI477_RS01590	1290	T	C	synonymous
325064	NTHI477_RS01590	1287	C	T	synonymous
325072	NTHI477_RS01590	1279	C	T	nonsynonymous
325078	NTHI477_RS01590	1273	T	C	nonsynonymous
325079	NTHI477_RS01590	1272	T	A	synonymous
325082	NTHI477_RS01590	1269	T	G	synonymous
325101	NTHI477_RS01590	1250	T	G	nonsynonymous
325103	NTHI477_RS01590	1248	T	A	synonymous
325111	NTHI477_RS01590	1240	T	G	synonymous
325112	NTHI477_RS01590	1239	T	C	synonymous
325112	NTHI477_RS01590	1239	T	A	synonymous
325127	NTHI477_RS01590	1224	C	T	synonymous
325145	NTHI477_RS01590	1206	C	T	synonymous
325160	NTHI477_RS01590	1191	T	C	synonymous
325193	NTHI477_RS01590	1158	G	A	synonymous

325199	NTHI477_RS01590	1152	C	T	synonymous
325226	NTHI477_RS01590	1125	G	A	synonymous
325247	NTHI477_RS01590	1104	C	A	synonymous
325247	NTHI477_RS01590	1104	C	T	synonymous
325251	NTHI477_RS01590	1100	G	A	nonsynonymous
325252	NTHI477_RS01590	1099	C	T	nonsynonymous
325267	NTHI477_RS01590	1084	G	A	nonsynonymous
325267	NTHI477_RS01590	1084	G	C	nonsynonymous
325268	NTHI477_RS01590	1083	C	T	synonymous
325270	NTHI477_RS01590	1081	A	G	synonymous
325271	NTHI477_RS01590	1080	C	T	synonymous
325361	NTHI477_RS01590	990	C	G	synonymous
325364	NTHI477_RS01590	987	C	T	synonymous
325370	NTHI477_RS01590	981	G	T	synonymous
325373	NTHI477_RS01590	978	C	T	synonymous
325388	NTHI477_RS01590	963	C	A	synonymous
325424	NTHI477_RS01590	927	A	T	nonsynonymous
325427	NTHI477_RS01590	924	T	C	synonymous
325439	NTHI477_RS01590	912	C	T	synonymous
325439	NTHI477_RS01590	912	C	A	synonymous
325451	NTHI477_RS01590	900	C	T	synonymous
325457	NTHI477_RS01590	894	A	G	synonymous
325458	NTHI477_RS01590	893	A	G	nonsynonymous
325459	NTHI477_RS01590	892	C	A	nonsynonymous
325487	NTHI477_RS01590	864	T	G	synonymous
325492	NTHI477_RS01590	859	C	T	nonsynonymous
325499	NTHI477_RS01590	852	T	C	synonymous
325505	NTHI477_RS01590	846	C	G	synonymous
325523	NTHI477_RS01590	828	T	C	synonymous
325529	NTHI477_RS01590	822	A	G	synonymous
325535	NTHI477_RS01590	816	A	G	synonymous
325544	NTHI477_RS01590	807	C	T	synonymous
325562	NTHI477_RS01590	789	G	A	synonymous
325570	NTHI477_RS01590	781	G	A	synonymous
325571	NTHI477_RS01590	780	T	C	synonymous
325571	NTHI477_RS01590	780	T	A	synonymous
325580	NTHI477_RS01590	771	C	A	synonymous
325580	NTHI477_RS01590	771	C	G	synonymous
325582	NTHI477_RS01590	769	G	T	synonymous
325583	NTHI477_RS01590	768	A	C	synonymous
325586	NTHI477_RS01590	765	A	C	nonsynonymous
325593	NTHI477_RS01590	758	G	A	nonsynonymous
325598	NTHI477_RS01590	753	A	C	synonymous
325600	NTHI477_RS01590	751	C	A	nonsynonymous
325601	NTHI477_RS01590	750	T	A	synonymous

325627	NTHI477_RS01590	724	G	T	nonsynonymous
325628	NTHI477_RS01590	723	A	C	nonsynonymous
325630	NTHI477_RS01590	721	A	C	nonsynonymous
325631	NTHI477_RS01590	720	G	A	synonymous
325694	NTHI477_RS01590	657	G	A	synonymous
325724	NTHI477_RS01590	627	A	G	synonymous
325748	NTHI477_RS01590	603	A	G	synonymous
325751	NTHI477_RS01590	600	G	A	synonymous
325794	NTHI477_RS01590	557	C	T	nonsynonymous
325802	NTHI477_RS01590	549	C	T	synonymous
325805	NTHI477_RS01590	546	G	A	synonymous
325817	NTHI477_RS01590	534	T	G	synonymous
325828	NTHI477_RS01590	523	G	A	synonymous
325834	NTHI477_RS01590	517	G	T	synonymous
325835	NTHI477_RS01590	516	G	A	synonymous
325838	NTHI477_RS01590	513	C	T	synonymous
325865	NTHI477_RS01590	486	G	A	synonymous
325880	NTHI477_RS01590	471	C	T	synonymous
325901	NTHI477_RS01590	450	T	C	synonymous
325916	NTHI477_RS01590	435	C	A	synonymous
325919	NTHI477_RS01590	432	T	G	synonymous
325973	NTHI477_RS01590	378	C	T	synonymous
325982	NTHI477_RS01590	369	A	T	synonymous
325991	NTHI477_RS01590	360	A	T	synonymous
325997	NTHI477_RS01590	354	A	G	synonymous
326018	NTHI477_RS01590	333	G	A	synonymous
326024	NTHI477_RS01590	327	C	T	synonymous
326039	NTHI477_RS01590	312	T	C	synonymous
326042	NTHI477_RS01590	309	T	C	synonymous
326045	NTHI477_RS01590	306	T	C	synonymous
326051	NTHI477_RS01590	300	C	T	synonymous
326057	NTHI477_RS01590	294	G	A	synonymous
326114	NTHI477_RS01590	237	C	T	synonymous
326126	NTHI477_RS01590	225	G	A	synonymous
326129	NTHI477_RS01590	222	G	A	synonymous
326132	NTHI477_RS01590	219	G	A	synonymous
326141	NTHI477_RS01590	210	T	A	synonymous
326144	NTHI477_RS01590	207	A	C	synonymous
326144	NTHI477_RS01590	207	A	G	synonymous
326150	NTHI477_RS01590	201	T	C	synonymous
326156	NTHI477_RS01590	195	G	A	synonymous
326159	NTHI477_RS01590	192	A	G	synonymous
326171	NTHI477_RS01590	180	A	G	synonymous
326174	NTHI477_RS01590	177	G	A	synonymous
326197	NTHI477_RS01590	154	C	A	nonsynonymous

326207	NTHI477_RS01590	144	G	A	synonymous
326216	NTHI477_RS01590	135	T	C	synonymous
326219	NTHI477_RS01590	132	T	C	synonymous
326231	NTHI477_RS01590	120	A	G	synonymous
326234	NTHI477_RS01590	117	G	A	synonymous
326261	NTHI477_RS01590	90	C	A	synonymous
326276	NTHI477_RS01590	75	G	A	synonymous
326355	intergenic	intergenic	C	A	intergenic
326356	intergenic	intergenic	T	C	intergenic
326422	intergenic	intergenic	T	G	intergenic
326429	intergenic	intergenic	C	T	intergenic
326431	intergenic	intergenic	A	C	intergenic
326438	intergenic	intergenic	G	A	intergenic
326509	NTHI477_RS01595	660	C	T	synonymous
326602	NTHI477_RS01595	567	G	A	synonymous
326823	NTHI477_RS01595	346	C	A	nonsynonymous
326824	NTHI477_RS01595	345	A	G	synonymous
326909	NTHI477_RS01595	260	C	T	nonsynonymous
326956	NTHI477_RS01595	213	G	T	synonymous
326965	NTHI477_RS01595	204	G	A	synonymous
326986	NTHI477_RS01595	183	C	T	synonymous
327022	NTHI477_RS01595	147	T	G	synonymous
327124	NTHI477_RS01595	45	G	A	synonymous
327193	NTHI477_RS01600	417	C	G	synonymous
327400	NTHI477_RS01600	210	T	C	synonymous
327430	NTHI477_RS01600	180	G	A	synonymous
327508	NTHI477_RS01600	102	T	A	synonymous
327551	NTHI477_RS01600	59	T	G	nonsynonymous
327666	intergenic	intergenic	G	T	intergenic
327669	intergenic	intergenic	A	G	intergenic
327698	intergenic	intergenic	C	T	intergenic
327748	NTHI477_RS01605	25	G	A	nonsynonymous
327804	NTHI477_RS01605	81	G	T	synonymous
327810	NTHI477_RS01605	87	T	A	synonymous
327840	NTHI477_RS01605	117	C	T	synonymous
327845	NTHI477_RS01605	122	G	T	nonsynonymous
327966	NTHI477_RS01605	243	C	T	synonymous
327969	NTHI477_RS01605	246	A	T	synonymous
327972	NTHI477_RS01605	249	T	C	synonymous
327976	NTHI477_RS01605	253	T	G	nonsynonymous
327977	NTHI477_RS01605	254	C	T	nonsynonymous
327978	NTHI477_RS01605	255	A	G	synonymous
327980	NTHI477_RS01605	257	T	G	nonsynonymous
328005	NTHI477_RS01605	282	A	C	synonymous
328011	NTHI477_RS01605	288	G	A	synonymous

328015	NTHI477_RS01605	292	A	G	nonsynonymous
328017	NTHI477_RS01605	294	T	G	nonsynonymous
328030	NTHI477_RS01605	307	G	A	nonsynonymous
328032	NTHI477_RS01605	309	T	A	nonsynonymous
328033	NTHI477_RS01605	310	A	C	nonsynonymous
328071	NTHI477_RS01605	348	A	T	synonymous
328083	NTHI477_RS01605	360	A	G	synonymous
328116	NTHI477_RS01605	393	G	A	synonymous
328158	NTHI477_RS01605	435	T	C	synonymous
328204	NTHI477_RS01605	481	T	C	nonsynonymous
328239	NTHI477_RS01610	32	A	C	nonsynonymous
328265	NTHI477_RS01610	58	T	G	nonsynonymous
328267	NTHI477_RS01610	60	T	G	synonymous
328271	NTHI477_RS01610	64	A	G	nonsynonymous
328279	NTHI477_RS01610	72	A	G	synonymous
328294	NTHI477_RS01610	87	C	T	synonymous
328297	NTHI477_RS01610	90	G	A	synonymous
328300	NTHI477_RS01610	93	A	G	synonymous
328311	NTHI477_RS01610	104	T	A	nonsynonymous
328319	NTHI477_RS01610	112	G	A	nonsynonymous
328327	NTHI477_RS01610	120	C	T	synonymous
328330	NTHI477_RS01610	123	T	C	synonymous
328340	NTHI477_RS01610	133	G	A	nonsynonymous
328342	NTHI477_RS01610	135	G	T	synonymous
328345	NTHI477_RS01610	138	T	G	synonymous
328353	NTHI477_RS01610	146	C	T	nonsynonymous
328360	NTHI477_RS01610	153	T	C	synonymous
328363	NTHI477_RS01610	156	C	T	synonymous
328378	NTHI477_RS01610	171	T	G	synonymous
328378	NTHI477_RS01610	171	T	A	synonymous
328381	NTHI477_RS01610	174	C	A	synonymous
328388	NTHI477_RS01610	181	G	A	nonsynonymous
328389	NTHI477_RS01610	182	T	C	nonsynonymous
328390	NTHI477_RS01610	183	G	C	synonymous
328414	NTHI477_RS01610	207	G	A	synonymous
328417	NTHI477_RS01610	210	T	A	synonymous
328421	NTHI477_RS01610	214	G	A	nonsynonymous
328430	NTHI477_RS01610	223	C	A	nonsynonymous
328435	NTHI477_RS01610	228	A	T	synonymous
328435	NTHI477_RS01610	228	A	G	synonymous
328446	NTHI477_RS01610	239	A	G	nonsynonymous
328448	NTHI477_RS01610	241	G	A	nonsynonymous
328451	NTHI477_RS01610	244	G	A	nonsynonymous
328469	NTHI477_RS01610	262	A	G	nonsynonymous
328477	NTHI477_RS01610	270	C	T	synonymous

328478	NTHI477_RS01610	271	G	A	nonsynonymous
328480	NTHI477_RS01610	273	A	G	synonymous
328483	NTHI477_RS01610	276	A	T	synonymous
328504	NTHI477_RS01610	297	G	A	synonymous
328505	NTHI477_RS01610	298	C	A	nonsynonymous
328507	NTHI477_RS01610	300	G	A	synonymous
328519	NTHI477_RS01610	312	C	T	synonymous
328522	NTHI477_RS01610	315	C	A	synonymous
328558	NTHI477_RS01610	351	T	C	synonymous
328561	NTHI477_RS01610	354	C	T	synonymous
328570	NTHI477_RS01610	363	C	T	synonymous
328575	NTHI477_RS01610	368	G	T	nonsynonymous
328579	NTHI477_RS01610	372	T	C	synonymous
328585	NTHI477_RS01610	378	G	A	synonymous
328601	NTHI477_RS01610	394	G	A	nonsynonymous
328637	NTHI477_RS01610	430	C	T	synonymous
328653	NTHI477_RS01610	446	T	C	nonsynonymous
328654	NTHI477_RS01610	447	G	A	synonymous
328657	NTHI477_RS01610	450	T	G	synonymous
328663	NTHI477_RS01610	456	T	A	synonymous
328664	NTHI477_RS01610	457	A	G	nonsynonymous
328687	NTHI477_RS01610	480	C	G	synonymous
328688	NTHI477_RS01610	481	G	A	nonsynonymous
328693	NTHI477_RS01610	486	G	A	synonymous
328717	NTHI477_RS01610	510	G	T	synonymous
328717	NTHI477_RS01610	510	G	A	synonymous
328723	NTHI477_RS01610	516	A	T	synonymous
328741	NTHI477_RS01610	534	C	A	synonymous
328744	NTHI477_RS01610	537	G	A	synonymous
328772	NTHI477_RS01610	565	G	T	nonsynonymous
328786	NTHI477_RS01610	579	G	A	synonymous
328789	NTHI477_RS01610	582	T	C	synonymous
328792	NTHI477_RS01610	585	A	T	synonymous
328801	NTHI477_RS01610	594	G	A	synonymous
328813	NTHI477_RS01610	606	T	C	synonymous
328819	NTHI477_RS01610	612	T	C	synonymous
328820	NTHI477_RS01610	613	G	A	nonsynonymous
328831	NTHI477_RS01610	624	T	C	synonymous
328837	NTHI477_RS01610	630	T	C	synonymous
328840	NTHI477_RS01610	633	C	T	synonymous
328840	NTHI477_RS01610	633	C	A	synonymous
328843	NTHI477_RS01610	636	T	C	synonymous
328846	NTHI477_RS01610	639	C	T	synonymous
328885	NTHI477_RS01610	678	A	G	synonymous
328909	NTHI477_RS01610	702	G	A	synonymous

328942	NTHI477_RS01610	735	T	C	synonymous
328945	NTHI477_RS01610	738	C	T	synonymous
328948	NTHI477_RS01610	741	A	G	synonymous
328960	NTHI477_RS01610	753	A	T	nonsynonymous
328960	NTHI477_RS01610	753	A	G	synonymous
328963	NTHI477_RS01610	756	T	G	synonymous
328966	NTHI477_RS01610	759	G	A	synonymous
328973	NTHI477_RS01610	766	A	G	nonsynonymous
328975	NTHI477_RS01610	768	T	G	nonsynonymous
328996	NTHI477_RS01610	789	G	A	synonymous
329004	NTHI477_RS01610	797	G	T	nonsynonymous
329014	NTHI477_RS01610	807	T	A	synonymous
329044	NTHI477_RS01610	837	C	A	synonymous
329053	NTHI477_RS01610	846	T	C	synonymous
329054	NTHI477_RS01610	847	G	A	nonsynonymous
329056	NTHI477_RS01610	849	G	A	synonymous
329059	NTHI477_RS01610	852	G	A	synonymous
329060	NTHI477_RS01610	853	G	A	nonsynonymous
329066	NTHI477_RS01610	859	C	T	synonymous
329083	NTHI477_RS01610	876	T	C	synonymous
329086	NTHI477_RS01610	879	C	T	synonymous
329089	NTHI477_RS01610	882	A	G	synonymous
329099	NTHI477_RS01610	892	T	C	nonsynonymous
329110	NTHI477_RS01610	903	C	T	synonymous
329121	NTHI477_RS01610	914	C	T	nonsynonymous
329122	NTHI477_RS01610	915	C	T	synonymous
329151	NTHI477_RS01610	944	A	G	nonsynonymous
329165	NTHI477_RS01610	958	A	G	nonsynonymous
329170	NTHI477_RS01610	963	T	C	synonymous
329177	NTHI477_RS01610	970	G	C	nonsynonymous
329179	NTHI477_RS01610	972	A	T	synonymous
329185	NTHI477_RS01610	978	T	C	synonymous
329186	NTHI477_RS01610	979	G	A	nonsynonymous
329188	NTHI477_RS01610	981	C	T	synonymous
329198	NTHI477_RS01610	991	G	A	nonsynonymous
329206	NTHI477_RS01610	999	C	T	synonymous
329221	NTHI477_RS01610	1014	T	C	synonymous
329230	NTHI477_RS01610	1023	T	A	synonymous
329239	NTHI477_RS01610	1032	G	A	synonymous
329257	NTHI477_RS01610	1050	G	A	synonymous
329260	NTHI477_RS01610	1053	T	C	synonymous
329263	NTHI477_RS01610	1056	T	A	synonymous
329266	NTHI477_RS01610	1059	G	A	synonymous
329269	NTHI477_RS01610	1062	C	A	synonymous
329278	NTHI477_RS01610	1071	C	T	synonymous

329296	NTHI477_RS01610	1089	A	T	synonymous
329320	NTHI477_RS01610	1113	G	A	synonymous
329323	NTHI477_RS01610	1116	T	C	synonymous
329326	NTHI477_RS01610	1119	T	G	synonymous
329335	NTHI477_RS01610	1128	G	C	synonymous
329335	NTHI477_RS01610	1128	G	A	synonymous
329350	NTHI477_RS01610	1143	A	G	synonymous
329351	NTHI477_RS01610	1144	A	G	nonsynonymous
329362	NTHI477_RS01610	1155	G	A	synonymous
329363	NTHI477_RS01610	1156	G	A	nonsynonymous
329368	NTHI477_RS01610	1161	C	T	synonymous
329371	NTHI477_RS01610	1164	T	C	synonymous
329381	NTHI477_RS01610	1174	G	A	nonsynonymous
329390	NTHI477_RS01610	1183	C	T	synonymous
329419	NTHI477_RS01615	16	A	C	nonsynonymous
329442	NTHI477_RS01615	39	T	A	nonsynonymous
329445	NTHI477_RS01615	42	C	T	synonymous
329454	NTHI477_RS01615	51	G	A	synonymous
329466	NTHI477_RS01615	63	A	T	synonymous
329472	NTHI477_RS01615	69	A	G	synonymous
329479	NTHI477_RS01615	76	C	T	nonsynonymous
329494	NTHI477_RS01615	91	A	G	nonsynonymous
329497	NTHI477_RS01615	94	G	A	nonsynonymous
329502	NTHI477_RS01615	99	C	T	synonymous
329514	NTHI477_RS01615	111	A	G	synonymous
329515	NTHI477_RS01615	112	C	T	nonsynonymous
329563	NTHI477_RS01615	160	C	A	nonsynonymous
329585	NTHI477_RS01615	182	G	A	nonsynonymous
329619	NTHI477_RS01615	216	G	A	synonymous
329632	NTHI477_RS01615	229	T	C	nonsynonymous
329638	NTHI477_RS01615	235	C	A	nonsynonymous
329649	NTHI477_RS01615	246	C	T	synonymous
329684	NTHI477_RS01615	281	T	C	nonsynonymous
329687	NTHI477_RS01615	284	C	T	nonsynonymous
329688	NTHI477_RS01615	285	C	A	synonymous
329717	NTHI477_RS01615	314	G	A	nonsynonymous
329724	NTHI477_RS01615	321	T	C	synonymous
329729	NTHI477_RS01615	326	T	C	nonsynonymous
329730	NTHI477_RS01615	327	G	A	nonsynonymous
329742	NTHI477_RS01615	339	C	A	synonymous
329755	NTHI477_RS01615	352	A	G	nonsynonymous
329804	NTHI477_RS01620	30	G	A	synonymous
329810	NTHI477_RS01620	36	A	G	synonymous
329813	NTHI477_RS01620	39	C	A	synonymous
329822	NTHI477_RS01620	48	T	C	synonymous

329837	NTHI477_RS01620	63	T	C	synonymous
329846	NTHI477_RS01620	72	C	T	synonymous
329848	NTHI477_RS01620	74	A	G	nonsynonymous
329912	NTHI477_RS01620	138	A	G	synonymous
329915	NTHI477_RS01620	141	G	A	synonymous
329927	NTHI477_RS01620	153	T	C	synonymous
329928	NTHI477_RS01620	154	G	A	nonsynonymous
329928	NTHI477_RS01620	154	G	T	nonsynonymous
329936	NTHI477_RS01620	162	T	C	synonymous
329949	NTHI477_RS01620	175	G	A	nonsynonymous
329960	NTHI477_RS01620	186	T	A	synonymous
329981	NTHI477_RS01620	207	A	G	synonymous
329994	NTHI477_RS01620	220	G	A	nonsynonymous
330006	NTHI477_RS01620	232	C	T	nonsynonymous
330007	NTHI477_RS01620	233	G	T	nonsynonymous
330008	NTHI477_RS01620	234	T	A	synonymous
330008	NTHI477_RS01620	234	T	G	synonymous
330008	NTHI477_RS01620	234	T	C	synonymous
330009	NTHI477_RS01620	235	G	A	nonsynonymous
330010	NTHI477_RS01620	236	C	T	nonsynonymous
330023	NTHI477_RS01620	249	A	C	synonymous
330035	NTHI477_RS01620	261	T	C	synonymous
330057	NTHI477_RS01620	283	C	G	nonsynonymous
330089	NTHI477_RS01620	315	A	G	synonymous
330092	NTHI477_RS01620	318	A	T	synonymous
330099	NTHI477_RS01620	325	A	G	nonsynonymous
330101	NTHI477_RS01620	327	A	C	synonymous
330101	NTHI477_RS01620	327	A	G	synonymous
330108	NTHI477_RS01620	334	G	A	nonsynonymous
330119	NTHI477_RS01620	345	G	A	synonymous
330128	NTHI477_RS01620	354	A	G	synonymous
330131	NTHI477_RS01620	357	C	T	synonymous
330152	NTHI477_RS01620	378	G	A	synonymous
330158	NTHI477_RS01620	384	T	C	synonymous
330160	NTHI477_RS01620	386	G	A	nonsynonymous
330161	NTHI477_RS01620	387	T	C	synonymous
330171	NTHI477_RS01620	397	T	C	nonsynonymous
330176	NTHI477_RS01620	402	A	G	synonymous
330178	NTHI477_RS01620	404	C	T	nonsynonymous
330179	NTHI477_RS01620	405	A	C	synonymous
330182	NTHI477_RS01620	408	T	C	synonymous
330185	NTHI477_RS01620	411	G	T	synonymous
330197	NTHI477_RS01620	423	G	A	synonymous
330198	NTHI477_RS01620	424	T	C	synonymous
330200	NTHI477_RS01620	426	G	A	synonymous

330209	NTHI477_RS01620	435	A	G	synonymous
330266	NTHI477_RS01620	492	A	G	synonymous
330275	NTHI477_RS01620	501	G	A	nonsynonymous
330281	NTHI477_RS01620	507	T	A	synonymous
330283	NTHI477_RS01620	509	C	T	nonsynonymous
330284	NTHI477_RS01620	510	G	A	synonymous
330285	NTHI477_RS01620	511	G	A	nonsynonymous
330317	NTHI477_RS01620	543	G	C	synonymous
330320	NTHI477_RS01620	546	G	T	nonsynonymous
330323	NTHI477_RS01620	549	C	T	synonymous
330329	NTHI477_RS01620	555	C	T	synonymous
330332	NTHI477_RS01620	558	A	G	synonymous
330344	NTHI477_RS01620	570	C	T	synonymous
330347	NTHI477_RS01620	573	A	G	synonymous
330350	NTHI477_RS01620	576	A	T	synonymous
330350	NTHI477_RS01620	576	A	C	synonymous
330359	NTHI477_RS01620	585	T	C	synonymous
330370	NTHI477_RS01620	596	A	C	nonsynonymous
330380	NTHI477_RS01620	606	T	A	synonymous
330380	NTHI477_RS01620	606	T	G	synonymous
330388	NTHI477_RS01620	614	C	T	nonsynonymous
330429	NTHI477_RS01620	655	A	G	nonsynonymous
330464	NTHI477_RS01620	690	T	G	synonymous
330467	NTHI477_RS01620	693	A	T	synonymous
330470	NTHI477_RS01620	696	T	C	synonymous
330483	NTHI477_RS01620	709	A	G	nonsynonymous
330485	NTHI477_RS01620	711	C	T	synonymous
330526	NTHI477_RS01620	752	G	T	nonsynonymous
330530	NTHI477_RS01620	756	C	A	nonsynonymous
330540	NTHI477_RS01620	766	C	T	synonymous
330558	NTHI477_RS01620	784	G	T	nonsynonymous
330576	intergenic	intergenic	G	C	intergenic
330582	intergenic	intergenic	A	C	intergenic
330583	intergenic	intergenic	T	C	intergenic
330592	intergenic	intergenic	G	C	intergenic
330593	intergenic	intergenic	T	G	intergenic
330595	intergenic	intergenic	G	A	intergenic
330598	intergenic	intergenic	C	T	intergenic
330598	intergenic	intergenic	C	A	intergenic
330601	intergenic	intergenic	C	T	intergenic
330601	intergenic	intergenic	C	A	intergenic
330605	intergenic	intergenic	T	A	intergenic
330606	intergenic	intergenic	C	A	intergenic
330606	intergenic	intergenic	C	G	intergenic
330610	intergenic	intergenic	G	A	intergenic

330615	intergenic	intergenic	G	A	intergenic
330654	NTHI477_RS01625	39	C	A	synonymous
330703	NTHI477_RS01625	88	C	T	nonsynonymous
330714	NTHI477_RS01625	99	T	C	synonymous
330717	NTHI477_RS01625	102	G	C	synonymous
330726	NTHI477_RS01625	111	T	C	synonymous
330735	NTHI477_RS01625	120	A	G	synonymous
330744	NTHI477_RS01625	129	T	C	synonymous
330753	NTHI477_RS01625	138	A	G	synonymous
330756	NTHI477_RS01625	141	A	T	synonymous
330762	NTHI477_RS01625	147	T	C	synonymous
330765	NTHI477_RS01625	150	C	T	synonymous
330768	NTHI477_RS01625	153	C	T	synonymous
330769	NTHI477_RS01625	154	G	A	nonsynonymous
330771	NTHI477_RS01625	156	C	A	synonymous
330798	NTHI477_RS01625	183	G	T	synonymous
330819	NTHI477_RS01625	204	A	G	synonymous
330822	NTHI477_RS01625	207	G	A	synonymous
330828	NTHI477_RS01625	213	A	G	synonymous
330840	NTHI477_RS01625	225	T	C	synonymous
330855	NTHI477_RS01625	240	T	C	synonymous
330876	NTHI477_RS01625	261	A	G	synonymous
330903	NTHI477_RS01625	288	T	C	synonymous
330909	NTHI477_RS01625	294	T	C	synonymous
330925	NTHI477_RS01625	310	G	A	nonsynonymous
330936	NTHI477_RS01625	321	C	T	synonymous
330958	NTHI477_RS01625	343	T	C	nonsynonymous
330960	NTHI477_RS01625	345	C	T	synonymous
330968	NTHI477_RS01625	353	T	G	nonsynonymous
330978	NTHI477_RS01625	363	G	T	synonymous
330978	NTHI477_RS01625	363	G	A	synonymous
330996	NTHI477_RS01625	381	G	A	synonymous
331024	NTHI477_RS01625	409	G	T	nonsynonymous
331036	NTHI477_RS01625	421	G	A	nonsynonymous
331038	NTHI477_RS01625	423	T	G	nonsynonymous
331080	NTHI477_RS01625	465	T	A	synonymous
331082	NTHI477_RS01625	467	G	C	nonsynonymous
331083	NTHI477_RS01625	468	T	C	synonymous
331095	NTHI477_RS01625	480	T	C	synonymous
331098	NTHI477_RS01625	483	C	T	synonymous
331102	NTHI477_RS01625	487	A	G	nonsynonymous
331125	NTHI477_RS01625	510	G	T	synonymous
331161	NTHI477_RS01625	546	A	G	synonymous
331162	NTHI477_RS01625	547	T	C	synonymous
331164	NTHI477_RS01625	549	G	A	synonymous

331170	NTHI477_RS01625	555	T	C	synonymous
331179	NTHI477_RS01625	564	C	A	synonymous
331191	NTHI477_RS01625	576	G	A	synonymous
331203	NTHI477_RS01625	588	C	A	synonymous
331203	NTHI477_RS01625	588	C	T	synonymous
331218	NTHI477_RS01625	603	C	T	synonymous
331222	NTHI477_RS01625	607	G	A	nonsynonymous
331237	NTHI477_RS01625	622	A	G	nonsynonymous
331242	NTHI477_RS01625	627	C	G	nonsynonymous
331249	NTHI477_RS01625	634	T	C	synonymous
331254	NTHI477_RS01625	639	G	A	synonymous
331264	NTHI477_RS01625	649	G	A	nonsynonymous
331272	NTHI477_RS01625	657	C	T	synonymous
331275	NTHI477_RS01625	660	T	C	synonymous
331278	NTHI477_RS01625	663	G	A	synonymous
331281	NTHI477_RS01625	666	T	C	synonymous
331317	NTHI477_RS01625	702	T	C	synonymous
331320	NTHI477_RS01625	705	C	T	synonymous
331356	NTHI477_RS01625	741	C	T	synonymous
331356	NTHI477_RS01625	741	C	A	synonymous
331359	NTHI477_RS01625	744	T	C	synonymous
331374	NTHI477_RS01625	759	C	T	synonymous
331392	NTHI477_RS01625	777	C	T	synonymous
331395	NTHI477_RS01625	780	T	C	synonymous
331398	NTHI477_RS01625	783	T	C	synonymous
331401	NTHI477_RS01625	786	A	G	synonymous
331407	NTHI477_RS01625	792	C	T	synonymous
331422	NTHI477_RS01625	807	T	C	synonymous
331443	NTHI477_RS01625	828	G	A	synonymous
331460	intergenic	intergenic	C	A	intergenic
331461	intergenic	intergenic	G	T	intergenic
331467	intergenic	intergenic	G	C	intergenic
331487	intergenic	intergenic	T	C	intergenic
331497	NTHI477_RS01630	1116	T	C	synonymous
331515	NTHI477_RS01630	1098	C	T	synonymous
331518	NTHI477_RS01630	1095	C	T	synonymous
331523	NTHI477_RS01630	1090	A	G	synonymous
331537	NTHI477_RS01630	1076	C	T	nonsynonymous
331551	NTHI477_RS01630	1062	T	A	synonymous
331551	NTHI477_RS01630	1062	T	G	synonymous
331563	NTHI477_RS01630	1050	A	G	synonymous
331573	NTHI477_RS01630	1040	C	T	nonsynonymous
331581	NTHI477_RS01630	1032	A	G	synonymous
331614	NTHI477_RS01630	999	A	C	nonsynonymous
331682	NTHI477_RS01630	931	A	G	nonsynonymous

331701	NTHI477_RS01630	912	T	G	synonymous
331722	NTHI477_RS01630	891	T	C	synonymous
331731	NTHI477_RS01630	882	G	A	synonymous
331737	NTHI477_RS01630	876	G	A	synonymous
331755	NTHI477_RS01630	858	C	G	synonymous
331767	NTHI477_RS01630	846	A	G	synonymous
331776	NTHI477_RS01630	837	C	T	synonymous
331791	NTHI477_RS01630	822	G	A	synonymous
331799	NTHI477_RS01630	814	C	T	nonsynonymous
331830	NTHI477_RS01630	783	A	G	synonymous
331836	NTHI477_RS01630	777	C	T	synonymous
331911	NTHI477_RS01630	702	G	T	synonymous
331911	NTHI477_RS01630	702	G	A	synonymous
331944	NTHI477_RS01630	669	A	G	synonymous
331953	NTHI477_RS01630	660	A	G	synonymous
331974	NTHI477_RS01630	639	T	C	synonymous
332004	NTHI477_RS01630	609	G	A	synonymous
332009	NTHI477_RS01630	604	C	A	nonsynonymous
332010	NTHI477_RS01630	603	A	C	synonymous
332019	NTHI477_RS01630	594	T	C	synonymous
332037	NTHI477_RS01630	576	A	T	synonymous
332045	NTHI477_RS01630	568	A	T	nonsynonymous
332049	NTHI477_RS01630	564	A	G	synonymous
332051	NTHI477_RS01630	562	C	T	nonsynonymous
332067	NTHI477_RS01630	546	T	G	synonymous
332076	NTHI477_RS01630	537	A	T	synonymous
332088	NTHI477_RS01630	525	G	A	synonymous
332103	NTHI477_RS01630	510	T	C	synonymous
332105	NTHI477_RS01630	508	G	A	synonymous
332109	NTHI477_RS01630	504	G	A	synonymous
332115	NTHI477_RS01630	498	C	T	synonymous
332132	NTHI477_RS01630	481	A	G	synonymous
332133	NTHI477_RS01630	480	G	T	synonymous
332134	NTHI477_RS01630	479	A	C	nonsynonymous
332136	NTHI477_RS01630	477	T	C	synonymous
332142	NTHI477_RS01630	471	T	C	synonymous
332148	NTHI477_RS01630	465	T	C	synonymous
332151	NTHI477_RS01630	462	G	A	synonymous
332154	NTHI477_RS01630	459	G	A	synonymous
332156	NTHI477_RS01630	457	G	T	nonsynonymous
332160	NTHI477_RS01630	453	A	G	synonymous
332163	NTHI477_RS01630	450	A	G	synonymous
332166	NTHI477_RS01630	447	G	A	synonymous
332169	NTHI477_RS01630	444	G	A	synonymous
332174	NTHI477_RS01630	439	C	T	nonsynonymous

332195	NTHI477_RS01630	418	C	T	nonsynonymous
332244	NTHI477_RS01630	369	T	G	synonymous
332259	NTHI477_RS01630	354	A	T	synonymous
332280	NTHI477_RS01630	333	G	A	synonymous
332283	NTHI477_RS01630	330	G	A	synonymous
332298	NTHI477_RS01630	315	G	A	synonymous
332301	NTHI477_RS01630	312	G	A	synonymous
332307	NTHI477_RS01630	306	A	G	synonymous
332327	NTHI477_RS01630	286	C	T	nonsynonymous
332330	NTHI477_RS01630	283	A	G	nonsynonymous
332337	NTHI477_RS01630	276	A	G	synonymous
332346	NTHI477_RS01630	267	G	A	synonymous
332355	NTHI477_RS01630	258	G	A	synonymous
332376	NTHI477_RS01630	237	G	A	synonymous
332388	NTHI477_RS01630	225	G	A	synonymous
332393	NTHI477_RS01630	220	A	G	synonymous
332406	NTHI477_RS01630	207	C	T	synonymous
332436	NTHI477_RS01630	177	T	C	synonymous
332448	NTHI477_RS01630	165	C	A	synonymous
332448	NTHI477_RS01630	165	C	T	synonymous
332453	NTHI477_RS01630	160	G	T	nonsynonymous
332463	NTHI477_RS01630	150	T	G	synonymous
332472	NTHI477_RS01630	141	C	T	synonymous
332502	NTHI477_RS01630	111	A	G	synonymous
332508	NTHI477_RS01630	105	A	C	synonymous
332605	NTHI477_RS01630	8	A	G	nonsynonymous
332618	intergenic	intergenic	C	T	intergenic
332625	intergenic	intergenic	C	T	intergenic
332628	intergenic	intergenic	A	T	intergenic
332629	intergenic	intergenic	A	G	intergenic
332629	intergenic	intergenic	A	T	intergenic
332634	intergenic	intergenic	G	T	intergenic
332635	intergenic	intergenic	T	C	intergenic
332666	intergenic	intergenic	T	G	intergenic
332677	intergenic	intergenic	G	A	intergenic
332685	intergenic	intergenic	T	C	intergenic
332687	intergenic	intergenic	A	G	intergenic
332708	NTHI477_RS01635	915	G	A	synonymous
332723	NTHI477_RS01635	900	G	C	synonymous
332723	NTHI477_RS01635	900	G	T	synonymous
332747	NTHI477_RS01635	876	G	A	synonymous
332756	NTHI477_RS01635	867	T	G	synonymous
332759	NTHI477_RS01635	864	C	T	synonymous
332801	NTHI477_RS01635	822	G	A	synonymous
332801	NTHI477_RS01635	822	G	T	synonymous

332804	NTHI477_RS01635	819	A	G	synonymous
332806	NTHI477_RS01635	817	T	C	nonsynonymous
332819	NTHI477_RS01635	804	G	A	synonymous
332820	NTHI477_RS01635	803	T	G	nonsynonymous
332846	NTHI477_RS01635	777	A	G	synonymous
332852	NTHI477_RS01635	771	A	G	synonymous
332861	NTHI477_RS01635	762	C	T	synonymous
332868	NTHI477_RS01635	755	G	A	nonsynonymous
332869	NTHI477_RS01635	754	C	T	nonsynonymous
332885	NTHI477_RS01635	738	G	A	synonymous
332909	NTHI477_RS01635	714	G	A	synonymous
332915	NTHI477_RS01635	708	T	C	synonymous
332915	NTHI477_RS01635	708	T	A	synonymous
332924	NTHI477_RS01635	699	A	T	synonymous
332925	NTHI477_RS01635	698	C	T	nonsynonymous
332929	NTHI477_RS01635	694	T	C	nonsynonymous
332930	NTHI477_RS01635	693	T	C	synonymous
332935	NTHI477_RS01635	688	C	T	nonsynonymous
332936	NTHI477_RS01635	687	A	G	synonymous
332949	NTHI477_RS01635	674	C	T	nonsynonymous
332957	NTHI477_RS01635	666	G	A	synonymous
332960	NTHI477_RS01635	663	C	T	synonymous
332965	NTHI477_RS01635	658	G	C	nonsynonymous
332968	NTHI477_RS01635	655	A	G	synonymous
332974	NTHI477_RS01635	649	T	C	nonsynonymous
332975	NTHI477_RS01635	648	G	A	synonymous
332981	NTHI477_RS01635	642	C	T	nonsynonymous
332995	NTHI477_RS01635	628	T	C	nonsynonymous
333017	NTHI477_RS01635	606	G	A	synonymous
333038	NTHI477_RS01635	585	C	T	synonymous
333062	NTHI477_RS01635	561	C	T	synonymous
333071	NTHI477_RS01635	552	G	T	synonymous
333077	NTHI477_RS01635	546	G	A	synonymous
333101	NTHI477_RS01635	522	A	G	synonymous
333107	NTHI477_RS01635	516	C	T	synonymous
333123	NTHI477_RS01635	500	T	A	nonsynonymous
333128	NTHI477_RS01635	495	T	C	synonymous
333131	NTHI477_RS01635	492	G	A	synonymous
333133	NTHI477_RS01635	490	G	T	nonsynonymous
333149	NTHI477_RS01635	474	A	G	synonymous
333151	NTHI477_RS01635	472	A	G	nonsynonymous
333173	NTHI477_RS01635	450	T	C	synonymous
333182	NTHI477_RS01635	441	C	A	nonsynonymous
333200	NTHI477_RS01635	423	G	T	synonymous
333200	NTHI477_RS01635	423	G	A	synonymous

333203	NTHI477_RS01635	420	C	G	synonymous
333224	NTHI477_RS01635	399	A	G	synonymous
333239	NTHI477_RS01635	384	T	C	synonymous
333242	NTHI477_RS01635	381	G	A	synonymous
333242	NTHI477_RS01635	381	G	T	synonymous
333251	NTHI477_RS01635	372	T	G	synonymous
333263	NTHI477_RS01635	360	C	T	synonymous
333272	NTHI477_RS01635	351	G	A	synonymous
333276	NTHI477_RS01635	347	C	T	nonsynonymous
333290	NTHI477_RS01635	333	T	C	synonymous
333306	NTHI477_RS01635	317	G	T	nonsynonymous
333316	NTHI477_RS01635	307	G	C	nonsynonymous
333319	NTHI477_RS01635	304	T	G	nonsynonymous
333319	NTHI477_RS01635	304	T	A	nonsynonymous
333326	NTHI477_RS01635	297	T	A	synonymous
333329	NTHI477_RS01635	294	A	C	synonymous
333338	NTHI477_RS01635	285	A	C	synonymous
333338	NTHI477_RS01635	285	A	G	synonymous
333350	NTHI477_RS01635	273	C	T	synonymous
333365	NTHI477_RS01635	258	G	A	synonymous
333371	NTHI477_RS01635	252	T	C	synonymous
333386	NTHI477_RS01635	237	C	T	synonymous
333407	NTHI477_RS01635	216	T	G	synonymous
333443	NTHI477_RS01635	180	A	G	synonymous
333455	NTHI477_RS01635	168	T	C	synonymous
333455	NTHI477_RS01635	168	T	A	synonymous
333464	NTHI477_RS01635	159	T	C	synonymous
333470	NTHI477_RS01635	153	A	T	synonymous
333476	NTHI477_RS01635	147	C	A	synonymous
333485	NTHI477_RS01635	138	C	T	synonymous
333488	NTHI477_RS01635	135	G	A	synonymous
333491	NTHI477_RS01635	132	T	A	synonymous
333500	NTHI477_RS01635	123	T	A	synonymous
333500	NTHI477_RS01635	123	T	C	synonymous
333506	NTHI477_RS01635	117	A	G	synonymous
333527	NTHI477_RS01635	96	C	T	synonymous
333531	NTHI477_RS01635	92	T	C	nonsynonymous
333539	NTHI477_RS01635	84	G	A	synonymous
333561	NTHI477_RS01635	62	T	C	nonsynonymous
333566	NTHI477_RS01635	57	T	C	synonymous
333584	NTHI477_RS01635	39	A	T	synonymous
333584	NTHI477_RS01635	39	A	C	synonymous
333670	NTHI477_RS01640	339	T	G	synonymous
333673	NTHI477_RS01640	336	T	C	synonymous
333691	NTHI477_RS01640	318	C	A	nonsynonymous

333715	NTHI477_RS01640	294	G	A	synonymous
333739	NTHI477_RS01640	270	G	A	synonymous
333760	NTHI477_RS01640	249	G	A	synonymous
333766	NTHI477_RS01640	243	T	C	synonymous
333769	NTHI477_RS01640	240	T	C	synonymous
333808	NTHI477_RS01640	201	G	A	synonymous
333838	NTHI477_RS01640	171	A	G	synonymous
333856	NTHI477_RS01640	153	G	A	synonymous
333877	NTHI477_RS01640	132	G	A	synonymous
333880	NTHI477_RS01640	129	C	T	synonymous
333883	NTHI477_RS01640	126	C	T	synonymous
334030	intergenic	intergenic	G	T	intergenic
334031	intergenic	intergenic	T	C	intergenic
334032	intergenic	intergenic	G	A	intergenic
334035	intergenic	intergenic	T	C	intergenic
334056	intergenic	intergenic	A	G	intergenic
334083	intergenic	intergenic	C	T	intergenic
334084	intergenic	intergenic	T	C	intergenic
334085	intergenic	intergenic	A	G	intergenic
334102	intergenic	intergenic	A	G	intergenic
334106	intergenic	intergenic	G	A	intergenic
334182	NTHI477_RS01645	60	G	A	synonymous
334185	NTHI477_RS01645	63	G	C	synonymous
334187	NTHI477_RS01645	65	A	G	nonsynonymous
334195	NTHI477_RS01645	73	G	A	nonsynonymous
334213	NTHI477_RS01645	91	C	T	nonsynonymous
334214	NTHI477_RS01645	92	A	G	nonsynonymous
334237	NTHI477_RS01645	115	C	T	nonsynonymous
334240	NTHI477_RS01645	118	T	A	nonsynonymous
334246	NTHI477_RS01645	124	C	T	nonsynonymous
334259	NTHI477_RS01645	137	G	T	nonsynonymous
334267	NTHI477_RS01645	145	C	G	nonsynonymous
334267	NTHI477_RS01645	145	C	T	nonsynonymous
334273	NTHI477_RS01645	151	A	G	nonsynonymous
334279	NTHI477_RS01645	157	C	T	nonsynonymous
334285	NTHI477_RS01645	163	G	A	nonsynonymous
334288	NTHI477_RS01645	166	G	T	nonsynonymous
334291	NTHI477_RS01645	169	C	T	nonsynonymous
334297	NTHI477_RS01645	175	T	C	nonsynonymous
334297	NTHI477_RS01645	175	T	A	nonsynonymous
334300	NTHI477_RS01645	178	C	T	synonymous
334309	NTHI477_RS01645	187	C	T	nonsynonymous
334324	NTHI477_RS01645	202	T	C	nonsynonymous
334330	NTHI477_RS01645	208	C	T	nonsynonymous
334345	NTHI477_RS01645	223	T	C	nonsynonymous

334348	NTHI477_RS01645	226	T	C	nonsynonymous
334350	NTHI477_RS01645	228	G	A	synonymous
334351	NTHI477_RS01645	229	T	C	nonsynonymous
334354	NTHI477_RS01645	232	C	T	nonsynonymous
334357	NTHI477_RS01645	235	C	T	nonsynonymous
334366	NTHI477_RS01645	244	G	A	nonsynonymous
334367	NTHI477_RS01645	245	G	A	nonsynonymous
334381	NTHI477_RS01645	259	G	A	nonsynonymous
334387	NTHI477_RS01645	265	C	T	nonsynonymous
334387	NTHI477_RS01645	265	C	G	nonsynonymous
334388	NTHI477_RS01645	266	T	A	nonsynonymous
334390	NTHI477_RS01645	268	C	T	nonsynonymous
334391	NTHI477_RS01645	269	A	G	nonsynonymous
334392	NTHI477_RS01645	270	C	A	nonsynonymous
334392	NTHI477_RS01645	270	C	T	synonymous
334393	NTHI477_RS01645	271	A	T	nonsynonymous
334394	NTHI477_RS01645	272	C	G	nonsynonymous
334401	NTHI477_RS01645	279	A	G	synonymous
334402	NTHI477_RS01645	280	T	C	nonsynonymous
334405	NTHI477_RS01645	283	C	A	nonsynonymous
334412	NTHI477_RS01645	290	C	T	nonsynonymous
334426	NTHI477_RS01645	304	C	T	synonymous
334427	NTHI477_RS01645	305	T	G	nonsynonymous
334428	NTHI477_RS01645	306	A	T	synonymous
334429	NTHI477_RS01645	307	C	T	nonsynonymous
334432	NTHI477_RS01645	310	T	C	nonsynonymous
334435	NTHI477_RS01645	313	G	T	nonsynonymous
334435	NTHI477_RS01645	313	G	C	nonsynonymous
334438	NTHI477_RS01645	316	C	A	synonymous
334444	NTHI477_RS01645	322	A	G	nonsynonymous
334451	NTHI477_RS01645	329	G	A	nonsynonymous
334453	NTHI477_RS01645	331	A	T	nonsynonymous
334456	NTHI477_RS01645	334	C	T	nonsynonymous
334462	NTHI477_RS01645	340	C	T	nonsynonymous
334464	NTHI477_RS01645	342	T	C	synonymous
334465	NTHI477_RS01645	343	C	T	nonsynonymous
334465	NTHI477_RS01645	343	C	A	synonymous
334468	NTHI477_RS01645	346	A	G	nonsynonymous
334471	NTHI477_RS01645	349	G	A	nonsynonymous
334477	NTHI477_RS01645	355	C	T	nonsynonymous
334480	NTHI477_RS01645	358	C	T	nonsynonymous
334483	NTHI477_RS01645	361	C	T	synonymous
334486	NTHI477_RS01645	364	C	T	synonymous
334492	NTHI477_RS01645	370	T	C	nonsynonymous
334495	NTHI477_RS01645	373	C	G	nonsynonymous

334495	NTHI477_RS01645	373	C	T	nonsynonymous
334498	NTHI477_RS01645	376	C	T	nonsynonymous
334501	NTHI477_RS01645	379	T	C	nonsynonymous
334504	NTHI477_RS01645	382	G	A	nonsynonymous
334507	NTHI477_RS01645	385	C	T	synonymous
334517	NTHI477_RS01645	395	C	T	nonsynonymous
334518	NTHI477_RS01645	396	A	T	synonymous
334519	NTHI477_RS01645	397	G	T	nonsynonymous
334522	NTHI477_RS01645	400	A	T	nonsynonymous
334525	NTHI477_RS01645	403	C	G	nonsynonymous
334525	NTHI477_RS01645	403	C	A	nonsynonymous
334532	NTHI477_RS01645	410	G	C	nonsynonymous
334534	NTHI477_RS01645	412	C	A	synonymous
334537	NTHI477_RS01645	415	G	C	nonsynonymous
334538	NTHI477_RS01645	416	A	C	nonsynonymous
334545	NTHI477_RS01645	423	C	A	nonsynonymous
334546	NTHI477_RS01645	424	C	A	nonsynonymous
334549	NTHI477_RS01645	427	T	C	nonsynonymous
334552	NTHI477_RS01645	430	C	T	nonsynonymous
334555	NTHI477_RS01645	433	C	T	nonsynonymous
334556	NTHI477_RS01645	434	T	C	nonsynonymous
334558	NTHI477_RS01645	436	A	C	nonsynonymous
334563	NTHI477_RS01645	441	T	C	synonymous
334564	NTHI477_RS01645	442	C	T	nonsynonymous
334567	NTHI477_RS01645	445	C	G	nonsynonymous
334567	NTHI477_RS01645	445	C	T	nonsynonymous
334573	NTHI477_RS01645	451	G	A	nonsynonymous
334574	NTHI477_RS01645	452	C	T	nonsynonymous
334576	NTHI477_RS01645	454	G	A	nonsynonymous
334585	NTHI477_RS01645	463	A	T	nonsynonymous
334588	NTHI477_RS01645	466	A	G	nonsynonymous
334588	NTHI477_RS01645	466	A	T	nonsynonymous
334594	NTHI477_RS01645	472	T	C	nonsynonymous
334595	NTHI477_RS01645	473	T	G	nonsynonymous
334597	NTHI477_RS01645	475	T	A	nonsynonymous
334600	NTHI477_RS01645	478	C	T	nonsynonymous
334607	NTHI477_RS01645	485	A	C	nonsynonymous
334608	NTHI477_RS01645	486	A	G	synonymous
334609	NTHI477_RS01645	487	A	T	nonsynonymous
334610	NTHI477_RS01645	488	C	T	nonsynonymous
334618	NTHI477_RS01645	496	C	T	nonsynonymous
334624	NTHI477_RS01645	502	C	T	nonsynonymous
334627	NTHI477_RS01645	505	C	T	nonsynonymous
334633	NTHI477_RS01645	511	C	T	nonsynonymous
334645	NTHI477_RS01645	523	C	T	nonsynonymous

334657	NTHI477_RS01645	535	C	G	nonsynonymous
334663	NTHI477_RS01645	541	C	T	nonsynonymous
334666	NTHI477_RS01645	544	A	C	nonsynonymous
334669	NTHI477_RS01645	547	C	T	nonsynonymous
334696	NTHI477_RS01645	574	C	T	nonsynonymous
334700	NTHI477_RS01645	578	G	A	nonsynonymous
334705	NTHI477_RS01645	583	C	T	nonsynonymous
334706	NTHI477_RS01645	584	C	T	nonsynonymous
334714	NTHI477_RS01645	592	G	T	nonsynonymous
334714	NTHI477_RS01645	592	G	A	nonsynonymous
334717	NTHI477_RS01645	595	G	T	nonsynonymous
334720	NTHI477_RS01645	598	T	C	nonsynonymous
334724	NTHI477_RS01645	602	T	C	nonsynonymous
334726	NTHI477_RS01645	604	G	C	nonsynonymous
334726	NTHI477_RS01645	604	G	T	nonsynonymous
334729	NTHI477_RS01645	607	T	C	nonsynonymous
334732	NTHI477_RS01645	610	C	T	nonsynonymous
334738	NTHI477_RS01645	616	C	T	nonsynonymous
334739	NTHI477_RS01645	617	A	C	nonsynonymous
334741	NTHI477_RS01645	619	C	T	nonsynonymous
334750	NTHI477_RS01645	628	T	C	nonsynonymous
334753	NTHI477_RS01645	631	G	A	nonsynonymous
334771	NTHI477_RS01645	649	C	T	nonsynonymous
334774	NTHI477_RS01645	652	A	G	nonsynonymous
334783	NTHI477_RS01645	661	C	A	nonsynonymous
334783	NTHI477_RS01645	661	C	T	nonsynonymous
334792	NTHI477_RS01645	670	T	C	nonsynonymous
334795	NTHI477_RS01645	673	A	T	nonsynonymous
334801	NTHI477_RS01645	679	C	G	nonsynonymous
334804	NTHI477_RS01645	682	C	T	nonsynonymous
334816	NTHI477_RS01645	694	C	T	nonsynonymous
334819	NTHI477_RS01645	697	C	T	nonsynonymous
334822	NTHI477_RS01645	700	C	T	nonsynonymous
334825	NTHI477_RS01645	703	T	C	nonsynonymous
334826	NTHI477_RS01645	704	C	A	nonsynonymous
334828	NTHI477_RS01645	706	C	A	nonsynonymous
334834	NTHI477_RS01645	712	C	T	nonsynonymous
334840	NTHI477_RS01645	718	A	C	synonymous
334846	NTHI477_RS01645	724	C	T	nonsynonymous
334852	NTHI477_RS01645	730	C	A	synonymous
334855	NTHI477_RS01645	733	C	T	nonsynonymous
334858	NTHI477_RS01645	736	A	G	nonsynonymous
334867	NTHI477_RS01645	745	C	T	nonsynonymous
334870	NTHI477_RS01645	748	G	A	nonsynonymous
334873	NTHI477_RS01645	751	G	A	nonsynonymous

334882	NTHI477_RS01645	760	A	C	nonsynonymous
334882	NTHI477_RS01645	760	A	T	nonsynonymous
334909	NTHI477_RS01645	787	G	A	nonsynonymous
334912	NTHI477_RS01645	790	A	C	nonsynonymous
334915	NTHI477_RS01645	793	C	T	nonsynonymous
334918	NTHI477_RS01645	796	C	T	nonsynonymous
334918	NTHI477_RS01645	796	C	A	synonymous
334921	NTHI477_RS01645	799	C	T	nonsynonymous
334921	NTHI477_RS01645	799	C	A	nonsynonymous
334924	NTHI477_RS01645	802	C	T	nonsynonymous
334936	NTHI477_RS01645	814	T	A	nonsynonymous
334939	NTHI477_RS01645	817	T	C	nonsynonymous
334948	NTHI477_RS01645	826	C	T	nonsynonymous
334951	NTHI477_RS01645	829	C	A	synonymous
334981	NTHI477_RS01645	859	C	T	nonsynonymous
335011	NTHI477_RS01645	889	G	T	nonsynonymous
335038	NTHI477_RS01645	916	A	C	nonsynonymous
335038	NTHI477_RS01645	916	A	G	nonsynonymous
335044	NTHI477_RS01645	922	A	C	nonsynonymous
335044	NTHI477_RS01645	922	A	T	nonsynonymous
335050	NTHI477_RS01645	928	A	G	nonsynonymous
335053	NTHI477_RS01645	931	T	C	nonsynonymous
335056	NTHI477_RS01645	934	G	C	nonsynonymous
335056	NTHI477_RS01645	934	G	A	nonsynonymous
335056	NTHI477_RS01645	934	G	T	nonsynonymous
335059	NTHI477_RS01645	937	G	C	nonsynonymous
335065	NTHI477_RS01645	943	T	C	nonsynonymous
335077	NTHI477_RS01645	955	T	C	nonsynonymous
335098	NTHI477_RS01645	976	C	T	nonsynonymous
335102	NTHI477_RS01645	980	C	T	nonsynonymous
335104	NTHI477_RS01645	982	C	T	nonsynonymous
335116	NTHI477_RS01645	994	T	C	nonsynonymous
335122	NTHI477_RS01645	1000	T	C	nonsynonymous
335128	NTHI477_RS01645	1006	G	A	nonsynonymous
335146	NTHI477_RS01645	1024	G	A	nonsynonymous
335158	NTHI477_RS01645	1036	T	C	nonsynonymous
335170	NTHI477_RS01645	1048	G	A	nonsynonymous
335179	NTHI477_RS01645	1057	C	T	nonsynonymous
335185	NTHI477_RS01645	1063	C	T	nonsynonymous
335212	NTHI477_RS01645	1090	T	C	nonsynonymous
335215	NTHI477_RS01645	1093	C	T	nonsynonymous
335230	NTHI477_RS01645	1108	C	T	nonsynonymous
335236	NTHI477_RS01645	1114	G	A	nonsynonymous
335239	NTHI477_RS01645	1117	G	A	nonsynonymous
335242	NTHI477_RS01645	1120	T	C	nonsynonymous

335246	NTHI477_RS01645	1124	G	A	nonsynonymous
335251	NTHI477_RS01645	1129	C	T	nonsynonymous
335284	NTHI477_RS01645	1162	C	T	nonsynonymous
335290	NTHI477_RS01645	1168	C	T	nonsynonymous
335293	NTHI477_RS01645	1171	A	G	nonsynonymous
335296	NTHI477_RS01645	1174	C	A	nonsynonymous
335314	NTHI477_RS01645	1192	G	A	nonsynonymous
335315	NTHI477_RS01645	1193	C	T	nonsynonymous
335317	NTHI477_RS01645	1195	A	G	nonsynonymous
335332	NTHI477_RS01645	1210	C	T	nonsynonymous
335338	NTHI477_RS01645	1216	C	T	nonsynonymous
335373	NTHI477_RS01645	1251	G	C	nonsynonymous
335374	NTHI477_RS01645	1252	C	T	nonsynonymous
335380	NTHI477_RS01645	1258	C	T	nonsynonymous
335387	NTHI477_RS01645	1265	G	A	nonsynonymous
335390	NTHI477_RS01645	1268	T	C	nonsynonymous
335395	NTHI477_RS01645	1273	G	C	nonsynonymous
335407	NTHI477_RS01645	1285	C	T	nonsynonymous
335413	NTHI477_RS01645	1291	G	A	nonsynonymous
335416	NTHI477_RS01645	1294	C	A	synonymous
335416	NTHI477_RS01645	1294	C	T	nonsynonymous
335419	NTHI477_RS01645	1297	C	T	nonsynonymous
335422	NTHI477_RS01645	1300	G	A	nonsynonymous
335428	NTHI477_RS01645	1306	C	T	nonsynonymous
335434	NTHI477_RS01645	1312	C	T	nonsynonymous
335446	NTHI477_RS01645	1324	T	C	nonsynonymous
335461	NTHI477_RS01645	1339	T	C	nonsynonymous
335467	NTHI477_RS01645	1345	C	T	nonsynonymous
335469	NTHI477_RS01645	1347	G	T	synonymous
335479	NTHI477_RS01645	1357	A	G	nonsynonymous
335480	NTHI477_RS01645	1358	C	T	nonsynonymous
335482	NTHI477_RS01645	1360	C	T	nonsynonymous
335494	NTHI477_RS01645	1372	A	G	nonsynonymous
335497	NTHI477_RS01645	1375	G	A	nonsynonymous
335506	NTHI477_RS01645	1384	T	C	nonsynonymous
335508	NTHI477_RS01645	1386	C	T	synonymous
335518	NTHI477_RS01645	1396	C	T	nonsynonymous
335524	NTHI477_RS01645	1402	C	T	nonsynonymous
335536	NTHI477_RS01645	1414	C	A	nonsynonymous
335537	NTHI477_RS01645	1415	A	G	nonsynonymous
335539	NTHI477_RS01645	1417	C	A	nonsynonymous
335545	NTHI477_RS01645	1423	T	C	nonsynonymous
335560	NTHI477_RS01645	1438	C	T	nonsynonymous
335570	NTHI477_RS01645	1448	C	A	nonsynonymous
335579	NTHI477_RS01645	1457	G	A	nonsynonymous

335581	NTHI477_RS01645	1459	T	C	nonsynonymous
335584	NTHI477_RS01645	1462	C	T	nonsynonymous
335590	NTHI477_RS01645	1468	C	T	nonsynonymous
335591	NTHI477_RS01645	1469	G	A	nonsynonymous
335601	NTHI477_RS01645	1479	C	A	nonsynonymous
335602	NTHI477_RS01645	1480	C	A	nonsynonymous
335608	NTHI477_RS01645	1486	C	T	nonsynonymous
335611	NTHI477_RS01645	1489	C	T	synonymous
335614	NTHI477_RS01645	1492	C	T	nonsynonymous
335623	NTHI477_RS01645	1501	A	G	nonsynonymous
335635	NTHI477_RS01645	1513	T	C	nonsynonymous
335642	NTHI477_RS01645	1520	A	G	nonsynonymous
335644	NTHI477_RS01645	1522	T	G	nonsynonymous
335647	NTHI477_RS01645	1525	T	G	nonsynonymous
335650	NTHI477_RS01645	1528	A	G	nonsynonymous
335656	NTHI477_RS01645	1534	G	A	nonsynonymous
335660	NTHI477_RS01645	1538	A	G	synonymous
335661	NTHI477_RS01645	1539	A	C	nonsynonymous
335662	NTHI477_RS01645	1540	A	G	nonsynonymous
335663	NTHI477_RS01645	1541	G	A	nonsynonymous
335664	NTHI477_RS01645	1542	A	G	synonymous
335665	NTHI477_RS01645	1543	C	T	nonsynonymous
335671	NTHI477_RS01645	1549	C	T	nonsynonymous
335686	NTHI477_RS01645	1564	A	G	nonsynonymous
335689	NTHI477_RS01645	1567	T	G	nonsynonymous
335689	NTHI477_RS01645	1567	T	A	nonsynonymous
335695	NTHI477_RS01645	1573	T	C	nonsynonymous
335698	NTHI477_RS01645	1576	C	A	synonymous
335704	NTHI477_RS01645	1582	C	T	nonsynonymous
335719	NTHI477_RS01645	1597	C	T	nonsynonymous
335725	NTHI477_RS01645	1603	C	T	nonsynonymous
335728	NTHI477_RS01645	1606	G	A	nonsynonymous
335761	NTHI477_RS01645	1639	T	A	nonsynonymous
335767	NTHI477_RS01645	1645	G	A	nonsynonymous
335770	NTHI477_RS01645	1648	G	A	nonsynonymous
335773	NTHI477_RS01645	1651	G	A	nonsynonymous
335776	NTHI477_RS01645	1654	A	G	nonsynonymous
335781	NTHI477_RS01645	1659	C	T	synonymous
335782	NTHI477_RS01645	1660	G	A	nonsynonymous
335789	NTHI477_RS01645	1667	G	A	nonsynonymous
335791	NTHI477_RS01645	1669	G	A	nonsynonymous
335794	NTHI477_RS01645	1672	A	G	nonsynonymous
335797	NTHI477_RS01645	1675	G	A	nonsynonymous
335803	NTHI477_RS01645	1681	G	A	nonsynonymous
335806	NTHI477_RS01645	1684	A	G	nonsynonymous

335812	NTHI477_RS01645	1690	G	A	nonsynonymous
335815	NTHI477_RS01645	1693	G	A	nonsynonymous
335821	NTHI477_RS01645	1699	A	G	nonsynonymous
335824	NTHI477_RS01645	1702	A	G	nonsynonymous
335833	NTHI477_RS01645	1711	G	T	nonsynonymous
335839	NTHI477_RS01645	1717	G	A	nonsynonymous
335839	NTHI477_RS09425	5	G	A	nonsynonymous
335841	NTHI477_RS01645	1719	G	A	synonymous
335841	NTHI477_RS09425	7	G	A	nonsynonymous
335855	NTHI477_RS09425	21	T	G	nonsynonymous
336353	NTHI477_RS09425	519	C	T	synonymous
336353	NTHI477_RS09425	519	C	A	synonymous
336354	NTHI477_RS09425	520	C	G	nonsynonymous
336359	NTHI477_RS09425	525	C	A	synonymous
336362	NTHI477_RS09425	528	T	C	synonymous
336395	NTHI477_RS09425	561	T	C	synonymous
336400	NTHI477_RS09425	566	C	T	nonsynonymous
336401	NTHI477_RS09425	567	C	G	synonymous
336413	NTHI477_RS09425	579	C	T	synonymous
336422	NTHI477_RS09425	588	C	T	synonymous
336428	NTHI477_RS09425	594	C	T	synonymous
336431	NTHI477_RS09425	597	T	C	synonymous
336434	NTHI477_RS09425	600	C	T	synonymous
336434	NTHI477_RS09425	600	C	A	synonymous
336440	NTHI477_RS09425	606	T	C	synonymous
336440	NTHI477_RS09425	606	T	A	synonymous
336441	NTHI477_RS09425	607	G	A	nonsynonymous
336449	NTHI477_RS09425	615	T	C	synonymous
336450	NTHI477_RS09425	616	C	G	nonsynonymous
336455	NTHI477_RS09425	621	C	G	synonymous
336462	NTHI477_RS09425	628	G	A	nonsynonymous
336464	NTHI477_RS09425	630	C	G	nonsynonymous
336467	NTHI477_RS09425	633	C	A	synonymous
336468	NTHI477_RS09425	634	C	A	nonsynonymous
336470	NTHI477_RS09425	636	G	T	synonymous
336473	NTHI477_RS09425	639	T	C	synonymous
336474	NTHI477_RS09425	640	T	C	nonsynonymous
336484	NTHI477_RS09425	650	C	T	nonsynonymous
336485	NTHI477_RS09425	651	A	G	synonymous
336488	NTHI477_RS09425	654	G	A	synonymous
336491	NTHI477_RS09425	657	G	T	synonymous
336492	NTHI477_RS09425	658	G	A	nonsynonymous
336494	NTHI477_RS09425	660	A	T	synonymous
336494	NTHI477_RS09425	660	A	C	synonymous
336500	NTHI477_RS09425	666	G	C	nonsynonymous

336504	NTHI477_RS09425	670	G	A	nonsynonymous
336512	NTHI477_RS09425	678	C	T	synonymous
336539	NTHI477_RS09425	705	A	T	synonymous
336544	NTHI477_RS09425	710	C	T	nonsynonymous
336548	NTHI477_RS09425	714	A	G	synonymous
336549	NTHI477_RS09425	715	A	G	nonsynonymous
336550	NTHI477_RS09425	716	C	A	nonsynonymous
336554	NTHI477_RS09425	720	G	A	synonymous
336560	NTHI477_RS09425	726	C	A	nonsynonymous
336561	NTHI477_RS09425	727	C	T	nonsynonymous
336562	NTHI477_RS09425	728	G	A	nonsynonymous
336567	NTHI477_RS09425	733	A	G	nonsynonymous
336569	NTHI477_RS09425	735	C	A	synonymous
336573	NTHI477_RS09425	739	T	C	synonymous
336577	NTHI477_RS09425	743	T	C	nonsynonymous
336585	NTHI477_RS09425	751	G	A	nonsynonymous
336614	NTHI477_RS09425	780	G	A	synonymous
336615	NTHI477_RS09425	781	G	A	nonsynonymous
336625	NTHI477_RS09425	791	T	G	nonsynonymous
336626	NTHI477_RS09425	792	T	C	synonymous
336629	NTHI477_RS09425	795	A	G	synonymous
336629	NTHI477_RS09425	795	A	T	nonsynonymous
336631	NTHI477_RS09425	797	T	C	nonsynonymous
336637	NTHI477_RS09425	803	A	G	nonsynonymous
336646	NTHI477_RS09425	812	G	A	nonsynonymous
336653	NTHI477_RS09425	819	G	A	synonymous
336654	NTHI477_RS09425	820	C	G	nonsynonymous
337884	NTHI477_RS01660	68	G	T	nonsynonymous
337884	NTHI477_RS01660	68	G	A	nonsynonymous
337888	NTHI477_RS01660	72	C	T	synonymous
337897	NTHI477_RS01660	81	T	C	synonymous
337898	NTHI477_RS01660	82	T	C	synonymous
337912	NTHI477_RS01660	96	A	G	synonymous
337916	NTHI477_RS01660	100	G	A	nonsynonymous
337918	NTHI477_RS01660	102	G	A	synonymous
337922	NTHI477_RS01660	106	G	T	nonsynonymous
337931	NTHI477_RS01660	115	G	A	nonsynonymous
337939	NTHI477_RS01660	123	T	C	synonymous
337941	NTHI477_RS01660	125	G	A	nonsynonymous
337942	NTHI477_RS01660	126	C	T	synonymous
337946	NTHI477_RS01660	130	G	A	nonsynonymous
337948	NTHI477_RS01660	132	C	T	synonymous
337952	NTHI477_RS01660	136	G	A	nonsynonymous
337972	NTHI477_RS01660	156	G	A	synonymous
337981	NTHI477_RS01660	165	G	A	synonymous

337982	NTHI477_RS01660	166	C	T	nonsynonymous
337990	NTHI477_RS01660	174	T	G	synonymous
337993	NTHI477_RS01660	177	G	A	synonymous
337996	NTHI477_RS01660	180	A	G	synonymous
338011	NTHI477_RS01660	195	G	T	synonymous
338020	NTHI477_RS01660	204	G	A	synonymous
338023	NTHI477_RS01660	207	G	C	synonymous
338029	NTHI477_RS01660	213	C	G	synonymous
338032	NTHI477_RS01660	216	G	A	synonymous
338035	NTHI477_RS01660	219	A	G	synonymous
338053	NTHI477_RS01660	237	C	T	synonymous
338059	NTHI477_RS01660	243	G	T	synonymous
338062	NTHI477_RS01660	246	G	A	synonymous
338065	NTHI477_RS01660	249	T	C	synonymous
338065	NTHI477_RS01660	249	T	A	synonymous
338072	NTHI477_RS01660	256	G	A	nonsynonymous
338074	NTHI477_RS01660	258	G	A	synonymous
338083	NTHI477_RS01660	267	G	A	synonymous
338084	NTHI477_RS01660	268	C	T	synonymous
338085	NTHI477_RS01660	269	T	C	nonsynonymous
338087	NTHI477_RS01660	271	C	T	synonymous
338098	NTHI477_RS01660	282	C	T	synonymous
338101	NTHI477_RS01660	285	G	A	synonymous
338104	NTHI477_RS01660	288	G	A	synonymous
338107	NTHI477_RS01660	291	G	A	synonymous
338107	NTHI477_RS01660	291	G	T	synonymous
338107	NTHI477_RS01660	291	G	C	synonymous
338114	NTHI477_RS01660	298	C	A	nonsynonymous
338115	NTHI477_RS01660	299	A	C	nonsynonymous
338117	NTHI477_RS01660	301	A	C	nonsynonymous
338117	NTHI477_RS01660	301	A	T	nonsynonymous
338118	NTHI477_RS01660	302	C	T	nonsynonymous
338118	NTHI477_RS01660	302	C	A	nonsynonymous
338119	NTHI477_RS01660	303	A	T	synonymous
338120	NTHI477_RS01660	304	A	G	nonsynonymous
338122	NTHI477_RS01660	306	C	T	synonymous
338125	NTHI477_RS01660	309	T	C	synonymous
338128	NTHI477_RS01660	312	G	A	synonymous
338131	NTHI477_RS01660	315	A	G	synonymous
338137	NTHI477_RS01660	321	C	T	synonymous
338145	NTHI477_RS01660	329	C	T	nonsynonymous
338146	NTHI477_RS01660	330	A	G	synonymous
338149	NTHI477_RS01660	333	C	T	synonymous
338161	NTHI477_RS01660	345	T	C	synonymous
338168	NTHI477_RS01660	352	T	G	nonsynonymous

338169	NTHI477_RS01660	353	G	T	nonsynonymous
338170	NTHI477_RS01660	354	G	T	nonsynonymous
338172	NTHI477_RS01660	356	A	G	nonsynonymous
338173	NTHI477_RS01660	357	A	G	synonymous
338176	NTHI477_RS01660	360	C	T	synonymous
338181	NTHI477_RS01660	365	A	G	nonsynonymous
338182	NTHI477_RS01660	366	C	T	synonymous
338188	NTHI477_RS01660	372	T	C	synonymous
338200	NTHI477_RS01660	384	A	G	synonymous
338201	NTHI477_RS01660	385	T	C	synonymous
338203	NTHI477_RS01660	387	G	A	synonymous
338215	NTHI477_RS01660	399	C	T	synonymous
338218	NTHI477_RS01660	402	C	T	synonymous
338224	NTHI477_RS01660	408	G	A	synonymous
338227	NTHI477_RS01660	411	C	T	synonymous
338230	NTHI477_RS01660	414	G	A	synonymous
338233	NTHI477_RS01660	417	G	A	synonymous
338239	NTHI477_RS01660	423	T	C	synonymous
338257	NTHI477_RS01660	441	A	C	synonymous
338263	NTHI477_RS01660	447	C	T	synonymous
338263	NTHI477_RS01660	447	C	G	synonymous
338264	NTHI477_RS01660	448	T	C	synonymous
338266	NTHI477_RS01660	450	A	G	synonymous
338269	NTHI477_RS01660	453	G	C	synonymous
338269	NTHI477_RS01660	453	G	T	synonymous
338272	NTHI477_RS01660	456	G	A	synonymous
338278	NTHI477_RS01660	462	A	G	synonymous
338287	NTHI477_RS01660	471	C	G	synonymous
338296	NTHI477_RS01660	480	G	A	synonymous
338297	NTHI477_RS01660	481	C	T	synonymous
338299	NTHI477_RS01660	483	G	A	synonymous
338311	NTHI477_RS01660	495	G	A	synonymous
338317	NTHI477_RS01660	501	G	A	synonymous
338323	NTHI477_RS01660	507	G	A	synonymous
338335	NTHI477_RS01660	519	A	G	synonymous
338351	NTHI477_RS01660	535	A	G	nonsynonymous
338374	NTHI477_RS01660	558	T	A	synonymous
338386	NTHI477_RS01660	570	T	C	synonymous
338387	NTHI477_RS01660	571	C	T	synonymous
338389	NTHI477_RS01660	573	G	A	synonymous
338398	NTHI477_RS01660	582	A	T	synonymous
338404	NTHI477_RS01660	588	C	T	synonymous
338409	NTHI477_RS01660	593	T	C	nonsynonymous
338410	NTHI477_RS01660	594	C	T	synonymous
338413	NTHI477_RS01660	597	C	A	synonymous

338419	NTHI477_RS01660	603	G	A	synonymous
338422	NTHI477_RS01660	606	T	C	synonymous
338431	NTHI477_RS01660	615	G	T	synonymous
338434	NTHI477_RS01660	618	A	G	synonymous
338440	NTHI477_RS01660	624	C	T	synonymous
338446	NTHI477_RS01660	630	C	T	synonymous
338452	NTHI477_RS01660	636	G	A	synonymous
338455	NTHI477_RS01660	639	G	T	synonymous
338464	NTHI477_RS01660	648	C	G	synonymous
338467	NTHI477_RS01660	651	G	C	synonymous
338470	NTHI477_RS01660	654	T	C	synonymous
338476	NTHI477_RS01660	660	A	G	synonymous
338477	NTHI477_RS01660	661	A	G	nonsynonymous
338478	NTHI477_RS01660	662	A	C	nonsynonymous
338479	NTHI477_RS01660	663	T	A	nonsynonymous
338485	NTHI477_RS01660	669	C	T	synonymous
338486	NTHI477_RS01660	670	G	A	nonsynonymous
338487	NTHI477_RS01660	671	C	T	nonsynonymous
338487	NTHI477_RS01660	671	C	A	nonsynonymous
338488	NTHI477_RS01660	672	G	A	synonymous
338491	NTHI477_RS01660	675	G	C	synonymous
338494	NTHI477_RS01660	678	A	G	synonymous
338495	NTHI477_RS01660	679	T	G	nonsynonymous
338502	NTHI477_RS01660	686	A	G	nonsynonymous
338503	NTHI477_RS01660	687	T	C	synonymous
338509	NTHI477_RS01660	693	A	G	synonymous
338517	NTHI477_RS01660	701	G	A	nonsynonymous
338518	NTHI477_RS01660	702	C	T	synonymous
338522	NTHI477_RS01660	706	C	T	nonsynonymous
338524	NTHI477_RS01660	708	C	T	synonymous
338524	NTHI477_RS01660	708	C	A	synonymous
338527	NTHI477_RS01660	711	T	C	synonymous
338531	NTHI477_RS01660	715	T	C	synonymous
338533	NTHI477_RS01660	717	G	A	synonymous
338534	NTHI477_RS01660	718	T	C	synonymous
338534	NTHI477_RS01660	718	T	A	nonsynonymous
338536	NTHI477_RS01660	720	G	A	synonymous
338537	NTHI477_RS01660	721	C	A	nonsynonymous
338542	NTHI477_RS01660	726	T	G	synonymous
338543	NTHI477_RS01660	727	A	G	nonsynonymous
338543	NTHI477_RS01660	727	A	C	nonsynonymous
338545	NTHI477_RS01660	729	G	A	synonymous
338546	NTHI477_RS01660	730	G	A	nonsynonymous
338548	NTHI477_RS01660	732	T	A	nonsynonymous
338549	NTHI477_RS01660	733	T	C	synonymous

338551	NTHI477_RS01660	735	A	G	synonymous
338552	NTHI477_RS01660	736	C	T	nonsynonymous
338554	NTHI477_RS01660	738	C	G	synonymous
338557	NTHI477_RS01660	741	C	T	synonymous
338561	NTHI477_RS01660	745	A	G	nonsynonymous
338563	NTHI477_RS01660	747	C	A	synonymous
338566	NTHI477_RS01660	750	C	T	synonymous
338572	NTHI477_RS01660	756	C	T	synonymous
338575	NTHI477_RS01660	759	C	G	synonymous
338576	NTHI477_RS01660	760	T	C	synonymous
338578	NTHI477_RS01660	762	G	A	synonymous
338584	NTHI477_RS01660	768	A	G	synonymous
338587	NTHI477_RS01660	771	G	C	synonymous
338589	NTHI477_RS01660	773	A	G	nonsynonymous
338594	NTHI477_RS01660	778	A	G	nonsynonymous
338595	NTHI477_RS01660	779	G	T	nonsynonymous
338599	NTHI477_RS01660	783	T	C	synonymous
338599	NTHI477_RS01660	783	T	A	synonymous
338600	NTHI477_RS01660	784	A	G	nonsynonymous
338601	NTHI477_RS01660	785	A	C	nonsynonymous
338602	NTHI477_RS01660	786	A	G	synonymous
338603	NTHI477_RS01660	787	A	T	nonsynonymous
338604	NTHI477_RS01660	788	C	T	nonsynonymous
338605	NTHI477_RS01660	789	C	A	synonymous
338608	NTHI477_RS01660	792	T	C	synonymous
338611	NTHI477_RS01660	795	A	G	synonymous
338620	NTHI477_RS01660	804	G	A	synonymous
338623	NTHI477_RS01660	807	G	A	synonymous
338641	NTHI477_RS01660	825	C	T	synonymous
338644	NTHI477_RS01660	828	A	G	synonymous
338653	NTHI477_RS01660	837	G	A	synonymous
338657	NTHI477_RS01660	841	A	G	nonsynonymous
338659	NTHI477_RS01660	843	T	A	nonsynonymous
338665	NTHI477_RS01660	849	C	G	synonymous
338665	NTHI477_RS01660	849	C	T	synonymous
338670	NTHI477_RS01660	854	G	T	nonsynonymous
338671	NTHI477_RS01660	855	G	T	nonsynonymous
338674	NTHI477_RS01660	858	G	C	synonymous
338677	NTHI477_RS01660	861	T	A	synonymous
338677	NTHI477_RS01660	861	T	C	synonymous
338678	NTHI477_RS01660	862	T	A	nonsynonymous
338679	NTHI477_RS01660	863	C	G	nonsynonymous
338680	NTHI477_RS01660	864	A	C	synonymous
338683	NTHI477_RS01660	867	A	G	synonymous
338684	NTHI477_RS01660	868	A	C	nonsynonymous

338692	NTHI477_RS01660	876	C	T	synonymous
338693	NTHI477_RS01660	877	C	A	nonsynonymous
338694	NTHI477_RS01660	878	G	A	nonsynonymous
338695	NTHI477_RS01660	879	C	A	synonymous
338701	NTHI477_RS01660	885	T	C	synonymous
338704	NTHI477_RS01660	888	C	T	synonymous
338704	NTHI477_RS01660	888	C	G	synonymous
338716	NTHI477_RS01660	900	T	C	synonymous
338722	NTHI477_RS01660	906	A	G	synonymous
338729	NTHI477_RS01660	913	G	A	nonsynonymous
338731	NTHI477_RS01660	915	T	C	synonymous
338734	NTHI477_RS01660	918	G	A	synonymous
338737	NTHI477_RS01660	921	G	A	synonymous
338737	NTHI477_RS01660	921	G	C	synonymous
338743	NTHI477_RS01660	927	G	C	synonymous
338743	NTHI477_RS01660	927	G	A	synonymous
338747	NTHI477_RS01660	931	C	T	synonymous
338758	NTHI477_RS01660	942	A	C	synonymous
338765	NTHI477_RS01660	949	C	T	synonymous
338770	NTHI477_RS01660	954	T	C	synonymous
338771	NTHI477_RS01660	955	G	T	nonsynonymous
338773	NTHI477_RS01660	957	A	C	synonymous
338776	NTHI477_RS01660	960	G	A	synonymous
338791	NTHI477_RS01660	975	T	C	synonymous
338794	NTHI477_RS01660	978	T	C	synonymous
338794	NTHI477_RS01660	978	T	G	synonymous
338797	NTHI477_RS01660	981	C	T	synonymous
338797	NTHI477_RS01660	981	C	G	synonymous
338801	NTHI477_RS01660	985	G	T	nonsynonymous
338803	NTHI477_RS01660	987	A	G	synonymous
338806	NTHI477_RS01660	990	G	C	synonymous
338809	NTHI477_RS01660	993	T	C	synonymous
338815	NTHI477_RS01660	999	C	T	synonymous
338818	NTHI477_RS01660	1002	C	T	synonymous
338821	NTHI477_RS01660	1005	T	C	synonymous
338827	NTHI477_RS01660	1011	A	G	synonymous
338830	NTHI477_RS01660	1014	C	T	synonymous
338839	NTHI477_RS01660	1023	C	T	synonymous
338848	NTHI477_RS01660	1032	T	C	synonymous
338863	NTHI477_RS01660	1047	A	G	synonymous
338866	NTHI477_RS01660	1050	T	G	nonsynonymous
338875	NTHI477_RS01660	1059	A	G	synonymous
338887	NTHI477_RS01660	1071	A	G	synonymous
338893	NTHI477_RS01660	1077	G	A	synonymous
338900	NTHI477_RS01660	1084	C	T	nonsynonymous

338902	NTHI477_RS01660	1086	C	T	synonymous
338902	NTHI477_RS01660	1086	C	A	synonymous
338905	NTHI477_RS01660	1089	T	A	nonsynonymous
338911	NTHI477_RS01660	1095	G	A	synonymous
338920	NTHI477_RS01660	1104	G	T	synonymous
338926	NTHI477_RS01660	1110	A	G	synonymous
338938	NTHI477_RS01660	1122	C	G	synonymous
338941	NTHI477_RS01660	1125	C	A	synonymous
338943	NTHI477_RS01660	1127	G	A	nonsynonymous
338947	NTHI477_RS01660	1131	G	C	synonymous
338947	NTHI477_RS01660	1131	G	A	synonymous
338956	NTHI477_RS01660	1140	G	T	synonymous
338956	NTHI477_RS01660	1140	G	C	synonymous
338959	NTHI477_RS01660	1143	G	C	synonymous
338965	NTHI477_RS01660	1149	G	A	synonymous
338974	NTHI477_RS01660	1158	C	T	synonymous
338977	NTHI477_RS01660	1161	C	A	synonymous
338983	NTHI477_RS01660	1167	G	A	synonymous
338992	NTHI477_RS01660	1176	C	T	synonymous
339002	NTHI477_RS01660	1186	A	G	nonsynonymous
339004	NTHI477_RS01660	1188	T	C	synonymous
339013	NTHI477_RS01660	1197	G	A	synonymous
339018	NTHI477_RS01660	1202	G	A	nonsynonymous
339031	NTHI477_RS01660	1215	G	A	synonymous
339034	NTHI477_RS01660	1218	C	T	synonymous
339037	NTHI477_RS01660	1221	C	T	synonymous
339046	NTHI477_RS01660	1230	T	A	synonymous
339052	NTHI477_RS01660	1236	C	T	synonymous
339055	NTHI477_RS01660	1239	C	T	synonymous
339061	NTHI477_RS01660	1245	T	C	synonymous
339064	NTHI477_RS01660	1248	C	T	synonymous
339070	NTHI477_RS01660	1254	G	A	synonymous
339076	NTHI477_RS01660	1260	G	A	synonymous
339077	NTHI477_RS01660	1261	C	T	synonymous
339091	NTHI477_RS01660	1275	G	A	synonymous
339094	NTHI477_RS01660	1278	T	C	synonymous
339097	NTHI477_RS01660	1281	G	A	synonymous
339098	NTHI477_RS01660	1282	A	G	nonsynonymous
339103	NTHI477_RS01660	1287	C	T	synonymous
339106	NTHI477_RS01660	1290	C	T	synonymous
339109	NTHI477_RS01660	1293	C	T	synonymous
339118	NTHI477_RS01660	1302	G	A	synonymous
339121	NTHI477_RS01660	1305	T	C	synonymous
339127	NTHI477_RS01660	1311	T	G	synonymous
339128	NTHI477_RS01660	1312	T	C	synonymous

339130	NTHI477_RS01660	1314	A	G	synonymous
339133	NTHI477_RS01660	1317	T	C	synonymous
339133	NTHI477_RS01660	1317	T	A	synonymous
339139	NTHI477_RS01660	1323	T	C	synonymous
339139	NTHI477_RS01660	1323	T	A	synonymous
339148	NTHI477_RS01660	1332	C	T	synonymous
339157	NTHI477_RS01660	1341	C	A	synonymous
339157	NTHI477_RS01660	1341	C	G	synonymous
339166	NTHI477_RS01660	1350	G	A	synonymous
339175	NTHI477_RS01660	1359	A	T	synonymous
339182	NTHI477_RS01660	1366	A	C	nonsynonymous
339183	NTHI477_RS01660	1367	A	G	nonsynonymous
339184	NTHI477_RS01660	1368	G	C	nonsynonymous
339187	NTHI477_RS01660	1371	T	C	synonymous
339190	NTHI477_RS01660	1374	A	G	synonymous
339199	NTHI477_RS01660	1383	T	G	synonymous
339207	NTHI477_RS01660	1391	G	A	nonsynonymous
339208	NTHI477_RS01660	1392	T	G	synonymous
339214	NTHI477_RS01660	1398	G	A	synonymous
339217	NTHI477_RS01660	1401	C	T	synonymous
339223	NTHI477_RS01660	1407	T	C	synonymous
339226	NTHI477_RS01660	1410	C	T	synonymous
339250	NTHI477_RS01660	1434	T	C	synonymous
339250	NTHI477_RS01660	1434	T	A	synonymous
339253	NTHI477_RS01660	1437	A	G	synonymous
339256	NTHI477_RS01660	1440	T	C	synonymous
339266	NTHI477_RS01660	1450	A	G	nonsynonymous
339268	NTHI477_RS01660	1452	T	G	nonsynonymous
339271	NTHI477_RS01660	1455	C	T	synonymous
339277	NTHI477_RS01660	1461	T	A	synonymous
339292	NTHI477_RS01660	1476	A	G	synonymous
339353	NTHI477_RS01660	1537	G	A	nonsynonymous
339386	NTHI477_RS01660	1570	C	T	nonsynonymous
339400	NTHI477_RS01660	1584	A	T	synonymous
339409	NTHI477_RS01660	1593	T	C	synonymous
339419	NTHI477_RS01660	1603	G	A	nonsynonymous
339427	NTHI477_RS01660	1611	T	A	nonsynonymous
339442	NTHI477_RS01660	1626	C	A	synonymous
339452	NTHI477_RS01660	1636	G	C	nonsynonymous
339454	NTHI477_RS01660	1638	A	G	synonymous
339455	NTHI477_RS01660	1639	G	C	nonsynonymous
339456	NTHI477_RS01660	1640	T	A	nonsynonymous
339457	NTHI477_RS01660	1641	G	A	synonymous
339458	NTHI477_RS01660	1642	G	C	nonsynonymous
339459	NTHI477_RS01660	1643	T	A	nonsynonymous

339460	NTHI477_RS01660	1644	G	A	synonymous
339460	NTHI477_RS01660	1644	G	T	synonymous
339464	NTHI477_RS01660	1648	A	G	nonsynonymous
339469	NTHI477_RS01660	1653	A	G	synonymous
339478	NTHI477_RS01660	1662	G	T	nonsynonymous
339481	NTHI477_RS01660	1665	G	T	synonymous
339496	NTHI477_RS01660	1680	G	A	synonymous
339508	NTHI477_RS01660	1692	C	T	synonymous
339511	NTHI477_RS01660	1695	A	G	synonymous
339514	NTHI477_RS01660	1698	C	T	synonymous
339518	NTHI477_RS01660	1702	G	C	nonsynonymous
339520	NTHI477_RS01660	1704	A	G	synonymous
339527	NTHI477_RS01660	1711	A	G	nonsynonymous
339528	NTHI477_RS01660	1712	A	C	nonsynonymous
339529	NTHI477_RS01660	1713	A	T	nonsynonymous
339530	NTHI477_RS01660	1714	T	C	synonymous
339533	NTHI477_RS01660	1717	T	C	nonsynonymous
339534	NTHI477_RS01660	1718	G	A	nonsynonymous
339550	NTHI477_RS01660	1734	G	A	synonymous
339553	NTHI477_RS01660	1737	C	T	synonymous
339559	NTHI477_RS01660	1743	T	C	synonymous
339562	NTHI477_RS01660	1746	C	T	synonymous
339563	NTHI477_RS01660	1747	A	G	nonsynonymous
339565	NTHI477_RS01660	1749	A	G	synonymous
339568	NTHI477_RS01660	1752	G	T	synonymous
339569	NTHI477_RS01660	1753	G	A	nonsynonymous
339589	NTHI477_RS01660	1773	A	T	synonymous
339595	NTHI477_RS01660	1779	T	G	synonymous
339598	NTHI477_RS01660	1782	T	A	synonymous
339599	NTHI477_RS01660	1783	T	A	nonsynonymous
339600	NTHI477_RS01660	1784	C	G	nonsynonymous
339605	NTHI477_RS01660	1789	G	A	nonsynonymous
339608	NTHI477_RS01660	1792	C	T	nonsynonymous
339610	NTHI477_RS01660	1794	G	T	synonymous
339610	NTHI477_RS01660	1794	G	A	synonymous
339619	NTHI477_RS01660	1803	G	A	synonymous
339622	NTHI477_RS01660	1806	A	G	synonymous
339625	NTHI477_RS01660	1809	C	T	synonymous
339626	NTHI477_RS01660	1810	G	A	nonsynonymous
339637	NTHI477_RS01660	1821	A	G	synonymous
339643	NTHI477_RS01660	1827	A	G	synonymous
339646	NTHI477_RS01660	1830	A	G	synonymous
339652	NTHI477_RS01660	1836	G	A	synonymous
339653	NTHI477_RS01660	1837	C	T	synonymous
339655	NTHI477_RS01660	1839	A	G	synonymous

339661	NTHI477_RS01660	1845	C	T	synonymous
339664	NTHI477_RS01660	1848	C	T	synonymous
339673	NTHI477_RS01660	1857	C	T	synonymous
339676	NTHI477_RS01660	1860	G	T	synonymous
339678	NTHI477_RS01660	1862	A	C	nonsynonymous
339691	NTHI477_RS01660	1875	A	G	synonymous
339692	NTHI477_RS01660	1876	G	C	nonsynonymous
339706	NTHI477_RS01660	1890	T	C	synonymous
339721	NTHI477_RS01660	1905	G	A	synonymous
339724	NTHI477_RS01660	1908	A	C	synonymous
339733	NTHI477_RS01660	1917	G	A	synonymous
339736	NTHI477_RS01660	1920	T	G	synonymous
339736	NTHI477_RS01660	1920	T	A	synonymous
339739	NTHI477_RS01660	1923	C	T	synonymous
339745	NTHI477_RS01660	1929	T	C	synonymous
339748	NTHI477_RS01660	1932	G	T	synonymous
339755	NTHI477_RS01660	1939	A	C	nonsynonymous
339757	NTHI477_RS01660	1941	C	T	synonymous
339760	NTHI477_RS01660	1944	C	T	synonymous
339766	NTHI477_RS01660	1950	G	A	synonymous
339770	NTHI477_RS01660	1954	C	A	nonsynonymous
339772	NTHI477_RS01660	1956	A	G	synonymous
339782	NTHI477_RS01660	1966	T	C	synonymous
339784	NTHI477_RS01660	1968	A	G	synonymous
339790	NTHI477_RS01660	1974	G	A	synonymous
339793	NTHI477_RS01660	1977	G	C	synonymous
339796	NTHI477_RS01660	1980	C	T	synonymous
339799	NTHI477_RS01660	1983	T	A	synonymous
339799	NTHI477_RS01660	1983	T	C	synonymous
339808	NTHI477_RS01660	1992	T	C	synonymous
339818	NTHI477_RS01660	2002	C	G	nonsynonymous
339819	NTHI477_RS01660	2003	G	C	nonsynonymous
339820	NTHI477_RS01660	2004	T	C	synonymous
339823	NTHI477_RS01660	2007	T	C	synonymous
339829	NTHI477_RS01660	2013	C	T	synonymous
339832	NTHI477_RS01660	2016	A	T	synonymous
339838	NTHI477_RS01660	2022	T	C	synonymous
339841	NTHI477_RS01660	2025	T	C	synonymous
339844	NTHI477_RS01660	2028	T	C	synonymous
339844	NTHI477_RS01660	2028	T	G	synonymous
339845	NTHI477_RS01660	2029	T	C	synonymous
339847	NTHI477_RS01660	2031	G	T	nonsynonymous
339850	NTHI477_RS01660	2034	T	C	synonymous
339862	NTHI477_RS01660	2046	A	T	synonymous
339865	NTHI477_RS01660	2049	C	T	synonymous

339866	NTHI477_RS01660	2050	A	T	nonsynonymous
339868	NTHI477_RS01660	2052	T	G	nonsynonymous
339871	NTHI477_RS01660	2055	T	A	synonymous
339874	NTHI477_RS01660	2058	C	A	nonsynonymous
339875	NTHI477_RS01660	2059	A	G	nonsynonymous
339877	NTHI477_RS01660	2061	G	A	synonymous
339878	NTHI477_RS01660	2062	C	A	nonsynonymous
339878	NTHI477_RS01660	2062	C	T	nonsynonymous
339880	NTHI477_RS01660	2064	C	T	synonymous
339880	NTHI477_RS01660	2064	C	A	synonymous
339881	NTHI477_RS01660	2065	A	G	nonsynonymous
339881	NTHI477_RS01660	2065	A	C	synonymous
339882	NTHI477_RS01660	2066	G	A	nonsynonymous
339884	NTHI477_RS01660	2068	G	A	nonsynonymous
339885	NTHI477_RS01660	2069	A	C	nonsynonymous
339892	NTHI477_RS01660	2076	C	T	synonymous
339892	NTHI477_RS01660	2076	C	A	synonymous
339894	NTHI477_RS01660	2078	A	G	nonsynonymous
339901	NTHI477_RS01660	2085	C	T	synonymous
339904	NTHI477_RS01660	2088	G	A	synonymous
339904	NTHI477_RS01660	2088	G	C	synonymous
339907	NTHI477_RS01660	2091	T	C	synonymous
339913	NTHI477_RS01660	2097	A	T	nonsynonymous
339913	NTHI477_RS01660	2097	A	C	nonsynonymous
339925	NTHI477_RS01660	2109	A	G	synonymous
339926	NTHI477_RS01660	2110	C	T	synonymous
339928	NTHI477_RS01660	2112	G	A	synonymous
339929	NTHI477_RS01660	2113	G	A	nonsynonymous
339931	NTHI477_RS01660	2115	T	G	synonymous
339937	NTHI477_RS01660	2121	C	T	synonymous
339942	NTHI477_RS01660	2126	T	A	nonsynonymous
339943	NTHI477_RS01660	2127	T	A	synonymous
339946	NTHI477_RS01660	2130	T	C	synonymous
339949	NTHI477_RS01660	2133	A	G	synonymous
339967	NTHI477_RS01660	2151	G	A	synonymous
339969	NTHI477_RS01660	2153	G	A	nonsynonymous
339970	NTHI477_RS01660	2154	T	C	synonymous
339977	NTHI477_RS01660	2161	G	T	nonsynonymous
339985	NTHI477_RS01660	2169	C	A	synonymous
339987	NTHI477_RS01660	2171	C	G	nonsynonymous
339988	NTHI477_RS01660	2172	T	C	synonymous
339989	NTHI477_RS01660	2173	C	T	synonymous
339997	NTHI477_RS01660	2181	A	G	synonymous
340000	NTHI477_RS01660	2184	G	A	synonymous
340004	NTHI477_RS01660	2188	G	A	nonsynonymous

340009	NTHI477_RS01660	2193	A	G	synonymous
340012	NTHI477_RS01660	2196	A	G	synonymous
340018	NTHI477_RS01660	2202	T	C	synonymous
340031	NTHI477_RS01660	2215	A	G	nonsynonymous
340032	NTHI477_RS01660	2216	C	T	nonsynonymous
340033	NTHI477_RS01660	2217	T	G	synonymous
340035	NTHI477_RS01660	2219	C	T	nonsynonymous
340054	NTHI477_RS01660	2238	G	A	synonymous
340055	NTHI477_RS01660	2239	A	C	nonsynonymous
340072	NTHI477_RS01660	2256	T	G	synonymous
340078	NTHI477_RS01660	2262	G	T	synonymous
340084	NTHI477_RS01660	2268	C	T	synonymous
340115	NTHI477_RS01660	2299	C	T	nonsynonymous
340141	NTHI477_RS01660	2325	A	G	synonymous
340165	NTHI477_RS01660	2349	A	C	synonymous
340168	NTHI477_RS01660	2352	A	G	synonymous
340171	NTHI477_RS01660	2355	T	A	synonymous
340183	NTHI477_RS01660	2367	C	T	synonymous
340192	NTHI477_RS01660	2376	A	G	synonymous
340193	NTHI477_RS01660	2377	G	A	nonsynonymous
340195	NTHI477_RS01660	2379	G	A	synonymous
340201	NTHI477_RS01660	2385	T	C	synonymous
340207	NTHI477_RS01660	2391	G	A	synonymous
340231	NTHI477_RS01660	2415	T	C	synonymous
340232	NTHI477_RS01660	2416	G	A	nonsynonymous
340238	NTHI477_RS01660	2422	G	A	nonsynonymous
340267	NTHI477_RS01660	2451	C	A	synonymous
340288	NTHI477_RS01660	2472	A	G	synonymous
340300	NTHI477_RS01660	2484	T	C	synonymous
340355	NTHI477_RS01660	2539	T	C	synonymous
340357	NTHI477_RS01660	2541	G	A	synonymous
340360	NTHI477_RS01660	2544	C	T	synonymous
340399	NTHI477_RS01660	2583	T	C	synonymous
340429	NTHI477_RS01660	2613	C	T	synonymous
340432	NTHI477_RS01660	2616	C	T	synonymous
340433	NTHI477_RS01660	2617	C	T	synonymous
340435	NTHI477_RS01660	2619	G	A	synonymous
340444	NTHI477_RS01660	2628	A	G	synonymous
340462	NTHI477_RS01660	2646	T	A	synonymous
340474	NTHI477_RS01660	2658	A	G	synonymous
340494	NTHI477_RS01660	2678	C	G	nonsynonymous
340495	NTHI477_RS01660	2679	G	C	synonymous
340498	NTHI477_RS01660	2682	A	G	synonymous
340504	NTHI477_RS01660	2688	A	G	synonymous
340513	NTHI477_RS01660	2697	A	G	synonymous

340519	NTHI477_RS01660	2703	T	C	synonymous
340525	NTHI477_RS01660	2709	C	T	synonymous
340530	NTHI477_RS01660	2714	C	T	nonsynonymous
340531	NTHI477_RS01660	2715	C	T	synonymous
340543	NTHI477_RS01660	2727	G	A	synonymous
340547	NTHI477_RS01660	2731	C	T	nonsynonymous
340549	NTHI477_RS01660	2733	C	A	synonymous
340552	NTHI477_RS01660	2736	C	A	synonymous
340571	NTHI477_RS01660	2755	C	A	nonsynonymous
340572	NTHI477_RS01660	2756	A	G	nonsynonymous
340582	NTHI477_RS01660	2766	A	G	synonymous
340589	NTHI477_RS01660	2773	C	T	synonymous
340606	NTHI477_RS01660	2790	T	C	synonymous
340621	NTHI477_RS01660	2805	G	T	synonymous
340667	NTHI477_RS01660	2851	G	A	nonsynonymous
340673	NTHI477_RS01660	2857	T	C	synonymous
340712	NTHI477_RS01660	2896	C	T	nonsynonymous
340714	NTHI477_RS01660	2898	T	A	synonymous
340729	NTHI477_RS01660	2913	C	T	synonymous
340811	NTHI477_RS01660	2995	A	C	synonymous
340819	NTHI477_RS01660	3003	G	A	synonymous
340837	NTHI477_RS01660	3021	A	G	synonymous
340846	NTHI477_RS01660	3030	G	A	synonymous
340849	NTHI477_RS01660	3033	G	T	synonymous
340852	NTHI477_RS01660	3036	A	G	synonymous
340855	NTHI477_RS01660	3039	A	G	synonymous
340860	NTHI477_RS01660	3044	A	G	nonsynonymous
340864	NTHI477_RS01660	3048	G	T	nonsynonymous
340865	NTHI477_RS01660	3049	C	A	nonsynonymous
340877	NTHI477_RS01660	3061	A	C	nonsynonymous
340878	NTHI477_RS01660	3062	A	G	nonsynonymous
340882	NTHI477_RS01660	3066	C	T	synonymous
340885	NTHI477_RS01660	3069	C	A	synonymous
340885	NTHI477_RS01660	3069	C	T	synonymous
340886	NTHI477_RS01660	3070	C	T	synonymous
340892	NTHI477_RS01660	3076	C	A	nonsynonymous
340893	NTHI477_RS01660	3077	G	A	nonsynonymous
340894	NTHI477_RS01660	3078	C	A	synonymous
340897	NTHI477_RS01660	3081	C	T	synonymous
340903	NTHI477_RS01660	3087	C	T	synonymous
340906	NTHI477_RS01660	3090	A	G	synonymous
340909	NTHI477_RS01660	3093	T	C	synonymous
340928	NTHI477_RS01660	3112	G	A	nonsynonymous
341061	intergenic	intergenic	A	G	intergenic
341062	intergenic	intergenic	G	A	intergenic

341066	NTHI477_RS01665	2487	G	A	synonymous
341069	NTHI477_RS01665	2484	T	C	synonymous
341075	NTHI477_RS01665	2478	T	C	synonymous
341081	NTHI477_RS01665	2472	T	C	synonymous
341096	NTHI477_RS01665	2457	C	T	synonymous
341102	NTHI477_RS01665	2451	G	A	synonymous
341114	NTHI477_RS01665	2439	G	A	synonymous
341126	NTHI477_RS01665	2427	A	G	synonymous
341162	NTHI477_RS01665	2391	G	A	synonymous
341180	NTHI477_RS01665	2373	A	G	synonymous
341183	NTHI477_RS01665	2370	A	G	synonymous
341210	NTHI477_RS01665	2343	T	C	synonymous
341213	NTHI477_RS01665	2340	C	T	synonymous
341222	NTHI477_RS01665	2331	G	T	synonymous
341246	NTHI477_RS01665	2307	G	A	synonymous
341249	NTHI477_RS01665	2304	A	G	synonymous
341255	NTHI477_RS01665	2298	A	G	synonymous
341279	NTHI477_RS01665	2274	G	A	synonymous
341291	NTHI477_RS01665	2262	A	G	synonymous
341321	NTHI477_RS01665	2232	G	A	synonymous
341345	NTHI477_RS01665	2208	A	G	synonymous
341411	NTHI477_RS01665	2142	C	T	synonymous
341420	NTHI477_RS01665	2133	G	A	synonymous
341435	NTHI477_RS01665	2118	A	G	synonymous
341455	NTHI477_RS01665	2098	C	A	nonsynonymous
341498	NTHI477_RS01665	2055	G	T	synonymous
341594	NTHI477_RS01665	1959	G	A	synonymous
341627	NTHI477_RS01665	1926	C	T	synonymous
341717	NTHI477_RS01665	1836	C	A	synonymous
341771	NTHI477_RS01665	1782	C	T	synonymous
341801	NTHI477_RS01665	1752	C	T	synonymous
341807	NTHI477_RS01665	1746	A	G	synonymous
341822	NTHI477_RS01665	1731	C	T	synonymous
341834	NTHI477_RS01665	1719	G	A	synonymous
341870	NTHI477_RS01665	1683	A	C	synonymous
341912	NTHI477_RS01665	1641	G	A	synonymous
341916	NTHI477_RS01665	1637	A	G	nonsynonymous
341921	NTHI477_RS01665	1632	G	A	synonymous
341951	NTHI477_RS01665	1602	A	G	synonymous
342044	NTHI477_RS01665	1509	G	C	synonymous
342056	NTHI477_RS01665	1497	A	G	synonymous
342059	NTHI477_RS01665	1494	G	A	synonymous
342116	NTHI477_RS01665	1437	T	A	synonymous
342137	NTHI477_RS01665	1416	T	A	synonymous
342176	NTHI477_RS01665	1377	G	T	synonymous

342213	NTHI477_RS01665	1340	A	G	nonsynonymous
342233	NTHI477_RS01665	1320	G	A	synonymous
342359	NTHI477_RS01665	1194	A	G	synonymous
342398	NTHI477_RS01665	1155	A	C	synonymous
342410	NTHI477_RS01665	1143	G	A	synonymous
342413	NTHI477_RS01665	1140	G	A	synonymous
342422	NTHI477_RS01665	1131	G	A	synonymous
342521	NTHI477_RS01665	1032	G	A	synonymous
342530	NTHI477_RS01665	1023	A	G	synonymous
342542	NTHI477_RS01665	1011	A	T	synonymous
342548	NTHI477_RS01665	1005	G	A	synonymous
342551	NTHI477_RS01665	1002	G	A	synonymous
342560	NTHI477_RS01665	993	T	A	synonymous
342569	NTHI477_RS01665	984	G	C	synonymous
342581	NTHI477_RS01665	972	T	C	synonymous
342593	NTHI477_RS01665	960	A	G	synonymous
342599	NTHI477_RS01665	954	G	A	synonymous
342632	NTHI477_RS01665	921	A	G	synonymous
342641	NTHI477_RS01665	912	C	T	synonymous
342662	NTHI477_RS01665	891	T	C	synonymous
342686	NTHI477_RS01665	867	G	A	synonymous
342704	NTHI477_RS01665	849	A	G	synonymous
342707	NTHI477_RS01665	846	A	G	synonymous
342716	NTHI477_RS01665	837	G	A	synonymous
342722	NTHI477_RS01665	831	C	T	synonymous
342733	NTHI477_RS01665	820	T	C	nonsynonymous
342737	NTHI477_RS01665	816	G	A	synonymous
342743	NTHI477_RS01665	810	C	T	synonymous
342749	NTHI477_RS01665	804	A	C	synonymous
342755	NTHI477_RS01665	798	T	C	synonymous
342760	NTHI477_RS01665	793	C	A	nonsynonymous
342764	NTHI477_RS01665	789	C	T	synonymous
342767	NTHI477_RS01665	786	A	G	synonymous
342785	NTHI477_RS01665	768	C	G	synonymous
342797	NTHI477_RS01665	756	T	G	synonymous
342803	NTHI477_RS01665	750	G	C	synonymous
342806	NTHI477_RS01665	747	T	A	synonymous
342811	NTHI477_RS01665	742	C	T	nonsynonymous
342812	NTHI477_RS01665	741	C	T	synonymous
342814	NTHI477_RS01665	739	A	C	nonsynonymous
342818	NTHI477_RS01665	735	A	T	synonymous
342828	NTHI477_RS01665	725	G	A	nonsynonymous
342830	NTHI477_RS01665	723	A	T	synonymous
342859	NTHI477_RS01665	694	C	T	nonsynonymous
342860	NTHI477_RS01665	693	A	G	synonymous

342869	NTHI477_RS01665	684	G	T	synonymous
342896	NTHI477_RS01665	657	G	A	synonymous
342959	NTHI477_RS01665	594	G	A	synonymous
342965	NTHI477_RS01665	588	G	T	synonymous
342974	NTHI477_RS01665	579	C	T	synonymous
342980	NTHI477_RS01665	573	C	T	synonymous
342995	NTHI477_RS01665	558	G	A	synonymous
342998	NTHI477_RS01665	555	A	T	synonymous
343001	NTHI477_RS01665	552	A	G	synonymous
343019	NTHI477_RS01665	534	A	G	synonymous
343063	NTHI477_RS01665	490	G	A	synonymous
343130	NTHI477_RS01665	423	C	A	synonymous
343140	NTHI477_RS01665	413	A	G	nonsynonymous
343178	NTHI477_RS01665	375	T	C	synonymous
343180	NTHI477_RS01665	373	A	C	nonsynonymous
343181	NTHI477_RS01665	372	T	C	synonymous
343205	NTHI477_RS01665	348	T	A	synonymous
343245	NTHI477_RS01665	308	G	A	nonsynonymous
343250	NTHI477_RS01665	303	A	C	synonymous
343251	NTHI477_RS01665	302	G	A	nonsynonymous
343275	NTHI477_RS01665	278	A	G	nonsynonymous
343286	NTHI477_RS01665	267	A	T	synonymous
343332	NTHI477_RS01665	221	G	A	nonsynonymous
343333	NTHI477_RS01665	220	C	T	nonsynonymous
343363	NTHI477_RS01665	190	T	C	nonsynonymous
343481	NTHI477_RS01665	72	G	A	synonymous
343526	NTHI477_RS01665	27	C	T	synonymous
343557	intergenic	intergenic	G	A	intergenic
343585	NTHI477_RS01670	1467	A	G	synonymous
343615	NTHI477_RS01670	1437	G	T	synonymous
343625	NTHI477_RS01670	1427	G	A	nonsynonymous
343627	NTHI477_RS01670	1425	G	T	synonymous
343645	NTHI477_RS01670	1407	A	T	synonymous
343647	NTHI477_RS01670	1405	T	C	nonsynonymous
343657	NTHI477_RS01670	1395	A	T	synonymous
343660	NTHI477_RS01670	1392	G	A	synonymous
343663	NTHI477_RS01670	1389	T	C	synonymous
343669	NTHI477_RS01670	1383	T	G	synonymous
343681	NTHI477_RS01670	1371	A	G	synonymous
343684	NTHI477_RS01670	1368	G	A	synonymous
343687	NTHI477_RS01670	1365	G	A	synonymous
343702	NTHI477_RS01670	1350	T	A	synonymous
343707	NTHI477_RS01670	1345	G	A	synonymous
343709	NTHI477_RS01670	1343	T	C	nonsynonymous
343713	NTHI477_RS01670	1339	T	A	nonsynonymous

343714	NTHI477_RS01670	1338	T	C	synonymous
343717	NTHI477_RS01670	1335	A	C	synonymous
343720	NTHI477_RS01670	1332	G	A	synonymous
343723	NTHI477_RS01670	1329	A	G	synonymous
343732	NTHI477_RS01670	1320	T	A	synonymous
343735	NTHI477_RS01670	1317	C	A	nonsynonymous
343750	NTHI477_RS01670	1302	A	G	synonymous
343761	NTHI477_RS01670	1291	C	T	nonsynonymous
343765	NTHI477_RS01670	1287	C	T	synonymous
343776	NTHI477_RS01670	1276	G	A	synonymous
343780	NTHI477_RS01670	1272	T	C	synonymous
343834	NTHI477_RS01670	1218	C	T	synonymous
343894	NTHI477_RS01670	1158	A	G	synonymous
343921	NTHI477_RS01670	1131	T	G	synonymous
343930	NTHI477_RS01670	1122	T	C	synonymous
343942	NTHI477_RS01670	1110	T	A	synonymous
343969	NTHI477_RS01670	1083	G	A	synonymous
343993	NTHI477_RS01670	1059	A	G	synonymous
344056	NTHI477_RS01670	996	C	T	synonymous
344080	NTHI477_RS01670	972	A	G	synonymous
344113	NTHI477_RS01670	939	A	G	synonymous
344182	NTHI477_RS01670	870	T	A	synonymous
344185	NTHI477_RS01670	867	T	C	synonymous
344191	NTHI477_RS01670	861	C	T	synonymous
344197	NTHI477_RS01670	855	A	G	synonymous
344209	NTHI477_RS01670	843	C	T	synonymous
344212	NTHI477_RS01670	840	G	T	synonymous
344218	NTHI477_RS01670	834	G	A	synonymous
344248	NTHI477_RS01670	804	G	A	synonymous
344251	NTHI477_RS01670	801	A	G	synonymous
344263	NTHI477_RS01670	789	C	G	synonymous
344269	NTHI477_RS01670	783	T	G	synonymous
344270	NTHI477_RS01670	782	A	G	nonsynonymous
344272	NTHI477_RS01670	780	C	T	synonymous
344299	NTHI477_RS01670	753	A	G	synonymous
344311	NTHI477_RS01670	741	T	A	synonymous
344317	NTHI477_RS01670	735	A	G	synonymous
344320	NTHI477_RS01670	732	T	A	synonymous
344323	NTHI477_RS01670	729	C	T	synonymous
344338	NTHI477_RS01670	714	C	T	synonymous
344341	NTHI477_RS01670	711	T	C	synonymous
344344	NTHI477_RS01670	708	A	G	synonymous
344353	NTHI477_RS01670	699	A	G	synonymous
344359	NTHI477_RS01670	693	G	A	synonymous
344362	NTHI477_RS01670	690	A	C	synonymous

344386	NTHI477_RS01670	666	T	C	synonymous
344506	NTHI477_RS01670	546	T	C	synonymous
344527	NTHI477_RS01670	525	C	A	synonymous
344563	NTHI477_RS01670	489	C	T	synonymous
344593	NTHI477_RS01670	459	A	G	synonymous
344629	NTHI477_RS01670	423	A	G	synonymous
344689	NTHI477_RS01670	363	G	A	synonymous
344725	NTHI477_RS01670	327	C	T	synonymous
344773	NTHI477_RS01670	279	T	C	synonymous
344806	NTHI477_RS01670	246	A	G	synonymous
344812	NTHI477_RS01670	240	C	G	synonymous
344815	NTHI477_RS01670	237	C	T	synonymous
344823	NTHI477_RS01670	229	A	G	synonymous
344833	NTHI477_RS01670	219	C	T	synonymous
344914	NTHI477_RS01670	138	G	T	synonymous
344950	NTHI477_RS01670	102	G	A	synonymous
344953	NTHI477_RS01670	99	A	G	synonymous
345085	NTHI477_RS01675	439	C	T	nonsynonymous
345167	NTHI477_RS01675	357	C	T	synonymous
345175	NTHI477_RS01675	349	T	G	nonsynonymous
345284	NTHI477_RS01675	240	A	G	synonymous
345284	NTHI477_RS01675	240	A	T	synonymous
345305	NTHI477_RS01675	219	A	G	synonymous
345373	NTHI477_RS01675	151	C	T	nonsynonymous
345380	NTHI477_RS01675	144	T	C	synonymous
345391	NTHI477_RS01675	133	C	T	nonsynonymous
345392	NTHI477_RS01675	132	T	A	nonsynonymous
345413	NTHI477_RS01675	111	A	C	synonymous
345416	NTHI477_RS01675	108	T	A	synonymous
345428	NTHI477_RS01675	96	A	C	synonymous
345432	NTHI477_RS01675	92	A	G	nonsynonymous
345433	NTHI477_RS01675	91	C	T	nonsynonymous
345449	NTHI477_RS01675	75	T	C	synonymous
345473	NTHI477_RS01675	51	C	T	synonymous
345475	NTHI477_RS01675	49	T	C	nonsynonymous
345479	NTHI477_RS01675	45	C	T	synonymous
345540	intergenic	intergenic	G	A	intergenic
345545	intergenic	intergenic	T	C	intergenic
345552	intergenic	intergenic	C	T	intergenic
345584	intergenic	intergenic	C	T	intergenic
345618	intergenic	intergenic	G	A	intergenic
345620	intergenic	intergenic	A	G	intergenic
345742	intergenic	intergenic	T	C	intergenic
345833	intergenic	intergenic	T	C	intergenic
345834	intergenic	intergenic	G	A	intergenic

345836	intergenic	intergenic	T	C	intergenic
345837	intergenic	intergenic	A	G	intergenic
345842	intergenic	intergenic	A	G	intergenic
345924	intergenic	intergenic	G	A	intergenic
345965	intergenic	intergenic	T	C	intergenic
346003	intergenic	intergenic	C	A	intergenic
346059	intergenic	intergenic	C	T	intergenic
346078	intergenic	intergenic	G	T	intergenic
346079	intergenic	intergenic	T	G	intergenic
346168	intergenic	intergenic	G	A	intergenic
346177	intergenic	intergenic	G	C	intergenic
346216	intergenic	intergenic	G	C	intergenic
346230	intergenic	intergenic	C	T	intergenic
346238	intergenic	intergenic	A	G	intergenic
346283	intergenic	intergenic	G	A	intergenic
346392	intergenic	intergenic	C	T	intergenic
346397	intergenic	intergenic	T	C	intergenic
346407	intergenic	intergenic	G	T	intergenic
346470	intergenic	intergenic	A	G	intergenic
346489	intergenic	intergenic	G	A	intergenic
346500	intergenic	intergenic	G	A	intergenic
346535	intergenic	intergenic	G	A	intergenic
346579	intergenic	intergenic	A	G	intergenic
346889	intergenic	intergenic	T	G	intergenic
346918	intergenic	intergenic	T	A	intergenic
346955	intergenic	intergenic	G	T	intergenic
347143	intergenic	intergenic	C	A	intergenic
373626	intergenic	intergenic	T	C	intergenic
373631	intergenic	intergenic	C	T	intergenic
373646	intergenic	intergenic	T	C	intergenic
373647	intergenic	intergenic	G	A	intergenic
373653	intergenic	intergenic	T	C	intergenic
373660	intergenic	intergenic	G	A	intergenic
373707	intergenic	intergenic	C	T	intergenic
373764	NTHI477_RS01850	30	T	G	synonymous
373773	NTHI477_RS01850	39	C	T	synonymous
373782	NTHI477_RS01850	48	C	T	synonymous
373784	NTHI477_RS01850	50	G	A	nonsynonymous
373785	NTHI477_RS01850	51	C	T	synonymous
373803	NTHI477_RS01850	69	C	T	synonymous
373806	NTHI477_RS01850	72	A	C	synonymous
373807	NTHI477_RS01850	73	A	G	nonsynonymous
373881	NTHI477_RS01850	147	T	C	synonymous
373893	NTHI477_RS01850	159	A	G	synonymous
373918	NTHI477_RS01850	184	T	C	synonymous

373920	NTHI477_RS01850	186	A	G	synonymous
373956	NTHI477_RS01850	222	A	G	nonsynonymous
373980	NTHI477_RS01850	246	T	C	synonymous
373989	NTHI477_RS01850	255	A	G	synonymous
373993	NTHI477_RS01850	259	C	T	nonsynonymous
373995	NTHI477_RS01850	261	T	C	synonymous
373995	NTHI477_RS01850	261	T	A	synonymous
374001	NTHI477_RS01850	267	A	G	synonymous
374019	NTHI477_RS01850	285	T	C	synonymous
374025	NTHI477_RS01850	291	G	A	synonymous
374040	NTHI477_RS01850	306	A	G	synonymous
374045	NTHI477_RS01850	311	C	T	nonsynonymous
374046	NTHI477_RS01850	312	G	A	synonymous
374046	NTHI477_RS01850	312	G	T	synonymous
374049	NTHI477_RS01850	315	C	T	synonymous
374082	NTHI477_RS01850	348	C	T	synonymous
374098	NTHI477_RS01850	364	A	G	nonsynonymous
374101	NTHI477_RS01850	367	C	T	synonymous
374124	NTHI477_RS01850	390	A	G	synonymous
374127	NTHI477_RS01850	393	G	C	synonymous
374132	NTHI477_RS01850	398	G	A	nonsynonymous
374178	NTHI477_RS01850	444	C	T	synonymous
374193	NTHI477_RS01850	459	T	C	synonymous
374199	NTHI477_RS01850	465	A	C	synonymous
374202	NTHI477_RS01850	468	T	C	synonymous
374203	NTHI477_RS01850	469	T	G	nonsynonymous
374205	NTHI477_RS01850	471	A	C	synonymous
374217	NTHI477_RS01850	483	C	T	synonymous
374220	NTHI477_RS01850	486	C	T	synonymous
374250	NTHI477_RS01850	516	C	T	synonymous
374269	NTHI477_RS01850	535	G	A	nonsynonymous
374278	NTHI477_RS01850	544	G	A	nonsynonymous
374286	NTHI477_RS01850	552	C	T	synonymous
374313	NTHI477_RS01850	579	C	T	synonymous
374331	NTHI477_RS01850	597	T	C	synonymous
374338	NTHI477_RS01850	604	C	T	nonsynonymous
374347	NTHI477_RS01850	613	G	A	nonsynonymous
374365	NTHI477_RS01850	631	A	G	nonsynonymous
374366	NTHI477_RS01850	632	C	G	nonsynonymous
374367	NTHI477_RS01850	633	G	A	synonymous
374370	NTHI477_RS01850	636	G	A	synonymous
374383	NTHI477_RS01850	649	A	C	synonymous
374384	NTHI477_RS01850	650	G	A	nonsynonymous
374393	NTHI477_RS01850	659	G	A	nonsynonymous
374405	intergenic	intergenic	A	G	intergenic

374420	intergenic	intergenic	C	T	intergenic
374422	intergenic	intergenic	A	C	intergenic
374442	intergenic	intergenic	G	A	intergenic
374471	NTHI477_RS01855	1086	G	A	synonymous
374516	NTHI477_RS01855	1041	T	C	synonymous
374519	NTHI477_RS01855	1038	T	G	synonymous
374530	NTHI477_RS01855	1027	A	G	synonymous
374536	NTHI477_RS01855	1021	C	T	nonsynonymous
374570	NTHI477_RS01855	987	G	A	synonymous
374573	NTHI477_RS01855	984	C	T	synonymous
374582	NTHI477_RS01855	975	T	A	synonymous
374588	NTHI477_RS01855	969	T	C	synonymous
374612	NTHI477_RS01855	945	C	T	synonymous
374614	NTHI477_RS01855	943	G	A	synonymous
374636	NTHI477_RS01855	921	C	T	synonymous
374651	NTHI477_RS01855	906	C	A	synonymous
374663	NTHI477_RS01855	894	T	A	synonymous
374666	NTHI477_RS01855	891	A	T	synonymous
374684	NTHI477_RS01855	873	T	C	synonymous
374693	NTHI477_RS01855	864	A	G	synonymous
374705	NTHI477_RS01855	852	A	G	synonymous
374714	NTHI477_RS01855	843	A	G	synonymous
374720	NTHI477_RS01855	837	C	T	synonymous
374734	NTHI477_RS01855	823	C	T	nonsynonymous
374737	NTHI477_RS01855	820	C	T	nonsynonymous
374738	NTHI477_RS01855	819	G	A	synonymous
374741	NTHI477_RS01855	816	T	C	synonymous
374744	NTHI477_RS01855	813	C	T	synonymous
374747	NTHI477_RS01855	810	T	C	synonymous
374747	NTHI477_RS01855	810	T	G	synonymous
374750	NTHI477_RS01855	807	T	C	synonymous
374750	NTHI477_RS01855	807	T	G	synonymous
374753	NTHI477_RS01855	804	G	A	synonymous
374765	NTHI477_RS01855	792	A	G	synonymous
374774	NTHI477_RS01855	783	G	A	synonymous
374783	NTHI477_RS01855	774	C	T	synonymous
374786	NTHI477_RS01855	771	T	C	synonymous
374789	NTHI477_RS01855	768	A	G	synonymous
374797	NTHI477_RS01855	760	G	A	synonymous
374810	NTHI477_RS01855	747	T	C	synonymous
374813	NTHI477_RS01855	744	A	G	synonymous
374822	NTHI477_RS01855	735	A	T	synonymous
374825	NTHI477_RS01855	732	C	G	synonymous
374828	NTHI477_RS01855	729	T	G	synonymous
374831	NTHI477_RS01855	726	A	G	synonymous

374840	NTHI477_RS01855	717	A	G	synonymous
374843	NTHI477_RS01855	714	G	A	synonymous
374852	NTHI477_RS01855	705	T	C	synonymous
374882	NTHI477_RS01855	675	A	C	synonymous
374899	NTHI477_RS01855	658	T	C	nonsynonymous
374948	NTHI477_RS01855	609	T	C	synonymous
374951	NTHI477_RS01855	606	A	G	synonymous
374954	NTHI477_RS01855	603	A	G	synonymous
374955	NTHI477_RS01855	602	C	T	nonsynonymous
374963	NTHI477_RS01855	594	T	C	synonymous
374969	NTHI477_RS01855	588	A	G	synonymous
374984	NTHI477_RS01855	573	G	A	synonymous
374987	NTHI477_RS01855	570	A	G	synonymous
375032	NTHI477_RS01855	525	T	G	synonymous
375047	NTHI477_RS01855	510	A	G	synonymous
375071	NTHI477_RS01855	486	C	T	synonymous
375074	NTHI477_RS01855	483	G	A	synonymous
375077	NTHI477_RS01855	480	T	C	synonymous
375080	NTHI477_RS01855	477	A	G	synonymous
375082	NTHI477_RS01855	475	C	T	nonsynonymous
375089	NTHI477_RS01855	468	A	G	synonymous
375098	NTHI477_RS01855	459	G	A	synonymous
375101	NTHI477_RS01855	456	T	A	synonymous
375113	NTHI477_RS01855	444	A	G	synonymous
375113	NTHI477_RS01855	444	A	C	synonymous
375137	NTHI477_RS01855	420	C	T	synonymous
375146	NTHI477_RS01855	411	T	A	synonymous
375149	NTHI477_RS01855	408	G	A	synonymous
375173	NTHI477_RS01855	384	T	G	synonymous
375182	NTHI477_RS01855	375	G	A	synonymous
375188	NTHI477_RS01855	369	C	T	synonymous
375197	NTHI477_RS01855	360	C	T	synonymous
375206	NTHI477_RS01855	351	G	A	synonymous
375212	NTHI477_RS01855	345	A	G	synonymous
375224	NTHI477_RS01855	333	A	C	synonymous
375233	NTHI477_RS01855	324	A	G	synonymous
375251	NTHI477_RS01855	306	A	G	synonymous
375251	NTHI477_RS01855	306	A	T	synonymous
375254	NTHI477_RS01855	303	A	T	synonymous
375254	NTHI477_RS01855	303	A	C	synonymous
375275	NTHI477_RS01855	282	A	G	synonymous
375295	NTHI477_RS01855	262	C	T	nonsynonymous
375312	NTHI477_RS01855	245	A	G	nonsynonymous
375313	NTHI477_RS01855	244	A	C	nonsynonymous
375314	NTHI477_RS01855	243	T	G	nonsynonymous

375317	NTHI477_RS01855	240	A	G	synonymous
375335	NTHI477_RS01855	222	C	T	synonymous
375339	NTHI477_RS01855	218	G	A	nonsynonymous
375340	NTHI477_RS01855	217	C	A	nonsynonymous
375362	NTHI477_RS01855	195	C	T	synonymous
375369	NTHI477_RS01855	188	G	A	nonsynonymous
375374	NTHI477_RS01855	183	A	T	synonymous
375383	NTHI477_RS01855	174	T	C	synonymous
375392	NTHI477_RS01855	165	C	T	synonymous
375397	NTHI477_RS01855	160	T	G	nonsynonymous
375407	NTHI477_RS01855	150	A	G	synonymous
375413	NTHI477_RS01855	144	C	A	synonymous
375413	NTHI477_RS01855	144	C	G	synonymous
375419	NTHI477_RS01855	138	A	T	synonymous
375419	NTHI477_RS01855	138	A	G	synonymous
375422	NTHI477_RS01855	135	G	T	synonymous
375453	NTHI477_RS01855	104	A	G	nonsynonymous
375472	NTHI477_RS01855	85	G	A	synonymous
375473	NTHI477_RS01855	84	A	G	synonymous
375494	NTHI477_RS01855	63	A	G	synonymous
375512	NTHI477_RS01855	45	G	A	synonymous
375521	NTHI477_RS01855	36	G	C	nonsynonymous
375591	intergenic	intergenic	G	A	intergenic
375612	intergenic	intergenic	G	A	intergenic
375613	intergenic	intergenic	T	C	intergenic
375654	intergenic	intergenic	C	T	intergenic
375742	NTHI477_RS01860	36	C	T	synonymous
375859	NTHI477_RS01860	153	A	G	synonymous
375862	NTHI477_RS01860	156	C	T	synonymous
375874	NTHI477_RS01860	168	C	T	synonymous
375877	NTHI477_RS01860	171	G	A	synonymous
375887	NTHI477_RS01860	181	T	C	synonymous
375895	NTHI477_RS01860	189	T	C	synonymous
375931	NTHI477_RS01860	225	T	C	synonymous
375950	NTHI477_RS01860	244	A	G	nonsynonymous
375952	NTHI477_RS01860	246	T	G	nonsynonymous
375963	NTHI477_RS01860	257	C	T	nonsynonymous
375970	NTHI477_RS01860	264	C	T	synonymous
375991	NTHI477_RS01860	285	T	C	synonymous
375997	NTHI477_RS01860	291	C	T	synonymous
376021	NTHI477_RS01860	315	G	A	synonymous
376027	NTHI477_RS01860	321	T	C	synonymous
376060	NTHI477_RS01860	354	C	T	synonymous
376096	NTHI477_RS01860	390	C	T	synonymous
376120	NTHI477_RS01860	414	T	C	synonymous

376153	NTHI477_RS01860	447	C	T	synonymous
376159	NTHI477_RS01860	453	A	G	synonymous
376189	NTHI477_RS01860	483	G	T	synonymous
376192	NTHI477_RS01860	486	T	C	synonymous
376195	NTHI477_RS01860	489	T	A	synonymous
376204	NTHI477_RS01860	498	T	C	synonymous
376207	NTHI477_RS01860	501	C	T	synonymous
376246	NTHI477_RS01860	540	C	T	synonymous
376247	NTHI477_RS01860	541	A	G	nonsynonymous
376256	NTHI477_RS01860	550	A	G	nonsynonymous
376258	NTHI477_RS01860	552	C	T	synonymous
376258	NTHI477_RS01860	552	C	A	synonymous
376291	NTHI477_RS01860	585	G	A	synonymous
376306	NTHI477_RS01860	600	G	C	synonymous
376324	NTHI477_RS01860	618	T	A	synonymous
376357	NTHI477_RS01860	651	T	C	synonymous
376378	NTHI477_RS01860	672	G	A	synonymous
376408	NTHI477_RS01860	702	T	C	synonymous
376414	NTHI477_RS01860	708	A	G	synonymous
376438	NTHI477_RS01860	732	C	G	synonymous
376438	NTHI477_RS01860	732	C	A	synonymous
376441	NTHI477_RS01860	735	C	T	synonymous
376442	NTHI477_RS01860	736	G	A	nonsynonymous
376477	NTHI477_RS01860	771	G	A	synonymous
376480	NTHI477_RS01860	774	A	T	synonymous
376513	NTHI477_RS01860	807	T	C	synonymous
376514	NTHI477_RS01860	808	G	A	nonsynonymous
376516	NTHI477_RS01860	810	G	T	nonsynonymous
376524	NTHI477_RS01860	818	G	A	nonsynonymous
376537	NTHI477_RS01860	831	C	T	synonymous
376549	NTHI477_RS01860	843	T	G	synonymous
376558	NTHI477_RS01860	852	C	T	synonymous
376559	NTHI477_RS01860	853	G	A	nonsynonymous
376591	NTHI477_RS01860	885	T	C	synonymous
376603	NTHI477_RS01860	897	C	T	synonymous
376609	NTHI477_RS01860	903	C	T	synonymous
376615	NTHI477_RS01860	909	C	T	synonymous
376621	NTHI477_RS01860	915	T	A	synonymous
376639	NTHI477_RS01860	933	T	C	synonymous
376645	NTHI477_RS01860	939	T	C	synonymous
376667	NTHI477_RS01860	961	G	A	nonsynonymous
376672	NTHI477_RS01860	966	T	C	synonymous
376675	NTHI477_RS01860	969	C	T	synonymous
376681	NTHI477_RS01860	975	C	A	synonymous
376699	NTHI477_RS01860	993	G	A	synonymous

376717	NTHI477_RS01860	1011	C	G	synonymous
376720	NTHI477_RS01860	1014	A	T	synonymous
376783	NTHI477_RS01860	1077	A	G	synonymous
376828	NTHI477_RS01860	1122	A	C	nonsynonymous
376852	NTHI477_RS01860	1146	A	G	synonymous
376858	NTHI477_RS01860	1152	C	T	synonymous
376864	NTHI477_RS01860	1158	C	T	synonymous
376879	NTHI477_RS01860	1173	A	G	synonymous
376894	NTHI477_RS01860	1188	A	G	synonymous
376897	NTHI477_RS01860	1191	T	C	synonymous
376906	NTHI477_RS01860	1200	G	C	synonymous
376928	NTHI477_RS01860	1222	G	T	nonsynonymous
376930	NTHI477_RS01860	1224	G	A	synonymous
376937	NTHI477_RS01860	1231	C	T	synonymous
376945	NTHI477_RS01860	1239	C	T	synonymous
376949	NTHI477_RS01860	1243	G	A	nonsynonymous
376949	NTHI477_RS01860	1243	G	T	nonsynonymous
376972	NTHI477_RS01860	1266	T	C	synonymous
376987	NTHI477_RS01860	1281	C	T	synonymous
376990	NTHI477_RS01860	1284	C	T	synonymous
376993	NTHI477_RS01860	1287	T	C	synonymous
377032	NTHI477_RS01860	1326	A	G	synonymous
377065	NTHI477_RS01860	1359	T	C	synonymous
377089	NTHI477_RS01860	1383	A	G	synonymous
377116	NTHI477_RS01860	1410	A	C	synonymous
377143	NTHI477_RS01860	1437	T	A	synonymous
377161	NTHI477_RS01860	1455	A	G	synonymous
377182	NTHI477_RS01860	1476	C	T	synonymous
377197	NTHI477_RS01860	1491	A	G	synonymous
377212	NTHI477_RS01860	1506	A	G	synonymous
377213	NTHI477_RS01860	1507	A	G	nonsynonymous
377260	NTHI477_RS01860	1554	G	A	synonymous
377264	NTHI477_RS01860	1558	G	A	nonsynonymous
377266	NTHI477_RS01860	1560	T	C	synonymous
377270	NTHI477_RS01860	1564	A	G	nonsynonymous
377272	NTHI477_RS01860	1566	C	T	synonymous
377281	NTHI477_RS01860	1575	G	A	synonymous
377284	NTHI477_RS01860	1578	T	C	synonymous
377321	NTHI477_RS01860	1615	G	A	nonsynonymous
377326	NTHI477_RS01860	1620	C	T	synonymous
377329	NTHI477_RS01860	1623	T	A	synonymous
377341	NTHI477_RS01860	1635	C	T	synonymous
377356	NTHI477_RS01860	1650	A	C	synonymous
377368	NTHI477_RS01860	1662	A	G	synonymous
377370	NTHI477_RS01860	1664	C	T	nonsynonymous

377371	NTHI477_RS01860	1665	C	A	synonymous
377374	NTHI477_RS01860	1668	T	A	synonymous
377383	NTHI477_RS01860	1677	A	T	synonymous
377408	NTHI477_RS01860	1702	A	G	nonsynonymous
377410	NTHI477_RS01860	1704	C	T	synonymous
377414	NTHI477_RS01860	1708	G	A	nonsynonymous
377437	NTHI477_RS01860	1731	G	C	synonymous
377437	NTHI477_RS01860	1731	G	T	synonymous
377447	NTHI477_RS01860	1741	T	C	nonsynonymous
377452	NTHI477_RS01860	1746	C	T	synonymous
377479	NTHI477_RS01860	1773	A	G	synonymous
377480	NTHI477_RS01860	1774	C	T	synonymous
377494	NTHI477_RS01860	1788	A	T	synonymous
377497	NTHI477_RS01860	1791	G	A	synonymous
377518	NTHI477_RS01860	1812	A	G	synonymous
377530	NTHI477_RS01860	1824	G	A	synonymous
377539	NTHI477_RS01860	1833	T	C	synonymous
377587	NTHI477_RS01860	1881	A	G	synonymous
377629	NTHI477_RS01860	1923	C	T	synonymous
377656	NTHI477_RS01860	1950	C	T	synonymous
377659	NTHI477_RS01860	1953	G	A	synonymous
377665	NTHI477_RS01860	1959	G	A	synonymous
377675	NTHI477_RS01860	1969	C	T	synonymous
377701	NTHI477_RS01860	1995	C	T	synonymous
377710	NTHI477_RS01860	2004	A	G	synonymous
377711	NTHI477_RS01860	2005	C	T	nonsynonymous
377713	NTHI477_RS01860	2007	T	A	synonymous
377719	NTHI477_RS01860	2013	T	C	synonymous
377724	NTHI477_RS01860	2018	G	A	nonsynonymous
377725	NTHI477_RS01860	2019	C	T	synonymous
377734	NTHI477_RS01860	2028	T	C	synonymous
377736	NTHI477_RS01860	2030	C	T	nonsynonymous
377737	NTHI477_RS01860	2031	G	A	synonymous
377740	NTHI477_RS01860	2034	C	T	synonymous
377772	intergenic	intergenic	G	T	intergenic
377792	intergenic	intergenic	C	T	intergenic
377816	intergenic	intergenic	G	A	intergenic
377817	intergenic	intergenic	T	C	intergenic
377819	intergenic	intergenic	A	G	intergenic
377832	intergenic	intergenic	T	C	intergenic
377854	intergenic	intergenic	G	A	intergenic
377885	NTHI477_RS01865	18	T	C	synonymous
377906	NTHI477_RS01865	39	T	G	synonymous
377921	NTHI477_RS01865	54	C	A	nonsynonymous
377945	NTHI477_RS01865	78	G	A	synonymous

377951	NTHI477_RS01865	84	C	T	synonymous
377962	NTHI477_RS01865	95	G	A	nonsynonymous
377963	NTHI477_RS01865	96	C	T	synonymous
377972	NTHI477_RS01865	105	A	G	synonymous
377981	NTHI477_RS01865	114	T	C	synonymous
377993	NTHI477_RS01865	126	C	T	synonymous
377996	NTHI477_RS01865	129	A	G	synonymous
377997	NTHI477_RS01865	130	C	A	nonsynonymous
377999	NTHI477_RS01865	132	C	T	synonymous
378011	NTHI477_RS01865	144	G	A	synonymous
378017	NTHI477_RS01865	150	C	T	synonymous
378017	NTHI477_RS01865	150	C	A	synonymous
378033	NTHI477_RS01865	166	A	G	nonsynonymous
378038	NTHI477_RS01865	171	C	T	synonymous
378053	NTHI477_RS01865	186	C	G	synonymous
378063	NTHI477_RS01865	196	G	C	nonsynonymous
378065	NTHI477_RS01865	198	G	C	nonsynonymous
378077	NTHI477_RS01865	210	T	C	synonymous
378089	NTHI477_RS01865	222	A	G	synonymous
378101	NTHI477_RS01865	234	T	A	synonymous
378107	NTHI477_RS01865	240	T	A	synonymous
378107	NTHI477_RS01865	240	T	G	synonymous
378119	NTHI477_RS01865	252	C	T	synonymous
378120	NTHI477_RS01865	253	C	T	synonymous
378131	NTHI477_RS01865	264	C	G	synonymous
378190	NTHI477_RS01865	323	T	C	nonsynonymous
378203	NTHI477_RS01865	336	G	A	synonymous
378257	NTHI477_RS01865	390	A	C	synonymous
378302	NTHI477_RS01865	435	G	A	synonymous
378377	NTHI477_RS01865	510	G	T	synonymous
378399	NTHI477_RS01865	532	G	T	nonsynonymous
378401	NTHI477_RS01865	534	C	T	synonymous
378443	NTHI477_RS01865	576	G	A	synonymous
378453	NTHI477_RS01865	586	T	C	nonsynonymous
378473	NTHI477_RS01865	606	G	A	synonymous
378485	NTHI477_RS01865	618	T	C	synonymous
378503	NTHI477_RS01865	636	T	C	synonymous
378518	NTHI477_RS01865	651	A	G	synonymous
378524	NTHI477_RS01865	657	C	T	synonymous
378536	NTHI477_RS01865	669	T	C	synonymous
378539	NTHI477_RS01865	672	A	T	synonymous
378539	NTHI477_RS01865	672	A	C	synonymous
378542	NTHI477_RS01865	675	G	A	nonsynonymous
378548	NTHI477_RS01865	681	T	A	synonymous
378555	NTHI477_RS01865	688	G	A	nonsynonymous

378572	NTHI477_RS01865	705	A	G	synonymous
378578	NTHI477_RS01865	711	T	A	synonymous
378596	NTHI477_RS01865	729	C	T	synonymous
378602	NTHI477_RS01865	735	G	A	synonymous
378650	NTHI477_RS01865	783	G	A	nonsynonymous
378668	NTHI477_RS01865	801	C	T	synonymous
378707	NTHI477_RS01865	840	C	G	synonymous
378707	NTHI477_RS01865	840	C	A	synonymous
378716	NTHI477_RS01865	849	G	A	synonymous
378718	NTHI477_RS01865	851	G	A	nonsynonymous
378729	NTHI477_RS01865	862	C	T	nonsynonymous
378731	intergenic	intergenic	C	T	intergenic
378748	intergenic	intergenic	A	G	intergenic
378760	intergenic	intergenic	C	G	intergenic
378761	intergenic	intergenic	A	C	intergenic
378762	intergenic	intergenic	T	C	intergenic
378764	intergenic	intergenic	A	C	intergenic
378787	NTHI477_RS01870	687	G	T	synonymous
378787	NTHI477_RS01870	687	G	A	synonymous
378790	NTHI477_RS01870	684	T	C	synonymous
378802	NTHI477_RS01870	672	A	G	synonymous
378853	NTHI477_RS01870	621	A	T	synonymous
378868	NTHI477_RS01870	606	T	C	synonymous
378877	NTHI477_RS01870	597	C	A	synonymous
378877	NTHI477_RS01870	597	C	T	synonymous
378878	NTHI477_RS01870	596	G	A	nonsynonymous
378945	NTHI477_RS01870	529	A	G	synonymous
378970	NTHI477_RS01870	504	C	T	synonymous
378976	NTHI477_RS01870	498	G	T	synonymous
378985	NTHI477_RS01870	489	A	G	synonymous
378991	NTHI477_RS01870	483	T	A	synonymous
379019	NTHI477_RS01870	455	G	C	nonsynonymous
379020	NTHI477_RS01870	454	T	C	nonsynonymous
379027	NTHI477_RS01870	447	A	C	synonymous
379033	NTHI477_RS01870	441	C	T	synonymous
379039	NTHI477_RS01870	435	T	A	nonsynonymous
379051	NTHI477_RS01870	423	C	T	synonymous
379054	NTHI477_RS01870	420	A	G	synonymous
379060	NTHI477_RS01870	414	G	A	synonymous
379066	NTHI477_RS01870	408	A	G	synonymous
379078	NTHI477_RS01870	396	G	A	synonymous
379084	NTHI477_RS01870	390	T	A	synonymous
379102	NTHI477_RS01870	372	G	A	synonymous
379105	NTHI477_RS01870	369	A	G	synonymous
379123	NTHI477_RS01870	351	T	C	synonymous

379156	NTHI477_RS01870	318	C	A	synonymous
379185	NTHI477_RS01870	289	T	G	nonsynonymous
379188	NTHI477_RS01870	286	C	T	nonsynonymous
379194	NTHI477_RS01870	280	G	A	nonsynonymous
379199	NTHI477_RS01870	275	T	G	nonsynonymous
379215	NTHI477_RS01870	259	T	C	nonsynonymous
379222	NTHI477_RS01870	252	C	T	synonymous
379260	NTHI477_RS01870	214	G	A	nonsynonymous
379264	NTHI477_RS01870	210	T	C	synonymous
379288	NTHI477_RS01870	186	C	T	synonymous
379309	NTHI477_RS01870	165	A	G	synonymous
379309	NTHI477_RS01870	165	A	C	nonsynonymous
379312	NTHI477_RS01870	162	A	G	synonymous
379341	NTHI477_RS01870	133	T	C	nonsynonymous
379342	NTHI477_RS01870	132	C	T	synonymous
379347	NTHI477_RS01870	127	C	A	nonsynonymous
379357	NTHI477_RS01870	117	C	A	nonsynonymous
379368	NTHI477_RS01870	106	C	T	nonsynonymous
379408	NTHI477_RS01870	66	C	A	synonymous
379412	NTHI477_RS01870	62	A	T	nonsynonymous
379459	NTHI477_RS01870	15	T	C	synonymous
379486	intergenic	intergenic	A	C	intergenic
379486	intergenic	intergenic	A	T	intergenic
379490	intergenic	intergenic	A	T	intergenic
379515	intergenic	intergenic	A	G	intergenic
379519	intergenic	intergenic	G	A	intergenic
383093	intergenic	intergenic	T	C	intergenic
383094	intergenic	intergenic	C	A	intergenic
383095	intergenic	intergenic	G	A	intergenic
383098	intergenic	intergenic	A	C	intergenic
383100	intergenic	intergenic	C	T	intergenic
383101	intergenic	intergenic	G	T	intergenic
383121	intergenic	intergenic	G	T	intergenic
383125	intergenic	intergenic	G	A	intergenic
383126	intergenic	intergenic	A	C	intergenic
383289	intergenic	intergenic	A	G	intergenic
383298	intergenic	intergenic	G	T	intergenic
383335	intergenic	intergenic	C	T	intergenic
383344	intergenic	intergenic	T	C	intergenic
383358	intergenic	intergenic	G	T	intergenic
383361	intergenic	intergenic	A	G	intergenic
383373	intergenic	intergenic	G	T	intergenic
383382	intergenic	intergenic	G	A	intergenic
383385	intergenic	intergenic	T	C	intergenic
383395	intergenic	intergenic	C	T	intergenic

383401	intergenic	intergenic	T	C	intergenic
383416	intergenic	intergenic	C	T	intergenic
383423	intergenic	intergenic	T	C	intergenic
383430	intergenic	intergenic	G	T	intergenic
383431	intergenic	intergenic	C	A	intergenic
383432	intergenic	intergenic	A	C	intergenic
383442	intergenic	intergenic	A	G	intergenic
383449	intergenic	intergenic	A	G	intergenic
383458	intergenic	intergenic	C	T	intergenic
383460	intergenic	intergenic	G	A	intergenic
383463	intergenic	intergenic	G	T	intergenic
383471	intergenic	intergenic	G	A	intergenic
383474	intergenic	intergenic	A	G	intergenic
383573	NTHI477_RS01895	31	C	T	nonsynonymous
383584	NTHI477_RS01895	42	C	T	synonymous
383659	NTHI477_RS01895	117	A	T	synonymous
383674	NTHI477_RS01895	132	A	T	synonymous
383689	NTHI477_RS01895	147	A	G	synonymous
383690	NTHI477_RS01895	148	C	T	synonymous
383692	NTHI477_RS01895	150	G	A	synonymous
383722	NTHI477_RS01895	180	C	T	synonymous
383734	NTHI477_RS01895	192	C	T	synonymous
383755	NTHI477_RS01895	213	G	A	synonymous
383758	NTHI477_RS01895	216	T	G	synonymous
383794	NTHI477_RS01895	252	A	T	synonymous
383809	NTHI477_RS01895	267	C	G	synonymous
383830	NTHI477_RS01895	288	A	C	synonymous
383884	NTHI477_RS01895	342	T	C	synonymous
383887	NTHI477_RS01895	345	T	C	synonymous
383893	NTHI477_RS01895	351	G	A	synonymous
383896	NTHI477_RS01895	354	A	C	synonymous
383947	NTHI477_RS01895	405	G	A	synonymous
383953	NTHI477_RS01895	411	T	G	synonymous
384010	NTHI477_RS01895	468	C	T	synonymous
384025	NTHI477_RS01895	483	A	G	synonymous
384070	NTHI477_RS01895	528	G	T	synonymous
384109	NTHI477_RS01895	567	C	T	synonymous
384184	NTHI477_RS01895	642	G	A	synonymous
384187	NTHI477_RS01895	645	C	G	synonymous
384190	NTHI477_RS01895	648	A	T	synonymous
384196	NTHI477_RS01895	654	T	C	synonymous
384199	NTHI477_RS01895	657	A	G	synonymous
384202	NTHI477_RS01895	660	C	G	synonymous
384202	NTHI477_RS01895	660	C	T	synonymous
384208	NTHI477_RS01895	666	A	G	synonymous

384219	NTHI477_RS01895	677	G	A	nonsynonymous
384238	NTHI477_RS01895	696	A	G	synonymous
384265	NTHI477_RS01895	723	T	G	synonymous
384280	NTHI477_RS01895	738	T	A	synonymous
384280	NTHI477_RS01895	738	T	C	synonymous
384286	NTHI477_RS01895	744	C	A	synonymous
384286	NTHI477_RS01895	744	C	G	synonymous
384313	NTHI477_RS01895	771	G	A	synonymous
384373	NTHI477_RS01895	831	T	C	synonymous
384379	NTHI477_RS01895	837	A	G	synonymous
384406	NTHI477_RS01895	864	C	T	synonymous
384433	NTHI477_RS01895	891	C	T	synonymous
384445	NTHI477_RS01895	903	C	T	synonymous
384454	NTHI477_RS01895	912	A	G	synonymous
384475	NTHI477_RS01895	933	A	T	synonymous
384478	NTHI477_RS01895	936	G	A	synonymous
384547	NTHI477_RS01895	1005	G	A	synonymous
384592	NTHI477_RS01895	1050	C	T	synonymous
384599	NTHI477_RS01895	1057	T	G	nonsynonymous
384601	NTHI477_RS01895	1059	C	G	synonymous
384604	NTHI477_RS01895	1062	C	T	synonymous
384625	NTHI477_RS01895	1083	G	C	synonymous
384628	NTHI477_RS01895	1086	G	T	synonymous
384649	NTHI477_RS01895	1107	G	A	synonymous
384661	NTHI477_RS01895	1119	A	G	synonymous
384676	NTHI477_RS01895	1134	C	T	synonymous
384700	NTHI477_RS01895	1158	T	A	synonymous
384727	NTHI477_RS01895	1185	A	G	synonymous
384730	NTHI477_RS01895	1188	T	C	synonymous
384736	NTHI477_RS01895	1194	C	T	synonymous
384757	NTHI477_RS01895	1215	G	A	synonymous
384767	NTHI477_RS01895	1225	G	T	nonsynonymous
384781	NTHI477_RS01895	1239	T	C	synonymous
384790	NTHI477_RS01895	1248	A	G	synonymous
384813	NTHI477_RS01895	1271	T	G	nonsynonymous
384826	NTHI477_RS01895	1284	C	T	synonymous
384868	NTHI477_RS01895	1326	T	A	synonymous
384868	NTHI477_RS01895	1326	T	C	synonymous
384874	NTHI477_RS01895	1332	G	A	synonymous
384892	NTHI477_RS01895	1350	G	A	synonymous
384901	NTHI477_RS01895	1359	C	T	synonymous
384919	NTHI477_RS01895	1377	C	T	synonymous
384925	NTHI477_RS01895	1383	C	T	synonymous
384934	NTHI477_RS01895	1392	G	T	synonymous
384943	NTHI477_RS01895	1401	G	C	synonymous

384956	NTHI477_RS01895	1414	A	G	nonsynonymous
384976	NTHI477_RS01895	1434	A	G	synonymous
384991	NTHI477_RS01895	1449	G	A	synonymous
385024	NTHI477_RS01895	1482	T	C	synonymous
385036	NTHI477_RS01895	1494	G	A	synonymous
385051	NTHI477_RS01895	1509	A	G	synonymous
385060	NTHI477_RS01895	1518	G	A	synonymous
385075	NTHI477_RS01895	1533	C	T	synonymous
385282	NTHI477_RS01895	1740	G	A	synonymous
385300	NTHI477_RS01895	1758	C	G	synonymous
385312	NTHI477_RS01895	1770	C	T	synonymous
385357	NTHI477_RS01895	1815	A	G	synonymous
385363	NTHI477_RS01895	1821	A	G	synonymous
385372	NTHI477_RS01895	1830	T	C	synonymous
385381	NTHI477_RS01895	1839	T	G	synonymous
385471	NTHI477_RS01895	1929	A	G	synonymous
385598	NTHI477_RS01895	2056	G	A	nonsynonymous
385654	NTHI477_RS01895	2112	T	A	synonymous
385663	NTHI477_RS01895	2121	C	A	synonymous
385663	NTHI477_RS01895	2121	C	T	synonymous
385762	NTHI477_RS01895	2220	T	A	nonsynonymous
385774	NTHI477_RS01895	2232	C	T	synonymous
385804	NTHI477_RS01895	2262	C	T	synonymous
385822	NTHI477_RS01895	2280	C	T	synonymous
385843	NTHI477_RS01895	2301	G	T	synonymous
385845	NTHI477_RS01895	2303	A	C	nonsynonymous
385855	NTHI477_RS01895	2313	A	C	synonymous
385906	NTHI477_RS01895	2364	T	G	synonymous
385966	NTHI477_RS01895	2424	C	A	synonymous
385972	NTHI477_RS01895	2430	T	C	synonymous
386011	NTHI477_RS01895	2469	C	T	synonymous
386026	NTHI477_RS01895	2484	G	T	synonymous
386083	NTHI477_RS01895	2541	T	C	synonymous
386086	NTHI477_RS01895	2544	C	T	synonymous
386098	NTHI477_RS01895	2556	T	C	synonymous
386101	NTHI477_RS01895	2559	C	A	synonymous
386128	NTHI477_RS01895	2586	A	G	synonymous
386143	NTHI477_RS01895	2601	A	G	synonymous
386152	NTHI477_RS01895	2610	A	T	nonsynonymous
386170	NTHI477_RS01895	2628	C	T	synonymous
386172	NTHI477_RS01895	2630	G	A	nonsynonymous
386177	NTHI477_RS01895	2635	G	A	nonsynonymous
386187	intergenic	intergenic	T	C	intergenic
386188	intergenic	intergenic	G	T	intergenic
386189	intergenic	intergenic	G	A	intergenic

386196	intergenic	intergenic	A	G	intergenic
386249	NTHI477_RS01900	1068	T	C	synonymous
386263	NTHI477_RS01900	1054	G	T	synonymous
386315	NTHI477_RS01900	1002	A	G	synonymous
386324	NTHI477_RS01900	993	G	A	synonymous
386348	NTHI477_RS01900	969	T	G	synonymous
386357	NTHI477_RS01900	960	C	T	synonymous
386389	NTHI477_RS01900	928	T	C	nonsynonymous
386390	NTHI477_RS01900	927	G	T	synonymous
386393	NTHI477_RS01900	924	T	C	synonymous
386417	NTHI477_RS01900	900	A	T	synonymous
386426	NTHI477_RS01900	891	C	T	synonymous
386444	NTHI477_RS01900	873	A	G	synonymous
386450	NTHI477_RS01900	867	T	C	synonymous
386456	NTHI477_RS01900	861	C	T	synonymous
386462	NTHI477_RS01900	855	G	A	synonymous
386468	NTHI477_RS01900	849	C	G	nonsynonymous
386483	NTHI477_RS01900	834	T	G	synonymous
386546	NTHI477_RS01900	771	G	A	synonymous
386561	NTHI477_RS01900	756	G	A	synonymous
386570	NTHI477_RS01900	747	A	G	synonymous
386579	NTHI477_RS01900	738	C	A	synonymous
386603	NTHI477_RS01900	714	G	A	synonymous
386621	NTHI477_RS01900	696	A	G	synonymous
386653	NTHI477_RS01900	664	C	T	nonsynonymous
386675	NTHI477_RS01900	642	T	C	synonymous
386684	NTHI477_RS01900	633	C	T	synonymous
386714	NTHI477_RS01900	603	C	A	synonymous
386720	NTHI477_RS01900	597	A	G	synonymous
386723	NTHI477_RS01900	594	A	G	synonymous
386732	NTHI477_RS01900	585	G	A	synonymous
386735	NTHI477_RS01900	582	G	A	synonymous
386744	NTHI477_RS01900	573	G	A	synonymous
386747	NTHI477_RS01900	570	A	G	synonymous
386756	NTHI477_RS01900	561	T	G	synonymous
386771	NTHI477_RS01900	546	A	C	synonymous
386771	NTHI477_RS01900	546	A	T	synonymous
386774	NTHI477_RS01900	543	A	G	synonymous
386777	NTHI477_RS01900	540	G	A	synonymous
386783	NTHI477_RS01900	534	G	A	synonymous
386816	NTHI477_RS01900	501	G	A	synonymous
386825	NTHI477_RS01900	492	C	T	synonymous
386879	NTHI477_RS01900	438	A	G	synonymous
386903	NTHI477_RS01900	414	G	C	synonymous
386920	NTHI477_RS01900	397	T	C	nonsynonymous

386921	NTHI477_RS01900	396	A	G	synonymous
386930	NTHI477_RS01900	387	T	A	nonsynonymous
386954	NTHI477_RS01900	363	A	G	synonymous
386972	NTHI477_RS01900	345	A	G	synonymous
386987	NTHI477_RS01900	330	G	A	synonymous
386990	NTHI477_RS01900	327	G	A	synonymous
387044	NTHI477_RS01900	273	A	G	synonymous
387047	NTHI477_RS01900	270	C	A	synonymous
387098	NTHI477_RS01900	219	T	C	synonymous
387128	NTHI477_RS01900	189	G	A	synonymous
387133	NTHI477_RS01900	184	C	T	nonsynonymous
387134	NTHI477_RS01900	183	T	G	synonymous
387164	NTHI477_RS01900	153	G	A	synonymous
387176	NTHI477_RS01900	141	A	G	synonymous
387203	NTHI477_RS01900	114	T	C	synonymous
387206	NTHI477_RS01900	111	G	A	synonymous
387207	NTHI477_RS01900	110	G	T	nonsynonymous
387215	NTHI477_RS01900	102	T	C	synonymous
387247	NTHI477_RS01900	70	G	A	nonsynonymous
387282	NTHI477_RS01900	35	T	A	nonsynonymous
387424	intergenic	intergenic	G	A	intergenic
387503	NTHI477_RS01905	30	A	C	nonsynonymous
387506	NTHI477_RS01905	33	T	C	synonymous
387520	NTHI477_RS01905	47	T	C	nonsynonymous
387531	NTHI477_RS01905	58	G	A	nonsynonymous
387537	NTHI477_RS01905	64	C	T	nonsynonymous
387590	NTHI477_RS01905	117	C	A	synonymous
387603	NTHI477_RS01905	130	C	T	nonsynonymous
387619	NTHI477_RS01905	146	C	T	nonsynonymous
387647	NTHI477_RS01905	174	C	T	synonymous
387649	NTHI477_RS01905	176	G	A	nonsynonymous
387659	NTHI477_RS01905	186	G	A	synonymous
387682	NTHI477_RS01905	209	T	C	nonsynonymous
387776	NTHI477_RS01905	303	T	C	synonymous
387839	NTHI477_RS01905	366	C	T	synonymous
387887	NTHI477_RS01905	414	A	T	synonymous
387893	NTHI477_RS01905	420	C	T	synonymous
387897	NTHI477_RS01905	424	A	G	nonsynonymous
387899	NTHI477_RS01905	426	C	T	synonymous
387902	NTHI477_RS01905	429	C	T	synonymous
387903	NTHI477_RS01905	430	T	G	nonsynonymous
387918	NTHI477_RS01905	445	C	A	nonsynonymous
387924	NTHI477_RS01905	451	A	C	nonsynonymous
387927	NTHI477_RS01905	454	A	G	nonsynonymous
387944	NTHI477_RS01905	471	T	C	synonymous

387968	NTHI477_RS01905	495	G	A	synonymous
387968	NTHI477_RS01905	495	G	T	synonymous
387981	NTHI477_RS01905	508	C	A	nonsynonymous
388001	NTHI477_RS01905	528	C	G	synonymous
388004	NTHI477_RS01905	531	T	C	synonymous
388007	NTHI477_RS01905	534	C	T	synonymous
388013	NTHI477_RS01905	540	G	A	synonymous
388016	NTHI477_RS01905	543	A	C	synonymous
388022	NTHI477_RS01905	549	G	A	synonymous
388025	NTHI477_RS01905	552	A	T	synonymous
388035	NTHI477_RS01905	562	T	C	nonsynonymous
388037	NTHI477_RS01905	564	T	A	nonsynonymous
388040	NTHI477_RS01905	567	C	T	synonymous
388043	NTHI477_RS01905	570	A	T	synonymous
388045	NTHI477_RS01905	572	A	G	nonsynonymous
388082	NTHI477_RS01905	609	T	G	nonsynonymous
388085	NTHI477_RS01905	612	C	T	synonymous
388161	intergenic	intergenic	T	A	intergenic
388162	intergenic	intergenic	T	C	intergenic
388176	intergenic	intergenic	C	A	intergenic
388179	intergenic	intergenic	C	A	intergenic
388187	intergenic	intergenic	C	G	intergenic
388256	intergenic	intergenic	A	C	intergenic
388308	intergenic	intergenic	A	G	intergenic
388339	intergenic	intergenic	C	T	intergenic
388345	intergenic	intergenic	T	C	intergenic
388402	intergenic	intergenic	G	A	intergenic
388427	intergenic	intergenic	C	T	intergenic
388431	intergenic	intergenic	G	A	intergenic
388435	intergenic	intergenic	G	A	intergenic
388438	intergenic	intergenic	C	A	intergenic
388466	NTHI477_RS01910	1281	A	G	synonymous
388469	NTHI477_RS01910	1278	A	T	synonymous
388499	NTHI477_RS01910	1248	C	A	synonymous
388500	NTHI477_RS01910	1247	A	G	nonsynonymous
388508	NTHI477_RS01910	1239	T	C	synonymous
388523	NTHI477_RS01910	1224	G	A	synonymous
388538	NTHI477_RS01910	1209	A	C	synonymous
388547	NTHI477_RS01910	1200	A	G	synonymous
388585	NTHI477_RS01910	1162	G	T	nonsynonymous
388586	NTHI477_RS01910	1161	A	G	synonymous
388594	NTHI477_RS01910	1153	A	G	synonymous
388598	NTHI477_RS01910	1149	C	A	nonsynonymous
388601	NTHI477_RS01910	1146	T	A	synonymous
388616	NTHI477_RS01910	1131	T	C	synonymous

388631	NTHI477_RS01910	1116	G	A	synonymous
388634	NTHI477_RS01910	1113	G	T	synonymous
388640	NTHI477_RS01910	1107	A	G	synonymous
388670	NTHI477_RS01910	1077	T	C	synonymous
388680	NTHI477_RS01910	1067	T	G	nonsynonymous
388691	NTHI477_RS01910	1056	A	G	synonymous
388721	NTHI477_RS01910	1026	C	T	synonymous
388736	NTHI477_RS01910	1011	A	G	synonymous
388742	NTHI477_RS01910	1005	G	A	synonymous
388742	NTHI477_RS01910	1005	G	T	synonymous
388745	NTHI477_RS01910	1002	G	A	synonymous
388746	NTHI477_RS01910	1001	C	T	nonsynonymous
388748	NTHI477_RS01910	999	A	G	synonymous
388749	NTHI477_RS01910	998	C	T	nonsynonymous
388750	NTHI477_RS01910	997	G	A	nonsynonymous
388787	NTHI477_RS01910	960	A	G	synonymous
388790	NTHI477_RS01910	957	G	A	synonymous
388805	NTHI477_RS01910	942	T	C	synonymous
388817	NTHI477_RS01910	930	A	G	synonymous
388821	NTHI477_RS01910	926	G	A	nonsynonymous
388840	NTHI477_RS01910	907	G	A	nonsynonymous
388865	NTHI477_RS01910	882	T	C	synonymous
388879	NTHI477_RS01910	868	T	C	nonsynonymous
388886	NTHI477_RS01910	861	A	T	synonymous
388962	NTHI477_RS01910	785	T	C	nonsynonymous
388970	NTHI477_RS01910	777	G	A	synonymous
388976	NTHI477_RS01910	771	C	A	synonymous
388997	NTHI477_RS01910	750	C	T	synonymous
389065	NTHI477_RS01910	682	C	T	nonsynonymous
389078	NTHI477_RS01910	669	T	C	synonymous
389079	NTHI477_RS01910	668	A	G	nonsynonymous
389123	NTHI477_RS01910	624	G	A	synonymous
389129	NTHI477_RS01910	618	A	C	nonsynonymous
389144	NTHI477_RS01910	603	C	T	synonymous
389146	NTHI477_RS01910	601	G	A	nonsynonymous
389159	NTHI477_RS01910	588	A	G	synonymous
389185	NTHI477_RS01910	562	G	A	nonsynonymous
389213	NTHI477_RS01910	534	G	A	synonymous
389214	NTHI477_RS01910	533	G	T	nonsynonymous
389214	NTHI477_RS01910	533	G	A	nonsynonymous
389216	NTHI477_RS01910	531	C	T	synonymous
389267	NTHI477_RS01910	480	C	T	synonymous
389270	NTHI477_RS01910	477	A	C	synonymous
389294	NTHI477_RS01910	453	G	A	synonymous
389297	NTHI477_RS01910	450	C	T	synonymous

389309	NTHI477_RS01910	438	G	A	synonymous
389312	NTHI477_RS01910	435	G	A	synonymous
389318	NTHI477_RS01910	429	A	C	synonymous
389348	NTHI477_RS01910	399	T	C	synonymous
389369	NTHI477_RS01910	378	T	G	synonymous
389414	NTHI477_RS01910	333	G	A	synonymous
389431	NTHI477_RS01910	316	C	T	nonsynonymous
389441	NTHI477_RS01910	306	G	A	synonymous
389441	NTHI477_RS01910	306	G	T	synonymous
389444	NTHI477_RS01910	303	A	G	synonymous
389525	NTHI477_RS01910	222	T	C	synonymous
389537	NTHI477_RS01910	210	G	A	synonymous
389642	NTHI477_RS01910	105	T	C	synonymous
389645	NTHI477_RS01910	102	C	T	synonymous
389653	NTHI477_RS01910	94	G	T	nonsynonymous
389669	NTHI477_RS01910	78	G	A	synonymous
389775	NTHI477_RS01915	864	T	C	synonymous
389778	NTHI477_RS01915	861	T	C	synonymous
389780	NTHI477_RS01915	859	C	T	nonsynonymous
389790	NTHI477_RS01915	849	A	T	synonymous
389850	NTHI477_RS01915	789	C	T	synonymous
389868	NTHI477_RS01915	771	C	A	synonymous
389882	NTHI477_RS01915	757	A	G	synonymous
389886	NTHI477_RS01915	753	C	T	synonymous
389892	NTHI477_RS01915	747	G	A	synonymous
389910	NTHI477_RS01915	729	T	C	synonymous
389922	NTHI477_RS01915	717	T	C	synonymous
389925	NTHI477_RS01915	714	G	A	synonymous
389940	NTHI477_RS01915	699	A	G	synonymous
389940	NTHI477_RS01915	699	A	C	synonymous
389943	NTHI477_RS01915	696	A	T	synonymous
389946	NTHI477_RS01915	693	G	C	synonymous
389949	NTHI477_RS01915	690	A	T	synonymous
389955	NTHI477_RS01915	684	C	A	synonymous
390021	NTHI477_RS01915	618	A	G	synonymous
390030	NTHI477_RS01915	609	T	C	synonymous
390045	NTHI477_RS01915	594	T	C	synonymous
390054	NTHI477_RS01915	585	C	T	synonymous
390063	NTHI477_RS01915	576	T	C	synonymous
390078	NTHI477_RS01915	561	G	A	synonymous
390084	NTHI477_RS01915	555	C	T	synonymous
390087	NTHI477_RS01915	552	C	G	synonymous
390087	NTHI477_RS01915	552	C	A	synonymous
390090	NTHI477_RS01915	549	A	G	synonymous
390093	NTHI477_RS01915	546	A	G	synonymous

390117	NTHI477_RS01915	522	A	G	synonymous
390120	NTHI477_RS01915	519	C	T	synonymous
390123	NTHI477_RS01915	516	G	T	synonymous
390129	NTHI477_RS01915	510	T	C	synonymous
390138	NTHI477_RS01915	501	C	T	synonymous
390138	NTHI477_RS01915	501	C	A	synonymous
390141	NTHI477_RS01915	498	A	C	synonymous
390144	NTHI477_RS01915	495	G	A	synonymous
390150	NTHI477_RS01915	489	T	C	synonymous
390162	NTHI477_RS01915	477	T	C	synonymous
390163	NTHI477_RS01915	476	G	A	nonsynonymous
390168	NTHI477_RS01915	471	G	T	synonymous
390168	NTHI477_RS01915	471	G	C	synonymous
390177	NTHI477_RS01915	462	T	C	nonsynonymous
390183	NTHI477_RS01915	456	C	T	nonsynonymous
390186	NTHI477_RS01915	453	T	C	synonymous
390200	NTHI477_RS01915	439	C	A	nonsynonymous
390216	NTHI477_RS01915	423	C	A	synonymous
390258	NTHI477_RS01915	381	T	C	synonymous
390273	NTHI477_RS01915	366	A	G	synonymous
390284	NTHI477_RS01915	355	T	C	nonsynonymous
390291	NTHI477_RS01915	348	T	C	synonymous
390294	NTHI477_RS01915	345	A	G	synonymous
390297	NTHI477_RS01915	342	T	A	synonymous
390312	NTHI477_RS01915	327	C	T	synonymous
390315	NTHI477_RS01915	324	A	G	synonymous
390327	NTHI477_RS01915	312	T	C	synonymous
390339	NTHI477_RS01915	300	C	T	synonymous
390346	NTHI477_RS01915	293	C	T	nonsynonymous
390378	NTHI477_RS01915	261	A	G	synonymous
390422	NTHI477_RS01915	217	G	C	nonsynonymous
390444	NTHI477_RS01915	195	T	A	nonsynonymous
390462	NTHI477_RS01915	177	T	A	synonymous
390465	NTHI477_RS01915	174	G	A	synonymous
390486	NTHI477_RS01915	153	G	A	synonymous
390492	NTHI477_RS01915	147	A	C	synonymous
390504	NTHI477_RS01915	135	A	C	synonymous
390552	NTHI477_RS01915	87	G	A	synonymous
390564	NTHI477_RS01915	75	T	C	synonymous
390573	NTHI477_RS01915	66	C	T	synonymous
390609	NTHI477_RS01915	30	T	C	synonymous
390669	intergenic	intergenic	C	T	intergenic
390711	intergenic	intergenic	G	A	intergenic
390720	intergenic	intergenic	A	G	intergenic
390741	intergenic	intergenic	T	A	intergenic

390747	intergenic	intergenic	G	A	intergenic
390780	intergenic	intergenic	C	T	intergenic
390796	intergenic	intergenic	T	C	intergenic
390807	intergenic	intergenic	G	A	intergenic
390816	intergenic	intergenic	C	T	intergenic
390888	NTHI477_RS01920	56	A	G	nonsynonymous
390926	NTHI477_RS01920	94	A	G	nonsynonymous
390952	NTHI477_RS01920	120	A	G	synonymous
390967	NTHI477_RS01920	135	T	A	synonymous
391011	NTHI477_RS01920	179	G	T	nonsynonymous
391011	NTHI477_RS01920	179	G	A	nonsynonymous
391030	NTHI477_RS01920	198	C	T	synonymous
391060	NTHI477_RS01920	228	T	C	synonymous
391066	NTHI477_RS01920	234	C	T	synonymous
391081	NTHI477_RS01920	249	G	C	synonymous
391099	NTHI477_RS01920	267	C	T	synonymous
391105	NTHI477_RS01920	273	G	A	synonymous
391111	NTHI477_RS01920	279	G	A	synonymous
391120	NTHI477_RS01920	288	T	C	synonymous
391138	NTHI477_RS01920	306	T	C	synonymous
391141	NTHI477_RS01920	309	C	T	synonymous
391195	NTHI477_RS01920	363	G	A	synonymous
391203	NTHI477_RS01920	371	A	C	nonsynonymous
391228	NTHI477_RS01920	396	T	G	synonymous
391264	NTHI477_RS01920	432	G	A	synonymous
391279	NTHI477_RS01920	447	G	A	synonymous
391285	NTHI477_RS01920	453	T	A	synonymous
391288	NTHI477_RS01920	456	A	G	synonymous
391298	NTHI477_RS01920	466	T	C	nonsynonymous
391357	NTHI477_RS01920	525	T	C	synonymous
391369	NTHI477_RS01920	537	T	C	synonymous
391409	NTHI477_RS01920	577	G	A	nonsynonymous
391438	NTHI477_RS01920	606	C	T	synonymous
391444	NTHI477_RS01920	612	T	C	synonymous
391447	NTHI477_RS01920	615	C	T	synonymous
391450	NTHI477_RS01920	618	T	C	synonymous
391465	NTHI477_RS01920	633	C	T	synonymous
391483	NTHI477_RS01920	651	C	A	synonymous
391501	NTHI477_RS01920	669	G	A	synonymous
391507	NTHI477_RS01920	675	T	A	synonymous
391510	NTHI477_RS01920	678	G	A	synonymous
391513	NTHI477_RS01920	681	G	A	synonymous
391516	NTHI477_RS01920	684	A	C	synonymous
391519	NTHI477_RS01920	687	T	C	synonymous
391520	NTHI477_RS01920	688	T	C	synonymous

391540	NTHI477_RS01920	708	C	A	synonymous
391561	NTHI477_RS01920	729	T	G	synonymous
391570	NTHI477_RS01920	738	T	A	synonymous
391624	NTHI477_RS01920	792	G	A	synonymous
391678	NTHI477_RS01920	846	T	G	synonymous
391687	NTHI477_RS01920	855	T	C	synonymous
391717	NTHI477_RS01920	885	G	A	synonymous
391723	NTHI477_RS01920	891	G	A	synonymous
391732	NTHI477_RS01920	900	C	T	synonymous
391741	NTHI477_RS01920	909	G	A	synonymous
391744	NTHI477_RS01920	912	C	T	synonymous
391756	NTHI477_RS01920	924	G	A	synonymous
391780	NTHI477_RS01920	948	T	A	synonymous
391834	NTHI477_RS01920	1002	T	C	synonymous
391843	NTHI477_RS01920	1011	G	T	nonsynonymous
391855	NTHI477_RS01920	1023	T	C	synonymous
391858	NTHI477_RS01920	1026	C	A	synonymous
391862	NTHI477_RS01920	1030	A	T	nonsynonymous
391879	NTHI477_RS01920	1047	G	A	synonymous
391900	NTHI477_RS01920	1068	A	T	synonymous
391903	NTHI477_RS01920	1071	C	T	synonymous
391918	NTHI477_RS01920	1086	T	C	synonymous
391925	NTHI477_RS01920	1093	G	A	nonsynonymous
391946	NTHI477_RS01920	1114	G	A	nonsynonymous
391963	NTHI477_RS01920	1131	T	A	synonymous
391972	NTHI477_RS01920	1140	A	G	synonymous
391975	NTHI477_RS01920	1143	A	G	synonymous
391977	NTHI477_RS01920	1145	T	C	nonsynonymous
391978	NTHI477_RS01920	1146	C	T	synonymous
391990	NTHI477_RS01920	1158	C	T	synonymous
391990	NTHI477_RS01920	1158	C	A	synonymous
392012	NTHI477_RS01920	1180	A	G	nonsynonymous
392014	NTHI477_RS01920	1182	A	T	synonymous
392029	NTHI477_RS01920	1197	C	A	synonymous
392033	NTHI477_RS01920	1201	G	A	nonsynonymous
392054	NTHI477_RS01920	1222	A	G	nonsynonymous
392059	NTHI477_RS01920	1227	A	G	synonymous
392084	NTHI477_RS01920	1252	G	T	nonsynonymous
392113	NTHI477_RS01920	1281	T	C	synonymous
392124	NTHI477_RS01920	1292	A	G	nonsynonymous
392153	NTHI477_RS01920	1321	G	A	nonsynonymous
392161	NTHI477_RS01920	1329	G	A	synonymous
392164	NTHI477_RS01920	1332	A	G	synonymous
392171	NTHI477_RS01920	1339	G	A	nonsynonymous
392195	NTHI477_RS01920	1363	A	G	nonsynonymous

392228	intergenic	intergenic	G	A	intergenic
392269	intergenic	intergenic	A	G	intergenic
392334	NTHI477_RS01925	3384	G	A	synonymous
392339	NTHI477_RS01925	3379	G	A	synonymous
392349	NTHI477_RS01925	3369	A	G	synonymous
392361	NTHI477_RS01925	3357	T	G	nonsynonymous
392367	NTHI477_RS01925	3351	A	G	synonymous
392421	NTHI477_RS01925	3297	T	G	synonymous
392460	NTHI477_RS01925	3258	G	A	synonymous
392466	NTHI477_RS01925	3252	T	C	synonymous
392490	NTHI477_RS01925	3228	T	G	nonsynonymous
392493	NTHI477_RS01925	3225	T	C	synonymous
392505	NTHI477_RS01925	3213	C	T	synonymous
392514	NTHI477_RS01925	3204	C	T	synonymous
392522	NTHI477_RS01925	3196	C	T	nonsynonymous
392535	NTHI477_RS01925	3183	G	A	synonymous
392580	NTHI477_RS01925	3138	G	A	synonymous
392625	NTHI477_RS01925	3093	G	A	synonymous
392655	NTHI477_RS01925	3063	G	A	synonymous
392658	NTHI477_RS01925	3060	T	A	synonymous
392663	NTHI477_RS01925	3055	G	A	synonymous
392673	NTHI477_RS01925	3045	A	T	synonymous
392678	NTHI477_RS01925	3040	G	A	synonymous
392688	NTHI477_RS01925	3030	A	C	synonymous
392691	NTHI477_RS01925	3027	G	A	synonymous
392723	NTHI477_RS01925	2995	G	A	synonymous
392733	NTHI477_RS01925	2985	G	T	synonymous
392742	NTHI477_RS01925	2976	T	A	synonymous
392757	NTHI477_RS01925	2961	C	T	synonymous
392769	NTHI477_RS01925	2949	C	T	synonymous
392771	NTHI477_RS01925	2947	C	A	nonsynonymous
392787	NTHI477_RS01925	2931	A	G	synonymous
392793	NTHI477_RS01925	2925	C	T	synonymous
392796	NTHI477_RS01925	2922	A	G	synonymous
392811	NTHI477_RS01925	2907	G	A	synonymous
392820	NTHI477_RS01925	2898	G	A	synonymous
392829	NTHI477_RS01925	2889	A	G	synonymous
392832	NTHI477_RS01925	2886	T	A	synonymous
392835	NTHI477_RS01925	2883	G	A	synonymous
392838	NTHI477_RS01925	2880	T	C	synonymous
392847	NTHI477_RS01925	2871	T	A	synonymous
392847	NTHI477_RS01925	2871	T	G	synonymous
392850	NTHI477_RS01925	2868	A	G	synonymous
392889	NTHI477_RS01925	2829	A	T	synonymous
392892	NTHI477_RS01925	2826	C	A	synonymous

392898	NTHI477_RS01925	2820	G	A	synonymous
392913	NTHI477_RS01925	2805	T	C	synonymous
392916	NTHI477_RS01925	2802	C	T	synonymous
392922	NTHI477_RS01925	2796	T	A	synonymous
392925	NTHI477_RS01925	2793	A	G	synonymous
392952	NTHI477_RS01925	2766	T	C	synonymous
392955	NTHI477_RS01925	2763	T	A	synonymous
392958	NTHI477_RS01925	2760	C	T	synonymous
392961	NTHI477_RS01925	2757	T	G	synonymous
393000	NTHI477_RS01925	2718	C	T	synonymous
393003	NTHI477_RS01925	2715	T	A	synonymous
393003	NTHI477_RS01925	2715	T	G	synonymous
393024	NTHI477_RS01925	2694	A	C	synonymous
393026	NTHI477_RS01925	2692	G	A	nonsynonymous
393036	NTHI477_RS01925	2682	G	A	synonymous
393156	NTHI477_RS01925	2562	A	G	synonymous
393159	NTHI477_RS01925	2559	C	T	synonymous
393165	NTHI477_RS01925	2553	T	C	synonymous
393174	NTHI477_RS01925	2544	G	T	synonymous
393176	NTHI477_RS01925	2542	G	T	nonsynonymous
393186	NTHI477_RS01925	2532	A	G	synonymous
393189	NTHI477_RS01925	2529	C	G	synonymous
393192	NTHI477_RS01925	2526	C	G	synonymous
393192	NTHI477_RS01925	2526	C	T	synonymous
393198	NTHI477_RS01925	2520	C	T	synonymous
393204	NTHI477_RS01925	2514	T	C	synonymous
393207	NTHI477_RS01925	2511	T	C	synonymous
393219	NTHI477_RS01925	2499	T	C	synonymous
393221	NTHI477_RS01925	2497	C	T	nonsynonymous
393225	NTHI477_RS01925	2493	G	C	synonymous
393249	NTHI477_RS01925	2469	A	G	synonymous
393264	NTHI477_RS01925	2454	A	G	synonymous
393270	NTHI477_RS01925	2448	G	A	synonymous
393282	NTHI477_RS01925	2436	T	C	synonymous
393285	NTHI477_RS01925	2433	G	A	synonymous
393288	NTHI477_RS01925	2430	A	T	synonymous
393291	NTHI477_RS01925	2427	C	G	nonsynonymous
393293	NTHI477_RS01925	2425	A	G	synonymous
393294	NTHI477_RS01925	2424	G	A	synonymous
393330	NTHI477_RS01925	2388	A	G	synonymous
393339	NTHI477_RS01925	2379	G	A	synonymous
393369	NTHI477_RS01925	2349	A	C	synonymous
393378	NTHI477_RS01925	2340	T	C	synonymous
393381	NTHI477_RS01925	2337	G	A	synonymous
393390	NTHI477_RS01925	2328	G	A	synonymous

393393	NTHI477_RS01925	2325	C	A	synonymous
393405	NTHI477_RS01925	2313	A	G	synonymous
393414	NTHI477_RS01925	2304	C	T	synonymous
393423	NTHI477_RS01925	2295	T	A	synonymous
393429	NTHI477_RS01925	2289	A	T	synonymous
393432	NTHI477_RS01925	2286	C	A	synonymous
393453	NTHI477_RS01925	2265	G	C	synonymous
393477	NTHI477_RS01925	2241	G	A	synonymous
393486	NTHI477_RS01925	2232	G	T	synonymous
393516	NTHI477_RS01925	2202	G	A	synonymous
393519	NTHI477_RS01925	2199	C	T	synonymous
393540	NTHI477_RS01925	2178	A	T	synonymous
393550	NTHI477_RS01925	2168	C	T	nonsynonymous
393558	NTHI477_RS01925	2160	G	T	synonymous
393591	NTHI477_RS01925	2127	C	T	synonymous
393593	NTHI477_RS01925	2125	G	A	synonymous
393606	NTHI477_RS01925	2112	G	T	synonymous
393672	NTHI477_RS01925	2046	T	G	synonymous
393675	NTHI477_RS01925	2043	G	A	synonymous
393684	NTHI477_RS01925	2034	C	T	synonymous
393687	NTHI477_RS01925	2031	G	A	synonymous
393734	NTHI477_RS01925	1984	A	G	synonymous
393749	NTHI477_RS01925	1969	G	A	synonymous
393762	NTHI477_RS01925	1956	T	C	synonymous
393831	NTHI477_RS01925	1887	G	A	synonymous
393852	NTHI477_RS01925	1866	T	C	synonymous
393855	NTHI477_RS01925	1863	T	A	synonymous
393906	NTHI477_RS01925	1812	A	G	synonymous
393920	NTHI477_RS01925	1798	G	A	nonsynonymous
393924	NTHI477_RS01925	1794	G	A	synonymous
393930	NTHI477_RS01925	1788	A	G	synonymous
393932	NTHI477_RS01925	1786	C	A	nonsynonymous
393939	NTHI477_RS01925	1779	T	C	synonymous
393966	NTHI477_RS01925	1752	G	A	synonymous
393978	NTHI477_RS01925	1740	T	C	synonymous
393996	NTHI477_RS01925	1722	A	G	synonymous
393999	NTHI477_RS01925	1719	A	G	synonymous
394007	NTHI477_RS01925	1711	G	A	synonymous
394026	NTHI477_RS01925	1692	A	T	synonymous
394041	NTHI477_RS01925	1677	T	A	synonymous
394060	NTHI477_RS01925	1658	G	A	nonsynonymous
394074	NTHI477_RS01925	1644	A	C	synonymous
394077	NTHI477_RS01925	1641	C	T	synonymous
394092	NTHI477_RS01925	1626	C	T	synonymous
394107	NTHI477_RS01925	1611	A	G	synonymous

394128	NTHI477_RS01925	1590	G	C	synonymous
394130	NTHI477_RS01925	1588	G	A	nonsynonymous
394143	NTHI477_RS01925	1575	G	C	synonymous
394161	NTHI477_RS01925	1557	T	C	synonymous
394182	NTHI477_RS01925	1536	T	C	synonymous
394194	NTHI477_RS01925	1524	T	G	synonymous
394206	NTHI477_RS01925	1512	G	A	synonymous
394221	NTHI477_RS01925	1497	A	G	synonymous
394223	NTHI477_RS01925	1495	C	T	nonsynonymous
394230	NTHI477_RS01925	1488	A	G	synonymous
394239	NTHI477_RS01925	1479	C	T	synonymous
394260	NTHI477_RS01925	1458	C	T	synonymous
394269	NTHI477_RS01925	1449	T	C	synonymous
394283	NTHI477_RS01925	1435	A	G	synonymous
394284	NTHI477_RS01925	1434	C	T	synonymous
394296	NTHI477_RS01925	1422	G	T	synonymous
394308	NTHI477_RS01925	1410	G	A	synonymous
394320	NTHI477_RS01925	1398	C	T	synonymous
394332	NTHI477_RS01925	1386	A	G	synonymous
394352	NTHI477_RS01925	1366	C	T	nonsynonymous
394353	NTHI477_RS01925	1365	A	G	synonymous
394356	NTHI477_RS01925	1362	T	C	synonymous
394359	NTHI477_RS01925	1359	G	A	synonymous
394365	NTHI477_RS01925	1353	T	C	synonymous
394397	NTHI477_RS01925	1321	A	G	nonsynonymous
394403	NTHI477_RS01925	1315	G	T	nonsynonymous
394410	NTHI477_RS01925	1308	A	T	synonymous
394456	NTHI477_RS01925	1262	T	A	nonsynonymous
394458	NTHI477_RS01925	1260	C	T	synonymous
394460	NTHI477_RS01925	1258	T	C	nonsynonymous
394462	NTHI477_RS01925	1256	G	A	nonsynonymous
394462	NTHI477_RS01925	1256	G	T	nonsynonymous
394463	NTHI477_RS01925	1255	C	T	nonsynonymous
394464	NTHI477_RS01925	1254	T	C	synonymous
394470	NTHI477_RS01925	1248	C	T	synonymous
394473	NTHI477_RS01925	1245	G	A	synonymous
394491	NTHI477_RS01925	1227	T	C	synonymous
394503	NTHI477_RS01925	1215	C	T	synonymous
394505	NTHI477_RS01925	1213	C	G	nonsynonymous
394512	NTHI477_RS01925	1206	C	T	synonymous
394524	NTHI477_RS01925	1194	C	A	synonymous
394551	NTHI477_RS01925	1167	C	A	synonymous
394559	NTHI477_RS01925	1159	G	A	synonymous
394562	NTHI477_RS01925	1156	C	A	nonsynonymous
394563	NTHI477_RS01925	1155	G	A	synonymous

394590	NTHI477_RS01925	1128	G	A	synonymous
394593	NTHI477_RS01925	1125	T	G	nonsynonymous
394595	NTHI477_RS01925	1123	A	G	synonymous
394599	NTHI477_RS01925	1119	C	T	synonymous
394629	NTHI477_RS01925	1089	A	G	synonymous
394632	NTHI477_RS01925	1086	T	C	synonymous
394638	NTHI477_RS01925	1080	T	A	synonymous
394643	NTHI477_RS01925	1075	A	G	synonymous
394695	NTHI477_RS01925	1023	T	C	synonymous
394707	NTHI477_RS01925	1011	A	G	synonymous
394710	NTHI477_RS01925	1008	T	C	synonymous
394734	NTHI477_RS01925	984	A	G	synonymous
394743	NTHI477_RS01925	975	A	T	synonymous
394749	NTHI477_RS01925	969	G	A	synonymous
394769	NTHI477_RS01925	949	A	G	nonsynonymous
394776	NTHI477_RS01925	942	C	G	synonymous
394785	NTHI477_RS01925	933	T	C	synonymous
394846	NTHI477_RS01925	872	G	A	nonsynonymous
394851	NTHI477_RS01925	867	G	A	synonymous
394854	NTHI477_RS01925	864	G	A	synonymous
394866	NTHI477_RS01925	852	T	C	synonymous
394881	NTHI477_RS01925	837	T	C	synonymous
394884	NTHI477_RS01925	834	T	C	synonymous
394899	NTHI477_RS01925	819	C	T	synonymous
394905	NTHI477_RS01925	813	C	T	synonymous
394916	NTHI477_RS01925	802	A	C	nonsynonymous
394926	NTHI477_RS01925	792	T	A	synonymous
394956	NTHI477_RS01925	762	G	T	synonymous
394956	NTHI477_RS01925	762	G	A	synonymous
394962	NTHI477_RS01925	756	C	T	synonymous
394995	NTHI477_RS01925	723	T	A	synonymous
394998	NTHI477_RS01925	720	A	G	synonymous
395001	NTHI477_RS01925	717	G	A	synonymous
395004	NTHI477_RS01925	714	C	T	synonymous
395007	NTHI477_RS01925	711	C	G	synonymous
395013	NTHI477_RS01925	705	G	A	synonymous
395028	NTHI477_RS01925	690	T	C	synonymous
395034	NTHI477_RS01925	684	G	A	synonymous
395052	NTHI477_RS01925	666	A	G	synonymous
395055	NTHI477_RS01925	663	A	G	synonymous
395058	NTHI477_RS01925	660	C	T	synonymous
395073	NTHI477_RS01925	645	C	A	synonymous
395076	NTHI477_RS01925	642	T	C	synonymous
395079	NTHI477_RS01925	639	T	C	synonymous
395085	NTHI477_RS01925	633	G	A	synonymous

395094	NTHI477_RS01925	624	G	T	nonsynonymous
395095	NTHI477_RS01925	623	T	C	nonsynonymous
395095	NTHI477_RS01925	623	T	G	nonsynonymous
395097	NTHI477_RS01925	621	G	A	synonymous
395112	NTHI477_RS01925	606	A	G	synonymous
395118	NTHI477_RS01925	600	C	G	synonymous
395121	NTHI477_RS01925	597	G	A	synonymous
395142	NTHI477_RS01925	576	G	A	synonymous
395169	NTHI477_RS01925	549	A	G	synonymous
395172	NTHI477_RS01925	546	A	G	synonymous
395181	NTHI477_RS01925	537	C	A	nonsynonymous
395183	NTHI477_RS01925	535	T	C	nonsynonymous
395196	NTHI477_RS01925	522	T	C	synonymous
395208	NTHI477_RS01925	510	C	T	synonymous
395214	NTHI477_RS01925	504	A	G	synonymous
395216	NTHI477_RS01925	502	C	A	nonsynonymous
395220	NTHI477_RS01925	498	A	T	synonymous
395226	NTHI477_RS01925	492	T	G	synonymous
395229	NTHI477_RS01925	489	G	A	synonymous
395235	NTHI477_RS01925	483	G	A	synonymous
395256	NTHI477_RS01925	462	C	T	synonymous
395259	NTHI477_RS01925	459	A	C	synonymous
395262	NTHI477_RS01925	456	A	T	synonymous
395271	NTHI477_RS01925	447	C	T	synonymous
395274	NTHI477_RS01925	444	G	T	synonymous
395274	NTHI477_RS01925	444	G	A	synonymous
395275	NTHI477_RS01925	443	A	G	nonsynonymous
395291	NTHI477_RS01925	427	G	A	nonsynonymous
395292	NTHI477_RS01925	426	T	C	nonsynonymous
395316	NTHI477_RS01925	402	G	A	synonymous
395330	NTHI477_RS01925	388	G	A	synonymous
395358	NTHI477_RS01925	360	C	G	synonymous
395388	NTHI477_RS01925	330	C	T	synonymous
395475	NTHI477_RS01925	243	G	A	synonymous
395505	NTHI477_RS01925	213	A	G	synonymous
395511	NTHI477_RS01925	207	A	G	synonymous
395515	NTHI477_RS01925	203	A	T	nonsynonymous
395523	NTHI477_RS01925	195	G	A	synonymous
395543	NTHI477_RS01925	175	T	C	nonsynonymous
395544	NTHI477_RS01925	174	C	A	synonymous
395556	NTHI477_RS01925	162	T	C	synonymous
395577	NTHI477_RS01925	141	T	A	synonymous
395580	NTHI477_RS01925	138	G	C	synonymous
395583	NTHI477_RS01925	135	T	C	synonymous
395589	NTHI477_RS01925	129	A	G	synonymous

395596	NTHI477_RS01925	122	T	C	nonsynonymous
395601	NTHI477_RS01925	117	A	G	synonymous
395612	NTHI477_RS01925	106	C	T	nonsynonymous
395612	NTHI477_RS01925	106	C	A	nonsynonymous
395622	NTHI477_RS01925	96	A	G	synonymous
395630	NTHI477_RS01925	88	G	A	synonymous
395640	NTHI477_RS01925	78	A	G	synonymous
395644	NTHI477_RS01925	74	T	G	nonsynonymous
395649	NTHI477_RS01925	69	G	T	synonymous
395667	NTHI477_RS01925	51	A	G	synonymous
395676	NTHI477_RS01925	42	A	G	synonymous
395683	NTHI477_RS01925	35	G	A	nonsynonymous
395685	NTHI477_RS01925	33	T	A	synonymous
395732	intergenic	intergenic	T	C	intergenic
395744	intergenic	intergenic	A	G	intergenic
395755	intergenic	intergenic	C	G	intergenic
395757	intergenic	intergenic	C	A	intergenic
395776	intergenic	intergenic	A	C	intergenic
395777	intergenic	intergenic	C	T	intergenic
395792	intergenic	intergenic	A	G	intergenic
395795	intergenic	intergenic	T	C	intergenic
395809	intergenic	intergenic	C	A	intergenic
395815	intergenic	intergenic	G	A	intergenic
395820	NTHI477_RS09435	174	T	C	synonymous
395826	NTHI477_RS09435	168	C	G	nonsynonymous
395834	NTHI477_RS09435	160	A	G	nonsynonymous
395840	NTHI477_RS09435	154	T	C	nonsynonymous
395843	NTHI477_RS09435	151	C	T	nonsynonymous
395845	NTHI477_RS09435	149	T	G	nonsynonymous
395856	NTHI477_RS09435	138	G	A	synonymous
395889	NTHI477_RS09435	105	G	A	synonymous
395892	NTHI477_RS09435	102	G	A	synonymous
395901	NTHI477_RS09435	93	A	C	synonymous
395910	NTHI477_RS09435	84	T	C	synonymous
395913	NTHI477_RS09435	81	A	T	synonymous
395913	NTHI477_RS09435	81	A	C	synonymous
395931	NTHI477_RS09435	63	G	A	synonymous
395946	NTHI477_RS09435	48	A	T	synonymous
395948	NTHI477_RS09435	46	G	A	nonsynonymous
395949	NTHI477_RS09435	45	T	A	synonymous
395951	NTHI477_RS09435	43	C	T	nonsynonymous
395981	NTHI477_RS09435	13	T	C	nonsynonymous
395985	NTHI477_RS09435	9	C	T	synonymous
396006	NTHI477_RS01930	1955	G	T	nonsynonymous
396006	NTHI477_RS01930	1955	G	A	nonsynonymous

396062	NTHI477_RS01930	1899	G	A	synonymous
396067	NTHI477_RS01930	1894	G	A	nonsynonymous
396069	NTHI477_RS01930	1892	T	G	nonsynonymous
396074	NTHI477_RS01930	1887	T	G	nonsynonymous
396104	NTHI477_RS01930	1857	G	A	synonymous
396104	NTHI477_RS01930	1857	G	C	nonsynonymous
396114	NTHI477_RS01930	1847	C	T	nonsynonymous
396116	NTHI477_RS01930	1845	C	T	synonymous
396137	NTHI477_RS01930	1824	T	C	nonsynonymous
396140	NTHI477_RS01930	1821	C	T	synonymous
396148	NTHI477_RS01930	1813	T	C	nonsynonymous
396193	NTHI477_RS01930	1768	T	C	nonsynonymous
396196	NTHI477_RS01930	1765	G	T	nonsynonymous
396199	NTHI477_RS01930	1762	C	A	nonsynonymous
396203	NTHI477_RS01930	1758	G	A	synonymous
396206	NTHI477_RS01930	1755	C	A	nonsynonymous
396206	NTHI477_RS01930	1755	C	T	synonymous
396226	NTHI477_RS01930	1735	A	G	nonsynonymous
396251	NTHI477_RS01930	1710	G	A	synonymous
396274	NTHI477_RS01930	1687	T	G	nonsynonymous
396295	NTHI477_RS01930	1666	G	A	nonsynonymous
396296	NTHI477_RS01930	1665	T	C	synonymous
396331	NTHI477_RS01930	1630	G	T	nonsynonymous
396332	NTHI477_RS01930	1629	A	T	synonymous
396347	NTHI477_RS01930	1614	C	G	synonymous
396347	NTHI477_RS01930	1614	C	A	synonymous
396350	NTHI477_RS01930	1611	T	C	synonymous
396377	NTHI477_RS01930	1584	A	G	synonymous
396379	NTHI477_RS01930	1582	A	G	nonsynonymous
396380	NTHI477_RS01930	1581	T	C	synonymous
396383	NTHI477_RS01930	1578	G	A	synonymous
396428	NTHI477_RS01930	1533	G	C	synonymous
396430	NTHI477_RS01930	1531	G	A	nonsynonymous
396434	NTHI477_RS01930	1527	G	A	synonymous
396437	NTHI477_RS01930	1524	A	G	synonymous
396443	NTHI477_RS01930	1518	A	G	synonymous
396452	NTHI477_RS01930	1509	T	C	synonymous
396455	NTHI477_RS01930	1506	C	T	synonymous
396470	NTHI477_RS01930	1491	T	A	nonsynonymous
396473	NTHI477_RS01930	1488	T	G	synonymous
396475	NTHI477_RS01930	1486	A	C	nonsynonymous
396476	NTHI477_RS01930	1485	A	C	nonsynonymous
396485	NTHI477_RS01930	1476	A	G	synonymous
396486	NTHI477_RS01930	1475	A	G	nonsynonymous
396487	NTHI477_RS01930	1474	A	C	nonsynonymous

396490	NTHI477_RS01930	1471	T	G	nonsynonymous
396494	NTHI477_RS01930	1467	T	C	synonymous
396496	NTHI477_RS01930	1465	G	A	synonymous
396503	NTHI477_RS01930	1458	A	C	synonymous
396511	NTHI477_RS01930	1450	C	T	nonsynonymous
396530	NTHI477_RS01930	1431	T	C	synonymous
396533	NTHI477_RS01930	1428	C	A	synonymous
396535	NTHI477_RS01930	1426	C	T	nonsynonymous
396536	NTHI477_RS01930	1425	G	C	synonymous
396539	NTHI477_RS01930	1422	G	T	synonymous
396545	NTHI477_RS01930	1416	T	A	synonymous
396551	NTHI477_RS01930	1410	G	A	synonymous
396554	NTHI477_RS01930	1407	G	T	synonymous
396556	NTHI477_RS01930	1405	G	A	nonsynonymous
396557	NTHI477_RS01930	1404	T	A	synonymous
396560	NTHI477_RS01930	1401	A	T	synonymous
396563	NTHI477_RS01930	1398	C	T	synonymous
396565	NTHI477_RS01930	1396	G	A	synonymous
396574	NTHI477_RS01930	1387	T	C	nonsynonymous
396580	NTHI477_RS01930	1381	A	C	nonsynonymous
396590	NTHI477_RS01930	1371	G	A	synonymous
396592	NTHI477_RS01930	1369	G	A	nonsynonymous
396599	NTHI477_RS01930	1362	C	T	synonymous
396602	NTHI477_RS01930	1359	G	C	synonymous
396605	NTHI477_RS01930	1356	T	C	synonymous
396611	NTHI477_RS01930	1350	C	A	synonymous
396632	NTHI477_RS01930	1329	G	A	synonymous
396662	NTHI477_RS01930	1299	C	T	synonymous
396665	NTHI477_RS01930	1296	A	G	synonymous
396671	NTHI477_RS01930	1290	A	C	synonymous
396671	NTHI477_RS01930	1290	A	T	synonymous
396673	NTHI477_RS01930	1288	C	A	nonsynonymous
396692	NTHI477_RS01930	1269	T	C	synonymous
396717	NTHI477_RS01930	1244	T	C	nonsynonymous
396728	NTHI477_RS01930	1233	G	A	synonymous
396748	NTHI477_RS01930	1213	T	G	nonsynonymous
396751	NTHI477_RS01930	1210	C	T	nonsynonymous
396752	NTHI477_RS01930	1209	T	C	synonymous
396758	NTHI477_RS01930	1203	A	C	synonymous
396763	NTHI477_RS01930	1198	A	C	nonsynonymous
396767	NTHI477_RS01930	1194	G	A	synonymous
396776	NTHI477_RS01930	1185	G	C	synonymous
396785	NTHI477_RS01930	1176	T	C	synonymous
396788	NTHI477_RS01930	1173	G	T	synonymous
396788	NTHI477_RS01930	1173	G	A	synonymous

396791	NTHI477_RS01930	1170	G	A	synonymous
396794	NTHI477_RS01930	1167	G	A	synonymous
396797	NTHI477_RS01930	1164	A	T	synonymous
396797	NTHI477_RS01930	1164	A	C	synonymous
396811	NTHI477_RS01930	1150	G	A	synonymous
396812	NTHI477_RS01930	1149	C	G	synonymous
396821	NTHI477_RS01930	1140	A	C	nonsynonymous
396830	NTHI477_RS01930	1131	G	T	nonsynonymous
396840	NTHI477_RS01930	1121	T	C	nonsynonymous
396843	NTHI477_RS01930	1118	C	T	nonsynonymous
396850	NTHI477_RS01930	1111	C	T	nonsynonymous
396851	NTHI477_RS01930	1110	C	T	synonymous
396852	NTHI477_RS01930	1109	T	C	nonsynonymous
396856	NTHI477_RS01930	1105	G	T	nonsynonymous
396856	NTHI477_RS01930	1105	G	C	nonsynonymous
396857	NTHI477_RS01930	1104	G	A	synonymous
396860	NTHI477_RS01930	1101	G	A	synonymous
396860	NTHI477_RS01930	1101	G	C	synonymous
396866	NTHI477_RS01930	1095	G	A	synonymous
396876	NTHI477_RS01930	1085	G	A	nonsynonymous
396878	NTHI477_RS01930	1083	T	A	synonymous
396881	NTHI477_RS01930	1080	T	C	synonymous
396882	NTHI477_RS01930	1079	A	T	nonsynonymous
396885	NTHI477_RS01930	1076	G	A	nonsynonymous
396888	NTHI477_RS01930	1073	G	A	nonsynonymous
396889	NTHI477_RS01930	1072	G	T	nonsynonymous
396889	NTHI477_RS01930	1072	G	C	nonsynonymous
396890	NTHI477_RS01930	1071	C	T	synonymous
396897	NTHI477_RS01930	1064	G	A	nonsynonymous
396898	NTHI477_RS01930	1063	A	G	nonsynonymous
396900	NTHI477_RS01930	1061	C	T	nonsynonymous
396902	NTHI477_RS01930	1059	G	A	synonymous
396914	NTHI477_RS01930	1047	G	A	synonymous
396917	NTHI477_RS01930	1044	T	C	synonymous
396930	NTHI477_RS01930	1031	A	T	nonsynonymous
396952	NTHI477_RS01930	1009	A	T	nonsynonymous
396961	NTHI477_RS01930	1000	A	C	nonsynonymous
396965	NTHI477_RS01930	996	G	A	synonymous
396983	NTHI477_RS01930	978	A	T	synonymous
397000	NTHI477_RS01930	961	C	T	nonsynonymous
397002	NTHI477_RS01930	959	C	T	nonsynonymous
397010	NTHI477_RS01930	951	G	A	synonymous
397043	NTHI477_RS01930	918	C	T	synonymous
397070	NTHI477_RS01930	891	C	G	synonymous
397073	NTHI477_RS01930	888	A	G	synonymous

397096	NTHI477_RS01930	865	G	C	nonsynonymous
397127	NTHI477_RS01930	834	G	A	synonymous
397129	NTHI477_RS01930	832	G	A	nonsynonymous
397163	NTHI477_RS01930	798	T	C	synonymous
397166	NTHI477_RS01930	795	C	A	synonymous
397169	NTHI477_RS01930	792	T	A	synonymous
397175	NTHI477_RS01930	786	G	A	synonymous
397190	NTHI477_RS01930	771	A	T	synonymous
397193	NTHI477_RS01930	768	A	G	synonymous
397196	NTHI477_RS01930	765	G	A	synonymous
397218	NTHI477_RS01930	743	T	C	nonsynonymous
397224	NTHI477_RS01930	737	T	C	nonsynonymous
397228	NTHI477_RS01930	733	G	C	nonsynonymous
397238	NTHI477_RS01930	723	G	C	synonymous
397250	NTHI477_RS01930	711	C	T	nonsynonymous
397252	NTHI477_RS01930	709	T	C	nonsynonymous
397253	NTHI477_RS01930	708	T	C	synonymous
397256	NTHI477_RS01930	705	G	C	synonymous
397256	NTHI477_RS01930	705	G	T	synonymous
397268	NTHI477_RS01930	693	C	G	synonymous
397280	NTHI477_RS01930	681	A	T	synonymous
397289	NTHI477_RS01930	672	A	G	synonymous
397295	NTHI477_RS01930	666	T	G	synonymous
397295	NTHI477_RS01930	666	T	A	synonymous
397297	NTHI477_RS01930	664	G	A	nonsynonymous
397298	NTHI477_RS01930	663	T	G	nonsynonymous
397302	NTHI477_RS01930	659	T	C	nonsynonymous
397302	NTHI477_RS01930	659	T	G	nonsynonymous
397303	NTHI477_RS01930	658	T	G	nonsynonymous
397311	NTHI477_RS01930	650	C	G	nonsynonymous
397312	NTHI477_RS01930	649	G	T	synonymous
397313	NTHI477_RS01930	648	A	C	nonsynonymous
397315	NTHI477_RS01930	646	T	A	nonsynonymous
397318	NTHI477_RS01930	643	T	C	nonsynonymous
397321	NTHI477_RS01930	640	G	A	synonymous
397328	NTHI477_RS01930	633	G	T	synonymous
397328	NTHI477_RS01930	633	G	A	synonymous
397331	NTHI477_RS01930	630	C	A	synonymous
397334	NTHI477_RS01930	627	A	G	synonymous
397343	NTHI477_RS01930	618	T	A	synonymous
397346	NTHI477_RS01930	615	C	T	synonymous
397346	NTHI477_RS01930	615	C	G	nonsynonymous
397347	NTHI477_RS01930	614	T	C	nonsynonymous
397349	NTHI477_RS01930	612	T	G	synonymous
397349	NTHI477_RS01930	612	T	C	synonymous

397351	NTHI477_RS01930	610	G	C	nonsynonymous
397367	NTHI477_RS01930	594	A	G	synonymous
397370	NTHI477_RS01930	591	C	G	synonymous
397376	NTHI477_RS01930	585	T	C	synonymous
397388	NTHI477_RS01930	573	A	C	nonsynonymous
397397	NTHI477_RS01930	564	A	G	synonymous
397400	NTHI477_RS01930	561	G	A	synonymous
397400	NTHI477_RS01930	561	G	C	synonymous
397412	NTHI477_RS01930	549	T	C	synonymous
397414	NTHI477_RS01930	547	C	T	nonsynonymous
397436	NTHI477_RS01930	525	T	C	synonymous
397436	NTHI477_RS01930	525	T	A	synonymous
397448	NTHI477_RS01930	513	A	G	synonymous
397454	NTHI477_RS01930	507	C	T	synonymous
397469	NTHI477_RS01930	492	A	G	synonymous
397471	NTHI477_RS01930	490	C	A	nonsynonymous
397472	NTHI477_RS01930	489	A	G	synonymous
397477	NTHI477_RS01930	484	G	A	synonymous
397498	NTHI477_RS01930	463	C	T	nonsynonymous
397499	NTHI477_RS01930	462	C	T	synonymous
397514	NTHI477_RS01930	447	G	A	synonymous
397529	NTHI477_RS01930	432	C	T	synonymous
397538	NTHI477_RS01930	423	G	A	synonymous
397552	NTHI477_RS01930	409	G	T	nonsynonymous
397553	NTHI477_RS01930	408	C	T	synonymous
397570	NTHI477_RS01930	391	C	T	nonsynonymous
397571	NTHI477_RS01930	390	C	G	synonymous
397573	NTHI477_RS01930	388	C	G	nonsynonymous
397573	NTHI477_RS01930	388	C	T	nonsynonymous
397619	NTHI477_RS01930	342	G	A	synonymous
397620	NTHI477_RS01930	341	C	T	nonsynonymous
397622	NTHI477_RS01930	339	T	C	synonymous
397628	NTHI477_RS01930	333	A	G	synonymous
397629	NTHI477_RS01930	332	C	T	nonsynonymous
397631	NTHI477_RS01930	330	T	C	synonymous
397645	NTHI477_RS01930	316	G	A	nonsynonymous
397645	NTHI477_RS01930	316	G	C	nonsynonymous
397667	NTHI477_RS01930	294	T	A	synonymous
397732	NTHI477_RS01930	229	T	C	nonsynonymous
397733	NTHI477_RS01930	228	G	A	synonymous
397739	NTHI477_RS01930	222	G	C	synonymous
397751	NTHI477_RS01930	210	T	C	synonymous
397754	NTHI477_RS01930	207	A	G	synonymous
397766	NTHI477_RS01930	195	T	C	synonymous
397780	NTHI477_RS01930	181	C	T	nonsynonymous

397790	NTHI477_RS01930	171	C	T	synonymous
397814	NTHI477_RS01930	147	T	C	synonymous
397815	NTHI477_RS01930	146	C	G	nonsynonymous
397826	NTHI477_RS01930	135	G	A	synonymous
397844	NTHI477_RS01930	117	T	C	synonymous
397847	NTHI477_RS01930	114	G	A	synonymous
397865	NTHI477_RS01930	96	C	A	synonymous
397886	NTHI477_RS01930	75	T	G	synonymous
397889	NTHI477_RS01930	72	G	A	synonymous
397895	NTHI477_RS01930	66	C	T	synonymous
397898	NTHI477_RS01930	63	A	T	synonymous
397903	NTHI477_RS01930	58	T	G	nonsynonymous
397916	NTHI477_RS01930	45	T	C	synonymous
397922	NTHI477_RS01930	39	G	A	synonymous
397928	NTHI477_RS01930	33	G	T	nonsynonymous
397930	NTHI477_RS01930	31	A	T	nonsynonymous
397946	NTHI477_RS01930	15	A	C	nonsynonymous
397946	NTHI477_RS01930	15	A	T	nonsynonymous
397957	NTHI477_RS01930	4	G	A	nonsynonymous
397962	intergenic	intergenic	G	A	intergenic
397964	intergenic	intergenic	G	T	intergenic
397971	NTHI477_RS01935	336	T	C	synonymous
397986	NTHI477_RS01935	321	T	C	nonsynonymous
398034	NTHI477_RS01935	273	G	T	synonymous
398064	NTHI477_RS01935	243	T	C	synonymous
398073	NTHI477_RS01935	234	C	T	synonymous
398075	NTHI477_RS01935	232	G	C	nonsynonymous
398076	NTHI477_RS01935	231	G	A	synonymous
398097	NTHI477_RS01935	210	A	G	synonymous
398115	NTHI477_RS01935	192	T	C	synonymous
398116	NTHI477_RS01935	191	A	C	nonsynonymous
398118	NTHI477_RS01935	189	C	T	synonymous
398125	NTHI477_RS01935	182	T	C	nonsynonymous
398139	NTHI477_RS01935	168	A	G	synonymous
398154	NTHI477_RS01935	153	A	G	synonymous
398181	NTHI477_RS01935	126	T	A	nonsynonymous
398262	NTHI477_RS01935	45	C	T	synonymous
398273	NTHI477_RS01935	34	C	A	nonsynonymous
398297	NTHI477_RS01935	10	C	G	nonsynonymous
398357	intergenic	intergenic	C	G	intergenic
398407	NTHI477_RS01940	1632	A	G	synonymous
398408	NTHI477_RS01940	1631	G	A	nonsynonymous
398410	NTHI477_RS01940	1629	G	T	synonymous
398430	NTHI477_RS01940	1609	T	C	nonsynonymous
398470	NTHI477_RS01940	1569	G	A	synonymous

398497	NTHI477_RS01940	1542	G	A	synonymous
398539	NTHI477_RS01940	1500	G	A	synonymous
398542	NTHI477_RS01940	1497	C	G	synonymous
398548	NTHI477_RS01940	1491	A	C	synonymous
398575	NTHI477_RS01940	1464	G	A	synonymous
398599	NTHI477_RS01940	1440	G	A	synonymous
398608	NTHI477_RS01940	1431	A	G	synonymous
398692	NTHI477_RS01940	1347	A	T	synonymous
398692	NTHI477_RS01940	1347	A	C	synonymous
398695	NTHI477_RS01940	1344	G	A	synonymous
398698	NTHI477_RS01940	1341	C	T	synonymous
398707	NTHI477_RS01940	1332	C	G	synonymous
398710	NTHI477_RS01940	1329	C	T	synonymous
398713	NTHI477_RS01940	1326	A	G	synonymous
398728	NTHI477_RS01940	1311	T	C	synonymous
398731	NTHI477_RS01940	1308	A	G	synonymous
398746	NTHI477_RS01940	1293	A	G	synonymous
398749	NTHI477_RS01940	1290	C	T	synonymous
398749	NTHI477_RS01940	1290	C	A	synonymous
398752	NTHI477_RS01940	1287	C	A	synonymous
398752	NTHI477_RS01940	1287	C	T	synonymous
398769	NTHI477_RS01940	1270	G	A	nonsynonymous
398782	NTHI477_RS01940	1257	G	A	synonymous
398785	NTHI477_RS01940	1254	G	A	synonymous
398791	NTHI477_RS01940	1248	A	G	synonymous
398797	NTHI477_RS01940	1242	G	A	synonymous
398800	NTHI477_RS01940	1239	T	C	synonymous
398806	NTHI477_RS01940	1233	A	G	synonymous
398812	NTHI477_RS01940	1227	C	G	synonymous
398827	NTHI477_RS01940	1212	A	G	synonymous
398836	NTHI477_RS01940	1203	G	A	synonymous
398845	NTHI477_RS01940	1194	T	A	synonymous
398847	NTHI477_RS01940	1192	A	C	nonsynonymous
398848	NTHI477_RS01940	1191	G	A	synonymous
398857	NTHI477_RS01940	1182	C	T	synonymous
398863	NTHI477_RS01940	1176	T	C	synonymous
398866	NTHI477_RS01940	1173	C	A	synonymous
398869	NTHI477_RS01940	1170	A	C	synonymous
398875	NTHI477_RS01940	1164	C	T	synonymous
398914	NTHI477_RS01940	1125	T	C	synonymous
398917	NTHI477_RS01940	1122	T	C	synonymous
398935	NTHI477_RS01940	1104	A	G	synonymous
398943	NTHI477_RS01940	1096	A	G	synonymous
398944	NTHI477_RS01940	1095	G	A	synonymous
398947	NTHI477_RS01940	1092	T	A	synonymous

398956	NTHI477_RS01940	1083	T	C	synonymous
398962	NTHI477_RS01940	1077	G	A	synonymous
398965	NTHI477_RS01940	1074	C	T	synonymous
398968	NTHI477_RS01940	1071	T	C	synonymous
398971	NTHI477_RS01940	1068	G	A	synonymous
398977	NTHI477_RS01940	1062	C	T	synonymous
398983	NTHI477_RS01940	1056	A	G	synonymous
398989	NTHI477_RS01940	1050	C	A	synonymous
399001	NTHI477_RS01940	1038	A	G	synonymous
399004	NTHI477_RS01940	1035	G	A	synonymous
399007	NTHI477_RS01940	1032	T	G	synonymous
399010	NTHI477_RS01940	1029	T	C	synonymous
399028	NTHI477_RS01940	1011	G	A	synonymous
399046	NTHI477_RS01940	993	G	A	synonymous
399052	NTHI477_RS01940	987	G	T	nonsynonymous
399079	NTHI477_RS01940	960	T	C	synonymous
399112	NTHI477_RS01940	927	C	A	synonymous
399118	NTHI477_RS01940	921	G	A	synonymous
399121	NTHI477_RS01940	918	T	A	synonymous
399130	NTHI477_RS01940	909	A	G	synonymous
399148	NTHI477_RS01940	891	T	C	synonymous
399157	NTHI477_RS01940	882	A	G	synonymous
399160	NTHI477_RS01940	879	A	C	synonymous
399160	NTHI477_RS01940	879	A	T	synonymous
399161	NTHI477_RS01940	878	G	A	nonsynonymous
399175	NTHI477_RS01940	864	G	A	synonymous
399187	NTHI477_RS01940	852	G	A	synonymous
399217	NTHI477_RS01940	822	T	C	synonymous
399232	NTHI477_RS01940	807	G	T	synonymous
399304	NTHI477_RS01940	735	A	G	synonymous
399325	NTHI477_RS01940	714	G	A	synonymous
399328	NTHI477_RS01940	711	C	T	synonymous
399331	NTHI477_RS01940	708	G	A	synonymous
399361	NTHI477_RS01940	678	C	T	synonymous
399364	NTHI477_RS01940	675	A	G	synonymous
399388	NTHI477_RS01940	651	A	G	synonymous
399391	NTHI477_RS01940	648	G	A	synonymous
399397	NTHI477_RS01940	642	T	C	synonymous
399400	NTHI477_RS01940	639	C	A	synonymous
399403	NTHI477_RS01940	636	T	A	synonymous
399406	NTHI477_RS01940	633	A	G	synonymous
399409	NTHI477_RS01940	630	C	T	synonymous
399412	NTHI477_RS01940	627	G	A	synonymous
399415	NTHI477_RS01940	624	G	A	synonymous
399418	NTHI477_RS01940	621	G	A	synonymous

399427	NTHI477_RS01940	612	T	A	synonymous
399439	NTHI477_RS01940	600	C	T	synonymous
399457	NTHI477_RS01940	582	T	C	synonymous
399466	NTHI477_RS01940	573	A	G	synonymous
399472	NTHI477_RS01940	567	T	C	synonymous
399475	NTHI477_RS01940	564	A	G	synonymous
399481	NTHI477_RS01940	558	C	T	synonymous
399484	NTHI477_RS01940	555	C	G	nonsynonymous
399486	NTHI477_RS01940	553	A	G	synonymous
399490	NTHI477_RS01940	549	A	G	synonymous
399508	NTHI477_RS01940	531	T	C	synonymous
399514	NTHI477_RS01940	525	G	A	synonymous
399520	NTHI477_RS01940	519	T	C	synonymous
399526	NTHI477_RS01940	513	T	A	synonymous
399529	NTHI477_RS01940	510	G	A	synonymous
399538	NTHI477_RS01940	501	A	T	synonymous
399541	NTHI477_RS01940	498	A	C	synonymous
399544	NTHI477_RS01940	495	A	T	synonymous
399553	NTHI477_RS01940	486	T	C	synonymous
399556	NTHI477_RS01940	483	A	G	synonymous
399562	NTHI477_RS01940	477	G	A	synonymous
399562	NTHI477_RS01940	477	G	C	synonymous
399566	NTHI477_RS01940	473	T	C	nonsynonymous
399571	NTHI477_RS01940	468	A	G	synonymous
399574	NTHI477_RS01940	465	A	T	synonymous
399586	NTHI477_RS01940	453	C	T	synonymous
399595	NTHI477_RS01940	444	C	G	synonymous
399598	NTHI477_RS01940	441	A	G	synonymous
399601	NTHI477_RS01940	438	C	T	synonymous
399625	NTHI477_RS01940	414	C	T	synonymous
399643	NTHI477_RS01940	396	G	A	synonymous
399645	NTHI477_RS01940	394	T	C	nonsynonymous
399646	NTHI477_RS01940	393	A	G	synonymous
399670	NTHI477_RS01940	369	C	T	synonymous
399670	NTHI477_RS01940	369	C	G	synonymous
399694	NTHI477_RS01940	345	A	G	synonymous
399877	NTHI477_RS01940	162	T	C	synonymous
400000	NTHI477_RS01940	39	G	A	synonymous
400148	intergenic	intergenic	C	A	intergenic
400168	intergenic	intergenic	T	C	intergenic
400242	NTHI477_RS01945	283	G	A	nonsynonymous
400375	NTHI477_RS01945	150	C	T	synonymous
400606	NTHI477_RS01950	235	G	A	nonsynonymous
400628	NTHI477_RS01950	213	A	G	synonymous
400677	NTHI477_RS01950	164	G	A	nonsynonymous

400735	NTHI477_RS01950	106	C	T	nonsynonymous
400978	intergenic	intergenic	T	C	intergenic
401018	intergenic	intergenic	G	A	intergenic
401171	NTHI477_RS01955	8	C	T	nonsynonymous
401197	NTHI477_RS01955	34	A	G	nonsynonymous
401274	NTHI477_RS01955	111	C	T	synonymous
401298	NTHI477_RS01955	135	G	A	synonymous
401356	NTHI477_RS01955	193	G	T	nonsynonymous
401385	NTHI477_RS01955	222	A	G	synonymous
401406	NTHI477_RS01955	243	G	A	synonymous
401433	NTHI477_RS01955	270	C	T	synonymous
401461	NTHI477_RS01955	298	C	A	nonsynonymous
401492	NTHI477_RS01955	329	G	A	nonsynonymous
401553	NTHI477_RS01955	390	C	T	synonymous
401595	NTHI477_RS01955	432	G	A	synonymous
401607	NTHI477_RS01955	444	T	G	synonymous
401757	NTHI477_RS01955	594	A	G	synonymous
401842	NTHI477_RS01960	56	T	C	nonsynonymous
401842	NTHI477_RS01960	56	T	G	nonsynonymous
401845	NTHI477_RS01960	59	A	C	nonsynonymous
401876	NTHI477_RS01960	90	T	C	synonymous
401877	NTHI477_RS01960	91	G	A	nonsynonymous
401878	NTHI477_RS01960	92	C	G	nonsynonymous
401879	NTHI477_RS01960	93	A	T	synonymous
401891	NTHI477_RS01960	105	T	G	nonsynonymous
401900	NTHI477_RS01960	114	A	G	synonymous
401957	NTHI477_RS01960	171	A	G	synonymous
401976	NTHI477_RS01960	190	A	G	nonsynonymous
401981	NTHI477_RS01960	195	T	C	synonymous
401990	NTHI477_RS01960	204	T	C	synonymous
401997	NTHI477_RS01960	211	C	T	nonsynonymous
402026	NTHI477_RS01960	240	T	C	synonymous
402044	NTHI477_RS01960	258	G	C	synonymous
402047	NTHI477_RS01960	261	T	C	synonymous
402080	NTHI477_RS01960	294	C	T	synonymous
402089	NTHI477_RS01960	303	T	A	synonymous
402113	NTHI477_RS01960	327	G	A	synonymous
402113	NTHI477_RS01960	327	G	C	synonymous
402125	NTHI477_RS01960	339	C	A	synonymous
402176	NTHI477_RS01960	390	T	C	synonymous
402246	NTHI477_RS01960	460	T	C	synonymous
402252	NTHI477_RS01960	466	G	A	nonsynonymous
402276	NTHI477_RS01960	490	G	A	nonsynonymous
402344	NTHI477_RS01960	558	G	A	synonymous
402353	NTHI477_RS01960	567	C	T	synonymous

402359	NTHI477_RS01960	573	T	C	synonymous
402362	NTHI477_RS01960	576	T	C	synonymous
402386	NTHI477_RS01960	600	G	C	synonymous
402389	NTHI477_RS01960	603	G	A	synonymous
402407	NTHI477_RS01960	621	C	T	synonymous
402411	NTHI477_RS01960	625	C	T	synonymous
402419	NTHI477_RS01960	633	G	A	synonymous
402425	NTHI477_RS01960	639	C	T	synonymous
402435	NTHI477_RS01960	649	C	T	synonymous
402446	NTHI477_RS01960	660	T	C	synonymous
402452	NTHI477_RS01960	666	C	A	synonymous
402455	NTHI477_RS01960	669	T	A	synonymous
402461	NTHI477_RS01960	675	C	T	synonymous
402467	NTHI477_RS01960	681	G	A	synonymous
402548	NTHI477_RS01960	762	G	A	synonymous
402590	NTHI477_RS01960	804	G	A	synonymous
402591	NTHI477_RS01960	805	A	C	synonymous
402616	NTHI477_RS01960	830	G	A	nonsynonymous
402633	NTHI477_RS01960	847	A	C	nonsynonymous
402656	NTHI477_RS01960	870	T	C	synonymous
402666	NTHI477_RS01960	880	T	C	synonymous
402674	NTHI477_RS01960	888	A	T	synonymous
402699	NTHI477_RS01960	913	T	C	synonymous
402704	NTHI477_RS01960	918	T	C	synonymous
402710	NTHI477_RS01960	924	G	A	synonymous
402713	NTHI477_RS01960	927	T	C	synonymous
402725	NTHI477_RS01960	939	C	T	synonymous
402728	NTHI477_RS01960	942	A	C	synonymous
402729	NTHI477_RS01960	943	A	G	nonsynonymous
402779	intergenic	intergenic	T	A	intergenic
402780	intergenic	intergenic	G	T	intergenic
402789	intergenic	intergenic	C	A	intergenic
403025	intergenic	intergenic	T	C	intergenic
403035	intergenic	intergenic	T	C	intergenic
403123	intergenic	intergenic	G	A	intergenic
403150	intergenic	intergenic	A	G	intergenic
403151	intergenic	intergenic	A	C	intergenic
403161	intergenic	intergenic	T	C	intergenic
403175	intergenic	intergenic	A	T	intergenic
403183	intergenic	intergenic	T	A	intergenic
403185	intergenic	intergenic	A	C	intergenic
403223	intergenic	intergenic	T	A	intergenic
403237	intergenic	intergenic	A	T	intergenic
403240	intergenic	intergenic	G	A	intergenic
403250	intergenic	intergenic	C	G	intergenic

403256	intergenic	intergenic	T	C	intergenic
403268	intergenic	intergenic	G	A	intergenic
403275	intergenic	intergenic	C	T	intergenic
403314	intergenic	intergenic	A	G	intergenic
403315	intergenic	intergenic	G	T	intergenic
403334	intergenic	intergenic	A	C	intergenic
403346	intergenic	intergenic	C	T	intergenic
403350	intergenic	intergenic	C	G	intergenic
403358	intergenic	intergenic	C	T	intergenic
403359	intergenic	intergenic	G	A	intergenic
403396	NTHI477_RS01970	27	A	G	synonymous
403423	NTHI477_RS01970	54	C	T	synonymous
403456	NTHI477_RS01970	87	G	A	synonymous
403477	NTHI477_RS01970	108	T	A	synonymous
403483	NTHI477_RS01970	114	G	A	synonymous
403483	NTHI477_RS01970	114	G	T	synonymous
403486	NTHI477_RS01970	117	T	C	synonymous
403495	NTHI477_RS01970	126	A	G	synonymous
403501	NTHI477_RS01970	132	G	A	synonymous
403507	NTHI477_RS01970	138	T	A	synonymous
403510	NTHI477_RS01970	141	C	A	synonymous
403516	NTHI477_RS01970	147	G	A	synonymous
403519	NTHI477_RS01970	150	G	A	synonymous
403522	NTHI477_RS01970	153	G	C	synonymous
403534	NTHI477_RS01970	165	C	T	synonymous
403543	NTHI477_RS01970	174	C	T	synonymous
403558	NTHI477_RS01970	189	C	T	synonymous
403561	NTHI477_RS01970	192	C	A	synonymous
403564	NTHI477_RS01970	195	G	A	synonymous
403594	NTHI477_RS01970	225	T	G	synonymous
403597	NTHI477_RS01970	228	T	C	synonymous
403600	NTHI477_RS01970	231	G	A	synonymous
403621	NTHI477_RS01970	252	T	C	synonymous
403693	NTHI477_RS01970	324	A	G	synonymous
403723	NTHI477_RS01970	354	G	A	synonymous
403789	NTHI477_RS01970	420	T	C	synonymous
403801	NTHI477_RS01970	432	G	A	synonymous
403804	NTHI477_RS01970	435	T	G	synonymous
403915	NTHI477_RS01970	546	G	A	synonymous
403921	NTHI477_RS01970	552	G	A	synonymous
403933	NTHI477_RS01970	564	A	C	synonymous
403972	NTHI477_RS01970	603	C	T	synonymous
403975	NTHI477_RS01970	606	A	C	synonymous
404005	NTHI477_RS01970	636	T	A	synonymous
404080	NTHI477_RS01970	711	T	C	synonymous

404098	NTHI477_RS01970	729	A	C	synonymous
404128	NTHI477_RS01970	759	C	T	synonymous
404134	NTHI477_RS01970	765	G	T	synonymous
404203	NTHI477_RS01970	834	C	T	synonymous
404213	NTHI477_RS01970	844	G	C	nonsynonymous
404218	NTHI477_RS01970	849	T	C	synonymous
404236	NTHI477_RS01970	867	G	A	synonymous
404248	NTHI477_RS01970	879	A	G	synonymous
404251	NTHI477_RS01970	882	A	T	synonymous
404299	NTHI477_RS01970	930	A	C	synonymous
404299	NTHI477_RS01970	930	A	T	synonymous
404323	NTHI477_RS01970	954	A	G	synonymous
404326	NTHI477_RS01970	957	G	A	synonymous
404329	NTHI477_RS01970	960	C	A	synonymous
404338	NTHI477_RS01970	969	G	A	synonymous
404344	NTHI477_RS01970	975	A	G	synonymous
404350	NTHI477_RS01970	981	A	C	synonymous
404359	NTHI477_RS01970	990	A	G	synonymous
404362	NTHI477_RS01970	993	T	C	synonymous
404368	NTHI477_RS01970	999	T	C	synonymous
404383	NTHI477_RS01970	1014	G	C	synonymous
404383	NTHI477_RS01970	1014	G	T	synonymous
404410	NTHI477_RS01970	1041	A	G	synonymous
404413	NTHI477_RS01970	1044	T	C	synonymous
404413	NTHI477_RS01970	1044	T	G	synonymous
404428	NTHI477_RS01970	1059	T	C	synonymous
404431	NTHI477_RS01970	1062	G	A	synonymous
404443	NTHI477_RS01970	1074	T	C	synonymous
404452	NTHI477_RS01970	1083	A	G	synonymous
404455	NTHI477_RS01970	1086	T	C	synonymous
404467	NTHI477_RS01970	1098	A	G	synonymous
404470	NTHI477_RS01970	1101	C	T	synonymous
404470	NTHI477_RS01970	1101	C	G	synonymous
404488	NTHI477_RS01970	1119	G	A	synonymous
404491	NTHI477_RS01970	1122	G	A	synonymous
404497	NTHI477_RS01970	1128	A	C	synonymous
404542	NTHI477_RS01970	1173	T	C	synonymous
404545	NTHI477_RS01970	1176	G	A	synonymous
404551	NTHI477_RS01970	1182	C	T	synonymous
404554	NTHI477_RS01970	1185	T	C	synonymous
404575	NTHI477_RS01970	1206	G	A	synonymous
404576	NTHI477_RS01970	1207	C	G	nonsynonymous
404578	NTHI477_RS01970	1209	T	C	synonymous
404590	NTHI477_RS01970	1221	A	G	synonymous
404599	NTHI477_RS01970	1230	C	T	synonymous

404629	NTHI477_RS01970	1260	C	A	synonymous
404638	NTHI477_RS01970	1269	G	A	synonymous
404647	NTHI477_RS01970	1278	G	A	synonymous
404650	NTHI477_RS01970	1281	T	C	synonymous
404668	NTHI477_RS01970	1299	G	A	synonymous
404668	NTHI477_RS01970	1299	G	C	synonymous
404671	NTHI477_RS01970	1302	A	T	synonymous
404695	NTHI477_RS01970	1326	C	T	synonymous
404700	NTHI477_RS01970	1331	C	T	nonsynonymous
404713	NTHI477_RS01970	1344	G	A	synonymous
404728	NTHI477_RS01970	1359	A	C	synonymous
404737	NTHI477_RS01970	1368	C	G	synonymous
404740	NTHI477_RS01970	1371	A	T	synonymous
404749	NTHI477_RS01970	1380	T	A	synonymous
404761	NTHI477_RS01970	1392	G	A	synonymous
404782	NTHI477_RS01970	1413	A	G	synonymous
404791	NTHI477_RS01970	1422	C	T	synonymous
404792	NTHI477_RS01970	1423	G	T	nonsynonymous
404794	NTHI477_RS01970	1425	G	A	synonymous
404797	NTHI477_RS01970	1428	T	C	synonymous
404800	NTHI477_RS01970	1431	T	A	synonymous
404803	NTHI477_RS01970	1434	G	A	synonymous
404812	NTHI477_RS01970	1443	A	G	synonymous
404818	NTHI477_RS01970	1449	T	C	synonymous
404830	NTHI477_RS01970	1461	C	G	synonymous
404830	NTHI477_RS01970	1461	C	T	synonymous
404836	NTHI477_RS01970	1467	A	G	synonymous
404839	NTHI477_RS01970	1470	C	T	synonymous
404839	NTHI477_RS01970	1470	C	A	synonymous
404842	NTHI477_RS01970	1473	C	G	synonymous
404842	NTHI477_RS01970	1473	C	A	synonymous
404869	NTHI477_RS01970	1500	C	A	synonymous
404869	NTHI477_RS01970	1500	C	G	synonymous
404875	NTHI477_RS01970	1506	T	C	synonymous
404881	NTHI477_RS01970	1512	A	G	synonymous
404884	NTHI477_RS01970	1515	A	G	synonymous
404887	NTHI477_RS01970	1518	G	C	synonymous
404887	NTHI477_RS01970	1518	G	T	synonymous
404890	NTHI477_RS01970	1521	A	C	synonymous
404893	NTHI477_RS01970	1524	T	C	synonymous
404899	NTHI477_RS01970	1530	A	G	synonymous
404900	NTHI477_RS01970	1531	T	C	synonymous
404902	NTHI477_RS01970	1533	G	A	synonymous
404908	NTHI477_RS01970	1539	A	G	synonymous
404911	NTHI477_RS01970	1542	T	A	synonymous

404914	NTHI477_RS01970	1545	A	G	synonymous
404923	NTHI477_RS01970	1554	A	T	synonymous
404953	NTHI477_RS01970	1584	A	G	synonymous
404953	NTHI477_RS01970	1584	A	C	synonymous
404953	NTHI477_RS01970	1584	A	T	synonymous
404956	NTHI477_RS01970	1587	C	T	synonymous
404959	NTHI477_RS01970	1590	A	G	synonymous
404965	NTHI477_RS01970	1596	A	T	synonymous
404965	NTHI477_RS01970	1596	A	G	synonymous
404971	NTHI477_RS01970	1602	A	G	synonymous
404974	NTHI477_RS01970	1605	T	C	synonymous
404977	NTHI477_RS01970	1608	C	T	synonymous
404983	NTHI477_RS01970	1614	T	C	synonymous
404989	NTHI477_RS01970	1620	A	G	synonymous
404992	NTHI477_RS01970	1623	T	C	synonymous
405004	NTHI477_RS01970	1635	T	C	synonymous
405013	NTHI477_RS01970	1644	A	G	synonymous
405034	NTHI477_RS01970	1665	G	A	synonymous
405040	NTHI477_RS01970	1671	A	G	synonymous
405046	NTHI477_RS01970	1677	G	A	synonymous
405051	NTHI477_RS01970	1682	A	G	nonsynonymous
405064	NTHI477_RS01970	1695	C	G	synonymous
405064	NTHI477_RS01970	1695	C	A	synonymous
405076	NTHI477_RS01970	1707	C	T	synonymous
405079	NTHI477_RS01970	1710	C	T	synonymous
405082	NTHI477_RS01970	1713	C	T	synonymous
405082	NTHI477_RS01970	1713	C	G	synonymous
405088	NTHI477_RS01970	1719	C	T	synonymous
405111	NTHI477_RS01970	1742	C	T	nonsynonymous
405118	NTHI477_RS01970	1749	C	T	synonymous
405123	NTHI477_RS01970	1754	A	G	nonsynonymous
405130	NTHI477_RS01970	1761	C	T	synonymous
405145	NTHI477_RS01970	1776	A	G	synonymous
405151	NTHI477_RS01970	1782	A	G	synonymous
405163	NTHI477_RS01970	1794	C	T	synonymous
405166	NTHI477_RS01970	1797	C	G	synonymous
405166	NTHI477_RS01970	1797	C	T	synonymous
405172	NTHI477_RS01970	1803	G	A	synonymous
405184	NTHI477_RS01970	1815	G	A	synonymous
405196	NTHI477_RS01970	1827	C	T	synonymous
405204	NTHI477_RS01970	1835	C	T	nonsynonymous
405205	NTHI477_RS01970	1836	G	A	synonymous
405209	NTHI477_RS01970	1840	G	T	nonsynonymous
405233	NTHI477_RS01970	1864	T	G	nonsynonymous
405234	NTHI477_RS01970	1865	C	T	nonsynonymous

405235	NTHI477_RS01970	1866	G	A	synonymous
405302	NTHI477_RS01970	1933	C	T	synonymous
405318	NTHI477_RS01970	1949	A	G	nonsynonymous
405328	NTHI477_RS01970	1959	G	A	synonymous
405352	NTHI477_RS01970	1983	C	T	synonymous
405355	NTHI477_RS01970	1986	G	A	synonymous
405358	NTHI477_RS01970	1989	A	T	synonymous
405365	NTHI477_RS01970	1996	G	A	nonsynonymous
405382	NTHI477_RS01970	2013	G	A	synonymous
405391	NTHI477_RS01970	2022	A	G	synonymous
405420	intergenic	intergenic	A	T	intergenic
405433	intergenic	intergenic	G	A	intergenic
405437	intergenic	intergenic	T	C	intergenic
405438	intergenic	intergenic	G	A	intergenic
405440	NTHI477_RS01975	1926	C	T	synonymous
405446	NTHI477_RS01975	1920	A	T	nonsynonymous
405459	NTHI477_RS01975	1907	G	A	nonsynonymous
405479	NTHI477_RS01975	1887	C	T	synonymous
405488	NTHI477_RS01975	1878	A	G	synonymous
405500	NTHI477_RS01975	1866	A	G	synonymous
405506	NTHI477_RS01975	1860	C	T	synonymous
405524	NTHI477_RS01975	1842	T	C	synonymous
405526	NTHI477_RS01975	1840	C	A	nonsynonymous
405536	NTHI477_RS01975	1830	C	G	nonsynonymous
405559	NTHI477_RS01975	1807	T	A	nonsynonymous
405560	NTHI477_RS01975	1806	T	A	synonymous
405562	NTHI477_RS01975	1804	A	C	nonsynonymous
405584	NTHI477_RS01975	1782	T	C	synonymous
405587	NTHI477_RS01975	1779	T	C	synonymous
405588	NTHI477_RS01975	1778	G	A	nonsynonymous
405608	NTHI477_RS01975	1758	T	C	synonymous
405611	NTHI477_RS01975	1755	C	T	synonymous
405631	NTHI477_RS01975	1735	T	G	nonsynonymous
405662	NTHI477_RS01975	1704	A	G	synonymous
405698	NTHI477_RS01975	1668	A	G	synonymous
405707	NTHI477_RS01975	1659	T	G	synonymous
405716	NTHI477_RS01975	1650	G	T	synonymous
405722	NTHI477_RS01975	1644	T	G	synonymous
405728	NTHI477_RS01975	1638	A	T	synonymous
405740	NTHI477_RS01975	1626	C	T	synonymous
405746	NTHI477_RS01975	1620	A	G	synonymous
405764	NTHI477_RS01975	1602	G	A	synonymous
405779	NTHI477_RS01975	1587	G	A	synonymous
405785	NTHI477_RS01975	1581	A	G	synonymous
405792	NTHI477_RS01975	1574	G	A	nonsynonymous

405793	NTHI477_RS01975	1573	T	C	nonsynonymous
405793	NTHI477_RS01975	1573	T	A	nonsynonymous
405797	NTHI477_RS01975	1569	G	T	synonymous
405800	NTHI477_RS01975	1566	A	C	nonsynonymous
405802	NTHI477_RS01975	1564	C	T	nonsynonymous
405803	NTHI477_RS01975	1563	A	C	synonymous
405808	NTHI477_RS01975	1558	G	T	nonsynonymous
405815	NTHI477_RS01975	1551	G	A	synonymous
405839	NTHI477_RS01975	1527	C	T	synonymous
405842	NTHI477_RS01975	1524	C	T	synonymous
405854	NTHI477_RS01975	1512	T	A	synonymous
405857	NTHI477_RS01975	1509	G	A	synonymous
405883	NTHI477_RS01975	1483	C	T	nonsynonymous
405926	NTHI477_RS01975	1440	C	T	nonsynonymous
405935	NTHI477_RS01975	1431	G	A	synonymous
405947	NTHI477_RS01975	1419	G	C	synonymous
405968	NTHI477_RS01975	1398	T	C	synonymous
405971	NTHI477_RS01975	1395	G	A	synonymous
406010	NTHI477_RS01975	1356	C	T	synonymous
406046	NTHI477_RS01975	1320	G	T	synonymous
406049	NTHI477_RS01975	1317	A	G	synonymous
406055	NTHI477_RS01975	1311	C	T	synonymous
406100	NTHI477_RS01975	1266	A	G	synonymous
406152	NTHI477_RS01975	1214	G	A	nonsynonymous
406166	NTHI477_RS01975	1200	C	T	synonymous
406169	NTHI477_RS01975	1197	A	G	synonymous
406172	NTHI477_RS01975	1194	G	A	synonymous
406199	NTHI477_RS01975	1167	G	A	synonymous
406205	NTHI477_RS01975	1161	A	G	synonymous
406208	NTHI477_RS01975	1158	G	A	synonymous
406217	NTHI477_RS01975	1149	G	A	synonymous
406220	NTHI477_RS01975	1146	C	A	synonymous
406259	NTHI477_RS01975	1107	G	C	synonymous
406296	NTHI477_RS01975	1070	A	C	nonsynonymous
406349	NTHI477_RS01975	1017	T	C	synonymous
406364	NTHI477_RS01975	1002	T	A	synonymous
406367	NTHI477_RS01975	999	G	C	synonymous
406370	NTHI477_RS01975	996	T	G	synonymous
406385	NTHI477_RS01975	981	A	T	synonymous
406388	NTHI477_RS01975	978	T	C	synonymous
406397	NTHI477_RS01975	969	C	T	synonymous
406403	NTHI477_RS01975	963	T	C	synonymous
406466	NTHI477_RS01975	900	T	A	synonymous
406478	NTHI477_RS01975	888	A	G	synonymous
406484	NTHI477_RS01975	882	T	A	synonymous

406493	NTHI477_RS01975	873	C	T	synonymous
406499	NTHI477_RS01975	867	A	G	synonymous
406541	NTHI477_RS01975	825	A	C	synonymous
406552	NTHI477_RS01975	814	T	C	nonsynonymous
406556	NTHI477_RS01975	810	G	A	synonymous
406565	NTHI477_RS01975	801	C	T	synonymous
406598	NTHI477_RS01975	768	A	G	synonymous
406601	NTHI477_RS01975	765	T	G	nonsynonymous
406613	NTHI477_RS01975	753	A	T	synonymous
406616	NTHI477_RS01975	750	G	A	synonymous
406619	NTHI477_RS01975	747	A	T	synonymous
406619	NTHI477_RS01975	747	A	G	synonymous
406643	NTHI477_RS01975	723	A	G	synonymous
406703	NTHI477_RS01975	663	C	A	synonymous
406705	NTHI477_RS01975	661	C	T	nonsynonymous
406712	NTHI477_RS01975	654	G	C	synonymous
406727	NTHI477_RS01975	639	A	G	synonymous
406730	NTHI477_RS01975	636	A	C	synonymous
406738	NTHI477_RS01975	628	G	A	synonymous
406739	NTHI477_RS01975	627	A	G	synonymous
406745	NTHI477_RS01975	621	C	A	synonymous
406756	NTHI477_RS01975	610	C	T	nonsynonymous
406760	NTHI477_RS01975	606	A	G	synonymous
406775	NTHI477_RS01975	591	G	A	synonymous
406778	NTHI477_RS01975	588	G	A	synonymous
406781	NTHI477_RS01975	585	G	A	synonymous
406784	NTHI477_RS01975	582	A	T	synonymous
406790	NTHI477_RS01975	576	A	G	synonymous
406793	NTHI477_RS01975	573	G	A	synonymous
406811	NTHI477_RS01975	555	T	C	synonymous
406814	NTHI477_RS01975	552	C	A	synonymous
406814	NTHI477_RS01975	552	C	G	synonymous
406820	NTHI477_RS01975	546	G	A	synonymous
406823	NTHI477_RS01975	543	G	A	synonymous
406826	NTHI477_RS01975	540	C	A	synonymous
406828	NTHI477_RS01975	538	C	A	nonsynonymous
406828	NTHI477_RS01975	538	C	T	nonsynonymous
406829	NTHI477_RS01975	537	G	A	synonymous
406829	NTHI477_RS01975	537	G	T	synonymous
406834	NTHI477_RS01975	532	T	C	nonsynonymous
406835	NTHI477_RS01975	531	C	A	synonymous
406837	NTHI477_RS01975	529	C	T	nonsynonymous
406844	NTHI477_RS01975	522	A	G	synonymous
406856	NTHI477_RS01975	510	G	A	synonymous
406871	NTHI477_RS01975	495	G	C	nonsynonymous

406879	NTHI477_RS01975	487	G	C	nonsynonymous
406879	NTHI477_RS01975	487	G	A	nonsynonymous
406899	NTHI477_RS01975	467	C	G	nonsynonymous
406933	NTHI477_RS01975	433	T	C	nonsynonymous
406934	NTHI477_RS01975	432	A	C	synonymous
406934	NTHI477_RS01975	432	A	T	synonymous
406955	NTHI477_RS01975	411	T	C	synonymous
406970	NTHI477_RS01975	396	T	C	synonymous
406979	NTHI477_RS01975	387	A	C	synonymous
407011	NTHI477_RS01975	355	G	A	synonymous
407012	NTHI477_RS01975	354	A	G	synonymous
407018	NTHI477_RS01975	348	G	A	synonymous
407036	NTHI477_RS01975	330	G	T	synonymous
407045	NTHI477_RS01975	321	G	A	synonymous
407090	NTHI477_RS01975	276	G	A	synonymous
407096	NTHI477_RS01975	270	G	A	synonymous
407099	NTHI477_RS01975	267	A	G	synonymous
407104	NTHI477_RS01975	262	A	G	nonsynonymous
407114	NTHI477_RS01975	252	T	A	synonymous
407117	NTHI477_RS01975	249	G	A	synonymous
407118	NTHI477_RS01975	248	C	T	nonsynonymous
407131	NTHI477_RS01975	235	C	T	nonsynonymous
407141	NTHI477_RS01975	225	A	G	synonymous
407142	NTHI477_RS01975	224	C	T	nonsynonymous
407150	NTHI477_RS01975	216	G	A	synonymous
407153	NTHI477_RS01975	213	C	T	synonymous
407156	NTHI477_RS01975	210	T	G	synonymous
407156	NTHI477_RS01975	210	T	C	synonymous
407171	NTHI477_RS01975	195	T	A	synonymous
407189	NTHI477_RS01975	177	A	G	synonymous
407203	NTHI477_RS01975	163	C	T	nonsynonymous
407216	NTHI477_RS01975	150	G	A	synonymous
407222	NTHI477_RS01975	144	C	A	nonsynonymous
407240	NTHI477_RS01975	126	G	A	synonymous
407273	NTHI477_RS01975	93	T	C	synonymous
407276	NTHI477_RS01975	90	A	C	synonymous
407279	NTHI477_RS01975	87	A	G	synonymous
407285	NTHI477_RS01975	81	C	A	nonsynonymous
407291	NTHI477_RS01975	75	G	C	synonymous
407297	NTHI477_RS01975	69	G	A	synonymous
407303	NTHI477_RS01975	63	G	T	synonymous
407305	NTHI477_RS01975	61	T	G	nonsynonymous
407306	NTHI477_RS01975	60	A	G	synonymous
407309	NTHI477_RS01975	57	G	A	synonymous
407318	NTHI477_RS01975	48	T	C	synonymous

407324	NTHI477_RS01975	42	G	A	synonymous
407348	NTHI477_RS01975	18	G	A	synonymous
407349	NTHI477_RS01975	17	G	A	nonsynonymous
407354	NTHI477_RS01975	12	C	A	synonymous
407354	NTHI477_RS01975	12	C	T	synonymous
407369	intergenic	intergenic	A	G	intergenic
407388	intergenic	intergenic	G	A	intergenic
407392	intergenic	intergenic	C	A	intergenic
407393	intergenic	intergenic	C	T	intergenic
407395	intergenic	intergenic	A	C	intergenic
407404	intergenic	intergenic	A	T	intergenic
407434	intergenic	intergenic	A	G	intergenic
407439	intergenic	intergenic	C	T	intergenic
407449	intergenic	intergenic	A	C	intergenic
407450	intergenic	intergenic	A	G	intergenic
407452	intergenic	intergenic	T	C	intergenic
407458	intergenic	intergenic	C	A	intergenic
407458	intergenic	intergenic	C	T	intergenic
407472	intergenic	intergenic	A	C	intergenic
407493	intergenic	intergenic	T	C	intergenic
407532	intergenic	intergenic	T	C	intergenic
407536	intergenic	intergenic	G	A	intergenic
407544	intergenic	intergenic	G	A	intergenic
407548	intergenic	intergenic	A	C	intergenic
407551	intergenic	intergenic	C	T	intergenic
407552	intergenic	intergenic	A	G	intergenic
407557	intergenic	intergenic	A	G	intergenic
407560	intergenic	intergenic	A	C	intergenic
407566	intergenic	intergenic	A	G	intergenic
407567	intergenic	intergenic	G	A	intergenic
407568	intergenic	intergenic	A	G	intergenic
407573	intergenic	intergenic	C	T	intergenic
407587	intergenic	intergenic	T	C	intergenic
407588	intergenic	intergenic	A	G	intergenic
407596	NTHI477_RS01980	2265	T	C	synonymous
407603	NTHI477_RS01980	2258	G	T	nonsynonymous
407653	NTHI477_RS01980	2208	A	G	synonymous
407671	NTHI477_RS01980	2190	C	T	synonymous
407686	NTHI477_RS01980	2175	T	A	synonymous
407707	NTHI477_RS01980	2154	G	A	synonymous
407716	NTHI477_RS01980	2145	C	G	synonymous
407719	NTHI477_RS01980	2142	A	G	synonymous
407722	NTHI477_RS01980	2139	A	T	synonymous
407722	NTHI477_RS01980	2139	A	G	synonymous
407725	NTHI477_RS01980	2136	G	T	synonymous

407727	NTHI477_RS01980	2134	C	T	nonsynonymous
407728	NTHI477_RS01980	2133	G	A	synonymous
407737	NTHI477_RS01980	2124	G	A	synonymous
407758	NTHI477_RS01980	2103	A	G	synonymous
407788	NTHI477_RS01980	2073	G	A	synonymous
407790	NTHI477_RS01980	2071	T	A	nonsynonymous
407810	NTHI477_RS01980	2051	A	G	nonsynonymous
407811	NTHI477_RS01980	2050	A	G	nonsynonymous
407815	NTHI477_RS01980	2046	T	G	nonsynonymous
407817	NTHI477_RS01980	2044	A	C	nonsynonymous
407826	NTHI477_RS01980	2035	T	G	synonymous
407830	NTHI477_RS01980	2031	T	A	synonymous
407836	NTHI477_RS01980	2025	A	G	synonymous
407851	NTHI477_RS01980	2010	T	G	synonymous
407863	NTHI477_RS01980	1998	A	C	synonymous
407869	NTHI477_RS01980	1992	G	A	synonymous
407878	NTHI477_RS01980	1983	A	G	synonymous
407881	NTHI477_RS01980	1980	T	C	synonymous
407887	NTHI477_RS01980	1974	G	A	synonymous
407888	NTHI477_RS01980	1973	C	T	nonsynonymous
407890	NTHI477_RS01980	1971	C	T	synonymous
407891	NTHI477_RS01980	1970	T	A	nonsynonymous
407896	NTHI477_RS01980	1965	G	T	synonymous
407902	NTHI477_RS01980	1959	C	T	synonymous
407908	NTHI477_RS01980	1953	T	C	synonymous
407944	NTHI477_RS01980	1917	T	C	synonymous
407959	NTHI477_RS01980	1902	A	G	synonymous
407962	NTHI477_RS01980	1899	A	T	synonymous
407968	NTHI477_RS01980	1893	C	T	synonymous
407971	NTHI477_RS01980	1890	T	C	synonymous
408073	NTHI477_RS01980	1788	T	A	synonymous
408091	NTHI477_RS01980	1770	A	T	synonymous
408091	NTHI477_RS01980	1770	A	C	synonymous
408094	NTHI477_RS01980	1767	C	A	synonymous
408099	NTHI477_RS01980	1762	A	G	synonymous
408106	NTHI477_RS01980	1755	A	G	synonymous
408118	NTHI477_RS01980	1743	A	G	synonymous
408133	NTHI477_RS01980	1728	G	A	synonymous
408136	NTHI477_RS01980	1725	G	A	synonymous
408151	NTHI477_RS01980	1710	A	G	synonymous
408157	NTHI477_RS01980	1704	T	G	synonymous
408166	NTHI477_RS01980	1695	G	T	synonymous
408211	NTHI477_RS01980	1650	G	A	synonymous
408235	NTHI477_RS01980	1626	T	G	synonymous
408253	NTHI477_RS01980	1608	A	T	synonymous

408280	NTHI477_RS01980	1581	G	A	synonymous
408283	NTHI477_RS01980	1578	T	C	synonymous
408288	NTHI477_RS01980	1573	G	A	nonsynonymous
408307	NTHI477_RS01980	1554	A	G	synonymous
408319	NTHI477_RS01980	1542	A	G	synonymous
408322	NTHI477_RS01980	1539	G	A	synonymous
408346	NTHI477_RS01980	1515	T	A	synonymous
408358	NTHI477_RS01980	1503	A	G	synonymous
408373	NTHI477_RS01980	1488	A	G	synonymous
408379	NTHI477_RS01980	1482	T	C	synonymous
408397	NTHI477_RS01980	1464	T	C	synonymous
408419	NTHI477_RS01980	1442	T	C	nonsynonymous
408430	NTHI477_RS01980	1431	G	A	synonymous
408465	NTHI477_RS01980	1396	C	T	nonsynonymous
408484	NTHI477_RS01980	1377	G	A	synonymous
408487	NTHI477_RS01980	1374	G	A	synonymous
408490	NTHI477_RS01980	1371	A	G	synonymous
408493	NTHI477_RS01980	1368	T	C	synonymous
408543	NTHI477_RS01980	1318	A	C	nonsynonymous
408556	NTHI477_RS01980	1305	G	A	synonymous
408565	NTHI477_RS01980	1296	A	G	synonymous
408578	NTHI477_RS01980	1283	C	T	nonsynonymous
408580	NTHI477_RS01980	1281	C	A	synonymous
408580	NTHI477_RS01980	1281	C	G	synonymous
408592	NTHI477_RS01980	1269	G	A	synonymous
408606	NTHI477_RS01980	1255	T	G	nonsynonymous
408607	NTHI477_RS01980	1254	T	G	nonsynonymous
408612	NTHI477_RS01980	1249	C	T	nonsynonymous
408617	NTHI477_RS01980	1244	G	A	nonsynonymous
408618	NTHI477_RS01980	1243	C	T	nonsynonymous
408634	NTHI477_RS01980	1227	T	C	synonymous
408637	NTHI477_RS01980	1224	A	G	synonymous
408649	NTHI477_RS01980	1212	A	G	synonymous
408655	NTHI477_RS01980	1206	T	C	synonymous
408661	NTHI477_RS01980	1200	T	C	synonymous
408697	NTHI477_RS01980	1164	C	T	synonymous
408709	NTHI477_RS01980	1152	A	G	synonymous
408734	NTHI477_RS01980	1127	G	C	nonsynonymous
408742	NTHI477_RS01980	1119	T	A	synonymous
408745	NTHI477_RS01980	1116	C	T	synonymous
408754	NTHI477_RS01980	1107	G	T	synonymous
408759	NTHI477_RS01980	1102	C	T	nonsynonymous
408769	NTHI477_RS01980	1092	G	A	synonymous
408775	NTHI477_RS01980	1086	C	A	synonymous
408826	NTHI477_RS01980	1035	A	G	synonymous

408856	NTHI477_RS01980	1005	G	T	synonymous
408870	NTHI477_RS01980	991	C	A	nonsynonymous
408871	NTHI477_RS01980	990	A	G	synonymous
408883	NTHI477_RS01980	978	A	G	synonymous
408901	NTHI477_RS01980	960	G	A	synonymous
408913	NTHI477_RS01980	948	G	A	synonymous
408919	NTHI477_RS01980	942	G	A	synonymous
408925	NTHI477_RS01980	936	G	A	synonymous
408928	NTHI477_RS01980	933	G	A	synonymous
408955	NTHI477_RS01980	906	A	G	synonymous
408961	NTHI477_RS01980	900	A	C	synonymous
408961	NTHI477_RS01980	900	A	T	synonymous
408967	NTHI477_RS01980	894	T	C	synonymous
408976	NTHI477_RS01980	885	C	T	synonymous
408997	NTHI477_RS01980	864	A	G	synonymous
409024	NTHI477_RS01980	837	C	T	synonymous
409033	NTHI477_RS01980	828	A	G	synonymous
409034	NTHI477_RS01980	827	C	G	nonsynonymous
409051	NTHI477_RS01980	810	C	T	synonymous
409063	NTHI477_RS01980	798	A	C	synonymous
409099	NTHI477_RS01980	762	A	C	nonsynonymous
409108	NTHI477_RS01980	753	A	G	synonymous
409115	NTHI477_RS01980	746	T	G	nonsynonymous
409132	NTHI477_RS01980	729	T	C	synonymous
409144	NTHI477_RS01980	717	A	C	nonsynonymous
409166	NTHI477_RS01980	695	T	C	nonsynonymous
409168	NTHI477_RS01980	693	G	T	nonsynonymous
409177	NTHI477_RS01980	684	A	G	synonymous
409178	NTHI477_RS01980	683	T	C	nonsynonymous
409192	NTHI477_RS01980	669	G	A	synonymous
409204	NTHI477_RS01980	657	G	A	synonymous
409210	NTHI477_RS01980	651	T	C	synonymous
409216	NTHI477_RS01980	645	A	G	synonymous
409231	NTHI477_RS01980	630	G	A	synonymous
409243	NTHI477_RS01980	618	T	C	synonymous
409255	NTHI477_RS01980	606	A	G	synonymous
409264	NTHI477_RS01980	597	A	T	synonymous
409267	NTHI477_RS01980	594	T	A	synonymous
409270	NTHI477_RS01980	591	A	T	synonymous
409270	NTHI477_RS01980	591	A	C	synonymous
409288	NTHI477_RS01980	573	C	A	synonymous
409297	NTHI477_RS01980	564	T	G	synonymous
409297	NTHI477_RS01980	564	T	A	synonymous
409318	NTHI477_RS01980	543	T	C	synonymous
409324	NTHI477_RS01980	537	A	T	synonymous

409326	NTHI477_RS01980	535	G	T	nonsynonymous
409330	NTHI477_RS01980	531	A	T	synonymous
409333	NTHI477_RS01980	528	A	G	synonymous
409336	NTHI477_RS01980	525	G	A	synonymous
409339	NTHI477_RS01980	522	G	A	synonymous
409345	NTHI477_RS01980	516	A	T	synonymous
409345	NTHI477_RS01980	516	A	G	synonymous
409348	NTHI477_RS01980	513	A	C	synonymous
409351	NTHI477_RS01980	510	A	G	synonymous
409354	NTHI477_RS01980	507	G	A	synonymous
409366	NTHI477_RS01980	495	G	A	synonymous
409366	NTHI477_RS01980	495	G	T	synonymous
409369	NTHI477_RS01980	492	A	G	synonymous
409372	NTHI477_RS01980	489	T	C	synonymous
409375	NTHI477_RS01980	486	G	A	synonymous
409381	NTHI477_RS01980	480	A	G	synonymous
409390	NTHI477_RS01980	471	A	T	synonymous
409396	NTHI477_RS01980	465	A	G	synonymous
409404	NTHI477_RS01980	457	G	T	synonymous
409423	NTHI477_RS01980	438	G	A	synonymous
409432	NTHI477_RS01980	429	G	A	synonymous
409438	NTHI477_RS01980	423	A	T	synonymous
409462	NTHI477_RS01980	399	T	C	synonymous
409465	NTHI477_RS01980	396	C	T	synonymous
409480	NTHI477_RS01980	381	C	T	synonymous
409501	NTHI477_RS01980	360	C	A	synonymous
409501	NTHI477_RS01980	360	C	T	synonymous
409521	NTHI477_RS01980	340	A	G	nonsynonymous
409522	NTHI477_RS01980	339	C	T	synonymous
409543	NTHI477_RS01980	318	T	C	synonymous
409549	NTHI477_RS01980	312	G	A	synonymous
409555	NTHI477_RS01980	306	C	T	synonymous
409570	NTHI477_RS01980	291	C	T	synonymous
409573	NTHI477_RS01980	288	C	T	synonymous
409582	NTHI477_RS01980	279	T	C	synonymous
409594	NTHI477_RS01980	267	G	A	synonymous
409603	NTHI477_RS01980	258	C	A	synonymous
409603	NTHI477_RS01980	258	C	G	synonymous
409606	NTHI477_RS01980	255	T	A	synonymous
409609	NTHI477_RS01980	252	T	C	synonymous
409612	NTHI477_RS01980	249	G	A	synonymous
409627	NTHI477_RS01980	234	C	T	synonymous
409633	NTHI477_RS01980	228	T	C	synonymous
409642	NTHI477_RS01980	219	G	A	synonymous
409648	NTHI477_RS01980	213	G	A	synonymous

409654	NTHI477_RS01980	207	C	A	synonymous
409657	NTHI477_RS01980	204	C	A	synonymous
409660	NTHI477_RS01980	201	C	T	synonymous
409660	NTHI477_RS01980	201	C	A	synonymous
409669	NTHI477_RS01980	192	G	A	synonymous
409675	NTHI477_RS01980	186	T	C	synonymous
409678	NTHI477_RS01980	183	T	G	synonymous
409678	NTHI477_RS01980	183	T	C	synonymous
409693	NTHI477_RS01980	168	G	A	synonymous
409696	NTHI477_RS01980	165	G	T	synonymous
409697	NTHI477_RS01980	164	G	A	nonsynonymous
409738	NTHI477_RS01980	123	C	T	synonymous
409741	NTHI477_RS01980	120	T	C	synonymous
409753	NTHI477_RS01980	108	G	A	synonymous
409756	NTHI477_RS01980	105	A	T	synonymous
409756	NTHI477_RS01980	105	A	C	synonymous
409758	NTHI477_RS01980	103	G	A	nonsynonymous
409762	NTHI477_RS01980	99	A	G	synonymous
409768	NTHI477_RS01980	93	G	T	synonymous
409774	NTHI477_RS01980	87	T	G	nonsynonymous
409776	NTHI477_RS01980	85	A	G	synonymous
409809	NTHI477_RS01980	52	G	A	nonsynonymous
409831	NTHI477_RS01980	30	T	C	synonymous
409856	NTHI477_RS01980	5	G	C	nonsynonymous
409866	intergenic	intergenic	A	G	intergenic
409898	intergenic	intergenic	T	C	intergenic
409949	intergenic	intergenic	T	C	intergenic
409951	intergenic	intergenic	T	C	intergenic
409953	intergenic	intergenic	C	T	intergenic
409983	intergenic	intergenic	G	A	intergenic
409985	intergenic	intergenic	G	A	intergenic
410013	intergenic	intergenic	C	T	intergenic
410042	intergenic	intergenic	C	G	intergenic
410047	intergenic	intergenic	A	G	intergenic
410050	intergenic	intergenic	A	G	intergenic
410052	intergenic	intergenic	G	A	intergenic
410078	NTHI477_RS01985	21	T	C	synonymous
410111	NTHI477_RS01985	54	C	T	synonymous
410114	NTHI477_RS01985	57	T	C	synonymous
410120	NTHI477_RS01985	63	C	T	synonymous
410121	NTHI477_RS01985	64	A	T	nonsynonymous
410123	NTHI477_RS01985	66	T	A	synonymous
410124	NTHI477_RS01985	67	C	T	synonymous
410126	NTHI477_RS01985	69	G	A	synonymous
410131	NTHI477_RS01985	74	G	A	nonsynonymous

410136	NTHI477_RS01985	79	C	T	synonymous
410138	NTHI477_RS01985	81	A	G	synonymous
410141	NTHI477_RS01985	84	A	T	synonymous
410144	NTHI477_RS01985	87	T	C	synonymous
410150	NTHI477_RS01985	93	T	C	synonymous
410153	NTHI477_RS01985	96	G	A	nonsynonymous
410163	NTHI477_RS01985	106	G	T	nonsynonymous
410174	NTHI477_RS01985	117	T	C	synonymous
410175	NTHI477_RS01985	118	A	G	nonsynonymous
410180	NTHI477_RS01985	123	T	G	synonymous
410189	NTHI477_RS01985	132	C	G	nonsynonymous
410195	NTHI477_RS01985	138	T	C	synonymous
410202	NTHI477_RS01985	145	C	T	nonsynonymous
410203	NTHI477_RS01985	146	A	G	nonsynonymous
410204	NTHI477_RS01985	147	C	T	synonymous
410219	NTHI477_RS01985	162	C	G	synonymous
410228	NTHI477_RS01985	171	G	A	synonymous
410236	NTHI477_RS01985	179	A	G	nonsynonymous
410276	NTHI477_RS01985	219	G	A	nonsynonymous
410285	NTHI477_RS01985	228	C	T	synonymous
410287	NTHI477_RS01985	230	G	T	nonsynonymous
410288	NTHI477_RS01985	231	T	C	synonymous
410290	NTHI477_RS01985	233	C	A	nonsynonymous
410298	NTHI477_RS01985	241	C	A	nonsynonymous
410300	NTHI477_RS01985	243	C	T	synonymous
410306	NTHI477_RS01985	249	C	T	synonymous
410309	NTHI477_RS01985	252	A	G	synonymous
410315	NTHI477_RS01985	258	A	G	synonymous
410330	NTHI477_RS01985	273	A	C	synonymous
410333	NTHI477_RS01985	276	G	A	synonymous
410345	NTHI477_RS01985	288	C	G	synonymous
410354	NTHI477_RS01985	297	A	C	nonsynonymous
410369	NTHI477_RS01985	312	T	C	synonymous
410375	NTHI477_RS01985	318	C	T	synonymous
410378	NTHI477_RS01985	321	T	C	synonymous
410390	NTHI477_RS01985	333	C	T	synonymous
410415	NTHI477_RS01985	358	C	T	nonsynonymous
410426	NTHI477_RS01985	369	G	T	nonsynonymous
410429	NTHI477_RS01985	372	A	C	nonsynonymous
410429	NTHI477_RS01985	372	A	T	nonsynonymous
410432	NTHI477_RS01985	375	C	T	synonymous
410451	NTHI477_RS01985	394	A	G	nonsynonymous
410465	NTHI477_RS01985	408	C	T	synonymous
410507	NTHI477_RS01985	450	C	T	synonymous
410519	NTHI477_RS01985	462	G	A	synonymous

410540	NTHI477_RS01985	483	T	C	synonymous
410543	NTHI477_RS01985	486	A	T	synonymous
410555	NTHI477_RS01985	498	G	A	synonymous
410560	NTHI477_RS01985	503	A	G	nonsynonymous
410576	NTHI477_RS01985	519	C	T	synonymous
410591	NTHI477_RS01985	534	G	A	synonymous
410594	NTHI477_RS01985	537	G	A	synonymous
410615	NTHI477_RS01985	558	T	A	synonymous
410618	NTHI477_RS01985	561	C	A	synonymous
410627	NTHI477_RS01985	570	G	A	synonymous
410648	NTHI477_RS01985	591	C	T	synonymous
410655	NTHI477_RS01985	598	G	C	nonsynonymous
410669	NTHI477_RS01985	612	T	C	synonymous
410672	NTHI477_RS01985	615	A	C	nonsynonymous
410673	NTHI477_RS01985	616	T	G	nonsynonymous
410673	NTHI477_RS01985	616	T	C	nonsynonymous
410679	NTHI477_RS01985	622	C	T	nonsynonymous
410685	intergenic	intergenic	G	A	intergenic
410700	intergenic	intergenic	A	T	intergenic
410721	intergenic	intergenic	A	C	intergenic
410740	intergenic	intergenic	A	C	intergenic
410747	intergenic	intergenic	T	C	intergenic
410760	intergenic	intergenic	C	T	intergenic
410804	NTHI477_RS01990	31	A	G	nonsynonymous
410805	NTHI477_RS01990	32	C	T	nonsynonymous
410808	NTHI477_RS01990	35	G	A	nonsynonymous
410809	NTHI477_RS01990	36	C	A	synonymous
410815	NTHI477_RS01990	42	G	T	synonymous
410828	NTHI477_RS01990	55	A	G	nonsynonymous
410830	NTHI477_RS01990	57	A	G	synonymous
410836	NTHI477_RS01990	63	G	A	synonymous
410842	NTHI477_RS01990	69	C	T	synonymous
410866	NTHI477_RS01990	93	T	C	synonymous
410875	NTHI477_RS01990	102	C	T	synonymous
410884	NTHI477_RS01990	111	T	C	synonymous
410885	NTHI477_RS01990	112	G	T	nonsynonymous
410887	NTHI477_RS01990	114	G	A	synonymous
410890	NTHI477_RS01990	117	T	A	synonymous
410908	NTHI477_RS01990	135	T	C	synonymous
410909	NTHI477_RS01990	136	A	G	nonsynonymous
410914	NTHI477_RS01990	141	C	T	synonymous
410937	NTHI477_RS01990	164	C	G	nonsynonymous
410938	NTHI477_RS01990	165	T	G	synonymous
410942	NTHI477_RS01990	169	G	A	nonsynonymous
410944	NTHI477_RS01990	171	A	G	synonymous

410944	NTHI477_RS01990	171	A	C	synonymous
410953	NTHI477_RS01990	180	C	T	synonymous
410969	NTHI477_RS01990	196	C	A	nonsynonymous
410974	NTHI477_RS01990	201	G	A	synonymous
411019	NTHI477_RS01990	246	A	T	synonymous
411034	NTHI477_RS01990	261	T	C	synonymous
411046	NTHI477_RS01990	273	A	G	synonymous
411049	NTHI477_RS01990	276	T	G	synonymous
411052	NTHI477_RS01990	279	A	C	synonymous
411067	NTHI477_RS01990	294	A	G	synonymous
411088	NTHI477_RS01990	315	G	A	synonymous
411095	NTHI477_RS01990	322	T	C	nonsynonymous
411109	NTHI477_RS01990	336	C	T	synonymous
411133	NTHI477_RS01990	360	C	T	synonymous
411136	NTHI477_RS01990	363	C	T	synonymous
411142	NTHI477_RS01990	369	G	C	synonymous
411145	NTHI477_RS01990	372	A	C	synonymous
411148	NTHI477_RS01990	375	A	G	synonymous
411166	NTHI477_RS01990	393	C	A	synonymous
411175	NTHI477_RS01990	402	A	G	synonymous
411178	NTHI477_RS01990	405	A	T	synonymous
411184	NTHI477_RS01990	411	T	A	synonymous
411190	NTHI477_RS01990	417	C	T	synonymous
411208	NTHI477_RS01990	435	A	T	synonymous
411208	NTHI477_RS01990	435	A	C	synonymous
411209	NTHI477_RS01990	436	G	A	nonsynonymous
411211	NTHI477_RS01990	438	G	A	synonymous
411218	NTHI477_RS01990	445	T	G	nonsynonymous
411250	NTHI477_RS01990	477	G	A	synonymous
411254	NTHI477_RS01990	481	A	T	nonsynonymous
411286	NTHI477_RS01990	513	T	C	synonymous
411295	NTHI477_RS01990	522	G	A	synonymous
411301	NTHI477_RS01990	528	G	A	synonymous
411310	NTHI477_RS01990	537	T	A	synonymous
411367	NTHI477_RS01990	594	T	C	synonymous
411392	NTHI477_RS01990	619	G	A	nonsynonymous
411407	NTHI477_RS01990	634	G	C	nonsynonymous
411418	NTHI477_RS01990	645	A	T	nonsynonymous
411424	NTHI477_RS01990	651	C	G	synonymous
411439	NTHI477_RS01990	666	A	G	synonymous
411448	NTHI477_RS01990	675	T	C	synonymous
411459	NTHI477_RS01990	686	A	G	nonsynonymous
411460	NTHI477_RS01990	687	G	T	nonsynonymous
411463	NTHI477_RS01990	690	A	G	synonymous
411518	NTHI477_RS01995	45	T	C	synonymous

411531	NTHI477_RS01995	58	C	T	synonymous
411539	NTHI477_RS01995	66	C	T	synonymous
411581	NTHI477_RS01995	108	G	A	synonymous
411584	NTHI477_RS01995	111	A	G	synonymous
411592	NTHI477_RS01995	119	G	A	nonsynonymous
411596	NTHI477_RS01995	123	G	A	synonymous
411626	NTHI477_RS01995	153	C	T	synonymous
411635	NTHI477_RS01995	162	T	C	synonymous
411638	NTHI477_RS01995	165	T	C	synonymous
411641	NTHI477_RS01995	168	C	T	synonymous
411644	NTHI477_RS01995	171	G	A	nonsynonymous
411647	NTHI477_RS01995	174	G	A	synonymous
411650	NTHI477_RS01995	177	T	C	synonymous
411662	NTHI477_RS01995	189	T	C	synonymous
411677	NTHI477_RS01995	204	T	C	synonymous
411683	NTHI477_RS01995	210	T	C	synonymous
411686	NTHI477_RS01995	213	C	T	synonymous
411716	NTHI477_RS01995	243	T	C	synonymous
411722	NTHI477_RS01995	249	G	A	synonymous
411725	NTHI477_RS01995	252	T	C	synonymous
411728	NTHI477_RS01995	255	A	G	synonymous
411729	NTHI477_RS01995	256	T	C	synonymous
411743	NTHI477_RS01995	270	G	A	synonymous
411746	NTHI477_RS01995	273	T	C	synonymous
411773	NTHI477_RS01995	300	T	C	synonymous
411815	NTHI477_RS01995	342	T	A	synonymous
411818	NTHI477_RS01995	345	A	T	synonymous
411821	NTHI477_RS01995	348	A	G	synonymous
411827	NTHI477_RS01995	354	T	A	synonymous
411839	NTHI477_RS01995	366	G	A	synonymous
411887	NTHI477_RS01995	414	T	C	synonymous
411887	NTHI477_RS01995	414	T	A	synonymous
411890	NTHI477_RS01995	417	G	C	synonymous
411896	NTHI477_RS01995	423	G	A	synonymous
411908	NTHI477_RS01995	435	G	A	synonymous
411920	NTHI477_RS01995	447	G	A	synonymous
411926	NTHI477_RS01995	453	T	C	synonymous
411932	NTHI477_RS01995	459	G	T	synonymous
411944	NTHI477_RS01995	471	T	C	synonymous
411947	NTHI477_RS01995	474	A	G	synonymous
411953	NTHI477_RS01995	480	C	T	synonymous
411956	NTHI477_RS01995	483	T	C	synonymous
411962	NTHI477_RS01995	489	T	C	synonymous
411965	NTHI477_RS01995	492	G	A	synonymous
411977	NTHI477_RS01995	504	T	C	synonymous

411983	NTHI477_RS01995	510	G	A	synonymous
411986	NTHI477_RS01995	513	T	C	synonymous
411986	NTHI477_RS01995	513	T	G	synonymous
411995	NTHI477_RS01995	522	A	G	synonymous
411996	NTHI477_RS01995	523	T	C	synonymous
412005	NTHI477_RS01995	532	G	A	nonsynonymous
412058	NTHI477_RS01995	585	G	T	synonymous
412064	NTHI477_RS01995	591	C	A	synonymous
412103	NTHI477_RS01995	630	G	A	synonymous
412118	NTHI477_RS01995	645	T	A	synonymous
412121	NTHI477_RS01995	648	C	T	synonymous
412124	NTHI477_RS01995	651	T	C	synonymous
412134	NTHI477_RS01995	661	G	A	nonsynonymous
412139	NTHI477_RS01995	666	T	C	synonymous
412143	NTHI477_RS01995	670	A	G	nonsynonymous
412151	NTHI477_RS01995	678	C	T	synonymous
412154	NTHI477_RS01995	681	G	A	synonymous
412157	NTHI477_RS01995	684	G	A	synonymous
412161	NTHI477_RS01995	688	G	A	nonsynonymous
412163	NTHI477_RS01995	690	T	C	synonymous
412169	NTHI477_RS01995	696	G	C	synonymous
412172	NTHI477_RS01995	699	G	C	synonymous
412172	NTHI477_RS01995	699	G	T	synonymous
412178	NTHI477_RS01995	705	A	C	synonymous
412181	NTHI477_RS01995	708	A	G	synonymous
412182	NTHI477_RS01995	709	A	G	nonsynonymous
412187	NTHI477_RS01995	714	C	A	synonymous
412205	NTHI477_RS01995	732	C	G	synonymous
412206	NTHI477_RS01995	733	G	A	nonsynonymous
412211	NTHI477_RS01995	738	C	T	synonymous
412211	NTHI477_RS01995	738	C	A	synonymous
412217	NTHI477_RS01995	744	T	A	nonsynonymous
412229	NTHI477_RS01995	756	T	C	synonymous
412248	NTHI477_RS01995	775	G	A	nonsynonymous
412265	NTHI477_RS01995	792	C	T	synonymous
412268	NTHI477_RS01995	795	G	C	synonymous
412274	NTHI477_RS01995	801	A	G	synonymous
412279	NTHI477_RS01995	806	G	C	nonsynonymous
412289	NTHI477_RS01995	816	T	C	synonymous
412292	NTHI477_RS01995	819	T	C	synonymous
412296	NTHI477_RS01995	823	G	A	nonsynonymous
412298	NTHI477_RS01995	825	G	T	synonymous
412301	NTHI477_RS01995	828	T	C	synonymous
412313	NTHI477_RS01995	840	T	C	synonymous
412318	NTHI477_RS01995	845	G	T	nonsynonymous

412339	NTHI477_RS01995	866	C	T	nonsynonymous
412340	NTHI477_RS01995	867	C	A	synonymous
412345	NTHI477_RS01995	872	T	A	nonsynonymous
412381	NTHI477_RS01995	908	T	C	nonsynonymous
412384	NTHI477_RS01995	911	T	C	nonsynonymous
412391	NTHI477_RS01995	918	C	T	synonymous
412393	NTHI477_RS01995	920	A	T	nonsynonymous
412408	NTHI477_RS01995	935	C	T	nonsynonymous
412412	NTHI477_RS01995	939	T	C	synonymous
412418	NTHI477_RS01995	945	G	A	synonymous
412432	NTHI477_RS01995	959	A	T	nonsynonymous
412432	NTHI477_RS01995	959	A	C	nonsynonymous
412448	NTHI477_RS01995	975	G	T	nonsynonymous
412470	NTHI477_RS01995	997	A	G	nonsynonymous
412489	NTHI477_RS01995	1016	A	G	nonsynonymous
412492	NTHI477_RS01995	1019	A	T	nonsynonymous
412516	NTHI477_RS01995	1043	A	G	nonsynonymous
412528	NTHI477_RS01995	1055	G	A	nonsynonymous
412537	NTHI477_RS01995	1064	T	G	nonsynonymous
412552	NTHI477_RS01995	1079	C	T	nonsynonymous
412555	NTHI477_RS01995	1082	G	A	nonsynonymous
412573	NTHI477_RS01995	1100	T	C	nonsynonymous
412576	NTHI477_RS01995	1103	C	T	nonsynonymous
412579	NTHI477_RS01995	1106	A	C	nonsynonymous
412600	NTHI477_RS01995	1127	G	A	nonsynonymous
412611	NTHI477_RS01995	1138	T	C	nonsynonymous
412621	NTHI477_RS01995	1148	G	A	nonsynonymous
412624	NTHI477_RS01995	1151	G	A	nonsynonymous
412625	NTHI477_RS01995	1152	G	T	nonsynonymous
412627	NTHI477_RS01995	1154	G	C	nonsynonymous
412633	NTHI477_RS01995	1160	C	T	nonsynonymous
412644	NTHI477_RS01995	1171	G	A	nonsynonymous
412652	NTHI477_RS01995	1179	C	T	synonymous
412654	NTHI477_RS01995	1181	T	A	nonsynonymous
412681	intergenic	intergenic	C	T	intergenic
412682	intergenic	intergenic	G	T	intergenic
412705	NTHI477_RS02000	797	A	G	nonsynonymous
412707	NTHI477_RS02000	795	A	G	synonymous
412712	NTHI477_RS02000	790	C	T	nonsynonymous
412728	NTHI477_RS02000	774	G	A	synonymous
412764	NTHI477_RS02000	738	G	A	synonymous
412766	NTHI477_RS02000	736	T	C	nonsynonymous
412774	NTHI477_RS02000	728	A	T	nonsynonymous
412774	NTHI477_RS02000	728	A	G	nonsynonymous
412802	NTHI477_RS02000	700	G	A	synonymous

412845	NTHI477_RS02000	657	G	A	synonymous
412848	NTHI477_RS02000	654	A	G	synonymous
412853	NTHI477_RS02000	649	C	T	nonsynonymous
412857	NTHI477_RS02000	645	G	C	nonsynonymous
412881	NTHI477_RS02000	621	A	G	synonymous
412902	NTHI477_RS02000	600	A	G	synonymous
412922	NTHI477_RS02000	580	G	A	synonymous
412926	NTHI477_RS02000	576	A	G	synonymous
412938	NTHI477_RS02000	564	C	T	nonsynonymous
412941	NTHI477_RS02000	561	T	G	synonymous
412947	NTHI477_RS02000	555	G	A	synonymous
412948	NTHI477_RS02000	554	G	A	nonsynonymous
412971	NTHI477_RS02000	531	T	C	synonymous
412995	NTHI477_RS02000	507	C	T	synonymous
412996	NTHI477_RS02000	506	G	A	nonsynonymous
413007	NTHI477_RS02000	495	T	C	synonymous
413024	NTHI477_RS02000	478	A	G	synonymous
413028	NTHI477_RS02000	474	T	C	synonymous
413031	NTHI477_RS02000	471	T	C	synonymous
413034	NTHI477_RS02000	468	A	G	synonymous
413037	NTHI477_RS02000	465	A	C	synonymous
413055	NTHI477_RS02000	447	A	G	synonymous
413056	NTHI477_RS02000	446	T	G	nonsynonymous
413058	NTHI477_RS02000	444	A	C	nonsynonymous
413089	NTHI477_RS02000	413	T	G	nonsynonymous
413097	NTHI477_RS02000	405	G	A	synonymous
413136	NTHI477_RS02000	366	G	A	synonymous
413145	NTHI477_RS02000	357	A	G	synonymous
413202	NTHI477_RS02000	300	G	A	synonymous
413208	NTHI477_RS02000	294	T	C	synonymous
413232	NTHI477_RS02000	270	T	C	synonymous
413256	NTHI477_RS02000	246	C	A	synonymous
413258	NTHI477_RS02000	244	C	G	nonsynonymous
413259	NTHI477_RS02000	243	T	C	synonymous
413271	NTHI477_RS02000	231	A	G	synonymous
413282	NTHI477_RS02000	220	T	G	nonsynonymous
413286	NTHI477_RS02000	216	C	A	synonymous
413303	NTHI477_RS02000	199	C	T	nonsynonymous
413307	NTHI477_RS02000	195	T	C	synonymous
413310	NTHI477_RS02000	192	T	C	synonymous
413328	NTHI477_RS02000	174	G	A	synonymous
413329	NTHI477_RS02000	173	C	A	nonsynonymous
413337	NTHI477_RS02000	165	C	A	synonymous
413343	NTHI477_RS02000	159	C	T	synonymous
413346	NTHI477_RS02000	156	C	A	synonymous

413448	NTHI477_RS02000	54	G	A	synonymous
413465	NTHI477_RS02000	37	G	A	nonsynonymous
413472	NTHI477_RS02000	30	T	C	synonymous
413473	NTHI477_RS02000	29	G	A	nonsynonymous
413512	intergenic	intergenic	T	A	intergenic
413526	intergenic	intergenic	G	A	intergenic
413528	intergenic	intergenic	G	A	intergenic
413529	intergenic	intergenic	T	C	intergenic
413563	intergenic	intergenic	A	G	intergenic
413570	intergenic	intergenic	A	G	intergenic
413610	NTHI477_RS02005	12	T	C	synonymous
413634	NTHI477_RS02005	36	C	T	synonymous
413658	NTHI477_RS02005	60	T	C	synonymous
413661	NTHI477_RS02005	63	G	C	synonymous
413664	NTHI477_RS02005	66	A	T	synonymous
413673	NTHI477_RS02005	75	G	A	synonymous
413697	NTHI477_RS02005	99	C	T	synonymous
413709	NTHI477_RS02005	111	T	C	synonymous
413712	NTHI477_RS02005	114	G	C	synonymous
413724	NTHI477_RS02005	126	T	C	synonymous
413727	NTHI477_RS02005	129	C	T	synonymous
413730	NTHI477_RS02005	132	C	T	synonymous
413751	NTHI477_RS02005	153	C	T	synonymous
413754	NTHI477_RS02005	156	A	G	synonymous
413760	NTHI477_RS02005	162	C	T	synonymous
413766	NTHI477_RS02005	168	C	A	synonymous
413802	NTHI477_RS02005	204	G	A	synonymous
413811	NTHI477_RS02005	213	G	A	synonymous
413817	NTHI477_RS02005	219	A	G	synonymous
413835	NTHI477_RS02005	237	A	G	synonymous
413841	NTHI477_RS02005	243	T	C	synonymous
413858	NTHI477_RS02005	260	G	A	nonsynonymous
413886	NTHI477_RS02005	288	T	C	synonymous
413898	NTHI477_RS02005	300	C	T	synonymous
413922	NTHI477_RS02005	324	C	T	synonymous
413934	NTHI477_RS02005	336	A	G	synonymous
413950	NTHI477_RS02005	352	A	G	nonsynonymous
413952	NTHI477_RS02005	354	C	T	synonymous
413955	NTHI477_RS02005	357	T	C	synonymous
413958	NTHI477_RS02005	360	C	T	synonymous
413973	NTHI477_RS02005	375	A	T	synonymous
413976	NTHI477_RS02005	378	C	T	synonymous
413985	NTHI477_RS02005	387	T	C	synonymous
413985	NTHI477_RS02005	387	T	G	synonymous
414000	NTHI477_RS02005	402	G	A	synonymous

414039	NTHI477_RS02005	441	G	A	synonymous
414042	NTHI477_RS02005	444	C	T	synonymous
414045	NTHI477_RS02005	447	A	G	synonymous
414057	NTHI477_RS02005	459	T	C	synonymous
414060	NTHI477_RS02005	462	C	T	synonymous
414070	NTHI477_RS02005	472	C	T	synonymous
414102	NTHI477_RS02005	504	T	C	synonymous
414123	NTHI477_RS02005	525	A	G	synonymous
414129	NTHI477_RS02005	531	C	A	synonymous
414132	NTHI477_RS02005	534	G	A	synonymous
414141	NTHI477_RS02005	543	A	G	synonymous
414150	NTHI477_RS02005	552	C	T	synonymous
414153	NTHI477_RS02005	555	C	T	synonymous
414165	NTHI477_RS02005	567	C	T	synonymous
414166	NTHI477_RS02005	568	C	A	nonsynonymous
414192	NTHI477_RS02005	594	G	T	synonymous
414195	NTHI477_RS02005	597	G	A	synonymous
414198	NTHI477_RS02005	600	G	A	synonymous
414198	NTHI477_RS02005	600	G	C	synonymous
414201	NTHI477_RS02005	603	G	A	synonymous
414225	NTHI477_RS02005	627	G	A	synonymous
414240	NTHI477_RS02005	642	G	A	synonymous
414274	intergenic	intergenic	G	A	intergenic
414317	NTHI477_RS02010	36	T	A	synonymous
414317	NTHI477_RS02010	36	T	G	synonymous
414329	NTHI477_RS02010	48	G	A	synonymous
414357	NTHI477_RS02010	76	T	C	nonsynonymous
414374	NTHI477_RS02010	93	T	A	synonymous
414387	NTHI477_RS02010	106	C	G	nonsynonymous
414401	NTHI477_RS02010	120	G	A	synonymous
414440	NTHI477_RS02010	159	T	C	synonymous
414451	NTHI477_RS02010	170	G	A	nonsynonymous
414458	NTHI477_RS02010	177	G	C	synonymous
414464	NTHI477_RS02010	183	C	T	synonymous
414467	NTHI477_RS02010	186	G	C	nonsynonymous
414477	NTHI477_RS02010	196	T	C	synonymous
414488	NTHI477_RS02010	207	A	G	synonymous
414494	NTHI477_RS02010	213	A	G	synonymous
414500	NTHI477_RS02010	219	A	G	synonymous
414504	NTHI477_RS02010	223	A	G	nonsynonymous
414519	NTHI477_RS02010	238	G	A	nonsynonymous
414527	NTHI477_RS02010	246	C	T	synonymous
414536	NTHI477_RS02010	255	T	A	synonymous
414539	NTHI477_RS02010	258	A	T	synonymous
414548	NTHI477_RS02010	267	C	T	synonymous

414566	NTHI477_RS02010	285	C	T	synonymous
414596	NTHI477_RS02010	315	T	C	synonymous
414599	NTHI477_RS02010	318	A	G	synonymous
414603	NTHI477_RS02010	322	G	A	nonsynonymous
414611	NTHI477_RS02010	330	T	G	synonymous
414620	NTHI477_RS02010	339	C	T	synonymous
414629	NTHI477_RS02010	348	T	C	synonymous
414656	NTHI477_RS02010	375	G	A	synonymous
414689	NTHI477_RS02010	408	T	C	synonymous
414689	NTHI477_RS02010	408	T	G	synonymous
414692	NTHI477_RS02010	411	C	T	synonymous
414698	NTHI477_RS02010	417	G	A	synonymous
414698	NTHI477_RS02010	417	G	C	synonymous
414710	NTHI477_RS02010	429	C	T	synonymous
414713	NTHI477_RS02010	432	C	T	synonymous
414719	NTHI477_RS02010	438	A	G	synonymous
414725	NTHI477_RS02010	444	A	G	synonymous
414728	NTHI477_RS02010	447	A	G	synonymous
414734	NTHI477_RS02010	453	T	C	synonymous
414740	NTHI477_RS02010	459	G	A	synonymous
414743	NTHI477_RS02010	462	T	C	synonymous
414744	NTHI477_RS02010	463	T	C	synonymous
414758	NTHI477_RS02010	477	A	G	synonymous
414770	NTHI477_RS02010	489	A	G	synonymous
414797	NTHI477_RS02010	516	G	T	synonymous
414800	NTHI477_RS02010	519	G	A	synonymous
414851	NTHI477_RS02010	570	G	A	synonymous
414863	NTHI477_RS02010	582	G	A	synonymous
414887	NTHI477_RS02010	606	T	A	synonymous
414888	NTHI477_RS02010	607	T	G	nonsynonymous
414892	NTHI477_RS02010	611	G	A	nonsynonymous
414920	NTHI477_RS02010	639	G	A	synonymous
414920	NTHI477_RS02010	639	G	T	synonymous
414923	NTHI477_RS02010	642	T	G	synonymous
414929	NTHI477_RS02010	648	T	A	synonymous
414938	NTHI477_RS02010	657	G	A	synonymous
414944	NTHI477_RS02010	663	T	C	synonymous
414956	NTHI477_RS02010	675	T	C	synonymous
414972	NTHI477_RS02010	691	A	G	nonsynonymous
414995	NTHI477_RS02010	714	G	A	synonymous
414996	NTHI477_RS02010	715	A	G	nonsynonymous
415013	NTHI477_RS02010	732	C	T	synonymous
415016	NTHI477_RS02010	735	C	T	synonymous
415022	NTHI477_RS02010	741	C	A	synonymous
415025	NTHI477_RS02010	744	A	G	synonymous

415037	NTHI477_RS02010	756	C	T	synonymous
415049	NTHI477_RS02010	768	G	A	synonymous
415052	NTHI477_RS02010	771	A	G	synonymous
415055	NTHI477_RS02010	774	A	G	synonymous
415064	NTHI477_RS02010	783	T	A	synonymous
415069	NTHI477_RS02010	788	A	G	nonsynonymous
415094	NTHI477_RS02010	813	A	T	synonymous
415094	NTHI477_RS02010	813	A	C	synonymous
415100	NTHI477_RS02010	819	C	T	synonymous
415100	NTHI477_RS02010	819	C	G	synonymous
415109	NTHI477_RS02010	828	C	T	synonymous
415112	NTHI477_RS02010	831	C	T	synonymous
415113	NTHI477_RS02010	832	G	T	nonsynonymous
415115	NTHI477_RS02010	834	T	C	synonymous
415116	NTHI477_RS02010	835	A	G	nonsynonymous
415118	NTHI477_RS02010	837	C	T	synonymous
415136	NTHI477_RS02010	855	C	T	synonymous
415163	NTHI477_RS02010	882	G	A	synonymous
415171	NTHI477_RS02010	890	C	T	nonsynonymous
415172	NTHI477_RS02010	891	A	G	synonymous
415177	NTHI477_RS02010	896	C	A	nonsynonymous
415178	NTHI477_RS02010	897	G	A	synonymous
415208	NTHI477_RS02010	927	G	A	synonymous
415211	NTHI477_RS02010	930	A	C	synonymous
415212	NTHI477_RS02010	931	A	G	nonsynonymous
415241	NTHI477_RS02010	960	G	C	synonymous
415265	NTHI477_RS02010	984	C	T	synonymous
415274	NTHI477_RS02010	993	G	T	nonsynonymous
415280	NTHI477_RS02010	999	T	C	synonymous
415319	NTHI477_RS02010	1038	T	G	synonymous
415319	NTHI477_RS02010	1038	T	C	synonymous
415320	NTHI477_RS02010	1039	T	G	nonsynonymous
415322	NTHI477_RS02010	1041	C	T	synonymous
415330	NTHI477_RS02010	1049	A	G	nonsynonymous
415331	NTHI477_RS02010	1050	C	T	synonymous
415337	NTHI477_RS02010	1056	A	C	synonymous
415337	NTHI477_RS02010	1056	A	T	synonymous
415349	NTHI477_RS02010	1068	C	T	synonymous
415358	NTHI477_RS02010	1077	T	C	synonymous
415364	NTHI477_RS02010	1083	G	T	synonymous
415364	NTHI477_RS02010	1083	G	C	synonymous
415364	NTHI477_RS02010	1083	G	A	synonymous
415367	NTHI477_RS02010	1086	A	T	synonymous
415367	NTHI477_RS02010	1086	A	C	synonymous
415388	NTHI477_RS02010	1107	T	C	synonymous

415391	NTHI477_RS02010	1110	A	C	synonymous
415410	NTHI477_RS02010	1129	C	G	nonsynonymous
415417	NTHI477_RS02010	1136	G	A	nonsynonymous
415424	NTHI477_RS02010	1143	C	T	synonymous
415442	NTHI477_RS02010	1161	T	C	synonymous
415457	NTHI477_RS02010	1176	T	C	synonymous
415458	NTHI477_RS02010	1177	G	A	nonsynonymous
415469	NTHI477_RS02010	1188	T	A	synonymous
415475	NTHI477_RS02010	1194	G	A	synonymous
415490	NTHI477_RS02010	1209	A	C	synonymous
415490	NTHI477_RS02010	1209	A	G	synonymous
415505	NTHI477_RS02010	1224	T	G	synonymous
415511	NTHI477_RS02010	1230	A	G	synonymous
415514	NTHI477_RS02010	1233	C	A	synonymous
415520	NTHI477_RS02010	1239	C	T	synonymous
415523	NTHI477_RS02010	1242	C	T	synonymous
415523	NTHI477_RS02010	1242	C	G	synonymous
415526	NTHI477_RS02010	1245	T	C	synonymous
415540	intergenic	intergenic	C	T	intergenic
415558	intergenic	intergenic	A	G	intergenic
415559	intergenic	intergenic	G	A	intergenic
415571	intergenic	intergenic	T	C	intergenic
415594	intergenic	intergenic	T	A	intergenic
415606	intergenic	intergenic	C	T	intergenic
415608	intergenic	intergenic	T	C	intergenic
415609	intergenic	intergenic	T	A	intergenic
415626	NTHI477_RS02015	1134	T	C	synonymous
415635	NTHI477_RS02015	1125	G	A	synonymous
415659	NTHI477_RS02015	1101	G	A	synonymous
415662	NTHI477_RS02015	1098	T	C	synonymous
415686	NTHI477_RS02015	1074	G	A	synonymous
415712	NTHI477_RS02015	1048	A	G	synonymous
415719	NTHI477_RS02015	1041	C	T	synonymous
415728	NTHI477_RS02015	1032	G	T	nonsynonymous
415731	NTHI477_RS02015	1029	G	A	synonymous
415746	NTHI477_RS02015	1014	T	C	synonymous
415764	NTHI477_RS02015	996	G	T	synonymous
415767	NTHI477_RS02015	993	C	T	synonymous
415773	NTHI477_RS02015	987	T	G	synonymous
415779	NTHI477_RS02015	981	G	A	synonymous
415791	NTHI477_RS02015	969	T	C	synonymous
415797	NTHI477_RS02015	963	G	A	synonymous
415806	NTHI477_RS02015	954	A	G	synonymous
415806	NTHI477_RS02015	954	A	C	synonymous
415808	NTHI477_RS02015	952	C	T	nonsynonymous

415821	NTHI477_RS02015	939	A	G	synonymous
415823	NTHI477_RS02015	937	C	T	nonsynonymous
415824	NTHI477_RS02015	936	A	G	synonymous
415833	NTHI477_RS02015	927	T	G	synonymous
415842	NTHI477_RS02015	918	G	A	synonymous
415857	NTHI477_RS02015	903	T	A	synonymous
415857	NTHI477_RS02015	903	T	G	synonymous
415857	NTHI477_RS02015	903	T	C	synonymous
415863	NTHI477_RS02015	897	C	G	synonymous
415863	NTHI477_RS02015	897	C	A	synonymous
415869	NTHI477_RS02015	891	A	T	synonymous
415870	NTHI477_RS02015	890	G	A	nonsynonymous
415872	NTHI477_RS02015	888	A	T	synonymous
415875	NTHI477_RS02015	885	G	A	synonymous
415881	NTHI477_RS02015	879	A	G	synonymous
415884	NTHI477_RS02015	876	T	C	synonymous
415887	NTHI477_RS02015	873	C	T	synonymous
415890	NTHI477_RS02015	870	T	C	synonymous
415892	NTHI477_RS02015	868	T	G	synonymous
415893	NTHI477_RS02015	867	G	A	synonymous
415902	NTHI477_RS02015	858	G	A	synonymous
415905	NTHI477_RS02015	855	C	T	synonymous
415920	NTHI477_RS02015	840	A	C	synonymous
415923	NTHI477_RS02015	837	G	A	synonymous
415926	NTHI477_RS02015	834	A	T	synonymous
415928	NTHI477_RS02015	832	G	A	nonsynonymous
415929	NTHI477_RS02015	831	G	T	synonymous
415929	NTHI477_RS02015	831	G	C	synonymous
415932	NTHI477_RS02015	828	T	C	synonymous
415936	NTHI477_RS02015	824	G	A	nonsynonymous
415938	NTHI477_RS02015	822	A	T	synonymous
415947	NTHI477_RS02015	813	A	G	synonymous
415950	NTHI477_RS02015	810	C	T	synonymous
415959	NTHI477_RS02015	801	A	G	synonymous
415974	NTHI477_RS02015	786	G	A	synonymous
415994	NTHI477_RS02015	766	T	G	nonsynonymous
416004	NTHI477_RS02015	756	C	T	synonymous
416013	NTHI477_RS02015	747	C	G	synonymous
416016	NTHI477_RS02015	744	A	C	synonymous
416022	NTHI477_RS02015	738	T	C	synonymous
416028	NTHI477_RS02015	732	T	G	synonymous
416031	NTHI477_RS02015	729	A	C	synonymous
416031	NTHI477_RS02015	729	A	T	synonymous
416037	NTHI477_RS02015	723	T	C	synonymous
416040	NTHI477_RS02015	720	G	A	synonymous

416049	NTHI477_RS02015	711	G	A	synonymous
416052	NTHI477_RS02015	708	T	C	synonymous
416053	NTHI477_RS02015	707	G	A	nonsynonymous
416058	NTHI477_RS02015	702	A	G	synonymous
416064	NTHI477_RS02015	696	A	T	synonymous
416064	NTHI477_RS02015	696	A	G	synonymous
416073	NTHI477_RS02015	687	G	A	synonymous
416079	NTHI477_RS02015	681	T	C	synonymous
416082	NTHI477_RS02015	678	G	A	synonymous
416088	NTHI477_RS02015	672	C	A	synonymous
416139	NTHI477_RS02015	621	T	G	synonymous
416139	NTHI477_RS02015	621	T	A	synonymous
416142	NTHI477_RS02015	618	T	C	synonymous
416148	NTHI477_RS02015	612	T	G	synonymous
416151	NTHI477_RS02015	609	A	G	synonymous
416157	NTHI477_RS02015	603	A	G	synonymous
416160	NTHI477_RS02015	600	G	A	synonymous
416161	NTHI477_RS02015	599	C	T	nonsynonymous
416187	NTHI477_RS02015	573	G	A	synonymous
416193	NTHI477_RS02015	567	G	A	synonymous
416200	NTHI477_RS02015	560	G	C	nonsynonymous
416208	NTHI477_RS02015	552	A	G	synonymous
416223	NTHI477_RS02015	537	G	A	synonymous
416241	NTHI477_RS02015	519	A	G	synonymous
416307	NTHI477_RS02015	453	G	A	synonymous
416361	NTHI477_RS02015	399	A	T	synonymous
416370	NTHI477_RS02015	390	G	A	synonymous
416379	NTHI477_RS02015	381	T	C	synonymous
416381	NTHI477_RS02015	379	G	A	synonymous
416382	NTHI477_RS02015	378	G	A	synonymous
416383	NTHI477_RS02015	377	C	G	nonsynonymous
416469	NTHI477_RS02015	291	A	G	synonymous
416481	NTHI477_RS02015	279	A	G	synonymous
416484	NTHI477_RS02015	276	A	G	synonymous
416496	NTHI477_RS02015	264	G	A	synonymous
416514	NTHI477_RS02015	246	G	A	synonymous
416520	NTHI477_RS02015	240	A	T	synonymous
416522	NTHI477_RS02015	238	T	C	nonsynonymous
416523	NTHI477_RS02015	237	G	T	synonymous
416526	NTHI477_RS02015	234	A	C	synonymous
416570	NTHI477_RS02015	190	C	T	nonsynonymous
416658	NTHI477_RS02015	102	G	A	synonymous
416661	NTHI477_RS02015	99	A	G	synonymous
416688	NTHI477_RS02015	72	C	A	synonymous
416697	NTHI477_RS02015	63	G	A	synonymous

416700	NTHI477_RS02015	60	G	A	synonymous
416703	NTHI477_RS02015	57	G	A	synonymous
416705	NTHI477_RS02015	55	C	T	nonsynonymous
416706	NTHI477_RS02015	54	C	T	synonymous
416726	NTHI477_RS02015	34	G	A	synonymous
416727	NTHI477_RS02015	33	G	A	synonymous
416733	NTHI477_RS02015	27	G	A	synonymous
416742	NTHI477_RS02015	18	G	A	synonymous
416829	intergenic	intergenic	C	T	intergenic
416882	NTHI477_RS02020	1872	T	C	synonymous
416912	NTHI477_RS02020	1842	T	C	synonymous
416951	NTHI477_RS02020	1803	A	C	synonymous
416952	NTHI477_RS02020	1802	A	G	nonsynonymous
416954	NTHI477_RS02020	1800	A	T	synonymous
416954	NTHI477_RS02020	1800	A	C	synonymous
416955	NTHI477_RS02020	1799	G	A	nonsynonymous
416966	NTHI477_RS02020	1788	T	C	synonymous
416999	NTHI477_RS02020	1755	C	T	synonymous
417032	NTHI477_RS02020	1722	T	C	synonymous
417035	NTHI477_RS02020	1719	T	C	synonymous
417044	NTHI477_RS02020	1710	C	T	synonymous
417049	NTHI477_RS02020	1705	C	A	nonsynonymous
417050	NTHI477_RS02020	1704	G	C	synonymous
417054	NTHI477_RS02020	1700	G	A	nonsynonymous
417056	NTHI477_RS02020	1698	A	T	synonymous
417058	NTHI477_RS02020	1696	C	T	nonsynonymous
417080	NTHI477_RS02020	1674	G	A	synonymous
417081	NTHI477_RS02020	1673	G	A	nonsynonymous
417083	NTHI477_RS02020	1671	T	A	nonsynonymous
417084	NTHI477_RS02020	1670	C	T	nonsynonymous
417090	NTHI477_RS02020	1664	G	A	nonsynonymous
417128	NTHI477_RS02020	1626	A	T	synonymous
417152	NTHI477_RS02020	1602	A	T	synonymous
417152	NTHI477_RS02020	1602	A	C	synonymous
417154	NTHI477_RS02020	1600	T	C	nonsynonymous
417188	NTHI477_RS02020	1566	A	G	synonymous
417203	NTHI477_RS02020	1551	A	G	synonymous
417210	NTHI477_RS02020	1544	A	T	nonsynonymous
417245	NTHI477_RS02020	1509	A	T	synonymous
417245	NTHI477_RS02020	1509	A	C	synonymous
417280	NTHI477_RS02020	1474	C	T	nonsynonymous
417293	NTHI477_RS02020	1461	T	C	synonymous
417296	NTHI477_RS02020	1458	C	T	synonymous
417296	NTHI477_RS02020	1458	C	A	synonymous
417323	NTHI477_RS02020	1431	G	A	synonymous

417341	NTHI477_RS02020	1413	G	A	synonymous
417356	NTHI477_RS02020	1398	A	G	synonymous
417359	NTHI477_RS02020	1395	T	C	synonymous
417362	NTHI477_RS02020	1392	T	C	synonymous
417377	NTHI477_RS02020	1377	T	C	synonymous
417383	NTHI477_RS02020	1371	G	A	synonymous
417428	NTHI477_RS02020	1326	A	G	synonymous
417497	NTHI477_RS02020	1257	G	A	synonymous
417506	NTHI477_RS02020	1248	C	A	synonymous
417512	NTHI477_RS02020	1242	G	A	synonymous
417539	NTHI477_RS02020	1215	G	A	synonymous
417542	NTHI477_RS02020	1212	A	G	synonymous
417560	NTHI477_RS02020	1194	A	T	synonymous
417562	NTHI477_RS02020	1192	G	A	nonsynonymous
417563	NTHI477_RS02020	1191	T	A	synonymous
417575	NTHI477_RS02020	1179	A	T	synonymous
417578	NTHI477_RS02020	1176	A	G	synonymous
417592	NTHI477_RS02020	1162	T	C	nonsynonymous
417593	NTHI477_RS02020	1161	G	A	synonymous
417605	NTHI477_RS02020	1149	G	A	synonymous
417626	NTHI477_RS02020	1128	T	A	synonymous
417638	NTHI477_RS02020	1116	A	G	synonymous
417644	NTHI477_RS02020	1110	T	C	synonymous
417662	NTHI477_RS02020	1092	A	T	synonymous
417662	NTHI477_RS02020	1092	A	C	synonymous
417674	NTHI477_RS02020	1080	A	C	synonymous
417674	NTHI477_RS02020	1080	A	T	synonymous
417683	NTHI477_RS02020	1071	A	G	synonymous
417686	NTHI477_RS02020	1068	A	G	synonymous
417692	NTHI477_RS02020	1062	C	T	synonymous
417710	NTHI477_RS02020	1044	T	A	synonymous
417731	NTHI477_RS02020	1023	A	T	synonymous
417731	NTHI477_RS02020	1023	A	G	synonymous
417734	NTHI477_RS02020	1020	A	G	synonymous
417761	NTHI477_RS02020	993	A	G	synonymous
417774	NTHI477_RS02020	980	C	T	nonsynonymous
417776	NTHI477_RS02020	978	A	T	synonymous
417791	NTHI477_RS02020	963	A	C	synonymous
417793	NTHI477_RS02020	961	T	C	nonsynonymous
417797	NTHI477_RS02020	957	G	C	synonymous
417799	NTHI477_RS02020	955	G	A	nonsynonymous
417800	NTHI477_RS02020	954	T	A	synonymous
417833	NTHI477_RS02020	921	C	T	synonymous
417839	NTHI477_RS02020	915	T	C	synonymous
417872	NTHI477_RS02020	882	G	A	synonymous

417881	NTHI477_RS02020	873	G	A	synonymous
417884	NTHI477_RS02020	870	T	A	synonymous
417887	NTHI477_RS02020	867	A	C	synonymous
417896	NTHI477_RS02020	858	A	C	synonymous
417902	NTHI477_RS02020	852	G	A	synonymous
417905	NTHI477_RS02020	849	A	C	synonymous
417908	NTHI477_RS02020	846	T	A	nonsynonymous
417910	NTHI477_RS02020	844	A	G	synonymous
417911	NTHI477_RS02020	843	A	G	synonymous
417914	NTHI477_RS02020	840	G	C	synonymous
417920	NTHI477_RS02020	834	G	A	synonymous
417929	NTHI477_RS02020	825	C	A	synonymous
417962	NTHI477_RS02020	792	C	A	synonymous
417989	NTHI477_RS02020	765	T	C	synonymous
417989	NTHI477_RS02020	765	T	A	synonymous
418007	NTHI477_RS02020	747	A	G	synonymous
418010	NTHI477_RS02020	744	A	G	synonymous
418028	NTHI477_RS02020	726	T	C	synonymous
418037	NTHI477_RS02020	717	G	A	synonymous
418049	NTHI477_RS02020	705	T	G	synonymous
418061	NTHI477_RS02020	693	A	G	synonymous
418070	NTHI477_RS02020	684	C	A	synonymous
418073	NTHI477_RS02020	681	G	A	synonymous
418091	NTHI477_RS02020	663	A	C	synonymous
418094	NTHI477_RS02020	660	G	C	synonymous
418094	NTHI477_RS02020	660	G	A	synonymous
418100	NTHI477_RS02020	654	C	T	synonymous
418103	NTHI477_RS02020	651	T	C	synonymous
418157	NTHI477_RS02020	597	G	A	synonymous
418178	NTHI477_RS02020	576	G	A	synonymous
418184	NTHI477_RS02020	570	T	A	synonymous
418196	NTHI477_RS02020	558	A	G	synonymous
418205	NTHI477_RS02020	549	G	A	synonymous
418220	NTHI477_RS02020	534	A	G	synonymous
418223	NTHI477_RS02020	531	C	T	synonymous
418226	NTHI477_RS02020	528	T	C	synonymous
418229	NTHI477_RS02020	525	T	C	synonymous
418250	NTHI477_RS02020	504	G	A	synonymous
418262	NTHI477_RS02020	492	A	T	synonymous
418265	NTHI477_RS02020	489	A	G	synonymous
418271	NTHI477_RS02020	483	A	G	synonymous
418277	NTHI477_RS02020	477	G	A	synonymous
418286	NTHI477_RS02020	468	C	T	synonymous
418295	NTHI477_RS02020	459	C	A	synonymous
418295	NTHI477_RS02020	459	C	T	synonymous

418313	NTHI477_RS02020	441	A	G	synonymous
418316	NTHI477_RS02020	438	G	A	synonymous
418319	NTHI477_RS02020	435	G	A	synonymous
418331	NTHI477_RS02020	423	A	C	synonymous
418334	NTHI477_RS02020	420	A	G	synonymous
418343	NTHI477_RS02020	411	C	T	synonymous
418349	NTHI477_RS02020	405	T	A	synonymous
418355	NTHI477_RS02020	399	A	C	synonymous
418357	NTHI477_RS02020	397	T	C	nonsynonymous
418357	NTHI477_RS02020	397	T	A	nonsynonymous
418358	NTHI477_RS02020	396	C	T	synonymous
418361	NTHI477_RS02020	393	T	G	synonymous
418361	NTHI477_RS02020	393	T	A	synonymous
418379	NTHI477_RS02020	375	C	A	synonymous
418400	NTHI477_RS02020	354	C	T	synonymous
418406	NTHI477_RS02020	348	A	T	synonymous
418409	NTHI477_RS02020	345	A	T	synonymous
418412	NTHI477_RS02020	342	G	A	synonymous
418418	NTHI477_RS02020	336	T	C	synonymous
418421	NTHI477_RS02020	333	C	T	synonymous
418424	NTHI477_RS02020	330	C	T	synonymous
418424	NTHI477_RS02020	330	C	G	synonymous
418430	NTHI477_RS02020	324	T	C	synonymous
418433	NTHI477_RS02020	321	A	G	synonymous
418436	NTHI477_RS02020	318	G	A	synonymous
418442	NTHI477_RS02020	312	T	C	synonymous
418445	NTHI477_RS02020	309	A	G	synonymous
418448	NTHI477_RS02020	306	G	T	synonymous
418454	NTHI477_RS02020	300	A	C	synonymous
418460	NTHI477_RS02020	294	G	A	synonymous
418469	NTHI477_RS02020	285	A	G	synonymous
418484	NTHI477_RS02020	270	A	G	synonymous
418520	NTHI477_RS02020	234	A	G	synonymous
418553	NTHI477_RS02020	201	A	C	synonymous
418562	NTHI477_RS02020	192	T	A	synonymous
418571	NTHI477_RS02020	183	C	T	synonymous
418589	NTHI477_RS02020	165	A	G	synonymous
418607	NTHI477_RS02020	147	A	C	synonymous
418607	NTHI477_RS02020	147	A	G	synonymous
418613	NTHI477_RS02020	141	A	G	synonymous
418619	NTHI477_RS02020	135	G	A	synonymous
418631	NTHI477_RS02020	123	T	A	synonymous
418637	NTHI477_RS02020	117	G	A	synonymous
418643	NTHI477_RS02020	111	T	C	synonymous
418643	NTHI477_RS02020	111	T	A	synonymous

418646	NTHI477_RS02020	108	A	G	synonymous
418649	NTHI477_RS02020	105	G	A	synonymous
418649	NTHI477_RS02020	105	G	T	synonymous
418655	NTHI477_RS02020	99	C	T	synonymous
418658	NTHI477_RS02020	96	A	C	synonymous
418661	NTHI477_RS02020	93	T	C	synonymous
418676	NTHI477_RS02020	78	T	A	synonymous
418679	NTHI477_RS02020	75	G	A	synonymous
418682	NTHI477_RS02020	72	C	A	synonymous
418688	NTHI477_RS02020	66	G	A	synonymous
418703	NTHI477_RS02020	51	T	A	synonymous
418784	intergenic	intergenic	A	G	intergenic
418801	intergenic	intergenic	G	A	intergenic
418804	intergenic	intergenic	T	G	intergenic
418804	intergenic	intergenic	T	C	intergenic
418821	intergenic	intergenic	C	T	intergenic
418873	intergenic	intergenic	A	G	intergenic
418879	intergenic	intergenic	C	T	intergenic
418895	intergenic	intergenic	A	C	intergenic
418915	intergenic	intergenic	C	G	intergenic
418921	intergenic	intergenic	G	A	intergenic
418929	intergenic	intergenic	C	T	intergenic
419036	NTHI477_RS02025	31	G	A	nonsynonymous
419038	NTHI477_RS02025	33	C	G	synonymous
419039	NTHI477_RS02025	34	G	A	nonsynonymous
419042	NTHI477_RS02025	37	G	T	nonsynonymous
419044	NTHI477_RS02025	39	G	C	synonymous
419044	NTHI477_RS02025	39	G	T	synonymous
419053	NTHI477_RS02025	48	C	T	synonymous
419062	NTHI477_RS02025	57	T	C	synonymous
419063	NTHI477_RS02025	58	A	G	nonsynonymous
419065	NTHI477_RS02025	60	C	A	synonymous
419066	NTHI477_RS02025	61	A	G	nonsynonymous
419089	NTHI477_RS02025	84	G	A	synonymous
419092	NTHI477_RS02025	87	T	G	synonymous
419108	NTHI477_RS02025	103	C	T	nonsynonymous
419122	NTHI477_RS02025	117	A	G	synonymous
419138	NTHI477_RS02025	133	G	A	nonsynonymous
419151	NTHI477_RS02025	146	C	A	nonsynonymous
419177	NTHI477_RS02025	172	A	G	nonsynonymous
419190	NTHI477_RS02025	185	A	T	nonsynonymous
419194	NTHI477_RS02025	189	C	A	nonsynonymous
419195	NTHI477_RS02025	190	A	T	nonsynonymous
419209	NTHI477_RS02025	204	G	C	synonymous
419215	NTHI477_RS02025	210	T	C	synonymous

419230	NTHI477_RS02025	225	T	A	synonymous
419231	NTHI477_RS02025	226	G	T	nonsynonymous
419239	NTHI477_RS02025	234	T	C	synonymous
419242	NTHI477_RS02025	237	A	G	synonymous
419277	NTHI477_RS02025	272	T	C	nonsynonymous
419278	NTHI477_RS02025	273	A	G	nonsynonymous
419308	NTHI477_RS02025	303	T	C	synonymous
419321	NTHI477_RS02025	316	A	C	nonsynonymous
419333	NTHI477_RS02025	328	A	G	nonsynonymous
419341	NTHI477_RS02025	336	C	T	synonymous
419353	NTHI477_RS02025	348	C	A	synonymous
419361	NTHI477_RS02025	356	G	A	nonsynonymous
419384	NTHI477_RS02025	379	C	T	nonsynonymous
419386	NTHI477_RS02025	381	T	C	synonymous
419392	NTHI477_RS02025	387	C	T	synonymous
419393	NTHI477_RS02025	388	G	A	nonsynonymous
419399	NTHI477_RS02025	394	A	G	nonsynonymous
419399	NTHI477_RS02025	394	A	C	nonsynonymous
419407	NTHI477_RS02025	402	A	G	synonymous
419414	NTHI477_RS02025	409	C	T	synonymous
419428	NTHI477_RS02025	423	C	T	synonymous
419429	NTHI477_RS02025	424	G	A	nonsynonymous
419441	NTHI477_RS02025	436	T	G	nonsynonymous
419443	NTHI477_RS02025	438	C	A	synonymous
419448	NTHI477_RS02025	443	A	G	nonsynonymous
419479	NTHI477_RS02025	474	G	A	synonymous
419494	NTHI477_RS02025	489	G	A	synonymous
419504	NTHI477_RS02025	499	G	A	nonsynonymous
419525	NTHI477_RS02025	520	A	G	nonsynonymous
419528	NTHI477_RS02025	523	A	G	nonsynonymous
419537	NTHI477_RS02025	532	G	A	nonsynonymous
419546	NTHI477_RS02025	541	G	A	nonsynonymous
419549	NTHI477_RS02025	544	A	C	nonsynonymous
419555	NTHI477_RS02025	550	G	A	nonsynonymous
419556	NTHI477_RS02025	551	A	C	nonsynonymous
419562	NTHI477_RS02025	557	G	A	nonsynonymous
419563	NTHI477_RS02025	558	C	T	synonymous
419566	NTHI477_RS02025	561	T	C	synonymous
419570	NTHI477_RS02025	565	A	G	nonsynonymous
419571	NTHI477_RS02025	566	C	T	nonsynonymous
419581	NTHI477_RS02025	576	A	C	synonymous
419585	NTHI477_RS02025	580	G	A	nonsynonymous
419601	NTHI477_RS02025	596	A	C	nonsynonymous
419605	NTHI477_RS02025	600	A	T	synonymous
419609	NTHI477_RS02025	604	T	C	nonsynonymous

419624	NTHI477_RS02025	619	G	A	nonsynonymous
419645	NTHI477_RS02025	640	G	A	nonsynonymous
419654	NTHI477_RS02025	649	A	G	nonsynonymous
419665	NTHI477_RS02025	660	G	A	synonymous
419666	NTHI477_RS02025	661	T	C	synonymous
419668	NTHI477_RS02025	663	G	A	synonymous
419701	NTHI477_RS02025	696	G	A	synonymous
419743	NTHI477_RS02025	738	A	G	synonymous
419745	NTHI477_RS02025	740	C	T	nonsynonymous
419747	NTHI477_RS02025	742	C	G	nonsynonymous
419760	NTHI477_RS02025	755	A	G	nonsynonymous
419785	NTHI477_RS02025	780	T	C	synonymous
419792	NTHI477_RS02025	787	G	A	nonsynonymous
419811	NTHI477_RS02025	806	T	C	nonsynonymous
419815	NTHI477_RS02025	810	G	A	synonymous
419818	NTHI477_RS02025	813	T	A	synonymous
419822	NTHI477_RS02025	817	A	G	nonsynonymous
419845	NTHI477_RS02025	840	G	A	synonymous
419891	NTHI477_RS02025	886	A	T	nonsynonymous
419921	NTHI477_RS02025	916	G	A	nonsynonymous
419922	NTHI477_RS02025	917	G	T	nonsynonymous
419926	NTHI477_RS02025	921	C	T	synonymous
419932	NTHI477_RS02025	927	A	G	synonymous
419938	NTHI477_RS02025	933	A	G	synonymous
419947	NTHI477_RS02025	942	T	C	synonymous
419956	NTHI477_RS02025	951	T	A	synonymous
419968	NTHI477_RS02025	963	A	G	synonymous
419971	NTHI477_RS02025	966	C	T	synonymous
419974	NTHI477_RS02025	969	C	T	synonymous
419982	NTHI477_RS02025	977	T	C	nonsynonymous
419985	NTHI477_RS02025	980	C	T	nonsynonymous
419986	NTHI477_RS02025	981	T	G	synonymous
419990	NTHI477_RS02025	985	T	C	nonsynonymous
419996	NTHI477_RS02025	991	A	C	nonsynonymous
420000	NTHI477_RS02025	995	T	C	nonsynonymous
420025	NTHI477_RS02025	1020	C	T	synonymous
420029	NTHI477_RS02025	1024	A	G	nonsynonymous
420030	NTHI477_RS02025	1025	T	A	nonsynonymous
420030	NTHI477_RS02025	1025	T	C	nonsynonymous
420031	NTHI477_RS02025	1026	C	A	synonymous
420058	NTHI477_RS02025	1053	A	G	synonymous
420062	NTHI477_RS02025	1057	A	T	nonsynonymous
420109	NTHI477_RS02025	1104	A	G	synonymous
420148	NTHI477_RS02025	1143	G	A	nonsynonymous
420150	NTHI477_RS02025	1145	G	A	nonsynonymous

420151	NTHI477_RS02025	1146	C	T	synonymous
420184	NTHI477_RS02025	1179	A	C	synonymous
420184	NTHI477_RS02025	1179	A	G	synonymous
420187	NTHI477_RS02025	1182	C	T	synonymous
420202	NTHI477_RS02025	1197	C	T	synonymous
420211	NTHI477_RS02025	1206	T	C	synonymous
420214	NTHI477_RS02025	1209	G	A	synonymous
420235	NTHI477_RS02025	1230	T	C	synonymous
420236	NTHI477_RS02025	1231	G	A	nonsynonymous
420237	NTHI477_RS02025	1232	T	C	nonsynonymous
420262	NTHI477_RS02025	1257	C	A	nonsynonymous
420263	NTHI477_RS02025	1258	G	A	nonsynonymous
420265	NTHI477_RS02025	1260	T	C	synonymous
420266	NTHI477_RS02025	1261	C	T	nonsynonymous
420267	NTHI477_RS02025	1262	A	T	nonsynonymous
420270	NTHI477_RS02025	1265	G	A	nonsynonymous
420272	NTHI477_RS02025	1267	T	A	nonsynonymous
420274	NTHI477_RS02025	1269	G	A	synonymous
420275	NTHI477_RS02025	1270	A	G	nonsynonymous
420287	NTHI477_RS02025	1282	A	G	nonsynonymous
420289	NTHI477_RS02025	1284	C	T	synonymous
420290	NTHI477_RS02025	1285	G	A	nonsynonymous
420295	NTHI477_RS02025	1290	T	C	synonymous
420300	NTHI477_RS02025	1295	T	C	nonsynonymous
420301	NTHI477_RS02025	1296	G	A	synonymous
420306	NTHI477_RS02025	1301	A	G	nonsynonymous
420320	NTHI477_RS02025	1315	A	G	nonsynonymous
420322	NTHI477_RS02025	1317	G	C	synonymous
420323	NTHI477_RS02025	1318	G	A	nonsynonymous
420325	NTHI477_RS02025	1320	C	T	synonymous
420329	NTHI477_RS02025	1324	A	G	nonsynonymous
420331	NTHI477_RS02025	1326	C	T	synonymous
420332	NTHI477_RS02025	1327	G	A	nonsynonymous
420333	NTHI477_RS02025	1328	T	C	nonsynonymous
420334	NTHI477_RS02025	1329	A	C	synonymous
420350	NTHI477_RS02025	1345	C	T	synonymous
420355	NTHI477_RS02025	1350	A	G	synonymous
420364	NTHI477_RS02025	1359	C	A	nonsynonymous
420367	NTHI477_RS02025	1362	G	A	synonymous
420400	NTHI477_RS02025	1395	G	A	synonymous
420407	NTHI477_RS02025	1402	G	A	nonsynonymous
420410	NTHI477_RS02025	1405	G	A	nonsynonymous
420410	NTHI477_RS02025	1405	G	C	nonsynonymous
420412	NTHI477_RS02025	1407	A	C	synonymous
420418	NTHI477_RS02025	1413	T	C	synonymous

420421	NTHI477_RS02025	1416	A	T	synonymous
420433	NTHI477_RS02025	1428	G	A	synonymous
420434	NTHI477_RS02025	1429	G	A	nonsynonymous
420451	intergenic	intergenic	G	A	intergenic
420458	intergenic	intergenic	G	T	intergenic
420481	intergenic	intergenic	T	C	intergenic
420483	intergenic	intergenic	T	C	intergenic
420484	intergenic	intergenic	A	G	intergenic
420494	intergenic	intergenic	A	C	intergenic
420523	intergenic	intergenic	G	A	intergenic
420533	intergenic	intergenic	T	C	intergenic
420553	intergenic	intergenic	T	C	intergenic
420577	intergenic	intergenic	A	C	intergenic
420614	NTHI477_RS02030	450	C	A	synonymous
420632	NTHI477_RS02030	432	T	C	synonymous
420644	NTHI477_RS02030	420	T	C	synonymous
420647	NTHI477_RS02030	417	G	T	nonsynonymous
420656	NTHI477_RS02030	408	C	A	synonymous
420701	NTHI477_RS02030	363	G	A	synonymous
420713	NTHI477_RS02030	351	C	G	synonymous
420713	NTHI477_RS02030	351	C	A	synonymous
420716	NTHI477_RS02030	348	A	G	synonymous
420722	NTHI477_RS02030	342	G	A	synonymous
420749	NTHI477_RS02030	315	A	G	synonymous
420758	NTHI477_RS02030	306	C	T	synonymous
420770	NTHI477_RS02030	294	A	G	synonymous
420776	NTHI477_RS02030	288	T	G	synonymous
420779	NTHI477_RS02030	285	A	G	synonymous
420815	NTHI477_RS02030	249	G	A	synonymous
420848	NTHI477_RS02030	216	T	C	synonymous
420851	NTHI477_RS02030	213	G	A	synonymous
420854	NTHI477_RS02030	210	A	G	synonymous
420857	NTHI477_RS02030	207	T	G	synonymous
420863	NTHI477_RS02030	201	C	T	synonymous
420866	NTHI477_RS02030	198	A	G	synonymous
420917	NTHI477_RS02030	147	C	G	synonymous
420917	NTHI477_RS02030	147	C	T	synonymous
420920	NTHI477_RS02030	144	T	A	synonymous
420923	NTHI477_RS02030	141	T	C	synonymous
420949	NTHI477_RS02030	115	G	A	nonsynonymous
420953	NTHI477_RS02030	111	A	G	synonymous
420956	NTHI477_RS02030	108	T	A	synonymous
420998	NTHI477_RS02030	66	A	G	synonymous
421001	NTHI477_RS02030	63	A	G	synonymous
421004	NTHI477_RS02030	60	A	G	synonymous

421007	NTHI477_RS02030	57	G	A	synonymous
421016	NTHI477_RS02030	48	A	T	synonymous
421049	NTHI477_RS02030	15	A	T	synonymous
421052	NTHI477_RS02030	12	T	G	synonymous
421058	NTHI477_RS02030	6	C	A	nonsynonymous
421088	intergenic	intergenic	C	T	intergenic
421133	intergenic	intergenic	T	G	intergenic
421134	intergenic	intergenic	G	A	intergenic
421160	intergenic	intergenic	A	G	intergenic
421174	intergenic	intergenic	A	T	intergenic
421195	intergenic	intergenic	G	C	intergenic
421304	intergenic	intergenic	G	T	intergenic
421310	intergenic	intergenic	G	T	intergenic
421312	intergenic	intergenic	G	T	intergenic
421387	NTHI477_RS02035	25	A	G	nonsynonymous
421470	NTHI477_RS02035	108	T	C	synonymous
421515	NTHI477_RS02035	153	G	A	synonymous
421590	NTHI477_RS02035	228	C	T	synonymous
421701	NTHI477_RS02035	339	A	T	synonymous
421707	NTHI477_RS02035	345	A	C	synonymous
421743	NTHI477_RS02035	381	T	C	synonymous
421758	NTHI477_RS02035	396	T	C	synonymous
421761	NTHI477_RS02035	399	C	T	synonymous
421764	NTHI477_RS02035	402	C	T	synonymous
421767	NTHI477_RS02035	405	T	C	synonymous
421794	NTHI477_RS02035	432	A	T	synonymous
421797	NTHI477_RS02035	435	A	T	synonymous
421815	NTHI477_RS02035	453	T	A	synonymous
421878	NTHI477_RS02035	516	C	T	synonymous
421899	NTHI477_RS02035	537	C	T	synonymous
421920	NTHI477_RS02035	558	C	T	synonymous
421929	NTHI477_RS02035	567	C	T	synonymous
421965	NTHI477_RS02035	603	C	A	synonymous
421992	NTHI477_RS02035	630	T	C	synonymous
421993	NTHI477_RS02035	631	C	T	nonsynonymous
421995	NTHI477_RS02035	633	C	A	synonymous
422007	NTHI477_RS02035	645	T	C	synonymous
422016	NTHI477_RS02035	654	T	C	synonymous
422025	NTHI477_RS02035	663	T	C	synonymous
422034	NTHI477_RS02035	672	T	C	synonymous
422040	NTHI477_RS02035	678	A	G	synonymous
422055	NTHI477_RS02035	693	C	G	synonymous
422058	NTHI477_RS02035	696	G	A	synonymous
422082	NTHI477_RS02035	720	T	C	synonymous
422082	NTHI477_RS02035	720	T	G	synonymous

422094	NTHI477_RS02035	732	T	C	synonymous
422097	NTHI477_RS02035	735	G	A	synonymous
422100	NTHI477_RS02035	738	T	A	synonymous
422106	NTHI477_RS02035	744	G	A	synonymous
422107	NTHI477_RS02035	745	A	C	nonsynonymous
422109	NTHI477_RS02035	747	G	C	nonsynonymous
422109	NTHI477_RS02035	747	G	A	synonymous
422121	NTHI477_RS02035	759	A	G	synonymous
422130	NTHI477_RS02035	768	T	A	synonymous
422130	NTHI477_RS02035	768	T	G	synonymous
422133	NTHI477_RS02035	771	C	T	synonymous
422136	NTHI477_RS02035	774	C	T	synonymous
422157	NTHI477_RS02035	795	T	C	synonymous
422160	NTHI477_RS02035	798	C	A	synonymous
422163	NTHI477_RS02035	801	T	G	synonymous
422166	NTHI477_RS02035	804	G	T	synonymous
422193	NTHI477_RS02035	831	A	G	synonymous
422202	NTHI477_RS02035	840	G	A	synonymous
422214	NTHI477_RS02035	852	T	C	synonymous
422219	NTHI477_RS02035	857	G	A	nonsynonymous
422270	NTHI477_RS02035	908	C	A	nonsynonymous
422313	NTHI477_RS02035	951	C	G	synonymous
422322	NTHI477_RS02035	960	A	T	synonymous
422325	NTHI477_RS02035	963	C	T	synonymous
422355	NTHI477_RS02035	993	A	T	synonymous
422359	NTHI477_RS02035	997	A	G	nonsynonymous
422364	NTHI477_RS02035	1002	C	T	synonymous
422376	NTHI477_RS02035	1014	C	T	synonymous
422397	NTHI477_RS02035	1035	T	C	synonymous
422454	NTHI477_RS02035	1092	T	C	synonymous
422475	NTHI477_RS02035	1113	A	G	synonymous
422698	NTHI477_RS02035	1336	T	C	nonsynonymous
422745	NTHI477_RS02035	1383	C	T	synonymous
422796	NTHI477_RS02035	1434	G	A	synonymous
422925	NTHI477_RS02035	1563	A	G	synonymous
422992	NTHI477_RS02035	1630	G	A	nonsynonymous
423048	NTHI477_RS02035	1686	A	G	synonymous
423049	NTHI477_RS02035	1687	T	C	synonymous
423096	NTHI477_RS02035	1734	A	T	synonymous
423102	NTHI477_RS02035	1740	G	A	synonymous
423105	NTHI477_RS02035	1743	C	T	synonymous
423108	NTHI477_RS02035	1746	G	A	synonymous
423111	NTHI477_RS02035	1749	C	T	synonymous
423144	NTHI477_RS02035	1782	T	C	synonymous
423162	NTHI477_RS02035	1800	C	T	synonymous

423231	NTHI477_RS02035	1869	G	A	synonymous
423261	NTHI477_RS02035	1899	C	A	synonymous
423267	NTHI477_RS02035	1905	T	C	synonymous
423282	NTHI477_RS02035	1920	C	T	synonymous
423297	NTHI477_RS02035	1935	T	C	synonymous
423306	NTHI477_RS02035	1944	C	T	synonymous
423307	NTHI477_RS02035	1945	G	A	nonsynonymous
423309	NTHI477_RS02035	1947	T	C	synonymous
423318	NTHI477_RS02035	1956	T	C	synonymous
423321	NTHI477_RS02035	1959	C	T	synonymous
423322	NTHI477_RS02035	1960	G	A	nonsynonymous
423324	NTHI477_RS02035	1962	T	C	synonymous
423342	NTHI477_RS02035	1980	T	C	synonymous
423345	NTHI477_RS02035	1983	A	T	synonymous
423354	NTHI477_RS02035	1992	T	C	synonymous
423360	NTHI477_RS02035	1998	T	A	synonymous
423411	NTHI477_RS02035	2049	A	G	synonymous
423441	NTHI477_RS02035	2079	G	A	synonymous
423456	NTHI477_RS02035	2094	A	G	synonymous
423462	NTHI477_RS02035	2100	T	A	synonymous
423486	NTHI477_RS02035	2124	T	C	synonymous
423519	NTHI477_RS02035	2157	T	C	synonymous
423536	NTHI477_RS02035	2174	A	G	nonsynonymous
423543	NTHI477_RS02035	2181	A	G	synonymous
423546	NTHI477_RS02035	2184	A	G	synonymous
423561	NTHI477_RS02035	2199	G	A	synonymous
423570	NTHI477_RS02035	2208	T	C	synonymous
423573	NTHI477_RS02035	2211	C	T	synonymous
423576	NTHI477_RS02035	2214	T	G	synonymous
423585	NTHI477_RS02035	2223	G	A	synonymous
423588	NTHI477_RS02035	2226	A	G	synonymous
423600	NTHI477_RS02035	2238	A	C	synonymous
423603	NTHI477_RS02035	2241	T	A	nonsynonymous
423606	NTHI477_RS02035	2244	C	T	synonymous
423609	NTHI477_RS02035	2247	C	T	synonymous
423612	NTHI477_RS02035	2250	T	C	synonymous
423615	NTHI477_RS02035	2253	G	T	synonymous
423618	NTHI477_RS02035	2256	T	A	synonymous
423630	NTHI477_RS02035	2268	T	C	synonymous
423636	NTHI477_RS02035	2274	G	C	synonymous
423642	NTHI477_RS02035	2280	A	T	synonymous
423654	NTHI477_RS02035	2292	A	G	synonymous
423657	NTHI477_RS02035	2295	A	T	synonymous
423660	NTHI477_RS02035	2298	C	T	synonymous
423669	NTHI477_RS02035	2307	T	C	synonymous

423674	NTHI477_RS02035	2312	C	T	nonsynonymous
423675	NTHI477_RS02035	2313	A	G	synonymous
423712	NTHI477_RS02035	2350	C	T	nonsynonymous
423714	NTHI477_RS02035	2352	C	T	synonymous
423717	NTHI477_RS02035	2355	A	G	synonymous
423720	NTHI477_RS02035	2358	A	T	synonymous
423723	NTHI477_RS02035	2361	C	T	synonymous
423727	NTHI477_RS02035	2365	G	A	nonsynonymous
423753	NTHI477_RS02035	2391	C	A	synonymous
423774	NTHI477_RS02035	2412	G	T	synonymous
423779	NTHI477_RS02035	2417	G	C	nonsynonymous
423780	NTHI477_RS02035	2418	T	A	synonymous
423783	NTHI477_RS02035	2421	T	C	synonymous
423786	NTHI477_RS02035	2424	T	A	synonymous
423795	NTHI477_RS02035	2433	C	T	synonymous
423801	NTHI477_RS02035	2439	A	G	synonymous
423804	NTHI477_RS02035	2442	T	C	synonymous
423807	NTHI477_RS02035	2445	A	T	synonymous
423812	NTHI477_RS02035	2450	G	A	nonsynonymous
423814	NTHI477_RS02035	2452	A	C	nonsynonymous
423815	NTHI477_RS02035	2453	A	G	nonsynonymous
423816	NTHI477_RS02035	2454	G	T	nonsynonymous
423819	NTHI477_RS02035	2457	C	T	synonymous
423825	NTHI477_RS02035	2463	T	C	synonymous
423828	NTHI477_RS02035	2466	A	G	synonymous
423840	NTHI477_RS02035	2478	C	T	synonymous
423843	NTHI477_RS02035	2481	T	C	synonymous
423858	NTHI477_RS02035	2496	T	C	synonymous
423885	NTHI477_RS02035	2523	C	T	synonymous
423891	NTHI477_RS02035	2529	T	C	synonymous
423897	NTHI477_RS02035	2535	A	T	synonymous
423906	NTHI477_RS02035	2544	T	G	synonymous
423915	NTHI477_RS02035	2553	T	A	synonymous
423924	NTHI477_RS02035	2562	T	A	synonymous
423933	NTHI477_RS02035	2571	G	A	synonymous
423948	NTHI477_RS02035	2586	A	T	synonymous
423949	NTHI477_RS02035	2587	T	A	nonsynonymous
423954	NTHI477_RS02035	2592	C	T	synonymous
423955	NTHI477_RS02035	2593	C	A	nonsynonymous
423960	NTHI477_RS02035	2598	G	A	synonymous
423963	NTHI477_RS02035	2601	T	G	synonymous
423966	NTHI477_RS02035	2604	A	T	synonymous
423977	NTHI477_RS02035	2615	C	T	nonsynonymous
423978	NTHI477_RS02035	2616	G	A	synonymous
423984	NTHI477_RS02035	2622	C	T	synonymous

423987	NTHI477_RS02035	2625	T	C	synonymous
423990	NTHI477_RS02035	2628	G	A	synonymous
424029	intergenic	intergenic	G	A	intergenic
424031	intergenic	intergenic	A	G	intergenic
424042	intergenic	intergenic	A	T	intergenic
424047	intergenic	intergenic	A	T	intergenic
424048	intergenic	intergenic	C	T	intergenic
424049	intergenic	intergenic	A	T	intergenic
424051	intergenic	intergenic	T	G	intergenic
424054	intergenic	intergenic	G	A	intergenic
424055	intergenic	intergenic	T	A	intergenic
424056	intergenic	intergenic	T	A	intergenic
424059	intergenic	intergenic	A	T	intergenic
424101	NTHI477_RS02040	16	C	A	nonsynonymous
424103	NTHI477_RS02040	18	A	T	nonsynonymous
424109	NTHI477_RS02040	24	T	A	synonymous
424121	NTHI477_RS02040	36	T	C	synonymous
424145	NTHI477_RS02040	60	G	C	synonymous
424154	NTHI477_RS02040	69	C	T	synonymous
424157	NTHI477_RS02040	72	A	G	synonymous
424160	NTHI477_RS02040	75	C	A	synonymous
424184	NTHI477_RS02040	99	T	C	synonymous
424187	NTHI477_RS02040	102	C	T	synonymous
424199	NTHI477_RS02040	114	A	G	synonymous
424223	NTHI477_RS02040	138	G	C	synonymous
424226	NTHI477_RS02040	141	T	A	synonymous
424238	NTHI477_RS02040	153	G	T	synonymous
424254	NTHI477_RS02040	169	A	G	nonsynonymous
424259	NTHI477_RS02040	174	A	G	synonymous
424262	NTHI477_RS02040	177	G	C	synonymous
424320	NTHI477_RS02040	235	G	A	nonsynonymous
424325	NTHI477_RS02040	240	T	A	synonymous
424330	NTHI477_RS02040	245	C	T	nonsynonymous
424339	NTHI477_RS02040	254	A	C	nonsynonymous
424354	NTHI477_RS02040	269	T	C	nonsynonymous
424363	NTHI477_RS02040	278	C	A	nonsynonymous
424371	NTHI477_RS02040	286	G	A	nonsynonymous
424378	NTHI477_RS02040	293	T	C	nonsynonymous
424397	NTHI477_RS02040	312	G	A	synonymous
424563	NTHI477_RS02040	478	G	A	nonsynonymous
424632	NTHI477_RS02040	547	C	G	nonsynonymous
424633	NTHI477_RS02040	548	T	C	nonsynonymous
424637	NTHI477_RS02040	552	T	C	synonymous
424638	NTHI477_RS02040	553	G	A	nonsynonymous
424650	NTHI477_RS02040	565	G	A	nonsynonymous

424658	NTHI477_RS02040	573	A	T	synonymous
424663	NTHI477_RS02040	578	C	A	nonsynonymous
424664	NTHI477_RS02040	579	T	A	synonymous
424665	NTHI477_RS02040	580	T	C	nonsynonymous
424668	NTHI477_RS02040	583	A	G	nonsynonymous
424674	NTHI477_RS02040	589	G	A	nonsynonymous
424683	NTHI477_RS02040	598	A	G	nonsynonymous
424696	NTHI477_RS02040	611	A	C	nonsynonymous
424697	NTHI477_RS02040	612	T	A	nonsynonymous
424698	NTHI477_RS02040	613	A	C	nonsynonymous
424713	NTHI477_RS02040	628	G	C	nonsynonymous
424724	NTHI477_RS02040	639	T	G	nonsynonymous
424772	NTHI477_RS02040	687	A	C	synonymous
424793	NTHI477_RS02040	708	T	A	synonymous
424808	NTHI477_RS02040	723	T	A	synonymous
424820	NTHI477_RS02040	735	T	C	synonymous
424826	NTHI477_RS02040	741	T	C	synonymous
424827	NTHI477_RS02040	742	C	T	synonymous
424847	NTHI477_RS02040	762	T	G	synonymous
424865	NTHI477_RS02040	780	C	T	synonymous
424877	NTHI477_RS02040	792	T	C	synonymous
424910	NTHI477_RS02040	825	T	C	synonymous
424922	NTHI477_RS02040	837	T	C	synonymous
424931	NTHI477_RS02040	846	A	C	synonymous
424935	NTHI477_RS02040	850	G	A	nonsynonymous
424937	NTHI477_RS02040	852	T	A	synonymous
424938	NTHI477_RS02040	853	A	T	nonsynonymous
424940	NTHI477_RS02040	855	T	A	synonymous
424949	NTHI477_RS02040	864	T	C	synonymous
424958	NTHI477_RS02040	873	T	C	synonymous
424967	NTHI477_RS02040	882	A	C	synonymous
425084	NTHI477_RS02040	999	A	G	nonsynonymous
425105	NTHI477_RS02040	1020	C	T	synonymous
425123	NTHI477_RS02040	1038	G	T	synonymous
425135	NTHI477_RS02040	1050	A	C	synonymous
425155	NTHI477_RS02040	1070	C	T	nonsynonymous
425156	NTHI477_RS02040	1071	G	A	synonymous
425177	NTHI477_RS02040	1092	C	T	synonymous
425180	NTHI477_RS02040	1095	C	T	synonymous
425204	NTHI477_RS02040	1119	C	T	synonymous
425216	NTHI477_RS02040	1131	G	A	synonymous
425218	NTHI477_RS02040	1133	T	C	nonsynonymous
425237	NTHI477_RS02040	1152	G	C	synonymous
425240	NTHI477_RS02040	1155	T	C	synonymous
425243	NTHI477_RS02040	1158	A	T	synonymous

425252	NTHI477_RS02040	1167	T	C	synonymous
425273	NTHI477_RS02040	1188	T	A	synonymous
425279	NTHI477_RS02040	1194	C	T	synonymous
425286	NTHI477_RS02040	1201	C	T	synonymous
425297	NTHI477_RS02040	1212	T	C	synonymous
425303	NTHI477_RS02040	1218	C	T	synonymous
425306	NTHI477_RS02040	1221	C	T	synonymous
425312	NTHI477_RS02040	1227	C	T	synonymous
425315	NTHI477_RS02040	1230	A	T	synonymous
425324	NTHI477_RS02040	1239	G	A	synonymous
425327	NTHI477_RS02040	1242	G	A	synonymous
425339	NTHI477_RS02040	1254	C	A	synonymous
425342	NTHI477_RS02040	1257	G	T	synonymous
425345	NTHI477_RS02040	1260	T	A	synonymous
425355	NTHI477_RS02040	1270	A	G	nonsynonymous
425360	NTHI477_RS02040	1275	G	A	synonymous
425375	NTHI477_RS02040	1290	T	C	synonymous
425378	NTHI477_RS02040	1293	C	T	synonymous
425405	NTHI477_RS02040	1320	A	G	synonymous
425417	NTHI477_RS02040	1332	A	C	synonymous
425441	NTHI477_RS02040	1356	T	A	synonymous
425453	NTHI477_RS02040	1368	T	C	synonymous
425462	NTHI477_RS02040	1377	C	T	synonymous
425477	NTHI477_RS02040	1392	T	C	synonymous
425495	NTHI477_RS02040	1410	G	T	synonymous
425495	NTHI477_RS02040	1410	G	C	synonymous
425498	NTHI477_RS02040	1413	C	T	synonymous
425504	NTHI477_RS02040	1419	G	A	synonymous
425516	NTHI477_RS02040	1431	T	C	synonymous
425519	NTHI477_RS02040	1434	G	A	synonymous
425528	NTHI477_RS02040	1443	A	T	synonymous
425531	NTHI477_RS02040	1446	T	C	synonymous
425537	NTHI477_RS02040	1452	T	C	synonymous
425555	NTHI477_RS02040	1470	A	G	synonymous
425561	NTHI477_RS02040	1476	G	A	synonymous
425573	NTHI477_RS02040	1488	C	T	synonymous
425588	NTHI477_RS02040	1503	G	A	synonymous
425606	NTHI477_RS02040	1521	A	G	synonymous
425621	NTHI477_RS02040	1536	C	T	synonymous
425645	NTHI477_RS02040	1560	A	T	synonymous
425645	NTHI477_RS02040	1560	A	G	synonymous
425651	NTHI477_RS02040	1566	A	C	synonymous
425651	NTHI477_RS02040	1566	A	T	synonymous
425696	NTHI477_RS02040	1611	T	C	synonymous
425714	intergenic	intergenic	T	C	intergenic

425715	intergenic	intergenic	G	A	intergenic
425728	intergenic	intergenic	A	C	intergenic
425737	intergenic	intergenic	G	T	intergenic
425741	intergenic	intergenic	A	G	intergenic
425760	intergenic	intergenic	A	T	intergenic
425761	intergenic	intergenic	G	A	intergenic
425764	intergenic	intergenic	A	G	intergenic
425769	intergenic	intergenic	T	C	intergenic
425774	intergenic	intergenic	T	C	intergenic
425799	intergenic	intergenic	A	G	intergenic
425804	intergenic	intergenic	T	G	intergenic
425875	NTHI477_RS02045	51	C	A	synonymous
426028	NTHI477_RS02045	204	C	T	synonymous
426049	NTHI477_RS02045	225	T	C	synonymous
426061	NTHI477_RS02045	237	A	T	synonymous
426070	NTHI477_RS02045	246	G	T	nonsynonymous
426082	NTHI477_RS02045	258	A	T	synonymous
426091	NTHI477_RS02045	267	T	A	synonymous
426100	NTHI477_RS02045	276	T	C	synonymous
426118	NTHI477_RS02045	294	T	A	synonymous
426130	NTHI477_RS02045	306	A	G	synonymous
426139	NTHI477_RS02045	315	A	G	synonymous
426145	NTHI477_RS02045	321	A	G	synonymous
426176	NTHI477_RS02045	352	A	G	nonsynonymous
426188	NTHI477_RS02045	364	T	C	nonsynonymous
426193	NTHI477_RS02045	369	C	T	synonymous
426223	NTHI477_RS02045	399	C	T	synonymous
426307	NTHI477_RS02045	483	T	A	synonymous
426331	NTHI477_RS02045	507	A	G	synonymous
426334	NTHI477_RS02045	510	T	C	synonymous
426373	NTHI477_RS02045	549	C	A	synonymous
426388	NTHI477_RS02045	564	T	C	synonymous
426439	NTHI477_RS02045	615	T	G	synonymous
426463	NTHI477_RS02045	639	C	T	synonymous
426517	NTHI477_RS02045	693	C	T	synonymous
426544	NTHI477_RS02045	720	T	G	synonymous
426552	NTHI477_RS02045	728	A	C	nonsynonymous
426568	NTHI477_RS02045	744	T	C	synonymous
426574	NTHI477_RS02045	750	C	T	synonymous
426595	NTHI477_RS02045	771	C	A	synonymous
426598	NTHI477_RS02045	774	T	C	synonymous
426604	NTHI477_RS02045	780	C	T	synonymous
426607	NTHI477_RS02045	783	C	A	synonymous
426622	NTHI477_RS02045	798	A	G	synonymous
426625	NTHI477_RS02045	801	A	G	synonymous

426625	NTHI477_RS02045	801	A	T	synonymous
426628	NTHI477_RS02045	804	T	G	synonymous
426631	NTHI477_RS02045	807	C	T	synonymous
426682	NTHI477_RS02045	858	C	T	synonymous
426691	NTHI477_RS02045	867	A	G	synonymous
426733	NTHI477_RS02045	909	C	A	synonymous
426736	NTHI477_RS02045	912	C	T	synonymous
426739	NTHI477_RS02045	915	G	A	synonymous
426751	NTHI477_RS02045	927	C	T	synonymous
426760	NTHI477_RS02045	936	G	A	synonymous
426832	NTHI477_RS02045	1008	A	G	synonymous
426856	NTHI477_RS02045	1032	T	C	synonymous
426937	NTHI477_RS02045	1113	C	T	synonymous
426952	NTHI477_RS02045	1128	G	C	synonymous
426982	NTHI477_RS02045	1158	C	T	synonymous
426991	NTHI477_RS02045	1167	A	T	synonymous
427018	NTHI477_RS02045	1194	G	A	synonymous
427039	NTHI477_RS02045	1215	G	A	synonymous
427099	NTHI477_RS02045	1275	T	C	synonymous
427103	NTHI477_RS02045	1279	T	C	synonymous
427105	NTHI477_RS02045	1281	G	T	nonsynonymous
427108	NTHI477_RS02045	1284	A	C	synonymous
427156	NTHI477_RS02045	1332	A	T	synonymous
427165	NTHI477_RS02045	1341	G	T	synonymous
427171	NTHI477_RS02045	1347	T	C	synonymous
427177	NTHI477_RS02045	1353	T	C	synonymous
427183	NTHI477_RS02045	1359	C	T	synonymous
427188	NTHI477_RS02045	1364	C	T	nonsynonymous
427213	NTHI477_RS02045	1389	T	C	synonymous
427222	NTHI477_RS02045	1398	T	G	synonymous
427252	intergenic	intergenic	C	T	intergenic
427253	intergenic	intergenic	T	C	intergenic
427257	intergenic	intergenic	T	C	intergenic
427258	intergenic	intergenic	G	T	intergenic
427264	intergenic	intergenic	T	G	intergenic
427274	intergenic	intergenic	T	A	intergenic
427275	intergenic	intergenic	G	T	intergenic
427280	intergenic	intergenic	G	A	intergenic
427284	intergenic	intergenic	A	G	intergenic
427285	intergenic	intergenic	A	T	intergenic
427289	intergenic	intergenic	T	C	intergenic
427292	intergenic	intergenic	C	A	intergenic
427294	intergenic	intergenic	G	A	intergenic
427296	intergenic	intergenic	C	T	intergenic
427315	intergenic	intergenic	T	A	intergenic

427364	intergenic	intergenic	A	G	intergenic
427365	intergenic	intergenic	T	C	intergenic
427370	intergenic	intergenic	A	G	intergenic
427379	intergenic	intergenic	T	A	intergenic
427380	intergenic	intergenic	T	A	intergenic
427432	NTHI477_RS02050	24	C	T	synonymous
427459	NTHI477_RS02050	51	T	C	synonymous
427483	NTHI477_RS02050	75	C	T	synonymous
427495	NTHI477_RS02050	87	G	T	synonymous
427558	NTHI477_RS02050	150	C	A	synonymous
427561	NTHI477_RS02050	153	T	C	synonymous
427570	NTHI477_RS02050	162	T	C	synonymous
427573	NTHI477_RS02050	165	C	A	synonymous
427579	NTHI477_RS02050	171	G	T	synonymous
427579	NTHI477_RS02050	171	G	A	synonymous
427585	NTHI477_RS02050	177	A	G	synonymous
427588	NTHI477_RS02050	180	T	A	synonymous
427612	NTHI477_RS02050	204	T	C	synonymous
427615	NTHI477_RS02050	207	C	A	synonymous
427618	NTHI477_RS02050	210	C	G	synonymous
427621	NTHI477_RS02050	213	T	A	synonymous
427624	NTHI477_RS02050	216	C	T	synonymous
427630	NTHI477_RS02050	222	A	C	synonymous
427631	NTHI477_RS02050	223	C	T	synonymous
427636	NTHI477_RS02050	228	T	A	synonymous
427639	NTHI477_RS02050	231	A	G	synonymous
427645	NTHI477_RS02050	237	C	T	synonymous
427654	NTHI477_RS02050	246	A	G	synonymous
427654	NTHI477_RS02050	246	A	C	synonymous
427672	NTHI477_RS02050	264	A	G	synonymous
427681	NTHI477_RS02050	273	G	A	synonymous
427690	NTHI477_RS02050	282	T	G	synonymous
427726	NTHI477_RS02050	318	G	A	synonymous
427751	NTHI477_RS02050	343	G	C	nonsynonymous
427756	NTHI477_RS02050	348	T	A	synonymous
427759	NTHI477_RS02050	351	C	T	synonymous
427792	NTHI477_RS02050	384	T	A	synonymous
427795	NTHI477_RS02050	387	T	C	synonymous
427801	NTHI477_RS02050	393	A	C	synonymous
427810	NTHI477_RS02050	402	G	A	synonymous
427813	NTHI477_RS02050	405	A	G	synonymous
427822	NTHI477_RS02050	414	A	G	synonymous
427840	NTHI477_RS02050	432	A	C	synonymous
427841	NTHI477_RS02050	433	G	A	nonsynonymous
427843	NTHI477_RS02050	435	G	T	synonymous

427862	NTHI477_RS02050	454	G	A	nonsynonymous
427948	NTHI477_RS02050	540	T	C	synonymous
427989	NTHI477_RS02055	27	G	A	synonymous
428031	NTHI477_RS02055	69	T	C	synonymous
428080	NTHI477_RS02055	118	G	T	nonsynonymous
428082	NTHI477_RS02055	120	T	C	synonymous
428085	NTHI477_RS02055	123	C	T	synonymous
428094	NTHI477_RS02055	132	A	T	synonymous
428097	NTHI477_RS02055	135	C	T	synonymous
428106	NTHI477_RS02055	144	T	A	synonymous
428112	NTHI477_RS02055	150	A	G	synonymous
428136	NTHI477_RS02055	174	C	T	synonymous
428139	NTHI477_RS02055	177	G	A	synonymous
428157	NTHI477_RS02055	195	T	A	synonymous
428160	NTHI477_RS02055	198	C	T	synonymous
428163	NTHI477_RS02055	201	T	C	synonymous
428169	NTHI477_RS02055	207	G	A	synonymous
428175	NTHI477_RS02055	213	G	A	synonymous
428175	NTHI477_RS02055	213	G	T	synonymous
428175	NTHI477_RS02055	213	G	C	synonymous
428184	NTHI477_RS02055	222	C	A	synonymous
428203	NTHI477_RS02055	241	A	G	nonsynonymous
428205	NTHI477_RS02055	243	T	C	synonymous
428205	NTHI477_RS02055	243	T	G	synonymous
428208	NTHI477_RS02055	246	T	C	synonymous
428223	NTHI477_RS02055	261	T	C	synonymous
428250	NTHI477_RS02055	288	G	A	synonymous
428253	NTHI477_RS02055	291	T	C	synonymous
428253	NTHI477_RS02055	291	T	A	synonymous
428274	NTHI477_RS02055	312	G	C	synonymous
428286	NTHI477_RS02055	324	G	A	synonymous
428307	NTHI477_RS02055	345	A	T	synonymous
428313	NTHI477_RS02055	351	G	C	synonymous
428316	NTHI477_RS02055	354	C	A	synonymous
428337	NTHI477_RS02055	375	C	T	synonymous
428346	NTHI477_RS02055	384	C	T	synonymous
428361	NTHI477_RS02055	399	T	C	synonymous
428385	NTHI477_RS02055	423	A	G	synonymous
428391	NTHI477_RS02055	429	A	G	synonymous
428409	NTHI477_RS02055	447	T	C	synonymous
428418	NTHI477_RS02055	456	T	C	synonymous
428427	NTHI477_RS02055	465	A	G	synonymous
428433	NTHI477_RS02055	471	A	G	synonymous
428446	NTHI477_RS02055	484	C	T	synonymous
428469	NTHI477_RS02055	507	C	T	synonymous

428472	NTHI477_RS02055	510	T	C	synonymous
428475	NTHI477_RS02055	513	A	G	synonymous
428505	NTHI477_RS02055	543	T	C	synonymous
428511	NTHI477_RS02055	549	C	A	synonymous
428533	NTHI477_RS02055	571	T	C	synonymous
428562	NTHI477_RS02055	600	A	T	nonsynonymous
428562	NTHI477_RS02055	600	A	G	synonymous
428565	NTHI477_RS02055	603	T	C	synonymous
428589	NTHI477_RS02055	627	A	G	synonymous
428592	NTHI477_RS02055	630	A	G	synonymous
428613	NTHI477_RS02055	651	C	T	synonymous
428661	NTHI477_RS02055	699	A	G	synonymous
428666	NTHI477_RS02055	704	A	G	nonsynonymous
428670	NTHI477_RS02055	708	T	G	synonymous
428676	NTHI477_RS02055	714	C	T	synonymous
428733	NTHI477_RS02055	771	T	C	synonymous
428745	NTHI477_RS02055	783	C	T	synonymous
428766	NTHI477_RS02055	804	A	G	synonymous
428778	NTHI477_RS02055	816	T	A	synonymous
428799	NTHI477_RS02055	837	T	C	synonymous
428841	NTHI477_RS02055	879	G	A	synonymous
428844	NTHI477_RS02055	882	T	C	synonymous
428875	NTHI477_RS02055	913	A	G	nonsynonymous
428878	NTHI477_RS02055	916	A	C	nonsynonymous
428920	NTHI477_RS02055	958	A	G	nonsynonymous
428943	NTHI477_RS02055	981	C	G	synonymous
428976	NTHI477_RS02055	1014	A	G	synonymous
428994	NTHI477_RS02055	1032	T	A	synonymous
428994	NTHI477_RS02055	1032	T	C	synonymous
429000	NTHI477_RS02055	1038	G	A	synonymous
429058	NTHI477_RS02055	1096	G	A	nonsynonymous
429066	NTHI477_RS02055	1104	C	T	synonymous
429069	NTHI477_RS02055	1107	T	A	synonymous
429090	NTHI477_RS02055	1128	G	A	synonymous
429111	NTHI477_RS02055	1149	G	A	synonymous
429111	NTHI477_RS02055	1149	G	T	synonymous
429113	NTHI477_RS02055	1151	G	A	nonsynonymous
429123	NTHI477_RS02055	1161	C	A	synonymous
429129	NTHI477_RS02055	1167	G	A	synonymous
429141	NTHI477_RS02055	1179	G	T	synonymous
429146	NTHI477_RS02055	1184	G	A	nonsynonymous
429150	NTHI477_RS02055	1188	C	A	synonymous
429156	NTHI477_RS02055	1194	T	A	synonymous
429170	NTHI477_RS02055	1208	T	C	nonsynonymous
429175	NTHI477_RS02055	1213	C	T	nonsynonymous

429189	NTHI477_RS02055	1227	C	T	synonymous
429213	NTHI477_RS02055	1251	G	C	synonymous
429228	NTHI477_RS02055	1266	T	G	synonymous
429231	NTHI477_RS02055	1269	A	G	synonymous
429244	NTHI477_RS02055	1282	C	T	synonymous
429246	NTHI477_RS02055	1284	G	C	synonymous
429303	NTHI477_RS02055	1341	C	T	synonymous
429324	NTHI477_RS02055	1362	A	G	synonymous
429327	NTHI477_RS02055	1365	C	T	synonymous
429363	NTHI477_RS02055	1401	C	T	synonymous
429381	NTHI477_RS02055	1419	G	A	synonymous
429384	NTHI477_RS02055	1422	G	A	synonymous
429405	NTHI477_RS02055	1443	G	A	synonymous
429412	NTHI477_RS02055	1450	G	T	nonsynonymous
429413	NTHI477_RS02055	1451	T	C	nonsynonymous
429416	NTHI477_RS02055	1454	A	C	nonsynonymous
429418	NTHI477_RS02055	1456	C	T	nonsynonymous
429432	NTHI477_RS02055	1470	G	A	synonymous
429442	NTHI477_RS02055	1480	A	G	nonsynonymous
429479	NTHI477_RS02055	1517	T	C	nonsynonymous
429484	NTHI477_RS02055	1522	A	G	nonsynonymous
429487	NTHI477_RS02055	1525	G	A	nonsynonymous
429498	NTHI477_RS02055	1536	G	A	synonymous
429518	NTHI477_RS02055	1556	A	G	nonsynonymous
429574	NTHI477_RS02055	1612	C	T	nonsynonymous
429639	NTHI477_RS02055	1677	T	C	synonymous
429643	NTHI477_RS02055	1681	A	C	nonsynonymous
429681	NTHI477_RS02055	1719	G	A	synonymous
429691	NTHI477_RS02055	1729	T	G	nonsynonymous
429717	NTHI477_RS02055	1755	G	A	synonymous
429770	NTHI477_RS02055	1808	A	G	nonsynonymous
429783	NTHI477_RS02055	1821	C	T	synonymous
429803	NTHI477_RS02055	1841	G	A	nonsynonymous
429816	NTHI477_RS02055	1854	A	G	synonymous
429818	NTHI477_RS02055	1856	C	T	nonsynonymous
429825	NTHI477_RS02055	1863	A	T	synonymous
429831	NTHI477_RS02055	1869	C	T	synonymous
429837	NTHI477_RS02055	1875	A	G	synonymous
429864	NTHI477_RS02055	1902	T	C	synonymous
429886	NTHI477_RS02055	1924	A	G	nonsynonymous
429909	NTHI477_RS02055	1947	C	T	synonymous
429943	NTHI477_RS02055	1981	A	C	nonsynonymous
429948	NTHI477_RS02055	1986	G	A	synonymous
429963	NTHI477_RS02055	2001	T	C	synonymous
429967	NTHI477_RS02055	2005	T	C	synonymous

429975	NTHI477_RS02055	2013	T	G	nonsynonymous
429976	NTHI477_RS02055	2014	C	T	synonymous
429978	NTHI477_RS02055	2016	A	G	synonymous
429987	NTHI477_RS02055	2025	G	A	synonymous
429990	NTHI477_RS02055	2028	T	C	synonymous
430005	NTHI477_RS02055	2043	T	C	synonymous
430006	NTHI477_RS02055	2044	G	A	nonsynonymous
430011	NTHI477_RS02055	2049	G	A	synonymous
430042	intergenic	intergenic	A	G	intergenic
430045	intergenic	intergenic	G	C	intergenic
430049	intergenic	intergenic	A	T	intergenic
430050	intergenic	intergenic	C	T	intergenic
430055	intergenic	intergenic	G	A	intergenic
430059	intergenic	intergenic	C	A	intergenic
430062	intergenic	intergenic	G	A	intergenic
430078	intergenic	intergenic	A	T	intergenic
430081	intergenic	intergenic	C	G	intergenic
430081	intergenic	intergenic	C	A	intergenic
430098	intergenic	intergenic	T	G	intergenic
430110	intergenic	intergenic	G	A	intergenic
430115	intergenic	intergenic	T	C	intergenic
430124	intergenic	intergenic	G	A	intergenic
430136	intergenic	intergenic	T	C	intergenic
430149	intergenic	intergenic	A	G	intergenic
430161	NTHI477_RS02060	9	G	A	synonymous
430170	NTHI477_RS02060	18	A	C	synonymous
430172	NTHI477_RS02060	20	G	A	nonsynonymous
430176	NTHI477_RS02060	24	T	C	synonymous
430245	NTHI477_RS02060	93	T	G	synonymous
430257	NTHI477_RS02060	105	C	T	synonymous
430318	NTHI477_RS02060	166	G	A	nonsynonymous
430329	NTHI477_RS02060	177	G	C	synonymous
430332	NTHI477_RS02060	180	A	T	synonymous
430341	NTHI477_RS02060	189	C	T	synonymous
430374	NTHI477_RS02060	222	A	G	synonymous
430389	NTHI477_RS02060	237	A	T	synonymous
430398	NTHI477_RS02060	246	A	T	synonymous
430422	NTHI477_RS02060	270	C	T	synonymous
430431	NTHI477_RS02060	279	C	T	synonymous
430434	NTHI477_RS02060	282	A	G	synonymous
430437	NTHI477_RS02060	285	C	T	synonymous
430443	NTHI477_RS02060	291	C	T	synonymous
430452	NTHI477_RS02060	300	G	A	synonymous
430477	NTHI477_RS02060	325	G	A	nonsynonymous
430479	NTHI477_RS02060	327	A	G	synonymous

430485	NTHI477_RS02060	333	T	C	synonymous
430491	NTHI477_RS02060	339	C	T	synonymous
430494	NTHI477_RS02060	342	C	T	synonymous
430506	NTHI477_RS02060	354	T	C	synonymous
430521	NTHI477_RS02060	369	G	A	synonymous
430524	NTHI477_RS02060	372	A	T	synonymous
430528	NTHI477_RS02060	376	T	G	nonsynonymous
430545	NTHI477_RS02060	393	G	A	synonymous
430557	NTHI477_RS02060	405	G	A	synonymous
430563	NTHI477_RS02060	411	C	T	synonymous
430575	NTHI477_RS02060	423	C	A	synonymous
430581	NTHI477_RS02060	429	T	C	synonymous
430632	NTHI477_RS02060	480	C	G	synonymous
430647	NTHI477_RS02060	495	A	T	synonymous
430653	NTHI477_RS02060	501	A	G	synonymous
430656	NTHI477_RS02060	504	T	A	synonymous
430662	NTHI477_RS02060	510	G	A	synonymous
430663	NTHI477_RS02060	511	G	A	nonsynonymous
430668	NTHI477_RS02060	516	G	A	synonymous
430671	NTHI477_RS02060	519	C	T	synonymous
430686	NTHI477_RS02060	534	T	C	synonymous
430692	NTHI477_RS02060	540	C	T	synonymous
430737	NTHI477_RS02060	585	C	G	synonymous
430833	intergenic	intergenic	G	A	intergenic
430897	NTHI477_RS02065	15	C	T	synonymous
430899	NTHI477_RS02065	17	C	T	nonsynonymous
430902	NTHI477_RS02065	20	C	T	nonsynonymous
430905	NTHI477_RS02065	23	C	T	nonsynonymous
430910	NTHI477_RS02065	28	G	T	nonsynonymous
430948	NTHI477_RS02065	66	T	C	synonymous
430951	NTHI477_RS02065	69	G	A	synonymous
430960	NTHI477_RS02065	78	T	C	synonymous
430963	NTHI477_RS02065	81	T	C	synonymous
430966	NTHI477_RS02065	84	T	C	synonymous
430978	NTHI477_RS02065	96	A	G	synonymous
430981	NTHI477_RS02065	99	A	T	synonymous
430984	NTHI477_RS02065	102	A	T	synonymous
430987	NTHI477_RS02065	105	C	T	synonymous
430990	NTHI477_RS02065	108	G	A	synonymous
431005	NTHI477_RS02065	123	C	A	synonymous
431032	NTHI477_RS02065	150	G	C	synonymous
431032	NTHI477_RS02065	150	G	A	synonymous
431035	NTHI477_RS02065	153	A	T	synonymous
431047	NTHI477_RS02065	165	C	T	synonymous
431053	NTHI477_RS02065	171	C	T	synonymous

431065	NTHI477_RS02065	183	C	T	synonymous
431074	NTHI477_RS02065	192	A	G	synonymous
431131	NTHI477_RS02065	249	C	T	synonymous
431134	NTHI477_RS02065	252	A	T	synonymous
431146	NTHI477_RS02065	264	A	C	synonymous
431146	NTHI477_RS02065	264	A	T	synonymous
431164	NTHI477_RS02065	282	C	T	synonymous
431176	NTHI477_RS02065	294	C	T	synonymous
431182	NTHI477_RS02065	300	T	G	synonymous
431200	NTHI477_RS02065	318	G	A	synonymous
431203	NTHI477_RS02065	321	C	T	synonymous
431206	NTHI477_RS02065	324	T	G	synonymous
431207	NTHI477_RS02065	325	T	C	synonymous
431224	NTHI477_RS02065	342	A	T	synonymous
431234	NTHI477_RS02065	352	T	C	synonymous
431308	NTHI477_RS02065	426	G	T	synonymous
431320	NTHI477_RS02065	438	C	A	synonymous
431326	NTHI477_RS02065	444	C	T	synonymous
431336	NTHI477_RS02065	454	G	A	nonsynonymous
431337	NTHI477_RS02065	455	C	A	nonsynonymous
431338	NTHI477_RS02065	456	T	C	synonymous
431353	NTHI477_RS02065	471	C	T	synonymous
431356	NTHI477_RS02065	474	T	A	synonymous
431356	NTHI477_RS02065	474	T	G	synonymous
431357	NTHI477_RS02065	475	A	G	nonsynonymous
431377	NTHI477_RS02065	495	A	G	synonymous
431401	NTHI477_RS02065	519	A	T	synonymous
431425	NTHI477_RS02065	543	A	G	synonymous
431454	NTHI477_RS02065	572	C	T	nonsynonymous
431473	NTHI477_RS02065	591	A	G	synonymous
431479	NTHI477_RS02065	597	G	A	synonymous
431503	NTHI477_RS02065	621	G	T	synonymous
431512	NTHI477_RS02065	630	G	A	synonymous
431530	NTHI477_RS02065	648	C	A	synonymous
431563	NTHI477_RS02065	681	C	T	synonymous
431587	NTHI477_RS02065	705	T	C	synonymous
431596	NTHI477_RS02065	714	C	T	synonymous
431596	NTHI477_RS02065	714	C	A	synonymous
431599	NTHI477_RS02065	717	C	A	synonymous
431611	NTHI477_RS02065	729	G	T	synonymous
431614	NTHI477_RS02065	732	T	C	synonymous
431617	NTHI477_RS02065	735	T	C	synonymous
431626	NTHI477_RS02065	744	G	A	synonymous
431629	NTHI477_RS02065	747	T	C	synonymous
431635	NTHI477_RS02065	753	G	T	synonymous

431638	NTHI477_RS02065	756	A	G	synonymous
431650	NTHI477_RS02065	768	C	T	synonymous
431654	NTHI477_RS02065	772	T	C	synonymous
431665	NTHI477_RS02065	783	A	G	synonymous
431704	NTHI477_RS02065	822	C	T	synonymous
431710	NTHI477_RS02065	828	T	A	synonymous
431713	NTHI477_RS02065	831	C	T	synonymous
431722	NTHI477_RS02065	840	T	G	synonymous
431725	NTHI477_RS02065	843	T	C	synonymous
431728	NTHI477_RS02065	846	C	T	synonymous
431761	NTHI477_RS02065	879	C	T	synonymous
431791	NTHI477_RS02065	909	A	C	synonymous
431812	NTHI477_RS02065	930	A	T	synonymous
431839	NTHI477_RS02065	957	T	C	synonymous
431875	NTHI477_RS02065	993	T	G	synonymous
431923	NTHI477_RS02065	1041	T	C	synonymous
431932	NTHI477_RS02065	1050	G	T	nonsynonymous
431934	NTHI477_RS02065	1052	G	A	nonsynonymous
431935	NTHI477_RS02065	1053	T	C	synonymous
431947	NTHI477_RS02065	1065	C	T	synonymous
431950	NTHI477_RS02065	1068	A	C	synonymous
431969	NTHI477_RS02065	1087	G	T	nonsynonymous
431971	NTHI477_RS02065	1089	T	G	synonymous
431971	NTHI477_RS02065	1089	T	A	synonymous
431980	NTHI477_RS02065	1098	G	T	synonymous
431980	NTHI477_RS02065	1098	G	A	synonymous
431995	NTHI477_RS02065	1113	C	T	synonymous
432047	NTHI477_RS02065	1165	A	C	nonsynonymous
432064	NTHI477_RS02065	1182	G	A	synonymous
432067	NTHI477_RS02065	1185	C	T	synonymous
432116	NTHI477_RS02065	1234	G	A	nonsynonymous
432128	NTHI477_RS02065	1246	G	T	nonsynonymous
432131	intergenic	intergenic	C	T	intergenic
432181	intergenic	intergenic	C	A	intergenic
432201	NTHI477_RS02070	9	G	A	synonymous
432204	NTHI477_RS02070	12	A	G	synonymous
432207	NTHI477_RS02070	15	C	T	synonymous
432211	NTHI477_RS02070	19	T	C	synonymous
432216	NTHI477_RS02070	24	T	C	synonymous
432229	NTHI477_RS02070	37	G	A	nonsynonymous
432237	NTHI477_RS02070	45	T	C	synonymous
432246	NTHI477_RS02070	54	G	A	synonymous
432279	NTHI477_RS02070	87	C	T	synonymous
432306	NTHI477_RS02070	114	T	A	nonsynonymous
432318	NTHI477_RS02070	126	G	T	synonymous

432327	NTHI477_RS02070	135	C	T	synonymous
432337	NTHI477_RS02070	145	G	A	nonsynonymous
432345	NTHI477_RS02070	153	C	T	synonymous
432348	NTHI477_RS02070	156	A	T	synonymous
432351	NTHI477_RS02070	159	T	A	synonymous
432357	NTHI477_RS02070	165	C	T	synonymous
432361	NTHI477_RS02070	169	T	C	synonymous
432363	NTHI477_RS02070	171	A	G	synonymous
432396	NTHI477_RS02070	204	T	A	nonsynonymous
432399	NTHI477_RS02070	207	A	G	synonymous
432408	NTHI477_RS02070	216	T	G	synonymous
432411	NTHI477_RS02070	219	T	C	synonymous
432417	NTHI477_RS02070	225	C	A	synonymous
432417	NTHI477_RS02070	225	C	T	synonymous
432420	NTHI477_RS02070	228	C	T	synonymous
432468	NTHI477_RS02070	276	G	A	synonymous
432471	NTHI477_RS02070	279	A	G	synonymous
432476	NTHI477_RS02070	284	A	C	nonsynonymous
432480	NTHI477_RS02070	288	A	G	synonymous
432504	NTHI477_RS02070	312	T	C	synonymous
432507	NTHI477_RS02070	315	A	C	synonymous
432510	NTHI477_RS02070	318	C	A	synonymous
432546	NTHI477_RS02070	354	G	A	synonymous
432549	NTHI477_RS02070	357	T	C	synonymous
432567	NTHI477_RS02070	375	T	C	synonymous
432570	NTHI477_RS02070	378	G	A	synonymous
432579	NTHI477_RS02070	387	T	G	synonymous
432603	NTHI477_RS02070	411	G	T	synonymous
432606	NTHI477_RS02070	414	T	C	synonymous
432621	NTHI477_RS02070	429	C	A	synonymous
432627	NTHI477_RS02070	435	G	A	synonymous
432648	NTHI477_RS02070	456	A	G	synonymous
432657	NTHI477_RS02070	465	G	C	synonymous
432687	NTHI477_RS02070	495	C	T	synonymous
432697	NTHI477_RS02070	505	A	G	nonsynonymous
432700	NTHI477_RS02070	508	A	G	nonsynonymous
432711	NTHI477_RS02070	519	A	G	synonymous
432713	NTHI477_RS02070	521	T	A	nonsynonymous
432714	NTHI477_RS02070	522	C	T	synonymous
432715	NTHI477_RS02070	523	G	A	nonsynonymous
432796	NTHI477_RS02070	604	T	C	synonymous
432802	NTHI477_RS02070	610	G	A	nonsynonymous
432834	NTHI477_RS02070	642	A	G	synonymous
432840	NTHI477_RS02070	648	A	T	synonymous
432852	NTHI477_RS02070	660	T	C	synonymous

432855	NTHI477_RS02070	663	A	G	synonymous
432858	NTHI477_RS02070	666	T	C	synonymous
432864	NTHI477_RS02070	672	C	T	synonymous
432871	NTHI477_RS02070	679	G	A	nonsynonymous
432879	NTHI477_RS02070	687	G	A	synonymous
432882	NTHI477_RS02070	690	T	C	synonymous
432885	NTHI477_RS02070	693	C	A	synonymous
432899	intergenic	intergenic	G	A	intergenic
432906	intergenic	intergenic	C	T	intergenic
432912	intergenic	intergenic	T	C	intergenic
432927	NTHI477_RS02075	311	G	A	nonsynonymous
432952	NTHI477_RS02075	286	G	T	nonsynonymous
433018	NTHI477_RS02075	220	C	A	nonsynonymous
433022	NTHI477_RS02075	216	A	G	synonymous
433025	NTHI477_RS02075	213	A	G	synonymous
433040	NTHI477_RS02075	198	T	C	synonymous
433049	NTHI477_RS02075	189	A	T	synonymous
433050	NTHI477_RS02075	188	G	A	nonsynonymous
433052	NTHI477_RS02075	186	C	A	synonymous
433067	NTHI477_RS02075	171	T	A	nonsynonymous
433073	NTHI477_RS02075	165	A	G	synonymous
433082	NTHI477_RS02075	156	A	G	synonymous
433100	NTHI477_RS02075	138	T	C	synonymous
433109	NTHI477_RS02075	129	C	A	synonymous
433118	NTHI477_RS02075	120	T	C	synonymous
433127	NTHI477_RS02075	111	C	T	synonymous
433139	NTHI477_RS02075	99	G	A	synonymous
433170	NTHI477_RS02075	68	G	A	nonsynonymous
433199	NTHI477_RS02075	39	A	C	synonymous
433211	NTHI477_RS02075	27	A	T	synonymous
433227	NTHI477_RS02075	11	G	A	nonsynonymous
433242	intergenic	intergenic	A	G	intergenic
433304	NTHI477_RS02080	633	T	A	synonymous
433334	NTHI477_RS02080	603	A	T	synonymous
433361	NTHI477_RS02080	576	C	T	synonymous
433369	NTHI477_RS02080	568	G	T	synonymous
433376	NTHI477_RS02080	561	G	A	synonymous
433406	NTHI477_RS02080	531	T	C	synonymous
433409	NTHI477_RS02080	528	G	T	synonymous
433412	NTHI477_RS02080	525	C	A	synonymous
433424	NTHI477_RS02080	513	G	C	nonsynonymous
433429	NTHI477_RS02080	508	C	T	nonsynonymous
433430	NTHI477_RS02080	507	T	A	synonymous
433438	NTHI477_RS02080	499	C	T	nonsynonymous
433439	NTHI477_RS02080	498	A	G	synonymous

433463	NTHI477_RS02080	474	C	A	synonymous
433466	NTHI477_RS02080	471	A	G	synonymous
433469	NTHI477_RS02080	468	G	A	synonymous
433472	NTHI477_RS02080	465	A	G	synonymous
433475	NTHI477_RS02080	462	C	T	synonymous
433484	NTHI477_RS02080	453	C	T	synonymous
433490	NTHI477_RS02080	447	T	C	synonymous
433492	NTHI477_RS02080	445	C	A	nonsynonymous
433568	NTHI477_RS02080	369	G	A	synonymous
433595	NTHI477_RS02080	342	A	G	synonymous
433610	NTHI477_RS02080	327	C	T	synonymous
433628	NTHI477_RS02080	309	T	C	synonymous
433643	NTHI477_RS02080	294	C	T	synonymous
433646	NTHI477_RS02080	291	G	T	nonsynonymous
433646	NTHI477_RS02080	291	G	C	nonsynonymous
433664	NTHI477_RS02080	273	G	A	synonymous
433757	NTHI477_RS02080	180	C	A	synonymous
433778	NTHI477_RS02080	159	A	G	synonymous
433787	NTHI477_RS02080	150	A	G	synonymous
433796	NTHI477_RS02080	141	T	C	synonymous
433801	NTHI477_RS02080	136	C	T	nonsynonymous
433820	NTHI477_RS02080	117	A	G	synonymous
433894	NTHI477_RS02080	43	C	T	nonsynonymous
433904	NTHI477_RS02080	33	G	A	synonymous
433905	NTHI477_RS02080	32	A	T	nonsynonymous
433928	NTHI477_RS02080	9	A	G	synonymous
433938	intergenic	intergenic	A	G	intergenic
433955	intergenic	intergenic	G	A	intergenic
434029	NTHI477_RS02085	1125	C	T	synonymous
434041	NTHI477_RS02085	1113	G	A	synonymous
434089	NTHI477_RS02085	1065	T	C	synonymous
434091	NTHI477_RS02085	1063	G	T	synonymous
434095	NTHI477_RS02085	1059	A	G	synonymous
434121	NTHI477_RS02085	1033	G	A	synonymous
434122	NTHI477_RS02085	1032	A	G	synonymous
434134	NTHI477_RS02085	1020	A	C	synonymous
434137	NTHI477_RS02085	1017	C	T	synonymous
434140	NTHI477_RS02085	1014	G	C	nonsynonymous
434164	NTHI477_RS02085	990	C	T	synonymous
434197	NTHI477_RS02085	957	C	A	nonsynonymous
434203	NTHI477_RS02085	951	C	T	synonymous
434206	NTHI477_RS02085	948	G	A	synonymous
434212	NTHI477_RS02085	942	C	T	synonymous
434230	NTHI477_RS02085	924	A	T	synonymous
434230	NTHI477_RS02085	924	A	C	synonymous

434239	NTHI477_RS02085	915	C	T	synonymous
434242	NTHI477_RS02085	912	G	A	synonymous
434266	NTHI477_RS02085	888	A	G	synonymous
434267	NTHI477_RS02085	887	G	A	nonsynonymous
434269	NTHI477_RS02085	885	T	C	synonymous
434271	NTHI477_RS02085	883	G	A	synonymous
434272	NTHI477_RS02085	882	T	A	synonymous
434275	NTHI477_RS02085	879	C	T	synonymous
434278	NTHI477_RS02085	876	C	T	synonymous
434319	NTHI477_RS02085	835	A	G	synonymous
434356	NTHI477_RS02085	798	G	A	synonymous
434375	NTHI477_RS02085	779	A	G	nonsynonymous
434425	NTHI477_RS02085	729	T	A	synonymous
434430	NTHI477_RS02085	724	T	C	nonsynonymous
434452	NTHI477_RS02085	702	G	A	synonymous
434488	NTHI477_RS02085	666	G	A	synonymous
434503	NTHI477_RS02085	651	A	T	synonymous
434510	NTHI477_RS02085	644	G	A	nonsynonymous
434557	NTHI477_RS02085	597	C	T	synonymous
434647	NTHI477_RS02085	507	G	A	synonymous
434671	NTHI477_RS02085	483	T	C	synonymous
434716	NTHI477_RS02085	438	T	C	synonymous
434755	NTHI477_RS02085	399	A	T	synonymous
434794	NTHI477_RS02085	360	C	T	synonymous
434860	NTHI477_RS02085	294	G	A	synonymous
434869	NTHI477_RS02085	285	A	G	synonymous
434902	NTHI477_RS02085	252	G	A	synonymous
434911	NTHI477_RS02085	243	A	C	synonymous
434914	NTHI477_RS02085	240	T	C	synonymous
434917	NTHI477_RS02085	237	G	T	synonymous
434919	NTHI477_RS02085	235	C	T	nonsynonymous
434929	NTHI477_RS02085	225	G	A	synonymous
434935	NTHI477_RS02085	219	G	A	synonymous
434953	NTHI477_RS02085	201	G	A	synonymous
434970	NTHI477_RS02085	184	A	G	synonymous
435010	NTHI477_RS02085	144	A	G	synonymous
435052	NTHI477_RS02085	102	T	C	synonymous
435106	NTHI477_RS02085	48	G	A	synonymous
435157	NTHI477_RS02090	290	G	A	nonsynonymous
435272	NTHI477_RS02090	175	G	A	synonymous
435306	NTHI477_RS02090	141	A	T	synonymous
435372	NTHI477_RS02090	75	G	A	synonymous
435408	NTHI477_RS02090	39	A	C	synonymous
435426	NTHI477_RS02090	21	G	A	synonymous
435435	NTHI477_RS02090	12	A	C	nonsynonymous

435494	intergenic	intergenic	A	T	intergenic
435539	NTHI477_RS02095	267	T	C	synonymous
435541	NTHI477_RS02095	265	G	T	synonymous
435561	NTHI477_RS02095	245	G	A	nonsynonymous
435629	NTHI477_RS02095	177	A	C	synonymous
435647	NTHI477_RS02095	159	T	C	synonymous
435673	NTHI477_RS02095	133	T	C	nonsynonymous
435685	NTHI477_RS02095	121	C	T	nonsynonymous
435740	NTHI477_RS02095	66	A	G	synonymous
435747	NTHI477_RS02095	59	T	G	nonsynonymous
435754	NTHI477_RS02095	52	G	A	nonsynonymous
435761	NTHI477_RS02095	45	C	T	synonymous
435774	NTHI477_RS02095	32	A	G	nonsynonymous
435785	NTHI477_RS02095	21	T	C	nonsynonymous
435807	intergenic	intergenic	G	C	intergenic
435813	intergenic	intergenic	G	A	intergenic
435828	intergenic	intergenic	C	T	intergenic
435981	NTHI477_RS02100	1597	T	C	nonsynonymous
436003	NTHI477_RS02100	1575	G	A	synonymous
436063	NTHI477_RS02100	1515	A	G	synonymous
436081	NTHI477_RS02100	1497	C	T	synonymous
436087	NTHI477_RS02100	1491	A	G	synonymous
436096	NTHI477_RS02100	1482	T	A	nonsynonymous
436102	NTHI477_RS02100	1476	A	G	synonymous
436102	NTHI477_RS02100	1476	A	C	synonymous
436113	NTHI477_RS02100	1465	C	T	nonsynonymous
436124	NTHI477_RS02100	1454	T	C	nonsynonymous
436195	NTHI477_RS02100	1383	T	C	synonymous
436206	NTHI477_RS02100	1372	T	C	nonsynonymous
436234	NTHI477_RS02100	1344	G	A	synonymous
436243	NTHI477_RS02100	1335	G	A	synonymous
436246	NTHI477_RS02100	1332	G	A	synonymous
436402	NTHI477_RS02100	1176	G	A	synonymous
436417	NTHI477_RS02100	1161	G	A	synonymous
436492	NTHI477_RS02100	1086	G	A	synonymous
436501	NTHI477_RS02100	1077	A	T	nonsynonymous
436510	NTHI477_RS02100	1068	T	C	synonymous
436519	NTHI477_RS02100	1059	A	T	synonymous
436615	NTHI477_RS02100	963	C	A	synonymous
436627	NTHI477_RS02100	951	G	A	synonymous
436639	NTHI477_RS02100	939	A	G	synonymous
436657	NTHI477_RS02100	921	A	T	synonymous
436660	NTHI477_RS02100	918	A	T	synonymous
436693	NTHI477_RS02100	885	T	C	synonymous
436705	NTHI477_RS02100	873	G	A	synonymous

436714	NTHI477_RS02100	864	A	T	synonymous
436717	NTHI477_RS02100	861	T	C	synonymous
436753	NTHI477_RS02100	825	T	A	synonymous
436759	NTHI477_RS02100	819	A	G	synonymous
436774	NTHI477_RS02100	804	A	T	synonymous
436780	NTHI477_RS02100	798	A	T	synonymous
436816	NTHI477_RS02100	762	G	A	synonymous
436855	NTHI477_RS02100	723	T	A	synonymous
436921	NTHI477_RS02100	657	A	G	synonymous
436942	NTHI477_RS02100	636	G	A	synonymous
436945	NTHI477_RS02100	633	T	A	nonsynonymous
436947	NTHI477_RS02100	631	A	G	synonymous
436986	NTHI477_RS02100	592	C	T	nonsynonymous
437008	NTHI477_RS02100	570	A	C	synonymous
437086	NTHI477_RS02100	492	G	A	synonymous
437128	NTHI477_RS02100	450	T	C	synonymous
437131	NTHI477_RS02100	447	A	G	synonymous
437179	NTHI477_RS02100	399	A	G	synonymous
437311	NTHI477_RS02100	267	A	C	synonymous
437338	NTHI477_RS02100	240	G	A	synonymous
437347	NTHI477_RS02100	231	A	G	synonymous
437365	NTHI477_RS02100	213	A	T	synonymous
437419	NTHI477_RS02100	159	A	G	synonymous
437505	NTHI477_RS02100	73	C	T	nonsynonymous
437534	NTHI477_RS02100	44	C	A	nonsynonymous
437681	NTHI477_RS02105	671	T	A	nonsynonymous
437709	NTHI477_RS02105	643	C	A	nonsynonymous
437710	NTHI477_RS02105	642	T	C	synonymous
437713	NTHI477_RS02105	639	C	T	synonymous
437716	NTHI477_RS02105	636	C	T	synonymous
437718	NTHI477_RS02105	634	T	G	nonsynonymous
437719	NTHI477_RS02105	633	A	C	nonsynonymous
437722	NTHI477_RS02105	630	G	A	synonymous
437725	NTHI477_RS02105	627	C	T	synonymous
437725	NTHI477_RS02105	627	C	A	synonymous
437732	NTHI477_RS02105	620	T	C	nonsynonymous
437733	NTHI477_RS02105	619	C	T	nonsynonymous
437734	NTHI477_RS02105	618	G	A	synonymous
437735	NTHI477_RS02105	617	G	A	nonsynonymous
437744	NTHI477_RS02105	608	C	G	nonsynonymous
437745	NTHI477_RS02105	607	T	C	nonsynonymous
437749	NTHI477_RS02105	603	G	A	synonymous
437773	NTHI477_RS02105	579	T	C	synonymous
437814	NTHI477_RS02105	538	A	G	nonsynonymous
437936	NTHI477_RS02105	416	T	G	nonsynonymous

437953	NTHI477_RS02105	399	A	C	synonymous
437956	NTHI477_RS02105	396	T	A	synonymous
437956	NTHI477_RS02105	396	T	C	synonymous
437959	NTHI477_RS02105	393	T	C	synonymous
437971	NTHI477_RS02105	381	A	G	synonymous
437974	NTHI477_RS02105	378	A	G	synonymous
438022	NTHI477_RS02105	330	C	T	synonymous
438046	NTHI477_RS02105	306	A	G	synonymous
438078	NTHI477_RS02105	274	C	T	nonsynonymous
438085	NTHI477_RS02105	267	G	A	synonymous
438105	NTHI477_RS02105	247	C	T	nonsynonymous
438118	NTHI477_RS02105	234	C	A	nonsynonymous
438169	NTHI477_RS02105	183	T	G	synonymous
438201	NTHI477_RS02105	151	G	A	synonymous
438238	NTHI477_RS02105	114	A	G	synonymous
438264	NTHI477_RS02105	88	G	A	synonymous
438292	NTHI477_RS02105	60	A	G	synonymous
438310	NTHI477_RS02105	42	C	T	synonymous
438325	NTHI477_RS02105	27	G	A	synonymous
438325	NTHI477_RS02105	27	G	C	synonymous
438370	intergenic	intergenic	A	C	intergenic
438400	intergenic	intergenic	T	G	intergenic
438464	intergenic	intergenic	G	A	intergenic
438632	intergenic	intergenic	C	A	intergenic
438660	intergenic	intergenic	C	T	intergenic
438673	intergenic	intergenic	G	T	intergenic
438701	intergenic	intergenic	A	G	intergenic
438728	intergenic	intergenic	C	A	intergenic
438729	intergenic	intergenic	G	T	intergenic
438741	intergenic	intergenic	A	T	intergenic
438799	intergenic	intergenic	C	T	intergenic
438817	intergenic	intergenic	G	A	intergenic
438876	NTHI477_RS02110	57	T	A	synonymous
438903	NTHI477_RS02110	84	A	G	synonymous
438994	NTHI477_RS02110	175	C	A	synonymous
438996	NTHI477_RS02110	177	A	C	synonymous
439002	NTHI477_RS02110	183	C	T	synonymous
439083	NTHI477_RS02110	264	G	A	synonymous
439119	NTHI477_RS02110	300	C	T	synonymous
439125	NTHI477_RS02110	306	G	A	synonymous
439128	NTHI477_RS02110	309	T	C	synonymous
439152	NTHI477_RS02110	333	C	T	synonymous
439272	NTHI477_RS02110	453	A	G	synonymous
439319	NTHI477_RS02110	500	G	A	nonsynonymous
439359	NTHI477_RS02110	540	G	A	synonymous

439377	NTHI477_RS02110	558	A	G	synonymous
439392	NTHI477_RS02110	573	T	C	synonymous
439419	NTHI477_RS02110	600	G	C	nonsynonymous
439420	NTHI477_RS02110	601	C	A	nonsynonymous
439479	NTHI477_RS02110	660	G	A	synonymous
439536	NTHI477_RS02110	717	G	A	synonymous
439560	NTHI477_RS02110	741	T	G	synonymous
439563	NTHI477_RS02110	744	C	T	synonymous
439611	NTHI477_RS02110	792	A	G	synonymous
439680	NTHI477_RS02110	861	T	C	synonymous
439734	NTHI477_RS02115	39	G	C	synonymous
439745	NTHI477_RS02115	50	G	A	nonsynonymous
439807	NTHI477_RS02115	112	C	T	nonsynonymous
439833	NTHI477_RS02115	138	T	C	synonymous
439836	NTHI477_RS02115	141	A	G	synonymous
439851	NTHI477_RS02115	156	C	A	synonymous
439882	NTHI477_RS02115	187	T	C	nonsynonymous
439899	NTHI477_RS02115	204	A	G	nonsynonymous
439935	NTHI477_RS02115	240	A	G	synonymous
439980	NTHI477_RS02115	285	G	A	synonymous
440001	NTHI477_RS02115	306	A	G	synonymous
440013	NTHI477_RS02115	318	G	A	synonymous
440043	NTHI477_RS02115	348	G	A	synonymous
440073	NTHI477_RS02115	378	A	G	synonymous
440113	NTHI477_RS02115	418	A	G	nonsynonymous
440115	NTHI477_RS02115	420	C	T	synonymous
440178	NTHI477_RS02115	483	C	T	synonymous
440398	intergenic	intergenic	T	A	intergenic
440459	intergenic	intergenic	T	C	intergenic
440474	intergenic	intergenic	C	T	intergenic
440503	intergenic	intergenic	C	G	intergenic
440540	intergenic	intergenic	A	G	intergenic
440557	intergenic	intergenic	C	T	intergenic
440563	intergenic	intergenic	C	T	intergenic
440594	intergenic	intergenic	C	T	intergenic
440659	intergenic	intergenic	A	G	intergenic
440663	intergenic	intergenic	A	G	intergenic
440692	intergenic	intergenic	G	A	intergenic
440732	intergenic	intergenic	G	T	intergenic
440810	intergenic	intergenic	G	A	intergenic
440820	intergenic	intergenic	G	T	intergenic
440830	NTHI477_RS02120	1688	G	A	nonsynonymous
440845	NTHI477_RS02120	1673	C	T	nonsynonymous
440905	NTHI477_RS02120	1613	T	C	nonsynonymous
441246	NTHI477_RS02120	1272	T	A	synonymous

441267	NTHI477_RS02120	1251	A	G	synonymous
441296	NTHI477_RS02120	1222	C	T	nonsynonymous
441443	NTHI477_RS02120	1075	G	A	nonsynonymous
441639	NTHI477_RS02120	879	G	A	synonymous
441711	NTHI477_RS02120	807	C	T	synonymous
441823	NTHI477_RS02120	695	C	T	nonsynonymous
441908	NTHI477_RS02120	610	G	A	nonsynonymous
442092	NTHI477_RS02120	426	G	A	synonymous
442107	NTHI477_RS02120	411	G	A	synonymous
442122	NTHI477_RS02120	396	G	A	synonymous
442149	NTHI477_RS02120	369	A	C	nonsynonymous
442170	NTHI477_RS02120	348	A	G	synonymous
442328	NTHI477_RS02120	190	C	T	nonsynonymous
442380	NTHI477_RS02120	138	A	T	synonymous
442389	NTHI477_RS02120	129	A	T	synonymous
442435	NTHI477_RS02120	83	G	A	nonsynonymous
442439	NTHI477_RS02120	79	C	A	nonsynonymous
442450	NTHI477_RS02120	68	G	T	nonsynonymous
442473	NTHI477_RS02120	45	T	G	synonymous
442534	intergenic	intergenic	T	A	intergenic
442536	intergenic	intergenic	T	C	intergenic
442545	intergenic	intergenic	G	A	intergenic
442549	intergenic	intergenic	T	C	intergenic
442583	intergenic	intergenic	C	T	intergenic
442584	intergenic	intergenic	A	G	intergenic
442600	intergenic	intergenic	G	A	intergenic
442662	intergenic	intergenic	T	C	intergenic
442739	NTHI477_RS09510	53	T	G	nonsynonymous
442769	NTHI477_RS09510	83	C	T	nonsynonymous
442786	NTHI477_RS09510	100	G	A	nonsynonymous
442888	NTHI477_RS09510	202	G	A	nonsynonymous
442893	intergenic	intergenic	G	T	intergenic
442995	NTHI477_RS09515	127	A	C	nonsynonymous
443017	NTHI477_RS09515	105	T	G	nonsynonymous
443035	NTHI477_RS09515	87	A	C	nonsynonymous
443085	NTHI477_RS09515	37	G	A	nonsynonymous
443131	NTHI477_RS09465	10	G	A	nonsynonymous
443141	NTHI477_RS09465	20	G	A	nonsynonymous
443185	NTHI477_RS09465	64	G	A	nonsynonymous
443223	NTHI477_RS09465	102	A	G	synonymous
443269	NTHI477_RS09465	148	G	A	nonsynonymous
443290	NTHI477_RS09465	169	G	T	nonsynonymous
443306	intergenic	intergenic	T	C	intergenic
443310	intergenic	intergenic	G	A	intergenic
443312	intergenic	intergenic	A	G	intergenic

443366	intergenic	intergenic	C	T	intergenic
443409	intergenic	intergenic	G	C	intergenic
443417	intergenic	intergenic	C	T	intergenic
443425	NTHI477_RS02130	549	C	T	nonsynonymous
443431	NTHI477_RS02130	543	A	G	synonymous
443435	NTHI477_RS02130	539	C	T	nonsynonymous
443437	NTHI477_RS02130	537	C	T	synonymous
443485	NTHI477_RS02130	489	T	C	synonymous
443491	NTHI477_RS02130	483	G	A	synonymous
443497	NTHI477_RS02130	477	C	T	synonymous
443509	NTHI477_RS02130	465	G	A	synonymous
443527	NTHI477_RS02130	447	A	G	synonymous
443536	NTHI477_RS02130	438	A	C	synonymous
443590	NTHI477_RS02130	384	T	G	synonymous
443599	NTHI477_RS02130	375	A	T	synonymous
443671	NTHI477_RS02130	303	A	T	synonymous
443677	NTHI477_RS02130	297	C	T	nonsynonymous
443728	NTHI477_RS02130	246	G	A	synonymous
443747	NTHI477_RS02130	227	T	C	nonsynonymous
443757	NTHI477_RS02130	217	T	C	nonsynonymous
443758	NTHI477_RS02130	216	G	T	synonymous
443763	NTHI477_RS02130	211	G	A	synonymous
443773	NTHI477_RS02130	201	G	T	synonymous
443782	NTHI477_RS02130	192	A	T	synonymous
443788	NTHI477_RS02130	186	G	T	synonymous
443812	NTHI477_RS02130	162	A	G	synonymous
443818	NTHI477_RS02130	156	G	A	synonymous
443823	NTHI477_RS02130	151	T	C	nonsynonymous
443824	NTHI477_RS02130	150	T	A	synonymous
443831	NTHI477_RS02130	143	T	C	nonsynonymous
443846	NTHI477_RS02130	128	T	C	nonsynonymous
443851	NTHI477_RS02130	123	T	C	synonymous
443862	NTHI477_RS02130	112	A	G	synonymous
443868	NTHI477_RS02130	106	T	C	nonsynonymous
443881	NTHI477_RS02130	93	T	C	synonymous
443889	NTHI477_RS02130	85	C	T	nonsynonymous
443897	NTHI477_RS02130	77	C	T	nonsynonymous
443900	NTHI477_RS02130	74	A	G	nonsynonymous
443906	NTHI477_RS02130	68	C	T	nonsynonymous
443913	NTHI477_RS02130	61	C	T	nonsynonymous
444002	intergenic	intergenic	T	C	intergenic
444004	intergenic	intergenic	C	A	intergenic
444008	intergenic	intergenic	G	A	intergenic
444009	intergenic	intergenic	C	G	intergenic
444009	intergenic	intergenic	C	T	intergenic

444029	intergenic	intergenic	A	G	intergenic
444034	intergenic	intergenic	G	A	intergenic
444052	intergenic	intergenic	C	T	intergenic
444054	intergenic	intergenic	A	G	intergenic
444055	intergenic	intergenic	A	G	intergenic
444057	intergenic	intergenic	G	A	intergenic
444058	intergenic	intergenic	G	C	intergenic
444060	intergenic	intergenic	A	C	intergenic
444061	intergenic	intergenic	T	A	intergenic
444071	intergenic	intergenic	C	T	intergenic
444124	NTHI477_RS02135	1411	G	A	synonymous
444134	NTHI477_RS02135	1401	T	C	synonymous
444146	NTHI477_RS02135	1389	A	G	synonymous
444158	NTHI477_RS02135	1377	C	T	synonymous
444176	NTHI477_RS02135	1359	C	T	synonymous
444194	NTHI477_RS02135	1341	T	C	synonymous
444221	NTHI477_RS02135	1314	C	T	synonymous
444242	NTHI477_RS02135	1293	A	G	synonymous
444245	NTHI477_RS02135	1290	G	A	synonymous
444248	NTHI477_RS02135	1287	A	G	synonymous
444260	NTHI477_RS02135	1275	G	A	synonymous
444266	NTHI477_RS02135	1269	A	C	synonymous
444284	NTHI477_RS02135	1251	T	C	synonymous
444296	NTHI477_RS02135	1239	T	A	nonsynonymous
444302	NTHI477_RS02135	1233	G	A	synonymous
444314	NTHI477_RS02135	1221	A	G	synonymous
444353	NTHI477_RS02135	1182	T	A	synonymous
444377	NTHI477_RS02135	1158	C	G	synonymous
444385	NTHI477_RS02135	1150	T	C	nonsynonymous
444437	NTHI477_RS02135	1098	G	A	synonymous
444440	NTHI477_RS02135	1095	A	G	synonymous
444448	NTHI477_RS02135	1087	G	T	synonymous
444458	NTHI477_RS02135	1077	C	T	synonymous
444464	NTHI477_RS02135	1071	T	C	synonymous
444464	NTHI477_RS02135	1071	T	G	synonymous
444467	NTHI477_RS02135	1068	A	G	synonymous
444473	NTHI477_RS02135	1062	C	T	synonymous
444476	NTHI477_RS02135	1059	A	T	synonymous
444476	NTHI477_RS02135	1059	A	G	synonymous
444479	NTHI477_RS02135	1056	T	A	synonymous
444482	NTHI477_RS02135	1053	T	A	synonymous
444482	NTHI477_RS02135	1053	T	G	synonymous
444488	NTHI477_RS02135	1047	C	T	synonymous
444491	NTHI477_RS02135	1044	T	C	synonymous
444495	NTHI477_RS02135	1040	G	T	nonsynonymous

444497	NTHI477_RS02135	1038	C	T	synonymous
444497	NTHI477_RS02135	1038	C	A	synonymous
444499	NTHI477_RS02135	1036	A	C	nonsynonymous
444499	NTHI477_RS02135	1036	A	T	nonsynonymous
444500	NTHI477_RS02135	1035	G	A	synonymous
444503	NTHI477_RS02135	1032	G	A	synonymous
444509	NTHI477_RS02135	1026	T	C	synonymous
444536	NTHI477_RS02135	999	A	G	synonymous
444548	NTHI477_RS02135	987	G	A	synonymous
444551	NTHI477_RS02135	984	C	A	synonymous
444554	NTHI477_RS02135	981	A	C	nonsynonymous
444554	NTHI477_RS02135	981	A	G	synonymous
444563	NTHI477_RS02135	972	A	T	synonymous
444563	NTHI477_RS02135	972	A	G	synonymous
444565	NTHI477_RS02135	970	G	A	nonsynonymous
444593	NTHI477_RS02135	942	G	A	synonymous
444605	NTHI477_RS02135	930	C	T	synonymous
444607	NTHI477_RS02135	928	A	G	synonymous
444611	NTHI477_RS02135	924	T	A	synonymous
444626	NTHI477_RS02135	909	A	T	synonymous
444629	NTHI477_RS02135	906	G	A	synonymous
444629	NTHI477_RS02135	906	G	C	synonymous
444632	NTHI477_RS02135	903	G	A	synonymous
444635	NTHI477_RS02135	900	G	A	synonymous
444641	NTHI477_RS02135	894	T	C	synonymous
444647	NTHI477_RS02135	888	T	C	synonymous
444650	NTHI477_RS02135	885	A	G	synonymous
444656	NTHI477_RS02135	879	T	A	synonymous
444656	NTHI477_RS02135	879	T	C	synonymous
444659	NTHI477_RS02135	876	A	T	synonymous
444665	NTHI477_RS02135	870	T	C	synonymous
444671	NTHI477_RS02135	864	T	C	synonymous
444674	NTHI477_RS02135	861	G	A	synonymous
444680	NTHI477_RS02135	855	C	T	synonymous
444683	NTHI477_RS02135	852	G	A	synonymous
444689	NTHI477_RS02135	846	A	G	synonymous
444719	NTHI477_RS02135	816	C	G	synonymous
444722	NTHI477_RS02135	813	C	T	synonymous
444734	NTHI477_RS02135	801	A	C	synonymous
444737	NTHI477_RS02135	798	T	A	synonymous
444737	NTHI477_RS02135	798	T	C	synonymous
444740	NTHI477_RS02135	795	T	C	synonymous
444740	NTHI477_RS02135	795	T	A	synonymous
444743	NTHI477_RS02135	792	T	C	synonymous
444751	NTHI477_RS02135	784	G	A	synonymous

444754	NTHI477_RS02135	781	A	G	synonymous
444755	NTHI477_RS02135	780	C	T	synonymous
444758	NTHI477_RS02135	777	C	A	synonymous
444758	NTHI477_RS02135	777	C	G	synonymous
444761	NTHI477_RS02135	774	A	C	synonymous
444770	NTHI477_RS02135	765	A	C	synonymous
444770	NTHI477_RS02135	765	A	T	synonymous
444773	NTHI477_RS02135	762	A	T	synonymous
444773	NTHI477_RS02135	762	A	G	synonymous
444779	NTHI477_RS02135	756	T	C	synonymous
444785	NTHI477_RS02135	750	A	T	synonymous
444791	NTHI477_RS02135	744	T	C	synonymous
444800	NTHI477_RS02135	735	T	G	synonymous
444800	NTHI477_RS02135	735	T	C	synonymous
444803	NTHI477_RS02135	732	T	G	synonymous
444811	NTHI477_RS02135	724	T	C	nonsynonymous
444821	NTHI477_RS02135	714	A	G	synonymous
444826	NTHI477_RS02135	709	C	T	nonsynonymous
444827	NTHI477_RS02135	708	G	A	synonymous
444827	NTHI477_RS02135	708	G	T	synonymous
444829	NTHI477_RS02135	706	G	A	nonsynonymous
444830	NTHI477_RS02135	705	T	G	synonymous
444833	NTHI477_RS02135	702	T	C	synonymous
444857	NTHI477_RS02135	678	T	A	nonsynonymous
444858	NTHI477_RS02135	677	C	A	nonsynonymous
444859	NTHI477_RS02135	676	T	G	synonymous
444863	NTHI477_RS02135	672	A	T	synonymous
444885	NTHI477_RS02135	650	T	A	nonsynonymous
444886	NTHI477_RS02135	649	C	T	nonsynonymous
444929	NTHI477_RS02135	606	G	A	synonymous
444938	NTHI477_RS02135	597	C	T	synonymous
444947	NTHI477_RS02135	588	G	T	synonymous
444956	NTHI477_RS02135	579	T	C	synonymous
444968	NTHI477_RS02135	567	T	A	synonymous
444992	NTHI477_RS02135	543	A	G	synonymous
445004	NTHI477_RS02135	531	G	T	synonymous
445009	NTHI477_RS02135	526	A	G	synonymous
445013	NTHI477_RS02135	522	G	A	synonymous
445019	NTHI477_RS02135	516	C	T	synonymous
445028	NTHI477_RS02135	507	A	G	synonymous
445030	NTHI477_RS02135	505	T	C	nonsynonymous
445036	NTHI477_RS02135	499	C	T	nonsynonymous
445049	NTHI477_RS02135	486	A	G	synonymous
445055	NTHI477_RS02135	480	G	A	synonymous
445067	NTHI477_RS02135	468	A	T	synonymous

445070	NTHI477_RS02135	465	C	G	nonsynonymous
445071	NTHI477_RS02135	464	T	C	nonsynonymous
445072	NTHI477_RS02135	463	T	G	nonsynonymous
445073	NTHI477_RS02135	462	G	A	synonymous
445091	NTHI477_RS02135	444	A	T	synonymous
445094	NTHI477_RS02135	441	T	G	synonymous
445097	NTHI477_RS02135	438	A	T	synonymous
445097	NTHI477_RS02135	438	A	C	synonymous
445112	NTHI477_RS02135	423	C	T	synonymous
445134	NTHI477_RS02135	401	T	C	nonsynonymous
445142	NTHI477_RS02135	393	A	G	synonymous
445144	NTHI477_RS02135	391	A	T	nonsynonymous
445145	NTHI477_RS02135	390	T	C	synonymous
445209	NTHI477_RS02135	326	G	A	nonsynonymous
445217	NTHI477_RS02135	318	T	C	synonymous
445238	NTHI477_RS02135	297	T	C	synonymous
445241	NTHI477_RS02135	294	A	G	synonymous
445244	NTHI477_RS02135	291	T	C	synonymous
445247	NTHI477_RS02135	288	T	C	synonymous
445250	NTHI477_RS02135	285	C	T	synonymous
445253	NTHI477_RS02135	282	G	C	synonymous
445256	NTHI477_RS02135	279	T	C	synonymous
445259	NTHI477_RS02135	276	T	C	synonymous
445295	NTHI477_RS02135	240	G	A	synonymous
445295	NTHI477_RS02135	240	G	C	synonymous
445307	NTHI477_RS02135	228	G	C	synonymous
445310	NTHI477_RS02135	225	T	C	synonymous
445319	NTHI477_RS02135	216	G	A	synonymous
445327	NTHI477_RS02135	208	T	C	nonsynonymous
445328	NTHI477_RS02135	207	T	A	synonymous
445329	NTHI477_RS02135	206	G	A	nonsynonymous
445331	NTHI477_RS02135	204	C	T	synonymous
445334	NTHI477_RS02135	201	C	A	synonymous
445337	NTHI477_RS02135	198	A	G	synonymous
445343	NTHI477_RS02135	192	A	G	synonymous
445346	NTHI477_RS02135	189	G	A	synonymous
445366	NTHI477_RS02135	169	C	A	nonsynonymous
445382	NTHI477_RS02135	153	G	A	synonymous
445388	NTHI477_RS02135	147	T	C	synonymous
445409	NTHI477_RS02135	126	G	A	synonymous
445445	NTHI477_RS02135	90	A	G	synonymous
445448	NTHI477_RS02135	87	A	G	synonymous
445457	NTHI477_RS02135	78	A	C	synonymous
445459	NTHI477_RS02135	76	C	T	nonsynonymous
445460	NTHI477_RS02135	75	A	G	synonymous

445483	NTHI477_RS02135	52	G	T	nonsynonymous
445485	NTHI477_RS02135	50	G	A	nonsynonymous
445496	NTHI477_RS02135	39	A	G	synonymous
445499	NTHI477_RS02135	36	C	T	synonymous
445502	NTHI477_RS02135	33	T	C	synonymous
445505	NTHI477_RS02135	30	G	C	synonymous
445563	intergenic	intergenic	G	A	intergenic
445564	intergenic	intergenic	T	C	intergenic
445573	intergenic	intergenic	G	A	intergenic
445579	intergenic	intergenic	T	G	intergenic
445584	intergenic	intergenic	A	G	intergenic
445588	intergenic	intergenic	A	T	intergenic
445607	intergenic	intergenic	G	A	intergenic
445611	intergenic	intergenic	G	T	intergenic
445626	intergenic	intergenic	G	A	intergenic
445672	NTHI477_RS02140	816	C	A	synonymous
445696	NTHI477_RS02140	792	C	T	synonymous
445699	NTHI477_RS02140	789	A	C	synonymous
445705	NTHI477_RS02140	783	T	A	synonymous
445711	NTHI477_RS02140	777	T	A	synonymous
445714	NTHI477_RS02140	774	T	C	synonymous
445723	NTHI477_RS02140	765	A	T	synonymous
445726	NTHI477_RS02140	762	T	C	synonymous
445729	NTHI477_RS02140	759	C	T	synonymous
445747	NTHI477_RS02140	741	C	T	synonymous
445750	NTHI477_RS02140	738	T	A	synonymous
445753	NTHI477_RS02140	735	T	C	synonymous
445756	NTHI477_RS02140	732	C	T	synonymous
445759	NTHI477_RS02140	729	T	C	synonymous
445762	NTHI477_RS02140	726	C	T	synonymous
445768	NTHI477_RS02140	720	C	T	synonymous
445771	NTHI477_RS02140	717	C	A	synonymous
445795	NTHI477_RS02140	693	C	T	synonymous
445801	NTHI477_RS02140	687	T	C	synonymous
445843	NTHI477_RS02140	645	G	T	synonymous
445853	NTHI477_RS02140	635	A	G	nonsynonymous
445864	NTHI477_RS02140	624	G	T	synonymous
445866	NTHI477_RS02140	622	G	A	nonsynonymous
445879	NTHI477_RS02140	609	C	G	synonymous
445882	NTHI477_RS02140	606	A	G	synonymous
445885	NTHI477_RS02140	603	C	G	synonymous
445885	NTHI477_RS02140	603	C	A	synonymous
445888	NTHI477_RS02140	600	C	T	synonymous
445903	NTHI477_RS02140	585	A	C	synonymous
445933	NTHI477_RS02140	555	G	A	synonymous

445936	NTHI477_RS02140	552	T	G	synonymous
445940	NTHI477_RS02140	548	T	C	nonsynonymous
445954	NTHI477_RS02140	534	C	T	synonymous
445956	NTHI477_RS02140	532	C	A	nonsynonymous
445960	NTHI477_RS02140	528	C	T	nonsynonymous
445971	NTHI477_RS02140	517	G	A	synonymous
445972	NTHI477_RS02140	516	T	C	synonymous
445973	NTHI477_RS02140	515	G	A	nonsynonymous
446026	NTHI477_RS02140	462	T	G	synonymous
446053	NTHI477_RS02140	435	C	T	synonymous
446068	NTHI477_RS02140	420	A	G	synonymous
446077	NTHI477_RS02140	411	G	A	synonymous
446080	NTHI477_RS02140	408	T	C	synonymous
446116	NTHI477_RS02140	372	A	G	synonymous
446122	NTHI477_RS02140	366	A	T	synonymous
446126	NTHI477_RS02140	362	C	G	nonsynonymous
446140	NTHI477_RS02140	348	A	G	synonymous
446146	NTHI477_RS02140	342	T	A	synonymous
446160	NTHI477_RS02140	328	C	T	nonsynonymous
446162	NTHI477_RS02140	326	C	T	nonsynonymous
446167	NTHI477_RS02140	321	G	A	synonymous
446179	NTHI477_RS02140	309	G	A	synonymous
446185	NTHI477_RS02140	303	G	A	synonymous
446188	NTHI477_RS02140	300	T	A	synonymous
446206	NTHI477_RS02140	282	A	G	synonymous
446212	NTHI477_RS02140	276	A	G	synonymous
446242	NTHI477_RS02140	246	G	A	synonymous
446249	NTHI477_RS02140	239	T	C	nonsynonymous
446263	NTHI477_RS02140	225	G	A	synonymous
446275	NTHI477_RS02140	213	C	T	synonymous
446284	NTHI477_RS02140	204	C	T	synonymous
446284	NTHI477_RS02140	204	C	G	synonymous
446308	NTHI477_RS02140	180	G	A	synonymous
446344	NTHI477_RS02140	144	G	A	synonymous
446345	NTHI477_RS02140	143	C	A	nonsynonymous
446347	NTHI477_RS02140	141	C	T	synonymous
446356	NTHI477_RS02140	132	T	G	synonymous
446365	NTHI477_RS02140	123	G	A	synonymous
446374	NTHI477_RS02140	114	T	C	synonymous
446398	NTHI477_RS02140	90	C	A	synonymous
446419	NTHI477_RS02140	69	C	T	synonymous
446422	NTHI477_RS02140	66	A	G	synonymous
446431	NTHI477_RS02140	57	T	C	synonymous
446440	NTHI477_RS02140	48	G	A	synonymous
446449	NTHI477_RS02140	39	C	T	synonymous

446523	intergenic	intergenic	C	T	intergenic
446579	NTHI477_RS02145	21	T	C	synonymous
446581	NTHI477_RS02145	23	G	A	nonsynonymous
446645	NTHI477_RS02145	87	A	C	synonymous
446651	NTHI477_RS02145	93	C	T	synonymous
446663	NTHI477_RS02145	105	A	G	synonymous
446676	NTHI477_RS02145	118	C	A	nonsynonymous
446711	NTHI477_RS02145	153	C	T	synonymous
446726	NTHI477_RS02145	168	A	G	synonymous
446765	NTHI477_RS02145	207	A	G	synonymous
446771	NTHI477_RS02145	213	G	C	synonymous
446771	NTHI477_RS02145	213	G	A	synonymous
446774	NTHI477_RS02145	216	A	G	synonymous
446792	NTHI477_RS02145	234	G	A	synonymous
446804	NTHI477_RS02145	246	A	G	synonymous
446806	NTHI477_RS02145	248	A	C	nonsynonymous
446807	NTHI477_RS02145	249	A	T	nonsynonymous
446810	NTHI477_RS02145	252	G	A	synonymous
446816	NTHI477_RS02145	258	A	G	synonymous
446843	NTHI477_RS02145	285	G	A	synonymous
446858	NTHI477_RS02145	300	T	A	synonymous
446873	NTHI477_RS02145	315	C	G	synonymous
446874	NTHI477_RS02145	316	C	A	synonymous
446885	NTHI477_RS02145	327	T	C	synonymous
446895	NTHI477_RS02145	337	A	G	nonsynonymous
446897	NTHI477_RS02145	339	C	A	synonymous
446897	NTHI477_RS02145	339	C	T	synonymous
446903	NTHI477_RS02145	345	A	C	nonsynonymous
446903	NTHI477_RS02145	345	A	G	synonymous
446904	NTHI477_RS02145	346	G	A	nonsynonymous
446906	NTHI477_RS02145	348	C	T	synonymous
446909	NTHI477_RS02145	351	G	A	synonymous
446909	NTHI477_RS02145	351	G	T	synonymous
446925	NTHI477_RS02145	367	G	A	nonsynonymous
446926	NTHI477_RS02145	368	C	T	nonsynonymous
446930	NTHI477_RS02145	372	A	G	synonymous
446936	NTHI477_RS02145	378	A	G	synonymous
446943	NTHI477_RS02145	385	C	T	synonymous
446977	NTHI477_RS02145	419	C	T	nonsynonymous
446984	NTHI477_RS02145	426	T	C	synonymous
447000	NTHI477_RS02145	442	C	A	nonsynonymous
447011	NTHI477_RS02145	453	G	T	nonsynonymous
447015	NTHI477_RS02145	457	A	G	nonsynonymous
447020	NTHI477_RS02145	462	A	G	synonymous
447047	NTHI477_RS02145	489	G	A	synonymous

447056	NTHI477_RS02145	498	A	G	synonymous
447068	NTHI477_RS02145	510	T	C	synonymous
447080	NTHI477_RS02145	522	T	C	synonymous
447113	NTHI477_RS02145	555	A	G	synonymous
447113	NTHI477_RS02145	555	A	T	synonymous
447119	NTHI477_RS02145	561	C	T	synonymous
447123	NTHI477_RS02145	565	A	G	nonsynonymous
447125	NTHI477_RS02145	567	A	T	synonymous
447128	NTHI477_RS02145	570	C	T	synonymous
447137	NTHI477_RS02145	579	T	C	synonymous
447140	NTHI477_RS02145	582	A	T	synonymous
447140	NTHI477_RS02145	582	A	C	synonymous
447149	NTHI477_RS02145	591	C	T	synonymous
447151	NTHI477_RS02145	593	G	A	nonsynonymous
447152	NTHI477_RS02145	594	C	T	synonymous
447153	NTHI477_RS02145	595	A	G	nonsynonymous
447155	NTHI477_RS02145	597	G	A	synonymous
447164	NTHI477_RS02145	606	T	C	synonymous
447167	NTHI477_RS02145	609	T	C	synonymous
447173	NTHI477_RS02145	615	C	A	synonymous
447176	NTHI477_RS02145	618	T	A	synonymous
447188	NTHI477_RS02145	630	T	C	synonymous
447215	NTHI477_RS02145	657	G	A	synonymous
447228	NTHI477_RS02145	670	A	G	nonsynonymous
447230	NTHI477_RS02145	672	A	C	synonymous
447231	NTHI477_RS02145	673	C	T	synonymous
447239	NTHI477_RS02145	681	C	T	synonymous
447251	NTHI477_RS02145	693	C	T	synonymous
447264	NTHI477_RS02145	706	T	G	nonsynonymous
447274	NTHI477_RS02145	716	T	C	nonsynonymous
447275	NTHI477_RS02145	717	T	G	nonsynonymous
447276	NTHI477_RS02145	718	T	C	synonymous
447278	NTHI477_RS02145	720	A	G	synonymous
447281	NTHI477_RS02145	723	T	C	synonymous
447293	NTHI477_RS02145	735	A	G	synonymous
447297	NTHI477_RS02145	739	T	A	nonsynonymous
447299	NTHI477_RS02145	741	T	A	synonymous
447302	NTHI477_RS02145	744	A	G	synonymous
447303	NTHI477_RS02145	745	T	C	synonymous
447305	NTHI477_RS02145	747	A	T	nonsynonymous
447314	NTHI477_RS02145	756	G	A	synonymous
447320	NTHI477_RS02145	762	A	C	synonymous
447320	NTHI477_RS02145	762	A	G	synonymous
447322	NTHI477_RS02145	764	C	A	nonsynonymous
447324	NTHI477_RS02145	766	C	A	nonsynonymous

447324	NTHI477_RS02145	766	C	G	nonsynonymous
447326	NTHI477_RS02145	768	T	G	synonymous
447341	NTHI477_RS02145	783	G	A	synonymous
447342	NTHI477_RS02145	784	C	T	synonymous
447344	NTHI477_RS02145	786	G	C	synonymous
447344	NTHI477_RS02145	786	G	A	synonymous
447345	NTHI477_RS02145	787	C	T	synonymous
447359	NTHI477_RS02145	801	T	A	synonymous
447362	NTHI477_RS02145	804	T	C	synonymous
447377	NTHI477_RS02145	819	T	C	synonymous
447398	NTHI477_RS02145	840	C	T	synonymous
447401	NTHI477_RS02145	843	C	T	synonymous
447404	NTHI477_RS02145	846	T	C	synonymous
447407	NTHI477_RS02145	849	G	A	synonymous
447423	NTHI477_RS02145	865	A	G	nonsynonymous
447428	NTHI477_RS02145	870	A	T	synonymous
447437	NTHI477_RS02145	879	A	G	synonymous
447461	NTHI477_RS02145	903	T	C	synonymous
447467	NTHI477_RS02145	909	T	A	nonsynonymous
447470	NTHI477_RS02145	912	T	C	synonymous
447475	NTHI477_RS02145	917	T	C	nonsynonymous
447476	NTHI477_RS02145	918	A	G	synonymous
447476	NTHI477_RS02145	918	A	T	synonymous
447479	NTHI477_RS02145	921	G	A	synonymous
447488	NTHI477_RS02145	930	G	A	synonymous
447491	NTHI477_RS02145	933	G	A	synonymous
447494	NTHI477_RS02145	936	T	A	synonymous
447494	NTHI477_RS02145	936	T	G	synonymous
447509	NTHI477_RS02145	951	C	T	synonymous
447512	NTHI477_RS02145	954	C	T	synonymous
447537	NTHI477_RS02145	979	A	G	nonsynonymous
447548	NTHI477_RS02145	990	G	A	synonymous
447555	NTHI477_RS02145	997	A	G	nonsynonymous
447572	NTHI477_RS02145	1014	A	G	nonsynonymous
447591	NTHI477_RS02145	1033	G	A	nonsynonymous
447600	NTHI477_RS02145	1042	A	C	nonsynonymous
447641	NTHI477_RS02145	1083	T	C	synonymous
447641	NTHI477_RS02145	1083	T	G	synonymous
447665	NTHI477_RS02145	1107	T	C	synonymous
447758	NTHI477_RS02145	1200	A	C	synonymous
447758	NTHI477_RS02145	1200	A	G	synonymous
447779	NTHI477_RS02145	1221	T	C	synonymous
447824	NTHI477_RS02145	1266	T	C	synonymous
447830	NTHI477_RS02145	1272	T	C	synonymous
447933	NTHI477_RS02145	1375	G	C	nonsynonymous

447933	NTHI477_RS02145	1375	G	T	nonsynonymous
447947	NTHI477_RS02145	1389	C	T	synonymous
447968	NTHI477_RS02145	1410	A	G	synonymous
447981	NTHI477_RS02145	1423	G	A	nonsynonymous
447983	NTHI477_RS02145	1425	T	C	synonymous
447992	NTHI477_RS02145	1434	C	T	synonymous
448046	NTHI477_RS02145	1488	T	C	synonymous
448061	NTHI477_RS02145	1503	G	T	nonsynonymous
448067	NTHI477_RS02145	1509	A	G	synonymous
448085	NTHI477_RS02145	1527	C	T	synonymous
448121	NTHI477_RS02145	1563	G	A	synonymous
448133	NTHI477_RS02145	1575	G	A	synonymous
448136	NTHI477_RS02145	1578	A	C	synonymous
448148	NTHI477_RS02145	1590	C	T	synonymous
448177	NTHI477_RS02145	1619	G	A	nonsynonymous
448181	NTHI477_RS02145	1623	G	A	synonymous
448193	NTHI477_RS02145	1635	G	A	synonymous
448196	NTHI477_RS02145	1638	T	G	synonymous
448349	NTHI477_RS02150	64	G	T	nonsynonymous
448435	NTHI477_RS02150	150	C	T	synonymous
448470	NTHI477_RS02150	185	T	A	nonsynonymous
448500	NTHI477_RS02150	215	G	A	nonsynonymous
448507	NTHI477_RS02150	222	G	A	synonymous
448519	NTHI477_RS02150	234	G	A	synonymous
448556	NTHI477_RS02150	271	A	G	nonsynonymous
448561	NTHI477_RS02150	276	A	G	synonymous
448576	NTHI477_RS02150	291	C	T	synonymous
448646	NTHI477_RS02150	361	C	T	nonsynonymous
448670	NTHI477_RS02155	13	G	A	nonsynonymous
448711	NTHI477_RS02155	54	A	T	synonymous
448714	NTHI477_RS02155	57	A	G	synonymous
448717	NTHI477_RS02155	60	A	T	synonymous
448720	NTHI477_RS02155	63	T	C	synonymous
448739	NTHI477_RS02155	82	A	G	nonsynonymous
448741	NTHI477_RS02155	84	C	T	synonymous
448744	NTHI477_RS02155	87	C	T	synonymous
448786	NTHI477_RS02155	129	A	G	synonymous
448789	NTHI477_RS02155	132	G	T	synonymous
448795	NTHI477_RS02155	138	T	C	synonymous
448804	NTHI477_RS02155	147	C	G	synonymous
448819	NTHI477_RS02155	162	C	T	synonymous
448834	NTHI477_RS02155	177	C	T	synonymous
448837	NTHI477_RS02155	180	C	A	synonymous
448840	NTHI477_RS02155	183	A	T	synonymous
448846	NTHI477_RS02155	189	A	G	synonymous

448849	NTHI477_RS02155	192	T	G	synonymous
448858	NTHI477_RS02155	201	T	C	synonymous
448861	NTHI477_RS02155	204	T	G	nonsynonymous
448873	NTHI477_RS02155	216	G	A	synonymous
448882	NTHI477_RS02155	225	T	G	synonymous
448882	NTHI477_RS02155	225	T	A	synonymous
448888	NTHI477_RS02155	231	G	A	synonymous
448889	NTHI477_RS02155	232	C	T	synonymous
448897	NTHI477_RS02155	240	T	C	synonymous
448909	NTHI477_RS02155	252	G	A	synonymous
448912	NTHI477_RS02155	255	G	A	synonymous
448921	NTHI477_RS02155	264	A	G	synonymous
448925	NTHI477_RS02155	268	A	G	nonsynonymous
448937	NTHI477_RS02155	280	T	C	nonsynonymous
448940	NTHI477_RS02155	283	C	T	nonsynonymous
448943	NTHI477_RS02155	286	A	G	nonsynonymous
448972	NTHI477_RS02155	315	T	C	synonymous
448973	NTHI477_RS02155	316	G	A	nonsynonymous
449008	NTHI477_RS02155	351	T	A	synonymous
449014	NTHI477_RS02155	357	A	G	synonymous
449032	NTHI477_RS02155	375	G	A	synonymous
449040	NTHI477_RS02155	383	C	T	nonsynonymous
449041	NTHI477_RS02155	384	G	A	synonymous
449041	NTHI477_RS02155	384	G	T	synonymous
449047	NTHI477_RS02155	390	C	T	synonymous
449050	NTHI477_RS02155	393	C	T	synonymous
449066	NTHI477_RS02155	409	A	G	nonsynonymous
449077	NTHI477_RS02155	420	T	C	synonymous
449079	NTHI477_RS02155	422	C	T	nonsynonymous
449080	NTHI477_RS02155	423	G	A	synonymous
449083	NTHI477_RS02155	426	A	C	synonymous
449125	NTHI477_RS02155	468	C	T	synonymous
449132	NTHI477_RS02155	475	T	C	synonymous
449154	NTHI477_RS02155	497	G	C	nonsynonymous
449164	NTHI477_RS02155	507	C	T	synonymous
449167	NTHI477_RS02155	510	C	T	synonymous
449170	NTHI477_RS02155	513	T	C	synonymous
449176	NTHI477_RS02155	519	A	G	synonymous
449179	NTHI477_RS02155	522	C	T	synonymous
449212	NTHI477_RS02155	555	A	G	synonymous
449235	NTHI477_RS02155	578	G	C	nonsynonymous
449245	intergenic	intergenic	G	A	intergenic
449313	NTHI477_RS02160	15	A	G	synonymous
449331	NTHI477_RS02160	33	A	G	nonsynonymous
449331	NTHI477_RS02160	33	A	C	synonymous

449332	NTHI477_RS02160	34	G	C	nonsynonymous
449334	NTHI477_RS02160	36	G	A	synonymous
449335	NTHI477_RS02160	37	T	C	nonsynonymous
449340	NTHI477_RS02160	42	G	A	synonymous
449341	NTHI477_RS02160	43	A	G	nonsynonymous
449343	NTHI477_RS02160	45	T	G	synonymous
449346	NTHI477_RS02160	48	C	T	synonymous
449353	NTHI477_RS02160	55	T	C	synonymous
449367	NTHI477_RS02160	69	C	T	synonymous
449370	NTHI477_RS02160	72	T	A	synonymous
449373	NTHI477_RS02160	75	A	T	synonymous
449376	NTHI477_RS02160	78	T	G	synonymous
449385	NTHI477_RS02160	87	A	T	synonymous
449388	NTHI477_RS02160	90	A	T	synonymous
449388	NTHI477_RS02160	90	A	C	synonymous
449394	NTHI477_RS02160	96	C	A	synonymous
449408	NTHI477_RS02160	110	G	C	nonsynonymous
449409	NTHI477_RS02160	111	A	T	synonymous
449412	NTHI477_RS02160	114	A	C	synonymous
449418	NTHI477_RS02160	120	T	C	synonymous
449424	NTHI477_RS02160	126	C	T	synonymous
449424	NTHI477_RS02160	126	C	G	synonymous
449427	NTHI477_RS02160	129	G	T	synonymous
449427	NTHI477_RS02160	129	G	A	synonymous
449430	NTHI477_RS02160	132	C	G	synonymous
449431	NTHI477_RS02160	133	T	A	nonsynonymous
449433	NTHI477_RS02160	135	C	A	synonymous
449445	NTHI477_RS02160	147	C	T	synonymous
449454	NTHI477_RS02160	156	A	T	synonymous
449454	NTHI477_RS02160	156	A	C	synonymous
449478	NTHI477_RS02160	180	T	A	synonymous
449490	NTHI477_RS02160	192	T	C	synonymous
449499	NTHI477_RS02160	201	A	G	synonymous
449500	NTHI477_RS02160	202	T	G	nonsynonymous
449500	NTHI477_RS02160	202	T	A	nonsynonymous
449508	NTHI477_RS02160	210	C	T	synonymous
449520	NTHI477_RS02160	222	C	A	synonymous
449523	NTHI477_RS02160	225	A	G	synonymous
449535	NTHI477_RS02160	237	T	C	synonymous
449550	NTHI477_RS02160	252	C	A	synonymous
449559	NTHI477_RS02160	261	A	T	synonymous
449562	NTHI477_RS02160	264	A	T	synonymous
449566	NTHI477_RS02160	268	G	A	nonsynonymous
449567	NTHI477_RS02160	269	A	G	nonsynonymous
449569	NTHI477_RS02160	271	A	G	nonsynonymous

449571	NTHI477_RS02160	273	C	T	synonymous
449574	NTHI477_RS02160	276	C	A	synonymous
449577	NTHI477_RS02160	279	G	A	synonymous
449580	NTHI477_RS02160	282	A	C	nonsynonymous
449583	NTHI477_RS02160	285	A	T	synonymous
449586	NTHI477_RS02160	288	A	C	synonymous
449589	NTHI477_RS02160	291	G	A	synonymous
449595	NTHI477_RS02160	297	A	T	nonsynonymous
449596	NTHI477_RS02160	298	A	G	nonsynonymous
449599	NTHI477_RS02160	301	C	A	nonsynonymous
449600	NTHI477_RS02160	302	G	A	nonsynonymous
449601	NTHI477_RS02160	303	T	A	synonymous
449601	NTHI477_RS02160	303	T	G	synonymous
449604	NTHI477_RS02160	306	C	T	synonymous
449608	NTHI477_RS02160	310	G	A	nonsynonymous
449613	NTHI477_RS02160	315	C	T	synonymous
449616	NTHI477_RS02160	318	T	C	synonymous
449616	NTHI477_RS02160	318	T	A	synonymous
449622	NTHI477_RS02160	324	T	A	nonsynonymous
449623	NTHI477_RS02160	325	A	G	nonsynonymous
449624	NTHI477_RS02160	326	C	T	nonsynonymous
449631	NTHI477_RS02160	333	C	T	synonymous
449632	NTHI477_RS02160	334	C	G	nonsynonymous
449637	NTHI477_RS02160	339	C	A	synonymous
449638	NTHI477_RS02160	340	A	G	nonsynonymous
449641	NTHI477_RS02160	343	A	G	nonsynonymous
449643	NTHI477_RS02160	345	A	T	synonymous
449644	NTHI477_RS02160	346	G	A	nonsynonymous
449649	NTHI477_RS02160	351	T	A	synonymous
449655	NTHI477_RS02160	357	T	C	synonymous
449658	NTHI477_RS02160	360	C	T	synonymous
449670	NTHI477_RS02160	372	A	C	synonymous
449686	NTHI477_RS02160	388	A	C	nonsynonymous
449700	NTHI477_RS02160	402	C	T	synonymous
449709	NTHI477_RS02160	411	G	T	nonsynonymous
449739	NTHI477_RS02160	441	A	T	synonymous
449757	NTHI477_RS02160	459	T	C	synonymous
449799	NTHI477_RS02160	501	C	T	synonymous
449841	NTHI477_RS02160	543	C	G	synonymous
449841	NTHI477_RS02160	543	C	T	synonymous
449844	NTHI477_RS02160	546	G	A	synonymous
449883	intergenic	intergenic	C	T	intergenic
449884	intergenic	intergenic	T	C	intergenic
449893	intergenic	intergenic	C	A	intergenic
449938	intergenic	intergenic	C	A	intergenic

449976	intergenic	intergenic	G	A	intergenic
450011	intergenic	intergenic	T	C	intergenic
450015	intergenic	intergenic	A	T	intergenic
450063	intergenic	intergenic	T	C	intergenic
450072	intergenic	intergenic	C	T	intergenic
450089	intergenic	intergenic	T	G	intergenic
450251	NTHI477_RS02165	117	C	T	synonymous
450320	NTHI477_RS02165	186	C	T	synonymous
450356	NTHI477_RS02165	222	G	A	synonymous
450389	NTHI477_RS02165	255	A	G	synonymous
450459	NTHI477_RS02165	325	T	C	synonymous
450472	NTHI477_RS02165	338	G	A	nonsynonymous
450509	NTHI477_RS02165	375	T	C	synonymous
450521	NTHI477_RS02165	387	C	T	synonymous
450522	NTHI477_RS02165	388	A	G	nonsynonymous
450523	NTHI477_RS02165	389	C	A	nonsynonymous
450531	NTHI477_RS02165	397	G	A	nonsynonymous
450532	NTHI477_RS02165	398	G	A	nonsynonymous
450533	NTHI477_RS02165	399	C	T	synonymous
450542	NTHI477_RS02165	408	T	C	synonymous
450587	NTHI477_RS02165	453	G	A	synonymous
450620	NTHI477_RS02165	486	G	T	synonymous
450659	NTHI477_RS02165	525	C	G	synonymous
450698	NTHI477_RS02165	564	T	A	synonymous
450731	NTHI477_RS02165	597	A	C	synonymous
450737	NTHI477_RS02165	603	T	G	synonymous
450740	NTHI477_RS02165	606	C	T	synonymous
450758	NTHI477_RS02165	624	A	T	synonymous
450761	NTHI477_RS02165	627	G	A	synonymous
450776	NTHI477_RS02165	642	T	C	synonymous
450785	NTHI477_RS02165	651	T	A	synonymous
450797	NTHI477_RS02165	663	A	G	synonymous
450908	NTHI477_RS02165	774	A	T	synonymous
451037	NTHI477_RS02165	903	T	A	synonymous
451109	NTHI477_RS02165	975	A	G	synonymous
451145	NTHI477_RS02165	1011	C	G	synonymous
451148	NTHI477_RS02165	1014	G	A	synonymous
451172	NTHI477_RS02165	1038	T	C	synonymous
451184	NTHI477_RS02165	1050	T	C	synonymous
451185	NTHI477_RS02165	1051	G	A	nonsynonymous
451253	NTHI477_RS02165	1119	T	C	synonymous
451291	NTHI477_RS02165	1157	C	T	nonsynonymous
451382	NTHI477_RS02165	1248	C	T	synonymous
451413	NTHI477_RS02165	1279	G	A	nonsynonymous
451448	NTHI477_RS02165	1314	A	G	synonymous

451460	NTHI477_RS02165	1326	T	C	synonymous
451589	NTHI477_RS02165	1455	T	G	synonymous
451589	NTHI477_RS02165	1455	T	A	synonymous
451592	NTHI477_RS02165	1458	T	G	synonymous
451598	NTHI477_RS02165	1464	T	A	synonymous
451717	NTHI477_RS02165	1583	G	C	nonsynonymous
451760	NTHI477_RS02165	1626	C	T	synonymous
451787	NTHI477_RS02165	1653	A	G	synonymous
451936	NTHI477_RS02165	1802	G	C	nonsynonymous
452048	NTHI477_RS02165	1914	G	A	synonymous
452051	NTHI477_RS02165	1917	T	C	synonymous
452054	NTHI477_RS02165	1920	A	T	synonymous
452231	NTHI477_RS02165	2097	T	C	synonymous
452286	NTHI477_RS02165	2152	C	T	nonsynonymous
452288	NTHI477_RS02165	2154	T	A	synonymous
452381	NTHI477_RS02165	2247	T	C	synonymous
452393	NTHI477_RS02165	2259	T	G	synonymous
452396	NTHI477_RS02165	2262	C	T	synonymous
452399	NTHI477_RS02165	2265	A	G	synonymous
452439	intergenic	intergenic	G	A	intergenic
452440	intergenic	intergenic	A	G	intergenic
452540	intergenic	intergenic	T	C	intergenic
452544	intergenic	intergenic	T	A	intergenic
452559	NTHI477_RS02170	12	C	T	synonymous
452565	NTHI477_RS02170	18	C	T	synonymous
452568	NTHI477_RS02170	21	A	C	synonymous
452574	NTHI477_RS02170	27	C	T	synonymous
452583	NTHI477_RS02170	36	T	C	synonymous
452598	NTHI477_RS02170	51	A	G	synonymous
452652	NTHI477_RS02170	105	A	G	synonymous
452661	NTHI477_RS02170	114	A	G	synonymous
452667	NTHI477_RS02170	120	C	G	synonymous
452688	NTHI477_RS02170	141	T	C	synonymous
452766	NTHI477_RS02170	219	C	T	synonymous
452814	NTHI477_RS02170	267	T	C	synonymous
452820	NTHI477_RS02170	273	C	T	synonymous
452833	NTHI477_RS02170	286	T	C	synonymous
452850	NTHI477_RS02170	303	G	T	synonymous
452868	NTHI477_RS02170	321	T	C	synonymous
452874	NTHI477_RS02170	327	T	C	synonymous
452877	NTHI477_RS02170	330	A	G	synonymous
452880	NTHI477_RS02170	333	T	G	synonymous
452988	NTHI477_RS02170	441	C	T	synonymous
452997	NTHI477_RS02170	450	C	T	synonymous
453061	NTHI477_RS02170	514	A	G	nonsynonymous

453108	NTHI477_RS02170	561	A	G	synonymous
453117	NTHI477_RS02170	570	A	G	synonymous
453207	NTHI477_RS02170	660	T	A	synonymous
453234	NTHI477_RS02170	687	A	G	synonymous
453267	NTHI477_RS02170	720	A	C	synonymous
453273	NTHI477_RS02170	726	C	T	synonymous
453315	NTHI477_RS02170	768	T	C	synonymous
453326	NTHI477_RS02170	779	T	C	nonsynonymous
453384	NTHI477_RS02170	837	A	T	synonymous
453405	NTHI477_RS02170	858	T	G	synonymous
453441	NTHI477_RS02170	894	C	T	synonymous
453627	NTHI477_RS02170	1080	T	C	synonymous
453643	NTHI477_RS02170	1096	G	A	nonsynonymous
453645	NTHI477_RS02170	1098	T	C	synonymous
453699	intergenic	intergenic	C	T	intergenic
453845	NTHI477_RS02175	1180	C	T	nonsynonymous
453881	NTHI477_RS02175	1144	A	G	synonymous
453882	NTHI477_RS02175	1143	A	G	synonymous
453891	NTHI477_RS02175	1134	G	A	synonymous
453900	NTHI477_RS02175	1125	A	G	synonymous
453906	NTHI477_RS02175	1119	G	A	synonymous
453915	NTHI477_RS02175	1110	C	A	synonymous
453918	NTHI477_RS02175	1107	A	G	synonymous
453927	NTHI477_RS02175	1098	C	A	synonymous
453948	NTHI477_RS02175	1077	C	T	synonymous
453966	NTHI477_RS02175	1059	G	A	synonymous
453981	NTHI477_RS02175	1044	C	T	synonymous
454008	NTHI477_RS02175	1017	A	G	synonymous
454026	NTHI477_RS02175	999	G	A	synonymous
454032	NTHI477_RS02175	993	G	A	synonymous
454035	NTHI477_RS02175	990	A	G	synonymous
454044	NTHI477_RS02175	981	A	G	synonymous
454047	NTHI477_RS02175	978	A	G	synonymous
454065	NTHI477_RS02175	960	A	G	synonymous
454077	NTHI477_RS02175	948	T	A	synonymous
454080	NTHI477_RS02175	945	T	C	synonymous
454100	NTHI477_RS02175	925	T	C	nonsynonymous
454185	NTHI477_RS02175	840	T	A	synonymous
454185	NTHI477_RS02175	840	T	C	synonymous
454188	NTHI477_RS02175	837	T	A	synonymous
454191	NTHI477_RS02175	834	T	C	synonymous
454197	NTHI477_RS02175	828	T	C	synonymous
454218	NTHI477_RS02175	807	G	A	synonymous
454229	NTHI477_RS02175	796	C	T	nonsynonymous
454235	NTHI477_RS02175	790	C	T	nonsynonymous

454257	NTHI477_RS02175	768	C	A	synonymous
454263	NTHI477_RS02175	762	A	C	synonymous
454275	NTHI477_RS02175	750	T	C	synonymous
454290	NTHI477_RS02175	735	C	T	synonymous
454296	NTHI477_RS02175	729	A	G	synonymous
454347	NTHI477_RS02175	678	T	G	nonsynonymous
454367	NTHI477_RS02175	658	G	A	synonymous
454368	NTHI477_RS02175	657	A	C	synonymous
454398	NTHI477_RS02175	627	G	A	synonymous
454404	NTHI477_RS02175	621	G	A	synonymous
454413	NTHI477_RS02175	612	C	T	synonymous
454416	NTHI477_RS02175	609	C	T	synonymous
454434	NTHI477_RS02175	591	A	C	synonymous
454496	NTHI477_RS02175	529	C	T	nonsynonymous
454497	NTHI477_RS02175	528	G	A	synonymous
454506	NTHI477_RS02175	519	C	T	synonymous
454537	NTHI477_RS02175	488	A	G	nonsynonymous
454572	NTHI477_RS02175	453	A	G	synonymous
454596	NTHI477_RS02175	429	T	C	synonymous
454608	NTHI477_RS02175	417	A	G	synonymous
454611	NTHI477_RS02175	414	C	T	synonymous
454614	NTHI477_RS02175	411	A	G	synonymous
454617	NTHI477_RS02175	408	C	T	synonymous
454626	NTHI477_RS02175	399	A	G	synonymous
454634	NTHI477_RS02175	391	C	T	nonsynonymous
454680	NTHI477_RS02175	345	C	T	synonymous
454692	NTHI477_RS02175	333	A	G	synonymous
454713	NTHI477_RS02175	312	C	T	synonymous
454718	NTHI477_RS02175	307	C	A	nonsynonymous
454731	NTHI477_RS02175	294	A	T	synonymous
454737	NTHI477_RS02175	288	T	C	synonymous
454740	NTHI477_RS02175	285	G	A	synonymous
454773	NTHI477_RS02175	252	G	A	synonymous
454803	NTHI477_RS02175	222	G	T	synonymous
454811	NTHI477_RS02175	214	A	G	synonymous
454815	NTHI477_RS02175	210	T	C	synonymous
454818	NTHI477_RS02175	207	A	G	synonymous
454821	NTHI477_RS02175	204	C	G	synonymous
454821	NTHI477_RS02175	204	C	T	synonymous
454824	NTHI477_RS02175	201	C	G	synonymous
454827	NTHI477_RS02175	198	T	A	synonymous
454832	NTHI477_RS02175	193	C	T	nonsynonymous
454833	NTHI477_RS02175	192	G	T	synonymous
454841	NTHI477_RS02175	184	C	A	nonsynonymous
454854	NTHI477_RS02175	171	A	T	synonymous

454855	NTHI477_RS02175	170	G	A	nonsynonymous
454859	NTHI477_RS02175	166	G	A	synonymous
454860	NTHI477_RS02175	165	T	C	synonymous
454862	NTHI477_RS02175	163	C	T	nonsynonymous
454863	NTHI477_RS02175	162	A	G	synonymous
454878	NTHI477_RS02175	147	G	T	synonymous
454881	NTHI477_RS02175	144	A	C	synonymous
454881	NTHI477_RS02175	144	A	G	synonymous
454893	NTHI477_RS02175	132	G	A	synonymous
454914	NTHI477_RS02175	111	C	A	synonymous
454920	NTHI477_RS02175	105	A	C	synonymous
454923	NTHI477_RS02175	102	C	T	synonymous
454935	NTHI477_RS02175	90	C	A	synonymous
454938	NTHI477_RS02175	87	A	G	synonymous
454944	NTHI477_RS02175	81	G	A	synonymous
454947	NTHI477_RS02175	78	C	T	synonymous
454949	NTHI477_RS02175	76	G	C	nonsynonymous
454965	NTHI477_RS02175	60	G	C	synonymous
454971	NTHI477_RS02175	54	A	G	synonymous
454980	NTHI477_RS02175	45	T	C	synonymous
454980	NTHI477_RS02175	45	T	A	synonymous
454982	NTHI477_RS02175	43	C	T	nonsynonymous
454986	NTHI477_RS02175	39	T	C	synonymous
455019	NTHI477_RS02175	6	T	A	synonymous
455019	NTHI477_RS02175	6	T	G	synonymous
455069	intergenic	intergenic	G	A	intergenic
455070	intergenic	intergenic	G	C	intergenic
455083	intergenic	intergenic	C	G	intergenic
455087	intergenic	intergenic	G	A	intergenic
455138	NTHI477_RS02180	2797	A	G	synonymous
455139	NTHI477_RS02180	2796	C	T	synonymous
455153	NTHI477_RS02180	2782	A	G	synonymous
455157	NTHI477_RS02180	2778	T	C	synonymous
455166	NTHI477_RS02180	2769	T	C	synonymous
455187	NTHI477_RS02180	2748	A	G	synonymous
455196	NTHI477_RS02180	2739	A	T	synonymous
455199	NTHI477_RS02180	2736	C	A	synonymous
455205	NTHI477_RS02180	2730	A	T	synonymous
455213	NTHI477_RS02180	2722	G	A	nonsynonymous
455214	NTHI477_RS02180	2721	A	G	synonymous
455229	NTHI477_RS02180	2706	G	A	synonymous
455233	NTHI477_RS02180	2702	T	C	nonsynonymous
455240	NTHI477_RS02180	2695	A	C	nonsynonymous
455250	NTHI477_RS02180	2685	T	A	synonymous
455253	NTHI477_RS02180	2682	T	C	synonymous

455255	NTHI477_RS02180	2680	A	C	nonsynonymous
455344	NTHI477_RS02180	2591	T	A	nonsynonymous
455350	NTHI477_RS02180	2585	G	A	nonsynonymous
455370	NTHI477_RS02180	2565	C	T	synonymous
455373	NTHI477_RS02180	2562	G	T	nonsynonymous
455376	NTHI477_RS02180	2559	T	G	synonymous
455394	NTHI477_RS02180	2541	A	G	synonymous
455397	NTHI477_RS02180	2538	A	G	synonymous
455424	NTHI477_RS02180	2511	T	G	synonymous
455505	NTHI477_RS02180	2430	A	G	synonymous
455517	NTHI477_RS02180	2418	G	A	synonymous
455525	NTHI477_RS02180	2410	T	C	nonsynonymous
455556	NTHI477_RS02180	2379	A	G	synonymous
455601	NTHI477_RS02180	2334	G	A	synonymous
455637	NTHI477_RS02180	2298	A	G	synonymous
455640	NTHI477_RS02180	2295	A	G	synonymous
455649	NTHI477_RS02180	2286	G	A	synonymous
455652	NTHI477_RS02180	2283	A	G	synonymous
455664	NTHI477_RS02180	2271	A	T	synonymous
455712	NTHI477_RS02180	2223	C	T	synonymous
455730	NTHI477_RS02180	2205	T	A	synonymous
455733	NTHI477_RS02180	2202	G	A	synonymous
455835	NTHI477_RS02180	2100	G	A	synonymous
455886	NTHI477_RS02180	2049	G	A	synonymous
456006	NTHI477_RS02180	1929	T	C	synonymous
456042	NTHI477_RS02180	1893	A	G	synonymous
456084	NTHI477_RS02180	1851	T	A	synonymous
456105	NTHI477_RS02180	1830	T	C	synonymous
456114	NTHI477_RS02180	1821	T	C	synonymous
456144	NTHI477_RS02180	1791	G	A	synonymous
456204	NTHI477_RS02180	1731	C	T	synonymous
456207	NTHI477_RS02180	1728	T	A	synonymous
456207	NTHI477_RS02180	1728	T	G	synonymous
456216	NTHI477_RS02180	1719	C	A	synonymous
456231	NTHI477_RS02180	1704	A	T	synonymous
456243	NTHI477_RS02180	1692	T	C	synonymous
456258	NTHI477_RS02180	1677	G	C	synonymous
456274	NTHI477_RS02180	1661	T	G	nonsynonymous
456294	NTHI477_RS02180	1641	C	A	synonymous
456297	NTHI477_RS02180	1638	C	T	synonymous
456312	NTHI477_RS02180	1623	G	A	synonymous
456315	NTHI477_RS02180	1620	G	C	synonymous
456354	NTHI477_RS02180	1581	T	A	synonymous
456363	NTHI477_RS02180	1572	A	T	synonymous
456369	NTHI477_RS02180	1566	C	G	synonymous

456372	NTHI477_RS02180	1563	G	T	synonymous
456420	NTHI477_RS02180	1515	A	G	synonymous
456426	NTHI477_RS02180	1509	C	T	synonymous
456456	NTHI477_RS02180	1479	G	A	synonymous
456480	NTHI477_RS02180	1455	T	G	synonymous
456522	NTHI477_RS02180	1413	G	A	synonymous
456531	NTHI477_RS02180	1404	T	C	synonymous
456537	NTHI477_RS02180	1398	T	G	synonymous
456537	NTHI477_RS02180	1398	T	A	synonymous
456540	NTHI477_RS02180	1395	C	T	synonymous
456543	NTHI477_RS02180	1392	G	A	synonymous
456555	NTHI477_RS02180	1380	G	A	synonymous
456558	NTHI477_RS02180	1377	A	G	synonymous
456570	NTHI477_RS02180	1365	C	T	synonymous
456576	NTHI477_RS02180	1359	A	G	synonymous
456594	NTHI477_RS02180	1341	G	A	synonymous
456615	NTHI477_RS02180	1320	C	A	synonymous
456629	NTHI477_RS02180	1306	C	T	nonsynonymous
456633	NTHI477_RS02180	1302	T	A	synonymous
456654	NTHI477_RS02180	1281	A	G	synonymous
456675	NTHI477_RS02180	1260	C	T	synonymous
456690	NTHI477_RS02180	1245	T	C	synonymous
456693	NTHI477_RS02180	1242	A	G	synonymous
456699	NTHI477_RS02180	1236	G	A	synonymous
456708	NTHI477_RS02180	1227	C	T	synonymous
456711	NTHI477_RS02180	1224	T	A	synonymous
456714	NTHI477_RS02180	1221	G	A	synonymous
456717	NTHI477_RS02180	1218	A	G	synonymous
456720	NTHI477_RS02180	1215	C	T	synonymous
456721	NTHI477_RS02180	1214	G	A	nonsynonymous
456723	NTHI477_RS02180	1212	G	A	synonymous
456729	NTHI477_RS02180	1206	C	A	synonymous
456732	NTHI477_RS02180	1203	A	G	synonymous
456768	NTHI477_RS02180	1167	G	A	synonymous
456771	NTHI477_RS02180	1164	G	A	synonymous
456777	NTHI477_RS02180	1158	A	C	synonymous
456792	NTHI477_RS02180	1143	A	G	synonymous
456945	NTHI477_RS02180	990	G	A	synonymous
456951	NTHI477_RS02180	984	G	A	synonymous
456963	NTHI477_RS02180	972	A	G	synonymous
456966	NTHI477_RS02180	969	T	G	synonymous
456966	NTHI477_RS02180	969	T	C	synonymous
456981	NTHI477_RS02180	954	C	A	synonymous
456990	NTHI477_RS02180	945	G	A	synonymous
456990	NTHI477_RS02180	945	G	C	synonymous

456995	NTHI477_RS02180	940	T	G	synonymous
457005	NTHI477_RS02180	930	T	C	synonymous
457011	NTHI477_RS02180	924	A	G	synonymous
457059	NTHI477_RS02180	876	G	A	synonymous
457122	NTHI477_RS02180	813	G	A	synonymous
457171	NTHI477_RS02180	764	A	C	nonsynonymous
457187	NTHI477_RS02180	748	A	C	nonsynonymous
457221	NTHI477_RS02180	714	C	T	synonymous
457266	NTHI477_RS02180	669	C	T	synonymous
457272	NTHI477_RS02180	663	A	G	synonymous
457296	NTHI477_RS02180	639	A	T	synonymous
457299	NTHI477_RS02180	636	T	C	synonymous
457302	NTHI477_RS02180	633	C	A	synonymous
457343	NTHI477_RS02180	592	G	T	nonsynonymous
457368	NTHI477_RS02180	567	C	T	synonymous
457371	NTHI477_RS02180	564	G	A	synonymous
457377	NTHI477_RS02180	558	G	A	synonymous
457383	NTHI477_RS02180	552	C	T	nonsynonymous
457485	NTHI477_RS02180	450	G	A	synonymous
457506	NTHI477_RS02180	429	T	A	synonymous
457515	NTHI477_RS02180	420	A	G	synonymous
457521	NTHI477_RS02180	414	G	A	synonymous
457545	NTHI477_RS02180	390	G	A	synonymous
457550	NTHI477_RS02180	385	G	A	nonsynonymous
457551	NTHI477_RS02180	384	G	A	synonymous
457572	NTHI477_RS02180	363	A	G	synonymous
457602	NTHI477_RS02180	333	A	G	synonymous
457608	NTHI477_RS02180	327	A	G	synonymous
457629	NTHI477_RS02180	306	A	T	synonymous
457632	NTHI477_RS02180	303	A	G	synonymous
457668	NTHI477_RS02180	267	T	C	synonymous
457683	NTHI477_RS02180	252	A	T	synonymous
457716	NTHI477_RS02180	219	A	G	synonymous
457724	NTHI477_RS02180	211	G	T	synonymous
457725	NTHI477_RS02180	210	C	T	synonymous
457731	NTHI477_RS02180	204	A	G	synonymous
457749	NTHI477_RS02180	186	C	T	synonymous
457755	NTHI477_RS02180	180	A	T	synonymous
457761	NTHI477_RS02180	174	G	A	synonymous
457764	NTHI477_RS02180	171	A	C	nonsynonymous
457801	NTHI477_RS02180	134	G	A	nonsynonymous
457830	NTHI477_RS02180	105	T	C	synonymous
457869	NTHI477_RS02180	66	A	G	synonymous
457884	NTHI477_RS02180	51	G	A	synonymous
457904	NTHI477_RS02180	31	A	G	synonymous

457986	intergenic	intergenic	C	T	intergenic
458027	intergenic	intergenic	A	G	intergenic
458050	intergenic	intergenic	C	A	intergenic
458053	intergenic	intergenic	G	A	intergenic
458080	intergenic	intergenic	A	C	intergenic
458086	intergenic	intergenic	C	T	intergenic
458097	intergenic	intergenic	C	T	intergenic
458330	intergenic	intergenic	G	A	intergenic
458350	intergenic	intergenic	G	A	intergenic
458379	intergenic	intergenic	T	A	intergenic
458380	intergenic	intergenic	G	A	intergenic
458382	intergenic	intergenic	T	A	intergenic
458386	intergenic	intergenic	C	T	intergenic
458397	intergenic	intergenic	A	T	intergenic
458568	NTHI477_RS02185	636	A	G	synonymous
458576	NTHI477_RS02185	628	G	A	nonsynonymous
458587	NTHI477_RS02185	617	G	C	nonsynonymous
458592	NTHI477_RS02185	612	G	A	synonymous
458593	NTHI477_RS02185	611	C	G	nonsynonymous
458596	NTHI477_RS02185	608	C	T	nonsynonymous
458619	NTHI477_RS02185	585	G	A	synonymous
458624	NTHI477_RS02185	580	A	G	nonsynonymous
458628	NTHI477_RS02185	576	G	A	synonymous
458631	NTHI477_RS02185	573	T	C	synonymous
458637	NTHI477_RS02185	567	G	A	synonymous
458648	NTHI477_RS02185	556	T	G	nonsynonymous
458658	NTHI477_RS02185	546	T	C	synonymous
458663	NTHI477_RS02185	541	G	A	nonsynonymous
458664	NTHI477_RS02185	540	G	T	synonymous
458666	NTHI477_RS02185	538	G	A	nonsynonymous
458667	NTHI477_RS02185	537	T	C	synonymous
458669	NTHI477_RS02185	535	A	G	synonymous
458679	NTHI477_RS02185	525	A	G	synonymous
458696	NTHI477_RS02185	508	T	C	nonsynonymous
458697	NTHI477_RS02185	507	T	A	nonsynonymous
458703	NTHI477_RS02185	501	A	G	synonymous
458706	NTHI477_RS02185	498	C	G	synonymous
458709	NTHI477_RS02185	495	G	A	synonymous
458715	NTHI477_RS02185	489	G	A	synonymous
458724	NTHI477_RS02185	480	A	G	synonymous
458733	NTHI477_RS02185	471	T	G	synonymous
458736	NTHI477_RS02185	468	G	A	synonymous
458757	NTHI477_RS02185	447	C	T	synonymous
458763	NTHI477_RS02185	441	C	T	synonymous
458778	NTHI477_RS02185	426	G	A	synonymous

458787	NTHI477_RS02185	417	A	G	synonymous
458793	NTHI477_RS02185	411	G	A	synonymous
458799	NTHI477_RS02185	405	T	C	synonymous
458802	NTHI477_RS02185	402	T	A	synonymous
458802	NTHI477_RS02185	402	T	G	synonymous
458808	NTHI477_RS02185	396	A	G	synonymous
458811	NTHI477_RS02185	393	C	T	synonymous
458841	NTHI477_RS02185	363	G	A	synonymous
458862	NTHI477_RS02185	342	G	A	synonymous
458862	NTHI477_RS02185	342	G	T	synonymous
458865	NTHI477_RS02185	339	C	T	synonymous
458889	NTHI477_RS02185	315	C	T	synonymous
458895	NTHI477_RS02185	309	A	G	synonymous
458898	NTHI477_RS02185	306	G	T	nonsynonymous
458910	NTHI477_RS02185	294	C	T	synonymous
458914	NTHI477_RS02185	290	C	T	nonsynonymous
458931	NTHI477_RS02185	273	T	C	synonymous
458932	NTHI477_RS02185	272	T	A	nonsynonymous
458933	NTHI477_RS02185	271	C	T	nonsynonymous
458963	NTHI477_RS02185	241	T	C	nonsynonymous
458967	NTHI477_RS02185	237	A	T	nonsynonymous
458969	NTHI477_RS02185	235	T	G	nonsynonymous
458973	NTHI477_RS02185	231	A	G	synonymous
459006	NTHI477_RS02185	198	T	C	synonymous
459012	NTHI477_RS02185	192	T	G	synonymous
459015	NTHI477_RS02185	189	A	T	synonymous
459018	NTHI477_RS02185	186	A	G	synonymous
459024	NTHI477_RS02185	180	G	A	synonymous
459036	NTHI477_RS02185	168	G	A	synonymous
459039	NTHI477_RS02185	165	A	G	synonymous
459042	NTHI477_RS02185	162	G	A	synonymous
459071	NTHI477_RS02185	133	C	G	nonsynonymous
459084	NTHI477_RS02185	120	A	G	synonymous
459102	NTHI477_RS02185	102	G	A	synonymous
459138	NTHI477_RS02185	66	T	G	nonsynonymous
459171	NTHI477_RS02185	33	G	A	synonymous
459180	NTHI477_RS02185	24	A	G	synonymous
459183	NTHI477_RS02185	21	G	A	synonymous
459204	NTHI477_RS02185	0	T	G	nonsynonymous
459208	intergenic	intergenic	G	A	intergenic
459209	intergenic	intergenic	C	A	intergenic
459220	NTHI477_RS02190	749	C	T	nonsynonymous
459228	NTHI477_RS02190	741	C	T	synonymous
459233	NTHI477_RS02190	736	C	G	nonsynonymous
459245	NTHI477_RS02190	724	G	A	nonsynonymous

459246	NTHI477_RS02190	723	G	C	nonsynonymous
459248	NTHI477_RS02190	721	T	G	nonsynonymous
459252	NTHI477_RS02190	717	C	A	nonsynonymous
459279	NTHI477_RS02190	690	C	T	synonymous
459293	NTHI477_RS02190	676	C	G	nonsynonymous
459297	NTHI477_RS02190	672	C	T	synonymous
459299	NTHI477_RS02190	670	T	G	nonsynonymous
459301	NTHI477_RS02190	668	G	A	nonsynonymous
459302	NTHI477_RS02190	667	C	A	nonsynonymous
459303	NTHI477_RS02190	666	T	G	synonymous
459306	NTHI477_RS02190	663	T	A	synonymous
459307	NTHI477_RS02190	662	G	T	nonsynonymous
459309	NTHI477_RS02190	660	A	C	nonsynonymous
459309	NTHI477_RS02190	660	A	G	synonymous
459318	NTHI477_RS02190	651	C	T	synonymous
459322	NTHI477_RS02190	647	G	A	nonsynonymous
459327	NTHI477_RS02190	642	T	G	synonymous
459328	NTHI477_RS02190	641	A	C	nonsynonymous
459329	NTHI477_RS02190	640	C	T	nonsynonymous
459335	NTHI477_RS02190	634	T	G	nonsynonymous
459345	NTHI477_RS02190	624	C	T	synonymous
459348	NTHI477_RS02190	621	G	A	synonymous
459366	NTHI477_RS02190	603	C	T	synonymous
459381	NTHI477_RS02190	588	G	A	synonymous
459384	NTHI477_RS02190	585	A	G	synonymous
459389	NTHI477_RS02190	580	A	G	synonymous
459390	NTHI477_RS02190	579	G	C	synonymous
459392	NTHI477_RS02190	577	G	A	nonsynonymous
459393	NTHI477_RS02190	576	T	C	synonymous
459402	NTHI477_RS02190	567	A	T	synonymous
459405	NTHI477_RS02190	564	G	A	synonymous
459411	NTHI477_RS02190	558	T	C	synonymous
459414	NTHI477_RS02190	555	G	T	synonymous
459414	NTHI477_RS02190	555	G	C	synonymous
459421	NTHI477_RS02190	548	G	T	nonsynonymous
459440	NTHI477_RS02190	529	G	A	nonsynonymous
459441	NTHI477_RS02190	528	G	C	synonymous
459462	NTHI477_RS02190	507	C	G	synonymous
459462	NTHI477_RS02190	507	C	T	synonymous
459468	NTHI477_RS02190	501	G	T	nonsynonymous
459484	NTHI477_RS02190	485	G	T	nonsynonymous
459507	NTHI477_RS02190	462	G	A	synonymous
459513	NTHI477_RS02190	456	A	G	synonymous
459516	NTHI477_RS02190	453	A	G	synonymous
459519	NTHI477_RS02190	450	C	A	synonymous

459520	NTHI477_RS02190	449	C	A	nonsynonymous
459521	NTHI477_RS02190	448	G	A	nonsynonymous
459522	NTHI477_RS02190	447	T	C	synonymous
459528	NTHI477_RS02190	441	T	A	nonsynonymous
459531	NTHI477_RS02190	438	G	A	synonymous
459558	NTHI477_RS02190	411	T	C	synonymous
459594	NTHI477_RS02190	375	T	C	synonymous
459596	NTHI477_RS02190	373	A	G	synonymous
459609	NTHI477_RS02190	360	C	T	synonymous
459612	NTHI477_RS02190	357	T	G	synonymous
459624	NTHI477_RS02190	345	T	G	synonymous
459624	NTHI477_RS02190	345	T	A	synonymous
459625	NTHI477_RS02190	344	C	T	nonsynonymous
459627	NTHI477_RS02190	342	C	T	synonymous
459630	NTHI477_RS02190	339	C	A	synonymous
459649	NTHI477_RS02190	320	G	A	nonsynonymous
459693	NTHI477_RS02190	276	G	C	synonymous
459708	NTHI477_RS02190	261	C	G	synonymous
459709	NTHI477_RS02190	260	C	G	nonsynonymous
459717	NTHI477_RS02190	252	C	T	synonymous
459718	NTHI477_RS02190	251	G	A	nonsynonymous
459720	NTHI477_RS02190	249	T	G	synonymous
459725	NTHI477_RS02190	244	G	A	nonsynonymous
459732	NTHI477_RS02190	237	G	A	synonymous
459752	NTHI477_RS02190	217	T	C	nonsynonymous
459798	NTHI477_RS02190	171	A	G	synonymous
459806	NTHI477_RS02190	163	A	T	nonsynonymous
459823	NTHI477_RS02190	146	T	G	nonsynonymous
459826	NTHI477_RS02190	143	G	T	nonsynonymous
459863	NTHI477_RS02190	106	G	A	nonsynonymous
459873	NTHI477_RS02190	96	C	T	synonymous
459874	NTHI477_RS02190	95	A	G	nonsynonymous
459888	NTHI477_RS02190	81	T	A	synonymous
459889	NTHI477_RS02190	80	A	G	nonsynonymous
459893	NTHI477_RS02190	76	T	G	nonsynonymous
459895	NTHI477_RS02190	74	T	C	nonsynonymous
459902	NTHI477_RS02190	67	G	C	nonsynonymous
459904	NTHI477_RS02190	65	G	C	nonsynonymous
459910	NTHI477_RS02190	59	A	G	nonsynonymous
459927	NTHI477_RS02190	42	G	A	synonymous
460059	NTHI477_RS02195	522	G	A	synonymous
460074	NTHI477_RS02195	507	G	A	synonymous
460106	NTHI477_RS02195	475	G	A	synonymous
460146	NTHI477_RS02195	435	T	C	synonymous
460216	NTHI477_RS02195	365	C	T	nonsynonymous

460235	NTHI477_RS02195	346	A	C	nonsynonymous
460268	NTHI477_RS02195	313	C	G	nonsynonymous
460311	NTHI477_RS02195	270	C	A	nonsynonymous
460449	NTHI477_RS02195	132	G	A	synonymous
460482	NTHI477_RS02195	99	T	C	synonymous
460506	NTHI477_RS02195	75	T	G	synonymous
460518	NTHI477_RS02195	63	T	C	nonsynonymous
460551	NTHI477_RS02195	30	T	C	synonymous
460563	NTHI477_RS02195	18	C	T	synonymous
460573	NTHI477_RS02195	8	T	G	nonsynonymous
460581	NTHI477_RS02195	0	T	G	synonymous
460608	NTHI477_RS02200	1458	T	C	synonymous
460635	NTHI477_RS02200	1431	A	C	synonymous
460650	NTHI477_RS02200	1416	A	G	synonymous
460683	NTHI477_RS02200	1383	A	G	synonymous
460697	NTHI477_RS02200	1369	C	T	nonsynonymous
460707	NTHI477_RS02200	1359	T	A	synonymous
460725	NTHI477_RS02200	1341	A	G	synonymous
460749	NTHI477_RS02200	1317	C	T	synonymous
460836	NTHI477_RS02200	1230	A	T	nonsynonymous
460839	NTHI477_RS02200	1227	A	T	synonymous
460850	NTHI477_RS02200	1216	C	T	nonsynonymous
460851	NTHI477_RS02200	1215	A	G	synonymous
460854	NTHI477_RS02200	1212	C	T	synonymous
460860	NTHI477_RS02200	1206	A	G	synonymous
460875	NTHI477_RS02200	1191	T	A	synonymous
460881	NTHI477_RS02200	1185	T	C	synonymous
460896	NTHI477_RS02200	1170	T	G	synonymous
460899	NTHI477_RS02200	1167	G	A	synonymous
460900	NTHI477_RS02200	1166	C	T	nonsynonymous
460965	NTHI477_RS02200	1101	C	T	synonymous
460988	NTHI477_RS02200	1078	G	T	synonymous
461103	NTHI477_RS02200	963	G	A	synonymous
461120	NTHI477_RS02200	946	A	G	synonymous
461160	NTHI477_RS02200	906	T	C	synonymous
461196	NTHI477_RS02200	870	T	C	synonymous
461199	NTHI477_RS02200	867	A	G	synonymous
461202	NTHI477_RS02200	864	T	G	synonymous
461205	NTHI477_RS02200	861	C	T	synonymous
461207	NTHI477_RS02200	859	T	G	synonymous
461208	NTHI477_RS02200	858	G	A	synonymous
461216	NTHI477_RS02200	850	C	T	nonsynonymous
461218	NTHI477_RS02200	848	G	T	nonsynonymous
461229	NTHI477_RS02200	837	T	G	synonymous
461232	NTHI477_RS02200	834	C	T	synonymous

461253	NTHI477_RS02200	813	G	A	synonymous
461301	NTHI477_RS02200	765	T	C	synonymous
461322	NTHI477_RS02200	744	C	T	synonymous
461325	NTHI477_RS02200	741	A	G	synonymous
461325	NTHI477_RS02200	741	A	T	synonymous
461328	NTHI477_RS02200	738	T	C	synonymous
461351	NTHI477_RS02200	715	A	G	synonymous
461358	NTHI477_RS02200	708	A	C	synonymous
461402	NTHI477_RS02200	664	A	C	nonsynonymous
461517	NTHI477_RS02200	549	T	C	synonymous
461519	NTHI477_RS02200	547	G	T	nonsynonymous
461529	NTHI477_RS02200	537	C	T	synonymous
461530	NTHI477_RS02200	536	T	A	nonsynonymous
461533	NTHI477_RS02200	533	G	T	nonsynonymous
461533	NTHI477_RS02200	533	G	A	nonsynonymous
461553	NTHI477_RS02200	513	A	C	synonymous
461564	NTHI477_RS02200	502	A	C	nonsynonymous
461576	NTHI477_RS02200	490	G	T	nonsynonymous
461589	NTHI477_RS02200	477	G	A	synonymous
461619	NTHI477_RS02200	447	A	G	synonymous
461626	NTHI477_RS02200	440	A	G	nonsynonymous
461634	NTHI477_RS02200	432	G	A	synonymous
461655	NTHI477_RS02200	411	C	A	nonsynonymous
461664	NTHI477_RS02200	402	T	C	synonymous
461666	NTHI477_RS02200	400	G	A	synonymous
461671	NTHI477_RS02200	395	G	T	nonsynonymous
461671	NTHI477_RS02200	395	G	A	nonsynonymous
461676	NTHI477_RS02200	390	C	T	synonymous
461683	NTHI477_RS02200	383	T	G	nonsynonymous
461709	NTHI477_RS02200	357	C	T	synonymous
461719	NTHI477_RS02200	347	C	T	nonsynonymous
461741	NTHI477_RS02200	325	C	A	nonsynonymous
461744	NTHI477_RS02200	322	T	A	nonsynonymous
461757	NTHI477_RS02200	309	C	T	synonymous
461760	NTHI477_RS02200	306	G	T	synonymous
461775	NTHI477_RS02200	291	A	T	synonymous
461778	NTHI477_RS02200	288	A	G	synonymous
461787	NTHI477_RS02200	279	C	T	synonymous
461790	NTHI477_RS02200	276	G	T	synonymous
461790	NTHI477_RS02200	276	G	A	synonymous
461796	NTHI477_RS02200	270	A	G	synonymous
461812	NTHI477_RS02200	254	T	G	nonsynonymous
461813	NTHI477_RS02200	253	T	C	nonsynonymous
461814	NTHI477_RS02200	252	A	G	synonymous
461816	NTHI477_RS02200	250	G	A	nonsynonymous

461829	NTHI477_RS02200	237	G	A	synonymous
461855	NTHI477_RS02200	211	T	C	nonsynonymous
461861	NTHI477_RS02200	205	T	G	nonsynonymous
461895	NTHI477_RS02200	171	G	A	synonymous
461919	NTHI477_RS02200	147	T	C	synonymous
461922	NTHI477_RS02200	144	G	T	nonsynonymous
461924	NTHI477_RS02200	142	A	G	nonsynonymous
461951	NTHI477_RS02200	115	A	T	nonsynonymous
461967	NTHI477_RS02200	99	G	T	nonsynonymous
461983	NTHI477_RS02200	83	C	T	nonsynonymous
461985	NTHI477_RS02200	81	T	G	synonymous
461986	NTHI477_RS02200	80	A	G	nonsynonymous
461988	NTHI477_RS02200	78	T	C	synonymous
461993	NTHI477_RS02200	73	C	T	nonsynonymous
462027	NTHI477_RS02200	39	G	A	synonymous
462060	NTHI477_RS02200	6	T	G	synonymous
462087	intergenic	intergenic	C	A	intergenic
462100	intergenic	intergenic	G	A	intergenic
462103	intergenic	intergenic	T	C	intergenic
462105	intergenic	intergenic	T	G	intergenic
462109	intergenic	intergenic	A	G	intergenic
462117	intergenic	intergenic	T	C	intergenic
462129	intergenic	intergenic	C	T	intergenic
462174	NTHI477_RS02205	2047	C	T	nonsynonymous
462180	NTHI477_RS02205	2041	C	T	nonsynonymous
462208	NTHI477_RS02205	2013	A	G	synonymous
462214	NTHI477_RS02205	2007	G	A	synonymous
462226	NTHI477_RS02205	1995	T	C	nonsynonymous
462298	NTHI477_RS02205	1923	T	C	synonymous
462409	NTHI477_RS02205	1812	A	T	nonsynonymous
462415	NTHI477_RS02205	1806	G	A	synonymous
462516	NTHI477_RS02205	1705	C	T	nonsynonymous
462520	NTHI477_RS02205	1701	A	G	synonymous
462522	NTHI477_RS02205	1699	A	C	nonsynonymous
462525	NTHI477_RS02205	1696	C	T	nonsynonymous
462526	NTHI477_RS02205	1695	G	A	synonymous
462542	NTHI477_RS02205	1679	A	G	nonsynonymous
462612	NTHI477_RS02205	1609	A	C	nonsynonymous
462625	NTHI477_RS02205	1596	G	A	synonymous
462628	NTHI477_RS02205	1593	G	A	synonymous
462640	NTHI477_RS02205	1581	G	T	synonymous
462643	NTHI477_RS02205	1578	T	G	synonymous
462678	NTHI477_RS02205	1543	T	C	nonsynonymous
462679	NTHI477_RS02205	1542	A	G	synonymous
462697	NTHI477_RS02205	1524	A	G	synonymous

462706	NTHI477_RS02205	1515	C	T	synonymous
462717	NTHI477_RS02205	1504	A	G	synonymous
462721	NTHI477_RS02205	1500	G	A	synonymous
462754	NTHI477_RS02205	1467	A	G	synonymous
462766	NTHI477_RS02205	1455	C	T	synonymous
462823	NTHI477_RS02205	1398	C	A	nonsynonymous
462844	NTHI477_RS02205	1377	T	C	synonymous
462850	NTHI477_RS02205	1371	G	A	synonymous
462907	NTHI477_RS02205	1314	A	G	synonymous
462919	NTHI477_RS02205	1302	A	G	synonymous
462970	NTHI477_RS02205	1251	G	A	synonymous
462994	NTHI477_RS02205	1227	C	T	synonymous
462997	NTHI477_RS02205	1224	C	G	synonymous
463042	NTHI477_RS02205	1179	A	T	synonymous
463054	NTHI477_RS02205	1167	T	C	synonymous
463087	NTHI477_RS02205	1134	G	A	synonymous
463138	NTHI477_RS02205	1083	G	T	nonsynonymous
463144	NTHI477_RS02205	1077	T	C	synonymous
463162	NTHI477_RS02205	1059	G	A	synonymous
463174	NTHI477_RS02205	1047	A	C	synonymous
463204	NTHI477_RS02205	1017	A	C	synonymous
463224	NTHI477_RS02205	997	A	G	nonsynonymous
463252	NTHI477_RS02205	969	T	C	synonymous
463257	NTHI477_RS02205	964	G	A	nonsynonymous
463321	NTHI477_RS02205	900	G	A	synonymous
463351	NTHI477_RS02205	870	A	G	synonymous
463369	NTHI477_RS02205	852	A	T	synonymous
463378	NTHI477_RS02205	843	C	T	synonymous
463418	NTHI477_RS02205	803	T	C	nonsynonymous
463420	NTHI477_RS02205	801	G	T	synonymous
463427	NTHI477_RS02205	794	A	G	nonsynonymous
463442	NTHI477_RS02205	779	G	A	nonsynonymous
463455	NTHI477_RS02205	766	T	C	nonsynonymous
463462	NTHI477_RS02205	759	G	A	synonymous
463471	NTHI477_RS02205	750	A	G	synonymous
463480	NTHI477_RS02205	741	T	C	nonsynonymous
463482	NTHI477_RS02205	739	T	C	nonsynonymous
463501	NTHI477_RS02205	720	T	C	synonymous
463525	NTHI477_RS02205	696	G	C	synonymous
463546	NTHI477_RS02205	675	C	T	synonymous
463633	NTHI477_RS02205	588	C	T	synonymous
463674	NTHI477_RS02205	547	G	A	nonsynonymous
463696	NTHI477_RS02205	525	A	G	synonymous
463704	NTHI477_RS02205	517	C	T	nonsynonymous
463717	NTHI477_RS02205	504	C	T	synonymous

463738	NTHI477_RS02205	483	C	A	synonymous
463765	NTHI477_RS02205	456	A	G	synonymous
463790	NTHI477_RS02205	431	G	A	nonsynonymous
463834	NTHI477_RS02205	387	G	A	synonymous
463843	NTHI477_RS02205	378	C	G	nonsynonymous
463845	NTHI477_RS02205	376	T	A	nonsynonymous
463861	NTHI477_RS02205	360	G	A	synonymous
463861	NTHI477_RS02205	360	G	C	nonsynonymous
463870	NTHI477_RS02205	351	C	T	synonymous
463870	NTHI477_RS02205	351	C	A	synonymous
463889	NTHI477_RS02205	332	C	T	nonsynonymous
463891	NTHI477_RS02205	330	C	T	synonymous
463897	NTHI477_RS02205	324	T	A	nonsynonymous
463910	NTHI477_RS02205	311	G	T	nonsynonymous
463934	NTHI477_RS02205	287	T	C	nonsynonymous
463964	NTHI477_RS02205	257	C	T	nonsynonymous
463991	NTHI477_RS02205	230	T	C	nonsynonymous
464056	NTHI477_RS02205	165	T	C	synonymous
464079	NTHI477_RS02205	142	A	G	synonymous
464084	NTHI477_RS02205	137	C	T	nonsynonymous
464105	NTHI477_RS02205	116	A	G	nonsynonymous
464115	NTHI477_RS02205	106	C	A	nonsynonymous
464122	NTHI477_RS02205	99	T	C	synonymous
464128	NTHI477_RS02205	93	T	C	synonymous
464129	NTHI477_RS02205	92	G	T	nonsynonymous
464170	NTHI477_RS02205	51	C	T	synonymous
464183	NTHI477_RS02205	38	G	A	nonsynonymous
464184	NTHI477_RS02205	37	C	T	nonsynonymous
464242	intergenic	intergenic	T	C	intergenic
464269	NTHI477_RS02210	618	A	C	synonymous
464270	NTHI477_RS02210	617	G	A	nonsynonymous
464290	NTHI477_RS02210	597	A	G	synonymous
464421	NTHI477_RS02210	466	A	C	nonsynonymous
464439	NTHI477_RS02210	448	G	A	nonsynonymous
464452	NTHI477_RS02210	435	A	T	synonymous
464461	NTHI477_RS02210	426	C	T	synonymous
464462	NTHI477_RS02210	425	G	A	nonsynonymous
464465	NTHI477_RS02210	422	T	C	nonsynonymous
464469	NTHI477_RS02210	418	T	G	nonsynonymous
464492	NTHI477_RS02210	395	G	A	nonsynonymous
464494	NTHI477_RS02210	393	A	G	synonymous
464503	NTHI477_RS02210	384	T	C	synonymous
464521	NTHI477_RS02210	366	A	G	synonymous
464524	NTHI477_RS02210	363	C	T	synonymous
464563	NTHI477_RS02210	324	G	A	synonymous

464584	NTHI477_RS02210	303	C	T	synonymous
464617	NTHI477_RS02210	270	A	G	synonymous
464656	NTHI477_RS02210	231	G	A	synonymous
464662	NTHI477_RS02210	225	G	A	synonymous
464680	NTHI477_RS02210	207	T	C	synonymous
464695	NTHI477_RS02210	192	G	A	synonymous
464698	NTHI477_RS02210	189	T	C	synonymous
464746	NTHI477_RS02210	141	T	C	synonymous
464761	NTHI477_RS02210	126	G	A	synonymous
464805	NTHI477_RS02210	82	T	C	nonsynonymous
464821	NTHI477_RS02210	66	G	A	synonymous
464888	intergenic	intergenic	G	T	intergenic
464914	intergenic	intergenic	A	G	intergenic
464926	intergenic	intergenic	C	T	intergenic
465038	intergenic	intergenic	C	T	intergenic
465039	intergenic	intergenic	A	G	intergenic
465081	NTHI477_RS02215	11	C	A	nonsynonymous
465148	NTHI477_RS02215	78	T	G	synonymous
465204	NTHI477_RS02215	134	A	C	nonsynonymous
465209	NTHI477_RS02215	139	G	A	nonsynonymous
465217	NTHI477_RS02215	147	G	A	synonymous
465283	NTHI477_RS02215	213	C	T	synonymous
465310	NTHI477_RS02215	240	A	G	synonymous
465313	NTHI477_RS02215	243	C	T	synonymous
465320	NTHI477_RS02215	250	A	G	nonsynonymous
465325	NTHI477_RS02215	255	C	T	synonymous
465343	NTHI477_RS02215	273	C	T	synonymous
465361	NTHI477_RS02215	291	G	A	synonymous
465379	NTHI477_RS02215	309	C	T	synonymous
465379	NTHI477_RS02215	309	C	A	synonymous
465421	NTHI477_RS02215	351	T	G	synonymous
465436	NTHI477_RS02215	366	T	G	synonymous
465475	NTHI477_RS02215	405	A	T	synonymous
465544	NTHI477_RS02215	474	T	C	synonymous
465547	NTHI477_RS02215	477	G	A	synonymous
465631	NTHI477_RS02215	561	C	T	synonymous
465634	NTHI477_RS02215	564	A	G	synonymous
465635	NTHI477_RS02215	565	C	T	synonymous
465676	NTHI477_RS02215	606	A	G	synonymous
465682	NTHI477_RS02215	612	T	C	synonymous
465691	NTHI477_RS02215	621	T	C	synonymous
465695	NTHI477_RS02215	625	C	T	nonsynonymous
465739	NTHI477_RS02215	669	T	C	synonymous
465745	NTHI477_RS02215	675	A	C	synonymous
465748	NTHI477_RS02215	678	T	C	synonymous

465764	NTHI477_RS02215	694	T	C	nonsynonymous
465784	NTHI477_RS02215	714	A	G	synonymous
465790	NTHI477_RS02215	720	T	C	synonymous
465844	NTHI477_RS02215	774	G	A	synonymous
465925	NTHI477_RS02215	855	T	C	synonymous
465943	NTHI477_RS02215	873	G	T	synonymous
465943	NTHI477_RS02215	873	G	A	synonymous
466003	NTHI477_RS02215	933	G	A	synonymous
466049	NTHI477_RS02215	979	C	T	nonsynonymous
466054	NTHI477_RS02215	984	T	C	synonymous
466061	NTHI477_RS02215	991	G	A	nonsynonymous
466114	NTHI477_RS02215	1044	A	G	synonymous
466150	NTHI477_RS02215	1080	C	T	synonymous
466219	NTHI477_RS02215	1149	G	C	synonymous
466219	NTHI477_RS02215	1149	G	A	synonymous
466225	NTHI477_RS02215	1155	C	T	synonymous
466226	NTHI477_RS02215	1156	G	A	nonsynonymous
466240	NTHI477_RS02215	1170	C	T	synonymous
466273	NTHI477_RS02215	1203	A	T	synonymous
466311	NTHI477_RS02215	1241	G	A	nonsynonymous
466311	NTHI477_RS02220	7	G	A	nonsynonymous
466313	NTHI477_RS02215	1243	G	C	nonsynonymous
466313	NTHI477_RS02220	9	G	C	nonsynonymous
466354	NTHI477_RS02220	50	T	G	nonsynonymous
466358	NTHI477_RS02220	54	A	G	synonymous
466361	NTHI477_RS02220	57	C	G	synonymous
466376	NTHI477_RS02220	72	A	C	synonymous
466388	NTHI477_RS02220	84	G	A	synonymous
466460	NTHI477_RS02220	156	C	T	synonymous
466475	NTHI477_RS02220	171	A	G	synonymous
466481	NTHI477_RS02220	177	T	C	synonymous
466485	NTHI477_RS02220	181	G	A	nonsynonymous
466487	NTHI477_RS02220	183	T	C	synonymous
466496	NTHI477_RS02220	192	T	A	synonymous
466499	NTHI477_RS02220	195	C	T	synonymous
466502	NTHI477_RS02220	198	T	C	synonymous
466505	NTHI477_RS02220	201	G	T	synonymous
466508	NTHI477_RS02220	204	A	G	synonymous
466558	NTHI477_RS02220	254	A	G	nonsynonymous
466580	NTHI477_RS02220	276	A	G	synonymous
466589	NTHI477_RS02220	285	A	G	synonymous
466596	NTHI477_RS02220	292	A	G	nonsynonymous
466598	NTHI477_RS02220	294	T	A	synonymous
466601	NTHI477_RS02220	297	A	C	synonymous
466616	NTHI477_RS02220	312	G	A	synonymous

466619	NTHI477_RS02220	315	T	A	synonymous
466624	NTHI477_RS02220	320	A	G	nonsynonymous
466631	NTHI477_RS02220	327	A	G	synonymous
466638	NTHI477_RS02220	334	G	A	nonsynonymous
466640	NTHI477_RS02220	336	C	T	synonymous
466658	NTHI477_RS02220	354	A	G	synonymous
466673	NTHI477_RS02220	369	C	A	synonymous
466673	NTHI477_RS02220	369	C	T	synonymous
466679	NTHI477_RS02220	375	A	T	synonymous
466682	NTHI477_RS02220	378	C	A	synonymous
466721	NTHI477_RS02220	417	C	T	synonymous
466733	NTHI477_RS02220	429	G	A	synonymous
466747	NTHI477_RS02220	443	G	A	nonsynonymous
466778	NTHI477_RS02220	474	G	C	synonymous
466787	NTHI477_RS02220	483	G	A	synonymous
466796	NTHI477_RS02220	492	A	C	synonymous
466799	NTHI477_RS02220	495	A	G	synonymous
466805	NTHI477_RS02220	501	C	T	synonymous
466808	NTHI477_RS02220	504	G	A	synonymous
466811	NTHI477_RS02220	507	G	A	synonymous
466814	NTHI477_RS02220	510	C	T	synonymous
466859	NTHI477_RS02220	555	C	T	synonymous
466880	NTHI477_RS02220	576	T	C	synonymous
466940	NTHI477_RS02220	636	C	T	synonymous
466956	NTHI477_RS02220	652	G	A	nonsynonymous
467009	NTHI477_RS02220	705	T	G	synonymous
467009	NTHI477_RS02220	705	T	A	synonymous
467012	NTHI477_RS02220	708	T	C	synonymous
467021	NTHI477_RS02220	717	G	A	synonymous
467022	NTHI477_RS02220	718	T	G	nonsynonymous
467033	NTHI477_RS02220	729	C	T	synonymous
467055	NTHI477_RS02220	751	G	T	nonsynonymous
467057	NTHI477_RS02220	753	C	A	synonymous
467057	NTHI477_RS02220	753	C	G	synonymous
467063	NTHI477_RS02220	759	A	G	synonymous
467066	NTHI477_RS02220	762	G	A	synonymous
467069	NTHI477_RS02220	765	G	T	synonymous
467072	NTHI477_RS02220	768	G	C	synonymous
467091	NTHI477_RS02220	787	G	A	nonsynonymous
467101	NTHI477_RS02220	797	C	T	nonsynonymous
467109	NTHI477_RS02220	805	A	G	nonsynonymous
467144	NTHI477_RS02220	840	A	G	synonymous
467175	NTHI477_RS02220	871	G	A	nonsynonymous
467180	NTHI477_RS02220	876	T	A	synonymous
467180	NTHI477_RS02220	876	T	C	synonymous

467186	NTHI477_RS02220	882	T	A	synonymous
467187	NTHI477_RS02220	883	G	A	nonsynonymous
467193	NTHI477_RS02220	889	A	T	nonsynonymous
467195	NTHI477_RS02220	891	C	A	synonymous
467206	NTHI477_RS02220	902	C	T	nonsynonymous
467207	NTHI477_RS02220	903	A	G	synonymous
467252	NTHI477_RS02220	948	C	T	synonymous
467270	NTHI477_RS02220	966	G	A	synonymous
467276	NTHI477_RS02220	972	T	C	synonymous
467294	NTHI477_RS02220	990	A	G	synonymous
467309	NTHI477_RS02220	1005	C	A	synonymous
467339	NTHI477_RS02220	1035	A	G	synonymous
467345	NTHI477_RS02220	1041	T	C	synonymous
467372	NTHI477_RS02220	1068	G	A	nonsynonymous
467378	NTHI477_RS02220	1074	A	T	synonymous
467379	NTHI477_RS02220	1075	G	A	nonsynonymous
467396	NTHI477_RS02220	1092	A	C	synonymous
467413	NTHI477_RS02220	1109	A	G	nonsynonymous
467498	NTHI477_RS02220	1194	A	G	synonymous
467522	NTHI477_RS02220	1218	C	T	synonymous
467534	NTHI477_RS02220	1230	G	A	synonymous
467540	NTHI477_RS02220	1236	C	T	synonymous
467543	NTHI477_RS02220	1239	C	T	synonymous
467546	NTHI477_RS02220	1242	C	A	synonymous
467576	NTHI477_RS02220	1272	T	C	synonymous
467609	NTHI477_RS02220	1305	T	A	synonymous
467624	NTHI477_RS02220	1320	G	A	synonymous
467657	NTHI477_RS02220	1353	C	T	synonymous
467670	NTHI477_RS02220	1366	G	A	nonsynonymous
467673	NTHI477_RS02220	1369	G	A	nonsynonymous
467678	NTHI477_RS02220	1374	A	T	synonymous
467690	NTHI477_RS02220	1386	A	T	synonymous
467702	NTHI477_RS02220	1398	T	C	synonymous
467712	NTHI477_RS02220	1408	A	G	nonsynonymous
467741	NTHI477_RS02220	1437	C	T	synonymous
467744	NTHI477_RS02220	1440	A	G	synonymous
467747	NTHI477_RS02220	1443	G	A	synonymous
467751	NTHI477_RS02220	1447	G	A	nonsynonymous
467768	NTHI477_RS02220	1464	C	T	synonymous
467771	NTHI477_RS02220	1467	T	C	synonymous
467780	NTHI477_RS02220	1476	A	G	synonymous
467783	NTHI477_RS02220	1479	A	G	synonymous
467784	NTHI477_RS02220	1480	A	G	nonsynonymous
467785	NTHI477_RS02220	1481	A	C	nonsynonymous
467794	NTHI477_RS02220	1490	C	G	nonsynonymous

467796	NTHI477_RS02220	1492	C	T	nonsynonymous
467797	NTHI477_RS02220	1493	A	T	nonsynonymous
467822	NTHI477_RS02220	1518	A	G	synonymous
467837	NTHI477_RS02220	1533	T	C	synonymous
467846	NTHI477_RS02220	1542	A	T	synonymous
467852	NTHI477_RS02220	1548	C	T	synonymous
467855	NTHI477_RS02220	1551	T	C	synonymous
467858	NTHI477_RS02220	1554	C	T	synonymous
467864	NTHI477_RS02220	1560	C	A	synonymous
467865	NTHI477_RS02220	1561	G	A	nonsynonymous
467882	NTHI477_RS02220	1578	A	G	synonymous
467894	NTHI477_RS02220	1590	T	C	synonymous
467903	NTHI477_RS02220	1599	A	C	synonymous
467903	NTHI477_RS02220	1599	A	T	synonymous
467911	NTHI477_RS02220	1607	G	A	nonsynonymous
467912	NTHI477_RS02220	1608	T	C	synonymous
467921	NTHI477_RS02220	1617	A	G	synonymous
467924	NTHI477_RS02220	1620	T	C	synonymous
467930	NTHI477_RS02220	1626	C	T	synonymous
467933	NTHI477_RS02220	1629	A	C	synonymous
467952	NTHI477_RS02220	1648	G	A	nonsynonymous
467954	NTHI477_RS02220	1650	A	G	synonymous
467984	NTHI477_RS02220	1680	G	A	synonymous
467989	NTHI477_RS02220	1685	G	A	nonsynonymous
468002	NTHI477_RS02220	1698	A	C	nonsynonymous
468020	NTHI477_RS02220	1716	C	T	synonymous
468039	NTHI477_RS02220	1735	G	A	nonsynonymous
468068	NTHI477_RS02220	1764	A	G	synonymous
468071	NTHI477_RS02220	1767	A	G	synonymous
468074	NTHI477_RS02220	1770	A	G	synonymous
468077	NTHI477_RS02220	1773	T	A	synonymous
468083	NTHI477_RS02220	1779	C	A	synonymous
468085	NTHI477_RS02220	1781	G	C	nonsynonymous
468086	NTHI477_RS02220	1782	T	A	nonsynonymous
468089	NTHI477_RS02220	1785	A	G	synonymous
468095	NTHI477_RS02220	1791	T	C	synonymous
468113	NTHI477_RS02220	1809	G	A	synonymous
468156	NTHI477_RS02220	1852	G	A	nonsynonymous
468158	NTHI477_RS02220	1854	C	T	synonymous
468161	NTHI477_RS02220	1857	A	T	synonymous
468167	NTHI477_RS02220	1863	T	C	synonymous
468197	NTHI477_RS02220	1893	C	T	synonymous
468212	NTHI477_RS02220	1908	C	T	synonymous
468215	NTHI477_RS02220	1911	G	A	synonymous
468215	NTHI477_RS02220	1911	G	T	synonymous

468221	NTHI477_RS02220	1917	C	A	synonymous
468224	NTHI477_RS02220	1920	C	T	synonymous
468227	NTHI477_RS02220	1923	G	A	synonymous
468272	NTHI477_RS02220	1968	G	A	synonymous
468275	NTHI477_RS02220	1971	T	C	synonymous
468279	NTHI477_RS02220	1975	T	C	synonymous
468281	NTHI477_RS02220	1977	A	T	nonsynonymous
468284	NTHI477_RS02220	1980	C	T	synonymous
468308	NTHI477_RS02220	2004	C	T	synonymous
468314	NTHI477_RS02220	2010	G	T	nonsynonymous
468329	NTHI477_RS02220	2025	A	G	synonymous
468338	NTHI477_RS02220	2034	T	C	synonymous
468364	NTHI477_RS02220	2060	A	G	nonsynonymous
468389	NTHI477_RS02220	2085	A	G	synonymous
468392	NTHI477_RS02220	2088	T	G	synonymous
468393	NTHI477_RS02220	2089	T	G	nonsynonymous
468396	NTHI477_RS02220	2092	C	A	nonsynonymous
468398	NTHI477_RS02220	2094	C	T	synonymous
468413	NTHI477_RS02220	2109	G	T	synonymous
468425	NTHI477_RS02220	2121	A	G	synonymous
468428	NTHI477_RS02220	2124	A	T	synonymous
468431	NTHI477_RS02220	2127	T	C	synonymous
468443	NTHI477_RS02220	2139	C	T	synonymous
468461	NTHI477_RS02220	2157	A	G	synonymous
468491	NTHI477_RS02220	2187	C	A	synonymous
468504	NTHI477_RS02220	2200	C	A	nonsynonymous
468518	NTHI477_RS02220	2214	G	A	synonymous
468524	NTHI477_RS02220	2220	A	G	synonymous
468533	NTHI477_RS02220	2229	A	G	synonymous
468557	NTHI477_RS02220	2253	G	A	synonymous
468575	NTHI477_RS02220	2271	G	A	synonymous
468644	NTHI477_RS02220	2340	G	A	synonymous
468666	NTHI477_RS02220	2362	G	A	nonsynonymous
468677	NTHI477_RS02220	2373	A	G	synonymous
468690	NTHI477_RS02220	2386	G	A	nonsynonymous
468692	NTHI477_RS02220	2388	C	A	synonymous
468782	NTHI477_RS02220	2478	G	A	synonymous
468785	NTHI477_RS02220	2481	A	C	synonymous
468794	NTHI477_RS02220	2490	A	G	synonymous
468800	NTHI477_RS02220	2496	T	C	synonymous
468857	NTHI477_RS02220	2553	T	C	synonymous
468869	NTHI477_RS02220	2565	A	G	synonymous
468869	NTHI477_RS02220	2565	A	T	synonymous
468890	NTHI477_RS02220	2586	G	A	synonymous
468912	NTHI477_RS02220	2608	G	A	nonsynonymous

468935	NTHI477_RS02220	2631	T	C	synonymous
468943	NTHI477_RS02220	2639	C	T	nonsynonymous
468983	intergenic	intergenic	G	A	intergenic
468997	intergenic	intergenic	G	A	intergenic
469003	intergenic	intergenic	G	T	intergenic
469032	NTHI477_RS02225	426	G	A	synonymous
469067	NTHI477_RS02225	391	T	G	nonsynonymous
469086	NTHI477_RS02225	372	G	A	synonymous
469089	NTHI477_RS02225	369	C	T	synonymous
469092	NTHI477_RS02225	366	A	T	synonymous
469098	NTHI477_RS02225	360	G	A	synonymous
469106	NTHI477_RS02225	352	A	G	synonymous
469107	NTHI477_RS02225	351	A	G	synonymous
469110	NTHI477_RS02225	348	A	G	synonymous
469116	NTHI477_RS02225	342	A	G	synonymous
469119	NTHI477_RS02225	339	A	G	synonymous
469125	NTHI477_RS02225	333	A	G	synonymous
469128	NTHI477_RS02225	330	A	G	synonymous
469131	NTHI477_RS02225	327	G	A	synonymous
469133	NTHI477_RS02225	325	G	A	nonsynonymous
469134	NTHI477_RS02225	324	G	A	synonymous
469137	NTHI477_RS02225	321	A	G	synonymous
469152	NTHI477_RS02225	306	C	T	synonymous
469152	NTHI477_RS02225	306	C	A	synonymous
469155	NTHI477_RS02225	303	G	A	synonymous
469158	NTHI477_RS02225	300	G	A	synonymous
469167	NTHI477_RS02225	291	T	C	synonymous
469170	NTHI477_RS02225	288	C	T	synonymous
469173	NTHI477_RS02225	285	C	A	synonymous
469185	NTHI477_RS02225	273	G	A	synonymous
469203	NTHI477_RS02225	255	A	G	synonymous
469242	NTHI477_RS02225	216	G	T	synonymous
469242	NTHI477_RS02225	216	G	C	synonymous
469242	NTHI477_RS02225	216	G	A	synonymous
469243	NTHI477_RS02225	215	G	A	nonsynonymous
469246	NTHI477_RS02225	212	T	G	nonsynonymous
469251	NTHI477_RS02225	207	T	C	synonymous
469253	NTHI477_RS02225	205	G	C	nonsynonymous
469255	NTHI477_RS02225	203	G	T	nonsynonymous
469257	NTHI477_RS02225	201	A	T	synonymous
469267	NTHI477_RS02225	191	T	C	nonsynonymous
469268	NTHI477_RS02225	190	T	G	nonsynonymous
469269	NTHI477_RS02225	189	C	T	synonymous
469272	NTHI477_RS02225	186	C	G	synonymous
469281	NTHI477_RS02225	177	A	T	synonymous

469305	NTHI477_RS02225	153	A	G	synonymous
469308	NTHI477_RS02225	150	C	T	synonymous
469311	NTHI477_RS02225	147	A	G	synonymous
469317	NTHI477_RS02225	141	T	C	synonymous
469321	NTHI477_RS02225	137	G	T	nonsynonymous
469322	NTHI477_RS02225	136	C	T	nonsynonymous
469326	NTHI477_RS02225	132	G	A	synonymous
469350	NTHI477_RS02225	108	G	A	synonymous
469353	NTHI477_RS02225	105	C	A	synonymous
469359	NTHI477_RS02225	99	T	A	synonymous
469371	NTHI477_RS02225	87	G	A	synonymous
469376	NTHI477_RS02225	82	C	T	nonsynonymous
469377	NTHI477_RS02225	81	C	T	synonymous
469377	NTHI477_RS02225	81	C	A	synonymous
469384	NTHI477_RS02225	74	A	T	nonsynonymous
469386	NTHI477_RS02225	72	C	T	synonymous
469387	NTHI477_RS02225	71	T	G	nonsynonymous
469389	NTHI477_RS02225	69	A	T	synonymous
469401	NTHI477_RS02225	57	A	G	synonymous
469407	NTHI477_RS02225	51	G	C	synonymous
469424	NTHI477_RS02225	34	A	G	nonsynonymous
469425	NTHI477_RS02225	33	C	A	nonsynonymous
469437	NTHI477_RS02225	21	C	T	synonymous
469439	NTHI477_RS02225	19	C	A	nonsynonymous
469452	NTHI477_RS02225	6	C	T	synonymous
469464	NTHI477_RS02230	240	T	C	synonymous
469473	NTHI477_RS02230	231	T	G	synonymous
469473	NTHI477_RS02230	231	T	C	synonymous
469485	NTHI477_RS02230	219	G	A	synonymous
469506	NTHI477_RS02230	198	T	G	synonymous
469521	NTHI477_RS02230	183	A	C	synonymous
469521	NTHI477_RS02230	183	A	T	synonymous
469527	NTHI477_RS02230	177	T	C	synonymous
469530	NTHI477_RS02230	174	G	A	synonymous
469539	NTHI477_RS02230	165	A	G	synonymous
469542	NTHI477_RS02230	162	T	A	synonymous
469542	NTHI477_RS02230	162	T	C	synonymous
469545	NTHI477_RS02230	159	C	G	synonymous
469545	NTHI477_RS02230	159	C	T	synonymous
469554	NTHI477_RS02230	150	T	C	synonymous
469557	NTHI477_RS02230	147	A	C	synonymous
469557	NTHI477_RS02230	147	A	G	synonymous
469566	NTHI477_RS02230	138	C	T	synonymous
469568	NTHI477_RS02230	136	G	A	synonymous
469569	NTHI477_RS02230	135	T	C	synonymous

469572	NTHI477_RS02230	132	C	T	synonymous
469575	NTHI477_RS02230	129	T	C	synonymous
469581	NTHI477_RS02230	123	T	C	synonymous
469582	NTHI477_RS02230	122	T	C	nonsynonymous
469587	NTHI477_RS02230	117	A	G	synonymous
469590	NTHI477_RS02230	114	C	T	synonymous
469602	NTHI477_RS02230	102	G	A	synonymous
469604	NTHI477_RS02230	100	G	A	nonsynonymous
469615	NTHI477_RS02230	89	G	T	nonsynonymous
469620	NTHI477_RS02230	84	C	T	synonymous
469623	NTHI477_RS02230	81	A	T	synonymous
469626	NTHI477_RS02230	78	G	C	synonymous
469636	NTHI477_RS02230	68	T	C	nonsynonymous
469641	NTHI477_RS02230	63	T	A	nonsynonymous
469643	NTHI477_RS02230	61	A	G	synonymous
469644	NTHI477_RS02230	60	T	C	synonymous
469650	NTHI477_RS02230	54	G	A	synonymous
469652	NTHI477_RS02230	52	C	A	nonsynonymous
469656	NTHI477_RS02230	48	A	T	synonymous
469657	NTHI477_RS02230	47	A	T	nonsynonymous
469658	NTHI477_RS02230	46	C	T	nonsynonymous
469659	NTHI477_RS02230	45	G	A	synonymous
469692	NTHI477_RS02230	12	A	T	synonymous
469694	NTHI477_RS02230	10	C	T	nonsynonymous
469720	NTHI477_RS02235	468	A	C	nonsynonymous
469722	NTHI477_RS02235	466	T	C	nonsynonymous
469729	NTHI477_RS02235	459	G	A	synonymous
469732	NTHI477_RS02235	456	T	G	synonymous
469732	NTHI477_RS02235	456	T	A	synonymous
469746	NTHI477_RS02235	442	T	A	nonsynonymous
469758	NTHI477_RS02235	430	G	A	synonymous
469759	NTHI477_RS02235	429	G	A	synonymous
469765	NTHI477_RS02235	423	G	T	nonsynonymous
469767	NTHI477_RS02235	421	G	T	nonsynonymous
469777	NTHI477_RS02235	411	T	A	synonymous
469777	NTHI477_RS02235	411	T	C	nonsynonymous
469786	NTHI477_RS02235	402	T	C	synonymous
469789	NTHI477_RS02235	399	T	C	synonymous
469792	NTHI477_RS02235	396	G	A	synonymous
469819	NTHI477_RS02235	369	G	A	synonymous
469822	NTHI477_RS02235	366	T	G	synonymous
469825	NTHI477_RS02235	363	T	A	synonymous
469832	NTHI477_RS02235	356	G	A	nonsynonymous
469834	NTHI477_RS02235	354	C	A	synonymous
469834	NTHI477_RS02235	354	C	G	synonymous

469834	NTHI477_RS02235	354	C	T	synonymous
469837	NTHI477_RS02235	351	A	G	synonymous
469837	NTHI477_RS02235	351	A	C	synonymous
469842	NTHI477_RS02235	346	G	A	synonymous
469843	NTHI477_RS02235	345	T	A	synonymous
469855	NTHI477_RS02235	333	T	C	synonymous
469870	NTHI477_RS02235	318	A	G	synonymous
469885	NTHI477_RS02235	303	A	G	synonymous
469894	NTHI477_RS02235	294	G	A	synonymous
469901	NTHI477_RS02235	287	T	C	nonsynonymous
469903	NTHI477_RS02235	285	G	A	synonymous
469906	NTHI477_RS02235	282	G	T	synonymous
469906	NTHI477_RS02235	282	G	A	synonymous
469916	NTHI477_RS02235	272	A	G	nonsynonymous
469918	NTHI477_RS02235	270	C	T	synonymous
469924	NTHI477_RS02235	264	T	C	synonymous
469928	NTHI477_RS02235	260	T	C	nonsynonymous
469930	NTHI477_RS02235	258	C	T	synonymous
469936	NTHI477_RS02235	252	T	C	synonymous
469942	NTHI477_RS02235	246	A	G	synonymous
469951	NTHI477_RS02235	237	C	T	synonymous
469954	NTHI477_RS02235	234	T	C	synonymous
469966	NTHI477_RS02235	222	C	T	synonymous
469975	NTHI477_RS02235	213	T	C	synonymous
469984	NTHI477_RS02235	204	G	A	synonymous
469993	NTHI477_RS02235	195	T	G	synonymous
469993	NTHI477_RS02235	195	T	A	synonymous
469993	NTHI477_RS02235	195	T	C	synonymous
470005	NTHI477_RS02235	183	C	T	synonymous
470014	NTHI477_RS02235	174	A	T	synonymous
470017	NTHI477_RS02235	171	C	A	synonymous
470017	NTHI477_RS02235	171	C	T	synonymous
470026	NTHI477_RS02235	162	A	T	synonymous
470026	NTHI477_RS02235	162	A	C	synonymous
470035	NTHI477_RS02235	153	T	C	synonymous
470038	NTHI477_RS02235	150	G	A	synonymous
470047	NTHI477_RS02235	141	A	G	synonymous
470056	NTHI477_RS02235	132	G	A	synonymous
470062	NTHI477_RS02235	126	A	G	synonymous
470064	NTHI477_RS02235	124	C	A	nonsynonymous
470068	NTHI477_RS02235	120	G	A	synonymous
470077	NTHI477_RS02235	111	A	T	synonymous
470083	NTHI477_RS02235	105	G	A	synonymous
470086	NTHI477_RS02235	102	A	T	synonymous
470086	NTHI477_RS02235	102	A	C	synonymous

470089	NTHI477_RS02235	99	G	A	synonymous
470092	NTHI477_RS02235	96	A	G	synonymous
470100	NTHI477_RS02235	88	C	A	nonsynonymous
470104	NTHI477_RS02235	84	A	G	synonymous
470110	NTHI477_RS02235	78	G	A	synonymous
470116	NTHI477_RS02235	72	A	T	synonymous
470116	NTHI477_RS02235	72	A	G	synonymous
470119	NTHI477_RS02235	69	C	T	synonymous
470122	NTHI477_RS02235	66	T	C	synonymous
470125	NTHI477_RS02235	63	T	C	synonymous
470128	NTHI477_RS02235	60	T	A	synonymous
470129	NTHI477_RS02235	59	G	A	nonsynonymous
470173	NTHI477_RS02235	15	T	C	synonymous
470191	intergenic	intergenic	A	G	intergenic
470193	intergenic	intergenic	G	A	intergenic
470216	intergenic	intergenic	A	G	intergenic
470239	intergenic	intergenic	A	G	intergenic
470241	intergenic	intergenic	A	G	intergenic
470244	intergenic	intergenic	G	A	intergenic
470287	NTHI477_RS02240	1005	G	A	synonymous
470293	NTHI477_RS02240	999	C	A	synonymous
470311	NTHI477_RS02240	981	A	G	synonymous
470315	NTHI477_RS02240	977	G	C	nonsynonymous
470320	NTHI477_RS02240	972	G	A	synonymous
470350	NTHI477_RS02240	942	G	A	synonymous
470356	NTHI477_RS02240	936	A	C	synonymous
470374	NTHI477_RS02240	918	C	T	synonymous
470374	NTHI477_RS02240	918	C	A	synonymous
470377	NTHI477_RS02240	915	T	C	synonymous
470387	NTHI477_RS02240	905	G	C	nonsynonymous
470398	NTHI477_RS02240	894	G	A	synonymous
470407	NTHI477_RS02240	885	C	T	synonymous
470449	NTHI477_RS02240	843	G	A	synonymous
470461	NTHI477_RS02240	831	A	G	synonymous
470464	NTHI477_RS02240	828	T	C	synonymous
470467	NTHI477_RS02240	825	T	C	synonymous
470496	NTHI477_RS02240	796	C	T	nonsynonymous
470497	NTHI477_RS02240	795	G	A	synonymous
470500	NTHI477_RS02240	792	A	G	synonymous
470509	NTHI477_RS02240	783	T	C	synonymous
470527	NTHI477_RS02240	765	A	G	synonymous
470539	NTHI477_RS02240	753	C	T	synonymous
470539	NTHI477_RS02240	753	C	A	synonymous
470541	NTHI477_RS02240	751	C	T	nonsynonymous
470549	NTHI477_RS02240	743	G	A	nonsynonymous

470550	NTHI477_RS02240	742	A	G	nonsynonymous
470554	NTHI477_RS02240	738	C	T	synonymous
470557	NTHI477_RS02240	735	A	G	synonymous
470560	NTHI477_RS02240	732	G	T	synonymous
470569	NTHI477_RS02240	723	A	T	synonymous
470572	NTHI477_RS02240	720	G	A	synonymous
470578	NTHI477_RS02240	714	C	A	synonymous
470584	NTHI477_RS02240	708	G	A	synonymous
470600	NTHI477_RS02240	692	A	G	nonsynonymous
470607	NTHI477_RS02240	685	C	T	nonsynonymous
470626	NTHI477_RS02240	666	C	G	synonymous
470631	NTHI477_RS02240	661	A	G	synonymous
470634	NTHI477_RS02240	658	C	T	nonsynonymous
470641	NTHI477_RS02240	651	A	T	synonymous
470646	NTHI477_RS02240	646	G	T	nonsynonymous
470651	NTHI477_RS02240	641	T	A	nonsynonymous
470654	NTHI477_RS02240	638	C	T	nonsynonymous
470659	NTHI477_RS02240	633	A	G	synonymous
470689	NTHI477_RS02240	603	C	G	nonsynonymous
470689	NTHI477_RS02240	603	C	T	synonymous
470691	NTHI477_RS02240	601	A	G	synonymous
470692	NTHI477_RS02240	600	C	T	synonymous
470695	NTHI477_RS02240	597	A	G	synonymous
470716	NTHI477_RS02240	576	G	A	synonymous
470721	NTHI477_RS02240	571	C	G	nonsynonymous
470725	NTHI477_RS02240	567	C	A	synonymous
470725	NTHI477_RS02240	567	C	T	synonymous
470732	NTHI477_RS02240	560	A	G	nonsynonymous
470734	NTHI477_RS02240	558	A	G	synonymous
470737	NTHI477_RS02240	555	G	A	synonymous
470743	NTHI477_RS02240	549	T	C	synonymous
470746	NTHI477_RS02240	546	A	G	synonymous
470749	NTHI477_RS02240	543	C	T	synonymous
470755	NTHI477_RS02240	537	G	A	synonymous
470763	NTHI477_RS02240	529	A	G	synonymous
470773	NTHI477_RS02240	519	T	C	synonymous
470791	NTHI477_RS02240	501	G	A	synonymous
470800	NTHI477_RS02240	492	A	G	synonymous
470803	NTHI477_RS02240	489	G	A	synonymous
470818	NTHI477_RS02240	474	C	A	synonymous
470824	NTHI477_RS02240	468	A	G	synonymous
470839	NTHI477_RS02240	453	A	G	synonymous
470844	NTHI477_RS02240	448	C	T	nonsynonymous
470854	NTHI477_RS02240	438	G	A	synonymous
470857	NTHI477_RS02240	435	A	G	synonymous

470857	NTHI477_RS02240	435	A	T	synonymous
470860	NTHI477_RS02240	432	C	T	synonymous
470860	NTHI477_RS02240	432	C	A	synonymous
470869	NTHI477_RS02240	423	G	A	synonymous
470881	NTHI477_RS02240	411	T	A	synonymous
470881	NTHI477_RS02240	411	T	G	synonymous
470881	NTHI477_RS02240	411	T	C	synonymous
470887	NTHI477_RS02240	405	T	A	nonsynonymous
470889	NTHI477_RS02240	403	A	G	synonymous
470896	NTHI477_RS02240	396	A	C	synonymous
470899	NTHI477_RS02240	393	A	G	synonymous
470902	NTHI477_RS02240	390	G	A	synonymous
470905	NTHI477_RS02240	387	A	G	synonymous
470920	NTHI477_RS02240	372	C	T	synonymous
470924	NTHI477_RS02240	368	A	G	nonsynonymous
470926	NTHI477_RS02240	366	C	T	synonymous
470928	NTHI477_RS02240	364	T	G	nonsynonymous
470929	NTHI477_RS02240	363	T	C	synonymous
470974	NTHI477_RS02240	318	A	G	synonymous
470977	NTHI477_RS02240	315	G	A	synonymous
470980	NTHI477_RS02240	312	T	C	synonymous
470987	NTHI477_RS02240	305	C	T	nonsynonymous
470989	NTHI477_RS02240	303	A	G	synonymous
470992	NTHI477_RS02240	300	G	A	synonymous
470992	NTHI477_RS02240	300	G	T	synonymous
470995	NTHI477_RS02240	297	C	A	nonsynonymous
471001	NTHI477_RS02240	291	G	A	synonymous
471006	NTHI477_RS02240	286	C	T	nonsynonymous
471016	NTHI477_RS02240	276	A	G	synonymous
471016	NTHI477_RS02240	276	A	T	synonymous
471022	NTHI477_RS02240	270	G	A	synonymous
471024	NTHI477_RS02240	268	A	G	nonsynonymous
471037	NTHI477_RS02240	255	A	G	synonymous
471045	NTHI477_RS02240	247	T	C	nonsynonymous
471045	NTHI477_RS02240	247	T	A	nonsynonymous
471046	NTHI477_RS02240	246	T	C	synonymous
471058	NTHI477_RS02240	234	T	C	synonymous
471073	NTHI477_RS02240	219	T	C	synonymous
471079	NTHI477_RS02240	213	A	G	synonymous
471090	NTHI477_RS02240	202	C	A	nonsynonymous
471094	NTHI477_RS02240	198	C	G	synonymous
471110	NTHI477_RS02240	182	G	A	nonsynonymous
471112	NTHI477_RS02240	180	A	G	synonymous
471124	NTHI477_RS02240	168	G	A	synonymous
471136	NTHI477_RS02240	156	C	T	synonymous

471142	NTHI477_RS02240	150	G	A	synonymous
471145	NTHI477_RS02240	147	A	G	synonymous
471147	NTHI477_RS02240	145	C	A	nonsynonymous
471153	NTHI477_RS02240	139	G	A	nonsynonymous
471178	NTHI477_RS02240	114	G	A	synonymous
471187	NTHI477_RS02240	105	G	A	synonymous
471193	NTHI477_RS02240	99	G	A	synonymous
471202	NTHI477_RS02240	90	G	A	synonymous
471214	NTHI477_RS02240	78	T	C	synonymous
471232	NTHI477_RS02240	60	A	G	synonymous
471253	NTHI477_RS02240	39	G	A	synonymous
471265	NTHI477_RS02240	27	T	C	synonymous
471271	NTHI477_RS02240	21	T	C	synonymous
471274	NTHI477_RS02240	18	A	G	synonymous
471283	NTHI477_RS02240	9	G	A	synonymous
471310	intergenic	intergenic	C	T	intergenic
471349	intergenic	intergenic	G	A	intergenic
471350	intergenic	intergenic	A	T	intergenic
471350	intergenic	intergenic	A	C	intergenic
471352	intergenic	intergenic	C	G	intergenic
471353	intergenic	intergenic	T	C	intergenic
471353	intergenic	intergenic	T	G	intergenic
471368	intergenic	intergenic	T	C	intergenic
471370	intergenic	intergenic	G	A	intergenic
471379	intergenic	intergenic	C	T	intergenic
471380	intergenic	intergenic	G	T	intergenic
471381	intergenic	intergenic	A	G	intergenic
471381	intergenic	intergenic	A	C	intergenic
471394	intergenic	intergenic	C	T	intergenic
471424	intergenic	intergenic	A	G	intergenic
471426	intergenic	intergenic	C	T	intergenic
471429	intergenic	intergenic	A	G	intergenic
471429	intergenic	intergenic	A	T	intergenic
471430	intergenic	intergenic	A	T	intergenic
471433	intergenic	intergenic	C	T	intergenic
471454	intergenic	intergenic	C	T	intergenic
471455	intergenic	intergenic	A	G	intergenic
471496	intergenic	intergenic	C	T	intergenic
471503	intergenic	intergenic	A	T	intergenic
471531	intergenic	intergenic	C	T	intergenic
471539	intergenic	intergenic	T	A	intergenic
471543	intergenic	intergenic	A	G	intergenic
471544	intergenic	intergenic	G	A	intergenic
471546	intergenic	intergenic	G	T	intergenic
471547	intergenic	intergenic	G	A	intergenic

471577	intergenic	intergenic	G	A	intergenic
471579	intergenic	intergenic	A	G	intergenic
471579	intergenic	intergenic	A	T	intergenic
471596	intergenic	intergenic	T	G	intergenic
471598	intergenic	intergenic	G	C	intergenic
471656	intergenic	intergenic	C	T	intergenic
471684	intergenic	intergenic	C	T	intergenic
471690	intergenic	intergenic	T	C	intergenic
471696	intergenic	intergenic	A	T	intergenic
471854	NTHI477_RS02245	119	C	T	nonsynonymous
471855	NTHI477_RS02245	120	G	A	synonymous
471917	NTHI477_RS02245	182	G	A	nonsynonymous
471963	NTHI477_RS02245	228	A	G	synonymous
471966	NTHI477_RS02245	231	A	G	synonymous
471997	NTHI477_RS02245	262	A	G	nonsynonymous
472038	NTHI477_RS02245	303	G	A	synonymous
472047	NTHI477_RS02245	312	A	G	synonymous
472060	NTHI477_RS02245	325	G	A	nonsynonymous
472062	NTHI477_RS02245	327	T	A	synonymous
472077	NTHI477_RS02245	342	C	T	synonymous
472081	NTHI477_RS02245	346	A	G	nonsynonymous
472083	NTHI477_RS02245	348	C	A	synonymous
472090	NTHI477_RS02245	355	G	A	nonsynonymous
472091	NTHI477_RS02245	356	C	T	nonsynonymous
472092	NTHI477_RS02245	357	A	C	synonymous
472098	NTHI477_RS02245	363	A	G	synonymous
472110	NTHI477_RS02245	375	T	C	synonymous
472158	NTHI477_RS02245	423	C	T	synonymous
472187	NTHI477_RS02245	452	T	C	nonsynonymous
472200	NTHI477_RS02245	465	A	C	synonymous
472213	NTHI477_RS02245	478	A	G	nonsynonymous
472233	NTHI477_RS02245	498	A	G	synonymous
472245	NTHI477_RS02245	510	G	A	synonymous
472260	NTHI477_RS02245	525	G	C	synonymous
472260	NTHI477_RS02245	525	G	A	synonymous
472269	NTHI477_RS02245	534	T	C	synonymous
472272	NTHI477_RS02245	537	C	T	synonymous
472276	NTHI477_RS02245	541	A	G	nonsynonymous
472278	NTHI477_RS02245	543	A	G	synonymous
472280	NTHI477_RS02245	545	C	T	nonsynonymous
472284	NTHI477_RS02245	549	T	A	synonymous
472311	NTHI477_RS02245	576	C	T	synonymous
472353	NTHI477_RS02245	618	C	T	synonymous
472410	intergenic	intergenic	T	C	intergenic
472411	intergenic	intergenic	A	C	intergenic

472414	intergenic	intergenic	A	T	intergenic
472418	intergenic	intergenic	G	A	intergenic
472448	intergenic	intergenic	C	T	intergenic
472473	intergenic	intergenic	C	T	intergenic
472476	intergenic	intergenic	C	T	intergenic
472477	intergenic	intergenic	C	T	intergenic
472479	intergenic	intergenic	C	T	intergenic
472482	intergenic	intergenic	A	G	intergenic
472483	intergenic	intergenic	A	C	intergenic
472484	intergenic	intergenic	A	G	intergenic
472538	intergenic	intergenic	G	A	intergenic
472546	intergenic	intergenic	A	G	intergenic
472574	intergenic	intergenic	C	T	intergenic
472575	intergenic	intergenic	T	C	intergenic
472579	intergenic	intergenic	G	A	intergenic
472580	intergenic	intergenic	A	G	intergenic
472589	intergenic	intergenic	C	T	intergenic
472595	intergenic	intergenic	A	G	intergenic
472596	intergenic	intergenic	G	A	intergenic
472638	intergenic	intergenic	A	C	intergenic
472639	intergenic	intergenic	C	A	intergenic
472640	intergenic	intergenic	G	A	intergenic
472641	intergenic	intergenic	T	C	intergenic
472642	intergenic	intergenic	T	C	intergenic
472649	intergenic	intergenic	A	G	intergenic
472650	intergenic	intergenic	A	G	intergenic
472651	intergenic	intergenic	C	A	intergenic
472671	intergenic	intergenic	G	A	intergenic
472803	intergenic	intergenic	T	C	intergenic
472863	intergenic	intergenic	G	A	intergenic
472864	intergenic	intergenic	C	T	intergenic
472905	intergenic	intergenic	C	T	intergenic
472906	intergenic	intergenic	A	T	intergenic
472913	intergenic	intergenic	G	A	intergenic
472914	intergenic	intergenic	A	C	intergenic
472920	intergenic	intergenic	G	T	intergenic
472921	intergenic	intergenic	C	T	intergenic
472923	intergenic	intergenic	T	A	intergenic
472925	intergenic	intergenic	A	G	intergenic
472926	intergenic	intergenic	T	G	intergenic
472936	intergenic	intergenic	A	C	intergenic
472946	intergenic	intergenic	G	A	intergenic
472973	intergenic	intergenic	T	G	intergenic
472978	intergenic	intergenic	C	T	intergenic
472981	intergenic	intergenic	C	T	intergenic

473006	intergenic	intergenic	A	T	intergenic
473016	intergenic	intergenic	A	C	intergenic
473020	intergenic	intergenic	G	A	intergenic
485716	intergenic	intergenic	C	T	intergenic
485722	intergenic	intergenic	C	T	intergenic
485725	intergenic	intergenic	T	C	intergenic
485756	intergenic	intergenic	T	C	intergenic
485757	intergenic	intergenic	A	C	intergenic
485772	intergenic	intergenic	C	T	intergenic
485773	intergenic	intergenic	G	A	intergenic
485774	intergenic	intergenic	C	T	intergenic
485781	intergenic	intergenic	T	C	intergenic
485783	intergenic	intergenic	T	G	intergenic
485800	intergenic	intergenic	C	T	intergenic
485804	intergenic	intergenic	C	T	intergenic
485809	intergenic	intergenic	T	C	intergenic
485810	intergenic	intergenic	T	C	intergenic
485826	intergenic	intergenic	G	T	intergenic
485833	intergenic	intergenic	G	A	intergenic
485840	intergenic	intergenic	C	T	intergenic
485857	intergenic	intergenic	A	G	intergenic
485861	intergenic	intergenic	C	G	intergenic
485881	intergenic	intergenic	T	A	intergenic
485902	intergenic	intergenic	T	G	intergenic
485911	intergenic	intergenic	T	A	intergenic
485912	intergenic	intergenic	T	A	intergenic
485919	intergenic	intergenic	A	C	intergenic
485928	intergenic	intergenic	C	A	intergenic
485930	intergenic	intergenic	G	T	intergenic
485944	intergenic	intergenic	G	A	intergenic
485951	intergenic	intergenic	T	G	intergenic
485959	intergenic	intergenic	T	A	intergenic
486005	intergenic	intergenic	A	G	intergenic
486029	intergenic	intergenic	T	A	intergenic
486114	NTHI477_RS02285	702	G	A	synonymous
486174	NTHI477_RS02285	642	C	G	synonymous
486174	NTHI477_RS02285	642	C	T	synonymous
486180	NTHI477_RS02285	636	A	G	synonymous
486201	NTHI477_RS02285	615	A	T	synonymous
486291	NTHI477_RS02285	525	C	A	nonsynonymous
486314	NTHI477_RS02285	502	T	C	nonsynonymous
486319	NTHI477_RS02285	497	A	G	nonsynonymous
486333	NTHI477_RS02285	483	A	G	synonymous
486372	NTHI477_RS02285	444	T	G	nonsynonymous
486396	NTHI477_RS02285	420	G	A	synonymous

486425	NTHI477_RS02285	391	C	T	nonsynonymous
486450	NTHI477_RS02285	366	A	G	synonymous
486468	NTHI477_RS02285	348	A	G	synonymous
486512	NTHI477_RS02285	304	C	T	nonsynonymous
486582	NTHI477_RS02285	234	C	G	synonymous
486597	NTHI477_RS02285	219	T	C	synonymous
486642	NTHI477_RS02285	174	C	A	nonsynonymous
486721	NTHI477_RS02285	95	T	C	nonsynonymous
486724	NTHI477_RS02285	92	G	T	nonsynonymous
486776	NTHI477_RS02285	40	G	A	synonymous
486797	NTHI477_RS02285	19	G	A	synonymous
486799	NTHI477_RS02285	17	A	G	nonsynonymous
486811	NTHI477_RS02285	5	T	A	nonsynonymous
486811	NTHI477_RS02290	918	T	A	synonymous
486904	NTHI477_RS02290	825	G	A	synonymous
486913	NTHI477_RS02290	816	T	C	synonymous
486928	NTHI477_RS02290	801	G	A	synonymous
486932	NTHI477_RS02290	797	A	C	nonsynonymous
486994	NTHI477_RS02290	735	A	T	synonymous
487034	NTHI477_RS02290	695	G	T	nonsynonymous
487036	NTHI477_RS02290	693	T	C	synonymous
487062	NTHI477_RS02290	667	C	T	nonsynonymous
487075	NTHI477_RS02290	654	C	T	synonymous
487087	NTHI477_RS02290	642	A	C	synonymous
487144	NTHI477_RS02290	585	C	A	synonymous
487161	NTHI477_RS02290	568	C	T	nonsynonymous
487233	NTHI477_RS02290	496	T	C	nonsynonymous
487246	NTHI477_RS02290	483	A	G	synonymous
487279	NTHI477_RS02290	450	A	G	synonymous
487285	NTHI477_RS02290	444	T	A	synonymous
487327	NTHI477_RS02290	402	C	T	synonymous
487363	NTHI477_RS02290	366	C	T	synonymous
487378	NTHI477_RS02290	351	A	T	synonymous
487462	NTHI477_RS02290	267	T	C	synonymous
487477	NTHI477_RS02290	252	C	A	nonsynonymous
487482	NTHI477_RS02290	247	G	A	synonymous
487489	NTHI477_RS02290	240	A	C	synonymous
487506	NTHI477_RS02290	223	T	C	nonsynonymous
487561	NTHI477_RS02290	168	T	C	synonymous
487597	NTHI477_RS02290	132	A	T	synonymous
487657	NTHI477_RS02290	72	G	A	synonymous
487662	NTHI477_RS02290	67	G	A	nonsynonymous
487707	NTHI477_RS02290	22	A	G	synonymous
487714	NTHI477_RS02290	15	A	G	synonymous
487765	intergenic	intergenic	C	G	intergenic

487770	intergenic	intergenic	A	G	intergenic
487833	NTHI477_RS02295	244	C	A	nonsynonymous
487837	NTHI477_RS02295	240	C	A	synonymous
487839	NTHI477_RS02295	238	G	C	nonsynonymous
487852	NTHI477_RS02295	225	A	G	synonymous
487858	NTHI477_RS02295	219	A	G	synonymous
487861	NTHI477_RS02295	216	C	T	synonymous
487864	NTHI477_RS02295	213	G	A	synonymous
487879	NTHI477_RS02295	198	A	T	synonymous
487894	NTHI477_RS02295	183	A	G	synonymous
487921	NTHI477_RS02295	156	C	T	synonymous
487933	NTHI477_RS02295	144	G	A	synonymous
487939	NTHI477_RS02295	138	C	T	synonymous
487960	NTHI477_RS02295	117	T	C	synonymous
487968	NTHI477_RS02295	109	G	A	nonsynonymous
487988	NTHI477_RS02295	89	T	C	nonsynonymous
488012	NTHI477_RS02295	65	A	G	nonsynonymous
488041	NTHI477_RS02295	36	A	G	synonymous
488043	NTHI477_RS02295	34	T	C	nonsynonymous
488059	NTHI477_RS02295	18	T	G	synonymous
488092	intergenic	intergenic	G	A	intergenic
488100	intergenic	intergenic	A	G	intergenic
488193	NTHI477_RS02300	363	T	C	synonymous
488308	NTHI477_RS02300	248	G	A	nonsynonymous
488337	NTHI477_RS02300	219	C	T	synonymous
488340	NTHI477_RS02300	216	T	C	synonymous
488355	NTHI477_RS02300	201	A	G	synonymous
488403	NTHI477_RS02300	153	A	G	synonymous
488406	NTHI477_RS02300	150	T	A	synonymous
488486	NTHI477_RS02300	70	C	A	nonsynonymous
488496	NTHI477_RS02300	60	C	T	synonymous
488502	NTHI477_RS02300	54	C	T	synonymous
488505	NTHI477_RS02300	51	A	G	synonymous
488549	NTHI477_RS02300	7	C	T	nonsynonymous
488633	intergenic	intergenic	T	C	intergenic
488657	intergenic	intergenic	A	G	intergenic
488662	intergenic	intergenic	G	C	intergenic
488682	intergenic	intergenic	T	G	intergenic
488728	intergenic	intergenic	A	G	intergenic
488760	intergenic	intergenic	G	A	intergenic
488825	NTHI477_RS02305	48	G	A	synonymous
488873	NTHI477_RS02305	96	G	A	synonymous
488891	NTHI477_RS02305	114	G	T	synonymous
488948	NTHI477_RS02305	171	C	T	synonymous
488954	NTHI477_RS02305	177	T	C	synonymous

488957	NTHI477_RS02305	180	G	A	synonymous
488975	NTHI477_RS02305	198	C	T	synonymous
488984	NTHI477_RS02305	207	G	A	synonymous
488993	NTHI477_RS02305	216	G	A	synonymous
489131	NTHI477_RS02305	354	G	A	synonymous
489137	NTHI477_RS02305	360	A	G	synonymous
489158	NTHI477_RS02305	381	T	A	synonymous
489170	NTHI477_RS02305	393	C	A	synonymous
489170	NTHI477_RS02305	393	C	T	synonymous
489182	NTHI477_RS02305	405	C	T	synonymous
489194	NTHI477_RS02305	417	C	A	synonymous
489233	NTHI477_RS02305	456	T	C	synonymous
489277	NTHI477_RS02305	500	T	C	nonsynonymous
489299	NTHI477_RS02305	522	A	T	nonsynonymous
489305	NTHI477_RS02305	528	G	A	synonymous
489341	NTHI477_RS02305	564	C	T	synonymous
489419	NTHI477_RS02305	642	A	T	synonymous
489446	NTHI477_RS02305	669	G	C	synonymous
489449	NTHI477_RS02305	672	G	A	synonymous
489463	intergenic	intergenic	C	T	intergenic
489478	intergenic	intergenic	T	A	intergenic
489532	NTHI477_RS02310	50	C	T	nonsynonymous
489566	NTHI477_RS02310	84	A	G	synonymous
489581	NTHI477_RS02310	99	C	T	synonymous
489590	NTHI477_RS02310	108	T	A	synonymous
489596	NTHI477_RS02310	114	C	A	synonymous
489611	NTHI477_RS02310	129	T	C	synonymous
489612	NTHI477_RS02310	130	G	A	nonsynonymous
489617	NTHI477_RS02310	135	A	C	synonymous
489619	NTHI477_RS02310	137	C	T	nonsynonymous
489629	NTHI477_RS02310	147	A	G	synonymous
489638	NTHI477_RS02310	156	T	C	synonymous
489641	NTHI477_RS02310	159	G	A	synonymous
489659	NTHI477_RS02310	177	A	T	synonymous
489665	NTHI477_RS02310	183	A	C	synonymous
489669	NTHI477_RS02310	187	T	C	synonymous
489674	NTHI477_RS02310	192	T	A	synonymous
489680	NTHI477_RS02310	198	C	T	synonymous
489698	NTHI477_RS02310	216	A	T	synonymous
489703	NTHI477_RS02310	221	G	A	nonsynonymous
489707	NTHI477_RS02310	225	C	T	synonymous
489716	NTHI477_RS02310	234	C	T	synonymous
489722	NTHI477_RS02310	240	G	A	nonsynonymous
489731	NTHI477_RS02310	249	T	C	synonymous
489737	NTHI477_RS02310	255	A	G	synonymous

489740	NTHI477_RS02310	258	C	A	synonymous
489752	NTHI477_RS02310	270	C	T	synonymous
489753	NTHI477_RS02310	271	T	C	synonymous
489782	NTHI477_RS02310	300	A	G	synonymous
489782	NTHI477_RS02310	300	A	T	synonymous
489785	NTHI477_RS02310	303	T	G	synonymous
489802	NTHI477_RS02310	320	C	T	nonsynonymous
489832	NTHI477_RS02310	350	T	C	nonsynonymous
489905	NTHI477_RS02310	423	T	G	nonsynonymous
489933	NTHI477_RS02310	451	G	A	nonsynonymous
489938	NTHI477_RS02310	456	G	A	synonymous
489947	NTHI477_RS02310	465	C	T	synonymous
489948	NTHI477_RS02310	466	T	G	nonsynonymous
489962	NTHI477_RS02310	480	T	C	synonymous
489983	NTHI477_RS02310	501	G	A	synonymous
490004	NTHI477_RS02310	522	T	C	synonymous
490010	NTHI477_RS02310	528	G	A	synonymous
490014	NTHI477_RS02310	532	T	C	synonymous
490026	NTHI477_RS02310	544	C	A	nonsynonymous
490059	NTHI477_RS02310	577	A	G	nonsynonymous
490079	NTHI477_RS02310	597	A	G	synonymous
490179	NTHI477_RS02310	697	C	T	nonsynonymous
490190	NTHI477_RS02310	708	C	T	synonymous
490196	NTHI477_RS02310	714	T	C	synonymous
490201	NTHI477_RS02310	719	C	T	nonsynonymous
490202	NTHI477_RS02310	720	A	C	synonymous
490205	NTHI477_RS02310	723	T	C	synonymous
490214	NTHI477_RS02310	732	T	A	synonymous
490219	NTHI477_RS02310	737	G	A	nonsynonymous
490220	NTHI477_RS02310	738	T	C	synonymous
490226	NTHI477_RS02310	744	C	T	synonymous
490229	NTHI477_RS02310	747	C	T	synonymous
490234	NTHI477_RS02310	752	T	G	nonsynonymous
490238	NTHI477_RS02310	756	A	C	synonymous
490259	NTHI477_RS02310	777	A	T	synonymous
490343	NTHI477_RS02310	861	G	T	synonymous
490409	NTHI477_RS02310	927	C	A	synonymous
490410	NTHI477_RS02310	928	T	C	synonymous
490430	NTHI477_RS02310	948	T	A	synonymous
490433	NTHI477_RS02310	951	G	A	synonymous
490475	NTHI477_RS02310	993	A	C	synonymous
490499	NTHI477_RS02310	1017	A	C	synonymous
490508	NTHI477_RS02310	1026	C	T	synonymous
490514	NTHI477_RS02310	1032	C	T	synonymous
490529	NTHI477_RS02310	1047	T	A	synonymous

490532	NTHI477_RS02310	1050	A	G	synonymous
490538	NTHI477_RS02310	1056	T	C	synonymous
490544	NTHI477_RS02310	1062	A	C	synonymous
490547	NTHI477_RS02310	1065	T	A	synonymous
490547	NTHI477_RS02310	1065	T	G	synonymous
490553	NTHI477_RS02310	1071	C	A	synonymous
490556	NTHI477_RS02310	1074	T	C	synonymous
490565	NTHI477_RS02310	1083	G	A	synonymous
490568	NTHI477_RS02310	1086	G	C	synonymous
490608	NTHI477_RS02310	1126	A	C	nonsynonymous
490613	NTHI477_RS02310	1131	C	T	synonymous
490622	NTHI477_RS02310	1140	C	T	synonymous
490631	NTHI477_RS02310	1149	T	A	synonymous
490634	NTHI477_RS02310	1152	A	G	synonymous
490652	NTHI477_RS02310	1170	C	T	synonymous
490658	NTHI477_RS02310	1176	C	T	synonymous
490676	NTHI477_RS02310	1194	T	C	synonymous
490694	NTHI477_RS02310	1212	T	C	synonymous
490709	NTHI477_RS02310	1227	T	C	synonymous
490723	NTHI477_RS02310	1241	T	C	nonsynonymous
490733	NTHI477_RS02310	1251	T	A	synonymous
490769	intergenic	intergenic	T	G	intergenic
490850	NTHI477_RS02315	40	T	C	synonymous
490855	NTHI477_RS02315	45	T	C	synonymous
490904	NTHI477_RS02315	94	T	C	synonymous
490906	NTHI477_RS02315	96	A	C	nonsynonymous
490912	NTHI477_RS02315	102	G	A	synonymous
490912	NTHI477_RS02315	102	G	T	synonymous
490921	NTHI477_RS02315	111	C	T	synonymous
490930	NTHI477_RS02315	120	T	A	synonymous
490933	NTHI477_RS02315	123	C	T	synonymous
490950	NTHI477_RS02315	140	C	T	nonsynonymous
490966	NTHI477_RS02315	156	A	G	synonymous
490973	NTHI477_RS02315	163	G	A	nonsynonymous
490975	NTHI477_RS02315	165	G	C	synonymous
490978	NTHI477_RS02315	168	G	A	synonymous
490990	NTHI477_RS02315	180	T	A	nonsynonymous
491062	NTHI477_RS02315	252	C	T	synonymous
491065	NTHI477_RS02315	255	T	C	synonymous
491110	NTHI477_RS02315	300	C	A	synonymous
491136	NTHI477_RS02315	326	G	A	nonsynonymous
491155	NTHI477_RS02315	345	G	A	synonymous
491158	NTHI477_RS02315	348	A	G	synonymous
491167	NTHI477_RS02315	357	C	A	synonymous
491221	NTHI477_RS02315	411	G	A	synonymous

491230	NTHI477_RS02315	420	T	A	synonymous
491234	NTHI477_RS02315	424	C	T	nonsynonymous
491236	NTHI477_RS02315	426	T	G	synonymous
491347	NTHI477_RS02315	537	A	G	synonymous
491374	NTHI477_RS02315	564	C	T	synonymous
491386	NTHI477_RS02315	576	G	A	synonymous
491413	NTHI477_RS02315	603	T	C	synonymous
491418	NTHI477_RS02315	608	C	A	nonsynonymous
491435	NTHI477_RS02320	7	A	G	nonsynonymous
491443	NTHI477_RS02320	15	A	G	synonymous
491449	NTHI477_RS02320	21	T	A	synonymous
491456	NTHI477_RS02320	28	A	G	nonsynonymous
491461	NTHI477_RS02320	33	T	C	synonymous
491513	NTHI477_RS02320	85	G	A	nonsynonymous
491517	NTHI477_RS02320	89	A	C	nonsynonymous
491520	NTHI477_RS02320	92	C	A	nonsynonymous
491547	NTHI477_RS02320	119	A	T	nonsynonymous
491569	NTHI477_RS02320	141	A	G	synonymous
491633	NTHI477_RS02320	205	A	G	nonsynonymous
491644	NTHI477_RS02320	216	G	T	synonymous
491761	NTHI477_RS02320	333	C	T	synonymous
491791	NTHI477_RS02320	363	G	A	synonymous
491830	NTHI477_RS02320	402	T	A	synonymous
491836	NTHI477_RS02320	408	G	A	synonymous
491854	NTHI477_RS02320	426	T	A	synonymous
491869	NTHI477_RS02320	441	C	T	synonymous
491878	NTHI477_RS02320	450	T	C	synonymous
491884	NTHI477_RS02320	456	C	T	synonymous
491887	NTHI477_RS02320	459	A	C	synonymous
491890	NTHI477_RS02320	462	A	G	synonymous
491897	NTHI477_RS02320	469	C	T	synonymous
491900	NTHI477_RS02320	472	T	G	nonsynonymous
491902	NTHI477_RS02320	474	C	T	synonymous
491909	NTHI477_RS02320	481	G	A	nonsynonymous
491922	NTHI477_RS02320	494	T	A	nonsynonymous
491948	NTHI477_RS02320	520	G	A	nonsynonymous
491957	NTHI477_RS02320	529	G	A	nonsynonymous
491986	NTHI477_RS02320	558	C	T	synonymous
492029	NTHI477_RS02320	601	G	A	nonsynonymous
492031	NTHI477_RS02320	603	T	C	synonymous
492050	NTHI477_RS02320	622	C	T	nonsynonymous
492058	NTHI477_RS02320	630	C	T	synonymous
492068	NTHI477_RS02320	640	T	C	nonsynonymous
492073	NTHI477_RS02320	645	C	G	synonymous
492076	NTHI477_RS02320	648	A	G	synonymous

492109	NTHI477_RS02320	681	T	C	synonymous
492143	NTHI477_RS02320	715	G	A	nonsynonymous
492145	NTHI477_RS02320	717	G	A	synonymous
492149	NTHI477_RS02320	721	A	C	nonsynonymous
492154	NTHI477_RS02320	726	G	A	synonymous
492168	NTHI477_RS02320	740	C	A	nonsynonymous
492171	NTHI477_RS02320	743	T	A	nonsynonymous
492172	NTHI477_RS02320	744	C	T	synonymous
492178	NTHI477_RS02320	750	T	C	synonymous
492190	NTHI477_RS02320	762	T	C	synonymous
492199	NTHI477_RS02320	771	C	G	synonymous
492202	NTHI477_RS02320	774	A	T	synonymous
492211	NTHI477_RS02320	783	T	C	synonymous
492313	NTHI477_RS02320	885	A	G	synonymous
492316	NTHI477_RS02320	888	C	T	synonymous
492364	NTHI477_RS02320	936	A	G	synonymous
492376	NTHI477_RS02320	948	A	G	synonymous
492385	NTHI477_RS02320	957	G	A	synonymous
492409	NTHI477_RS02320	981	G	A	synonymous
492440	NTHI477_RS02320	1012	T	C	nonsynonymous
492473	NTHI477_RS02320	1045	C	T	synonymous
492475	NTHI477_RS02320	1047	A	C	synonymous
492496	NTHI477_RS02320	1068	A	T	synonymous
492507	NTHI477_RS02320	1079	A	C	nonsynonymous
492541	NTHI477_RS02320	1113	A	G	synonymous
492547	NTHI477_RS02320	1119	G	A	synonymous
492550	NTHI477_RS02320	1122	T	C	synonymous
492551	NTHI477_RS02320	1123	G	T	nonsynonymous
492551	NTHI477_RS02320	1123	G	A	nonsynonymous
492553	NTHI477_RS02320	1125	G	T	synonymous
492571	NTHI477_RS02320	1143	A	G	synonymous
492574	NTHI477_RS02320	1146	G	A	synonymous
492586	NTHI477_RS02320	1158	T	C	synonymous
492625	NTHI477_RS02320	1197	G	T	synonymous
492659	NTHI477_RS02320	1231	A	G	nonsynonymous
492669	NTHI477_RS02320	1241	C	A	nonsynonymous
492672	NTHI477_RS02320	1244	C	A	nonsynonymous
492683	intergenic	intergenic	A	T	intergenic
492727	intergenic	intergenic	A	G	intergenic
492759	NTHI477_RS02325	18	G	C	synonymous
492769	NTHI477_RS02325	28	T	C	nonsynonymous
492790	NTHI477_RS02325	49	G	C	nonsynonymous
492796	NTHI477_RS02325	55	C	T	synonymous
492860	NTHI477_RS02325	119	T	C	nonsynonymous
492959	NTHI477_RS02325	218	A	G	nonsynonymous

492975	NTHI477_RS02325	234	C	T	synonymous
492984	NTHI477_RS02325	243	G	A	synonymous
492996	NTHI477_RS02325	255	G	A	synonymous
493053	NTHI477_RS02325	312	C	T	synonymous
493077	NTHI477_RS02325	336	C	T	synonymous
493080	NTHI477_RS02325	339	T	C	synonymous
493094	NTHI477_RS02325	353	C	T	nonsynonymous
493095	NTHI477_RS02325	354	A	T	synonymous
493104	NTHI477_RS02325	363	G	T	synonymous
493109	NTHI477_RS02325	368	A	T	nonsynonymous
493111	NTHI477_RS02325	370	T	C	synonymous
493124	NTHI477_RS02325	383	T	G	nonsynonymous
493125	NTHI477_RS02325	384	C	T	synonymous
493131	NTHI477_RS02325	390	A	C	synonymous
493137	NTHI477_RS02325	396	C	T	synonymous
493138	NTHI477_RS02325	397	A	G	nonsynonymous
493140	NTHI477_RS02325	399	T	A	synonymous
493140	NTHI477_RS02325	399	T	G	nonsynonymous
493143	NTHI477_RS02325	402	T	A	synonymous
493157	NTHI477_RS02325	416	T	C	nonsynonymous
493188	NTHI477_RS02325	447	T	C	synonymous
493191	NTHI477_RS02325	450	A	G	synonymous
493203	NTHI477_RS02325	462	T	G	synonymous
493206	NTHI477_RS02325	465	C	T	synonymous
493224	NTHI477_RS02325	483	C	T	synonymous
493236	NTHI477_RS02325	495	C	T	synonymous
493237	NTHI477_RS02325	496	C	T	nonsynonymous
493250	NTHI477_RS02325	509	C	T	nonsynonymous
493272	NTHI477_RS02325	531	A	C	synonymous
493275	NTHI477_RS02325	534	C	T	synonymous
493293	NTHI477_RS02325	552	G	A	synonymous
493314	NTHI477_RS02325	573	A	G	synonymous
493329	NTHI477_RS02325	588	A	G	synonymous
493338	NTHI477_RS02325	597	C	T	synonymous
493339	NTHI477_RS02325	598	T	C	synonymous
493355	NTHI477_RS02325	614	T	A	nonsynonymous
493428	NTHI477_RS02330	46	C	G	nonsynonymous
493439	NTHI477_RS02330	57	T	G	synonymous
493463	NTHI477_RS02330	81	A	T	synonymous
493466	NTHI477_RS02330	84	C	A	synonymous
493493	NTHI477_RS02330	111	C	T	synonymous
493510	NTHI477_RS02330	128	G	A	nonsynonymous
493632	NTHI477_RS02330	250	G	A	nonsynonymous
493648	NTHI477_RS02330	266	A	G	nonsynonymous
493652	NTHI477_RS02330	270	T	C	synonymous

493730	NTHI477_RS02330	348	C	G	synonymous
493733	NTHI477_RS02330	351	T	G	synonymous
493742	NTHI477_RS02330	360	C	T	synonymous
493769	NTHI477_RS02330	387	A	G	synonymous
493773	NTHI477_RS02330	391	T	G	nonsynonymous
493793	NTHI477_RS02330	411	A	G	synonymous
493817	NTHI477_RS02330	435	G	A	synonymous
493841	NTHI477_RS02330	459	C	T	synonymous
493863	NTHI477_RS02330	481	T	C	nonsynonymous
493865	NTHI477_RS02330	483	C	T	synonymous
493871	NTHI477_RS02330	489	G	C	nonsynonymous
493874	NTHI477_RS02330	492	T	A	synonymous
493892	NTHI477_RS02330	510	T	C	synonymous
493920	NTHI477_RS02330	538	G	T	nonsynonymous
493925	NTHI477_RS02330	543	G	A	synonymous
493973	NTHI477_RS02330	591	C	G	synonymous
493976	NTHI477_RS02330	594	C	A	synonymous
494099	NTHI477_RS02330	717	G	A	synonymous
494112	NTHI477_RS02330	730	C	A	nonsynonymous
494123	NTHI477_RS02330	741	G	A	synonymous
494186	NTHI477_RS02330	804	G	A	synonymous
494229	NTHI477_RS02330	847	G	A	nonsynonymous
494267	NTHI477_RS02330	885	G	A	nonsynonymous
494298	NTHI477_RS02330	916	C	A	synonymous
494334	NTHI477_RS02335	9	C	T	synonymous
494358	NTHI477_RS02335	33	T	A	synonymous
494562	NTHI477_RS02335	237	C	T	synonymous
494589	NTHI477_RS02335	264	G	A	synonymous
494595	NTHI477_RS02335	270	C	T	synonymous
494652	NTHI477_RS02335	327	T	C	synonymous
494664	NTHI477_RS02335	339	G	A	synonymous
494709	NTHI477_RS02335	384	T	C	synonymous
494754	NTHI477_RS02335	429	A	G	synonymous
494812	NTHI477_RS02335	487	G	A	nonsynonymous
494820	NTHI477_RS02335	495	A	T	synonymous
494820	NTHI477_RS02335	495	A	G	synonymous
494823	NTHI477_RS02335	498	T	C	synonymous
494847	NTHI477_RS02335	522	C	T	synonymous
494853	NTHI477_RS02335	528	G	A	synonymous
494862	NTHI477_RS02335	537	C	T	synonymous
494868	NTHI477_RS02335	543	C	A	synonymous
494868	NTHI477_RS02335	543	C	T	synonymous
494871	NTHI477_RS02335	546	T	G	synonymous
494883	NTHI477_RS02335	558	C	T	synonymous
494901	NTHI477_RS02335	576	T	C	synonymous

494916	NTHI477_RS02335	591	C	T	synonymous
494919	NTHI477_RS02335	594	T	A	synonymous
494928	NTHI477_RS02335	603	T	C	synonymous
494959	NTHI477_RS02335	634	A	G	nonsynonymous
494967	NTHI477_RS02335	642	C	T	synonymous
494982	NTHI477_RS02335	657	G	A	synonymous
494985	NTHI477_RS02335	660	T	A	synonymous
495054	NTHI477_RS02335	729	A	G	synonymous
495060	NTHI477_RS02335	735	A	T	synonymous
495102	NTHI477_RS02335	777	C	T	synonymous
495108	NTHI477_RS02335	783	C	T	synonymous
495121	NTHI477_RS02335	796	G	A	nonsynonymous
495138	NTHI477_RS02335	813	T	A	synonymous
495144	NTHI477_RS02335	819	C	T	synonymous
495147	NTHI477_RS02335	822	T	C	synonymous
495150	NTHI477_RS02335	825	T	G	synonymous
495177	NTHI477_RS02335	852	A	G	synonymous
495180	NTHI477_RS02335	855	C	T	synonymous
495189	NTHI477_RS02335	864	C	T	synonymous
495192	NTHI477_RS02335	867	T	C	synonymous
495195	NTHI477_RS02335	870	A	G	synonymous
495196	NTHI477_RS02335	871	T	C	synonymous
495198	NTHI477_RS02335	873	A	T	nonsynonymous
495201	NTHI477_RS02335	876	A	G	synonymous
495210	NTHI477_RS02335	885	T	C	synonymous
495214	NTHI477_RS02335	889	T	C	synonymous
495216	NTHI477_RS02335	891	A	G	synonymous
495225	NTHI477_RS02335	900	C	T	synonymous
495231	NTHI477_RS02335	906	C	T	synonymous
495234	NTHI477_RS02335	909	A	C	synonymous
495306	intergenic	intergenic	A	T	intergenic
495308	intergenic	intergenic	C	T	intergenic
495309	intergenic	intergenic	A	G	intergenic
495310	intergenic	intergenic	G	C	intergenic
495312	intergenic	intergenic	T	A	intergenic
495331	intergenic	intergenic	C	T	intergenic
495343	NTHI477_RS02340	1182	C	T	synonymous
495343	NTHI477_RS02340	1182	C	A	synonymous
495346	NTHI477_RS02340	1179	A	C	synonymous
495349	NTHI477_RS02340	1176	C	T	synonymous
495355	NTHI477_RS02340	1170	C	T	synonymous
495379	NTHI477_RS02340	1146	G	A	synonymous
495391	NTHI477_RS02340	1134	G	A	synonymous
495397	NTHI477_RS02340	1128	T	C	synonymous
495406	NTHI477_RS02340	1119	G	A	synonymous

495412	NTHI477_RS02340	1113	C	T	synonymous
495415	NTHI477_RS02340	1110	G	A	synonymous
495421	NTHI477_RS02340	1104	T	G	synonymous
495430	NTHI477_RS02340	1095	G	A	synonymous
495433	NTHI477_RS02340	1092	G	A	synonymous
495442	NTHI477_RS02340	1083	C	T	synonymous
495445	NTHI477_RS02340	1080	T	A	synonymous
495459	NTHI477_RS02340	1066	C	T	nonsynonymous
495463	NTHI477_RS02340	1062	A	G	synonymous
495469	NTHI477_RS02340	1056	T	C	synonymous
495475	NTHI477_RS02340	1050	G	A	synonymous
495493	NTHI477_RS02340	1032	T	A	synonymous
495493	NTHI477_RS02340	1032	T	G	synonymous
495496	NTHI477_RS02340	1029	C	T	synonymous
495496	NTHI477_RS02340	1029	C	A	synonymous
495511	NTHI477_RS02340	1014	A	G	synonymous
495514	NTHI477_RS02340	1011	A	G	synonymous
495523	NTHI477_RS02340	1002	A	C	nonsynonymous
495529	NTHI477_RS02340	996	G	T	synonymous
495529	NTHI477_RS02340	996	G	C	synonymous
495535	NTHI477_RS02340	990	G	A	synonymous
495568	NTHI477_RS02340	957	G	A	synonymous
495580	NTHI477_RS02340	945	G	A	synonymous
495583	NTHI477_RS02340	942	A	G	synonymous
495604	NTHI477_RS02340	921	T	A	synonymous
495606	NTHI477_RS02340	919	C	A	nonsynonymous
495607	NTHI477_RS02340	918	A	G	synonymous
495609	NTHI477_RS02340	916	T	C	nonsynonymous
495613	NTHI477_RS02340	912	T	C	synonymous
495616	NTHI477_RS02340	909	T	C	synonymous
495636	NTHI477_RS02340	889	A	C	nonsynonymous
495654	NTHI477_RS02340	871	G	A	synonymous
495664	NTHI477_RS02340	861	T	G	synonymous
495673	NTHI477_RS02340	852	A	T	synonymous
495682	NTHI477_RS02340	843	A	G	synonymous
495685	NTHI477_RS02340	840	G	C	nonsynonymous
495703	NTHI477_RS02340	822	T	C	synonymous
495718	NTHI477_RS02340	807	G	A	synonymous
495719	NTHI477_RS02340	806	C	T	nonsynonymous
495727	NTHI477_RS02340	798	G	A	synonymous
495730	NTHI477_RS02340	795	G	A	synonymous
495742	NTHI477_RS02340	783	A	G	synonymous
495753	NTHI477_RS02340	772	C	T	nonsynonymous
495760	NTHI477_RS02340	765	C	G	synonymous
495769	NTHI477_RS02340	756	T	A	synonymous

495775	NTHI477_RS02340	750	A	C	synonymous
495775	NTHI477_RS02340	750	A	T	synonymous
495820	NTHI477_RS02340	705	G	A	synonymous
495841	NTHI477_RS02340	684	T	A	synonymous
495841	NTHI477_RS02340	684	T	G	synonymous
495859	NTHI477_RS02340	666	A	G	synonymous
495865	NTHI477_RS02340	660	A	G	synonymous
495865	NTHI477_RS02340	660	A	T	synonymous
495874	NTHI477_RS02340	651	C	T	synonymous
495880	NTHI477_RS02340	645	A	G	synonymous
495919	NTHI477_RS02340	606	T	C	synonymous
495940	NTHI477_RS02340	585	C	T	synonymous
495964	NTHI477_RS02340	561	G	A	synonymous
495969	NTHI477_RS02340	556	A	G	synonymous
495973	NTHI477_RS02340	552	T	C	synonymous
495979	NTHI477_RS02340	546	T	C	synonymous
495982	NTHI477_RS02340	543	A	G	synonymous
495985	NTHI477_RS02340	540	C	T	synonymous
495994	NTHI477_RS02340	531	A	G	synonymous
496000	NTHI477_RS02340	525	A	G	synonymous
496009	NTHI477_RS02340	516	T	C	synonymous
496018	NTHI477_RS02340	507	G	A	synonymous
496042	NTHI477_RS02340	483	A	G	synonymous
496048	NTHI477_RS02340	477	G	A	synonymous
496051	NTHI477_RS02340	474	A	G	synonymous
496054	NTHI477_RS02340	471	G	T	synonymous
496075	NTHI477_RS02340	450	A	G	synonymous
496078	NTHI477_RS02340	447	A	G	synonymous
496093	NTHI477_RS02340	432	C	T	synonymous
496099	NTHI477_RS02340	426	G	C	synonymous
496102	NTHI477_RS02340	423	G	A	synonymous
496117	NTHI477_RS02340	408	A	G	synonymous
496168	NTHI477_RS02340	357	A	G	synonymous
496183	NTHI477_RS02340	342	T	A	synonymous
496222	NTHI477_RS02340	303	A	C	synonymous
496228	NTHI477_RS02340	297	A	G	synonymous
496245	NTHI477_RS02340	280	G	T	nonsynonymous
496249	NTHI477_RS02340	276	G	A	synonymous
496261	NTHI477_RS02340	264	C	A	synonymous
496264	NTHI477_RS02340	261	G	C	synonymous
496273	NTHI477_RS02340	252	A	G	synonymous
496276	NTHI477_RS02340	249	T	C	synonymous
496285	NTHI477_RS02340	240	G	A	synonymous
496288	NTHI477_RS02340	237	C	T	synonymous
496300	NTHI477_RS02340	225	A	G	synonymous

496306	NTHI477_RS02340	219	G	A	synonymous
496357	NTHI477_RS02340	168	G	A	synonymous
496501	NTHI477_RS02340	24	G	A	synonymous
496518	NTHI477_RS02340	7	C	T	nonsynonymous
496572	intergenic	intergenic	A	G	intergenic
496575	intergenic	intergenic	A	G	intergenic
496682	intergenic	intergenic	T	C	intergenic
496683	intergenic	intergenic	C	A	intergenic
496694	intergenic	intergenic	C	A	intergenic
496710	intergenic	intergenic	A	C	intergenic
496734	NTHI477_RS02345	705	A	G	synonymous
496740	NTHI477_RS02345	699	C	T	synonymous
496795	NTHI477_RS02345	644	G	A	nonsynonymous
496807	NTHI477_RS02345	632	G	C	nonsynonymous
496815	NTHI477_RS02345	624	A	G	synonymous
496860	NTHI477_RS02345	579	A	G	synonymous
496869	NTHI477_RS02345	570	C	T	synonymous
496872	NTHI477_RS02345	567	A	G	synonymous
496881	NTHI477_RS02345	558	A	G	synonymous
496890	NTHI477_RS02345	549	C	A	synonymous
496917	NTHI477_RS02345	522	T	C	synonymous
496918	NTHI477_RS02345	521	A	G	nonsynonymous
496925	NTHI477_RS02345	514	G	T	synonymous
496926	NTHI477_RS02345	513	G	A	synonymous
496929	NTHI477_RS02345	510	A	G	synonymous
496947	NTHI477_RS02345	492	C	G	nonsynonymous
496949	NTHI477_RS02345	490	A	G	synonymous
496956	NTHI477_RS02345	483	T	A	synonymous
496995	NTHI477_RS02345	444	A	G	synonymous
497001	NTHI477_RS02345	438	A	G	synonymous
497010	NTHI477_RS02345	429	G	A	synonymous
497022	NTHI477_RS02345	417	C	T	synonymous
497053	NTHI477_RS02345	386	A	G	nonsynonymous
497058	NTHI477_RS02345	381	G	A	synonymous
497061	NTHI477_RS02345	378	T	C	synonymous
497085	NTHI477_RS02345	354	T	G	synonymous
497097	NTHI477_RS02345	342	C	T	synonymous
497118	NTHI477_RS02345	321	G	A	synonymous
497137	NTHI477_RS02345	302	A	G	nonsynonymous
497139	NTHI477_RS02345	300	C	T	synonymous
497154	NTHI477_RS02345	285	T	C	synonymous
497175	NTHI477_RS02345	264	G	A	synonymous
497208	NTHI477_RS02345	231	G	A	synonymous
497214	NTHI477_RS02345	225	C	T	synonymous
497217	NTHI477_RS02345	222	T	C	synonymous

497224	NTHI477_RS02345	215	G	T	nonsynonymous
497226	NTHI477_RS02345	213	G	A	synonymous
497232	NTHI477_RS02345	207	A	T	synonymous
497235	NTHI477_RS02345	204	G	A	synonymous
497238	NTHI477_RS02345	201	T	C	synonymous
497259	NTHI477_RS02345	180	T	G	synonymous
497259	NTHI477_RS02345	180	T	A	synonymous
497262	NTHI477_RS02345	177	A	G	synonymous
497268	NTHI477_RS02345	171	A	G	synonymous
497274	NTHI477_RS02345	165	G	A	synonymous
497292	NTHI477_RS02345	147	T	G	synonymous
497295	NTHI477_RS02345	144	G	A	synonymous
497298	NTHI477_RS02345	141	A	G	synonymous
497307	NTHI477_RS02345	132	A	T	synonymous
497310	NTHI477_RS02345	129	G	A	synonymous
497313	NTHI477_RS02345	126	T	G	synonymous
497316	NTHI477_RS02345	123	A	T	synonymous
497325	NTHI477_RS02345	114	A	C	synonymous
497328	NTHI477_RS02345	111	T	A	synonymous
497334	NTHI477_RS02345	105	G	T	synonymous
497337	NTHI477_RS02345	102	A	G	synonymous
497352	NTHI477_RS02345	87	A	C	synonymous
497367	NTHI477_RS02345	72	C	T	synonymous
497369	NTHI477_RS02345	70	A	G	synonymous
497442	intergenic	intergenic	G	A	intergenic
497453	NTHI477_RS02350	1389	A	G	synonymous
497477	NTHI477_RS02350	1365	A	G	synonymous
497483	NTHI477_RS02350	1359	G	A	synonymous
497487	NTHI477_RS02350	1355	T	C	nonsynonymous
497492	NTHI477_RS02350	1350	A	G	synonymous
497495	NTHI477_RS02350	1347	T	C	synonymous
497528	NTHI477_RS02350	1314	T	C	synonymous
497543	NTHI477_RS02350	1299	C	T	synonymous
497552	NTHI477_RS02350	1290	A	G	synonymous
497555	NTHI477_RS02350	1287	G	A	synonymous
497573	NTHI477_RS02350	1269	C	T	synonymous
497582	NTHI477_RS02350	1260	G	A	synonymous
497588	NTHI477_RS02350	1254	C	A	synonymous
497590	NTHI477_RS02350	1252	C	T	nonsynonymous
497601	NTHI477_RS02350	1241	G	A	nonsynonymous
497609	NTHI477_RS02350	1233	T	G	synonymous
497612	NTHI477_RS02350	1230	C	T	synonymous
497615	NTHI477_RS02350	1227	A	G	synonymous
497621	NTHI477_RS02350	1221	A	C	synonymous
497629	NTHI477_RS02350	1213	C	T	nonsynonymous

497648	NTHI477_RS02350	1194	G	A	synonymous
497654	NTHI477_RS02350	1188	A	G	synonymous
497660	NTHI477_RS02350	1182	A	G	synonymous
497669	NTHI477_RS02350	1173	C	G	nonsynonymous
497671	NTHI477_RS02350	1171	T	C	nonsynonymous
497675	NTHI477_RS02350	1167	G	A	synonymous
497687	NTHI477_RS02350	1155	A	G	synonymous
497690	NTHI477_RS02350	1152	A	C	synonymous
497699	NTHI477_RS02350	1143	G	A	synonymous
497702	NTHI477_RS02350	1140	A	G	synonymous
497708	NTHI477_RS02350	1134	C	T	synonymous
497758	NTHI477_RS02350	1084	G	A	synonymous
497765	NTHI477_RS02350	1077	T	C	synonymous
497771	NTHI477_RS02350	1071	G	T	synonymous
497777	NTHI477_RS02350	1065	G	C	synonymous
497789	NTHI477_RS02350	1053	T	G	synonymous
497792	NTHI477_RS02350	1050	T	C	synonymous
497798	NTHI477_RS02350	1044	A	G	synonymous
497801	NTHI477_RS02350	1041	A	C	synonymous
497804	NTHI477_RS02350	1038	G	T	synonymous
497822	NTHI477_RS02350	1020	G	A	synonymous
497832	NTHI477_RS02350	1010	A	G	nonsynonymous
497845	NTHI477_RS02350	997	T	C	nonsynonymous
497849	NTHI477_RS02350	993	T	C	synonymous
497855	NTHI477_RS02350	987	A	C	synonymous
497876	NTHI477_RS02350	966	G	A	synonymous
497884	NTHI477_RS02350	958	C	T	nonsynonymous
497885	NTHI477_RS02350	957	A	C	nonsynonymous
497891	NTHI477_RS02350	951	C	T	synonymous
497906	NTHI477_RS02350	936	A	T	synonymous
497914	NTHI477_RS02350	928	C	T	nonsynonymous
497921	NTHI477_RS02350	921	T	C	synonymous
497927	NTHI477_RS02350	915	T	C	synonymous
497935	NTHI477_RS02350	907	A	G	synonymous
497944	NTHI477_RS02350	898	T	C	nonsynonymous
497987	NTHI477_RS02350	855	G	A	synonymous
497995	NTHI477_RS02350	847	A	G	synonymous
497996	NTHI477_RS02350	846	T	C	synonymous
497999	NTHI477_RS02350	843	G	A	synonymous
498014	NTHI477_RS02350	828	G	C	synonymous
498020	NTHI477_RS02350	822	A	G	synonymous
498047	NTHI477_RS02350	795	G	A	synonymous
498047	NTHI477_RS02350	795	G	C	synonymous
498050	NTHI477_RS02350	792	C	A	synonymous
498060	NTHI477_RS02350	782	A	T	nonsynonymous

498074	NTHI477_RS02350	768	G	A	synonymous
498083	NTHI477_RS02350	759	T	C	synonymous
498131	NTHI477_RS02350	711	A	C	synonymous
498132	NTHI477_RS02350	710	G	A	nonsynonymous
498137	NTHI477_RS02350	705	C	T	synonymous
498146	NTHI477_RS02350	696	A	G	synonymous
498155	NTHI477_RS02350	687	A	G	synonymous
498184	NTHI477_RS02350	658	C	T	nonsynonymous
498191	NTHI477_RS02350	651	G	A	synonymous
498200	NTHI477_RS02350	642	A	G	synonymous
498215	NTHI477_RS02350	627	A	G	synonymous
498242	NTHI477_RS02350	600	C	T	synonymous
498263	NTHI477_RS02350	579	A	C	synonymous
498275	NTHI477_RS02350	567	A	T	synonymous
498275	NTHI477_RS02350	567	A	C	nonsynonymous
498278	NTHI477_RS02350	564	A	G	synonymous
498311	NTHI477_RS02350	531	C	T	synonymous
498323	NTHI477_RS02350	519	C	T	synonymous
498325	NTHI477_RS02350	517	A	G	synonymous
498326	NTHI477_RS02350	516	T	C	synonymous
498353	NTHI477_RS02350	489	A	T	synonymous
498359	NTHI477_RS02350	483	G	A	synonymous
498365	NTHI477_RS02350	477	G	A	synonymous
498365	NTHI477_RS02350	477	G	C	synonymous
498366	NTHI477_RS02350	476	G	T	nonsynonymous
498371	NTHI477_RS02350	471	A	C	synonymous
498386	NTHI477_RS02350	456	G	A	synonymous
498410	NTHI477_RS02350	432	G	A	synonymous
498426	NTHI477_RS02350	416	A	G	nonsynonymous
498431	NTHI477_RS02350	411	C	T	synonymous
498434	NTHI477_RS02350	408	A	G	synonymous
498436	NTHI477_RS02350	406	G	A	nonsynonymous
498446	NTHI477_RS02350	396	C	G	synonymous
498449	NTHI477_RS02350	393	T	A	synonymous
498515	NTHI477_RS02350	327	C	T	synonymous
498518	NTHI477_RS02350	324	A	C	synonymous
498568	NTHI477_RS02350	274	C	G	nonsynonymous
498572	NTHI477_RS02350	270	G	T	synonymous
498585	NTHI477_RS02350	257	C	T	nonsynonymous
498614	NTHI477_RS02350	228	T	C	synonymous
498644	NTHI477_RS02350	198	C	T	synonymous
498652	NTHI477_RS02350	190	G	A	nonsynonymous
498659	NTHI477_RS02350	183	T	C	synonymous
498713	NTHI477_RS02350	129	A	T	synonymous
498716	NTHI477_RS02350	126	A	C	synonymous

498758	NTHI477_RS02350	84	G	A	synonymous
498764	NTHI477_RS02350	78	T	C	synonymous
498778	NTHI477_RS02350	64	G	A	nonsynonymous
498797	NTHI477_RS02350	45	A	C	nonsynonymous
498811	NTHI477_RS02350	31	C	T	nonsynonymous
498815	NTHI477_RS02350	27	A	G	synonymous
498821	NTHI477_RS02350	21	G	A	synonymous
498902	NTHI477_RS02355	18	A	C	synonymous
498917	NTHI477_RS02355	33	C	T	synonymous
498937	NTHI477_RS02355	53	A	C	nonsynonymous
498959	NTHI477_RS02355	75	G	A	synonymous
498970	NTHI477_RS02355	86	T	C	nonsynonymous
499008	NTHI477_RS02355	124	A	G	nonsynonymous
499022	NTHI477_RS02355	138	T	C	synonymous
499028	NTHI477_RS02355	144	T	C	synonymous
499046	NTHI477_RS02355	162	C	T	synonymous
499049	NTHI477_RS02355	165	T	C	synonymous
499103	NTHI477_RS02355	219	C	T	synonymous
499103	NTHI477_RS02355	219	C	A	synonymous
499109	NTHI477_RS02355	225	A	G	synonymous
499110	NTHI477_RS02355	226	G	A	nonsynonymous
499176	NTHI477_RS02355	292	T	C	synonymous
499215	NTHI477_RS02355	331	C	G	nonsynonymous
499242	NTHI477_RS02355	358	C	A	nonsynonymous
499253	NTHI477_RS02355	369	T	C	synonymous
499256	NTHI477_RS02355	372	A	G	synonymous
499259	NTHI477_RS02355	375	G	A	synonymous
499259	NTHI477_RS02355	375	G	C	synonymous
499264	NTHI477_RS02355	380	G	A	nonsynonymous
499265	NTHI477_RS02355	381	C	T	synonymous
499266	NTHI477_RS02355	382	G	A	nonsynonymous
499268	NTHI477_RS02355	384	A	G	synonymous
499274	NTHI477_RS02355	390	A	G	synonymous
499283	NTHI477_RS02355	399	C	T	synonymous
499307	NTHI477_RS02355	423	G	T	synonymous
499316	NTHI477_RS02355	432	C	T	synonymous
499322	NTHI477_RS02355	438	T	C	synonymous
499334	NTHI477_RS02355	450	G	T	synonymous
499334	NTHI477_RS02355	450	G	C	synonymous
499342	NTHI477_RS02355	458	A	G	nonsynonymous
499344	NTHI477_RS02355	460	G	A	nonsynonymous
499345	NTHI477_RS02355	461	G	A	nonsynonymous
499349	NTHI477_RS02355	465	G	A	synonymous
499349	NTHI477_RS02355	465	G	T	synonymous
499361	NTHI477_RS02355	477	G	A	synonymous

499370	NTHI477_RS02355	486	C	T	synonymous
499379	NTHI477_RS02355	495	T	C	synonymous
499386	NTHI477_RS02355	502	C	A	synonymous
499391	NTHI477_RS02355	507	C	G	synonymous
499392	NTHI477_RS02355	508	C	T	synonymous
499403	NTHI477_RS02355	519	G	A	synonymous
499412	NTHI477_RS02355	528	A	G	synonymous
499442	NTHI477_RS02355	558	T	G	synonymous
499448	NTHI477_RS02355	564	G	A	synonymous
499454	NTHI477_RS02355	570	T	C	synonymous
499457	NTHI477_RS02355	573	T	A	synonymous
499466	NTHI477_RS02355	582	T	G	synonymous
499469	NTHI477_RS02355	585	A	G	synonymous
499502	intergenic	intergenic	C	T	intergenic
499516	intergenic	intergenic	A	C	intergenic
499517	intergenic	intergenic	A	G	intergenic
499519	intergenic	intergenic	C	T	intergenic
499520	intergenic	intergenic	A	C	intergenic
499541	intergenic	intergenic	G	T	intergenic
499548	intergenic	intergenic	G	A	intergenic
499565	intergenic	intergenic	T	C	intergenic
499592	NTHI477_RS02360	2589	T	C	synonymous
499613	NTHI477_RS02360	2568	A	C	synonymous
499625	NTHI477_RS02360	2556	A	G	synonymous
499627	NTHI477_RS02360	2554	T	C	nonsynonymous
499628	NTHI477_RS02360	2553	G	A	synonymous
499646	NTHI477_RS02360	2535	G	A	synonymous
499649	NTHI477_RS02360	2532	C	T	synonymous
499655	NTHI477_RS02360	2526	T	C	synonymous
499657	NTHI477_RS02360	2524	G	A	synonymous
499658	NTHI477_RS02360	2523	T	C	synonymous
499658	NTHI477_RS02360	2523	T	G	synonymous
499670	NTHI477_RS02360	2511	A	G	synonymous
499672	NTHI477_RS02360	2509	C	T	nonsynonymous
499688	NTHI477_RS02360	2493	A	T	synonymous
499703	NTHI477_RS02360	2478	A	G	synonymous
499709	NTHI477_RS02360	2472	A	G	synonymous
499712	NTHI477_RS02360	2469	C	T	synonymous
499715	NTHI477_RS02360	2466	A	T	synonymous
499715	NTHI477_RS02360	2466	A	C	synonymous
499718	NTHI477_RS02360	2463	C	T	synonymous
499724	NTHI477_RS02360	2457	T	C	synonymous
499727	NTHI477_RS02360	2454	A	G	synonymous
499751	NTHI477_RS02360	2430	C	A	synonymous
499769	NTHI477_RS02360	2412	A	G	synonymous

499775	NTHI477_RS02360	2406	A	T	synonymous
499778	NTHI477_RS02360	2403	A	T	synonymous
499781	NTHI477_RS02360	2400	G	A	synonymous
499787	NTHI477_RS02360	2394	G	A	synonymous
499790	NTHI477_RS02360	2391	G	A	synonymous
499793	NTHI477_RS02360	2388	A	G	synonymous
499805	NTHI477_RS02360	2376	A	G	synonymous
499820	NTHI477_RS02360	2361	A	G	synonymous
499826	NTHI477_RS02360	2355	C	T	synonymous
499834	NTHI477_RS02360	2347	A	C	nonsynonymous
499859	NTHI477_RS02360	2322	A	G	synonymous
499874	NTHI477_RS02360	2307	A	G	synonymous
499901	NTHI477_RS02360	2280	C	T	synonymous
499949	NTHI477_RS02360	2232	C	G	synonymous
499961	NTHI477_RS02360	2220	C	G	nonsynonymous
499987	NTHI477_RS02360	2194	C	T	nonsynonymous
499991	NTHI477_RS02360	2190	T	C	synonymous
500003	NTHI477_RS02360	2178	G	A	synonymous
500012	NTHI477_RS02360	2169	T	C	synonymous
500015	NTHI477_RS02360	2166	A	C	synonymous
500039	NTHI477_RS02360	2142	T	G	synonymous
500039	NTHI477_RS02360	2142	T	C	synonymous
500039	NTHI477_RS02360	2142	T	A	synonymous
500042	NTHI477_RS02360	2139	C	T	synonymous
500072	NTHI477_RS02360	2109	A	G	synonymous
500084	NTHI477_RS02360	2097	C	T	synonymous
500084	NTHI477_RS02360	2097	C	G	synonymous
500089	NTHI477_RS02360	2092	A	G	synonymous
500093	NTHI477_RS02360	2088	G	A	synonymous
500096	NTHI477_RS02360	2085	A	G	synonymous
500105	NTHI477_RS02360	2076	C	A	synonymous
500105	NTHI477_RS02360	2076	C	G	synonymous
500108	NTHI477_RS02360	2073	A	G	synonymous
500135	NTHI477_RS02360	2046	A	T	synonymous
500153	NTHI477_RS02360	2028	G	A	synonymous
500156	NTHI477_RS02360	2025	G	A	synonymous
500177	NTHI477_RS02360	2004	A	C	nonsynonymous
500185	NTHI477_RS02360	1996	G	A	nonsynonymous
500186	NTHI477_RS02360	1995	A	G	synonymous
500219	NTHI477_RS02360	1962	T	G	nonsynonymous
500225	NTHI477_RS02360	1956	C	A	synonymous
500240	NTHI477_RS02360	1941	C	T	synonymous
500240	NTHI477_RS02360	1941	C	A	synonymous
500242	NTHI477_RS02360	1939	C	T	nonsynonymous
500253	NTHI477_RS02360	1928	G	T	nonsynonymous

500261	NTHI477_RS02360	1920	G	A	synonymous
500262	NTHI477_RS02360	1919	A	G	nonsynonymous
500285	NTHI477_RS02360	1896	A	G	synonymous
500297	NTHI477_RS02360	1884	T	C	synonymous
500300	NTHI477_RS02360	1881	C	G	synonymous
500324	NTHI477_RS02360	1857	T	C	synonymous
500336	NTHI477_RS02360	1845	T	G	synonymous
500344	NTHI477_RS02360	1837	G	A	synonymous
500360	NTHI477_RS02360	1821	G	A	synonymous
500365	NTHI477_RS02360	1816	T	G	nonsynonymous
500366	NTHI477_RS02360	1815	A	G	synonymous
500371	NTHI477_RS02360	1810	G	A	synonymous
500416	NTHI477_RS02360	1765	C	G	nonsynonymous
500416	NTHI477_RS02360	1765	C	A	nonsynonymous
500420	NTHI477_RS02360	1761	G	T	synonymous
500450	NTHI477_RS02360	1731	T	C	synonymous
500459	NTHI477_RS02360	1722	C	A	synonymous
500467	NTHI477_RS02360	1714	G	A	synonymous
500471	NTHI477_RS02360	1710	T	C	synonymous
500474	NTHI477_RS02360	1707	T	A	synonymous
500477	NTHI477_RS02360	1704	T	C	synonymous
500495	NTHI477_RS02360	1686	T	C	synonymous
500501	NTHI477_RS02360	1680	G	A	synonymous
500502	NTHI477_RS02360	1679	T	C	nonsynonymous
500513	NTHI477_RS02360	1668	C	A	synonymous
500531	NTHI477_RS02360	1650	T	C	synonymous
500540	NTHI477_RS02360	1641	A	G	synonymous
500552	NTHI477_RS02360	1629	C	A	synonymous
500564	NTHI477_RS02360	1617	C	T	synonymous
500567	NTHI477_RS02360	1614	A	G	synonymous
500570	NTHI477_RS02360	1611	A	G	synonymous
500581	NTHI477_RS02360	1600	A	G	synonymous
500582	NTHI477_RS02360	1599	T	C	synonymous
500588	NTHI477_RS02360	1593	T	G	synonymous
500593	NTHI477_RS02360	1588	T	C	nonsynonymous
500597	NTHI477_RS02360	1584	G	A	synonymous
500603	NTHI477_RS02360	1578	A	G	synonymous
500609	NTHI477_RS02360	1572	G	A	synonymous
500612	NTHI477_RS02360	1569	G	A	synonymous
500614	NTHI477_RS02360	1567	G	T	nonsynonymous
500618	NTHI477_RS02360	1563	T	C	synonymous
500621	NTHI477_RS02360	1560	A	G	synonymous
500642	NTHI477_RS02360	1539	G	A	synonymous
500648	NTHI477_RS02360	1533	T	C	synonymous
500662	NTHI477_RS02360	1519	A	G	synonymous

500667	NTHI477_RS02360	1514	T	C	nonsynonymous
500669	NTHI477_RS02360	1512	G	A	synonymous
500683	NTHI477_RS02360	1498	A	G	synonymous
500696	NTHI477_RS02360	1485	G	A	synonymous
500699	NTHI477_RS02360	1482	A	G	synonymous
500703	NTHI477_RS02360	1478	G	A	nonsynonymous
500704	NTHI477_RS02360	1477	C	A	nonsynonymous
500714	NTHI477_RS02360	1467	C	T	synonymous
500716	NTHI477_RS02360	1465	C	A	nonsynonymous
500717	NTHI477_RS02360	1464	G	C	nonsynonymous
500719	NTHI477_RS02360	1462	T	C	nonsynonymous
500759	NTHI477_RS02360	1422	T	A	synonymous
500786	NTHI477_RS02360	1395	C	T	synonymous
500813	NTHI477_RS02360	1368	G	C	nonsynonymous
500819	NTHI477_RS02360	1362	A	C	synonymous
500825	NTHI477_RS02360	1356	G	A	synonymous
500834	NTHI477_RS02360	1347	T	C	synonymous
500846	NTHI477_RS02360	1335	A	T	synonymous
500873	NTHI477_RS02360	1308	C	T	synonymous
500876	NTHI477_RS02360	1305	G	A	synonymous
500876	NTHI477_RS02360	1305	G	T	nonsynonymous
500897	NTHI477_RS02360	1284	A	T	synonymous
500897	NTHI477_RS02360	1284	A	C	synonymous
500915	NTHI477_RS02360	1266	A	G	synonymous
500918	NTHI477_RS02360	1263	A	G	synonymous
500939	NTHI477_RS02360	1242	G	A	synonymous
500945	NTHI477_RS02360	1236	A	G	synonymous
500949	NTHI477_RS02360	1232	T	C	nonsynonymous
500963	NTHI477_RS02360	1218	C	T	synonymous
500981	NTHI477_RS02360	1200	G	A	synonymous
500983	NTHI477_RS02360	1198	C	T	nonsynonymous
500987	NTHI477_RS02360	1194	T	C	synonymous
500990	NTHI477_RS02360	1191	A	C	synonymous
500999	NTHI477_RS02360	1182	G	A	synonymous
501002	NTHI477_RS02360	1179	A	G	synonymous
501017	NTHI477_RS02360	1164	C	G	synonymous
501023	NTHI477_RS02360	1158	T	C	synonymous
501041	NTHI477_RS02360	1140	G	T	synonymous
501044	NTHI477_RS02360	1137	T	C	synonymous
501047	NTHI477_RS02360	1134	G	C	synonymous
501050	NTHI477_RS02360	1131	A	G	synonymous
501056	NTHI477_RS02360	1125	A	G	synonymous
501086	NTHI477_RS02360	1095	A	G	synonymous
501095	NTHI477_RS02360	1086	T	C	synonymous
501122	NTHI477_RS02360	1059	T	C	synonymous

501128	NTHI477_RS02360	1053	A	G	synonymous
501128	NTHI477_RS02360	1053	A	C	synonymous
501131	NTHI477_RS02360	1050	G	A	synonymous
501161	NTHI477_RS02360	1020	C	A	synonymous
501161	NTHI477_RS02360	1020	C	T	synonymous
501164	NTHI477_RS02360	1017	T	G	synonymous
501164	NTHI477_RS02360	1017	T	C	synonymous
501170	NTHI477_RS02360	1011	C	A	synonymous
501176	NTHI477_RS02360	1005	T	C	synonymous
501182	NTHI477_RS02360	999	T	A	synonymous
501191	NTHI477_RS02360	990	T	C	synonymous
501200	NTHI477_RS02360	981	A	G	synonymous
501209	NTHI477_RS02360	972	C	T	synonymous
501215	NTHI477_RS02360	966	G	A	synonymous
501218	NTHI477_RS02360	963	T	C	synonymous
501224	NTHI477_RS02360	957	C	T	synonymous
501245	NTHI477_RS02360	936	A	T	synonymous
501251	NTHI477_RS02360	930	A	G	synonymous
501260	NTHI477_RS02360	921	A	G	synonymous
501263	NTHI477_RS02360	918	G	A	synonymous
501308	NTHI477_RS02360	873	A	G	synonymous
501320	NTHI477_RS02360	861	C	T	synonymous
501326	NTHI477_RS02360	855	T	G	synonymous
501344	NTHI477_RS02360	837	G	T	synonymous
501347	NTHI477_RS02360	834	T	C	synonymous
501350	NTHI477_RS02360	831	T	C	synonymous
501362	NTHI477_RS02360	819	A	G	synonymous
501368	NTHI477_RS02360	813	A	G	synonymous
501374	NTHI477_RS02360	807	C	T	synonymous
501377	NTHI477_RS02360	804	C	T	synonymous
501383	NTHI477_RS02360	798	G	A	synonymous
501395	NTHI477_RS02360	786	A	G	synonymous
501404	NTHI477_RS02360	777	G	A	synonymous
501407	NTHI477_RS02360	774	A	G	synonymous
501416	NTHI477_RS02360	765	A	G	synonymous
501419	NTHI477_RS02360	762	G	A	synonymous
501428	NTHI477_RS02360	753	A	G	synonymous
501481	NTHI477_RS02360	700	C	A	nonsynonymous
501490	NTHI477_RS02360	691	A	G	synonymous
501524	NTHI477_RS02360	657	A	G	synonymous
501527	NTHI477_RS02360	654	G	A	synonymous
501527	NTHI477_RS02360	654	G	T	synonymous
501536	NTHI477_RS02360	645	A	G	synonymous
501542	NTHI477_RS02360	639	C	T	synonymous
501548	NTHI477_RS02360	633	C	T	synonymous

501548	NTHI477_RS02360	633	C	A	synonymous
501548	NTHI477_RS02360	633	C	G	synonymous
501549	NTHI477_RS02360	632	G	T	nonsynonymous
501549	NTHI477_RS02360	632	G	A	nonsynonymous
501557	NTHI477_RS02360	624	A	T	synonymous
501559	NTHI477_RS02360	622	G	A	nonsynonymous
501560	NTHI477_RS02360	621	G	C	synonymous
501560	NTHI477_RS02360	621	G	T	synonymous
501569	NTHI477_RS02360	612	A	G	synonymous
501572	NTHI477_RS02360	609	A	T	synonymous
501574	NTHI477_RS02360	607	T	C	nonsynonymous
501575	NTHI477_RS02360	606	A	G	synonymous
501602	NTHI477_RS02360	579	C	T	synonymous
501605	NTHI477_RS02360	576	T	C	synonymous
501641	NTHI477_RS02360	540	T	A	synonymous
501644	NTHI477_RS02360	537	G	A	synonymous
501664	NTHI477_RS02360	517	G	A	nonsynonymous
501665	NTHI477_RS02360	516	A	G	synonymous
501695	NTHI477_RS02360	486	G	A	synonymous
501701	NTHI477_RS02360	480	G	A	synonymous
501707	NTHI477_RS02360	474	A	T	synonymous
501728	NTHI477_RS02360	453	G	A	synonymous
501729	NTHI477_RS02360	452	C	A	nonsynonymous
501734	NTHI477_RS02360	447	G	A	synonymous
501737	NTHI477_RS02360	444	C	T	synonymous
501755	NTHI477_RS02360	426	A	G	synonymous
501758	NTHI477_RS02360	423	G	A	synonymous
501760	NTHI477_RS02360	421	A	G	nonsynonymous
501764	NTHI477_RS02360	417	C	T	synonymous
501767	NTHI477_RS02360	414	T	C	synonymous
501773	NTHI477_RS02360	408	T	A	synonymous
501779	NTHI477_RS02360	402	G	A	synonymous
501824	NTHI477_RS02360	357	C	G	synonymous
501833	NTHI477_RS02360	348	A	G	synonymous
501836	NTHI477_RS02360	345	C	T	synonymous
501839	NTHI477_RS02360	342	G	T	synonymous
501848	NTHI477_RS02360	333	A	G	synonymous
501851	NTHI477_RS02360	330	C	T	synonymous
501853	NTHI477_RS02360	328	A	G	synonymous
501854	NTHI477_RS02360	327	T	C	synonymous
501857	NTHI477_RS02360	324	T	C	synonymous
501866	NTHI477_RS02360	315	A	C	synonymous
501875	NTHI477_RS02360	306	A	G	synonymous
501881	NTHI477_RS02360	300	C	T	synonymous
501888	NTHI477_RS02360	293	A	G	nonsynonymous

501890	NTHI477_RS02360	291	A	G	synonymous
501905	NTHI477_RS02360	276	T	C	synonymous
501926	NTHI477_RS02360	255	G	A	synonymous
501932	NTHI477_RS02360	249	C	T	synonymous
501938	NTHI477_RS02360	243	G	A	synonymous
501968	NTHI477_RS02360	213	A	G	synonymous
501969	NTHI477_RS02360	212	G	T	nonsynonymous
501972	NTHI477_RS02360	209	G	A	nonsynonymous
501973	NTHI477_RS02360	208	A	T	nonsynonymous
502022	NTHI477_RS02360	159	G	A	synonymous
502031	NTHI477_RS02360	150	T	C	synonymous
502036	NTHI477_RS02360	145	A	C	nonsynonymous
502048	NTHI477_RS02360	133	C	T	nonsynonymous
502060	NTHI477_RS02360	121	G	T	nonsynonymous
502069	NTHI477_RS02360	112	T	A	nonsynonymous
502071	NTHI477_RS02360	110	C	G	nonsynonymous
502076	NTHI477_RS02360	105	G	T	synonymous
502076	NTHI477_RS02360	105	G	A	synonymous
502079	NTHI477_RS02360	102	T	C	synonymous
502098	NTHI477_RS02360	83	T	G	nonsynonymous
502100	NTHI477_RS02360	81	A	T	synonymous
502106	NTHI477_RS02360	75	A	T	nonsynonymous
502119	NTHI477_RS02360	62	A	G	nonsynonymous
502124	NTHI477_RS02360	57	T	A	synonymous
502133	NTHI477_RS02360	48	A	C	nonsynonymous
502139	NTHI477_RS02360	42	T	A	synonymous
502262	NTHI477_RS02365	307	G	T	nonsynonymous
502266	NTHI477_RS02365	303	C	T	synonymous
502275	NTHI477_RS02365	294	A	C	synonymous
502290	NTHI477_RS02365	279	A	T	synonymous
502302	NTHI477_RS02365	267	T	A	synonymous
502308	NTHI477_RS02365	261	G	T	synonymous
502314	NTHI477_RS02365	255	A	T	synonymous
502426	NTHI477_RS02365	143	C	T	nonsynonymous
502442	NTHI477_RS02365	127	G	A	synonymous
502470	NTHI477_RS02365	99	C	T	synonymous
502503	NTHI477_RS02365	66	C	T	synonymous
502608	NTHI477_RS02370	206	A	C	nonsynonymous
502616	NTHI477_RS02370	198	A	G	synonymous
502640	NTHI477_RS02370	174	A	G	synonymous
502661	NTHI477_RS02370	153	A	G	synonymous
502664	NTHI477_RS02370	150	T	C	synonymous
502666	NTHI477_RS02370	148	C	T	nonsynonymous
502711	NTHI477_RS02370	103	G	A	nonsynonymous
502732	NTHI477_RS02370	82	C	T	nonsynonymous

502743	NTHI477_RS02370	71	A	G	nonsynonymous
502811	NTHI477_RS02370	3	C	A	nonsynonymous
502899	intergenic	intergenic	T	G	intergenic
502902	intergenic	intergenic	C	T	intergenic
502924	intergenic	intergenic	A	G	intergenic
502950	intergenic	intergenic	G	A	intergenic
502951	intergenic	intergenic	C	T	intergenic
502955	intergenic	intergenic	C	G	intergenic
502992	NTHI477_RS02375	9	A	C	nonsynonymous
503007	NTHI477_RS02375	24	T	C	synonymous
503013	NTHI477_RS02375	30	G	A	synonymous
503019	NTHI477_RS02375	36	T	G	synonymous
503022	NTHI477_RS02375	39	C	A	synonymous
503028	NTHI477_RS02375	45	C	T	synonymous
503040	NTHI477_RS02375	57	C	T	synonymous
503049	NTHI477_RS02375	66	G	A	synonymous
503076	NTHI477_RS02375	93	A	C	nonsynonymous
503079	NTHI477_RS02375	96	A	G	synonymous
503095	NTHI477_RS02375	112	A	G	nonsynonymous
503103	NTHI477_RS02375	120	T	A	synonymous
503106	NTHI477_RS02375	123	T	C	synonymous
503115	NTHI477_RS02375	132	G	A	synonymous
503130	NTHI477_RS02375	147	T	C	synonymous
503139	NTHI477_RS02375	156	T	C	synonymous
503160	NTHI477_RS02375	177	C	T	synonymous
503163	NTHI477_RS02375	180	T	C	synonymous
503169	NTHI477_RS02375	186	A	G	synonymous
503176	NTHI477_RS02375	193	G	T	nonsynonymous
503181	NTHI477_RS02375	198	G	T	synonymous
503184	NTHI477_RS02375	201	T	A	synonymous
503187	NTHI477_RS02375	204	C	A	synonymous
503190	NTHI477_RS02375	207	T	C	synonymous
503205	NTHI477_RS02375	222	C	T	synonymous
503208	NTHI477_RS02375	225	C	T	synonymous
503220	NTHI477_RS02375	237	C	G	synonymous
503226	NTHI477_RS02375	243	C	A	synonymous
503226	NTHI477_RS02375	243	C	G	synonymous
503235	NTHI477_RS02375	252	T	A	synonymous
503244	NTHI477_RS02375	261	T	C	synonymous
503244	NTHI477_RS02375	261	T	G	synonymous
503247	NTHI477_RS02375	264	G	A	synonymous
503274	NTHI477_RS02375	291	C	T	synonymous
503277	NTHI477_RS02375	294	A	G	synonymous
503313	NTHI477_RS02375	330	A	G	synonymous
503316	NTHI477_RS02375	333	C	T	synonymous

503322	NTHI477_RS02375	339	C	A	synonymous
503331	NTHI477_RS02375	348	G	A	synonymous
503334	NTHI477_RS02375	351	T	G	synonymous
503334	NTHI477_RS02375	351	T	A	synonymous
503340	NTHI477_RS02375	357	C	T	synonymous
503346	NTHI477_RS02375	363	G	A	synonymous
503349	NTHI477_RS02375	366	T	C	synonymous
503352	NTHI477_RS02375	369	G	A	synonymous
503352	NTHI477_RS02375	369	G	C	synonymous
503364	NTHI477_RS02375	381	A	G	synonymous
503367	NTHI477_RS02375	384	G	A	synonymous
503373	NTHI477_RS02375	390	C	A	synonymous
503373	NTHI477_RS02375	390	C	G	synonymous
503376	NTHI477_RS02375	393	A	C	synonymous
503389	NTHI477_RS02375	406	A	G	nonsynonymous
503392	NTHI477_RS02375	409	T	G	nonsynonymous
503399	NTHI477_RS02375	416	C	A	nonsynonymous
503400	NTHI477_RS02375	417	A	T	synonymous
503402	NTHI477_RS02375	419	A	C	nonsynonymous
503403	NTHI477_RS02375	420	A	G	synonymous
503406	NTHI477_RS02375	423	G	A	synonymous
503407	NTHI477_RS02375	424	C	T	nonsynonymous
503409	NTHI477_RS02375	426	T	C	synonymous
503410	NTHI477_RS02375	427	T	G	nonsynonymous
503412	NTHI477_RS02375	429	A	T	synonymous
503416	NTHI477_RS02375	433	T	C	synonymous
503418	NTHI477_RS02375	435	G	A	synonymous
503422	NTHI477_RS02375	439	T	G	nonsynonymous
503424	NTHI477_RS02375	441	T	A	synonymous
503430	NTHI477_RS02375	447	T	C	synonymous
503435	NTHI477_RS02375	452	G	A	nonsynonymous
503436	NTHI477_RS02375	453	C	T	synonymous
503442	NTHI477_RS02375	459	G	T	synonymous
503442	NTHI477_RS02375	459	G	C	synonymous
503443	NTHI477_RS02375	460	A	C	nonsynonymous
503444	NTHI477_RS02375	461	G	A	nonsynonymous
503446	NTHI477_RS02375	463	A	G	nonsynonymous
503447	NTHI477_RS02375	464	A	C	nonsynonymous
503447	NTHI477_RS02375	464	A	T	nonsynonymous
503448	NTHI477_RS02375	465	T	G	nonsynonymous
503451	NTHI477_RS02375	468	G	A	synonymous
503452	NTHI477_RS02375	469	C	T	nonsynonymous
503453	NTHI477_RS02375	470	T	A	nonsynonymous
503454	NTHI477_RS02375	471	T	G	synonymous
503457	NTHI477_RS02375	474	T	C	synonymous

503463	NTHI477_RS02375	480	C	T	synonymous
503482	intergenic	intergenic	T	C	intergenic
503488	intergenic	intergenic	C	T	intergenic
503507	intergenic	intergenic	A	G	intergenic
503541	intergenic	intergenic	A	C	intergenic
503574	NTHI477_RS02380	27	G	C	synonymous
503583	NTHI477_RS02380	36	A	T	synonymous
503586	NTHI477_RS02380	39	T	C	synonymous
503622	NTHI477_RS02380	75	T	A	synonymous
503625	NTHI477_RS02380	78	T	C	synonymous
503626	NTHI477_RS02380	79	G	A	nonsynonymous
503652	NTHI477_RS02380	105	C	T	synonymous
503658	NTHI477_RS02380	111	C	T	synonymous
503706	NTHI477_RS02380	159	A	G	synonymous
503730	NTHI477_RS02380	183	A	G	synonymous
503731	NTHI477_RS02380	184	T	C	nonsynonymous
503749	NTHI477_RS02380	202	G	A	nonsynonymous
503769	NTHI477_RS02380	222	T	C	synonymous
503776	NTHI477_RS02380	229	C	T	nonsynonymous
503779	NTHI477_RS02380	232	G	A	nonsynonymous
503793	NTHI477_RS02380	246	A	G	synonymous
503796	NTHI477_RS02380	249	C	A	synonymous
503806	NTHI477_RS02380	259	G	A	nonsynonymous
503808	NTHI477_RS02380	261	T	G	synonymous
503820	NTHI477_RS02380	273	G	A	synonymous
503823	NTHI477_RS02380	276	T	A	synonymous
503832	NTHI477_RS02380	285	C	G	synonymous
503836	NTHI477_RS02380	289	C	A	nonsynonymous
503847	NTHI477_RS02380	300	G	A	synonymous
503848	NTHI477_RS02380	301	C	T	nonsynonymous
503887	NTHI477_RS02380	340	G	C	nonsynonymous
503889	NTHI477_RS02380	342	T	C	synonymous
503897	NTHI477_RS02380	350	C	T	nonsynonymous
503906	NTHI477_RS02380	359	A	G	nonsynonymous
503913	NTHI477_RS02380	366	C	T	synonymous
503919	NTHI477_RS02380	372	C	T	synonymous
503925	NTHI477_RS02380	378	T	C	synonymous
503931	NTHI477_RS02380	384	A	C	synonymous
503934	NTHI477_RS02380	387	C	T	synonymous
503946	NTHI477_RS02380	399	G	A	synonymous
503949	NTHI477_RS02380	402	T	A	synonymous
503971	NTHI477_RS02380	424	G	A	nonsynonymous
503991	NTHI477_RS02380	444	T	C	synonymous
503997	NTHI477_RS02380	450	C	T	synonymous
504003	NTHI477_RS02380	456	T	C	synonymous

504012	NTHI477_RS02380	465	G	A	synonymous
504036	NTHI477_RS02380	489	T	C	synonymous
504045	NTHI477_RS02380	498	A	G	synonymous
504046	NTHI477_RS02380	499	G	A	nonsynonymous
504048	NTHI477_RS02380	501	T	G	synonymous
504060	NTHI477_RS02380	513	C	T	synonymous
504063	NTHI477_RS02380	516	A	G	synonymous
504078	NTHI477_RS02380	531	C	T	synonymous
504085	NTHI477_RS02380	538	C	T	nonsynonymous
504096	NTHI477_RS02380	549	C	T	synonymous
504102	NTHI477_RS02380	555	G	A	synonymous
504120	NTHI477_RS02380	573	T	C	synonymous
504142	NTHI477_RS02380	595	A	G	nonsynonymous
504149	NTHI477_RS02380	602	A	G	nonsynonymous
504149	NTHI477_RS02380	602	A	T	nonsynonymous
504151	NTHI477_RS02380	604	G	A	nonsynonymous
504152	NTHI477_RS02380	605	C	T	nonsynonymous
504153	NTHI477_RS02380	606	A	T	synonymous
504153	NTHI477_RS02380	606	A	G	synonymous
504180	NTHI477_RS02380	633	A	G	synonymous
504186	NTHI477_RS02380	639	T	A	synonymous
504186	NTHI477_RS02380	639	T	G	synonymous
504189	NTHI477_RS02380	642	A	C	nonsynonymous
504190	NTHI477_RS02380	643	A	G	nonsynonymous
504192	NTHI477_RS02380	645	A	C	synonymous
504199	NTHI477_RS02380	652	G	A	nonsynonymous
504216	NTHI477_RS02380	669	G	A	synonymous
504232	NTHI477_RS02380	685	G	A	nonsynonymous
504234	NTHI477_RS02380	687	G	C	synonymous
504258	NTHI477_RS02380	711	A	T	synonymous
504273	NTHI477_RS02380	726	A	G	synonymous
504288	NTHI477_RS02380	741	G	A	synonymous
504294	NTHI477_RS02380	747	C	T	synonymous
504309	NTHI477_RS02380	762	A	G	synonymous
504324	NTHI477_RS02380	777	G	A	synonymous
504327	NTHI477_RS02380	780	A	C	synonymous
504330	NTHI477_RS02380	783	T	C	synonymous
504331	NTHI477_RS02380	784	G	T	nonsynonymous
504333	NTHI477_RS02380	786	T	G	synonymous
504339	NTHI477_RS02380	792	T	C	synonymous
504360	NTHI477_RS02380	813	C	T	synonymous
504361	NTHI477_RS02380	814	A	G	nonsynonymous
504362	NTHI477_RS02380	815	C	T	nonsynonymous
504363	NTHI477_RS02380	816	G	T	synonymous
504381	NTHI477_RS02380	834	A	G	synonymous

504387	NTHI477_RS02380	840	C	A	nonsynonymous
504395	NTHI477_RS02380	848	T	C	nonsynonymous
504396	NTHI477_RS02380	849	T	G	nonsynonymous
504396	NTHI477_RS02380	849	T	A	synonymous
504399	NTHI477_RS02380	852	T	C	synonymous
504400	NTHI477_RS02380	853	G	T	nonsynonymous
504403	NTHI477_RS02380	856	C	T	nonsynonymous
504411	NTHI477_RS02380	864	A	G	synonymous
504415	NTHI477_RS02380	868	G	A	nonsynonymous
504417	NTHI477_RS02380	870	A	T	synonymous
504441	NTHI477_RS02380	894	T	C	synonymous
504444	NTHI477_RS02380	897	A	T	synonymous
504458	NTHI477_RS02380	911	T	C	nonsynonymous
504462	NTHI477_RS02380	915	T	C	synonymous
504471	NTHI477_RS02380	924	A	G	synonymous
504483	NTHI477_RS02380	936	C	T	synonymous
504498	NTHI477_RS02380	951	G	A	synonymous
504510	NTHI477_RS02380	963	G	A	synonymous
504522	NTHI477_RS02380	975	C	T	synonymous
504531	NTHI477_RS02380	984	T	C	synonymous
504537	NTHI477_RS02380	990	T	C	synonymous
504540	NTHI477_RS02380	993	T	C	synonymous
504542	NTHI477_RS02380	995	A	G	nonsynonymous
504570	NTHI477_RS02380	1023	G	A	synonymous
504573	NTHI477_RS02380	1026	C	T	synonymous
504576	NTHI477_RS02380	1029	A	C	synonymous
504577	NTHI477_RS02380	1030	A	G	nonsynonymous
504578	NTHI477_RS02380	1031	T	C	nonsynonymous
504582	NTHI477_RS02380	1035	A	G	synonymous
504588	NTHI477_RS02380	1041	C	A	synonymous
504588	NTHI477_RS02380	1041	C	G	synonymous
504603	NTHI477_RS02380	1056	A	G	synonymous
504603	NTHI477_RS02380	1056	A	T	synonymous
504638	intergenic	intergenic	G	T	intergenic
504663	intergenic	intergenic	A	G	intergenic
504679	intergenic	intergenic	T	C	intergenic
504684	intergenic	intergenic	T	C	intergenic
504686	intergenic	intergenic	C	T	intergenic
504725	intergenic	intergenic	C	T	intergenic
504727	intergenic	intergenic	G	A	intergenic
504748	intergenic	intergenic	T	C	intergenic
504790	NTHI477_RS02385	15	T	C	synonymous
504811	NTHI477_RS02385	36	A	C	synonymous
504841	NTHI477_RS02385	66	C	T	synonymous
504846	NTHI477_RS02385	71	C	T	nonsynonymous

504862	NTHI477_RS02385	87	T	C	synonymous
504865	NTHI477_RS02385	90	C	T	synonymous
504871	NTHI477_RS02385	96	T	C	synonymous
504907	NTHI477_RS02385	132	G	A	synonymous
504916	NTHI477_RS02385	141	T	C	synonymous
504919	NTHI477_RS02385	144	A	G	synonymous
504922	NTHI477_RS02385	147	G	A	synonymous
504949	NTHI477_RS02385	174	T	C	synonymous
504961	NTHI477_RS02385	186	C	G	synonymous
504964	NTHI477_RS02385	189	G	A	synonymous
504979	NTHI477_RS02385	204	C	T	synonymous
504991	NTHI477_RS02385	216	T	G	synonymous
505054	NTHI477_RS02385	279	T	C	synonymous
505057	NTHI477_RS02385	282	T	A	synonymous
505060	NTHI477_RS02385	285	A	G	synonymous
505084	NTHI477_RS02385	309	A	T	synonymous
505156	NTHI477_RS02385	381	C	A	synonymous
505162	NTHI477_RS02385	387	T	A	synonymous
505180	NTHI477_RS02385	405	C	G	synonymous
505323	NTHI477_RS02385	548	A	C	nonsynonymous
505342	NTHI477_RS02385	567	T	A	synonymous
505345	NTHI477_RS02385	570	T	C	synonymous
505348	NTHI477_RS02385	573	A	T	synonymous
505349	NTHI477_RS02385	574	G	A	nonsynonymous
505359	NTHI477_RS02385	584	A	G	nonsynonymous
505370	NTHI477_RS02385	595	G	A	nonsynonymous
505378	NTHI477_RS02385	603	G	A	synonymous
505381	NTHI477_RS02385	606	G	A	synonymous
505387	NTHI477_RS02385	612	C	T	synonymous
505396	NTHI477_RS02385	621	G	A	synonymous
505399	NTHI477_RS02385	624	A	G	synonymous
505429	NTHI477_RS02385	654	C	A	synonymous
505432	NTHI477_RS02385	657	T	C	synonymous
505435	NTHI477_RS02385	660	A	C	synonymous
505447	NTHI477_RS02385	672	T	C	synonymous
505456	NTHI477_RS02385	681	C	T	synonymous
505459	NTHI477_RS02385	684	G	A	synonymous
505471	NTHI477_RS02385	696	G	A	synonymous
505487	NTHI477_RS02385	712	T	C	synonymous
505489	NTHI477_RS02385	714	G	A	synonymous
505495	NTHI477_RS02385	720	A	G	synonymous
505495	NTHI477_RS02385	720	A	T	synonymous
505496	NTHI477_RS02385	721	G	T	nonsynonymous
505498	NTHI477_RS02385	723	G	A	synonymous
505525	NTHI477_RS02385	750	A	G	synonymous

505528	NTHI477_RS02385	753	T	C	synonymous
505534	NTHI477_RS02385	759	A	G	synonymous
505540	NTHI477_RS02385	765	T	A	synonymous
505543	NTHI477_RS02385	768	T	C	synonymous
505546	NTHI477_RS02385	771	C	A	synonymous
505552	NTHI477_RS02385	777	T	C	synonymous
505561	NTHI477_RS02385	786	A	T	synonymous
505569	NTHI477_RS02385	794	C	A	nonsynonymous
505579	NTHI477_RS02385	804	A	T	synonymous
505585	NTHI477_RS02385	810	C	T	synonymous
505586	NTHI477_RS02385	811	G	A	nonsynonymous
505594	NTHI477_RS02385	819	C	A	synonymous
505609	NTHI477_RS02385	834	T	C	synonymous
505621	NTHI477_RS02385	846	C	T	synonymous
505633	NTHI477_RS02385	858	T	A	synonymous
505642	NTHI477_RS02385	867	C	T	synonymous
505645	NTHI477_RS02385	870	C	T	synonymous
505648	NTHI477_RS02385	873	C	G	synonymous
505651	NTHI477_RS02385	876	A	G	synonymous
505657	NTHI477_RS02385	882	A	C	synonymous
505676	NTHI477_RS02385	901	A	G	nonsynonymous
505685	NTHI477_RS02385	910	C	T	nonsynonymous
505693	NTHI477_RS02385	918	G	C	nonsynonymous
505712	NTHI477_RS02385	937	G	A	nonsynonymous
505720	NTHI477_RS02385	945	C	T	synonymous
505726	NTHI477_RS02385	951	T	C	synonymous
505760	NTHI477_RS02385	985	T	C	synonymous
505771	NTHI477_RS02385	996	C	T	synonymous
505777	NTHI477_RS02385	1002	G	A	synonymous
505792	NTHI477_RS02385	1017	C	T	synonymous
505798	NTHI477_RS02385	1023	C	T	synonymous
505801	NTHI477_RS02385	1026	T	G	nonsynonymous
505837	NTHI477_RS02385	1062	T	A	synonymous
505855	NTHI477_RS02385	1080	C	T	synonymous
505856	NTHI477_RS02385	1081	T	C	synonymous
505862	NTHI477_RS02385	1087	G	A	nonsynonymous
505864	NTHI477_RS02385	1089	A	T	nonsynonymous
505872	NTHI477_RS02385	1097	C	T	nonsynonymous
505873	NTHI477_RS02385	1098	C	A	synonymous
505879	NTHI477_RS02385	1104	T	C	synonymous
505903	NTHI477_RS02385	1128	T	C	synonymous
505906	NTHI477_RS02385	1131	G	A	synonymous
505912	NTHI477_RS02385	1137	T	A	synonymous
505912	NTHI477_RS02385	1137	T	C	synonymous
505915	NTHI477_RS02385	1140	T	C	synonymous

505918	NTHI477_RS02385	1143	T	A	synonymous
505932	NTHI477_RS02385	1157	G	A	nonsynonymous
505937	NTHI477_RS02385	1162	T	C	synonymous
505948	NTHI477_RS02385	1173	C	T	synonymous
505951	NTHI477_RS02385	1176	T	C	synonymous
505975	intergenic	intergenic	G	C	intergenic
506000	intergenic	intergenic	G	A	intergenic
506040	intergenic	intergenic	T	C	intergenic
506044	intergenic	intergenic	T	C	intergenic
506047	intergenic	intergenic	A	G	intergenic
506055	intergenic	intergenic	C	T	intergenic
506063	intergenic	intergenic	C	T	intergenic
506081	NTHI477_RS02390	619	T	G	nonsynonymous
506124	NTHI477_RS02390	576	A	G	synonymous
506145	NTHI477_RS02390	555	C	A	nonsynonymous
506148	NTHI477_RS02390	552	G	A	synonymous
506151	NTHI477_RS02390	549	T	C	synonymous
506153	NTHI477_RS02390	547	A	G	synonymous
506160	NTHI477_RS02390	540	C	T	synonymous
506163	NTHI477_RS02390	537	A	T	synonymous
506164	NTHI477_RS02390	536	C	T	nonsynonymous
506190	NTHI477_RS02390	510	A	G	synonymous
506205	NTHI477_RS02390	495	T	G	synonymous
506232	NTHI477_RS02390	468	A	G	synonymous
506241	NTHI477_RS02390	459	C	T	synonymous
506253	NTHI477_RS02390	447	G	A	synonymous
506256	NTHI477_RS02390	444	A	G	synonymous
506259	NTHI477_RS02390	441	A	T	synonymous
506259	NTHI477_RS02390	441	A	C	synonymous
506268	NTHI477_RS02390	432	C	G	synonymous
506268	NTHI477_RS02390	432	C	A	synonymous
506271	NTHI477_RS02390	429	G	A	synonymous
506280	NTHI477_RS02390	420	C	T	synonymous
506283	NTHI477_RS02390	417	A	C	synonymous
506294	NTHI477_RS02390	406	G	A	synonymous
506301	NTHI477_RS02390	399	C	T	synonymous
506321	NTHI477_RS02390	379	C	G	nonsynonymous
506325	NTHI477_RS02390	375	G	A	synonymous
506328	NTHI477_RS02390	372	A	G	synonymous
506346	NTHI477_RS02390	354	C	T	synonymous
506355	NTHI477_RS02390	345	G	A	synonymous
506364	NTHI477_RS02390	336	C	T	synonymous
506375	NTHI477_RS02390	325	T	C	nonsynonymous
506376	NTHI477_RS02390	324	G	A	synonymous
506378	NTHI477_RS02390	322	G	A	nonsynonymous

506381	NTHI477_RS02390	319	T	C	nonsynonymous
506400	NTHI477_RS02390	300	G	A	synonymous
506403	NTHI477_RS02390	297	G	A	synonymous
506408	NTHI477_RS02390	292	C	T	nonsynonymous
506418	NTHI477_RS02390	282	C	T	synonymous
506445	NTHI477_RS02390	255	G	C	synonymous
506472	NTHI477_RS02390	228	G	A	synonymous
506481	NTHI477_RS02390	219	G	T	synonymous
506484	NTHI477_RS02390	216	C	T	synonymous
506501	NTHI477_RS02390	199	C	T	nonsynonymous
506508	NTHI477_RS02390	192	A	C	synonymous
506522	NTHI477_RS02390	178	A	G	synonymous
506535	NTHI477_RS02390	165	T	C	synonymous
506544	NTHI477_RS02390	156	A	T	synonymous
506565	NTHI477_RS02390	135	A	G	synonymous
506568	NTHI477_RS02390	132	C	G	synonymous
506586	NTHI477_RS02390	114	T	C	synonymous
506589	NTHI477_RS02390	111	A	G	synonymous
506589	NTHI477_RS02390	111	A	T	synonymous
506604	NTHI477_RS02390	96	T	G	synonymous
506610	NTHI477_RS02390	90	G	A	synonymous
506613	NTHI477_RS02390	87	G	A	synonymous
506637	NTHI477_RS02390	63	A	G	synonymous
506640	NTHI477_RS02390	60	C	T	synonymous
506677	NTHI477_RS02390	23	C	T	nonsynonymous
506700	NTHI477_RS02390	0	A	G	synonymous
506722	NTHI477_RS02395	612	A	G	synonymous
506726	NTHI477_RS02395	608	T	C	nonsynonymous
506746	NTHI477_RS02395	588	C	A	synonymous
506755	NTHI477_RS02395	579	C	T	synonymous
506758	NTHI477_RS02395	576	C	A	synonymous
506758	NTHI477_RS02395	576	C	G	synonymous
506764	NTHI477_RS02395	570	G	A	synonymous
506770	NTHI477_RS02395	564	T	C	synonymous
506772	NTHI477_RS02395	562	A	G	synonymous
506773	NTHI477_RS02395	561	T	C	synonymous
506782	NTHI477_RS02395	552	A	G	synonymous
506809	NTHI477_RS02395	525	G	C	synonymous
506809	NTHI477_RS02395	525	G	T	synonymous
506820	NTHI477_RS02395	514	G	T	nonsynonymous
506830	NTHI477_RS02395	504	A	G	synonymous
506838	NTHI477_RS02395	496	C	T	nonsynonymous
506840	NTHI477_RS02395	494	C	G	nonsynonymous
506850	NTHI477_RS02395	484	C	T	nonsynonymous
506857	NTHI477_RS02395	477	T	G	synonymous

506863	NTHI477_RS02395	471	A	G	synonymous
506886	NTHI477_RS02395	448	A	G	synonymous
506898	NTHI477_RS02395	436	G	A	synonymous
506914	NTHI477_RS02395	420	A	G	synonymous
506926	NTHI477_RS02395	408	A	C	synonymous
506932	NTHI477_RS02395	402	A	C	nonsynonymous
506959	NTHI477_RS02395	375	G	A	synonymous
506977	NTHI477_RS02395	357	C	T	synonymous
506980	NTHI477_RS02395	354	C	T	synonymous
506996	NTHI477_RS02395	338	C	A	nonsynonymous
506998	NTHI477_RS02395	336	T	C	synonymous
507004	NTHI477_RS02395	330	A	G	synonymous
507013	NTHI477_RS02395	321	A	C	synonymous
507019	NTHI477_RS02395	315	T	C	synonymous
507021	NTHI477_RS02395	313	A	G	synonymous
507034	NTHI477_RS02395	300	A	C	synonymous
507037	NTHI477_RS02395	297	C	T	synonymous
507040	NTHI477_RS02395	294	A	G	synonymous
507043	NTHI477_RS02395	291	T	C	synonymous
507061	NTHI477_RS02395	273	T	G	synonymous
507061	NTHI477_RS02395	273	T	A	synonymous
507064	NTHI477_RS02395	270	G	A	synonymous
507094	NTHI477_RS02395	240	G	T	synonymous
507102	NTHI477_RS02395	232	G	T	nonsynonymous
507106	NTHI477_RS02395	228	A	G	synonymous
507114	NTHI477_RS02395	220	T	C	nonsynonymous
507115	NTHI477_RS02395	219	T	C	synonymous
507116	NTHI477_RS02395	218	G	A	nonsynonymous
507139	NTHI477_RS02395	195	A	G	synonymous
507141	NTHI477_RS02395	193	C	T	nonsynonymous
507158	NTHI477_RS02395	176	T	C	nonsynonymous
507163	NTHI477_RS02395	171	A	G	synonymous
507166	NTHI477_RS02395	168	G	A	synonymous
507189	NTHI477_RS02395	145	A	G	nonsynonymous
507191	NTHI477_RS02395	143	A	T	nonsynonymous
507224	NTHI477_RS02395	110	G	A	nonsynonymous
507225	NTHI477_RS02395	109	C	T	nonsynonymous
507231	NTHI477_RS02395	103	G	A	nonsynonymous
507234	NTHI477_RS02395	100	G	A	nonsynonymous
507247	NTHI477_RS02395	87	G	A	synonymous
507265	NTHI477_RS02395	69	A	G	synonymous
507268	NTHI477_RS02395	66	A	G	synonymous
507269	NTHI477_RS02395	65	A	G	nonsynonymous
507280	NTHI477_RS02395	54	G	A	synonymous
507286	NTHI477_RS02395	48	T	C	synonymous

507308	NTHI477_RS02395	26	G	A	nonsynonymous
507319	NTHI477_RS02395	15	G	A	synonymous
507320	NTHI477_RS02395	14	T	C	nonsynonymous
507322	NTHI477_RS02395	12	A	G	synonymous
507324	NTHI477_RS02395	10	G	A	nonsynonymous
507341	NTHI477_RS02400	613	C	A	nonsynonymous
507347	NTHI477_RS02400	607	C	G	nonsynonymous
507348	NTHI477_RS02400	606	G	T	synonymous
507349	NTHI477_RS02400	605	A	G	nonsynonymous
507396	NTHI477_RS02400	558	T	C	synonymous
507422	NTHI477_RS02400	532	T	C	nonsynonymous
507429	NTHI477_RS02400	525	C	T	synonymous
507434	NTHI477_RS02400	520	T	G	nonsynonymous
507453	NTHI477_RS02400	501	C	A	nonsynonymous
507477	NTHI477_RS02400	477	T	A	synonymous
507510	NTHI477_RS02400	444	T	C	synonymous
507511	NTHI477_RS02400	443	G	A	nonsynonymous
507513	NTHI477_RS02400	441	G	A	synonymous
507513	NTHI477_RS02400	441	G	T	synonymous
507516	NTHI477_RS02400	438	T	A	synonymous
507519	NTHI477_RS02400	435	C	T	synonymous
507534	NTHI477_RS02400	420	T	A	synonymous
507542	NTHI477_RS02400	412	A	C	nonsynonymous
507543	NTHI477_RS02400	411	T	A	synonymous
507552	NTHI477_RS02400	402	G	A	synonymous
507555	NTHI477_RS02400	399	G	T	nonsynonymous
507573	NTHI477_RS02400	381	A	T	synonymous
507579	NTHI477_RS02400	375	G	T	nonsynonymous
507581	NTHI477_RS02400	373	T	G	nonsynonymous
507582	NTHI477_RS02400	372	T	C	synonymous
507585	NTHI477_RS02400	369	T	C	synonymous
507611	NTHI477_RS02400	343	G	A	nonsynonymous
507615	NTHI477_RS02400	339	A	G	synonymous
507621	NTHI477_RS02400	333	T	C	synonymous
507621	NTHI477_RS02400	333	T	A	synonymous
507624	NTHI477_RS02400	330	A	T	synonymous
507627	NTHI477_RS02400	327	C	T	synonymous
507628	NTHI477_RS02400	326	G	A	nonsynonymous
507639	NTHI477_RS02400	315	G	A	synonymous
507642	NTHI477_RS02400	312	T	A	synonymous
507651	NTHI477_RS02400	303	G	A	synonymous
507660	NTHI477_RS02400	294	T	G	synonymous
507669	NTHI477_RS02400	285	G	A	synonymous
507684	NTHI477_RS02400	270	G	A	synonymous
507693	NTHI477_RS02400	261	C	T	synonymous

507705	NTHI477_RS02400	249	G	A	synonymous
507708	NTHI477_RS02400	246	G	A	synonymous
507711	NTHI477_RS02400	243	G	A	synonymous
507714	NTHI477_RS02400	240	T	C	synonymous
507717	NTHI477_RS02400	237	A	T	synonymous
507726	NTHI477_RS02400	228	A	G	synonymous
507735	NTHI477_RS02400	219	G	A	synonymous
507738	NTHI477_RS02400	216	A	G	synonymous
507747	NTHI477_RS02400	207	A	T	synonymous
507753	NTHI477_RS02400	201	G	A	synonymous
507774	NTHI477_RS02400	180	A	G	synonymous
507786	NTHI477_RS02400	168	G	T	synonymous
507789	NTHI477_RS02400	165	T	G	synonymous
507789	NTHI477_RS02400	165	T	A	synonymous
507792	NTHI477_RS02400	162	C	G	synonymous
507801	NTHI477_RS02400	153	G	A	synonymous
507807	NTHI477_RS02400	147	T	A	synonymous
507825	NTHI477_RS02400	129	G	C	synonymous
507870	NTHI477_RS02400	84	C	T	synonymous
507872	NTHI477_RS02400	82	A	C	nonsynonymous
507879	NTHI477_RS02400	75	G	A	synonymous
507889	NTHI477_RS02400	65	G	A	nonsynonymous
507904	NTHI477_RS02400	50	G	A	nonsynonymous
507906	NTHI477_RS02400	48	G	A	synonymous
507910	NTHI477_RS02400	44	G	C	nonsynonymous
507980	NTHI477_RS02405	462	T	C	synonymous
508025	NTHI477_RS02405	417	G	A	synonymous
508031	NTHI477_RS02405	411	A	G	synonymous
508070	NTHI477_RS02405	372	C	T	synonymous
508105	NTHI477_RS02405	337	T	C	nonsynonymous
508118	NTHI477_RS02405	324	G	T	synonymous
508124	NTHI477_RS02405	318	C	T	synonymous
508124	NTHI477_RS02405	318	C	G	synonymous
508125	NTHI477_RS02405	317	G	A	nonsynonymous
508142	NTHI477_RS02405	300	A	C	synonymous
508145	NTHI477_RS02405	297	G	T	synonymous
508145	NTHI477_RS02405	297	G	C	synonymous
508148	NTHI477_RS02405	294	C	T	synonymous
508151	NTHI477_RS02405	291	A	G	synonymous
508166	NTHI477_RS02405	276	G	C	synonymous
508178	NTHI477_RS02405	264	A	C	synonymous
508193	NTHI477_RS02405	249	A	G	synonymous
508199	NTHI477_RS02405	243	T	C	synonymous
508206	NTHI477_RS02405	236	G	A	nonsynonymous
508226	NTHI477_RS02405	216	A	G	synonymous

508238	NTHI477_RS02405	204	T	A	synonymous
508304	NTHI477_RS02405	138	T	A	synonymous
508307	NTHI477_RS02405	135	G	A	synonymous
508337	NTHI477_RS02405	105	A	G	synonymous
508373	NTHI477_RS02405	69	C	T	synonymous
508400	NTHI477_RS02405	42	G	A	synonymous
508407	NTHI477_RS02405	35	G	A	nonsynonymous
508428	NTHI477_RS02405	14	G	A	nonsynonymous
508429	NTHI477_RS02405	13	C	T	nonsynonymous
508432	NTHI477_RS02405	10	A	T	nonsynonymous
508460	NTHI477_RS02410	678	C	T	synonymous
508461	NTHI477_RS02410	677	G	A	nonsynonymous
508489	NTHI477_RS02410	649	T	A	nonsynonymous
508490	NTHI477_RS02410	648	T	G	synonymous
508490	NTHI477_RS02410	648	T	A	synonymous
508505	NTHI477_RS02410	633	T	C	synonymous
508520	NTHI477_RS02410	618	T	G	nonsynonymous
508565	NTHI477_RS02410	573	G	C	synonymous
508592	NTHI477_RS02410	546	A	G	synonymous
508619	NTHI477_RS02410	519	C	T	synonymous
508622	NTHI477_RS02410	516	A	G	synonymous
508625	NTHI477_RS02410	513	C	T	synonymous
508631	NTHI477_RS02410	507	A	T	synonymous
508634	NTHI477_RS02410	504	G	C	synonymous
508643	NTHI477_RS02410	495	T	G	synonymous
508651	NTHI477_RS02410	487	C	T	nonsynonymous
508652	NTHI477_RS02410	486	A	G	synonymous
508676	NTHI477_RS02410	462	T	C	synonymous
508682	NTHI477_RS02410	456	A	G	synonymous
508693	NTHI477_RS02410	445	C	G	nonsynonymous
508697	NTHI477_RS02410	441	T	C	synonymous
508715	NTHI477_RS02410	423	G	A	synonymous
508718	NTHI477_RS02410	420	T	A	synonymous
508719	NTHI477_RS02410	419	G	A	nonsynonymous
508763	NTHI477_RS02410	375	C	T	synonymous
508787	NTHI477_RS02410	351	A	G	synonymous
508799	NTHI477_RS02410	339	T	A	synonymous
508802	NTHI477_RS02410	336	T	A	synonymous
508849	NTHI477_RS02410	289	A	G	synonymous
508871	NTHI477_RS02410	267	G	A	synonymous
508879	NTHI477_RS02410	259	C	T	nonsynonymous
508883	NTHI477_RS02410	255	A	T	synonymous
508892	NTHI477_RS02410	246	T	A	synonymous
508910	NTHI477_RS02410	228	G	T	synonymous
508931	NTHI477_RS02410	207	G	A	synonymous

508940	NTHI477_RS02410	198	A	G	synonymous
508940	NTHI477_RS02410	198	A	T	synonymous
508943	NTHI477_RS02410	195	A	G	synonymous
508952	NTHI477_RS02410	186	G	T	synonymous
508960	NTHI477_RS02410	178	C	A	nonsynonymous
508973	NTHI477_RS02410	165	G	A	synonymous
509015	NTHI477_RS02410	123	A	G	synonymous
509030	NTHI477_RS02410	108	T	C	synonymous
509041	NTHI477_RS02410	97	C	T	nonsynonymous
509044	NTHI477_RS02410	94	G	T	nonsynonymous
509045	NTHI477_RS02410	93	C	A	synonymous
509047	NTHI477_RS02410	91	A	C	nonsynonymous
509053	NTHI477_RS02410	85	C	T	nonsynonymous
509057	NTHI477_RS02410	81	C	T	synonymous
509072	NTHI477_RS02410	66	A	G	synonymous
509114	NTHI477_RS02410	24	T	C	synonymous
509117	NTHI477_RS02410	21	A	G	synonymous
509118	NTHI477_RS02410	20	G	A	nonsynonymous
509120	NTHI477_RS02410	18	G	A	synonymous
509129	NTHI477_RS02410	9	C	T	synonymous
509168	intergenic	intergenic	C	T	intergenic
509277	NTHI477_RS02415	51	C	T	synonymous
509305	NTHI477_RS02415	79	C	T	nonsynonymous
509307	NTHI477_RS02415	81	G	A	synonymous
509309	NTHI477_RS02415	83	C	T	nonsynonymous
509313	NTHI477_RS02415	87	A	C	synonymous
509319	NTHI477_RS02415	93	T	C	synonymous
509324	NTHI477_RS02415	98	T	C	nonsynonymous
509337	NTHI477_RS02415	111	G	C	synonymous
509355	NTHI477_RS02415	129	C	T	synonymous
509408	NTHI477_RS02415	182	G	A	nonsynonymous
509415	NTHI477_RS02415	189	A	C	synonymous
509440	NTHI477_RS02415	214	G	A	nonsynonymous
509442	NTHI477_RS02415	216	C	T	synonymous
509445	NTHI477_RS02415	219	C	A	synonymous
509451	NTHI477_RS02415	225	A	G	synonymous
509472	NTHI477_RS02415	246	T	C	synonymous
509481	NTHI477_RS02415	255	T	C	synonymous
509502	NTHI477_RS02415	276	A	G	synonymous
509542	NTHI477_RS02415	316	A	G	nonsynonymous
509544	NTHI477_RS02415	318	T	G	nonsynonymous
509556	NTHI477_RS02415	330	A	G	synonymous
509560	NTHI477_RS02415	334	A	G	nonsynonymous
509613	NTHI477_RS02415	387	G	A	synonymous
509634	NTHI477_RS02415	408	G	A	synonymous

509658	NTHI477_RS02420	534	A	G	synonymous
509684	NTHI477_RS02420	508	T	G	nonsynonymous
509688	NTHI477_RS02420	504	G	A	synonymous
509700	NTHI477_RS02420	492	T	A	synonymous
509700	NTHI477_RS02420	492	T	G	synonymous
509700	NTHI477_RS02420	492	T	C	synonymous
509706	NTHI477_RS02420	486	T	C	synonymous
509711	NTHI477_RS02420	481	G	A	synonymous
509718	NTHI477_RS02420	474	C	T	synonymous
509721	NTHI477_RS02420	471	T	G	synonymous
509729	NTHI477_RS02420	463	C	T	nonsynonymous
509733	NTHI477_RS02420	459	T	C	synonymous
509742	NTHI477_RS02420	450	T	C	synonymous
509743	NTHI477_RS02420	449	A	G	nonsynonymous
509746	NTHI477_RS02420	446	C	A	nonsynonymous
509772	NTHI477_RS02420	420	C	T	synonymous
509778	NTHI477_RS02420	414	C	T	synonymous
509790	NTHI477_RS02420	402	T	C	synonymous
509791	NTHI477_RS02420	401	C	T	nonsynonymous
509793	NTHI477_RS02420	399	A	C	synonymous
509820	NTHI477_RS02420	372	T	C	synonymous
509853	NTHI477_RS02420	339	T	C	synonymous
509892	NTHI477_RS02420	300	A	G	synonymous
509934	NTHI477_RS02420	258	A	G	synonymous
509937	NTHI477_RS02420	255	G	A	synonymous
509943	NTHI477_RS02420	249	C	T	synonymous
509944	NTHI477_RS02420	248	A	G	nonsynonymous
509976	NTHI477_RS02420	216	T	C	synonymous
509978	NTHI477_RS02420	214	G	A	synonymous
509982	NTHI477_RS02420	210	G	A	synonymous
509991	NTHI477_RS02420	201	A	C	synonymous
509994	NTHI477_RS02420	198	G	T	synonymous
509995	NTHI477_RS02420	197	G	A	nonsynonymous
509997	NTHI477_RS02420	195	G	A	synonymous
510003	NTHI477_RS02420	189	C	T	synonymous
510015	NTHI477_RS02420	177	C	T	synonymous
510024	NTHI477_RS02420	168	G	A	synonymous
510029	NTHI477_RS02420	163	C	T	nonsynonymous
510036	NTHI477_RS02420	156	A	G	synonymous
510037	NTHI477_RS02420	155	G	T	nonsynonymous
510038	NTHI477_RS02420	154	A	C	nonsynonymous
510040	NTHI477_RS02420	152	C	A	nonsynonymous
510048	NTHI477_RS02420	144	C	T	synonymous
510048	NTHI477_RS02420	144	C	A	nonsynonymous
510051	NTHI477_RS02420	141	T	G	nonsynonymous

510069	NTHI477_RS02420	123	C	T	synonymous
510104	NTHI477_RS02420	88	A	G	nonsynonymous
510106	NTHI477_RS02420	86	T	C	nonsynonymous
510116	NTHI477_RS02420	76	T	C	nonsynonymous
510122	NTHI477_RS02420	70	T	C	nonsynonymous
510129	NTHI477_RS02420	63	C	T	nonsynonymous
510132	NTHI477_RS02420	60	C	T	synonymous
510140	NTHI477_RS02420	52	C	T	nonsynonymous
510222	NTHI477_RS02425	687	A	G	synonymous
510225	NTHI477_RS02425	684	T	C	synonymous
510246	NTHI477_RS02425	663	T	G	synonymous
510247	NTHI477_RS02425	662	G	A	nonsynonymous
510287	NTHI477_RS02425	622	G	A	synonymous
510294	NTHI477_RS02425	615	C	G	synonymous
510347	NTHI477_RS02425	562	C	T	nonsynonymous
510348	NTHI477_RS02425	561	C	A	synonymous
510361	NTHI477_RS02425	548	A	G	nonsynonymous
510381	NTHI477_RS02425	528	C	T	synonymous
510432	NTHI477_RS02425	477	T	C	synonymous
510516	NTHI477_RS02425	393	G	A	synonymous
510522	NTHI477_RS02425	387	G	A	synonymous
510539	NTHI477_RS02425	370	G	A	synonymous
510540	NTHI477_RS02425	369	G	A	synonymous
510612	NTHI477_RS02425	297	C	T	synonymous
510616	NTHI477_RS02425	293	G	A	nonsynonymous
510617	NTHI477_RS02425	292	C	T	nonsynonymous
510618	NTHI477_RS02425	291	A	T	synonymous
510624	NTHI477_RS02425	285	C	A	synonymous
510627	NTHI477_RS02425	282	T	C	synonymous
510633	NTHI477_RS02425	276	G	A	synonymous
510648	NTHI477_RS02425	261	C	A	nonsynonymous
510650	NTHI477_RS02425	259	A	G	synonymous
510654	NTHI477_RS02425	255	C	T	synonymous
510672	NTHI477_RS02425	237	C	T	synonymous
510675	NTHI477_RS02425	234	G	A	synonymous
510693	NTHI477_RS02425	216	G	T	synonymous
510766	NTHI477_RS02425	143	G	T	nonsynonymous
510810	NTHI477_RS02425	99	C	T	nonsynonymous
510810	NTHI477_RS02425	99	C	G	nonsynonymous
510812	NTHI477_RS02425	97	T	A	nonsynonymous
510816	NTHI477_RS02425	93	C	T	synonymous
510822	NTHI477_RS02425	87	T	C	synonymous
510825	NTHI477_RS02425	84	T	A	synonymous
510826	NTHI477_RS02425	83	G	A	nonsynonymous
510831	NTHI477_RS02425	78	A	C	synonymous

510837	NTHI477_RS02425	72	C	T	synonymous
510843	NTHI477_RS02425	66	A	G	synonymous
510846	NTHI477_RS02425	63	A	G	synonymous
510860	NTHI477_RS02425	49	G	C	nonsynonymous
510861	NTHI477_RS02425	48	T	C	synonymous
510863	NTHI477_RS02425	46	G	A	synonymous
510879	NTHI477_RS02425	30	A	G	synonymous
510882	NTHI477_RS02425	27	G	A	synonymous
510891	NTHI477_RS02425	18	A	G	synonymous
510894	NTHI477_RS02425	15	G	A	synonymous
510902	NTHI477_RS02425	7	G	A	synonymous
510932	intergenic	intergenic	T	C	intergenic
510934	intergenic	intergenic	G	A	intergenic
510939	intergenic	intergenic	A	G	intergenic
510944	intergenic	intergenic	T	C	intergenic
510949	intergenic	intergenic	C	T	intergenic
510972	intergenic	intergenic	C	T	intergenic
510973	intergenic	intergenic	A	G	intergenic
511001	intergenic	intergenic	C	T	intergenic
511016	intergenic	intergenic	C	T	intergenic
511045	NTHI477_RS02430	26	A	G	nonsynonymous
511046	NTHI477_RS02430	27	T	C	synonymous
511047	NTHI477_RS02430	28	G	A	nonsynonymous
511060	NTHI477_RS02430	41	A	G	nonsynonymous
511070	NTHI477_RS02430	51	T	G	nonsynonymous
511076	NTHI477_RS02430	57	C	T	synonymous
511109	NTHI477_RS02430	90	A	G	synonymous
511110	NTHI477_RS02430	91	T	C	synonymous
511128	NTHI477_RS02430	109	A	G	nonsynonymous
511157	NTHI477_RS02430	138	T	A	synonymous
511179	NTHI477_RS02430	160	G	A	nonsynonymous
511199	NTHI477_RS02430	180	C	A	nonsynonymous
511202	NTHI477_RS02430	183	T	C	synonymous
511213	NTHI477_RS02430	194	C	T	nonsynonymous
511226	NTHI477_RS02430	207	G	A	synonymous
511229	NTHI477_RS02430	210	A	T	synonymous
511236	NTHI477_RS02430	217	C	A	nonsynonymous
511246	NTHI477_RS02430	227	T	C	nonsynonymous
511247	NTHI477_RS02430	228	T	A	synonymous
511253	NTHI477_RS02430	234	C	A	nonsynonymous
511310	NTHI477_RS02430	291	G	C	synonymous
511333	NTHI477_RS02430	314	G	A	nonsynonymous
511353	NTHI477_RS02430	334	A	G	nonsynonymous
511364	NTHI477_RS02430	345	T	C	synonymous
511367	NTHI477_RS02430	348	T	C	synonymous

511380	intergenic	intergenic	C	T	intergenic
511382	intergenic	intergenic	G	A	intergenic
511383	intergenic	intergenic	A	C	intergenic
511386	intergenic	intergenic	A	T	intergenic
511389	intergenic	intergenic	C	T	intergenic
511390	intergenic	intergenic	A	G	intergenic
511397	intergenic	intergenic	T	C	intergenic
511411	NTHI477_RS02435	11	G	T	nonsynonymous
511414	NTHI477_RS02435	14	C	G	nonsynonymous
511415	NTHI477_RS02435	15	A	C	synonymous
511433	NTHI477_RS02435	33	A	G	synonymous
511439	NTHI477_RS02435	39	T	C	synonymous
511457	NTHI477_RS02435	57	G	A	synonymous
511496	NTHI477_RS02435	96	C	T	synonymous
511496	NTHI477_RS02435	96	C	A	synonymous
511499	NTHI477_RS02435	99	G	C	synonymous
511523	NTHI477_RS02435	123	G	A	synonymous
511550	NTHI477_RS02435	150	G	A	synonymous
511565	NTHI477_RS02435	165	G	A	synonymous
511586	NTHI477_RS02435	186	T	C	synonymous
511601	NTHI477_RS02435	201	G	A	synonymous
511622	NTHI477_RS02435	222	C	G	nonsynonymous
511642	NTHI477_RS02435	242	G	A	nonsynonymous
511655	NTHI477_RS02435	255	T	C	synonymous
511658	NTHI477_RS02435	258	C	T	synonymous
511667	NTHI477_RS02435	267	T	C	synonymous
511688	NTHI477_RS02435	288	C	A	nonsynonymous
511700	NTHI477_RS02435	300	C	T	synonymous
511712	NTHI477_RS02435	312	A	G	synonymous
511721	NTHI477_RS02435	321	A	T	synonymous
511738	NTHI477_RS02435	338	A	G	nonsynonymous
511745	NTHI477_RS02435	345	G	A	synonymous
511748	NTHI477_RS02435	348	T	A	synonymous
511760	NTHI477_RS02435	360	G	T	synonymous
511763	NTHI477_RS02435	363	T	A	nonsynonymous
511763	NTHI477_RS02435	363	T	G	nonsynonymous
511765	NTHI477_RS02435	365	G	A	nonsynonymous
511766	NTHI477_RS02435	366	T	C	synonymous
511767	NTHI477_RS02435	367	G	A	nonsynonymous
511768	NTHI477_RS02435	368	C	A	nonsynonymous
511774	NTHI477_RS02435	374	G	A	nonsynonymous
511775	NTHI477_RS02435	375	C	T	synonymous
511827	intergenic	intergenic	C	A	intergenic
511837	intergenic	intergenic	A	G	intergenic
511841	intergenic	intergenic	A	G	intergenic

511871	NTHI477_RS02440	599	G	A	nonsynonymous
511872	NTHI477_RS02440	598	T	C	nonsynonymous
511902	NTHI477_RS02440	568	A	G	nonsynonymous
511911	NTHI477_RS02440	559	G	C	nonsynonymous
511920	NTHI477_RS02440	550	A	G	synonymous
511921	NTHI477_RS02440	549	C	A	synonymous
511926	NTHI477_RS02440	544	C	T	nonsynonymous
511942	NTHI477_RS02440	528	A	T	synonymous
511965	NTHI477_RS02440	505	T	C	nonsynonymous
512054	NTHI477_RS02440	416	A	G	nonsynonymous
512110	NTHI477_RS02440	360	A	G	synonymous
512179	NTHI477_RS02440	291	A	C	nonsynonymous
512221	NTHI477_RS02440	249	G	A	synonymous
512305	NTHI477_RS02440	165	C	T	synonymous
512356	NTHI477_RS02440	114	G	A	synonymous
512359	NTHI477_RS02440	111	A	T	synonymous
512368	NTHI477_RS02440	102	C	T	synonymous
512371	NTHI477_RS02440	99	G	T	synonymous
512386	NTHI477_RS02440	84	C	A	synonymous
512389	NTHI477_RS02440	81	C	T	synonymous
512524	intergenic	intergenic	T	C	intergenic
512536	intergenic	intergenic	A	G	intergenic
512537	intergenic	intergenic	A	G	intergenic
512538	intergenic	intergenic	T	G	intergenic
512539	intergenic	intergenic	C	T	intergenic
512545	intergenic	intergenic	G	A	intergenic
512562	intergenic	intergenic	A	C	intergenic
512571	intergenic	intergenic	T	G	intergenic
512573	intergenic	intergenic	A	G	intergenic
512589	intergenic	intergenic	C	G	intergenic
512590	intergenic	intergenic	A	C	intergenic
512593	intergenic	intergenic	G	T	intergenic
512599	intergenic	intergenic	A	C	intergenic
512616	intergenic	intergenic	G	A	intergenic
512617	intergenic	intergenic	G	A	intergenic
512619	intergenic	intergenic	T	A	intergenic
512646	intergenic	intergenic	G	A	intergenic
512673	intergenic	intergenic	C	A	intergenic
512684	intergenic	intergenic	A	T	intergenic
512720	NTHI477_RS02445	33	A	T	synonymous
512754	NTHI477_RS02445	67	A	C	nonsynonymous
512756	NTHI477_RS02445	69	C	T	synonymous
512768	NTHI477_RS02445	81	C	T	synonymous
512798	NTHI477_RS02445	111	G	A	synonymous
512801	NTHI477_RS02445	114	C	A	synonymous

512822	NTHI477_RS02445	135	C	T	synonymous
512837	NTHI477_RS02445	150	C	T	synonymous
512849	NTHI477_RS02445	162	C	T	synonymous
512873	NTHI477_RS02445	186	T	C	synonymous
512882	NTHI477_RS02445	195	G	A	synonymous
512883	NTHI477_RS02445	196	A	G	nonsynonymous
512885	NTHI477_RS02445	198	C	A	synonymous
512887	NTHI477_RS02445	200	G	A	nonsynonymous
512918	NTHI477_RS02445	231	A	T	synonymous
512930	NTHI477_RS02445	243	C	T	synonymous
512969	NTHI477_RS02445	282	C	T	synonymous
512970	NTHI477_RS02445	283	G	A	nonsynonymous
512972	NTHI477_RS02445	285	G	A	synonymous
512982	intergenic	intergenic	A	C	intergenic
512987	intergenic	intergenic	G	A	intergenic
512987	intergenic	intergenic	G	T	intergenic
512990	intergenic	intergenic	C	T	intergenic
512994	intergenic	intergenic	G	A	intergenic
513009	intergenic	intergenic	G	C	intergenic
513012	intergenic	intergenic	C	T	intergenic
513013	intergenic	intergenic	A	C	intergenic
513033	intergenic	intergenic	G	T	intergenic
513036	intergenic	intergenic	T	C	intergenic
513039	intergenic	intergenic	T	A	intergenic
513042	intergenic	intergenic	A	C	intergenic
513046	intergenic	intergenic	A	G	intergenic
513046	intergenic	intergenic	A	C	intergenic
513051	intergenic	intergenic	C	T	intergenic
513061	intergenic	intergenic	C	T	intergenic
513078	intergenic	intergenic	T	G	intergenic
513205	intergenic	intergenic	T	G	intergenic
513221	intergenic	intergenic	T	C	intergenic
513232	intergenic	intergenic	C	T	intergenic
513233	intergenic	intergenic	T	C	intergenic
513235	intergenic	intergenic	T	A	intergenic
513333	intergenic	intergenic	A	C	intergenic
513334	intergenic	intergenic	T	C	intergenic
513346	intergenic	intergenic	A	G	intergenic
513607	intergenic	intergenic	A	T	intergenic
513672	intergenic	intergenic	A	G	intergenic
513705	intergenic	intergenic	C	T	intergenic
513763	intergenic	intergenic	C	A	intergenic
513764	intergenic	intergenic	C	T	intergenic
513765	intergenic	intergenic	T	G	intergenic
513792	intergenic	intergenic	C	A	intergenic

513813	intergenic	intergenic	G	A	intergenic
513814	intergenic	intergenic	G	A	intergenic
513831	NTHI477_RS02470	9	T	G	nonsynonymous
513839	NTHI477_RS02470	17	T	C	nonsynonymous
513840	NTHI477_RS02470	18	C	T	synonymous
513856	NTHI477_RS02470	34	A	C	nonsynonymous
513862	NTHI477_RS02470	40	G	A	nonsynonymous
513864	NTHI477_RS02470	42	T	C	synonymous
513865	NTHI477_RS02470	43	G	A	nonsynonymous
513871	NTHI477_RS02470	49	T	C	nonsynonymous
513874	NTHI477_RS02470	52	A	G	nonsynonymous
513877	NTHI477_RS02470	55	G	T	nonsynonymous
513922	NTHI477_RS02470	100	C	T	nonsynonymous
513925	NTHI477_RS02470	103	G	A	nonsynonymous
513977	NTHI477_RS02470	155	T	C	nonsynonymous
513999	NTHI477_RS02470	177	A	G	synonymous
514032	NTHI477_RS02470	210	C	T	synonymous
514050	NTHI477_RS02470	228	A	G	synonymous
514079	NTHI477_RS02470	257	A	G	nonsynonymous
514126	NTHI477_RS02470	304	T	C	synonymous
514156	NTHI477_RS02470	334	G	A	nonsynonymous
514164	NTHI477_RS02470	342	G	A	synonymous
514190	intergenic	intergenic	A	G	intergenic
514221	intergenic	intergenic	A	G	intergenic
514224	intergenic	intergenic	C	A	intergenic
514253	intergenic	intergenic	C	T	intergenic
514254	intergenic	intergenic	C	T	intergenic
514281	intergenic	intergenic	C	T	intergenic
514286	intergenic	intergenic	G	A	intergenic
514391	NTHI477_RS02475	75	G	A	synonymous
514427	NTHI477_RS02475	111	C	T	synonymous
514460	NTHI477_RS02475	144	C	T	synonymous
514649	NTHI477_RS02475	333	C	T	synonymous
514652	NTHI477_RS02475	336	T	G	synonymous
514655	NTHI477_RS02475	339	A	T	synonymous
514664	NTHI477_RS02475	348	C	T	synonymous
514667	NTHI477_RS02475	351	A	G	synonymous
514667	NTHI477_RS02475	351	A	T	synonymous
514670	NTHI477_RS02475	354	C	T	synonymous
514679	NTHI477_RS02475	363	C	A	synonymous
514682	NTHI477_RS02475	366	T	C	synonymous
514697	NTHI477_RS02475	381	A	C	synonymous
514700	NTHI477_RS02475	384	T	C	synonymous
514712	NTHI477_RS02475	396	T	C	synonymous
514721	NTHI477_RS02475	405	C	T	synonymous

514724	NTHI477_RS02475	408	A	T	synonymous
514742	NTHI477_RS02475	426	A	G	synonymous
514772	NTHI477_RS02475	456	A	G	synonymous
514775	NTHI477_RS02475	459	T	C	synonymous
514776	NTHI477_RS02475	460	A	G	nonsynonymous
514778	NTHI477_RS02475	462	T	C	synonymous
514787	NTHI477_RS02475	471	C	T	synonymous
514808	NTHI477_RS02475	492	C	T	synonymous
514835	NTHI477_RS02475	519	G	A	synonymous
514847	NTHI477_RS02475	531	C	T	synonymous
514850	NTHI477_RS02475	534	A	G	synonymous
514902	NTHI477_RS02475	586	G	A	nonsynonymous
514910	NTHI477_RS02475	594	C	T	synonymous
514913	NTHI477_RS02475	597	A	T	synonymous
514913	NTHI477_RS02475	597	A	G	synonymous
514914	NTHI477_RS02475	598	G	A	nonsynonymous
514916	NTHI477_RS02475	600	T	C	synonymous
514916	NTHI477_RS02475	600	T	A	synonymous
514928	NTHI477_RS02475	612	T	C	synonymous
514949	NTHI477_RS02475	633	T	A	synonymous
514949	NTHI477_RS02475	633	T	C	synonymous
514952	NTHI477_RS02475	636	G	A	synonymous
514953	NTHI477_RS02475	637	C	T	nonsynonymous
514964	NTHI477_RS02475	648	C	T	synonymous
514967	NTHI477_RS02475	651	A	G	synonymous
514970	NTHI477_RS02475	654	T	C	synonymous
514985	NTHI477_RS02475	669	C	T	synonymous
514988	NTHI477_RS02475	672	A	G	synonymous
514994	NTHI477_RS02475	678	G	T	synonymous
514997	NTHI477_RS02475	681	A	C	synonymous
515003	NTHI477_RS02475	687	A	G	synonymous
515033	NTHI477_RS02475	717	A	C	synonymous
515036	NTHI477_RS02475	720	C	T	synonymous
515045	NTHI477_RS02475	729	C	T	synonymous
515063	NTHI477_RS02475	747	T	C	synonymous
515072	NTHI477_RS02475	756	C	T	synonymous
515090	NTHI477_RS02475	774	T	C	synonymous
515093	NTHI477_RS02475	777	T	C	synonymous
515108	NTHI477_RS02475	792	T	A	synonymous
515111	NTHI477_RS02475	795	T	A	synonymous
515114	NTHI477_RS02475	798	C	T	synonymous
515132	NTHI477_RS02475	816	T	C	synonymous
515141	NTHI477_RS02475	825	T	C	synonymous
515156	NTHI477_RS02475	840	A	G	synonymous
515159	NTHI477_RS02475	843	T	A	synonymous

515159	NTHI477_RS02475	843	T	G	synonymous
515177	NTHI477_RS02475	861	A	G	synonymous
515192	NTHI477_RS02475	876	A	T	synonymous
515192	NTHI477_RS02475	876	A	G	synonymous
515207	NTHI477_RS02475	891	A	G	synonymous
515213	NTHI477_RS02475	897	T	C	synonymous
515216	NTHI477_RS02475	900	G	A	synonymous
515222	NTHI477_RS02475	906	A	C	synonymous
515225	NTHI477_RS02475	909	C	T	synonymous
515237	NTHI477_RS02475	921	T	C	synonymous
515240	NTHI477_RS02475	924	C	T	synonymous
515249	NTHI477_RS02475	933	C	T	synonymous
515258	NTHI477_RS02475	942	C	T	synonymous
515261	NTHI477_RS02475	945	C	A	synonymous
515264	NTHI477_RS02475	948	T	C	synonymous
515267	NTHI477_RS02475	951	T	C	synonymous
515282	NTHI477_RS02475	966	T	C	synonymous
515285	NTHI477_RS02475	969	C	T	synonymous
515288	NTHI477_RS02475	972	T	C	synonymous
515294	NTHI477_RS02475	978	T	A	synonymous
515318	NTHI477_RS02475	1002	C	T	synonymous
515321	NTHI477_RS02475	1005	A	G	synonymous
515322	NTHI477_RS02475	1006	T	C	synonymous
515342	NTHI477_RS02475	1026	A	T	synonymous
515351	NTHI477_RS02475	1035	T	C	synonymous
515417	NTHI477_RS02475	1101	A	T	nonsynonymous
515447	NTHI477_RS02475	1131	A	T	synonymous
515450	NTHI477_RS02475	1134	T	C	synonymous
515504	NTHI477_RS02475	1188	C	T	synonymous
515534	NTHI477_RS02475	1218	A	G	synonymous
515541	NTHI477_RS02475	1225	G	A	nonsynonymous
515546	NTHI477_RS02475	1230	T	A	synonymous
515564	NTHI477_RS02475	1248	T	A	synonymous
515579	NTHI477_RS02475	1263	A	G	synonymous
515585	NTHI477_RS02475	1269	A	C	synonymous
515603	NTHI477_RS02475	1287	G	A	synonymous
515607	NTHI477_RS02475	1291	G	T	nonsynonymous
515609	NTHI477_RS02475	1293	T	A	synonymous
515609	NTHI477_RS02475	1293	T	C	synonymous
515618	intergenic	intergenic	T	G	intergenic
515624	intergenic	intergenic	G	A	intergenic
515626	intergenic	intergenic	T	A	intergenic
515631	intergenic	intergenic	A	T	intergenic
515648	intergenic	intergenic	T	C	intergenic
515681	NTHI477_RS02480	819	A	G	synonymous

515699	NTHI477_RS02480	801	A	G	synonymous
515702	NTHI477_RS02480	798	G	A	synonymous
515705	NTHI477_RS02480	795	G	A	synonymous
515708	NTHI477_RS02480	792	C	T	synonymous
515714	NTHI477_RS02480	786	T	A	synonymous
515750	NTHI477_RS02480	750	G	T	synonymous
515753	NTHI477_RS02480	747	A	G	synonymous
515756	NTHI477_RS02480	744	A	G	synonymous
515761	NTHI477_RS02480	739	G	A	synonymous
515795	NTHI477_RS02480	705	T	A	synonymous
515831	NTHI477_RS02480	669	A	G	synonymous
515885	NTHI477_RS02480	615	G	T	synonymous
515942	NTHI477_RS02480	558	C	T	synonymous
515942	NTHI477_RS02480	558	C	A	synonymous
515945	NTHI477_RS02480	555	G	A	synonymous
515966	NTHI477_RS02480	534	G	A	synonymous
515972	NTHI477_RS02480	528	G	A	synonymous
516011	NTHI477_RS02480	489	G	T	synonymous
516062	NTHI477_RS02480	438	T	A	synonymous
516086	NTHI477_RS02480	414	G	T	synonymous
516089	NTHI477_RS02480	411	G	A	synonymous
516152	NTHI477_RS02480	348	A	G	synonymous
516158	NTHI477_RS02480	342	G	A	synonymous
516197	NTHI477_RS02480	303	T	A	nonsynonymous
516269	NTHI477_RS02480	231	G	A	synonymous
516272	NTHI477_RS02480	228	G	A	synonymous
516414	NTHI477_RS02480	86	G	A	nonsynonymous
516416	NTHI477_RS02480	84	A	G	synonymous
516515	intergenic	intergenic	T	C	intergenic
516533	intergenic	intergenic	A	G	intergenic
516626	intergenic	intergenic	G	A	intergenic
516627	intergenic	intergenic	A	G	intergenic
517073	NTHI477_RS02485	213	A	T	synonymous
517157	NTHI477_RS02485	297	T	C	synonymous
517178	NTHI477_RS02485	318	T	C	synonymous
517184	NTHI477_RS02485	324	A	G	synonymous
517304	NTHI477_RS02485	444	C	G	synonymous
517311	NTHI477_RS02485	451	G	A	nonsynonymous
517313	NTHI477_RS02485	453	G	C	synonymous
517319	NTHI477_RS02485	459	C	A	synonymous
517325	NTHI477_RS02485	465	G	T	synonymous
517352	NTHI477_RS02485	492	T	A	synonymous
517409	NTHI477_RS02485	549	C	T	synonymous
517556	NTHI477_RS02485	696	G	A	synonymous
517566	NTHI477_RS02485	706	G	A	nonsynonymous

517607	NTHI477_RS02485	747	A	G	synonymous
517607	NTHI477_RS02485	747	A	T	synonymous
517625	NTHI477_RS02485	765	A	G	synonymous
517638	NTHI477_RS02485	778	G	T	nonsynonymous
517676	NTHI477_RS02485	816	T	C	synonymous
517694	NTHI477_RS02485	834	A	G	synonymous
517703	NTHI477_RS02485	843	C	T	synonymous
517706	NTHI477_RS02485	846	A	G	synonymous
517769	NTHI477_RS02485	909	A	G	synonymous
517797	NTHI477_RS02485	937	G	A	nonsynonymous
517799	NTHI477_RS02485	939	T	C	synonymous
517820	NTHI477_RS02485	960	C	T	synonymous
517841	NTHI477_RS02485	981	C	T	synonymous
517861	NTHI477_RS02485	1001	C	T	nonsynonymous
517877	intergenic	intergenic	G	T	intergenic
517886	intergenic	intergenic	G	A	intergenic
517902	intergenic	intergenic	T	C	intergenic
518069	NTHI477_RS02490	16	A	C	nonsynonymous
518071	NTHI477_RS02490	18	T	C	synonymous
518077	NTHI477_RS02490	24	G	A	synonymous
518122	NTHI477_RS02490	69	C	T	synonymous
518149	NTHI477_RS02490	96	C	T	synonymous
518217	NTHI477_RS02490	164	C	T	nonsynonymous
518217	NTHI477_RS02490	164	C	A	nonsynonymous
518224	NTHI477_RS02490	171	C	A	synonymous
518227	NTHI477_RS02490	174	T	C	synonymous
518233	NTHI477_RS02490	180	C	A	synonymous
518263	NTHI477_RS02490	210	C	T	synonymous
518281	NTHI477_RS02490	228	T	C	synonymous
518308	NTHI477_RS02490	255	A	T	synonymous
518323	NTHI477_RS02490	270	C	A	synonymous
518323	NTHI477_RS02490	270	C	T	synonymous
518347	NTHI477_RS02490	294	A	T	synonymous
518352	NTHI477_RS02490	299	G	A	nonsynonymous
518365	NTHI477_RS02490	312	C	T	synonymous
518404	NTHI477_RS02490	351	T	C	synonymous
518416	NTHI477_RS02490	363	G	C	nonsynonymous
518456	NTHI477_RS02490	403	C	T	synonymous
518492	NTHI477_RS02490	439	T	C	synonymous
518578	NTHI477_RS02490	525	A	G	synonymous
518579	NTHI477_RS02490	526	C	T	nonsynonymous
518593	NTHI477_RS02490	540	T	C	synonymous
518605	NTHI477_RS02490	552	A	G	synonymous
518608	NTHI477_RS02490	555	A	T	synonymous
518638	NTHI477_RS02490	585	C	T	synonymous

518644	NTHI477_RS02490	591	G	A	synonymous
518656	NTHI477_RS02490	603	G	A	synonymous
518659	NTHI477_RS02490	606	T	G	synonymous
518659	NTHI477_RS02490	606	T	C	synonymous
518680	NTHI477_RS02490	627	A	G	synonymous
518686	NTHI477_RS02490	633	C	T	synonymous
518701	NTHI477_RS02490	648	T	A	synonymous
518710	NTHI477_RS02490	657	A	G	synonymous
518725	NTHI477_RS02490	672	C	A	synonymous
518735	NTHI477_RS02490	682	G	A	nonsynonymous
518755	NTHI477_RS02490	702	T	C	synonymous
518758	NTHI477_RS02490	705	C	T	synonymous
518762	NTHI477_RS02490	709	C	T	nonsynonymous
518764	NTHI477_RS02490	711	A	G	synonymous
518767	NTHI477_RS02490	714	A	G	synonymous
518842	NTHI477_RS02490	789	T	C	synonymous
518866	NTHI477_RS02490	813	G	C	synonymous
518869	NTHI477_RS02490	816	C	T	synonymous
518881	NTHI477_RS02490	828	A	G	synonymous
518913	NTHI477_RS02490	860	C	T	nonsynonymous
518955	NTHI477_RS02490	902	A	C	nonsynonymous
518971	NTHI477_RS02490	918	T	G	nonsynonymous
519017	NTHI477_RS02490	964	A	G	nonsynonymous
519091	NTHI477_RS02490	1038	G	A	synonymous
519110	NTHI477_RS02490	1057	G	A	nonsynonymous
519112	NTHI477_RS02490	1059	G	A	synonymous
519147	NTHI477_RS02490	1094	A	G	nonsynonymous
519148	NTHI477_RS02490	1095	C	T	synonymous
519166	NTHI477_RS02490	1113	G	A	synonymous
519179	NTHI477_RS02490	1126	C	T	synonymous
519182	NTHI477_RS02490	1129	A	G	nonsynonymous
519211	NTHI477_RS02490	1158	C	T	synonymous
519217	NTHI477_RS02490	1164	A	G	synonymous
519223	NTHI477_RS02490	1170	T	C	synonymous
519250	NTHI477_RS02490	1197	T	A	synonymous
519250	NTHI477_RS02490	1197	T	C	synonymous
519256	NTHI477_RS02490	1203	C	T	synonymous
519265	NTHI477_RS02490	1212	C	A	synonymous
519265	NTHI477_RS02490	1212	C	G	synonymous
519268	NTHI477_RS02490	1215	T	C	synonymous
519295	NTHI477_RS02490	1242	G	A	synonymous
519298	NTHI477_RS02490	1245	C	T	synonymous
519305	NTHI477_RS02490	1252	T	G	nonsynonymous
519315	NTHI477_RS02490	1262	T	C	nonsynonymous
519316	NTHI477_RS02490	1263	G	A	nonsynonymous

519322	NTHI477_RS02490	1269	C	T	synonymous
519346	NTHI477_RS02490	1293	T	C	synonymous
519361	NTHI477_RS02490	1308	C	T	synonymous
519370	NTHI477_RS02490	1317	G	A	synonymous
519388	NTHI477_RS02490	1335	A	G	synonymous
519424	NTHI477_RS02490	1371	T	C	synonymous
519435	NTHI477_RS02490	1382	C	T	nonsynonymous
519454	NTHI477_RS02490	1401	A	C	synonymous
519457	NTHI477_RS02490	1404	A	T	synonymous
519460	NTHI477_RS02490	1407	T	G	synonymous
519475	NTHI477_RS02490	1422	G	A	synonymous
519481	NTHI477_RS02490	1428	G	A	synonymous
519486	NTHI477_RS02490	1433	G	A	nonsynonymous
519490	NTHI477_RS02490	1437	A	C	synonymous
519527	NTHI477_RS02490	1474	G	T	nonsynonymous
519529	NTHI477_RS02490	1476	A	T	synonymous
519530	NTHI477_RS02490	1477	G	A	nonsynonymous
519544	NTHI477_RS02490	1491	T	C	synonymous
519565	NTHI477_RS02490	1512	T	C	synonymous
519574	NTHI477_RS02490	1521	A	G	synonymous
519581	NTHI477_RS02490	1528	T	C	synonymous
519592	NTHI477_RS02490	1539	C	T	synonymous
519601	NTHI477_RS02490	1548	G	C	synonymous
519615	NTHI477_RS02490	1562	T	A	nonsynonymous
519640	NTHI477_RS02490	1587	G	C	synonymous
519640	NTHI477_RS02490	1587	G	T	synonymous
519643	NTHI477_RS02490	1590	C	T	synonymous
519670	NTHI477_RS02490	1617	C	T	synonymous
519676	NTHI477_RS02490	1623	A	G	synonymous
519682	NTHI477_RS02490	1629	G	A	synonymous
519706	NTHI477_RS02490	1653	T	A	synonymous
519709	NTHI477_RS02490	1656	C	T	synonymous
519718	NTHI477_RS02490	1665	G	A	synonymous
519724	NTHI477_RS02490	1671	C	T	synonymous
519730	NTHI477_RS02490	1677	A	G	synonymous
519745	NTHI477_RS02490	1692	G	A	synonymous
519754	NTHI477_RS02490	1701	A	G	synonymous
519766	NTHI477_RS02490	1713	C	T	synonymous
519823	NTHI477_RS02490	1770	A	G	synonymous
519838	NTHI477_RS02490	1785	A	G	synonymous
519847	NTHI477_RS02490	1794	G	A	synonymous
519850	NTHI477_RS02490	1797	A	G	synonymous
519859	NTHI477_RS02490	1806	C	A	synonymous
519860	NTHI477_RS02490	1807	T	C	synonymous
519904	NTHI477_RS02490	1851	C	T	synonymous

519922	NTHI477_RS02490	1869	C	G	synonymous
519925	NTHI477_RS02490	1872	A	T	synonymous
519928	NTHI477_RS02490	1875	A	G	synonymous
519931	NTHI477_RS02490	1878	A	G	synonymous
519949	NTHI477_RS02490	1896	T	C	synonymous
519955	NTHI477_RS02490	1902	C	T	synonymous
519958	NTHI477_RS02490	1905	C	T	synonymous
519961	NTHI477_RS02490	1908	C	T	synonymous
519979	NTHI477_RS02490	1926	T	G	synonymous
519985	NTHI477_RS02490	1932	G	A	synonymous
520014	NTHI477_RS02490	1961	C	A	nonsynonymous
520015	NTHI477_RS02490	1962	T	C	synonymous
520120	NTHI477_RS02490	2067	A	G	synonymous
520123	NTHI477_RS02490	2070	A	C	synonymous
520132	NTHI477_RS02490	2079	A	G	synonymous
520174	NTHI477_RS02490	2121	T	C	synonymous
520192	NTHI477_RS02490	2139	G	C	synonymous
520195	NTHI477_RS02490	2142	C	T	synonymous
520198	NTHI477_RS02490	2145	G	T	synonymous
520210	NTHI477_RS02490	2157	T	C	synonymous
520222	NTHI477_RS02490	2169	C	T	synonymous
520225	NTHI477_RS02490	2172	T	C	synonymous
520234	NTHI477_RS02490	2181	A	G	synonymous
520249	NTHI477_RS02490	2196	G	A	synonymous
520271	NTHI477_RS02490	2218	G	A	nonsynonymous
520360	NTHI477_RS02490	2307	C	A	synonymous
520420	NTHI477_RS02490	2367	G	A	synonymous
520420	NTHI477_RS02490	2367	G	T	nonsynonymous
520435	NTHI477_RS02490	2382	T	C	synonymous
520499	NTHI477_RS02490	2446	G	A	nonsynonymous
520537	NTHI477_RS02490	2484	T	C	synonymous
520543	NTHI477_RS02490	2490	G	A	synonymous
520558	NTHI477_RS02490	2505	A	G	synonymous
520564	NTHI477_RS02490	2511	A	C	synonymous
520573	NTHI477_RS02490	2520	A	T	synonymous
520582	NTHI477_RS02490	2529	G	A	synonymous
520588	NTHI477_RS02490	2535	G	A	synonymous
520591	NTHI477_RS02490	2538	T	C	synonymous
520621	NTHI477_RS02490	2568	C	T	synonymous
520654	NTHI477_RS02490	2601	C	T	synonymous
520669	NTHI477_RS02490	2616	G	A	synonymous
520687	NTHI477_RS02490	2634	G	A	synonymous
520696	intergenic	intergenic	A	G	intergenic
520785	NTHI477_RS02495	1359	A	G	synonymous
520791	NTHI477_RS02495	1353	G	A	synonymous

520813	NTHI477_RS02495	1331	T	A	nonsynonymous
520830	NTHI477_RS02495	1314	A	G	synonymous
520865	NTHI477_RS02495	1279	T	C	nonsynonymous
520867	NTHI477_RS02495	1277	T	C	nonsynonymous
520878	NTHI477_RS02495	1266	A	G	synonymous
520881	NTHI477_RS02495	1263	C	T	synonymous
520887	NTHI477_RS02495	1257	G	C	synonymous
520893	NTHI477_RS02495	1251	T	C	synonymous
520901	NTHI477_RS02495	1243	T	G	nonsynonymous
520902	NTHI477_RS02495	1242	C	T	synonymous
520909	NTHI477_RS02495	1235	A	G	nonsynonymous
520911	NTHI477_RS02495	1233	C	T	synonymous
520931	NTHI477_RS02495	1213	C	T	nonsynonymous
520941	NTHI477_RS02495	1203	T	C	synonymous
520947	NTHI477_RS02495	1197	A	C	nonsynonymous
520968	NTHI477_RS02495	1176	C	T	synonymous
520971	NTHI477_RS02495	1173	A	G	synonymous
520980	NTHI477_RS02495	1164	C	T	synonymous
520998	NTHI477_RS02495	1146	C	T	synonymous
521004	NTHI477_RS02495	1140	A	G	synonymous
521004	NTHI477_RS02495	1140	A	C	synonymous
521016	NTHI477_RS02495	1128	C	T	synonymous
521019	NTHI477_RS02495	1125	A	G	synonymous
521022	NTHI477_RS02495	1122	G	A	synonymous
521037	NTHI477_RS02495	1107	C	T	synonymous
521049	NTHI477_RS02495	1095	A	G	synonymous
521064	NTHI477_RS02495	1080	G	T	nonsynonymous
521070	NTHI477_RS02495	1074	C	T	nonsynonymous
521079	NTHI477_RS02495	1065	C	T	synonymous
521081	NTHI477_RS02495	1063	C	T	nonsynonymous
521091	NTHI477_RS02495	1053	A	C	nonsynonymous
521100	NTHI477_RS02495	1044	A	G	synonymous
521118	NTHI477_RS02495	1026	C	T	synonymous
521121	NTHI477_RS02495	1023	T	G	synonymous
521121	NTHI477_RS02495	1023	T	C	synonymous
521149	NTHI477_RS02495	995	C	T	nonsynonymous
521150	NTHI477_RS02495	994	G	T	synonymous
521151	NTHI477_RS02495	993	C	T	synonymous
521154	NTHI477_RS02495	990	A	G	synonymous
521178	NTHI477_RS02495	966	T	C	synonymous
521181	NTHI477_RS02495	963	G	A	synonymous
521184	NTHI477_RS02495	960	G	A	synonymous
521211	NTHI477_RS02495	933	A	G	synonymous
521217	NTHI477_RS02495	927	G	A	synonymous
521247	NTHI477_RS02495	897	A	G	synonymous

521252	NTHI477_RS02495	892	G	T	nonsynonymous
521283	NTHI477_RS02495	861	A	C	synonymous
521289	NTHI477_RS02495	855	A	G	synonymous
521298	NTHI477_RS02495	846	G	A	synonymous
521299	NTHI477_RS02495	845	G	T	nonsynonymous
521351	NTHI477_RS02495	793	C	T	nonsynonymous
521359	NTHI477_RS02495	785	T	G	nonsynonymous
521370	NTHI477_RS02495	774	A	G	synonymous
521385	NTHI477_RS02495	759	T	C	synonymous
521391	NTHI477_RS02495	753	T	G	synonymous
521410	NTHI477_RS02495	734	C	T	nonsynonymous
521415	NTHI477_RS02495	729	C	A	nonsynonymous
521424	NTHI477_RS02495	720	T	C	synonymous
521436	NTHI477_RS02495	708	T	C	synonymous
521442	NTHI477_RS02495	702	G	A	synonymous
521451	NTHI477_RS02495	693	T	C	synonymous
521455	NTHI477_RS02495	689	G	A	nonsynonymous
521457	NTHI477_RS02495	687	G	A	synonymous
521457	NTHI477_RS02495	687	G	T	synonymous
521460	NTHI477_RS02495	684	C	A	synonymous
521462	NTHI477_RS02495	682	C	T	nonsynonymous
521463	NTHI477_RS02495	681	A	G	synonymous
521466	NTHI477_RS02495	678	C	G	nonsynonymous
521478	NTHI477_RS02495	666	C	T	synonymous
521484	NTHI477_RS02495	660	T	C	synonymous
521496	NTHI477_RS02495	648	A	G	synonymous
521521	NTHI477_RS02495	623	A	G	nonsynonymous
521526	NTHI477_RS02495	618	G	A	synonymous
521532	NTHI477_RS02495	612	A	G	synonymous
521559	NTHI477_RS02495	585	T	A	synonymous
521606	NTHI477_RS02495	538	C	T	nonsynonymous
521610	NTHI477_RS02495	534	C	T	synonymous
521616	NTHI477_RS02495	528	A	G	synonymous
521618	NTHI477_RS02495	526	C	A	nonsynonymous
521619	NTHI477_RS02495	525	G	A	synonymous
521621	NTHI477_RS02495	523	G	A	nonsynonymous
521630	NTHI477_RS02495	514	C	G	nonsynonymous
521634	NTHI477_RS02495	510	C	T	synonymous
521641	NTHI477_RS02495	503	T	G	nonsynonymous
521642	NTHI477_RS02495	502	C	T	nonsynonymous
521643	NTHI477_RS02495	501	A	G	synonymous
521643	NTHI477_RS02495	501	A	T	synonymous
521648	NTHI477_RS02495	496	T	C	nonsynonymous
521649	NTHI477_RS02495	495	G	A	synonymous
521652	NTHI477_RS02495	492	A	T	synonymous

521655	NTHI477_RS02495	489	T	G	synonymous
521658	NTHI477_RS02495	486	G	T	synonymous
521659	NTHI477_RS02495	485	A	G	nonsynonymous
521664	NTHI477_RS02495	480	A	G	synonymous
521666	NTHI477_RS02495	478	T	C	nonsynonymous
521670	NTHI477_RS02495	474	C	T	synonymous
521671	NTHI477_RS02495	473	T	C	nonsynonymous
521672	NTHI477_RS02495	472	C	T	nonsynonymous
521692	NTHI477_RS02495	452	T	C	nonsynonymous
521696	NTHI477_RS02495	448	G	A	synonymous
521703	NTHI477_RS02495	441	T	C	synonymous
521717	NTHI477_RS02495	427	G	T	nonsynonymous
521718	NTHI477_RS02495	426	C	T	synonymous
521720	NTHI477_RS02495	424	G	A	synonymous
521727	NTHI477_RS02495	417	C	A	synonymous
521727	NTHI477_RS02495	417	C	T	synonymous
521730	NTHI477_RS02495	414	T	G	synonymous
521730	NTHI477_RS02495	414	T	C	synonymous
521748	NTHI477_RS02495	396	T	C	synonymous
521758	NTHI477_RS02495	386	A	G	nonsynonymous
521762	NTHI477_RS02495	382	C	T	nonsynonymous
521763	NTHI477_RS02495	381	T	G	synonymous
521778	NTHI477_RS02495	366	A	G	synonymous
521796	NTHI477_RS02495	348	G	A	synonymous
521811	NTHI477_RS02495	333	T	G	synonymous
521835	NTHI477_RS02495	309	A	T	synonymous
521844	NTHI477_RS02495	300	T	C	synonymous
521856	NTHI477_RS02495	288	A	C	synonymous
521859	NTHI477_RS02495	285	C	T	synonymous
521904	NTHI477_RS02495	240	T	C	synonymous
521937	NTHI477_RS02495	207	G	A	synonymous
521946	NTHI477_RS02495	198	A	G	synonymous
521949	NTHI477_RS02495	195	T	C	synonymous
521951	NTHI477_RS02495	193	A	C	nonsynonymous
521961	NTHI477_RS02495	183	C	A	synonymous
521964	NTHI477_RS02495	180	T	C	synonymous
521984	NTHI477_RS02495	160	T	G	nonsynonymous
521992	NTHI477_RS02495	152	G	A	nonsynonymous
521996	NTHI477_RS02495	148	G	T	nonsynonymous
522006	NTHI477_RS02495	138	A	G	synonymous
522015	NTHI477_RS02495	129	T	C	synonymous
522021	NTHI477_RS02495	123	G	A	synonymous
522054	NTHI477_RS02495	90	C	T	synonymous
522066	NTHI477_RS02495	78	T	C	synonymous
522069	NTHI477_RS02495	75	T	C	synonymous

522075	NTHI477_RS02495	69	C	T	synonymous
522078	NTHI477_RS02495	66	G	A	synonymous
522090	NTHI477_RS02495	54	G	A	synonymous
522185	intergenic	intergenic	A	G	intergenic
522253	NTHI477_RS02500	41	G	C	nonsynonymous
522254	NTHI477_RS02500	42	T	C	synonymous
522259	NTHI477_RS02500	47	T	G	nonsynonymous
522267	NTHI477_RS02500	55	G	A	nonsynonymous
522278	NTHI477_RS02500	66	G	A	synonymous
522291	NTHI477_RS02500	79	G	A	nonsynonymous
522314	NTHI477_RS02500	102	T	C	synonymous
522335	NTHI477_RS02500	123	C	T	synonymous
522365	NTHI477_RS02500	153	T	A	synonymous
522365	NTHI477_RS02500	153	T	G	synonymous
522395	NTHI477_RS02500	183	C	T	synonymous
522401	NTHI477_RS02500	189	A	G	synonymous
522464	NTHI477_RS02500	252	A	G	synonymous
522479	NTHI477_RS02500	267	T	C	synonymous
522484	NTHI477_RS02500	272	C	A	nonsynonymous
522494	NTHI477_RS02500	282	A	G	synonymous
522495	NTHI477_RS02500	283	G	A	nonsynonymous
522572	NTHI477_RS02500	360	T	G	synonymous
522578	NTHI477_RS02500	366	C	T	synonymous
522608	NTHI477_RS02500	396	G	A	synonymous
522635	NTHI477_RS02500	423	T	A	synonymous
522639	NTHI477_RS02500	427	A	G	nonsynonymous
522659	NTHI477_RS02500	447	T	C	synonymous
522670	NTHI477_RS02500	458	G	A	nonsynonymous
522761	NTHI477_RS02500	549	G	A	synonymous
522790	NTHI477_RS02500	578	C	A	nonsynonymous
522791	NTHI477_RS02500	579	A	G	synonymous
522806	NTHI477_RS02500	594	C	T	synonymous
522812	NTHI477_RS02500	600	T	G	synonymous
522833	NTHI477_RS02500	621	C	A	synonymous
522881	NTHI477_RS02500	669	C	T	synonymous
522885	NTHI477_RS02500	673	G	A	nonsynonymous
522899	NTHI477_RS02500	687	C	T	synonymous
522902	NTHI477_RS02500	690	C	T	synonymous
522911	NTHI477_RS02500	699	A	C	synonymous
522947	NTHI477_RS02500	735	C	T	synonymous
522956	NTHI477_RS02500	744	T	C	synonymous
522964	NTHI477_RS02500	752	T	C	nonsynonymous
522965	NTHI477_RS02500	753	G	A	synonymous
522968	NTHI477_RS02500	756	C	T	synonymous
522971	NTHI477_RS02500	759	T	C	synonymous

522977	NTHI477_RS02500	765	T	C	synonymous
522995	NTHI477_RS02500	783	T	C	synonymous
522995	NTHI477_RS02500	783	T	A	synonymous
522998	NTHI477_RS02500	786	G	A	synonymous
523001	NTHI477_RS02500	789	A	T	synonymous
523002	NTHI477_RS02500	790	G	C	nonsynonymous
523005	NTHI477_RS02500	793	C	A	nonsynonymous
523013	NTHI477_RS02500	801	T	C	synonymous
523020	NTHI477_RS02500	808	C	T	nonsynonymous
523022	NTHI477_RS02500	810	T	A	synonymous
523032	NTHI477_RS02500	820	C	A	nonsynonymous
523034	NTHI477_RS02500	822	C	A	synonymous
523036	NTHI477_RS02500	824	G	A	nonsynonymous
523040	NTHI477_RS02500	828	A	G	synonymous
523043	NTHI477_RS02500	831	T	A	synonymous
523044	NTHI477_RS02500	832	T	C	synonymous
523046	NTHI477_RS02500	834	A	C	nonsynonymous
523062	NTHI477_RS02500	850	A	C	nonsynonymous
523064	NTHI477_RS02500	852	T	C	synonymous
523075	NTHI477_RS02500	863	T	G	nonsynonymous
523076	NTHI477_RS02500	864	C	A	synonymous
523079	NTHI477_RS02500	867	C	T	synonymous
523094	NTHI477_RS02500	882	A	G	synonymous
523101	NTHI477_RS02500	889	A	C	nonsynonymous
523115	NTHI477_RS02500	903	A	G	synonymous
523117	NTHI477_RS02500	905	A	G	nonsynonymous
523118	NTHI477_RS02500	906	T	C	synonymous
523119	NTHI477_RS02500	907	G	A	nonsynonymous
523121	NTHI477_RS02500	909	T	C	synonymous
523124	NTHI477_RS02500	912	C	T	synonymous
523127	NTHI477_RS02500	915	A	G	synonymous
523130	NTHI477_RS02500	918	T	C	synonymous
523133	NTHI477_RS02500	921	T	C	synonymous
523138	NTHI477_RS02500	926	T	C	nonsynonymous
523145	NTHI477_RS02500	933	T	C	synonymous
[truncated: 496,141 more chars]
